# Neurotoxicity, α-synuclein pathology, and mitochondrial dysfunction: A comparative study of different mouse models of Parkinson’s disease

**DOI:** 10.4103/NRR.NRR-D-25-00648

**Published:** 2025-09-29

**Authors:** Xiwen Tang, Yifei He, Min Liang, Penggang Ning, Jiayin Zhao, Yunhe Zhang, Xin Yan, Ruilin Sun, Gang Wei, Ruling Shen, Fang Huang, Mei Yu

**Affiliations:** 1 Department of Translational Neuroscience, Jing’an District Center Hospital of Shanghai, State Key Laboratory of Medical Neurobiology and MOE Frontiers Center for Brain Science, Institutes of Brain Science, Fudan University, Shanghai, China; 2 Shanghai Laboratory Animal Research Center, Shanghai, China; 3 Shanghai Engineering Research Center for Model Organisms, Shanghai Model Organisms Center, Inc., Shanghai, China; 4 State Key Laboratory of Advanced Drug Formulations for Overcoming Delivery Barriers, Fudan University, Shanghai, China

**Keywords:** 1-methyl-4-phenyl-1, 2, 3, 6-tetrahydropyridine-induced model, ferroptosis pathway, glial cell activation, interleukin 17 pathway, mitochondrial dysfunction, MitoPark model, nerve regeneration, neurodegeneration, nigrostriatal pathway, pS129-α-syn, α-syn A53T transgenic model

## Abstract

The causes of Parkinson’s disease are complex, and it is difficult for a single animal model to fully mimic its pathological characteristics. In this study, a comprehensive analysis of behaviors, Parkinson’s disease–like pathologies, and gene and protein expression profiles was carried out in three mouse models of disease: 1-methyl-4-phenyl-1,2,3,6-tetrahydropyridine-induced, α-synuclein (α-syn) A53T transgenic, and MitoPark, revealing both shared and model-specific pathogenic pathways to guide model selection and identify potential therapeutic targets. All three Parkinson’s disease models exhibited motor impairments, with particularly pronounced age-related decline observed in MitoPark mice. Pathologically, nigrostriatal pathway damage was observed in all models, yet with distinct patterns of glial cell activation. Sixteen-month-old α-syn A53T mice displayed a few pS129-α-syn-positive signals in the substantia nigra, while no α-syn aggregates were observed in any of the models. RNA sequencing and proteomics analysis revealed significant changes in gene and protein expression, with both unique and common features among the three models. Five common differentially expressed genes (*Ifi27l2a*, *Ifitm3*, *Oasl2*, *Rtp4*, and *Ankk1*) and two common differentially expressed proteins (*Timm8a1* and *Sephs1*) were identified. Functional enrichment analysis indicated that immune responses, cytokines, and neurotransmitter transport were crucial in Parkinson’s disease pathogenesis. Notably, multiple iron-related cell damage (ferroptosis)-related differentially expressed genes were identified across all three models, while interleukin 17 pathway activation was altered in MitoPark mice. In summary, we analyzed the commonalities and specificities of pathological simulation capabilities and common disease mechanisms in different mouse models of Parkinson’s disease from multiple perspectives. Our findings offer valuable insights into the multifaceted characteristics of Parkinson’s disease and will assist in model selection for mechanistic exploration in the future.

## Graphical abstract

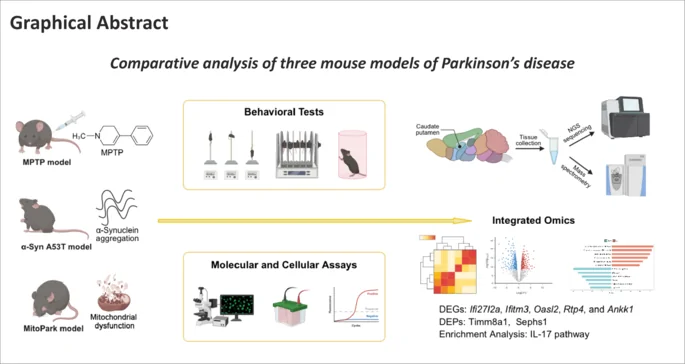

## Introduction

Parkinson’s disease (PD) is characterized by motor symptoms such as rigidity and tremors that result from degeneration of midbrain dopaminergic neurons in the substantia nigra pars compacta (SNc) and subsequent depletion of striatal dopamine (Ye et al., 2024). Surviving neurons contain Lewy bodies, which are mainly composed of misfolded proteins, with α-synuclein (α-syn) aggregates as a key component (Li et al., 2024; Zhu et al., 2024). The pathogenesis of PD is highly intricate, and is influenced by genetics, environment, aging, and mitochondrial dysfunction. Despite extensive research, the molecular mechanisms underlying PD pathogenesis remain elusive.

While animal models are indispensable tools for PD research, each system has distinct limitations with regard to recapitulating the complex pathology of the disease (Zhu et al., 2023). In this study, we systematically evaluated three established PD mouse models: 1-methyl-4-phenyl-1,2,3,6-tetrahydropyridine (MPTP)-induced, α-syn A53T transgenic, and MitoPark. These models represent, respectively, three core pathological pathways of PD: environmental toxin–induced pathogenesis (Pan-Montojo and Reichmann, 2014), abnormal α-syn aggregation–related pathogenesis (Tofaris, 2022), and mitochondrial dysfunction–associated pathogenesis (Banerjee et al., 2009).

The neurotoxin MPTP rapidly damages dopaminergic neurons (Ransom et al., 1987; Bardien et al., 2009). Although acute and subacute protocols enable efficient model generation, they fail to recapitulate chronic disease progression, metabolic alterations, or α-syn inclusion formation, which are key characteristics of human PD (Halliday et al., 2009).

Transgenic mice expressing human A53T-mutant *SNCA* under control of the prion protein promoter demonstrate age-dependent, modest dopaminergic neuron loss (Lee et al., 2002). However, this model exhibits variable motor phenotypes across transgenic lines and requires extended experimental timelines due to late-onset pathology.

Mitochondrial DNA encodes the 13 key respiratory chain subunits crucial for mitochondrial biogenesis and energy-related processes (Wculek et al., 2023). Transcription factor A, mitochondrial (TFAM) is essential for mitochondrial DNA transcription and maintenance. The MitoPark model, in which TFAM is knocked out in dopamine neurons, exhibits progressive dopaminergic neuron loss and PD-like symptoms but lacks α-syn inclusions (Beckstead and Howell, 2021; Fifel et al., 2023).

The aim of this study was to comprehensively analyze these models using behavioral, pathological, and molecular methods. We performed a variety of behavioral tests to assess behavioral impairments as precisely as possible. Immunohistochemistry and western blot assays were conducted to examine the status of dopaminergic neurons, astrocytes, microglia, and α-syn expression within the nigrostriatal pathway. RNA sequencing (RNA-seq) and proteomics were used to profile gene and protein expression changes in the striatum. Functional enrichment analysis was used to identify key PD-related processes and pathways.

Using this multidimensional experimental approach, we focused on differences in the pathological characteristics of the three PD models and analyzed their commonalities and specificities. The behavioral, pathological, and molecular characteristics of the three mouse models were compared systematically to provide new insights into the multifactorial characteristics of PD and aid in model selection.

## Methods

### Animals

Male C57BL/6 mice (10–12 weeks old, weighing 22–28 g) were obtained from the Shanghai Model Organisms Center (Shanghai, China). The mice were randomly divided into two groups (*n* = 12/group): the normal saline (NS) control group and the MPTP-treated group.

Wild-type (WT) mice were used as controls for the transgenic mice. MitoPark mice were generated by crossing C57BL/6Smoc-*Tfam*^tm1(flox)^ mice (Shanghai Model Organisms Center, Cat. No. NM-CKO-200173; RRID: IMSR_NM-CKO-200173) with B6.SJL-*Slc6a3*^tm1.1(cre)Bkmn^/J mice (Jackson Laboratory, Bar Harbor, ME, USA; Strain 006660; RRID:IMSR_JAX:006660). Male WT control mice and male MitoPark transgenic mice were used at the following ages: 3 months (*n* = 11, body weight: WT 25–32 g, MitoPark 23–30 g), 4 months (*n* = 11, body weight: WT 25–32 g, MitoPark 26–32 g), 5 months (*n* = 7, body weight: WT 24–32 g, MitoPark 23–32 g), 6 months (*n* = 12, body weight: WT 22–38 g, MitoPark 19–33 g), and 7 months (*n* = 10, body weight: WT 32–39 g, MitoPark 21–29 g).

Human α-syn A53T (α-syn A53T or A53T) transgenic line G2–3 (B6. Cg-*2310039L15Rik*^Tg(Prnp-SNCA*A53T)23Mkle^/J, Strain 006823; RRID:IMSR_JAX:006823) was acquired from the Jackson Laboratory. Male WT control mice and male α-syn A53T mice at 12 months of age were used (*n* = 8, body weight: WT 30–35 g, α-syn A53T 28–34 g). Given that estrogen exerts a protective effect on the pathological progression of PD (Thadathil et al., 2021), it is critical to account for its potential influence in experimental designs. Mice exhibit a gradual decline in estrogen production starting around 12 months of age, with circulating estrogen levels essentially depleted by 16 months (Aslam et al., 2012). This physiological pattern indicates that mice aged 16 months or older are minimally affected by estrogen. Thus, given the limited number of available mice, female 16-month-old α-syn A53T transgenic mice were included in the experiments (*n* = 12, three male and nine female; body weight: WT 28–39 g, α-syn A53T 24–35 g).

Genotyping was performed by PCR using tail DNA. Animals were housed in individually ventilated cages under controlled conditions (temperature: 22 ± 1°C; humidity: 55% ± 5%) with a 12/12-hour light/dark cycle (lights on at 7:00 a.m.). All experimental procedures were approved by the Institutional Animal Care and Use Committee of Shanghai Medical College, Fudan University (approval No. 20220228-133; approval date: February 28, 2022) and conducted in accordance with the Guide for the Care and Use of Laboratory Animals (National Research Council, 2011). An experimental timeline/flow chart detailing the procedures is included in **Additional Figure 1**.

### Establishment of 1-methyl-4-phenyl-1,2,3,6-tetrahydropyridine model

For the acute MPTP model, C57BL/6J mice received four intraperitoneal injections of 15 mg/kg MPTP-HCl (Merck, Kenilworth, NJ, USA) at 2-hour intervals on a single day (Jackson-Lewis and Przedborski, 2007; Meredith and Rademacher, 2011). For the subacute model, mice were injected with 20 mg/kg MPTP-HCl intraperitoneally once daily for 5 consecutive days. Control mice were administered an equivalent volume of normal saline (Santoro et al., 2023).

### Behavioral tests

#### Pole test

Mice were habituated to the testing environment for 2 hours prior to experimentation. They were placed head-up on a rough-surfaced wooden pole (1.5 cm in diameter, 50 cm in height). The turning time and time taken to climb down were recorded in three trials performed at 1-hour intervals, and the average value was taken as the result (Matsuura et al., 1997; Glajch et al., 2012).

#### Cylinder test

Individual mice were placed in a 9-cm-diameter, 20-cm-high transparent acrylic cylinder for 3 minutes. A blinded observer quantified rearing episodes, representing spontaneous exploratory behavior and sensorimotor integration (Han et al., 2020; Chen et al., 2023).

#### Wire hanging test

Mice were placed on a horizontal wire (2 mm in diameter × 50 cm in length) suspended between two elevated platforms (40 cm in height). The number of times each mouse successfully reached either platform or fell within 3 minutes was recorded. Upon reaching a platform or falling, the mouse was immediately repositioned at the wire’s midpoint. Reaching a platform earned a score of one point, while one point was deducted for each fall. The test was repeated three times, and the average score was calculated (Dorchies et al., 2013; Aartsma-Rus and van Putten, 2014; Zhang et al., 2024).

#### Y-maze food reward test

After 24 hours of food restriction, mice were placed in a modified Y-maze to assess olfactory function. The latency to locating hidden food in the maze (maximum 3 minutes) was recorded (Conrad et al., 1996; Roberge et al., 2008).

#### Open field test

An open field test was employed to assess locomotor activity (Walsh and Cummins, 1976). Mice were placed at the center of a 40 cm × 40 cm × 40 cm open field and allowed to explore for 5 minutes. Their activities, such as total distance traveled, average speed, frequency of entering the central area, and time spent in the central area, were recorded and analyzed using Noldus EthoVision XT software (Noldus, Wageningen, the Netherlands).

#### Rotarod test

Motor coordination and endurance were assessed using a rotarod apparatus (KW-6C Mouse Rotarod Apparatus, KEWBASIS, Nanjing, China). The speed was increased from 4 to 40 r/min over 300 seconds, with a total test duration of 300 seconds. Each mouse underwent three trials at 1-hour intervals, and the average fall time, distance, and speed were recorded (Kucinski et al., 2013, 2015; Leem et al., 2022).

#### Tail suspension test

Mice were suspended by adhesive tape 30 cm above a surface, and the duration of immobility was recorded over 6 minutes to evaluate depressive-like behaviors (Ishola et al., 2012).

### Mouse weight measurement

Body weight was measured using a calibrated electronic balance (ZC-DST, Beijing Zhecheng Technology Co., Ltd., Beijing, China). Mice were briefly placed in a clean container, allowed to settle for 5 seconds, and weighed. Measurements were taken at the same time each morning to minimize variability.

### Immunohistochemical and immunofluorescence staining

Mice were anesthetized by intraperitoneal injection with a Zoletil 50/xylazine cocktail (BN 998LA, Virbac Trading [Shanghai] Co., Ltd., Shanghai, China; 5 mg/kg tiletamine/zolazepam + 1.25 mg/kg xylazine dissolved in normal saline). After confirming successful anesthesia by toe pinch, transcardial perfusion was performed with ice-cold phosphate-buffered saline (PBS), followed by 4% paraformaldehyde fixation. The brains were post-fixed in 4% paraformaldehyde, dehydrated in sucrose solutions, and sectioned. For immunofluorescence staining, the sections were blocked with 5% bovine serum albumin in PBS for 1 hour at 23 ± 2°C, then incubated overnight at 4°C with primary antibodies. Following three washes with PBS (5 minutes each), the sections were incubated with Alexa Fluor–conjugated secondary antibodies (1:1000) for 1 hour at 23 ± 2°C in the dark. After washing with PBS, the nuclei were counterstained with 4′,6-diamidino-2-phenylindole (DAPI) for 5 minutes. Images were acquired using a FLUOVIEW FV3000 confocal microscope (Olympus, Tokyo, Japan) and analyzed with ImageJ (version 1.54f; National Institutes of Health, Bethesda, MD, USA) (Schneider et al., 2012).

For immunohistochemical staining, endogenous peroxidase was quenched with 0.3% hydrogen peroxide for 30 minutes. Antigen retrieval was performed via heat-induced epitope retrieval in citrate buffer (pH 6.0) at 95°C for 10 minutes (if required). After blocking, the sections were incubated with primary antibodies overnight at 4°C, washed thrice with PBS, and then incubated with biotin-conjugated secondary antibodies (1:200) for 1 hour at room temperature. The sections were then treated with VECTASTAIN Elite ABC Reagent (PK-4001 or PK-4002, Vector Laboratories, Newark, CA, USA) for 30 minutes. Signal development was performed using a DAB kit (SK-4100, Vector Laboratories) according to the manufacturer’s protocol. Images were captured using an Olympus BX53 microscope. Antibody details are provided in **Table 1**.

**Table 1:** Antibodies used in WB, IHC and IF staining

Antibody	Species	Dilution	Manufacturer	Catalog number	RRID number	Application
β-actin	Mouse	1:1000	Santa Cruz (Dallas, TX, USA)	sc-47778	AB_626632	WB
GAPDH	Rabbit	1:3000	Epizyme (Shanghai, China)	LF206	AB_3697174	WB
TH	Mouse	1:1000	IMMUNOSTAR (Hudson, WI, USA)	P22941	AB_572268	IF/IHC/WB
GFAP	Rabbit	1:1000	Proteintech (Rosemont, IL, USA)	16825-1-AP	AB_2109646	WB
GFAP	Rat	1:1000	Thermo Fisher Scientific (Waltham, MA USA)	13-0300	AB_2532994	IF
IBA1	Rabbit	1:1000	Abcam (Cambridge, UK)	ab178846	AB_2636859	IF/WB
Alpha-synuclein	Rabbit	1:10000	Abcam (Cambridge, UK)	ab138501	AB_2537217	WB
Alpha-synuclein	Rabbit	1:100	Abcam (Cambridge, UK)	ab138501	AB_2537217	IHC
Alpha-synuclein (pS129)	Rabbit	1:400	Abcam (Cambridge, UK)	ab51253	AB_869973	IHC
TFAM	Rabbit	1:100	Abcam (Cambridge, UK)	ab131607	AB_11154693	IF
Goat anti-Rabbit IgG (H+L) Alexa FluorTM Plus 488	Goat	1:1000	Thermo Fisher Scientific (Waltham, MA USA)	A32731	AB_2633280	IF
Goat anti-Mouse IgG (H+L) Alexa FluorTM Plus 647	Goat	1:1000	Thermo Fisher Scientific (Waltham, MA USA)	A32728	AB_2633277	IF
Cy^TM^3 AffiniPure^®^ Donkey Anti-Rat IgG (H+L)	N/A	1:1000	Jackson ImmunoResearch (West Grove, PA, USA)	712-165-153	AB_2340667	IF
IRDye^®^ 800CW Goat anti-Rabbit	Goat	1:20000	LICORbio (Lincoln, NE, USA)	926-32211	AB_621843	WB
IRDye^®^ 680RD Goat anti-Mouse	Goat	1:20000	LICORbio (Lincoln, NE, USA)	926-68070	AB_10956588	WB

GAPDH: Glyceraldehyde-3-phosphate dehydrogenase; GFAP: glial fibrillary acidic protein; IBA1: ionized calcium-binding adapter molecule 1; IF: immunofluorescence; IHC: Immunohistochemistry; TFAM: transcription factor A, mitochondrial; TH: tyrosine hydroxylase; WB: Western blot.

### Thioflavin S staining

Thioflavin S staining was performed by incubating striatum sections in 0.1% Thioflavin S (1326-12-1, Merck) for 5–10 minutes, differentiating in 80% ethanol (10–20 seconds), then mounting for fluorescence microscopy (excitation **~**440 nm/emission **~**521 nm). All steps were performed in a light-protected manner. This method specifically labels amyloid deposits, including senile plaques and α-syn aggregates (Christensen and Pike, 2020).

### Quantification of dopaminergic neurons and glial cells in the substantia nigra

Immunohistochemically labeled dopaminergic neurons (tyrosine hydroxylase–positive, TH^+^) in the SNc were quantified using the Stereo Investigator system (MBF Bioscience, Williston, VT, USA) with an Olympus microscope. One section out of every four 30 μm-thick slices was selected, with a total of six sections collected from the bregma range of –2.54 to –3.88 mm (Stutz et al., 2019). TH^+^ neurons were counted in the SNc (double-blinded analysis).

Immunofluorescently labeled glial cells and TH^+^ neurons in the SNc were quantified in four coronal sections per animal. The boundaries of the SNc were defined by TH immunofluorescence signals. Glial cells were identified by staining with glial fibrillary acidic protein (GFAP) for astrocytes or ionized calcium-binding adapter molecule 1 (IBA1) for microglia. The fluorescently labeled positive cells within the TH-demarcated SNc were counted in a double-blind manner using ImageJ software with the Cell Counter plugin. Cell density was defined as the number of positive glial cells divided by the area of the SNc in mm^2^, and the final value represented the mean density calculated across all analyzed sections.

### Western blot assay

Striatal tissues were lysed in radioimmunoprecipitation assay buffer (89900, Thermo Fisher Scientific, Waltham, MA, USA) supplemented with protease inhibitor cocktail (78440, Thermo Fisher Scientific). Following centrifugation (12,000 × *g*, 15 minutes, 4°C), the protein concentration was determined using a bicinchoninic acid protein assay kit (23225, Thermo Fisher Scientific). Equal protein amounts (30 μg) were denatured at 95°C for 5 minutes, separated by sodium dodecyl sulfate–polyacrylamide gel electrophoresis using a CFAS Any KD PAGE gel (PE008, ZHHC, Xi’an, China) at 170 V for 70 minutes, and transferred to PVDF membranes (IPFL00005 or ISEQ00010, MilliporeSigma, Burlington, MA, USA) at 100 V for 45 minutes with ice cooling. The membranes were blocked with 5% skim milk in Tris-buffered saline with Tween 20 for 1 hour, incubated with primary antibodies overnight at 4°C, washed with Tris-buffered saline with Tween 20, probed with IRDye-conjugated secondary antibodies for 1 hour at 23 ± 2°C, and imaged using an Odyssey CLx infrared imaging system (LI-COR Biosciences, Lincoln, NE, USA). Protein levels were normalized to β-actin or glyceraldehyde 3-phosphate dehydrogenase and analyzed using ImageJ software. Antibody details are provided in **Table 1**.

### Quantitative reverse transcription-polymerase chain reaction

Total RNA from the mouse striatum was extracted with Total RNA Isolation Reagent (YY101, Epizyme, Shanghai, China), and its concentration was measured using a BioTek Microplate Reader (Agilent Technologies, Santa Clara, CA, USA). Next, 1 µg of RNA was reverse-transcribed to complementary DNA using HiFiScript gDNA Removal RT MasterMix (CW 2020M, CWBIO, Taizhou, China) according to the manufacturer’s instructions. Relative mRNA levels were detected by quantitative reverse transcription-polymerase chain reaction (qRT-PCR) on a Mastercycler ep realplex (Eppendorf, Hamburg, Germany). Relative gene expression was calculated using the 2^–∆∆Ct^ method (Livak and Schmittgen, 2001) and normalized to β*-actin*. The primer sequences are listed in **Table 2**.

**Table 2:** The primer sequences for polymerase chain reaction

Gene	Primer sequence (5'–3')
*Slc1a5*	Forward: TCC TGG TCA CCA CAC TGC TC
	Reverse: GAA GGC AGC AGA CAC CAG ATT G
*Flt3*	Forward: GTC AGT AAT GAT TCT TGA GAC
	Reverse: GAG CTG CAC TTG CAG GGT GAT G
*Lcn2*	Forward: GCA GGT GGT ACG TTG TGG G
	Reverse: CTC TTG TAG CTC ATA GAT GGT GC
*Prnp*	Forward: CTG CCT TCC TAG TGG TAC CAG TC
	Reverse: CCA ACT ACC ACC ATG AGG TTG
*NOS2*	Forward: GGA CAA GCT GCA TGT GAC ATC G
	Reverse: GGA GCC ATA ATA CTG GTT GAT G
*Drd4*	Forward: GAA CTC GCT CGT GTG CGT GAG
	Reverse: GAT GGC GCA CAG GTT GAA GAT G
*Hspb1*	Forward: GAT GAG TGG TCG CAG TGG TTC
	Reverse: CTT CGT GCT TGC CAG TGA TCT C
*IL17a*	Forward: CCG TTC CAC GTC ACC CTG GAC
	Reverse: GGT CCA GCT TTC CCT CCG CAT TG
*IL17ra*	Forward: GGT GGG ATC TGT CAT CGT GC
	Reverse: GAC CTT CCT GGG CCT CAG GG
*Ndufaf3*	Forward: CCT AGG ATA GAG ATT GTT GTG
	Reverse: GAA GCC AGT GCA GTC TCT CCA G
*β-actin*	Forward: CAG GAT GCA GAA GGA GAT TAC
	Reverse: AAC GCA GCT CAG TAA CAG TC

### RNA sequencing and proteomic analysis

Libraries were prepared from 1 µg total RNA isolated from striatal brain tissue and sequenced (2 × 150 bp paired-end, Illumina Nova-seq, San Diego, CA, USA). Adapters and low-quality reads were trimmed (Cutadapt, https://cutadapt.readthedocs.io/en/stable/; Martin, 2011), and reads were aligned to the mouse genome (Hisat2, https://daehwankimlab.github.io/hisat2/). Genes were annotated using the GENCODE database (https://www.gencodegenes.org/mouse/).

For principal component analysis (PCA), FPKM values (RNA-seq) and normalized protein expression (mass spectrometry) were analyzed in R (version 4.2.1; https://www.r-project.org). Differential expression (DESeq2, https://bioconductor.org/packages/release/bioc/html/DESeq2.html) was defined as *P* < 0.05 and |log_2_fold-change (FC)| > 0.58. Gene Ontology (GO) and Kyoto Encyclopedia of Genes and Genomes (KEGG) pathway analyses were performed using clusterProfiler (https://guangchuangyu.github.io/software/clusterProfiler/; Yu et al., 2012) and ClueGO (https://apps.cytoscape.org/apps/cluego; Bindea et al., 2009), respectively.

Xiantao Platform (https://www.xiantaozi.com), a web-based bioinformatics tool for omics data analysis, was used to perform integrated analysis of RNA-seq data. Statistical analyses were conducted utilizing R, and data were visualized by PCA, volcano plots, and GO/KEGG enrichment lollipop plots generated with the ‘ggplot2’ package (version 3.3.6, https://ggplot2.tidyverse.org; Wickham, 2016). Heatmaps were created using the ‘ComplexHeatmap’ package (version 2.13.1, https://github.com/jokergoo/ComplexHeatmap; Gu et al., 2016), and gene set enrichment analysis (GSEA) bubble plots, which depicted pathway enrichment statistics, were visualized with the ‘ggplot2’ package (version 3.4.4). To illustrate KEGG pathway networks, Cytoscape (version 3.10.3, https://cytoscape.org; Shannon et al., 2003) was utilized, operating within the Java Environment: Java 17.0.5 (Eclipse Adoptium).

Striatal proteins were extracted and analyzed by LC-MS/MS (GENEWIZ, Suzhou, China). Differentially expressed proteins (DEPs) were identified (*P* < 0.05, |log_2_FC| > 0.25). The RNA-seq results were visualized with a protein–protein interaction plot generated using the Cytoscape package ‘stringApp’ (version 2.2.0, https://apps.cytoscape.org/apps/stringapp; Doncheva et al., 2019).

### Postmortem transcriptomic data from patients with Parkinson’s disease

Transcriptomic data from the caudate nucleus and putamen of postmortem patients with PD (*n* = 35) and neurologically healthy controls (*n* = 40) were sourced from the publicly available dataset GSE205450 (Irmady et al., 2023). This dataset, deposited in the Gene Expression Omnibus (GEO) database, comprises bulk RNA-seq data derived from PD and control striatal tissues. Clinical metadata, including disease duration, motor/cognitive complications, and age at onset, were extracted from the Supplementary Data 1 from Irmady et al. (2023). All human data in this study were obtained from these previously generated and annotated datasets.

### Statistical analysis

Sample size was determined on the basis of the literature (González-Rodríguez et al., 2021; Zhang et al., 2022). No animals were excluded, because of factors such as death, failure to meet criteria, or incomplete data. All mice were ultimately included in the statistical analysis. Statistical analyses were performed by an investigator blinded to the group assignments. All statistical analyses were performed using GraphPad Prism version 9.5.0 for Windows (GraphPad Software, Boston, MA, USA, www.graphpad.com), with data expressed as mean ± standard error of the mean (SEM). Before intergroup comparisons, normality (Shapiro–Wilk test) and homogeneity of variance (Levene’s test) assumptions were verified. Unpaired Student’s *t*-test with Welch’s correction was used as appropriate. Statistical significance was defined as *P* < 0.05 for all analyses.

## Results

### Behavioral comparisons of the three distinct Parkinson’s disease mouse models

**Figure 1:**
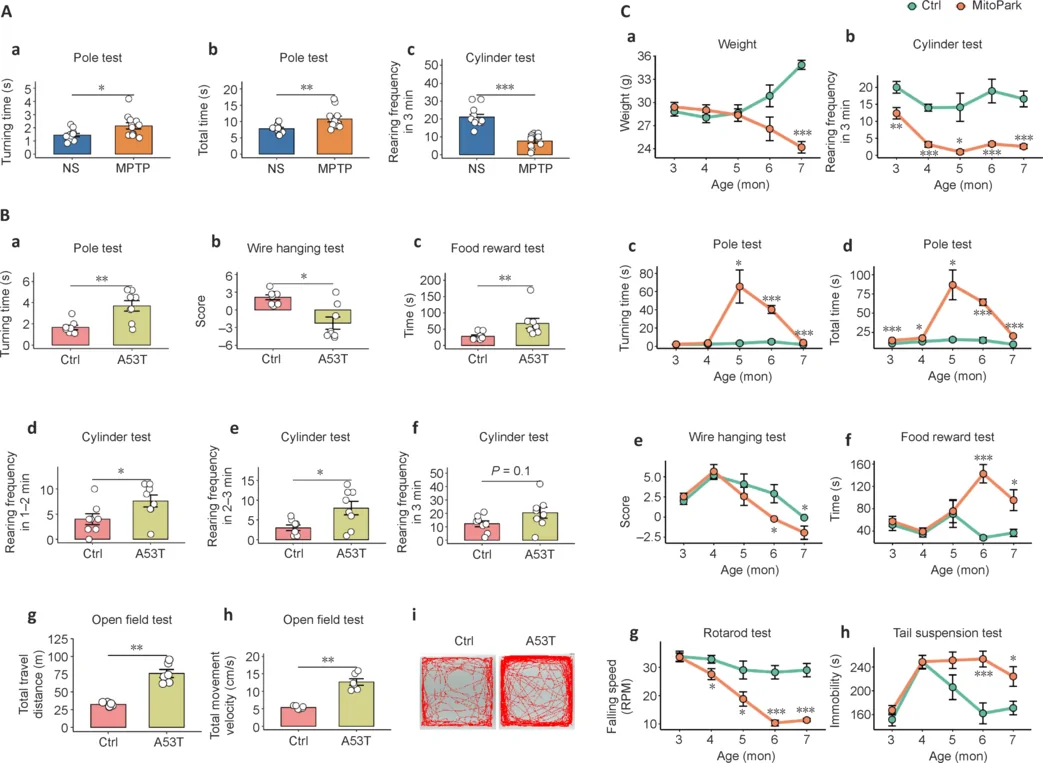
Motor behavior impairments in the three PD mouse models. (A) Results from the behavioral tests in mice 3 days after subacute MPTP administration. (a, b) Turning time (a) and total time (b) in the pole test. (c) Rearing times within 3 minutes in the cylinder test (*n* = 12). (B) Results from the behavioral tests in 12-month-old α-syn A53T mice (A53T). (a) Turning time in the pole test. (b) Scores in the wire hanging test. (c) Time taken to locate food pellets in the Y-maze after fasting. (d–f) Rearing times from 1 to 2 minutes (d), from 2 to 3 minutes (e), and during the entire 3-minute period (f) in the cylinder test. (g–i) Results from the open field test. The total travel distance (g), total movement velocity (h), and representative trajectory plots (i) are shown (*n* = 8). For all data in Aa–c, and Ba–i, unpaired *t*-test with Welch’s correction was used. (C) Body weight and results from the behavioral tests in 3-, 4-, 5-, 6-, and 7-month-old MitoPark mice (*n* = 7–12). (a) Body weight. (b) Rearing times within 3 minutes in the cylinder test. (c, d) Turning time (c) and total time (d) in the pole test. (e) Scores in the wire hanging test. (f) Time taken to locate food pellets in the Y-maze after fasting. (g) Rod speed at which the mice fell in the rotarod test. (h) Immobile time in the tail suspension test. Data are expressed as mean ± SEM. *P < 0.05, **P < 0.01, ***P < 0.001. All data in Ca–h were analyzed by unpaired *t*-test (Welch correction). Ctrl: Control; MPTP: 1-methyl-4-phenyl-1,2,3,6-tetrahydropyridine; PD: Parkinson’s disease; α-syn: α-synuclein.

To explore behavioral changes in PD model mice, we conducted multiple tests and observed distinct differences. Acute and subacute MPTP regimens are standard protocols for modeling PD, with dopaminergic neuron damage stabilizing at 3–7 days post-injection (Przedborski et al., 2001). Thus, we performed behavioral assessments at this time point. In subacute MPTP-treated adult male C57BL/6 mice, the turning (**Figure 1Aa**) and total climbing (**Figure 1Ab**) times in the pole test were significantly longer than those in the pole test in the normal saline-treated controls at 3 days post-injection. In the cylinder test, PD mice reared significantly fewer times within 3 minutes compared with the normal saline-treated controls (**Figure 1Ac**). However, no significant differences were found between the normal saline- and MPTP-injected groups in the wire hanging test, the Y-maze food reward test, the rotarod test, or body weight (data not shown). These results indicate that MPTP injection can quickly induce motor impairments in mice. We previously reported similar acute MPTP-induced impairments (Zhang et al., 2024), and thus do not elaborate on them here.

According to Jackson Laboratory information (The Jackson Laboratory, 2002), α-syn A53T (G2–3) heterozygous mice develop PD between 9 and 16 months (mean 13 months), and thus we made our observations at 12 and 16 months (Lee et al., 2002). Compared with wild-type (WT) control mice, 12-month-old A53T mice exhibited a significantly longer turning time in the pole test (**Figure 1Ba**), although there was no difference in total time (data not shown). In the wire hanging test, A53T mice had lower scores (**Figure 1Bb**), and after a 24-hour fast, they took considerably longer to find food in the Y-maze compared with WT mice (**Figure 1Bc**). These findings suggest a decline in motor function and possible olfactory impairment. Unlike MPTP-induced PD mice, in the cylinder test, 12-month-old A53T mice reared more often in the 1- to 2- and 2- to 3-minute intervals compared with WT mice, although the total standing time in 3 minutes remained unchanged (**Figure 1Bd–f**). In the open field test, A53T mice demonstrated greater total movement distance and velocity compared with WT mice (**Figure 1Bg–i**), indicating hyperactivity in a new environment. In the accelerating rotarod test, there were no differences between genotypes (**Additional Figure 2**). Additionally, 16-month-old A53T mice weighed significantly less than WT mice (**Additional Figure 3A**). Similar to the 12-month-old group, they also had lower scores in the wire hanging test (**Additional Figure 3B**) and reared more often in the cylinder test compared with WT mice (**Additional Figure 3C**). No differences were found in the accelerating rotarod and tail suspension tests (data not shown).

The MitoPark model exhibited significant behavioral impairments from 3 months of age, with 5- and 7-month time points defined as mid- and late-stage PD (Galter et al., 2010; Beckstead and Howell, 2021). Immunofluorescence analysis confirmed TFAM deficiency in the dopaminergic neurons of MitoPark mice (**Additional Figure 4**). We assessed male MitoPark and wild-type mice at 3, 4, 5, 6, and 7 months. MitoPark mice showed a plateau in body weight followed by a decline from 5 months, with a significant drop at 7 months (**Figure 1Ca**). In the cylinder test, 3- to 7-month-old MitoPark mice reared less often compared with WT mice (**Figure 1Cb**). In the pole test, 5- to 7-month-old mice exhibited longer turning times (**Figure 1Cc**), while 3- to 7-month-old mice exhibited increased total climbing times compared with WT mice (**Figure 1Cd**). In the wire hanging test, the 6- and 7-month-old mice performed worse compared with WT mice (**Figure 1Ce**). In the Y-maze test, after fasting, 6- and 7-month-old mice took longer to find food (**Figure 1Cf**). In the rotarod test, 4- to 7-month-old mice fell at lower rotation speeds compared with WT mice (**Figure 1Cg**). In the tail suspension test, the 6- and 7-month-old mice exhibited longer immobility times compared with WT mice (**Figure 1Ch**). The behavioral results of the three PD models are summarized in **Additional Table 1**.

**Additional Table 1:** Comparison of behavioral performance among the three PD model mice

		Subacute MPTP model (3 d)	A53T	MitoPark
Body weight		-	16 mon↓	7 mon↓↓↓
Pole test				
	Turning time	↑	12 mon↑↑	5 mon↑
	Total time	↑↑	-	3 mon↑↑↑
Rearing test in 3 min			16 mon↑	3 mon↓↓
Wire hanging test		-	16 mon↓	6 mon↓
Y-maze food reward test		-	12 mon↑↑	6 mon↑↑↑
Rotarod test		-	-	4 mon↓
Tail suspension test		-	-	6 mon↑↑↑

"-" indicates no significant difference; "↓" indicates decrease compared with the control group; "↑" indicates increase compared with the control group. One, two, and three arrows represent *P* < 0.05, *P* < 0.01, and *P* < 0.001, respectively. For all data, the unpaired *t*-test (Welch correction) was used. A53T: α-Synuclein A53T mutant; MitoPark: MitoPark transgenic mouse model (a Parkinson’s disease model).

### Nigrostriatal pathway pathology in the three Parkinson’s disease mouse models

Nigrostriatal injury is a key manifestation of PD pathology (Blesa et al., 2011). We used immunofluorescence staining to label and quantify dopaminergic neurons (TH^+^), astrocytes (GFAP^+^), and microglia (IBA1^+^) in the substantia nigra and western blot to detect TH, GFAP, and IBA1 protein levels in the striatum. The three PD models showed different features of nigrostriatal pathway damage.

**Figure 2:**
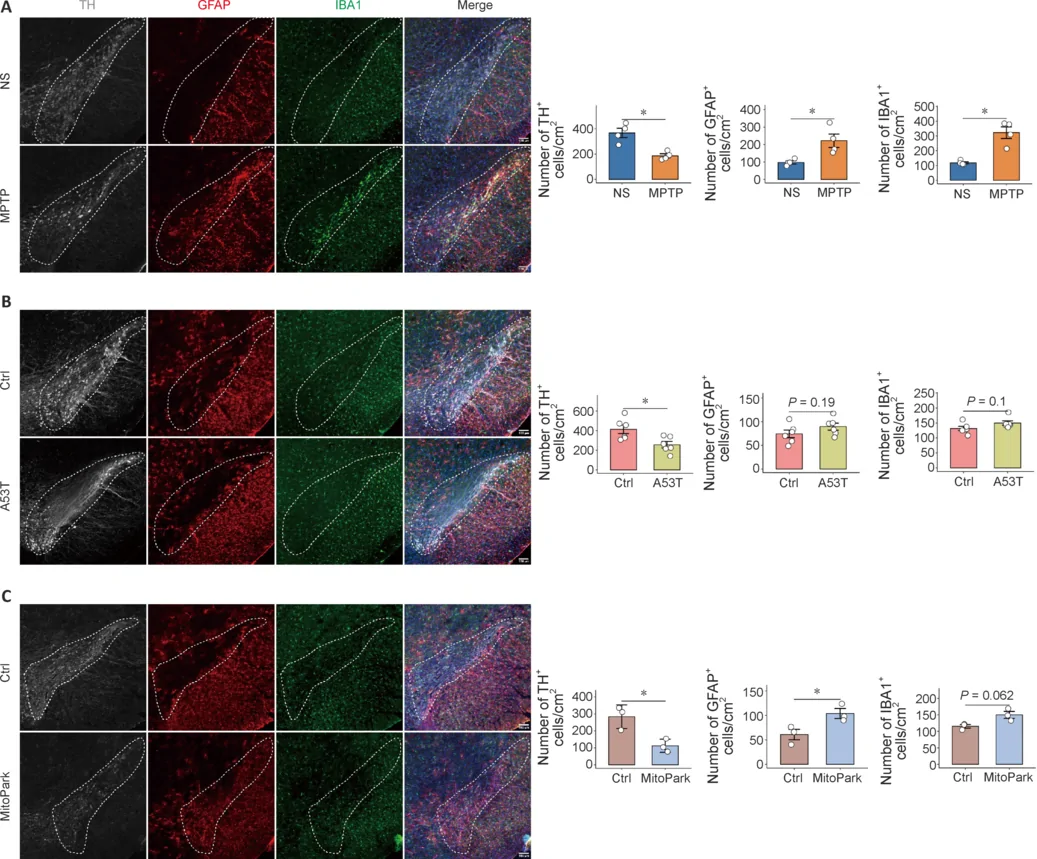
Differences in nigral dopaminergic neuron loss and glial activation among the three PD mouse models. (A–C) Immunofluorescence staining showing fewer TH^+^ dopaminergic neurons (labeled with Alexa Fluor^TM^ Plus 647, gray) and more GFAP^+^ astrocytes (labeled with Cy^TM^3, red) and IBA1^+^ microglia (labeled with Alexa Fluor^TM^ Plus 488, green) in the substantia nigra of PD models *versus* controls. Notably, the A53T group was an exception to this pattern because no significant increase was observed in the number of GFAP^+^ astrocytes. DAPI (blue) was used to label cell nuclei. (A) Cell staining and counting in mice 3 days after NS or subacute MPTP administration (*n* = 4). (B) Cell staining and counting in 16-month-old WT and A53T mice (*n* = 6). (C) Cell staining and counting in 5-month-old WT and MitoPark mice (*n* = 3). The circled area indicates the SNc. Scale bars: 100 μm. Data are expressed as mean ± SEM. *P < 0.05. For all data in A–C, unpaired *t*-test with Welch’s correction was used. A53T: α-Synuclein A53T mutant; Ctrl: control; DAPI: 4′,6-diamidino-2-phenylindole; GFAP: glial fibrillary acidic protein; IBA1: ionized calcium-binding adapter molecule 1; MPTP: 1-methyl-4-phenyl-1,2,3,6-tetrahydropyridine; MitoPark: MitoPark transgenic mouse model; NS: normal saline; PD: Parkinson’s disease; SNc: substantia nigra pars compacta; TH: tyrosine hydroxylase; WT: wild-type.

**Figure 3:**
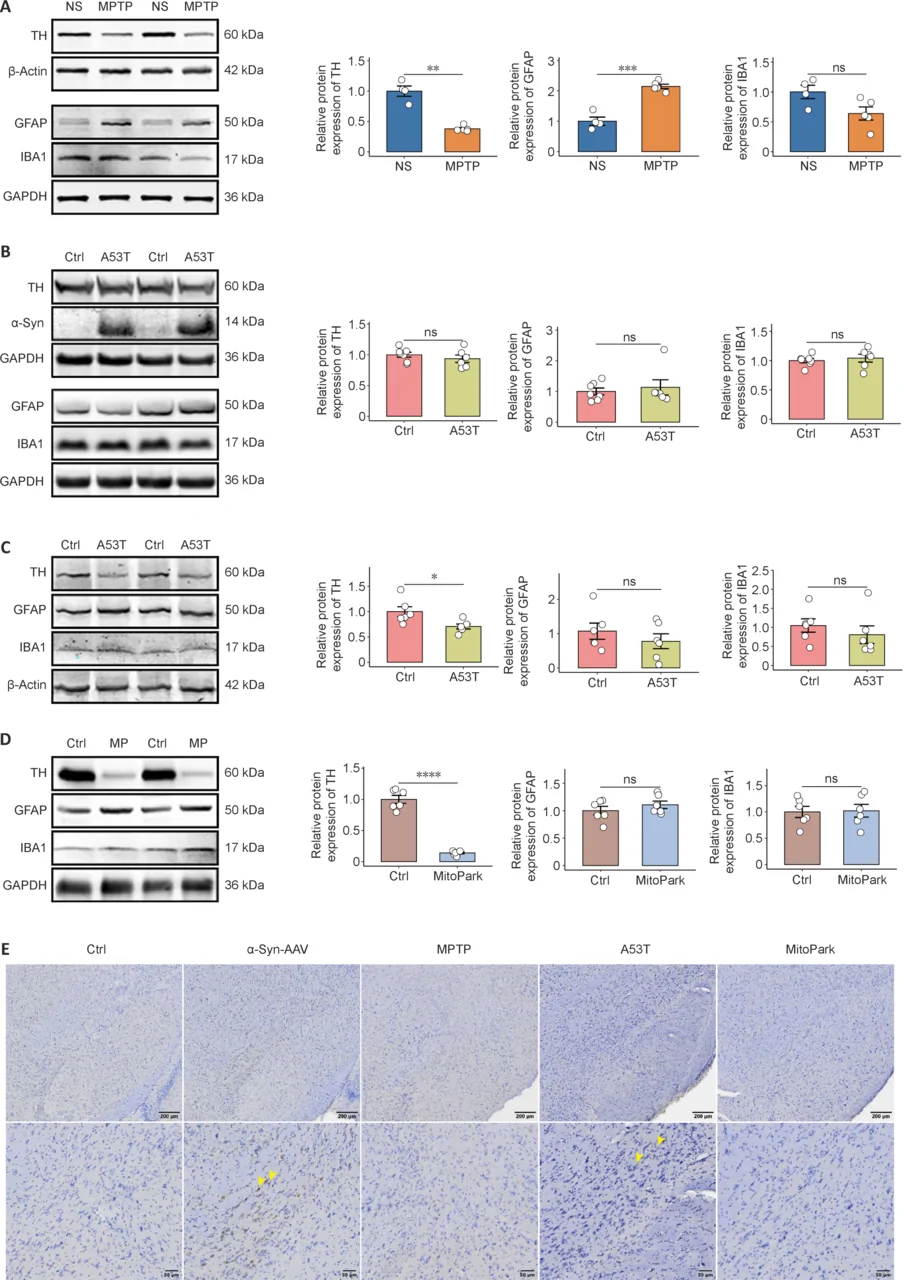
Differences in striatal PD-related protein expression and nigral pS129-α-syn in PD mouse models. (A–D) Western blot analysis of TH, GFAP, and IBA1 expression in the mouse striatum. (A) TH, GFAP, and IBA1 expression 3 days after subacute MPTP administration (*n* = 4). (B) TH, GFAP, α-syn, and IBA1 expression in 12-month-old A53T mice (*n* = 6 or 7). (C, D) TH, GFAP, and IBA1 expression in 16-month-old A53T mice (C) and 7-month-old MitoPark mice (D) (*n* = 6). Data are expressed as mean ± SEM. *P < 0.05, **P < 0.01, ***P < 0.001, ****P < 0.0001. For all of the data presented in A–D, unpaired *t*-test (with Welch’s correction) was used. (E) α-Syn pS129 staining in the substantia nigra of PD model mice. Immunohistochemistry combined with hematoxylin staining shows pS129-α-syn expression and aggregation in the substantia nigra of different mouse models (upper: original magnification 4×, scale bar: 200 μm; lower: scale bar: 50 μm). The positive control mice (α-syn-AAV–injected) showed some cytoplasmic pS129-α-syn–positive signals due to overexpression; α-syn A53T mice exhibited a few pS129-α-syn-positive cells; no pS129-α-syn was detected in MPTP-treated or MitoPark mice. Yellow arrows indicate pS129-α-syn–positive signals. AAV: Adeno-associated virus; A53T: α-synuclein A53T mutant; Ctrl: control; DEG: differentially expressed gene; GAPDH: glyceraldehyde-3-phosphate dehydrogenase; GFAP: glial fibrillary acidic protein; IBA1: ionized calcium-binding adapter molecule 1; MP: MitoPark; MPTP: 1-methyl-4-phenyl-1,2,3,6-tetrahydropyridine; MitoPark: MitoPark transgenic mouse model; NS: normal saline; ns: not significant; PD: Parkinson’s disease; pS129-α-syn: phosphorylated α-synuclein at Ser129; TH: tyrosine hydroxylase; WB: western blot; α-syn: α-synuclein.

In MPTP-induced PD mice, there was a significant reduction in the number of TH^+^ neurons in the SNc and in striatal TH protein levels at 3 days after subacute administration or 7 days after acute administration compared with the normal saline-treated controls. The numbers of GFAP^+^ astrocytes and IBA1^+^ microglia in the SNc were increased compared with the normal saline-treated controls, and astrocytes and microglia showed morphology consistent with activation. Moreover, striatal GFAP protein levels were increased, whereas IBA1 levels were unchanged compared with the normal saline-treated controls (**Figures 2A**, **3A** and **Additional Figure 5**). These findings indicate that MPTP intoxication damages the dopaminergic system and triggers neuroinflammation.

In 12-month-old A53T mice, there were no significant changes in the numbers and morphologies of TH^+^ neurons, GFAP^+^ astrocytes, and IBA1^+^ microglia in the substantia nigra (data not shown). At 16 months, the number of TH^+^ neurons in the substantia nigra decreased significantly, but the numbers of astrocytes and microglia showed no obvious alterations compared with WT mice (**Figure 2B**). In the striatum of 12-month-old A53T mice, the expression levels of TH, GFAP, and IBA1 remained relatively stable, while human α-syn was overexpressed (**Figure 3B**). Notably, at 16 months, striatal TH protein levels declined significantly, but GFAP and IBA1 levels remained unchanged compared with WT mice (**Figure 3C**). These findings indicate that the α-syn A53T transgene has minimal effects on the nigrostriatal pathway at 12 months, while dopaminergic neuron damage progresses with aging.

In 5-month-old MitoPark mice, the number of TH^+^ neurons in the substantia nigra decreased significantly, and astrocytes increased and were activated compared with WT mice (**Figure 2C**). In 7-month-old mice, striatal TH protein levels decreased, while GFAP and IBA1 levels were unchanged compared with WT mice (**Figure 3D**). Immunohistology staining and cell counting showed that 12.1% of dopaminergic neurons were preserved in 7-month-old MitoPark mice compared with controls (**Additional Figure 6**). Thus, in this model, dopaminergic neuron damage and neuroinflammation were evident in the substantia nigra. **Additional Table 2** summarizes the changes seen in the nigrostriatal pathway in these three models.

**Additional Table 2:** Comparison of nigrostriatal pathway damage among the three PD models

PD models		TH	GFAP	Iba1
Subacute MPTP model (3 d)	Str	↓↓	↑↑↑	-
	SNc	↓	↑	↑
A53T (16 mon)	Str	↓	-	-
	SNc	↓	-	-
MitoPark (5 mon)	Str	↓↓↓↓	-	-
	SNc	↓	↑	-

"↓" indicates significant decrease vs. control; "↑" indicates significant increase vs. control; "-": no significant difference. One, two, three, and four arrows represent *P* < 0.05, *P* < 0.01, *P* < 0.001, and *P* < 0.0001, respectively. For all data, the unpaired *t*-test (Welch correction) was used. A53T: α-Synuclein A53T mutant; GFAP: glial fibrillary acidic protein; IBA1: ionized calcium-binding adapter molecule 1; MitoPark: MitoPark transgenic mouse model (a Parkinson’s disease model); MPTP: 1-methyl-4-phenyl-1,2,3,6-tetrahydropyridine; PD: Parkinson’s disease; SNc: substantia nigra pars compacta; Str: striatum; TH: tyrosine hydroxylase.

### No obvious accumulation of pathological α-synuclein is observed in the three Parkinson’s disease mouse models

α-Syn aggregation is an important pathological feature of PD (Srinivasan et al., 2021). Ser129-phosphorylated α-syn (pS129-α-syn) is crucial for enhancing α-syn aggregation and toxicity (Dutta et al., 2023; Hu et al., 2024). Immunohistochemistry was utilized to label α-syn and pS129-α-syn in the substantia nigra and striatum of the three PD mouse models. No obvious α-syn aggregates were detected in these regions, as assessed by thioflavin S staining (data not shown). The positive control mice, in which AAV-α-syn-A53T injection led to α-syn A53T overexpression in the substantia nigra, displayed numerous pS129-α-syn–positive cytoplasmic signals. α-Syn A53T transgenic mice exhibited a few pS129-α-syn–positive cells in the SN, while MPTP-treated and MitoPark mice showed no detectable pS129-α-syn (**Figure 3E**). No pS129-α-syn labeling was observed in the striatum of any of the three mouse types (data not shown).

### Differentially expressed genes in the striatum of three Parkinson’s disease mouse models

**Figure 4:**
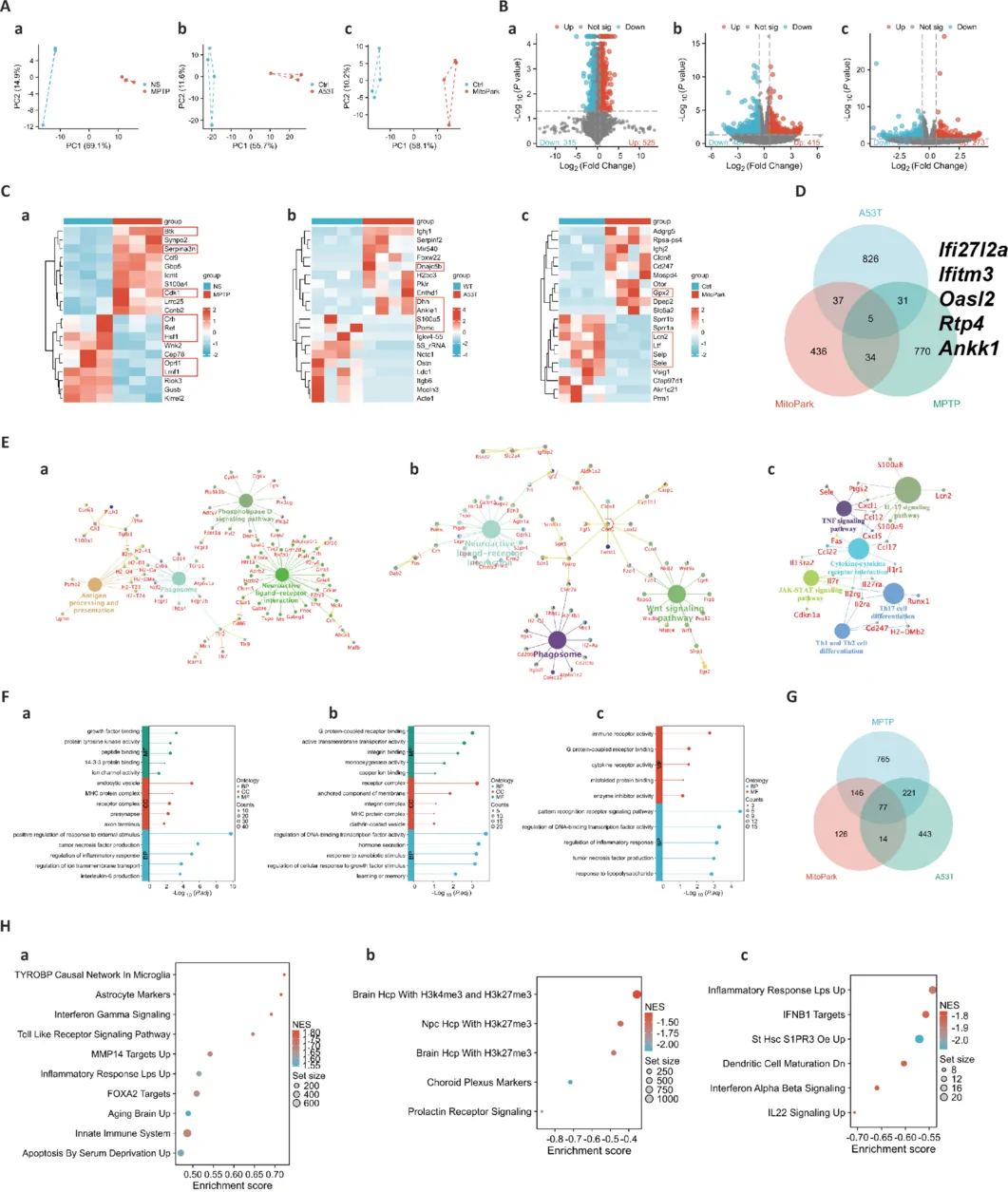
Comprehensive transcriptomic analysis of the striatum in the three PD mouse models. (A) Principal component analysis (model groups: red; NS/WT controls: blue). (B) Volcano plots of DEGs (downregulated: blue; upregulated: red). (C) Heatmap of the top 10 up-/downregulated genes (PD-related genes are marked with red boxes). (D) Shared DEGs among the three PD mouse models. (E, F) Enrichment analyses: (E) KEGG pathway networks, (F) GO term bar graphs. (G) Results from intersection analysis of the GO enrichment results from the three PD models. (H) GSEA of biological pathways. Subpanels (a–c) represent the (a) acute MPTP, (b) 16-month-old A53T, and (c) 5-month-old MitoPark models in A–C, E, F, and H. A53T: α-Synuclein A53T mutant; Ctrl: control; DEG: differentially expressed gene; GSEA: gene set enrichment analysis; GO: Gene Ontology; KEGG: Kyoto encyclopedia of genes and genomes; MPTP: 1-methyl-4-phenyl-1,2,3,6-tetrahydropyridine; MitoPark: MitoPark transgenic mouse model; NES: normalized enrichment score; PC: principal component; PCA: principal component analysis; PD: Parkinson’s disease.

To detect molecular differences in three PD mouse models, we used RNA-seq to analyze striatal gene expression profiles. PCA indicated significant alterations in striatal gene expression in all three PD models compared with their respective controls (**Figure 4A**, and **Additional Figure 7Aa** and **Ba**).

Differentially expressed gene (DEG) analysis showed substantial gene expression changes in each model. In the acute MPTP model, 525 genes were upregulated, and 315 genes were downregulated (**Figure 4Ba**). The subacute MPTP model exhibited 243 upregulated and 414 downregulated DEGs (**Additional Figure 7Ab**). The 12-month-old A53T model exhibited 155 upregulated and 215 downregulated DEGs (**Additional Figure 7Bb**), whereas the 16-month-old A53T model exhibited 415 upregulated and 484 downregulated DEGs (**Figure 4Bb**). The 5-month-old MitoPark model exhibited 273 upregulated and 239 downregulated DEGs (**Figure 4Bc**).

On the basis of FC, we identified the top 10 upregulated and downregulated genes and used them to generate a heatmap (**Figure 4C**). In the acute MPTP model, *Btk*, *Serpina3n*, and *Cdk1* were upregulated, and *Crh*, *Ret*, *Hsf1*, *Oprl1*, and *Lmf1* were downregulated (**Figure 4Ca**). In the subacute MPTP model, *Rab44* and *Esrrb* were among the top 10 downregulated genes (**Additional Figure 7Ac**). In the 12-month-old A53T model, *Nnmt*, *Ghsr*, and *Prnp* were upregulated, and *Muc19* was downregulated (**Additional Figure 7Bc**). In the 16-month-old A53T model, *Dnajc5b*, *Dhh*, and *Ankle1* were upregulated, and *S100a5* and *POMC* were downregulated (**Figure 4Cb**). In the 5-month-old MitoPark model, *Gpx2* was upregulated, and *Lcn2*, *Ltf*, *Selp*, and *Sele* were downregulated (**Figure 4Cc**). Intersection analysis of the acute MPTP, 16-month-old A53T, and 5-month-old MitoPark models identified five genes––*Ifi27l2a*, *Ifitm3*, *Oasl2*, *Rtp4*, and *Ankk1*––whose expression levels were significantly altered in all three models (**Figure 4D**), suggesting their potential significance in PD pathology. Detailed information regarding the DEGs identified by the intersection analysis is shown in **Additional Table 3**.

Next, GO and KEGG enrichment analyses were performed to identify PD-related pathways. In the acute MPTP model, phagosome, neuroactive ligand-receptor interaction and chemokine signaling pathways were enriched (**Figure 4Ea** and **Fa**). The 16-month-old A53T model showed enrichment of phagosome and Wnt signaling (**Figure 4Eb** and **Fb**). The 5-month-old MitoPark model exhibited enrichment of the interleukin 17 (IL17) and tumor necrosis factor (TNF) signaling pathways (KEGG) and GO terms such as immune receptor and cytokine receptor activity (**Figure 4Ec** and **Fc**). Detailed information regarding the GO and KEGG enrichment analyses is shown in **Additional Tables 4** and **5**.

Intersection analysis of the enriched KEGG and GO terms from the three models yielded 77 common pathways/functions, mainly related to the immune response, cytokine regulation, transcription factor regulation, receptor-related aspects, and gliogenesis (**Figure 4G** and **Additional Tables 4** and **5**). The overlap suggests that these pathways and functions play crucial roles in PD pathogenesis, and they exhibited common and model-specific molecular changes.

The GSEA shown in **Figure 4H** yielded model-specific functional profiles: acute MPTP mice exhibited significantly enriched immunity-inflammation, astrocyte-related, and apoptosis pathways; 16-month-old A53T mice exhibited dysregulated H3K27me3/H3K4me3 epigenetic and prolactin receptor signaling pathways; and 5-month-old MitoPark mice exhibited activated immune-inflammatory responses (**Additional Table 6**).

**Figure 5:**
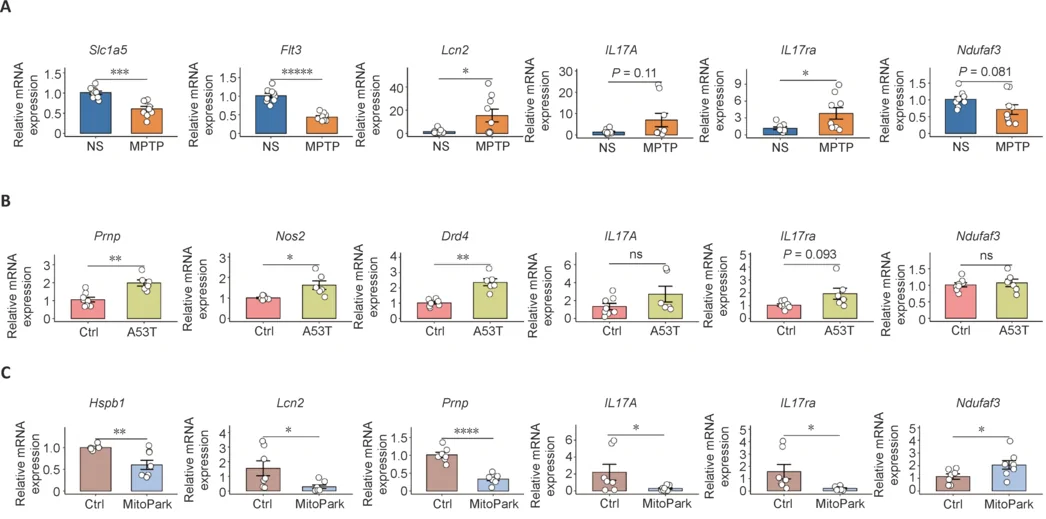
Ferroptosis pathway in the three PD mouse models. (A) *Slc1a5*, *Flt3*, *Lcn2*, *IL17A*, *IL17ra*, and *Ndufaf3* expression in the subacute MPTP model, as detected by qRT-PCR (*n* = 8). (B) *Prnp*, *Nos2*, *Drd4*, *IL17A*, *IL17ra*, and *Ndufaf3* expression in the 16-month-old A53T model (*n* = 6 or 8). (C) *Hspb1*, *Lcn2*, *Prnp*, *IL17A*, *IL17ra*, and *Ndufaf3* expression in the 5-month-old MitoPark model (*n* = 7 or 8). Data are expressed as mean ± SEM. *P < 0.05, **P < 0.01, ***P < 0.001, ****P < 0.0001. For all data in A–C, unpaired *t*-test (with Welch’s correction) was used. A53T: α-Synuclein A53T mutant; Ctrl: control; Flt3: FMS-like tyrosine kinase 3; Hspb1: heat shock protein beta-1; IL17A: interleukin-17A; IL17ra: interleukin-17 receptor A; Lcn2: lipocalin-2; MPTP: 1-methyl-4-phenyl-1,2,3,6-tetrahydropyridine; MitoPark: MitoPark transgenic mouse model; Ndufaf3: NADH dehydrogenase (ubiquinone) complex assembly factor 3; Nos2: nitric oxide synthase 2, inducible; ns: not significant; PD: Parkinson’s disease; Prnp: prion protein gene; qPCR: quantitative polymerase chain reaction; RT-qPCR: reverse transcription-quantitative polymerase chain reaction; Slc1a5: solute carrier family 1 member 5.

Each model exhibited altered expression of a set of genes associated with the ferroptosis pathway (**Additional Table 7**), consistent with the concept that ferroptosis contributes to dopaminergic neuron loss in PD. We selected some DEGs in the ferroptosis pathway and verified them by RT-qPCR. Given the IL17 pathway enrichment in the MitoPark model, we also verified Il17a and interleukin 17 receptor A (Il17ra) expression levels. The RT-qPCR results showed that, in the MPTP model, *Slc1a5* and *Flt3* were downregulated, and *Lcn2* and *Il17ra* were upregulated (**Figure 5A**). In the A53T model, *Prnp*, *Nos2*, and *Drd4* were upregulated (**Figure 5B**). In the MitoPark model, *Hspb1*, *Lcn2*, *Prnp*, *Il17a*, and *Il17ra* were downregulated, and *Ndufaf3* was upregulated (**Figure 5C**). These results suggest that there is a transcription-level link between the three PD models and the ferroptosis pathway, and that IL17 pathway is involved in PD pathogenesis in the MitoPark model.

**Additional Table 7:** DEGs involved in the ferroptosis pathway and their logFC values from RNA-seq analysis in the MPTP, A53T and MitoPark mouse models

Model	Gene	Log_2_FC	P value
	*Slc1a5*	-0.713	0.017
	*Heph*	-0.817	2.098 × 10^-4^
Subacute MPTP	*Hfe*	-0.907	0.024
	*Flt3*	-1.307	0.035
	*Steap2*	-1.753	0.020
	*Prnp*	2.867	2.436 × 10^-29^
	*Nnmt*	2.783	0.006
12 mon A53T	*Nos2*	1.923	0.028
	*Drd4*	1.899	0.043
	*Trib3*	0.717	0.031
	*Flt3*	-0.646	0.001
12 mon A53T	*Alox12b*	-0.750	0.016
16 mon A53T		-0.776	0.003
12 mon A53T	*Cybb*	-1.291	0.043
16 mon A53T		-0.542	0.085
	*Gpx2*	2.962	0.019
	*Tfr2*	0.800	0.014
	*Cdkn1a*	-0.758	0.002
5 mon MitoPark	*Ptgs2*	-0.817	0.001
	*Hspb1*	-0.937	0.006
	*Ltf*	-3.249	0.001
	*Lcn2*	-4.41	2.088 × 10^-22^

A53T: α-Synuclein A53T mutant; DEG: differentially expressed gene; FC: fold-change; MitoPark: MitoPark transgenic mouse model (a Parkinson’s disease model); MPTP: 1-methyl-4-phenyl-1,2,3,6-tetrahydropyridine; RNA-seq: RNA sequencing.

### Differentially expressed proteins in the striatum in the three Parkinson’s disease mouse models

**Figure 6:**
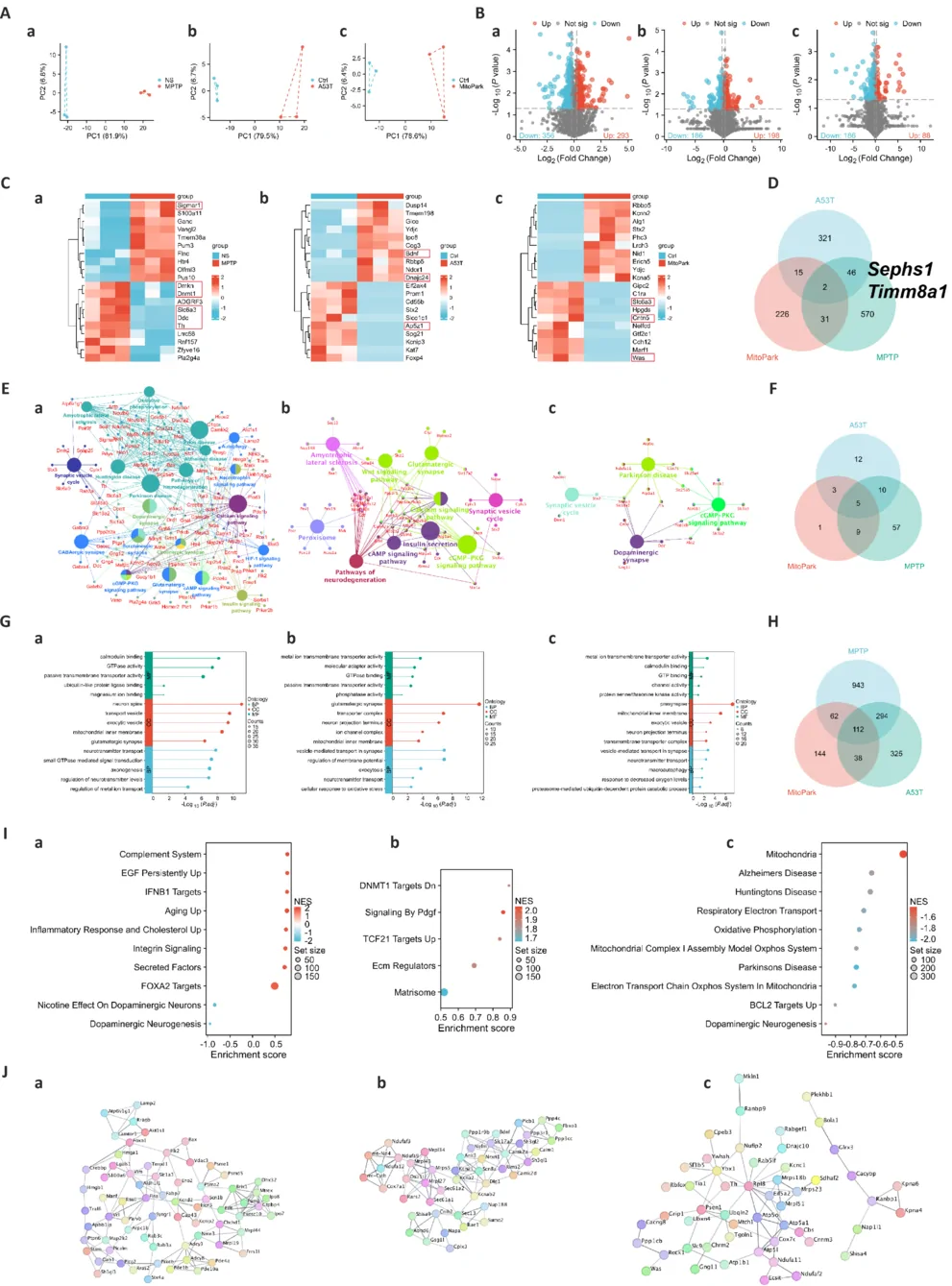
Comprehensive proteomic analysis of the striatum in the PD mouse models. (A) Principal component analysis (model groups: red; controls: blue). (B) Volcano plots of DEPs (downregulated: blue; upregulated: red). (C) Heatmap of the top 10 up-/downregulated proteins (PD-related proteins marked with red boxes). (D) Shared DEPs among the three PD mouse models. (E–J) Enrichment analyses: (E) KEGG pathway networks, (G) GO term bar graphs. Results from intersection analysis of the KEGG (F) and GO (H) enrichment results from the three PD models. (I) GSEA of biological pathways. (J) PPI networks of the DEPs. Subpanels (a–c) represent the (a) acute MPTP, (b) 16-month-old A53T, and (c) 5-month-old MitoPark models in A–C, E, G, I, and J. A53T: α-Synuclein A53T mutant; Ctrl: control; DEP: differentially expressed protein; GSEA: Gene set enrichment analysis; GO: Gene Ontology; KEGG: Kyoto Encyclopedia of Genes and Genomes; MPTP: 1-methyl-4-phenyl-1,2,3,6-tetrahydropyridine; MitoPark: MitoPark transgenic mouse model; PC: principal component; PCA: principal component analysis; PD: Parkinson’s disease; PPI: protein-protein interaction.

Next, we performed proteomic analysis of the striatal tissues of the PD model mice. The results showed significant protein expression differences between the model and control groups. PCA plots clearly separated the acute MPTP group from the normal saline control (**Figure 6Aa**), the 16-month-old A53T group from the WT control (**Figure 6Ab**), and the 5-month-old MitoPark group from the WT control (**Figure 6Ac**).

DEP analysis revealed substantial protein expression changes in each model. In the acute MPTP model, 293 proteins were upregulated, and 356 proteins were downregulated (**Figure 6Ba**). In the 16-month-old A53T model, 198 proteins were upregulated, and 186 proteins were downregulated (**Figure 6Bb**). In the 5-month-old MitoPark model, 88 proteins were upregulated, and 186 proteins were downregulated (**Figure 6Bc**).

A heatmap of the top 10 significantly upregulated and downregulated proteins in each model is shown in **Figure 6C**. Many of these DEPs have already been reported as being associated with PD. For example, in the acute MPTP model, Sigmar1, which was upregulated, and Dmkn, Dnmt1, Slc6a3, DDC, and TH, which were downregulated, are related to PD pathology. In the 16-month-old A53T model, brain-derived neurotrophic factor (BDNF) and Dnajc24 were upregulated, and Ap5z1 was downregulated; all three proteins are disease-related. In the 5-month-old MitoPark model, Slc6a3, Cntn5, and WAS, which are linked to PD, were downregulated. Intersection analysis of the models showed that Timm8a1 and Sephs1 were altered in all three (**Figure 6D**), indicating characteristic PD-relevant protein changes. Detailed information regarding the DEPs obtained from the intersection analysis is shown in **Additional Table 8**.

KEGG and GO enrichment analyses showed that the DEPs in the three PD models were enriched in neurodegeneration-related pathways and functions. Neurotransmitter transport (a shared biological process) and mitochondrial inner membrane organization (a common cellular component) were both significantly enriched in all three models. Select commonly enriched KEGG pathways are visualized in **Figure 6E**. The neurodegeneration pathway was enriched in the acute MPTP and 16-month-old A53T models (**Figure 6Ea** and **Eb**), while the 5-month-old MitoPark model exhibited Parkinson disease pathway enrichment (**Figure 6Ec**). Molecular function analysis revealed model-specific patterns: metal ion transmembrane transporter activity was enriched in both the MitoPark model and the A53T model, and passive transmembrane transporter activity was enriched in both the MPTP model and the A53T model (**Figure 6G**). Detailed information regarding the KEGG and GO enrichment analyses is shown in **Additional Tables 9** and **10**.

Intersection analysis of the KEGG enrichment results from the three PD models identified five shared pathways (**Figure 6F**), and intersection analysis of the GO results identified 112 shared functional annotations (**Figure 6H**). They covered categories such as vesicle-mediated transport, neurotransmitter transport and secretion, ion homeostasis (especially metal ion transmembrane transport), and mitochondrial inner membrane, all of which are likely related to PD pathogenesis.

GSEA revealed distinct pathological mechanisms: acute MPTP mice showed prominent complement activation, inflammatory signaling, and dopaminergic regulation; 16-month-old A53T mice exhibited extracellular matrix reorganization, growth factor dysregulation, and increased platelet-derived growth factor (PDGF) signaling; and 5-month-old MitoPark mice demonstrated pronounced mitochondrial dysfunction across neurodegeneration pathways, including those for Parkinson’s, Alzheimer’s, and Huntington’s diseases (**Figure 6I** and **Additional Table 11**).

Protein–protein interaction network analysis was performed to identify model-specific hub proteins: the hub proteins in the acute MPTP model were FOXO1, LAMP2, and GAP43, interacting with proteins including AKT1S1, RRAGB, and HCN3 and forming modules regulating cellular stress response, protein degradation, and neurotransmitter release; the hub proteins in the 16-month-old A53T model were NDUFAF3, CAMK2A, SEC61A1, BDNF, NEFM, and PLCB1, interacting with proteins such as NDUFA9, NDUFA12, SLC17A7, and ANK3 and participating in networks governing mitochondrial function, neurotransmission, neurotrophic support, cytoskeletal dynamics, and signaling pathways; the hub proteins in 5-month-old MitoPark model were RPL8, ATP5O, and PSEN1, interacting with TH, MRPS18B, MRPS23, ATP5A1, and UBQLN2 and forming modules regulating protein synthesis, energy metabolism, mitochondrial DNA repair, and neurotransmitter pathways (**Figure 6J**). Detailed information regarding the results from the protein–protein interaction network analysis is shown in **Additional Table 12**.

### Intersection analysis of the pathways enriched differentially expressed genes among the three Parkinson’s disease models and patients with Parkinson’s disease

**Figure 7:**
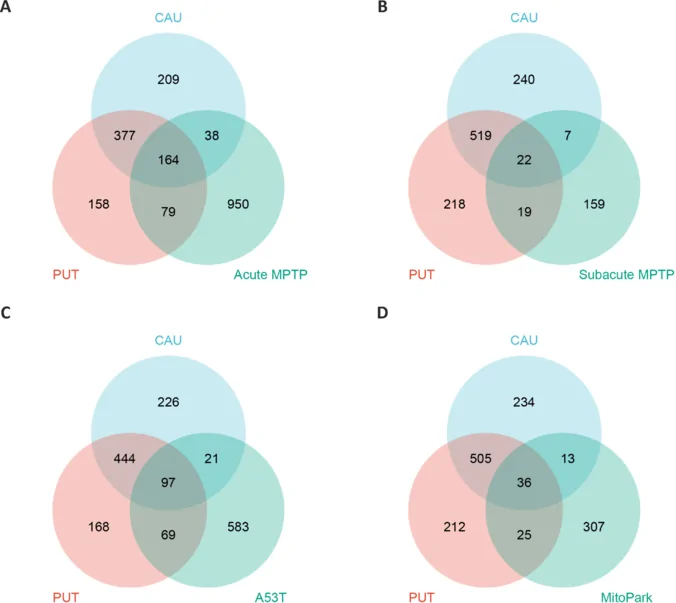
Venn diagrams illustrating intersections among the GO and KEGG signaling pathways enriched in DEGs in the brains of the three PD mouse models, as well as the PUT and CAU of autopsied patients with PD. (A) MPTP acute model. (B) MPTP subacute model. (C) 16-month-old A53T model. (D) 5-month-old MitoPark model. The numbers in the diagrams represent the counts of unique and overlapping GO and KEGG categories in corresponding regions or models. A53T: α-Synuclein A53T mutant; CAU: caudate nucleus; GO: Gene Ontology; KEGG: Kyoto Encyclopedia of Genes and Genomes; MPTP: 1-methyl-4-phenyl-1,2,3,6-tetrahydropyridine; MitoPark: MitoPark transgenic mouse model; PD: Parkinson’s disease; PUT: putamen.

To assess the extent to which each of the three PD mouse models recapitulates the unique pathological signaling pathways active in human PD pathology, we performed an overlap analysis of GO and KEGG pathways enriched in DEGs in the striatum of the three PD models and those reported in the putamen and caudate nucleus of postmortem brains from patients with PD (Irmady et al., 2023). The acute MPTP model shared 164 common categories with human PD, encompassing the neurotransmitter homeostasis, calcium ion regulation, synaptic plasticity, neuronal development, cellular stress and protein processing, immune inflammation, signal transduction, learning and memory, and locomotor behavior-related pathways (**Figure 7A**). The subacute MPTP model exhibited 22 shared GO and KEGG categories with human PD, involving cellular ion homeostasis, signal transduction, cellular architecture, extracellular matrix organization, substance transport, receptor activity, and stimulus responses (**Figure 7B**). The 16-month-old A53T transgenic model harbored 97 common categories with human PD, including nervous system-associated signaling pathways, neural function and behavioral processes, and select cellular physiological pathways (**Figure 7C**). Finally, the 5-month-old MitoPark model displayed 36 common categories with human PD, spanning neurotransmission and signal transduction, ion transport, intracellular signaling, protein refolding, transmembrane transport, immune inflammation, and membrane structure-related pathways (**Figure 7D**). This suggests that the extent to which each PD model simulates human PD pathology and the characteristics of the simulated pathological signaling pathways vary by model. Detailed information regarding the common GO/KEGG signaling pathways obtained from the intersection analysis is shown in **Additional Table 13**.

## Discussion

The comparative analysis of the MPTP-induced, α-syn A53T transgenic, and MitoPark mouse models that we performed in this study identified key PD-related signaling pathways and will help researchers select the right model for their specific research aims. In this study, we used a range of methods, including behavioral tests, immunohistochemistry, immunofluorescence, western blot, RNA-seq, and proteomics, to comprehensively analyze PD pathogenesis in these three models, providing new insights into the underlying mechanisms by which they simulate the human disease.

Distinct motor phenotypes were observed among the three PD models. In the MPTP model, rapid neurotoxic damage to the dopaminergic system impaired balance and coordination, as evidenced by poor performance in the pole test and reduced activity in the cylinder test. By contrast, A53T mice exhibited complex motor abnormalities: 12-month-old transgenic mice displayed hyperactivity in both the open field test and cylinder test, yet showed delayed turning in the pole test and reduced scores in the wire hanging test. The observed hyperactivity in A53T mice is consistent with findings from other studies (Unger et al., 2006; Graham and Sidhu, 2010; Paumier et al., 2013), although the underlying mechanism requires further investigation. In the MitoPark model, mitochondrial dysfunction within dopaminergic neurons led to progressive motor impairments, which were consistently evident across all of the behavioral tests that we performed and worsened with age.

All three models exhibited similar dopaminergic neuronal damage but different patterns of glial cell activation. In the MPTP model, astrocytes and microglia were quickly and strongly activated by neurotoxin exposure, which might have damaged neurons through excessive stress. The 16-month-old α-syn A53T transgenic (G2–3 line) model, however, showed no significant glial activation. This could be due to low levels of pathological α-syn. The MitoPark model exhibited a unique sequence of bioenergetic failure, starting with mitochondrial defects, then metabolic stress, and finally neuroinflammatory priming (Ekstrand and Galter, 2009; Chen et al., 2019). These differences demonstrate the complex link between neurodegenerative triggers and neuroinflammatory processes, with mitochondrial homeostasis playing a crucial role in controlling inflammation.

Transcriptomic and proteomic analyses revealed distinct molecular profiles in the three PD models. In the MPTP-induced acute model, the *Btk* and *Cdk1* upregulations were observed alongside neuroinflammatory activation and cell cycle dysregulation (Chen et al., 2024; Sun et al., 2024), while elevated *Serpina3n* levels may reflect a compensatory stress response (Hong et al., 2025). *Crh*, *Ret*, and *Hsf1* downregulation was associated with reduced neurotrophic support and proteostatic capacity (Hsieh et al., 2011; Gong et al., 2024). At the protein level, Sigmar1, which has been linked to protein quality control and neuroprotection (Li et al., 2023a), was upregulated, while Dmkn downregulation correlated with disrupted cellular homeostasis (Liu et al., 2023). Dnmt1 downregulation may also disrupt gene expression, affecting neuron survival (Wang et al., 2023). The decrease in Slc6a3 expression observed in both the MPTP acute model and the 5-month-old MitoPark model, was consistent with disrupted dopamine equilibrium, a characteristic of PD (Xie et al., 2024). The decline in DDC levels that we observed has been associated with neurotransmitter synthesis dysregulation and could serve as a PD biomarker (Bolsewig et al., 2025). The decrease in TH, a marker of dopaminergic neurons, was consistent with our western blot results.

The 16-month-old A53T transgenic model showed age-dependent molecular adaptations. *Dnajc5* upregulation could enhance neuronal resilience (Chandra et al., 2005), while increased *Ankle1* expression might be linked to mitochondrial dysfunction (Przanowski et al., 2023). *Dhh*, which was upregulated, has been associated with hedgehog signaling–mediated cellular homeostasis, but its direct neuroprotective role in PD requires validation (Dong et al., 2019). *S100a5* downregulation could worsen neuroinflammation through chemokine dysregulation (Zimmer et al., 2005), and *Pomc* reduction may disrupt neuroendocrine balance (Li et al., 2021). Our proteomic analysis indicated elevated BDNF, which has been previously correlated with dopaminergic neuron survival, and increased DNAJC24, a chaperone implicated in protein folding. AP5Z1/SPG48 downregulation could trigger mitochondrial dysfunction and neurofilament aggregation.

In the 5-month-old MitoPark model, we noted upregulation of *Gpx2*, which is involved in antioxidant defense (Zhu et al., 2025). Conversely, we also observed downregulation of the *Sele*, *SelP*, and *Ltf*, which could affect PD progression: diminished *Sele* impairs neurovascular inflammatory modulation (Rajkumar et al., 2020), *Selp* downregulation disrupts selenium trafficking for glutathione peroxidase function (Salaramoli et al., 2024), and *Ltf* suppression worsens iron-driven Fenton reactions (Xu et al., 2024). Other proteomic changes noted in this model have been correlated with neurodegenerative processes: CNTN5 loss disrupts axon–glia synchronization, an early sign of PD (Chatterjee et al., 2019), and WAS protein downregulation promotes α-syn aggregation (Jackson et al., 2024). Further analysis revealed that, among the five genes whose expression was altered in all three models, changes in the expression of *Ifi27l2a*, *Ifitm3*, and *RTP4* have been previously implicated in neuroinflammation or the immune response (Fujita and Kuroiwa, 2024; Feng et al., 2025; Kim et al., 2025), while altered *Oasl2* expression may affect immune-related functions (Zhu et al., 2014), and *Ankk1* is related to genetic susceptibility to PD (Pérez-Santamarina et al., 2021). Intersection analysis identified two DEPs that were shared among all three models: SEPHS1 and TIMM8A1. SEPHS1 is engaged in selenoprotein synthesis, while TIMM8A1 is involved in the maintenance of mitochondrial function. Selenoproteins play important roles in various biological processes, including antioxidant defense, cell homeostasis, and neurodevelopment. These findings suggest a shared regulatory mechanism related to cell homeostasis and mitochondrial function across the three models.

Accumulating evidence demonstrates that, in the MPTP model, suppression of ferroptosis-related proteins exerts neuroprotective effects on dopaminergic neurons (Lin et al., 2022; Li et al., 2023b). Meanwhile, a study involving the A53T model reported that deficiency in the ferroptosis-associated enzyme calcium-independent phospholipase A2β promotes dopaminergic neuron susceptibility to ferroptosis (Sun et al., 2021). Additionally, mitochondria have been implicated in ferroptosis regulation (Liu et al., 2024; Peng et al., 2025; Wang et al., 2025). Our RNA-seq analysis identified multiple ferroptosis-related DEGs across the three models, with different activation mechanisms and key gene alterations. In the MPTP model, reduced *Slc1a5* expression was observed in conjunction with impaired cystine uptake, decreasing GSH synthesis and GPX4 activity, which would increase sensitivity to ferroptosis; *Flt3* downregulation may promote ferroptosis by negatively regulating the anti-ferroptosis pathway; and *Lcn2* upregulation may enhance iron uptake and oxidative stress, thereby facilitating ferroptosis (Qiu et al., 2024). In the A53T model, *Prnp* upregulation coincided with its reported role in maintaining iron homeostasis and boosting antioxidant capacity, while *Nos2* upregulation mirrored prior links to NO production, oxidative stress, and ferroptosis. *Drd4* upregulation may influence signaling and redox balance, increasing susceptibility to ferroptosis. In the MitoPark model, *Hspb1* downregulation was detected - an expression pattern previously associated with the cell stress response, contributing to mitochondrial dysfunction and ferroptosis; while *Lcn2* and *Prnp* downregulation may disrupt iron metabolism, the cell defense, and cellular homeostasis, exacerbating ferroptosis. Taken together, these findings suggest that ferroptosis is a common mechanism in PD models, offering a promising target for neuroprotective therapies.

The enrichment of IL17 pathway genes coupled with downregulation of *IL17a* and *IL17ra* in the MitoPark model reveals a unique neuroinflammatory signature distinct from the acute neuroinflammation observed in the MPTP model. IL17a has been associated with exacerbated PD pathology through pro-inflammatory signaling pathways (Meredith and Rademacher, 2011). Although IL17a typically promotes neuroinflammation through the NF-κB and MAPK pathways (Chen et al., 2013), emerging evidence suggests that it may also confer neuroprotection through immunomodulation, thereby reducing neuronal damage under neurodegenerative conditions (Chen et al., 2020). Given these contrasting roles, the precise function of the IL17 pathway in the MitoPark model warrants further investigation.

Owing to the limited number of mice included in our study, which resulted from challenges in breeding, low survival rates among homozygous pups, and variable transgene expression, pathological progression in α-syn A53T mice could not be assessed beyond 16 months. We aim to perform a more detailed analysis once an adequate number of mice becomes available in the future. These subsequent studies will also allow for more comprehensive analysis and validation of the five common DEGs (*Ifi27l2a*, *Ifitm3*, *Oasl2*, *Rtp4*, and *Ankk1*) and two common DEPs (Timm8a1 and Sephs1), with a particular focus on elucidating their mechanistic roles across different PD models to better clarify their contribution to disease progression.

Overall, the three PD models exhibit similarities and differences in behaviors, nigrostriatal changes, and gene/protein expression profiles. They not only share aspects such as neurodegeneration, immune regulation, and energy metabolism processes, but also all possess unique molecular traits, highlighting PD’s complex pathogenesis. Although each model replicates certain features of PD, they also each have limitations. The MPTP model rapidly induces dopaminergic neuron damage and neuroinflammation, but lacks Lewy bodies and phosphorylated α-syn accumulation. The homozygous α-syn A53T TgM83 mouse model recapitulates Lewy body formation and increased phosphorylated α-syn (Lee et al., 2002; Bencsik et al., 2014); however, the age of onset and disease severity vary significantly among different transgenic lines. In the heterozygous strain (Line G2–3) used in this study (Lee et al., 2002; Rota et al., 2019), dopaminergic neuron loss is moderate, and pathological features, including phosphorylated pathological α-syn accumulation, develop slowly. Given that homozygous G2–3 mice are non-viable, future studies aiming to observe significant α-syn aggregation should either extend the observation period beyond 16 months or adopt alternative models. The MitoPark model demonstrates mitochondrial dysfunction and progressive neuronal damage, but without the typical Lewy bodies and phosphorylated α-syn changes. Thus, integrating multiple models is essential for studying PD pathogenesis and treatment.

## Additional files:

***Additional Figure 1:*** *Experimental design.*

Additional Figure 1 Experimental design.
(A) Acute MPTP model. (B) Subacute MPTP model. (C) α-Syn A53T model. (D) MitoPark model. MitoPark: MitoPark transgenic mouse model; MPTP: 1-methyl-4-phenyl-1,2,3,6-tetrahydropyridine; α-Syn A53T: α-Synuclein A53T mutant.

***Additional Figure 2:*** *Results from the rotarod test in 12-month-old α-Syn A53T mice.*

Additional Figure 2 Results from the rotarod test in 12-month-old α-Syn A53T mice.
(A) Falling time; (B) distance fallen; and (C) falling speed at which the mice fell in the rotarod test. Data are expressed as mean ± SEM (*n* = 8). ^*^P < 0.05. For all data in A-C, unpaired *t*-test (with Welch’s correction) was used. Ctrl: Control; ns: not significant; α-Syn A53T: α-synuclein A53T mutant.

***Additional Figure 3:*** *Body weight and behavior in 16-month-old α-Syn A53T mice.*

Additional Figure 3 Body weight and behavior in 16-month-old α-Syn A53T mice.
(A) Body weight. (B) Scores in the wire hanging test. (C) Rearing times within 3 minutes in the cylinder test. Data are expressed as mean ± SEM (*n* = 12). ^*^P < 0.05. For all data in A-C, unpaired *t*-test (with Welch’s correction) was used. Ctrl: Control; α-Syn A53T: α-synuclein A53T mutant.

***Additional Figure 4:*** *TFAM deficiency in dopaminergic neurons of the MitoPark model.*

Additional Figure 4 TFAM deficiency in dopaminergic neurons in the MitoPark model.
Immunofluorescence staining of dopaminergic neurons (Alexa Fluor^TM^ Plus 488, green for TH^+^), mitochondrial transcription factor A (Alexa Fluor^TM^ Plus 594, red for TFAM^+^), and cell nuclei (blue (DAPI)) in the substantia nigra of mice. The circled area indicates dopaminergic neurons. Virtually no TFAM signals were detected in dopaminergic neurons in MitoPark mice. Scale bars: 5 μm. Ctrl: Control; DAPI: 4',6-diamidino-2-phenylindole; GFAP: glial fibrillary acidic protein; IBA1: ionized calcium-binding adapter molecule 1; MitoPark: MitoPark transgenic mouse model; SNc: substantia nigra pars compacta; TH: tyrosine hydroxylase; TFAM: transcription factor A, mitochondrial.

***Additional Figure 5:*** *Lesions in the substantia nigra and striatum of mice 7 days after acute MPTP administration.*

Additional Figure 5 Lesions in the substantia nigra and striatum of mice 7 days after acute MPTP administration.
(A) Immunofluorescence staining of dopaminergic neurons (Alexa Fluor^TM^ Plus 647, gray for TH^+^), astrocytes (Cy^TM^3, red for GFAP^+^), and microglia (Alexa Fluor^TM^ Plus 488, green). GFAP: Glial fibrillary acidic protein; IBA1: ionized calcium-binding adapter molecule 1; MitoPark: MitoPark transgenic mouse model (a Parkinson’s disease model); MPTP: 1-Methyl-4-phenyl-1,2,3,6-tetrahydropyridine; NS: normal saline; SNc: Substantia nigra pars compacta; TH: tyrosine hydroxylase.

***Additional Figure 6:*** *Lesions in the substantia nigra of MitoPark model.*

Additional Figure 6 Lesions in the substantia nigra of the MitoPark model.
Immunohistochemical staining of dopaminergic neurons (brown for TH^+^) in the SNc of mice. MPTP treatment significantly decreased the number of TH^+^ dopaminergic neurons. Scale bar: 100 μm. Data are expressed as mean ± SEM (*n* = 3). ^****^P < 0.0001. For all data presented in the bar graph, unpaired *t*-test (with Welch’s correction) was used. Ctrl: control; MitoPark: MitoPark transgenic mouse model (a Parkinson’s disease model); MPTP: 1-Methyl-4-phenyl-1,2,3,6-tetrahydropyridine; SNc: Substantia nigra pars compacta; TH: tyrosine hydroxylase.

***Additional Figure 7:*** *RNA-seq analysis of the striatum in subacute MPTP and 12-month-old A53T models..*

Additional Figure 7 RNA-seq analysis of the striatum in subacute MPTP and 12-month-old A53T models.
(A) Analysis of the subacute MPTP model. (B) 12-month-old A53T model. (a) Principal component analysis. (b) Volcano plot: DEGs in the model vs. control groups (blue: downregulated; red: upregulated). (c) Heatmap: top 10 up- and downregulated DEGs in the model vs. control groups. The genes highlighted in red boxes are associated with PD. A53T: α-Synuclein A53T mutant; Ctrl: Control; DEG: differentially expressed gene; MPTP: 1-methyl-4-phenyl-1,2,3,6-tetrahydropyridine; NS: normal saline; PC: principal component; PCA: principal component analysis; PD: Parkinson’s disease; RNA-seq: RNA sequencing.

***Additional Table 1:*** *Comparison of behavioral performance among the three PD model mice.*

***Additional Table 2:*** *Comparison of nigrostriatal pathway damage among the three PD models.*

***Additional Table 3:*** *Intersection analysis of DEGs across the three PD mouse models.*

Additional Table 3 Intersection analysis of DEGs across the three PD mouse models

***Additional Table 4:*** *GO pathway identified from RNA-Seq data analysis of the striatum in three PD mouse models.*

Additional Table 4 GO pathway identified from RNA-Seq data analysis of the striatum in three PD mouse models

***Additional Table 5:*** *KEGG pathway identified from RNA-Seq data analysis of the striatum in three PD mouse models.*

Additional Table 5 KEGG pathway identified from RNA-Seq data analysis of the striatum in three PD mouse models

***Additional Table 6:*** *Pathway enrichment results from GSEA analysis of RNA-Seq data in the striatum of three PD mouse models.*

Additional Table 6 Pathway enrichment results from GSEA analysis of RNA-Seq data in the striatum of three PD mouse models.

***Additional Table 7:*** *DEGs involved in the ferroptosis pathway and their logFC values from RNA-seq analysis in the MPTP, A53T, and MitoPark mouse models.*

***Additional Table 8:*** *Intersection analysis of DEPs across the three PD mouse models.*

Additional Table 8 Intersection analysis of DEPs across the three PD mouse models

***Additional Table 9:*** *GO pathway identified from proteomic data analysis of the striatum in the three PD mouse models.*

Additional Table 9 GO pathway identified from proteomic data analysis of the striatum in three PD mouse models

***Additional Table 10:*** *KEGG pathway identified from proteomic data analysis of the striatum in the three PD mouse models.*

Additional Table 10 KEGG pathway identified from proteomic data analysis of the striatum in three PD mouse models

***Additional Table 11:*** *Pathway enrichment results from GSEA analysis of proteomic data in the striatum of the three PD mouse models.*

Additional Table 11 Pathway enrichment results from GSEA analysis of proteomic data in the striatum of three PD mouse models

***Additional Table 12:*** *PPI nodes identified from proteomic data analysis of the stratum in the three PD mouse models.*

Additional Table 12 PPI nodes identified from proteomic data analysis of the stratum in three PD mouse models

***Additional Table 13:*** *Common GO/KEGG pathways revealed in the intersection analysis between human samples and mouse models.*

Additional Table 13 Common GO/KEGG pathways revealed in the intersection analysis between human samples and mouse models

## Data Availability

*The raw data and intermediate analysis files from both RNA-seq and proteomic analyses of this study have been deposited in the Gene Expression Omnibus (GEO, accession number: GSE300519, https://www.ncbi.nlm.nih.gov/geo/query/acc.cgi?acc=GSE300519) and the iProx (https://www.iprox.cn//page/SCV017.html?query=IPX0012353000), ensuring full traceability and reproducibility of the research data. All other data are provided in Additional files.*
