# Supplementary material for: Neurotoxicity, α-synuclein pathology, and mitochondrial dysfunction: A comparative study of different mouse models of Parkinson’s disease
Source: Neural Regen Res. 2025 Sep 29;21(10):5000–12. doi: 10.4103/NRR.NRR-D-25-00648 (PMC13568611; doi:10.4103/NRR.NRR-D-25-00648)
Supplement: Supplementary file 1 [file NRR-21-5000_Suppl1.pdf]

Additional Table 3. Intersection analysis of DEGs across the three PD mouse models

Only A53T			Only MitoPark			Only MPTP			A53T AND MitoPark NOT MPTP			A53T AND MPTP NOT MitoPark			MitoPark AND MPTP NOT A53T			A53T AND MitoPark AND MPTP		
**GeneID**	**GeneSymbol**	**Description**	**GeneID**	**GeneSymbol**	**Description**	**GeneID**	**GeneSymbol**	**Description**	**GeneID**	**GeneSymbol**	**Description**	**GeneID**	**GeneSymbol**	**Description**	**GeneID**	**GeneSymbol**	**Description**	**GeneID**	**GeneSymbol**	**Description**
ENSMUSG00000096833	Igkv4-55	immunoglobulin kappa variable 4-55 [Source:MGI Symbol%3BAcc:MGI:2686370]	ENSMUSG00000113047	Gm47469	predicted gene%2C 47469 [Source:MGI Symbol%3BAcc:MGI:6096437]	ENSMUSG00000000120	Ngfr	nerve growth factor receptor (TNFR superfamily%2C member 16) [Source:MGI Symbol%3BAcc:MGI:97323	ENSMUSG00000001023	S100a5	S100 calcium binding protein A5 [Source:MGI Symbol%3BAcc:MGI:1338915]	ENSMUSG00000047370	Gm7367	predicted pseudogene 7367 [Source:MGI Symbol%3BAcc:MGI:3646404]	ENSMUSG00000070327	Rnf213	ring finger protein 213 [Source:MGI Symbol%3BAcc:MGI:1289196]	ENSMUSG00000079017	Ifi27l2a	interferon%2C alpha-inducible protein 27 like 2A [Source:MGI Symbol%3BAcc:MGI:1924183]
ENSMUSG00000026971	Itgb6	integrin beta 6 [Source:MGI Symbol%3BAcc:MGI:96615]	ENSMUSG00000026822	Lcn2	lipocalin 2 [Source:MGI Symbol%3BAcc:MGI:96757]	ENSMUSG00000038257	Glra3	glycine receptor%2C alpha 3 subunit [Source:MGI Symbol%3BAcc:MGI:95749]	ENSMUSG00000111652	Gm48726	predicted gene%2C 48726 [Source:MGI Symbol%3BAcc:MGI:6098384]	ENSMUSG00000004655	Aqp1	aquaporin 1 [Source:MGI Symbol%3BAcc:MGI:103201]	ENSMUSG00000038550	Ciart	circadian associated repressor of transcription [Source:MGI Symbol%3BAcc:MGI:2684975]	ENSMUSG00000025492	Ifitm3	interferon induced transmembrane protein 3 [Source:MGI Symbol%3BAcc:MGI:1913391]
ENSMUSG00000089887	4930428N03Rik	RIKEN cDNA 4930428N03 gene [Source:MGI Symbol%3BAcc:MGI:1921121]	ENSMUSG00000031167	Rbm3	RNA binding motif (RNP1%2C RRM) protein 3 [Source:MGI Symbol%3BAcc:MGI:1099460]	ENSMUSG00000074340	Ovgp1	oviductal glycoprotein 1 [Source:MGI Symbol%3BAcc:MGI:106661]	ENSMUSG00000099950	9130227L01Rik	RIKEN cDNA 9130227L01 gene [Source:MGI Symbol%3BAcc:MGI:2445084]	ENSMUSG00000039323	Igfbp2	insulin-like growth factor binding protein 2 [Source:MGI Symbol%3BAcc:MGI:96437]	ENSMUSG00000049502	Dtx3l	deltex 3-like%2C E3 ubiquitin ligase [Source:MGI Symbol%3BAcc:MGI:2656973]	ENSMUSG00000029561	Oasl2	2'-5' oligoadenylate synthetase-like 2 [Source:MGI Symbol%3BAcc:MGI:1344390]
ENSMUSG00000036853	Mcoln3	mucolipin 3 [Source:MGI Symbol%3BAcc:MGI:1890500]	ENSMUSG00000047246	H2bc6	H2B clustered histone 6 [Source:MGI Symbol%3BAcc:MGI:2448380]	ENSMUSG00000000628	Hk2	hexokinase 2 [Source:MGI Symbol%3BAcc:MGI:1315197]	ENSMUSG00000050370	Ch25h	cholesterol 25-hydroxylase [Source:MGI Symbol%3BAcc:MGI:1333869]	ENSMUSG00000109588	Lnp1	leukemia NUP98 fusion partner 1 [Source:MGI Symbol%3BAcc:MGI:5011982]	ENSMUSG00000054404	Slfn5	schlafen 5 [Source:MGI Symbol%3BAcc:MGI:1329004]	ENSMUSG00000033355	Rtp4	receptor transporter protein 4 [Source:MGI Symbol%3BAcc:MGI:1915025]
ENSMUSG00000105545	4930599N24Rik	RIKEN cDNA 4930599N24 gene [Source:MGI Symbol%3BAcc:MGI:1922633]	ENSMUSG00000045193	Cirbp	cold inducible RNA binding protein [Source:MGI Symbol%3BAcc:MGI:893588]	ENSMUSG00000000682	Cd52	CD52 antigen [Source:MGI Symbol%3BAcc:MGI:1346088]	ENSMUSG00000112947	Gm47493	predicted gene%2C 47493 [Source:MGI Symbol%3BAcc:MGI:6096474]	ENSMUSG00000032334	Loxl1	lysyl oxidase-like 1 [Source:MGI Symbol%3BAcc:MGI:106096]	ENSMUSG00000051627	H1f4	H1.4 linker histone%2C cluster member [Source:MGI Symbol%3BAcc:MGI:1931527]	ENSMUSG00000032257	Ankk1	ankyrin repeat and kinase domain containing 1 [Source:MGI Symbol%3BAcc:MGI:3045301]
ENSMUSG00000113336	Gm16497	predicted gene 16497 [Source:MGI Symbol%3BAcc:MGI:3642481]	ENSMUSG00000043872	Zmym1	zinc finger%2C MYM domain containing 1 [Source:MGI Symbol%3BAcc:MGI:19155	ENSMUSG00000000693	Loxl3	lysyl oxidase-like 3 [Source:MGI Symbol%3BAcc:MGI:1337004]	ENSMUSG00000091172	Gm17120	predicted gene 17120 [Source:MGI Symbol%3BAcc:MGI:4937947]	ENSMUSG00000083798	Gm14584	predicted gene 14584 [Source:MGI Symbol%3BAcc:MGI:3705780]	ENSMUSG00000026656	Fcgr2b	Fc receptor%2C IgG%2C low affinity IIb [Source:MGI Symbol%3BAcc:MGI:95499			
ENSMUSG00000020660	Pomc	pro-opiomelanocortin-alpha [Source:MGI Symbol%3BAcc:MGI:97742]	ENSMUSG00000056071	S100a9	S100 calcium binding protein A9 (calgranulin B) [Source:MGI Symbol%3BAcc:MGI:1338947]	ENSMUSG00000001025	S100a6	S100 calcium binding protein A6 (calcyclin) [Source:MGI Symbol%3BAcc:MGI:1339467]	ENSMUSG00000111118	Gm6545	predicted gene 6545 [Source:MGI Symbol%3BAcc:MGI:3643874]	ENSMUSG00000031530	Dusp4	dual specificity phosphatase 4 [Source:MGI Symbol%3BAcc:MGI:2442191]	ENSMUSG00000030921	Trim30a	tripartite motif-containing 30A [Source:MGI Symbol%3BAcc:MGI:98178]			
ENSMUSG00000117652	Gm2350	predicted gene 2350 [Source:MGI Symbol%3BAcc:MGI:3826781]	ENSMUSG00000089774	Slc5a3	solute carrier family 5 (inositol transporters)%2C member 3 [Source:MGI Symbol%3BAcc:MGI:1858226]	ENSMUSG00000029994	Anxa4	annexin A4 [Source:MGI Symbol%3BAcc:MGI:88030]	ENSMUSG00000030708	Dnajb13	DnaJ heat shock protein family (Hsp40) member B13 [Source:MGI Symbol%3BAcc:MGI:1916637]	ENSMUSG00000016526	Dyrk3	dual-specificity tyrosine-(Y)-phosphorylation regulated kinase 3 [Source:MGI Symbol%3BAcc:MGI:1330300]	ENSMUSG00000073489	Ifi204	interferon activated gene 204 [Source:MGI Symbol%3BAcc:MGI:96429]			
ENSMUSG00000087618	Gm14114	predicted gene 14114 [Source:MGI Symbol%3BAcc:MGI:3652257]	ENSMUSG00000026864	Hspa5	heat shock protein 5 [Source:MGI Symbol%3BAcc:MGI:95835]	ENSMUSG00000001289	Pfdn5	prefoldin 5 [Source:MGI Symbol%3BAcc:MGI:1928753]	ENSMUSG00000028341	Nr4a3	nuclear receptor subfamily 4%2C group A%2C member 3 [Source:MGI Symbol%3BAcc:MGI:1352457]	ENSMUSG00000032122	Slc37a2	solute carrier family 37 (glycerol-3-phosphate transporter)%2C member 2 [Source:MGI Symbol%3BAcc:MGI:1929693]	ENSMUSG00000034459	Ifit1	interferon-induced protein with tetratricopeptide repeats 1 [Source:MGI Symbol%3BAcc:MGI:99450]			
ENSMUSG00000086170	Gm12144	predicted gene 12144 [Source:MGI Symbol%3BAcc:MGI:3649685]	ENSMUSG00000086503	Xist	inactive X specific transcripts [Source:MGI Symbol%3BAcc:MGI:98974]	ENSMUSG00000022219	Cideb	cell death-inducing DNA fragmentation factor%2C alpha subunit-like effector B [Source:MGI Symbol%3BAcc:MGI:1270844]	ENSMUSG00000085945	2310014F06Rik	RIKEN cDNA 2310014F06 gene [Source:MGI Symbol%3BAcc:MGI:1916875]	ENSMUSG00000033033	Calhm2	calcium homeostasis modulator family member 2 [Source:MGI Symbol%3BAcc:MGI:1919941]	ENSMUSG00000030107	Usp18	ubiquitin specific peptidase 18 [Source:MGI Symbol%3BAcc:MGI:1344364]			
ENSMUSG00000074469	Gm15348	predicted gene 15348 [Source:MGI Symbol%3BAcc:MGI:3642533]	ENSMUSG00000096768	Gm47283	predicted gene%2C 47283 [Source:MGI Symbol%3BAcc:MGI:6096131]	ENSMUSG00000023186	Vwa5a	von Willebrand factor A domain containing 5A [Source:MGI Symbol%3BAcc:MGI:1915026]	ENSMUSG00000070605	Zfp992	zinc finger protein 992 [Source:MGI Symbol%3BAcc:MGI:3700963]	ENSMUSG00000023192	Grm2	glutamate receptor%2C metabotropic 2 [Source:MGI Symbol%3BAcc:MGI:1351339]	ENSMUSG00000035692	Isg15	ISG15 ubiquitin-like modifier [Source:MGI Symbol%3BAcc:MGI:1855694]			
ENSMUSG00000023120	Ldc1	leucine decarboxylase 1 [Source:MGI Symbol%3BAcc:MGI:2685699]	ENSMUSG00000109812	Gm45640	predicted gene 45640 [Source:MGI Symbol%3BAcc:MGI:5791476]	ENSMUSG00000001506	Col1a1	collagen%2C type I%2C alpha 1 [Source:MGI Symbol%3BAcc:MGI:88467]	ENSMUSG00000110735	Gm47173	predicted gene%2C 47173 [Source:MGI Symbol%3BAcc:MGI:6095954]	ENSMUSG00000046718	Bst2	bone marrow stromal cell antigen 2 [Source:MGI Symbol%3BAcc:MGI:1916800]	ENSMUSG00000001020	S100a4	S100 calcium binding protein A4 [Source:MGI Symbol%3BAcc:MGI:1330282]			
ENSMUSG00000117852	4930471G03Rik	RIKEN cDNA 4930471G03 gene [Source:MGI Symbol%3BAcc:MGI:1922216]	ENSMUSG00000095562	Erdr1	erythroid differentiation regulator 1 [Source:NCBI gene (formerly Entrezgene)%3BAcc:17094	ENSMUSG00000001520	Nrip2	nuclear receptor interacting protein 2 [Source:MGI Symbol%3BAcc:MGI:18918	ENSMUSG00000111163	Gm46136	predicted gene%2C 46136 [Source:MGI Symbol%3BAcc:MGI:5825773]	ENSMUSG00000040978	Gm11992	predicted gene 11992 [Source:MGI Symbol%3BAcc:MGI:3651127]	ENSMUSG00000045509	Gpr150	G protein-coupled receptor 150 [Source:MGI Symbol%3BAcc:MGI:2441872]			
ENSMUSG00000117118	Gm49878	predicted gene%2C 49878 [Source:MGI Symbol%3BAcc:MGI:6270558]	ENSMUSG00000114277	Gm48583	predicted gene%2C 48583 [Source:MGI Symbol%3BAcc:MGI:6098152]	ENSMUSG00000001663	Gstt1	glutathione S-transferase%2C theta 1 [Source:MGI Symbol%3BAcc:MGI:107379]	ENSMUSG00000056144	Trim34a	tripartite motif-containing 34A [Source:MGI Symbol%3BAcc:MGI:2137359]	ENSMUSG00000019880	Rspo3	R-spondin 3 [Source:MGI Symbol%3BAcc:MGI:1920030]	ENSMUSG00000047735	Samd9l	sterile alpha motif domain containing 9-like [Source:MGI Symbol%3BAcc:MGI:1343184]			
ENSMUSG00000103560	Gm38070	predicted gene%2C 38070 [Source:MGI Symbol%3BAcc:MGI:5611298]	ENSMUSG00000097098	9330111N05Rik	RIKEN cDNA 9330111N05 gene [Source:MGI Symbol%3BAcc:MGI:2443112]	ENSMUSG00000001750	Tcirg1	T cell%2C immune regulator 1%2C ATPase%2C H+ transporting%2C lysosomal V0 protein A3 [Source:MGI Symbol%3BAcc:MGI:13509	ENSMUSG00000063268	Parp10	poly (ADP-ribose) polymerase family%2C member 10 [Source:MGI Symbol%3BAcc:MGI:3712326]	ENSMUSG00000049796	Crh	corticotropin releasing hormone [Source:MGI Symbol%3BAcc:MGI:88496]	ENSMUSG00000023067	Cdkn1a	cyclin-dependent kinase inhibitor 1A (P21) [Source:MGI Symbol%3BAcc:MGI:104556]			
ENSMUSG00000052276	Ostn	osteocrin [Source:MGI Symbol%3BAcc:MGI:2677164]	ENSMUSG00000056054	S100a8	S100 calcium binding protein A8 (calgranulin A) [Source:MGI Symbol%3BAcc:MGI:88244	ENSMUSG00000001755	Coasy	Coenzyme A synthase [Source:MGI Symbol%3BAcc:MGI:1918993]	ENSMUSG00000025888	Casp1	caspase 1 [Source:MGI Symbol%3BAcc:MGI:96544]	ENSMUSG00000041736	Tspo	translocator protein [Source:MGI Symbol%3BAcc:MGI:88222]	ENSMUSG00000096936	Gm3510	predicted gene 3510 [Source:MGI Symbol%3BAcc:MGI:3781687]			
ENSMUSG00000113179	1700030L22Rik	RIKEN cDNA 1700030L22 gene [Source:MGI Symbol%3BAcc:MGI:1922826]	ENSMUSG00000110070	Gm45619	predicted gene 45619 [Source:MGI Symbol%3BAcc:MGI:5791455]	ENSMUSG00000002006	Pdzd4	PDZ domain containing 4 [Source:MGI Symbol%3BAcc:MGI:2443483]	ENSMUSG00000034738	Nostrin	nitric oxide synthase trafficker [Source:MGI Symbol%3BAcc:MGI:3606242]	ENSMUSG00000062098	Btbd3	BTB (POZ) domain containing 3 [Source:MGI Symbol%3BAcc:MGI:2385155]	ENSMUSG00000024079	Eif2ak2	eukaryotic translation initiation factor 2-alpha kinase 2 [Source:MGI Symbol%3BAcc:MGI:1353449]			
ENSMUSG00002076760	5S_rRNA	5S ribosomal RNA [Source:RFAM%3BAcc:RF00001]	ENSMUSG00000050359	Sprr1a	small proline-rich protein 1A [Source:MGI Symbol%3BAcc:MGI:106660]	ENSMUSG00000002032	Tmem25	transmembrane protein 25 [Source:MGI Symbol%3BAcc:MGI:1918937]	ENSMUSG00000067929	Gm10226	predicted gene 10226 [Source:MGI Symbol%3BAcc:MGI:3704470]	ENSMUSG00000056158	Car10	carbonic anhydrase 10 [Source:MGI Symbol%3BAcc:MGI:1919855]	ENSMUSG00000066245	Gm10156	predicted gene 10156 [Source:MGI Symbol%3BAcc:MGI:3642344]			
ENSMUSG00000031085	Acte1	actin%2C epsilon 1 [Source:MGI Symbol%3BAcc:MGI:2685344]	ENSMUSG00000032484	Ngp	neutrophilic granule protein [Source:MGI Symbol%3BAcc:MGI:105983]	ENSMUSG00000002266	Zim1	zinc finger%2C imprinted 1 [Source:MGI Symbol%3BAcc:MGI:1341879]	ENSMUSG00000097535	Gm26592	predicted gene%2C 26592 [Source:MGI Symbol%3BAcc:MGI:5477086]	ENSMUSG00000106904	Gm43029	predicted gene 43029 [Source:MGI Symbol%3BAcc:MGI:5663166]	ENSMUSG00000025407	Gli1	GLI-Kruppel family member GLI1 [Source:MGI Symbol%3BAcc:MGI:95727]			
ENSMUSG00000120513	-	novel transcript	ENSMUSG00000029727	Cyp3a13	cytochrome P450%2C family 3%2C subfamily a%2C polypeptide 13 [Source:MGI Symbol%3BAcc:MGI:88610	ENSMUSG00000002332	Dhrs1	dehydrogenase/reductase (SDR family) member 1 [Source:MGI Symbol%3BAcc:MGI:1196314]	ENSMUSG00000056023	Gm9989	predicted gene 9989 [Source:MGI Symbol%3BAcc:MGI:3642361]	ENSMUSG00000038732	Mboat1	membrane bound O-acyltransferase domain containing 1 [Source:MGI Symbol%3BAcc:MGI:2387184]	ENSMUSG00000086291	Gm15513	predicted gene 15513 [Source:MGI Symbol%3BAcc:MGI:3782961]			
ENSMUSG00000087061	A130030D18Rik	RIKEN cDNA A130030D18 gene [Source:MGI Symbol%3BAcc:MGI:2685404]	ENSMUSG00000021587	Pcsk1	proprotein convertase subtilisin/kexin type 1 [Source:MGI Symbol%3BAcc:MGI:97511	ENSMUSG00000002603	Tgfb1	transforming growth factor%2C beta 1 [Source:MGI Symbol%3BAcc:MGI:98725	ENSMUSG00000057457	Phex	phosphate regulating endopeptidase homolog%2C X-linked [Source:MGI	ENSMUSG00000030110	Ret	ret proto-oncogene [Source:MGI Symbol%3BAcc:MGI:97902]	ENSMUSG00000093507	Gm20627	predicted gene 20627 [Source:MGI Symbol%3BAcc:MGI:5313074]			

ENSMUSG00000118000	4933403J19Rik	RIKEN cDNA 4933403J19 gene [Source:MGI Symbol%3BAcc:MGI:1918271]	ENSMUSG00000052776	Oas1a	2'-5' oligoadenylate synthetase 1A [Source:MGI Symbol%3BAcc:MGI:21808	ENSMUSG00000002732	Fkbp7	FK506 binding protein 7 [Source:MGI Symbol%3BAcc:MGI:1336879]	ENSMUSG00000006310	Zbtb32	zinc finger and BTB domain containing 32 [Source:MGI Symbol%3BAcc:MGI:18918	ENSMUSG00000001496	Nkx2-1	NK2 homeobox 1 [Source:MGI Symbol%3BAcc:MGI:108067]	ENSMUSG00000040296	Ddx58	DEAD/H box helicase 58 [Source:MGI Symbol%3BAcc:MGI:2442858]
ENSMUSG00000105877	Gm42479	predicted gene 42479 [Source:MGI Symbol%3BAcc:MGI:5662616]	ENSMUSG00000074006	Omp	olfactory marker protein [Source:MGI Symbol%3BAcc:MGI:97436]	ENSMUSG00000002771	Grin2d	glutamate receptor%2C ionotropic%2C NMDA2D (epsilon 4) [Source:MGI Symbol%3BAcc:MGI:95823]	ENSMUSG00000112168	Gm34776	predicted gene%2C 34776 [Source:MGI Symbol%3BAcc:MGI:5593935]	ENSMUSG00000007030	Vwa7	von Willebrand factor A domain containing 7 [Source:MGI Symbol%3BAcc:MGI:1306798]	ENSMUSG00000032744	Heyl	hairy/enhancer-of-split related with YRPW motif-like [Source:MGI Symbol%3BAcc:MGI:1860511]
ENSMUSG00000087090	Nctc1	non-coding transcript 1 [Source:MGI Symbol%3BAcc:MGI:1306816]	ENSMUSG00000035208	Slfn8	schlafen 8 [Source:MGI Symbol%3BAcc:MGI:2672859]	ENSMUSG00000029925	Tbxas1	thromboxane A synthase 1%2C platelet [Source:MGI Symbol%3BAcc:MGI:98497]	ENSMUSG00000120522	-	novel transcript	ENSMUSG00000086006	Gm13293	predicted gene 13293 [Source:MGI Symbol%3BAcc:MGI:3649469]	ENSMUSG00000048502	Duxbl1	double homeobox B-like 1 [Source:MGI Symbol%3BAcc:MGI:1916048]
ENSMUSG00000040283	Btnl9	butyrophilin-like 9 [Source:MGI Symbol%3BAcc:MGI:2442439]	ENSMUSG00000068245	Phf11d	PHD finger protein 11D [Source:MGI Symbol%3BAcc:MGI:1277133]	ENSMUSG00000003031	Cdkn1b	cyclin-dependent kinase inhibitor 1B [Source:MGI Symbol%3BAcc:MGI:104565]	ENSMUSG00000056316	Gm9996	predicted gene 9996 [Source:MGI Symbol%3BAcc:MGI:3642549]	ENSMUSG00000054986	Sec14l3	SEC14-like lipid binding 3 [Source:MGI Symbol%3BAcc:MGI:3617848]	ENSMUSG00000045730	Adrb2	adrenergic receptor%2C beta 2 [Source:MGI Symbol%3BAcc:MGI:87938]
ENSMUSG00000030165	Klrd1	killer cell lectin-like receptor%2C subfamily D%2C member 1 [Source:MGI Symbol%3BAcc:MGI:11962	ENSMUSG00000072774	Zfp951	zinc finger protein 951 [Source:MGI Symbol%3BAcc:MGI:2441896]	ENSMUSG00000003657	Calb2	calbindin 2 [Source:MGI Symbol%3BAcc:MGI:101914]	ENSMUSG00000079363	Gbp4	guanylate binding protein 4 [Source:MGI Symbol%3BAcc:MGI:97072]	ENSMUSG00000067724	Gbx1	gastrulation brain homeobox 1 [Source:MGI Symbol%3BAcc:MGI:95667]	ENSMUSG00000018822	Sfrp5	secreted frizzled-related sequence protein 5 [Source:MGI Symbol%3BAcc:MGI:1860298]
ENSMUSG00000085553	Gm14808	predicted gene 14808 [Source:MGI Symbol%3BAcc:MGI:3705252]	ENSMUSG00000106734	Gm20559	predicted gene%2C 20559 [Source:MGI Symbol%3BAcc:MGI:5295666]	ENSMUSG00000003808	Farsa	phenylalanyl-tRNA synthetase%2C alpha subunit [Source:MGI Symbol%3BAcc:MGI:1913840]	ENSMUSG00000094732	1500015L24Rik	RIKEN cDNA 1500015L24 gene [Source:MGI Symbol%3BAcc:MGI:1916244]	ENSMUSG00000073418	C4b	complement component 4B (Chido blood group) [Source:MGI Symbol%3BAcc:MGI:88228]	ENSMUSG00000120908	-	novel transcript
ENSMUSG00000083767	Gm11405	predicted gene 11405 [Source:MGI Symbol%3BAcc:MGI:3651226]	ENSMUSG00000085167	Gm12063	predicted gene 12063 [Source:MGI Symbol%3BAcc:MGI:3649572]	ENSMUSG00000029771	Irf5	interferon regulatory factor 5 [Source:MGI Symbol%3BAcc:MGI:1350924]	ENSMUSG00000095366	Gm21860	predicted gene%2C 21860 [Source:MGI Symbol%3BAcc:MGI:5434024]	ENSMUSG00000108393	Gm32633	predicted gene%2C 32633 [Source:MGI Symbol%3BAcc:MGI:5591792]	ENSMUSG00000120954	-	novel transcript
ENSMUSG00000107197	Gm43312	predicted gene 43312 [Source:MGI Symbol%3BAcc:MGI:5663449]	ENSMUSG00000090675	Olfr111	olfactory receptor 111 [Source:MGI Symbol%3BAcc:MGI:2177494]	ENSMUSG00000069633	Pex11g	peroxisomal biogenesis factor 11 gamma [Source:MGI Symbol%3BAcc:MGI:1920905]	ENSMUSG00000086389	Gm15998	predicted gene 15998 [Source:MGI Symbol%3BAcc:MGI:3802094]	ENSMUSG00000032343	Impg1	interphotoreceptor matrix proteoglycan 1 [Source:MGI Symbol%3BAcc:MGI:1926876]	ENSMUSG00000054545	Ugt1a6a	UDP glucuronosyltransferase 1 family%2C polypeptide A6A [Source:MGI Symbol%3BAcc:MGI:21376
ENSMUSG00000040966	Slc22a2	solute carrier family 22 (organic cation transporter)%2C member 2 [Source:MGI Symbol%3BAcc:MGI:1335072]	ENSMUSG00000069132	Nxph2	neurexophilin 2 [Source:MGI Symbol%3BAcc:MGI:107491]	ENSMUSG00000005357	Slc1a6	solute carrier family 1 (high affinity aspartate/glutamate transporter)%2C member 6 [Source:MGI Symbol%3BAcc:MGI:10963	ENSMUSG00000023267	Gabrr2	gamma-aminobutyric acid (GABA) C receptor%2C subunit rho 2 [Source:MGI Symbol%3BAcc:MGI:95626]	ENSMUSG00000029762	Akr1b8	aldo-keto reductase family 1%2C member B8 [Source:MGI Symbol%3BAcc:MGI:107673]	ENSMUSG00000108197	Gm44214	predicted gene%2C 44214 [Source:MGI Symbol%3BAcc:MGI:5690606]
ENSMUSG00000083283	Gm15361	predicted pseudogene 15361 [Source:MGI Symbol%3BAcc:MGI:3642247]	ENSMUSG00000099875	Rbm3-ps	RNA binding motif (RNP1%2C RRM) protein 3 [Source:MGI Symbol%3BAcc:MGI:3642874]	ENSMUSG00000005373	Mlxipl	MLX interacting protein-like [Source:MGI Symbol%3BAcc:MGI:1927999]	ENSMUSG00000040919	4930505A04Rik	RIKEN cDNA 4930505A04 gene [Source:MGI Symbol%3BAcc:MGI:1922337]	ENSMUSG00000089665	Fcor	Foxo1 corepressor [Source:MGI Symbol%3BAcc:MGI:1915484]	ENSMUSG00000028307	Aldob	aldolase B%2C fructose-bisphosphate [Source:MGI Symbol%3BAcc:MGI:87995]
ENSMUSG00000120802	-	novel transcript	ENSMUSG00000027580	Helz2	helicase with zinc finger 2%2C transcriptional coactivator [Source:MGI Symbol%3BAcc:MGI:2385169]	ENSMUSG00000005575	Ube2m	ubiquitin-conjugating enzyme E2M [Source:MGI Symbol%3BAcc:MGI:108278]	ENSMUSG00000095134	Mid1-ps1	midline 1%2C pseudogene 1 [Source:MGI Symbol%3BAcc:MGI:5780070]				ENSMUSG00000023031	Cela1	chymotrypsin-like elastase family%2C member 1 [Source:MGI Symbol%3BAcc:MGI:95314]
ENSMUSG00000106071	Gm43826	predicted gene 43826 [Source:MGI Symbol%3BAcc:MGI:5663963]	ENSMUSG00000022769	Sdf2l1	stromal cell-derived factor 2-like 1 [Source:MGI Symbol%3BAcc:MGI:2149842]	ENSMUSG00000005583	Mef2c	myocyte enhancer factor 2C [Source:MGI Symbol%3BAcc:MGI:99458]	ENSMUSG00000117893	Gm46658	predicted gene%2C 46658 [Source:MGI Symbol%3BAcc:MGI:5826295]				ENSMUSG00000069270	H2ac6	H2A clustered histone 6 [Source:MGI Symbol%3BAcc:MGI:2448287]
ENSMUSG00000005696	Sh2d1a	SH2 domain containing 1A [Source:MGI Symbol%3BAcc:MGI:1328352]	ENSMUSG00000026582	Sele	selectin%2C endothelial cell [Source:MGI Symbol%3BAcc:MGI:98278]	ENSMUSG00000005716	Pvalb	parvalbumin [Source:MGI Symbol%3BAcc:MGI:97821]	ENSMUSG00000117023	Gm49876	predicted gene%2C 49876 [Source:MGI Symbol%3BAcc:MGI:6270555]				ENSMUSG00000064215	Ifi27	interferon%2C alpha-inducible protein 27 [Source:MGI Symbol%3BAcc:MGI:1277180]
ENSMUSG00000106342	Gm42891	predicted gene 42891 [Source:MGI Symbol%3BAcc:MGI:5663028]	ENSMUSG00000087263	Gm15726	predicted gene 15726 [Source:MGI Symbol%3BAcc:MGI:3783169]	ENSMUSG00000005718	Tfap4	transcription factor AP4 [Source:MGI Symbol%3BAcc:MGI:103239]	ENSMUSG00000082932	Cyp2j8	cytochrome P450%2C family 2%2C subfamily j%2C polypeptide 8 [Source:MGI Symbol%3BAcc:MGI:24498						
ENSMUSG00000084024	Gm15937	predicted gene 15937 [Source:MGI Symbol%3BAcc:MGI:3802031]	ENSMUSG00000016194	Hsd11b1	hydroxysteroid 11-beta dehydrogenase 1 [Source:MGI Symbol%3BAcc:MGI:10356	ENSMUSG00000005892	Trh	thyrotropin releasing hormone [Source:MGI Symbol%3BAcc:MGI:98823]	ENSMUSG00000025014	Dntt	deoxynucleotidyltransferase%2C terminal [Source:MGI Symbol%3BAcc:MGI:98659						
ENSMUSG00000075370	Igll1	immunoglobulin lambda-like polypeptide 1 [Source:MGI Symbol%3BAcc:MGI:96529	ENSMUSG00000032690	Oas2	2'-5' oligoadenylate synthetase 2 [Source:MGI Symbol%3BAcc:MGI:2180852]	ENSMUSG00000020128	Vps54	VPS54 GARP complex subunit [Source:MGI Symbol%3BAcc:MGI:2178798]	ENSMUSG00000076621	Ighj1	immunoglobulin heavy joining 1 [Source:MGI Symbol%3BAcc:MGI:4439802]						
ENSMUSG00000058773	H1f5	H1.5 linker histone%2C cluster member [Source:MGI Symbol%3BAcc:MGI:18614	ENSMUSG00000028037	Ifi44	interferon-induced protein 44 [Source:MGI Symbol%3BAcc:MGI:2443016]	ENSMUSG00000006281	Tep1	telomerase associated protein 1 [Source:MGI Symbol%3BAcc:MGI:109573]									
ENSMUSG00000023902	Zscan10	zinc finger and SCAN domain containing 10 [Source:MGI Symbol%3BAcc:MGI:3040700]	ENSMUSG00000034774	Dsg1c	desmoglein 1 gamma [Source:MGI Symbol%3BAcc:MGI:2664358]	ENSMUSG00000035274	Tpbg	trophoblast glycoprotein [Source:MGI Symbol%3BAcc:MGI:1341264]									
ENSMUSG00000108055	Gm43876	predicted gene%2C 43876 [Source:MGI Symbol%3BAcc:MGI:5690268]	ENSMUSG00000090877	Hspa1b	heat shock protein 1B [Source:MGI Symbol%3BAcc:MGI:99517]	ENSMUSG00000006463	Zdhhc24	zinc finger%2C DHHC domain containing 24 [Source:MGI Symbol%3BAcc:MGI:1917855]									
ENSMUSG00000103552	Gm38217	predicted gene%2C 38217 [Source:MGI Symbol%3BAcc:MGI:5611445]	ENSMUSG00000032496	Ltf	lactotransferrin [Source:MGI Symbol%3BAcc:MGI:96837]	ENSMUSG00000006587	Snai3	snail family zinc finger 3 [Source:MGI Symbol%3BAcc:MGI:1353563]									
ENSMUSG00000104753	Gm35172	predicted gene%2C 35172 [Source:MGI Symbol%3BAcc:MGI:5594331]	ENSMUSG00000025498	Irf7	interferon regulatory factor 7 [Source:MGI Symbol%3BAcc:MGI:1859212]	ENSMUSG00000007039	Ddah2	dimethylarginine dimethylaminohydrolase 2 [Source:MGI Symbol%3BAcc:MGI:1859016]									
ENSMUSG00000117979	Gm50341	predicted gene%2C 50341 [Source:MGI Symbol%3BAcc:MGI:6303218]	ENSMUSG00000026963	Tmem210	transmembrane protein 210 [Source:MGI Symbol%3BAcc:MGI:1925467]	ENSMUSG00000007041	Clic1	chloride intracellular channel 1 [Source:MGI Symbol%3BAcc:MGI:2148924]									
ENSMUSG00000055210	Foxd2	forkhead box D2 [Source:MGI Symbol%3BAcc:MGI:1347471]	ENSMUSG00000075514	Gm13375	predicted gene 13375 [Source:MGI Symbol%3BAcc:MGI:3649913]	ENSMUSG00000007837	Prrg2	proline-rich Gla (G-carboxyglutamic acid) polypeptide 2 [Source:MGI Symbol%3BAcc:MGI:19295									

ENSMUSG00000029445	Hpd	4-hydroxyphenylpyruvic acid dioxygenase [Source:MGI Symbol%3BAcc:MGI:96213	ENSMUSG00000061577	Adgrg5	adhesion G protein-coupled receptor G5 [Source:MGI Symbol%3BAcc:MGI:26859	ENSMUSG00000007872	Id3	inhibitor of DNA binding 3 [Source:MGI Symbol%3BAcc:MGI:96398]
ENSMUSG00000113224	Gm48444	predicted gene%2C 48444 [Source:MGI Symbol%3BAcc:MGI:6097954]	ENSMUSG00000032487	Ptgs2	prostaglandin-endoperoxide synthase 2 [Source:MGI Symbol%3BAcc:MGI:97798	ENSMUSG00000008540	Mgst1	microsomal glutathione S-transferase 1 [Source:MGI Symbol%3BAcc:MGI:19138
ENSMUSG00000090628	Gm17083	predicted gene 17083 [Source:MGI Symbol%3BAcc:MGI:4937910]	ENSMUSG00000035299	Mid1	midline 1 [Source:MGI Symbol%3BAcc:MGI:1100537]	ENSMUSG00000027895	Kcnc4	potassium voltage gated channel%2C Shaw-related subfamily%2C member 4 [Source:MGI Symbol%3BAcc:MGI:96670
ENSMUSG00000001508	Sgca	sarcoglycan%2C alpha (dystrophin-associated glycoprotein) [Source:MGI Symbol%3BAcc:MGI:89469	ENSMUSG00000099583	H3c4	H3 clustered histone 4 [Source:MGI Symbol%3BAcc:MGI:2448322]	ENSMUSG00000009876	Cox4i2	cytochrome c oxidase subunit 4I2 [Source:MGI Symbol%3BAcc:MGI:2135755]
ENSMUSG00000053375	Atp6v1e2	ATPase%2C H+ transporting%2C lysosomal V1 subunit E2 [Source:MGI Symbol%3BAcc:MGI:1922165]	ENSMUSG00000037921	Ddx60	DExD/H box helicase 60 [Source:MGI Symbol%3BAcc:MGI:2384570]	ENSMUSG00000010142	Tnfrsf13b	tumor necrosis factor receptor superfamily%2C member 13b [Source:MGI Symbol%3BAcc:MGI:1889411]
ENSMUSG00000029595	Lhx5	LIM homeobox protein 5 [Source:MGI Symbol%3BAcc:MGI:107792]	ENSMUSG00000091408	Gm6728	predicted gene 6728 [Source:MGI Symbol%3BAcc:MGI:3643418]	ENSMUSG00000010358	Ifi35	interferon-induced protein 35 [Source:MGI Symbol%3BAcc:MGI:1917360]
ENSMUSG00000075217	Fads2b	fatty acid desaturase 2B [Source:MGI Symbol%3BAcc:MGI:2687041]	ENSMUSG00000111994	A330049N07Rik	RIKEN cDNA A330049N07 gene [Source:MGI Symbol%3BAcc:MGI:3041175]	ENSMUSG00000010529	Gm266	predicted gene 266 [Source:MGI Symbol%3BAcc:MGI:2685112]
ENSMUSG00000102831	Gm37382	predicted gene%2C 37382 [Source:MGI Symbol%3BAcc:MGI:5610610]	ENSMUSG00000075591	Gm10874	predicted gene 10874 [Source:MGI Symbol%3BAcc:MGI:3704261]	ENSMUSG00000012296	Tjap1	tight junction associated protein 1 [Source:MGI Symbol%3BAcc:MGI:1921344]
ENSMUSG00000046814	Gchfr	GTP cyclohydrolase I feedback regulator [Source:MGI Symbol%3BAcc:MGI:2443977]	ENSMUSG00000050211	Pla2g4e	phospholipase A2%2C group IVE [Source:MGI Symbol%3BAcc:MGI:1919144]	ENSMUSG00000015202	Cnksr3	Cnksr family member 3 [Source:MGI Symbol%3BAcc:MGI:2674130]
ENSMUSG00000064201	Krt2	keratin 2 [Source:MGI Symbol%3BAcc:MGI:96699]	ENSMUSG00000026072	Il1r1	interleukin 1 receptor%2C type I [Source:MGI Symbol%3BAcc:MGI:96545	ENSMUSG00000028111	Ctsk	cathepsin K [Source:MGI Symbol%3BAcc:MGI:107823]
ENSMUSG00000110929	Gm47757	predicted gene%2C 47757 [Source:MGI Symbol%3BAcc:MGI:6096905]	ENSMUSG00000000204	Slfn4	schlafen 4 [Source:MGI Symbol%3BAcc:MGI:1329010]	ENSMUSG00000015745	Plekho1	pleckstrin homology domain containing%2C family O member 1 [Source:MGI Symbol%3BAcc:MGI:19144
ENSMUSG00000028707	Dmbx1	diencephalon/mesencephalon homeobox 1 [Source:MGI Symbol%3BAcc:MGI:21535	ENSMUSG00000108308	Gm45218	predicted gene 45218 [Source:MGI Symbol%3BAcc:MGI:5753794]	ENSMUSG00000016024	Lbp	lipopolysaccharide binding protein [Source:MGI Symbol%3BAcc:MGI:10987
ENSMUSG00000062905	Vmn1r32	vomeronasal 1 receptor 32 [Source:MGI Symbol%3BAcc:MGI:2159451]	ENSMUSG00000017830	Dhx58	DEXH (Asp-Glu-X-His) box polypeptide 58 [Source:MGI Symbol%3BAcc:MGI:19315	ENSMUSG00000041132	N4bp2l1	NEDD4 binding protein 2-like 1 [Source:MGI Symbol%3BAcc:MGI:2140872]
ENSMUSG00000045362	Tnfrsf26	tumor necrosis factor receptor superfamily%2C member 26 [Source:MGI Symbol%3BAcc:MGI:2651928]	ENSMUSG00000113450	Zfp935	zinc finger protein 935 [Source:MGI Symbol%3BAcc:MGI:1918758]	ENSMUSG00000016487	Ppfibp1	PTPRF interacting protein%2C binding protein 1 (liprin beta 1) [Source:MGI Symbol%3BAcc:MGI:19147
ENSMUSG00000106815	Gm43658	predicted gene 43658 [Source:MGI Symbol%3BAcc:MGI:5663795]	ENSMUSG00000086013	Gm15706	predicted gene 15706 [Source:MGI Symbol%3BAcc:MGI:3783146]	ENSMUSG00000016520	Lnx2	ligand of numb-protein X 2 [Source:MGI Symbol%3BAcc:MGI:2155959]
ENSMUSG00000101242	Gm28319	predicted gene 28319 [Source:MGI Symbol%3BAcc:MGI:5579025]	ENSMUSG00000083718	Ccnb2-ps	cyclin B2%2C pseudogene [Source:MGI Symbol%3BAcc:MGI:3779896]	ENSMUSG00000006519	Cyba	cytochrome b-245%2C alpha polypeptide [Source:MGI Symbol%3BAcc:MGI:1316658]
ENSMUSG00000085638	Gm15521	predicted gene 15521 [Source:MGI Symbol%3BAcc:MGI:3782968]	ENSMUSG00000005107	Slc2a9	solute carrier family 2 (facilitated glucose transporter)%2C member 9 [Source:MGI Symbol%3BAcc:MGI:2152844]	ENSMUSG00000020707	Rnf135	ring finger protein 135 [Source:MGI Symbol%3BAcc:MGI:1919206]
ENSMUSG00000085977	Gm5970	predicted gene 5970 [Source:MGI Symbol%3BAcc:MGI:3646761]	ENSMUSG00000026580	Selp	selectin%2C platelet [Source:MGI Symbol%3BAcc:MGI:98280]	ENSMUSG00000017756	Slc12a7	solute carrier family 12%2C member 7 [Source:MGI Symbol%3BAcc:MGI:1342283]
ENSMUSG00000120832	-	novel transcript	ENSMUSG00000043263	Ifi209	interferon activated gene 209 [Source:MGI Symbol%3BAcc:MGI:2138243]	ENSMUSG00000017801	Mlx	MAX-like protein X [Source:MGI Symbol%3BAcc:MGI:108398]
ENSMUSG00000029373	Pf4	platelet factor 4 [Source:MGI Symbol%3BAcc:MGI:1888711]	ENSMUSG00000021765	Fst	follistatin [Source:MGI Symbol%3BAcc:MGI:95586]	ENSMUSG00000020828	Pld2	phospholipase D2 [Source:MGI Symbol%3BAcc:MGI:892877]
ENSMUSG00000095318	Gm8024	predicted gene 8024 [Source:MGI Symbol%3BAcc:MGI:3647989]	ENSMUSG00000050121	Opalin	oligodendrocytic myelin paranodal and inner loop protein [Source:MGI Symbol%3BAcc:MGI:2657025]	ENSMUSG00000020388	Pdlim4	PDZ and LIM domain 4 [Source:MGI Symbol%3BAcc:MGI:1353470]
ENSMUSG00000044199	S1pr4	sphingosine-1-phosphate receptor 4 [Source:MGI Symbol%3BAcc:MGI:1333809]	ENSMUSG00000039236	Isg20	interferon-stimulated protein [Source:MGI Symbol%3BAcc:MGI:1928895]	ENSMUSG00000018774	Cd68	CD68 antigen [Source:MGI Symbol%3BAcc:MGI:88342]
ENSMUSG00000094462	Gm21028	predicted gene%2C 21028 [Source:MGI Symbol%3BAcc:MGI:5434383]	ENSMUSG00000116594	Gm49601	predicted gene%2C 49601 [Source:MGI Symbol%3BAcc:MGI:6215011]	ENSMUSG00000018927	Ccl6	chemokine (C-C motif) ligand 6 [Source:MGI Symbol%3BAcc:MGI:98263]
ENSMUSG00000120535	-	novel transcript	ENSMUSG00000112146	Gm46210	predicted gene%2C 46210 [Source:MGI Symbol%3BAcc:MGI:5825847]	ENSMUSG00000019122	Ccl9	chemokine (C-C motif) ligand 9 [Source:MGI Symbol%3BAcc:MGI:104533]

ENSMUSG00000102856	Gm37084	predicted gene%2C 37084 [Source:MGI Symbol%3BAcc:MGI:5610312]	ENSMUSG00000113536	Gm49327	predicted gene%2C 49327 [Source:MGI Symbol%3BAcc:MGI:6121512]	ENSMUSG00000019832	Rab32	RAB32%2C member RAS oncogene family [Source:MGI Symbol%3BAcc:MGI:1915094]
ENSMUSG00000093390	Gm20652	predicted gene 20652 [Source:MGI Symbol%3BAcc:MGI:5313099]	ENSMUSG00000081126	Gm15784	predicted gene 15784 [Source:MGI Symbol%3BAcc:MGI:3783226]	ENSMUSG00000019890	Nts	neurotensin [Source:MGI Symbol%3BAcc:MGI:1328351]
ENSMUSG00000032725	Folr2	folate receptor 2 (fetal) [Source:MGI Symbol%3BAcc:MGI:95569]	ENSMUSG00000079343	C1s2	complement component 1%2C s subcomponent 2 [Source:MGI Symbol%3BAcc:MGI:3644269]	ENSMUSG00000019944	Rhobtb1	Rho-related BTB domain containing 1 [Source:MGI Symbol%3BAcc:MGI:1916538]
ENSMUSG00000117350	Gm49975	predicted gene%2C 49975 [Source:MGI Symbol%3BAcc:MGI:6275248]	ENSMUSG00000031430	Vsig1	V-set and immunoglobulin domain containing 1 [Source:MGI Symbol%3BAcc:MGI:1926039]	ENSMUSG00000019988	Nedd1	neural precursor cell expressed%2C developmentally down-regulated gene 1 [Source:MGI Symbol%3BAcc:MGI:97293
ENSMUSG00000044172	Ptx4	pentraxin 4 [Source:MGI Symbol%3BAcc:MGI:1915759]	ENSMUSG00000104728	Gm42462	predicted gene 42462 [Source:MGI Symbol%3BAcc:MGI:5662599]	ENSMUSG00000020022	Ndufa12	NADH:ubiquinone oxidoreductase subunit A12 [Source:MGI Symbol%3BAcc:MGI:1913664]
ENSMUSG00000111598	Gm47095	predicted gene%2C 47095 [Source:MGI Symbol%3BAcc:MGI:6095827]	ENSMUSG00000102317	Gm37628	predicted gene%2C 37628 [Source:MGI Symbol%3BAcc:MGI:5610856]	ENSMUSG00000020098	Pcbd1	pterin 4 alpha carbinolamine dehydratase/dimerization cofactor of hepatocyte nuclear factor 1 alpha (TCF1) 1 [Source:MGI Symbol%3BAcc:MGI:94873
ENSMUSG00000103331	Gm37995	predicted gene%2C 37995 [Source:MGI Symbol%3BAcc:MGI:5611223]	ENSMUSG00000040113	Mettl11b	methyltransferase like 11B [Source:MGI Symbol%3BAcc:MGI:2685053]	ENSMUSG00000020120	Plek	pleckstrin [Source:MGI Symbol%3BAcc:MGI:1860485]
ENSMUSG00000087541	Hopxos	HOP homeobox%2C opposite strand [Source:MGI Symbol%3BAcc:MGI:3801917]	ENSMUSG00000117301	Gm35883	predicted gene%2C 35883 [Source:MGI Symbol%3BAcc:MGI:5595042]	ENSMUSG00000020256	Aldh1l2	aldehyde dehydrogenase 1 family%2C member L2 [Source:MGI Symbol%3BAcc:MGI:2444680]
ENSMUSG00000095195	Gm3005	predicted gene 3005 [Source:MGI Symbol%3BAcc:MGI:3781183]	ENSMUSG00000020131	Pcsk4	proprotein convertase subtilisin/kexin type 4 [Source:MGI Symbol%3BAcc:MGI:97514	ENSMUSG00000020287	Mpg	N-methylpurine-DNA glycosylase [Source:MGI Symbol%3BAcc:MGI:97073]
ENSMUSG00000048368	Omd	osteomodulin [Source:MGI Symbol%3BAcc:MGI:1350918]	ENSMUSG00000109981	Gm36849	predicted gene%2C 36849 [Source:MGI Symbol%3BAcc:MGI:5596008]	ENSMUSG00000054206	Gzmm	granzyme M (lymphocyte met-ase 1) [Source:MGI Symbol%3BAcc:MGI:99549]
ENSMUSG00000052180	Serpinb6c	serine (or cysteine) peptidase inhibitor%2C clade B%2C member 6c [Source:MGI Symbol%3BAcc:MGI:2145481]	ENSMUSG00000034997	Htr2a	5-hydroxytryptamine (serotonin) receptor 2A [Source:MGI Symbol%3BAcc:MGI:109521]	ENSMUSG00000020329	Polrmt	polymerase (RNA) mitochondrial (DNA directed) [Source:MGI Symbol%3BAcc:MGI:1915843]
ENSMUSG00000040329	Il7	interleukin 7 [Source:MGI Symbol%3BAcc:MGI:96561]	ENSMUSG00000090113	Nhlrc4	NHL repeat containing 4 [Source:MGI Symbol%3BAcc:MGI:3687200]	ENSMUSG00000020467	Efemp1	epidermal growth factor-containing fibulin-like extracellular matrix protein 1 [Source:MGI Symbol%3BAcc:MGI:1339998]
ENSMUSG00000108132	Gm44175	predicted gene%2C 44175 [Source:MGI Symbol%3BAcc:MGI:5690567]	ENSMUSG00000034161	Scx	scleraxis [Source:MGI Symbol%3BAcc:MGI:102934]	ENSMUSG00000020649	Rrm2	ribonucleotide reductase M2 [Source:MGI Symbol%3BAcc:MGI:98181]
ENSMUSG00000031173	Otc	ornithine transcarbamylase [Source:MGI Symbol%3BAcc:MGI:97448]	ENSMUSG00000105572	Gm43300	predicted gene 43300 [Source:MGI Symbol%3BAcc:MGI:5663437]	ENSMUSG00000020696	Rffl	ring finger and FYVE like domain containing protein [Source:MGI Symbol%3BAcc:MGI:1914588]
ENSMUSG00000079507	H2-Q1	histocompatibility 2%2C Q region locus 1 [Source:MGI Symbol%3BAcc:MGI:95928]	ENSMUSG00000020227	Irak3	interleukin-1 receptor-associated kinase 3 [Source:MGI Symbol%3BAcc:MGI:1921164]	ENSMUSG00000020709	Adap2	ArfGAP with dual PH domains 2 [Source:MGI Symbol%3BAcc:MGI:2663075]
ENSMUSG00000086266	Igf2os	insulin-like growth factor 2%2C opposite strand [Source:MGI Symbol%3BAcc:MGI:1195257]	ENSMUSG00000097370	4930467K11Rik	RIKEN cDNA 4930467K11 gene [Source:MGI Symbol%3BAcc:MGI:1922178]	ENSMUSG00000078619	Smarcd2	SWI/SNF related%2C matrix associated%2C actin dependent regulator of chromatin%2C subfamily d%2C member 2 [Source:MGI Symbol%3BAcc:MGI:1933621]
ENSMUSG00000097754	Ptgs2os2	prostaglandin-endoperoxide synthase 2%2C opposite strand 2 [Source:MGI Symbol%3BAcc:MGI:5477181]	ENSMUSG00000013766	Ly6g6e	lymphocyte antigen 6 complex%2C locus G6E [Source:MGI Symbol%3BAcc:MGI:1917524]	ENSMUSG00000020805	Slc13a5	solute carrier family 13 (sodium-dependent citrate transporter)%2C member 5 [Source:MGI Symbol%3BAcc:MGI:3037150]
ENSMUSG00000029556	Hnf1a	HNF1 homeobox A [Source:MGI Symbol%3BAcc:MGI:98504]	ENSMUSG00000030935	Acsm3	acyl-CoA synthetase medium-chain family member 3 [Source:MGI Symbol%3BAcc:MGI:99538	ENSMUSG00000020808	Pimreg	PICALM interacting mitotic regulator [Source:MGI Symbol%3BAcc:MGI:1924434]
ENSMUSG00000091105	Mrto4-ps2	mRNA turnover 4%2C pseudogene 2 [Source:MGI Symbol%3BAcc:MGI:37795	ENSMUSG00000031780	Ccl17	chemokine (C-C motif) ligand 17 [Source:MGI Symbol%3BAcc:MGI:1329039]	ENSMUSG00000020836	Coro6	coronin 6 [Source:MGI Symbol%3BAcc:MGI:2183448]
ENSMUSG00000111369	Gm47043	predicted gene%2C 47043 [Source:MGI Symbol%3BAcc:MGI:6095746]	ENSMUSG00000114360	Gm48596	predicted gene%2C 48596 [Source:MGI Symbol%3BAcc:MGI:6098172]	ENSMUSG00000021023	Prorp	protein only RNase P catalytic subunit [Source:MGI Symbol%3BAcc:MGI:1913382]
ENSMUSG00000015316	Slamf1	signaling lymphocytic activation molecule family member 1 [Source:MGI Symbol%3BAcc:MGI:1351314]	ENSMUSG00000103947	Gm37345	predicted gene%2C 37345 [Source:MGI Symbol%3BAcc:MGI:5610573]	ENSMUSG00000021032	Ngb	neuroglobin [Source:MGI Symbol%3BAcc:MGI:2151886]

ENSMUSG00000102594	Gm38381	predicted gene%2C 38381 [Source:MGI Symbol%3BAcc:MGI:5611609]	ENSMUSG00000004951	Hspb1	heat shock protein 1 [Source:MGI Symbol%3BAcc:MGI:96240]	ENSMUSG00000021091	Serpina3n	serine (or cysteine) peptidase inhibitor%2C clade A%2C member 3N [Source:MGI Symbol%3BAcc:MGI:10504
ENSMUSG00000044378	Slc15a5	solute carrier family 15%2C member 5 [Source:MGI Symbol%3BAcc:MGI:3607714]	ENSMUSG00000030406	Gipr	gastric inhibitory polypeptide receptor [Source:MGI Symbol%3BAcc:MGI:13527	ENSMUSG00000021190	Lgmn	legumain [Source:MGI Symbol%3BAcc:MGI:1330838]
ENSMUSG00000103209	Gm37331	predicted gene%2C 37331 [Source:MGI Symbol%3BAcc:MGI:5610559]	ENSMUSG00000020325	Fstl3	follistatin-like 3 [Source:MGI Symbol%3BAcc:MGI:1890391]	ENSMUSG00000021287	Xrcc3	X-ray repair complementing defective repair in Chinese hamster cells 3 [Source:MGI Symbol%3BAcc:MGI:19215
ENSMUSG00000096963	B230364G03Rik	RIKEN cDNA B230364G03 gene [Source:MGI Symbol%3BAcc:MGI:3704350]	ENSMUSG00000050520	Cldn8	claudin 8 [Source:MGI Symbol%3BAcc:MGI:1859286]	ENSMUSG00000021423	Ly86	lymphocyte antigen 86 [Source:MGI Symbol%3BAcc:MGI:1321404]
ENSMUSG00000089769	Gm16574	predicted gene 16574 [Source:MGI Symbol%3BAcc:MGI:4414994]	ENSMUSG00000021207	Akr1c21	aldo-keto reductase family 1%2C member C21 [Source:MGI Symbol%3BAcc:MGI:1924587]	ENSMUSG00000021466	Ptch1	patched 1 [Source:MGI Symbol%3BAcc:MGI:105373]
ENSMUSG00000086179	Gm14317	predicted gene 14317 [Source:MGI Symbol%3BAcc:MGI:3651690]	ENSMUSG00000111360	Gm38642	predicted gene%2C 38642 [Source:MGI Symbol%3BAcc:MGI:5621527]	ENSMUSG00000021624	Cd180	CD180 antigen [Source:MGI Symbol%3BAcc:MGI:1194924]
ENSMUSG00000026697	Myoc	myocilin [Source:MGI Symbol%3BAcc:MGI:1202864]	ENSMUSG00000024778	Fas	Fas (TNF receptor superfamily member 6) [Source:MGI Symbol%3BAcc:MGI:95484	ENSMUSG00000021665	Hexb	hexosaminidase B [Source:MGI Symbol%3BAcc:MGI:96074]
ENSMUSG00000026686	Lmx1a	LIM homeobox transcription factor 1 alpha [Source:MGI Symbol%3BAcc:MGI:18885	ENSMUSG00000041255	Tmco5b	transmembrane and coiled-coil domains 5B [Source:MGI Symbol%3BAcc:MGI:19225	ENSMUSG00000021702	Thbs4	thrombospondin 4 [Source:MGI Symbol%3BAcc:MGI:1101779]
ENSMUSG00000021342	Prl	prolactin [Source:MGI Symbol%3BAcc:MGI:97762]	ENSMUSG00000034329	Brip1	BRCA1 interacting protein C-terminal helicase 1 [Source:MGI Symbol%3BAcc:MGI:2442836]	ENSMUSG00000021721	Htr1a	5-hydroxytryptamine (serotonin) receptor 1A [Source:MGI Symbol%3BAcc:MGI:96273]
ENSMUSG00000069456	Rdh16	retinol dehydrogenase 16 [Source:MGI Symbol%3BAcc:MGI:1201375]	ENSMUSG00000040140	Tdrd6	tudor domain containing 6 [Source:MGI Symbol%3BAcc:MGI:2679727]	ENSMUSG00000049985	Ankrd55	ankyrin repeat domain 55 [Source:MGI Symbol%3BAcc:MGI:1924568]
ENSMUSG00000037005	Xpnpep2	X-prolyl aminopeptidase (aminopeptidase P) 2%2C membrane-bound [Source:MGI Symbol%3BAcc:MGI:2180001]	ENSMUSG00000014444	Piezo1	piezo-type mechanosensitive ion channel component 1 [Source:MGI Symbol%3BAcc:MGI:3603204]	ENSMUSG00000021823	Vcl	vinculin [Source:MGI Symbol%3BAcc:MGI:98927]
ENSMUSG00000022993	Tex49	testis expressed 49 [Source:MGI Symbol%3BAcc:MGI:1921113]	ENSMUSG00000029371	Cxcl5	chemokine (C-X-C motif) ligand 5 [Source:MGI Symbol%3BAcc:MGI:1096868]	ENSMUSG00000021876	Rnase4	ribonuclease%2C RNase A family 4 [Source:MGI Symbol%3BAcc:MGI:1926217]
ENSMUSG00000099553	Gm29538	predicted gene 29538 [Source:MGI Symbol%3BAcc:MGI:5580244]	ENSMUSG00000000365	Rnf17	ring finger protein 17 [Source:MGI Symbol%3BAcc:MGI:1353419]	ENSMUSG00000022014	Epsti1	epithelial stromal interaction 1 (breast) [Source:MGI Symbol%3BAcc:MGI:19151
ENSMUSG00000115261	Gm35769	predicted gene%2C 35769 [Source:MGI Symbol%3BAcc:MGI:5594928]	ENSMUSG00000116793	Gm34256	predicted gene%2C 34256 [Source:MGI Symbol%3BAcc:MGI:5593415]	ENSMUSG00000022033	Pbk	PDZ binding kinase [Source:MGI Symbol%3BAcc:MGI:1289156]
ENSMUSG00000060787	Olfr464	olfactory receptor 464 [Source:MGI Symbol%3BAcc:MGI:3030298]	ENSMUSG00000087331	1810021B22Rik	RIKEN cDNA 1810021B22 gene [Source:MGI Symbol%3BAcc:MGI:1916370]	ENSMUSG00000022146	Osmr	oncostatin M receptor [Source:MGI Symbol%3BAcc:MGI:1330819]
ENSMUSG00000036687	Tmem184a	transmembrane protein 184a [Source:MGI Symbol%3BAcc:MGI:2385897]	ENSMUSG00000005763	Cd247	CD247 antigen [Source:MGI Symbol%3BAcc:MGI:88334]	ENSMUSG00000022364	Tbc1d31	TBC1 domain family%2C member 31 [Source:MGI Symbol%3BAcc:MGI:2684931]
ENSMUSG00000099284	Mir7026	microRNA 7026 [Source:MGI Symbol%3BAcc:MGI:5531370]	ENSMUSG00000082985	Gm14042	predicted gene 14042 [Source:MGI Symbol%3BAcc:MGI:3650956]	ENSMUSG00000022439	Parvg	parvin%2C gamma [Source:MGI Symbol%3BAcc:MGI:2158329]
ENSMUSG00000117290	Gm34883	predicted gene%2C 34883 [Source:MGI Symbol%3BAcc:MGI:5594042]	ENSMUSG00000075555	Gm10863	predicted gene 10863 [Source:MGI Symbol%3BAcc:MGI:3641623]	ENSMUSG00000022575	Gsdmd	gasdermin D [Source:MGI Symbol%3BAcc:MGI:1916396]
ENSMUSG00000086284	Frmpd1os	FERM and PDZ domain containing 1%2C opposite strand [Source:MGI Symbol%3BAcc:MGI:1925758]	ENSMUSG00000118263	Gm42064	predicted gene%2C 42064 [Source:MGI Symbol%3BAcc:MGI:5624949]	ENSMUSG00000075602	Ly6a	lymphocyte antigen 6 complex%2C locus A [Source:MGI Symbol%3BAcc:MGI:107527]
ENSMUSG00000103189	Gm37092	predicted gene%2C 37092 [Source:MGI Symbol%3BAcc:MGI:5610320]	ENSMUSG00000101166	Gm28496	predicted gene 28496 [Source:MGI Symbol%3BAcc:MGI:5579202]	ENSMUSG00000022906	Parp9	poly (ADP-ribose) polymerase family%2C member 9 [Source:MGI Symbol%3BAcc:MGI:1933117]
ENSMUSG00000021751	Acox2	acyl-Coenzyme A oxidase 2%2C branched chain [Source:MGI Symbol%3BAcc:MGI:1934852]	ENSMUSG00000086632	1700112J05Rik	RIKEN cDNA 1700112J05 gene [Source:MGI Symbol%3BAcc:MGI:3696786]	ENSMUSG00000022912	Pros1	protein S (alpha) [Source:MGI Symbol%3BAcc:MGI:1095733]
ENSMUSG00000087312	Gm13833	predicted gene 13833 [Source:MGI Symbol%3BAcc:MGI:3650920]	ENSMUSG00000073430	Gm10505	predicted gene 10505 [Source:MGI Symbol%3BAcc:MGI:3642687]	ENSMUSG00000022969	Il10rb	interleukin 10 receptor%2C beta [Source:MGI Symbol%3BAcc:MGI:109380]
ENSMUSG00000086103	Gm11832	predicted gene 11832 [Source:MGI Symbol%3BAcc:MGI:3650310]	ENSMUSG00000043501	Lgals2	lectin%2C galactose-binding%2C soluble 2 [Source:MGI Symbol%3BAcc:MGI:89506	ENSMUSG00000023064	Sncg	synuclein%2C gamma [Source:MGI Symbol%3BAcc:MGI:1298397]
ENSMUSG00000110685	Gm5373	predicted gene 5373 [Source:MGI Symbol%3BAcc:MGI:3779485]	ENSMUSG00000111938	2900045O20Rik	RIKEN cDNA 2900045O20 gene [Source:MGI Symbol%3BAcc:MGI:1920205]	ENSMUSG00000023066	Rttn	rotatin [Source:MGI Symbol%3BAcc:MGI:2179288]

ENSMUSG00000078091	Gm10912	predicted gene 10912 [Source:MGI Symbol%3BAcc:MGI:3809094]	ENSMUSG00000115023	Gm48967	predicted gene%2C 48967 [Source:MGI Symbol%3BAcc:MGI:6118304]	ENSMUSG00000023224	Serping1	serine (or cysteine) peptidase inhibitor%2C clade G%2C member 1 [Source:MGI Symbol%3BAcc:MGI:89469
ENSMUSG00000049241	Hcar1	hydrocarboxylic acid receptor 1 [Source:MGI Symbol%3BAcc:MGI:2441671]	ENSMUSG00000105741	C79130	expressed sequence C79130 [Source:MGI Symbol%3BAcc:MGI:2141307]	ENSMUSG00000023391	Dlx2	distal-less homeobox 2 [Source:MGI Symbol%3BAcc:MGI:94902]
ENSMUSG00000087440	4930577N17Rik	RIKEN cDNA 4930577N17 gene [Source:MGI Symbol%3BAcc:MGI:1914996]	ENSMUSG00000106450	Gm43018	predicted gene 43018 [Source:MGI Symbol%3BAcc:MGI:5663155]	ENSMUSG00000023911	Flywch2	FLYWCH family member 2 [Source:MGI Symbol%3BAcc:MGI:1924167]
ENSMUSG00000117546	-	TEC	ENSMUSG00000120721	-	novel transcript%2C antisense to Kcna3	ENSMUSG00000023992	Trem2	triggering receptor expressed on myeloid cells 2 [Source:MGI Symbol%3BAcc:MGI:1913150]
ENSMUSG00000061048	Cdh3	cadherin 3 [Source:MGI Symbol%3BAcc:MGI:88356]	ENSMUSG00000120154	-	novel transcript%2C antisense to Prr3	ENSMUSG00000024066	Xdh	xanthine dehydrogenase [Source:MGI Symbol%3BAcc:MGI:98973
ENSMUSG00000113330	Gm34237	predicted gene%2C 34237 [Source:MGI Symbol%3BAcc:MGI:5593396]	ENSMUSG00000102681	Gm37221	predicted gene%2C 37221 [Source:MGI Symbol%3BAcc:MGI:5610449]	ENSMUSG00000024076	Vit	vitrin [Source:MGI Symbol%3BAcc:MGI:1921449]
ENSMUSG00000107688	Gm44091	predicted gene%2C 44091 [Source:MGI Symbol%3BAcc:MGI:5690483]	ENSMUSG00000116739	Gm49733	predicted gene%2C 49733 [Source:MGI Symbol%3BAcc:MGI:6215218]	ENSMUSG00000117406	Ntn3	netrin 3 [Source:MGI Symbol%3BAcc:MGI:1341188]
ENSMUSG00000086283	2810433D01Rik	RIKEN cDNA 2810433D01 gene [Source:MGI Symbol%3BAcc:MGI:1924382]	ENSMUSG00000007617	Homer1	homer scaffolding protein 1 [Source:MGI Symbol%3BAcc:MGI:1347345]	ENSMUSG00000091845	Rpl36-ps12	ribosomal protein L36%2C pseudogene 12 [Source:MGI Symbol%3BAcc:MGI:37827
ENSMUSG00000096870	Gm21816	predicted gene%2C 21816 [Source:MGI Symbol%3BAcc:MGI:5433980]	ENSMUSG00000027801	Tm4sf4	transmembrane 4 superfamily member 4 [Source:MGI Symbol%3BAcc:MGI:2385173]	ENSMUSG00000061232	H2-K1	histocompatibility 2%2C K1%2C K region [Source:MGI Symbol%3BAcc:MGI:95904]
ENSMUSG00000109239	Gm45176	predicted gene 45176 [Source:MGI Symbol%3BAcc:MGI:5753752]	ENSMUSG00000035352	Ccl12	chemokine (C-C motif) ligand 12 [Source:MGI Symbol%3BAcc:MGI:108224]	ENSMUSG00000024334	H2-Oa	histocompatibility 2%2C O region alpha locus [Source:MGI Symbol%3BAcc:MGI:95924
ENSMUSG00000001739	Cldn15	claudin 15 [Source:MGI Symbol%3BAcc:MGI:1913103]	ENSMUSG00000000766	Oprm1	opioid receptor%2C mu 1 [Source:MGI Symbol%3BAcc:MGI:97441	ENSMUSG00000024335	Brd2	bromodomain containing 2 [Source:MGI Symbol%3BAcc:MGI:99495
ENSMUSG00000044976	Wdr72	WD repeat domain 72 [Source:MGI Symbol%3BAcc:MGI:3583957]	ENSMUSG00000037578	Pkd2l1	polycystic kidney disease 2-like 1 [Source:MGI Symbol%3BAcc:MGI:1352448]	ENSMUSG00000024395	Lims2	LIM and senescent cell antigen like domains 2 [Source:MGI Symbol%3BAcc:MGI:2385067]
ENSMUSG00000062329	Cytl1	cytokine-like 1 [Source:MGI Symbol%3BAcc:MGI:2684993]	ENSMUSG00000119750	Gm26498	predicted gene%2C 26498 [Source:MGI Symbol%3BAcc:MGI:5456275]	ENSMUSG00000024538	Ppic	peptidylprolyl isomerase C [Source:MGI Symbol%3BAcc:MGI:97751]
ENSMUSG00000025927	Tfap2b	transcription factor AP-2 beta [Source:MGI Symbol%3BAcc:MGI:104672]	ENSMUSG00000117804	Gm6252	predicted gene 6252 [Source:MGI Symbol%3BAcc:MGI:3644748]	ENSMUSG00000024621	Csf1r	colony stimulating factor 1 receptor [Source:MGI Symbol%3BAcc:MGI:1339758]
ENSMUSG00000029843	Slc13a4	solute carrier family 13 (sodium/sulfate symporters)%2C member 4 [Source:MGI Symbol%3BAcc:MGI:2442367]	ENSMUSG00000120816	-	novel transcript	ENSMUSG00000024737	Slc15a3	solute carrier family 15%2C member 3 [Source:MGI Symbol%3BAcc:MGI:1929691]
ENSMUSG00000118462	Lhb	luteinizing hormone beta [Source:MGI Symbol%3BAcc:MGI:96782	ENSMUSG00000040809	Chil3	chitinase-like 3 [Source:MGI Symbol%3BAcc:MGI:13308	ENSMUSG00000024758	Rtn3	reticulon 3 [Source:MGI Symbol%3BAcc:MGI:1339970]
ENSMUSG00002075586	SNORA21B	small nucleolar RNA%2C H/ACA box 21B [Source:HGNC Symbol%3BAcc:HGNC:52195]	ENSMUSG00000109021	Gm44812	predicted gene 44812 [Source:MGI Symbol%3BAcc:MGI:5753388]	ENSMUSG00000024831	Ighmbp2	immunoglobulin mu binding protein 2 [Source:MGI Symbol%3BAcc:MGI:99954]
ENSMUSG00000079355	Ackr4	atypical chemokine receptor 4 [Source:MGI Symbol%3BAcc:MGI:2181676]	ENSMUSG00000116557	4933432I09Rik	RIKEN cDNA 4933432I09 gene [Source:MGI Symbol%3BAcc:MGI:1914029]	ENSMUSG00000024827	Gldc	glycine decarboxylase [Source:MGI Symbol%3BAcc:MGI:1341155]
ENSMUSG00000007888	Crlf1	cytokine receptor-like factor 1 [Source:MGI Symbol%3BAcc:MGI:1340030]	ENSMUSG00000086368	Gm13830	predicted gene 13830 [Source:MGI Symbol%3BAcc:MGI:3651141]	ENSMUSG00000024867	Pip5k1b	phosphatidylinositol-4-phosphate 5-kinase%2C type 1 beta [Source:MGI Symbol%3BAcc:MGI:10793
ENSMUSG00000078190	Dnm3os	dynamin 3%2C opposite strand [Source:MGI Symbol%3BAcc:MGI:3052332]	ENSMUSG00000109359	Gm44797	predicted gene 44797 [Source:MGI Symbol%3BAcc:MGI:5753373]	ENSMUSG00000024871	Doc2g	double C2%2C gamma [Source:MGI Symbol%3BAcc:MGI:1926250]
ENSMUSG00000102801	Gm37478	predicted gene%2C 37478 [Source:MGI Symbol%3BAcc:MGI:5610706]	ENSMUSG00000036196	Slc26a8	solute carrier family 26%2C member 8 [Source:MGI Symbol%3BAcc:MGI:2385046]	ENSMUSG00000024875	Yif1a	Yip1 interacting factor homolog A (S. cerevisiae) [Source:MGI Symbol%3BAcc:MGI:1915340]
ENSMUSG00000039092	Sptlc3	serine palmitoyltransferase%2C long chain base subunit 3 [Source:MGI Symbol%3BAcc:MGI:2444678]	ENSMUSG00000022705	Drd3	dopamine receptor D3 [Source:MGI Symbol%3BAcc:MGI:94925]	ENSMUSG00000052085	Dock8	dedicator of cytokinesis 8 [Source:MGI Symbol%3BAcc:MGI:1921396]
ENSMUSG00000111094	Gm34425	predicted gene%2C 34425 [Source:MGI Symbol%3BAcc:MGI:5593584]	ENSMUSG00000052631	Sh2d6	SH2 domain containing 6 [Source:MGI Symbol%3BAcc:MGI:1918380]	ENSMUSG00000025142	Aspscr1	alveolar soft part sarcoma chromosome region%2C candidate 1 (human) [Source:MGI Symbol%3BAcc:MGI:1916188]
ENSMUSG00000105620	Gm43047	predicted gene 43047 [Source:MGI Symbol%3BAcc:MGI:5663184]	ENSMUSG00000097330	Gm26672	predicted gene%2C 26672 [Source:MGI Symbol%3BAcc:MGI:5477166]	ENSMUSG00000025400	Tac2	tachykinin 2 [Source:MGI Symbol%3BAcc:MGI:98476]

ENSMUSG00000042793	Lgr6	leucine-rich repeat-containing G protein-coupled receptor 6 [Source:MGI Symbol%3BAcc:MGI:2441805]	ENSMUSG00000086206	Gm13783	predicted gene 13783 [Source:MGI Symbol%3BAcc:MGI:3649551]	ENSMUSG00000025534	Gusb	glucuronidase%2C beta [Source:MGI Symbol%3BAcc:MGI:95872]
ENSMUSG00000073197	5730507C01Rik	RIKEN cDNA 5730507C01 gene [Source:MGI Symbol%3BAcc:MGI:1917882]	ENSMUSG00000059659	Gm10069	predicted gene 10069 [Source:MGI Symbol%3BAcc:MGI:3641875]	ENSMUSG00000025572	Tmc6	transmembrane channel-like gene family 6 [Source:MGI Symbol%3BAcc:MGI:1098686]
ENSMUSG00000041831	Sytl3	synaptotagmin-like 3 [Source:MGI Symbol%3BAcc:MGI:1933367]	ENSMUSG00000009596	Taf7l	TATA-box binding protein associated factor 7 like [Source:MGI Symbol%3BAcc:MGI:1921719]	ENSMUSG00000025731	Mettl26	methyltransferase like 26 [Source:MGI Symbol%3BAcc:MGI:1915597]
ENSMUSG00000090667	Mdfic2	MyoD family inhibitor domain containing 2 [Source:MGI Symbol%3BAcc:MGI:2685611]	ENSMUSG00000097745	AI115009	expressed sequence AI115009 [Source:MGI Symbol%3BAcc:MGI:2139623]	ENSMUSG00000025860	Xiap	X-linked inhibitor of apoptosis [Source:MGI Symbol%3BAcc:MGI:107572]
ENSMUSG00000113959	Gm46339	predicted gene%2C 46339 [Source:MGI Symbol%3BAcc:MGI:5825976]	ENSMUSG00000048455	#N/A	#N/A	ENSMUSG00000025892	Gria4	glutamate receptor%2C ionotropic%2C AMPA4 (alpha 4) [Source:MGI Symbol%3BAcc:MGI:95811
ENSMUSG00000082185	Gm12428	predicted gene 12428 [Source:MGI Symbol%3BAcc:MGI:3650848]	ENSMUSG00000000386	Mx1	MX dynamin-like GTPase 1 [Source:MGI Symbol%3BAcc:MGI:97243]	ENSMUSG00000026094	Stk17b	serine/threonine kinase 17b (apoptosis-inducing) [Source:MGI Symbol%3BAcc:MGI:2138162]
ENSMUSG00000028487	Bnc2	basonuclin 2 [Source:MGI Symbol%3BAcc:MGI:2443805]	ENSMUSG00000024658	Gm9750	predicted gene 9750 [Source:MGI Symbol%3BAcc:MGI:3642669]	ENSMUSG00000026185	Igfbp5	insulin-like growth factor binding protein 5 [Source:MGI Symbol%3BAcc:MGI:96440
ENSMUSG00000000157	Itgb2l	integrin beta 2-like [Source:MGI Symbol%3BAcc:MGI:1277979]	ENSMUSG00000108553	Gm38569	predicted gene%2C 38569 [Source:MGI Symbol%3BAcc:MGI:5621454]	ENSMUSG00000026241	Nppc	natriuretic peptide type C [Source:MGI Symbol%3BAcc:MGI:97369]
ENSMUSG00000120402	-	novel transcript%2C antisense to Dusp1	ENSMUSG00000045629	Sh3tc2	SH3 domain and tetratricopeptide repeats 2 [Source:MGI Symbol%3BAcc:MGI:2444417]	ENSMUSG00000026480	Ncf2	neutrophil cytosolic factor 2 [Source:MGI Symbol%3BAcc:MGI:97284]
ENSMUSG00000049353	Rd3	retinal degeneration 3 [Source:MGI Symbol%3BAcc:MGI:1921273]	ENSMUSG00000097617	Gm10687	predicted gene 10687 [Source:MGI Symbol%3BAcc:MGI:3642689]	ENSMUSG00000026548	Slamf9	SLAM family member 9 [Source:MGI Symbol%3BAcc:MGI:1923692]
ENSMUSG00000102816	Gm37814	predicted gene%2C 37814 [Source:MGI Symbol%3BAcc:MGI:5611042]	ENSMUSG00000020609	Apob	apolipoprotein B [Source:MGI Symbol%3BAcc:MGI:88052]	ENSMUSG00000026676	Ccdc3	coiled-coil domain containing 3 [Source:MGI Symbol%3BAcc:MGI:1921436]
ENSMUSG00000114306	Gm3227	predicted gene 3227 [Source:MGI Symbol%3BAcc:MGI:3781405]	ENSMUSG00000076620	Ighj2	immunoglobulin heavy joining 2 [Source:MGI Symbol%3BAcc:MGI:4439803]	ENSMUSG00000026683	Nuf2	NUF2%2C NDC80 kinetochore complex component [Source:MGI Symbol%3BAcc:MGI:1914227]
ENSMUSG00000034438	Gbp8	guanylate-binding protein 8 [Source:MGI Symbol%3BAcc:MGI:1923324]	ENSMUSG00000033585	Ndn	necdin%2C MAGE family member [Source:MGI Symbol%3BAcc:MGI:97290]	ENSMUSG00000026728	Vim	vimentin [Source:MGI Symbol%3BAcc:MGI:98932]
ENSMUSG00000075296	Aldh3b2	aldehyde dehydrogenase 3 family%2C member B2 [Source:MGI Symbol%3BAcc:MGI:2147613]	ENSMUSG00000119955	-	novel transcript	ENSMUSG00000026842	Abl1	c-abl oncogene 1%2C non-receptor tyrosine kinase [Source:MGI Symbol%3BAcc:MGI:87859]
ENSMUSG00000084007	Gm9003	predicted pseudogene 9003 [Source:MGI Symbol%3BAcc:MGI:3647163]	ENSMUSG00000047363	Cstad	CSA-conditional%2C T cell activation-dependent protein [Source:MGI Symbol%3BAcc:MGI:1925867]	ENSMUSG00000026880	Stom	stomatin [Source:MGI Symbol%3BAcc:MGI:95403]
ENSMUSG00000102691	Gm37780	predicted gene%2C 37780 [Source:MGI Symbol%3BAcc:MGI:5611008]	ENSMUSG00000104301	Wdr49	WD repeat domain 49 [Source:MGI Symbol%3BAcc:MGI:3645287]	ENSMUSG00000026885	Ttll11	tubulin tyrosine ligase-like family%2C member 11 [Source:MGI Symbol%3BAcc:MGI:1921660]
ENSMUSG00000086233	Gm11816	predicted gene 11816 [Source:MGI Symbol%3BAcc:MGI:3650294]	ENSMUSG00000059089	Fcgr4	Fc receptor%2C IgG%2C low affinity IV [Source:MGI Symbol%3BAcc:MGI:2179523]	ENSMUSG00000026896	Ifih1	interferon induced with helicase C domain 1 [Source:MGI Symbol%3BAcc:MGI:1918836]
ENSMUSG00000032057	Hoatz	HOATZ cilia and flagella associated protein [Source:MGI Symbol%3BAcc:MGI:1921013]	ENSMUSG00000099923	1700105P06Rik	RIKEN cDNA 1700105P06 gene [Source:MGI Symbol%3BAcc:MGI:1915479]	ENSMUSG00000026837	Col5a1	collagen%2C type V%2C alpha 1 [Source:MGI Symbol%3BAcc:MGI:88457]
ENSMUSG00000048583	Igf2	insulin-like growth factor 2 [Source:MGI Symbol%3BAcc:MGI:96434]	ENSMUSG00000029716	Tfr2	transferrin receptor 2 [Source:MGI Symbol%3BAcc:MGI:1354956]	ENSMUSG00000015085	Entpd2	ectonucleoside triphosphate diphosphohydrolase 2 [Source:MGI Symbol%3BAcc:MGI:10968
ENSMUSG00000059060	Rad51b	RAD51 paralog B [Source:MGI Symbol%3BAcc:MGI:1099436]	ENSMUSG00000033327	Tnxb	tenascin XB [Source:MGI Symbol%3BAcc:MGI:1932137]	ENSMUSG00000027186	Elf5	E74-like factor 5 [Source:MGI Symbol%3BAcc:MGI:1335079]
ENSMUSG00000024650	Slc22a6	solute carrier family 22 (organic anion transporter)%2C member 6 [Source:MGI Symbol%3BAcc:MGI:892001]	ENSMUSG00000035298	Klhl35	kelch-like 35 [Source:MGI Symbol%3BAcc:MGI:1919434]	ENSMUSG00000027188	Pamr1	peptidase domain containing associated with muscle regeneration 1 [Source:MGI Symbol%3BAcc:MGI:2445082]
ENSMUSG00000029618	Ocm	oncomodulin [Source:MGI Symbol%3BAcc:MGI:97401]	ENSMUSG00000041827	Oasl1	2'-5' oligoadenylate synthetase-like 1 [Source:MGI Symbol%3BAcc:MGI:2180849]	ENSMUSG00000027333	Smox	spermine oxidase [Source:MGI Symbol%3BAcc:MGI:2445356]

ENSMUSG00000030450	Oca2	oculocutaneous albinism II [Source:MGI Symbol%3BAcc:MGI:97454]	ENSMUSG00000072620	Slfn2	schlafen 2 [Source:MGI Symbol%3BAcc:MGI:1313258]	ENSMUSG00000027353	Mcm8	minichromosome maintenance 8 homologous recombination repair factor [Source:MGI Symbol%3BAcc:MGI:19138
ENSMUSG00000056904	Gm5620	predicted gene 5620 [Source:MGI Symbol%3BAcc:MGI:3647798]	ENSMUSG00000047369	Dnah14	dynein%2C axonemal%2C heavy chain 14 [Source:MGI Symbol%3BAcc:MGI:2444525]	ENSMUSG00000027424	Mgme1	mitochondrial genome maintenance exonuclease 1 [Source:MGI Symbol%3BAcc:MGI:1921778]
ENSMUSG00000035799	Twist1	twist basic helix-loop-helix transcription factor 1 [Source:MGI Symbol%3BAcc:MGI:98872	ENSMUSG00000081303	Gm16011	predicted gene 16011 [Source:MGI Symbol%3BAcc:MGI:3801796]	ENSMUSG00000027456	Sdcbp2	syndecan binding protein (syntenin) 2 [Source:MGI Symbol%3BAcc:MGI:2385156]
ENSMUSG00000030344	Akap3	A kinase (PRKA) anchor protein 3 [Source:MGI Symbol%3BAcc:MGI:1341149]	ENSMUSG00000084159	Gm12696	predicted gene 12696 [Source:MGI Symbol%3BAcc:MGI:3649288]	ENSMUSG00000027546	Atp9a	ATPase%2C class II%2C type 9A [Source:MGI Symbol%3BAcc:MGI:1330826]
ENSMUSG00000036412	Arsi	arylsulfatase i [Source:MGI Symbol%3BAcc:MGI:2670959]	ENSMUSG00000073490	Ifi207	interferon activated gene 207 [Source:MGI Symbol%3BAcc:MGI:2138302]	ENSMUSG00000027650	Tti1	TELO2 interacting protein 1 [Source:MGI Symbol%3BAcc:MGI:1922675]
ENSMUSG00000082160	Gm11578	predicted gene 11578 [Source:MGI Symbol%3BAcc:MGI:3650505]	ENSMUSG00000097443	Gm17529	predicted gene%2C 17529 [Source:MGI Symbol%3BAcc:MGI:4937163]	ENSMUSG00000018459	Slc13a3	solute carrier family 13 (sodium-dependent dicarboxylate transporter)%2C member 3 [Source:MGI Symbol%3BAcc:MGI:2149635]
ENSMUSG00000109529	Gm44801	predicted gene 44801 [Source:MGI Symbol%3BAcc:MGI:5753377]	ENSMUSG00000103723	Gm37095	predicted gene%2C 37095 [Source:MGI Symbol%3BAcc:MGI:5610323]	ENSMUSG00000027676	Ccdc39	coiled-coil domain containing 39 [Source:MGI Symbol%3BAcc:MGI:1289263]
ENSMUSG00000109194	Gm44570	predicted gene 44570 [Source:MGI Symbol%3BAcc:MGI:5753146]	ENSMUSG00000108137	Gm44053	predicted gene%2C 44053 [Source:MGI Symbol%3BAcc:MGI:5690445]	ENSMUSG00000027715	Ccna2	cyclin A2 [Source:MGI Symbol%3BAcc:MGI:108069]
ENSMUSG00000072679	D6Ertd474e	DNA segment%2C Chr 6%2C ERATO Doi 474%2C expressed [Source:MGI Symbol%3BAcc:MGI:1196315]	ENSMUSG00000109245	Gm44860	predicted gene 44860 [Source:MGI Symbol%3BAcc:MGI:5753436]	ENSMUSG00000027751	Supt20	SPT20 SAGA complex component [Source:MGI Symbol%3BAcc:MGI:1929651]
ENSMUSG00000044951	Mylk4	myosin light chain kinase family%2C member 4 [Source:MGI Symbol%3BAcc:MGI:3643758]	ENSMUSG00000111110	Gm36855	predicted gene%2C 36855 [Source:MGI Symbol%3BAcc:MGI:5596014]	ENSMUSG00000027765	P2ry1	purinergic receptor P2Y%2C G-protein coupled 1 [Source:MGI Symbol%3BAcc:MGI:105049]
ENSMUSG00000038403	Hjv	hemojuvelin BMP co-receptor [Source:MGI Symbol%3BAcc:MGI:1916835]	ENSMUSG00000079654	Prrt4	proline-rich transmembrane protein 4 [Source:MGI Symbol%3BAcc:MGI:21416	ENSMUSG00000027800	Tm4sf1	transmembrane 4 superfamily member 1 [Source:MGI Symbol%3BAcc:MGI:10467
ENSMUSG00000030787	Lyve1	lymphatic vessel endothelial hyaluronan receptor 1 [Source:MGI Symbol%3BAcc:MGI:2136348]	ENSMUSG00000059647	Cbx3-ps7	chromobox 3%2C pseudogene 7 [Source:MGI Symbol%3BAcc:MGI:3704187]	ENSMUSG00000027803	Wwtr1	WW domain containing transcription regulator 1 [Source:MGI Symbol%3BAcc:MGI:1917649]
ENSMUSG00000033268	Duox1	dual oxidase 1 [Source:MGI Symbol%3BAcc:MGI:2139422]	ENSMUSG00000020286	1700093K21Rik	RIKEN cDNA 1700093K21 gene [Source:MGI Symbol%3BAcc:MGI:1914608]	ENSMUSG00000027848	Olfml3	olfactomedin-like 3 [Source:MGI Symbol%3BAcc:MGI:1914877]
ENSMUSG00000021791	Dydc2	DPY30 domain containing 2 [Source:MGI Symbol%3BAcc:MGI:1918450]	ENSMUSG00000110016	Gm20751	predicted gene%2C 20751 [Source:MGI Symbol%3BAcc:MGI:5434107]	ENSMUSG00000027907	S100a11	S100 calcium binding protein A11 [Source:MGI Symbol%3BAcc:MGI:1338798]
ENSMUSG00000031383	Dusp9	dual specificity phosphatase 9 [Source:MGI Symbol%3BAcc:MGI:23871	ENSMUSG00000086512	Gm12600	predicted gene 12600 [Source:MGI Symbol%3BAcc:MGI:3651471]	ENSMUSG00000027954	Efna1	ephrin A1 [Source:MGI Symbol%3BAcc:MGI:103236]
ENSMUSG00000121005	-	novel transcript	ENSMUSG00000110205	Gm33594	predicted gene%2C 33594 [Source:MGI Symbol%3BAcc:MGI:5592753]	ENSMUSG00000027978	Prss12	protease%2C serine 12 neurotrypsin (motopsin) [Source:MGI Symbol%3BAcc:MGI:1100881]
ENSMUSG00000051906	Cd209f	CD209f antigen [Source:MGI Symbol%3BAcc:MGI:1916392]	ENSMUSG00000118501	Gm53048	predicted gene%2C 53048 [Source:MGI Symbol%3BAcc:MGI:6388943]	ENSMUSG00000027995	Tlr2	toll-like receptor 2 [Source:MGI Symbol%3BAcc:MGI:1346060]
ENSMUSG00000065118	Gm23297	predicted gene%2C 23297 [Source:MGI Symbol%3BAcc:MGI:5453074]	ENSMUSG00000086472	Gm16172	predicted gene 16172 [Source:MGI Symbol%3BAcc:MGI:3801735]	ENSMUSG00000028051	Hcn3	hyperpolarization-activated%2C cyclic nucleotide-gated K+ 3 [Source:MGI Symbol%3BAcc:MGI:1298211]
ENSMUSG00000046463	5930403N24Rik	RIKEN cDNA 5930403N24 gene [Source:MGI Symbol%3BAcc:MGI:2444171]	ENSMUSG00000090778	Gm3235	predicted gene 3235 [Source:MGI Symbol%3BAcc:MGI:3781413]	ENSMUSG00000028063	Lmna	lamin A [Source:MGI Symbol%3BAcc:MGI:96794]
ENSMUSG00000041559	Fmod	fibromodulin [Source:MGI Symbol%3BAcc:MGI:1328364]	ENSMUSG00000006360	Crip1	cysteine-rich protein 1 (intestinal) [Source:MGI Symbol%3BAcc:MGI:88501	ENSMUSG00000015947	Fcgr1	Fc receptor%2C IgG%2C high affinity I [Source:MGI Symbol%3BAcc:MGI:95498
ENSMUSG00000120554	-	novel transcript	ENSMUSG00000030623	Prss23os	protease%2C serine 23%2C opposite strand [Source:MGI Symbol%3BAcc:MGI:1916654]	ENSMUSG00000028159	Dapp1	dual adaptor for phosphotyrosine and 3-phosphoinositides 1 [Source:MGI Symbol%3BAcc:MGI:1347063]
ENSMUSG00000003436	Dll3	delta like canonical Notch ligand 3 [Source:MGI Symbol%3BAcc:MGI:1096877]	ENSMUSG00000028475	Spaar	small regulatory polypeptide of amino acid response [Source:MGI Symbol%3BAcc:MGI:1918656]	ENSMUSG00000028197	Col24a1	collagen%2C type XXIV%2C alpha 1 [Source:MGI Symbol%3BAcc:MGI:1918605]

ENSMUSG00000021553	Slc28a3	solute carrier family 28 (sodium-coupled nucleoside transporter)%2C member 3 [Source:MGI Symbol%3BAcc:MGI:21373	ENSMUSG00000103920	Gm37897	predicted gene%2C 37897 [Source:MGI Symbol%3BAcc:MGI:5611125]	ENSMUSG00000028256	Odf2l	outer dense fiber of sperm tails 2-like [Source:MGI Symbol%3BAcc:MGI:1098600]
ENSMUSG00000033383	Rtp1	receptor transporter protein 1 [Source:MGI Symbol%3BAcc:MGI:2685450]	ENSMUSG00000044556	Tex38	testis expressed 38 [Source:MGI Symbol%3BAcc:MGI:1922423]	ENSMUSG00000015243	Abca1	ATP-binding cassette%2C sub-family A (ABC1)%2C member 1 [Source:MGI Symbol%3BAcc:MGI:99607
ENSMUSG00000109625	3110080E11Rik	RIKEN cDNA 3110080E11 gene [Source:MGI Symbol%3BAcc:MGI:1920469]	ENSMUSG00000106961	Gm43128	predicted gene 43128 [Source:MGI Symbol%3BAcc:MGI:5663265]	ENSMUSG00000028559	Osbpl9	oxysterol binding protein-like 9 [Source:MGI Symbol%3BAcc:MGI:1923784]
ENSMUSG00000031492	Chrnb3	cholinergic receptor%2C nicotinic%2C beta polypeptide 3 [Source:MGI Symbol%3BAcc:MGI:10621	ENSMUSG00000035594	Chrna5	cholinergic receptor%2C nicotinic%2C alpha polypeptide 5 [Source:MGI Symbol%3BAcc:MGI:87889	ENSMUSG00000028655	Mfsd2a	major facilitator superfamily domain containing 2A [Source:MGI Symbol%3BAcc:MGI:19238
ENSMUSG00000041372	B4galnt3	beta-1%2C4-N-acetyl-galactosaminyl transferase 3 [Source:MGI Symbol%3BAcc:MGI:3041155]	ENSMUSG00000105260	Gm40040	predicted gene%2C 40040 [Source:MGI Symbol%3BAcc:MGI:5622925]	ENSMUSG00000028859	Csf3r	colony stimulating factor 3 receptor (granulocyte) [Source:MGI Symbol%3BAcc:MGI:1339755]
ENSMUSG00000034127	Tspan8	tetraspanin 8 [Source:MGI Symbol%3BAcc:MGI:2384918]	ENSMUSG00000078763	Slfn1	schlafen 1 [Source:MGI Symbol%3BAcc:MGI:1313259]	ENSMUSG00000028871	Rspo1	R-spondin 1 [Source:MGI Symbol%3BAcc:MGI:2183426]
ENSMUSG00000035694	Caps2	calcyphosphine 2 [Source:MGI Symbol%3BAcc:MGI:2441980]	ENSMUSG00000030413	Pglyrp1	peptidoglycan recognition protein 1 [Source:MGI Symbol%3BAcc:MGI:1345092]	ENSMUSG00000028927	Padi2	peptidyl arginine deiminase%2C type II [Source:MGI Symbol%3BAcc:MGI:1338892]
ENSMUSG00000096957	E230013L22Rik	RIKEN cDNA E230013L22 gene [Source:MGI Symbol%3BAcc:MGI:2142601]	ENSMUSG00000103755	Gm37805	predicted gene%2C 37805 [Source:MGI Symbol%3BAcc:MGI:5611033]	ENSMUSG00000063077	Kif1b	kinesin family member 1B [Source:MGI Symbol%3BAcc:MGI:108426]
ENSMUSG00000028125	Abca4	ATP-binding cassette%2C sub-family A (ABC1)%2C member 4 [Source:MGI Symbol%3BAcc:MGI:109424]	ENSMUSG00000042808	Gpx2	glutathione peroxidase 2 [Source:MGI Symbol%3BAcc:MGI:106609]	ENSMUSG00000028976	Slc2a5	solute carrier family 2 (facilitated glucose transporter)%2C member 5 [Source:MGI Symbol%3BAcc:MGI:1928369]
ENSMUSG00000087017	4930417H01Rik	RIKEN cDNA 4930417H01 gene [Source:MGI Symbol%3BAcc:MGI:3041209]	ENSMUSG00000100534	Gm816	predicted gene 816 [Source:MGI Symbol%3BAcc:MGI:2685662]	ENSMUSG00000029020	Mfn2	mitofusin 2 [Source:MGI Symbol%3BAcc:MGI:2442230]
ENSMUSG00000030862	Cpxm2	carboxypeptidase X 2 (M14 family) [Source:MGI Symbol%3BAcc:MGI:1926006]	ENSMUSG00000112297	Gm17816	predicted gene%2C 17816 [Source:MGI Symbol%3BAcc:MGI:5010001]	ENSMUSG00000029070	Mxra8	matrix-remodelling associated 8 [Source:MGI Symbol%3BAcc:MGI:1922011]
ENSMUSG00000071489	Ptgdr	prostaglandin D receptor [Source:MGI Symbol%3BAcc:MGI:102966]	ENSMUSG00000109556	Gm38843	predicted gene%2C 38843 [Source:MGI Symbol%3BAcc:MGI:5621728]	ENSMUSG00000029122	Evc	EvC ciliary complex subunit 1 [Source:MGI Symbol%3BAcc:MGI:1890596]
ENSMUSG00000078920	Ifi47	interferon gamma inducible protein 47 [Source:MGI	ENSMUSG00000120716	-	novel transcript	ENSMUSG00000029193	Cckar	cholecystokinin A receptor [Source:MGI Symbol%3BAcc:MGI:99478
ENSMUSG00000016756	Cmah	cytidine monophospho-N-acetylneuraminic acid hydroxylase [Source:MGI Symbol%3BAcc:MGI:103227]	ENSMUSG00000097496	Gm26653	predicted gene%2C 26653 [Source:MGI Symbol%3BAcc:MGI:5477147]	ENSMUSG00000001260	Gabrg1	gamma-aminobutyric acid (GABA) A receptor%2C subunit gamma 1 [Source:MGI Symbol%3BAcc:MGI:10315
ENSMUSG00000104693	Gm42941	predicted gene 42941 [Source:MGI Symbol%3BAcc:MGI:5663078]	ENSMUSG00000067356	B430203G13Rik	RIKEN cDNA B430203G13 gene [Source:MGI Symbol%3BAcc:MGI:3642832]	ENSMUSG00000029343	Crybb1	crystallin%2C beta B1 [Source:MGI Symbol%3BAcc:MGI:104992]
ENSMUSG00000037490	Slc2a12	solute carrier family 2 (facilitated glucose transporter)%2C member 12 [Source:MGI Symbol%3BAcc:MGI:3052471]	ENSMUSG00000102277	A130050O07Rik	RIKEN cDNA A130050O07 gene [Source:MGI Symbol%3BAcc:MGI:2443458]	ENSMUSG00000029570	Lfng	LFNG O-fucosylpeptide 3-beta-N-acetylglucosaminyltransferase [Source:MGI Symbol%3BAcc:MGI:1095413]
ENSMUSG00000102900	Gm37811	predicted gene%2C 37811 [Source:MGI Symbol%3BAcc:MGI:5611039]	ENSMUSG00000005465	Il27ra	interleukin 27 receptor%2C alpha [Source:MGI Symbol%3BAcc:MGI:1355318]	ENSMUSG00000029669	Tspan12	tetraspanin 12 [Source:MGI Symbol%3BAcc:MGI:1889818]
ENSMUSG00000013584	Aldh1a2	aldehyde dehydrogenase family 1%2C subfamily A2 [Source:MGI Symbol%3BAcc:MGI:107928]	ENSMUSG00000113019	Gm47467	predicted gene%2C 47467 [Source:MGI Symbol%3BAcc:MGI:6096433]	ENSMUSG00000029695	Aass	aminoadipate-semialdehyde synthase [Source:MGI Symbol%3BAcc:MGI:1353573]
ENSMUSG00000105745	Gm42521	predicted gene 42521 [Source:MGI Symbol%3BAcc:MGI:5662658]	ENSMUSG00000022952	Runx1	runt related transcription factor 1 [Source:MGI Symbol%3BAcc:MGI:99852]	ENSMUSG00000029759	Pon3	paraoxonase 3 [Source:MGI Symbol%3BAcc:MGI:106686]
ENSMUSG00000025491	Ifitm1	interferon induced transmembrane protein 1 [Source:MGI Symbol%3BAcc:MGI:1915963]	ENSMUSG00000078995	Zfp456	zinc finger protein 456 [Source:MGI Symbol%3BAcc:MGI:3040694]	ENSMUSG00000029861	Fam131b	family with sequence similarity 131%2C member B [Source:MGI Symbol%3BAcc:MGI:1923406]
ENSMUSG00000097282	5031415H12Rik	RIKEN cDNA 5031415H12 gene [Source:MGI Symbol%3BAcc:MGI:1923234]	ENSMUSG00000027322	Siglec1	sialic acid binding Ig-like lectin 1%2C sialoadhesin [Source:MGI Symbol%3BAcc:MGI:99668]	ENSMUSG00000029915	Clec5a	C-type lectin domain family 5%2C member a [Source:MGI Symbol%3BAcc:MGI:1345151]
ENSMUSG00000036446	Lum	lumican [Source:MGI Symbol%3BAcc:MGI:109347]	ENSMUSG00000002944	Cd36	CD36 molecule [Source:MGI Symbol%3BAcc:MGI:107899]	ENSMUSG00000029919	Hpgds	hematopoietic prostaglandin D synthase [Source:MGI Symbol%3BAcc:MGI:18593
ENSMUSG00000056328	Myh1	myosin%2C heavy polypeptide 1%2C skeletal muscle%2C adult [Source:MGI Symbol%3BAcc:MGI:1339711]	ENSMUSG00000110652	Gm33023	predicted gene%2C 33023 [Source:MGI Symbol%3BAcc:MGI:5592182]	ENSMUSG00000030111	A2m	alpha-2-macroglobulin [Source:MGI Symbol%3BAcc:MGI:2449119]

ENSMUSG00000085180	AI838599	expressed sequence AI838599 [Source:MGI Symbol%3BAcc:MGI:3510989]	ENSMUSG00000117575	Gm36486	predicted gene%2C 36486 [Source:MGI Symbol%3BAcc:MGI:5595645]	ENSMUSG00000030124	Lag3	lymphocyte-activation gene 3 [Source:MGI Symbol%3BAcc:MGI:106588]
ENSMUSG00000096596	Gm10591	predicted gene 10591 [Source:MGI Symbol%3BAcc:MGI:3711256]	ENSMUSG00000117263	Gm35290	predicted gene%2C 35290 [Source:MGI Symbol%3BAcc:MGI:5594449]	ENSMUSG00000030208	Emp1	epithelial membrane protein 1 [Source:MGI Symbol%3BAcc:MGI:107941]
ENSMUSG00000022512	Cldn1	claudin 1 [Source:MGI Symbol%3BAcc:MGI:1276109]	ENSMUSG00000071235	Vrtn	vertebrae development associated [Source:MGI Symbol%3BAcc:MGI:3588197]	ENSMUSG00000030220	Arhgdib	Rho%2C GDP dissociation inhibitor (GDI) beta [Source:MGI Symbol%3BAcc:MGI:10194
ENSMUSG00000058806	Col13a1	collagen%2C type XIII%2C alpha 1 [Source:MGI Symbol%3BAcc:MGI:1277201]	ENSMUSG00000037613	Tnfrsf23	tumor necrosis factor receptor superfamily%2C member 23 [Source:MGI Symbol%3BAcc:MGI:1930269]	ENSMUSG00000030235	Slco1c1	solute carrier organic anion transporter family%2C member 1c1 [Source:MGI Symbol%3BAcc:MGI:18896
ENSMUSG00000098785	Mir6395	microRNA 6395 [Source:MGI Symbol%3BAcc:MGI:5530943]	ENSMUSG00000038543	BC028528	cDNA sequence BC028528 [Source:MGI Symbol%3BAcc:MGI:2385885]	ENSMUSG00000057604	Lmcd1	LIM and cysteine-rich domains 1 [Source:MGI Symbol%3BAcc:MGI:1353635]
ENSMUSG00000110928	Gm48114	predicted gene%2C 48114 [Source:MGI Symbol%3BAcc:MGI:6097467]	ENSMUSG00000072919	Noxred1	NADP+ dependent oxidoreductase domain containing 1 [Source:MGI Symbol%3BAcc:MGI:1918525]	ENSMUSG00000030337	Vamp1	vesicle-associated membrane protein 1 [Source:MGI Symbol%3BAcc:MGI:1313276]
ENSMUSG00000044177	Wfikkn2	WAP%2C follistatin/kazal%2C immunoglobulin%2C kunitz and netrin domain containing 2 [Source:MGI Symbol%3BAcc:MGI:26692	ENSMUSG00000044719	E230025N22Rik	Riken cDNA E230025N22 gene [Source:MGI Symbol%3BAcc:MGI:3687212]	ENSMUSG00000030341	Tnfrsf1a	tumor necrosis factor receptor superfamily%2C member 1a [Source:MGI Symbol%3BAcc:MGI:1314884]
ENSMUSG00000047420	Fam180a	family with sequence similarity 180%2C member A [Source:MGI Symbol%3BAcc:MGI:3039626]	ENSMUSG00000108551	Gm20274	predicted gene%2C 20274 [Source:MGI Symbol%3BAcc:MGI:5012459]	ENSMUSG00000030342	Cd9	CD9 antigen [Source:MGI Symbol%3BAcc:MGI:88348]
ENSMUSG00000034701	Neurod1	neurogenic differentiation 1 [Source:MGI Symbol%3BAcc:MGI:1339708]	ENSMUSG00000117130	Gm49802	predicted gene%2C 49802 [Source:MGI Symbol%3BAcc:MGI:6270463]	ENSMUSG00000030410	Dmwd	dystrophia myotonica-containing WD repeat motif [Source:MGI Symbol%3BAcc:MGI:94907
ENSMUSG00000003227	Edar	ectodysplasin-A receptor [Source:MGI Symbol%3BAcc:MGI:1343498]	ENSMUSG00000063245	Zfp993	zinc finger protein 993 [Source:MGI Symbol%3BAcc:MGI:3713585]	ENSMUSG00000030500	Slc17a6	solute carrier family 17 (sodium-dependent inorganic phosphate cotransporter)%2C member 6 [Source:MGI Symbol%3BAcc:MGI:2156052]
ENSMUSG00000042816	Gpr151	G protein-coupled receptor 151 [Source:MGI Symbol%3BAcc:MGI:2441887]	ENSMUSG00000080759	Gm15573	predicted gene 15573 [Source:MGI Symbol%3BAcc:MGI:3783021]	ENSMUSG00000030551	Nr2f2	nuclear receptor subfamily 2%2C group F%2C member 2 [Source:MGI Symbol%3BAcc:MGI:1352452]
ENSMUSG00000078502	Zfp268	zinc finger protein 268 [Source:MGI Symbol%3BAcc:MGI:3651014]	ENSMUSG00000107709	9430018G01Rik	RIKEN cDNA 9430018G01 gene [Source:MGI Symbol%3BAcc:MGI:2442776]	ENSMUSG00000030717	Nupr1	nuclear protein transcription regulator 1 [Source:MGI Symbol%3BAcc:MGI:18918
ENSMUSG00000105983	Gm42770	predicted gene 42770 [Source:MGI Symbol%3BAcc:MGI:5662907]	ENSMUSG00000086359	9630013K17Rik	RIKEN cDNA 9630013K17 gene [Source:MGI Symbol%3BAcc:MGI:1926133]	ENSMUSG00000030745	Il21r	interleukin 21 receptor [Source:MGI Symbol%3BAcc:MGI:1890475]
ENSMUSG00000021390	Ogn	osteoglycin [Source:MGI Symbol%3BAcc:MGI:109278]	ENSMUSG00000022218	Tgm1	transglutaminase 1%2C K polypeptide [Source:MGI Symbol%3BAcc:MGI:98730]	ENSMUSG00000030793	Pycard	PYD and CARD domain containing [Source:MGI Symbol%3BAcc:MGI:1931465]
ENSMUSG00000085772	D630024D03Rik	RIKEN cDNA D630024D03 gene [Source:MGI Symbol%3BAcc:MGI:3041224]	ENSMUSG00000031779	Ccl22	chemokine (C-C motif) ligand 22 [Source:MGI Symbol%3BAcc:MGI:1306779]	ENSMUSG00000030867	Plk1	polo like kinase 1 [Source:MGI Symbol%3BAcc:MGI:97621]
ENSMUSG00000073405	H2-T-ps	histocompatibility 2%2C T region locus%2C pseudogene [Source:MGI Symbol%3BAcc:MGI:2442805]	ENSMUSG00000024986	Hhex	hematopoietically expressed homeobox [Source:MGI Symbol%3BAcc:MGI:96086]	ENSMUSG00000030872	Gga2	golgi associated%2C gamma adaptin ear containing%2C ARF binding protein 2 [Source:MGI Symbol%3BAcc:MGI:19213
ENSMUSG00000042436	Mfap4	microfibrillar-associated protein 4 [Source:MGI Symbol%3BAcc:MGI:1342276]	ENSMUSG00000101741	Gm28055	predicted gene 28055 [Source:MGI Symbol%3BAcc:MGI:5578761]	ENSMUSG00000031004	Mki67	antigen identified by monoclonal antibody Ki 67 [Source:MGI Symbol%3BAcc:MGI:10603
ENSMUSG00000015090	Ptgds	prostaglandin D2 synthase (brain) [Source:MGI Symbol%3BAcc:MGI:99261]	ENSMUSG00000109209	Gm45104	predicted gene 45104 [Source:MGI Symbol%3BAcc:MGI:5753680]	ENSMUSG00000031101	Sash3	SAM and SH3 domain containing 3 [Source:MGI Symbol%3BAcc:MGI:1921381]
ENSMUSG00000121208	-	novel transcript	ENSMUSG00002076680	mmu-mir-1194	mmu-mir-1194 [Source:miRBase%3BAcc:MI0006299]	ENSMUSG00000031129	Slc9a9	solute carrier family 9 (sodium/hydrogen exchanger)%2C member 9 [Source:MGI Symbol%3BAcc:MGI:2679732]
ENSMUSG00000100627	A830008E24Rik	RIKEN cDNA A830008E24 gene [Source:MGI Symbol%3BAcc:MGI:3041183]	ENSMUSG00000110795	Gm47179	predicted gene%2C 47179 [Source:MGI Symbol%3BAcc:MGI:6095965]	ENSMUSG00000031146	Plp2	proteolipid protein 2 [Source:MGI Symbol%3BAcc:MGI:1298382]
ENSMUSG00000021760	Gpx8	glutathione peroxidase 8 (putative) [Source:MGI Symbol%3BAcc:MGI:1916840]	ENSMUSG00000041566	Tssk1	testis-specific serine kinase 1 [Source:MGI Symbol%3BAcc:MGI:1347557]	ENSMUSG00000031165	Was	Wiskott-Aldrich syndrome [Source:MGI Symbol%3BAcc:MGI:105059]
ENSMUSG00000043168	4930426D05Rik	RIKEN cDNA 4930426D05 gene [Source:MGI Symbol%3BAcc:MGI:1921894]	ENSMUSG00000114193	Gm48139	predicted gene%2C 48139 [Source:MGI Symbol%3BAcc:MGI:6097504]	ENSMUSG00000031189	Aff2	AF4/FMR2 family%2C member 2 [Source:MGI Symbol%3BAcc:MGI:1202294]
ENSMUSG00000037254	Itih2	inter-alpha trypsin inhibitor%2C heavy chain 2 [Source:MGI Symbol%3BAcc:MGI:96619	ENSMUSG00000096233	Gm13238	predicted gene 13238 [Source:MGI Symbol%3BAcc:MGI:3652104]	ENSMUSG00000031210	Gpr165	G protein-coupled receptor 165 [Source:MGI Symbol%3BAcc:MGI:1923456]

ENSMUSG00000006014	Prg4	proteoglycan 4 (megakaryocyte stimulating factor%2C articular superficial zone protein) [Source:MGI Symbol%3BAcc:MGI:1891344]	ENSMUSG00000025494	Sigirr	single immunoglobulin and toll-interleukin 1 receptor (TIR) domain [Source:MGI Symbol%3BAcc:MGI:1344402]	ENSMUSG00000031232	Magt1	magnesium transporter 1 [Source:MGI Symbol%3BAcc:MGI:1914325]
ENSMUSG00000120798	-	novel transcript	ENSMUSG00000006378	Gcat	glycine C-acetyltransferase (2-amino-3-ketobutyrate-coenzyme A ligase) [Source:MGI Symbol%3BAcc:MGI:13493	ENSMUSG00000031264	Btk	Bruton agammaglobulinemia tyrosine kinase [Source:MGI Symbol%3BAcc:MGI:88216]
ENSMUSG00000057722	Lepr	leptin receptor [Source:MGI Symbol%3BAcc:MGI:104993]	ENSMUSG00000029697	Fezf1	Fez family zinc finger 1 [Source:MGI Symbol%3BAcc:MGI:1920441]	ENSMUSG00000031278	Acsl4	acyl-CoA synthetase long-chain family member 4 [Source:MGI Symbol%3BAcc:MGI:1354713]
ENSMUSG00000117943	Gm50403	predicted gene%2C 50403 [Source:MGI Symbol%3BAcc:MGI:6303316]	ENSMUSG00000107932	Gm44432	predicted gene%2C 44432 [Source:MGI Symbol%3BAcc:MGI:5690824]	ENSMUSG00000031373	Car5b	carbonic anhydrase 5b%2C mitochondrial [Source:MGI Symbol%3BAcc:MGI:19262
ENSMUSG00000026870	Cutal	cutA divalent cation tolerance homolog-like [Source:MGI Symbol%3BAcc:MGI:1925246]	ENSMUSG00000104394	Gm37254	predicted gene%2C 37254 [Source:MGI Symbol%3BAcc:MGI:5610482]	ENSMUSG00000031458	Coprs	coordinator of PRMT5%2C differentiation stimulator [Source:MGI Symbol%3BAcc:MGI:1913673]
ENSMUSG00000020218	Wif1	Wnt inhibitory factor 1 [Source:MGI Symbol%3BAcc:MGI:1344332]	ENSMUSG00000120174	4931408D14Rik	RIKEN cDNA 4931408D14 gene [Source:NCBI gene (formerly Entrezgene)%3BAcc:77059]	ENSMUSG00000031502	Col4a1	collagen%2C type IV%2C alpha 1 [Source:MGI Symbol%3BAcc:MGI:88454]
ENSMUSG00000048373	Fgfbp1	fibroblast growth factor binding protein 1 [Source:MGI Symbol%3BAcc:MGI:1096350]	ENSMUSG00000090122	Kcne1l	potassium voltage-gated channel%2C Isk-related family%2C member 1-like%2C pseudogene [Source:MGI Symbol%3BAcc:MGI:1913490]	ENSMUSG00000031616	Ednra	endothelin receptor type A [Source:MGI Symbol%3BAcc:MGI:105923]
ENSMUSG00000103492	Gm37412	predicted gene%2C 37412 [Source:MGI Symbol%3BAcc:MGI:5610640]	ENSMUSG00000116260	1700041B01Rik	RIKEN cDNA 1700041B01 gene [Source:MGI Symbol%3BAcc:MGI:1925873]	ENSMUSG00000031644	Nek1	NIMA (never in mitosis gene a)-related expressed kinase 1 [Source:MGI Symbol%3BAcc:MGI:97303]
ENSMUSG00000059187	Tafa1	TAFA chemokine like family member 1 [Source:MGI Symbol%3BAcc:MGI:24436	ENSMUSG00000058246	Gm10037	predicted gene 10037 [Source:MGI Symbol%3BAcc:MGI:3645096]	ENSMUSG00000031662	Snx20	sorting nexin 20 [Source:MGI Symbol%3BAcc:MGI:1918857]
ENSMUSG00000048489	Depp1	DEPP1 autophagy regulator [Source:MGI Symbol%3BAcc:MGI:1918730]	ENSMUSG00000104699	Rps4x-ps	Rps4x retrotransposed pseudogene [Source:MGI Symbol%3BAcc:MGI:3644569]	ENSMUSG00000031762	Mt2	metallothionein 2 [Source:MGI Symbol%3BAcc:MGI:97172]
ENSMUSG00000033196	Myh2	myosin%2C heavy polypeptide 2%2C skeletal muscle%2C adult [Source:MGI Symbol%3BAcc:MGI:1339710]	ENSMUSG00000102937	Gm38116	predicted gene%2C 38116 [Source:MGI Symbol%3BAcc:MGI:5611344]	ENSMUSG00000031788	Kifc3	kinesin family member C3 [Source:MGI Symbol%3BAcc:MGI:109202]
ENSMUSG00000000303	Cdh1	cadherin 1 [Source:MGI Symbol%3BAcc:MGI:88354]	ENSMUSG00000022501	Prm1	protamine 1 [Source:MGI Symbol%3BAcc:MGI:97765]	ENSMUSG00000031922	Cep57	centrosomal protein 57 [Source:MGI Symbol%3BAcc:MGI:1915551]
ENSMUSG00000103632	Gm38329	predicted gene%2C 38329 [Source:MGI Symbol%3BAcc:MGI:5611557]	ENSMUSG00000100891	2810049E08Rik	RIKEN cDNA 2810049E08 gene [Source:MGI Symbol%3BAcc:MGI:1919927]	ENSMUSG00000031976	Urb2	URB2 ribosome biogenesis 2 homolog (S. cerevisiae) [Source:MGI Symbol%3BAcc:MGI:2681124]
ENSMUSG00000031494	Cd209a	CD209a antigen [Source:MGI Symbol%3BAcc:MGI:2157942]	ENSMUSG00000110605	Gm32856	predicted gene%2C 32856 [Source:MGI Symbol%3BAcc:MGI:5592015]	ENSMUSG00000032089	Il10ra	interleukin 10 receptor%2C alpha [Source:MGI Symbol%3BAcc:MGI:96538]
ENSMUSG00000062248	Cks2	CDC28 protein kinase regulatory subunit 2 [Source:MGI Symbol%3BAcc:MGI:1913447]	ENSMUSG00000054901	Arhgef33	Rho guanine nucleotide exchange factor (GEF) 33 [Source:MGI Symbol%3BAcc:MGI:2685787]	ENSMUSG00000048537	Phldb1	pleckstrin homology like domain%2C family B%2C member 1 [Source:MGI Symbol%3BAcc:MGI:2143230]
ENSMUSG00000022206	Npr3	natriuretic peptide receptor 3 [Source:MGI Symbol%3BAcc:MGI:97373]	ENSMUSG00000046219	C230012O17Rik	RIKEN cDNA C230012O17 gene [Source:MGI Symbol%3BAcc:MGI:2442283]	ENSMUSG00000032218	Ccnb2	cyclin B2 [Source:MGI Symbol%3BAcc:MGI:88311]
ENSMUSG00000036814	Slc6a20a	solute carrier family 6 (neurotransmitter transporter)%2C member 20A [Source:MGI Symbol%3BAcc:MGI:2143217]	ENSMUSG00000097413	A830052D11Rik	RIKEN cDNA A830052D11 gene [Source:MGI Symbol%3BAcc:MGI:3028074]	ENSMUSG00000032297	Celf6	CUGBP%2C Elav-like family member 6 [Source:MGI Symbol%3BAcc:MGI:1923433]
ENSMUSG00000116753	Gm36001	predicted gene%2C 36001 [Source:MGI Symbol%3BAcc:MGI:5595160]	ENSMUSG00000049709	Nlrp10	NLR family%2C pyrin domain containing 10 [Source:MGI Symbol%3BAcc:MGI:24440	ENSMUSG00000032417	Rwdd2a	RWD domain containing 2A [Source:MGI Symbol%3BAcc:MGI:1916769]
ENSMUSG00000094530	Gm21399	predicted gene%2C 21399 [Source:MGI Symbol%3BAcc:MGI:5434754]	ENSMUSG00000030781	Slc5a2	solute carrier family 5 (sodium/glucose cotransporter)%2C member 2 [Source:MGI Symbol%3BAcc:MGI:2181411]	ENSMUSG00000032436	Cmtm7	CKLF-like MARVEL transmembrane domain containing 7 [Source:MGI Symbol%3BAcc:MGI:2447166]
ENSMUSG00000006585	Cdt1	chromatin licensing and DNA replication factor 1 [Source:MGI Symbol%3BAcc:MGI:1914427]	ENSMUSG00000010841	Cfap97d1	CFAP97 domain containing 1 [Source:MGI Symbol%3BAcc:MGI:1922687]	ENSMUSG00000032491	Nradd	neurotrophin receptor associated death domain [Source:MGI Symbol%3BAcc:MGI:1914419]
ENSMUSG00000051985	Igfn1	immunoglobulin-like and fibronectin type III domain containing 1 [Source:MGI Symbol%3BAcc:MGI:3045352]	ENSMUSG00000015880	Ncapg	non-SMC condensin I complex%2C subunit G [Source:MGI Symbol%3BAcc:MGI:1930197]	ENSMUSG00000042787	Exog	endo/exonuclease (5'-3')%2C endonuclease G-like [Source:MGI Symbol%3BAcc:MGI:2143333]

ENSMUSG00000110170	St6galnac2	ST6 (alpha-N-acetyl-neuraminyl-2%2C3-beta-galactosyl-1%2C3)-N-acetylgalactosaminide alpha-2%2C6-sialyltransferase 2 [Source:MGI Symbol%3BAcc:MGI:10755	ENSMUSG00000104106	Gm10848	predicted gene 10848 [Source:MGI Symbol%3BAcc:MGI:3641917]	ENSMUSG00000032609	Klhdc8b	kelch domain containing 8B [Source:MGI Symbol%3BAcc:MGI:1925517]
ENSMUSG00000021250	Fos	FBJ osteosarcoma oncogene [Source:MGI Symbol%3BAcc:MGI:95574]	ENSMUSG00000048349	Pou4f1	POU domain%2C class 4%2C transcription factor 1 [Source:MGI Symbol%3BAcc:MGI:10252	ENSMUSG00000078853	Igtp	interferon gamma induced GTPase [Source:MGI Symbol%3BAcc:MGI:10772
ENSMUSG00000004105	Angptl2	angiopoietin-like 2 [Source:MGI Symbol%3BAcc:MGI:1347002]	ENSMUSG00000086450	Macrod2os1	mono-ADP ribosylhydrolase 2%2C opposite strand 1 [Source:MGI Symbol%3BAcc:MGI:36501	ENSMUSG00000037989	Wnk2	WNK lysine deficient protein kinase 2 [Source:MGI Symbol%3BAcc:MGI:1922857]
ENSMUSG00000072662	Mansc4	MANSC domain containing 4 [Source:MGI Symbol%3BAcc:MGI:3645619]	ENSMUSG00000120826	-	novel transcript%2C antisense to Hexim2	ENSMUSG00000039158	Akna	AT-hook transcription factor [Source:MGI Symbol%3BAcc:MGI:2140340]
ENSMUSG00000030972	Acsm5	acyl-CoA synthetase medium-chain family member 5 [Source:MGI Symbol%3BAcc:MGI:2444086]	ENSMUSG00000039728	Slc6a5	solute carrier family 6 (neurotransmitter transporter%2C glycine)%2C member 5 [Source:MGI	ENSMUSG00000041355	Ssr2	signal sequence receptor%2C beta [Source:MGI Symbol%3BAcc:MGI:1913506]
ENSMUSG00000024043	Arhgap28	Rho GTPase activating protein 28 [Source:MGI Symbol%3BAcc:MGI:2147003]	ENSMUSG00000081076	#N/A	#N/A	ENSMUSG00000037892	Pcdh18	protocadherin 18 [Source:MGI Symbol%3BAcc:MGI:1920423]
ENSMUSG00000027171	Prrg4	proline rich Gla (G-carboxyglutamic acid) 4 (transmembrane) [Source:MGI Symbol%3BAcc:MGI:2442211]	ENSMUSG00000113456	Gm30738	predicted gene%2C 30738 [Source:MGI Symbol%3BAcc:MGI:5589897]	ENSMUSG00000028521	Slc35d1	solute carrier family 35 (UDP-glucuronic acid/UDP-N-acetylgalactosamine dual transporter)%2C member D1 [Source:MGI Symbol%3BAcc:MGI:21403
ENSMUSG00000021647	Cartpt	CART prepropeptide [Source:MGI Symbol%3BAcc:MGI:1351330]	ENSMUSG00000002204	Napsa	napsin A aspartic peptidase [Source:MGI Symbol%3BAcc:MGI:1093665]	ENSMUSG00000045672	Col27a1	collagen%2C type XXVII%2C alpha 1 [Source:MGI Symbol%3BAcc:MGI:26721
ENSMUSG00000121093	-	novel transcript	ENSMUSG00000031170	Slc38a5	solute carrier family 38%2C member 5 [Source:MGI Symbol%3BAcc:MGI:2148066]	ENSMUSG00000033478	Fhip2a	FHF complex subunit HOOK interacting protein 2A [Source:MGI Symbol%3BAcc:MGI:2147545]
ENSMUSG00000030108	Slc6a13	solute carrier family 6 (neurotransmitter transporter%2C GABA)%2C member 13 [Source:MGI Symbol%3BAcc:MGI:95629	ENSMUSG00000110148	5830408C22Rik	RIKEN cDNA 5830408C22 gene [Source:MGI Symbol%3BAcc:MGI:1921994]	ENSMUSG00000042286	Stab1	stabilin 1 [Source:MGI Symbol%3BAcc:MGI:2178742]
ENSMUSG00000032179	Bmp5	bone morphogenetic protein 5 [Source:MGI Symbol%3BAcc:MGI:88181]	ENSMUSG00000027824	Vmn2r1	vomeronasal 2%2C receptor 1 [Source:MGI Symbol%3BAcc:MGI:3645892]	ENSMUSG00000036907	C1ql2	complement component 1%2C q subcomponent-like 2 [Source:MGI Symbol%3BAcc:MGI:3032521]
ENSMUSG00000053783	1700016K19Rik	RIKEN cDNA 1700016K19 gene [Source:MGI Symbol%3BAcc:MGI:1921480]	ENSMUSG00000107859	Gm30731	predicted gene%2C 30731 [Source:MGI Symbol%3BAcc:MGI:5589890]	ENSMUSG00000040354	Mars1	methionine-tRNA synthetase 1 [Source:MGI Symbol%3BAcc:MGI:1345633]
ENSMUSG00000036972	Zic4	zinc finger protein of the cerebellum 4 [Source:MGI Symbol%3BAcc:MGI:107201]	ENSMUSG00000106201	Gm7815	predicted gene 7815 [Source:MGI Symbol%3BAcc:MGI:3646285]	ENSMUSG00000040035	Disp2	dispatched RND transporter family member 2 [Source:MGI Symbol%3BAcc:MGI:2388733]
ENSMUSG00000090021	Gm6493	predicted gene 6493 [Source:MGI Symbol%3BAcc:MGI:3644797]	ENSMUSG00000051212	Gpr183	G protein-coupled receptor 183 [Source:MGI Symbol%3BAcc:MGI:2442034]	ENSMUSG00000071172	Srsf3	serine and arginine-rich splicing factor 3 [Source:MGI Symbol%3BAcc:MGI:98285
ENSMUSG00000112121	C230072F16Rik	RIKEN cDNA C230072F16 gene [Source:MGI Symbol%3BAcc:MGI:2444727]	ENSMUSG00000083603	Gm14810	predicted gene 14810 [Source:MGI Symbol%3BAcc:MGI:3705403]	ENSMUSG00000034796	Cpne7	copine VII [Source:MGI Symbol%3BAcc:MGI:2142747]
ENSMUSG00000105152	Gm42864	predicted gene 42864 [Source:MGI Symbol%3BAcc:MGI:5663001]	ENSMUSG00000087054	Gm12405	predicted gene 12405 [Source:MGI Symbol%3BAcc:MGI:3649392]	ENSMUSG00000035051	Dhx57	DEAH (Asp-Glu-Ala-Asp/His) box polypeptide 57 [Source:MGI Symbol%3BAcc:MGI:2147067]
ENSMUSG00000112812	Gm47415	predicted gene%2C 47415 [Source:MGI Symbol%3BAcc:MGI:6096352]	ENSMUSG00000096351	Samd11	sterile alpha motif domain containing 11 [Source:MGI Symbol%3BAcc:MGI:2446220]	ENSMUSG00000035849	Krt222	keratin 222 [Source:MGI Symbol%3BAcc:MGI:2442728]
ENSMUSG00000098050	Gm5345	predicted gene 5345 [Source:MGI Symbol%3BAcc:MGI:3644320]	ENSMUSG00000116237	Gm30339	predicted gene%2C 30339 [Source:MGI Symbol%3BAcc:MGI:5589498]	ENSMUSG00000034098	Fstl5	follistatin-like 5 [Source:MGI Symbol%3BAcc:MGI:2442179]
ENSMUSG00000097286	Gm26684	predicted gene%2C 26684 [Source:MGI Symbol%3BAcc:MGI:5477178]	ENSMUSG00000113650	Gm47826	predicted gene%2C 47826 [Source:MGI Symbol%3BAcc:MGI:6097020]	ENSMUSG00000035851	Ythdc1	YTH domain containing 1 [Source:MGI Symbol%3BAcc:MGI:2443713]
ENSMUSG00000051456	Hspb3	heat shock protein 3 [Source:MGI Symbol%3BAcc:MGI:1928479]	ENSMUSG00000114496	#N/A	#N/A	ENSMUSG00000038507	Parp12	poly (ADP-ribose) polymerase family%2C member 12 [Source:MGI Symbol%3BAcc:MGI:2143990]
ENSMUSG00000022032	Scara5	scavenger receptor class A%2C member 5 [Source:MGI Symbol%3BAcc:MGI:1918395]	ENSMUSG00000115970	8430426J06Rik	RIKEN cDNA 8430426J06 gene [Source:MGI Symbol%3BAcc:MGI:1925352]	ENSMUSG00000039182	AW209491	expressed sequence AW209491 [Source:MGI Symbol%3BAcc:MGI:2145422]
ENSMUSG00000091243	Vgll3	vestigial like family member 3 [Source:MGI Symbol%3BAcc:MGI:1920819]	ENSMUSG00000031304	Il2rg	interleukin 2 receptor%2C gamma chain [Source:MGI Symbol%3BAcc:MGI:96551]	ENSMUSG00000039307	Hexdc	hexosaminidase (glycosyl hydrolase family 20%2C catalytic domain) containing [Source:MGI Symbol%3BAcc:MGI:3605542]

ENSMUSG00000036594	H2-Aa	histocompatibility 2%2C class II antigen A%2C alpha [Source:MGI Symbol%3BAcc:MGI:95895	ENSMUSG00000037548	H2-DMb2	histocompatibility 2%2C class II%2C locus Mb2 [Source:MGI Symbol%3BAcc:MGI:95923	ENSMUSG00000040747	Cd53	CD53 antigen [Source:MGI Symbol%3BAcc:MGI:88341]
ENSMUSG00000055523	Gucy2g	guanylate cyclase 2g [Source:MGI Symbol%3BAcc:MGI:106025]	ENSMUSG00000096255	Dynlt1b	dynein light chain Tctex-type 1B [Source:MGI Symbol%3BAcc:MGI:98643]	ENSMUSG00000038760	Trhr	thyrotropin releasing hormone receptor [Source:MGI Symbol%3BAcc:MGI:98824
ENSMUSG00000097697	4833412C05Rik	RIKEN cDNA 4833412C05 gene [Source:MGI Symbol%3BAcc:MGI:1921154]	ENSMUSG00000045551	Fpr1	formyl peptide receptor 1 [Source:MGI Symbol%3BAcc:MGI:107443]	ENSMUSG00000042613	Pbxip1	pre B cell leukemia transcription factor interacting protein 1 [Source:MGI Symbol%3BAcc:MGI:2441670]
ENSMUSG00000057068	Fam47e	family with sequence similarity 47%2C member E [Source:MGI Symbol%3BAcc:MGI:2686227]	ENSMUSG00000120309	-	novel transcript	ENSMUSG00000035342	Lzts2	leucine zipper%2C putative tumor suppressor 2 [Source:MGI Symbol%3BAcc:MGI:2385095]
ENSMUSG00000041703	Zic5	zinc finger protein of the cerebellum 5 [Source:MGI Symbol%3BAcc:MGI:1929518]	ENSMUSG00000093726	Gm20667	predicted gene 20667 [Source:MGI Symbol%3BAcc:MGI:5313114]	ENSMUSG00000033061	Resp18	regulated endocrine-specific protein 18 [Source:MGI Symbol%3BAcc:MGI:10982
ENSMUSG00000111340	Gm47171	predicted gene%2C 47171 [Source:MGI Symbol%3BAcc:MGI:6095951]	ENSMUSG00000114970	Gm49069	predicted gene%2C 49069 [Source:MGI Symbol%3BAcc:MGI:6118452]	ENSMUSG00000039031	Arhgap18	Rho GTPase activating protein 18 [Source:MGI Symbol%3BAcc:MGI:1921160]
ENSMUSG00000029718	Pcolce	procollagen C-endopeptidase enhancer protein [Source:MGI Symbol%3BAcc:MGI:10509	ENSMUSG00000068794	Col28a1	collagen%2C type XXVIII%2C alpha 1 [Source:MGI Symbol%3BAcc:MGI:26853	ENSMUSG00000036422	Pcdh8	protocadherin 8 [Source:MGI Symbol%3BAcc:MGI:1306800]
ENSMUSG00000039903	Eva1c	eva-1 homolog C (C. elegans) [Source:MGI Symbol%3BAcc:MGI:1918217]	ENSMUSG00000100833	Gm28988	predicted gene 28988 [Source:MGI Symbol%3BAcc:MGI:5579694]	ENSMUSG00000042353	Frem3	Fras1 related extracellular matrix protein 3 [Source:MGI Symbol%3BAcc:MGI:2685641]
ENSMUSG00000048424	Ranbp3l	RAN binding protein 3-like [Source:MGI Symbol%3BAcc:MGI:2444654]	ENSMUSG00000104761	Gm43511	predicted gene 43511 [Source:MGI Symbol%3BAcc:MGI:5663648]	ENSMUSG00000038563	Efl1	elongation factor like GTPase 1 [Source:MGI Symbol%3BAcc:MGI:2141969]
ENSMUSG00000056162	Cndp1	carnosine dipeptidase 1 (metallopeptidase M20 family) [Source:MGI Symbol%3BAcc:MGI:2451097]	ENSMUSG00000106607	Gm43178	predicted gene 43178 [Source:MGI Symbol%3BAcc:MGI:5663315]	ENSMUSG00000038168	P3h2	prolyl 3-hydroxylase 2 [Source:MGI Symbol%3BAcc:MGI:2146663]
ENSMUSG00000026726	Cubn	cubilin (intrinsic factor-cobalamin receptor) [Source:MGI Symbol%3BAcc:MGI:1931256]	ENSMUSG00000120747	-	novel transcript	ENSMUSG00000037086	Prr32	proline rich 32 [Source:MGI Symbol%3BAcc:MGI:1916050]
ENSMUSG00000083692	Gm9575	predicted gene 9575 [Source:MGI Symbol%3BAcc:MGI:3779984]	ENSMUSG00000121045	-	novel transcript%2C antisense to Tab1	ENSMUSG00000041025	Iffo2	intermediate filament family orphan 2 [Source:MGI Symbol%3BAcc:MGI:21406
ENSMUSG00000078238	Gm12854	predicted gene 12854 [Source:MGI Symbol%3BAcc:MGI:3650410]	ENSMUSG00000093383	Gm20642	predicted gene 20642 [Source:MGI Symbol%3BAcc:MGI:5313089]	ENSMUSG00000041710	Trpc5	transient receptor potential cation channel%2C subfamily C%2C member 5 [Source:MGI
ENSMUSG00000059852	Kcng2	potassium voltage-gated channel%2C subfamily G%2C member 2 [Source:MGI Symbol%3BAcc:MGI:36946	ENSMUSG00000039873	Neurl2	neuralized E3 ubiquitin protein ligase 2 [Source:MGI Symbol%3BAcc:MGI:3043305]	ENSMUSG00000041974	Spidr	scaffolding protein involved in DNA repair [Source:MGI Symbol%3BAcc:MGI:1924834]
ENSMUSG00000037868	Egr2	early growth response 2 [Source:MGI Symbol%3BAcc:MGI:95296]	ENSMUSG00000121211	-	novel transcript	ENSMUSG00000040723	Rcsd1	RCSD domain containing 1 [Source:MGI Symbol%3BAcc:MGI:2676394]
ENSMUSG00000038151	Prdm1	PR domain containing 1%2C with ZNF domain [Source:MGI Symbol%3BAcc:MGI:99655	ENSMUSG00000058163	Gm5431	predicted gene 5431 [Source:MGI Symbol%3BAcc:MGI:3645205]	ENSMUSG00000025597	Klhl4	kelch-like 4 [Source:MGI Symbol%3BAcc:MGI:2442829]
ENSMUSG00000046794	Ppp1r3b	protein phosphatase 1%2C regulatory subunit 3B [Source:MGI Symbol%3BAcc:MGI:2177268]	ENSMUSG00000086021	Gm15767	predicted gene 15767 [Source:MGI Symbol%3BAcc:MGI:3783209]	ENSMUSG00000035181	Heatr5a	HEAT repeat containing 5A [Source:MGI Symbol%3BAcc:MGI:2444133]
ENSMUSG00000028214	Gem	GTP binding protein (gene overexpressed in skeletal muscle) [Source:MGI Symbol%3BAcc:MGI:99844	ENSMUSG00000046610	Oacyl	O-acyltransferase like [Source:MGI Symbol%3BAcc:MGI:2442915]	ENSMUSG00000054737	Zfp182	zinc finger protein 182 [Source:MGI Symbol%3BAcc:MGI:2442220]
ENSMUSG00000019929	Dcn	decorin [Source:MGI Symbol%3BAcc:MGI:94872]	ENSMUSG00000079259	Trim71	tripartite motif-containing 71 [Source:MGI Symbol%3BAcc:MGI:2685973]	ENSMUSG00000034118	Tpst1	protein-tyrosine sulfotransferase 1 [Source:MGI Symbol%3BAcc:MGI:1298231]
ENSMUSG00000046318	Ccbe1	collagen and calcium binding EGF domains 1 [Source:MGI Symbol%3BAcc:MGI:2445053]	ENSMUSG00000035683	Melk	maternal embryonic leucine zipper kinase [Source:MGI Symbol%3BAcc:MGI:106924]	ENSMUSG00000024965	Fermt3	fermitin family member 3 [Source:MGI Symbol%3BAcc:MGI:2147790]
ENSMUSG00000097182	A830009L08Rik	RIKEN cDNA A830009L08 gene [Source:MGI Symbol%3BAcc:MGI:2443762]	ENSMUSG00000039997	Ifi203	interferon activated gene 203 [Source:MGI Symbol%3BAcc:MGI:96428]	ENSMUSG00000005501	Usp40	ubiquitin specific peptidase 40 [Source:MGI Symbol%3BAcc:MGI:2443184]
ENSMUSG00000040152	Thbs1	thrombospondin 1 [Source:MGI Symbol%3BAcc:MGI:98737]	ENSMUSG00000090016	Gm16578	predicted gene 16578 [Source:MGI Symbol%3BAcc:MGI:4414998]	ENSMUSG00000030270	Cpne9	copine family member IX [Source:MGI Symbol%3BAcc:MGI:2443052]
ENSMUSG00000090523	Gypc	glycophorin C [Source:MGI Symbol%3BAcc:MGI:1098566]	ENSMUSG00000051147	Nat2	N-acetyltransferase 2 (arylamine N-acetyltransferase) [Source:MGI Symbol%3BAcc:MGI:10920	ENSMUSG00000042647	Acad12	acyl-Coenzyme A dehydrogenase family%2C member 12 [Source:MGI Symbol%3BAcc:MGI:2443320]

ENSMUSG00000100789	Gm29508	predicted gene 29508 [Source:MGI Symbol%3BAcc:MGI:5580214]	ENSMUSG00000108166	Gm44017	predicted gene%2C 44017 [Source:MGI Symbol%3BAcc:MGI:5690409]	ENSMUSG00000038255	Neurod2	neurogenic differentiation 2 [Source:MGI Symbol%3BAcc:MGI:107755]
ENSMUSG00002075524	-	-	ENSMUSG00000085039	Gm15927	predicted gene 15927 [Source:MGI Symbol%3BAcc:MGI:3801889]	ENSMUSG00000039405	Prss23	protease%2C serine 23 [Source:MGI Symbol%3BAcc:MGI:1923703]
ENSMUSG00000033726	Emx1	empty spiracles homeobox 1 [Source:MGI Symbol%3BAcc:MGI:95387]	ENSMUSG00000097616	1110019D14Rik	RIKEN cDNA 1110019D14 gene [Source:MGI Symbol%3BAcc:MGI:1923561]	ENSMUSG00000036555	Iqce	IQ motif containing E [Source:MGI Symbol%3BAcc:MGI:1921489]
ENSMUSG00000071497	Nutf2-ps1	nuclear transport factor 2%2C pseudogene 1 [Source:MGI Symbol%3BAcc:MGI:108008]	ENSMUSG00000105748	Gm43088	predicted gene 43088 [Source:MGI Symbol%3BAcc:MGI:5663225]	ENSMUSG00000035595	Fam174c	family with sequence similarity 174%2C member C [Source:MGI Symbol%3BAcc:MGI:1917020]
ENSMUSG00000046618	Olfml2a	olfactomedin-like 2A [Source:MGI Symbol%3BAcc:MGI:2444741]	ENSMUSG00000033080	Vsx1	visual system homeobox 1 [Source:MGI Symbol%3BAcc:MGI:1890816]	ENSMUSG00000033420	Antxr1	anthrax toxin receptor 1 [Source:MGI Symbol%3BAcc:MGI:1916788]
ENSMUSG00000092086	Gm6793	predicted gene 6793 [Source:MGI Symbol%3BAcc:MGI:3643578]	ENSMUSG00000096780	Tmem181b-ps	transmembrane protein 181B%2C pseudogene [Source:MGI Symbol%3BAcc:MGI:3779544]	ENSMUSG00000034463	Scara3	scavenger receptor class A%2C member 3 [Source:MGI Symbol%3BAcc:MGI:2444418]
ENSMUSG00000031375	Bgn	biglycan [Source:MGI Symbol%3BAcc:MGI:88158]	ENSMUSG00000109942	Gm45262	predicted gene 45262 [Source:MGI Symbol%3BAcc:MGI:5791098]	ENSMUSG00000040552	C3ar1	complement component 3a receptor 1 [Source:MGI Symbol%3BAcc:MGI:1097680]
ENSMUSG00000092094	Zfp804b	zinc finger protein 804B [Source:MGI Symbol%3BAcc:MGI:2685287]	ENSMUSG00000066315	Gm12918	predicted gene 12918 [Source:MGI Symbol%3BAcc:MGI:3652005]	ENSMUSG00000037649	H2-DMa	histocompatibility 2%2C class II%2C locus DMa [Source:MGI Symbol%3BAcc:MGI:95921
ENSMUSG00000019892	Lrriq1	leucine-rich repeats and IQ motif containing 1 [Source:MGI Symbol%3BAcc:MGI:1922228]	ENSMUSG00000072844	G530011O06Rik	RIKEN cDNA G530011O06 gene [Source:MGI Symbol%3BAcc:MGI:3603513]	ENSMUSG00000042708	Shcbp1l	Shc SH2-domain binding protein 1-like [Source:MGI Symbol%3BAcc:MGI:1919086]
ENSMUSG00000103358	Gm37593	predicted gene%2C 37593 [Source:MGI Symbol%3BAcc:MGI:5610821]	ENSMUSG00000110266	Gm32742	predicted gene%2C 32742 [Source:MGI Symbol%3BAcc:MGI:5591901]	ENSMUSG00000035367	Rmi1	RecQ mediated genome instability 1 [Source:MGI Symbol%3BAcc:MGI:1921636]
ENSMUSG00000120873	-	novel transcript	ENSMUSG00000051455	Meioc	meiosis specific with coiled-coil domain [Source:MGI Symbol%3BAcc:MGI:26864	ENSMUSG00000032878	Ccdc85a	coiled-coil domain containing 85A [Source:MGI Symbol%3BAcc:MGI:24450
ENSMUSG00000044471	Lncpint	long non-protein coding RNA%2C Trp53 induced transcript [Source:MGI Symbol%3BAcc:MGI:2673128]	ENSMUSG00000094122	Rpl31-ps9	ribosomal protein L31%2C pseudogene 9 [Source:MGI Symbol%3BAcc:MGI:3704195]	ENSMUSG00000034422	Parp14	poly (ADP-ribose) polymerase family%2C member 14 [Source:MGI Symbol%3BAcc:MGI:1919489]
ENSMUSG00000007877	Tcap	titin-cap [Source:MGI Symbol%3BAcc:MGI:1330233]	ENSMUSG00000117442	1810073O08Rik	RIKEN cDNA 1810073O08 gene [Source:MGI Symbol%3BAcc:MGI:1919535]	ENSMUSG00000041120	Nbl1	NBL1%2C DAN family BMP antagonist [Source:MGI Symbol%3BAcc:MGI:104591]
ENSMUSG00000097146	Gm4211	predicted gene 4211 [Source:MGI Symbol%3BAcc:MGI:3782387]	ENSMUSG00000029784	Ssmem1	serine-rich single-pass membrane protein 1 [Source:MGI Symbol%3BAcc:MGI:1922897]	ENSMUSG00000024451	Arap3	ArfGAP with RhoGAP domain%2C ankyrin repeat and PH domain 3 [Source:MGI Symbol%3BAcc:MGI:2147274]
ENSMUSG00000063409	Lrrc43	leucine rich repeat containing 43 [Source:MGI Symbol%3BAcc:MGI:2685907]	ENSMUSG00000005540	Fcer2a	Fc receptor%2C IgE%2C low affinity II%2C alpha polypeptide [Source:MGI Symbol%3BAcc:MGI:95497	ENSMUSG00000034957	Cebpa	CCAAT/enhancer binding protein (C/EBP)%2C alpha [Source:MGI Symbol%3BAcc:MGI:99480
ENSMUSG00000061524	Zic2	zinc finger protein of the cerebellum 2 [Source:MGI Symbol%3BAcc:MGI:106679]	ENSMUSG00000102224	4930447F24Rik	RIKEN cDNA 4930447F24 gene [Source:MGI Symbol%3BAcc:MGI:1924123]	ENSMUSG00000018008	Cyth4	cytohesin 4 [Source:MGI Symbol%3BAcc:MGI:2441702]
ENSMUSG00000024210	Ip6k3	inositol hexaphosphate kinase 3 [Source:MGI Symbol%3BAcc:MGI:3045325]	ENSMUSG00000046080	Clec9a	C-type lectin domain family 9%2C member a [Source:MGI Symbol%3BAcc:MGI:2444608]	ENSMUSG00000033220	Rac2	Rac family small GTPase 2 [Source:MGI Symbol%3BAcc:MGI:97846]
ENSMUSG00000039110	Mycbpap	MYCBP associated protein [Source:MGI Symbol%3BAcc:MGI:2388726]	ENSMUSG00000029380	Cxcl1	chemokine (C-X-C motif) ligand 1 [Source:MGI Symbol%3BAcc:MGI:108068]	ENSMUSG00000042607	Asb4	ankyrin repeat and SOCS box-containing 4 [Source:MGI Symbol%3BAcc:MGI:1929751]
ENSMUSG00000029026	Trp73	transformation related protein 73 [Source:MGI Symbol%3BAcc:MGI:1336991]	ENSMUSG00000109602	Gm31597	predicted gene%2C 31597 [Source:MGI Symbol%3BAcc:MGI:5590756]	ENSMUSG00000036764	Dnajc12	DnaJ heat shock protein family (Hsp40) member C12 [Source:MGI Symbol%3BAcc:MGI:1353428]
ENSMUSG00000050737	Ptges	prostaglandin E synthase [Source:MGI Symbol%3BAcc:MGI:1927593]	ENSMUSG00000026715	Serpinc1	serine (or cysteine) peptidase inhibitor%2C clade C (antithrombin)%2C member 1 [Source:MGI Symbol%3BAcc:MGI:88095	ENSMUSG00000033880	Lgals3bp	lectin%2C galactoside-binding%2C soluble%2C 3 binding protein [Source:MGI Symbol%3BAcc:MGI:99554
ENSMUSG00000052387	Trpm3	transient receptor potential cation channel%2C subfamily M%2C member 3 [Source:MGI Symbol%3BAcc:MGI:24431	ENSMUSG00000092246	Gm20493	predicted gene 20493 [Source:MGI Symbol%3BAcc:MGI:5141958]	ENSMUSG00000038235	F11r	F11 receptor [Source:MGI Symbol%3BAcc:MGI:1321398]
ENSMUSG00000061878	Sphk1	sphingosine kinase 1 [Source:MGI Symbol%3BAcc:MGI:1316649]	ENSMUSG00000074065	Gm10617	predicted gene 10617 [Source:MGI Symbol%3BAcc:MGI:3708793]	ENSMUSG00000039648	Kyat1	kynurenine aminotransferase 1 [Source:MGI Symbol%3BAcc:MGI:1917516]
ENSMUSG00000082675	Gm6382	predicted gene 6382 [Source:MGI Symbol%3BAcc:MGI:3646438]	ENSMUSG00000034057	Myrfl	myelin regulatory factor-like [Source:MGI Symbol%3BAcc:MGI:2685085]	ENSMUSG00000036854	Hspb6	heat shock protein%2C alpha-crystallin-related%2C B6 [Source:MGI Symbol%3BAcc:MGI:26853

ENSMUSG00000090066	1110002E22Rik	RIKEN cDNA 1110002E22 gene [Source:MGI Symbol%3BAcc:MGI:1915066]	ENSMUSG00000107917	Gm44235	predicted gene%2C 44235 [Source:MGI Symbol%3BAcc:MGI:5690627]	ENSMUSG00000039313	Minar1	membrane integral NOTCH2 associated receptor 1 [Source:MGI Symbol%3BAcc:MGI:2667167]
ENSMUSG00000050931	Sgms2	sphingomyelin synthase 2 [Source:MGI Symbol%3BAcc:MGI:1921692]	ENSMUSG00000032717	Mdfi	MyoD family inhibitor [Source:MGI Symbol%3BAcc:MGI:107687]	ENSMUSG00000036480	Prss56	protease%2C serine 56 [Source:MGI Symbol%3BAcc:MGI:1916703]
ENSMUSG00000029661	Col1a2	collagen%2C type I%2C alpha 2 [Source:MGI Symbol%3BAcc:MGI:88468]	ENSMUSG00000085415	Selenok-ps1	selenoprotein K%2C pseudogene 1 [Source:MGI Symbol%3BAcc:MGI:36493	ENSMUSG00000033467	Crlf2	cytokine receptor-like factor 2 [Source:MGI Symbol%3BAcc:MGI:1889506]
ENSMUSG00000051435	Fhad1	forkhead-associated (FHA) phosphopeptide binding domain 1 [Source:MGI Symbol%3BAcc:MGI:19203	ENSMUSG00000089812	Gm15867	predicted gene 15867 [Source:MGI Symbol%3BAcc:MGI:3801862]	ENSMUSG00000037984	Neurod6	neurogenic differentiation 6 [Source:MGI Symbol%3BAcc:MGI:106593]
ENSMUSG00000034205	Loxl2	lysyl oxidase-like 2 [Source:MGI Symbol%3BAcc:MGI:2137913]	ENSMUSG00000087516	Tbx3os1	T-box 3%2C opposite strand 1 [Source:MGI Symbol%3BAcc:MGI:3780472]	ENSMUSG00000041797	Abca9	ATP-binding cassette%2C sub-family A (ABC1)%2C member 9 [Source:MGI Symbol%3BAcc:MGI:2386796]
ENSMUSG00000019102	Aldh3a1	aldehyde dehydrogenase family 3%2C subfamily A1 [Source:MGI Symbol%3BAcc:MGI:1353451]	ENSMUSG00000024939	Fam89b	family with sequence similarity 89%2C member B [Source:MGI Symbol%3BAcc:MGI:106595]	ENSMUSG00000040253	Gbp7	guanylate binding protein 7 [Source:MGI Symbol%3BAcc:MGI:2444421]
ENSMUSG00000023411	Nfatc4	nuclear factor of activated T cells%2C cytoplasmic%2C calcineurin dependent 4 [Source:MGI Symbol%3BAcc:MGI:1920431]	ENSMUSG00000102659	Gm37077	predicted gene%2C 37077 [Source:MGI Symbol%3BAcc:MGI:5610305]	ENSMUSG00000037731	Themis2	thymocyte selection associated family member 2 [Source:MGI Symbol%3BAcc:MGI:2446213]
ENSMUSG00000045775	Slc16a5	solute carrier family 16 (monocarboxylic acid transporters)%2C member 5 [Source:MGI Symbol%3BAcc:MGI:2443515]	ENSMUSG00000109337	Gm45047	predicted gene 45047 [Source:MGI Symbol%3BAcc:MGI:5753623]	ENSMUSG00000041459	Tardbp	TAR DNA binding protein [Source:MGI Symbol%3BAcc:MGI:2387629]
ENSMUSG00000026875	Traf1	TNF receptor-associated factor 1 [Source:MGI Symbol%3BAcc:MGI:101836]	ENSMUSG00000101997	Gm28722	predicted gene 28722 [Source:MGI Symbol%3BAcc:MGI:5579428]	ENSMUSG00000036915	Kirrel2	kirre like nephrin family adhesion molecule 2 [Source:MGI Symbol%3BAcc:MGI:2442334]
ENSMUSG00000041390	Mdfic	MyoD family inhibitor domain containing [Source:MGI Symbol%3BAcc:MGI:104611]	ENSMUSG00000086108	Gm5602	predicted gene 5602 [Source:MGI Symbol%3BAcc:MGI:3645136]	ENSMUSG00000034617	Mtrr	5-methyltetrahydrofolate-homocysteine methyltransferase reductase [Source:MGI Symbol%3BAcc:MGI:1891037]
ENSMUSG00000046352	Gjb2	gap junction protein%2C beta 2 [Source:MGI Symbol%3BAcc:MGI:95720]	ENSMUSG00000121083	-	novel transcript%2C antisense to Tymp	ENSMUSG00000040482	Dxo	decapping exoribonuclease [Source:MGI Symbol%3BAcc:MGI:18904
ENSMUSG00000036395	Glb1l2	galactosidase%2C beta 1-like 2 [Source:MGI Symbol%3BAcc:MGI:2388283]	ENSMUSG00000083849	Gm13477	predicted gene 13477 [Source:MGI Symbol%3BAcc:MGI:3652109]	ENSMUSG00000057738	Sptan1	spectrin alpha%2C non-erythrocytic 1 [Source:MGI Symbol%3BAcc:MGI:98386]
ENSMUSG00000048616	Nog	noggin [Source:MGI Symbol%3BAcc:MGI:104327]	ENSMUSG00000112162	Gm48758	predicted gene%2C 48758 [Source:MGI Symbol%3BAcc:MGI:6098439]	ENSMUSG00000036896	C1qc	complement component 1%2C q subcomponent%2C C chain [Source:MGI
ENSMUSG00000024087	Cyp1b1	cytochrome P450%2C family 1%2C subfamily b%2C polypeptide 1 [Source:MGI Symbol%3BAcc:MGI:88590	ENSMUSG00000057058	Skap1	src family associated phosphoprotein 1 [Source:MGI Symbol%3BAcc:MGI:1925723]	ENSMUSG00000036905	C1qb	complement component 1%2C q subcomponent%2C beta polypeptide [Source:MGI Symbol%3BAcc:MGI:88224
ENSMUSG00000085609	1700016P03Rik	RIKEN cDNA 1700016P03 gene [Source:MGI Symbol%3BAcc:MGI:1922792]	ENSMUSG00000114893	Gm48684	predicted gene%2C 48684 [Source:MGI Symbol%3BAcc:MGI:6098310]	ENSMUSG00000039033	Tasp1	taspase%2C threonine aspartase 1 [Source:MGI Symbol%3BAcc:MGI:1923062]
ENSMUSG00000031273	Col4a6	collagen%2C type IV%2C alpha 6 [Source:MGI Symbol%3BAcc:MGI:2152695]	ENSMUSG00000112054	Gm33680	predicted gene%2C 33680 [Source:MGI Symbol%3BAcc:MGI:5592839]	ENSMUSG00000037725	Ckap2	cytoskeleton associated protein 2 [Source:MGI Symbol%3BAcc:MGI:1931797]
ENSMUSG00000032355	Mlip	muscular LMNA-interacting protein [Source:MGI Symbol%3BAcc:MGI:1916892]	ENSMUSG00000046487	Mospd4	motile sperm domain containing 4 [Source:MGI Symbol%3BAcc:MGI:1919326]	ENSMUSG00000041895	Wipi1	WD repeat domain%2C phosphoinositide interacting 1 [Source:MGI Symbol%3BAcc:MGI:1261864]
ENSMUSG00000024747	Aldh1a7	aldehyde dehydrogenase family 1%2C subfamily A7 [Source:MGI Symbol%3BAcc:MGI:1347050]	ENSMUSG00000068397	Gm10240	predicted gene 10240 [Source:MGI Symbol%3BAcc:MGI:3704454]	ENSMUSG00000035004	Igsf6	immunoglobulin superfamily%2C member 6 [Source:MGI Symbol%3BAcc:MGI:1891393]
ENSMUSG00000032419	Tbx18	T-box18 [Source:MGI Symbol%3BAcc:MGI:1923615]	ENSMUSG00000097921	Gm26576	predicted gene%2C 26576 [Source:MGI Symbol%3BAcc:MGI:5477070]	ENSMUSG00000022488	Nckap1l	NCK associated protein 1 like [Source:MGI Symbol%3BAcc:MGI:1926063]
ENSMUSG00000000126	Wnt9a	wingless-type MMTV integration site family%2C member 9A [Source:MGI Symbol%3BAcc:MGI:2446084]	ENSMUSG00000101122	Gm17971	predicted gene%2C 17971 [Source:MGI Symbol%3BAcc:MGI:5010156]	ENSMUSG00000041515	Irf8	interferon regulatory factor 8 [Source:MGI Symbol%3BAcc:MGI:96395]
ENSMUSG00000070336	Fbxo47	F-box protein 47 [Source:MGI Symbol%3BAcc:MGI:1920223]	ENSMUSG00000003882	Il7r	interleukin 7 receptor [Source:MGI Symbol%3BAcc:MGI:96562]	ENSMUSG00000036699	Zcchc12	zinc finger%2C CCHC domain containing 12 [Source:MGI Symbol%3BAcc:MGI:1919943]
ENSMUSG00000020641	Rsad2	radical S-adenosyl methionine domain containing 2 [Source:MGI Symbol%3BAcc:MGI:1929628]	ENSMUSG00000046408	1700067K01Rik	RIKEN cDNA 1700067K01 gene [Source:MGI Symbol%3BAcc:MGI:1920703]	ENSMUSG00000042312	S100a13	S100 calcium binding protein A13 [Source:MGI Symbol%3BAcc:MGI:109581]

ENSMUSG00000034460	Six4	sine oculis-related homeobox 4 [Source:MGI Symbol%3BAcc:MGI:106034]	ENSMUSG00000082309	Gm12123	predicted gene 12123 [Source:MGI Symbol%3BAcc:MGI:3652210]	ENSMUSG00000042106	Inka1	inka box actin regulator 1 [Source:MGI Symbol%3BAcc:MGI:1915426]
ENSMUSG00000062980	Cped1	cadherin-like and PC-esterase domain containing 1 [Source:MGI Symbol%3BAcc:MGI:2444814]	ENSMUSG00000026009	Icos	inducible T cell co-stimulator [Source:MGI Symbol%3BAcc:MGI:1858745]	ENSMUSG00000056608	Chd9	chromodomain helicase DNA binding protein 9 [Source:MGI Symbol%3BAcc:MGI:1924001]
ENSMUSG00000103050	Gm38273	predicted gene%2C 38273 [Source:MGI Symbol%3BAcc:MGI:5611501]	ENSMUSG00000049160	Tex50	testis expressed 50 [Source:MGI Symbol%3BAcc:MGI:1922183]	ENSMUSG00000042195	Slc35f2	solute carrier family 35%2C member F2 [Source:MGI Symbol%3BAcc:MGI:1919272]
ENSMUSG00000029135	Fosl2	fos-like antigen 2 [Source:MGI Symbol%3BAcc:MGI:102858]	ENSMUSG00000007080	Pole	polymerase (DNA directed)%2C epsilon [Source:MGI Symbol%3BAcc:MGI:1196391]	ENSMUSG00000037070	Rbmxl1	RNA binding motif protein%2C X-linked like-1 [Source:MGI Symbol%3BAcc:MGI:1343045]
ENSMUSG00000026712	Mrc1	mannose receptor%2C C type 1 [Source:MGI Symbol%3BAcc:MGI:97142]	ENSMUSG00000080006	Rps19-ps7	ribosomal protein S19%2C pseudogene 7 [Source:MGI Symbol%3BAcc:MGI:3652235]	ENSMUSG00000031506	Ptpn7	protein tyrosine phosphatase%2C non-receptor type 7 [Source:MGI Symbol%3BAcc:MGI:21568
ENSMUSG00000044071	Tafa2	TAFA chemokine like family member 2 [Source:MGI Symbol%3BAcc:MGI:21436	ENSMUSG00000117430	Gm49968	predicted gene%2C 49968 [Source:MGI Symbol%3BAcc:MGI:6275235]	ENSMUSG00000040841	Six5	sine oculis-related homeobox 5 [Source:MGI Symbol%3BAcc:MGI:106220]
ENSMUSG00000078249	Hmga1b	high mobility group AT-hook 1B [Source:MGI Symbol%3BAcc:MGI:96161]	ENSMUSG00000117050	1700023B13Rik	RIKEN cDNA 1700023B13 gene [Source:MGI Symbol%3BAcc:MGI:1920529]	ENSMUSG00000034708	Grn	granulin [Source:MGI Symbol%3BAcc:MGI:95832]
ENSMUSG00000019970	Sgk1	serum/glucocorticoid regulated kinase 1 [Source:MGI Symbol%3BAcc:MGI:1340062]	ENSMUSG00000106010	Gm42616	predicted gene 42616 [Source:MGI Symbol%3BAcc:MGI:5662753]	ENSMUSG00000071203	Naip5	NLR family%2C apoptosis inhibitory protein 5 [Source:MGI Symbol%3BAcc:MGI:1298220]
ENSMUSG00000026604	Ptpn14	protein tyrosine phosphatase%2C non-receptor type 14 [Source:MGI Symbol%3BAcc:MGI:10246	ENSMUSG00000113722	Snhg10	small nucleolar RNA host gene 10 [Source:MGI Symbol%3BAcc:MGI:1916684]	ENSMUSG00000048406	B330016D10Rik	RIKEN cDNA B330016D10 gene [Source:MGI Symbol%3BAcc:MGI:2444063]
ENSMUSG00000032807	Alox12b	arachidonate 12-lipoxygenase%2C 12R type [Source:MGI Symbol%3BAcc:MGI:1274782]	ENSMUSG00000109293	Dcst2	DC-STAMP domain containing 2 [Source:MGI Symbol%3BAcc:MGI:2685606]	ENSMUSG00000049387	Cox7b2	cytochrome c oxidase subunit 7B2 [Source:MGI Symbol%3BAcc:MGI:1925424]
ENSMUSG00000039676	Capsl	calcyphosine-like [Source:MGI Symbol%3BAcc:MGI:1922818]	ENSMUSG00000054885	4930578G10Rik	RIKEN cDNA 4930578G10 gene [Source:MGI Symbol%3BAcc:MGI:1923202]	ENSMUSG00000051669	AU021092	expressed sequence AU021092 [Source:MGI Symbol%3BAcc:MGI:2146559]
ENSMUSG00000018752	Tnfsfm13	tumor necrosis factor (ligand) superfamily%2C membrane-bound member 13 [Source:MGI Symbol%3BAcc:MGI:3845075]	ENSMUSG00000091996	BC049352	cDNA sequence BC049352 [Source:MGI Symbol%3BAcc:MGI:3040681]	ENSMUSG00000028399	Ptprd	protein tyrosine phosphatase%2C receptor type%2C D [Source:MGI Symbol%3BAcc:MGI:97812]
ENSMUSG00000046561	Arsj	arylsulfatase J [Source:MGI Symbol%3BAcc:MGI:2443513]	ENSMUSG00000118521	Gm52948	predicted gene%2C 52948 [Source:MGI Symbol%3BAcc:MGI:6388827]	ENSMUSG00000047146	Tet1	tet methylcytosine dioxygenase 1 [Source:MGI Symbol%3BAcc:MGI:10986
ENSMUSG00000027004	Frzb	frizzled-related protein [Source:MGI Symbol%3BAcc:MGI:892032]	ENSMUSG00000083670	Gm6829	predicted pseudogene 6829 [Source:MGI Symbol%3BAcc:MGI:3648590]	ENSMUSG00000049555	Tmie	transmembrane inner ear [Source:MGI Symbol%3BAcc:MGI:2159400]
ENSMUSG00000040562	Gstm2	glutathione S-transferase%2C mu 2 [Source:MGI Symbol%3BAcc:MGI:95861]	ENSMUSG00000077347	Gm24671	predicted gene%2C 24671 [Source:MGI Symbol%3BAcc:MGI:5454448]	ENSMUSG00000047773	Ankfn1	ankyrin-repeat and fibronectin type III domain containing 1 [Source:MGI Symbol%3BAcc:MGI:2686021]
ENSMUSG00000031216	Stard8	START domain containing 8 [Source:MGI Symbol%3BAcc:MGI:2448556]	ENSMUSG00000101210	Gm28720	predicted gene 28720 [Source:MGI Symbol%3BAcc:MGI:5579426]	ENSMUSG00000047090	Tmem198b	transmembrane protein 198b [Source:MGI Symbol%3BAcc:MGI:1921077]
ENSMUSG00000030887	Pdzd9	PDZ domain containing 9 [Source:MGI Symbol%3BAcc:MGI:1915233]	ENSMUSG00000098758	7630403G23Rik	RIKEN cDNA 7630403G23 gene [Source:MGI Symbol%3BAcc:MGI:1924369]	ENSMUSG00000050315	Synpo2	synaptopodin 2 [Source:MGI Symbol%3BAcc:MGI:2153070]
ENSMUSG00000056531	Ccdc18	coiled-coil domain containing 18 [Source:MGI Symbol%3BAcc:MGI:1922974]	ENSMUSG00000085735	Mettl5os	methyltransferase like 5%2C opposite strand [Source:MGI Symbol%3BAcc:MGI:19225	ENSMUSG00000044795	Cyb5d1	cytochrome b5 domain containing 1 [Source:MGI Symbol%3BAcc:MGI:2685586]
ENSMUSG00000079550	Mpp4	membrane protein%2C palmitoylated 4 (MAGUK p55 subfamily member 4) [Source:MGI Symbol%3BAcc:MGI:2386681]	ENSMUSG00000083633	Gm13312	predicted gene 13312 [Source:MGI Symbol%3BAcc:MGI:3651094]	ENSMUSG00000057895	Zfp105	zinc finger protein 105 [Source:MGI Symbol%3BAcc:MGI:1277119]
ENSMUSG00000060586	H2-Eb1	histocompatibility 2%2C class II antigen E beta [Source:MGI Symbol%3BAcc:MGI:95901]	ENSMUSG00000022861	Dgkg	diacylglycerol kinase%2C gamma [Source:MGI Symbol%3BAcc:MGI:105060]	ENSMUSG00000024885	Aldh3b1	aldehyde dehydrogenase 3 family%2C member B1 [Source:MGI Symbol%3BAcc:MGI:1914939]
ENSMUSG00000040121	Rep15	RAB15 effector protein [Source:MGI Symbol%3BAcc:MGI:1913782]	ENSMUSG00000081221	Gm14760	predicted gene 14760 [Source:MGI Symbol%3BAcc:MGI:3709612]	ENSMUSG00000049988	Lrrc25	leucine rich repeat containing 25 [Source:MGI Symbol%3BAcc:MGI:2445284]
ENSMUSG00000005251	Ripk4	receptor-interacting serine-threonine kinase 4 [Source:MGI Symbol%3BAcc:MGI:1919638]	ENSMUSG00000102193	Gm7299	predicted gene 7299 [Source:MGI Symbol%3BAcc:MGI:3648716]	ENSMUSG00000048327	Ckap2l	cytoskeleton associated protein 2-like [Source:MGI Symbol%3BAcc:MGI:1917716]
ENSMUSG00000033542	Arhgef5	Rho guanine nucleotide exchange factor (GEF) 5 [Source:MGI Symbol%3BAcc:MGI:1858952]	ENSMUSG00000059645	Gm7361	predicted gene 7361 [Source:MGI Symbol%3BAcc:MGI:3805965]	ENSMUSG00000051146	Camk2n2	calcium/calmodulin-dependent protein kinase II inhibitor 2 [Source:MGI Symbol%3BAcc:MGI:1920297]

ENSMUSG00000086022	Rad51ap2	RAD51 associated protein 2 [Source:MGI Symbol%3BAcc:MGI:3644580]	ENSMUSG00000046242	Nme9	NME/NM23 family member 9 [Source:MGI Symbol%3BAcc:MGI:4359686]	ENSMUSG00000020573	Pik3cg	phosphatidylinositol-4%2C5-bisphosphate 3-kinase catalytic subunit gamma [Source:MGI Symbol%3BAcc:MGI:1353576]
ENSMUSG00000035202	Lars2	leucyl-tRNA synthetase%2C mitochondrial [Source:MGI	ENSMUSG00000032380	Dapk2	death-associated protein kinase 2 [Source:MGI Symbol%3BAcc:MGI:1341297]	ENSMUSG00000024457	Trim26	tripartite motif-containing 26 [Source:MGI Symbol%3BAcc:MGI:1337056]
ENSMUSG00000121280	-	novel transcript%2C sense intronic to Kansl1l	ENSMUSG00000068129	Cst7	cystatin F (leukocystatin) [Source:MGI Symbol%3BAcc:MGI:1298217]	ENSMUSG00000079470	Utp14b	UTP14B small subunit processome component [Source:MGI Symbol%3BAcc:MGI:2445092]
ENSMUSG00000099760	Gm28800	predicted gene 28800 [Source:MGI Symbol%3BAcc:MGI:5579506]	ENSMUSG00000031147	Magix	MAGI family member%2C X-linked [Source:MGI Symbol%3BAcc:MGI:1859644]	ENSMUSG00000049404	Rarres1	retinoic acid receptor responder (tazarotene induced) 1 [Source:MGI Symbol%3BAcc:MGI:1924461]
ENSMUSG00000118257	9630014M24Rik	RIKEN cDNA 9630014M24 gene [Source:MGI Symbol%3BAcc:MGI:3588234]	ENSMUSG00000086655	C330018A13Rik	RIKEN cDNA C330018A13 gene [Source:MGI Symbol%3BAcc:MGI:2444781]	ENSMUSG00000058070	Eml1	echinoderm microtubule associated protein like 1 [Source:MGI Symbol%3BAcc:MGI:1915769]
ENSMUSG00000031698	Mylk3	myosin light chain kinase 3 [Source:MGI Symbol%3BAcc:MGI:2443063]	ENSMUSG00000103739	Gm37653	predicted gene%2C 37653 [Source:MGI Symbol%3BAcc:MGI:5610881]	ENSMUSG00000038668	Lpar1	lysophosphatidic acid receptor 1 [Source:MGI Symbol%3BAcc:MGI:108429]
ENSMUSG00000045319	Proser2	proline and serine rich 2 [Source:MGI Symbol%3BAcc:MGI:2442238]	ENSMUSG00000110713	Gm30606	predicted gene%2C 30606 [Source:MGI Symbol%3BAcc:MGI:5589765]	ENSMUSG00000040219	Ttc12	tetratricopeptide repeat domain 12 [Source:MGI Symbol%3BAcc:MGI:2444588]
ENSMUSG00000006369	Fbln1	fibulin 1 [Source:MGI Symbol%3BAcc:MGI:95487]	ENSMUSG00000099895	Gm28153	predicted gene 28153 [Source:MGI Symbol%3BAcc:MGI:5578859]	ENSMUSG00000046997	Spsb4	splA/ryanodine receptor domain and SOCS box containing 4 [Source:MGI Symbol%3BAcc:MGI:2183445]
ENSMUSG00000119584	Rn18s-rs5	18s RNA%2C related sequence 5 [Source:MGI Symbol%3BAcc:MGI:107222]	ENSMUSG00000025432	Avil	advillin [Source:MGI Symbol%3BAcc:MGI:1333798]	ENSMUSG00000050188	Lsm10	U7 snRNP-specific Sm-like protein LSM10 [Source:MGI Symbol%3BAcc:MGI:21510
ENSMUSG00000009378	Slc16a12	solute carrier family 16 (monocarboxylic acid transporters)%2C member 12 [Source:MGI Symbol%3BAcc:MGI:2147716]	ENSMUSG00000087698	Gm13031	predicted gene 13031 [Source:MGI Symbol%3BAcc:MGI:3702680]	ENSMUSG00000027104	Atf2	activating transcription factor 2 [Source:MGI Symbol%3BAcc:MGI:109349]
ENSMUSG00000055723	Rras2	related RAS viral (r-ras) oncogene 2 [Source:MGI Symbol%3BAcc:MGI:1914172]	ENSMUSG00000068014	Gm14648	predicted gene 14648 [Source:MGI Symbol%3BAcc:MGI:3705508]	ENSMUSG00000048706	Lurap1l	leucine rich adaptor protein 1-like [Source:MGI Symbol%3BAcc:MGI:106510]
ENSMUSG00000048416	Mlf1	myeloid leukemia factor 1 [Source:MGI Symbol%3BAcc:MGI:1341819]	ENSMUSG00000034683	Ppp1r1c	protein phosphatase 1%2C regulatory inhibitor subunit 1C [Source:MGI Symbol%3BAcc:MGI:1923185]	ENSMUSG00000040856	Dlk1	delta like non-canonical Notch ligand 1 [Source:MGI Symbol%3BAcc:MGI:94900]
ENSMUSG00000022602	Arc	activity regulated cytoskeletal-associated protein [Source:MGI Symbol%3BAcc:MGI:88067	ENSMUSG00000109616	Gm45393	predicted gene 45393 [Source:MGI Symbol%3BAcc:MGI:5791229]	ENSMUSG00000045165	AI467606	expressed sequence AI467606 [Source:MGI Symbol%3BAcc:MGI:2141979]
ENSMUSG00000078897	Gm4724	predicted gene 4724 [Source:MGI Symbol%3BAcc:MGI:3782904]	ENSMUSG00000020067	Mypn	myopalladin [Source:MGI Symbol%3BAcc:MGI:1916052]	ENSMUSG00000050919	Zfp366	zinc finger protein 366 [Source:MGI Symbol%3BAcc:MGI:2178429]
ENSMUSG00000022687	Boc	biregional cell adhesion molecule-related/down-regulated by oncogenes (Cdon) binding protein [Source:MGI Symbol%3BAcc:MGI:2151153]	ENSMUSG00000085611	Ap3s1-ps1	adaptor-related protein complex 3%2C sigma 1 subunit%2C pseudogene 1 [Source:MGI Symbol%3BAcc:MGI:1929216]	ENSMUSG00000047180	Neurl3	neuralized E3 ubiquitin protein ligase 3 [Source:MGI Symbol%3BAcc:MGI:2429944]
ENSMUSG00000024610	Cd74	CD74 antigen (invariant polypeptide of major histocompatibility complex%2C class II antigen-associated) [Source:MGI Symbol%3BAcc:MGI:96534	ENSMUSG00000006056	Calcoco2	calcium binding and coiled-coil domain 2 [Source:MGI Symbol%3BAcc:MGI:1343177]	ENSMUSG00000051048	P4ha3	procollagen-proline%2C 2-oxoglutarate 4-dioxygenase (proline 4-hydroxylase)%2C alpha polypeptide III [Source:MGI Symbol%3BAcc:MGI:24440
ENSMUSG00000031548	Sfrp1	secreted frizzled-related protein 1 [Source:MGI Symbol%3BAcc:MGI:892014]	ENSMUSG00000031289	Il13ra2	interleukin 13 receptor%2C alpha 2 [Source:MGI Symbol%3BAcc:MGI:1277954]	ENSMUSG00000043346	Gm6741	predicted gene 6741 [Source:MGI Symbol%3BAcc:MGI:3643504]
ENSMUSG00000006179	Prss16	protease%2C serine 16 (thymus) [Source:MGI Symbol%3BAcc:MGI:1859181]	ENSMUSG00000086509	Nkx2-2os	NK2 homeobox 2%2C opposite strand [Source:MGI Symbol%3BAcc:MGI:3652259]	ENSMUSG00000050549	Fam241a	family with sequence similarity 241%2C member A [Source:MGI Symbol%3BAcc:MGI:1917867]
ENSMUSG00000036103	Colec12	collectin sub-family member 12 [Source:MGI Symbol%3BAcc:MGI:2152907]	ENSMUSG00000110124	Gm4247	predicted gene 4247 [Source:MGI Symbol%3BAcc:MGI:3782424]	ENSMUSG00000036357	Gpr101	G protein-coupled receptor 101 [Source:MGI Symbol%3BAcc:MGI:2685211]
ENSMUSG00000060429	Sntb1	syntrophin%2C basic 1 [Source:MGI Symbol%3BAcc:MGI:10178	ENSMUSG00000021485	Mxd3	Max dimerization protein 3 [Source:MGI Symbol%3BAcc:MGI:10498	ENSMUSG00000047259	Mc4r	melanocortin 4 receptor [Source:MGI Symbol%3BAcc:MGI:99457
ENSMUSG00000026674	Ddr2	discoidin domain receptor family%2C member 2 [Source:MGI Symbol%3BAcc:MGI:1345277]	ENSMUSG00000006724	Cyp27b1	cytochrome P450%2C family 27%2C subfamily b%2C polypeptide 1 [Source:MGI Symbol%3BAcc:MGI:10982	ENSMUSG00000032854	Ugt8a	UDP galactosyltransferase 8A [Source:MGI Symbol%3BAcc:MGI:109522]
ENSMUSG00000033715	Akr1c14	aldo-keto reductase family 1%2C member C14 [Source:MGI Symbol%3BAcc:MGI:2145458]	ENSMUSG00000104214	Gm36638	predicted gene%2C 36638 [Source:MGI Symbol%3BAcc:MGI:5595797]	ENSMUSG00000050875	Minar2	membrane integral NOTCH2 associated receptor 2 [Source:MGI Symbol%3BAcc:MGI:2442934]

ENSMUSG00000039114	Nrn1	neuritin 1 [Source:MGI Symbol%3BAcc:MGI:1915654]	ENSMUSG00000113501	Gm18940	predicted gene%2C 18940 [Source:MGI Symbol%3BAcc:MGI:5011125]	ENSMUSG00000043008	Klhl6	kelch-like 6 [Source:MGI Symbol%3BAcc:MGI:2686922]
ENSMUSG00000028971	Cort	cortistatin [Source:MGI Symbol%3BAcc:MGI:109538]	ENSMUSG00000099784	Dalir	DNMT1 associated long intergenic non-coding RNA [Source:MGI Symbol%3BAcc:MGI:5579231]	ENSMUSG00000047557	Lxn	latexin [Source:MGI Symbol%3BAcc:MGI:107633]
ENSMUSG00000027931	Npr1	natriuretic peptide receptor 1 [Source:MGI Symbol%3BAcc:MGI:97371]	ENSMUSG00000039720	Got1l1	glutamic-oxaloacetic transaminase 1-like 1 [Source:MGI Symbol%3BAcc:MGI:1923865]	ENSMUSG00000044646	Zbtb7c	zinc finger and BTB domain containing 7C [Source:MGI Symbol%3BAcc:MGI:2443302]
ENSMUSG00000074825	Itpripl1	inositol 1%2C4%2C5-triphosphate receptor interacting protein-like 1 [Source:MGI Symbol%3BAcc:MGI:1920588]	ENSMUSG00000087166	L1td1	LINE-1 type transposase domain containing 1 [Source:MGI Symbol%3BAcc:MGI:3578435]	ENSMUSG00000040374	Pex2	peroxisomal biogenesis factor 2 [Source:MGI Symbol%3BAcc:MGI:107486]
ENSMUSG00000083325	Gm14121	predicted gene 14121 [Source:MGI Symbol%3BAcc:MGI:3651769]	ENSMUSG00000111077	Gm48743	predicted gene%2C 48743 [Source:MGI Symbol%3BAcc:MGI:6098412]	ENSMUSG00000057143	Trim12c	tripartite motif-containing 12C [Source:MGI Symbol%3BAcc:MGI:4821183]
ENSMUSG00000070564	Ntn5	netrin 5 [Source:MGI Symbol%3BAcc:MGI:2685330]	ENSMUSG00000098383	Gm27197	predicted gene 27197 [Source:MGI Symbol%3BAcc:MGI:5521040]	ENSMUSG00000028664	Ephb2	Eph receptor B2 [Source:MGI Symbol%3BAcc:MGI:99611]
ENSMUSG00000114469	C730002L08Rik	RIKEN cDNA C730002L08 gene [Source:MGI Symbol%3BAcc:MGI:2443493]	ENSMUSG00000065637	Gm26397	predicted gene%2C 26397 [Source:MGI Symbol%3BAcc:MGI:5456174]	ENSMUSG00000045731	Pnoc	prepronociceptin [Source:MGI Symbol%3BAcc:MGI:105308]
ENSMUSG00000019768	Esr1	estrogen receptor 1 (alpha) [Source:MGI Symbol%3BAcc:MGI:1352467]	ENSMUSG00000026770	Il2ra	interleukin 2 receptor%2C alpha chain [Source:MGI Symbol%3BAcc:MGI:96549]	ENSMUSG00000028876	Epha10	Eph receptor A10 [Source:MGI Symbol%3BAcc:MGI:3586824]
ENSMUSG00000050623	Catsperz	cation channel sperm associated auxiliary subunit zeta [Source:MGI Symbol%3BAcc:MGI:1914327]	ENSMUSG00000046589	Lrrc8e	leucine rich repeat containing 8 family%2C member E [Source:MGI Symbol%3BAcc:MGI:1919517]	ENSMUSG00000055430	Nap1l5	nucleosome assembly protein 1-like 5 [Source:MGI Symbol%3BAcc:MGI:1923555]
ENSMUSG00000034765	Dusp5	dual specificity phosphatase 5 [Source:MGI Symbol%3BAcc:MGI:2685183]	ENSMUSG00000081161	Gm12783	predicted gene 12783 [Source:MGI Symbol%3BAcc:MGI:3649566]	ENSMUSG00000025017	Pik3ap1	phosphoinositide-3-kinase adaptor protein 1 [Source:MGI Symbol%3BAcc:MGI:1933177]
ENSMUSG00000110447	Gm31518	predicted gene%2C 31518 [Source:MGI Symbol%3BAcc:MGI:5590677]	ENSMUSG00000104488	Gm38062	predicted gene%2C 38062 [Source:MGI Symbol%3BAcc:MGI:5611290]	ENSMUSG00000017754	Pltp	phospholipid transfer protein [Source:MGI Symbol%3BAcc:MGI:103151]
ENSMUSG00000066150	Slc31a1	solute carrier family 31%2C member 1 [Source:MGI Symbol%3BAcc:MGI:1333843]	ENSMUSG00000097111	Peak1os	pseudopodium-enriched atypical kinase 1%2C opposite strand [Source:MGI Symbol%3BAcc:MGI:54770	ENSMUSG00000058192	Zfp846	zinc finger protein 846 [Source:MGI Symbol%3BAcc:MGI:1924012]
ENSMUSG00002076173	-	-	ENSMUSG00000027416	Otor	otoraplin [Source:MGI Symbol%3BAcc:MGI:1888678]	ENSMUSG00000048779	P2ry6	pyrimidinergic receptor P2Y%2C G-protein coupled%2C 6 [Source:MGI Symbol%3BAcc:MGI:2673874]
ENSMUSG00000019851	Perp	PERP%2C TP53 apoptosis effector [Source:MGI Symbol%3BAcc:MGI:1929938]	ENSMUSG00000053687	Dpep2	dipeptidase 2 [Source:MGI Symbol%3BAcc:MGI:2442042]	ENSMUSG00000051504	Siglech	sialic acid binding Ig-like lectin H [Source:MGI Symbol%3BAcc:MGI:2443256]
ENSMUSG00000032006	Pdgfd	platelet-derived growth factor%2C D polypeptide [Source:MGI Symbol%3BAcc:MGI:1919035]	ENSMUSG00000104481	Gm38063	predicted gene%2C 38063 [Source:MGI Symbol%3BAcc:MGI:5611291]	ENSMUSG00000046287	Pnma3	paraneoplastic antigen MA3 [Source:MGI Symbol%3BAcc:MGI:2180565]
ENSMUSG00000030125	Lrrc23	leucine rich repeat containing 23 [Source:MGI Symbol%3BAcc:MGI:1315192]	ENSMUSG00000113589	Gm48374	predicted gene%2C 48374 [Source:MGI Symbol%3BAcc:MGI:6097849]	ENSMUSG00000051730	Mettl5	methyltransferase like 5 [Source:MGI Symbol%3BAcc:MGI:1922672]
ENSMUSG00000086555	Gm13446	predicted gene 13446 [Source:MGI Symbol%3BAcc:MGI:3649869]	ENSMUSG00000017868	Sgk2	serum/glucocorticoid regulated kinase 2 [Source:MGI Symbol%3BAcc:MGI:1351318]	ENSMUSG00000049891	Gm7984	predicted gene 7984 [Source:MGI Symbol%3BAcc:MGI:3645839]
ENSMUSG00000000440	Pparg	peroxisome proliferator activated receptor gamma [Source:MGI Symbol%3BAcc:MGI:97747]	ENSMUSG00000039063	Echdc3	enoyl Coenzyme A hydratase domain containing 3 [Source:MGI Symbol%3BAcc:MGI:1915106]	ENSMUSG00000044583	Tlr7	toll-like receptor 7 [Source:MGI Symbol%3BAcc:MGI:2176882]
ENSMUSG00000021708	Rasgrf2	RAS protein-specific guanine nucleotide-releasing factor 2 [Source:MGI Symbol%3BAcc:MGI:10913	ENSMUSG00000079048	4933413L06Rik	RIKEN cDNA 4933413L06 gene [Source:MGI Symbol%3BAcc:MGI:1918350]	ENSMUSG00000044080	S100a1	S100 calcium binding protein A1 [Source:MGI Symbol%3BAcc:MGI:1338917]
ENSMUSG00000042757	Tmem108	transmembrane protein 108 [Source:MGI Symbol%3BAcc:MGI:1932411]	ENSMUSG00000114424	Gm47414	predicted gene%2C 47414 [Source:MGI Symbol%3BAcc:MGI:6096350]	ENSMUSG00000036606	Plxnb2	plexin B2 [Source:MGI Symbol%3BAcc:MGI:2154239]
ENSMUSG00000020061	Mybpc1	myosin binding protein C%2C slow-type [Source:MGI Symbol%3BAcc:MGI:1336213]	ENSMUSG00000050050	Ccdc158	coiled-coil domain containing 158 [Source:MGI Symbol%3BAcc:MGI:2444555]	ENSMUSG00000050022	Amz1	archaelysin family metallopeptidase 1 [Source:MGI Symbol%3BAcc:MGI:2442258]
ENSMUSG00000041809	Efhc1	EF-hand domain (C-terminal) containing 1 [Source:MGI Symbol%3BAcc:MGI:1919127]	ENSMUSG00000117751	Gm50256	predicted gene%2C 50256 [Source:MGI Symbol%3BAcc:MGI:6303077]	ENSMUSG00000022665	Ccdc80	coiled-coil domain containing 80 [Source:MGI Symbol%3BAcc:MGI:1915146]
ENSMUSG00000044674	Fzd1	frizzled class receptor 1 [Source:MGI Symbol%3BAcc:MGI:1196625]	ENSMUSG00000002289	Angptl4	angiopoietin-like 4 [Source:MGI Symbol%3BAcc:MGI:1888999]	ENSMUSG00000017561	Crlf3	cytokine receptor-like factor 3 [Source:MGI Symbol%3BAcc:MGI:1860086]

ENSMUSG00000032224	Fam81a	family with sequence similarity 81%2C member A [Source:MGI Symbol%3BAcc:MGI:1924136]	ENSMUSG00000113866	Gm47450	predicted gene%2C 47450 [Source:MGI Symbol%3BAcc:MGI:6096407]	ENSMUSG00000043388	Tmem130	transmembrane protein 130 [Source:MGI Symbol%3BAcc:MGI:3607706]
ENSMUSG00000037206	Islr	immunoglobulin superfamily containing leucine-rich repeat [Source:MGI Symbol%3BAcc:MGI:13496	ENSMUSG00000029082	Bst1	bone marrow stromal cell antigen 1 [Source:MGI Symbol%3BAcc:MGI:105370]	ENSMUSG00000044468	Tent5c	terminal nucleotidyltransferase 5C [Source:MGI Symbol%3BAcc:MGI:1921895]
ENSMUSG00000032796	Lama1	laminin%2C alpha 1 [Source:MGI Symbol%3BAcc:MGI:99892]	ENSMUSG00002075945	5S_rRNA	5S ribosomal RNA [Source:RFAM%3BAcc:RF00001]	ENSMUSG00000050505	Pcdh20	protocadherin 20 [Source:MGI Symbol%3BAcc:MGI:2443376]
ENSMUSG00000047821	Trim16	tripartite motif-containing 16 [Source:MGI Symbol%3BAcc:MGI:2137356]	ENSMUSG00000047109	Cldn14	claudin 14 [Source:MGI Symbol%3BAcc:MGI:1860425]	ENSMUSG00000045246	Kcng4	potassium voltage-gated channel%2C subfamily G%2C member 4 [Source:MGI Symbol%3BAcc:MGI:19139
ENSMUSG00000029337	Fgf5	fibroblast growth factor 5 [Source:MGI Symbol%3BAcc:MGI:95519]	ENSMUSG00000109015	Gm31024	predicted gene%2C 31024 [Source:MGI Symbol%3BAcc:MGI:5590183]	ENSMUSG00000051439	Cd14	CD14 antigen [Source:MGI Symbol%3BAcc:MGI:88318]
ENSMUSG00000030796	Tead2	TEA domain family member 2 [Source:MGI Symbol%3BAcc:MGI:104904]	ENSMUSG00000103272	Gm37914	predicted gene%2C 37914 [Source:MGI Symbol%3BAcc:MGI:5611142]	ENSMUSG00000049148	Plcxd3	phosphatidylinositol-specific phospholipase C%2C X domain containing 3 [Source:MGI Symbol%3BAcc:MGI:2442605]
ENSMUSG00000049791	Fzd4	frizzled class receptor 4 [Source:MGI Symbol%3BAcc:MGI:108520]	ENSMUSG00000086740	Gm17029	predicted gene 17029 [Source:MGI Symbol%3BAcc:MGI:4937856]	ENSMUSG00000047419	Cmya5	cardiomyopathy associated 5 [Source:MGI Symbol%3BAcc:MGI:1923719]
ENSMUSG00000024033	Rsph1	radial spoke head 1 homolog (Chlamydomonas) [Source:MGI Symbol%3BAcc:MGI:11949	ENSMUSG00000098108	Gm27008	predicted gene%2C 27008 [Source:MGI Symbol%3BAcc:MGI:5504123]	ENSMUSG00000043969	Emx2	empty spiracles homeobox 2 [Source:MGI Symbol%3BAcc:MGI:95388]
ENSMUSG00000024647	Cbln2	cerebellin 2 precursor protein [Source:MGI Symbol%3BAcc:MGI:88282]	ENSMUSG00000117731	Gm50145	predicted gene%2C 50145 [Source:MGI Symbol%3BAcc:MGI:6302898]	ENSMUSG00000045322	Tlr9	toll-like receptor 9 [Source:MGI Symbol%3BAcc:MGI:1932389]
ENSMUSG00000097596	Gm26673	predicted gene%2C 26673 [Source:MGI Symbol%3BAcc:MGI:5477167]	ENSMUSG00000027863	Cd2	CD2 antigen [Source:MGI Symbol%3BAcc:MGI:88320]	ENSMUSG00000044350	Lacc1	laccase domain containing 1 [Source:MGI Symbol%3BAcc:MGI:2445077]
ENSMUSG00000025813	Homer2	homer scaffolding protein 2 [Source:MGI Symbol%3BAcc:MGI:1347354]	ENSMUSG00000119087	Gm25311	predicted gene%2C 25311 [Source:MGI Symbol%3BAcc:MGI:5455088]	ENSMUSG00000019843	Fyn	Fyn proto-oncogene [Source:MGI Symbol%3BAcc:MGI:95602]
ENSMUSG00000022150	Dab2	disabled 2%2C mitogen-responsive phosphoprotein [Source:MGI	ENSMUSG00000040564	Apoc1	apolipoprotein C-I [Source:MGI Symbol%3BAcc:MGI:88053]	ENSMUSG00000032360	Hcrtr2	hypocretin (orexin) receptor 2 [Source:MGI Symbol%3BAcc:MGI:2680765]
ENSMUSG00000027524	Edn3	endothelin 3 [Source:MGI Symbol%3BAcc:MGI:95285]	ENSMUSG00000116796	Gm49784	predicted gene%2C 49784 [Source:MGI Symbol%3BAcc:MGI:6215305]	ENSMUSG00000020151	Ptprr	protein tyrosine phosphatase%2C receptor type%2C R [Source:MGI Symbol%3BAcc:MGI:10955
ENSMUSG00000048572	Tmem252	transmembrane protein 252 [Source:MGI Symbol%3BAcc:MGI:3583948]	ENSMUSG00000120568	-	novel transcript	ENSMUSG00000052459	Atp6v1a	ATPase%2C H+ transporting%2C lysosomal V1 subunit A [Source:MGI Symbol%3BAcc:MGI:1201780]
ENSMUSG00000021567	Nkd2	naked cuticle 2 [Source:MGI Symbol%3BAcc:MGI:1919543]	ENSMUSG00000086997	Gm12981	predicted gene 12981 [Source:MGI Symbol%3BAcc:MGI:3651528]	ENSMUSG00000052160	Pld4	phospholipase D family%2C member 4 [Source:MGI Symbol%3BAcc:MGI:21447
ENSMUSG00000053965	Pde5a	phosphodiesterase 5A%2C cGMP-specific [Source:MGI Symbol%3BAcc:MGI:26514	ENSMUSG00000117478	BC051408	cDNA sequence BC051408 [Source:MGI Symbol%3BAcc:MGI:3040685]	ENSMUSG00000052229	Gpr17	G protein-coupled receptor 17 [Source:MGI Symbol%3BAcc:MGI:3584514]
ENSMUSG00000029307	Dmp1	dentin matrix protein 1 [Source:MGI Symbol%3BAcc:MGI:94910]	ENSMUSG00000120582	-	novel transcript	ENSMUSG00000028555	Ttc39a	tetratricopeptide repeat domain 39A [Source:MGI Symbol%3BAcc:MGI:2444350]
ENSMUSG00000035357	Pdzrn3	PDZ domain containing RING finger 3 [Source:MGI Symbol%3BAcc:MGI:1933157]	ENSMUSG00002076474	7SK	7SK RNA [Source:RFAM%3BAcc:RF00100]	ENSMUSG00000052336	Cx3cr1	chemokine (C-X3-C motif) receptor 1 [Source:MGI Symbol%3BAcc:MGI:1333815]
ENSMUSG00000074227	Spint2	serine protease inhibitor%2C Kunitz type 2 [Source:MGI Symbol%3BAcc:MGI:13380	ENSMUSG00000045036	Tmem232	transmembrane protein 232 [Source:MGI Symbol%3BAcc:MGI:2685786]	ENSMUSG00000052364	B630019K06Rik	RIKEN cDNA B630019K06 gene [Source:MGI Symbol%3BAcc:MGI:2147918]
ENSMUSG00000030340	Scnn1a	sodium channel%2C nonvoltage-gated 1 alpha [Source:MGI Symbol%3BAcc:MGI:101782]	ENSMUSG00000081683	Fzd10	frizzled class receptor 10 [Source:MGI Symbol%3BAcc:MGI:2136761]	ENSMUSG00000079055	Slc8a3	solute carrier family 8 (sodium/calcium exchanger)%2C member 3 [Source:MGI Symbol%3BAcc:MGI:10797
ENSMUSG00000021806	Nid2	nidogen 2 [Source:MGI Symbol%3BAcc:MGI:1298229]	ENSMUSG00000107336	Gm43461	predicted gene 43461 [Source:MGI Symbol%3BAcc:MGI:5663598]	ENSMUSG00000040591	1110051M20Rik	RIKEN cDNA 1110051M20 gene [Source:MGI Symbol%3BAcc:MGI:1915079]
ENSMUSG00000040998	Npnt	nephronectin [Source:MGI Symbol%3BAcc:MGI:2148811]	ENSMUSG00000091561	Gm6665	predicted gene 6665 [Source:MGI Symbol%3BAcc:MGI:3643253]	ENSMUSG00000031340	Gabre	gamma-aminobutyric acid (GABA) A receptor%2C subunit epsilon [Source:MGI Symbol%3BAcc:MGI:13302
ENSMUSG00000025795	Rassf3	Ras association (RalGDS/AF-6) domain family member 3 [Source:MGI Symbol%3BAcc:MGI:2179722]	ENSMUSG00000117860	Gm30018	predicted gene%2C 30018 [Source:MGI Symbol%3BAcc:MGI:5589177]	ENSMUSG00000053470	Kdm3a	lysine (K)-specific demethylase 3A [Source:MGI Symbol%3BAcc:MGI:98847]

ENSMUSG00000097610	A930012L18Rik	RIKEN cDNA A930012L18 gene [Source:MGI Symbol%3BAcc:MGI:2442887]	ENSMUSG00000103959	Gm37720	predicted gene%2C 37720 [Source:MGI Symbol%3BAcc:MGI:5610948]	ENSMUSG00000049265	Kcnk3	potassium channel%2C subfamily K%2C member 3 [Source:MGI Symbol%3BAcc:MGI:1100509]
ENSMUSG00000046761	Fam83h	family with sequence similarity 83%2C member H [Source:MGI Symbol%3BAcc:MGI:2145900]	ENSMUSG00000109956	Gm45428	predicted gene 45428 [Source:MGI Symbol%3BAcc:MGI:5791264]	ENSMUSG00000024968	Rcor2	REST corepressor 2 [Source:MGI Symbol%3BAcc:MGI:1859854]
ENSMUSG00000092083	Kcnb2	potassium voltage gated channel%2C Shab-related subfamily%2C member 2 [Source:MGI Symbol%3BAcc:MGI:99632	ENSMUSG00000120259	-	novel transcript	ENSMUSG00000026824	Kcnj3	potassium inwardly-rectifying channel%2C subfamily J%2C member 3 [Source:MGI Symbol%3BAcc:MGI:10474
ENSMUSG00000060591	Ifitm2	interferon induced transmembrane protein 2 [Source:MGI Symbol%3BAcc:MGI:1933382]	ENSMUSG00000104556	Gm43192	predicted gene 43192 [Source:MGI Symbol%3BAcc:MGI:5663329]	ENSMUSG00000020932	Gfap	glial fibrillary acidic protein [Source:MGI Symbol%3BAcc:MGI:95697]
ENSMUSG00000032265	Tent5a	terminal nucleotidyltransferase 5A [Source:MGI Symbol%3BAcc:MGI:2670964]	ENSMUSG00000027115	Kif18a	kinesin family member 18A [Source:MGI Symbol%3BAcc:MGI:2446977]	ENSMUSG00000054517	Trim65	tripartite motif-containing 65 [Source:MGI Symbol%3BAcc:MGI:2442815]
ENSMUSG00000039246	Lyplal1	lysophospholipase-like 1 [Source:MGI Symbol%3BAcc:MGI:2385115]	ENSMUSG00000111509	Gm47266	predicted gene%2C 47266 [Source:MGI Symbol%3BAcc:MGI:6096101]	ENSMUSG00000054555	Adam12	a disintegrin and metallopeptidase domain 12 (meltrin alpha) [Source:MGI
ENSMUSG00000021025	Nfkbia	nuclear factor of kappa light polypeptide gene enhancer in B cells inhibitor%2C alpha [Source:MGI	ENSMUSG00000090005	Gm16540	predicted gene 16540 [Source:MGI Symbol%3BAcc:MGI:4414960]	ENSMUSG00000054667	Irs4	insulin receptor substrate 4 [Source:MGI Symbol%3BAcc:MGI:1338009]
ENSMUSG00000005124	Ccn4	cellular communication network factor 4 [Source:MGI Symbol%3BAcc:MGI:1197008]				ENSMUSG00000054717	Hmgb2	high mobility group box 2 [Source:MGI Symbol%3BAcc:MGI:96157]
ENSMUSG00000043668	Tox3	TOX high mobility group box family member 3 [Source:MGI Symbol%3BAcc:MGI:3039593]				ENSMUSG00000020914	Top2a	topoisomerase (DNA) II alpha [Source:MGI Symbol%3BAcc:MGI:98790]
ENSMUSG00000097326	A330048O09Rik	RIKEN cDNA A330048O09 gene [Source:MGI Symbol%3BAcc:MGI:2443792]				ENSMUSG00000050953	Gja1	gap junction protein%2C alpha 1 [Source:MGI Symbol%3BAcc:MGI:95713]
ENSMUSG00000042256	Ptchd4	patched domain containing 4 [Source:MGI Symbol%3BAcc:MGI:1920485]				ENSMUSG00000055184	Fam72a	family with sequence similarity 72%2C member A [Source:MGI Symbol%3BAcc:MGI:1919669]
ENSMUSG00000039813	Tbc1d2	TBC1 domain family%2C member 2 [Source:MGI Symbol%3BAcc:MGI:2652885]				ENSMUSG00000066357	Wdr6	WD repeat domain 6 [Source:MGI Symbol%3BAcc:MGI:1930140]
ENSMUSG00000068744	Psrc1	proline/serine-rich coiled-coil 1 [Source:MGI Symbol%3BAcc:MGI:1913099]				ENSMUSG00000036466	Megf11	multiple EGF-like-domains 11 [Source:MGI Symbol%3BAcc:MGI:1920951]
ENSMUSG00000043439	Epop	elongin BC and polycomb repressive complex 2 associated protein [Source:MGI Symbol%3BAcc:MGI:2143991]				ENSMUSG00000055407	Map6	microtubule-associated protein 6 [Source:MGI Symbol%3BAcc:MGI:1201690]
ENSMUSG00000031303	Map3k15	mitogen-activated protein kinase kinase kinase 15 [Source:MGI Symbol%3BAcc:MGI:2448588]				ENSMUSG00000068457	Uty	ubiquitously transcribed tetratricopeptide repeat containing%2C Y-linked [Source:MGI Symbol%3BAcc:MGI:89481
ENSMUSG00000098066	Gm26944	predicted gene%2C 26944 [Source:MGI Symbol%3BAcc:MGI:5504059]				ENSMUSG00000055653	Gpc3	glypican 3 [Source:MGI Symbol%3BAcc:MGI:104903]
ENSMUSG00000040701	Ap1g2	adaptor protein complex AP-1%2C gamma 2 subunit [Source:MGI Symbol%3BAcc:MGI:1328307]				ENSMUSG00000056131	Pgm3	phosphoglucomutase 3 [Source:MGI Symbol%3BAcc:MGI:97566]
ENSMUSG00000074796	Slc4a11	solute carrier family 4%2C sodium bicarbonate transporter-like%2C member 11 [Source:MGI Symbol%3BAcc:MGI:2138987]				ENSMUSG00000056204	Pgpep1	pyroglutamyl-peptidase I [Source:MGI Symbol%3BAcc:MGI:1913772]
ENSMUSG00000026787	Gad2	glutamic acid decarboxylase 2 [Source:MGI Symbol%3BAcc:MGI:95634]				ENSMUSG00000056306	Sertm1	serine rich and transmembrane domain containing 1 [Source:MGI Symbol%3BAcc:MGI:3607715]
ENSMUSG00000110945	Gm9856	predicted gene 9856 [Source:MGI Symbol%3BAcc:MGI:3704347]				ENSMUSG00000029860	Zyx	zyxin [Source:MGI Symbol%3BAcc:MGI:103072]
ENSMUSG00000047534	Mis18bp1	MIS18 binding protein 1 [Source:MGI Symbol%3BAcc:MGI:2145099]				ENSMUSG00000056529	Ptafr	platelet-activating factor receptor [Source:MGI Symbol%3BAcc:MGI:106066]
ENSMUSG00000051111	Sv2c	synaptic vesicle glycoprotein 2c [Source:MGI Symbol%3BAcc:MGI:19224				ENSMUSG00000029778	Adcyap1r1	adenylate cyclase activating polypeptide 1 receptor 1 [Source:MGI Symbol%3BAcc:MGI:10844

ENSMUSG00000020121	Srgap1	SLIT-ROBO Rho GTPase activating protein 1 [Source:MGI Symbol%3BAcc:MGI:2152936]
ENSMUSG00000110702	Gm45767	predicted gene 45767 [Source:MGI Symbol%3BAcc:MGI:5804882]
ENSMUSG00000115222	Gm49747	predicted gene%2C 49747 [Source:MGI Symbol%3BAcc:MGI:6215240]
ENSMUSG00000046056	Sbsn	suprabasin [Source:MGI Symbol%3BAcc:MGI:2446326]
ENSMUSG00000021263	Degs2	delta(4)-desaturase%2C sphingolipid 2 [Source:MGI Symbol%3BAcc:MGI:1917309]
ENSMUSG00000058260	Serpina9	serine (or cysteine) peptidase inhibitor%2C clade A (alpha-1 antiproteinase%2C antitrypsin)%2C member 9 [Source:MGI Symbol%3BAcc:MGI:1919157]
ENSMUSG00000021108	Prkch	protein kinase C%2C eta [Source:MGI Symbol%3BAcc:MGI:976001]
ENSMUSG00000049538	Adamts16	a disintegrin-like and metallopeptidase (reprolysin type) with thrombospondin type 1 motif%2C 16 [Source:MGI Symbol%3BAcc:MGI:2429637]
ENSMUSG00000097961	Gm27000	predicted gene%2C 27000 [Source:MGI Symbol%3BAcc:MGI:5504115]
ENSMUSG00000105736	Gm42456	predicted gene 42456 [Source:MGI Symbol%3BAcc:MGI:5662593]
ENSMUSG00000105218	Gm38413	predicted gene%2C 38413 [Source:MGI Symbol%3BAcc:MGI:5621298]
ENSMUSG00000091345	Col6a5	collagen%2C type VI%2C alpha 5 [Source:MGI Symbol%3BAcc:MGI:3648134]
ENSMUSG00000104157	Gm38101	predicted gene%2C 38101 [Source:MGI Symbol%3BAcc:MGI:5611329]
ENSMUSG00000066510	Ankdd1a	ankyrin repeat and death domain containing 1A [Source:MGI Symbol%3BAcc:MGI:2686319]
ENSMUSG00000079293	Clec7a	C-type lectin domain family 7%2C member a [Source:MGI Symbol%3BAcc:MGI:1861431]
ENSMUSG00000070687	Htr1d	5-hydroxytryptamine (serotonin) receptor 1D [Source:MGI Symbol%3BAcc:MGI:962761]
ENSMUSG00000089707	Slain1os	SLAIN motif family%2C member 1%2C opposite strand [Source:MGI Symbol%3BAcc:MGI:1918148]
ENSMUSG00000044749	Abca6	ATP-binding cassette%2C sub-family A (ABC1)%2C member 6 [Source:MGI Symbol%3BAcc:MGI:1923434]
ENSMUSG00000109849	Gm45645	predicted gene 45645 [Source:MGI Symbol%3BAcc:MGI:5791481]
ENSMUSG00000089832	Shkbp1	Sh3kbp1 binding protein 1 [Source:MGI Symbol%3BAcc:MGI:2385803]
ENSMUSG00000104494	Gm37111	predicted gene%2C 37111 [Source:MGI Symbol%3BAcc:MGI:5610339]

ENSMUSG00000022235	Cmbl	carboxymethylenebutenolidase-like (Pseudomonas) [Source:MGI Symbol%3BAcc:MGI:1916824]
ENSMUSG00000056737	Capg	capping protein (actin filament)%2C gelsolin-like [Source:MGI Symbol%3BAcc:MGI:1098259]
ENSMUSG00000032890	Rims3	regulating synaptic membrane exocytosis 3 [Source:MGI Symbol%3BAcc:MGI:2443331]
ENSMUSG00000060063	Alox5ap	arachidonate 5-lipoxygenase activating protein [Source:MGI Symbol%3BAcc:MGI:10750
ENSMUSG00000029376	Mthfd2l	methylenetetrahydrofolate dehydrogenase (NADP+ dependent) 2-like [Source:MGI Symbol%3BAcc:MGI:1915871]
ENSMUSG00000060073	Psma3	proteasome subunit alpha 3 [Source:MGI Symbol%3BAcc:MGI:104883]
ENSMUSG00000071379	Hpcal1	hippocalcin-like 1 [Source:MGI Symbol%3BAcc:MGI:1855689]
ENSMUSG00000063362	Alg11	asparagine-linked glycosylation 11 (alpha-1%2C2-mannosyltransferase) [Source:MGI Symbol%3BAcc:MGI:2142632]
ENSMUSG00000057359	Gm17494	predicted gene%2C 17494 [Source:MGI Symbol%3BAcc:MGI:4937128]
ENSMUSG00000015149	Sirt2	sirtuin 2 [Source:MGI Symbol%3BAcc:MGI:1927664]
ENSMUSG00000060019	Gm10073	predicted pseudogene 10073 [Source:MGI Symbol%3BAcc:MGI:3641908]
ENSMUSG00000037465	Klf10	Kruppel-like factor 10 [Source:MGI Symbol%3BAcc:MGI:1101353]
ENSMUSG00000022212	Cpne6	copine VI [Source:MGI Symbol%3BAcc:MGI:1334445]
ENSMUSG00000059751	Rps3a3	ribosomal protein S3A3 [Source:MGI Symbol%3BAcc:MGI:3643406]
ENSMUSG00000061724	Gm2423	predicted gene 2423 [Source:MGI Symbol%3BAcc:MGI:3805957]
ENSMUSG00000059734	Ndufs8	NADH:ubiquinone oxidoreductase core subunit S8 [Source:MGI Symbol%3BAcc:MGI:2385079]
ENSMUSG00000040540	Ctdsp2-ps	CTD (carboxy-terminal domain%2C RNA polymerase II%2C polypeptide A) small phosphatase 2%2C pseudogene [Source:MGI Symbol%3BAcc:MGI:3708545]
ENSMUSG00000058153	Sez6l	seizure related 6 homolog like [Source:MGI Symbol%3BAcc:MGI:1935121]
ENSMUSG00000034437	Gm9761	predicted gene 9761 [Source:MGI Symbol%3BAcc:MGI:3708640]
ENSMUSG00000094446	Gm6344	predicted gene 6344 [Source:MGI Symbol%3BAcc:MGI:3779586]
ENSMUSG00000020895	Tmem107	transmembrane protein 107 [Source:MGI Symbol%3BAcc:MGI:1914160]

ENSMUSG00000028681	Ptch2	patched 2 [Source:MGI Symbol%3BAcc:MGI:1095405]
ENSMUSG00000107320	Gm42549	predicted gene 42549 [Source:MGI Symbol%3BAcc:MGI:5662686]
ENSMUSG00000109792	Gm45426	predicted gene 45426 [Source:MGI Symbol%3BAcc:MGI:5791262]
ENSMUSG00000020599	Rgs9	regulator of G-protein signaling 9 [Source:MGI Symbol%3BAcc:MGI:1338824]
ENSMUSG00000026639	Lamb3	laminin%2C beta 3 [Source:MGI Symbol%3BAcc:MGI:99915]
ENSMUSG00000050473	Slc35d3	solute carrier family 35%2C member D3 [Source:MGI Symbol%3BAcc:MGI:1923407]
ENSMUSG00000008028	1700008O03Rik	RIKEN cDNA 1700008O03 gene [Source:MGI Symbol%3BAcc:MGI:1916599]
ENSMUSG00000017491	Rarb	retinoic acid receptor%2C beta [Source:MGI Symbol%3BAcc:MGI:97857]
ENSMUSG00000097746	Gm6225	predicted gene 6225 [Source:MGI Symbol%3BAcc:MGI:3643252]
ENSMUSG00000037994	Slc9b2	solute carrier family 9%2C subfamily B (NHA2%2C cation proton antiporter 2)%2C member 2 [Source:MGI Symbol%3BAcc:MGI:2140077]
ENSMUSG00000097268	Gm26805	predicted gene%2C 26805 [Source:MGI Symbol%3BAcc:MGI:5477299]
ENSMUSG00000049357	Brd8dc	BRD8 domain containing [Source:MGI Symbol%3BAcc:MGI:3045347]
ENSMUSG00000115360	Gm48932	predicted gene%2C 48932 [Source:MGI Symbol%3BAcc:MGI:6118246]
ENSMUSG00000097835	Gm26910	predicted gene%2C 26910 [Source:MGI Symbol%3BAcc:MGI:5477404]
ENSMUSG00000097466	D430036J16Rik	RIKEN cDNA D430036J16 gene [Source:MGI Symbol%3BAcc:MGI:2441977]
ENSMUSG00000030775	Trat1	T cell receptor associated transmembrane adaptor 1 [Source:MGI Symbol%3BAcc:MGI:1924897]
ENSMUSG00000111752	Gm38575	predicted gene%2C 38575 [Source:MGI Symbol%3BAcc:MGI:5621460]
ENSMUSG00000085913	Gm15601	predicted gene 15601 [Source:MGI Symbol%3BAcc:MGI:3783048]
ENSMUSG00000097547	B230110C06Rik	RIKEN cDNA B230110C06 gene [Source:MGI Symbol%3BAcc:MGI:1925231]
ENSMUSG00000027966	Col11a1	collagen%2C type XI%2C alpha 1 [Source:MGI Symbol%3BAcc:MGI:88446]
ENSMUSG00000100937	Nscme3l	NSE3 homolog%2C SMC5-SMC6 complex component like [Source:MGI Symbol%3BAcc:MGI:19228
ENSMUSG00000112343	Sfta3-ps	surfactant associated 3%2C pseudogene [Source:MGI Symbol%3BAcc:MGI:2144804]
ENSMUSG00000022483	Col2a1	collagen%2C type II%2C alpha 1 [Source:MGI Symbol%3BAcc:MGI:88452]
ENSMUSG00000116766	Gm49702	predicted gene%2C 49702 [Source:MGI Symbol%3BAcc:MGI:6215162]
ENSMUSG00000062488	Ifit3b	interferon-induced protein with tetratricopeptide repeats 3B [Source:MGI Symbol%3BAcc:MGI:36984
ENSMUSG00000022847	Thpo	thrombopoietin [Source:MGI Symbol%3BAcc:MGI:101875]
ENSMUSG00000063328	Gm9396	predicted gene 9396 [Source:MGI Symbol%3BAcc:MGI:3645563]
ENSMUSG00000059775	Rps26-ps1	ribosomal protein S26%2C pseudogene 1 [Source:MGI Symbol%3BAcc:MGI:37043
ENSMUSG00000058050	Gm9234	predicted pseudogene 9234 [Source:MGI Symbol%3BAcc:MGI:3648545]
ENSMUSG00000064208	Gm10145	predicted gene 10145 [Source:MGI Symbol%3BAcc:MGI:3704353]
ENSMUSG00000062456	Rpl9-ps6	ribosomal protein L9%2C pseudogene 6 [Source:MGI Symbol%3BAcc:MGI:36426
ENSMUSG00000058586	Serhl	serine hydrolase-like [Source:MGI Symbol%3BAcc:MGI:1890404]
ENSMUSG00000026825	Dnm1	dynamin 1 [Source:MGI Symbol%3BAcc:MGI:107384]
ENSMUSG00000037784	Dzip1l	DAZ interacting protein 1-like [Source:MGI Symbol%3BAcc:MGI:1919757]
ENSMUSG00000058248	Kcnh1	potassium voltage-gated channel%2C subfamily H (eag-related)%2C member 1 [Source:MGI Symbol%3BAcc:MGI:1341721]
ENSMUSG00000034009	Rxfp1	relaxin/insulin-like family peptide receptor 1 [Source:MGI Symbol%3BAcc:MGI:2682211]
ENSMUSG00000064220	#N/A	#N/A
ENSMUSG00000060791	Gmfg	glia maturation factor%2C gamma [Source:MGI Symbol%3BAcc:MGI:1927135]
ENSMUSG00000024411	Aqp4	aquaporin 4 [Source:MGI Symbol%3BAcc:MGI:107387]
ENSMUSG00000037686	Aspg	asparaginase [Source:MGI Symbol%3BAcc:MGI:2144822]
ENSMUSG00000062031	Pgghg	protein glucosylgalactosylhydroxylysine glucosidase [Source:MGI Symbol%3BAcc:MGI:24440
ENSMUSG00000060519	Tor3a	torsin family 3%2C member A [Source:MGI Symbol%3BAcc:MGI:1353652]
ENSMUSG00000063234	Gpr84	G protein-coupled receptor 84 [Source:MGI Symbol%3BAcc:MGI:1934129]
ENSMUSG00000058715	Fcer1g	Fc receptor%2C IgE%2C high affinity I%2C gamma polypeptide [Source:MGI Symbol%3BAcc:MGI:95496
ENSMUSG00000028962	Slc4a2	solute carrier family 4 (anion exchanger)%2C member 2 [Source:MGI Symbol%3BAcc:MGI:109351]
ENSMUSG00000009927	Rps25	ribosomal protein S25 [Source:MGI Symbol%3BAcc:MGI:1922867]
ENSMUSG00000056972	Magel2	MAGE family member L2 [Source:MGI Symbol%3BAcc:MGI:1351648]
ENSMUSG00000039952	Dag1	dystroglycan 1 [Source:MGI Symbol%3BAcc:MGI:101864]

ENSMUSG00000086245	Gm16170	predicted gene 16170 [Source:MGI Symbol%3BAcc:MGI:3805548]
ENSMUSG00000049608	Gpr55	G protein-coupled receptor 55 [Source:MGI Symbol%3BAcc:MGI:2685064]
ENSMUSG00000057280	Musk	muscle%2C skeletal%2C receptor tyrosine kinase [Source:MGI Symbol%3BAcc:MGI:10358
ENSMUSG00000043635	Adamts3	a disintegrin-like and metallopeptidase (reprolysin type) with thrombospondin type 1 motif%2C 3 [Source:MGI Symbol%3BAcc:MGI:3045353]
ENSMUSG00000104277	Gm38299	predicted gene%2C 38299 [Source:MGI Symbol%3BAcc:MGI:5611527]
ENSMUSG00000028072	Ntrk1	neurotrophic tyrosine kinase%2C receptor%2C type 1 [Source:MGI Symbol%3BAcc:MGI:97383
ENSMUSG00000014782	Plekhg4	pleckstrin homology domain containing%2C family G (with RhoGef domain) member 4 [Source:MGI Symbol%3BAcc:MGI:21425
ENSMUSG00000104988	Gm43622	predicted gene 43622 [Source:MGI Symbol%3BAcc:MGI:5663759]
ENSMUSG00000000555	Itga5	integrin alpha 5 (fibronectin receptor alpha) [Source:MGI
ENSMUSG00000120002	-	novel transcript
ENSMUSG00000104939	Gm33651	predicted gene%2C 33651 [Source:MGI Symbol%3BAcc:MGI:5592810]
ENSMUSG00000103329	Gm42492	predicted gene 42492 [Source:MGI Symbol%3BAcc:MGI:5662629]
ENSMUSG00000012123	Crybg2	crystallin beta-gamma domain containing 2 [Source:MGI Symbol%3BAcc:MGI:1334463]
ENSMUSG00000108802	Gm44769	predicted gene 44769 [Source:MGI Symbol%3BAcc:MGI:5753345]
ENSMUSG00000087611	4930458D05Rik	RIKEN cDNA 4930458D05 gene [Source:MGI Symbol%3BAcc:MGI:1923052]
ENSMUSG00000004347	Pde1c	phosphodiesterase 1C [Source:MGI Symbol%3BAcc:MGI:108413]
ENSMUSG00000032946	Rasgrp2	RAS%2C guanyl releasing protein 2 [Source:MGI Symbol%3BAcc:MGI:1333849]
ENSMUSG00000112163	Gm8188	predicted gene 8188 [Source:MGI Symbol%3BAcc:MGI:3646272]
ENSMUSG00000028459	Cd72	CD72 antigen [Source:MGI Symbol%3BAcc:MGI:88345]
ENSMUSG00000107560	Gm44199	predicted gene%2C 44199 [Source:MGI Symbol%3BAcc:MGI:5690591]
ENSMUSG00000116624	Gm49706	predicted gene%2C 49706 [Source:MGI Symbol%3BAcc:MGI:6215169]
ENSMUSG00000039328	Rnf122	ring finger protein 122 [Source:MGI Symbol%3BAcc:MGI:1916117]
ENSMUSG00000028885	Smpdl3b	sphingomyelin phosphodiesterase%2C acid-like 3B [Source:MGI Symbol%3BAcc:MGI:1916022]
ENSMUSG00000094840	Muc3a	mucin 3A%2C cell surface associated [Source:MGI Symbol%3BAcc:MGI:3588263]
ENSMUSG00000057657	Rps18-ps3	ribosomal protein S18%2C pseudogene 3 [Source:MGI Symbol%3BAcc:MGI:36424
ENSMUSG00000062760	Shisal1	shisa like 1 [Source:MGI Symbol%3BAcc:MGI:1919551]
ENSMUSG00000053158	Fes	feline sarcoma oncogene [Source:MGI Symbol%3BAcc:MGI:95514]
ENSMUSG00000046805	Mpeg1	macrophage expressed gene 1 [Source:MGI Symbol%3BAcc:MGI:1333743]
ENSMUSG00000010505	Myt1	myelin transcription factor 1 [Source:MGI Symbol%3BAcc:MGI:1100535]
ENSMUSG00000034330	Plcg2	phospholipase C%2C gamma 2 [Source:MGI Symbol%3BAcc:MGI:97616]
ENSMUSG00000067629	Syngap1	synaptic Ras GTPase activating protein 1 homolog (rat) [Source:MGI Symbol%3BAcc:MGI:3039785]
ENSMUSG00000030528	Blm	Bloom syndrome%2C RecQ like helicase [Source:MGI Symbol%3BAcc:MGI:13283
ENSMUSG00000035929	H2-Q4	histocompatibility 2%2C Q region locus 4 [Source:MGI
ENSMUSG00000060512	0610040J01Rik	RIKEN cDNA 0610040J01 gene [Source:MGI Symbol%3BAcc:MGI:1923511]
ENSMUSG00000064246	Chil1	chitinase-like 1 [Source:MGI Symbol%3BAcc:MGI:1340899]
ENSMUSG00000018339	Gpx3	glutathione peroxidase 3 [Source:MGI Symbol%3BAcc:MGI:105102]
ENSMUSG00000068466	Gm5518	predicted gene 5518 [Source:MGI Symbol%3BAcc:MGI:3648673]
ENSMUSG00000010021	Kif19a	kinesin family member 19A [Source:MGI Symbol%3BAcc:MGI:2447024]
ENSMUSG00000096256	4933409K07Rik	RIKEN cDNA 4933409K07 gene [Source:MGI Symbol%3BAcc:MGI:1918429]
ENSMUSG00000061079	Zfp143	zinc finger protein 143 [Source:MGI Symbol%3BAcc:MGI:1277969]
ENSMUSG00000032593	Amigo3	adhesion molecule with Ig like domain 3 [Source:MGI Symbol%3BAcc:MGI:2444854]
ENSMUSG00000021198	Unc79	unc-79 homolog [Source:MGI Symbol%3BAcc:MGI:2684729]
ENSMUSG00000038406	Scaf1	SR-related CTD-associated factor 1 [Source:MGI Symbol%3BAcc:MGI:21419
ENSMUSG00000041696	Rasl12	RAS-like%2C family 12 [Source:MGI Symbol%3BAcc:MGI:1918034]
ENSMUSG00000046410	Kcnk6	potassium inwardly-rectifying channel%2C subfamily K%2C member 6 [Source:MGI Symbol%3BAcc:MGI:1891291]
ENSMUSG00000026579	F5	coagulation factor V [Source:MGI Symbol%3BAcc:MGI:88382]
ENSMUSG00000037405	Icam1	intercellular adhesion molecule 1 [Source:MGI Symbol%3BAcc:MGI:96392]
ENSMUSG00000064247	Plcxd1	phosphatidylinositol-specific phospholipase C%2C X domain containing 1 [Source:MGI Symbol%3BAcc:MGI:2685422]

ENSMUSG00000085776	5430402O13Rik	RIKEN cDNA 5430402O13 gene [Source:MGI Symbol%3BAcc:MGI:1918631]
ENSMUSG00000066197	Gpr139	G protein-coupled receptor 139 [Source:MGI Symbol%3BAcc:MGI:2685341]
ENSMUSG00000089718	2310075C17Rik	RIKEN cDNA 2310075C17 gene [Source:MGI Symbol%3BAcc:MGI:1917444]
ENSMUSG00000041669	Prima1	proline rich membrane anchor 1 [Source:MGI Symbol%3BAcc:MGI:1926097]
ENSMUSG00000087202	Gm15813	predicted gene 15813 [Source:MGI Symbol%3BAcc:MGI:3801815]
ENSMUSG00000021367	Edn1	endothelin 1 [Source:MGI Symbol%3BAcc:MGI:95283]
ENSMUSG00000068062	Gm14164	predicted gene 14164 [Source:MGI Symbol%3BAcc:MGI:3650730]
ENSMUSG00000104727	Gm42500	predicted gene 42500 [Source:MGI Symbol%3BAcc:MGI:5662637]
ENSMUSG00000118049	Gm50103	predicted gene%2C 50103 [Source:MGI Symbol%3BAcc:MGI:6302834]
ENSMUSG00000073158	9030624G23Rik	RIKEN cDNA 9030624G23 gene [Source:MGI Symbol%3BAcc:MGI:1914058]
ENSMUSG00000043340	6530409C15Rik	RIKEN cDNA 6530409C15 gene [Source:MGI Symbol%3BAcc:MGI:1923474]
ENSMUSG00000091003	Gm7244	predicted gene 7244 [Source:MGI Symbol%3BAcc:MGI:3644192]
ENSMUSG00000027315	Spint1	serine protease inhibitor%2C Kunitz type 1 [Source:MGI Symbol%3BAcc:MGI:13380
ENSMUSG00000097032	4930539J05Rik	RIKEN cDNA 4930539J05 gene [Source:MGI Symbol%3BAcc:MGI:1922477]
ENSMUSG00000115373	Gm49194	predicted gene%2C 49194 [Source:MGI Symbol%3BAcc:MGI:6118637]
ENSMUSG00000023868	Pde10a	phosphodiesterase 10A [Source:MGI Symbol%3BAcc:MGI:1345143]
ENSMUSG00000117905	Gm50230	predicted gene%2C 50230 [Source:MGI Symbol%3BAcc:MGI:6303033]
ENSMUSG00000109598	Gm36356	predicted gene%2C 36356 [Source:MGI Symbol%3BAcc:MGI:5595515]
ENSMUSG00000086924	Gm11766	predicted gene 11766 [Source:MGI Symbol%3BAcc:MGI:3650673]
ENSMUSG00000006219	Fblim1	filamin binding LIM protein 1 [Source:MGI Symbol%3BAcc:MGI:1921452]
ENSMUSG00000117486	Gm30938	predicted gene%2C 30938 [Source:MGI Symbol%3BAcc:MGI:5590097]
ENSMUSG00000025905	Oprk1	opioid receptor%2C kappa 1 [Source:MGI Symbol%3BAcc:MGI:97439]
ENSMUSG00000112433	Gm30122	predicted gene%2C 30122 [Source:MGI Symbol%3BAcc:MGI:5589281]
ENSMUSG00000031962	Cdh15	cadherin 15 [Source:MGI Symbol%3BAcc:MGI:106672]

ENSMUSG00000029328	Hnrnpdl	heterogeneous nuclear ribonucleoprotein D-like [Source:MGI Symbol%3BAcc:MGI:1355299]
ENSMUSG00000023919	Cenpq	centromere protein Q [Source:MGI Symbol%3BAcc:MGI:1933744]
ENSMUSG00000067279	Ppp1r3c	protein phosphatase 1%2C regulatory subunit 3C [Source:MGI Symbol%3BAcc:MGI:1858229]
ENSMUSG00000029096	Htra3	HtrA serine peptidase 3 [Source:MGI Symbol%3BAcc:MGI:1925808]
ENSMUSG00000041763	Tpp2	tripeptidyl peptidase II [Source:MGI Symbol%3BAcc:MGI:102724]
ENSMUSG00000067578	Cbln4	cerebellin 4 precursor protein [Source:MGI Symbol%3BAcc:MGI:2154433]
ENSMUSG00000043832	Clec4a3	C-type lectin domain family 4%2C member a3 [Source:MGI Symbol%3BAcc:MGI:1920399]
ENSMUSG00000061167	Rpl15-ps3	ribosomal protein L15%2C pseudogene 3 [Source:MGI Symbol%3BAcc:MGI:37829
ENSMUSG00000067924	Rtl8b	retrotransposon Gag like 8B [Source:MGI Symbol%3BAcc:MGI:3576504]
ENSMUSG00000094724	Rnaset2b	ribonuclease T2B [Source:MGI Symbol%3BAcc:MGI:3702087]
ENSMUSG00000068220	Lgals1	lectin%2C galactose binding%2C soluble 1 [Source:MGI Symbol%3BAcc:MGI:96777
ENSMUSG00000033174	Mgll	monoglyceride lipase [Source:MGI Symbol%3BAcc:MGI:1346042]
ENSMUSG00000022901	Cd86	CD86 antigen [Source:MGI Symbol%3BAcc:MGI:101773]
ENSMUSG00000046598	Bdh1	3-hydroxybutyrate dehydrogenase%2C type 1 [Source:MGI Symbol%3BAcc:MGI:1919161]
ENSMUSG00000105504	Gbp5	guanylate binding protein 5 [Source:MGI Symbol%3BAcc:MGI:2429943]
ENSMUSG00000021900	Btd	biotinidase [Source:MGI Symbol%3BAcc:MGI:1347001]
ENSMUSG00000040093	Bmf	BCL2 modifying factor [Source:MGI Symbol%3BAcc:MGI:2176433]
ENSMUSG00000068615	Gjd2	gap junction protein%2C delta 2 [Source:MGI Symbol%3BAcc:MGI:1334209]
ENSMUSG00000022543	Dnaaf8	dynein axonemal assembly factor 8 [Source:MGI Symbol%3BAcc:MGI:19219
ENSMUSG00000022148	Fyb	FYN binding protein [Source:MGI Symbol%3BAcc:MGI:1346327]
ENSMUSG00000027074	Slc43a3	solute carrier family 43%2C member 3 [Source:MGI Symbol%3BAcc:MGI:1931054]
ENSMUSG00000027875	Hmgcs2	3-hydroxy-3-methylglutaryl-Coenzyme A synthase 2 [Source:MGI Symbol%3BAcc:MGI:10193
ENSMUSG00000070392	Gm20634	predicted gene 20634 [Source:MGI Symbol%3BAcc:MGI:5313081]
ENSMUSG00000038777	Sema6c	sema domain%2C transmembrane domain (TM)%2C and cytoplasmic domain%2C (semaphorin) 6C [Source:MGI Symbol%3BAcc:MGI:1338032]

ENSMUSG00000105742	Gm42748	predicted gene 42748 [Source:MGI Symbol%3BAcc:MGI:5662885]
ENSMUSG00000019312	Grb7	growth factor receptor bound protein 7 [Source:MGI Symbol%3BAcc:MGI:10268
ENSMUSG00000116836	Gm49727	predicted gene%2C 49727 [Source:MGI Symbol%3BAcc:MGI:6215207]
ENSMUSG00000113965	A730091E23Rik	RIKEN cDNA A730091E23 gene [Source:MGI Symbol%3BAcc:MGI:2442493]
ENSMUSG00000112977	Gm34081	predicted gene%2C 34081 [Source:MGI Symbol%3BAcc:MGI:5593240]
ENSMUSG00000084941	Gm11944	predicted gene 11944 [Source:MGI Symbol%3BAcc:MGI:3702312]
ENSMUSG00000100053	Gm28154	predicted gene 28154 [Source:MGI Symbol%3BAcc:MGI:5578860]
ENSMUSG00000108825	Gm45838	predicted gene 45838 [Source:MGI Symbol%3BAcc:MGI:5804953]
ENSMUSG00000083808	Gm3828	predicted gene 3828 [Source:MGI Symbol%3BAcc:MGI:3782000]
ENSMUSG00000085687	Gm16153	predicted gene 16153 [Source:MGI Symbol%3BAcc:MGI:3802057]
ENSMUSG00000026979	Psd4	pleckstrin and Sec7 domain containing 4 [Source:MGI Symbol%3BAcc:MGI:26740
ENSMUSG00000108594	Gm44770	predicted gene 44770 [Source:MGI Symbol%3BAcc:MGI:5753346]
ENSMUSG00000101054	Gm18406	predicted gene%2C 18406 [Source:MGI Symbol%3BAcc:MGI:5010591]
ENSMUSG00000100241	Slc18a3	solute carrier family 18 (vesicular monoamine)%2C member 3 [Source:MGI Symbol%3BAcc:MGI:1101061]
ENSMUSG00000029204	Rhoh	ras homolog family member H [Source:MGI Symbol%3BAcc:MGI:1921984]
ENSMUSG00000120795	-	novel transcript%2C antisense to Ric8b
ENSMUSG00000087461	C230014O12Rik	RIKEN cDNA C230014O12 gene [Source:MGI Symbol%3BAcc:MGI:3045377]
ENSMUSG00000079045	Prox1os	prospero homeobox 1%2C opposite strand [Source:MGI Symbol%3BAcc:MGI:49372
ENSMUSG00000046456	Tmem150b	transmembrane protein 150B [Source:MGI Symbol%3BAcc:MGI:2679718]
ENSMUSG00000114907	Gm47794	predicted gene%2C 47794 [Source:MGI Symbol%3BAcc:MGI:6096966]
ENSMUSG00000099384	1700110C19Rik	RIKEN cDNA 1700110C19 gene [Source:MGI Symbol%3BAcc:MGI:1923884]
ENSMUSG00000107937	Gm44200	predicted gene%2C 44200 [Source:MGI Symbol%3BAcc:MGI:5690592]
ENSMUSG00000005547	Cyp2a5	cytochrome P450%2C family 2%2C subfamily a%2C polypeptide 5 [Source:MGI
ENSMUSG00000029607	Ankrd61	ankyrin repeat domain 61 [Source:MGI Symbol%3BAcc:MGI:1913979]
ENSMUSG00000015852	Fcrls	Fc receptor-like S%2C scavenger receptor [Source:MGI Symbol%3BAcc:MGI:1933397]
ENSMUSG00000069008	Gm5537	predicted gene 5537 [Source:MGI Symbol%3BAcc:MGI:3643179]
ENSMUSG00000037016	Frem2	Fras1 related extracellular matrix protein 2 [Source:MGI Symbol%3BAcc:MGI:2444465]
ENSMUSG00000026924	Sec16a	SEC16 homolog A%2C endoplasmic reticulum export factor [Source:MGI Symbol%3BAcc:MGI:2139207]
ENSMUSG00000059336	Slc14a1	solute carrier family 14 (urea transporter)%2C member 1 [Source:MGI Symbol%3BAcc:MGI:1351654]
ENSMUSG00000002147	Stat6	signal transducer and activator of transcription 6 [Source:MGI Symbol%3BAcc:MGI:10303
ENSMUSG00000069516	Lyz2	lysozyme 2 [Source:MGI Symbol%3BAcc:MGI:96897]
ENSMUSG00000069769	Msi2	musashi RNA-binding protein 2 [Source:MGI Symbol%3BAcc:MGI:1923876]
ENSMUSG00000069844	Sco1	SCO1 cytochrome c oxidase assembly protein [Source:MGI Symbol%3BAcc:MGI:10636
ENSMUSG00000020143	Dock2	dedicator of cyto-kinesis 2 [Source:MGI Symbol%3BAcc:MGI:2149010]
ENSMUSG00000041126	H2az2	H2A.Z histone variant 2 [Source:MGI Symbol%3BAcc:MGI:1924855]
ENSMUSG00000048148	Nwd1	NACHT and WD repeat domain containing 1 [Source:MGI Symbol%3BAcc:MGI:2442268]
ENSMUSG00000070509	Rgma	repulsive guidance molecule family member A [Source:MGI Symbol%3BAcc:MGI:2679262]
ENSMUSG00000041117	Ccdc8	coiled-coil domain containing 8 [Source:MGI Symbol%3BAcc:MGI:3612184]
ENSMUSG00000006019	Dhx34	DEAH (Asp-Glu-Ala-His) box polypeptide 34 [Source:MGI Symbol%3BAcc:MGI:1918973]
ENSMUSG00000029123	Stk32b	serine/threonine kinase 32B [Source:MGI Symbol%3BAcc:MGI:1927552]
ENSMUSG00000095427	Rps2-ps6	ribosomal protein S2%2C pseudogene 6 [Source:MGI Symbol%3BAcc:MGI:36448
ENSMUSG00000022123	Scel	sciellin [Source:MGI Symbol%3BAcc:MGI:1891228]
ENSMUSG00000071573	Rnls	renalase%2C FAD-dependent amine oxidase [Source:MGI Symbol%3BAcc:MGI:1915045]
ENSMUSG00000071691	Gm960	predicted gene 960 [Source:MGI Symbol%3BAcc:MGI:2685806]
ENSMUSG00000072407	Gm6419	predicted gene 6419 [Source:MGI Symbol%3BAcc:MGI:3647564]
ENSMUSG00000095687	Rnaset2a	ribonuclease T2A [Source:MGI Symbol%3BAcc:MGI:1915445]
ENSMUSG00000033594	Spata2l	spermatogenesis associated 2-like [Source:MGI Symbol%3BAcc:MGI:19260
ENSMUSG00000031659	Adcy7	adenylate cyclase 7 [Source:MGI Symbol%3BAcc:MGI:102891]

ENSMUSG00000109450	Gm39043	predicted gene%2C 39043 [Source:MGI Symbol%3BAcc:MGI:5621928]
ENSMUSG00000029219	Slc10a4	solute carrier family 10 (sodium/bile acid cotransporter family)%2C member 4 [Source:MGI Symbol%3BAcc:MGI:3606480]
ENSMUSG00000032691	Nlrp3	NLR family%2C pyrin domain containing 3 [Source:MGI Symbol%3BAcc:MGI:26538
ENSMUSG00000032715	Trib3	tribbles pseudokinase 3 [Source:MGI Symbol%3BAcc:MGI:1345675]
ENSMUSG00000104002	Gm38336	predicted gene%2C 38336 [Source:MGI Symbol%3BAcc:MGI:5611564]
ENSMUSG00000104164	Gm38248	predicted gene%2C 38248 [Source:MGI Symbol%3BAcc:MGI:5611476]
ENSMUSG00000090264	Eif4ebp3	eukaryotic translation initiation factor 4E binding protein 3 [Source:MGI Symbol%3BAcc:MGI:1270847]
ENSMUSG00000032572	Col6a4	collagen%2C type VI%2C alpha 4 [Source:MGI Symbol%3BAcc:MGI:1915803]
ENSMUSG00000103160	C130012C08Rik	RIKEN cDNA C130012C08 gene [Source:MGI Symbol%3BAcc:MGI:2145684]
ENSMUSG00000110827	Gm32281	predicted gene%2C 32281 [Source:MGI Symbol%3BAcc:MGI:5591440]
ENSMUSG00000044361	BC024139	cDNA sequence BC024139 [Source:MGI Symbol%3BAcc:MGI:2442591]
ENSMUSG00000029015	Slc26a5	solute carrier family 26%2C member 5 [Source:MGI Symbol%3BAcc:MGI:1933154]
ENSMUSG00000117926	C030004G16Rik	RIKEN cDNA C030004G16 gene [Source:MGI Symbol%3BAcc:MGI:1925786]
ENSMUSG00000081418	Gm14830	predicted gene 14830 [Source:MGI Symbol%3BAcc:MGI:3705386]
ENSMUSG00000085944	1700003D09Rik	RIKEN cDNA 1700003D09 gene [Source:MGI Symbol%3BAcc:MGI:1916588]
ENSMUSG00000048292	Olfr1417	olfactory receptor 1417 [Source:MGI Symbol%3BAcc:MGI:3031251]
ENSMUSG00000079478	Znrd2	zinc ribbon domain containing 2 [Source:MGI Symbol%3BAcc:MGI:1913482]
ENSMUSG00000029720	Gm20605	predicted gene 20605 [Source:MGI Symbol%3BAcc:MGI:5306917]
ENSMUSG00000102605	Gm37264	predicted gene%2C 37264 [Source:MGI Symbol%3BAcc:MGI:5610492]
ENSMUSG00000081344	Gm14303	predicted gene 14303 [Source:MGI Symbol%3BAcc:MGI:3651379]
ENSMUSG00000089997	1810020O05Rik	Riken cDNA 1810020O05 gene [Source:MGI Symbol%3BAcc:MGI:3586838]
ENSMUSG00000085041	BB031773	expressed sequence BB031773 [Source:MGI Symbol%3BAcc:MGI:2140631]
ENSMUSG00000042064	Myo3b	myosin IIIB [Source:MGI Symbol%3BAcc:MGI:2448580]
ENSMUSG00000097923	Gm26577	predicted gene%2C 26577 [Source:MGI Symbol%3BAcc:MGI:5477071]
ENSMUSG00000074342	I830077J02Rik	RIKEN cDNA I830077J02 gene [Source:MGI Symbol%3BAcc:MGI:3588284]
ENSMUSG00000078307	AI593442	expressed sequence AI593442 [Source:MGI Symbol%3BAcc:MGI:2143099]
ENSMUSG00000060594	Layn	layilin [Source:MGI Symbol%3BAcc:MGI:2685357]
ENSMUSG00000030386	Zfp606	zinc finger protein 606 [Source:MGI Symbol%3BAcc:MGI:1914620]
ENSMUSG00000074622	Mafb	v-maf musculoaponeurotic fibrosarcoma oncogene family%2C protein B (avian) [Source:MGI
ENSMUSG00000074743	Thbd	thrombomodulin [Source:MGI Symbol%3BAcc:MGI:98736]
ENSMUSG00000074785	Plxnc1	plexin C1 [Source:MGI Symbol%3BAcc:MGI:1890127]
ENSMUSG00000074800	Gm4149	predicted pseudogene 4149 [Source:MGI Symbol%3BAcc:MGI:3782325]
ENSMUSG00000074872	Ctxn2	cortexin 2 [Source:MGI Symbol%3BAcc:MGI:2139444]
ENSMUSG00000074890	Lcmt2	leucine carboxyl methyltransferase 2 [Source:MGI Symbol%3BAcc:MGI:1353659]
ENSMUSG00000040820	Hlcs	holocarboxylase synthetase (biotin-[propriony-Coenzyme A-carboxylase (ATP-hydrolysing)] ligase) [Source:MGI
ENSMUSG00000074939	Chrm5	cholinergic receptor%2C muscarinic 5 [Source:MGI Symbol%3BAcc:MGI:109248]
ENSMUSG00000075012	Fjx1	four jointed box 1 [Source:MGI Symbol%3BAcc:MGI:1341907]
ENSMUSG00000075266	Cenpw	centromere protein W [Source:MGI Symbol%3BAcc:MGI:1913561]
ENSMUSG00000075334	Rprm	reprimo%2C TP53 dependent G2 arrest mediator candidate [Source:MGI Symbol%3BAcc:MGI:1915124]
ENSMUSG00000022372	Sla	src-like adaptor [Source:MGI Symbol%3BAcc:MGI:104295]
ENSMUSG00000048163	Selplg	selectin%2C platelet (p-selectin) ligand [Source:MGI Symbol%3BAcc:MGI:10668
ENSMUSG00000023367	Tmem176a	transmembrane protein 176A [Source:MGI Symbol%3BAcc:MGI:1913308]
ENSMUSG00000029838	Ptn	pleiotrophin [Source:MGI Symbol%3BAcc:MGI:97804]
ENSMUSG00000060802	B2m	beta-2 microglobulin [Source:MGI Symbol%3BAcc:MGI:88127]
ENSMUSG00000027166	Dnajc24	DnaJ heat shock protein family (Hsp40) member C24 [Source:MGI Symbol%3BAcc:MGI:1919522]
ENSMUSG00000027078	Ube2l6	ubiquitin-conjugating enzyme E2L 6 [Source:MGI Symbol%3BAcc:MGI:1914500]
ENSMUSG00000067212	H2-T23	histocompatibility 2%2C T region locus 23 [Source:MGI
ENSMUSG00000074896	Ifit3	interferon-induced protein with tetratricopeptide repeats 3 [Source:MGI Symbol%3BAcc:MGI:11010

ENSMUSG00000069581	Tspear	thrombospondin type laminin G domain and EAR repeats [Source:MGI Symbol%3BAcc:MGI:2671932]
ENSMUSG00000039079	Trhr2	thyrotropin releasing hormone receptor 2 [Source:MGI Symbol%3BAcc:MGI:2177284]
ENSMUSG00000083015	Gm16518	predicted gene%2C 16518 [Source:MGI Symbol%3BAcc:MGI:4360828]
ENSMUSG00000097404	Gm10814	predicted gene 10814 [Source:MGI Symbol%3BAcc:MGI:3642580]
ENSMUSG00000028392	Bspry	B-box and SPRY domain containing [Source:MGI Symbol%3BAcc:MGI:2177191]
ENSMUSG00000074817	Papolb	poly (A) polymerase beta (testis specific) [Source:MGI Symbol%3BAcc:MGI:19321
ENSMUSG00000053063	Clec12a	C-type lectin domain family 12%2C member a [Source:MGI Symbol%3BAcc:MGI:3040968]
ENSMUSG00000115234	Gm49130	predicted gene%2C 49130 [Source:MGI Symbol%3BAcc:MGI:6118539]
ENSMUSG00000086128	St8sia3os	ST8 alpha-N-acetyl-neuraminide alpha-2%2C8-sialyltransferase 3%2C opposite strand [Source:MGI Symbol%3BAcc:MGI:19208
ENSMUSG00000116223	Gm41396	predicted gene%2C 41396 [Source:MGI Symbol%3BAcc:MGI:5624281]
ENSMUSG00000056508	1700001K19Rik	RIKEN cDNA 1700001K19 gene [Source:MGI Symbol%3BAcc:MGI:1913573]
ENSMUSG00000038540	Tmc3	transmembrane channel-like gene family 3 [Source:MGI Symbol%3BAcc:MGI:2669033]
ENSMUSG00000018566	Slc2a4	solute carrier family 2 (facilitated glucose transporter)%2C member 4 [Source:MGI Symbol%3BAcc:MGI:95758
ENSMUSG00000106887	A930005G22Rik	RIKEN cDNA A930005G22 gene [Source:MGI Symbol%3BAcc:MGI:3528178]
ENSMUSG00000035211	Xrra1	X-ray radiation resistance associated 1 [Source:MGI Symbol%3BAcc:MGI:2181647]
ENSMUSG00000096992	Gm26788	predicted gene%2C 26788 [Source:MGI Symbol%3BAcc:MGI:5477282]
ENSMUSG00000087322	Gm16075	predicted gene 16075 [Source:MGI Symbol%3BAcc:MGI:3801977]
ENSMUSG00000029658	Wdr95	WD40 repeat domain 95 [Source:MGI Symbol%3BAcc:MGI:1923042]
ENSMUSG00000018168	Ikzf3	IKAROS family zinc finger 3 [Source:MGI Symbol%3BAcc:MGI:1342542]
ENSMUSG00000097993	Ptprv	protein tyrosine phosphatase%2C receptor type%2C V [Source:MGI Symbol%3BAcc:MGI:108027]
ENSMUSG00000115856	Gm18095	predicted gene%2C 18095 [Source:MGI Symbol%3BAcc:MGI:5010280]
ENSMUSG00000074629	4930518I15Rik	RIKEN cDNA 4930518I15 gene [Source:MGI Symbol%3BAcc:MGI:1921954]
ENSMUSG00000044139	Prss53	protease%2C serine 53 [Source:MGI Symbol%3BAcc:MGI:2652890]
ENSMUSG00000045932	Ifit2	interferon-induced protein with tetratricopeptide repeats 2 [Source:MGI Symbol%3BAcc:MGI:99449
ENSMUSG00000020473	Aebp1	AE binding protein 1 [Source:MGI Symbol%3BAcc:MGI:1197012]
ENSMUSG00000027570	Col9a3	collagen%2C type IX%2C alpha 3 [Source:MGI Symbol%3BAcc:MGI:894686]
ENSMUSG00000020937	Plcd3	phospholipase C%2C delta 3 [Source:MGI Symbol%3BAcc:MGI:107451]
ENSMUSG00000006930	Hap1	huntingtin-associated protein 1 [Source:MGI Symbol%3BAcc:MGI:1261831]
ENSMUSG00000076435	Acsf2	acyl-CoA synthetase family member 2 [Source:MGI Symbol%3BAcc:MGI:23882
ENSMUSG00000076617	Ighm	immunoglobulin heavy constant mu [Source:MGI Symbol%3BAcc:MGI:96448]
ENSMUSG00000035640	Cbarp	calcium channel%2C voltage-dependent%2C beta subunit associated regulatory protein [Source:MGI Symbol%3BAcc:MGI:1354170]
ENSMUSG00000000732	Icosl	icos ligand [Source:MGI Symbol%3BAcc:MGI:1354701]
ENSMUSG00000019986	Ahi1	Abelson helper integration site 1 [Source:MGI Symbol%3BAcc:MGI:87971]
ENSMUSG00000040715	#N/A	#N/A
ENSMUSG00000050063	Klk6	kallikrein related-peptidase 6 [Source:MGI Symbol%3BAcc:MGI:1343166]
ENSMUSG00000070604	Vsig10l	V-set and immunoglobulin domain containing 10 like [Source:MGI Symbol%3BAcc:MGI:19229
ENSMUSG00000002068	Ccne1	cyclin E1 [Source:MGI Symbol%3BAcc:MGI:88316]
ENSMUSG00000027669	Gnb4	guanine nucleotide binding protein (G protein)%2C beta 4 [Source:MGI
ENSMUSG00000016356	Col20a1	collagen%2C type XX%2C alpha 1 [Source:MGI Symbol%3BAcc:MGI:1920618]
ENSMUSG00000078867	Gm14418	predicted gene 14418 [Source:MGI Symbol%3BAcc:MGI:3702408]
ENSMUSG00000095648	Gm2004	predicted gene 2004 [Source:MGI Symbol%3BAcc:MGI:3780174]
ENSMUSG00000047907	Tshz2	teashirt zinc finger family member 2 [Source:MGI Symbol%3BAcc:MGI:2153084]
ENSMUSG00000021662	Arhgef28	Rho guanine nucleotide exchange factor (GEF) 28 [Source:MGI Symbol%3BAcc:MGI:1346016]
ENSMUSG00000053693	Mast1	microtubule associated serine/threonine kinase 1 [Source:MGI Symbol%3BAcc:MGI:1861901]
ENSMUSG00000079197	Psme2	proteasome (prosome%2C macropain) activator subunit 2 (PA28 beta) [Source:MGI Symbol%3BAcc:MGI:10963
ENSMUSG00000041688	Amot	angiomotin [Source:MGI Symbol%3BAcc:MGI:108440]

ENSMUSG00000055341	Zfp457	zinc finger protein 457 [Source:MGI Symbol%3BAcc:MGI:2664334]
ENSMUSG00000111435	Gm47358	predicted gene%2C 47358 [Source:MGI Symbol%3BAcc:MGI:6096264]
ENSMUSG00000108486	Gm44836	predicted gene 44836 [Source:MGI Symbol%3BAcc:MGI:5753412]
ENSMUSG00000055994	Nod2	nucleotide-binding oligomerization domain containing 2 [Source:MGI Symbol%3BAcc:MGI:2429397]
ENSMUSG00000120903	-	novel transcript
ENSMUSG00000108808	Gm45204	predicted gene 45204 [Source:MGI Symbol%3BAcc:MGI:5753780]
ENSMUSG00000074508	Gm10706	predicted gene 10706 [Source:MGI Symbol%3BAcc:MGI:3642794]
ENSMUSG00000040441	Slc26a10	solute carrier family 26%2C member 10 [Source:MGI Symbol%3BAcc:MGI:2143920]
ENSMUSG00000109366	Gm44698	predicted gene 44698 [Source:MGI Symbol%3BAcc:MGI:5753274]
ENSMUSG00000078887	Gm6710	predicted gene 6710 [Source:MGI Symbol%3BAcc:MGI:3779623]
ENSMUSG00000072982	9230009I02Rik	RIKEN cDNA 9230009I02 gene [Source:MGI Symbol%3BAcc:MGI:3588229]
ENSMUSG00000112237	D630033A02Rik	RIKEN cDNA D630033A02 gene [Source:MGI Symbol%3BAcc:MGI:3028060]
ENSMUSG00000025479	Cyp2e1	cytochrome P450%2C family 2%2C subfamily e%2C polypeptide 1 [Source:MGI
ENSMUSG00000097069	Gm16998	predicted gene%2C 16998 [Source:MGI Symbol%3BAcc:MGI:4439922]
ENSMUSG00000030789	Itgax	integrin alpha X [Source:MGI Symbol%3BAcc:MGI:96609]
ENSMUSG00000110396	Gm36737	predicted gene%2C 36737 [Source:MGI Symbol%3BAcc:MGI:5595896]
ENSMUSG00000115610	Gm31251	predicted gene%2C 31251 [Source:MGI Symbol%3BAcc:MGI:5590410]
ENSMUSG00000111873	Gm39463	predicted gene%2C 39463 [Source:MGI Symbol%3BAcc:MGI:5622348]
ENSMUSG00000086662	BC046251	cDNA sequence BC046251 [Source:MGI Symbol%3BAcc:MGI:3039598]
ENSMUSG00000120013	-	novel transcript
ENSMUSG00000112721	Gm35608	predicted gene%2C 35608 [Source:MGI Symbol%3BAcc:MGI:5594767]
ENSMUSG00000009356	Lpo	lactoperoxidase [Source:MGI Symbol%3BAcc:MGI:1923363]
ENSMUSG00000027196	Alkbh3os1	alkB homolog 3%2C opposite strand 1 [Source:MGI Symbol%3BAcc:MGI:19181
ENSMUSG00000075297	H60b	histocompatibility 60b [Source:MGI Symbol%3BAcc:MGI:3649078]
ENSMUSG00000086855	Gm13844	predicted gene 13844 [Source:MGI Symbol%3BAcc:MGI:3651305]
ENSMUSG00000079418	Atg4a	autophagy related 4A%2C cysteine peptidase [Source:MGI Symbol%3BAcc:MGI:2147903]
ENSMUSG00000079427	Mthfsl	5%2C 10-methenyltetrahydrofolate synthetase-like [Source:MGI Symbol%3BAcc:MGI:37805
ENSMUSG00000043384	Gprasp1	G protein-coupled receptor associated sorting protein 1 [Source:MGI Symbol%3BAcc:MGI:19174
ENSMUSG00000053835	H2-T24	histocompatibility 2%2C T region locus 24 [Source:MGI Symbol%3BAcc:MGI:95958]
ENSMUSG00000022876	Samsn1	SAM domain%2C SH3 domain and nuclear localization signals%2C 1 [Source:MGI Symbol%3BAcc:MGI:1914992]
ENSMUSG00000022791	Tnk2	tyrosine kinase%2C non-receptor%2C 2 [Source:MGI Symbol%3BAcc:MGI:18583
ENSMUSG00000103770	Pcdha9	protocadherin alpha 9 [Source:MGI Symbol%3BAcc:MGI:2447322]
ENSMUSG00000024347	Psd2	pleckstrin and Sec7 domain containing 2 [Source:MGI Symbol%3BAcc:MGI:19212
ENSMUSG00000082414	Gm13303	predicted gene 13303 [Source:MGI Symbol%3BAcc:MGI:3705775]
ENSMUSG00000032850	Rnft2	ring finger protein%2C transmembrane 2 [Source:MGI Symbol%3BAcc:MGI:2442859]
ENSMUSG00000083563	Gm13340	predicted gene 13340 [Source:MGI Symbol%3BAcc:MGI:3650227]
ENSMUSG00000082998	Gm11221	predicted gene 11221 [Source:MGI Symbol%3BAcc:MGI:3651633]
ENSMUSG00000083679	Gm12892	predicted gene 12892 [Source:MGI Symbol%3BAcc:MGI:3649649]
ENSMUSG00000031207	Msn	moesin [Source:MGI Symbol%3BAcc:MGI:97167]
ENSMUSG00000081049	Rps24-ps3	ribosomal protein S24%2C pseudogene 3 [Source:MGI Symbol%3BAcc:MGI:36444
ENSMUSG00000067147	Rpl7a-ps11	ribosomal protein L7A%2C pseudogene 11 [Source:MGI Symbol%3BAcc:MGI:36440
ENSMUSG00000082035	Rpl17-ps8	ribosomal protein L17%2C pseudogene 8 [Source:MGI Symbol%3BAcc:MGI:38021
ENSMUSG00000034324	Tmem132c	transmembrane protein 132C [Source:MGI Symbol%3BAcc:MGI:2443061]
ENSMUSG00000058809	Hspd1-ps3	heat shock protein 1 (chaperonin)%2C pseudogene 3 [Source:MGI Symbol%3BAcc:MGI:36512
ENSMUSG00000082100	Glns-ps1	glutamine synthetase pseudogene 1 [Source:MGI
ENSMUSG00000087701	Gm13493	predicted gene 13493 [Source:MGI Symbol%3BAcc:MGI:3650824]
ENSMUSG00000101431	Gm7901	predicted gene 7901 [Source:MGI Symbol%3BAcc:MGI:3645047]
ENSMUSG00000082274	Gm14026	predicted gene 14026 [Source:MGI Symbol%3BAcc:MGI:3649712]
ENSMUSG00000081170	Gm12922	predicted gene 12922 [Source:MGI Symbol%3BAcc:MGI:3651537]
ENSMUSG00000082884	Gm13339	predicted gene 13339 [Source:MGI Symbol%3BAcc:MGI:3650221]

ENSMUSG00000117848	Gm35867	predicted gene%2C 35867 [Source:MGI Symbol%3BAcc:MGI:5595026]
ENSMUSG00000032494	Tdgf1	teratocarcinoma-derived growth factor 1 [Source:MGI Symbol%3BAcc:MGI:98658
ENSMUSG00000059406	Tmprss9	transmembrane protease%2C serine 9 [Source:MGI Symbol%3BAcc:MGI:3612246]
ENSMUSG00000049115	Agtr1a	angiotensin II receptor%2C type 1a [Source:MGI Symbol%3BAcc:MGI:87964]
ENSMUSG00000025197	Cyp2c23	cytochrome P450%2C family 2%2C subfamily c%2C polypeptide 23 [Source:MGI Symbol%3BAcc:MGI:18888
ENSMUSG00000001333	Sync	syncoilin [Source:MGI Symbol%3BAcc:MGI:1916078]
ENSMUSG00000022056	Adam7	a disintegrin and metallopeptidase domain 7 [Source:MGI Symbol%3BAcc:MGI:10724
ENSMUSG00000081378	Rps13-ps4	ribosomal protein S13%2C pseudogene 4 [Source:MGI Symbol%3BAcc:MGI:36488
ENSMUSG00000026828	Galnt5	polypeptide N-acetylgalactosaminyltransferase 5 [Source:MGI Symbol%3BAcc:MGI:2179403]
ENSMUSG00000097439	Gm16754	predicted gene%2C 16754 [Source:MGI Symbol%3BAcc:MGI:4439678]
ENSMUSG00000111246	4930517E14Rik	Riken cDNA 4930517E14 gene [Source:MGI Symbol%3BAcc:MGI:5608878]
ENSMUSG00000064389	Gm26446	predicted gene%2C 26446 [Source:MGI Symbol%3BAcc:MGI:5456223]
ENSMUSG00000040752	Myh6	myosin%2C heavy polypeptide 6%2C cardiac muscle%2C alpha [Source:MGI Symbol%3BAcc:MGI:97255
ENSMUSG00000022479	Vdr	vitamin D (1%2C25-dihydroxyvitamin D3) receptor [Source:MGI Symbol%3BAcc:MGI:10307
ENSMUSG00000114796	A930028N01Rik	RIKEN cDNA A930028N01 gene [Source:MGI Symbol%3BAcc:MGI:1925688]
ENSMUSG00000101179	Gm29455	predicted gene 29455 [Source:MGI Symbol%3BAcc:MGI:5580161]
ENSMUSG00000092286	Dtnbos	dystrobrevin%2C beta%2C opposite strand [Source:MGI Symbol%3BAcc:MGI:51419
ENSMUSG00000087059	Gm12339	predicted gene 12339 [Source:MGI Symbol%3BAcc:MGI:3650628]
ENSMUSG00000091160	Gm17133	predicted gene 17133 [Source:MGI Symbol%3BAcc:MGI:4937960]
ENSMUSG00000036961	Wnt8b	wingless-type MMTV integration site family%2C member 8B [Source:MGI Symbol%3BAcc:MGI:10948
ENSMUSG00000117321	Gm49927	predicted gene%2C 49927 [Source:MGI Symbol%3BAcc:MGI:6270635]
ENSMUSG00000042800	Spata46	spermatogenesis associated 46 [Source:MGI Symbol%3BAcc:MGI:1924175]
ENSMUSG00000012705	Retn	resistin [Source:MGI Symbol%3BAcc:MGI:1888506]
ENSMUSG00000106565	Gm43582	predicted gene 43582 [Source:MGI Symbol%3BAcc:MGI:5663719]
ENSMUSG00000055632	Hmcn2	hemicentin 2 [Source:MGI Symbol%3BAcc:MGI:2677838]
ENSMUSG00000082070	Gm1866	predicted gene 1866 [Source:MGI Symbol%3BAcc:MGI:3037724]
ENSMUSG00000082895	Rpsa-ps9	ribosomal protein SA%2C pseudogene 9 [Source:MGI Symbol%3BAcc:MGI:36503
ENSMUSG00000050900	Gm7327	predicted gene 7327 [Source:MGI Symbol%3BAcc:MGI:3646274]
ENSMUSG00000081021	Gm11964	predicted gene 11964 [Source:MGI Symbol%3BAcc:MGI:3652241]
ENSMUSG00000083889	E530001F21Rik	RIKEN cDNA E530001F21 gene [Source:MGI Symbol%3BAcc:MGI:3036288]
ENSMUSG00000081113	Gm7308	predicted pseudogene 7308 [Source:MGI Symbol%3BAcc:MGI:3644558]
ENSMUSG00000082194	Gm12444	predicted gene 12444 [Source:MGI Symbol%3BAcc:MGI:3650375]
ENSMUSG00000083284	Gm13688	predicted gene 13688 [Source:MGI Symbol%3BAcc:MGI:3652062]
ENSMUSG00000080888	Gm14387	predicted gene 14387 [Source:MGI Symbol%3BAcc:MGI:3650318]
ENSMUSG00000086158	Ccpg1os	cell cycle progression 1%2C opposite strand [Source:MGI Symbol%3BAcc:MGI:36487
ENSMUSG00000060989	Gm11847	predicted gene 11847 [Source:MGI Symbol%3BAcc:MGI:3651787]
ENSMUSG00000086728	Man2c1os	mannosidase%2C alpha%2C class 2C%2C member 1%2C opposite strand [Source:MGI Symbol%3BAcc:MGI:19240
ENSMUSG00000096696	Zfp960	zinc finger protein 960 [Source:MGI Symbol%3BAcc:MGI:3052731]
ENSMUSG00000084808	9430091E24Rik	RIKEN cDNA 9430091E24 gene [Source:MGI Symbol%3BAcc:MGI:1924618]
ENSMUSG00000087141	Plcxd2	phosphatidylinositol-specific phospholipase C%2C X domain containing 2 [Source:MGI Symbol%3BAcc:MGI:3647874]
ENSMUSG00000056999	Ide	insulin degrading enzyme [Source:MGI Symbol%3BAcc:MGI:96412]
ENSMUSG00000064193	Gm4735	predicted gene 4735 [Source:MGI Symbol%3BAcc:MGI:3645521]
ENSMUSG00000087437	Gm13112	predicted gene 13112 [Source:MGI Symbol%3BAcc:MGI:3701127]
ENSMUSG00000069862	Rps12-ps9	ribosomal protein S12%2C pseudogene 9 [Source:MGI Symbol%3BAcc:MGI:37043
ENSMUSG00000085389	1700003M07Rik	RIKEN cDNA 1700003M07 gene [Source:MGI Symbol%3BAcc:MGI:1919475]
ENSMUSG00000091556	Gm14569	predicted gene 14569 [Source:MGI Symbol%3BAcc:MGI:3705243]
ENSMUSG00000062393	Dgkk	diacylglycerol kinase kappa [Source:MGI Symbol%3BAcc:MGI:3580254]
ENSMUSG00000087014	Gm16364	predicted gene 16364 [Source:MGI Symbol%3BAcc:MGI:3840142]
ENSMUSG00000087445	Gm14286	predicted gene 14286 [Source:MGI Symbol%3BAcc:MGI:3650190]
ENSMUSG00000089840	Gm4491	predicted gene 4491 [Source:MGI Symbol%3BAcc:MGI:3782676]

ENSMUSG00000103445	Gm36948	predicted gene%2C 36948 [Source:MGI Symbol%3BAcc:MGI:5610176]
ENSMUSG00000087094	Gm13093	predicted gene 13093 [Source:MGI Symbol%3BAcc:MGI:3650866]
ENSMUSG00000112596	Gm48804	predicted gene%2C 48804 [Source:MGI Symbol%3BAcc:MGI:6098514]
ENSMUSG00000002500	Rpl3l	ribosomal protein L3-like [Source:MGI Symbol%3BAcc:MGI:1913461]
ENSMUSG00000078308	Gm47854	predicted gene%2C 47854 [Source:MGI Symbol%3BAcc:MGI:6097063]
ENSMUSG00000026940	Ccdc183	coiled-coil domain containing 183 [Source:MGI Symbol%3BAcc:MGI:19243
ENSMUSG00000036816	Atoh7	atonal bHLH transcription factor 7 [Source:MGI Symbol%3BAcc:MGI:1355553]
ENSMUSG00000078721	Fam205a1	family with sequence similarity 205%2C member A1 [Source:MGI Symbol%3BAcc:MGI:3651059]
ENSMUSG00000043192	Gpi-ps	glucose-6-phosphate isomerase%2C pseudogene [Source:MGI Symbol%3BAcc:MGI:3037698]
ENSMUSG00000058669	Nkx2-9	NK2 homeobox 9 [Source:MGI Symbol%3BAcc:MGI:1270158]
ENSMUSG00000109040	Gm44542	predicted gene 44542 [Source:MGI Symbol%3BAcc:MGI:5753118]
ENSMUSG00000058046	4933430I17Rik	RIKEN cDNA 4933430I17 gene [Source:MGI Symbol%3BAcc:MGI:3045314]
ENSMUSG00000105137	Gm42869	predicted gene 42869 [Source:MGI Symbol%3BAcc:MGI:5663006]
ENSMUSG00000039878	Slc39a5	solute carrier family 39 (metal ion transporter)%2C member 5 [Source:MGI Symbol%3BAcc:MGI:1919336]
ENSMUSG00000049685	Cyp2g1	cytochrome P450%2C family 2%2C subfamily g%2C polypeptide 1 [Source:MGI Symbol%3BAcc:MGI:109612]
ENSMUSG00000086806	Gm13054	predicted gene 13054 [Source:MGI Symbol%3BAcc:MGI:3702728]
ENSMUSG00000100108	Gm28100	predicted gene 28100 [Source:MGI Symbol%3BAcc:MGI:5578806]
ENSMUSG00000094152	Slc6a16	solute carrier family 6%2C member 16 [Source:MGI Symbol%3BAcc:MGI:2685930]
ENSMUSG00000013974	Mcemp1	mast cell expressed membrane protein 1 [Source:MGI Symbol%3BAcc:MGI:1916439]
ENSMUSG00000110516	Gm45758	predicted gene 45758 [Source:MGI Symbol%3BAcc:MGI:5804873]
ENSMUSG00000048442	Smim5	small integral membrane protein 5 [Source:MGI Symbol%3BAcc:MGI:1913778]
ENSMUSG00000062438	Adam1b	a disintegrin and metallopeptidase domain 1b [Source:MGI Symbol%3BAcc:MGI:2429506]
ENSMUSG00000102628	Gm37671	predicted gene%2C 37671 [Source:MGI Symbol%3BAcc:MGI:5610899]

ENSMUSG00000090061	Nwd2	NACHT and WD repeat domain containing 2 [Source:MGI Symbol%3BAcc:MGI:1920464]
ENSMUSG00000090164	BC035044	cDNA sequence BC035044 [Source:MGI Symbol%3BAcc:MGI:2448540]
ENSMUSG00000091002	Tcerg1l	transcription elongation regulator 1-like [Source:MGI Symbol%3BAcc:MGI:19178
ENSMUSG00000089767	4930556I23Rik	RIKEN cDNA 4930556I23 gene [Source:MGI Symbol%3BAcc:MGI:1922520]
ENSMUSG00000089886	Gm16184	predicted gene 16184 [Source:MGI Symbol%3BAcc:MGI:3802154]
ENSMUSG00000039735	Fnbp1l	formin binding protein 1-like [Source:MGI Symbol%3BAcc:MGI:1925642]
ENSMUSG00000059498	Fcgr3	Fc receptor%2C IgG%2C low affinity III [Source:MGI Symbol%3BAcc:MGI:95500]
ENSMUSG00000015176	Nolc1	nucleolar and coiled-body phosphoprotein 1 [Source:MGI Symbol%3BAcc:MGI:1918019]
ENSMUSG00000090958	Lrrc32	leucine rich repeat containing 32 [Source:MGI Symbol%3BAcc:MGI:93882]
ENSMUSG00000094628	Gm3252	predicted gene 3252 [Source:MGI Symbol%3BAcc:MGI:3781430]
ENSMUSG00000091472	Gm3739	predicted gene 3739 [Source:MGI Symbol%3BAcc:MGI:3781914]
ENSMUSG00000028270	Gbp2	guanylate binding protein 2 [Source:MGI Symbol%3BAcc:MGI:102772]
ENSMUSG00000085584	Rtl9	retrotransposon Gag like 9 [Source:MGI Symbol%3BAcc:MGI:2685231]
ENSMUSG00000075289	Carns1	carnosine synthase 1 [Source:MGI Symbol%3BAcc:MGI:2147595]
ENSMUSG00000026458	Ppfia4	protein tyrosine phosphatase%2C receptor type%2C f polypeptide (PTPRF)%2C interacting protein (liprin)%2C alpha 4 [Source:MGI Symbol%3BAcc:MGI:1915757]
ENSMUSG00000063626	Unc5d	unc-5 netrin receptor D [Source:MGI Symbol%3BAcc:MGI:2389364]
ENSMUSG00000092118	Fancf	Fanconi anemia%2C complementation group F [Source:MGI Symbol%3BAcc:MGI:3689889]
ENSMUSG00000090610	Gm3571	predicted gene 3571 [Source:MGI Symbol%3BAcc:MGI:3781748]
ENSMUSG00000091387	Gcnt4	glucosaminyl (N-acetyl) transferase 4%2C core 2 (beta-1%2C6-N-acetylglucosaminyltransferase) [Source:MGI Symbol%3BAcc:MGI:2684919]
ENSMUSG00000039004	Bmp6	bone morphogenetic protein 6 [Source:MGI Symbol%3BAcc:MGI:88182]
ENSMUSG00000090936	Gm17705	predicted gene%2C 17705 [Source:MGI Symbol%3BAcc:MGI:4937339]
ENSMUSG00000073411	H2-D1	histocompatibility 2%2C D region locus 1 [Source:MGI Symbol%3BAcc:MGI:95896]
ENSMUSG00000092274	Neat1	nuclear paraspeckle assembly transcript 1 (non-protein coding) [Source:MGI Symbol%3BAcc:MGI:19142

ENSMUSG00000116635	Gm49699	predicted gene%2C 49699 [Source:MGI Symbol%3BAcc:MGI:6215157]
ENSMUSG00000082860	Gm13204	predicted gene 13204 [Source:MGI Symbol%3BAcc:MGI:3651444]
ENSMUSG00000106967	Gm42477	predicted gene 42477 [Source:MGI Symbol%3BAcc:MGI:5662614]
ENSMUSG00000085336	Gm11732	predicted gene 11732 [Source:MGI Symbol%3BAcc:MGI:3652087]
ENSMUSG00000087476	Rap1gapos	RAP1 GTPase activating protein%2C opposite strand [Source:MGI Symbol%3BAcc:MGI:1917058]
ENSMUSG00000051076	Vtcn1	V-set domain containing T cell activation inhibitor 1 [Source:MGI Symbol%3BAcc:MGI:3039619]
ENSMUSG00000114774	Gm35281	predicted gene%2C 35281 [Source:MGI Symbol%3BAcc:MGI:5594440]
ENSMUSG00000108520	Gm44684	predicted gene 44684 [Source:MGI Symbol%3BAcc:MGI:5753260]
ENSMUSG00000108035	Gm44021	predicted gene%2C 44021 [Source:MGI Symbol%3BAcc:MGI:5690413]
ENSMUSG00000097323	4930426I24Rik	RIKEN cDNA 4930426I24 gene [Source:MGI Symbol%3BAcc:MGI:1921125]
ENSMUSG00000103636	Gm38331	predicted gene%2C 38331 [Source:MGI Symbol%3BAcc:MGI:5611559]
ENSMUSG00000030159	Clec1b	C-type lectin domain family 1%2C member b [Source:MGI Symbol%3BAcc:MGI:1913287]
ENSMUSG00000090535	Gm8540	predicted gene 8540 [Source:MGI Symbol%3BAcc:MGI:3645422]
ENSMUSG00000010651	Acaa1b	acetyl-Coenzyme A acyltransferase 1B [Source:MGI Symbol%3BAcc:MGI:3605455]
ENSMUSG00000085887	Arhgap27os3	Rho GTPase activating protein 27%2C opposite strand 3 [Source:MGI Symbol%3BAcc:MGI:3650159]
ENSMUSG00000120142	-	novel transcript
ENSMUSG00000087148	1700030C14Rik	RIKEN cDNA 1700030C14 gene [Source:MGI Symbol%3BAcc:MGI:1922912]
ENSMUSG00000036198	Arhgap36	Rho GTPase activating protein 36 [Source:MGI Symbol%3BAcc:MGI:1922654]
ENSMUSG00000089827	1700023H06Rik	RIKEN cDNA 1700023H06 gene [Source:MGI Symbol%3BAcc:MGI:1916692]
ENSMUSG00000116288	Gm41349	predicted gene%2C 41349 [Source:MGI Symbol%3BAcc:MGI:5624234]
ENSMUSG00000013418	B4galnt2	beta-1%2C4-N-acetyl-galactosaminyl transferase 2 [Source:MGI Symbol%3BAcc:MGI:1342058]
ENSMUSG00000090120	A730090N16Rik	RIKEN cDNA A730090N16 gene [Source:MGI Symbol%3BAcc:MGI:2442748]
ENSMUSG00000064919	Gm22572	predicted gene%2C 22572 [Source:MGI Symbol%3BAcc:MGI:5452349]
ENSMUSG00000102324	Gm19721	predicted gene%2C 19721 [Source:MGI Symbol%3BAcc:MGI:5011906]
ENSMUSG00000001493	Meox1	mesenchyme homeobox 1 [Source:MGI Symbol%3BAcc:MGI:103220]
ENSMUSG00000101932	Gm10074	predicted gene 10074 [Source:MGI Symbol%3BAcc:MGI:3708632]
ENSMUSG00000093392	#N/A	#N/A
ENSMUSG00000093637	Gm20636	predicted gene 20636 [Source:MGI Symbol%3BAcc:MGI:5313083]
ENSMUSG00000093577	Gm20632	predicted gene 20632 [Source:MGI Symbol%3BAcc:MGI:5313079]
ENSMUSG00000094090	Gm8494	predicted gene 8494 [Source:MGI Symbol%3BAcc:MGI:3645114]
ENSMUSG00000096726	Gm5558	predicted gene 5558 [Source:MGI Symbol%3BAcc:MGI:3644616]
ENSMUSG00000097042	Gm17491	predicted gene%2C 17491 [Source:MGI Symbol%3BAcc:MGI:4937125]
ENSMUSG00000097929	Tunar	Tcl1 upstream neural differentiation associated RNA [Source:MGI Symbol%3BAcc:MGI:1917202]
ENSMUSG00000097195	Snhg5	small nucleolar RNA host gene 5 [Source:MGI Symbol%3BAcc:MGI:1919905]
ENSMUSG00000097305	Gm17276	predicted gene%2C 17276 [Source:MGI Symbol%3BAcc:MGI:4936910]
ENSMUSG00000097280	AI849053	expressed sequence AI849053 [Source:MGI Symbol%3BAcc:MGI:2139818]
ENSMUSG00000097515	1700040D17Rik	RIKEN cDNA 1700040D17 gene [Source:MGI Symbol%3BAcc:MGI:1923852]
ENSMUSG00000097649	Gm10561	predicted gene 10561 [Source:MGI Symbol%3BAcc:MGI:3648163]
ENSMUSG00000097890	4930547M16Rik	RIKEN cDNA 4930547M16 gene [Source:MGI Symbol%3BAcc:MGI:1922549]
ENSMUSG00000097520	4930488L21Rik	RIKEN cDNA 4930488L21 gene [Source:MGI Symbol%3BAcc:MGI:1923059]
ENSMUSG00000098424	Gm27202	predicted gene 27202 [Source:MGI Symbol%3BAcc:MGI:5521045]
ENSMUSG00000100671	Gm28322	predicted gene 28322 [Source:MGI Symbol%3BAcc:MGI:5579028]
ENSMUSG00000101439	Gm336	predicted pseudogene 336 [Source:MGI Symbol%3BAcc:MGI:2685182]
ENSMUSG00000101249	Gm29216	predicted gene 29216 [Source:MGI Symbol%3BAcc:MGI:5579922]
ENSMUSG00000099839	Gm29374	predicted gene 29374 [Source:MGI Symbol%3BAcc:MGI:5580080]
ENSMUSG00000100131	Gm28439	predicted gene 28439 [Source:MGI Symbol%3BAcc:MGI:5579145]
ENSMUSG00000102070	Gm28661	predicted gene 28661 [Source:MGI Symbol%3BAcc:MGI:5579367]
ENSMUSG00000101939	Gm28438	predicted gene 28438 [Source:MGI Symbol%3BAcc:MGI:5579144]
ENSMUSG00000100862	Gm10925	predicted gene 10925 [Source:MGI Symbol%3BAcc:MGI:3809095]
ENSMUSG00000101111	Gm28437	predicted gene 28437 [Source:MGI Symbol%3BAcc:MGI:5579143]

ENSMUSG00000113571	Gm48137	predicted gene%2C 48137 [Source:MGI Symbol%3BAcc:MGI:6097500]
ENSMUSG00000076144	Mir709	microRNA 709 [Source:MGI Symbol%3BAcc:MGI:3629717]
ENSMUSG00000054612	Mgmt	O-6-methylguanine-DNA methyltransferase [Source:MGI Symbol%3BAcc:MGI:96977
ENSMUSG00000121097	-	novel transcript%2C antisense to Il17rc
ENSMUSG00000100546	Gm29483	predicted gene 29483 [Source:MGI Symbol%3BAcc:MGI:5580189]
ENSMUSG00000031390	Avpr2	arginine vasopressin receptor 2 [Source:MGI Symbol%3BAcc:MGI:88123]
ENSMUSG00000120189	-	novel transcript%2C antisense to Cdkn2c
ENSMUSG00000035930	Chst4	carbohydrate sulfotransferase 4 [Source:MGI Symbol%3BAcc:MGI:1349479]
ENSMUSG00000097603	A430010J10Rik	RIKEN cDNA A430010J10 gene [Source:MGI Symbol%3BAcc:MGI:2442501]
ENSMUSG00000108347	Gm19187	predicted gene%2C 19187 [Source:MGI Symbol%3BAcc:MGI:5011372]
ENSMUSG00000120441	-	novel transcript%2C antisense to Slc39a13
ENSMUSG00000114050	Gm47666	predicted gene%2C 47666 [Source:MGI Symbol%3BAcc:MGI:6096758]
ENSMUSG00000016458	Wt1	WT1 transcription factor [Source:MGI Symbol%3BAcc:MGI:98968]
ENSMUSG00000086053	Gm15178	predicted gene 15178 [Source:MGI Symbol%3BAcc:MGI:3705132]
ENSMUSG00000087013	2610027K06Rik	RIKEN cDNA 2610027K06 gene [Source:MGI Symbol%3BAcc:MGI:1917159]
ENSMUSG00000042554	Zp3r	zona pellucida 3 receptor [Source:MGI Symbol%3BAcc:MGI:104965]
ENSMUSG00000105120	Gm26413	predicted gene%2C 26413 [Source:MGI Symbol%3BAcc:MGI:5456190]
ENSMUSG00000117380	E430002N23Rik	RIKEN cDNA E430002N23 gene [Source:MGI Symbol%3BAcc:MGI:2442036]
ENSMUSG00000064435	Gm25588	predicted gene%2C 25588 [Source:MGI Symbol%3BAcc:MGI:5455365]
ENSMUSG00000102425	Gm26616	predicted gene%2C 26616 [Source:MGI Symbol%3BAcc:MGI:5477110]
ENSMUSG00000111280	Gm47239	predicted gene%2C 47239 [Source:MGI Symbol%3BAcc:MGI:6096060]
ENSMUSG00000101565	1700003L19Rik	RIKEN cDNA 1700003L19 gene [Source:MGI Symbol%3BAcc:MGI:1922743]
ENSMUSG00000071893	Vmn1r4	vomeronasal 1 receptor 4 [Source:MGI Symbol%3BAcc:MGI:2159457]
ENSMUSG00000112105	Rps7-ps2	ribosomal protein S7%2C pseudogene 2 [Source:MGI Symbol%3BAcc:MGI:36460
ENSMUSG00000056569	Mpz	myelin protein zero [Source:MGI Symbol%3BAcc:MGI:103177]
ENSMUSG00000117779	Gm19209	predicted gene%2C 19209 [Source:MGI Symbol%3BAcc:MGI:5011394]
ENSMUSG00000099632	2900093K20Rik	RIKEN cDNA 2900093K20 gene [Source:MGI Symbol%3BAcc:MGI:1915287]
ENSMUSG00000104117	Gm20743	predicted gene%2C 20743 [Source:MGI Symbol%3BAcc:MGI:5434099]
ENSMUSG00000104415	Gm37069	predicted gene%2C 37069 [Source:MGI Symbol%3BAcc:MGI:5610297]
ENSMUSG00000103922	Gm6123	predicted gene 6123 [Source:MGI Symbol%3BAcc:MGI:3647047]
ENSMUSG00000103901	Gm37499	predicted gene%2C 37499 [Source:MGI Symbol%3BAcc:MGI:5610727]
ENSMUSG00000103451	Gm33973	predicted gene%2C 33973 [Source:MGI Symbol%3BAcc:MGI:5593132]
ENSMUSG00000103047	Gm37310	predicted gene%2C 37310 [Source:MGI Symbol%3BAcc:MGI:5610538]
ENSMUSG00000103735	Gm38317	predicted gene%2C 38317 [Source:MGI Symbol%3BAcc:MGI:5611545]
ENSMUSG00000105469	A930036I15Rik	RIKEN cDNA A930036I15 gene [Source:MGI Symbol%3BAcc:MGI:1925217]
ENSMUSG00000105843	Gm19439	predicted gene%2C 19439 [Source:MGI Symbol%3BAcc:MGI:5011624]
ENSMUSG00000105895	Gm42829	predicted gene 42829 [Source:MGI Symbol%3BAcc:MGI:5662966]
ENSMUSG00000106535	Gm43031	predicted gene 43031 [Source:MGI Symbol%3BAcc:MGI:5663168]
ENSMUSG00000105879	Gm6204	predicted gene 6204 [Source:MGI Symbol%3BAcc:MGI:3649038]
ENSMUSG00000107134	Gm42528	predicted gene 42528 [Source:MGI Symbol%3BAcc:MGI:5662665]
ENSMUSG00000106988	Tsg101-ps	tumor susceptibility gene 101%2C pseudogene [Source:MGI Symbol%3BAcc:MGI:1335078]
ENSMUSG00000107216	2210412B16Rik	RIKEN cDNA 2210412B16 gene [Source:MGI Symbol%3BAcc:MGI:1919625]
ENSMUSG00000107369	Gstm2-ps1	glutathione S-transferase mu 2 (muscle)%2C pseudogene 1 [Source:MGI Symbol%3BAcc:MGI:36482
ENSMUSG00000107881	Gm44250	predicted gene%2C 44250 [Source:MGI Symbol%3BAcc:MGI:5690642]
ENSMUSG00000107770	Gm44126	predicted gene%2C 44126 [Source:MGI Symbol%3BAcc:MGI:5690518]
ENSMUSG00000108528	Gm8319	predicted gene 8319 [Source:MGI Symbol%3BAcc:MGI:3646693]
ENSMUSG00000108799	Glud-ps	glutamate dehydrogenase%2C pseudogene [Source:MGI Symbol%3BAcc:MGI:95754
ENSMUSG00000108772	Gm6063	predicted gene 6063 [Source:MGI Symbol%3BAcc:MGI:3645395]
ENSMUSG00000109243	Gm45867	predicted gene 45867 [Source:MGI Symbol%3BAcc:MGI:5804982]
ENSMUSG00000108897	Gm44861	predicted gene 44861 [Source:MGI Symbol%3BAcc:MGI:5753437]
ENSMUSG00000110332	Gm19935	predicted gene%2C 19935 [Source:MGI Symbol%3BAcc:MGI:5012120]
ENSMUSG00000110373	Gm42303	predicted gene%2C 42303 [Source:MGI Symbol%3BAcc:MGI:5625188]

ENSMUSG00000087968	Gm25395	predicted gene%2C 25395 [Source:MGI Symbol%3BAcc:MGI:5455172]
ENSMUSG00000115965	Gm30564	predicted gene%2C 30564 [Source:MGI Symbol%3BAcc:MGI:5589723]
ENSMUSG00000095550	Gm21671	predicted gene%2C 21671 [Source:MGI Symbol%3BAcc:MGI:5435026]
ENSMUSG00000048070	Pirt	phosphoinositide-interacting regulator of transient receptor potential channels [Source:MGI Symbol%3BAcc:MGI:24436
ENSMUSG00000099591	Gm17969	predicted gene%2C 17969 [Source:MGI Symbol%3BAcc:MGI:5010154]
ENSMUSG00000065162	Gm23645	predicted gene%2C 23645 [Source:MGI Symbol%3BAcc:MGI:5453422]
ENSMUSG00000097429	Gm26520	predicted gene%2C 26520 [Source:MGI Symbol%3BAcc:MGI:5477014]
ENSMUSG00000104244	2900018N21Rik	RIKEN cDNA 2900018N21 gene [Source:MGI Symbol%3BAcc:MGI:1920114]
ENSMUSG00000077557	Gm23706	predicted gene%2C 23706 [Source:MGI Symbol%3BAcc:MGI:5453483]
ENSMUSG00000104528	Gm43314	predicted gene 43314 [Source:MGI Symbol%3BAcc:MGI:5663451]
ENSMUSG00000108859	Gm44776	predicted gene 44776 [Source:MGI Symbol%3BAcc:MGI:5753352]
ENSMUSG00000084713	Mir1911	microRNA 1911 [Source:MGI Symbol%3BAcc:MGI:5454404]
ENSMUSG00000121158	Gm35162	predicted gene%2C 35162 [Source:NCBI gene (formerly Entrezgene)%3BAcc:10263
ENSMUSG00000120233	-	novel transcript
ENSMUSG00000090665	Gad1-ps	glutamate decarboxylase 1%2C pseudogene [Source:MGI Symbol%3BAcc:MGI:95633
ENSMUSG00000111792	Gm33858	predicted gene%2C 33858 [Source:MGI Symbol%3BAcc:MGI:5593017]
ENSMUSG00000102927	Gm37152	predicted gene%2C 37152 [Source:MGI Symbol%3BAcc:MGI:5610380]
ENSMUSG00000112770	Gm48210	predicted gene%2C 48210 [Source:MGI Symbol%3BAcc:MGI:6097604]
ENSMUSG00000079037	Prnp	prion protein [Source:MGI Symbol%3BAcc:MGI:97769]
ENSMUSG00000065061	Gm22884	predicted gene%2C 22884 [Source:MGI Symbol%3BAcc:MGI:5452661]
ENSMUSG00000097472	Gm26586	predicted gene%2C 26586 [Source:MGI Symbol%3BAcc:MGI:5477080]
ENSMUSG00000080790	Gm7638	predicted gene 7638 [Source:MGI Symbol%3BAcc:MGI:3643470]
ENSMUSG00000041730	Prrxl1	paired related homeobox protein-like 1 [Source:MGI Symbol%3BAcc:MGI:2148204]
ENSMUSG00000028874	Fgr	FGR proto-oncogene%2C Src family tyrosine kinase [Source:MGI Symbol%3BAcc:MGI:95527
ENSMUSG00000115357	Gm49214	predicted gene%2C 49214 [Source:MGI Symbol%3BAcc:MGI:6118668]
ENSMUSG00000038421	Fcrla	Fc receptor-like A [Source:MGI Symbol%3BAcc:MGI:2138647]
ENSMUSG00000110235	Gm5086	predicted gene 5086 [Source:MGI Symbol%3BAcc:MGI:3644417]
ENSMUSG00000109882	Gm18706	predicted gene%2C 18706 [Source:MGI Symbol%3BAcc:MGI:5010891]
ENSMUSG00000110483	Gm45702	predicted gene 45702 [Source:MGI Symbol%3BAcc:MGI:5804817]
ENSMUSG00000110760	Gm39456	predicted gene%2C 39456 [Source:MGI Symbol%3BAcc:MGI:5622341]
ENSMUSG00000110768	Gm18541	predicted gene%2C 18541 [Source:MGI Symbol%3BAcc:MGI:5010726]
ENSMUSG00000113425	Gm48653	predicted gene%2C 48653 [Source:MGI Symbol%3BAcc:MGI:6098263]
ENSMUSG00000113527	Gm48079	predicted gene%2C 48079 [Source:MGI Symbol%3BAcc:MGI:6097416]
ENSMUSG00000115584	Gm5854	predicted gene 5854 [Source:MGI Symbol%3BAcc:MGI:3645469]
ENSMUSG00000115518	Gm10791	predicted gene 10791 [Source:MGI Symbol%3BAcc:MGI:3641949]
ENSMUSG00000115263	Gm5089	predicted gene 5089 [Source:MGI Symbol%3BAcc:MGI:3644731]
ENSMUSG00000115777	Gm32815	predicted gene%2C 32815 [Source:MGI Symbol%3BAcc:MGI:5591974]
ENSMUSG00000116174	Gm10362	predicted gene 10362 [Source:MGI Symbol%3BAcc:MGI:3642619]
ENSMUSG00000116757	Gm4786	predicted gene 4786 [Source:MGI Symbol%3BAcc:MGI:3644879]
ENSMUSG00000117027	Gm49886	predicted gene%2C 49886 [Source:MGI Symbol%3BAcc:MGI:6270570]
ENSMUSG00000117322	6330415G19Rik	RIKEN cDNA 6330415G19 gene [Source:MGI Symbol%3BAcc:MGI:2443182]
ENSMUSG00000117484	Gm5500	predicted pseudogene 5500 [Source:MGI Symbol%3BAcc:MGI:3647082]
ENSMUSG00000117465	Gm49980	predicted gene%2C 49980 [Source:MGI Symbol%3BAcc:MGI:6275257]
ENSMUSG00000117548	Gm41662	predicted gene%2C 41662 [Source:MGI Symbol%3BAcc:MGI:5624547]
ENSMUSG00000117438	Rpsa-ps7	ribosomal protein SA%2C pseudogene 7 [Source:MGI Symbol%3BAcc:MGI:36438
ENSMUSG00000117896	AA387883	expressed sequence AA387883 [Source:MGI Symbol%3BAcc:MGI:3035466]
ENSMUSG00000117789	Gm50388	predicted gene%2C 50388 [Source:MGI Symbol%3BAcc:MGI:6303291]
ENSMUSG00000118154	9330117O12Rik	RIKEN cDNA 9330117O12 gene [Source:MGI Symbol%3BAcc:MGI:5439411]
ENSMUSG00000119968	-	novel transcript%2C antisense to Syvn1
ENSMUSG00000120224	-	novel transcript
ENSMUSG00000120298	-	novel transcript
ENSMUSG00000120310	-	novel transcript

ENSMUSG00000103011	Gm8860	predicted gene 8860 [Source:MGI Symbol%3BAcc:MGI:3648010]
ENSMUSG00000079580	Tmem217	transmembrane protein 217 [Source:MGI Symbol%3BAcc:MGI:3646280]
ENSMUSG00000109431	Gm45161	predicted gene 45161 [Source:MGI Symbol%3BAcc:MGI:5753737]
ENSMUSG00000111817	Gm47154	predicted gene%2C 47154 [Source:MGI Symbol%3BAcc:MGI:6095923]
ENSMUSG00000103476	Strit1	small transmembrane regulator of ion transport 1 [Source:MGI Symbol%3BAcc:MGI:5593461]
ENSMUSG00000084782	Gm11213	predicted gene 11213 [Source:MGI Symbol%3BAcc:MGI:3701774]
ENSMUSG00000112468	Gm47720	predicted gene%2C 47720 [Source:MGI Symbol%3BAcc:MGI:6096849]
ENSMUSG00000107121	1110006O24Rik	RIKEN cDNA 1110006O24 gene [Source:MGI Symbol%3BAcc:MGI:1913373]
ENSMUSG00000070526	Peg12	paternally expressed 12 [Source:MGI Symbol%3BAcc:MGI:1351637]
ENSMUSG00000112058	Gm47593	predicted gene%2C 47593 [Source:MGI Symbol%3BAcc:MGI:6096640]
ENSMUSG00000013611	Snx31	sorting nexin 31 [Source:MGI Symbol%3BAcc:MGI:1913946]
ENSMUSG00000082561	Gm15580	predicted gene 15580 [Source:MGI Symbol%3BAcc:MGI:3783028]
ENSMUSG00000102191	Gm36569	predicted gene%2C 36569 [Source:MGI Symbol%3BAcc:MGI:5595728]
ENSMUSG00000075031	H2bc3	H2B clustered histone 3 [Source:MGI Symbol%3BAcc:MGI:2448377]
ENSMUSG00000086559	Gm14426	predicted gene 14426 [Source:MGI Symbol%3BAcc:MGI:3652025]
ENSMUSG00000082715	Gm11633	predicted gene 11633 [Source:MGI Symbol%3BAcc:MGI:3650676]
ENSMUSG00000120555	-	novel transcript%2C antisense to Hspa12a
ENSMUSG00000113794	Gm36607	predicted gene%2C 36607 [Source:MGI Symbol%3BAcc:MGI:5595766]
ENSMUSG00000041237	Pklr	pyruvate kinase liver and red blood cell [Source:MGI Symbol%3BAcc:MGI:97604
ENSMUSG00000084601	Gm23111	predicted gene%2C 23111 [Source:MGI Symbol%3BAcc:MGI:5452888]
ENSMUSG00000113568	Gm46369	predicted gene%2C 46369 [Source:MGI Symbol%3BAcc:MGI:5826006]
ENSMUSG00000109970	Gm17995	predicted gene%2C 17995 [Source:MGI Symbol%3BAcc:MGI:5010180]
ENSMUSG00000116307	Gm49490	predicted gene%2C 49490 [Source:MGI Symbol%3BAcc:MGI:6155166]
ENSMUSG00000023000	Dhh	desert hedgehog [Source:MGI Symbol%3BAcc:MGI:94891
ENSMUSG00000121070	-	novel transcript%2C antisense to Fbxl18and KO:Fbxl18
ENSMUSG000	-	novel transcript
ENSMUSG00000061272	Gm14173	predicted gene 14173 [Source:MGI Symbol%3BAcc:MGI:3650930]
ENSMUSG00000102894	Gm37851	predicted gene%2C 37851 [Source:MGI Symbol%3BAcc:MGI:5611079]
ENSMUSG00000120369	-	novel transcript
ENSMUSG00000120414	Gm32261	predicted gene%2C 32261 [Source:NCBI gene (formerly Entrezgene)%3BAcc:10263
ENSMUSG00000120630	-	novel transcript
ENSMUSG00000120906	-	novel transcript
ENSMUSG00000121191	-	novel transcript

ENSMUSG00000114853	D030051J21Rik	RIKEN cDNA D030051J21 gene [Source:MGI Symbol%3BAcc:MGI:2685588]
ENSMUSG00000099423	Gm17826	predicted gene%2C 17826 [Source:MGI Symbol%3BAcc:MGI:5010011]
ENSMUSG00000112734	Gm47644	predicted gene%2C 47644 [Source:MGI Symbol%3BAcc:MGI:6096722]
ENSMUSG00000106255	Gm36793	predicted gene%2C 36793 [Source:MGI Symbol%3BAcc:MGI:5595952]
ENSMUSG00000038224	Serpinf2	serine (or cysteine) peptidase inhibitor%2C clade F%2C member 2 [Source:MGI Symbol%3BAcc:MGI:10717
ENSMUSG00000050439	Enthd1	ENTH domain containing 1 [Source:MGI Symbol%3BAcc:MGI:2686088]
ENSMUSG00000046295	Ankle1	ankyrin repeat and LEM domain containing 1 [Source:MGI Symbol%3BAcc:MGI:1918775]
ENSMUSG00000100621	Gm28374	predicted gene 28374 [Source:MGI Symbol%3BAcc:MGI:5579080]
ENSMUSG00000112279	Gm18774	predicted gene%2C 18774 [Source:MGI Symbol%3BAcc:MGI:5010959]
ENSMUSG00000113434	3200001D21Rik	RIKEN cDNA 3200001D21 gene [Source:MGI Symbol%3BAcc:MGI:1919068]
ENSMUSG00000065130	Gm24457	predicted gene%2C 24457 [Source:MGI Symbol%3BAcc:MGI:5454234]
ENSMUSG00000070324	Fbxw22	F-box and WD-40 domain protein 22 [Source:MGI Symbol%3BAcc:MGI:36432
ENSMUSG00000095836	Gm21960	predicted gene%2C 21960 [Source:MGI Symbol%3BAcc:MGI:5439429]
ENSMUSG00000085113	B130011K05Rik	RIKEN cDNA B130011K05 gene [Source:MGI Symbol%3BAcc:MGI:2443995]
ENSMUSG00000106603	Gm43509	predicted gene 43509 [Source:MGI Symbol%3BAcc:MGI:5663646]
ENSMUSG00000087638	Gm11773	predicted gene 11773 [Source:MGI Symbol%3BAcc:MGI:3651310]
ENSMUSG00000107010	Gm43293	predicted gene 43293 [Source:MGI Symbol%3BAcc:MGI:5663430]
ENSMUSG00000081861	Gm14441	predicted gene 14441 [Source:MGI Symbol%3BAcc:MGI:3652321]
ENSMUSG00000112916	Gm18622	predicted gene%2C 18622 [Source:MGI Symbol%3BAcc:MGI:5010807]
ENSMUSG00000118625	-	polycystic kidney disease 2 (Pkd2) pseudogene
ENSMUSG00000101648	Gm29388	predicted gene 29388 [Source:MGI Symbol%3BAcc:MGI:5580094]
ENSMUSG00000088377	Gm25183	predicted gene%2C 25183 [Source:MGI Symbol%3BAcc:MGI:5454960]
ENSMUSG00000089697	Gm15947	predicted gene 15947 [Source:MGI Symbol%3BAcc:MGI:3801748]
ENSMUSG00000102684	Gm37374	predicted gene%2C 37374 [Source:MGI Symbol%3BAcc:MGI:5610602]
ENSMUSG00000072900	Mir540	microRNA 540 [Source:MGI Symbol%3BAcc:MGI:36194
ENSMUSG00000081972	Gm16397	predicted pseudogene 16397 [Source:MGI Symbol%3BAcc:MGI:3648067]
ENSMUSG000	-	novel transcript

ENSMUSG00000027606	Dnajc5b	DnaJ heat shock protein family (Hsp40) member C5 beta [Source:MGI Symbol%3BAcc:MGI:1913576]						
ENSMUSG000	-	novel transcript						
ENSMUSG00000098004	Gm27027	predicted gene%2C 27027 [Source:MGI Symbol%3BAcc:MGI:5504142]						
ENSMUSG00000113844	Gm47943	predicted gene%2C 47943 [Source:MGI Symbol%3BAcc:MGI:6097209]						
ENSMUSG00000104846	Gm43020	predicted gene 43020 [Source:MGI Symbol%3BAcc:MGI:5663157]						
ENSMUSG00000108751	Gm45217	predicted gene 45217 [Source:MGI Symbol%3BAcc:MGI:5753793]						

Categories include "Only MPTP/A53T/MitoPark" (DEGs exclusive to each model), intersections such as "MPTP AND MitoPark NOT A53T" (shared by two models but not the third), and "MPTP AND A53T AND MitoPark" (common to all three); "MPTP" refers to the acute MPTP model, "A53T" to the 16 months A53T-α-synuclein model, and "MitoPark" to the 5 months MitoPark model.
