# Supplementary material for: Neurotoxicity, α-synuclein pathology, and mitochondrial dysfunction: A comparative study of different mouse models of Parkinson’s disease
Source: Neural Regen Res. 2025 Sep 29;21(10):5000–12. doi: 10.4103/NRR.NRR-D-25-00648 (PMC13568611; doi:10.4103/NRR.NRR-D-25-00648)
Supplement: Supplementary file 2 [file NRR-21-5000_Suppl10.pdf]

Additional Table 13 Common GO/KEGG pathways revealed in the intersection analysis between human samples and mouse models

CAU AND PUT AND MPTP acute		CAU		PUT		MPTP acute		CAU AND PUT AND MPTP subacute		CAU		PUT		MPTP subacute		CAU AND PUT AND 16m A53T		CAU		PUT		16m A53T
**ID**	**Description**	**Risk Factor**	**qvalue**	**Risk Factor**	**qvalue**	**Risk Factor**	**qvalue**	**ID**	**Description**	**Risk Factor**	**qvalue**	**Risk Factor**	**qvalue**	**Risk Factor**	**qvalue**	**ID**	**Description**	**Risk Factor**	**qvalue**	**Risk Factor**	**qvalue**	**Risk Factor**
GO:0048167	regulation of synaptic plasticity	3.077486014	5.62612E-15	2.453163571	4.01711E-09	1.836807548	0.05793282	GO:0006874	cellular calcium ion homeostasis	2.141973141	2.72437E-12	1.920833426	2.50618E-09	2.273030162	0.054146981	GO:0007611	learning or memory	2.613600254	5.61682E-13	2.201814845	1.00398E-08	2.83515159
GO:0099003	vesicle-mediated transport in synapse	2.911160254	5.61682E-13	2.58687599	2.0326E-10	2.220982355	0.03151624	GO:0043270	positive regulation of ion transport	2.133551228	2.74241E-07	1.983390039	2.53864E-06	2.438437507	0.087305451	GO:0050890	cognition	2.430575026	2.73452E-12	2.029714893	1.09615E-07	2.542949337
GO:0006836	neurotransmitter transport	2.857103383	5.61682E-13	2.577307986	1.13097E-10	2.51043015	0.01203416	GO:0019932	second-messenger-mediated signaling	2.040604442	5.87104E-07	1.865853676	1.52E-05	2.812620608	0.035014251	GO:0007613	memory	3.084905218	1.85056E-09	2.61073038	8.58076E-07	4.252727385
GO:0007611	learning or memory	2.613600254	5.61682E-13	2.201814845	1.00398E-08	2.29101811	0.01133079	GO:0071248	cellular response to metal ion	2.20504195	9.05695E-06	2.042794006	6.5238E-05	3.164678263	0.085425328	GO:0007200	phospholipase C-activating G protein-coupled receptor signaling pathway	3.274180538	2.073E-09	2.832893494	3.06786E-07	4.519400059
GO:0042391	regulation of membrane potential	2.19278865	2.48785E-12	1.985997957	8.33544E-10	2.313143833	0.00163371	GO:0003012	muscle system process	1.536727098	0.00258949	1.401014443	0.020698407	2.487584309	0.035014251	GO:0043270	positive regulation of ion transport	2.133551228	2.74241E-07	1.983390039	2.53864E-06	2.081121912
GO:0006874	cellular calcium ion homeostasis	2.141973141	2.72437E-12	1.920833426	2.50618E-09	2.603674699	0.00011025	GO:0060249	anatomical structure homeostasis	1.573076015	0.009256357	1.572576309	0.005023352	2.445871767	0.086323093	GO:0010038	response to metal ion	1.965784807	4.79885E-07	1.746809201	6.2087E-05	2.32098003
GO:0050890	cognition	2.430575026	2.73452E-12	2.029714893	1.09615E-07	2.296648992	0.00709822	GO:0098657	import into cell	1.656639777	0.011705844	1.806669301	0.000566834	3.899806651	0.00503361	GO:0019932	second-messenger-mediated signaling	2.040604442	5.87104E-07	1.865853676	1.52E-05	3.323733538
GO:0099504	synaptic vesicle cycle	2.88903822	5.62588E-12	2.61073038	5.2322E-10	2.102820976	0.0629437	GO:0019935	cyclic-nucleotide-mediated signaling	2.159256664	0.013688603	2.302475467	0.002315366	4.05174717	0.081354273	GO:0007631	feeding behavior	2.78971887	2.40583E-06	2.779442673	5.18444E-07	3.5112262
GO:0051480	regulation of cytosolic calcium ion concentration	2.240228789	4.18677E-11	2.035416339	4.95185E-09	2.95126855	0.000043686	GO:0098659	inorganic cation import across plasma membrane	1.898498974	0.021230601	2.024422286	0.003547702	4.610608849	0.021485342	GO:0009914	hormone transport	1.977581276	5.62686E-06	1.92203771	5.76816E-06	3.153173528
GO:0001505	regulation of neurotransmitter levels	2.608341501	1.89284E-10	2.467324063	6.37451E-10	2.391827151	0.01605149	GO:0099587	inorganic ion import across plasma membrane	1.898498974	0.021230601	2.024422286	0.003547702	4.610608849	0.021485342	GO:0007409	axonogenesis	1.771343694	7.7352E-06	1.629581474	0.00015685	2.198967726
GO:0060078	regulation of postsynaptic membrane potential	3.078176962	5.02219E-10	2.492151404	2.294E-06	2.942005309	0.0302825	GO:0098739	import across plasma membrane	1.620591039	0.046086752	1.821262177	0.002406562	3.850220478	0.01474007	GO:0071248	cellular response to metal ion	2.20504195	9.05695E-06	2.042794006	6.5238E-05	2.70094323
GO:0006887	exocytosis	2.158651831	7.95834E-10	1.895887538	5.45037E-07	1.991538618	0.02141541	GO:0062023	collagen-containing extracellular matrix	1.735476563	3.00663E-06	1.719777118	1.35075E-06	3.837205387	1.48756E-05	GO:0046879	hormone secretion	1.945252045	1.62952E-05	1.870249201	3.47999E-05	3.220983711
GO:0007204	positive regulation of cytosolic calcium ion concentration	2.205763731	1.34983E-09	1.984556739	1.55397E-07	2.819421755	0.00030717	GO:0016324	apical plasma membrane	1.590330725	0.000871544	1.518521225	0.002427757	3.222764228	0.001188753	GO:0015833	peptide transport	1.979318609	3.28366E-05	1.854771904	0.000150632	2.674566832
GO:0007269	neurotransmitter secretion	2.904572499	1.39408E-09	2.635937432	2.44827E-08	2.58021817	0.02206256	GO:0045177	apical part of cell	1.528702524	0.001074621	1.410362402	0.008921648	2.71321013	0.003772295	GO:0030072	peptide hormone secretion	2.036571627	4.31696E-05	1.918293481	0.000146073	2.557746671
GO:0099643	signal release from synapse	2.904572499	1.39408E-09	2.635937432	2.44827E-08	2.58021817	0.02206256	GO:0005614	interstitial matrix	3.98138741	0.026397535	3.530068821	0.052290257	11.65882353	0.03541525	GO:0071241	cellular response to inorganic substance	2.02735637	4.73792E-05	1.945643862	8.41231E-05	2.387339989
GO:0034765	regulation of ion transmembrane transport	1.957847009	1.98445E-09	1.840126139	2.44827E-08	2.614131224	0.00010583	GO:0005539	glycosaminoglycan binding	1.903432589	0.000107519	1.674653335	0.003057096	3.126162165	0.043357869	GO:0042886	amide transport	1.873362054	6.41466E-05	1.886639306	1.72256E-05	2.586172272
GO:0007200	phospholipase C-activating G protein-coupled receptor signaling pathway	3.274180538	2.073E-09	2.832893494	3.06786E-07	3.437194322	0.01549871	GO:0008201	heparin binding	2.079380139	0.0001203	1.866042287	0.001396715	3.505648161	0.046264451	GO:0002790	peptide secretion	1.991314479	7.47863E-05	1.875664737	0.000257444	2.680550203
GO:0006813	potassium ion transport	2.396952614	2.073E-09	2.228253007	2.36287E-08	2.678677674	0.00568355	GO:0015293	symporter activity	2.001403384	0.001451848	2.013770635	0.000562993	4.630615286	0.007108361	GO:0007193	adenylate cyclase-inhibiting G protein-coupled receptor signaling pathway	2.757204664	0.000128061	1.939638788	0.042865598	4.018128573
GO:0045055	regulated exocytosis	2.412554081	3.05947E-09	2.279926295	1.14699E-08	2.419209941	0.00939016	GO:0001653	peptide receptor activity	1.871442125	0.004822499	2.13746662	4.16942E-05	3.462368554	0.056705228	GO:0046883	regulation of hormone secretion	1.900800185	0.000314891	1.79247549	0.000948251	3.345386526
GO:0007612	learning	2.842773084	4.00921E-09	2.306445253	1.52E-05	2.552622254	0.02340031	GO:0008509	anion transmembrane transporter activity	1.552603837	0.008704574	1.517714394	0.008576829	2.961818468	0.019224612	GO:0007411	axon guidance	1.876430952	0.000424645	1.701437534	0.003547702	3.538445007

GO:0010959	regulation of metal ion transport	2.001196933	1.23531E-08	1.69924213	5.06534E-05	2.335134259	0.00179218	GO:0015291	secondary active transmembrane transporter activity	1.564733555	0.020738524	1.600131262	0.008576829	3.830781737	0.003774643	GO:0097485	neuron projection guidance	1.868446139	0.000471259	1.694197374	0.003814596	3.524783057	
GO:0051588	regulation of neurotransmitter transport	3.168000308	3.1824E-08	2.734674145	2.75425E-06	3.121912109	0.01138832	GO:0022853	active ion transmembrane transporter activity	1.5472553	0.020738524	1.63535832	0.003499906	3.06048649	0.046264451	GO:0007601	visual perception	1.899548835	0.000549482	1.76134068	0.002315366	3.543939487	
GO:0046928	regulation of neurotransmitter secretion	3.334294012	4.01685E-08	2.962875408	8.72032E-07	3.201239384	0.01450902									GO:0050953	sensory perception of light stimulus	1.865167861	0.000861632	1.765491653	0.002040734	3.458025803	
GO:0050808	synapse organization	1.946165344	4.01685E-08	1.55833811	0.00134632	2.898828106	5.9537E-06									GO:0046942	carboxylic acid transport	1.734370676	0.001413358	1.683874308	0.001750596	2.369166467	
GO:0007218	neuropeptide signaling pathway	2.986971719	1.05126E-07	2.506784633	2.61946E-05	3.413056721	0.02418495									GO:0015711	organic anion transport	1.613461178	0.002233638	1.521961796	0.006279104	2.262900738	
GO:0007188	adenylate cyclase-modulating G protein-coupled receptor signaling pathway	2.240228789	1.26098E-07	1.860145395	0.000157315	2.293986519	0.03245663									GO:1901214	regulation of neuron death	1.660489071	0.002345375	1.65360159	0.001373923	2.32887452	
GO:0043270	positive regulation of ion transport	2.133551228	2.74241E-07	1.983390039	2.53864E-06	2.242273044	0.0129444									GO:0003012	muscle system process	1.536727098	0.00258949	1.401014443	0.020698407	2.123067004	
GO:0010038	response to metal ion	1.965784807	4.79885E-07	1.746809201	6.2087E-05	3.089105575	0.00022283									GO:0043523	regulation of neuron apoptotic	1.818156699	0.002773828	1.807962318	0.001715305	2.419396145	
GO:0071805	potassium ion transmembrane transport	2.250458145	5.86817E-07	2.181569221	5.18444E-07	2.317186221	0.03896382									GO:0090183	regulation of kidney development	3.360343184	0.002982614	3.48369335	0.000700772	8.15106082	
GO:0006816	calcium ion transport	1.85981258	6.20386E-07	1.671421607	6.37297E-05	2.169728916	0.00629147									GO:0019722	calcium-mediated signaling	1.818799611	0.003103822	1.655616642	0.013851766	2.91109315	
GO:0051932	synaptic transmission, GABAergic	3.865492813	7.21097E-07	3.278770212	3.55701E-05	3.499562767	0.05453962									GO:0070997	neuron death	1.599256813	0.003252505	1.57901115	0.002455316	2.084289525	
GO:0008306	associative learning	3.136320305	1.68893E-06	2.488352393	0.000561614	2.531350402	0.07246111									GO:0042220	response to cocaine	2.85986654	0.00330453	2.541296061	0.010403385	5.307667511	
GO:0007631	feeding behavior	2.78971887	2.40583E-06	2.779442673	5.18444E-07	2.670435589	0.04506826									GO:0051047	positive regulation of secretion	1.642834446	0.003973879	1.539460681	0.011692906	2.326082105	
GO:0017157	regulation of exocytosis	2.196302735	5.3056E-06	1.951648936	0.00015685	2.81098483	0.00277684									GO:1904064	positive regulation of cation transmembrane	1.937495169	0.004613442	1.775473059	0.014265727	2.685055329	
GO:0009914	hormone transport	1.977581276	5.62686E-06	1.92203771	5.76816E-06	1.82714014	0.0599974									GO:0051402	neuron apoptotic process	1.710382146	0.004939314	1.68505855	0.003806807	2.153110405	
GO:0060079	excitatory postsynaptic potential	2.829762681	6.39391E-06	2.598363762	3.47999E-05	2.830081194	0.06952887									GO:0035094	response to nicotine	2.743137293	0.005002862	2.925083629	0.000788208	7.362248483	
GO:0051592	response to calcium ion	2.426914522	7.70154E-06	2.433055673	1.60947E-06	2.981306907	0.02018506									GO:0015849	organic acid transport	1.578022795	0.008579146	1.602561541	0.002923354	2.132987878	
GO:0007409	axonogenesis	1.771343694	7.7352E-06	1.629581474	0.00015685	2.464600797	0.00039408									GO:0060249	anatomical structure homeostasis	1.573076015	0.009256357	1.572576309	0.005023352	2.261422362	
GO:1904062	regulation of cation transmembrane	1.858371609	8.60057E-06	1.628739748	0.000948251	2.619620213	0.00068024									GO:0098657	import into cell	1.656639777	0.011705844	1.806669301	0.000566834	2.615132013	
GO:0070588	calcium ion transmembrane transport	1.912042406	9.05695E-06	1.673694348	0.000860957	2.31437751	0.01277946									GO:1903532	positive regulation of secretion by cell	1.602499426	0.012631835	1.540235642	0.016477164	2.200561874	
GO:0071248	cellular response to metal ion	2.20504195	9.05695E-06	2.042794006	6.5238E-05	4.621907749	7.7448E-06									GO:1905330	regulation of morphogenesis of an epithelium	2.343623964	0.012974532	1.960056039	0.065391918	5.471260003	
GO:0015844	monoamine transport	2.914996497	1.16028E-05	2.110602512	0.010758843	3.275062514	0.01927877									GO:0019935	cyclic-nucleotide-mediated signaling	2.159256664	0.013688603	2.302475467	0.002315366	3.458025803	
GO:0051924	regulation of calcium ion transport	1.999248003	1.90806E-05	1.808268831	0.000352302	2.872811805	0.00088091									GO:0009410	response to xenobiotic stimulus	1.46078179	0.018004587	1.530548625	0.002550428	3.33566489	
GO:0015833	peptide transport	1.979318609	3.28366E-05	1.854771904	0.000150632	1.898512801	0.06328796									GO:0055078	sodium ion homeostasis	2.400245131	0.018004587	2.13287348	0.042943753	4.754785479	
GO:0019233	sensory perception of pain	2.624914541	4.49133E-05	2.493379368	7.70465E-05	3.494865368	0.00243822									GO:0007616	long-term memory	2.816287621	0.019936348	2.50257155	0.039904889	5.187038704	
GO:0071241	cellular response to inorganic substance	2.02735637	4.73792E-05	1.945643862	8.41231E-05	3.631345466	0.000049096									GO:0015850	organic hydroxy compound transport	1.589839786	0.023034594	1.509065322	0.036178118	2.685055329	

GO:0032409	regulation of transporter activity	1.850943131	5.65534E-05	1.879725873	9.4238E-06	2.621149026	0.00276109
GO:0007626	locomotory behavior	2.10021449	5.77585E-05	1.866264295	0.001162504	1.965037509	0.07192444
GO:0042886	amide transport	1.873362054	6.41466E-05	1.886639306	1.72256E-05	1.966893068	0.03800562
GO:1903305	regulation of regulated secretory	2.304235326	6.6099E-05	2.218188419	8.61626E-05	3.283914035	0.00163998
GO:2000300	regulation of synaptic vesicle exocytosis	3.200326842	6.86926E-05	2.986022872	0.000149049	3.099612737	0.05228763
GO:0006814	sodium ion transport	1.907343387	0.000136375	1.7268566	0.001763779	2.274715799	0.02736488
GO:0032412	regulation of ion transmembrane transporter activity	1.839883721	0.000305052	1.816591861	0.000186851	2.692364348	0.00289355
GO:0042755	eating behavior	3.44650583	0.000408267	3.266760065	0.000500767	4.01801651	0.03686157
GO:0022898	regulation of transmembrane transporter	1.805312577	0.000422632	1.837552536	8.88282E-05	2.750360597	0.001752
GO:0007411	axon guidance	1.876430952	0.000424645	1.701437534	0.003547702	2.85933268	0.00170757
GO:0097485	neuron projection guidance	1.868446139	0.000471259	1.694197374	0.003814596	2.848292785	0.001752
GO:0051928	positive regulation of calcium ion	2.259054241	0.000550658	2.007410334	0.004021943	2.992729539	0.01412923
GO:2001257	regulation of cation channel activity	1.991314479	0.000607683	1.725257659	0.010318632	2.876988617	0.00911757
GO:0060402	calcium ion transport into cytosol	1.991314479	0.000951968	1.629798059	0.034215115	2.909133742	0.0085599
GO:0010977	negative regulation of neuron projection	2.108450625	0.001209383	1.697934574	0.039754998	4.285884278	0.000065779
GO:1903169	regulation of calcium ion transmembrane transport	2.010461734	0.001472933	1.735466284	0.016550085	3.242123667	0.0027394
GO:1901214	regulation of neuron death	1.660489071	0.002345375	1.65360159	0.001373923	1.992608188	0.02503526
GO:0045926	negative regulation of growth	1.755607868	0.002345375	1.722549248	0.002050695	2.29254376	0.02110017
GO:0043523	regulation of neuron apoptotic process	1.818156699	0.002773828	1.807962318	0.001715305	2.300065988	0.01645935
GO:0071277	cellular response to calcium ion	2.346906351	0.003025866	2.464653799	0.000477795	4.943686137	0.00140419
GO:0071692	protein localization to extracellular	1.597489053	0.003057385	1.508260392	0.008407566	1.923897561	0.03244876
GO:0070997	neuron death	1.599256813	0.003252505	1.57901115	0.002455316	1.882413215	0.03254744
GO:0060401	cytosolic calcium ion transport	1.839345743	0.003366089	1.592545532	0.034935511	2.590721093	0.01645935
GO:0051047	positive regulation of secretion	1.642834446	0.003973879	1.539460681	0.011692906	1.873147265	0.03910584
GO:0009306	protein secretion	1.587362114	0.004165623	1.52429358	0.007209983	1.848891808	0.04841211
GO:0035592	establishment of protein localization to extracellular	1.582839714	0.004495972	1.519950863	0.007634514	1.844269578	0.04907263
GO:1904064	positive regulation of cation transmembrane	1.937495169	0.004613442	1.775473059	0.014265727	2.807884479	0.01421905

GO:0003018	vascular process in circulatory system	1.567308355	0.024297199	1.453273489	0.058539413	2.67077312
GO:0030900	forebrain development	1.453765491	0.029760525	1.376535366	0.056306194	2.150405493
GO:0060541	respiratory system development	1.63327025	0.030388774	1.647460895	0.014765443	2.350680461
GO:0070371	ERK1 and ERK2 cascade	1.471195026	0.035281021	1.568776195	0.004021943	2.288836563
GO:0022600	digestive system process	1.895578206	0.036311985	2.37350536	0.000184052	3.356319161
GO:0007586	digestion	1.766019776	0.036901515	2.208639788	0.000121744	2.936779266
GO:0006721	terpenoid metabolic process	1.920196105	0.037812909	2.193812722	0.002270962	4.258016846
GO:0034308	primary alcohol metabolic process	1.877525081	0.039929229	1.971723039	0.010758843	4.226475981
GO:0006066	alcohol metabolic process	1.43970382	0.042138124	1.544019213	0.00412893	2.315373798
GO:0098739	import across plasma membrane	1.620591039	0.046086752	1.821262177	0.002406562	2.987299777
GO:0070372	regulation of ERK1 and ERK2 cascade	1.469474833	0.046937118	1.536217555	0.010429108	2.275296119
GO:0072210	metanephric nephron development	2.464251668	0.050244042	2.78695468	0.006279104	6.006044815
GO:0009743	response to carbohydrate	1.546352616	0.053500064	1.691198795	0.00504643	2.422249206
GO:0030902	hindbrain development	1.672704163	0.057901375	1.592545532	0.071709027	2.564378685
GO:0015718	monocarboxylic acid transport	1.761547424	0.061331764	2.109782542	0.001715305	3.220983711
GO:1905332	positive regulation of morphogenesis of an epithelium	2.489143099	0.061500034	2.654242553	0.019400611	5.705742574
GO:0046888	negative regulation of hormone	2.090880203	0.066877684	2.123394042	0.03553402	5.705742574
GO:0035270	endocrine system development	1.697143022	0.067784139	2.171652998	0.000287172	3.260424328
GO:0050730	regulation of peptidyl-tyrosine phosphorylation	1.476319355	0.069102086	1.555935289	0.016333064	2.359517606
GO:0090189	regulation of branching involved in ureteric bud	2.986971719	0.070909595	3.412597568	0.012252405	6.846891089
GO:0060993	kidney morphogenesis	1.830724602	0.075018375	2.226138915	0.002281851	3.323733538
GO:0003014	renal system process	1.729299416	0.081129841	1.885909182	0.015420607	4.638815101
GO:0062023	collagen-containing extracellular	1.735476563	3.00663E-06	1.719777118	1.35075E-06	4.754315798
GO:0031225	anchored component of membrane	2.073148474	5.09309E-05	2.113863697	8.01819E-06	3.365258306
GO:0005539	glycosaminoglycan binding	1.903432589	0.000107519	1.674653335	0.003057096	3.852442264
GO:0008201	heparin binding	2.079380139	0.0001203	1.866042287	0.001396715	3.300070028
GO:0005179	hormone activity	2.219141755	0.000328344	2.312668802	3.31541E-05	4.675099206

GO:0010524	positive regulation of calcium ion transport into	2.655085973	0.004830392	2.211868794	0.032352897	3.944961665	0.02305044
GO:0051402	neuron apoptotic	1.710382146	0.004939314	1.68505855	0.003806807	2.183375009	0.0190905
GO:0031644	regulation of nervous system process	1.948025034	0.006203449	1.961831452	0.002711444	2.236833934	0.05738767
GO:1900271	regulation of long-term synaptic	2.787840271	0.006491315	2.477293049	0.016823998	4.567850349	0.01379996
GO:0014074	response to purine-containing	1.939302534	0.008051125	2.258086948	7.70465E-05	2.761473165	0.05402226
GO:1901019	regulation of calcium ion transmembrane transporter	2.166375093	0.008106417	2.01255754	0.014714492	2.92547719	0.06300136
GO:0070374	positive regulation of ERK1 and	1.710720166	0.00824447	1.773516615	0.001904854	2.867483148	0.00329715
GO:0060249	anatomical structure	1.573076015	0.009256357	1.572576309	0.005023352	2.249109242	0.01277946
GO:0034767	positive regulation of ion transmembrane transport	1.814173007	0.011154106	1.807490327	0.006279104	2.698108496	0.01316583
GO:0098657	import into cell	1.656639777	0.011705844	1.806669301	0.000566834	2.531350402	0.01138832
GO:1903532	positive regulation of secretion by cell	1.602499426	0.012631835	1.540235642	0.016477164	1.912708686	0.04513503
GO:0099509	regulation of presynaptic cytosolic calcium ion	4.181760407	0.013061644	3.715939574	0.02231602	13.01837349	0.01050696
GO:0045744	negative regulation of G protein-coupled receptor signaling pathway	2.489143099	0.013159849	2.211868794	0.032352897	3.444014152	0.05745486
GO:0098661	inorganic anion transmembrane	2.090880203	0.015649966	1.857969787	0.045302221	3.381395713	0.03933296
GO:0015698	inorganic anion transport	1.723252915	0.017664778	1.575045031	0.047565915	2.854906468	0.01786402
GO:0007190	activation of adenylate	2.986971719	0.020027482	2.919666808	0.014265727	6.199225473	0.04841211
GO:0046683	response to organophosphor	1.878901565	0.020233533	2.183328551	0.000433409	3.338044486	0.0268291
GO:0098659	inorganic cation import across plasma	1.898498974	0.021230601	2.024422286	0.003547702	2.618638346	0.06560044
GO:0099587	inorganic ion import across plasma	1.898498974	0.021230601	2.024422286	0.003547702	2.618638346	0.06560044
GO:0015850	organic hydroxy compound transport	1.589839786	0.023034594	1.509065322	0.036178118	2.382447437	0.01645935
GO:0003018	vascular process in circulatory system	1.567308355	0.024297199	1.453273489	0.058539413	2.400551141	0.01996486
GO:0031623	receptor internalization	1.903203219	0.024752271	1.761665411	0.043203766	2.850738721	0.02447735
GO:0001656	metanephros development	2.013688799	0.028410774	2.236721252	0.002584498	2.861180988	0.06752691
GO:0030900	forebrain development	1.453765491	0.029760525	1.376535366	0.056306194	1.853537265	0.04788981
GO:0060541	respiratory system	1.63327025	0.030388774	1.647460895	0.014765443	2.112844637	0.04189795
GO:0043271	negative regulation of ion	1.734370676	0.030573205	1.746662841	0.01543544	2.451671091	0.03762956

GO:1901681	sulfur compound binding	1.766304927	0.000489358	1.61444108	0.003713321	2.370472837
GO:0015293	symporter activity	2.001403384	0.001451848	2.013770635	0.000562993	3.530844156
GO:0001664	G protein-coupled receptor binding	1.667836153	0.001854391	1.660000118	0.001022536	3.331008185
GO:0004867	serine-type endopeptidase inhibitor activity	2.138791	0.004227004	1.839384541	0.029878456	3.020833333
GO:0001653	peptide receptor activity	1.871442125	0.004822499	2.13746662	4.16942E-05	2.93339558
GO:0015291	secondary active transmembrane transporter activity	1.564733555	0.020738524	1.600131262	0.008576829	3.477346517
GO:0022853	active ion transmembrane transporter	1.5472553	0.020738524	1.63535832	0.003499906	2.828631453
GO:0004497	monooxygenase activity	1.78059542	0.044639116	1.978367163	0.00701557	3.321781015
GO:0019842	vitamin binding	1.593254782	0.063432719	1.80047864	0.00701557	3.300070028
GO:0042277	peptide binding	1.383239832	0.067437891	1.436041239	0.029878456	2.550054113
map05033	Nicotine addiction	4.052142111	1.90976E-06	2.409900091	0.025025508	#N/A
map04726	Serotonergic synapse	2.373794921	7.14575E-05	1.740931169	0.029690461	#N/A
map04727	GABAergic synapse	2.58800324	7.14575E-05	2.082886855	0.004632246	#N/A
map05031	Amphetamine addiction	2.719973347	0.000173547	1.934367966	0.04084766	#N/A
map04010	MAPK signaling	1.740984795	0.000217401	1.437617009	0.029690461	#N/A
map04921	Oxytocin signaling	1.772639064	0.009938493	1.637094967	0.029080707	#N/A
map04970	Salivary secretion	1.947253646	0.019364108	1.853769301	0.029080707	#N/A
map05143	African trypanosomiasis	2.536191364	0.025284247	2.404889904	0.029690461	#N/A
map04972	Pancreatic secretion	1.83998197	0.028328242	1.817420883	0.026917987	#N/A
map04270	Vascular smooth muscle contraction	1.655234798	0.048240295	1.660091911	0.034974354	#N/A
map04961	Endocrine and other factor-regulated calcium	2.092466631	0.050260824	2.238513872	0.020766197	#N/A
map04925	Aldosterone synthesis and	1.740984795	0.059926229	1.891601327	0.018280604	#N/A
map04022	cGMP-PKG signaling	1.532483622	0.071306825	1.642861416	0.021703965	#N/A
map04964	Proximal tubule bicarbonate	2.596338195	0.080945927	3.22394661	0.008705863	#N/A

GO:0051953	negative regulation of amino transport	2.986971719	0.031281108	3.244074231	0.006349624	4.691305764	0.04392398
GO:0010522	regulation of calcium ion transport into	1.913981878	0.0324783	1.778084817	0.051209914	3.518479323	0.00926387
GO:0044703	multi-organism reproductive process	1.617335906	0.035115659	1.786758401	0.002315366	2.539044476	0.02418495
GO:0070371	ERK1 and ERK2 cascade	1.471195026	0.035281021	1.568776195	0.004021943	2.611135085	0.00122023
GO:0022600	digestive system process	1.895578206	0.036311985	2.37350536	0.000184052	2.552622254	0.07134738
GO:0044706	multi-multicellular organism	1.59866092	0.037552413	1.757033802	0.002687081	2.577895741	0.01710197
GO:0098815	modulation of excitatory postsynaptic	2.527437608	0.042609728	2.450070049	0.03379206	3.616214859	0.04957674
GO:0098739	import across plasma membrane	1.620591039	0.046086752	1.821262177	0.002406562	2.499164196	0.02619857
GO:0140013	meiotic nuclear division	1.620591039	0.046086752	1.524777637	0.071091264	2.148246451	0.06827956
GO:0070372	regulation of ERK1 and ERK2 cascade	1.469474833	0.046937118	1.536217555	0.010429108	2.795356272	0.00059952
GO:0070098	chemokine-mediated signaling	1.913004359	0.053690446	2.057783552	0.011590753	4.199475321	0.01855386
GO:0034764	positive regulation of transmembrane	1.556590896	0.056674056	1.532731615	0.04530491	2.401281013	0.01557174
GO:0030902	hindbrain development	1.672704163	0.057901375	1.592545532	0.071709027	2.437897658	0.03872089
GO:0032760	positive regulation of tumor necrosis factor	1.826982702	0.057901375	1.855392852	0.029254886	5.917442497	4.5937E-06
GO:0030324	lung development	1.601951313	0.061331764	1.690411458	0.015420607	2.206503982	0.04008081
GO:0032411	positive regulation of transporter	1.761547424	0.061331764	1.973667539	0.006226473	3.562241503	0.00339768
GO:0050730	regulation of peptidyl-tyrosine phosphorylation	1.476319355	0.069102086	1.555935289	0.016333064	2.773337712	0.00226145
GO:0032640	tumor necrosis factor production	1.575545522	0.07213884	1.53129378	0.072672028	4.771131647	1.0876E-06
GO:0032680	regulation of tumor necrosis factor	1.575545522	0.07213884	1.53129378	0.072672028	4.691305764	0.00000237
GO:0060993	kidney morphogenesis	1.830724602	0.075018375	2.226138915	0.002281851	2.949146099	0.04345268
GO:0030323	respiratory tube development	1.566935984	0.077248092	1.653462574	0.020983951	2.178807279	0.04347993
GO:1903557	positive regulation of tumor necrosis factor superfamily cytokine	1.758684283	0.079306054	1.786032372	0.043066058	5.811773881	5.1432E-06
GO:0003014	renal system process	1.729299416	0.081129841	1.885909182	0.015420607	2.822411598	0.03601776
GO:0007565	female pregnancy	1.549996135	0.08671581	1.721670845	0.009553029	2.297360028	0.06372775
GO:0002295	T-helper cell lineage commitment	3.162675938	0.08671581	3.747165957	0.011800006	7.657866761	0.03174598
GO:0098793	presynapse	2.545822112	1.84585E-24	2.146741039	4.15382E-16	2.232175344	0.0042421
GO:0097060	synaptic membrane	2.723994818	1.4712E-22	2.16536125	1.45407E-12	1.844956761	0.07354361
GO:0043679	axon terminus	3.720659385	2.44795E-15	2.955261155	6.43676E-10	2.895953802	0.01305926
GO:0044306	neuron projection terminus	3.462881143	8.85793E-15	2.889730756	1.21138E-10	2.595430294	0.02202893
GO:0150034	distal axon	2.530392887	1.42597E-13	2.042210185	6.71677E-08	2.144490918	0.02202893
GO:0042734	presynaptic membrane	2.940101472	3.89244E-11	2.389585048	4.71634E-07	2.565180524	0.0785566
GO:0062023	collagen-containing extracellular	1.735476563	3.00663E-06	1.719777118	1.35075E-06	3.420240699	7.1111E-07
GO:0044304	main axon	3.15900247	5.16715E-06	2.928221022	1.41548E-05	3.485627653	0.03543747

GO:0044298	cell body membrane	3.95570104	2.67073E-05	3.757815197	2.70875E-05	6.193972485	0.00883481
GO:0032809	neuronal cell body membrane	4.066702855	3.93832E-05	3.883075704	3.37894E-05	5.569141927	0.02202893
GO:0032589	neuron projection membrane	2.86934472	0.00012832	2.811882406	7.40413E-05	3.52712322	0.04977132
GO:0098688	parallel fiber to Purkinje cell synapse	4.671494561	0.000773021	3.62420399	0.012117688	7.469202114	0.04977132
GO:0016324	apical plasma membrane	1.590330725	0.000871544	1.518521225	0.002427757	1.949954301	0.04977132
GO:0045177	apical part of cell	1.528702524	0.001074621	1.410362402	0.008921648	1.998944577	0.02202893
GO:0031253	cell projection membrane	1.498598919	0.007616795	1.489085078	0.005386974	2.513075295	0.0070758
GO:0045121	membrane raft	1.450885964	0.020244709	1.500820671	0.005369732	2.907857311	0.000058783
GO:0098857	membrane microdomain	1.446449004	0.021360826	1.496231005	0.005608106	2.900476963	0.000058783
GO:0005216	ion channel activity	2.331540265	1.70386E-16	1.95047859	3.31959E-10	1.950295907	0.05341923
GO:0022836	gated channel activity	2.542959594	1.70386E-16	2.212906619	1.36522E-11	1.98350528	0.07489739
GO:0015267	channel activity	2.214603633	7.40165E-16	1.923282848	1.74559E-10	2.215325745	0.00950597
GO:0022803	passive transmembrane transporter activity	2.210084034	7.40165E-16	1.919357781	1.74559E-10	2.215325745	0.00950597
GO:0046873	metal ion transmembrane transporter activity	2.244564543	1.53996E-14	1.941032772	8.31755E-10	1.826144054	0.07607859
GO:0030594	neurotransmitter receptor activity	3.540566183	4.73325E-13	2.400638186	2.81482E-05	3.106289481	0.05341923
GO:0005230	extracellular ligand-gated ion channel activity	3.828338229	2.14546E-10	2.791394764	2.47643E-05	3.714041771	0.05607674
GO:0015079	potassium ion transmembrane transporter activity	2.835518372	2.14546E-10	2.544603119	1.4244E-08	2.720476456	0.05341923
GO:0022824	transmitter-gated ion channel activity	4.075585073	6.29716E-10	2.873705123	7.70505E-05	3.746621084	0.07558861
GO:0022835	transmitter-gated channel activity	4.075585073	6.29716E-10	2.873705123	7.70505E-05	3.746621084	0.07558861
GO:0005267	potassium channel activity	2.81489642	4.43537E-08	2.720412062	3.69807E-08	2.823899528	0.06478298
GO:0005539	glycosaminoglycan binding	1.903432589	0.000107519	1.674653335	0.003057096	2.749666118	0.01457214
GO:0001664	G protein-coupled receptor binding	1.667836153	0.001854391	1.660000118	0.001022536	2.135574018	0.05341923
GO:0008081	phosphoric diester hydrolase activity	2.282592289	0.002075552	1.781222184	0.060024585	3.476515843	0.05341923
GO:0008528	G protein-coupled peptide receptor activity	1.947311401	0.002332531	2.224120672	1.86307E-05	2.632899474	0.06478298
GO:0001653	peptide receptor activity	1.871442125	0.004822499	2.13746662	4.16942E-05	2.512440021	0.07558861
GO:0008509	anion transmembrane transporter activity	1.552603837	0.008704574	1.517714394	0.008576829	2.319792863	0.03261321
GO:0015291	secondary active transmembrane transporter activity	1.564733555	0.020738524	1.600131262	0.008576829	2.294418367	0.05556944
GO:0022853	active ion transmembrane transporter activity	1.5472553	0.020738524	1.63535832	0.003499906	2.153520018	0.07558861
GO:0140375	immune receptor activity	1.711273655	0.025693233	1.747523385	0.012766182	3.768660032	0.00844513
GO:0004896	cytokine receptor activity	1.800700327	0.046448363	1.858347268	0.027889885	3.082271779	0.06478298
GO:0042277	peptide binding	1.383239832	0.067437891	1.436041239	0.029878456	2.74084366	0.00220274
map04080	Neuroactive ligand-receptor interaction	2.545108159	4.11421E-19	2.273683894	8.13577E-16	2.030753802	0.02258387

This table lists significantly enriched Gene Ontology (GO) pathways. "CAU AND PUT AND MPTP acute" means the common path is from analyzing human CAU/PUT samples and MPTP acute models. Similarly, "CAU AND PUT AND MPTP subacute" is from human CAU/PUT and MPTP subacute models; "CAU AND PUT AND 16m A53T" from human CAU/PUT and 16m A53T models; "CAU
human Putamen samples.

	CAU AND PUT AND 5m MitoPark		CAU		PUT		5m MitoPark	
qvalue	ID	Description	Risk Factor	qvalue	Risk Factor	qvalue	Risk Factor	qvalue
0.005376113	GO:0071867	response to monoamine	2.821028846	1.17395E-06	1.990681914	0.00799955	7.105444861	0.014495608
0.011318664	GO:0071869	response to catecholamine	2.821028846	1.17395E-06	1.990681914	0.00799955	7.105444861	0.014495608
0.001366492	GO:0071868	cellular response to monoamine	2.816287621	1.89145E-06	1.971723039	0.010758843	5.842254663	0.052396014
0.008312577	GO:0071870	cellular response to catecholamine stimulus	2.816287621	1.89145E-06	1.971723039	0.010758843	5.842254663	0.052396014
0.066808064	GO:0019233	sensory perception of	2.624914541	4.49133E-05	2.493379368	7.70465E-05	4.940430118	0.013333886
0.041963032	GO:0043266	regulation of potassium ion	2.443885952	0.000128061	2.026876131	0.005224488	4.694668926	0.048061224
0.000714496	GO:0009266	response to temperature stimulus	2.090880203	0.000130623	1.769495035	0.006091682	4.330141692	0.02039719
0.022142472	GO:0006814	sodium ion transport	1.907343387	0.000136375	1.7268566	0.001763779	2.968242289	0.077047573
0.000400355	GO:0051966	regulation of synaptic transmission,	2.560261474	0.001869123	1.933805288	0.06131123	4.948733362	0.071822374
0.016996687	GO:0042220	response to cocaine	2.85986654	0.00330453	2.541296061	0.010403385	7.336784926	0.067337318
0.057418731	GO:0031644	regulation of nervous system process	1.948025034	0.006203449	1.961831452	0.002711444	3.252389194	0.083410006
0.000342237	GO:0070374	positive regulation of ERK1 and	1.710720166	0.00824447	1.773516615	0.001904854	3.24283739	0.058492519
0.010426744	GO:0098657	import into cell	1.656639777	0.011705844	1.806669301	0.000566834	3.505352798	0.03081824
0.021791996	GO:0008277	regulation of G protein-coupled receptor signaling pathway	1.883090866	0.012443728	1.673326827	0.046279706	4.639437527	0.028821095
0.056703034	GO:0045744	negative regulation of G protein-coupled receptor signaling pathway	2.489143099	0.013159849	2.211868794	0.032352897	8.346078091	0.008083048
0.010309801	GO:0042026	protein refolding	3.360343184	0.015076445	2.654242553	0.07583494	15.02294056	0.017497391
0.013512721	GO:0007422	peripheral nervous system development	2.182787025	0.015649966	1.837552536	0.07583494	5.608564477	0.055167351
0.049642518	GO:0009612	response to mechanical stimulus	1.694103363	0.015956609	1.505391298	0.071306681	3.544738785	0.064960961
0.000669409	GO:0098739	import across plasma	1.620591039	0.046086752	1.821262177	0.002406562	3.303473841	0.078584079
0.0009767	GO:0070098	chemokine-mediated signaling	1.913004359	0.053690446	2.057783552	0.011590753	8.480692253	0.0076407

0.000989907	GO:0032760	positive regulation of tumor necrosis factor	1.826982702	0.057901375	1.855392852	0.029254886	5.736031851	0.013968122
0.010419623	GO:0038034	signal transduction in absence of	2.036571627	0.065970251	1.93035822	0.073330839	5.129784583	0.066750769
0.011575405	GO:0097192	extrinsic apoptotic signaling pathway in absence of	2.036571627	0.065970251	1.93035822	0.073330839	5.129784583	0.066750769
0.038003774	GO:0032640	tumor necrosis factor	1.575545522	0.07213884	1.53129378	0.072672028	5.505789735	0.000826473
0.032983786	GO:0032680	regulation of tumor necrosis factor	1.575545522	0.07213884	1.53129378	0.072672028	5.684355889	0.000706609
0.019592143	GO:1903557	positive regulation of tumor necrosis factor superfamily cytokine production	1.758684283	0.079306054	1.786032372	0.043066058	5.633602711	0.014495608
0.033177749	GO:0045121	membrane raft	1.450885964	0.020244709	1.500820671	0.005369732	3.191006246	0.056400924
0.03489907	GO:0098857	membrane microdomain	1.446449004	0.021360826	1.496231005	0.005608106	3.182907245	0.056400924
0.007618671	GO:0001664	G protein-coupled receptor binding	1.667836153	0.001854391	1.660000118	0.001022536	3.613208643	0.024943487
0.024900088	GO:0140375	immune receptor activity	1.711273655	0.025693233	1.747523385	0.012766182	6.955909687	0.001508274
0.036355302	GO:0008009	chemokine activity	2.317023584	0.027140529	2.399197227	0.012285934	12.81847856	0.006550554
0.043504006	GO:0042379	chemokine receptor binding	1.968089371	0.04361151	1.986940802	0.033350688	6.825423647	0.055024823
0.01683549	GO:0004896	cytokine receptor activity	1.800700327	0.046448363	1.858347268	0.027889885	6.501743763	0.0258834
0.057964623	map04061	Viral protein interaction with cytokine and cytokine	2.303322884	0.000438449	2.224523161	0.000595407	5.921052632	0.004159194
0.059727206	map04060	Cytokine-cytokine receptor	1.532656784	0.010392789	1.58355886	0.002991203	3.302348337	0.005682473
0.021791996	map05144	Malaria	2.047398119	0.071470785	2.076221617	0.047939211	5.639097744	0.058194823
0.062059848								
0.039368333								
0.030475252								
0.037532611								
0.006747321								
0.049322269								
0.000448731								
0.032632456								
0.046293865								
0.021646078								

0.028308688

0.036171744
0.048707088

0.031030773

0.03707703

0.057478863
0.042368348

0.028202323

0.029684996

0.022051442

0.038003774

0.033605596

0.064066498

0.066011716

0.042304783

0.037395363

0.001366492

0.029684996

0.047881308

0.053771802

0.055463998

0.002350615
2.17692E-11

0.011897327

0.000749858
0.016910412

0.001942466

0.050598678

0.018072082

0.000749858

0.071910868

0.057320644

0.002003661

0.019398518

0.021576355

0.03348074

0.010833292

#N/A

#N/A

#N/A

#N/A

#N/A

#N/A

#N/A

#N/A

#N/A

#N/A

#N/A

#N/A

#N/A

#N/A

J AND PUT AND 5m MitoPark" from human CAU/PUT and 5m MitoPark models. "CAU" represented human Caudate samples, and "PUT" represented
