# Supplementary material for: Neurotoxicity, α-synuclein pathology, and mitochondrial dysfunction: A comparative study of different mouse models of Parkinson’s disease
Source: Neural Regen Res. 2025 Sep 29;21(10):5000–12. doi: 10.4103/NRR.NRR-D-25-00648 (PMC13568611; doi:10.4103/NRR.NRR-D-25-00648)
Supplement: Supplementary file 3 [file NRR-21-5000_Suppl2.pdf]

Additional Table 4. GO pathway identified from RNA-Seq data analysis of the striatum in three PD mouse models

MPTP

ONTOLOGY	ID	Description	GeneRatio	BgRatio	Rich Factor	pvalue	p.adjust	qvalue	geneID	Count
BP	GO:0034341	response to interferon-gamma	21/574	139/28814	7.58	6.08166E-13	2.4576E-09	1.93397E-09	Vim/Ifitm3/Gbp3/Gbp9/Gbp5/Gbp4/Irgm1/Parp14/Ccl6/Ccl5/Ccl9/Gch1/Ccl4/Rab7b/Bst2/Nlrc5/Igtp/Ccl3/Gbp2/Ccl12/Gbp6	21
BP	GO:0071346	cellular response to interferon-gamma	18/574	112/28814	8.07	9.39191E-12	1.89764E-08	1.49331E-08	Vim/Gbp3/Gbp9/Gbp5/Gbp4/Irgm1/Parp14/Ccl6/Ccl5/Ccl9/Ccl4/Rab7b/Nlrc5/Igtp/Ccl3/Gbp2/Ccl12/Gbp6	18
BP	GO:0019221	cytokine-mediated signaling pathway	31/574	421/28814	3.7	5.51757E-10	7.43216E-07	5.84862E-07	Osmr/Ifitm3/Plvap/Cxcl12/Il1r1/Oasl2/Hspa1b/Traip/Irf7/Irgm1/Ptprc/Parp14/Ccl6/Ccl5/Ikbke/Il2rg/Ccl9/Ccl4/Il7r/Il12rb2/Nlrc5/Igtp/Ccl3/Csf2rb/Ccl12/Oas2/Cxcr4/Ticam2/Traf1/Trim6/Eda2r	31
BP	GO:0001961	positive regulation of cytokine-mediated signaling pathway	11/574	55/28814	10.04	9.65018E-09	8.73588E-06	6.87456E-06	Il1r1/Hspa1b/Irf7/Irgm1/Parp14/Ikbke/Nlrc5/Igtp/Cxcr4/Ticam2/Trim6	11
BP	GO:0070371	ERK1 and ERK2 cascade	26/574	349/28814	3.74	1.08091E-08	8.73588E-06	6.87456E-06	Cnksr3/Fn1/Tek/Cxcl12/Kdr/Ctsh/Klf4/Icam1/Flt4/Ptprc/Dynlt1b/Ccl6/Ccl5/Prkd2/Nod2/Ccl9/Ccl4/Ccl3/Fgfr4/Ccl12/Avp/Cxcr4/Calcr/Ager/Htr2b/Agt	26
BP	GO:0060326	cell chemotaxis	24/574	314/28814	3.84	2.43804E-08	1.4042E-05	1.10501E-05	Flt1/Eng/Ackr4/Cxcl12/Kdr/Arhgef5/Hspb1/Lef1/Ccl6/Ccl5/Prkd2/Nod2/Ccl9/Ccl4/Rarres2/Ccl3/Cmklr1/Ccr6/Ccl12/Hsd3b7/Ch25h/Cxcr	24
BP	GO:0060760	positive regulation of response to cytokine stimulus	11/574	61/28814	9.05	3.02691E-08	1.4042E-05	1.10501E-05	Il1r1/Hspa1b/Irf7/Irgm1/Parp14/Ikbke/Nlrc5/Igtp/Cxcr4/Ticam2/Trim6	11
BP	GO:0002040	sprouting angiogenesis	15/574	125/28814	6.02	3.11406E-08	1.4042E-05	1.10501E-05	Flt1/Tek/Rnf213/Kdr/Klf4/Lef1/Gata2/Flt4/Mmrn2/Klf2/Ngfr/Clec14a/Dll4/E2f7/Anxa1	15
BP	GO:0071674	mononuclear cell migration	18/574	183/28814	4.94	3.12739E-08	1.4042E-05	1.10501E-05	Flt1/Pecam1/Cxcl12/Arhgef5/Icam1/Ccl6/Ccl5/Ccl9/Ccl4/Rarres2/Ccl3/Cmklr1/Ccr6/Ccl12/Hsd3b7/Ch25h/Ager/Anxa1	18
BP	GO:0035456	response to interferon-beta	11/574	64/28814	8.63	5.10073E-08	2.0612E-05	1.62203E-05	Ifitm3/Gbp3/Irgm1/Ikbke/Bst2/Igtp/Ifit1/Gbp2/Gm4951/Trim6/Gbp6	11
BP	GO:0032103	positive regulation of response to external stimulus	29/574	458/28814	3.18	5.63875E-08	2.07147E-05	1.63011E-05	Fn1/Cxcl12/Kdr/Gbp5/Hspb1/Irf7/Irgm1/Gpr4/Ccl5/Prkd2/Nod2/Lpl/Ikbke/Mmrn2/Trim30a/Nupr1/Rarres2/Nlrc5/Igtp/Ccl3/Cmklr1/Ifi35/Ccr6/Ddx60/Cxcr4/S100a	29
BP	GO:0003018	vascular process in circulatory system	20/574	235/28814	4.27	6.21663E-08	2.09345E-05	1.64741E-05	Cdh5/Abcb1a/Plvap/Kdr/Per2/Adm/Icam1/Gpr4/Gch1/Klf2/Ccl4/Mylk3/Avpr1a/Abcg3/Kcnmb1/Avp/Tacr2/Ager/Htr2b/Agt	20
BP	GO:0001667	ameboidal-type cell migration	28/574	452/28814	3.11	1.50092E-07	4.62852E-05	3.64234E-05	Fn1/Pecam1/Cdh5/Lcn2/Tek/Cxcl12/Kdr/Ctsh/Lama5/Klf4/Hspb1/Fbn2/Gata2/Flt4/Sema3g/Prkd2/Mmrn2/Clec14a/Dll4/Tbx1/Ccr6/Angpt2/Cxcr4/Ager/Pkn3/Htr2b/	28
BP	GO:0001763	morphogenesis of a branching structure	20/574	249/28814	4.03	1.60355E-07	4.62852E-05	3.64234E-05	Flt1/Eng/Tek/Cxcl12/Kdr/Ctsh/Lama5/Tie1/Rasip1/Adm/Lef1/Wnt6/Prdm1/Dll4/Tbx1/Grhl2/Hs3st3a1/Llgl2/Cxcr4/Agt	20
BP	GO:0070374	positive regulation of ERK1 and ERK2 cascade	19/574	227/28814	4.2	1.72084E-07	4.63595E-05	3.64819E-05	Tek/Cxcl12/Kdr/Icam1/Flt4/Ptprc/Ccl6/Ccl5/Prkd2/Nod2/Ccl9/Ccl4/Ccl3/Fgfr4/Ccl12/Cxcr4/Calcr/Ager/Htr2b	19
BP	GO:0070372	regulation of ERK1 and ERK2 cascade	23/574	326/28814	3.54	2.02464E-07	4.87501E-05	3.83631E-05	Cnksr3/Fn1/Tek/Cxcl12/Kdr/Klf4/Icam1/Flt4/Ptprc/Dynlt1b/Ccl6/Ccl5/Prkd2/Nod2/Ccl9/Ccl4/Ccl3/Fgfr4/Ccl12/Cxcr4/Calcr/Ager/Htr2b	23
BP	GO:0061138	morphogenesis of a branching epithelium	19/574	230/28814	4.15	2.11171E-07	4.87501E-05	3.83631E-05	Flt1/Eng/Tek/Cxcl12/Kdr/Ctsh/Lama5/Tie1/Rasip1/Adm/Lef1/Wnt6/Dll4/Tbx1/Grhl2/Hs3st3a1/Llgl2/Cxcr4/Agt	19
BP	GO:0001569	branching involved in blood vessel morphogenesis	9/574	45/28814	10.04	2.1715E-07	4.87501E-05	3.83631E-05	Flt1/Eng/Tek/Cxcl12/Kdr/Lef1/Dll4/Tbx1/Cxcr4	9
BP	GO:0045071	negative regulation of viral genome replication	10/574	59/28814	8.51	2.38029E-07	5.0625E-05	3.98385E-05	Apobec3/Ifitm3/Oasl2/Ccl5/Oas1b/Rsad2/Bst2/Oas2/Trim6/Slpi	10
BP	GO:0001936	regulation of endothelial cell proliferation	16/574	167/28814	4.81	2.60822E-07	5.26991E-05	4.14707E-05	Flt1/Eng/Tek/Cxcl12/Kdr/Tie1/Gata2/Flt4/Prkd2/Mmrn2/Ngfr/Dll4/Lrg1/Ccl12/Ager/Htr2b	16
BP	GO:0009615	response to virus	23/574	337/28814	3.43	3.64052E-07	7.00541E-05	5.51279E-05	Lcn2/Apobec3/Ifitm3/Oasl2/Gbp4/Irf7/Irgm1/Slfn8/Ptprc/Ccl5/Kcnj8/Ikbke/Oas1b/Trim30a/Rsad2/Bst2/Igtp/Bcl3/Ifit1/Ddx60/Oas2/Ticam	23
BP	GO:0048010	vascular endothelial growth factor receptor signaling pathway	9/574	48/28814	9.41	3.89708E-07	7.15822E-05	5.63305E-05	Flt1/Tek/Kdr/Hspb1/Flt4/Tmem204/Prkd2/Mmrn2/Clec14a	9
BP	GO:0030198	extracellular matrix organization	22/574	315/28814	3.51	4.41647E-07	7.75955E-05	6.10626E-05	Fn1/Eng/Myh11/Impg1/Tie1/Hspg2/Egflam/Foxf2/Hmcn1/Col9a1/Mmp23/Fgfr4/Impg2/Bcl3/Col6a4/Kazald1/Adamtsl3/Tnxb/Col14a1/Sox/Serpinb5/Agt	22
BP	GO:0043062	extracellular structure organization	22/574	316/28814	3.49	4.66022E-07	7.84664E-05	6.17479E-05	Fn1/Eng/Myh11/Impg1/Tie1/Hspg2/Egflam/Foxf2/Hmcn1/Col9a1/Mmp23/Fgfr4/Impg2/Bcl3/Col6a4/Kazald1/Adamtsl3/Tnxb/Col14a1/Sox/Serpinb5/Agt	22
BP	GO:0045229	external encapsulating structure organization	22/574	317/28814	3.48	4.91625E-07	7.94663E-05	6.25348E-05	Fn1/Eng/Myh11/Impg1/Tie1/Hspg2/Egflam/Foxf2/Hmcn1/Col9a1/Mmp23/Fgfr4/Impg2/Bcl3/Col6a4/Kazald1/Adamtsl3/Tnxb/Col14a1/Sc	22
BP	GO:0001818	negative regulation of cytokine production	21/574	296/28814	3.56	6.2371E-07	9.6939E-05	7.62846E-05	Flt1/Fn1/Foxj1/Klf4/Gbp4/Serpinb1b/Traip/Lef1/Ptprc/Nod2/Pglyrp1/Fcgr2b/Trim30a/Klf2/Bst2/Fgfr4/Cmklr1/Bcl3/Lilrb4a/Ager/Anxa1	21
BP	GO:0002460	adaptive immune response based on somatic recombination of immune receptors built from immunoglobulin superfamily domains	28/574	486/28814	2.89	6.47817E-07	9.69566E-05	7.62985E-05	C4b/H2-D1/Ctsh/Il1r1/Foxj1/Icam1/Lef1/Irf7/Ly9/Ptprc/Nod2/H2-Q4/Fcgr2b/Il7r/Rsad2/Bcl3/Lilrb4a/Fas/H2-T10/6030468B19Rik/Csf2rb/Ccr6/Hlx/Ager/Il4i1/Igha/	28
BP	GO:0050900	leukocyte migration	24/574	380/28814	3.17	8.23001E-07	0.000115424	9.08311E-05	Flt1/Pecam1/Podxl/Plvap/Cxcl12/Il1r1/Foxj1/Arhgef5/Icam1/Ccl6/Ccl5/Nod2/Ccl9/Ccl4/Rarres2/Ccl3/Cmklr1/Ccr6/Ccl12/Hsd3b7/Ch25h/	24
BP	GO:0001935	endothelial cell proliferation	16/574	183/28814	4.39	8.99724E-07	0.000115424	9.08311E-05	Flt1/Eng/Tek/Cxcl12/Kdr/Tie1/Gata2/Flt4/Prkd2/Mmrn2/Ngfr/Dll4/Lrg1/Ccl12/Ager/Htr2b	16
BP	GO:0045766	positive regulation of angiogenesis	16/574	183/28814	4.39	8.99724E-07	0.000115424	9.08311E-05	Flt1/Eng/Cdh5/Tek/Kdr/Ctsh/Tie1/Klf4/Hspb1/Adm/Gata2/Ccl5/Prkd2/Lrg1/Angpt2/Cxcr4	16
BP	GO:1904018	positive regulation of vasculature development	16/574	183/28814	4.39	8.99724E-07	0.000115424	9.08311E-05	Flt1/Eng/Cdh5/Tek/Kdr/Ctsh/Tie1/Klf4/Hspb1/Adm/Gata2/Ccl5/Prkd2/Lrg1/Angpt2/Cxcr4	16
BP	GO:0048525	negative regulation of viral process	12/574	102/28814	5.91	9.23348E-07	0.000115424	9.08311E-05	Apobec3/Ifitm3/Oasl2/Ccl5/Oas1b/Trim30a/Rsad2/Bst2/Oas2/Ch25h/Trim6/Slpi	12
BP	GO:2000351	regulation of endothelial cell apoptotic process	9/574	53/28814	8.52	9.42587E-07	0.000115424	9.08311E-05	Cdh5/Tek/Kdr/Icam1/Gata2/Ecscr/Cd248/Ccl12/Ager	9

A53T

ONTOLOGY	ID	Description	GeneRatio	BgRatio	Rich Factor	pvalue	p.adjust	qvalue	geneID	Count
BP	GO:0050886	endocrine process	16/556	109/28814	7.61	3.40674E-10	7.46758E-07	5.89252E-07	Pomc/Aqp1/Crh/Pparg/Tspo/Fzd4/Dab2/Edn3/Nkx2-1/Edn1/Oprk1/Agtr1a/Vdr/Retn/Avpr2/Serpinf2	16
BP	GO:0030198	extracellular matrix organization	26/556	315/28814	4.28	6.49121E-10	7.46758E-07	5.89252E-07	Wdr72/Lum/Col13a1/Mfap4/Loxl1/Olfml2a/Col1a2/Loxl2/Cyp1b1/Col4a6/Fbln1/Ddr2/Dmp1/Spint2/Npnt/Adamts16/Col6a5/Lamb3/Col11a1/Col2a1/Adamts3/Spint1/Impg1/Col6a4/Wt1/Serpinf2	26
BP	GO:0043062	extracellular structure organization	26/556	316/28814	4.26	6.94767E-10	7.46758E-07	5.89252E-07	Wdr72/Lum/Col13a1/Mfap4/Loxl1/Olfml2a/Col1a2/Loxl2/Cyp1b1/Col4a6/Fbln1/Ddr2/Dmp1/Spint2/Npnt/Adamts16/Col6a5/Lamb3/Col11a1/Col2a1/Adamts3/Spint1/Impg1/Col6a4/Wt1/Serpinf2	26
BP	GO:0045229	external encapsulating structure organization	26/556	317/28814	4.25	7.43413E-10	7.46758E-07	5.89252E-07	Wdr72/Lum/Col13a1/Mfap4/Loxl1/Olfml2a/Col1a2/Loxl2/Cyp1b1/Col4a6/Fbln1/Ddr2/Dmp1/Spint2/Npnt/Adamts16/Col6a5/Lamb3/Col11a1/Col2a1/Adamts3/Spint1/Impg1/Col6a4/Wt1/Serpinf2	26
BP	GO:0001503	ossification	30/556	421/28814	3.69	1.07937E-09	8.67382E-07	6.84434E-07	Ostn/Omd/Myoc/Prl/Dnm3os/Igf2/Twist1/Ifitm1/Col13a1/Bmp5/Rarbp3/Egr2/Lncpint/Sgms2/Col1a2/Nog/Rad2/Sfrp1/Ddr2/Pparg/Fzd1/Dmp1/Npnt/Tent5a/Ccn4/Phex/Col2a1/Vdr/Fgr/Dhh	30
BP	GO:0060986	endocrine hormone secretion	11/556	67/28814	8.51	6.05778E-08	4.05669E-05	3.20106E-05	Pomc/Aqp1/Crh/Pparg/Tspo/Fzd4/Dab2/Oprk1/Agtr1a/Vdr/Retn	11
BP	GO:0060485	mesenchyme development	22/556	299/28814	3.81	1.0466E-07	6.0075E-05	4.7404E-05	Hnf1a/Bmp2/Twist1/Olfml3/Aldh1a2/Bmp5/Rarbp3/Thbs1/Zic2/Loxl2/Nog/Sox4/Frzb/Sfrp1/Tead2/Dab2/Edn3/Ret/Nkx2-1/Edn1/Meox1/Wt1	22
BP	GO:0030199	collagen fibril organization	10/556	59/28814	8.78	1.77567E-07	8.91832E-05	7.03728E-05	Lum/Col13a1/Loxl1/Col1a2/Loxl2/Cyp1b1/Ddr2/Col11a1/Col2a1/Serpinf2	10
BP	GO:0031214	biomineral tissue development	16/556	176/28814	4.71	3.49514E-07	0.000156039	0.000123127	Itgb6/Ostn/Omd/Wdr72/Dnm3os/Twist1/Lncpint/Sgms2/Col1a2/Ddr2/Dmp1/Fam83h/Tent5a/Phex/Tspear/Fgr	16
BP	GO:0001763	morphogenesis of a branching structure	19/556	249/28814	3.95	4.43172E-07	0.000178066	0.000140509	Col13a1/Cdh1/Prdm1/Nfatc4/Sox9/Bmp5/Rarbp3/Sfrp1/Esr1/Lama1/Spint2/Npnt/Adamts16/Edn1/Spint1/Tdgf1/Agtr1a/Vdr/Wt1	19
BP	GO:0110148	biomineralization	16/556	185/28814	4.48	6.85759E-07	0.000250489	0.000197656	Itgb6/Ostn/Omd/Wdr72/Dnm3os/Twist1/Lncpint/Sgms2/Col1a2/Ddr2/Dmp1/Fam83h/Tent5a/Phex/Tspear/Fgr	16
BP	GO:0060562	epithelial tube morphogenesis	24/556	395/28814	3.15	9.32669E-07	0.000312289	0.000246421	Twist1/Aldh1a2/Bmp5/Zic5/Tcap/Zic2/Nfatc4/Nog/Sox4/Sfrp1/Sfrp2/Fzd1/Lama1/Tead2/Spint2/Npnt/Adamts16/Ret/Edn1/Spint1/Itgax/Agtr1a/Vdr/Wt1	24
BP	GO:0035929	steroid hormone secretion	7/556	30/28814	12.09	1.32851E-06	0.00036766	0.000290113	Pomc/Crh/Tspo/Fzd4/Dab2/Agtr1a/Retn	7
BP	GO:0048145	regulation of fibroblast proliferation	12/556	109/28814	5.71	1.35761E-06	0.00036766	0.000290113	Aqp1/Sphk1/Fosl2/Fbln1/Parp10/Cd74/Sfrp1/Ddr2/Esr1/Pdgfd/Pparg/Tdgf1	12
BP	GO:0140353	lipid export from cell	9/556	57/28814	8.18	1.37255E-06	0.00036766	0.000290113	Pomc/Ptges/Crh/Tspo/Fzd4/Dab2/Edn1/Agtr1a/Retn	9
BP	GO:0051091	positive regulation of DNA-binding transcription factor activity	19/556	272/28814	3.62	1.67701E-06	0.00042114	0.000332313	Cyp1b1/Itgb2l/Neurod1/Zic2/Sphk1/Traf1/Rps6ka4/Arhgef5/Trim34a/Ddr2/Esr1/Pparg/Fzd1/Fzd4/Prkch/Nbrk1/Edn1/Nlrp3/Nod2	19
BP	GO:0048144	fibroblast proliferation	12/556	112/28814	5.55	1.81605E-06	0.000429229	0.000338696	Aqp1/Sphk1/Fosl2/Fbln1/Parp10/Cd74/Sfrp1/Ddr2/Esr1/Pdgfd/Pparg/Tdgf1	12
BP	GO:0033559	unsaturated fatty acid metabolic process	13/556	133/28814	5.07	1.93603E-06	0.000432166	0.000341014	Ptgds/Ptges/Sphk1/Cyp1b1/Alox12b/Cd74/Akr1c14/Cbr1/Cyp2a5/Cyp2e1/Cyp2c23/Cyp2g1/Cyp2j6	13
BP	GO:0061053	somite development	11/556	95/28814	6	2.26315E-06	0.000478596	0.000377651	Rad51b/Dll3/Aldh1a2/Ifitm1/Tcap/Nog/Tbx18/Six4/Frzb/Sfrp1/Meox1	11
BP	GO:0035148	tube formation	15/556	182/28814	4.27	2.78177E-06	0.000558857	0.000440983	Twist1/Edar/Cdh1/Bmp5/Zic5/Tcap/Zic2/Nog/Sox4/Sfrp1/Fzd1/Tead2/Spint2/Ret/Spint1	15
BP	GO:0120254	olefinic compound metabolic process	14/556	162/28814	4.48	3.41836E-06	0.000654046	0.000516095	Rdh16/Abca4/Aldh1a2/Bmp5/Sphk1/Cyp1b1/Alox12b/Akr1c14/Dab2/Cyp2a5/Cyp2e1/Cyp2c23/Cyp2g1/Cyp2j6	14
BP	GO:0006690	icosanoid metabolic process	13/556	142/28814	4.74	4.0307E-06	0.000736152	0.000580884	Ptgds/Ptges/Sphk1/Cyp1b1/Alox12b/Cd74/Akr1c14/Cbr1/Cyp2a5/Cyp2e1/Cyp2c23/Cyp2g1/Cyp2j6	13
BP	GO:0030111	regulation of Wnt signaling pathway	20/556	318/28814	3.26	4.4671E-06	0.000780383	0.000615785	Hnf1a/Lmx1a/Cdh3/Lgr6/Cmah/Wif1/Cdh1/Nfatc4/Mdfic/Nog/Tbx18/Frzb/Rspo3/Sfrp1/Fzd1/Fzd4/Dab2/Nkd2/Ccn4/Peg12	20
BP	GO:0051090	regulation of DNA-binding transcription factor activity	24/556	436/28814	2.85	5.13798E-06	0.000860184	0.000678754	Cyp1b1/Itgb2l/Twist1/Neurod1/Zic2/Sphk1/Traf1/Rps6ka4/Arhgef5/Trim34a/Parp10/Ddr2/Esr1/Pparg/Fzd1/Fzd4/Prkch/Nbrk1/Edn1/Nlrp3/Nod2	24
BP	GO:0035265	organ growth	16/556	217/28814	3.82	5.5349E-06	0.000889569	0.000701942	Ostn/Irf7/Bmp10/Igf2/Fup9/Lepr/Thbs1/Tnp73/Nog/Cdh1/Ccn4/Rarb/Edn1/Myh6/Wt1	16
BP	GO:2000831	regulation of steroid hormone secretion	6/556	26/28814	11.96	8.3289E-06	0.001253702	0.000989272	Pomc/Crh/Tspo/Dab2/Agtr1a/Retn	6
BP	GO:0044060	regulation of endocrine process	8/556	54/28814	7.68	8.71432E-06	0.001253702	0.000989272	Pomc/Crh/Tspo/Dab2/Oprk1/Agtr1a/Vdr/Retn	8
BP	GO:0048146	positive regulation of fibroblast proliferation	9/556	71/28814	6.57	8.94451E-06	0.001253702	0.000989272	Aqp1/Sphk1/Fosl2/Fbln1/Cd74/Ddr2/Esr1/Pdgfd/Tdgf1	9
BP	GO:0016055	Wnt signaling pathway	24/556	451/28814	2.76	9.04862E-06	0.001253702	0.000989272	Hnf1a/Myoc/Lmx1a/Cdh3/Lgr6/Cmah/Wif1/Cdh1/Nfatc4/Mdfic/Nog/Tbx18/Wnt8a/Frzb/Rspo3/Sfrp1/Fzd1/Fzd4/Dab2/Nkd2/Ccn4/Edn1/Wnt8b/Peg12	24
BP	GO:0198738	cell-cell signaling by wnt	24/556	453/28814	2.75	9.7369E-06	0.001304096	0.001029037	Hnf1a/Myoc/Lmx1a/Cdh3/Lgr6/Cmah/Wif1/Cdh1/Nfatc4/Mdfic/Nog/Tbx18/Wnt8a/Frzb/Rspo3/Sfrp1/Fzd1/Fzd4/Dab2/Nkd2/Ccn4/Edn1/Wnt8b/Peg12	24
BP	GO:0061138	morphogenesis of a branching epithelium	16/556	230/28814	3.61	1.15356E-05	0.001495164	0.001179805	Cdh1/Nfatc4/Nog/Sox4/Rspo3/Sfrp1/Esr1/Lama1/Spint2/Npnt/Adamts16/Edn1/Spint1/Agtr1a/Vdr/Wt1	16
BP	GO:0035282	segmentation	11/556	113/28814	5.04	1.23365E-05	0.001548998	0.001222284	Dll3/Aldh1a2/Ifitm1/Egr2/Tcap/Nog/Tbx18/Sfrp1/Tdgf1/Meox1/Wt1	11
BP	GO:0046879	hormone secretion	21/556	372/28814	2.93	1.35934E-05	0.001655094	0.001306003	Pomc/Hnf1a/Ttpg2b/Aqp1/Neurod1/Lepr/Cartpt/Crh/Sfrp1/Pparg/Tspo/Fzd4/Dab2/Edn3/Slc9b2/Pde1c/Edn1/Oprk1/Agtr1a/Vdr/Retn	21

MitoPark

ONTOLOGY	ID	Description	GeneRatio	BgRatio	Rich Factor	pvalue	p.adjust	qvalue	geneID	Count
BP	GO:0009615	response to virus	26/305	337/28814	7.29	3.83192E-15	1.20322E-11	1.03502E-11	Lcn2/Oasl1/Isg20/Oas1a/Ifi207/Mx1/Ifit1/Ifi204/Oas2/Isg15/Ifi27l2a/Irf7/Dhx58/Ifitm3/Trim34a/Rtp4/Trim30a/Slfn8/Ddx60/Dtx3l/Eif2ak2/Oasl2/Ifi27/Ddx58/Irak3/Gbp4	26
BP	GO:0051607	defense response to virus	23/305	281/28814	7.73	4.4604E-14	5.03523E-11	4.33135E-11	Oasl1/Isg20/Oas1a/Ifi207/Mx1/Ifit1/Ifi204/Oas2/Isg15/Irf7/Dhx58/Ifitm3/Trim34a/Rtp4/Trim30a/Slfn8/Ddx60/Dtx3l/Eif2ak2/Oasl2/Ifi27/Ddx58/Gbp4	23
BP	GO:0140546	defense response to symbiont	23/305	282/28814	7.71	4.81073E-14	5.03523E-11	4.33135E-11	Oasl1/Isg20/Oas1a/Ifi207/Mx1/Ifit1/Ifi204/Oas2/Isg15/Irf7/Dhx58/Ifitm3/Trim34a/Rtp4/Trim30a/Slfn8/Ddx60/Dtx3l/Eif2ak2/Oasl2/Ifi27/Ddx58/Gbp4	23
BP	GO:0045071	negative regulation of viral genome replication	12/305	59/28814	19.21	1.14477E-12	8.98647E-10	7.73023E-10	Ltf/Oasl1/Isg20/Oas1a/Ifi207/Ifi204/Oas2/Isg15/Ifitm3/Eif2ak2/Oasl2/Parp10	12
BP	GO:0048525	negative regulation of viral process	14/305	102/28814	12.97	4.24479E-12	2.66573E-09	2.29308E-09	Ltf/Oasl1/Isg20/Ch25h/Oas1a/Ifi207/Ifi204/Oas2/Isg15/Ifitm3/Trim30a/Eif2ak2/Oasl2/Parp10	14
BP	GO:0050792	regulation of viral process	16/305	171/28814	8.84	4.75659E-11	2.48928E-08	2.1413E-08	Ltf/Oasl1/Isg20/Ch25h/Oas1a/Ifi207/Ifi204/Oas2/Isg15/Ifitm3/Trim34a/Trim30a/Eif2ak2/Oasl2/Parp10/Dynlt1b	16
BP	GO:1903900	regulation of viral life cycle	15/305	147/28814	9.64	5.60893E-11	2.51601E-08	2.16429E-08	Ltf/Oasl1/Isg20/Ch25h/Oas1a/Ifi207/Ifi204/Oas2/Isg15/Ifitm3/Trim34a/Trim30a/Eif2ak2/Oasl2/Parp10	15
BP	GO:0019079	viral genome replication	14/305	128/28814	10.33	9.70727E-11	3.8101E-08	3.27748E-08	Ltf/Oasl1/Isg20/Oas1a/Ifi207/Ifi204/Ccl12/Oas2/Isg15/Ifitm3/Eif2ak2/Oasl2/Ifi27/Parp10	14
BP	GO:0045069	regulation of viral genome replication	12/305	91/28814	12.46	2.38673E-10	8.32703E-08	7.16298E-08	Ltf/Oasl1/Isg20/Oas1a/Ifi207/Ifi204/Oas2/Isg15/Ifitm3/Eif2ak2/Oasl2/Parp10	12
BP	GO:0019058	viral life cycle	18/305	261/28814	6.52	4.52576E-10	1.42109E-07	1.22243E-07	Ltf/Oasl1/Isg20/Ch25h/Oas1a/Ifi207/Ifi204/Siglec1/Ccl12/Oas2/Isg15/Ifitm3/Trim34a/Trim30a/Eif2ak2/Oasl2/Ifi27/Parp10	18
BP	GO:0019221	cytokine-mediated signaling pathway	22/305	421/28814	4.94	1.02251E-09	2.91879E-07	2.51077E-07	Cxcl1/Oasl1/Cxcl5/Oas1a/Hspa1b/Il2rg/Sigirr/Ccl12/Oas2/Isg15/Irf7/Ifitm3/Il1r1/Oasl2/Ifi27/Casp1/Irak3/Il7r/Ccl17/Il27ra/Ccl22/Il13ra2	22
BP	GO:0016032	viral process	19/305	355/28814	5.06	9.87733E-09	2.58457E-06	2.22327E-06	Ltf/Oasl1/Isg20/Ch25h/Oas1a/Ifi207/Ifi204/Siglec1/Ccl12/Oas2/Isg15/Ifitm3/Trim34a/Trim30a/Eif2ak2/Oasl2/Ifi27/Parp10/Dynlt1b	19
BP	GO:0002831	regulation of response to biotic stimulus	19/305	371/28814	4.84	2.00337E-08	4.8389E-06	4.16246E-06	Ltf/Cxcl1/Oasl1/Cxcl5/Oas1a/Ifi207/Ifi209/Ifi204/Isg15/Irf7/Dhx58/Nlrp10/Trim30a/Ddx60/Dtx3l/Ifi203/Ddx58/Irak3/Gbp4	19
BP	GO:0002683	negative regulation of immune system process	20/305	448/28814	4.22	8.11208E-08	1.81942E-05	1.56508E-05	Slfn1/Il2ra/Oas1a/Ifi207/Ifi204/Ccl12/Isg15/Hspb1/Dhx58/Fas/Fcgr2b/Pglyrp1/Runx1/Irak3/Il7r/Fstl3/Il27ra/Il13ra2/Cst7/Gpx2	20
BP	GO:0035456	response to interferon-beta	8/305	64/28814	11.81	3.80578E-07	7.96677E-05	6.85308E-05	Oas1a/Ifi207/Gm5431/Ifit1/Ifi209/Ifi204/Ifitm3/Ifi203	8
BP	GO:0002221	pattern recognition receptor signaling pathway	12/305	177/28814	6.4	4.54413E-07	8.91785E-05	7.6712E-05	Ltf/Oasl1/Oas1a/Hspa1b/Irf7/Dhx58/Trim30a/Ddx60/Ddx58/Casp1/Irak3/Cd36	12
BP	GO:0032103	positive regulation of response to external stimulus	19/305	458/28814	3.92	5.25461E-07	9.70557E-05	8.34881E-05	S100a9/S100a8/Cxcl1/Lgals2/Oasl1/Ifi207/Ifi209/Ifi204/Hspb1/Irf7/Dhx58/Nlrp10/Trim30a/Ptgs2/Oprm1/Ddx60/Ifi203/Casp1/Dapk2	19
BP	GO:0050777	negative regulation of immune response	12/305	189/28814	6	9.14407E-07	0.000159513	0.000137214	Oas1a/Ifi207/Ifi204/Isg15/Dhx58/Fcgr2b/Pglyrp1/Irak3/Il7r/Il27ra/Il13ra2/Gpx2	12
BP	GO:0062207	regulation of pattern recognition receptor signaling pathway	9/305	101/28814	8.42	1.32058E-06	0.000208123	0.000179029	Ltf/Oasl1/Hspa1b/Irf7/Dhx58/Trim30a/Ddx60/Irak3/Cd36	9
BP	GO:1990266	neutrophil migration	10/305	130/28814	7.27	1.32563E-06	0.000208123	0.000179029	S100a9/S100a8/Cxcl1/Cxcl5/Bst1/Ccl12/Il1r1/Dapk2/Ccl17/Ccl22	10
BP	GO:0030593	neutrophil chemotaxis	9/305	103/28814	8.25	1.55802E-06	0.000232961	0.000200395	S100a9/S100a8/Cxcl1/Cxcl5/Bst1/Ccl12/Dapk2/Ccl17/Ccl22	9
BP	GO:0035458	cellular response to interferon-beta	7/305	56/28814	11.81	2.06756E-06	0.000295098	0.000253845	Oas1a/Ifi207/Gm5431/Ifit1/Ifi209/Ifi204/Ifi203	7
BP	GO:0034340	response to type I interferon	7/305	62/28814	10.67	4.15303E-06	0.000566978	0.000487719	Ch25h/Oas1a/Oas2/Isg15/Irf7/Ifitm3/Ifi27	7
BP	GO:0043903	regulation of biological process involved in symbiotic interaction	7/305	67/28814	9.87	7.01965E-06	0.000910483	0.000783205	Ltf/Cxcl1/Ch25h/Cxcl5/Ifitm3/Trim34a/Trim30a	7
BP	GO:0002833	positive regulation of response to biotic stimulus	11/305	193/28814	5.38	7.35888E-06	0.000910483	0.000783205	Cxcl1/Oasl1/Ifi207/Ifi209/Ifi204/Irf7/Dhx58/Nlrp10/Trim30a/Ddx60/Ifi203	11
BP	GO:0044403	biological process involved in symbiotic interaction	13/305	272/28814	4.52	7.53903E-06	0.000910483	0.000783205	Ltf/Cxcl1/Ch25h/Cxcl5/Siglec1/Ccl12/Ifitm3/Trim34a/Trim30a/Pglyrp1/Ifi27/Dynlt1b/Gpx2	13
BP	GO:0097530	granulocyte migration	10/305	159/28814	5.94	8.10424E-06	0.000942493	0.00081074	S100a9/S100a8/Cxcl1/Cxcl5/Bst1/Ccl12/Il1r1/Dapk2/Ccl17/Ccl22	10
BP	GO:0071621	granulocyte chemotaxis	9/305	128/28814	6.64	9.39755E-06	0.001053868	0.000906546	S100a9/S100a8/Cxcl1/Cxcl5/Bst1/Ccl12/Dapk2/Ccl17/Ccl22	9
BP	GO:0031348	negative regulation of defense response	12/305	241/28814	4.7	1.12228E-05	0.00118349	0.001018048	Il2ra/Oas1a/Ifi207/Ifi204/Isg15/Dhx58/Fcgr2b/Pglyrp1/Adrb2/Irak3/Cst7/Gpx2	12
BP	GO:0032728	positive regulation of interferon-beta production	6/305	48/28814	11.81	1.13072E-05	0.00118349	0.001018048	Oas1a/Oas2/Isg15/Irf7/Dhx58/Ddx58	6
BP	GO:0050900	leukocyte migration	15/305	380/28814	3.73	1.50694E-05	0.001503765	0.00129355	Sele/Selp/S100a9/S100a8/Cxcl1/Ch25h/Cxcl5/Bst1/Ccl12/Il1r1/Gpr183/Dapk2/Ccl17/Il27ra/Ccl22	15
BP	GO:0060700	regulation of ribonuclease activity	4/305	15/28814	25.19	1.5325E-05	0.001503765	0.00129355	Oasl1/Oas1a/Oas2/Oasl2	4
BP	GO:0007159	leukocyte cell-cell adhesion	15/305	383/28814	3.7	1.65194E-05	0.001571842	0.001352111	Sele/Selp/Slfn1/S100a9/S100a8/Il2ra/Il2rg/Hspb1/Runx1/Nr4a3/Il7r/H2-DMb2/Skap1/Il27ra/Icos	15

BP	GO:0048247	lymphocyte chemotaxis	9/574	54/28814	8.37	1.11128E-06	0.000129947	0.00010226	Cxcl12/Ccl6/Ccl5/Ccl9/Ccl4/Ccl3/Ccl12/Hsd3b7/Ch25h	9
BP	GO:0045765	regulation of angiogenesis	21/574	307/28814	3.43	1.1255E-06	0.000129947	0.00010226	Flt1/Eng/Cdh5/Tek/Kdr/Ctsh/Tie1/Klf4/Hspb1/Adm/Gata2/Gpr4/Ccl5/Prkd2/Klf2/Ngfr/Ecscr/Lrg1/Angpt2/Cxcr4/Agt	21
BP	GO:1901342	regulation of vasculature development	21/574	311/28814	3.39	1.3849E-06	0.000155455	0.000122333	Flt1/Eng/Cdh5/Tek/Kdr/Ctsh/Tie1/Klf4/Hspb1/Adm/Gata2/Gpr4/Ccl5/Prkd2/Klf2/Ngfr/Ecscr/Lrg1/Angpt2/Cxcr4/Agt	21
BP	GO:0072577	endothelial cell apoptotic process	9/574	56/28814	8.07	1.52814E-06	0.00016588	0.000130537	Cdh5/Tek/Kdr/Icam1/Gata2/Ecscr/Cd248/Ccl12/Ager	9
BP	GO:0060759	regulation of response to cytokine stimulus	14/574	147/28814	4.78	1.55987E-06	0.00016588	0.000130537	Il1r1/Klf4/Hspa1b/Traip/Irf7/Irgm1/Ptprc/Parp14/Ikbke/Nlrc5/Igtp/Cxcr4/Ticam2/Trim6	14
BP	GO:0090132	epithelium migration	21/574	315/28814	3.35	1.69779E-06	0.000171746	0.000135153	Pecam1/Cdh5/Lcn2/Tek/Kdr/Ctsh/Klf4/Hspb1/Gata2/Flt4/Prkd2/Mmrn2/Clec14a/Dll4/Grhl2/Ccr6/Angpt2/Ager/Pkn3/Agt/Anxa1	21
BP	GO:0048754	branching morphogenesis of an epithelial tube	16/574	192/28814	4.18	1.70004E-06	0.000171746	0.000135153	Flt1/Eng/Tek/Cxcl12/Kdr/Ctsh/Lama5/Tie1/Rasip1/Lef1/Wnt6/Dll4/Tbx1/Hs3st3a1/Cxcr4/Agt	16
BP	GO:0045785	positive regulation of cell adhesion	27/574	482/28814	2.81	1.74788E-06	0.000172272	0.000135567	Fn1/Podxl/Tek/Cxcl12/Kdr/Rasal3/Icam1/Lef1/Piezo1/Ptprc/Egflam/Ccl5/Prkd2/Myoc/Il2rg/Il7r/Il12rb2/Rag1/Lilrb4a/Hlx/Olfm4/Ager/Il4i1/H2-DMb1/Tnfsf9/Slc4a1/Anxa	27
BP	GO:0090130	tissue migration	21/574	317/28814	3.33	1.87726E-06	0.000177223	0.000139463	Pecam1/Cdh5/Lcn2/Tek/Kdr/Ctsh/Klf4/Hspb1/Gata2/Flt4/Prkd2/Mmrn2/Clec14a/Dll4/Grhl2/Ccr6/Angpt2/Ager/Pkn3/Agt/Anxa1	21
BP	GO:0050792	regulation of viral process	15/574	171/28814	4.4	1.88582E-06	0.000177223	0.000139463	Apobec3/Ifitm3/Top2a/Oasl2/Dynlt1b/Ccl5/Oas1b/Trim30a/Rsad2/Bst2/Oas2/Ch25h/Ccr4/Trim6/Slpi	15
BP	GO:0045069	regulation of viral genome replication	11/574	91/28814	6.07	1.99936E-06	0.000183623	0.0001445	Apobec3/Ifitm3/Top2a/Oasl2/Ccl5/Oas1b/Rsad2/Bst2/Oas2/Trim6/Slpi	11
BP	GO:0001945	lymph vessel development	7/574	31/28814	11.34	2.08387E-06	0.000187132	0.00014726	Kdr/Tie1/Flt4/Tmem204/Sox18/Clec14a/Tbx1	7
BP	GO:1903706	regulation of hemopoiesis	24/574	402/28814	3	2.20602E-06	0.000193794	0.000152503	Foxj1/Hspb1/Hspa1b/Lef1/Gata2/Ptprc/Ccl5/Pglyrp1/Il2rg/H4c17/Il7r/Rab7b/Rag1/Prdm1/Ccl3/Lilrb4a/Fas/Ctla2a/Hlx/Ager/Il4i1/Tnfsf9/Tal1/Anxa1	24
BP	GO:1902105	regulation of leukocyte differentiation	21/574	323/28814	3.26	2.52437E-06	0.000217042	0.000170798	Foxj1/Hspb1/Lef1/Gata2/Ptprc/Ccl5/Pglyrp1/Il2rg/Il7r/Rag1/Prdm1/Ccl3/Lilrb4a/Fas/Ctla2a/Hlx/Ager/Il4i1/Tnfsf9/Tal1/Anxa1	21
BP	GO:0003158	endothelium development	13/574	133/28814	4.91	2.74333E-06	0.000225754	0.000177653	Pecam1/Cdh5/Lcn2/Apold1/Kdr/Tie1/Robo4/Icam1/Sox18/Dll4/Cldn3/Cxcr4/Agt	13
BP	GO:0002548	monocyte chemotaxis	9/574	60/28814	7.53	2.77842E-06	0.000225754	0.000177653	Flt1/Cxcl12/Ccl6/Ccl5/Ccl9/Ccl4/Ccl3/Ccl12/Anxa1	9
BP	GO:0002697	regulation of immune effector process	25/574	436/28814	2.88	2.79329E-06	0.000225754	0.000177653	H2-D1/Tek/Il1r1/Foxj1/Gata2/Ptprc/Nod2/H2-Q4/Pglyrp1/Tap1/Fcgr2b/Il7r/Rsad2/Bst2/Nppc/Lilrb4a/H2-T10/6030468B19Rik/Hlx/Ddx60/Ager/Il4i1/H2-	25
BP	GO:0045619	regulation of lymphocyte differentiation	16/574	201/28814	4	3.09216E-06	0.000245008	0.000192805	Foxj1/Hspb1/Lef1/Ptprc/Pglyrp1/Il2rg/Il7r/Rag1/Prdm1/Lilrb4a/Fas/Ctla2a/Hlx/Il4i1/Tnfsf9/Anxa1	16
BP	GO:0007159	leukocyte cell-cell adhesion	23/574	383/28814	3.01	3.24751E-06	0.000247157	0.000194496	Pecam1/Cxcl12/Foxj1/Rasal3/Klf4/Hspb1/Icam1/Lef1/Ptprc/Ccl5/Il2rg/Il7r/Il12rb2/Rag1/Lilrb4a/Hlx/S100a9/Ager/Il4i1/H2-DMb1/Tnfsf9/Slc4a1/Anxa	23
BP	GO:0030217	T cell differentiation	20/574	302/28814	3.32	3.30276E-06	0.000247157	0.000194496	Foxj1/Hspb1/Lef1/Ly9/Ptprc/Il2rg/Il7r/Rag1/Prdm1/Rsad2/Dll4/Bcl3/Lilrb4a/Fas/Ctla2a/Ccr6/Hlx/Il4i1/Tnfsf9/Anxa1	20
BP	GO:0031349	positive regulation of defense response	20/574	302/28814	3.32	3.30276E-06	0.000247157	0.000194496	Gbp5/Irf7/Irgm1/Gpr4/Ccl5/Crh/Nod2/Lpl/Ikbke/Mmrn2/Trim30a/Nupr1/Nlrc5/Igtp/Ccl3/Ifi35/S100a9/Ager/Trim6/Lgals2	20
BP	GO:0030595	leukocyte chemotaxis	17/574	228/28814	3.74	3.72573E-06	0.000268852	0.000211568	Flt1/Cxcl12/Arhgef5/Ccl6/Ccl5/Nod2/Ccl9/Ccl4/Rarres2/Ccl3/Cmklr1/Ccr6/Ccl12/Hsd3b7/Ch25h/S100a9/Anxa1	17
BP	GO:0043542	endothelial cell migration	17/574	228/28814	3.74	3.72573E-06	0.000268852	0.000211568	Pecam1/Cdh5/Lcn2/Tek/Kdr/Klf4/Hspb1/Gata2/Flt4/Prkd2/Mmrn2/Clec14a/Dll4/Angpt2/Ager/Agt/Anxa1	17
BP	GO:0001959	regulation of cytokine-mediated signaling pathway	13/574	137/28814	4.76	3.82027E-06	0.000270837	0.000213131	Il1r1/Hspa1b/Traip/Irf7/Irgm1/Ptprc/Parp14/Ikbke/Nlrc5/Igtp/Cxcr4/Ticam2/Trim6	13
BP	GO:0097529	myeloid leukocyte migration	17/574	230/28814	3.71	4.18965E-06	0.000291903	0.000229708	Flt1/Pecam1/Cxcl12/Il1r1/Arhgef5/Ccl6/Ccl5/Nod2/Ccl9/Ccl4/Rarres2/Ccl3/Cmklr1/Ccl12/S100a9/Ager/Anxa1	17
BP	GO:0010631	epithelial cell migration	20/574	313/28814	3.21	5.6477E-06	0.00038682	0.000304402	Pecam1/Cdh5/Lcn2/Tek/Kdr/Ctsh/Klf4/Hspb1/Gata2/Flt4/Prkd2/Mmrn2/Clec14a/Dll4/Ccr6/Angpt2/Ager/Pkn3/Agt/Anxa1	20
BP	GO:0002819	regulation of adaptive immune response	17/574	236/28814	3.62	5.90919E-06	0.000397984	0.000313187	H2-D1/Il1r1/Foxj1/Irf7/Ptprc/Nod2/H2-Q4/Fcgr2b/Il7r/Rsad2/Lilrb4a/H2-T10/6030468B19Rik/Hlx/A	17
BP	GO:1903037	regulation of leukocyte cell-cell adhesion	21/574	343/28814	3.07	6.41858E-06	0.000425205	0.000334608	Cxcl12/Foxj1/Rasal3/Klf4/Hspb1/Icam1/Lef1/Ptprc/Ccl5/Il2rg/Il7r/Il12rb2/Rag1/Lilrb4a/Hlx/Ager/Il4i1/H2-DMb1/Tnfsf9/Slc4a1/Anxa1	21
BP	GO:0045580	regulation of T cell differentiation	14/574	167/28814	4.21	7.0025E-06	0.000456405	0.00035916	Foxj1/Hspb1/Lef1/Ptprc/Il2rg/Il7r/Rag1/Prdm1/Lilrb4a/Ctla2a/Hlx/Il4i1/Tnfsf9/Anxa1	14
BP	GO:0002456	T cell mediated immunity	13/574	147/28814	4.44	8.30347E-06	0.000524286	0.000412579	H2-D1/Ctsh/Il1r1/Icam1/Ptprc/Nod2/H2-Q4/Il7r/Rsad2/Lilrb4a/H2-T10/Ager/Il4i1	13
BP	GO:1903900	regulation of viral life cycle	13/574	147/28814	4.44	8.30347E-06	0.000524286	0.000412579	Apobec3/Ifitm3/Top2a/Oasl2/Ccl5/Oas1b/Trim30a/Rsad2/Bst2/Oas2/Ch25h/Trim6/Slpi	13
BP	GO:1903131	mononuclear cell differentiation	26/574	495/28814	2.64	8.55293E-06	0.000531729	0.000418436	Foxj1/Hspb1/Lef1/Ly9/Ptprc/Mfng/Pglyrp1/Il2rg/Cmtm7/Il7r/Rag1/Prdm1/Rsad2/Dll4/Bcl3/Lilrb4a/Fas/Ctla2a/6030468B19Rik/Ccr6/Hlx/Batf2/Ager/Il4i1/Tnfsf9/Anx	26
BP	GO:0042832	defense response to protozoan	7/574	38/28814	9.25	8.86366E-06	0.000542698	0.000427067	Gbp3/Gbp9/Gbp4/Bcl3/Gbp2/Batf2/Gbp6	7
BP	GO:0050727	regulation of inflammatory response	21/574	351/28814	3	9.12281E-06	0.000550228	0.000432993	Cdh5/Uaca/Il1r1/Cst7/Wfdc1/Gpr4/Ccl5/Nod2/Lpl/Pglyrp1/Fcgr2b/Nupr1/Bcl6b/Ccl3/Ifi35/Ctla2a/S100a9/Ager/Lgals2/Agt/Anxa1	21
BP	GO:0002822	regulation of adaptive immune response based on somatic recombination of immune receptors built from immunoglobulin superfamily domains	16/574	220/28814	3.65	9.79396E-06	0.000581673	0.000457738	H2-D1/Il1r1/Foxj1/Ptprc/Nod2/H2-Q4/Fcgr2b/Il7r/Rsad2/Lilrb4a/H2-T10/6030468B19Rik/Hlx/A	16
BP	GO:0022409	positive regulation of cell-cell adhesion	19/574	298/28814	3.2	9.93205E-06	0.000581673	0.000457738	Podxl/Rasal3/Icam1/Lef1/Piezo1/Ptprc/Ccl5/Il2rg/Il7r/Il12rb2/Rag1/Lilrb4a/Hlx/Ager/Il4i1/H2-DMb1/Tnfsf9/Slc4a1/Anxa1	19
BP	GO:0019079	viral genome replication	12/574	128/28814	4.71	1.01544E-05	0.000586197	0.000461298	Apobec3/Ifitm3/Top2a/Oasl2/Ccl5/Oas1b/Rsad2/Bst2/Ccl12/Oas2/Trim6/Slpi	12
BP	GO:0072676	lymphocyte migration	11/574	108/28814	5.11	1.07505E-05	0.000611869	0.000481501	Cxcl12/Icam1/Ccl6/Ccl5/Ccl9/Ccl4/Ccl3/Ccr6/Ccl12/Hsd3b7/Ch25h	11

BP	GO:0050678	regulation of epithelial cell proliferation	22/556	405/28814	2.82	1.5486E-05	0.001810228	0.001428416	Prl/Cdh3/Igf2/Twist1/Aldh1a2/Ccnd1/Fgfbp1/Cdh1/Bmp5/Nr4a3/Thbs1/Nog/Tbx18/Six4/Sfrp1/Esr1/Pparg/Dab2/Nox4/Agtr1a/Vdr/Nkx2-9	22
BP	GO:0016331	morphogenesis of embryonic epithelium	14/556	185/28814	3.92	1.57685E-05	0.001810228	0.001428416	Twist1/Aldh1a2/Bmp5/Zic5/Zic2/Nog/Tbx18/Six4/Sfrp1/Fzd1/Tead2/Spint2/Ret/Spint1	14
BP	GO:0034341	response to interferon-gamma	12/556	139/28814	4.47	1.71219E-05	0.001910997	0.001507931	Gbp8/Ifitm1/H2-Aa/Mrc1/Ifitm3/H2-Eb1/Bst2/Cd74/Pparg/Ccl1/Ifitm2/Gbp4	12
BP	GO:0009914	hormone transport	21/556	380/28814	2.86	1.8662E-05	0.002026594	0.001599147	Pomc/Hnf1a/Tfap2b/Aqp1/Neurod1/Lepr/Cartpt/Crh/Sfrp1/Pparg/Tapo/Fzd4/Dab2/Edn3/Slc9b2/Pde1c/Edn1/Oprk1/Anxa1/Vdr/Retn	21
BP	GO:0009410	response to xenobiotic stimulus	19/556	325/28814	3.03	2.14532E-05	0.002252407	0.001777331	Igf2/Fos/Errfi1/Aldh3a1/Cyp1b1/Gstm2/Grm2/Slc1a1/Casp1/Fzd1/Ntrk1/Cyp2a5/Slc10a4/Cyp2e1/Cyp2c23/Cyp2j6/Acadm/Pcx	19
BP	GO:0050673	epithelial cell proliferation	24/556	476/28814	2.61	2.18626E-05	0.002252407	0.001777331	Krt2/Prl/Cdh3/Igf2/Twist1/Aldh1a2/Ccnd1/Fgfbp1/Cdh1/Bmp5/Nr4a3/Thbs1/Lox/Nog/Tbx18/Six4/Sfrp1/Esr1/Pparg/Dab2/Nox4/Agtr1a/Vdr/Nkx2-9	24
BP	GO:0072175	epithelial tube formation	13/556	167/28814	4.03	2.33892E-05	0.002349441	0.001853898	Twist1/Bmp5/Zic5/Tcap/Zic2/Nog/Six4/Sfrp1/Fzd1/Tead2/Spint2/Ret/Spint1	13
BP	GO:0090287	regulation of cellular response to growth factor stimulus	18/556	300/28814	3.11	2.49537E-05	0.002445463	0.001929668	Tfap2b/Hpx/Wfikkn2/Fgfbp1/Ccnd1/Cdh1/Thbs1/Nog/Sfrp1/Pparg/Tmem100/Fzd1/Fzd4/Dab2/Nprn/Nkx2-1/Adamts3/Tdgf1	18
BP	GO:0008217	regulation of blood pressure	15/556	220/28814	3.53	2.72129E-05	0.002603363	0.002054264	Pomc/Npr3/Cartpt/Col1a2/Crh/Npr1/Pparg/Edn3/Adamts16/Nkx2-1/Edn1/Agtr1a/Myh6/Avpr2/Serpinf2	15
BP	GO:0048525	negative regulation of viral process	10/556	102/28814	5.08	2.8704E-05	0.002682156	0.002116438	Ch25h/Ifitm1/Rsad2/Ifitm3/Bst2/Pbln1/Oas2/Parp10/Cd74/Ifitm2	10
BP	GO:2000846	regulation of corticosteroid hormone secretion	5/556	20/28814	12.96	3.20463E-05	0.002926412	0.002309176	Pomc/Crh/Tapo/Dab2/Agtr1a	5
BP	GO:0046883	regulation of hormone secretion	18/556	307/28814	3.04	3.38004E-05	0.003017998	0.002381444	Pomc/Hnf1a/Tfap2b/Lepr/Cartpt/Crh/Sfrp1/Pparg/Tapo/Dab2/Edn3/Slc9b2/Pde1c/Edn1/Oprk1/Agtr1a/Vdr/Retn	18
BP	GO:0019932	second-messenger-mediated signaling	18/556	309/28814	3.02	3.67942E-05	0.003213892	0.00253602	Pomc/Adcy4/Agp1/Neurod1/Thbs1/Sphk1/Nfatc4/Crh/Npr1/Homer2/Edn3/Pde5a/Tox3/Trat1/Edn1/Pde10a/Agtr1a/Prnp	18
BP	GO:0030282	bone mineralization	11/556	128/28814	4.45	3.98425E-05	0.003325603	0.002624169	Ostn/Omd/Dmm3os/Twist1/Lncpint/Sgms2/Col1a2/Ddr2/Tent5a/Phex/Fgr	11
BP	GO:0072073	kidney epithelium development	12/556	152/28814	4.09	4.16915E-05	0.003325603	0.002624169	Tfap2b/Crif1/Agp1/Nog/Six4/Sfrp1/Nprt/Adamts16/Rarb/Ret/Agtr1a/Wt1	12
BP	GO:1903901	negative regulation of viral life cycle	6/556	34/28814	9.15	4.26813E-05	0.003325603	0.002624169	Ch25h/Ifitm1/Ifitm3/Bst2/Cd74/Ifitm2	6
BP	GO:0001657	ureteric bud development	10/556	107/28814	4.84	4.34557E-05	0.003325603	0.002624169	Crif1/Nog/Six4/Sfrp1/Nprt/Adamts16/Rarb/Ret/Agtr1a/Wt1	10
BP	GO:0072163	mesonephric epithelium development	10/556	107/28814	4.84	4.34557E-05	0.003325603	0.002624169	Crif1/Nog/Six4/Sfrp1/Nprt/Adamts16/Rarb/Ret/Agtr1a/Wt1	10
BP	GO:0072164	mesonephric tubule development	10/556	107/28814	4.84	4.34557E-05	0.003325603	0.002624169	Crif1/Nog/Six4/Sfrp1/Nprt/Adamts16/Rarb/Ret/Agtr1a/Wt1	10
BP	GO:0043903	regulation of biological process involved in symbiotic interaction	8/556	67/28814	6.19	4.38668E-05	0.003325603	0.002624169	Pomc/Ch25h/Ifitm1/Ifitm3/Rtn1/Tmem34a/Cd74/Ifitm2	8
BP	GO:0007411	axon guidance	16/556	258/28814	3.21	4.69649E-05	0.003494535	0.00275747	Lmx1a/Ocm/Nr4a3/Egr2/Zic2/Nog/Boc/Ntrn5/Lama1/Edn3/Nkx2-1/Ntrk1/Edn1/Rhoh/Gbx1/Prrxl1	16
BP	GO:0097485	neuron projection guidance	16/556	259/28814	3.2	4.9184E-05	0.003593111	0.002835255	Lmx1a/Ocm/Nr4a3/Egr2/Zic2/Nog/Boc/Ntrn5/Lama1/Edn3/Nkx2-1/Ntrk1/Edn1/Rhoh/Gbx1/Prrxl1	16
BP	GO:0048660	regulation of smooth muscle cell proliferation	13/556	180/28814	3.74	5.11886E-05	0.003672781	0.002898121	Ogn/Npr3/Nr4a3/Thbs1/Ddr2/Npr1/Esr1/Pdgfd/Pparg/Ccn4/Edn1/Retn/Serpinf2	13
BP	GO:0035930	corticosteroid hormone secretion	5/556	22/28814	11.78	5.27221E-05	0.003716448	0.002932577	Pomc/Crh/Tapo/Dab2/Agtr1a	5
BP	GO:0001838	embryonic epithelial tube formation	12/556	156/28814	3.99	5.3748E-05	0.003723441	0.002938096	Twist1/Bmp5/Zic5/Zic2/Nog/Six4/Sfrp1/Fzd1/Tead2/Spint2/Ret/Spint1	12
BP	GO:0001823	mesonephros development	10/556	110/28814	4.71	5.50913E-05	0.003751808	0.002960479	Crif1/Nog/Six4/Sfrp1/Nprt/Adamts16/Rarb/Ret/Agtr1a/Wt1	10
BP	GO:0008643	carbohydrate transport	12/556	158/28814	3.94	6.08317E-05	0.004042045	0.003189499	Ostn/Hnf1a/Agp1/Slc2a12/Nr4a3/Slc37a2/Esr1/Slc35d3/Edn1/Trib3/Slc26a5/Slc2a4	12
BP	GO:0046888	negative regulation of hormone secretion	9/556	90/28814	5.18	6.1365E-05	0.004042045	0.003189499	Cartpt/Crh/Sfrp1/Pparg/Tapo/Edn3/Pde1c/Edn1/Oprk1	9
BP	GO:0006631	fatty acid metabolic process	22/556	446/28814	2.56	6.57808E-05	0.004263019	0.003363866	Fads2b/Hnf1a/Acox2/Twist1/Ptgds/Acsm5/Nr4a3/Ptges/Sphk1/Cyp1b1/Alox12b/Cd74/Akr1c14/Pparg/Edn1/Cyp2a5/Trib3/Cyp2e1/Cyp2c23/Cyp2j6/Acaa1b/Cyp2j8	22
BP	GO:0061448	connective tissue development	17/556	295/28814	2.99	6.86328E-05	0.00437725	0.003454004	Cyt1l/Bmp5/Thbs1/Bgn/Lncpint/Lox2/Nog/Wnt9b/Frzb/Pdgfd/Pparg/Ccn4/Rarb/Col11a1/Col2a1/Edn1/Wt1	17
BP	GO:0007613	memory	12/556	161/28814	3.86	7.29645E-05	0.004542252	0.003584203	Lmx1a/Igf2/Nfatc4/Nog/Tafa2/Ascl/Asic1a/Casp1/Pde5a/Musk/Itga5/Slc2a4	12
BP	GO:0030278	regulation of ossification	11/556	137/28814	4.16	7.4209E-05	0.004542252	0.003584203	Ostn/Omd/Prl/Twist1/Egr2/Sgms2/Rsad2/Sfrp1/Ddr2/Tent5a/Vdr	11
BP	GO:0045785	positive regulation of cell adhesion	23/556	482/28814	2.47	7.46114E-05	0.004542252	0.003584203	Il7/Slamf1/Myoc/Igf2/Igfbp2/Cdh1/Nr4a3/H2-Aa/Thbs1/Tbx18/H2-Eb1/Rtn1/Cd74/Dab2/Dmp1/Nprn/Ret/Itga5/Rhoh/Nlrp3/Tdgf1/Vtcn1/Chst4	23
BP	GO:0048659	smooth muscle cell proliferation	13/556	188/28814	3.58	7.98997E-05	0.004791598	0.003780957	Ogn/Npr3/Nr4a3/Thbs1/Ddr2/Npr1/Esr1/Pdgfd/Pparg/Ccn4/Edn1/Retn/Serpinf2	13
BP	GO:0098868	bone growth	6/556	38/28814	8.18	8.20697E-05	0.004849356	0.003826533	Ostn/Bnc2/Lepr/Thbs1/Ddr2/Rarb	6
BP	GO:0007178	transmembrane receptor protein serine/threonine kinase signaling pathway	20/556	391/28814	2.65	8.56131E-05	0.004985411	0.003933892	Itgb6/Hnf1a/Tfap2b/Akap3/Hpx/Wfikkn2/Fos/Bmp5/Thbs1/Zic2/Col1a2/Nog/Sfrp1/Pparg/Fzd1/Dab2/Nprn/Nkx2-1/Tdgf1/Slc39a5	20
BP	GO:0050679	positive regulation of epithelial cell proliferation	14/556	217/28814	3.34	9.06987E-05	0.005206104	0.004108036	Prl/Cdh3/Igf2/Twist1/Ccnd1/Fgfbp1/Bmp5/Nr4a3/Nog/Tbx18/Six4/Sfrp1/Esr1/Nox4/Agtr1a	14
BP	GO:0014020	primary neural tube formation	10/556	119/28814	4.35	0.000107123	0.005974179	0.00471411	Twist1/Bmp5/Zic5/Zic2/Nog/Sfrp1/Fzd1/Tead2/Spint2/Spint1	10

BP	GO:0035455	response to interferon-alpha	5/305	31/28814	15.24	1.74344E-05	0.001610115	0.001385033	Oas1a/Ifit1/Ifi204/Ifitm3/Eif2ak2	5
BP	GO:0002697	regulation of immune effector process	16/305	436/28814	3.47	1.92533E-05	0.001679318	0.001444563	Cxcl1/Cxcl5/Dhx58/Il1r1/Ddx60/Fcgr2b/Pglyrp1/Ddx58/Nr4a3/Irak3/Il7r/H2-DMb2/Fcer2a/Cd36/Il27ra/Il13ra2	16
BP	GO:0051090	regulation of DNA-binding transcription factor activity	16/305	436/28814	3.47	1.92533E-05	0.001679318	0.001444563	Ltf/Hspa1b/Sigirr/Trim34a/Trim30a/Fcgr2b/Ndn/Eif2ak2/Parp10/Ddx58/Heyl/Irak3/Sfrp5/Gli1/Cd36/Pou4f1	16
BP	GO:0022409	positive regulation of cell-cell adhesion	13/305	298/28814	4.12	1.9855E-05	0.001684995	0.001449446	Sele/Selp/Il2ra/Il2rg/Piezo1/Runx1/Nr4a3/Il7r/H2-DMb2/Skap1/Fstl3/Il27ra/Icos	13
BP	GO:0051085	chaperone cofactor-dependent protein refolding	5/305	32/28814	14.76	2.04847E-05	0.001692683	0.001456059	Hspa1b/Dnajb13/Hspa5/Sdf2l1/H2-DMb2	5
BP	GO:0031349	positive regulation of defense response	13/305	302/28814	4.07	2.2824E-05	0.001837622	0.001580737	S100a9/S100a8/Cxcl1/Lgals2/Ifi207/Ifi209/Ifi204/Irf7/Nlrp10/Trim30a/Ptgs2/Ifi203/Casp1	13
BP	GO:0050727	regulation of inflammatory response	14/305	351/28814	3.77	2.58498E-05	0.002029208	0.001745541	S100a9/S100a8/Lgals2/Il2ra/Bst1/Nlrp10/Ptgs2/Il1r1/Fcgr2b/Pglyrp1/Casp1/Adrb2/Cst7/Gpx2	14
BP	GO:0060337	type I interferon signaling pathway	6/305	56/28814	10.12	2.7868E-05	0.002079519	0.001788818	Oas1a/Oas2/Isg15/Irf7/Ifitm3/Ifi27	6
BP	GO:0071357	cellular response to type I interferon	6/305	56/28814	10.12	2.7868E-05	0.002079519	0.001788818	Oas1a/Oas2/Isg15/Irf7/Ifitm3/Ifi27	6
BP	GO:0060759	regulation of response to cytokine stimulus	9/305	147/28814	5.78	2.84775E-05	0.002079519	0.001788818	Oas1a/Hspa1b/Sigirr/Isg15/Irf7/Il1r1/Ddx58/Casp1/Irak3	9
BP	GO:0032102	negative regulation of response to external stimulus	15/305	403/28814	3.52	2.97648E-05	0.00210602	0.001811615	Ltf/Il2ra/Oas1a/Drd3/Ifi207/Ifi204/Ccl12/Isg15/Dhx58/Fcgr2b/Pglyrp1/Adrb2/Irak3/Cst7/Gpx2	15
BP	GO:0032680	regulation of tumor necrosis factor production	10/305	185/28814	5.11	3.01818E-05	0.00210602	0.001811615	Ltf/Oas1a/Oas2/Hspb1/Trim30a/Ddx58/Irak3/Cd36/Il27ra/Cd2	10
BP	GO:0002237	response to molecule of bacterial origin	15/305	407/28814	3.48	3.33273E-05	0.002273417	0.001955611	Ltf/S100a9/S100a8/Cxcl1/Cxcl5/Fcgr4/Cyp27b1/Ccl12/Trim30a/Ptgs2/Fcgr2b/Eif2ak2/Casp1/Irak3/Cd36	15
BP	GO:1903555	regulation of tumor necrosis factor superfamily cytokine production	10/305	188/28814	5.03	3.46169E-05	0.002273417	0.001955611	Ltf/Oas1a/Oas2/Hspb1/Trim30a/Ddx58/Irak3/Cd36/Il27ra/Cd2	10
BP	GO:0030595	leukocyte chemotaxis	11/305	228/28814	4.56	3.47529E-05	0.002273417	0.001955611	S100a9/S100a8/Cxcl1/Ch25h/Cxcl5/Bst1/Ccl12/Gpr183/Dapk2/Ccl17/Ccl22	11
BP	GO:0032640	tumor necrosis factor production	10/305	191/28814	4.95	3.95979E-05	0.002537498	0.002182775	Ltf/Oas1a/Oas2/Hspb1/Trim30a/Ddx58/Irak3/Cd36/Il27ra/Cd2	10
BP	GO:0051084	'de novo' post-translational protein folding	5/305	37/28814	12.77	4.24624E-05	0.002666642	0.002293866	Hspa1b/Dnajb13/Hspa5/Sdf2l1/H2-DMb2	5
BP	GO:0022407	regulation of cell-cell adhesion	16/305	468/28814	3.23	4.5051E-05	0.002728099	0.002346732	Sele/Selp/Slfn1/Il2ra/Il2rg/Hspb1/Piezo1/Runx1/Nr4a3/Adrb2/Il7r/H2-DMb2/Skap1/Fstl3/Il27ra/Icos	16
BP	GO:0071706	tumor necrosis factor superfamily cytokine production	10/305	194/28814	4.87	4.51787E-05	0.002728099	0.002346732	Ltf/Oas1a/Oas2/Hspb1/Trim30a/Ddx58/Irak3/Cd36/Il27ra/Cd2	10
BP	GO:0006458	'de novo' protein folding	5/305	38/28814	12.43	4.84749E-05	0.002871911	0.00247044	Hspa1b/Dnajb13/Hspa5/Sdf2l1/H2-DMb2	5
BP	GO:2000116	regulation of cysteine-type endopeptidase activity	11/305	240/28814	4.33	5.5327E-05	0.003217161	0.002767427	Ltf/S100a9/S100a8/Ifi207/Hspa1b/Ifi204/Tnfrsf23/Fas/Ptgs2/Casp1/Cst7	11
BP	GO:0061077	chaperone-mediated protein folding	6/305	64/28814	8.86	5.99496E-05	0.003422578	0.002944128	Hspa1b/Hspb1/Dnajb13/Hspa5/Sdf2l1/H2-DMb2	6
BP	GO:0032496	response to lipopolysaccharide	14/305	386/28814	3.43	7.20899E-05	0.004042182	0.003477117	Ltf/S100a9/S100a8/Cxcl1/Cxcl5/Fcgr4/Cyp27b1/Ccl12/Trim30a/Ptgs2/Eif2ak2/Casp1/Irak3/Cd36	14
BP	GO:1903039	positive regulation of leukocyte cell-cell adhesion	11/305	248/28814	4.19	7.42296E-05	0.004089139	0.00351751	Sele/Selp/Il2ra/Il2rg/Runx1/Nr4a3/Il7r/H2-DMb2/Skap1/Il27ra/Icos	11
BP	GO:0032608	interferon-beta production	6/305	67/28814	8.46	7.77066E-05	0.00413557	0.003557449	Oas1a/Oas2/Isg15/Irf7/Dhx58/Ddx58	6
BP	GO:0032648	regulation of interferon-beta production	6/305	67/28814	8.46	7.77066E-05	0.00413557	0.003557449	Oas1a/Oas2/Isg15/Irf7/Dhx58/Ddx58	6
BP	GO:1903037	regulation of leukocyte cell-cell adhesion	13/305	343/28814	3.58	8.39255E-05	0.004348441	0.003740563	Sele/Selp/Slfn1/Il2ra/Il2rg/Hspb1/Runx1/Nr4a3/Il7r/H2-DMb2/Skap1/Il27ra/Icos	13
BP	GO:0032481	positive regulation of type I interferon production	6/305	68/28814	8.34	8.44761E-05	0.004348441	0.003740563	Oas1a/Oas2/Isg15/Irf7/Dhx58/Ddx58	6
BP	GO:0061844	antimicrobial humoral immune response mediated by antimicrobial peptide	8/305	134/28814	5.64	9.45652E-05	0.004789272	0.004119769	Ltf/S100a9/Cxcl1/Cxcl5/Ccl12/Pglyrp1/Ccl17/Ccl22	8
BP	GO:0001959	regulation of cytokine-mediated signaling pathway	8/305	137/28814	5.52	0.000110376	0.005501277	0.004732241	Oas1a/Hspa1b/Sigirr/Isg15/Irf7/Il1r1/Casp1/Irak3	8
BP	GO:0032479	regulation of type I interferon production	7/305	103/28814	6.42	0.00011565	0.005674087	0.004880895	Oas1a/Oas2/Isg15/Irf7/Dhx58/Ddx58/Gbp4	7
BP	GO:0034341	response to interferon-gamma	8/305	139/28814	5.44	0.000122084	0.005897584	0.005073148	Calcoco2/Cyp27b1/Ccl12/Ifitm3/Casp1/Gbp4/Ccl17/Ccl22	8
BP	GO:0032732	positive regulation of interleukin-1 production	6/305	73/28814	7.76	0.000125703	0.005980429	0.005144412	Ifi207/Ifi204/Hspb1/Nlrp10/Casp1/Cd36	6
BP	GO:1900246	positive regulation of RIG-I signaling pathway	3/305	10/28814	28.34	0.000133375	0.006250703	0.005376904	Oasl1/Dhx58/Ddx60	3
BP	GO:0062208	positive regulation of pattern recognition receptor signaling pathway	5/305	47/28814	10.05	0.000137055	0.00632872	0.005444015	Ltf/Oasl1/Hspa1b/Dhx58/Ddx60	5
BP	GO:0060326	cell chemotaxis	12/305	314/28814	3.61	0.000144952	0.006586545	0.005665798	S100a9/S100a8/Cxcl1/Ch25h/Cxcl5/Bst1/Ccl12/Hspb1/Gpr183/Dapk2/Ccl17/Ccl22	12
BP	GO:0032606	type I interferon production	7/305	107/28814	6.18	0.000146834	0.006586545	0.005665798	Oas1a/Oas2/Isg15/Irf7/Dhx58/Ddx58/Gbp4	7
BP	GO:0039529	RIG-I signaling pathway	4/305	26/28814	14.53	0.000153117	0.006732998	0.005791778	Oasl1/Dhx58/Ddx60/Ddx58	4

BP	GO:0010594	regulation of endothelial cell migration	14/574	174/28814	4.04	1.12168E-05	0.000628549	0.000494626	Lcn2/Tek/Kdr/Klf4/Hspb1/Gata2/Flt4/Prkd2/Mmrn2/Dll4/Angpt2/Ager/Agt/Anxa1	14
BP	GO:1903039	positive regulation of leukocyte cell-cell adhesion	17/574	248/28814	3.44	1.13546E-05	0.000628549	0.000494626	Rasal3/Icam1/Lef1/Ptprc/Ccl5/Il2rg/Il7r/Il12rb2/Rag1/Lilrb4a/Hlx/Ager/Il4i1/H2-DMb1/Tnfsf9/Slc4a1/Anxa1	17
BP	GO:0050870	positive regulation of T cell activation	16/574	223/28814	3.6	1.16057E-05	0.000633767	0.000498733	Rasal3/Lef1/Ptprc/Ccl5/Il2rg/Il7r/Il12rb2/Rag1/Lilrb4a/Hlx/Ager/Il4i1/H2-DMb1/Tnfsf9/Slc4a1/Anxa	16
BP	GO:0002698	negative regulation of immune effector process	12/574	130/28814	4.63	1.18989E-05	0.00064111	0.000504512	Foxj1/Ptprc/Nod2/Pglyrp1/Tap1/Fcgr2b/Il7r/Bst2/Lilrb4a/Hlx/Il4i1/Anxa1	12
BP	GO:0030098	lymphocyte differentiation	24/574	447/28814	2.7	1.31887E-05	0.000701255	0.000551841	Foxj1/Hspb1/Lef1/Ly9/Ptprc/Mfng/Pglyrp1/Il2rg/Cmtm7/Il7r/Rag1/Prdm1/Rsad2/Dll4/Bcl3/Lilrb4a/Fas/Ctla2a/6030468B19Rik/Ccr6/Hlx/I	24
BP	GO:0002709	regulation of T cell mediated immunity	11/574	111/28814	4.97	1.39771E-05	0.000733523	0.000577234	H2-D1/Il1r1/Ptprc/Nod2/H2-Q4/Il7r/Rsad2/Lilrb4a/H2-T10/Ager/Il4i1	11
BP	GO:0035458	cellular response to interferon-beta	8/574	56/28814	7.17	1.44711E-05	0.000749712	0.000589974	Gbp3/Irgm1/Igtp/Ifit1/Gbp2/Gm4951/Trim6/Gbp6	8
BP	GO:0051607	defense response to virus	18/574	281/28814	3.22	1.58868E-05	0.000812639	0.000639493	Apobec3/Ifitm3/Oasl2/Gbp4/Irf7/Slfn8/Ptprc/Ccl5/Kcnj8/Oas1b/Trim30a/Rsad2/Bst2/Ifit1/Ddx60/Oas2/Ticam2/	18
BP	GO:0140546	defense response to symbiont	18/574	282/28814	3.2	1.66623E-05	0.000833657	0.000656033	Apobec3/Ifitm3/Oasl2/Gbp4/Irf7/Slfn8/Ptprc/Ccl5/Kcnj8/Oas1b/Trim30a/Rsad2/Bst2/Ifit1/Ddx60/Oas2/Ticam2/	18
BP	GO:1904035	regulation of epithelial cell apoptotic process	10/574	93/28814	5.4	1.67103E-05	0.000833657	0.000656033	Cdh5/Tek/Kdr/Icam1/Gata2/Nupr1/Ecscr/Cd248/Ccl12/Ager	10
BP	GO:0060562	epithelial tube morphogenesis	22/574	395/28814	2.8	1.71641E-05	0.000845854	0.000665631	Flt1/Eng/Lcn2/Tek/Cxcl12/Kdr/Ctsh/Lama5/Tie1/Rasip1/Adm/Lef1/Mmrn2/Wnt6/Tcap/Stil/Dll4/Tbx1/Grhl2/	22
BP	GO:0001562	response to protozoan	7/574	42/28814	8.37	1.76864E-05	0.000861094	0.000677624	Gbp3/Gbp9/Gbp4/Bcl3/Gbp2/Batf2/Gbp6	7
BP	GO:0002449	lymphocyte mediated immunity	25/574	488/28814	2.57	1.93024E-05	0.000919826	0.000723842	C4b/H2-D1/Ctsh/Il1r1/Foxj1/Icam1/Irf7/Ptprc/Nod2/H2-Q4/Tap1/Fcgr2b/Il7r/Rsad2/Bcl3/Lilrb4a/Fas/H2-T10/6030468B19Rik/Csf2rb/Ccr6/Ager/Il4i1/Igha/H2-	25
BP	GO:0097530	granulocyte migration	13/574	159/28814	4.1	1.9348E-05	0.000919826	0.000723842	Pecam1/Il1r1/Ccl6/Ccl5/Nod2/Ccl9/Ccl4/Rarres2/Ccl3/Cmklr1/Ccl12/S100a9/Anxa1	13
BP	GO:0002704	negative regulation of leukocyte mediated immunity	9/574	76/28814	5.94	2.01926E-05	0.000948817	0.000746656	Foxj1/Ptprc/Nod2/Tap1/Fcgr2b/Il7r/Bst2/Lilrb4a/Il4i1	9
BP	GO:0042092	type 2 immune response	7/574	43/28814	8.17	2.0765E-05	0.000964497	0.000758995	Nod2/Rsad2/Bcl6b/Bcl3/Lilrb4a/Hlx/Anxa1	7
BP	GO:0043534	blood vessel endothelial cell migration	11/574	116/28814	4.76	2.1232E-05	0.000967486	0.000761348	Cdh5/Kdr/Klf4/Hspb1/Gata2/Prkd2/Mmrn2/Clec14a/Dll4/Angpt2/Anxa1	11
BP	GO:0044406	adhesion of symbiont to host	5/574	18/28814	13.94	2.13082E-05	0.000967486	0.000761348	Gbp3/Gbp9/Icam1/Gbp2/Gbp6	5
BP	GO:0045446	endothelial cell differentiation	11/574	117/28814	4.72	2.30204E-05	0.001033617	0.000813388	Pecam1/Cdh5/Apold1/Kdr/Tie1/Robo4/Icam1/Sox18/Cldn3/Cxcr4/Agt	11
BP	GO:0070555	response to interleukin-1	10/574	97/28814	5.18	2.42285E-05	0.001075906	0.000846667	Fn1/Il1r1/Ccl6/Ccl5/Ccl9/Ccl4/Mylk3/Ccl3/Ccl12/Anxa1	10
BP	GO:0050678	regulation of epithelial cell proliferation	22/574	405/28814	2.73	2.51252E-05	0.001103599	0.00086846	Flt1/Eng/Tek/Cxcl12/Kdr/Tie1/Gata2/Flt4/Wfdc1/Ccl5/Prkd2/Nod2/Mmrn2/Nupr1/Ngfr/Dll4/Lrg1/Tbx1/Ccl12/	22
BP	GO:0050863	regulation of T cell activation	20/574	348/28814	2.88	2.63715E-05	0.001145885	0.000901736	Foxj1/Rasal3/Hspb1/Lef1/Ptprc/Ccl5/Il2rg/Il7r/Il12rb2/Rag1/Prdm1/Lilrb4a/Ctla2a/Hlx/Ager/Il4i1/H2-DMb1/Tnfsf9/Slc4a1/Anxa	20
BP	GO:0022407	regulation of cell-cell adhesion	24/574	468/28814	2.57	2.77378E-05	0.001184083	0.000931795	Podxl/Cxcl12/Foxj1/Rasal3/Klf4/Hspb1/Icam1/Ccm2l/Lef1/Piezo1/Ptprc/Ccl5/Il2rg/Il7r/Il12rb2/Rag1/Lilrb4a/Hlx/Ager/Il4i1/H2-DMb1/Tnfsf9/Slc4a1/Anxa	24
BP	GO:0050777	negative regulation of immune response	14/574	189/28814	3.72	2.84227E-05	0.001184083	0.000931795	Foxj1/Ptprc/Parp14/Nod2/Pglyrp1/Tap1/Fcgr2b/Il7r/Nlrc5/Lilrb4a/Hlx/Olfm4/Il4i1/Anxa1	14
BP	GO:1902107	positive regulation of leukocyte differentiation	14/574	189/28814	3.72	2.84227E-05	0.001184083	0.000931795	Lef1/Ptprc/Ccl5/Il2rg/Il7r/Rag1/Prdm1/Ccl3/Lilrb4a/Hlx/Ager/Il4i1/Tnfsf9/Anxa1	14
BP	GO:1903708	positive regulation of hemopoiesis	14/574	189/28814	3.72	2.84227E-05	0.001184083	0.000931795	Lef1/Ptprc/Ccl5/Il2rg/Il7r/Rag1/Prdm1/Ccl3/Lilrb4a/Hlx/Ager/Il4i1/Tnfsf9/Anxa1	14
BP	GO:0010632	regulation of epithelial cell migration	16/574	241/28814	3.33	3.01982E-05	0.001245214	0.000979901	Lcn2/Tek/Kdr/Ctsh/Klf4/Hspb1/Gata2/Flt4/Prkd2/Mmrn2/Dll4/Ccr6/Angpt2/Ager/Agt/Anxa1	16
BP	GO:0071347	cellular response to interleukin-1	9/574	80/28814	5.65	3.06337E-05	0.001250414	0.000983993	Fn1/Il1r1/Ccl6/Ccl5/Ccl9/Ccl4/Mylk3/Ccl3/Ccl12	9
BP	GO:0070098	chemokine-mediated signaling pathway	8/574	62/28814	6.48	3.10237E-05	0.001253666	0.000986552	Cxcl12/Ccl6/Ccl5/Ccl9/Ccl4/Ccl3/Ccl12/Cxcr4	8
BP	GO:1904019	epithelial cell apoptotic process	11/574	121/28814	4.56	3.15402E-05	0.001261921	0.000993048	Cdh5/Tek/Kdr/Icam1/Gata2/Nupr1/Ecscr/Cd248/Fas/Ccl12/Ager	11
BP	GO:0052547	regulation of peptidase activity	23/574	443/28814	2.61	3.3537E-05	0.001328658	0.001045566	Acer2/Uaca/Ctsh/Klf4/Hspa1b/Serpinb1b/Cst7/Wfdc1/Ngfr/Rag1/Tnfsf10/Sox7/Fas/Rcn3/Spink4/Cldn3/Avp/S100a9/Ager/Wfikkn2/Serpin	23
BP	GO:0045621	positive regulation of lymphocyte differentiation	11/574	122/28814	4.53	3.40526E-05	0.001335984	0.001051331	Lef1/Ptprc/Il2rg/Il7r/Rag1/Prdm1/Lilrb4a/Hlx/Il4i1/Tnfsf9/Anxa1	11
BP	GO:0002707	negative regulation of lymphocyte mediated immunity	8/574	63/28814	6.37	3.49226E-05	0.001356943	0.001067824	Foxj1/Ptprc/Nod2/Tap1/Fcgr2b/Il7r/Lilrb4a/Il4i1	8
BP	GO:0003014	renal system process	11/574	123/28814	4.49	3.67355E-05	0.001413793	0.001112562	Adgrf5/Adm/Sctr/Avpr1a/Abcg3/Aqp3/Avp/Oxt/Slc4a1/Tfap2b/Agt	11
BP	GO:0043114	regulation of vascular permeability	7/574	47/28814	7.48	3.78325E-05	0.001442273	0.001134974	Cdh5/Plvap/Adm/Gpr4/Ccl4/Mylk3/Tacr2	7
BP	GO:0002683	negative regulation of immune system process	23/574	448/28814	2.58	3.98595E-05	0.001505349	0.00118461	Adgrf5/Cxcl12/Foxj1/Hspb1/Gata2/Cst7/Ptprc/Parp14/Nod2/Pglyrp1/Tap1/Fcgr2b/Il7r/Prdm1/Bst2/Nlrc5/Lilrb4a/Fas/Hlx/Ccl12/Olfm4/Il4i	23
BP	GO:0002703	regulation of leukocyte mediated immunity	18/574	304/28814	2.97	4.48817E-05	0.001679324	0.001321517	H2-D1/Il1r1/Foxj1/Gata2/Ptprc/Nod2/H2-Q4/Tap1/Fcgr2b/Il7r/Rsad2/Bst2/Nppc/Lilrb4a/H2-T10/6030468B19Rik/Ager/I	18
BP	GO:0045582	positive regulation of T cell differentiation	10/574	106/28814	4.74	5.23142E-05	0.001939466	0.001526232	Lef1/Ptprc/Il2rg/Il7r/Rag1/Lilrb4a/Hlx/Il4i1/Tnfsf9/Anxa1	10
BP	GO:0071621	granulocyte chemotaxis	11/574	128/28814	4.31	5.30568E-05	0.001949113	0.001533823	Ccl6/Ccl5/Nod2/Ccl9/Ccl4/Rarres2/Ccl3/Cmklr1/Ccl12/S100a9/Anxa1	11
BP	GO:0070661	leukocyte proliferation	20/574	368/28814	2.73	5.76265E-05	0.002097917	0.001650922	Cxcl12/Foxj1/Rasal3/Lef1/Ptprc/Ccl5/Fcgr2b/Il7r/Il12rb2/Prdm1/Lilrb4a/Csf2rb/Ccl12/Cxcr4/Ager/Il4i1/H2-DMb1/Tnfsf9/Slc4a1/Anxa1	20
BP	GO:0050920	regulation of chemotaxis	15/574	230/28814	3.27	6.4993E-05	0.002342938	0.001843737	Fn1/Cxcl12/Kdr/Hspb1/Sema3g/Ccl5/Prkd2/Nod2/Rarres2/Cmklr1/Ccr6/Angpt2/C	15
BP	GO:0034612	response to tumor necrosis factor	14/574	204/28814	3.45	6.55165E-05	0.002342938	0.001843737	Plvap/Gbp3/Hspa1b/Traip/Ccl6/Ccl5/Ccl9/Gch1/Ccl4/Ccl3/Ccl12/Mir103-2/	14
BP	GO:0002706	regulation of lymphocyte mediated immunity	15/574	232/28814	3.25	7.16653E-05	0.002521363	0.001984146	H2-D1/Il1r1/Foxj1/Ptprc/Nod2/H2-Q4/Tap1/Fcgr2b/Il7r/Rsad2/Lilrb4a/H2-T10/6030468B19Rik/Ager/I	15
BP	GO:0016525	negative regulation of angiogenesis	10/574	110/28814	4.56	7.17537E-05	0.002521363	0.001984146	Flt1/Tek/Tie1/Klf4/Gpr4/Klf2/Ngfr/Ecscr/Angpt2/Agt	10
BP	GO:0019058	viral life cycle	16/574	261/28814	3.08	7.82136E-05	0.002724664	0.002144131	Apobec3/Ifitm3/Top2a/Oasl2/Icam1/Ccl5/Oas1b/Trim30a/Rsad2/Bst2/Ccl12/Oas2/Ch25h/Avp/Trim6/Slpi	16

BP	GO:1903036	positive regulation of response to wounding	8/556	76/28814	5.46	0.000108989	0.005974179	0.00471411	Ocm/Duox1/Cldn1/Thbs1/Ddr2/Ccn4/Clec7a/Serpinf2	8
BP	GO:0035567	non-canonical Wnt signaling pathway	7/556	57/28814	6.36	0.000110027	0.005974179	0.00471411	Myoc/Frzb/Rspo3/Sfrp1/Fzd1/Fzd4/Dab2	7
BP	GO:0051353	positive regulation of oxidoreductase activity	7/556	57/28814	6.36	0.000110027	0.005974179	0.00471411	Cdh3/Npr3/Esr1/Edn3/Edn1/Nod2/Vdr	7
BP	GO:0001649	osteoblast differentiation	14/556	222/28814	3.27	0.000115464	0.006185781	0.004881081	Ostn/Myoc/Igf2/Twist1/Ranbp3l/Nog/Sfrp1/Ddr2/Pparg/Fzd1/Npnt/Tent5a/Ccn4/Dhh	14
BP	GO:0045793	positive regulation of cell size	4/556	14/28814	14.81	0.000117712	0.006223225	0.004910627	Trp73/Ret/Edn1/Slc26a5	4
BP	GO:0043583	ear development	15/556	251/28814	3.1	0.000121349	0.006332214	0.004996628	Mcoln3/Cytl1/Neurod1/Cdh1/Bmp5/Nr4a3/Tcap/Nog/Tbx18/Six4/Frzb/Col11a1/Col2a1/Edn1/Mvo3b	15
BP	GO:0090303	positive regulation of wound healing	7/556	58/28814	6.25	0.000123069	0.006339655	0.005002499	Duox1/Cldn1/Thbs1/Ddr2/Ccn4/Clec7a/Serpinf2	7
BP	GO:0050792	regulation of viral process	12/556	171/28814	3.64	0.000129662	0.006581719	0.005193507	Ch25h/Ifitm1/Mdfic/Rsad2/Ifitm3/Bst2/Fbln1/Oasl2/Trim34a/Parp10/Cd74/Ifitm2	12
BP	GO:0019369	arachidonic acid metabolic process	8/556	78/28814	5.32	0.000131045	0.006581719	0.005193507	Sphk1/Cyp1b1/Alox12b/Cyp2a5/Cyp2e1/Cyp2c23/Cyp2g1/Cyp2j8	8
BP	GO:0045071	negative regulation of viral genome replication	7/556	59/28814	6.15	0.000137339	0.006812685	0.005375759	Ifitm1/Rsad2/Ifitm3/Bst2/Oasl2/Parp10/Ifitm2	7
BP	GO:0003014	renal system process	10/556	123/28814	4.21	0.000141048	0.006911374	0.005453632	Hnf1a/Tfap2b/Aqp1/Slc22a6/Npr3/Dab2/Edn1/Agtr1a/Avpr2/Serpinf2	10
BP	GO:0043405	regulation of MAP kinase activity	13/556	200/28814	3.37	0.000148761	0.007199879	0.005681286	Thbs1/Trp73/Arhgef5/Sfrp1/Pdgfd/Pparg/Fzd4/Edn3/Pde5a/Edn1/Trib3/Nod2/Tdgf1	13
BP	GO:0050795	regulation of behavior	9/556	101/28814	4.62	0.00015052	0.007199879	0.005681286	Ptgds/Lepr/Nr4a3/Crh/Cort/Casp1/Htr1d/Oprk1/Retn	9
BP	GO:0010226	response to lithium ion	4/556	15/28814	13.82	0.000158071	0.007472105	0.005896094	Cdh1/Nfatc4/Pparg/Pklr	4
BP	GO:0045165	cell fate commitment	16/556	287/28814	2.89	0.00016284	0.007578587	0.005980117	Il7/Dll3/Neurod1/Prdm1/Tbx18/Wnt9a/Sfrp1/Pparg/Epop/Ptch2/Nkx2-1/Edn1/Gbx1/Wnt8b/Wt1/Dhh	16
BP	GO:0060348	bone development	15/556	258/28814	3.01	0.000164096	0.007578587	0.005980117	Itgb6/Ostn/Myoc/Bnc2/Twist1/Col13a1/Lepr/Ranbp3l/Thbs1/Bgn/Ddr2/Ccn4/Rarb/Slc9b2/Col2a1	15
BP	GO:0033002	muscle cell proliferation	15/556	259/28814	3	0.00017116	0.007815014	0.006166677	Ogn/Npr3/Nr4a3/Thbs1/Trp73/Nog/Ddr2/Npr1/Esr1/Pdgfd/Pparg/Ccn4/Edn1/Retn/Serpinf2	15
BP	GO:0007530	sex determination	5/556	28/28814	9.25	0.000178831	0.008073535	0.006370671	Tmem184a/Ptgdr/Six4/Wt1/Dhh	5
BP	GO:0014706	striated muscle tissue development	16/556	290/28814	2.86	0.000183269	0.008181959	0.006456226	Twist1/Aldh1a2/Bmp5/Tcap/Trp73/Nog/Tbx18/Mylk3/Boc/Ccn4/Rarb/Col11a1/Edn1/Tdgf1/Myh6/Wt1	16
BP	GO:0034219	carbohydrate transmembrane transport	10/556	129/28814	4.02	0.000208658	0.009213056	0.007269845	Ostn/Hnf1a/Aqp1/Slc2a12/Nr4a3/Esr1/Edn1/Trib3/Slc26a5/Slc2a4	10
BP	GO:0072330	monocarboxylic acid biosynthetic process	13/556	208/28814	3.24	0.000218937	0.009561816	0.007545045	Fads2b/Hnf1a/Rdh16/Aldh1a2/Ptgds/Acsm5/Ptges/Sphk1/Alox12b/Cd74/Edn1/Trib3/Pklr	13
BP	GO:0001990	regulation of systemic arterial blood pressure by hormone	6/556	46/28814	6.76	0.000244412	0.010559653	0.008332419	Edn3/Nkx2-1/Edn1/Agtr1a/Avpr2/Serpinf2	6
BP	GO:0001516	prostaglandin biosynthetic process	5/556	30/28814	8.64	0.000251192	0.010620051	0.008380078	Ptgds/Ptges/Sphk1/Cd74/Edn1	5
BP	GO:0046457	prostanoid biosynthetic process	5/556	30/28814	8.64	0.000251192	0.010620051	0.008380078	Ptgds/Ptges/Sphk1/Cd74/Edn1	5
BP	GO:0006081	cellular aldehyde metabolic process	7/556	65/28814	5.58	0.000253739	0.010620051	0.008380078	Aldh3b2/Abca4/Aldh1a2/Bmp5/Aldh3a1/Cyp1b1/Dab2	7
BP	GO:0060537	muscle tissue development	22/556	493/28814	2.31	0.000272248	0.011277235	0.008898649	Tfap2b/Igf2/Twist1/Aldh1a2/Fos/Bmp5/Egr2/Lncpint/Tcap/Trp73/Nog/Tbx18/Six4/Mylk3/Boc/Ccn4/Rarb/Col11a1/Edn1/Tdgf1/Myh6/Wt1	22
BP	GO:0051216	cartilage development	13/556	213/28814	3.16	0.000275862	0.011310324	0.008924758	Cytl1/Bmp5/Thbs1/Bgn/Loxl2/Nog/Wnt9a/Frzb/Ccn4/Rarb/Col11a1/Col2a1/Edn1	13
BP	GO:0001841	neural tube formation	10/556	134/28814	3.87	0.00028407	0.011529241	0.009097502	Twist1/Bmp5/Zic5/Zic2/Nog/Sfrp1/Fzd1/Tead2/Spint2/Spint1	10
BP	GO:0046597	negative regulation of viral entry into host cell	5/556	31/28814	8.36	0.000294778	0.01172693	0.009253495	Ch25h/Ifitm1/Ifitm3/Cd74/Ifitm2	5
BP	GO:0061037	negative regulation of cartilage development	5/556	31/28814	8.36	0.000294778	0.01172693	0.009253495	Nog/Wnt9a/Frzb/Ccn4/Rarb	5
BP	GO:0014897	striated muscle hypertrophy	9/556	111/28814	4.2	0.000307411	0.012109572	0.00955543	Myoc/Nr4a3/Tcap/Mlip/Pparg/Pde5a/Ccn4/Edn1/Myh6	9
BP	GO:0060070	canonical Wnt signaling pathway	16/556	304/28814	2.73	0.000310871	0.012126998	0.00956918	Lmx1a/Cdh3/Cdh1/Nog/Tbx18/Wnt9a/Frzb/Rspo3/Sfrp1/Fzd1/Fzd4/Dab2/Nkd2/Edn1/Wnt8b/Peg12	16
BP	GO:0001843	neural tube closure	9/556	112/28814	4.16	0.000328659	0.012697599	0.01001943	Twist1/Zic5/Zic2/Nog/Sfrp1/Fzd1/Tead2/Spint2/Spint1	9
BP	GO:0002830	positive regulation of type 2 immune response	4/556	18/28814	11.52	0.00033843	0.012942562	0.010212726	Rsad2/Cd74/Nlrp3/Nod2	4
BP	GO:0003416	endochondral bone growth	5/556	32/28814	8.1	0.000343864	0.012942562	0.010212726	Ostn/Bnc2/Thbs1/Ddr2/Rarb	5
BP	GO:0008202	steroid metabolic process	17/556	338/28814	2.61	0.000345748	0.012942562	0.010212726	Hnf1a/Ch25h/Rdh16/Igf2/Lepr/Bmp5/Cubn/Cyp1b1/Crh/Akr1c14/Esr1/Tspo/Dab2/Cyp2e1/Agtr1a/Acaa1b/Dhh	17
BP	GO:0014896	muscle hypertrophy	9/556	113/28814	4.13	0.000351105	0.012942562	0.010212726	Myoc/Nr4a3/Tcap/Mlip/Pparg/Pde5a/Ccn4/Edn1/Myh6	9
BP	GO:0060606	tube closure	9/556	113/28814	4.13	0.000351105	0.012942562	0.010212726	Twist1/Zic5/Zic2/Nog/Sfrp1/Fzd1/Tead2/Spint2/Spint1	9
BP	GO:0050878	regulation of body fluid levels	18/556	373/28814	2.5	0.000384528	0.014045772	0.011083248	Pf4/Prl/Tfap2b/Aqp1/Tspan8/Cldn1/Cdh1/Npr3/Thbs1/Trp73/Alox12b/Fbln1/Scnn1a/Edn1/Oprk1/Vdr/Avpr2/Serpinf2	18
BP	GO:0060669	embryonic placenta morphogenesis	5/556	33/28814	7.85	0.000398889	0.014439048	0.011393574	Igf2/Bmp5/Rspo3/Spint2/Spint1	5
BP	GO:0030178	negative regulation of Wnt signaling pathway	11/556	167/28814	3.41	0.000421742	0.014996095	0.01183313	Wif1/Cdh1/Nfatc4/Nog/Tbx18/Frzb/Sfrp1/Fzd1/Fzd4/Dab2/Nkd2	11
BP	GO:1905952	regulation of lipid localization	11/556	167/28814	3.41	0.000421742	0.014996095	0.01183313	Pomc/Thbs1/Ptges/Crh/Pparg/Tspo/Dab2/Nfkbia/Edn1/Agtr1a/Retn	11
BP	GO:0046456	icosanoid biosynthetic process	6/556	51/28814	6.1	0.00043323	0.015269452	0.01204883	Ptgds/Ptges/Sphk1/Cd74/Edn1/Cyp2c23	6
BP	GO:0042471	ear morphogenesis	10/556	143/28814	3.62	0.000477157	0.016592686	0.013092969	Nr4a3/Tcap/Nog/Tbx18/Six4/Frzb/Col11a1/Col2a1/Edn1/Mvo3b	10
BP	GO:0046596	regulation of viral entry into host cell	6/556	52/28814	5.98	0.000481847	0.016592686	0.013092969	Ch25h/Ifitm1/Ifitm3/Trim34a/Cd74/Ifitm2	6

BP	GO:0050866	negative regulation of cell activation	10/305	225/28814	4.2	0.000154387	0.006732998	0.005791778	Slfn1/Il2ra/Hspb1/Fas/Fcgr2b/Pglyrp1/Runx1/Adrb2/Il13ra2/Cst7	10
BP	GO:0031640	killing of cells of another organism	6/305	76/28814	7.46	0.000157213	0.006762311	0.005816993	Ltf/Ccl12/Pglyrp1/Fcer2a/Ccl17/Ccl22	6
BP	GO:0032069	regulation of nuclease activity	4/305	27/28814	14	0.000178254	0.007563767	0.006506411	Oasl1/Oas1a/Oas2/Oasl2	4
BP	GO:0097529	myeloid leukocyte migration	10/305	230/28814	4.11	0.000184486	0.007723794	0.006644068	S100a9/S100a8/Cxcl1/Cxcl5/Bst1/Ccl12/Il1r1/Dapk2/Ccl17/Ccl22	10
BP	GO:0002753	cytoplasmic pattern recognition receptor signaling pathway	5/305	52/28814	9.08	0.00022241	0.009189034	0.007904479	Oasl1/Hspa1b/Dhx58/Ddx60/Ddx58	5
BP	GO:0048247	lymphocyte chemotaxis	5/305	54/28814	8.75	0.000266011	0.010430337	0.008972258	Ch25h/Ccl12/Gpr183/Ccl17/Ccl22	5
BP	GO:0002029	desensitization of G protein-coupled receptor signaling pathway	4/305	30/28814	12.6	0.000271484	0.010430337	0.008972258	Ly6g6e/Drd3/Gipr/Adrb2	4
BP	GO:0002507	tolerance induction	4/305	30/28814	12.6	0.000271484	0.010430337	0.008972258	Il2ra/Runx1/Irak3/Icos	4
BP	GO:0022401	negative adaptation of signaling pathway	4/305	30/28814	12.6	0.000271484	0.010430337	0.008972258	Ly6g6e/Drd3/Gipr/Adrb2	4
BP	GO:0039528	cytoplasmic pattern recognition receptor signaling pathway in response to virus	4/305	30/28814	12.6	0.000271484	0.010430337	0.008972258	Oasl1/Dhx58/Ddx60/Ddx58	4
BP	GO:0045824	negative regulation of innate immune response	6/305	84/28814	6.75	0.000272385	0.010430337	0.008972258	Oas1a/Ifi207/Ifi204/Isg15/Dhx58/Irak3	6
BP	GO:0045088	regulation of innate immune response	10/305	242/28814	3.9	0.000277391	0.010494071	0.009027082	Oas1a/Ifi207/Ifi209/Ifi204/Isg15/Irf7/Dhx58/Trim30a/Ifi203/Irak3	10
BP	GO:0002832	negative regulation of response to biotic stimulus	7/305	119/28814	5.56	0.000283246	0.010588019	0.009107897	Ltf/Oas1a/Ifi207/Ifi204/Isg15/Dhx58/Irak3	7
BP	GO:0052547	regulation of peptidase activity	14/305	443/28814	2.99	0.000299446	0.011061892	0.009515526	Ltf/Ngp/S100a9/S100a8/Ifi207/Hspa1b/Ifi204/Tnfrsf23/Fas/Ptgs2/Casp1/Col28a1/Serpinc1/Cst7	14
BP	GO:0061082	myeloid leukocyte cytokine production	4/305	31/28814	12.19	0.000309121	0.011286514	0.009708748	Ddx58/Nr4a3/Irak3/Cd36	4
BP	GO:0002695	negative regulation of leukocyte activation	9/305	202/28814	4.21	0.000321831	0.011615496	0.009991741	Slfn1/Il2ra/Hspb1/Fas/Fcgr2b/Pglyrp1/Runx1/Il13ra2/Cst7	9
BP	GO:0001776	leukocyte homeostasis	7/305	122/28814	5.42	0.000329713	0.011764766	0.010120144	Lgals2/Il2ra/Cxcl5/Tnfrsf23/Fas/Fcgr2b/Gpr183	7
BP	GO:0023058	adaptation of signaling pathway	4/305	32/28814	11.81	0.000350355	0.012360834	0.010632887	Ly6g6e/Drd3/Gipr/Adrb2	4
BP	GO:0002218	activation of innate immune response	5/305	58/28814	8.14	0.000372367	0.012991475	0.011175369	Ifi207/Ifi209/Ifi204/Trim30a/Ifi203	5
BP	GO:0002517	T cell tolerance induction	3/305	14/28814	20.24	0.000392051	0.013527904	0.011636809	Il2ra/Runx1/Icos	3
BP	GO:0032731	positive regulation of interleukin-1 beta production	5/305	61/28814	7.74	0.000471138	0.015738013	0.013537962	Ifi207/Ifi204/Hspb1/Casp1/Cd36	5
BP	GO:0060760	positive regulation of response to cytokine stimulus	5/305	61/28814	7.74	0.000471138	0.015738013	0.013537962	Hspa1b/Irf7/Il1r1/Ddx58/Casp1	5
BP	GO:0070231	T cell apoptotic process	5/305	61/28814	7.74	0.000471138	0.015738013	0.013537962	Il2ra/Siglec1/Tnfrsf23/Fas/Il7r	5
BP	GO:0052548	regulation of endopeptidase activity	12/305	358/28814	3.17	0.000476553	0.015739886	0.013539573	Ltf/S100a9/S100a8/Ifi207/Hspa1b/Ifi204/Tnfrsf23/Fas/Ptgs2/Casp1/Serpinc1/Cst7	12
BP	GO:0097193	intrinsic apoptotic signaling pathway	11/305	308/28814	3.37	0.000482078	0.015739886	0.013539573	S100a9/S100a8/Ifi207/Ifi204/Hspb1/Ptgs2/Cdkn1a/Melk/Crip1/Dapk2/Pou4f1	11
BP	GO:0006924	activation-induced cell death of T cells	3/305	15/28814	18.89	0.000486232	0.015739886	0.013539573	Il2ra/Tnfrsf23/Fas	3
BP	GO:0019730	antimicrobial humoral response	8/305	171/28814	4.42	0.00049814	0.01584592	0.013630785	Ltf/S100a9/Cxcl1/Cxcl5/Ccl12/Pglyrp1/Ccl17/Ccl22	8
BP	GO:2000117	negative regulation of cysteine-type endopeptidase activity	6/305	94/28814	6.03	0.000499601	0.01584592	0.013630785	Ltf/Ifi207/Hspa1b/Ifi204/Ptgs2/Cst7	6
BP	GO:0070098	chemokine-mediated signaling pathway	5/305	62/28814	7.62	0.000508077	0.015953617	0.013723427	Cxcl1/Cxcl5/Ccl12/Ccl17/Ccl22	5
BP	GO:0045744	negative regulation of G protein-coupled receptor signaling pathway	5/305	63/28814	7.5	0.000547152	0.017010453	0.014632525	Ly6g6e/Drd3/Oprm1/Gipr/Adrb2	5
BP	GO:0051346	negative regulation of hydrolase activity	12/305	366/28814	3.1	0.000579101	0.017754591	0.015272638	Ltf/Ngp/Ifi207/Hspa1b/Ifi204/Ptgs2/Angptl4/Apoc1/Fzd10/Col28a1/Serpinc1/Cst7	12
BP	GO:0006898	receptor-mediated endocytosis	10/305	266/28814	3.55	0.000582396	0.017754591	0.015272638	Sele/Drd3/Siglec1/Ifitm3/Fcgr2b/Adrb2/Tfr2/Apoc1/Cd36/Clec9a	10
BP	GO:0070555	response to interleukin-1	6/305	97/28814	5.84	0.000590544	0.017829888	0.015337409	Sele/Ccl12/Il1r1/Irak3/Ccl17/Ccl22	6
BP	GO:0070661	leukocyte proliferation	12/305	368/28814	3.08	0.000607452	0.018165715	0.01562629	Slfn1/Il2ra/Bst1/Ccl12/Cdkn1a/Fcgr2b/Hhex/Zbtb32/Gpr183/Il7r/H2-DMb2/Il27ra	12
BP	GO:0002700	regulation of production of molecular mediator of immune response	8/305	177/28814	4.27	0.000625265	0.018522001	0.015932771	Il1r1/Fcgr2b/Ddx58/Nr4a3/Irak3/Cd36/Il27ra/Il13ra2	8
BP	GO:0070663	regulation of leukocyte proliferation	10/305	269/28814	3.51	0.000635004	0.018634683	0.0160297	Slfn1/Il2ra/Bst1/Ccl12/Cdkn1a/Fcgr2b/Hhex/Gpr183/H2-DMb2/Il27ra	10
BP	GO:0045785	positive regulation of cell adhesion	14/305	482/28814	2.74	0.000688567	0.020019458	0.017220895	Sele/Selp/Il2ra/Il2rg/Piezo1/Runx1/Nr4a3/Il7r/H2-DMb2/Skap1/Cd36/Fstl3/Il27ra/Icos	14
BP	GO:0042742	defense response to bacterium	14/305	483/28814	2.74	0.000702512	0.020237512	0.017408466	Lcn2/Ltf/Cxcl5/Oas1a/Oas2/Isg15/Nlrp10/Pglyrp1/Rnf213/Casp1/Il7r/Cd36/Gbp4/Il27ra	14
BP	GO:0071887	leukocyte apoptotic process	7/305	141/28814	4.69	0.000784806	0.022402641	0.019270927	Il2ra/Siglec1/Tnfrsf23/Fas/Fcgr2b/Nr4a3/Il7r	7
BP	GO:0039535	regulation of RIG-I signaling pathway	3/305	18/28814	15.75	0.00085174	0.023879149	0.020541031	Oasl1/Dhx58/Ddx60	3
BP	GO:0070486	leukocyte aggregation	3/305	18/28814	15.75	0.00085174	0.023879149	0.020541031	S100a9/S100a8/Nr4a3	3
BP	GO:0034121	regulation of toll-like receptor signaling pathway	5/305	71/28814	6.65	0.000946215	0.026293061	0.022617497	Ltf/Irf7/Trim30a/Irak3/Cd36	5
BP	GO:0002827	positive regulation of T-helper 1 type immune response	3/305	19/28814	14.92	0.001003551	0.026601396	0.02288273	Nlrp10/Il1r1/Il27ra	3
BP	GO:0033623	regulation of integrin activation	3/305	19/28814	14.92	0.001003551	0.026601396	0.02288273	Selp/Piezo1/Skap1	3
BP	GO:0035457	cellular response to interferon-alpha	3/305	19/28814	14.92	0.001003551	0.026601396	0.02288273	Oas1a/Ifit1/Ifi204	3

BP	GO:0002237	response to molecule of bacterial origin	21/574	407/28814	2.59	7.95368E-05	0.002747078	0.002161769	Vim/Adm/Irgm1/Ccl5/Nod2/Kcnj8/Fcgr2b/Trim30a/Gch1/Il12rb2/Prdm1/Igtp/Tlr1/Lilrb4a/Ldoc1/Gbp2/Ccl12/S	21
BP	GO:0002064	epithelial cell development	15/574	235/28814	3.2	8.2797E-05	0.00281678	0.00221662	Vim/Pecam1/Cdh5/Abcb1a/Robo4/Bfsp2/Icam1/Gpr4/Hydin/Sox18/Prdm1/Grhl2/Cldn3/Cxcr4/Agt	15
BP	GO:2000181	negative regulation of blood vessel morphogenesis	10/574	112/28814	4.48	8.35771E-05	0.00281678	0.00221662	Flt1/Tek/Tie1/Klf4/Gpr4/Klf2/Ngfr/Ecscr/Angpt2/Agt	10
BP	GO:0002042	cell migration involved in sprouting angiogenesis	7/574	53/28814	6.63	8.3646E-05	0.00281678	0.00221662	Kdr/Klf4/Gata2/Mmrn2/Clec14a/Dll4/Anxa1	7
BP	GO:1901343	negative regulation of vasculature development	10/574	113/28814	4.44	9.00828E-05	0.003008468	0.002367466	Flt1/Tek/Tie1/Klf4/Gpr4/Klf2/Ngfr/Ecscr/Angpt2/Agt	10
BP	GO:1990868	response to chemokine	8/574	72/28814	5.58	9.22839E-05	0.003031865	0.002385878	Cxcl12/Ccl6/Ccl5/Ccl9/Ccl4/Ccl3/Ccl12/Cxcr4	8
BP	GO:1990869	cellular response to chemokine	8/574	72/28814	5.58	9.22839E-05	0.003031865	0.002385878	Cxcl12/Ccl6/Ccl5/Ccl9/Ccl4/Ccl3/Ccl12/Cxcr4	8
BP	GO:0071356	cellular response to tumor necrosis factor	13/574	185/28814	3.53	9.32805E-05	0.003039892	0.002392194	Plvap/Gbp3/Hspa1b/Traip/Ccl6/Ccl5/Ccl9/Ccl4/Ccl3/Ccl12/Mir103-2/Traf1/Eda2r	13
BP	GO:0002823	negative regulation of adaptive immune response based on somatic recombination of immune receptors built from immunoglobulin superfamily domains	7/574	54/28814	6.51	9.44692E-05	0.003046826	0.002397651	Foxj1/Ptprc/Nod2/Fcgr2b/Il7r/Lilrb4a/Il4i1	7
BP	GO:0002693	positive regulation of cellular extravasation	5/574	24/28814	10.46	9.57552E-05	0.003046826	0.002397651	Pecam1/Plvap/Il1r1/Icam1/Ager	5
BP	GO:0002710	negative regulation of T cell mediated immunity	5/574	24/28814	10.46	9.57552E-05	0.003046826	0.002397651	Ptprc/Nod2/Il7r/Lilrb4a/Il4i1	5
BP	GO:0046631	alpha-beta T cell activation	13/574	186/28814	3.51	9.85023E-05	0.003109748	0.002447166	Rasal3/Lef1/Ly9/Ptprc/Il2rg/Il12rb2/Prdm1/Rsad2/Bcl3/Lilrb4a/Hlx/Ager/Anxa1	13
BP	GO:0050673	epithelial cell proliferation	23/574	476/28814	2.43	9.93868E-05	0.003113348	0.00245	Flt1/Eng/Tek/Cxcl12/Kdr/Tie1/Serpinb1b/Gata2/Flt4/Wfdc1/Ccl5/Prkd2/Nod2/Mmrn2/Nupr1/Ngfr/Dll4/Lrg1/Tbx1/Ccl12/Ager/Serpinb5/H	23
BP	GO:0043535	regulation of blood vessel endothelial cell migration	9/574	93/28814	4.86	0.000101107	0.003142862	0.002473225	Kdr/Klf4/Hspb1/Gata2/Prkd2/Mmrn2/Dll4/Angpt2/Anxa1	9
BP	GO:0045777	positive regulation of blood pressure	7/574	55/28814	6.39	0.000106405	0.003277676	0.002579315	Eng/Rarres2/Avpr1a/Avp/Oxt/Glp1r/Agt	7
BP	GO:0016032	viral process	19/574	355/28814	2.69	0.000107066	0.003277676	0.002579315	Apobec3/Ifitm3/Top2a/Oasl2/Icam1/Lef1/Dynlt1b/Ccl5/Oas1b/Trim30a/Rsad2/Bst2/Ccl12/Oas2/Ch25h/Avp/Cxcr4/Trim6/Slpi	19
BP	GO:0070663	regulation of leukocyte proliferation	16/574	269/28814	2.99	0.000111207	0.003378865	0.002658943	Foxj1/Rasal3/Ptprc/Ccl5/Fcgr2b/Il12rb2/Prdm1/Lilrb4a/Csf2rb/Ccl12/Ager/Il4i1/H2-DMb1/Tnfsf9/Slc4a1/Anxa	16
BP	GO:0090049	regulation of cell migration involved in sprouting angiogenesis	6/574	39/28814	7.72	0.000113585	0.003425362	0.002695534	Kdr/Klf4/Gata2/Mmrn2/Dll4/Anxa1	6
BP	GO:0003084	positive regulation of systemic arterial blood pressure	5/574	25/28814	10.04	0.000117741	0.003498477	0.00275307	Eng/Rarres2/Avpr1a/Avp/Agt	5
BP	GO:0150105	protein localization to cell-cell junction	5/574	25/28814	10.04	0.000117741	0.003498477	0.00275307	Pecam1/Cdh5/Abcb1a/Lsr/Dsp	5
BP	GO:0043270	positive regulation of ion transport	18/574	329/28814	2.75	0.000122717	0.003619714	0.002848475	Cnksr3/Lcn2/Abcb1a/Cxcl12/Ccl5/Crh/Avpr1a/Fhl4/Trdn/Ccl12/Kcnmb1/Avp/Tacr2/Cxcr4/Calcr/Ager/Oxt/Agt	18
BP	GO:0044403	biological process involved in symbiotic interaction	16/574	272/28814	2.95	0.000126405	0.003701457	0.002912802	Fn1/Ifitm3/Gbp3/Gbp9/Icam1/Lef1/Dynlt1b/Ccl5/Pglyrp1/Trim30a/Gbp2/Ccl12/Ch25h/Avp/Trim6/Gbp6	16
BP	GO:0006925	inflammatory cell apoptotic process	5/574	26/28814	9.65	0.000143398	0.004168859	0.003280617	Ccl5/Nod2/Fcgr2b/Fas/Anxa1	5
BP	GO:0046651	lymphocyte proliferation	18/574	335/28814	2.7	0.000153607	0.004433755	0.003489072	Cxcl12/Foxj1/Rasal3/Lef1/Ptprc/Ccl5/Fcgr2b/Il7r/Il12rb2/Prdm1/Lilrb4a/Cxcr4/Ager/Il4i1/H2-DMb1/Tnfsf9/Slc4a1/Anxa	18
BP	GO:0032677	regulation of interleukin-8 production	8/574	78/28814	5.15	0.000162892	0.004668416	0.003673735	Klf4/Hspa1b/Ptprc/Prkd2/Nod2/Tlr1/Bcl3/Anxa1	8
BP	GO:0002820	negative regulation of adaptive immune response	7/574	59/28814	5.96	0.00016703	0.004753292	0.003740527	Foxj1/Ptprc/Nod2/Fcgr2b/Il7r/Lilrb4a/Il4i1	7
BP	GO:0002699	positive regulation of immune effector process	17/574	309/28814	2.76	0.000175735	0.004910322	0.003864099	H2-D1/Tek/Il1r1/Gata2/Ptprc/Nod2/H2-Q4/Rsad2/Nppc/Lilrb4a/H2-T10/6030468B19Rik/Hlx/Ddx60/H2-	17
BP	GO:0032943	mononuclear cell proliferation	18/574	339/28814	2.67	0.000177808	0.004910322	0.003864099	Cxcl12/Foxj1/Rasal3/Lef1/Ptprc/Ccl5/Fcgr2b/Il7r/Il12rb2/Prdm1/Lilrb4a/Cxcr4/Ager/Il4i1/H2-DMb1/Tnfsf9/Slc4a1/Anxa	18
BP	GO:0032637	interleukin-8 production	8/574	79/28814	5.08	0.000178128	0.004910322	0.003864099	Klf4/Hspa1b/Ptprc/Prkd2/Nod2/Tlr1/Bcl3/Anxa1	8
BP	GO:0060330	regulation of response to interferon-gamma	4/574	15/28814	13.39	0.000178623	0.004910322	0.003864099	Irgm1/Parp14/Nlrc5/Igtp	4
BP	GO:0060334	regulation of interferon-gamma-mediated signaling pathway	4/574	15/28814	13.39	0.000178623	0.004910322	0.003864099	Irgm1/Parp14/Nlrc5/Igtp	4
BP	GO:0035924	cellular response to vascular endothelial growth factor stimulus	7/574	60/28814	5.86	0.00018588	0.005075269	0.003993901	Flt1/Kdr/Hspb1/Flt4/Prkd2/Dll4/Anxa1	7
BP	GO:0002831	regulation of response to biotic stimulus	19/574	371/28814	2.57	0.000188708	0.005117905	0.004027453	Apobec3/Gbp5/Gbp4/Irf7/Irgm1/Parp14/Ccl5/Nod2/Ikbke/Mmrn2/Tap1/Trim30a/Prdm1/Nlrc5/Igtp/Ifi35/Ddx60/Il4i1/Trim6	19
BP	GO:0062207	regulation of pattern recognition receptor signaling pathway	9/574	101/28814	4.47	0.000190786	0.00513978	0.004044667	Hspa1b/Irf7/Nod2/Trim30a/Rab7b/Rsad2/Tlr1/Ifi35/Ddx60	9
BP	GO:0042098	T cell proliferation	14/574	226/28814	3.11	0.000193325	0.005173671	0.004071337	Cxcl12/Foxj1/Rasal3/Ptprc/Ccl5/Il12rb2/Lilrb4a/Cxcr4/Ager/Il4i1/H2-DMb1/Tnfsf9/Slc4a1/Anxa1	14
BP	GO:0051606	detection of stimulus	16/574	283/28814	2.84	0.000198738	0.005283548	0.004157803	Eng/Cxcl12/Piezo1/Nod2/Pglyrp1/Tcap/Ngfr/Clec7a/Tlr1/Kcnmb2/Guca1b/Rpe65/Rho/Kcnmb1/Cxcr4/Srpx	16
BP	GO:0007162	negative regulation of cell adhesion	17/574	313/28814	2.73	0.000204529	0.005401969	0.004250993	Acer2/Podxl/Cxcl12/Foxj1/Klf4/Hspb1/Ccm2l/Lef1/Ptprc/Myoc/Muc1/Lilrb4a/Angpt2/Hlx/Ager/Il4i1/Anxa1	17
BP	GO:0042060	wound healing	19/574	374/28814	2.55	0.000208984	0.005483802	0.00431539	Fn1/Eng/Pecam1/Kdr/Vwf/Ccm2l/Gata2/Dsp/Wfdc1/Gpr4/Pear1/Clec7a/Lilrb4a/Cldn3/Cxcr4/S100a9/Ager/Slc4a1/Anxa1	19
BP	GO:0034340	response to type I interferon	7/574	62/28814	5.67	0.000228733	0.00596329	0.004692715	Ifitm3/Irf7/Ikbke/Nlrc5/Oas2/Ch25h/Trim6	7
BP	GO:0050921	positive regulation of chemotaxis	11/574	151/28814	3.66	0.000232436	0.006020978	0.004738112	Fn1/Cxcl12/Kdr/Hspb1/Ccl5/Prkd2/Rarres2/Cmklr1/Ccr6/Cxcr4/Ager	11
BP	GO:0090280	positive regulation of calcium ion import	5/574	29/28814	8.65	0.000246435	0.006342953	0.004991485	Cxcl12/Crh/Ccl12/Cxcr4/Calcr	5
BP	GO:0030336	negative regulation of cell migration	16/574	292/28814	2.75	0.00028238	0.007222137	0.005683345	Eng/Arap3/Cxcl12/Tie1/Robo4/Klf4/Mmrn2/Ngfr/Dll4/Bst2/Fas/Angpt2/Ccl12/Mapk15/Cldn3/Ager	16
BP	GO:1990266	neutrophil migration	10/574	130/28814	3.86	0.000286222	0.00727436	0.00572444	Pecam1/Il1r1/Ccl6/Ccl5/Nod2/Ccl9/Ccl4/Ccl3/Ccl12/S100a9	10
BP	GO:0002691	regulation of cellular extravasation	6/574	46/28814	6.55	0.00028986	0.007301643	0.005745911	Pecam1/Plvap/Cxcl12/Il1r1/Icam1/Ager	6
BP	GO:0030947	regulation of vascular endothelial growth factor receptor signaling pathway	5/574	30/28814	8.37	0.000290909	0.007301643	0.005745911	Tek/Kdr/Tmem204/Prkd2/Mmrn2	5
BP	GO:0007204	positive regulation of cytosolic calcium ion concentration	18/574	354/28814	2.55	0.000300824	0.007480511	0.005886668	Ackr4/Kdr/Adm/Gata2/Gpr4/Ptprc/Ccl3/Cmklr1/Avpr1a/Trdn/Ccr6/Avp/Cxcr4/Calcr/Oxt/Htr2b/Glp1r/Agt	18
BP	GO:1903587	regulation of blood vessel endothelial cell proliferation involved in sprouting angiogenesis	4/574	17/28814	11.81	0.000301738	0.007480511	0.005886668	Gata2/Mmrn2/Ngfr/Dll4	4
BP	GO:0010634	positive regulation of epithelial cell migration	11/574	156/28814	3.54	0.000308082	0.007591212	0.005973782	Lcn2/Tek/Kdr/Ctsh/Hspb1/Gata2/Flt4/Prkd2/Ccr6/Agt/Anxa1	11
BP	GO:0032496	response to lipopolysaccharide	19/574	386/28814	2.47	0.000310431	0.007602728	0.005982845	Vim/Adm/Irgm1/Ccl5/Nod2/Kcnj8/Trim30a/Gch1/Il12rb2/Prdm1/Igtp/Lilrb4a/Ldoc1/Gbp2/Ccl12/S100a9/Trim6/Gbp6/Slpi	19

BP	GO:0008645	hexose transmembrane transport	9/556	118/28814	3.95	0.000483162	0.016592686	0.013092969	Ostn/Hnf1a/Slc2a12/Nr4a3/Esr1/Edn1/Trib3/Slc26a5/Slc2a4	9
BP	GO:0090092	regulation of transmembrane receptor protein serine/threonine kinase signaling pathway	14/556	256/28814	2.83	0.000496544	0.016907728	0.013341562	Tfap2b/Hjv/Wfikkn2/Bmp5/Thbs1/Zic2/Nog/Sfrp1/Pparg/Fzd1/Dab2/Npnt/Nkx2-1/Tdgf1	14
BP	GO:0002683	negative regulation of immune system process	20/556	448/28814	2.31	0.000506026	0.017085821	0.013482092	Klrd1/Sh2d1a/Slamf1/Igf2/Cartpt/H2-Aa/Prdm1/Thbs1/Bst2/Cd74/Sfrp1/Pparg/Pde5a/Gpr55/Smpdl3b/Nod2/Vtcn1/Zp3r/Prnp/Fgr	20
BP	GO:1905330	regulation of morphogenesis of an epithelium	7/556	73/28814	4.97	0.000520245	0.01741955	0.013745431	Cdh1/Nog/Six4/Sfrp1/Esr1/Itgax/Agtr1a	7
BP	GO:0090183	regulation of kidney development	5/556	35/28814	7.4	0.000528575	0.017552177	0.013850085	Nog/Six4/Ret/Agtr1a/Wt1	5
BP	GO:0048608	reproductive structure development	21/556	484/28814	2.25	0.000536136	0.017657341	0.013933067	Hnf1a/Lhb/Igf2/Cdh1/Bmp5/Prdm1/Nog/Six4/Rspo3/Sfrp1/Esr1/Pparg/Fzd4/Spint2/Nkx2-1/Ntrk1/Spint1/Retn/Adam1b/Wt1/Dhh	21
BP	GO:0015749	monosaccharide transmembrane transport	9/556	121/28814	3.85	0.00058029	0.018562943	0.014647661	Ostn/Hnf1a/Slc2a12/Nr4a3/Esr1/Edn1/Trib3/Slc26a5/Slc2a4	9
BP	GO:0007611	learning or memory	16/556	322/28814	2.58	0.000582387	0.018562943	0.014647661	Lmx1a/Igf2/Nfatc4/Nog/Tafa2/Sgk1/Crh/Arc/Casp1/Pde5a/Musk/Ntrk1/Itga5/Oprk1/Slc2a4/Prnp	16
BP	GO:0006692	prostanoid metabolic process	6/556	54/28814	5.76	0.00059175	0.018562943	0.014647661	Ptgds/Ptges/Sphk1/Cd74/Akr1c14/Edn1	6
BP	GO:0006693	prostaglandin metabolic process	6/556	54/28814	5.76	0.00059175	0.018562943	0.014647661	Ptgds/Ptges/Sphk1/Cd74/Akr1c14/Edn1	6
BP	GO:1903900	regulation of viral life cycle	10/556	147/28814	3.53	0.000592589	0.018562943	0.014647661	Ch25h/Ifitm1/Rsad2/Ifitm3/Bst2/Oasl2/Trim34a/Parp10/Cd74/Ifitm2	10
BP	GO:0061458	reproductive system development	21/556	488/28814	2.23	0.000595921	0.018562943	0.014647661	Hnf1a/Lhb/Igf2/Cdh1/Bmp5/Prdm1/Nog/Six4/Rspo3/Sfrp1/Esr1/Pparg/Fzd4/Spint2/Nkx2-1/Ntrk1/Spint1/Retn/Adam1b/Wt1/Dhh	21
BP	GO:0051048	negative regulation of secretion	12/556	202/28814	3.08	0.000595973	0.018562943	0.014647661	Cartpt/Rsad2/Crh/Cd74/Sfrp1/Pparg/Tspo/Edn3/Pde1c/Edn1/Oprk1/Avpr2	12
BP	GO:0019373	epoxygenase P450 pathway	5/556	36/28814	7.2	0.000604179	0.018673789	0.014735127	Cyp2a5/Cyp2e1/Cyp2c23/Cyp2g1/Cyp2j8	5
BP	GO:0007492	endoderm development	7/556	75/28814	4.84	0.000613159	0.018806653	0.014839967	Nog/Dusp4/Arc/Dusp5/Dab2/Nkx2-1/Itga5	7
BP	GO:0018108	peptidyl-tyrosine phosphorylation	16/556	324/28814	2.56	0.000622388	0.018945117	0.014949226	Il7/Crlf1/Itgb2l/Igf2/Dyrk3/Cd74/Sfrp1/Ddr2/Pdgfd/Musk/Ntrk1/Itga5/Nod2/Agtr1a/Prnp/Fgr	16
BP	GO:0032368	regulation of lipid transport	9/556	123/28814	3.79	0.000653506	0.019595836	0.015462696	Pomc/Thbs1/Ptges/Crh/Tspo/Dab2/Edn1/Agtr1a/Retn	9
BP	GO:0003044	regulation of systemic arterial blood pressure mediated by a chemical signal	6/556	55/28814	5.65	0.00065352	0.019595836	0.015462696	Edn3/Nkx2-1/Edn1/Agtr1a/Avpr2/Serpinf2	6
BP	GO:0001756	somitogenesis	7/556	76/28814	4.77	0.000664306	0.019626344	0.015486769	Dll3/Aldh1a2/Ifitm1/Tcap/Tbx18/Sfrp1/Meox1	7
BP	GO:0021766	hippocampus development	7/556	76/28814	4.77	0.000664306	0.019626344	0.015486769	Lhx5/Lmx1a/Neurod1/Nr4a3/Trp73/Tmem108/Nkx2-1	7
BP	GO:0046394	carboxylic acid biosynthetic process	15/556	295/28814	2.64	0.000676374	0.019837025	0.015653014	Fads2b/Otc/Hnf1a/Rdh16/Aldh1a2/Ptgds/Acsm5/Ptges/Sphk1/Alox12b/Cd74/Edn1/Trib3/Cyp2c23/Pklr	15
BP	GO:0018212	peptidyl-tyrosine modification	16/556	327/28814	2.54	0.000686785	0.019951911	0.015743668	Il7/Crlf1/Itgb2l/Igf2/Dyrk3/Cd74/Sfrp1/Ddr2/Pdgfd/Musk/Ntrk1/Itga5/Nod2/Agtr1a/Prnp/Fgr	16
BP	GO:0046620	regulation of organ growth	9/556	124/28814	3.76	0.000692847	0.019951911	0.015743668	Il7/Igf2/Dusp9/Trp73/Nog/Ccn4/Edn1/Myh6/Wt1	9
BP	GO:0001818	negative regulation of cytokine production	15/556	296/28814	2.63	0.000700154	0.019951911	0.015743668	Pomc/Slamf1/Cdh3/Twist1/Prg4/Thbs1/Bst2/Fbln1/Pparg/Tspo/Homer2/Gbp4/Nlrp3/Nod2/Prnp	15
BP	GO:0016053	organic acid biosynthetic process	15/556	296/28814	2.63	0.000700154	0.019951911	0.015743668	Fads2b/Otc/Hnf1a/Rdh16/Aldh1a2/Ptgds/Acsm5/Ptges/Sphk1/Alox12b/Cd74/Edn1/Trib3/Cyp2c23/Pklr	15
BP	GO:0007200	phospholipase C-activating G protein-coupled receptor signaling pathway	8/556	101/28814	4.1	0.000768198	0.021736767	0.017152063	Npr3/Esr1/Gpr55/Gpr139/Edn1/Oprk1/Trhr2/Agtr1a	8
BP	GO:0002604	regulation of dendritic cell antigen processing and presentation	3/556	10/28814	15.55	0.000775066	0.021746137	0.017159457	Thbs1/Cd74/Nod2	3
BP	GO:0071634	regulation of transforming growth factor beta production	5/556	38/28814	6.82	0.000779354	0.021746137	0.017159457	Itgb6/Cdh3/Lum/Fbln1/Serpinf2	5
BP	GO:0052372	modulation by symbiont of entry into host	6/556	57/28814	5.46	0.000791947	0.021945136	0.017316483	Ch25h/Ifitm1/Ifitm3/Trim34a/Cd74/Ifitm2	6
BP	GO:0006633	fatty acid biosynthetic process	10/556	153/28814	3.39	0.000808287	0.02224451	0.017552714	Fads2b/Hnf1a/Ptgds/Acsm5/Ptges/Sphk1/Alox12b/Cd74/Edn1/Trib3	10
BP	GO:0072080	nephron tubule development	8/556	102/28814	4.06	0.000819763	0.022406866	0.017680826	Tfap2b/Aqp1/Nog/Six4/Npnt/Adamts16/Agtr1a/Wt1	8
BP	GO:0048562	embryonic organ morphogenesis	16/556	333/28814	2.49	0.000832781	0.022544869	0.017789722	Twist1/Aldh1a2/Neurod1/Nr4a3/Tcap/Nog/Tbx18/Wnt9a/Six4/Frzb/Tead2/Rarb/Col11a1/Col2a1/Edn1/Myo3b	16
BP	GO:0048738	cardiac muscle tissue development	14/556	270/28814	2.69	0.000836034	0.022544869	0.017789722	Aldh1a2/Bmp5/Tcap/Trp73/Nog/Tbx18/Mylk3/Ccn4/Rarb/Col11a1/Edn1/Tdgf1/Myh6/Wt1	14
BP	GO:0048863	stem cell differentiation	13/556	240/28814	2.81	0.000850708	0.022787633	0.017981282	Zscan10/Twist1/Aldh1a2/Frzb/Sfrp1/Esr1/Fzd1/Tead2/Edn3/Epop/Slc4a11/Ret/Edn1	13
BP	GO:0033674	positive regulation of kinase activity	19/556	435/28814	2.26	0.000896285	0.023730664	0.018725409	Cartpt/Thbs1/Arhgef5/Cd74/Ddr2/Pdgfd/Fzd4/Edn3/Pde5a/Psrc1/Ret/Musk/Ntrk1/Edn1/Nod2/Tdgf1/Agtr1a/Prnp/Fgr	19
BP	GO:0045766	positive regulation of angiogenesis	11/556	183/28814	3.12	0.000903632	0.023730664	0.018725409	Aqp1/Itgb2l/Igf2/Ccbe1/Thbs1/Sphk1/Cyp1b1/Ntrk1/Itga5/Itgax/Agtr1a	11
BP	GO:1904018	positive regulation of vasculature development	11/556	183/28814	3.12	0.000903632	0.023730664	0.018725409	Aqp1/Itgb2l/Igf2/Ccbe1/Thbs1/Sphk1/Cyp1b1/Ntrk1/Itga5/Itgax/Agtr1a	11
BP	GO:0043500	muscle adaptation	9/556	129/28814	3.62	0.000919622	0.023993786	0.018933033	Myoc/Nr4a3/Tcap/Mlip/Pparg/Pde5a/Ccn4/Edn1/Myh6	9
BP	GO:0043406	positive regulation of MAP kinase activity	9/556	130/28814	3.59	0.000971496	0.025183675	0.019871952	Thbs1/Arhgef5/Pdgfd/Fzd4/Edn3/Pde5a/Edn1/Nod2/Tdgf1	9
BP	GO:0072006	nephron development	10/556	157/28814	3.3	0.000985114	0.025210094	0.019892799	Tfap2b/Aqp1/Nog/Six4/Pdgfd/Npnt/Adamts16/Ret/Agtr1a/Wt1	10
BP	GO:0048265	response to pain	5/556	40/28814	6.48	0.000989859	0.025210094	0.019892799	Thbs1/Tspo/Ret/Ntrk1/Pirt	5
BP	GO:0046718	viral entry into host cell	8/556	105/28814	3.95	0.000991338	0.025210094	0.019892799	Ch25h/Ifitm1/Ifitm3/Trim34a/Cd74/Ifitm2/Spint1/Agtr1a	8
BP	GO:0045667	regulation of osteoblast differentiation	10/556	158/28814	3.28	0.001033943	0.025894578	0.020432912	Ostn/Twist1/Ranbp3l/Nog/Sfrp1/Ddr2/Pparg/Npnt/Tent5a/Ccn4	10
BP	GO:0031589	cell-substrate adhesion	17/556	373/28814	2.36	0.001038269	0.025894578	0.020432912	Itgb6/Myoc/Itgb2l/Col13a1/Thbs1/Fbln1/Sfrp1/Fzd4/Dab2/Dmp1/Nid2/Npnt/Lamb3/Itga5/BC024139/Itgax/Tdgf1	17
BP	GO:0014015	positive regulation of gliogenesis	7/556	82/28814	4.42	0.0010464	0.025894578	0.020432912	Egr2/Trp73/Nog/Pparg/Tspo/Prkch/Spint1	7
BP	GO:0051901	positive regulation of mitochondrial depolarization	3/556	11/28814	14.13	0.001050477	0.025894578	0.020432912	Myoc/Dcn/Tspo	3
BP	GO:0061299	retina vasculature morphogenesis in camera-type eye	3/556	11/28814	14.13	0.001050477	0.025894578	0.020432912	Cyp1b1/Lama1/Fzd4	3
BP	GO:0030903	notochord development	4/556	24/28814	8.64	0.001072245	0.026110784	0.020603516	Col13a1/Nog/Tead2/Col2a1	4
BP	GO:0051882	mitochondrial depolarization	4/556	24/28814	8.64	0.001072245	0.026110784	0.020603516	Myoc/Dcn/Casp1/Tspo	4

BP	GO:1900225	regulation of NLRP3 inflammasome complex assembly	3/305	19/28814	14.92	0.001003551	0.026601396	0.02288273	Trim30a/Eif2ak2/Cd36	3
BP	GO:1990868	response to chemokine	5/305	72/28814	6.56	0.001008142	0.026601396	0.02288273	Cxcl1/Cxcl5/Ccl12/Ccl17/Ccl22	5
BP	GO:1990869	cellular response to chemokine	5/305	72/28814	6.56	0.001008142	0.026601396	0.02288273	Cxcl1/Cxcl5/Ccl12/Ccl17/Ccl22	5
BP	GO:0072676	lymphocyte migration	6/305	108/28814	5.25	0.00103821	0.027166495	0.023368832	Ch25h/Ccl12/Gpr183/Ccl17/Il27ra/Ccl22	6
BP	GO:0019233	sensory perception of pain	7/305	149/28814	4.44	0.001084175	0.027904187	0.024003401	Fcgr4/Chrna5/Htr2a/Ptgs2/Oprm1/Ndn/Adrb2	7
BP	GO:2001056	positive regulation of cysteine-type endopeptidase activity	7/305	149/28814	4.44	0.001084175	0.027904187	0.024003401	S100a9/S100a8/Ifi207/Ifi204/Tnfrsf23/Fas/Casp1	7
BP	GO:0071867	response to monoamine	5/305	74/28814	6.38	0.001140957	0.02868345	0.024673729	Drd3/Htr2a/Oprm1/Nr4a3/Adrb2	5
BP	GO:0071869	response to catecholamine	5/305	74/28814	6.38	0.001140957	0.02868345	0.024673729	Drd3/Htr2a/Oprm1/Nr4a3/Adrb2	5
BP	GO:0032760	positive regulation of tumor necrosis factor production	6/305	110/28814	5.15	0.001141857	0.02868345	0.024673729	Oas1a/Oas2/Hspb1/Ddx58/Cd36/Cd2	6
BP	GO:0002643	regulation of tolerance induction	3/305	20/28814	14.17	0.001171442	0.028963294	0.024914453	Il2ra/Runx1/Irak3	3
BP	GO:0051701	biological process involved in interaction with host	7/305	151/28814	4.38	0.001171445	0.028963294	0.024914453	Ch25h/Siglec1/Ifitm3/Trim34a/Trim30a/Pglyrp1/Dynlt1b	7
BP	GO:1903557	positive regulation of tumor necrosis factor superfamily cytokine production	6/305	112/28814	5.06	0.001253252	0.030743848	0.026446099	Oas1a/Oas2/Hspb1/Ddx58/Cd36/Cd2	6
BP	GO:1903706	regulation of hemopoiesis	12/305	402/28814	2.82	0.001298974	0.031618434	0.027198425	Il2ra/Hspa1b/Il2rg/Isg15/Hspb1/Fas/Pglyrp1/Runx1/Il7r/Duxbl1/Fstl3/Pou4f1	12
BP	GO:0039531	regulation of viral-induced cytoplasmic pattern recognition receptor signaling pathway	3/305	21/28814	13.5	0.00135603	0.032014552	0.027539169	Oasl1/Dhx58/Ddx60	3
BP	GO:0042026	protein refolding	3/305	21/28814	13.5	0.00135603	0.032014552	0.027539169	Hspa1b/Hspb1/Hspa5	3
BP	GO:0044546	NLRP3 inflammasome complex assembly	3/305	21/28814	13.5	0.00135603	0.032014552	0.027539169	Trim30a/Eif2ak2/Cd36	3
BP	GO:0048148	behavioral response to cocaine	3/305	21/28814	13.5	0.00135603	0.032014552	0.027539169	Drd3/Htr2a/Homer1	3
BP	GO:0007286	spermatid development	9/305	248/28814	3.43	0.001386362	0.032486385	0.027945043	Cfap97d1/Prm1/Brip1/Tssk1/Rnf17/Meioc/Pcsk4/Slc26a8/Taf7l	9
BP	GO:0002691	regulation of cellular extravasation	4/305	46/28814	8.21	0.001415555	0.032771439	0.028190249	Sele/Selp/Il1r1/Il27ra	4
BP	GO:0045619	regulation of lymphocyte differentiation	8/305	201/28814	3.76	0.0014194	0.032771439	0.028190249	Il2ra/Il2rg/Hspb1/Fas/Pglyrp1/Runx1/Il7r/Duxbl1	8
BP	GO:2000379	positive regulation of reactive oxygen species metabolic process	5/305	78/28814	6.06	0.00144488	0.033116224	0.028486835	Lcn2/Cxcl1/Cdkn1a/Slc5a3/Cd36	5
BP	GO:0051092	positive regulation of NF-kappaB transcription factor activity	7/305	157/28814	4.21	0.00146666	0.033371828	0.028706708	Ltf/Hspa1b/Trim34a/Trim30a/Eif2ak2/Irak3/Cd36	7
BP	GO:0071496	cellular response to external stimulus	10/305	301/28814	3.14	0.001483004	0.03350096	0.028817788	Ifi207/Cyp27b1/Ifi204/Fas/Cdkn1a/Angptl4/Piezo1/Eif2ak2/Hspa5/Scx	10
BP	GO:0032652	regulation of interleukin-1 production	6/305	116/28814	4.89	0.001500797	0.033660739	0.028955232	Ifi207/Ifi204/Hspb1/Nlrp10/Casp1/Cd36	6
BP	GO:0061081	positive regulation of myeloid leukocyte cytokine production involved in immune response	3/305	22/28814	12.88	0.00155791	0.034693877	0.029843946	Ddx58/Nr4a3/Cd36	3
BP	GO:0031669	cellular response to nutrient levels	8/305	205/28814	3.69	0.001607352	0.035515031	0.030550308	Ifi207/Cyp27b1/Ifi204/Fas/Cdkn1a/Angptl4/Eif2ak2/Hspa5	8
BP	GO:0071347	cellular response to interleukin-1	5/305	80/28814	5.9	0.001617404	0.035515031	0.030550308	Ccl12/Il1r1/Irak3/Ccl17/Ccl22	5
BP	GO:0051702	biological process involved in interaction with symbiont	6/305	119/28814	4.76	0.001709637	0.037279575	0.032068183	Ltf/Cxcl1/Cxcl5/Ccl12/Ifi27/Gpx2	6
BP	GO:0048515	spermatid differentiation	9/305	257/28814	3.31	0.001769648	0.037685568	0.032417421	Cfap97d1/Prm1/Brip1/Tssk1/Rnf17/Meioc/Pcsk4/Slc26a8/Taf7l	9
BP	GO:0140632	inflammasome complex assembly	3/305	23/28814	12.32	0.001777648	0.037685568	0.032417421	Trim30a/Eif2ak2/Cd36	3
BP	GO:1903428	positive regulation of reactive oxygen species biosynthetic process	3/305	23/28814	12.32	0.001777648	0.037685568	0.032417421	Lcn2/Slc5a3/Cd36	3
BP	GO:0043029	T cell homeostasis	4/305	49/28814	7.71	0.001792883	0.037685568	0.032417421	Lgals2/Il2ra/Tnfrsf23/Fas	4
BP	GO:0002699	positive regulation of immune effector process	10/305	309/28814	3.06	0.001798527	0.037685568	0.032417421	Cxcl1/Dhx58/Il1r1/Ddx60/Ddx58/Nr4a3/H2-DMb2/Fcer2a/Cd36/Il13ra2	10
BP	GO:0006959	humoral immune response	12/305	418/28814	2.71	0.001800266	0.037685568	0.032417421	Ltf/S100a9/Cxcl1/Cxcl5/Ccl12/Fcgr2b/Pglyrp1/Gpr183/Fcer2a/Ccl17/Ccl22/C1s2	12
BP	GO:0032612	interleukin-1 production	6/305	122/28814	4.65	0.001939827	0.040338114	0.034699162	Ifi207/Ifi204/Hspb1/Nlrp10/Casp1/Cd36	6
BP	GO:0001906	cell killing	9/305	261/28814	3.26	0.001965187	0.040596632	0.034921541	Ltf/Cxcl1/Cxcl5/Ccl12/Pglyrp1/Il7r/Fcer2a/Ccl17/Ccl22	9
BP	GO:0010466	negative regulation of peptidase activity	9/305	262/28814	3.25	0.002016671	0.041387898	0.035602194	Ltf/Ngp/Ifi207/Hspa1b/Ifi204/Ptgs2/Col28a1/Serpinc1/Cst7	9
BP	GO:0009267	cellular response to starvation	7/305	167/28814	3.96	0.002084299	0.042498045	0.036557152	Ifi207/Ifi204/Fas/Cdkn1a/Angptl4/Eif2ak2/Hspa5	7
BP	GO:0002224	toll-like receptor signaling pathway	6/305	124/28814	4.57	0.00210582	0.042659839	0.036696328	Ltf/Oas1a/Irf7/Trim30a/Irak3/Cd36	6
BP	GO:0032649	regulation of interferon-gamma production	6/305	125/28814	4.53	0.00219273	0.044135724	0.037965896	Isg15/Il1r1/Pglyrp1/Runx1/Il27ra/Cd2	6
BP	GO:0032729	positive regulation of interferon-gamma production	5/305	86/28814	5.49	0.002225887	0.044298157	0.038105622	Isg15/Il1r1/Runx1/Il27ra/Cd2	5
BP	GO:0046596	regulation of viral entry into host cell	4/305	52/28814	7.27	0.002234774	0.044298157	0.038105622	Ch25h/Ifitm3/Trim34a/Trim30a	4
BP	GO:0031667	response to nutrient levels	11/305	373/28814	2.79	0.002243123	0.044298157	0.038105622	Ifi207/Cyp27b1/Ifi204/Fas/Cdkn1a/Oprm1/Angptl4/Eif2ak2/Hspa5/Adrb2/Serpinc1	11
BP	GO:0140448	signaling receptor ligand precursor processing	3/305	25/28814	11.34	0.002272854	0.044384598	0.038179979	Pcsk1/Casp1/Pcsk4	3
BP	GO:0009266	response to temperature stimulus	7/305	170/28814	3.89	0.002304041	0.044384598	0.038179979	Hspa1b/Hspb1/Htr2a/Il1r1/Adrb2/Cirbp/Rbm3	7
BP	GO:0010950	positive regulation of endopeptidase activity	7/305	170/28814	3.89	0.002304041	0.044384598	0.038179979	S100a9/S100a8/Ifi207/Ifi204/Tnfrsf23/Fas/Casp1	7
BP	GO:0051250	negative regulation of lymphocyte activation	7/305	170/28814	3.89	0.002304041	0.044384598	0.038179979	Slfn1/Il2ra/Hspb1/Fas/Fcgr2b/Pglyrp1/Runx1	7
BP	GO:0043123	positive regulation of I-kappaB kinase/NF-kappaB signaling	6/305	127/28814	4.46	0.002374636	0.045465586	0.039109854	Ltf/Trim34a/Trim30a/Casp1/Cd36/S100a4	6
BP	GO:0031397	negative regulation of protein ubiquitination	5/305	88/28814	5.37	0.002461453	0.046842199	0.040294027	Hspa1b/Isg15/Dtx3l/Parp10/Gbp4	5

BP	GO:0042102	positive regulation of T cell proliferation	9/574	108/28814	4.18	0.00031612	0.007695434	0.006055798	Rasal3/Ptprc/Ccl5/Il12rb2/Ager/H2-DMb1/Tnfsf9/Slc4a1/Anxa	9
BP	GO:0002687	positive regulation of leukocyte migration	11/574	157/28814	3.52	0.000325463	0.007875425	0.006197439	Pecam1/Plvap/Cxcl12/Il1r1/Icam1/Ccl5/Rarres2/Cmklr1/Ccr6/Ccl12/Ager	11
BP	GO:0010595	positive regulation of endothelial cell migration	9/574	109/28814	4.14	0.000338595	0.00814443	0.006409128	Lcn2/Tek/Kdr/Hspb1/Gata2/Flt4/Prkd2/Agt/Anxa1	9
BP	GO:2000352	negative regulation of endothelial cell apoptotic process	5/574	31/28814	8.1	0.000341211	0.008158791	0.006420429	Cdh5/Tek/Kdr/Icam1/Gata2	5
BP	GO:0032680	regulation of tumor necrosis factor production	12/574	185/28814	3.26	0.000359283	0.008540368	0.006720705	Hspb1/Ptprc/Nod2/Lpl/Trim30a/Ccl4/Ccl3/Tlr1/Bcl3/Lilrb4a/Oas2/Ager	12
BP	GO:0032760	positive regulation of tumor necrosis factor production	9/574	110/28814	4.11	0.000362373	0.008563456	0.006738874	Hspb1/Ptprc/Nod2/Lpl/Ccl4/Ccl3/Tlr1/Oas2/Ager	9
BP	GO:0090287	regulation of cellular response to growth factor stimulus	16/574	300/28814	2.68	0.000380736	0.00891965	0.007019175	Eng/Cdh5/Tek/Kdr/Fbn2/Tmem204/Prkd2/Mmrn2/Ngfr/Chrdl2/Lrg1/Kcp/Wfikkn2	16
BP	GO:0070431	nucleotide-binding oligomerization domain containing 2 signaling pathway	4/574	18/28814	11.16	0.000381861	0.00891965	0.007019175	Hspa1b/Irgm1/Nod2/Igtp	4
BP	GO:0042129	regulation of T cell proliferation	12/574	187/28814	3.22	0.000396008	0.009196943	0.007237387	Foxj1/Rasal3/Ptprc/Ccl5/Il12rb2/Lilrb4a/Ager/Il4i1/H2-DMb1/Tnfsf9/Slc4a1/Anxa1	12
BP	GO:0014065	phosphatidylinositol 3-kinase signaling	10/574	136/28814	3.69	0.000410841	0.009486725	0.007465426	Flt1/Fn1/Tek/Kdr/Klf4/Ccl5/Pear1/Myoc/Htr2b/Agt	10
BP	GO:1903557	positive regulation of tumor necrosis factor superfamily cytokine production	9/574	112/28814	4.03	0.000414069	0.009486725	0.007465426	Hspb1/Ptprc/Nod2/Lpl/Ccl4/Ccl3/Tlr1/Oas2/Ager	9
BP	GO:1903555	regulation of tumor necrosis factor superfamily cytokine production	12/574	188/28814	3.2	0.000415528	0.009486725	0.007465426	Hspb1/Ptprc/Nod2/Lpl/Trim30a/Ccl4/Ccl3/Tlr1/Bcl3/Lilrb4a/Oas2/Ager	12
BP	GO:0051480	regulation of cytosolic calcium ion concentration	19/574	397/28814	2.4	0.000438725	0.009913269	0.007801088	Ackr4/Kdr/Adm/Gata2/Gpr4/Ptprc/Scgn/Ccl3/Cmklr1/Avpr1a/Trdn/Ccr6/Avp/Cxcr4/Calcr/Oxt/Htr2b/Glp1r/A	19
BP	GO:0050670	regulation of lymphocyte proliferation	14/574	245/28814	2.87	0.000439118	0.009913269	0.007801088	Foxj1/Rasal3/Ptprc/Ccl5/Fcgr2b/Il12rb2/Prdm1/Lilrb4a/Ager/Il4i1/H2-DMb1/Tnfsf9/Slc4a1/Anxa	14
BP	GO:2000146	negative regulation of cell motility	16/574	305/28814	2.63	0.000456147	0.010240499	0.008058596	Eng/Arap3/Cxcl12/Tie1/Robo4/Klf4/Mmrn2/Ngfr/Dll4/Bst2/Fas/Angpt2/Ccl12/Ma	16
BP	GO:0060340	positive regulation of type I interferon-mediated signaling pathway	4/574	19/28814	10.57	0.000476105	0.010577972	0.008324165	Irf7/Ikbke/Nlrc5/Trim6	4
BP	GO:0032640	tumor necrosis factor production	12/574	191/28814	3.15	0.000479032	0.010577972	0.008324165	Hspb1/Ptprc/Nod2/Lpl/Trim30a/Ccl4/Ccl3/Tlr1/Bcl3/Lilrb4a/Oas2/Ager	12
BP	GO:0043122	regulation of I-kappaB kinase/NF-kappaB signaling	12/574	191/28814	3.15	0.000479032	0.010577972	0.008324165	Hspb1/Nod2/Trim30a/Tnfsf10/Lilrb4a/Edar/Olfm4/Unc5cl/Ticam2/Traf1/Trim6/Ed	12
BP	GO:0001937	negative regulation of endothelial cell proliferation	7/574	70/28814	5.02	0.000486295	0.010622267	0.008359022	Flt1/Eng/Mmrn2/Ngfr/Dll4/Ccl12/Ager	7
BP	GO:0035904	aorta development	7/574	70/28814	5.02	0.000486295	0.010622267	0.008359022	Eng/Ngfr/Prdm1/Dll4/Tbx1/Cxcr4/Tfap2b	7
BP	GO:0046634	regulation of alpha-beta T cell activation	9/574	115/28814	3.93	0.000502871	0.010887354	0.008567628	Rasal3/Ptprc/Il2rg/Il12rb2/Prdm1/Lilrb4a/Hlx/Ager/Anxa1	9
BP	GO:0006874	cellular calcium ion homeostasis	22/574	500/28814	2.21	0.000506646	0.010887354	0.008567628	Cdh5/Ackr4/Kdr/Adm/Gata2/Gpr4/Ptprc/Ccl5/Scgn/Ccl3/Cmklr1/Avpr1a/Trdn/Ccr6/Ccl12/Avp/Cxcr4/Calcr/Oxt/Htr2b/Glp1r/Agt	22
BP	GO:0032944	regulation of mononuclear cell proliferation	14/574	249/28814	2.82	0.000515808	0.010887354	0.008567628	Foxj1/Rasal3/Ptprc/Ccl5/Fcgr2b/Il12rb2/Prdm1/Lilrb4a/Ager/Il4i1/H2-DMb1/Tnfsf9/Slc4a1/Anxa1	14
BP	GO:0032675	regulation of interleukin-6 production	11/574	166/28814	3.33	0.000522484	0.010887354	0.008567628	Foxj1/Nod2/Lpl/Trim30a/Klf2/Rab7b/Tlr1/Lilrb4a/Ager/Ticam2/Tnfsf9	11
BP	GO:0019932	second-messenger-mediated signaling	16/574	309/28814	2.6	0.000525393	0.010887354	0.008567628	Ackr4/Kdr/Irgm1/Sctr/Ptprc/Crh/Nppc/Igtp/Cmklr1/Avpr1a/3425401B19Rik/Rxfp2/Ccr6/Cxcr4/Htr2b/Glp1r	16
BP	GO:0002833	positive regulation of response to biotic stimulus	12/574	193/28814	3.12	0.000525757	0.010887354	0.008567628	Gbp5/Irf7/Irgm1/Nod2/Ikbke/Mmrn2/Trim30a/Nlrc5/Igtp/Ifi35/Ddx60/Trim6	12
BP	GO:0040013	negative regulation of locomotion	17/574	340/28814	2.51	0.00052983	0.010887354	0.008567628	Eng/Arap3/Cxcl12/Tie1/Robo4/Klf4/Sema3g/Mmrn2/Ngfr/Dll4/Bst2/Fas/Angpt2/Ccl12/Mapk15/Cldn3/Ager	17
BP	GO:0001913	T cell mediated cytotoxicity	7/574	71/28814	4.95	0.000530355	0.010887354	0.008567628	H2-D1/Ctsh/Ptprc/H2-Q4/Il7r/H2-T10/Ager	7
BP	GO:0034121	regulation of toll-like receptor signaling pathway	7/574	71/28814	4.95	0.000530355	0.010887354	0.008567628	Irf7/Nod2/Trim30a/Rab7b/Rsad2/Tlr1/Ifi35	7
BP	GO:0032652	regulation of interleukin-1 production	9/574	116/28814	3.89	0.000535729	0.010887354	0.008567628	Gbp5/Hspb1/Serpinb1b/Nod2/Lpl/Ccl3/Lilrb4a/Ager/Anxa1	9
BP	GO:0035710	CD4-positive, alpha-beta T cell activation	9/574	116/28814	3.89	0.000535729	0.010887354	0.008567628	Lef1/Ly9/Il2rg/Rsad2/Bcl3/Lilrb4a/Hlx/Ager/Anxa1	9
BP	GO:0051952	regulation of amine transport	9/574	116/28814	3.89	0.000535729	0.010887354	0.008567628	Cxcl12/Per2/Crh/Syt15/Avpr1a/Avp/Tacr2/Oxt/Agt	9
BP	GO:0060840	artery development	9/574	116/28814	3.89	0.000535729	0.010887354	0.008567628	Adgrf5/Eng/Hpgd/Ngfr/Prdm1/Dll4/Tbx1/Cxcr4/Tfap2b	9
BP	GO:0051090	regulation of DNA-binding transcription factor activity	20/574	436/28814	2.3	0.00053615	0.010887354	0.008567628	Foxj1/Arhgef5/Klf4/Hspa1b/Icam1/Traip/Prkd2/Nod2/Ikbke/Fcgr2b/Trim30a/Nupr1/Rab7b/Nlrc5/Cmklr1/Bcl3/Ager/Traf1/Trim6/Eda2r	20
BP	GO:0071706	tumor necrosis factor superfamily cytokine production	12/574	194/28814	3.11	0.000550521	0.011113722	0.008745765	Hspb1/Ptprc/Nod2/Lpl/Trim30a/Ccl4/Ccl3/Tlr1/Bcl3/Lilrb4a/Oas2/Ager	12
BP	GO:0002285	lymphocyte activation involved in immune response	13/574	222/28814	2.94	0.000552798	0.011113722	0.008745765	Icam1/Lef1/Ly9/Ptprc/Mfng/Pglyrp1/Bcl3/Lilrb4a/6030468B19Rik/Ccr6/Hlx/H2-DMb1/Anxa1	13
BP	GO:0051954	positive regulation of amine transport	6/574	52/28814	5.79	0.000569642	0.011339533	0.008923463	Cxcl12/Avpr1a/Avp/Tacr2/Oxt/Agt	6
BP	GO:0061028	establishment of endothelial barrier	6/574	52/28814	5.79	0.000569642	0.011339533	0.008923463	Pecam1/Cdh5/Robo4/Icam1/Sox18/Cldn3	6
BP	GO:0002923	regulation of humoral immune response mediated by circulating immunoglobulin	4/574	20/28814	10.04	0.000585805	0.011435925	0.008999317	Foxj1/Ptprc/Nod2/Fcgr2b	4
BP	GO:0003159	morphogenesis of an endothelium	4/574	20/28814	10.04	0.000585805	0.011435925	0.008999317	Lcn2/Kdr/Dll4/Cxcr4	4
BP	GO:0061154	endothelial tube morphogenesis	4/574	20/28814	10.04	0.000585805	0.011435925	0.008999317	Lcn2/Kdr/Dll4/Cxcr4	4
BP	GO:0070423	nucleotide-binding oligomerization domain containing signaling pathway	4/574	20/28814	10.04	0.000585805	0.011435925	0.008999317	Hspa1b/Irgm1/Nod2/Igtp	4
BP	GO:0051271	negative regulation of cellular component movement	16/574	314/28814	2.56	0.000624464	0.012132012	0.009547092	Eng/Arap3/Cxcl12/Tie1/Robo4/Klf4/Mmrn2/Ngfr/Dll4/Bst2/Fas/Angpt2/Ccl12/Mapk15/Cldn3/Ager	16
BP	GO:0032651	regulation of interleukin-1 beta production	8/574	95/28814	4.23	0.00062911	0.012155416	0.009565509	Gbp5/Hspb1/Serpinb1b/Nod2/Lpl/Ccl3/Lilrb4a/Ager	8
BP	GO:1904036	negative regulation of epithelial cell apoptotic process	6/574	53/28814	5.68	0.000631685	0.012155416	0.009565509	Cdh5/Tek/Kdr/Icam1/Gata2/Nupr1	6
BP	GO:0051251	positive regulation of lymphocyte activation	20/574	443/28814	2.27	0.000653457	0.012514777	0.009848303	Rasal3/Lef1/Ptprc/Ccl5/Nod2/Il2rg/Il7r/Il12rb2/Rag1/Prdm1/Clec7a/Lilrb4a/Hlx/Ager/Il4i1/Igha/H2-DMb1/Tnfsf9/Slc4a1/Anxa	20
BP	GO:0002685	regulation of leukocyte migration	13/574	227/28814	2.87	0.000680659	0.012875789	0.010132395	Pecam1/Plvap/Cxcl12/Il1r1/Icam1/Ccl5/Nod2/Rarres2/Cmklr1/Ccr6/Ccl12/Ager/Anx	13
BP	GO:0001894	tissue homeostasis	15/574	286/28814	2.63	0.000680851	0.012875789	0.010132395	Adgrf5/Prom1/Abcb1a/Kdr/Ctsh/Lsr/Lrrk1/Gata2/Nod2/Col9a1/Rpe65/Rcn3/Rho/C	15
BP	GO:0001885	endothelial cell development	7/574	74/28814	4.75	0.000681866	0.012875789	0.010132395	Pecam1/Cdh5/Robo4/Icam1/Sox18/Cldn3/Agt	7
BP	GO:0001946	lymphangiogenesis	4/574	21/28814	9.56	0.000712308	0.013203841	0.01039055	Tie1/Flt4/Sox18/Clec14a	4
BP	GO:0035872	nucleotide-binding domain, leucine rich repeat containing receptor signaling pathway	4/574	21/28814	9.56	0.000712308	0.013203841	0.01039055	Hspa1b/Irgm1/Nod2/Igtp	4

BP	GO:0006636	unsaturated fatty acid biosynthetic process	5/556	41/28814	6.32	0.001109661	0.02664869	0.021027967	Ptgds/Ptges/Sphk1/Cd74/Edn1	5
BP	GO:0071604	transforming growth factor beta production	5/556	41/28814	6.32	0.001109661	0.02664869	0.021027967	Itgb6/Cdh3/Lum/Fbln1/Serpinf2	5
BP	GO:0003300	cardiac muscle hypertrophy	8/556	107/28814	3.87	0.001120863	0.02664869	0.021027967	Nr4a3/Tcap/Mlip/Pparg/Pde5a/Ccn4/Edn1/Myh6	8
BP	GO:0048661	positive regulation of smooth muscle cell proliferation	8/556	107/28814	3.87	0.001120863	0.02664869	0.021027967	Nr4a3/Thbs1/Ddr2/Pdgfd/Ccn4/Edn1/Retn/Serpinf2	8
BP	GO:2000027	regulation of animal organ morphogenesis	9/556	133/28814	3.51	0.001141513	0.026979992	0.021289391	Cdh1/Nog/Sox4/Sfrp1/Fzd1/Dab2/Edn1/Agtr1a/Wt1	9
BP	GO:0060828	regulation of canonical Wnt signaling pathway	13/556	249/28814	2.71	0.0011893	0.027511799	0.02170903	Lmx1a/Cdh3/Cdh1/Nog/Tbx18/Frzb/Rspo3/Sfrp1/Fzd1/Fzd4/Dab2/Nkd2/Prox1	13
BP	GO:0061326	renal tubule development	8/556	108/28814	3.84	0.001190503	0.027511799	0.02170903	Tfap2b/Aqp1/Nog/Sox4/Npnt/Adamts16/Agtr1a/Wt1	8
BP	GO:0007601	visual perception	10/556	161/28814	3.22	0.001192444	0.027511799	0.02170903	Rd3/Abca4/Lum/Zic2/Rgs9/Col11a1/Col2a1/Impg1/Myo3b/Gabrr2	10
BP	GO:0090090	negative regulation of canonical Wnt signaling pathway	9/556	134/28814	3.48	0.001203249	0.027511799	0.02170903	Cdh1/Nog/Tbx18/Frzb/Sfrp1/Fzd1/Fzd4/Dab2/Nkd2	9
BP	GO:0048839	inner ear development	12/556	219/28814	2.84	0.001203889	0.027511799	0.02170903	Mcoln3/Cyt1l/Neurod1/Cdh1/Nr4a3/Tcap/Tbx18/Sox4/Frzb/Col11a1/Col2a1/Myo3b	12
BP	GO:0072001	renal system development	16/556	345/28814	2.4	0.001205096	0.027511799	0.02170903	Tfap2b/Crlf1/Aqp1/Aldh1a2/Prdm1/Nog/Tbx18/Sox4/Sfrp1/Pdgfd/Npnt/Adamts16/Ret/Agtr1a/Wt1	16
BP	GO:0045684	positive regulation of epidermis development	4/556	25/28814	8.29	0.001257167	0.028522113	0.02250625	Krt2/Prkch/Ptch2/Vdr	4
BP	GO:0044409	entry into host	8/556	109/28814	3.8	0.001263548	0.028522113	0.02250625	Ch25h/Ifitm1/Ifitm3/Trim34a/Cd74/Ifitm2/Spint1/Agtr1a	8
BP	GO:0021543	pallium development	10/556	163/28814	3.18	0.001308723	0.029376802	0.023180668	Lhx5/Lmx1a/Neurod1/Nr4a3/Emx1/Trp73/Tmem108/Efhc1/Btbd3/Nkx2-1	10
BP	GO:0048754	branching morphogenesis of an epithelial tube	11/556	192/28814	2.97	0.001332921	0.029753756	0.023478115	Nfatc4/Nog/Sox4/Esr1/Lama1/Npnt/Adamts16/Edn1/Agtr1a/Vdr/Wt1	11
BP	GO:0019216	regulation of lipid metabolic process	17/556	382/28814	2.31	0.001341796	0.029786397	0.023503871	Rdh16/Hcar1/Igf2/Twist1/Bmp5/Nr4a3/Sphk1/Cd74/Esr1/Pparg/Tspo/Dab2/Trib3/Nkd2/Agtr1a/Fcor/Dhh	17
BP	GO:0007159	leukocyte cell-cell adhesion	17/556	383/28814	2.3	0.0013797	0.030460407	0.024035719	Il7/Slamf1/Itgb2l/Igf2/Itgb2/Nr4a3/H2-Aa/Thbs1/H2-Eb1/Cd74/Pde5a/Itga5/Rhoh/Nlrp3/Vcan/Prnp	17
BP	GO:0050953	sensory perception of light stimulus	10/556	165/28814	3.14	0.001434071	0.031486881	0.024845691	Rd3/Abca4/Lum/Zic2/Rgs9/Col11a1/Col2a1/Impg1/Myo3b/Gabrr2	10
BP	GO:0014911	positive regulation of smooth muscle cell migration	6/556	64/28814	4.86	0.001460938	0.03160992	0.024942778	Nr4a3/Cyp1b1/Ddr2/Pdgfd/Ccn4/Retn	6
BP	GO:0002504	antigen processing and presentation of peptide or polysaccharide antigen via MHC class II	4/556	26/28814	7.97	0.001463277	0.03160992	0.024942778	H2-Aa/Thbs1/H2-Eb1/Cd74	4
BP	GO:0070293	renal absorption	4/556	26/28814	7.97	0.001463277	0.03160992	0.024942778	Hnf1a/Aqp1/Dab2/Edn1	4
BP	GO:0090596	sensory organ morphogenesis	15/556	319/28814	2.44	0.001479262	0.03178435	0.025080417	Aqp1/Twist1/Igfn1/Nr4a3/Prdm1/Tcap/Nog/Tbx18/Sox4/Frzb/Rarb/Col11a1/Col2a1/Edn1/Myo3b	15
BP	GO:0042886	amide transport	16/556	353/28814	2.35	0.001524572	0.032425223	0.025586118	Foxl2/Hnf1a/Slc15a5/Tfap2b/Aqp1/Neurod1/Lepr/Cartpt/Crh/Cd74/Sfrp1/Pparg/Edn3/Slc9b2/Pde1c/Edn1	16
BP	GO:0015833	peptide transport	15/556	320/28814	2.43	0.001525228	0.032425223	0.025586118	Hnf1a/Slc15a5/Tfap2b/Aqp1/Neurod1/Lepr/Cartpt/Crh/Cd74/Sfrp1/Pparg/Edn3/Slc9b2/Pde1c/Edn1	15
BP	GO:0001655	urogenital system development	17/556	387/28814	2.28	0.001540719	0.032582146	0.025709943	Tfap2b/Crlf1/Aqp1/Aldh1a2/Prdm1/Nog/Tbx18/Sox4/Sfrp1/Esr1/Pdgfd/Npnt/Adamts16/Rarb/Ret/Agtr1a/Wt1	17
BP	GO:0021675	nerve development	7/556	88/28814	4.12	0.001583023	0.0331423	0.02615195	Egr2/Sox4/Ret/Ntrk1/Edn1/Atoh7/Prrxl1	7
BP	GO:0002792	negative regulation of peptide secretion	6/556	65/28814	4.78	0.001583704	0.0331423	0.02615195	Cartpt/Crh/Cd74/Sfrp1/Pparg/Pde1c	6
BP	GO:0033627	cell adhesion mediated by integrin	7/556	89/28814	4.08	0.001690144	0.03426606	0.027038687	Itgb6/Itgb2l/Cyp1b1/Npnt/Ret/Itga5/Itgax	7
BP	GO:0035809	regulation of urine volume	4/556	27/28814	7.68	0.001691802	0.03426606	0.027038687	Tfap2b/Npr3/Edn1/Avpr2	4
BP	GO:0048266	behavioral response to pain	4/556	27/28814	7.68	0.001691802	0.03426606	0.027038687	Thbs1/Tspo/Ntrk1/Pirt	4
BP	GO:0045840	positive regulation of mitotic nuclear division	5/556	45/28814	5.76	0.001698403	0.03426606	0.027038687	Igf2/Cdc1/Sphk1/Edn3/Edn1	5
BP	GO:0042593	glucose homeostasis	14/556	291/28814	2.49	0.001699131	0.03426606	0.027038687	Pomc/Hnf1a/Tfap2b/Neurod1/Lepr/Cartpt/Crh/Pparg/Ccn4/Slc9b2/Pde1c/Oprk1/Slc2a4/Fcor	14
BP	GO:0021915	neural tube development	11/556	198/28814	2.88	0.001702856	0.03426606	0.027038687	Twist1/Aldh1a2/Bmp5/Zic5/Zic2/Nog/Sfrp1/Fzd1/Tead2/Spint2/Spint1	11
BP	GO:0002720	positive regulation of cytokine production involved in immune response	6/556	66/28814	4.71	0.001714156	0.03426606	0.027038687	Slamf1/Nr4a3/Rsad2/Cd74/Nlrp3/Nod2	6
BP	GO:0050891	multicellular organismal water homeostasis	6/556	66/28814	4.71	0.001714156	0.03426606	0.027038687	Tfap2b/Aqp1/Cldn1/Cdh1/Alox12b/Scnn1a	6
BP	GO:0060688	regulation of morphogenesis of a branching structure	6/556	66/28814	4.71	0.001714156	0.03426606	0.027038687	Cdh1/Nog/Sox4/Sfrp1/Esr1/Agtr1a	6
BP	GO:0033500	carbohydrate homeostasis	14/556	292/28814	2.48	0.001754024	0.034676346	0.027362436	Pomc/Hnf1a/Tfap2b/Neurod1/Lepr/Cartpt/Crh/Pparg/Ccn4/Slc9b2/Pde1c/Oprk1/Slc2a4/Fcor	14
BP	GO:0035810	positive regulation of urine volume	3/556	13/28814	11.96	0.001769201	0.034676346	0.027362436	Tfap2b/Npr3/Edn1	3
BP	GO:1904181	positive regulation of membrane depolarization	3/556	13/28814	11.96	0.001769201	0.034676346	0.027362436	Myoc/Dcn/Tspo	3
BP	GO:2000849	regulation of glucocorticoid secretion	3/556	13/28814	11.96	0.001769201	0.034676346	0.027362436	Pomc/Crh/Tspo	3
BP	GO:0007589	body fluid secretion	8/556	115/28814	3.61	0.001780057	0.034719751	0.027396686	Prl/Aqp1/Npr3/Trp73/Alox12b/Edn1/Oprk1/Vdr	8
BP	GO:0050890	cognition	16/556	359/28814	2.31	0.001808494	0.035077725	0.027679156	Lmx1a/Igf2/Nfatc4/Nog/Tafa2/Sgk1/Crh/Arc/Casp1/Pde5a/Musk/Ntrk1/Itga5/Oprk1/Slc2a4/Prnp	16
BP	GO:0006820	anion transport	20/556	498/28814	2.08	0.00181587	0.035077725	0.027679156	Slc22a2/Foxl2/Hnf1a/Slc13a4/Slc22a6/Slc6a20a/Slc6a13/Ptges/Slc16a5/Slc37a2/Grm2/Slc16a12/Tspo/Slc4a11/Edn1/Slc10a4/Slc26a5/Gabrr2/Slc26a10/Prnp	20
BP	GO:0001658	branching involved in ureteric bud morphogenesis	6/556	67/28814	4.64	0.0018526	0.035616019	0.028103914	Nog/Sox4/Npnt/Adamts16/Agtr1a/Wt1	6
BP	GO:0045069	regulation of viral genome replication	7/556	91/28814	3.99	0.001921224	0.036329527	0.028666929	Ifitm1/Rsad2/Ifitm3/Bst2/Oasl2/Parp10/Ifitm2	7
BP	GO:0060420	regulation of heart growth	7/556	91/28814	3.99	0.001921224	0.036329527	0.028666929	Dusp6/Trp73/Nog/Ccn4/Edn1/Myh6/Wt1	7
BP	GO:0003299	muscle hypertrophy in response to stress	4/556	28/28814	7.4	0.001943964	0.036329527	0.028666929	Tcap/Mlip/Pparg/Myh6	4
BP	GO:0014898	cardiac muscle hypertrophy in response to stress	4/556	28/28814	7.4	0.001943964	0.036329527	0.028666929	Tcap/Mlip/Pparg/Myh6	4
BP	GO:0060713	labyrinthine layer morphogenesis	4/556	28/28814	7.4	0.001943964	0.036329527	0.028666929	Bmp5/Rspo3/Spint2/Spint1	4
BP	GO:0097186	amelogenesis	4/556	28/28814	7.4	0.001943964	0.036329527	0.028666929	Itgb6/Wdr72/Ferp/Dmp1	4
BP	GO:0072009	nephron epithelium development	8/556	117/28814	3.54	0.001985038	0.036755211	0.029002828	Tfap2b/Aqp1/Nog/Sox4/Npnt/Adamts16/Agtr1a/Wt1	8

BP	GO:1902105	regulation of leukocyte differentiation	10/305	323/28814	2.92	0.002480013	0.04691108	0.040353279	Il2ra/Il2rg/Hspb1/Fas/Pglyrp1/Runx1/Il7r/Duxbl1/Fstl3/Pou4f1	10
BP	GO:0051091	positive regulation of DNA-binding transcription factor activity	9/305	272/28814	3.13	0.002593068	0.048755897	0.041940205	Ltf/Hspa1b/Trim34a/Trim30a/Fcgr2b/Eif2ak2/Ddx58/Irak3/Cd36	9
BP	GO:0019216	regulation of lipid metabolic process	11/305	382/28814	2.72	0.002693285	0.050338782	0.043301815	Apob/Drd3/Cyp27b1/Htr2a/Ptgs2/Angptl4/Rnf213/Nr4a3/Apoc1/Avil/Cd36	11
BP	GO:0001961	positive regulation of cytokine-mediated signaling pathway	4/305	55/28814	6.87	0.002746481	0.051029297	0.043895802	Hspa1b/Irf7/Il1r1/Casp1	4
BP	GO:0002440	production of molecular mediator of immune response	11/305	386/28814	2.69	0.002915484	0.053850711	0.046322804	Fas/Il1r1/Fcgr2b/Ddx58/Nr4a3/Irak3/Il7r/H2-DMb2/Cd36/Il27ra/Il13ra2	11
BP	GO:0052372	modulation by symbiont of entry into host	4/305	57/28814	6.63	0.003128946	0.057455505	0.049423676	Ch25h/Ifitm3/Trim34a/Trim30a	4
BP	GO:0033622	integrin activation	3/305	28/28814	10.12	0.003162466	0.057493174	0.049456079	Selp/Piezo1/Skap1	3
BP	GO:0010952	positive regulation of peptidase activity	7/305	180/28814	3.67	0.003167618	0.057493174	0.049456079	S100a9/S100a8/Ifi207/Ifi204/Tnfrsf23/Fas/Casp1	7
BP	GO:0046651	lymphocyte proliferation	10/305	335/28814	2.82	0.003216637	0.058047358	0.049932793	Slfn1/Il2ra/Bst1/Cdkn1a/Fcgr2b/Zbtb32/Gpr183/Il7r/H2-DMb2/Il27ra	10
BP	GO:0008277	regulation of G protein-coupled receptor signaling pathway	6/305	136/28814	4.17	0.003337015	0.05987558	0.051505443	Ly6g6e/Drd3/Oprm1/Gipr/Adrb2/Dynlt1b	6
BP	GO:0032651	regulation of interleukin-1 beta production	5/305	95/28814	4.97	0.003429359	0.061182877	0.052629991	Ifi207/Ifi204/Hspb1/Casp1/Cd36	5
BP	GO:0032943	mononuclear cell proliferation	10/305	339/28814	2.79	0.003497599	0.061396483	0.052813736	Slfn1/Il2ra/Bst1/Cdkn1a/Fcgr2b/Zbtb32/Gpr183/Il7r/H2-DMb2/Il27ra	10
BP	GO:0032647	regulation of interferon-alpha production	3/305	29/28814	9.77	0.003499991	0.061396483	0.052813736	Irf7/Ddx58/Gbp4	3
BP	GO:0048875	chemical homeostasis within a tissue	3/305	29/28814	9.77	0.003499991	0.061396483	0.052813736	Oas1a/Homer1/Napsa	3
BP	GO:0071216	cellular response to biotic stimulus	10/305	340/28814	2.78	0.003570799	0.06219926	0.053504292	Ltf/Cxcl1/Cxcl5/Fcgr4/Ccl12/Trim30a/Fcgr2b/Hspa5/Casp1/Cd36	10
BP	GO:0032609	interferon-gamma production	6/305	138/28814	4.11	0.003585371	0.06219926	0.053504292	Isg15/Il1r1/Pglyrp1/Runx1/Il27ra/Cd2	6
BP	GO:0002718	regulation of cytokine production involved in immune response	5/305	97/28814	4.87	0.003749931	0.064659151	0.055620309	Il1r1/Ddx58/Nr4a3/Irak3/Cd36	5
BP	GO:0032757	positive regulation of interleukin-8 production	4/305	60/28814	6.3	0.003768352	0.064659151	0.055620309	Hspa1b/Nlrp10/Ddx58/Cd2	4
BP	GO:0002819	regulation of adaptive immune response	8/305	236/28814	3.2	0.003821358	0.065212301	0.056096133	Irf7/Nlrp10/Il1r1/Fcgr2b/Il7r/Skap1/Fcer2a/Il27ra	8
BP	GO:0010951	negative regulation of endopeptidase activity	7/305	187/28814	3.54	0.003905642	0.065814215	0.056613904	Ltf/Ifi207/Hspa1b/Ifi204/Ptgs2/Serpinc1/Cst7	7
BP	GO:0042594	response to starvation	7/305	187/28814	3.54	0.003905642	0.065814215	0.056613904	Ifi207/Ifi204/Fas/Cdkn1a/Angptl4/Eif2ak2/Hspa5	7
BP	GO:0031668	cellular response to extracellular stimulus	8/305	237/28814	3.19	0.003919509	0.065814215	0.056613904	Ifi207/Cyp27b1/Ifi204/Fas/Cdkn1a/Angptl4/Eif2ak2/Hspa5	8
BP	GO:1903321	negative regulation of protein modification by small protein conjugation or removal	5/305	99/28814	4.77	0.004091422	0.068335461	0.058782699	Hspa1b/Isg15/Dtx3l/Parp10/Gbp4	5
BP	GO:0050863	regulation of T cell activation	10/305	348/28814	2.71	0.004201186	0.069050492	0.059397775	Slfn1/Il2ra/Il2rg/Hspb1/Runx1/Il7r/H2-DMb2/Duxbl1/Il27ra/Icos	10
BP	GO:0098657	import into cell	8/305	240/28814	3.15	0.00422568	0.069050492	0.059397775	Lcn2/Slc6a5/Drd3/Adrb2/Tfr2/Cd36/Lrrc8e/Slc5a2	8
BP	GO:0046597	negative regulation of viral entry into host cell	3/305	31/28814	9.14	0.004239027	0.069050492	0.059397775	Ch25h/Ifitm3/Trim30a	3
BP	GO:0050982	detection of mechanical stimulus	4/305	62/28814	6.09	0.004240539	0.069050492	0.059397775	Chrna5/Htr2a/Piezo1/Pkd2l1	4
BP	GO:0009991	response to extracellular stimulus	11/305	406/28814	2.56	0.004258391	0.069050492	0.059397775	Ifi207/Cyp27b1/Ifi204/Fas/Cdkn1a/Oprm1/Angptl4/Eif2ak2/Hspa5/Adrb2/Serpinc1	11
BP	GO:0050729	positive regulation of inflammatory response	6/305	143/28814	3.96	0.004266177	0.069050492	0.059397775	S100a9/S100a8/Lgals2/Nlrp10/Ptgs2/Casp1	6
BP	GO:0042063	gliogenesis	10/305	350/28814	2.7	0.004371747	0.07020349	0.060389593	S100a9/S100a8/Drd3/Ccl12/Fas/Ndn/Sh3tc2/Gpr183/Nkx2-2os/Opalin	10
BP	GO:0043122	regulation of I-kappaB kinase/NF-kappaB signaling	7/305	191/28814	3.46	0.004382129	0.07020349	0.060389593	Ltf/Hspb1/Trim34a/Trim30a/Casp1/Cd36/S100a4	7
BP	GO:0050728	negative regulation of inflammatory response	6/305	144/28814	3.94	0.004413045	0.070339897	0.060506931	Il2ra/Fcgr2b/Pglyrp1/Adrb2/Cst7/Gpx2	6
BP	GO:0032611	interleukin-1 beta production	5/305	101/28814	4.68	0.004454568	0.070643143	0.060767786	Ifi207/Ifi204/Hspb1/Casp1/Cd36	5
BP	GO:0001818	negative regulation of cytokine production	9/305	296/28814	2.87	0.004513657	0.071220521	0.061264451	Oas1a/Sigirr/Dhx58/Trim30a/Fcgr2b/Pglyrp1/Irak3/Gbp4/Il27ra	9
BP	GO:0002825	regulation of T-helper 1 type immune response	3/305	32/28814	8.86	0.004641303	0.072777827	0.062604057	Nlrp10/Il1r1/Il27ra	3
BP	GO:0008347	glial cell migration	4/305	64/28814	5.9	0.004751105	0.072777827	0.062604057	Ccl12/Fas/Ndn/Gpr183	4
BP	GO:0007191	adenylate cyclase-activating dopamine receptor signaling pathway	2/305	10/28814	18.89	0.004751419	0.072777827	0.062604057	Drd3/Oprm1	2
BP	GO:0035811	negative regulation of urine volume	2/305	10/28814	18.89	0.004751419	0.072777827	0.062604057	Adrb2/Slc5a2	2
BP	GO:1902033	regulation of hematopoietic stem cell proliferation	2/305	10/28814	18.89	0.004751419	0.072777827	0.062604057	Cxcl1/Eif2ak2	2
BP	GO:2001269	positive regulation of cysteine-type endopeptidase activity involved in apoptotic signaling pathway	2/305	10/28814	18.89	0.004751419	0.072777827	0.062604057	Tnfrsf23/Fas	2
BP	GO:0050670	regulation of lymphocyte proliferation	8/305	245/28814	3.08	0.004776539	0.072807432	0.062629524	Slfn1/Il2ra/Bst1/Cdkn1a/Fcgr2b/Gpr183/H2-DMb2/Il27ra	8
BP	GO:0006639	acylglycerol metabolic process	6/305	147/28814	3.86	0.004876043	0.073609502	0.063319471	Apob/Dgkg/Tnxb/Apoc1/Avil/Cd36	6
BP	GO:0050671	positive regulation of lymphocyte proliferation	6/305	147/28814	3.86	0.004876043	0.073609502	0.063319471	Il2ra/Bst1/Cdkn1a/Gpr183/H2-DMb2/Il27ra	6
BP	GO:0045089	positive regulation of innate immune response	6/305	148/28814	3.83	0.005038024	0.07503261	0.06454364	Ifi207/Ifi209/Ifi204/Irf7/Trim30a/Ifi203	6
BP	GO:0070227	lymphocyte apoptotic process	5/305	104/28814	4.54	0.005041479	0.07503261	0.06454364	Il2ra/Siglec1/Tnfrsf23/Fas/Il7r	5
BP	GO:0032607	interferon-alpha production	3/305	33/28814	8.59	0.005065896	0.07503261	0.06454364	Irf7/Ddx58/Gbp4	3
BP	GO:1904646	cellular response to amyloid-beta	3/305	33/28814	8.59	0.005065896	0.07503261	0.06454364	Fcgr2b/Adrb2/Cd36	3
BP	GO:0006638	neutral lipid metabolic process	6/305	149/28814	3.8	0.00520392	0.076356582	0.065682531	Apob/Dgkg/Tnxb/Apoc1/Avil/Cd36	6
BP	GO:0032946	positive regulation of mononuclear cell proliferation	6/305	149/28814	3.8	0.00520392	0.076356582	0.065682531	Il2ra/Bst1/Cdkn1a/Gpr183/H2-DMb2/Il27ra	6
BP	GO:0046718	viral entry into host cell	5/305	105/28814	4.5	0.00524873	0.076400615	0.065720408	Ch25h/Siglec1/Ifitm3/Trim34a/Trim30a	5
BP	GO:0032944	regulation of mononuclear cell proliferation	8/305	249/28814	3.04	0.005255584	0.076400615	0.065720408	Slfn1/Il2ra/Bst1/Cdkn1a/Fcgr2b/Gpr183/H2-DMb2/Il27ra	8

BP	GO:0060333	interferon-gamma-mediated signaling pathway	4/574	21/28814	9.56	0.000712308	0.013203841	0.01039055	Irgm1/Parp14/Nlrc5/Igtp	4
BP	GO:2000353	positive regulation of endothelial cell apoptotic process	4/574	21/28814	9.56	0.000712308	0.013203841	0.01039055	Ecscr/Cd248/Ccl12/Ager	4
BP	GO:0001570	vasculogenesis	8/574	97/28814	4.14	0.000722592	0.013333299	0.010492425	Eng/Tek/Kdr/Tie1/Rasip1/Adm/Sox18/Foxm1	8
BP	GO:0015837	amine transport	9/574	121/28814	3.73	0.000727506	0.013337953	0.010496088	Cxcl12/Per2/Crh/Syt15/Avpr1a/Avp/Tacr2/Oxt/Agt	9
BP	GO:0035296	regulation of tube diameter	11/574	173/28814	3.19	0.000737225	0.013337953	0.010496088	Kdr/Per2/Adm/Icam1/Gch1/Klf2/Avpr1a/Kcnmb1/Avp/Htr2b/Agt	11
BP	GO:0097746	blood vessel diameter maintenance	11/574	173/28814	3.19	0.000737225	0.013337953	0.010496088	Kdr/Per2/Adm/Icam1/Gch1/Klf2/Avpr1a/Kcnmb1/Avp/Htr2b/Agt	11
BP	GO:0007249	I-kappaB kinase/NF-kappaB signaling	13/574	229/28814	2.85	0.000738354	0.013337953	0.010496088	Hspb1/Nod2/Trim30a/Tnfsf10/Bcl3/Lilrb4a/Edar/Olfm4/Unc5cl/Ticam2/Traf1/Trim6/Eda2r	13
BP	GO:0014068	positive regulation of phosphatidylinositol 3-kinase signaling	7/574	75/28814	4.69	0.000739347	0.013337953	0.010496088	Flt1/Fn1/Tek/Kdr/Ccl5/Myoc/Agt	7
BP	GO:0032612	interleukin-1 production	9/574	122/28814	3.7	0.000771866	0.013824584	0.010879034	Gbp5/Hspb1/Serpinb1b/Nod2/Lpl/Ccl3/Lilrb4a/Ager/Anxa1	9
BP	GO:0035150	regulation of tube size	11/574	174/28814	3.17	0.000773164	0.013824584	0.010879034	Kdr/Per2/Adm/Icam1/Gch1/Klf2/Avpr1a/Kcnmb1/Avp/Htr2b/Agt	11
BP	GO:0002695	negative regulation of leukocyte activation	12/574	202/28814	2.98	0.000786256	0.013996746	0.011014514	Adgrf5/Foxj1/Hspb1/Cst7/Pglyrp1/Fcgr2b/Prdm1/Lilrb4a/Fas/Hlx/Il4i1/Anxa1	12
BP	GO:0050869	negative regulation of B cell activation	5/574	37/28814	6.78	0.000793465	0.014060725	0.011064862	Foxj1/Fcgr2b/Prdm1/Lilrb4a/Fas	5
BP	GO:0045089	positive regulation of innate immune response	10/574	148/28814	3.39	0.000796809	0.014060725	0.011064862	Gbp5/Irf7/Irgm1/Nod2/Ikbke/Trim30a/Nlrc5/Igtp/Ifi35/Trim6	10
BP	GO:0048608	reproductive structure development	21/574	484/28814	2.18	0.000804089	0.014127497	0.011117407	Rec8/Abcb1a/Kdr/Vwf/Fbn2/Adm/Icam1/Lef1/Gata2/Foxf2/Nupr1/Prdm1/E2f7/Ldoc1/Grhl2/Rxfp2/Llgl2/Serp	21
BP	GO:0032635	interleukin-6 production	11/574	175/28814	3.16	0.000810545	0.014179276	0.011158153	Foxj1/Nod2/Lpl/Trim30a/Klf2/Rab7b/Tlr1/Lilrb4a/Ager/Ticam2/Tnfsf9	11
BP	GO:0060337	type I interferon signaling pathway	6/574	56/28814	5.38	0.0008496	0.014673863	0.01154736	Ifitm3/Irf7/Ikbke/Nlrc5/Oas2/Trim6	6
BP	GO:0071357	cellular response to type I interferon	6/574	56/28814	5.38	0.0008496	0.014673863	0.01154736	Ifitm3/Irf7/Ikbke/Nlrc5/Oas2/Trim6	6
BP	GO:0035907	dorsal aorta development	3/574	10/28814	15.06	0.000850133	0.014673863	0.01154736	Eng/Ngfr/Dll4	3
BP	GO:0060055	angiogenesis involved in wound healing	4/574	22/28814	9.13	0.000856974	0.014673863	0.01154736	Kdr/Gata2/Gpr4/Cxcr4	4
BP	GO:0098543	detection of other organism	4/574	22/28814	9.13	0.000856974	0.014673863	0.01154736	Nod2/Pglyrp1/Clec7a/Tlr1	4
BP	GO:0046635	positive regulation of alpha-beta T cell activation	7/574	77/28814	4.56	0.000865779	0.014724181	0.011586957	Rasal3/Ptprc/Il2rg/Il12rb2/Lilrb4a/Hlx/Anxa1	7
BP	GO:0032963	collagen metabolic process	9/574	124/28814	3.64	0.0008672	0.014724181	0.011586957	Fn1/Vim/Mrc2/Nppc/Mmp23/Rcn3/Tnxb/Scx/Ager	9
BP	GO:0002221	pattern recognition receptor signaling pathway	11/574	177/28814	3.12	0.000889811	0.014947986	0.011763078	Hspa1b/Irf7/Irgm1/Nod2/Trim30a/Rab7b/Rsad2/Igtp/Tlr1/Ifi35/Ddx60	11
BP	GO:0061458	reproductive system development	21/574	488/28814	2.16	0.000891697	0.014947986	0.011763078	Rec8/Abcb1a/Kdr/Vwf/Fbn2/Adm/Icam1/Lef1/Gata2/Foxf2/Nupr1/Prdm1/E2f7/Ldoc1/Grhl2/Rxfp2/Llgl2/Serp	21
BP	GO:0009595	detection of biotic stimulus	5/574	38/28814	6.61	0.000898877	0.014947986	0.011763078	Nod2/Pglyrp1/Clec7a/Tlr1/Srpx	5
BP	GO:0045589	regulation of regulatory T cell differentiation	5/574	38/28814	6.61	0.000898877	0.014947986	0.011763078	Hspb1/Il2rg/Lilrb4a/Ctla2a/Il4i1	5
BP	GO:0097254	renal tubular secretion	5/574	38/28814	6.61	0.000898877	0.014947986	0.011763078	Avpr1a/Abcg3/Avp/Oxt/Agt	5
BP	GO:0032964	collagen biosynthetic process	6/574	57/28814	5.28	0.000933793	0.015464991	0.012169926	Fn1/Vim/Nppc/Rcn3/Scx/Ager	6
BP	GO:0032611	interleukin-1 beta production	8/574	101/28814	3.98	0.000943055	0.015554641	0.012240475	Gbp5/Hspb1/Serpinb1b/Nod2/Lpl/Ccl3/Lilrb4a/Ager	8
BP	GO:0002366	leukocyte activation involved in immune response	16/574	327/28814	2.46	0.000959703	0.015764875	0.012405915	Icam1/Lef1/Gata2/Ly9/Ptprc/Mfng/Pglyrp1/Nppc/Bcl3/Lilrb4a/Ifi35/6030468B19Rik/Ccr6/Hlx/H2-	16
BP	GO:0032872	regulation of stress-activated MAPK cascade	12/574	207/28814	2.91	0.000972538	0.015911042	0.012520939	Mid1/Flt4/Mecom/Nod2/Fcgr2b/Fas/Foxm1/Edar/Unc5cl/Ager/Traf1/Eda2r	12
BP	GO:0060249	anatomical structure homeostasis	16/574	328/28814	2.45	0.000990852	0.016145301	0.012705285	Adgrf5/Prom1/Abcb1a/Kdr/Ctsh/Lsr/Lrrk1/Gata2/Nod2/Scgn/Col9a1/Rpe65/Rcn3/Rho/Col14a1/Scx	16
BP	GO:0043491	protein kinase B signaling	12/574	208/28814	2.9	0.001013884	0.016227094	0.012769651	Eng/Tek/Cxcl12/Kdr/Klf4/Ccl5/Pear1/Myoc/Ngfr/Rcn3/Ccl12/Calcr	12
BP	GO:0002335	mature B cell differentiation	5/574	39/28814	6.44	0.001014361	0.016227094	0.012769651	Mfng/Cmtm7/Bcl3/Lilrb4a/6030468B19Rik	5
BP	GO:1904645	response to amyloid-beta	5/574	39/28814	6.44	0.001014361	0.016227094	0.012769651	Icam1/Fcgr2b/Ngfr/Calcr/Ager	5
BP	GO:0010952	positive regulation of peptidase activity	11/574	180/28814	3.07	0.001020658	0.016227094	0.012769651	Acer2/Uaca/Ctsh/Ngfr/Tnfsf10/Sox7/Fas/Rcn3/Cldn3/S100a9/Ager	11
BP	GO:0002043	blood vessel endothelial cell proliferation involved in sprouting angiogenesis	4/574	23/28814	8.73	0.001021165	0.016227094	0.012769651	Gata2/Mmrn2/Ngfr/Dll4	4
BP	GO:0001914	regulation of T cell mediated cytotoxicity	6/574	58/28814	5.19	0.001024259	0.016227094	0.012769651	H2-D1/Ptprc/H2-Q4/Il7r/H2-T10/Ager	6
BP	GO:0003073	regulation of systemic arterial blood pressure	9/574	127/28814	3.56	0.001027946	0.016227094	0.012769651	Eng/Kdr/Adm/Crh/Rarres2/Enpep/Avpr1a/Avp/Agt	9
BP	GO:0050830	defense response to Gram-positive bacterium	10/574	153/28814	3.28	0.001027997	0.016227094	0.012769651	Gbp3/Gbp9/Gbp4/Adm/Nod2/Pglyrp1/Il7r/Rarres2/Gbp2/Gbp6	10
BP	GO:0014066	regulation of phosphatidylinositol 3-kinase signaling	8/574	103/28814	3.9	0.001071873	0.016788527	0.013211462	Flt1/Fn1/Tek/Kdr/Klf4/Ccl5/Myoc/Agt	8
BP	GO:0030593	neutrophil chemotaxis	8/574	103/28814	3.9	0.001071873	0.016788527	0.013211462	Ccl6/Ccl5/Nod2/Ccl9/Ccl4/Ccl3/Ccl12/S100a9	8
BP	GO:0002263	cell activation involved in immune response	16/574	331/28814	2.43	0.001089496	0.016998659	0.013376822	Icam1/Lef1/Gata2/Ly9/Ptprc/Mfng/Pglyrp1/Nppc/Bcl3/Lilrb4a/Ifi35/6030468B19Rik/Ccr6/Hlx/H2-	16
BP	GO:0070302	regulation of stress-activated protein kinase signaling cascade	12/574	210/28814	2.87	0.001100966	0.017111555	0.013465663	Mid1/Flt4/Mecom/Nod2/Fcgr2b/Fas/Foxm1/Edar/Unc5cl/Ager/Traf1/Eda2r	12
BP	GO:0046632	alpha-beta T cell differentiation	9/574	129/28814	3.5	0.001147921	0.017561997	0.013820131	Lef1/Ly9/Il2rg/Prdm1/Rsad2/Bcl3/Lilrb4a/Hlx/Anxa1	9
BP	GO:0031077	post-embryonic camera-type eye development	3/574	11/28814	13.69	0.001151678	0.017561997	0.013820131	Flt1/Kdr/Klf4	3
BP	GO:0045924	regulation of female receptivity	3/574	11/28814	13.69	0.001151678	0.017561997	0.013820131	Avpr1a/Avp/Oxt	3
BP	GO:0060856	establishment of blood-brain barrier	3/574	11/28814	13.69	0.001151678	0.017561997	0.013820131	Abcb1a/Lsr/Cldn3	3
BP	GO:2001280	positive regulation of unsaturated fatty acid biosynthetic process	3/574	11/28814	13.69	0.001151678	0.017561997	0.013820131	Avpr1a/Avp/Anxa1	3
BP	GO:0032088	negative regulation of NF-kappaB transcription factor activity	7/574	81/28814	4.34	0.001169474	0.017699791	0.013928566	Foxj1/Klf4/Traip/Nod2/Nlrc5/Cmklr1/Bcl3	7
BP	GO:0048844	artery morphogenesis	7/574	81/28814	4.34	0.001169474	0.017699791	0.013928566	Adgrf5/Eng/Hpgd/Prdm1/Dll4/Tbx1/Tfap2b	7
BP	GO:0036303	lymph vessel morphogenesis	4/574	24/28814	8.37	0.001206248	0.018188232	0.014312937	Tie1/Flt4/Sox18/Clec14a	4
BP	GO:0046330	positive regulation of JNK cascade	8/574	105/28814	3.82	0.001214371	0.018242659	0.014355767	Flt4/Nod2/Fcgr2b/Edar/Unc5cl/Ager/Traf1/Eda2r	8
BP	GO:0051092	positive regulation of NF-kappaB transcription factor activity	10/574	157/28814	3.2	0.001250151	0.018641546	0.014669665	Hspa1b/Icam1/Prkd2/Nod2/Trim30a/Rab7b/Ager/Traf1/Trim6/Eda2r	10
BP	GO:0072006	nephron development	10/574	157/28814	3.2	0.001250151	0.018641546	0.014669665	Prom1/Tek/Lama5/Gpr4/Wnt6/Enpep/Angpt2/Hs3st3a1/Tfap2b/Agt	10
BP	GO:0045123	cellular extravasation	7/574	82/28814	4.29	0.001257064	0.018675712	0.014696551	Pecam1/Plvap/Cxcl12/Il1r1/Icam1/Ccl5/Ager	7
BP	GO:0032731	positive regulation of interleukin-1 beta production	6/574	61/28814	4.94	0.001336385	0.019709234	0.015509865	Gbp5/Hspb1/Nod2/Lpl/Ccl3/Ager	6
BP	GO:0045620	negative regulation of lymphocyte differentiation	6/574	61/28814	4.94	0.001336385	0.019709234	0.015509865	Foxj1/Hspb1/Pglyrp1/Lilrb4a/Hlx/Anxa1	6
BP	GO:0048872	homeostasis of number of cells	17/574	372/28814	2.29	0.001418382	0.020842472	0.016401649	Adgrf5/Arhgef5/Hspa1b/Emcn/Gata2/Mecom/Fcgr2b/Klf2/Il7r/Rag1/Lilrb4a/Fas/Col14a1/Slc4a1/Lgals2/Tal1/Anxa1	17

BP	GO:1904659	glucose transmembrane transport	8/556	117/28814	3.54	0.001985038	0.036755211	0.029002828	Ostn/Hnf1a/Slc2a12/Nr4a3/Esr1/Edn1/Trib3/Slc2a4	8
BP	GO:0045444	fat cell differentiation	13/556	264/28814	2.55	0.001999969	0.036861819	0.029086951	Tfap2b/Wif1/Nr4a3/Egr2/Frzb/Sfrp1/Pparg/Ccn4/Lamb3/Trib3/Slc2a4/Fcor/Retn	13
BP	GO:1903531	negative regulation of secretion by cell	10/556	173/28814	3	0.002036727	0.037367904	0.029486293	Cartpt/Rsad2/Crh/Sfrp1/Pparg/Tspo/Edn3/Pde1c/Edn1/Oprk1	10
BP	GO:0002790	peptide secretion	14/556	298/28814	2.43	0.002115228	0.038456953	0.03034564	Hnf1a/Tfap2b/Aqp1/Neurod1/Lepr/Cartpt/Crh/Cd74/Sfrp1/Pparg/Edn3/Slc9b2/Pde1c/Edn1	14
BP	GO:0022409	positive regulation of cell-cell adhesion	14/556	298/28814	2.43	0.002115228	0.038456953	0.03034564	Il7/Slamf1/Igf2/Igfbp2/Cdh1/Nr4a3/H2-Aa/Tbx18/H2-Eb1/Cd74/Rhoh/Nlrp3/Vtcn1/Chst4	14
BP	GO:0045668	negative regulation of osteoblast differentiation	6/556	69/28814	4.51	0.00215471	0.038799377	0.03061584	Ostn/Twist1/Ranbp3l/Nog/Sfrp1/Pparg	6
BP	GO:0009952	anterior/posterior pattern specification	12/556	235/28814	2.65	0.002177105	0.038799377	0.03061584	Dll3/Aldh1a2/Ifitm1/Neurod1/Tcap/Nog/Tbx18/Arc/Sfrp1/Tdgf1/Meox1/Wt1	12
BP	GO:0060670	branching involved in labyrinthine layer morphogenesis	3/556	14/28814	11.11	0.002219608	0.038799377	0.03061584	Rspo3/Spint2/Spint1	3
BP	GO:0071285	cellular response to lithium ion	3/556	14/28814	11.11	0.002219608	0.038799377	0.03061584	Cdh1/Nfatc4/Pparg	3
BP	GO:0072224	metanephric glomerulus development	3/556	14/28814	11.11	0.002219608	0.038799377	0.03061584	Aqp1/Ret/Wt1	3
BP	GO:2000052	positive regulation of non-canonical Wnt signaling pathway	3/556	14/28814	11.11	0.002219608	0.038799377	0.03061584	Rspo3/Sfrp1/Dab2	3
BP	GO:2000833	positive regulation of steroid hormone secretion	3/556	14/28814	11.11	0.002219608	0.038799377	0.03061584	Crh/Dab2/Retn	3
BP	GO:0014887	cardiac muscle adaptation	4/556	29/28814	7.15	0.00222097	0.038799377	0.03061584	Tcap/Mlip/Pparg/Myh6	4
BP	GO:0032770	positive regulation of monooxygenase activity	4/556	29/28814	7.15	0.00222097	0.038799377	0.03061584	Cdh3/Npr3/Esr1/Vdr	4
BP	GO:0014812	muscle cell migration	8/556	120/28814	3.45	0.002326869	0.040298956	0.031799129	Nr4a3/Cyp1b1/Six4/Ddr2/Pdgfd/Pparg/Ccn4/Retn	8
BP	GO:0042472	inner ear morphogenesis	8/556	120/28814	3.45	0.002326869	0.040298956	0.031799129	Nr4a3/Tcap/Tbx18/Six4/Frzb/Col11a1/Col2a1/Myo3b	8
BP	GO:0032872	regulation of stress-activated MAPK cascade	11/556	207/28814	2.75	0.002411256	0.041581222	0.032810941	Slamf1/Edar/Trp73/Sphk1/Traf1/Mdfic/Sfrp1/Dab2/Oprk1/Nod2/Serpinf2	11
BP	GO:0014910	regulation of smooth muscle cell migration	7/556	95/28814	3.82	0.002456203	0.041995845	0.033138111	Nr4a3/Cyp1b1/Ddr2/Pdgfd/Pparg/Ccn4/Retn	7
BP	GO:0051341	regulation of oxidoreductase activity	7/556	95/28814	3.82	0.002456203	0.041995845	0.033138111	Cdh3/Npr3/Esr1/Edn3/Edn1/Nod2/Vdr	7
BP	GO:0055007	cardiac muscle cell differentiation	9/556	149/28814	3.13	0.002498762	0.042542476	0.033569447	Tcap/Tbx18/Mylk3/Ccn4/Rarb/Edn1/Tdgf1/Myh6/Wt1	9
BP	GO:0002703	regulation of leukocyte mediated immunity	14/556	304/28814	2.39	0.002535911	0.042992791	0.033924782	Pomc/Klrd1/Sh2d1a/H2-Q1/Slamf1/Itgb2l/Igf2/Rsad2/Tnfsfm13/Bst2/Nlrp3/Nod2/Zp3r/Fgr	14
BP	GO:0052547	regulation of peptidase activity	18/556	443/28814	2.11	0.002656524	0.044671532	0.035249445	Slc22a2/Serpinb6c/Aqp1/Wfikkn2/Itih2/Cdh1/Thbs1/Wnt9a/Perp/Pparg/Casp1/Spint2/Serpina9/Ret/Spint1/Nlrp3/Mgmt/Serpinf2	18
BP	GO:0061035	regulation of cartilage development	6/556	72/28814	4.32	0.002675678	0.044671532	0.035249445	Loxl2/Nog/Wnt9a/Frzb/Ccn4/Rarb	6
BP	GO:0070302	regulation of stress-activated protein kinase signaling cascade	11/556	210/28814	2.71	0.002694445	0.044671532	0.035249445	Slamf1/Edar/Trp73/Sphk1/Traf1/Mdfic/Sfrp1/Dab2/Oprk1/Nod2/Serpinf2	11
BP	GO:0042060	wound healing	16/556	374/28814	2.22	0.002717319	0.044671532	0.035249445	Itgb6/Pf4/Aqp1/Duox1/Tspan8/Cldn1/Myh2/Thbs1/Nog/Fbln1/Ddr2/Pparg/Ccn4/Clec7a/BC024139/Serpinf2	16
BP	GO:0045214	sarcomere organization	5/556	50/28814	5.18	0.002722309	0.044671532	0.035249445	Tcap/Six4/Mylk3/Edn1/Myh6	5
BP	GO:0051701	biological process involved in interaction with host	9/556	151/28814	3.09	0.002733383	0.044671532	0.035249445	Ch25h/Ifitm1/Ifitm3/Fbln1/Trim34a/Cd74/Ifitm2/Spint1/Agtr1a	9
BP	GO:0035933	glucocorticoid secretion	3/556	15/28814	10.36	0.002734992	0.044671532	0.035249445	Pomc/Crh/Tspo	3
BP	GO:0042574	retinal metabolic process	3/556	15/28814	10.36	0.002734992	0.044671532	0.035249445	Abca4/Aldh1a2/Cyp1b1	3
BP	GO:0110096	cellular response to aldehyde	3/556	15/28814	10.36	0.002734992	0.044671532	0.035249445	Aldh1a7/Sgk1/Scnn1a	3
BP	GO:0002718	regulation of cytokine production involved in immune response	7/556	97/28814	3.74	0.002763316	0.04471069	0.035280344	Slamf1/Nr4a3/Rsad2/Bst2/Cd74/Nlrp3/Nod2	7
BP	GO:0048762	mesenchymal cell differentiation	12/556	242/28814	2.57	0.002769123	0.04471069	0.035280344	Twist1/Aldh1a2/Ranbp3l/Loxl2/Nog/Frzb/Sfrp1/Dab2/Edn3/Ret/Nkx2-1/Edn1	12
BP	GO:0048511	rhythmic process	14/556	307/28814	2.36	0.002770772	0.04471069	0.035280344	Twist1/Ptgds/Lepr/Cartpt/Egr2/Cort/Esr1/Pparg/Casp1/Fzd4/Nkx2-1/Ntrk1/Retn/Atoh7	14
BP	GO:0001676	long-chain fatty acid metabolic process	8/556	124/28814	3.34	0.002852559	0.044842317	0.035384207	Sphk1/Cyp1b1/Alox12b/Cyp2a5/Cyp2e1/Cyp2c23/Cyp2g1/Cyp2j8	8
BP	GO:0006805	xenobiotic metabolic process	8/556	124/28814	3.34	0.002852559	0.044842317	0.035384207	Cyp1b1/Gstm2/Cyp2a5/Cyp2e1/Cyp2c23/Cyp2g1/Acaa1b/Cyp2j8	8
BP	GO:0035094	response to nicotine	4/556	31/28814	6.69	0.002854269	0.044842317	0.035384207	Igf2/Chrnb3/Grm2/Ntrk1	4
BP	GO:0035455	response to interferon-alpha	4/556	31/28814	6.69	0.002854269	0.044842317	0.035384207	Ifitm1/Ifitm3/Bst2/Ifitm2	4
BP	GO:0060037	pharyngeal system development	4/556	31/28814	6.69	0.002854269	0.044842317	0.035384207	Bmp5/Nog/Six4/Edn1	4
BP	GO:0072337	modified amino acid transport	4/556	31/28814	6.69	0.002854269	0.044842317	0.035384207	Folr2/Slc6a20a/Slc6a13/Slc16a12	4
BP	GO:0046328	regulation of JNK cascade	9/556	152/28814	3.07	0.002857052	0.044842317	0.035384207	Slamf1/Edar/Trp73/Traf1/Mdfic/Sfrp1/Dab2/Nod2/Serpinf2	9
BP	GO:0050954	sensory perception of mechanical stimulus	14/556	310/28814	2.34	0.00302322	0.047265745	0.037296488	Mcoln3/Serpinb6c/Cdh1/Gjb2/Tbx18/Fzd4/Homer2/Col11a1/Col2a1/Ntrk1/Slc26a5/Myo3b/Tspear/Prrxl1	14
BP	GO:0050670	regulation of lymphocyte proliferation	12/556	245/28814	2.54	0.003059954	0.047473496	0.03746042	Il7/Slamf1/Igf2/Igfbp2/Cd209a/H2-Aa/Prdm1/Cd74/Pde5a/Ikzf3/Vtcn1/Prnp	12
BP	GO:0030104	water homeostasis	6/556	74/28814	4.2	0.003071953	0.047473496	0.03746042	Tfap2b/Aqp1/Cldn1/Cdh1/Alox12b/Scnn1a	6
BP	GO:0060675	ureteric bud morphogenesis	6/556	74/28814	4.2	0.003071953	0.047473496	0.03746042	Nog/Six4/Npnt/Adamts16/Agtr1a/Wt1	6
BP	GO:0014033	neural crest cell differentiation	7/556	99/28814	3.66	0.003099044	0.047708661	0.037645984	Twist1/Aldh1a2/Frzb/Sfrp1/Edn3/Ret/Edn1	7
BP	GO:0016485	protein processing	13/556	278/28814	2.42	0.003123289	0.047898375	0.037795684	Hjv/Cpxm2/Cdh1/Ccbe1/Thbs1/Perp/Casp1/Nkd2/Phex/Adamts3/Tmprss9/Dhh/Serpinf2	13
BP	GO:0014888	striated muscle adaptation	5/556	52/28814	4.98	0.003235939	0.049008227	0.038671447	Myoc/Tcap/Mlip/Pparg/Myh6	5
BP	GO:0032941	secretion by tissue	5/556	52/28814	4.98	0.003235939	0.049008227	0.038671447	Aqp1/Npr3/Trp73/Alox12b/Oprk1	5
BP	GO:0010611	regulation of cardiac muscle hypertrophy	6/556	75/28814	4.15	0.003285762	0.049008227	0.038671447	Nr4a3/Mlip/Pparg/Pde5a/Ccn4/Edn1	6
BP	GO:0072171	mesonephric tubule morphogenesis	6/556	75/28814	4.15	0.003285762	0.049008227	0.038671447	Nog/Six4/Npnt/Adamts16/Agtr1a/Wt1	6
BP	GO:0003073	regulation of systemic arterial blood pressure	8/556	127/28814	3.26	0.003304249	0.049008227	0.038671447	Crh/Edn3/Adamts16/Nkx2-1/Edn1/Agtr1a/Avpr2/Serpinf2	8
BP	GO:0035051	cardiocyte differentiation	10/556	185/28814	2.8	0.003307787	0.049008227	0.038671447	Twist1/Tcap/Tbx18/Mylk3/Ccn4/Rarb/Edn1/Tdgf1/Myh6/Wt1	10
BP	GO:0045834	positive regulation of lipid metabolic process	10/556	185/28814	2.8	0.003307787	0.049008227	0.038671447	Rdh16/Igf2/Twist1/Nr4a3/Cd74/Pparg/Dab2/Nod2/Agtr1a/Fgr	10
BP	GO:0010752	regulation of cGMP-mediated signaling	3/556	16/28814	9.72	0.003318247	0.049008227	0.038671447	Thbs1/Npr1/Pde10a	3
BP	GO:0030238	male sex determination	3/556	16/28814	9.72	0.003318247	0.049008227	0.038671447	Ptgdr/Six4/Dhh	3
BP	GO:0043518	negative regulation of DNA damage response, signal transduction by p53 class mediator	3/556	16/28814	9.72	0.003318247	0.049008227	0.038671447	Twist1/Dyrk3/Cd74	3
BP	GO:0051047	positive regulation of secretion	17/556	417/28814	2.11	0.003329827	0.049008227	0.038671447	Prl/Aqp1/Itgb2l/Lepr/Cartpt/Ptges/Sphk1/Alox12b/Crh/Pparg/Dab2/Edn3/Edn1/Oprk1/Vdr/Retn/Fgr	17
BP	GO:1903039	positive regulation of leukocyte cell-cell adhesion	12/556	248/28814	2.51	0.003375048	0.049492488	0.039053568	Il7/Slamf1/Igf2/Igfbp2/Nr4a3/H2-Aa/H2-Eb1/Cd74/Rhoh/Nlrp3/Vtcn1/Chst4	12
BP	GO:0007389	pattern specification process	19/556	490/28814	2.01	0.003424727	0.050037358	0.039483514	Dll3/Aldh1a2/Ifitm1/Neurod1/Bmp5/Egr2/Emx1/Tcap/Zic2/Nog/Tbx18/Arc/Sfrp1/Nkx2-1/Edn1/Tdgf1/Meox1/Wt1/Peg12	19

BP	GO:0002720	positive regulation of cytokine production involved in immune response	4/305	66/28814	5.73	0.005301417	0.076711754	0.065988053	Il1r1/Ddx58/Nr4a3/Cd36	4
BP	GO:0002703	regulation of leukocyte mediated immunity	9/305	304/28814	2.8	0.005354591	0.077125764	0.066344187	Cxcl1/Cxcl5/Il1r1/Fcgr2b/Ddx58/Il7r/Fcer2a/Il27ra/Il13ra2	9
BP	GO:0044788	modulation by host of viral process	3/305	34/28814	8.34	0.005513139	0.078687527	0.067687628	Ltf/Ccl12/Ifi27	3
BP	GO:1903901	negative regulation of viral life cycle	3/305	34/28814	8.34	0.005513139	0.078687527	0.067687628	Ch25h/Ifitm3/Trim30a	3
BP	GO:0001503	ossification	11/305	421/28814	2.47	0.005557304	0.078958977	0.067921131	Ltf/Cyp27b1/Ifi204/Isg15/Ptgs2/Phex/Runx1/Adrb2/Gli1/Scx/Fstl3	11
BP	GO:0030193	regulation of blood coagulation	4/305	67/28814	5.64	0.005591896	0.079092579	0.068036057	S100a9/Adrb2/Cd36/Serpinc1	4
BP	GO:1903265	positive regulation of tumor necrosis factor-mediated signaling pathway	2/305	11/28814	17.18	0.005766855	0.081201459	0.069850132	Hspa1b/Casp1	2
BP	GO:0000209	protein polyubiquitination	8/305	254/28814	2.98	0.005905058	0.082445892	0.070920603	Nhlrc4/Trim34a/Trim30a/Dtx3l/Ifi27/Parp10/Rnf213/Trim71	8
BP	GO:0050830	defense response to Gram-positive bacterium	6/305	153/28814	3.7	0.005907747	0.082445892	0.070920603	Ltf/Pglyrp1/Il7r/Cd36/Gbp4/Il27ra	6
BP	GO:0044409	entry into host	5/305	109/28814	4.33	0.006138188	0.08528279	0.073360925	Ch25h/Siglec1/Ifitm3/Trim34a/Trim30a	5
BP	GO:0001960	negative regulation of cytokine-mediated signaling pathway	4/305	69/28814	5.48	0.006204308	0.085445299	0.073500717	Oas1a/Sigirr/Isg15/Irak3	4
BP	GO:1900046	regulation of hemostasis	4/305	69/28814	5.48	0.006204308	0.085445299	0.073500717	S100a9/Adrb2/Cd36/Serpinc1	4
BP	GO:0071219	cellular response to molecule of bacterial origin	9/305	314/28814	2.71	0.006570867	0.089026784	0.076581538	Ltf/Cxcl1/Cxcl5/Fcgr4/Ccl12/Trim30a/Fcgr2b/Casp1/Cd36	9
BP	GO:0032642	regulation of chemokine production	5/305	111/28814	4.26	0.006620427	0.089026784	0.076581538	Chil3/Cxcl5/Oas1a/Sigirr/Eif2ak2	5
BP	GO:0048872	homeostasis of number of cells	10/305	372/28814	2.54	0.006631681	0.089026784	0.076581538	Lgals2/Il2ra/Cxcl5/Hspa1b/Isg15/Tnfrsf23/Fas/Fcgr2b/Gpr183/Il7r	10
BP	GO:0032872	regulation of stress-activated MAPK cascade	7/305	207/28814	3.19	0.006738207	0.089026784	0.076581538	Tnfrsf23/Fas/Fcgr2b/Eif2ak2/Sfrp5/Mid1/Mdfi	7
BP	GO:0030888	regulation of B cell proliferation	4/305	71/28814	5.32	0.006859715	0.089026784	0.076581538	Bst1/Cdkn1a/Fcgr2b/Gpr183	4
BP	GO:0050818	regulation of coagulation	4/305	71/28814	5.32	0.006859715	0.089026784	0.076581538	S100a9/Adrb2/Cd36/Serpinc1	4
BP	GO:0043266	regulation of potassium ion transport	5/305	112/28814	4.22	0.006871229	0.089026784	0.076581538	Cxcl1/Drd3/Htr2a/Kcne1l/Adrb2	5
BP	GO:0071346	cellular response to interferon-gamma	5/305	112/28814	4.22	0.006871229	0.089026784	0.076581538	Ccl12/Casp1/Gbp4/Ccl17/Ccl22	5
BP	GO:0002664	regulation of T cell tolerance induction	2/305	12/28814	15.75	0.006872085	0.089026784	0.076581538	Il2ra/Runx1	2
BP	GO:0010744	positive regulation of macrophage derived foam cell differentiation	2/305	12/28814	15.75	0.006872085	0.089026784	0.076581538	Apob/Cd36	2
BP	GO:0033089	positive regulation of T cell differentiation in thymus	2/305	12/28814	15.75	0.006872085	0.089026784	0.076581538	Il2rg/Il7r	2
BP	GO:0033212	iron import into cell	2/305	12/28814	15.75	0.006872085	0.089026784	0.076581538	Lcn2/Tfr2	2
BP	GO:0033632	regulation of cell-cell adhesion mediated by integrin	2/305	12/28814	15.75	0.006872085	0.089026784	0.076581538	Piezo1/Skap1	2
BP	GO:0035726	common myeloid progenitor cell proliferation	2/305	12/28814	15.75	0.006872085	0.089026784	0.076581538	Samd9l/Nr4a3	2
BP	GO:1903131	mononuclear cell differentiation	12/305	495/28814	2.29	0.006889652	0.089026784	0.076581538	Il2ra/Ifi207/Il2rg/Ifi204/Hspb1/Fas/Pglyrp1/Runx1/Hhex/Gpr183/Il7r/Duxbl1	12
BP	GO:0016540	protein autoprocessing	3/305	37/28814	7.66	0.006993839	0.090002686	0.077421017	Myrfl/Pcsk1/Casp1	3
BP	GO:0071868	cellular response to monoamine stimulus	4/305	72/28814	5.25	0.007203925	0.091952536	0.079098293	Drd3/Htr2a/Oprm1/Nr4a3	4
BP	GO:0071870	cellular response to catecholamine stimulus	4/305	72/28814	5.25	0.007203925	0.091952536	0.079098293	Drd3/Htr2a/Oprm1/Nr4a3	4
BP	GO:0043281	regulation of cysteine-type endopeptidase activity involved in apoptotic process	7/305	210/28814	3.15	0.007268136	0.092023974	0.079159744	S100a9/S100a8/Hspa1b/Tnfrsf23/Fas/Ptgs2/Casp1	7
BP	GO:0070302	regulation of stress-activated protein kinase signaling cascade	7/305	210/28814	3.15	0.007268136	0.092023974	0.079159744	Tnfrsf23/Fas/Fcgr2b/Eif2ak2/Sfrp5/Mid1/Mdfi	7
BP	GO:2000377	regulation of reactive oxygen species metabolic process	6/305	160/28814	3.54	0.007302778	0.092091255	0.07921762	Lcn2/Cxcl1/Trim30a/Cdkn1a/Slc5a3/Cd36	6
BP	GO:0002701	negative regulation of production of molecular mediator of immune response	3/305	38/28814	7.46	0.007534665	0.093819466	0.08070424	Fcgr2b/Irak3/Il13ra2	3
BP	GO:0034381	plasma lipoprotein particle clearance	3/305	38/28814	7.46	0.007534665	0.093819466	0.08070424	Apob/Apoc1/Cd36	3
BP	GO:0060761	negative regulation of response to cytokine stimulus	4/305	73/28814	5.18	0.007559339	0.093819466	0.08070424	Oas1a/Sigirr/Isg15/Irak3	4
BP	GO:0098586	cellular response to virus	4/305	73/28814	5.18	0.007559339	0.093819466	0.08070424	Oasl1/Dhx58/Ddx60/Ddx58	4
BP	GO:0002367	cytokine production involved in immune response	5/305	115/28814	4.11	0.007663401	0.094736532	0.081493108	Il1r1/Ddx58/Nr4a3/Irak3/Cd36	5
BP	GO:0032650	regulation of interleukin-1 alpha production	2/305	13/28814	14.53	0.008065106	0.098156722	0.084435182	Nlrp10/Casp1	2
BP	GO:0032926	negative regulation of activin receptor signaling pathway	2/305	13/28814	14.53	0.008065106	0.098156722	0.084435182	Fst/Fstl3	2
BP	GO:0033625	positive regulation of integrin activation	2/305	13/28814	14.53	0.008065106	0.098156722	0.084435182	Piezo1/Skap1	2
BP	GO:1902950	regulation of dendritic spine maintenance	2/305	13/28814	14.53	0.008065106	0.098156722	0.084435182	Fcgr2b/Homer1	2
BP	GO:1904645	response to amyloid-beta	3/305	39/28814	7.27	0.008099546	0.098195268	0.08446834	Fcgr2b/Adrb2/Cd36	3
BP	GO:0035249	synaptic transmission, glutamatergic	5/305	117/28814	4.04	0.008225499	0.098771579	0.084964087	Drd3/Htr2a/Ptgs2/Homer1/Adrb2	5
BP	GO:0042100	B cell proliferation	5/305	117/28814	4.04	0.008225499	0.098771579	0.084964087	Bst1/Cdkn1a/Fcgr2b/Gpr183/Il7r	5
BP	GO:0002886	regulation of myeloid leukocyte mediated immunity	4/305	75/28814	5.04	0.008304362	0.098771579	0.084964087	Cxcl1/Cxcl5/Ddx58/Il13ra2	4
BP	GO:0007422	peripheral nervous system development	4/305	75/28814	5.04	0.008304362	0.098771579	0.084964087	Slc5a3/Sh3tc2/Heyl/Pou4f1	4
BP	GO:0051865	protein autoubiquitination	4/305	75/28814	5.04	0.008304362	0.098771579	0.084964087	Trim30a/Dtx3l/Rnf213/Trim71	4
BP	GO:0070665	positive regulation of leukocyte proliferation	6/305	165/28814	3.44	0.008435157	0.099948649	0.085976612	Il2ra/Bst1/Cdkn1a/Gpr183/H2-DMb2/Il27ra	6
MF	GO:0003725	double-stranded RNA binding	8/300	84/28275	8.98	3.17166E-06	0.001658777	0.001515719	Oasl1/Oas1a/Oas2/Dhx58/Ddx60/Eif2ak2/Oasl2/Ddx58	8
MF	GO:0140375	immune receptor activity	9/300	136/28275	6.24	1.55908E-05	0.004076981	0.003725369	Il2ra/Fcgr4/Il2rg/Il1r1/Fcgr2b/Il7r/Fpr1/Il27ra/Il13ra2	9
MF	GO:0008009	chemokine activity	5/300	41/28275	11.49	7.12689E-05	0.012424553	0.011353018	Cxcl1/Cxcl5/Ccl12/Ccl17/Ccl22	5
MF	GO:0050786	RAGE receptor binding	3/300	10/28275	28.27	0.000134282	0.017557366	0.01604316	S100a8/Fpr1/S100a4	3
MF	GO:0004896	cytokine receptor activity	6/300	97/28275	5.83	0.000597469	0.058673533	0.053613332	Il2ra/Il2rg/Il1r1/Il7r/Il27ra/Il13ra2	6
MF	GO:0001664	G protein-coupled receptor binding	11/300	320/28275	3.24	0.000673119	0.058673533	0.053613332	Cxcl1/Cxcl5/Drd3/Hspa1b/Ccl12/Rtp4/Homer1/Adrb2/Fpr1/Ccl17/Ccl22	11
MF	GO:0042379	chemokine receptor binding	5/300	77/28275	6.12	0.001377495	0.085462764	0.07809217	Cxcl1/Cxcl5/Ccl12/Ccl17/Ccl22	5
MF	GO:0005355	glucose transmembrane transporter activity	3/300	22/28275	12.85	0.001568181	0.085462764	0.07809217	Slc5a3/Slc2a9/Slc5a2	3
MF	GO:0015149	hexose transmembrane transporter activity	3/300	23/28275	12.29	0.001789338	0.085462764	0.07809217	Slc5a3/Slc2a9/Slc5a2	3
MF	GO:0016779	nucleotidyltransferase activity	6/300	120/28275	4.71	0.001804101	0.085462764	0.07809217	Oasl1/Oas1a/Oas2/Oasl2/Pole/Dntt	6

BP	GO:0045066	regulatory T cell differentiation	5/574	42/28814	5.98	0.001427103	0.020894645	0.016442705	Hspb1/Il2rg/Lilrb4a/Ctla2a/Il4i1	5
BP	GO:0071216	cellular response to biotic stimulus	16/574	340/28814	2.36	0.001436721	0.020959522	0.016493759	Vim/Irgm1/Ccl5/Nod2/Fcgr2b/Trim30a/Gch1/Prdm1/Clec7a/Igtp/Tlr1/Lilrb4a/Ldoc	16
BP	GO:0003341	cilium movement	12/574	217/28814	2.78	0.001456113	0.021131328	0.016628959	Ak7/Spag17/Dnah10/Hydin/Dnah12/Dnali1/Ttc29/Ccr6/Tacr2/Enkur/Daw1/Dnah11	12
BP	GO:2001233	regulation of apoptotic signaling pathway	18/574	406/28814	2.23	0.001458956	0.021131328	0.016628959	Cxcl12/Ctsh/Hspb1/Hspa1b/Icam1/Ptprc/Pam16l/Ddias/Muc1/Nupr1/Ngfr/Tnfsf10/Fas/Avp/S100a9/Traf1/Agt/	18
BP	GO:0031392	regulation of prostaglandin biosynthetic process	3/574	12/28814	12.55	0.001512926	0.021757062	0.01712137	Avpr1a/Avp/Anxa1	3
BP	GO:0033632	regulation of cell-cell adhesion mediated by integrin	3/574	12/28814	12.55	0.001512926	0.021757062	0.01712137	Podxl/Piezo1/Ccl5	3
BP	GO:0051403	stress-activated MAPK cascade	13/574	248/28814	2.63	0.001522528	0.021817506	0.017168936	Mid1/Flt4/Mecom/Nod2/Fcgr2b/Fas/Foxm1/Edar/Unc5	13
BP	GO:0008217	regulation of blood pressure	12/574	220/28814	2.74	0.001634853	0.023344316	0.018370435	Eng/Kdr/Adm/Crh/Gch1/Rarres2/Enpep/Avpr1a/Avp/Oxt/Glp1r/Agt	12
BP	GO:0035813	regulation of renal sodium excretion	4/574	26/28814	7.72	0.001644525	0.023399732	0.018414043	Avpr1a/Avp/Oxt/Agt	4
BP	GO:0043087	regulation of GTPase activity	16/574	345/28814	2.33	0.001666972	0.023635909	0.018599898	Dock6/Pecam1/Arap3/Foxj1/Arhgef5/Rasal3/Rasip1/Icam1/Asap3/Dynlt1b/Ccl6/Ccl	16
BP	GO:0071219	cellular response to molecule of bacterial origin	15/574	314/28814	2.4	0.001726822	0.024398915	0.019200334	Vim/Irgm1/Ccl5/Nod2/Fcgr2b/Trim30a/Gch1/Prdm1/Igtp/Tlr1/Lilrb4a/Ldoc1/Gbp2/Ccl12/Gbp6	15
BP	GO:0048469	cell maturation	12/574	222/28814	2.71	0.001763757	0.024833938	0.019542668	Rec8/Kdr/Bfsp2/Gata2/Myoc/Ccnb1/Klf2/Sox18/Nppc/Rxfp2/Ccr6/Tal1	12
BP	GO:0070665	positive regulation of leukocyte proliferation	10/574	165/28814	3.04	0.001811973	0.025424242	0.020007198	Rasal3/Ptprc/Ccl5/Il12rb2/Csf2rb/Ager/H2-DMb1/Tnfsf9/Slc4a1/Anxa1	10
BP	GO:0050766	positive regulation of phagocytosis	7/574	88/28814	3.99	0.001895697	0.025944598	0.020416685	Gata2/Ptprc/Nod2/Il2rg/Fcgr2b/Clec7a/Ager	7
BP	GO:0120192	tight junction assembly	7/574	88/28814	3.99	0.001895697	0.025944598	0.020416685	Pecam1/Cdh5/Lsr/Frmpd2/Grhl2/Cldn23/Cldn3	7
BP	GO:0001780	neutrophil homeostasis	4/574	27/28814	7.44	0.001900421	0.025944598	0.020416685	Mecom/Fcgr2b/Lilrb4a/Anxa1	4
BP	GO:0016048	detection of temperature stimulus	4/574	27/28814	7.44	0.001900421	0.025944598	0.020416685	Cxcl12/Ngfr/Rho/Cxcr4	4
BP	GO:0035809	regulation of urine volume	4/574	27/28814	7.44	0.001900421	0.025944598	0.020416685	Adm/Oxt/Slc4a1/Tfap2b	4
BP	GO:0043129	surfactant homeostasis	4/574	27/28814	7.44	0.001900421	0.025944598	0.020416685	Adgrf5/Kdr/Ctsh/Rcn3	4
BP	GO:0045723	positive regulation of fatty acid biosynthetic process	4/574	27/28814	7.44	0.001900421	0.025944598	0.020416685	Avpr1a/Avp/Agt/Anxa1	4
BP	GO:0050926	regulation of positive chemotaxis	4/574	27/28814	7.44	0.001900421	0.025944598	0.020416685	Cxcl12/Kdr/Angpt2/Ager	4
BP	GO:0032874	positive regulation of stress-activated MAPK cascade	9/574	139/28814	3.25	0.001928865	0.026159215	0.020585574	Mid1/Flt4/Nod2/Fcgr2b/Edar/Unc5cl/Ager/Traf1/Eda2r	9
BP	GO:0030949	positive regulation of vascular endothelial growth factor receptor signaling pathway	3/574	13/28814	11.58	0.00193783	0.026159215	0.020585574	Tek/Kdr/Prkd2	3
BP	GO:0070471	uterine smooth muscle contraction	3/574	13/28814	11.58	0.00193783	0.026159215	0.020585574	Tacr2/Oxt/Agt	3
BP	GO:0008630	intrinsic apoptotic signaling pathway in response to DNA damage	8/574	113/28814	3.55	0.001942035	0.026159215	0.020585574	Uaca/Cxcl12/Ikbke/Ddias/Hic1/Muc1/Nupr1/Bcl3	8
BP	GO:0050866	negative regulation of cell activation	12/574	225/28814	2.68	0.001972675	0.026483655	0.020840887	Adgrf5/Foxj1/Hspb1/Cst7/Pglyrp1/Fcgr2b/Prdm1/Lilrb4a/Fas/Hlx/Il4i1/Anxa1	12
BP	GO:0050891	multicellular organismal water homeostasis	6/574	66/28814	4.56	0.002011722	0.026869966	0.021144888	Klf4/Lsr/Sctr/Aqp3/Avp/Tfap2b	6
BP	GO:0019722	calcium-mediated signaling	11/574	196/28814	2.82	0.002014749	0.026869966	0.021144888	Ackr4/Kdr/Irgm1/Ptprc/Igtp/Cmklr1/Avpr1a/3425401B19Rik/Ccr6/Cxcr4/Htr2b	11
BP	GO:0070304	positive regulation of stress-activated protein kinase signaling cascade	9/574	141/28814	3.2	0.002126751	0.028270402	0.022246938	Mid1/Flt4/Nod2/Fcgr2b/Edar/Unc5cl/Ager/Traf1/Eda2r	9
BP	GO:0001655	urogenital system development	17/574	387/28814	2.21	0.002148704	0.028451301	0.022389294	Prom1/Tek/Lama5/Gata2/Gpr4/Mecom/Wnt6/Prdm1/Enpep/Angpt2/Hs3st3a1/Cxcr4/Serpinb5/Sim1/Tfap2b/A	17
BP	GO:0031098	stress-activated protein kinase signaling cascade	13/574	258/28814	2.53	0.002156605	0.028451301	0.022389294	Mid1/Flt4/Mecom/Nod2/Fcgr2b/Fas/Foxm1/Edar/Unc5cl/Ager/Traf1/Eda2r/Agt	13
BP	GO:0030330	DNA damage response, signal transduction by p53 class mediator	6/574	67/28814	4.5	0.002173072	0.028451301	0.022389294	Acer2/Hic1/Muc1/Bcl3/E2f7/Foxm1	6
BP	GO:0033622	integrin activation	4/574	28/28814	7.17	0.002182604	0.028451301	0.022389294	Fn1/Cxcl12/Rasip1/Piezo1	4
BP	GO:0060713	labyrinthine layer morphogenesis	4/574	28/28814	7.17	0.002182604	0.028451301	0.022389294	Adm/Lef1/Grhl2/Llgl2	4
BP	GO:0098581	detection of external biotic stimulus	4/574	28/28814	7.17	0.002182604	0.028451301	0.022389294	Nod2/Pglyrp1/Clec7a/Tlr1	4
BP	GO:0009266	response to temperature stimulus	10/574	170/28814	2.95	0.00225625	0.029129419	0.022922928	Cxcl12/Il1r1/Hspb1/Hspa1b/Ngfr/Slc52a3/Rho/Cxcr4/Htr2b/Agt	10
BP	GO:0048015	phosphatidylinositol-mediated signaling	10/574	170/28814	2.95	0.00225625	0.029129419	0.022922928	Flt1/Fn1/Tek/Kdr/Klf4/Ccl5/Pear1/Myoc/Htr2b/Agt	10
BP	GO:0051250	negative regulation of lymphocyte activation	10/574	170/28814	2.95	0.00225625	0.029129419	0.022922928	Foxj1/Hspb1/Pglyrp1/Fcgr2b/Prdm1/Lilrb4a/Fas/Hlx/Il4i1/Anxa1	10
BP	GO:0032526	response to retinoic acid	7/574	91/28814	3.86	0.002297064	0.029561892	0.023263255	Acer2/Tek/Ctsh/Tie1/Klf4/Tbx1/Aqp3	7
BP	GO:0018108	peptidyl-tyrosine phosphorylation	15/574	324/28814	2.32	0.002333819	0.029929992	0.023552926	Flt1/Pecam1/Tek/Kdr/Lrrk1/Icam1/Flt4/Ptprc/Parp14/Ccl5/Nod2/Il12rb2/Fgfr4/Lilrb4a/Agt	15
BP	GO:0050729	positive regulation of inflammatory response	9/574	143/28814	3.16	0.002340479	0.029929992	0.023552926	Gpr4/Ccl5/Lpl/Nupr1/Ccl3/Ifi35/S100a9/Ager/Lgals2	9
BP	GO:0062208	positive regulation of pattern recognition receptor signaling pathway	5/574	47/28814	5.34	0.00237218	0.030239681	0.023796631	Hspa1b/Rsad2/Tlr1/Ifi35/Ddx60	5
BP	GO:0052548	regulation of endopeptidase activity	16/574	358/28814	2.24	0.002414544	0.030591175	0.024073234	Acer2/Uaca/Klf4/Hspa1b/Serpinb1b/Cst7/Ngfr/Rag1/Tnfsf10/Sox7/Fas/Avp/S100a9/Ager/Serpinb5/Agt	16
BP	GO:0060180	female mating behavior	3/574	14/28814	10.76	0.002430034	0.030591175	0.024073234	Avpr1a/Avp/Oxt	3
BP	GO:0060670	branching involved in labyrinthine layer morphogenesis	3/574	14/28814	10.76	0.002430034	0.030591175	0.024073234	Adm/Grhl2/Llgl2	3
BP	GO:2001279	regulation of unsaturated fatty acid biosynthetic process	3/574	14/28814	10.76	0.002430034	0.030591175	0.024073234	Avpr1a/Avp/Anxa1	3
BP	GO:0043367	CD4-positive, alpha-beta T cell differentiation	7/574	92/28814	3.82	0.002444448	0.03067706	0.02414082	Lef1/Ly9/Il2rg/Rsad2/Bcl3/Hlx/Anxa1	7
BP	GO:0007018	microtubule-based movement	18/574	426/28814	2.12	0.002457109	0.030740485	0.02419073	Kif19a/Ak7/Hspb1/Spag17/Dnah10/Hydin/Dynlt1b/Casc1/Dnah12/Mreg/Dnah3/Dnali1/Ttc29/Ccr6/Tacr2/Enkur/Dcxr?	18
BP	GO:0035812	renal sodium excretion	4/574	29/28814	6.92	0.002492391	0.030990009	0.02438709	Avpr1a/Avp/Oxt/Agt	4
BP	GO:0048875	chemical homeostasis within a tissue	4/574	29/28814	6.92	0.002492391	0.030990009	0.02438709	Adgrf5/Kdr/Ctsh/Rcn3	4
BP	GO:0018212	peptidyl-tyrosine modification	15/574	327/28814	2.3	0.002547104	0.031483641	0.024775545	Flt1/Pecam1/Tek/Kdr/Lrrk1/Icam1/Flt4/Ptprc/Parp14/Ccl5/Nod2/Il12rb2/Fgfr4/Lilrb4a/Agt	15
BP	GO:0038061	NIK/NF-kappaB signaling	8/574	118/28814	3.4	0.002547674	0.031483641	0.024775545	Uaca/Tek/Nod2/Tlr1/Ifi35/Edar/Ager/Agt	8
BP	GO:0048017	inositol lipid-mediated signaling	10/574	173/28814	2.9	0.002562284	0.031567651	0.024841656	Flt1/Fn1/Tek/Kdr/Klf4/Ccl5/Pear1/Myoc/Htr2b/Agt	10
BP	GO:0032965	regulation of collagen biosynthetic process	5/574	48/28814	5.23	0.002605317	0.031806906	0.025029933	Fn1/Vim/Nppc/Scx/Ager	5
BP	GO:0033628	regulation of cell adhesion mediated by integrin	5/574	48/28814	5.23	0.002605317	0.031806906	0.025029933	Acer2/Podxl/Piezo1/Ccl5/Muc1	5
BP	GO:0048246	macrophage chemotaxis	5/574	48/28814	5.23	0.002605317	0.031806906	0.025029933	Ccl5/Rarres2/Ccl3/Cmklr1/Ccl12	5
BP	GO:0045444	fat cell differentiation	13/574	264/28814	2.47	0.002631651	0.032031636	0.025206781	Adgrf5/Lama4/Klf4/Per2/Cebpd/Gata2/Mecom/Lpl/Rarres2/Lrg1/Tph1/Cmklr1/Tfa	13
BP	GO:0043254	regulation of protein-containing complex assembly	18/574	429/28814	2.11	0.002647363	0.032126107	0.025281124	Hjurp/Pecam1/Cdh5/Plekhg2/Arhgef5/Gbp5/Rasip1/Hspa1b/Icam1/Piezo1/Irgm1/Ikbke/Trim30a/Igtp/Fas/Avil/	18
BP	GO:0120193	tight junction organization	7/574	94/28814	3.74	0.002760954	0.033404234	0.026286925	Pecam1/Cdh5/Lsr/Frmpd2/Grhl2/Cldn23/Cldn3	7

BP	GO:0007179	transforming growth factor beta receptor signaling pathway	10/556	186/28814	2.79	0.003437111	0.050037358	0.039483514	Itgb6/Wfikkn2/Fos/Thbs1/Col1a2/Pparg/Dab2/Npnt/Nkx2-1/Tdgf1	10
BP	GO:0071621	granulocyte chemotaxis	8/556	128/28814	3.24	0.003466531	0.050283468	0.039677715	Pf4/Slamf1/Itgb2l/Thbs1/Cd74/Edn3/Edn1/Nod2	8
BP	GO:0032944	regulation of mononuclear cell proliferation	12/556	249/28814	2.5	0.003485718	0.050379917	0.039753821	Il7/Slamf1/Igf2/Igfbp2/Cd209a/H2-Aa/Prdm1/Cd74/Pde5a/Ikzf3/Vtcn1/Prnp	12
BP	GO:0002704	negative regulation of leukocyte mediated immunity	6/556	76/28814	4.09	0.003510458	0.050555626	0.03989247	Klrd1/Slamf1/Igf2/Bst2/Nod2/Zp3r	6
BP	GO:0051092	positive regulation of NF-kappaB transcription factor activity	9/556	157/28814	2.97	0.003543236	0.050845432	0.04012115	Itgb2l/Sphk1/Traf1/Ripk4/Trim34a/Prkch/Ntrk1/Nlrp3/Nod2	9
BP	GO:0002828	regulation of type 2 immune response	4/556	33/28814	6.28	0.003601038	0.051066833	0.040295853	Rsad2/Cd74/Nlrp3/Nod2	4
BP	GO:0034505	tooth mineralization	4/556	33/28814	6.28	0.003601038	0.051066833	0.040295853	Itgb6/Wdr72/Dmp1/Tspear	4
BP	GO:0003002	regionalization	16/556	385/28814	2.15	0.003601318	0.051066833	0.040295853	Dll3/Aldh1a2/Ifitm1/Neurod1/Egr2/Emx1/Tcap/Nog/Tbx18/Arc/Sfrp1/Nkx2-1/Edn1/Tdgf1/Meox1/Wt1	16
BP	GO:0014013	regulation of gliogenesis	8/556	129/28814	3.21	0.003634921	0.051066833	0.040295853	Dll3/Egr2/Trp73/Nog/Pparg/Tspo/Prkch/Spint1	8
BP	GO:0042476	odontogenesis	8/556	129/28814	3.21	0.003634921	0.051066833	0.040295853	Itgb6/Wdr72/Edar/Perp/Dmp1/Phex/Edn1/Tspear	8
BP	GO:0090101	negative regulation of transmembrane receptor protein serine/threonine kinase signaling pathway	8/556	129/28814	3.21	0.003634921	0.051066833	0.040295853	Hjv/Wfikkn2/Nog/Sfrp1/Pparg/Fzd1/Nkx2-1/Tdgf1	8
BP	GO:0007409	axonogenesis	19/556	493/28814	2	0.003656644	0.051193012	0.040395419	Lmx1a/Ocm/Nr4a3/Egr2/Zic2/Nog/Boc/Nrn1/Ntn5/Lama1/Edn3/Ret/Nkx2-1/Ntrk1/Edn1/Rhoh/Gbx1/Nkx2-9/Prrxl1	19
BP	GO:0003007	heart morphogenesis	13/556	284/28814	2.37	0.003740815	0.052189559	0.041181774	Twist1/Aldh1a2/Bmp5/Thbs1/Tcap/Nog/Fzd1/Tead2/Rarb/Col11a1/Col2a1/Edn1/Myh6	13
BP	GO:0071900	regulation of protein serine/threonine kinase activity	15/556	352/28814	2.21	0.003767687	0.052382586	0.041334088	Thbs1/Trp73/Arhgef5/Sfrp1/Pdgfd/Pparg/Fzd4/Dab2/Edn3/Pde5a/Psrc1/Edn1/Trib3/Nod2/Tdgf1	15
BP	GO:0007631	feeding behavior	8/556	130/28814	3.19	0.003809572	0.052600898	0.041506354	Dmbx1/Lepr/Cartpt/Nr4a3/Nkx2-1/Oprk1/Agtr1a/Retn	8
BP	GO:0052126	movement in host environment	8/556	130/28814	3.19	0.003809572	0.052600898	0.041506354	Ch25h/Ifitm1/Ifitm3/Trim34a/Cd74/Ifitm2/Spint1/Agtr1a	8
BP	GO:0097530	granulocyte migration	9/556	159/28814	2.93	0.003851408	0.052976412	0.041802665	Pf4/Slamf1/Itgb2l/Thbs1/Cd74/Edn3/Edn1/Rhoh/Nod2	9
BP	GO:0021761	limbic system development	7/556	103/28814	3.52	0.003863138	0.052976412	0.041802665	Lhx5/Lmx1a/Neurod1/Nr4a3/Trp73/Tmem108/Nkx2-1	7
BP	GO:0001894	tissue homeostasis	13/556	286/28814	2.36	0.003967367	0.053507704	0.042221898	Itgb6/Il7/Hnf1a/Cdh3/Cytl1/Slc28a3/Abca4/Neurod1/Cartpt/Homer2/Col2a1/Gpr55/Nod2	13
BP	GO:0002468	dendritic cell antigen processing and presentation	3/556	17/28814	9.15	0.003972024	0.053507704	0.042221898	Thbs1/Cd74/Nod2	3
BP	GO:0046068	cGMP metabolic process	3/556	17/28814	9.15	0.003972024	0.053507704	0.042221898	Gucy2g/Npr1/Pde5a	3
BP	GO:0070206	protein trimerization	3/556	17/28814	9.15	0.003972024	0.053507704	0.042221898	Scara5/Col1a2/Cd74	3
BP	GO:1903587	regulation of blood vessel endothelial cell proliferation involved in sprouting angiogenesis	3/556	17/28814	9.15	0.003972024	0.053507704	0.042221898	Fgfbp1/Thbs1/Agtr1a	3
BP	GO:0014743	regulation of muscle hypertrophy	6/556	78/28814	3.99	0.003993846	0.053507704	0.042221898	Nr4a3/Mlip/Pparg/Pde5a/Ccn4/Edn1	6
BP	GO:0002705	positive regulation of leukocyte mediated immunity	10/556	190/28814	2.73	0.0039951	0.053507704	0.042221898	Pomc/Klrd1/Sh2d1a/H2-Q1/Slamf1/Itgb2l/Rsad2/Tnfsfm13/Nlrp3/Nod2	10
BP	GO:0070167	regulation of biomineral tissue development	7/556	104/28814	3.49	0.004074719	0.054212656	0.042778161	Ostn/Omd/Twist1/Sgms2/Ddr2/Dmp1/Tent5a	7
BP	GO:0090288	negative regulation of cellular response to growth factor stimulus	7/556	104/28814	3.49	0.004074719	0.054212656	0.042778161	Hjv/Dcn/Thbs1/Nog/Sfrp1/Pparg/Fzd1	7
BP	GO:0010712	regulation of collagen metabolic process	5/556	55/28814	4.71	0.004132089	0.054787586	0.043231827	Mfap4/Ddr2/Pparg/Retn/Serpinf2	5
BP	GO:0098739	import across plasma membrane	10/556	191/28814	2.71	0.004145203	0.054787586	0.043231827	Slc22a2/Folr2/Slc6a20a/Slc6a13/Thbs1/Slc31a1/Scnn1a/Agtr1a/Slc39a5/Prnp	10
BP	GO:0015850	organic hydroxy compound transport	12/556	255/28814	2.44	0.004212791	0.055498341	0.043792671	Pomc/Slc22a2/Hnf1a/Aqp1/Cartpt/Grm2/Crh/Tspo/Dab2/Oprk1/Slc10a4/Agtr1a	12
BP	GO:1901214	regulation of neuron death	16/556	392/28814	2.12	0.004278154	0.056175233	0.044326793	Tfap2b/Crlf1/Fos/Nr4a3/Trp73/Nfatc4/Six4/Rras2/Ddr2/Esr1/Casp1/Fzd1/Tox3/Musk/Ntrk1/Prnp	16
BP	GO:0061041	regulation of wound healing	8/556	133/28814	3.12	0.004372619	0.057228608	0.045157991	Duox1/Tspan8/Cldn1/Thbs1/Ddr2/Ccn4/Clec7a/Serpinf2	8
BP	GO:0030072	peptide hormone secretion	13/556	290/28814	2.32	0.004453765	0.058080142	0.04582992	Hnf1a/Tfap2b/Aqp1/Neurod1/Lepr/Cartpt/Crh/Sfrp1/Pparg/Edn3/Slc9b2/Pde1c/Edn1	13
BP	GO:0048016	inositol phosphate-mediated signaling	5/556	56/28814	4.63	0.004466591	0.058080142	0.04582992	Nfatc4/Homer2/Edn3/Edn1/Prnp	5
BP	GO:0014909	smooth muscle cell migration	7/556	106/28814	3.42	0.004524083	0.058449412	0.046121304	Nr4a3/Cyp1b1/Ddr2/Pdgfd/Pparg/Ccn4/Retn	7
BP	GO:0110149	regulation of biomineralization	7/556	106/28814	3.42	0.004524083	0.058449412	0.046121304	Ostn/Omd/Twist1/Sgms2/Ddr2/Dmp1/Tent5a	7
BP	GO:0021537	telencephalon development	12/556	258/28814	2.41	0.004619394	0.059489503	0.04694202	Lhx5/Lmx1a/Aqp1/Neurod1/Nr4a3/Emx1/Trp73/Tmem108/Efhc1/Btbd3/Rarb/Nkx2-1	12
BP	GO:0071560	cellular response to transforming growth factor beta stimulus	11/556	226/28814	2.52	0.004691124	0.060083556	0.047410775	Itgb6/Wfikkn2/Fos/Thbs1/Col1a2/Pdgfd/Pparg/Dab2/Npnt/Nkx2-1/Tdgf1	11
BP	GO:0022407	regulation of cell-cell adhesion	18/556	468/28814	1.99	0.00469543	0.060083556	0.047410775	Il7/Slamf1/Igf2/Igfbp2/Cdh1/Nr4a3/H2-Aa/Tbx18/H2-Eb1/Cd74/Pde5a/Spint2/Rhoh/Nlrp3/Vtcn1/B4galnt2/Chst4/Prnp	18
BP	GO:0034308	primary alcohol metabolic process	6/556	81/28814	3.84	0.004808936	0.061092897	0.048207227	Rdh16/Bmp5/Cyp1b1/Slc37a2/Akr1c14/Dab2	6
BP	GO:0019748	secondary metabolic process	5/556	57/28814	4.55	0.004819922	0.061092897	0.048207227	Cdh3/Oca2/Cyp1b1/Akr1c14/Cyp2a5	5
BP	GO:0030514	negative regulation of BMP signaling pathway	5/556	57/28814	4.55	0.004819922	0.061092897	0.048207227	Hjv/Nog/Sfrp1/Pparg/Fzd1	5
BP	GO:0008045	motor neuron axon guidance	4/556	36/28814	5.76	0.004953547	0.062326403	0.049180563	Egr2/Nog/Ntn5/Rhoh	4
BP	GO:0045577	regulation of B cell differentiation	4/556	36/28814	5.76	0.004953547	0.062326403	0.049180563	Il7/Prdm1/Sfrp1/Ikzf3	4
BP	GO:0019722	calcium-mediated signaling	10/556	196/28814	2.64	0.004963775	0.062326403	0.049180563	Pomc/Ackr4/Sphk1/Nfatc4/Homer2/Tox3/Trat1/Edn1/Agtr1a/Prnp	10
BP	GO:0019058	viral life cycle	12/556	261/28814	2.38	0.005056792	0.063099967	0.049790967	Ch25h/Ifitm1/Rsad2/Ifitm3/Bst2/Oasl2/Trim34a/Parp10/Cd74/Ifitm2/Spint1/Agtr1a	12
BP	GO:0048705	skeletal system morphogenesis	12/556	261/28814	2.38	0.005056792	0.063099967	0.049790967	Twist1/Col13a1/Thbs1/Nog/Wnt9a/Six4/Sfrp1/Rarb/Col11a1/Col2a1/Vdr/Fgr	12
BP	GO:0007249	I-kappaB kinase/NF-kappaB signaling	11/556	229/28814	2.49	0.005170851	0.064323464	0.050756405	Edar/Traf1/Trim34a/Cd74/Esr1/Casp1/Nfkbia/Edn1/Rhoh/Nod2/Avpr2	11
BP	GO:0001822	kidney development	14/556	330/28814	2.2	0.005230014	0.064858633	0.051178696	Tfap2b/Crlf1/Aqp1/Prdm1/Nog/Six4/Sfrp1/Pdgfd/Npnt/Adamts16/Rarb/Ret/Agtr1a/Wt1	14
BP	GO:0042310	vasoconstriction	7/556	109/28814	3.33	0.00526696	0.065115826	0.051381642	Dusp5/Edn3/Pde5a/Htr1d/Edn1/Agtr1a/Avpr2	7
BP	GO:0071559	response to transforming growth factor beta	11/556	230/28814	2.48	0.005339101	0.065604002	0.051766853	Itgb6/Wfikkn2/Fos/Thbs1/Col1a2/Pdgfd/Pparg/Dab2/Npnt/Nkx2-1/Tdgf1	11
BP	GO:0097529	myeloid leukocyte migration	11/556	230/28814	2.48	0.005339101	0.065604002	0.051766853	Pf4/Slamf1/Itgb2l/Thbs1/Arhgef5/Cd74/Pdgfd/Edn3/Edn1/Rhoh/Nod2	11
BP	GO:0072078	nephron tubule morphogenesis	6/556	83/28814	3.75	0.005416254	0.066349106	0.0523548	Nog/Six4/Npnt/Adamts16/Agtr1a/Wt1	6
BP	GO:0019934	cGMP-mediated signaling	4/556	37/28814	5.6	0.00547063	0.066771644	0.052688217	Aqp1/Thbs1/Npr1/Pde10a	4
BP	GO:0003228	atrial cardiac muscle tissue development	3/556	19/28814	8.18	0.005500601	0.066771644	0.052688217	Nog/Tbx18/Myh6	3
BP	GO:0021542	dentate gyrus development	3/556	19/28814	8.18	0.005500601	0.066771644	0.052688217	Lmx1a/Neurod1/Tmem108	3
BP	GO:0071622	regulation of granulocyte chemotaxis	5/556	59/28814	4.39	0.005585294	0.067595513	0.053338315	Slamf1/Thbs1/Cd74/Edn1/Nod2	5
BP	GO:0030177	positive regulation of Wnt signaling pathway	8/556	139/28814	2.98	0.005688684	0.068434524	0.054000363	Lmx1a/Cdh3/Lgr6/Rspo3/Sfrp1/Dab2/Ccn4/Peg12	8
BP	GO:0032874	positive regulation of stress-activated MAPK cascade	8/556	139/28814	2.98	0.005688684	0.068434524	0.054000363	Slamf1/Edar/Sphk1/Traf1/Dab2/Oprk1/Nod2/Serpinf2	8

MF	GO:0015459	potassium channel regulator activity	4/300	50/28275	7.54	0.001948794	0.085462764	0.07809217	Slc5a3/Sgk2/Kcne1l/Adrb2	4
MF	GO:0015145	monosaccharide transmembrane transporter activity	3/300	25/28275	11.31	0.002287722	0.085462764	0.07809217	Slc5a3/Slc2a9/Slc5a2	3
MF	GO:0019865	immunoglobulin binding	3/300	25/28275	11.31	0.002287722	0.085462764	0.07809217	Fcgr4/Fcgr2b/Fcer2a	3
MF	GO:0051787	misfolded protein binding	3/300	25/28275	11.31	0.002287722	0.085462764	0.07809217	Hspa1b/Hspa5/Sdf2l1	3
MF	GO:0051119	sugar transmembrane transporter activity	3/300	26/28275	10.88	0.002565975	0.089466979	0.081751048	Slc5a3/Slc2a9/Slc5a2	3

BP	GO:0030183	B cell differentiation	10/574	175/28814	2.87	0.002784154	0.033584381	0.026428689	Ptprc/Mfng/Il2rg/Cmtm7/Rag1/Prdm1/Bcl3/Lilrb4a/6030468B19Rik/Il4i1	10
BP	GO:0050671	positive regulation of lymphocyte proliferation	9/574	147/28814	3.07	0.002819003	0.033903544	0.026679849	Rasal3/Ptprc/Ccl5/Il12rb2/Ager/H2-DMb1/Tnfsf9/Slc4a1/Anxa1	9
BP	GO:0060541	respiratory system development	13/574	267/28814	2.44	0.002899463	0.034767739	0.027359913	Kdr/Ctsh/Foxj1/Lama5/Lef1/Flt4/Hydin/Crh/Klf2/Fgfr4/Rcn3/Grhl2/Fendrr	13
BP	GO:0033077	T cell differentiation in thymus	7/574	95/28814	3.7	0.002930532	0.035036334	0.02757128	Foxj1/Ptprc/Il2rg/Il7r/Rag1/Fas/Ccr6	7
BP	GO:0003091	renal water homeostasis	3/574	15/28814	10.04	0.002992887	0.035267729	0.027753373	Sctr/Aqp3/Tfap2b	3
BP	GO:0035815	positive regulation of renal sodium excretion	3/574	15/28814	10.04	0.002992887	0.035267729	0.027753373	Avpr1a/Oxt/Agt	3
BP	GO:0060700	regulation of ribonuclease activity	3/574	15/28814	10.04	0.002992887	0.035267729	0.027753373	Oasl2/Oas1b/Oas2	3
BP	GO:0071695	anatomical structure maturation	14/574	300/28814	2.34	0.002993471	0.035267729	0.027753373	Rec8/Cdh5/Kdr/Bfsp2/Gata2/Myoc/Ccnb1/Mreg/Klf2/Sox18/Nppc/Rxfp2/Ccr6/Tal1	14
BP	GO:0002274	myeloid leukocyte activation	13/574	268/28814	2.44	0.002993524	0.035267729	0.027753373	Adgrf5/Fn1/Gata2/Cst7/Ccl5/Nppc/Tlr1/Lilrb4a/Ifi35/Batf2/Ager/Tnfsf9/Anxa1	13
BP	GO:0042326	negative regulation of phosphorylation	17/574	400/28814	2.13	0.003014707	0.035386486	0.027846826	Ptprb/Cnksr3/Eng/Pecam1/Hspb1/Rasip1/Gbp4/Lrrk1/Ptprc/Parp14/Ccnb1/Nupr1/Acp4/Lilrb4a/Ager/Slc4a1/	17
BP	GO:0002700	regulation of production of molecular mediator of immune response	10/574	177/28814	2.84	0.003021118	0.035386486	0.027846826	Tek/Il1r1/Ptprc/Nod2/Fcgr2b/Rsad2/Bst2/Lilrb4a/6030468B19Rik/Trim6	10
BP	GO:0032946	positive regulation of mononuclear cell proliferation	9/574	149/28814	3.03	0.003085623	0.036037585	0.028359198	Rasal3/Ptprc/Ccl5/Il12rb2/Ager/H2-DMb1/Tnfsf9/Slc4a1/Anxa1	9
BP	GO:0001890	placenta development	10/574	178/28814	2.82	0.003145483	0.036630825	0.028826039	Vwf/Fbn2/Adm/Lef1/Gata2/Prdm1/E2f7/Ldoc1/Grhl2/Llgl2	10
BP	GO:0022408	negative regulation of cell-cell adhesion	11/574	208/28814	2.65	0.003188359	0.03702345	0.029135009	Podxl/Cxcl12/Foxj1/Klf4/Hspb1/Ccm2l/Lef1/Lilrb4a/Hlx/Il4i1/Anxa1	11
BP	GO:0002313	mature B cell differentiation involved in immune response	4/574	31/28814	6.48	0.00319995	0.037051573	0.02915714	Mfng/Bcl3/Lilrb4a/6030468B19Rik	4
BP	GO:0042113	B cell activation	18/574	437/28814	2.07	0.003215765	0.0371283	0.029217519	Foxj1/Lef1/Ptprc/Mfng/Nod2/Il2rg/Fcgr2b/Cmtm7/Il7r/Rag1/Prdm1/Bcl3/Lilrb4a/Fas/6030468B19Rik/Ccr6/Il4	18
BP	GO:0072331	signal transduction by p53 class mediator	9/574	150/28814	3.01	0.003226183	0.037142466	0.029228667	Acer2/Hic1/Muc1/Nupr1/Bcl3/E2f7/Fhit/Foxm1/Eda2r	9
BP	GO:0002718	regulation of cytokine production involved in immune response	7/574	97/28814	3.62	0.003293499	0.037809739	0.029753766	Tek/Il1r1/Nod2/Rsad2/Bst2/Lilrb4a/Trim6	7
BP	GO:2000116	regulation of cysteine-type endopeptidase activity	12/574	240/28814	2.51	0.003343491	0.03827492	0.030119833	Acer2/Uaca/Klf4/Hspa1b/Cst7/Ngfr/Rag1/Tnfsf10/Sox7/Fas/Avp/S100a9	12
BP	GO:0032732	positive regulation of interleukin-1 production	6/574	73/28814	4.13	0.003354526	0.038292762	0.030133873	Gbp5/Hspb1/Nod2/Lpl/Ccl3/Ager	6
BP	GO:0051091	positive regulation of DNA-binding transcription factor activity	13/574	272/28814	2.4	0.00339499	0.038641682	0.03040845	Arhgef5/Hspa1b/Icam1/Prkd2/Nod2/Ikbke/Fcgr2b/Trim30a/Rab7b/Ager/Traf1/Trim6/Eda2r	13
BP	GO:0042088	T-helper 1 type immune response	5/574	51/28814	4.92	0.003404216	0.038641682	0.03040845	Il1r1/Lef1/Bcl3/Hlx/Anxa1	5
BP	GO:0043281	regulation of cysteine-type endopeptidase activity involved in apoptotic process	11/574	210/28814	2.63	0.003428716	0.038810758	0.030541503	Acer2/Uaca/Klf4/Hspa1b/Ngfr/Rag1/Tnfsf10/Sox7/Fas/Avp/S100a9	11
BP	GO:0046328	regulation of JNK cascade	9/574	152/28814	2.97	0.0035224	0.039759824	0.031288354	Flt4/Mecom/Nod2/Fcgr2b/Edar/Unc5cl/Ager/Traf1/Eda2r	9
BP	GO:0045088	regulation of innate immune response	12/574	242/28814	2.49	0.003573547	0.039783932	0.031307326	Gbp5/Irf7/Irgm1/Parp14/Nod2/Ikbke/Tap1/Trim30a/Nlrc5/Igtp/Ifi35/Trim6	12
BP	GO:0030104	water homeostasis	6/574	74/28814	4.07	0.003590348	0.039783932	0.031307326	Klf4/Lsr/Sctr/Aqp3/Avp/Tfap2b	6
BP	GO:2000107	negative regulation of leukocyte apoptotic process	6/574	74/28814	4.07	0.003590348	0.039783932	0.031307326	Cxcl12/Ccl5/Nod2/Fcgr2b/Il7r/Rag1	6
BP	GO:0044062	regulation of excretion	4/574	32/28814	6.27	0.003600254	0.039783932	0.031307326	Avpr1a/Avp/Oxt/Agt	4
BP	GO:0031589	cell-substrate adhesion	16/574	373/28814	2.15	0.003604307	0.039783932	0.031307326	Rpl29/Fn1/Acer2/Pecam1/Parvb/Tek/Kdr/Lama5/Vwf/Egflam/Nid2/Myoc/Angpt2/Tnxb/Olfm4/Agt	16
BP	GO:0050878	regulation of body fluid levels	16/574	373/28814	2.15	0.003604307	0.039783932	0.031307326	Fn1/Klf4/Lsr/Vwf/Adm/Sctr/Pear1/Tph1/Lilrb4a/Aqp3/Oas2/Avp/S100a9/Oxt/Slc4a1/Tfap2b	16
BP	GO:0032717	negative regulation of interleukin-8 production	3/574	16/28814	9.41	0.003629455	0.039783932	0.031307326	Klf4/Bcl3/Anxa1	3
BP	GO:0043084	penile erection	3/574	16/28814	9.41	0.003629455	0.039783932	0.031307326	Avpr1a/Avp/Oxt	3
BP	GO:0061042	vascular wound healing	3/574	16/28814	9.41	0.003629455	0.039783932	0.031307326	Kdr/Gata2/Cxcr4	3
BP	GO:0090051	negative regulation of cell migration involved in sprouting angiogenesis	3/574	16/28814	9.41	0.003629455	0.039783932	0.031307326	Klf4/Mmrn2/Dll4	3
BP	GO:0032649	regulation of interferon-gamma production	8/574	125/28814	3.21	0.003632831	0.039783932	0.031307326	Il1r1/Nod2/Pglyrp1/Il12rb2/Bcl3/Lilrb4a/Ticam2/Tnfsf9	8
BP	GO:0001938	positive regulation of endothelial cell proliferation	7/574	99/28814	3.55	0.003689779	0.040040118	0.031508927	Cxcl12/Kdr/Gata2/Flt4/Prkd2/Lrg1/Htr2b	7
BP	GO:0002753	cytoplasmic pattern recognition receptor signaling pathway	5/574	52/28814	4.83	0.003705767	0.040040118	0.031508927	Hspa1b/Irgm1/Nod2/Igtp/Ddx60	5
BP	GO:0045581	negative regulation of T cell differentiation	5/574	52/28814	4.83	0.003705767	0.040040118	0.031508927	Foxj1/Hspb1/Lilrb4a/Hlx/Anxa1	5
BP	GO:0060428	lung epithelium development	5/574	52/28814	4.83	0.003705767	0.040040118	0.031508927	Foxj1/Klf2/Fgfr4/Rcn3/Grhl2	5
BP	GO:0090279	regulation of calcium ion import	5/574	52/28814	4.83	0.003705767	0.040040118	0.031508927	Cxcl12/Crh/Ccl12/Cxcr4/Calcr	5
BP	GO:0002828	regulation of type 2 immune response	4/574	33/28814	6.08	0.004033221	0.043117051	0.03393027	Nod2/Rsad2/Hlx/Anxa1	4
BP	GO:0033032	regulation of myeloid cell apoptotic process	4/574	33/28814	6.08	0.004033221	0.043117051	0.03393027	Ccl5/Nod2/Fcgr2b/Anxa1	4
BP	GO:0060669	embryonic placenta morphogenesis	4/574	33/28814	6.08	0.004033221	0.043117051	0.03393027	Adm/Lef1/Grhl2/Llgl2	4
BP	GO:1904646	cellular response to amyloid-beta	4/574	33/28814	6.08	0.004033221	0.043117051	0.03393027	Icam1/Fcgr2b/Ngfr/Ager	4
BP	GO:0042742	defense response to bacterium	19/574	483/28814	1.97	0.004126072	0.04396667	0.034598864	Lcn2/Rnf213/Gbp3/Gbp9/Gbp4/Adm/Irgm1/Nod2/Pglyrp1/Mmrn2/Il7r/Rarres2/Igtp/Bcl3/Gbp2/Oas2/Igha/Gbp6/Slpi	19
BP	GO:0045834	positive regulation of lipid metabolic process	10/574	185/28814	2.71	0.004134455	0.04396667	0.034598864	Flt1/Adgrf5/Tek/Sctr/Nod2/Avpr1a/Avp/Htr2b/Agt/Anxa1	10
BP	GO:0019882	antigen processing and presentation	8/574	128/28814	3.14	0.00419474	0.044490663	0.035011212	H2-D1/Icam1/Nod2/H2-Q4/Tap1/Fcgr2b/H2-T10/H2-DMb1	8
BP	GO:0003222	ventricular trabecula myocardium morphogenesis	3/574	17/28814	8.86	0.004342538	0.045142469	0.035524141	Eng/Ccm2l/Dll4	3
BP	GO:0010713	negative regulation of collagen metabolic process	3/574	17/28814	8.86	0.004342538	0.045142469	0.035524141	Fn1/Nppc/Ager	3
BP	GO:0032966	negative regulation of collagen biosynthetic process	3/574	17/28814	8.86	0.004342538	0.045142469	0.035524141	Fn1/Nppc/Ager	3
BP	GO:0042711	maternal behavior	3/574	17/28814	8.86	0.004342538	0.045142469	0.035524141	Avpr1a/Avp/Oxt	3
BP	GO:0045064	T-helper 2 cell differentiation	3/574	17/28814	8.86	0.004342538	0.045142469	0.035524141	Bcl3/Hlx/Anxa1	3
BP	GO:1901569	fatty acid derivative catabolic process	3/574	17/28814	8.86	0.004342538	0.045142469	0.035524141	Hpgd/Cyp4f18/Oxct2b	3
BP	GO:2001028	positive regulation of endothelial cell chemotaxis	3/574	17/28814	8.86	0.004342538	0.045142469	0.035524141	Kdr/Hspb1/Prkd2	3
BP	GO:0008360	regulation of cell shape	9/574	157/28814	2.88	0.004356734	0.045142469	0.035524141	Fn1/Parvb/Arap3/Kdr/Palmd/Icam1/Ccl3/Ccl12/Anxa1	9
BP	GO:2001235	positive regulation of apoptotic signaling pathway	9/574	157/28814	2.88	0.004356734	0.045142469	0.035524141	Ctsh/Ptprc/Nupr1/Ngfr/Tnfsf10/Fas/S100a9/Agt/Srpx	9
BP	GO:1903901	negative regulation of viral life cycle	4/574	34/28814	5.91	0.004500052	0.046508209	0.036598888	Ifitm3/Trim30a/Bst2/Ch25h	4
BP	GO:0019216	regulation of lipid metabolic process	16/574	382/28814	2.1	0.004525025	0.046647007	0.036708112	Flt1/Adgrf5/Tek/Rnf213/Igfbp7/Lsr/Sctr/Nod2/Rarres2/Fgfr4/Avpr1a/Avil/Avp/Htr2b/Agt/Anxa1	16
BP	GO:0033138	positive regulation of peptidyl-serine phosphorylation	8/574	130/28814	3.09	0.00460498	0.047350443	0.03726167	Tek/Irgm1/Prkd2/Igtp/Avp/Calcr/Trim6/Agt	8
BP	GO:0010596	negative regulation of endothelial cell migration	5/574	55/28814	4.56	0.0047249	0.048337517	0.038038432	Klf4/Mmrn2/Dll4/Angpt2/Ager	5
BP	GO:0010712	regulation of collagen metabolic process	5/574	55/28814	4.56	0.0047249	0.048337517	0.038038432	Fn1/Vim/Nppc/Scx/Ager	5
BP	GO:0042063	gliogenesis	15/574	350/28814	2.15	0.004777725	0.048754516	0.038366583	9630013A20Rik/Flt1/Fn1/Vim/Lef1/Myoc/Ccl3/Fas/Ccl12/Cxcr4/S100a9/Ager/Tal1/Agt/Anxa1	15
BP	GO:0043409	negative regulation of MAPK cascade	10/574	189/28814	2.66	0.004801004	0.048868658	0.038456405	Cnksr3/Klf4/Mecom/Ptprc/Dynlt1b/Dusp2/Lilrb4a/Foxm1/Dusp13/Agt	10
BP	GO:1901216	positive regulation of neuron death	8/574	131/28814	3.07	0.004821306	0.048952006	0.038521994	Lcn2/Fcgr2b/Nupr1/Ngfr/Ccl3/Fas/Ager/Tfap2b	8

BP	GO:0010811	positive regulation of cell-substrate adhesion	8/556	140/28814	2.96	0.005934503	0.070814077	0.055878023	Myoc/Thbs1/Fbln1/Dab2/Dmp1/Npnt/Itga5/Tdgf1	8
BP	GO:0035270	endocrine system development	8/556	140/28814	2.96	0.005934503	0.070814077	0.055878023	Hnf1a/Neurod1/Bmp5/Nog/Crh/Nkx2-1/Edn1/Wt1	8
BP	GO:0048015	phosphatidylinositol-mediated signaling	9/556	170/28814	2.74	0.005939359	0.070814077	0.055878023	Myoc/Twist1/Npr3/Dcn/Pdgfd/Ntrk1/Edn1/Pirt/Fgr	9
BP	GO:0055078	sodium ion homeostasis	5/556	60/28814	4.32	0.005998434	0.071306826	0.056266841	Tfap2b/Sgk1/Scnn1a/Edn1/Avpr2	5
BP	GO:0072210	metanephric nephron development	4/556	38/28814	5.46	0.006022545	0.071382264	0.056326368	Tfap2b/Aqp1/Ret/Wt1	4
BP	GO:0007160	cell-matrix adhesion	11/556	234/28814	2.44	0.006055791	0.071405818	0.056344954	Itgb6/Myoc/Itgb2l/Col13a1/Thbs1/Nid2/Npnt/Itga5/BC024139/Itgax/Tdgf1	11
BP	GO:0030500	regulation of bone mineralization	6/556	85/28814	3.66	0.006077847	0.071405818	0.056344954	Ostn/Omd/Twist1/Sgms2/Ddr2/Tent5a	6
BP	GO:0072088	nephron epithelium morphogenesis	6/556	85/28814	3.66	0.006077847	0.071405818	0.056344954	Nog/Six4/Npnt/Adamts16/Agtr1a/Wt1	6
BP	GO:0098656	anion transmembrane transport	10/556	202/28814	2.57	0.006107804	0.071548565	0.056457593	Slc22a2/Folr2/Slc13a4/Slc6a20a/Slc6a13/Slc37a2/Slc16a12/Slc26a5/Gabrr2/Prnp	10
BP	GO:0008584	male gonad development	8/556	141/28814	2.94	0.006188316	0.072018655	0.056828532	Six4/Sfrp1/Esr1/Nkx2-1/Ntrk1/Adam1b/Wt1/Dhh	8
BP	GO:0070304	positive regulation of stress-activated protein kinase signaling cascade	8/556	141/28814	2.94	0.006188316	0.072018655	0.056828532	Slamf1/Edar/Sphk1/Traf1/Dab2/Oprk1/Nod2/Serpinf2	8
BP	GO:0002274	myeloid leukocyte activation	12/556	268/28814	2.32	0.006206238	0.072018655	0.056828532	Itgb6/Slamf1/Itgb2l/Ptgdr/Ptgds/Nr4a3/Thbs1/Sphk1/Pparg/Casp1/Rhoh/Fgr	12
BP	GO:0002064	epithelial cell development	11/556	235/28814	2.43	0.006246262	0.072018655	0.056828532	Krt2/Cldn1/Cdh1/Bmp5/Prdm1/Fosl2/Esr1/Spint2/Rarb/Ntrk1/Wt1	11
BP	GO:0003018	vascular process in circulatory system	11/556	235/28814	2.43	0.006246262	0.072018655	0.056828532	Slc22a2/Npr3/Mylk3/Dusp5/Edn3/Pde5a/Htr1d/Edn1/Agtr1a/Avpr2/Serpinf2	11
BP	GO:0009615	response to virus	14/556	337/28814	2.15	0.006255478	0.072018655	0.056828532	Itgb6/Ifitm1/Ifi27l2a/Rsad2/Ifitm3/Bst2/Oasl2/Trim34a/Rtp4/Ifitm2/Gbp4/Oprk1/Nlrc3/Itgax	14
BP	GO:0048771	tissue remodeling	10/556	203/28814	2.55	0.006316932	0.072212279	0.056981317	Il7/Hnf1a/Lepr/Cartpt/Rspo3/Sfrp1/Ddr2/Pparg/Gpr55/Vdr	10
BP	GO:0019886	antigen processing and presentation of exogenous peptide antigen via MHC class II	3/556	20/28814	7.77	0.006379584	0.072212279	0.056981317	H2-Aa/H2-Eb1/Cd74	3
BP	GO:0032695	negative regulation of interleukin-12 production	3/556	20/28814	7.77	0.006379584	0.072212279	0.056981317	Slamf1/Thbs1/Nod2	3
BP	GO:0072189	ureter development	3/556	20/28814	7.77	0.006379584	0.072212279	0.056981317	Aldh1a2/Tbx18/Ret	3
BP	GO:0090030	regulation of steroid hormone biosynthetic process	3/556	20/28814	7.77	0.006379584	0.072212279	0.056981317	Igf2/Bmp5/Dab2	3
BP	GO:0070663	regulation of leukocyte proliferation	12/556	269/28814	2.31	0.00638607	0.072212279	0.056981317	Il7/Slamf1/Igf2/Igfbp2/Cd209a/H2-Aa/Prdm1/Cd74/Pde5a/Ikzf3/Vtcn1/Prnp	12
BP	GO:0046661	male sex differentiation	9/556	172/28814	2.71	0.006398101	0.072212279	0.056981317	Bmp5/Six4/Sfrp1/Esr1/Nkx2-1/Ntrk1/Adam1b/Wt1/Dhh	9
BP	GO:0019229	regulation of vasoconstriction	6/556	86/28814	3.62	0.006429835	0.072367167	0.057103536	Dusp5/Edn3/Pde5a/Edn1/Agtr1a/Avpr2	6
BP	GO:0046546	development of primary male sexual characteristics	8/556	142/28814	2.92	0.006450283	0.072394513	0.057125114	Six4/Sfrp1/Esr1/Nkx2-1/Ntrk1/Adam1b/Wt1/Dhh	8
BP	GO:0001706	endoderm formation	4/556	39/28814	5.32	0.006610273	0.073671973	0.058133133	Nog/Dusp4/Dusp5/Itga5	4
BP	GO:0035296	regulation of tube diameter	9/556	173/28814	2.7	0.006637445	0.073671973	0.058133133	Npr3/Dusp5/Edn3/Pde5a/Htr1d/Edn1/Agtr1a/Avpr2/Serpinf2	9
BP	GO:0048017	inositol lipid-mediated signaling	9/556	173/28814	2.7	0.006637445	0.073671973	0.058133133	Myoc/Twist1/Npr3/Dcn/Pdgfd/Ntrk1/Edn1/Pirt/Fgr	9
BP	GO:0097746	blood vessel diameter maintenance	9/556	173/28814	2.7	0.006637445	0.073671973	0.058133133	Npr3/Dusp5/Edn3/Pde5a/Htr1d/Edn1/Agtr1a/Avpr2/Serpinf2	9
BP	GO:2000146	negative regulation of cell motility	13/556	305/28814	2.21	0.006721974	0.074404655	0.058711279	Cdh1/Dcn/Thbs1/Nog/Cyp1b1/Bst2/Fbln1/Cd74/Sfrp1/Pparg/Spint2/[illegible]/Nkx2-1	13
BP	GO:0051881	regulation of mitochondrial membrane potential	6/556	87/28814	3.57	0.006796396	0.074816221	0.059036037	Myoc/Dcn/Casp1/Tspo/Slc4a11/Rnf122	6
BP	GO:0072028	nephron morphogenesis	6/556	87/28814	3.57	0.006796396	0.074816221	0.059036037	Nog/Six4/Npnt/Adamts16/Agtr1a/Wt1	6
BP	GO:0035150	regulation of tube size	9/556	174/28814	2.68	0.006883601	0.075210119	0.059346854	Npr3/Dusp5/Edn3/Pde5a/Htr1d/Edn1/Agtr1a/Avpr2/Serpinf2	9
BP	GO:1903034	regulation of response to wounding	9/556	174/28814	2.68	0.006883601	0.075210119	0.059346854	Dcn/Duox1/Tspan8/Ccn1/Thbs1/Ddr2/Ccn4/Clec7a/Serpinf2	9
BP	GO:0046622	positive regulation of organ growth	5/556	62/28814	4.18	0.006888333	0.075210119	0.059346854	Il7/Igf2/Ccn4/Edn1/Wt1	5
BP	GO:0002062	chondrocyte differentiation	7/556	115/28814	3.15	0.00702267	0.075503131	0.059578064	Cytl1/Loxl2/Wnt9a/Ccn4/Rarb/Col11a1/Col2a1	7
BP	GO:0002367	cytokine production involved in immune response	7/556	115/28814	3.15	0.00702267	0.075503131	0.059578064	Slamf1/Nr4a3/Rsad2/Bst2/Cd74/Nlrp3/Nod2	7
BP	GO:0045765	regulation of angiogenesis	13/556	307/28814	2.19	0.007083463	0.075503131	0.059578064	Aqp1/Itgb2l/Igf2/Dcn/Ccbe1/Thbs1/Sphk1/Cyp1b1/Pparg/Ntrk1/Itga5/Itgax/Agtr1a	13
BP	GO:0010720	positive regulation of cell development	15/556	378/28814	2.06	0.007143629	0.075503131	0.059578064	Myoc/Dcn/Dll3/Egr2/Trp73/Nog/Sgk1/Pparg/Tspo/Dab2/Pde5a/Prkch/Slc9b2/Ret/Spint1	15
BP	GO:0003151	outflow tract morphogenesis	6/556	88/28814	3.53	0.007177863	0.075503131	0.059578064	Twist1/Thbs1/Nog/Fzd1/Rarb/Edn1	6
BP	GO:0071902	positive regulation of protein serine/threonine kinase activity	10/556	207/28814	2.5	0.00720966	0.075503131	0.059578064	Thbs1/Arhgef5/Pdgfd/Fzd4/Edn3/Pde5a/Pirc1/Edn1/Nod2/Tdgf1	10
BP	GO:0001881	receptor recycling	4/556	40/28814	5.18	0.007234763	0.075503131	0.059578064	Rep15/Dab2/Traf1/Agtr1a	4
BP	GO:0030431	sleep	4/556	40/28814	5.18	0.007234763	0.075503131	0.059578064	Ptgdr/Ptgds/Cort/Casp1	4
BP	GO:0048520	positive regulation of behavior	4/556	40/28814	5.18	0.007234763	0.075503131	0.059578064	Nr4a3/Crh/Casp1/Oprk1	4
BP	GO:0060259	regulation of feeding behavior	4/556	40/28814	5.18	0.007234763	0.075503131	0.059578064	Lepr/Nr4a3/Oprk1/Retn	4
BP	GO:1905332	positive regulation of morphogenesis of an epithelium	4/556	40/28814	5.18	0.007234763	0.075503131	0.059578064	Nog/Six4/Itgax/Agtr1a	4
BP	GO:1903037	regulation of leukocyte cell-cell adhesion	14/556	343/28814	2.12	0.007257275	0.075503131	0.059578064	Il7/Slamf1/Igf2/Igfbp2/Nr4a3/H2-Aa/H2-Eb1/Cd74/Pde5a/Rhoh/Nlrp3/Vtcn1/Chst4/Prnp	14
BP	GO:0008406	gonad development	11/556	240/28814	2.38	0.007270235	0.075503131	0.059578064	Lhb/Six4/Sfrp1/Esr1/Fzd4/Nkx2-1/Ntrk1/Retn/Adam1b/Wt1/Dhh	11
BP	GO:0098657	import into cell	11/556	240/28814	2.38	0.007270235	0.075503131	0.059578064	Slc22a2/Folr2/Slc6a20a/Slc6a13/Cubn/Thbs1/Slc31a1/Scnn1a/Agtr1a/Slc39a5/Prnp	11
BP	GO:2000116	regulation of cysteine-type endopeptidase activity	11/556	240/28814	2.38	0.007270235	0.075503131	0.059578064	Slc22a2/Aqp1/Cdh1/Thbs1/Wnt9a/Pparg/Casp1/Ret/Nlrp3/Mgmt	11
BP	GO:0002577	regulation of antigen processing and presentation	3/556	21/28814	7.4	0.007337475	0.075503131	0.059578064	Thbs1/Cd74/Nod2	3
BP	GO:0035994	response to muscle stretch	3/556	21/28814	7.4	0.007337475	0.075503131	0.059578064	Fos/Tcap/Nfkbia	3
BP	GO:0045606	positive regulation of epidermal cell differentiation	3/556	21/28814	7.4	0.007337475	0.075503131	0.059578064	Prkch/Ptch2/Vdr	3
BP	GO:0046321	positive regulation of fatty acid oxidation	3/556	21/28814	7.4	0.007337475	0.075503131	0.059578064	Twist1/Nr4a3/Pparg	3
BP	GO:0051900	regulation of mitochondrial depolarization	3/556	21/28814	7.4	0.007337475	0.075503131	0.059578064	Myoc/Dcn/Tspo	3
BP	GO:0070166	enamel mineralization	3/556	21/28814	7.4	0.007337475	0.075503131	0.059578064	Itgb6/Wdr72/Dmp1	3
BP	GO:0002707	negative regulation of lymphocyte mediated immunity	5/556	63/28814	4.11	0.007366159	0.075503131	0.059578064	Klrd1/Slamf1/Igf2/Nod2/Zp3	5
BP	GO:0051785	positive regulation of nuclear division	5/556	63/28814	4.11	0.007366159	0.075503131	0.059578064	Igf2/Cdt1/Sphk1/Edn3/Edn1	5
BP	GO:0090278	negative regulation of peptide hormone secretion	5/556	63/28814	4.11	0.007366159	0.075503131	0.059578064	Cartpt/Crh/Sfrp1/Pparg/Pde1c	5
BP	GO:0002699	positive regulation of immune effector process	13/556	309/28814	2.18	0.007460187	0.076272347	0.060185038	Pomc/Klrd1/Sh2d1a/H2-Q1/Slamf1/Itgb2l/Nr4a3/Rsad2/Tnfsf13/Cd74/Nlrp3/Nod2/Fgr	13
BP	GO:0061333	renal tubule morphogenesis	6/556	89/28814	3.49	0.007574565	0.077245186	0.060952687	Nog/Six4/Npnt/Adamts16/Agtr1a/Wt1	6
BP	GO:0006066	alcohol metabolic process	14/556	345/28814	2.1	0.007618214	0.077493626	0.061148726	Hnf1a/Ch25h/Rdh16/Sptlc3/Lepr/Bmp5/Cubn/Ip6k3/Sphk1/Cyp1b1/Slc37a2/Akr1c14/Dab2/[illegible]	14
BP	GO:1901342	regulation of vasculature development	13/556	311/28814	2.17	0.007852581	0.079523533	0.062750486	Aqp1/Itgb2l/Igf2/Dcn/Ccbe1/Thbs1/Sphk1/Cyp1b1/Pparg/Ntrk1/Itga5/Itgax/Agtr1a	13
BP	GO:0035456	response to interferon-beta	5/556	64/28814	4.05	0.007866602	0.079523533	0.062750486	Ifi47/Ifitm1/Ifitm3/Bst2/Ifitm2	5
BP	GO:0010469	regulation of signaling receptor activity	8/556	147/28814	2.82	0.007888132	0.079523533	0.062750486	Tafa1/Nog/Crh/Arc/Ppara/[illegible]/Edn1	8

BP	GO:0048002	antigen processing and presentation of peptide antigen	6/574	79/28814	3.81	0.004958192	0.050215672	0.039516416	H2-D1/H2-Q4/Tap1/Fcgr2b/H2-T10/H2-DMb1	6
BP	GO:0001556	oocyte maturation	4/574	35/28814	5.74	0.00500192	0.050280492	0.039567425	Rec8/Ccnb1/Nppc/Rxfp2	4
BP	GO:0014002	astrocyte development	4/574	35/28814	5.74	0.00500192	0.050280492	0.039567425	Vim/S100a9/Ager/Agt	4
BP	GO:0043537	negative regulation of blood vessel endothelial cell migration	4/574	35/28814	5.74	0.00500192	0.050280492	0.039567425	Klf4/Mmrn2/Dll4/Angpt2	4
BP	GO:0001933	negative regulation of protein phosphorylation	15/574	352/28814	2.14	0.005029849	0.050435781	0.039689627	Ptprb/Cnksr3/Eng/Pecam1/Hspb1/Rasip1/Gbp4/Lrrk1/Ptprc/Parp14/Ccnb1/Acp4/Lilrb4a/Ager/Agt	15
BP	GO:0007588	excretion	5/574	56/28814	4.48	0.005104831	0.050934869	0.040082376	Avpr1a/Abcg3/Avp/Oxt/Agt	5
BP	GO:0043536	positive regulation of blood vessel endothelial cell migration	5/574	56/28814	4.48	0.005104831	0.050934869	0.040082376	Kdr/Hspb1/Gata2/Prkd2/Anxa1	5
BP	GO:0033631	cell-cell adhesion mediated by integrin	3/574	18/28814	8.37	0.005134673	0.050980868	0.040118575	Podxl/Piezo1/Ccl5	3
BP	GO:0060746	parental behavior	3/574	18/28814	8.37	0.005134673	0.050980868	0.040118575	Avpr1a/Avp/Oxt	3
BP	GO:0050732	negative regulation of peptidyl-tyrosine phosphorylation	5/574	57/28814	4.4	0.005505892	0.054399293	0.042808649	Pecam1/Lrrk1/Ptprc/Parp14/Lilrb4a	5
BP	GO:0061383	trabecula morphogenesis	5/574	57/28814	4.4	0.005505892	0.054399293	0.042808649	Eng/Tek/Fbn2/Ccm2l/Dll4	5
BP	GO:0045577	regulation of B cell differentiation	4/574	36/28814	5.58	0.005539966	0.05446959	0.042863968	Il2rg/Prdm1/Lilrb4a/Il4i1	4
BP	GO:1904037	positive regulation of epithelial cell apoptotic process	4/574	36/28814	5.58	0.005539966	0.05446959	0.042863968	Ecscr/Cd248/Ccl12/Ager	4
BP	GO:0043547	positive regulation of GTPase activity	12/574	256/28814	2.35	0.005564638	0.054579371	0.042950359	Dock6/Arap3/Foxj1/Arhgef5/Icam1/Asap3/Ccl6/Ccl5/Ccl9/Ccl4/Ccl3/Ccl12	12
BP	GO:0006820	anion transport	19/574	498/28814	1.92	0.005684781	0.055622765	0.043771441	Abcb1a/Slc38a5/Abcc6/Per2/Slco1a4/Clic3/Slc25a24/Gabrr2/Fgfr4/Avpr1a/Abcg3/Slc52a3/Gabrq/Avp/Slc26a7/Oxt/Slc4a1/Agt/Anxa1	19
BP	GO:0045862	positive regulation of proteolysis	15/574	357/28814	2.11	0.005707605	0.055711186	0.043841022	Acer2/Uaca/Ctsh/Hspa1b/Nupr1/Ngfr/Ecscr/Tnfsf10/Sox7/Fgfr4/Fas/Rcn3/Cldn3/S100a9/Ager	15
BP	GO:0045861	negative regulation of proteolysis	15/574	358/28814	2.1	0.005851616	0.05685707	0.044742757	Klf4/Hspa1b/Serpinb1b/Cst7/Wfdc1/Acp4/Rag1/Fhit/Ctla2a/Spink4/Avp/Wfikkn2/Serpinb5/Agt/Slpi	15
BP	GO:0042311	vasodilation	5/574	58/28814	4.33	0.005928688	0.05685707	0.044742757	Kdr/Gch1/Klf2/Kcnmb1/Agt	5
BP	GO:0090303	positive regulation of wound healing	5/574	58/28814	4.33	0.005928688	0.05685707	0.044742757	Clec7a/Cldn3/Cxcr4/S100a9/Anxa1	5
BP	GO:0019724	B cell mediated immunity	13/574	291/28814	2.24	0.005941349	0.05685707	0.044742757	C4b/Foxj1/Irf7/Ptprc/Nod2/Fcgr2b/Bcl3/Fas/6030468B19Rik/Csf2rb/Ccr6/Igha/H2-DMb1	13
BP	GO:0002711	positive regulation of T cell mediated immunity	6/574	82/28814	3.67	0.00594319	0.05685707	0.044742757	H2-D1/Il1r1/Ptprc/H2-Q4/Rsad2/H2-T10	6
BP	GO:0070830	bicellular tight junction assembly	6/574	82/28814	3.67	0.00594319	0.05685707	0.044742757	Pecam1/Cdh5/Frmpd2/Grhl2/Cldn23/Cldn3	6
BP	GO:0014061	regulation of norepinephrine secretion	3/574	19/28814	7.93	0.006008154	0.05685707	0.044742757	Crh/Oxt/Agt	3
BP	GO:0016045	detection of bacterium	3/574	19/28814	7.93	0.006008154	0.05685707	0.044742757	Nod2/Pglyrp1/Tlr1	3
BP	GO:0048245	eosinophil chemotaxis	3/574	19/28814	7.93	0.006008154	0.05685707	0.044742757	Ccl5/Ccl4/Ccl12	3
BP	GO:0055062	phosphate ion homeostasis	3/574	19/28814	7.93	0.006008154	0.05685707	0.044742757	Abcc6/Fgfr4/Tfap2b	3
BP	GO:0060033	anatomical structure regression	3/574	19/28814	7.93	0.006008154	0.05685707	0.044742757	Flt1/Lef1/Cd248	3
BP	GO:0072506	trivalent inorganic anion homeostasis	3/574	19/28814	7.93	0.006008154	0.05685707	0.044742757	Abcc6/Fgfr4/Tfap2b	3
BP	GO:0009582	detection of abiotic stimulus	8/574	136/28814	2.95	0.006021981	0.05685707	0.044742757	Cxcl12/Piezo1/Tcap/Ngfr/Guca1b/Rpe65/Rho/Cxcr4	8
BP	GO:0009791	post-embryonic development	8/574	136/28814	2.95	0.006021981	0.05685707	0.044742757	Flt1/Kdr/Klf4/Mecom/Prdm1/Nppc/Llgl2/Fendrr	8
BP	GO:0035909	aorta morphogenesis	4/574	37/28814	5.43	0.006115299	0.057469591	0.045224771	Eng/Dll4/Tbx1/Tfap2b	4
BP	GO:0061384	heart trabecula morphogenesis	4/574	37/28814	5.43	0.006115299	0.057469591	0.045224771	Eng/Tek/Ccm2l/Dll4	4
BP	GO:0032755	positive regulation of interleukin-6 production	7/574	109/28814	3.22	0.006238382	0.058224492	0.045818828	Nod2/Lpl/Rab7b/Tlr1/Ager/Ticam2/Tnfsf9	7
BP	GO:0042310	vasoconstriction	7/574	109/28814	3.22	0.006238382	0.058224492	0.045818828	Per2/Adm/Icam1/Avpr1a/Avp/Htr2b/Agt	7
BP	GO:0033135	regulation of peptidyl-serine phosphorylation	9/574	166/28814	2.72	0.006238853	0.058224492	0.045818828	Cnksr3/Tek/Irgm1/Prkd2/Igtp/Avp/Calcr/Trim6/Agt	9
BP	GO:0009581	detection of external stimulus	8/574	137/28814	2.93	0.006287161	0.058540128	0.046067213	Cxcl12/Piezo1/Tcap/Ngfr/Guca1b/Rpe65/Rho/Cxcr4	8
BP	GO:1903793	positive regulation of anion transport	5/574	59/28814	4.25	0.006373816	0.059210553	0.046594793	Abcb1a/Avpr1a/Avp/Oxt/Agt	5
BP	GO:0001906	cell killing	12/574	261/28814	2.31	0.006458708	0.059861556	0.047107089	H2-D1/Cxcl12/Ctsh/Ptprc/H2-Q4/Pglyrp1/Tap1/Il7r/Clec7a/H2-T10/Ccl12/Ager	12
BP	GO:0032609	interferon-gamma production	8/574	138/28814	2.91	0.006561103	0.060671436	0.047744411	Il1r1/Nod2/Pglyrp1/Il12rb2/Bcl3/Lilrb4a/Ticam2/Tnfsf9	8
BP	GO:0007259	receptor signaling pathway via JAK-STAT	10/574	198/28814	2.54	0.006609169	0.060976371	0.047984375	Pecam1/Ptprc/Parp14/Ccl5/Il7r/Il12rb2/Bcl3/Csf2rb/Ager/Agt	10
BP	GO:0010466	negative regulation of peptidase activity	12/574	262/28814	2.3	0.006650424	0.061217226	0.048173912	Klf4/Hspa1b/Serpinb1b/Cst7/Wfdc1/Rag1/Spink4/Avp/Wfikkn2/Serpinb5/Agt/Slpi	12
BP	GO:0072089	stem cell proliferation	6/574	84/28814	3.59	0.006674473	0.061298967	0.048238237	Nes/Abcb1a/Etv6/Mecom/Ptprc/Sox18	6
BP	GO:0055078	sodium ion homeostasis	5/574	60/28814	4.18	0.006841868	0.062693856	0.049335922	Avpr1a/Avp/Oxt/Tfap2b/Agt	5
BP	GO:0032642	regulation of chemokine production	7/574	111/28814	3.17	0.00687529	0.062715678	0.049353095	Klf4/Ccl5/Nod2/Lpl/Lilrb4a/Ager/Ticam2	7
BP	GO:1902106	negative regulation of leukocyte differentiation	7/574	111/28814	3.17	0.00687529	0.062715678	0.049353095	Foxj1/Hspb1/Gata2/Pglyrp1/Lilrb4a/Hlx/Anxa1	7
BP	GO:0032495	response to muramyl dipeptide	3/574	20/28814	7.53	0.00696504	0.063107008	0.049661046	Vim/Nod2/Ldoc1	3
BP	GO:0033033	negative regulation of myeloid cell apoptotic process	3/574	20/28814	7.53	0.00696504	0.063107008	0.049661046	Ccl5/Nod2/Fcgr2b	3
BP	GO:0048243	norepinephrine secretion	3/574	20/28814	7.53	0.00696504	0.063107008	0.049661046	Crh/Oxt/Agt	3
BP	GO:0003205	cardiac chamber development	10/574	200/28814	2.51	0.007074647	0.06395671	0.050329705	Eng/Tek/Ccm2l/Dsp/Prdm1/Dll4/Tbx1/Grhl2/Cxcr4/Dnah11	10
BP	GO:0002244	hematopoietic progenitor cell differentiation	9/574	170/28814	2.66	0.007252776	0.065420689	0.05148176	Flt1/Apobec3/Kdr/Top2a/Cebpd/Flt4/Ptprc/Kcp/Tal1	9
BP	GO:0002262	myeloid cell homeostasis	10/574	201/28814	2.5	0.0073167	0.065850299	0.051819835	Adgrf5/Hspa1b/Gata2/Mecom/Fcgr2b/Klf2/Lilrb4a/Slc4a1/Tal1/Anxa1	10
BP	GO:0033028	myeloid cell apoptotic process	4/574	39/28814	5.15	0.007382097	0.066291232	0.05216682	Ccl5/Nod2/Fcgr2b/Anxa1	4
BP	GO:0032102	negative regulation of response to external stimulus	16/574	403/28814	1.99	0.00743255	0.066479608	0.052315059	Cdh5/Uaca/Cst7/Wfdc1/Sema3g/Parp14/Nod2/Pglyrp1/Tap1/Fcgr2b/Prdm1/Nlrc5/Ctla2a/Angpt2/Ccl12/Cldn3	16
BP	GO:0071887	leukocyte apoptotic process	8/574	141/28814	2.85	0.007437302	0.066479608	0.052315059	Cxcl12/Ccl5/Nod2/Fcgr2b/Il7r/Rag1/Fas/Anxa1	8
BP	GO:0043525	positive regulation of neuron apoptotic process	6/574	86/28814	3.5	0.007468879	0.066479608	0.052315059	Lcn2/Nupr1/Ccl3/Fas/Ager/Tfap2b	6
BP	GO:0060415	muscle tissue morphogenesis	6/574	86/28814	3.5	0.007468879	0.066479608	0.052315059	Eng/Ccm2l/Dsp/Tcap/Dll4/Tbx1	6
BP	GO:0030099	myeloid cell differentiation	17/574	439/28814	1.94	0.007490233	0.066523147	0.052349321	Adgrf5/Hspa1b/Lrrk1/Lef1/Gata2/Ccl5/Klf2/H4c17/Rab7b/Ccl3/Lilrb4a/Fas/Batf2/Calcr/Tnfsf9/Slc4a1/Tal1	17
BP	GO:0019730	antimicrobial humoral response	9/574	171/28814	2.64	0.007524886	0.066550778	0.052371065	Cxcl12/Adm/Nod2/Pglyrp1/Rarres2/Bcl3/Ccl12/S100a9/Slpi	9

BP	GO:0010613	positive regulation of cardiac muscle hypertrophy	4/556	41/28814	5.06	0.007896936	0.079523533	0.062750486	Nr4a3/Pde5a/Ccn4/Edn1	4
BP	GO:0001890	placenta development	9/556	178/28814	2.62	0.00793881	0.079567806	0.062785421	Hnf1a/Igf2/Cdh1/Bmp5/Prdm1/Rspo3/Pparg/Spint2/Spint1	9
BP	GO:0043281	regulation of cysteine-type endopeptidase activity involved in apoptotic process	10/556	210/28814	2.47	0.007940938	0.079567806	0.062785421	Slc22a2/Aqp1/Cdh1/Tbx1/Wnt9a/Pparg/Casp1/Ret/Nlrp3/Mgmt	10
BP	GO:1901652	response to peptide	16/556	420/28814	1.97	0.008102092	0.080980613	0.06390024	Pomc/Igf2/Nr4a3/Egr2/Sgk1/Pparg/Nfkbia/Edn1/Oprk1/Trib3/Slc2a4/Nod2/Agtr1a/Retn/Prnp/Pklr	16
BP	GO:0045137	development of primary sexual characteristics	11/556	244/28814	2.34	0.008180021	0.081416351	0.064244072	[illegible]/Sox4/Sfrp1/Esr1/Fzd4/Nkx2-1/Ntrk1/Retn/Adam1b/Wt1/Dhh	11
BP	GO:0050863	regulation of T cell activation	14/556	348/28814	2.08	0.008186213	0.081416351	0.064244072	Il7/Slamf1/Igf2/Igfbp2/Cd209a/H2-Aa/Prdm1/H2-Eb1/Cd74/Pde5a/Rhoh/Nlrp3/Vtcn1/Prnp	14
BP	GO:0007162	negative regulation of cell adhesion	13/556	313/28814	2.15	0.008261081	0.081824584	0.064566202	Myoc/Cdh1/H2-Aa/Thbs1/Cyp1b1/Fbln1/Cd74/Fzd4/Pde5a/Spint2/Vtcn1/B4galnt2/Prnp	13
BP	GO:0019221	cytokine-mediated signaling pathway	16/556	421/28814	1.97	0.008277423	0.081824584	0.064566202	Pf4/Il7/Crlf1/Duox1/Ifitm1/Lepr/Sphk1/Traf1/Ifitm3/Oasl2/Cd74/Pparg/Casp1/Ifitm2/Nfkbia/Edn1	16
BP	GO:0010743	regulation of macrophage derived foam cell differentiation	3/556	22/28814	7.07	0.008375863	0.081824584	0.064566202	Pparg/Nfkbia/Prkch	3
BP	GO:0046519	sphingoid metabolic process	3/556	22/28814	7.07	0.008375863	0.081824584	0.064566202	Sptlc3/Sphk1/Degs2	3
BP	GO:0048339	paraxial mesoderm development	3/556	22/28814	7.07	0.008375863	0.081824584	0.064566202	Hnf1a/Dll3/Tead2	3
BP	GO:1900746	regulation of vascular endothelial growth factor signaling pathway	3/556	22/28814	7.07	0.008375863	0.081824584	0.064566202	Dcn/Ccbe1/Adamts3	3
BP	GO:0070371	ERK1 and ERK2 cascade	14/556	349/28814	2.08	0.008382826	0.081824584	0.064566202	Slamf1/Dusp9/Dusp4/Fbln1/Cd74/Ddr2/Esr1/Pdgfd/Dab2/Npnt/Gpr55/Edn1/Nod2/Serpinf2	14
BP	GO:0032835	glomerulus development	5/556	65/28814	3.99	0.008390176	0.081824584	0.064566202	Aqp1/Nog/Pdgfd/Ret/Wt1	5
BP	GO:0022600	digestive system process	7/556	119/28814	3.05	0.00841264	0.081845008	0.064582317	Aqp1/Neurod1/Nlrp3/Cftr/Oprk1/Nod2/Vdr	7
BP	GO:0051271	negative regulation of cellular component movement	13/556	314/28814	2.15	0.00847151	0.082002892	0.064706901	Cdh1/Dcn/Thbs1/Nog/Cyp1b1/Bst2/Fbln1/Cd74/Sfrp1/Pparg/Spint2/[illegible]/Nkx2-1	13
BP	GO:0060326	cell chemotaxis	13/556	314/28814	2.15	0.00847151	0.082002892	0.064706901	Pf4/Slamf1/Ch25h/[illegible]/Itgb2l/Thbs1/Arhgef5/Cd74/Pdgfd/Edn3/Edn1/Nod2/Agtr1a	13
BP	GO:0007254	JNK cascade	9/556	180/28814	2.59	0.008510504	0.082002892	0.064706901	Slamf1/Edar/Trp73/Traf1/Mdfic/Sfrp1/Dab2/Nod2/Serpinf2	9
BP	GO:0071466	cellular response to xenobiotic stimulus	9/556	180/28814	2.59	0.008510504	0.082002892	0.064706901	Cyp1b1/Gstm2/Cyp2a5/Cyp2e1/Cyp2c23/Cyp2g1/Acaa1b/Cyp2j8/Prnp	9
BP	GO:0001562	response to protozoan	4/556	42/28814	4.94	0.00859768	0.082251136	0.064902785	Pf4/Gbp8/Gbp4/Vtcn1	4
BP	GO:0014742	positive regulation of muscle hypertrophy	4/556	42/28814	4.94	0.00859768	0.082251136	0.064902785	Nr4a3/Pde5a/Ccn4/Edn1	4
BP	GO:0018149	peptide cross-linking	4/556	42/28814	4.94	0.00859768	0.082251136	0.064902785	Krt2/Dcn/Thbs1/Bgn	4
BP	GO:0007605	sensory perception of sound	12/556	280/28814	2.22	0.008648526	0.082541042	0.065131545	Mcoln3/Serpinb6c/Cdh1/Gjb2/Tbx18/Fzd4/Homer2/Col11a1/Col2a1/Slc26a5/Myo3b/Tspear	12
BP	GO:0051607	defense response to virus	12/556	281/28814	2.21	0.008881736	0.084565913	0.066729331	Ifitm1/Rsad2/Ifitm3/Bst2/Oasl2/Trim34a/Rtp4/Ifitm2/Gbp4/Oprk1/Nlrp3/Itgax	12
BP	GO:0140546	defense response to symbiont	12/556	282/28814	2.21	0.009119819	0.086627504	0.068356093	Ifitm1/Rsad2/Ifitm3/Bst2/Oasl2/Trim34a/Rtp4/Ifitm2/Gbp4/Oprk1/Nlrp3/Itgax	12
BP	GO:0051403	stress-activated MAPK cascade	11/556	248/28814	2.3	0.00917582	0.0867782	0.068475004	Slamf1/Edar/Trp73/Sphk1/Traf1/Mdfic/Sfrp1/Dab2/Oprk1/Nod2/Serpinf2	11
BP	GO:0017015	regulation of transforming growth factor beta receptor signaling pathway	7/556	121/28814	3	0.009178879	0.0867782	0.068475004	Wfikkn2/Thbs1/Pparg/Dab2/Npnt/Nkx2-1/Tdgf1	7
BP	GO:0015711	organic anion transport	14/556	353/28814	2.06	0.009206938	0.086839148	0.068523097	Slc22a2/Folr2/Hnf1a/Slc22a6/Slc6a20a/Slc6a13/Ptges/Slc16a5/Slc37a2/Grm2/Slc16a12/Slc4a11/Edn1/Slc10a4	14
BP	GO:0070301	cellular response to hydrogen peroxide	6/556	93/28814	3.34	0.009320291	0.087285158	0.068875035	Aqp1/Nr4a3/Sphk1/Cyp1b1/Ddr2/Edn1	6
BP	GO:0042092	type 2 immune response	4/556	43/28814	4.82	0.009337848	0.087285158	0.068875035	Rsad2/Cd74/Nlrp3/Nod2	4
BP	GO:0042220	response to cocaine	4/556	43/28814	4.82	0.009337848	0.087285158	0.068875035	Grm2/Crh/Homer2/Oprk1	4
BP	GO:0006869	lipid transport	15/556	390/28814	1.99	0.009360129	0.087285158	0.068875035	Pomc/Hnf1a/Abca4/Thbs1/Ptges/Crh/Pparg/[illegible]/Fzd4/Dab2/Abca6/Edn1/Slc10a4/Agtr1a/Retn	15
BP	GO:0043523	regulation of neuron apoptotic process	12/556	283/28814	2.2	0.009362843	0.087285158	0.068875035	Tfap2b/Crlf1/Nr4a3/Trp73/Nfatc4/Sox4/Esr1/Casp1/Tox3/Musk/Ntrk1/Prnp	12
BP	GO:0002043	blood vessel endothelial cell proliferation involved in sprouting angiogenesis	3/556	23/28814	6.76	0.009496151	0.087628758	0.069146163	Fgfbp1/Thbs1/Agtr1a	3
BP	GO:0090190	positive regulation of branching involved in ureteric bud morphogenesis	3/556	23/28814	6.76	0.009496151	0.087628758	0.069146163	Nog/Six4/Agtr1a	3
BP	GO:0006611	protein export from nucleus	5/556	67/28814	3.87	0.009508745	0.087628758	0.069146163	Ranbp3l/Egr2/Ptpn14/[illegible]/Ankle1	5
BP	GO:0006721	terpenoid metabolic process	5/556	67/28814	3.87	0.009508745	0.087628758	0.069146163	Rdh16/Abca4/Aldh1a2/Cyp1b1/Cyp2e1	5
BP	GO:0007622	rhythmic behavior	5/556	67/28814	3.87	0.009508745	0.087628758	0.069146163	Ptgds/Lepr/Egr2/Cort/Casp1	5
BP	GO:0030258	lipid modification	10/556	216/28814	2.4	0.009573669	0.08768754	0.069192547	Acox2/Twist1/Nr4a3/Alox12b/Pparg/Mboat1/Cyp2e1/Agtr1a/Acaa1b/B4galnt2	10
BP	GO:0034754	cellular hormone metabolic process	7/556	122/28814	2.97	0.009580595	0.08768754	0.069192547	Rdh16/Aldh1a2/Bmp5/Cyp1b1/Akr1c14/Esr1/Dab2	7
BP	GO:0060419	heart growth	7/556	122/28814	2.97	0.009580595	0.08768754	0.069192547	Dusp6/Trp73/Nog/Ccn4/Edn1/Myh6/Wt1	7
BP	GO:0016032	viral process	14/556	355/28814	2.04	0.009642263	0.088051389	0.069479653	Ch25h/Ifitm1/Mdfic/Rsad2/Ifitm3/Bst2/Fbln1/Oasl2/Trim34a/Parp10/Cd74/Ifitm2/Spint1/Agtr1a	14
BP	GO:0010565	regulation of cellular ketone metabolic process	8/556	153/28814	2.71	0.009916763	0.090352733	0.071295599	Rdh16/Twist1/Bmp5/Nr4a3/Cd74/Pparg/Dab2/Trib3	8
BP	GO:0003012	muscle system process	16/556	430/28814	1.93	0.009995652	0.090865452	0.071700176	Myoc/Myh2/Nr4a3/Tcap/Sphk1/Mlip/Pparg/Mybpc1/Edn3/Pde5a/Npnt/Ccn4/Htr1d/Edn1/Myh6/Strit1	16
BP	GO:0007616	long-term memory	4/556	44/28814	4.71	0.010118264	0.09156673	0.072252752	Nfatc4/Sgk1/Arc/Slc2a4	4
BP	GO:0046006	regulation of activated T cell proliferation	4/556	44/28814	4.71	0.010118264	0.09156673	0.072252752	Slamf1/Igf2/Igfbp2/Prnp	4
BP	GO:0010717	regulation of epithelial to mesenchymal transition	6/556	95/28814	3.27	0.010293028	0.092399887	0.072910969	Twist1/Loxl2/Nog/Sfrp1/Dab2/Nkx2-1	6
BP	GO:0045685	regulation of glial cell differentiation	6/556	95/28814	3.27	0.010293028	0.092399887	0.072910969	Dll3/Egr2/Trp73/Nog/[illegible]/Spint1	6
BP	GO:0043588	skin development	13/556	322/28814	2.09	0.010310592	0.092399887	0.072910969	Itgb6/Krt2/Cdh3/Tfap2b/[illegible]/Edar/[illegible]/Col1a2/Fosl2/Alox12b/Prkch/[illegible]/Vdr	13
BP	GO:0045860	positive regulation of protein kinase activity	14/556	358/28814	2.03	0.010325423	0.092399887	0.072910969	Cartpt/Thbs1/Arhgef5/Ddr2/Pdgfd/Fzd4/Edn3/Pde5a/Parc1/Edn1/Nod2/Tdgf1/Agtr1a/Prnp	14
BP	GO:0052548	regulation of endopeptidase activity	14/556	358/28814	2.03	0.010325423	0.092399887	0.072910969	Slc22a2/Serpinb6c/Aqp1/Cdh1/Thbs1/Wnt9a/Perp/Pparg/Casp1/Serpina9/Ret/Nlrp3/Mgmt/Serpinf2	14
BP	GO:0015718	monocarboxylic acid transport	7/556	124/28814	2.93	0.010422331	0.092648066	0.073106802	Hnf1a/Slc6a13/Ptges/Slc16a5/Slc16a12/Edn1/Slc10a4	7
BP	GO:0032963	collagen metabolic process	7/556	124/28814	2.93	0.010422331	0.092648066	0.073106802	Mfap4/Col1a2/Ddr2/Pparg/Adamts3/Retn/Serpinf2	7
BP	GO:1903844	regulation of cellular response to transforming growth factor beta stimulus	7/556	124/28814	2.93	0.010422331	0.092648066	0.073106802	Wfikkn2/Thbs1/Pparg/Dab2/Npnt/Nkx2-1/Tdgf1	7
BP	GO:0002495	antigen processing and presentation of peptide antigen via MHC class II	3/556	24/28814	6.48	0.010699567	0.093781995	0.074001564	H2-Aa/H2-Eb1/Cd74	3
BP	GO:0002710	negative regulation of T cell mediated immunity	3/556	24/28814	6.48	0.010699567	0.093781995	0.074001564	Klrd1/Slamf1/Nod2	3
BP	GO:0032331	negative regulation of chondrocyte differentiation	3/556	24/28814	6.48	0.010699567	0.093781995	0.074001564	Wnt9a/Ccn4/Rarb	3
BP	GO:0032352	positive regulation of hormone metabolic process	3/556	24/28814	6.48	0.010699567	0.093781995	0.074001564	Rdh16/Igf2/Dab2	3

BP	GO:0045639	positive regulation of myeloid cell differentiation	7/574	113/28814	3.11	0.00755922	0.066550778	0.052371065	Hspa1b/Lef1/Gata2/Ccl5/Rab7b/Ccl3/Tal1	7
BP	GO:1903707	negative regulation of hemopoiesis	7/574	113/28814	3.11	0.00755922	0.066550778	0.052371065	Foxj1/Hspb1/Gata2/Pglyrp1/Lilrb4a/Hlx/Anxa1	7
BP	GO:2000106	regulation of leukocyte apoptotic process	7/574	113/28814	3.11	0.00755922	0.066550778	0.052371065	Cxcl12/Ccl5/Nod2/Fcgr2b/Il7r/Rag1/Anxa1	7
BP	GO:0045652	regulation of megakaryocyte differentiation	3/574	21/28814	7.17	0.008007162	0.0698854	0.054995192	Gata2/H4c17/Rab7b	3
BP	GO:0048569	post-embryonic animal organ development	3/574	21/28814	7.17	0.008007162	0.0698854	0.054995192	Flt1/Kdr/Klf4	3
BP	GO:0051900	regulation of mitochondrial depolarization	3/574	21/28814	7.17	0.008007162	0.0698854	0.054995192	Kdr/Myoc/Ngfr	3
BP	GO:0090050	positive regulation of cell migration involved in sprouting angiogenesis	3/574	21/28814	7.17	0.008007162	0.0698854	0.054995192	Kdr/Gata2/Anxa1	3
BP	GO:0030324	lung development	11/574	236/28814	2.34	0.008082614	0.070325687	0.055341669	Kdr/Ctsh/Foxj1/Lama5/Flt4/Crh/Klf2/Fgfr4/Rcn3/Grhl2/Fendrr	11
BP	GO:0050680	negative regulation of epithelial cell proliferation	9/574	173/28814	2.61	0.008092414	0.070325687	0.055341669	Flt1/Eng/Wfdc1/Mmrn2/Nupr1/Ngfr/Dll4/Ccl12/Ager	9
BP	GO:0002367	cytokine production involved in immune response	7/574	115/28814	3.06	0.008292159	0.071754112	0.056465745	Tek/Il1r1/Nod2/Rsad2/Bst2/Lilrb4a/Trim6	7
BP	GO:0051937	catecholamine transport	6/574	88/28814	3.42	0.008329321	0.071754112	0.056465745	Cxcl12/Crh/Syt15/Mapk15/Oxt/Agt	6
BP	GO:0097696	receptor signaling pathway via STAT	10/574	205/28814	2.45	0.0083495	0.071754112	0.056465745	Pecam1/Ptprc/Parp14/Ccl5/Il7r/Il12rb2/Bcl3/Csf2rb/Ager/Agt	10
BP	GO:0001755	neural crest cell migration	5/574	63/28814	3.98	0.008389376	0.071754112	0.056465745	Fn1/Lama5/Sema3g/Tbx1/Htr2b	5
BP	GO:0010517	regulation of phospholipase activity	5/574	63/28814	3.98	0.008389376	0.071754112	0.056465745	Flt1/Ccl5/Htr2b/Agt/Anxa1	5
BP	GO:0032653	regulation of interleukin-10 production	5/574	63/28814	3.98	0.008389376	0.071754112	0.056465745	Nod2/Fcgr2b/Bcl3/Lilrb4a/Ager	5
BP	GO:1905517	macrophage migration	5/574	63/28814	3.98	0.008389376	0.071754112	0.056465745	Ccl5/Rarres2/Ccl3/Cmklr1/Ccl12	5
BP	GO:0050728	negative regulation of inflammatory response	8/574	144/28814	2.79	0.008398836	0.071754112	0.056465745	Cdh5/Uaca/Cst7/Wfdc1/Nod2/Pglyrp1/Fcgr2b/Ctla2a	8
BP	GO:0071222	cellular response to lipopolysaccharide	13/574	304/28814	2.15	0.008428272	0.071853684	0.056544102	Vim/Irgm1/Ccl5/Nod2/Trim30a/Gch1/Prdm1/Igtp/Lilrb4a/Ldoc1/Gbp2/Ccl12/Gbp6	13
BP	GO:0060047	heart contraction	11/574	238/28814	2.32	0.008581126	0.073002797	0.057448378	Adm/Dsp/Kcnj8/Gch1/Tcap/Avpr1a/Trdn/Cxcr4/Oxt/Glp1r/Agt	11
BP	GO:0010959	regulation of metal ion transport	17/574	446/28814	1.91	0.008686221	0.073741636	0.058029795	Cnksr3/Ahnak/Lcn2/Cxcl12/Fxyd5/Icam1/Ccl5/Crh/Fhl4/Trdn/Ccl12/Kcnmb1/Cxcr4/Calcr/Ager/Glp1r/Agt	17
BP	GO:0033627	cell adhesion mediated by integrin	6/574	89/28814	3.38	0.008785207	0.074173377	0.058369547	Acer2/Podxl/Icam1/Piezo1/Ccl5/Muc1	6
BP	GO:0002483	antigen processing and presentation of endogenous peptide antigen	4/574	41/28814	4.9	0.008810498	0.074173377	0.058369547	H2-D1/H2-Q4/Tap1/H2-T10	4
BP	GO:0019885	antigen processing and presentation of endogenous peptide antigen via MHC class I	4/574	41/28814	4.9	0.008810498	0.074173377	0.058369547	H2-D1/H2-Q4/Tap1/H2-T10	4
BP	GO:0030513	positive regulation of BMP signaling pathway	4/574	41/28814	4.9	0.008810498	0.074173377	0.058369547	Eng/Cdh5/Kdr/Kcp	4
BP	GO:0030323	respiratory tube development	11/574	239/28814	2.31	0.008839147	0.074259864	0.058437607	Kdr/Ctsh/Foxj1/Lama5/Flt4/Crh/Klf2/Fgfr4/Rcn3/Grhl2/Fendrr	11
BP	GO:0071902	positive regulation of protein serine/threonine kinase activity	10/574	207/28814	2.43	0.008906164	0.074667653	0.05875851	Flt1/Arhgef5/Irgm1/Ptprc/Nod2/Stil/Igtp/Ager/Htr2b/Agt	10
BP	GO:0008347	glial cell migration	5/574	64/28814	3.92	0.008954888	0.074920709	0.058957648	Fn1/Vim/Ccl3/Fas/Ccl12	5
BP	GO:0001892	embryonic placenta development	7/574	117/28814	3	0.009076089	0.075423729	0.059353491	Adm/Lef1/Gata2/Prdm1/E2f7/Grhl2/Llgl2	7
BP	GO:0044070	regulation of anion transport	7/574	117/28814	3	0.009076089	0.075423729	0.059353491	Abcb1a/Per2/Fgfr4/Avpr1a/Avp/Oxt/Agt	7
BP	GO:0031341	regulation of cell killing	8/574	146/28814	2.75	0.00908967	0.075423729	0.059353491	H2-D1/Ptprc/H2-Q4/Tap1/Il7r/Clec7a/H2-T10/Ager	8
BP	GO:2000045	regulation of G1/S transition of mitotic cell cycle	8/574	146/28814	2.75	0.00908967	0.075423729	0.059353491	Klf4/Rrm2/Muc1/Stil/Fhl4/E2f7/Ccl12/Anxa1	8
BP	GO:0045591	positive regulation of regulatory T cell differentiation	3/574	22/28814	6.85	0.009136139	0.075499256	0.059412926	Il2rg/Lilrb4a/Il4i1	3
BP	GO:0150104	transport across blood-brain barrier	3/574	22/28814	6.85	0.009136139	0.075499256	0.059412926	Abcb1a/Abcg3/Ager	3
BP	GO:0031348	negative regulation of defense response	11/574	241/28814	2.29	0.009373164	0.077299914	0.060829925	Cdh5/Uaca/Cst7/Wfdc1/Parp14/Nod2/Pglyrp1/Tap1/Fcgr2b/Nlrc5/Ctla2a	11
BP	GO:1903038	negative regulation of leukocyte cell-cell adhesion	8/574	147/28814	2.73	0.009450572	0.077487768	0.060977754	Cxcl12/Foxj1/Klf4/Hspb1/Lilrb4a/Hlx/Il4i1/Anxa1	8
BP	GO:0032602	chemokine production	7/574	118/28814	2.98	0.009487791	0.077487768	0.060977754	Klf4/Ccl5/Nod2/Lpl/Lilrb4a/Ager/Ticam2	7
BP	GO:0071675	regulation of mononuclear cell migration	7/574	118/28814	2.98	0.009487791	0.077487768	0.060977754	Cxcl12/Ccl5/Rarres2/Cmklr1/Ccr6/Ccl12/Ager	7
BP	GO:0003229	ventricular cardiac muscle tissue development	5/574	65/28814	3.86	0.009546163	0.077487768	0.060977754	Eng/Ccm2l/Dsp/Dll4/Col14a1	5
BP	GO:0009994	oocyte differentiation	5/574	65/28814	3.86	0.009546163	0.077487768	0.060977754	Rec8/Ccnb1/Nppc/Rxfp2/Sohlh1	5
BP	GO:0032613	interleukin-10 production	5/574	65/28814	3.86	0.009546163	0.077487768	0.060977754	Nod2/Fcgr2b/Bcl3/Lilrb4a/Ager	5
BP	GO:0032835	glomerulus development	5/574	65/28814	3.86	0.009546163	0.077487768	0.060977754	Prom1/Tek/Gpr4/Enpep/Angpt2	5
BP	GO:0018149	peptide cross-linking	4/574	42/28814	4.78	0.009587697	0.077487768	0.060977754	Fn1/Dsp/Egflam/Anxa1	4
BP	GO:0038084	vascular endothelial growth factor signaling pathway	4/574	42/28814	4.78	0.009587697	0.077487768	0.060977754	Kdr/Hspb1/Flt4/Prkd2	4
BP	GO:0060338	regulation of type I interferon-mediated signaling pathway	4/574	42/28814	4.78	0.009587697	0.077487768	0.060977754	Irf7/Ikbke/Nlrc5/Trim6	4
BP	GO:0035082	axoneme assembly	6/574	91/28814	3.31	0.009750085	0.078486241	0.061763486	Foxj1/Ak7/Spag17/Hydin/Daw1/Cfap91	6
BP	GO:0043297	apical junction assembly	6/574	91/28814	3.31	0.009750085	0.078486241	0.061763486	Pecam1/Cdh5/Frmpd2/Grhl2/Cldn23/Cldn3	6
BP	GO:2000241	regulation of reproductive process	10/574	210/28814	2.39	0.009793935	0.078682488	0.061917919	Abcb1a/Fbn2/Rspo1/Nupr1/Nppc/Zp1/Ccr6/Mapk15/Tacr2/Oxt	10
BP	GO:0072001	renal system development	14/574	345/28814	2.04	0.009895205	0.079323448	0.062422312	Prom1/Tek/Lama5/Gpr4/Mecom/Wnt6/Prdm1/Enpep/Angpt2/Hs3st3a1/Cxcr4/Sim1/Tfap2b/Agt	14
BP	GO:0050764	regulation of phagocytosis	7/574	119/28814	2.95	0.009912977	0.079323448	0.062422312	Gata2/Ptprc/Nod2/Il2rg/Fcgr2b/Clec7a/Ager	7
BP	GO:0002720	positive regulation of cytokine production involved in immune response	5/574	66/28814	3.8	0.01016374	0.081009218	0.063748902	Tek/Il1r1/Nod2/Rsad2/Trim6	5
BP	GO:0045776	negative regulation of blood pressure	5/574	66/28814	3.8	0.01016374	0.081009218	0.063748902	Kdr/Crh/Gch1/Oxt/Glp1r	5
BP	GO:0007254	JNK cascade	9/574	180/28814	2.51	0.010337939	0.081038788	0.063772172	Flt4/Mecom/Nod2/Fcgr2b/Edar/Unc5cl/Ager/Traf1/Eda2r	9
BP	GO:0007625	grooming behavior	3/574	23/28814	6.55	0.010353385	0.081038788	0.063772172	Avpr1a/Avp/Oxt	3
BP	GO:0010232	vascular transport	3/574	23/28814	6.55	0.010353385	0.081038788	0.063772172	Abcb1a/Abcg3/Ager	3

BP	GO:1902547	regulation of cellular response to vascular endothelial growth factor stimulus	3/556	24/28814	6.48	0.010699567	0.093781995	0.074001564	Dcn/Ccbe1/Adamts3	3
BP	GO:0046513	ceramide biosynthetic process	5/556	69/28814	3.76	0.010725824	0.093781995	0.074001564	Sptlc3/Sphk1/Sgms2/Alox12b/Degs2	5
BP	GO:0070613	regulation of protein processing	5/556	69/28814	3.76	0.010725824	0.093781995	0.074001564	Cdh1/Ccbe1/Thbs1/Nkd2/Serpinf2	5
BP	GO:0071772	response to BMP	9/556	187/28814	2.49	0.010759955	0.093781995	0.074001564	Tfap2b/Hjv/Bmp5/Nog/Sfrp1/Pparg/Fzd1/Col2a1/Slc39a5	9
BP	GO:0071773	cellular response to BMP stimulus	9/556	187/28814	2.49	0.010759955	0.093781995	0.074001564	Tfap2b/Hjv/Bmp5/Nog/Sfrp1/Pparg/Fzd1/Col2a1/Slc39a5	9
BP	GO:0030522	intracellular receptor signaling pathway	10/556	220/28814	2.36	0.010796774	0.093899217	0.074094061	Twist1/Aldh1a2/Sfrp1/Esr1/Pparg/Dab2/Nfkbia/Rarb/Nod2/Vdr	10
BP	GO:0002702	positive regulation of production of molecular mediator of immune response	7/556	125/28814	2.9	0.010862792	0.094269329	0.07438611	Slamf1/Nr4a3/Rsad2/Tnfsfm13/Cd74/Nlrp3/Nod2	7
BP	GO:0046942	carboxylic acid transport	12/556	289/28814	2.15	0.010928481	0.094325686	0.07443058	Slc22a2/Folr2/Hnf1a/Slc22a6/Slc6a20a/Slc6a13/Ptges/Slc16a5/Grm2/Slc16a12/Edn1/Slc10a4	12
BP	GO:0042554	superoxide anion generation	4/556	45/28814	4.61	0.010939714	0.094325686	0.07443058	Itgb2l/Duox1/Edn1/Agtr1a	4
BP	GO:0097009	energy homeostasis	4/556	45/28814	4.61	0.010939714	0.094325686	0.07443058	Lepr/Nr4a3/Slc35d3/Fcor	4
BP	GO:0030900	forebrain development	15/556	398/28814	1.95	0.011117894	0.095656742	0.075480891	Lhx5/Lmx1a/Aqp1/Aldh1a2/Neurod1/Nr4a3/Zic5/Emx1/Trp73/Nog/Tmem108/Efhc1/Btbd3/Rarb/Nkx2-1	15
BP	GO:0002697	regulation of immune effector process	16/556	436/28814	1.9	0.011290207	0.096931737	0.076486964	Pomc/Klrd1/Sh2d1a/H2-Q1/Slamf1/Itgb2l/Igf2/Nr4a3/Rsad2/Tnfsfm13/Bst2/Cd74/Nlrp3/Nod2/Zp3r/Fgr	16
BP	GO:0070372	regulation of ERK1 and ERK2 cascade	13/556	326/28814	2.07	0.011339782	0.097016383	0.076553757	Slamf1/Dusp9/Dusp4/Fbln1/Cd74/Ddr2/Esr1/Pdgfd/Dab2/Npnt/Gpr55/Nod2/Serpinf2	13
BP	GO:0032720	negative regulation of tumor necrosis factor production	5/556	70/28814	3.7	0.011372503	0.097016383	0.076553757	Pomc/Slamf1/Twist1/Tspo/Nod2	5
BP	GO:0048662	negative regulation of smooth muscle cell proliferation	5/556	70/28814	3.7	0.011372503	0.097016383	0.076553757	Ogn/Npr3/Npr1/Esr1/Pparg	5
BP	GO:1903532	positive regulation of secretion by cell	14/556	363/28814	2	0.011547744	0.098302617	0.0775687	Itgb2l/Lepr/Cartpt/Ptges/Sphk1/Crh/Pparg/Dab2/Edn3/Edn1/Oprk1/Vdr/Retn/Fgr	14
BP	GO:0042692	muscle cell differentiation	16/556	438/28814	1.89	0.011749918	0.099220249	0.078292785	Igf2/Wfikkn2/Lncpint/Tcap/Nfatc4/Tbx18/Six4/Mylk3/Casp1/Npnt/Ccn4/Rarb/Edn1/Tdgf1/Myh6/Wt1	16
BP	GO:0070997	neuron death	16/556	438/28814	1.89	0.011749918	0.099220249	0.078292785	Tfap2b/Crlf1/Fos/Nr4a3/Trp73/Nfatc4/Six4/Rras2/Ddr2/Esr1/Casp1/Fzd1/Tox3/Musk/Ntrk1/Prnp	16
BP	GO:0050870	positive regulation of T cell activation	10/556	223/28814	2.32	0.011789297	0.099220249	0.078292785	Il7/Slamf1/Igf2/Igfbp2/H2-Aa/H2-Eb1/Cd74/Rhoh/Nlrp3/Vtcn1	10
BP	GO:0032768	regulation of monooxygenase activity	4/556	46/28814	4.51	0.011802951	0.099220249	0.078292785	Cdh3/Npr3/Esr1/Vdr	4
BP	GO:0035136	forelimb morphogenesis	4/556	46/28814	4.51	0.011802951	0.099220249	0.078292785	Tfap2b/Twist1/Aldh1a2/Wnt9a	4
BP	GO:0051146	striated muscle cell differentiation	13/556	328/28814	2.05	0.011883388	0.099220249	0.078292785	Igf2/Lncpint/Tcap/Tbx18/Six4/Mylk3/Casp1/Ccn4/Rarb/Edn1/Tdgf1/Myh6/Wt1	13
BP	GO:0060249	anatomical structure homeostasis	13/556	328/28814	2.05	0.011883388	0.099220249	0.078292785	Itgb6/Il7/Hnf1a/Cdh3/Cytl1/Slc28a3/Abca4/Neurod1/Cartpt/Homer2/Col2a1/Gpr55/Nod2	13
BP	GO:0007229	integrin-mediated signaling pathway	6/556	98/28814	3.17	0.011884093	0.099220249	0.078292785	Itgb6/Itgb2l/Dab2/Itga5/Itgax/Fgr	6
BP	GO:0007263	nitric oxide mediated signal transduction	3/556	25/28814	6.22	0.011987166	0.099220249	0.078292785	Neurod1/Thbs1/Pde5a	3
BP	GO:0043567	regulation of insulin-like growth factor receptor signaling pathway	3/556	25/28814	6.22	0.011987166	0.099220249	0.078292785	Cdh3/Igfbp2/Bmp5	3
BP	GO:0090189	regulation of branching involved in ureteric bud morphogenesis	3/556	25/28814	6.22	0.011987166	0.099220249	0.078292785	Nog/Six4/Agtr1a	3
BP	GO:0098743	cell aggregation	3/556	25/28814	6.22	0.011987166	0.099220249	0.078292785	Col11a1/Col2a1/Mpz	3
BP	GO:0007193	adenylate cyclase-inhibiting G protein-coupled receptor signaling pathway	5/556	71/28814	3.65	0.012045226	0.099220249	0.078292785	Npr3/Cort/Htr1d/Edn1/Oprk1	5
BP	GO:0030239	myofibril assembly	5/556	71/28814	3.65	0.012045226	0.099220249	0.078292785	Tcap/Six4/Mylk3/Edn1/Myh6	5
BP	GO:1903556	negative regulation of tumor necrosis factor superfamily cytokine production	5/556	71/28814	3.65	0.012045226	0.099220249	0.078292785	Pomc/Slamf1/Twist1/Tspo/Nod2	5
BP	GO:0007517	muscle organ development	14/556	365/28814	1.99	0.012067124	0.099220249	0.078292785	H1f5/Igf2/Twist1/Fos/Egr2/Lncpint/Tcap/Nog/Six4/Boc/Fzd1/Col11a1/Myh6/Wt1	14
BP	GO:0031098	stress-activated protein kinase signaling cascade	11/556	258/28814	2.21	0.012075336	0.099220249	0.078292785	Slamf1/Edar/Trp73/Sphk1/Traf1/Mdfic/Sfrp1/Dab2/Oprk1/Nod2/Serpinf2	11
CC	GO:0062023	collagen-containing extracellular matrix	34/569	396/28739	4.34	9.86962E-13	3.38528E-10	3.044E-10	Pf4/Omd/Myoc/Igf2/Fmod/Lum/Col13a1/Ogn/Mfap4/Itih2/Prg4/Angptl2/Loxl1/Dcn/Thbs1/Bgn/Col1a2/Loxl2/Col4a6/Fbln1/Sfrp1/Colec12/Ntn5/Lama1/Nid2/Npnt/Col6a5/Lamb3/Col11a1/Col2a1/Impg1/Col6a4/Hmcn2/Serpinf2	34
CC	GO:0005581	collagen trimer	11/569	81/28739	6.86	5.76869E-07	9.89331E-05	8.89593E-05	Lum/Col13a1/Dcn/Ccbe1/Col1a2/Col4a6/Colec12/Col6a5/Col11a1/Col2a1/Col6a4	11
CC	GO:0016323	basolateral plasma membrane	18/569	246/28739	3.7	2.32745E-06	0.000245569	0.000220812	Slc22a2/Aqp1/Slc22a6/Hjv/Cldn1/Cdh1/Slc6a13/Slc16a12/Nkd2/Slc4a11/Slc9b2/Slc26a5/Nod2/Slc26a10/Lpo/Agtr1a/Slc39a5/Mpz	18
CC	GO:0045178	basal part of cell	20/569	301/28739	3.36	2.86378E-06	0.000245569	0.000220812	Slc22a2/Aqp1/Slc22a6/Hjv/Cldn1/Cdh1/Slc6a13/Mpp4/Slc16a12/Nkd2/Slc4a11/Slc9b2/Edn1/Slc26a5/Nod2/Slc26a10/Lpo/Agtr1a/Slc39a5/Mpz	20
CC	GO:0009925	basal plasma membrane	18/569	276/28739	3.29	1.14833E-05	0.000787754	0.000708338	Slc22a2/Aqp1/Slc22a6/Hjv/Cldn1/Cdh1/Slc6a13/Slc16a12/Nkd2/Slc4a11/Slc9b2/Slc26a5/Nod2/Slc26a10/Lpo/Agtr1a/Slc39a5/Mpz	18
CC	GO:0005604	basement membrane	11/569	119/28739	4.67	2.55162E-05	0.001353397	0.001216956	Loxl1/Loxl2/Col4a6/Fbln1/Ntn5/Lama1/Nid2/Npnt/Lamb3/Col2a1/Hmcn2	11
CC	GO:0098644	complex of collagen trimers	5/569	19/28739	13.29	2.76203E-05	0.001353397	0.001216956	Lum/Col1a2/Col4a6/Col11a1/Col2a1	5
CC	GO:0043235	receptor complex	22/569	423/28739	2.63	4.39776E-05	0.001540868	0.001385528	Itgb6/Klrd1/Crlf1/Itgb2l/Hjv/Chrnb3/Lepr/Cubn/Traf1/Cd74/Ddr2/Nrn1/Npr1/Pparg/Trat1/Ret/Musk/Ntrk1/Itga5/Gabrr2/Itgax/Vdr	22
CC	GO:0005583	fibrillar collagen trimer	4/569	11/28739	18.37	4.49233E-05	0.001540868	0.001385528	Lum/Col1a2/Col11a1/Col2a1	4
CC	GO:0098643	banded collagen fibril	4/569	11/28739	18.37	4.49233E-05	0.001540868	0.001385528	Lum/Col1a2/Col11a1/Col2a1	4
CC	GO:0016328	lateral plasma membrane	7/569	67/28739	5.28	0.00035788	0.011159355	0.010034344	Cldn15/Cldn1/Cdh1/Gjb2/Mpp4/Nkd2/Slc26a5	7
CC	GO:0031225	anchored component of membrane	11/569	181/28739	3.07	0.001016192	0.029046153	0.026117916	Folr2/Xpnpep2/Hjv/Bst2/Nrn1/Gad2/Smpdl3b/Nod2/Tdgf1/Vtcn1/Prnp	11
CC	GO:0042613	MHC class II protein complex	3/569	12/28739	12.63	0.001486468	0.039219882	0.035265998	H2-Aa/H2-Eb1/Cd74	3
CC	GO:0030665	clathrin-coated vesicle membrane	5/569	44/28739	5.74	0.001716694	0.042059013	0.037818907	Dab2/Ap1g2/Gad2/Slc18a3/Enthd1	5
MF	GO:0005201	extracellular matrix structural constituent	19/555	150/28275	6.45	1.38846E-10	9.13607E-08	7.52692E-08	Lum/Col13a1/Ogn/Mfap4/Dcn/Thbs1/Bgn/Col1a2/Col4a6/Fbln1/Lama1/Nid2/Npnt/Col6a5/Lamb3/Col11a1/Col2a1/Col6a4/Hmcn2	19
MF	GO:0030020	extracellular matrix structural constituent conferring tensile strength	7/555	38/28275	9.38	8.04769E-06	0.002435446	0.002006487	Col13a1/Col1a2/Col4a6/Col6a5/Col11a1/Col2a1/Col6a4	7
MF	GO:0005539	glycosaminoglycan binding	16/555	233/28275	3.5	1.66695E-05	0.002435446	0.002006487	Pf4/Lyve1/Col13a1/Itih2/Pcolce/Eva1c/Dcn/Thbs1/Bgn/Rspo3/Sfrp1/Ccn4/Col11a1/Impg1/Nod2/Prnp	16
MF	GO:0017147	Wnt-protein binding	6/555	29/28275	10.54	1.80072E-05	0.002435446	0.002006487	Wif1/Frzb/Sfrp1/Fzd1/Fzd4/Tdgf1	6
MF	GO:0001664	G protein-coupled receptor binding	19/555	320/28275	3.02	2.18232E-05	0.002435446	0.002006487	Pomc/Pf4/Myoc/Rtp1/Wnt9a/Rspo3/Crh/Sfrp1/Cort/Rtp4/Esr1/Fzd1/Homer2/Edn3/Ptch2/Edn1/Agtr1a/Wnt8b/Prnp	19
MF	GO:0038024	cargo receptor activity	9/555	78/28275	5.88	2.22077E-05	0.002435446	0.002006487	Folr2/Ackr4/Prg4/Scara5/Cubn/Loxl2/Mrc1/Colec12/Dab2	9
MF	GO:0005179	hormone activity	11/555	132/28275	4.25	6.15386E-05	0.005784628	0.004765771	Pomc/Ostn/Prl/Lhb/Igf2/Cartpt/Crh/Cort/Edn3/Edn1/Retn	11

BP	GO:0032693	negative regulation of interleukin-10 production	3/574	23/28814	6.55	0.010353385	0.081038788	0.063772172	Fcgr2b/Lilrb4a/Ager	3
BP	GO:0033630	positive regulation of cell adhesion mediated by integrin	3/574	23/28814	6.55	0.010353385	0.081038788	0.063772172	Podxl/Piezo1/Ccl5	3
BP	GO:0050961	detection of temperature stimulus involved in sensory perception	3/574	23/28814	6.55	0.010353385	0.081038788	0.063772172	Cxcl12/Rho/Cxcr4	3
BP	GO:0072677	eosinophil migration	3/574	23/28814	6.55	0.010353385	0.081038788	0.063772172	Ccl5/Ccl4/Ccl12	3
BP	GO:2001026	regulation of endothelial cell chemotaxis	3/574	23/28814	6.55	0.010353385	0.081038788	0.063772172	Kdr/Hspb1/Prkd2	3
BP	GO:0003351	epithelial cilium movement involved in extracellular fluid movement	4/574	43/28814	4.67	0.0104081	0.081038788	0.063772172	Ak7/Spag17/Daw1/Dnah11	4
BP	GO:0045823	positive regulation of heart contraction	4/574	43/28814	4.67	0.0104081	0.081038788	0.063772172	Adm/Gch1/Avpr1a/Glp1r	4
BP	GO:0085029	extracellular matrix assembly	4/574	43/28814	4.67	0.0104081	0.081038788	0.063772172	Myh11/Tie1/Tnxb/Agt	4
BP	GO:0098801	regulation of renal system process	4/574	43/28814	4.67	0.0104081	0.081038788	0.063772172	Avpr1a/Avp/Oxt/Agt	4
BP	GO:0060537	muscle tissue development	18/574	493/28814	1.83	0.010685119	0.083035702	0.065343611	Eng/Myh11/Hspg2/Ccm2l/Lef1/Dsp/Maff/Ccnb1/Nupr1/Tcap/Dll4/Mylk3/Tbx1/Hlx/Col14a1/Scx/Tfap2b/Agt	18
BP	GO:0032663	regulation of interleukin-2 production	5/574	67/28814	3.75	0.010808149	0.083830574	0.065969123	Ptprc/Prkd2/Nod2/Lilrb4a/Anxa1	5
BP	GO:0010563	negative regulation of phosphorus metabolic process	17/574	457/28814	1.87	0.01086998	0.083987745	0.066092806	Ptprb/Cnksr3/Eng/Pecam1/Hspb1/Rasip1/Gbp4/Lrrk1/Ptprc/Parp14/Ccnb1/Nupr1/Acp4/Lilrb4a/Ager/Slc4a1/Agt	17
BP	GO:0045936	negative regulation of phosphate metabolic process	17/574	457/28814	1.87	0.01086998	0.083987745	0.066092806	Ptprb/Cnksr3/Eng/Pecam1/Hspb1/Rasip1/Gbp4/Lrrk1/Ptprc/Parp14/Ccnb1/Nupr1/Acp4/Lilrb4a/Ager/Slc4a1/Agt	17
BP	GO:0006909	phagocytosis	15/574	386/28814	1.95	0.011227203	0.08643722	0.068020381	Pecam1/Gata2/Ptprc/Nod2/Pear1/Il2rg/Fcgr2b/Psg16/Rab7b/Clec7a/Ager/Igha/Ticam2/Srpx/Anxa1	15
BP	GO:0001776	leukocyte homeostasis	7/574	122/28814	2.88	0.011271879	0.08643722	0.068020381	Mecom/Fcgr2b/Rag1/Lilrb4a/Fas/Lgals2/Anxa1	7
BP	GO:0009636	response to toxic substance	7/574	122/28814	2.88	0.011271879	0.08643722	0.068020381	Lcn2/Abcb1a/Ccl5/Nupr1/Gch1/Abcg3/Fas	7
BP	GO:0019883	antigen processing and presentation of endogenous antigen	4/574	44/28814	4.56	0.01127256	0.08643722	0.068020381	H2-D1/H2-Q4/Tap1/H2-T10	4
BP	GO:0042475	odontogenesis of dentin-containing tooth	6/574	94/28814	3.2	0.011335416	0.086590579	0.068141065	Lama5/Lef1/Wnt6/Ngfr/Tbx1/Edar	6
BP	GO:0048644	muscle organ morphogenesis	6/574	94/28814	3.2	0.011335416	0.086590579	0.068141065	Eng/Ccm2l/Dsp/Tcap/Dll4/Tbx1	6
BP	GO:0002824	positive regulation of adaptive immune response based on somatic recombination of immune receptors built from immunoglobulin superfamily domains	8/574	152/28814	2.64	0.011417118	0.08672288	0.068245177	H2-D1/Il1r1/Ptprc/Nod2/H2-Q4/Rsad2/H2-T10/6030468B19Rik	8
BP	GO:0003206	cardiac chamber morphogenesis	8/574	152/28814	2.64	0.011417118	0.08672288	0.068245177	Eng/Tek/Ccm2l/Dsp/Dll4/Tbx1/Grhl2/Dnah11	8
BP	GO:0015698	inorganic anion transport	8/574	152/28814	2.64	0.011417118	0.08672288	0.068245177	Abcb1a/Abcc6/Clic3/Gabrr2/Fgfr4/Gabrq/Slc26a7/Slc4a1	8
BP	GO:0050433	regulation of catecholamine secretion	5/574	68/28814	3.69	0.011479907	0.087036221	0.068491755	Cxcl12/Crh/Syt15/Oxt/Agt	5
BP	GO:0051882	mitochondrial depolarization	3/574	24/28814	6.27	0.011660119	0.088236964	0.069436661	Kdr/Myoc/Ngfr	3
BP	GO:0001558	regulation of cell growth	17/574	461/28814	1.85	0.01176421	0.088858268	0.069925586	Fn1/Cxcl12/Igfbp7/Hspa1b/Flt4/Wfdc1/Sema3g/Bst2/Avpr1a/Fhl4/Kazald1/Csf2rb/Hsd3b7/Avp/Cxcr4/Col14a1/Agt	17
BP	GO:0010506	regulation of autophagy	12/574	283/28814	2.13	0.011817902	0.08893136	0.069983104	Acer2/Kdr/Rasip1/Irgm1/Nod2/Trim30a/Nupr1/Igtp/Mapk15/Ager/Htr2b/Trim6	12
BP	GO:0043523	regulation of neuron apoptotic process	12/574	283/28814	2.13	0.011817902	0.08893136	0.069983104	Nes/Lcn2/Kdr/Nupr1/Ngfr/Ccl3/Fas/Ccl12/Ager/Glp1r/Tfap2b/Agt	12
BP	GO:0048864	stem cell development	6/574	95/28814	3.17	0.011902038	0.089104951	0.070119709	Fn1/Lama5/Sema3g/Ptprc/Tbx1/Htr2b	6
BP	GO:0015711	organic anion transport	14/574	353/28814	1.99	0.01190712	0.089104951	0.070119709	Slc38a5/Abcc6/Per2/Slco1a4/Slc25a24/Avpr1a/Abcg3/Slc52a3/Avp/Slc26a7/Oxt/Slc4a1/Agt/Anxa1	14
BP	GO:0042886	amide transport	14/574	353/28814	1.99	0.01190712	0.089104951	0.070119709	Abcb1a/Slc38a5/Per2/Ccl5/Crh/Tap1/Pde4c/Aqp3/Tacr2/S100a9/Glp1r/Tfap2b/Agt/Anxa1	14
BP	GO:0003015	heart process	11/574	250/28814	2.21	0.012090401	0.090309259	0.07106742	Adm/Dsp/Kcnj8/Gch1/Tcap/Avpr1a/Trdn/Cxcr4/Oxt/Glp1r/Agt	11
BP	GO:0050679	positive regulation of epithelial cell proliferation	10/574	217/28814	2.31	0.012126988	0.090324828	0.071079672	Cxcl12/Kdr/Gata2/Flt4/Ccl5/Prkd2/Nod2/Lrg1/Tbx1/Htr2b	10
BP	GO:0042093	T-helper cell differentiation	5/574	69/28814	3.64	0.012179523	0.090324828	0.071079672	Lef1/Ly9/Bcl3/Hlx/Anxa1	5
BP	GO:0045600	positive regulation of fat cell differentiation	5/574	69/28814	3.64	0.012179523	0.090324828	0.071079672	Mecom/Lpl/Rarres2/Tph1/Cmklr1	5
BP	GO:0050918	positive chemotaxis	4/574	45/28814	4.46	0.012181893	0.090324828	0.071079672	Cxcl12/Kdr/Angpt2/Ager	4
BP	GO:0002224	toll-like receptor signaling pathway	7/574	124/28814	2.83	0.012249672	0.09049529	0.071213814	Irf7/Nod2/Trim30a/Rab7b/Rsad2/Tlr1/Ifi35	7
BP	GO:0002286	T cell activation involved in immune response	7/574	124/28814	2.83	0.012249672	0.09049529	0.071213814	Icam1/Lef1/Ly9/Bcl3/Hlx/H2-DMb1/Anxa1	7
BP	GO:0021700	developmental maturation	14/574	355/28814	1.98	0.012456661	0.091856508	0.072285003	Rec8/Cdh5/Kdr/Bfsp2/Gata2/Myoc/Ccnb1/Mmp/Klf2/Sox18/Nppc/Rxfp2/Ccr6/Tal1	14
BP	GO:0002312	B cell activation involved in immune response	6/574	96/28814	3.14	0.012488309	0.091922141	0.072336652	Ptprc/Msg/Bcl3/Lilrb4a/6030468B19Rik/Ccr6	6
BP	GO:0007043	cell-cell junction assembly	8/574	155/28814	2.59	0.012733107	0.09348767	0.07356862	Pecam1/Cdh5/Lsr/Frmpd2/Grhl2/Cldn23/Cldn3/Agt	8
BP	GO:0002702	positive regulation of production of molecular mediator of immune response	7/574	125/28814	2.81	0.01276084	0.09348767	0.07356862	Tek/Il1r1/Ptprc/Nod2/Rsad2/6030468B19Rik/Trim6	7
BP	GO:1901214	regulation of neuron death	15/574	392/28814	1.92	0.012770402	0.09348767	0.07356862	Rras2/Nes/Lcn2/Kdr/Ccl5/Fcgr2b/Nupr1/Ngfr/Ccl3/Fas/Ccl12/Ager/Glp1r/Tfap2b/Agt	15
BP	GO:0002294	CD4-positive, alpha-beta T cell differentiation involved in immune response	5/574	70/28814	3.59	0.012907491	0.094320383	0.07422391	Lef1/Ly9/Bcl3/Hlx/Anxa1	5
BP	GO:0007620	copulation	3/574	25/28814	6.02	0.013057373	0.094560656	0.074412989	Avpr1a/Avp/Oxt	3
BP	GO:0010759	positive regulation of macrophage chemotaxis	3/574	25/28814	6.02	0.013057373	0.094560656	0.074412989	Ccl5/Rarres2/Cmklr1	3
BP	GO:0035024	negative regulation of Rho protein signal transduction	3/574	25/28814	6.02	0.013057373	0.094560656	0.074412989	Arap3/Rasip1/Myoc	3
BP	GO:0045063	T-helper 1 cell differentiation	3/574	25/28814	6.02	0.013057373	0.094560656	0.074412989	Lef1/Hlx/Anxa1	3
BP	GO:0051969	regulation of transmission of nerve impulse	3/574	25/28814	6.02	0.013057373	0.094560656	0.074412989	Avpr1a/Avp/Agt	3
BP	GO:0006858	extracellular transport	4/574	46/28814	4.37	0.013136876	0.094796633	0.074598686	Ak7/Spag17/Daw1/Dnah11	4
BP	GO:0048821	erythrocyte development	4/574	46/28814	4.37	0.013136876	0.094796633	0.074598686	Adgrf5/Klf2/Slc4a1/Tal1	4
BP	GO:0030522	intracellular receptor signaling pathway	10/574	220/28814	2.28	0.013246097	0.095414403	0.075084831	Flt1/Hspa1b/Irgm1/Zfp366/Nod2/Klf2/Srarp/Igtp/Tbx1/Ddx60	10
BP	GO:0002688	regulation of leukocyte chemotaxis	7/574	126/28814	2.79	0.01328717	0.095539954	0.075183631	Cxcl12/Ccl5/Nod2/Rarres2/Cmklr1/Ccr6/Ccl12	7
BP	GO:0016064	immunoglobulin mediated immune response	12/574	288/28814	2.09	0.013412301	0.096268397	0.075756867	C4b/Foxj1/Irf7/Ptprc/Nod2/Fcgr2b/Bcl3/6030468B19Rik/Csf2rb/Ccr6/Igha/H2-DMb1	12
BP	GO:0002293	alpha-beta T cell differentiation involved in immune response	5/574	71/28814	3.54	0.013664295	0.097385212	0.076635727	Lef1/Ly9/Bcl3/Hlx/Anxa1	5
BP	GO:0048146	positive regulation of fibroblast proliferation	5/574	71/28814	3.54	0.013664295	0.097385212	0.076635727	Fn1/Ccnb1/Ngfr/Ager/Agt	5
BP	GO:0050432	catecholamine secretion	5/574	71/28814	3.54	0.013664295	0.097385212	0.076635727	Cxcl12/Crh/Syt15/Oxt/Agt	5
BP	GO:2000134	negative regulation of G1/S transition of mitotic cell cycle	5/574	71/28814	3.54	0.013664295	0.097385212	0.076635727	Klf4/Muc1/Fhl4/E2f7/Ccl12	5
BP	GO:0070664	negative regulation of leukocyte proliferation	6/574	98/28814	3.07	0.013721117	0.097446454	0.076683921	Foxj1/Fcgr2b/Prdm1/Lilrb4a/Ccl12/Il4i1	6
BP	GO:1901222	regulation of NIK/NF-kappaB signaling	6/574	98/28814	3.07	0.013721117	0.097446454	0.076683921	Uaca/Tek/Nod2/Ifi35/Edar/Ager	6
BP	GO:0001910	regulation of leukocyte mediated cytotoxicity	7/574	127/28814	2.77	0.013828894	0.097526281	0.07674674	H2-D1/Ptprc/H2-Q4/Tap1/Il7r/H2-T10/Ager	7

MF	GO:0015291	secondary active transmembrane transporter activity	15/555	242/28275	3.16	9.74586E-05	0.00765618	0.006307683	Slc15a5/Slc22a6/Slc28a3/Slc6a20a/Slc6a13/Slc16a5/Slc37a2/Slc16a12/Slc4a11/Slc35d3/Slc9b2/Slc10a4/Slc26a5/Slc2a4/Slc26a10	15
MF	GO:0030021	extracellular matrix structural constituent conferring compression resistance	4/555	14/28275	14.56	0.000125699	0.00765618	0.006307683	Lum/Ogn/Dcn/Bgn	4
MF	GO:0022804	active transmembrane transporter activity	20/555	396/28275	2.57	0.000127403	0.00765618	0.006307683	Slc22a2/Atp6v1e2/Slc15a5/Slc22a6/Slc28a3/Abca4/Slc6a20a/Slc6a13/Slc16a5/Slc37a2/Slc16a12/Slc4a11/Abca6/Slc35d3/Slc9b2/Slc18a3/Slc10a4/Slc26a5/Slc2a4/Slc26a10	20
MF	GO:0030246	carbohydrate binding	16/555	277/28275	2.94	0.000131473	0.00765618	0.006307683	Klrd1/Cd209f/Prg4/Cd209a/Eva1c/Ppp1r3b/Loxl2/Mrc1/H2-Eb1/Colec12/Clec7a/Cd72/C4b/Clec12a/Galnt5/Clec1b	16
MF	GO:0005109	frizzled binding	6/555	41/28275	7.46	0.000139626	0.00765618	0.006307683	Myoc/Wnt9a/Rspo3/Sfrp1/Fzd1/Wnt8b	6
MF	GO:0005518	collagen binding	8/555	80/28275	5.09	0.000175854	0.008900941	0.007333202	Lum/Pcolce/Dcn/Ccbe1/Thbs1/Ddr2/Musk/Col6a4	8
MF	GO:0005178	integrin binding	11/555	155/28275	3.62	0.000256852	0.011835039	0.009750512	Itgb6/Itgb2l/Igf2/Tspan8/Thbs1/Fbln1/Dab2/Npnt/Ccn4/Itga5/Itgax	11
MF	GO:0004029	aldehyde dehydrogenase (NAD+) activity	4/555	17/28275	11.99	0.000285197	0.011835039	0.009750512	Aldh3b2/Aldh1a2/Aldh3a1/Aldh1a7	4
MF	GO:0001221	transcription coregulator binding	10/555	132/28275	3.86	0.000287782	0.011835039	0.009750512	Hnf1a/Twist1/Fos/Nr4a3/Trp73/Hmga1b/Esr1/Pparg/Tead2/Nkx2-1	10
MF	GO:0004030	aldehyde dehydrogenase [NAD(P)+] activity	4/555	19/28275	10.73	0.000450218	0.017426073	0.014356787	Aldh3b2/Aldh1a2/Aldh3a1/Aldh1a7	4
MF	GO:0019955	cytokine binding	10/555	143/28275	3.56	0.000544138	0.019891271	0.016387785	Ackr4/Crlf1/Hjv/Wfikkn2/Lepr/Tcap/Nog/Cd74/Trim16/Fzd4	10
MF	GO:0008395	steroid hydroxylase activity	7/555	73/28275	4.89	0.000575306	0.019923766	0.016414556	Ch25h/Cyp1b1/Cyp2a5/Cyp2e1/Cyp2c23/Cyp2g1/Cyp2j8	7
MF	GO:0016620	oxidoreductase activity, acting on the aldehyde or oxo group of donors, NAD or NADP as acceptor	6/555	56/28275	5.46	0.000786717	0.02540968	0.020934227	Aldh3b2/Aldh1a2/Aldh3a1/Aldh1a7/Akr1c14/Akr1b8	6
MF	GO:0046965	nuclear retinoid X receptor binding	4/555	22/28275	9.26	0.000810947	0.02540968	0.020934227	Hmga1b/Pparg/Rarb/Vdr	4
MF	GO:0019838	growth factor binding	10/555	153/28275	3.33	0.000919101	0.027489462	0.022647693	Igfbp2/Wfikkn2/Fgfbp1/Thbs1/Col1a2/Trim16/Nkd2/Ccn4/Col2a1/Ntrk1	10
MF	GO:0023023	MHC protein complex binding	4/555	23/28275	8.86	0.000966546	0.027651621	0.022781291	Klrd1/H2-Aa/H2-Eb1/Cd74	4
MF	GO:0008330	protein tyrosine/threonine phosphatase activity	3/555	11/28275	13.89	0.0011035	0.030254299	0.024925555	Dusp9/Dusp4/Dusp5	3
MF	GO:0050839	cell adhesion molecule binding	15/555	307/28275	2.49	0.001197023	0.030372302	0.025022773	Itgb6/Cdh3/Itgb2l/Igf2/Tspan8/Cdh1/Thbs1/Fbln1/Dab2/Npnt/Ccn4/Tbc1d2/Itga5/Cdh15/Itgax	15
MF	GO:0015297	antiporter activity	7/555	83/28275	4.3	0.001239607	0.030372302	0.025022773	Slc22a6/Slc37a2/Slc4a11/Slc35d3/Slc9b2/Slc26a5/Slc26a10	7
MF	GO:0042277	peptide binding	17/555	374/28275	2.32	0.001281552	0.030372302	0.025022773	H2-Q1/Itgb2l/Lepr/Npr3/H2-Aa/Ptges/Gstm2/H2-Eb1/Cd74/Npr1/Pparg/Fzd4/Nfkbia/Oprk1/Nod2/Avpr2/Prnp	17
MF	GO:0015301	anion:anion antiporter activity	4/555	25/28275	8.15	0.001338597	0.030372302	0.025022773	Slc22a6/Slc4a11/Slc26a5/Slc26a10	4
MF	GO:0140323	solute:anion antiporter activity	4/555	25/28275	8.15	0.001338597	0.030372302	0.025022773	Slc22a6/Slc4a11/Slc26a5/Slc26a10	4
MF	GO:0005044	scavenger receptor activity	5/555	43/28275	5.92	0.001488562	0.032649124	0.026898575	Ackr4/Prg4/Scara5/Loxl2/Colec12	5
MF	GO:0017017	MAP kinase tyrosine/serine/threonine phosphatase activity	3/555	13/28275	11.76	0.001857584	0.037039089	0.030515327	Dusp9/Dusp4/Dusp5	3
MF	GO:0023026	MHC class II protein complex binding	3/555	13/28275	11.76	0.001857584	0.037039089	0.030515327	H2-Aa/H2-Eb1/Cd74	3
MF	GO:0035256	G protein-coupled glutamate receptor binding	3/555	13/28275	11.76	0.001857584	0.037039089	0.030515327	Esr1/Homer2/Prnp	3
MF	GO:0016903	oxidoreductase activity, acting on the aldehyde or oxo group of donors	6/555	67/28275	4.56	0.002017693	0.037675293	0.031039475	Aldh3b2/Aldh1a2/Aldh3a1/Aldh1a7/Akr1c14/Akr1b8	6
MF	GO:0008201	heparin binding	10/555	170/28275	3	0.002026147	0.037675293	0.031039475	Pf4/Col13a1/Pcolce/Eva1c/Thbs1/Rspo3/Sfrp1/Ccn4/Col11a1/Impg1	10
MF	GO:0030247	polysaccharide binding	4/555	28/28275	7.28	0.002068265	0.037675293	0.031039475	Prg4/Ppp1r3b/H2-Eb1/Clec7a	4
MF	GO:0015293	symporter activity	9/555	143/28275	3.21	0.00211852	0.037675293	0.031039475	Slc15a5/Slc28a3/Slc6a20a/Slc6a13/Slc16a5/Slc16a12/Slc4a11/Slc10a4/Slc2a4	9
MF	GO:0008392	arachidonic acid epoxygenase activity	5/555	48/28275	5.31	0.002443916	0.042318343	0.034864736	Cyp2a5/Cyp2e1/Cyp2c23/Cyp2g1/Cyp2j8	5
MF	GO:0022853	active ion transmembrane transporter activity	12/555	238/28275	2.57	0.002773049	0.046786311	0.038545753	Atp6v1e2/Slc22a6/Slc28a3/Slc6a20a/Slc6a13/Slc37a2/Slc4a11/Slc9b2/Slc10a4/Slc26a5/Slc2a4/Slc26a10	12
MF	GO:0008391	arachidonic acid monooxygenase activity	5/555	51/28275	4.99	0.003195609	0.051286466	0.042253287	Cyp2a5/Cyp2e1/Cyp2c23/Cyp2g1/Cyp2j8	5
MF	GO:0004497	monooxygenase activity	9/555	152/28275	3.02	0.003195661	0.051286466	0.042253287	Ch25h/Cmah/Cyp1b1/Akr1c14/Cyp2a5/Cyp2e1/Cyp2c23/Cyp2g1/Cyp2j8	9
MF	GO:0008514	organic anion transmembrane transporter activity	10/555	185/28275	2.75	0.003727117	0.054733356	0.04509307	Slc22a2/Slc22a6/Slc6a20a/Slc6a13/Slc37a2/Slc16a12/Slc4a11/Slc35d3/Slc26a5/Slc26a10	10
MF	GO:0004879	nuclear receptor activity	5/555	53/28275	4.81	0.003781072	0.054733356	0.04509307	Nr4a3/Esr1/Pparg/Rarb/Vdr	5
MF	GO:0098531	ligand-activated transcription factor activity	5/555	53/28275	4.81	0.003781072	0.054733356	0.04509307	Nr4a3/Esr1/Pparg/Rarb/Vdr	5
MF	GO:0016712	oxidoreductase activity, acting on paired donors, with incorporation or reduction of molecular oxygen, reduced flavin or flavoprotein as one donor, and incorporation of one atom of oxygen	6/555	76/28275	4.02	0.003813937	0.054733356	0.04509307	Cyp1b1/Cyp2a5/Cyp2e1/Cyp2c23/Cyp2g1/Cyp2j8	6
MF	GO:0042974	nuclear retinoic acid receptor binding	4/555	33/28275	6.18	0.003826344	0.054733356	0.04509307	Hmga1b/Pparg/Rarb/Vdr	4
MF	GO:0033218	amide binding	18/555	452/28275	2.03	0.003911087	0.054755213	0.045111078	Folr2/H2-Q1/Itgb2l/Lepr/Npr3/H2-Aa/Ptges/Gstm2/H2-Eb1/Cd74/Npr1/Pparg/Fzd4/Nfkbia/Oprk1/Nod2/Avpr2/Prnp	18
MF	GO:0001758	retinal dehydrogenase activity	3/555	18/28275	8.49	0.004927419	0.066168203	0.054513877	Aldh1a2/Akr1c14/Akr1b8	3
MF	GO:0033549	MAP kinase phosphatase activity	3/555	18/28275	8.49	0.004927419	0.066168203	0.054513877	Dusp9/Dusp4/Dusp5	3
MF	GO:0016705	oxidoreductase activity, acting on paired donors, with incorporation or reduction of molecular oxygen	11/555	227/28275	2.47	0.005487226	0.070540393	0.058115985	Fads2b/Ch25h/Cmah/Cyp1b1/Akr1c14/Degs2/Cyp2a5/Cyp2e1/Cyp2c23/Cyp2g1/Cyp2j8	11
MF	GO:0019842	vitamin binding	8/555	136/28275	3	0.005522189	0.070540393	0.058115985	Folr2/Spcs3/Abca4/Aldh1a2/Cubn/Gad2/Bspry/Vdr	8
MF	GO:0050840	extracellular matrix binding	5/555	58/28275	4.39	0.005574621	0.070540393	0.058115985	Dcn/Thbs1/Olfml2a/Bgn/Dmp1	5
MF	GO:0070330	aromatase activity	4/555	37/28275	5.51	0.005806942	0.070758661	0.058295809	Cyp1b1/Cyp2e1/Cyp2c23/Cyp2j8	4
MF	GO:0071855	neuropeptide receptor binding	4/555	37/28275	5.51	0.005806942	0.070758661	0.058295809	Pomc/Crh/Edn3/Edn1	4
MF	GO:0015106	bicarbonate transmembrane transporter activity	3/555	21/28275	7.28	0.007688954	0.090345212	0.074432545	Slc4a11/Slc26a5/Slc26a10	3
MF	GO:0072349	modified amino acid transmembrane transporter activity	3/555	21/28275	7.28	0.007688954	0.090345212	0.074432545	Slc6a20a/Slc6a13/Slc16a12	3

BP	GO:0043123	positive regulation of I-kappaB kinase/NF-kappaB signaling	7/574	127/28814	2.77	0.013828894	0.097526281	0.07674674	Nod2/Trim30a/Tnfsf10/Edar/Unc5cl/Trim6/Eda2r	7
BP	GO:0043280	positive regulation of cysteine-type endopeptidase activity involved in apoptotic process	7/574	127/28814	2.77	0.013828894	0.097526281	0.07674674	Acer2/Uaca/Ngfr/Tnfsf10/Sox7/Fas/S100a9	7
BP	GO:0051101	regulation of DNA binding	7/574	127/28814	2.77	0.013828894	0.097526281	0.07674674	Hjurp/Klf4/Per2/Lef1/Ager/Wfikkn2/Trim6	7
BP	GO:0045637	regulation of myeloid cell differentiation	10/574	222/28814	2.26	0.014034101	0.098801051	0.077749899	Hspa1b/Lef1/Gata2/Ccl5/H4c17/Rab7b/Ccl3/Lilrb4a/Fas/Tal1	10
BP	GO:0014706	striated muscle tissue development	12/574	290/28814	2.08	0.014094129	0.099051087	0.077946661	Eng/Myh11/Hspg2/Ccm2l/Lef1/Dsp/Ccnb1/Tcap/Dll4/Mylk3/Col14a1/Agt	12
BP	GO:1903524	positive regulation of blood circulation	4/574	47/28814	4.27	0.014138243	0.099188609	0.078054881	Adm/Gch1/Avpr1a/Glp1r	4
CC	GO:0062023	collagen-containing extracellular matrix	29/574	396/28739	3.67	2.39791E-09	8.39268E-07	7.5471E-07	Fn1/Lama5/Impg1/Lama4/Igfbp7/Vwf/Hspg2/Fbn2/Tinagl1/Egflam/Nid2/Myoc/Fmod/Mmrn2/Muc1/Hmcn1/Rarres2/Clec14a/Col9a1/Impg2/Col6a4/Zp1/Kazald1/Anxa9/Angpt2/Gpc2/Tnxb/Col	29
CC	GO:0043230	extracellular organelle	11/574	125/28739	4.41	4.36579E-05	0.005093426	0.004580254	Fn1/Ahnak/Prom1/Podxl/Gbp3/Gbp9/Icam1/Muc1/Gbp2/Gbp6/Anxa1	11
CC	GO:0065010	extracellular membrane-bounded organelle	11/574	125/28739	4.41	4.36579E-05	0.005093426	0.004580254	Fn1/Ahnak/Prom1/Podxl/Gbp3/Gbp9/Icam1/Muc1/Gbp2/Gbp6/Anxa1	11
CC	GO:0036156	inner dynein arm	4/574	12/28739	16.69	6.86211E-05	0.006004347	0.005399398	Dnah10/Casc1/Dnah12/Dnah3	4
CC	GO:0043235	receptor complex	21/574	423/28739	2.49	0.0001409	0.008876193	0.0079819	Flt1/Ptprb/Osmr/Eng/Tek/Kdr/Tie1/Flt4/Chrna2/Il2rg/Gabrr2/Il12rb2/Fgfr4/Tlr1/Impg2/Csf2rb/Gabrq/Calcr/Ticam2/Traf1/Htr2b	21
CC	GO:0030286	dynein complex	7/574	58/28739	6.04	0.000152163	0.008876193	0.0079819	Dnah10/Dynlt1b/Casc1/Dnah12/Dnah3/Dnali1/Dnah11	7
CC	GO:0031514	motile cilium	15/574	258/28739	2.91	0.000237383	0.011869164	0.010673323	Ctsh/Ak7/Spag17/Dnali1/Oxct2b/Ttc29/Ccr6/Rho/Tacr2/Iqca/Il4i1/Enkur/Fbxl13/Dnah11/Anxa1	15
CC	GO:0005604	basement membrane	9/574	119/28739	3.79	0.000656993	0.028743452	0.025847495	Fn1/Lama5/Lama4/Hspg2/Egflam/Nid2/Mmrn2/Hmcn1/Col9a1	9
CC	GO:0005858	axonemal dynein complex	4/574	27/28739	7.42	0.001918512	0.062457211	0.05616453	Dnah10/Casc1/Dnah12/Dnah3	4
CC	GO:0042622	photoreceptor outer segment membrane	3/574	13/28739	11.55	0.00195228	0.062457211	0.05616453	Prom1/Rho/Prph	3
CC	GO:0005930	axoneme	9/574	139/28739	3.24	0.001962941	0.062457211	0.05616453	Ctsh/Spag17/Dnah10/Hydin/Casc1/Dnah12/Dnah3/Dnali1/Dnah11	9
CC	GO:0097014	ciliary plasm	9/574	141/28739	3.2	0.002164131	0.063120493	0.056760985	Ctsh/Spag17/Dnah10/Hydin/Casc1/Dnah12/Dnah3/Dnali1/Dnah11	9
MF	GO:0005201	extracellular matrix structural constituent	17/565	150/28275	5.67	9.44727E-09	5.78173E-06	5.00208E-06	Fn1/Lama5/Lama4/Igfbp7/Vwf/Hspg2/Fbn2/Tinagl1/Nid2/Mmrn2/Hmcn1/Col9a1/Impg2/Col6a4/Zp1/Tnxb/Col14a1	17
MF	GO:0019838	growth factor binding	14/565	153/28275	4.58	2.5993E-06	0.000795387	0.000688132	Flt1/Osmr/Eng/Tek/Kdr/Il1r1/Igfbp7/Flt4/Il2rg/Ngfr/Trim16/Fgfr4/Kazald1/Wfikkn2	14
MF	GO:0005539	glycosaminoglycan binding	17/565	233/28275	3.65	5.17607E-06	0.000969059	0.000838384	Rpl29/Fn1/Eng/Impg1/Ptprc/Rspo1/Egflam/Ccl5/Nod2/Lpl/Pglyrp1/Liph/Layn/Fgfr4/Impg2/Tnxb/Ager	17
MF	GO:0019955	cytokine binding	13/565	143/28275	4.55	6.33372E-06	0.000969059	0.000838384	Osmr/Eng/Ackr4/Il1r1/Il2rg/Tcap/Trim16/Il12rb2/Gbp2/Ccr6/Cxcr4/Ager/Wfikkn2	13
MF	GO:0008009	chemokine activity	7/565	41/28275	8.54	1.52864E-05	0.001871055	0.001618749	Cxcl12/Ccl6/Ccl5/Ccl9/Ccl4/Ccl3/Ccl12	7
MF	GO:0140375	immune receptor activity	12/565	136/28275	4.42	1.93512E-05	0.001973827	0.001707663	Osmr/Ackr4/Ctsh/Il1r1/Il2rg/Fcgr2b/Il7r/Il12rb2/Cmklr1/Csf2rb/Ccr6/Cxcr4	12
MF	GO:0001664	G protein-coupled receptor binding	19/565	320/28275	2.97	2.78319E-05	0.0024333	0.002105177	Nes/Cxcl12/Hspa1b/Adm/Rspo1/Ccl6/Ccl5/Crh/Myoc/Wnt6/Ccl9/Ccl4/Npb/Ccl3/Avpr1a/Ccl12/Avp/Oxt/Agt	19
MF	GO:0005126	cytokine receptor binding	18/565	316/28275	2.85	7.65694E-05	0.005215625	0.004512314	Osmr/Eng/Nes/Cdh5/Cxcl12/Ccl6/Ccl5/Ccl9/Ccl4/Ngfr/Tnfsf10/Lrg1/Ccl3/Angpt2/Ccl12/Ticam2/Tnfsf9/Traf1	18
MF	GO:0030547	signaling receptor inhibitor activity	6/565	37/28275	8.12	8.51054E-05	0.005215625	0.004512314	Ly6c1/Ly6a/Ccl5/Lilrb4a/Angpt2/Wfikkn2	6
MF	GO:0017046	peptide hormone binding	7/565	53/28275	6.61	8.52226E-05	0.005215625	0.004512314	Sctr/Prlhr/Cmklr1/Avpr1a/Rxfp2/Calcr/Glp1r	7
MF	GO:0048020	CCR chemokine receptor binding	7/565	56/28275	6.26	0.000121774	0.006775045	0.005861451	Nes/Ccl6/Ccl5/Ccl9/Ccl4/Ccl3/Ccl12	7
MF	GO:0004896	cytokine receptor activity	9/565	97/28275	4.64	0.000143204	0.007146685	0.006182977	Osmr/Ackr4/Il1r1/Il2rg/Il7r/Il12rb2/Csf2rb/Ccr6/Cxcr4	9
MF	GO:0042379	chemokine receptor binding	8/565	77/28275	5.2	0.000151809	0.007146685	0.006182977	Nes/Cxcl12/Ccl6/Ccl5/Ccl9/Ccl4/Ccl3/Ccl12	8
MF	GO:0008201	heparin binding	12/565	170/28275	3.53	0.000169302	0.007246227	0.006269096	Rpl29/Fn1/Impg1/Ptprc/Rspo1/Ccl5/Lpl/Liph/Fgfr4/Impg2/Tnxb/Ager	12
MF	GO:0008528	G protein-coupled peptide receptor activity	11/565	146/28275	3.77	0.000177604	0.007246227	0.006269096	Ackr4/Sctr/Prlhr/Avpr1a/Rxfp2/Ccr6/Brs3/Tacr2/Cxcr4/Calcr/Glp1r	11
MF	GO:0001653	peptide receptor activity	11/565	153/28275	3.6	0.000267203	0.010220514	0.008842309	Ackr4/Sctr/Prlhr/Avpr1a/Rxfp2/Ccr6/Brs3/Tacr2/Cxcr4/Calcr/Glp1r	11
MF	GO:0042562	hormone binding	8/565	87/28275	4.6	0.000354294	0.012754586	0.011034669	Ctsh/Sctr/Prlhr/Cmklr1/Avpr1a/Rxfp2/Calcr/Glp1r	8
MF	GO:0008569	minus-end-directed microtubule motor activity	4/565	20/28275	10.01	0.000592519	0.019181886	0.016595267	Dnah10/Dnah12/Dnah3/Dnah11	4
MF	GO:0042277	peptide binding	18/565	374/28275	2.41	0.000595516	0.019181886	0.016595267	H2-D1/Sctr/Gstm6/Nod2/H2-Q4/Tap1/Fcgr2b/Ngfr/Enpep/Prlhr/Tlr1/Cmklr1/Avpr1a/H2-T10/Rxfp2/Calcr/Ager/Glp1	18
MF	GO:0005125	cytokine activity	13/565	228/28275	2.85	0.000728494	0.022291907	0.019285912	Cxcl12/Ccl6/Ccl5/Pglyrp1/Wnt6/Ccl9/Cmtm7/Ccl4/Tnfsf10/Ccl3/6030468B19Rik/Ccl12/Tnfsf9	13
MF	GO:0019199	transmembrane receptor protein kinase activity	7/565	76/28275	4.61	0.000814757	0.023744346	0.020542494	Flt1/Eng/Tek/Kdr/Tie1/Flt4/Fgfr4	7
MF	GO:0061134	peptidase regulator activity	14/565	266/28275	2.63	0.00100942	0.027679842	0.0239473	Fn1/Ctsh/Serpinb1b/Cst7/Wfdc1/Acrbp/Bst2/Rarres1/Spink4/Avp/Wfikkn2/Serpinb5/Agt/Slpi	14
MF	GO:0004714	transmembrane receptor protein tyrosine kinase activity	6/565	58/28275	5.18	0.001040255	0.027679842	0.0239473	Flt1/Tek/Kdr/Tie1/Flt4/Fgfr4	6
MF	GO:0045505	dynein intermediate chain binding	5/565	40/28275	6.26	0.001155911	0.028442191	0.024606849	Dnah10/Dynlt1b/Dnah12/Dnah3/Dnah11	5
MF	GO:0005035	death receptor activity	3/565	11/28275	13.65	0.001161854	0.028442191	0.024606849	Ngfr/Fas/Eda2r	3
MF	GO:0042826	histone deacetylase binding	9/565	133/28275	3.39	0.001451248	0.034160136	0.029553747	Klf4/Top2a/Hspa1b/Per2/Lef1/Hic1/Prdm1/Bcl3/Tal1	9
MF	GO:0050700	CARD domain binding	3/565	13/28275	11.55	0.001954779	0.044308329	0.038333487	Irgm1/Nod2/Igtp	3
MF	GO:0140359	ABC-type transporter activity	4/565	28/28275	7.15	0.002206797	0.048234267	0.041730025	Abcb1a/Abcc6/Tap1/Abcg3	4
MF	GO:0008191	metalloendopeptidase inhibitor activity	3/565	14/28275	10.72	0.002451179	0.050596472	0.043773694	Bst2/Rarres1/Wfikkn2	3
MF	GO:0038187	pattern recognition receptor activity	4/565	29/28275	6.9	0.0025199	0.050596472	0.043773694	Nod2/Pglyrp1/Trim30a/Clec7a	4
MF	GO:0045296	cadherin binding	6/565	69/28275	4.35	0.002562893	0.050596472	0.043773694	Ptprb/Prom1/Cdh5/Kdr/Fxyd5/Olfm4	6
MF	GO:0051959	dynein light intermediate chain binding	4/565	31/28275	6.46	0.003234969	0.061868786	0.053525971	Dnah10/Dnah12/Dnah3/Dnah11	4
MF	GO:0070566	adenylyltransferase activity	4/565	32/28275	6.26	0.003639485	0.064010177	0.055378601	Oasl2/Oas1b/Fhit/Oas2	4
MF	GO:0004698	calcium-dependent protein kinase C activity	3/565	16/28275	9.38	0.003660713	0.064010177	0.055378601	Prkd2/Ccl3/Pkn3	3
MF	GO:0048019	receptor antagonist activity	3/565	16/28275	9.38	0.003660713	0.064010177	0.055378601	Ccl5/Angpt2/Wfikkn2	3
MF	GO:0050839	cell adhesion molecule binding	14/565	307/28275	2.28	0.003771277	0.064111709	0.055466443	Ptprb/Fn1/Prom1/Cdh5/Cxcl12/Kdr/Fxyd5/Lama5/Vwf/Icam1/Dsp/Icam2/Lilrb4a/Olfm4	14
MF	GO:0004866	endopeptidase inhibitor activity	11/565	216/28275	2.55	0.004332633	0.067010063	0.057973962	Serpinb1b/Cst7/Wfdc1/Bst2/Rarres1/Spink4/Avp/Wfikkn2/Serpinb5/Agt/Slpi	11
MF	GO:0004697	protein kinase C activity	3/565	17/28275	8.83	0.004379743	0.067010063	0.057973962	Prkd2/Ccl3/Pkn3	3
MF	GO:0008559	ABC-type xenobiotic transporter activity	3/565	17/28275	8.83	0.004379743	0.067010063	0.057973962	Abcb1a/Abcc6/Abcg3	3
MF	GO:0016004	phospholipase activator activity	3/565	17/28275	8.83	0.004379743	0.067010063	0.057973962	Ccl5/Ccl3/Ccl12	3
MF	GO:0033218	amide binding	18/565	452/28275	1.99	0.004693544	0.070059728	0.06061239	H2-D1/Sctr/Gstm6/Nod2/H2-Q4/Tap1/Fcgr2b/Ngfr/Enpep/Prlhr/Tlr1/Cmklr1/Avpr1a/H2-T10/Rxfp2/Calcr/Ager/Glp1	18
MF	GO:1901681	sulfur compound binding	13/565	284/28275	2.29	0.004988499	0.07268955	0.062887588	Rpl29/Fn1/Impg1/Ptprc/Rspo1/Gstm6/Ccl5/Lpl/Liph/Fgfr4/Impg2/Tnxb/Ager	13
MF	GO:0003774	cytoskeletal motor activity	7/565	106/28275	3.3	0.005453809	0.077621654	0.067154613	Myh11/Myo5c/Kif19a/Dnah10/Dnah12/Dnah3/Dnah11	7
MF	GO:0030414	peptidase inhibitor activity	11/565	224/28275	2.46	0.00566755	0.078830462	0.068200417	Serpinb1b/Cst7/Wfdc1/Bst2/Rarres1/Spink4/Avp/Wfikkn2/Serpinb5/Agt/Slpi	11
MF	GO:0060229	lipase activator activity	3/565	19/28275	7.9	0.006059096	0.082403707	0.071291821	Ccl5/Ccl3/Ccl12	3
MF	GO:0003924	GTPase activity	13/565	294/28275	2.21	0.006611065	0.087955911	0.076095327	Rras2/Gbp3/Gbp9/Gbp5/Gbp4/Irgm1/Tuba1c/Gch1/Rab7b/Igtp/Gbp2/Gm4951/Gbp6	13
MF	GO:0061135	endopeptidase regulator activity	11/565	232/28275	2.37	0.007307235	0.095149523	0.082318902	Serpinb1b/Cst7/Wfdc1/Bst2/Rarres1/Spink4/Avp/Wfikkn2/Serpinb5/Agt/Slpi	11

This table lists significantly enriched Gene Ontology (GO) pathways. "MPTP" refers to the acute MPTP model, "A53T" to the 16 months A53T-α-synuclein model, and "MitoPark" to the 5-month MitoPark model.
