# Supplementary material for: Neurotoxicity, α-synuclein pathology, and mitochondrial dysfunction: A comparative study of different mouse models of Parkinson’s disease
Source: Neural Regen Res. 2025 Sep 29;21(10):5000–12. doi: 10.4103/NRR.NRR-D-25-00648 (PMC13568611; doi:10.4103/NRR.NRR-D-25-00648)
Supplement: Supplementary file 4 [file NRR-21-5000_Suppl3.pdf]

Additional Table 5. KEGG pathway identified from RNA-Seq data analysis of the striatum in three PD mouse models

MPTP

ID	Term	Ontology Source	Term PValue	Term PValue Corrected with Benjamini-Hochberg	Group PValue	Group PValue Corrected with Benjamini-Hochberg	GOLevels	GOGroups	% Associated Genes	Nr. Genes	Rich Factor	Associated Genes Found
KEGG:04080	Neuroactive ligand-receptor interaction	KEGG_25.05.2022	0.000422866	0.012093973	0.000422866	0.001127643	[-1]	Group1	6.994818687	27	3.055052666	[Adcyap1r1, Adrb2, C3ar1, Cckar, Chrm5, Crh, Ednra, Gabre, Gabrg1, Glra3, Gria4, Grin2d, Grm2, Hcrtr2, Htr1a, Lpar1, Mc4r, Nts, P2ry1, P2ry6, Pnoc, Ptafr, Rxfp1, Tac2, Trh, Trhr, Tspo]
KEGG:05150	Staphylococcus aureus infection	KEGG_25.05.2022	0.001340977	0.017432697	0.001340977	0.002145563	[-1]	Group2	9.375	12	4.158266129	[C1qb, C1qc, C3ar1, C4b, Fcgr1, Fcgr2b, Fcgr3, H2-DMa, H2-Oa, Icam1, Ptafr, Selplg]
KEGG:04360	Axon guidance	KEGG_25.05.2022	0.001264204	0.018078115	0.001264204	0.002528408	[-1]	Group3	8.28729248	15	3.675815363	[Abl1, Efna1, Ephb2, Fes, Fyn, Ntn3, Plcg2, Plxnb2, Plxnc1, Ptch1, Rac2, Rgma, Sema6c, Trpc5, Unc5d]
KEGG:04610	Complement and coagulation cascades	KEGG_25.05.2022	0.004242008	0.037912942	0.004242008	0.00565601	[-1]	Group4	9.677419662	9	4.246739876	[A2m, C1qb, C1qc, C3ar1, C4b, F5, Pros1, Serping1, Thbd]
KEGG:04640	Hematopoietic cell lineage	KEGG_25.05.2022	0.004555026	0.038315807	0.004555026	0.005205744	[-1]	Group5	9.574467659	9	4.246739876	[Cd14, Cd9, Csf1r, Csf3r, Fcgr1, H2-DMa, H2-Oa, Siglech, Thpo]
KEGG:04145	Phagosome	KEGG_25.05.2022	0.000148544	0.007080609	5.02479E-06	2.00992E-05	[-1]	Group6	9.340659142	17	4.120394853	[Atp6v1a, Cd14, Cyba, Fcgr1, Fcgr2b, Fcgr3, H2-D1, H2-DMa, H2-K1, H2-Oa, H2-Q4, H2-T23, H2-T24, Ncf2, Tcirg1, Thbs4, Tlr2]
KEGG:04380	Osteoclast differentiation	KEGG_25.05.2022	0.000396984	0.01419218	5.02479E-06	2.00992E-05	[-1]	Group6	10.15625	13	4.239800759	[Btk, Csf1r, Ctsk, Cyba, Fcgr1, Fcgr2b, Fcgr3, Fyn, Ncf2, Plcg2, Tgfb1, Tnfrsf1a, Trem2]
KEGG:04672	Intestinal immune network for IgA production	KEGG_25.05.2022	0.003104669	0.031711977	5.02479E-06	2.00992E-05	[-1]	Group6	13.95348835	6	6.189047262	[Cd86, H2-DMa, H2-Oa, Icosl, Tgfb1, Tnfrsf13b]
KEGG:05140	Leishmaniasis	KEGG_25.05.2022	0.002461253	0.029329928	5.02479E-06	2.00992E-05	[-1]	Group6	11.4285717	8	5.069124424	[Cyba, Fcgr1, Fcgr3, H2-DMa, H2-Oa, Ncf2, Tgfb1, Tlr2]
KEGG:05145	Toxoplasmosis	KEGG_25.05.2022	0.004142337	0.039490286	5.02479E-06	2.00992E-05	[-1]	Group6	9.090909004	10	4.069251258	[H2-DMa, H2-Oa, Igtp, Il10ra, Il10rb, Pik3cg, Tgfb1, Tlr2, Tnfrsf1a, Xiap]
KEGG:05152	Tuberculosis	KEGG_25.05.2022	0.001195325	0.021366438	5.02479E-06	2.00992E-05	[-1]	Group6	8.333333015	15	3.696236559	[Cd14, Fcer1g, Fcgr1, Fcgr2b, Fcgr3, H2-DMa, H2-Oa, Il10ra, Il10rb, Lbp, Tcirg1, Tgfb1, Tlr2, Tlr9, Tnfrsf1a]
KEGG:05323	Rheumatoid arthritis	KEGG_25.05.2022	0.002699959	0.029699551	5.02479E-06	2.00992E-05	[-1]	Group6	10.34482765	9	4.588431591	[Atp6v1a, Cd86, Ctsk, H2-DMa, H2-Oa, Icam1, Tcirg1, Tgfb1, Tlr2]
KEGG:04145	Phagosome	KEGG_25.05.2022	0.000148544	0.007080609	8.16331E-08	6.53065E-07	[-1]	Group7	9.340659142	17	4.120394853	[Atp6v1a, Cd14, Cyba, Fcgr1, Fcgr2b, Fcgr3, H2-D1, H2-DMa, H2-K1, H2-Oa, H2-Q4, H2-T23, H2-T24, Ncf2, Tcirg1, Thbs4, Tlr2]
KEGG:04612	Antigen processing and presentation	KEGG_25.05.2022	0.000916696	0.02184793	8.16331E-08	6.53065E-07	[-1]	Group7	11.11111069	10	5.098257323	[B2m, H2-D1, H2-DMa, H2-K1, H2-Oa, H2-Q4, H2-T23, H2-T24, Lgmn, Psme2]
KEGG:04672	Intestinal immune network for IgA production	KEGG_25.05.2022	0.003104669	0.031711977	8.16331E-08	6.53065E-07	[-1]	Group7	13.95348835	6	6.189047262	[Cd86, H2-DMa, H2-Oa, Icosl, Tgfb1, Tnfrsf13b]
KEGG:04940	Type I diabetes mellitus	KEGG_25.05.2022	0.002461253	0.029329928	8.16331E-08	6.53065E-07	[-1]	Group7	11.4285717	8	5.296100144	[Cd86, H2-D1, H2-DMa, H2-K1, H2-Oa, H2-Q4, H2-T23, H2-T24]
KEGG:05140	Leishmaniasis	KEGG_25.05.2022	0.002461253	0.029329928	8.16331E-08	6.53065E-07	[-1]	Group7	11.4285717	8	5.069124424	[Cyba, Fcgr1, Fcgr3, H2-DMa, H2-Oa, Ncf2, Tgfb1, Tlr2]
KEGG:05152	Tuberculosis	KEGG_25.05.2022	0.001195325	0.021366438	8.16331E-08	6.53065E-07	[-1]	Group7	8.333333015	15	3.696236559	[Cd14, Fcer1g, Fcgr1, Fcgr2b, Fcgr3, H2-DMa, H2-Oa, Il10ra, Il10rb, Lbp, Tcirg1, Tgfb1, Tlr2, Tlr9, Tnfrsf1a]
KEGG:05166	Human T-cell leukemia virus 1 infection	KEGG_25.05.2022	0.001017175	0.020779438	8.16331E-08	6.53065E-07	[-1]	Group7	7.599999905	19	3.41191067	[Adcy7, Atf2, B2m, Ccna2, Ccnb2, Ccne1, Cdkn1a, H2-D1, H2-DMa, H2-K1, H2-Oa, H2-Q4, H2-T23, H2-T24, Icam1, Tgfb1, Tnfrsf1a, Tspo, Xiap]
KEGG:05169	Epstein-Barr virus infection	KEGG_25.05.2022	0.000113239	0.008096562	8.16331E-08	6.53065E-07	[-1]	Group7	8.658008575	20	3.890775325	[B2m, Btk, Ccna2, Ccne1, Cdkn1a, Cdkn1b, Ddx58, Eif2ak2, H2-D1, H2-DMa, H2-K1, H2-Oa, H2-Q4, H2-T23, H2-T24, Icam1, Isg15, Plcg2, Tlr2, Vim]
KEGG:05320	Autoimmune thyroid disease	KEGG_25.05.2022	0.005230225	0.039364323	8.16331E-08	6.53065E-07	[-1]	Group7	10.12658215	8	4.66893039	[Cd86, H2-D1, H2-DMa, H2-K1, H2-Oa, H2-Q4, H2-T23, H2-T24]
KEGG:05323	Rheumatoid arthritis	KEGG_25.05.2022	0.002699959	0.029699551	8.16331E-08	6.53065E-07	[-1]	Group7	10.34482765	9	4.588431591	[Atp6v1a, Cd86, Ctsk, H2-DMa, H2-Oa, Icam1, Tcirg1, Tgfb1, Tlr2]
KEGG:05330	Allograft rejection	KEGG_25.05.2022	0.001239552	0.019695107	8.16331E-08	6.53065E-07	[-1]	Group7	12.6984129	8	5.913978495	[Cd86, H2-D1, H2-DMa, H2-K1, H2-Oa, H2-Q4, H2-T23, H2-T24]
KEGG:05332	Graft-versus-host disease	KEGG_25.05.2022	0.001239552	0.019695107	8.16331E-08	6.53065E-07	[-1]	Group7	12.6984129	8	5.913978495	[Cd86, H2-D1, H2-DMa, H2-K1, H2-Oa, H2-Q4, H2-T23, H2-T24]
KEGG:05416	Viral myocarditis	KEGG_25.05.2022	7.61542E-06	0.001089004	8.16331E-08	6.53065E-07	[-1]	Group7	14.77272701	13	6.134179822	[Abl1, Cd86, Dag1, Fyn, H2-D1, H2-DMa, H2-K1, H2-Oa, H2-Q4, H2-T23, H2-T24, Icam1, Rac2]

A53T

ID	Term	Ontology Source	Term PValue	Term PValue Corrected with Benjamini-Hochberg	Group PValue	Group PValue Corrected with Benjamini-Hochberg	GOLevels	GOGroups	% Associated Genes	Nr. Genes	Rich Factor	Associated Genes Found
KEGG:00590	Arachidonic acid metabolism	KEGG_25.05.2022	0.005571099	0.035283629	0.005571099	0.006963874	[-1]	Group01	8.13953495	7	3.48858282	[Alox12b, Cyp2c23, Cyp2e1, Cyp2j8, Gpx8, Ptgds, Ptges]
KEGG:05133	Pertussis	KEGG_25.05.2022	0.003010122	0.025896506	0.003010122	0.00501687	[-1]	Group02	9.090909004	7	4.032258065	[C4b, Casp1, Fos, Itga5, Itgb2l, Nlrp3, Zp3r]
KEGG:05200	Pathways in cancer	KEGG_25.05.2022	0.000541157	0.012852481	0.000541157	0.001082314	[-1]	Group03	4.97237587	27	2.18138551	[Agtr1a, Cdh1, Cks2, Col4a6, Dll3, Edn1, Esr1, Fgf5, Fos, Fzd1, Fzd4, Gstm2, Igf2, Il7, Lama1, Lamb3, Nfkbia, Ntrk1, Peg12, Pparg, Ptch2, Rarb, Rasgrp2, Ret, Traf1, Wnt8b, Wnt9a]
KEGG:05222	Small cell lung cancer	KEGG_25.05.2022	0.008487944	0.044797484	0.008487944	0.008487944	[-1]	Group04	7.526881695	7	3.303019904	[Cks2, Col4a6, Lama1, Lamb3, Nfkbia, Rarb, Traf1]
KEGG:04080	Neuroactive ligand-receptor interaction	KEGG_25.05.2022	0.004137808	0.037757644	0.004137808	0.005911154	[-1]	Group05	4.922279835	19	2.149851876	[Agtr1a, Avpr2, Chrnb3, Cort, Crh, Edn1, Edn3, Gabrr2, Grm2, Htr1d, Lepr, Lhb, Oprk1, Pomc, Prl, Ptgdr, S1pr4, Trhr2, Tspo]
KEGG:04625	C-type lectin receptor signaling pathway	KEGG_25.05.2022	0.000471943	0.014944863	0.000471943	0.001179858	[-1]	Group06	8.928571701	10	3.960253456	[Casp1, Cd209a, Cd209f, Clec1b, Clec7a, Egr2, Nfatc4, Nfkbia, Nlrp3, Rras2]
KEGG:04310	Wnt signaling pathway	KEGG_25.05.2022	0.000342202	0.016254611	0.000174695	0.000582317	[-1]	Group07	7.602339268	13	3.294930876	[Ccn4, Frzb, Fzd1, Fzd4, Lgr6, Nfatc4, Nkd2, Peg12, Rspo3, Sfrp1, Wif1, Wnt8b, Wnt9a]
KEGG:04390	Hippo signaling pathway	KEGG_25.05.2022	0.005906657	0.035070777	0.000174695	0.000582317	[-1]	Group07	6.369426727	10	2.807268273	[Bmp5, Cdh1, Fzd1, Fzd4, Itgb2l, Nkd2, Tead2, Trp73, Wnt8b, Wnt9a]
KEGG:04145	Phagosome	KEGG_25.05.2022	0.000622243	0.011822626	8.71492E-06	8.71492E-05	[-1]	Group08	7.142857075	13	3.150890182	[Atp6v1e2, Cd209a, Cd209f, Clec7a, Colec12, H2-Aa, H2-Eb1, H2-Q1, H2-T-ps, Itga5, Itgb2l, Mrc1, Thbs1]
KEGG:04514	Cell adhesion molecules	KEGG_25.05.2022	0.001359223	0.021521024	8.71492E-06	8.71492E-05	[-1]	Group08	6.896551609	12	2.94065229	[Cdh1, Cdh15, Cdh3, Cldn1, Cldn15, H2-Aa, H2-Eb1, H2-Q1, H2-T-ps, Itgb2l, Mpz, Vtcn1]
KEGG:05152	Tuberculosis	KEGG_25.05.2022	0.001814885	0.019157123	8.71492E-06	8.71492E-05	[-1]	Group08	6.666666508	12	2.956989247	[Cd209a, Cd209f, Cd74, Clec7a, H2-Aa, H2-Eb1, Itgax, Itgb2l, Mrc1, Nod2, Sphk1, Vdr]
KEGG:05416	Viral myocarditis	KEGG_25.05.2022	0.001541192	0.020916179	8.71492E-06	8.71492E-05	[-1]	Group08	9.090909004	8	3.77487989	[H2-Aa, H2-Eb1, H2-Q1, H2-T-ps, Itgb2l, Lama1, Myh6, Sgca]
KEGG:04510	Focal adhesion	KEGG_25.05.2022	0.001556317	0.018481268	0.000135311	0.000676557	[-1]	Group09	6.467661858	13	2.854519323	[Col1a2, Col2a1, Col4a6, Col6a4, Col6a5, Itga5, Itgb6, Lama1, Lamb3, Mylk3, Mylk4, Pdgfd, Thbs1]
KEGG:04512	ECM-receptor interaction	KEGG_25.05.2022	2.37748E-07	2.25881E-05	0.000135311	0.000676557	[-1]	Group09	14.77272701	13	6.478796665	[Col1a2, Col2a1, Col4a6, Col6a4, Col6a5, Dmp1, Itga5, Itgb6, Lama1, Lamb3, Npnt, Sv2c, Thbs1]
KEGG:04974	Protein digestion and absorption	KEGG_25.05.2022	0.005522708	0.037475523	0.000135311	0.000676557	[-1]	Group09	7.407407284	8	3.285543608	[Col11a1, Col13a1, Col1a2, Col2a1, Col4a6, Col6a4, Col6a5, Xpnpep2]
KEGG:05165	Human papillomavirus infection	KEGG_25.05.2022	0.005054959	0.036940083	0.000135311	0.000676557	[-1]	Group09	4.97237587	18	2.22391949	[Atp6v1e2, Col1a2, Col2a1, Col4a6, Col6a4, Col6a5, Fzd1, Fzd4, H2-Q1, H2-T-ps, Itga5, Itgb6, Lama1, Lamb3, Oasl2, Thbs1, Wnt8b, Wnt9a]
KEGG:05205	Proteoglycans in cancer	KEGG_25.05.2022	0.001856709	0.017638734	0.000135311	0.000676557	[-1]	Group09	6.341463566	13	2.826533639	[Col1a2, Dcn, Esr1, Fzd1, Fzd4, Igf2, Itga5, Lum, Rras2, Thbs1, Twist1, Wnt8b, Wnt9a]

MitoPark

ID	Term	Ontology Source	Term PValue	Term PValue Corrected with Benjamini-Hochberg	Group PValue	Group PValue Corrected with Benjamini-Hochberg	GOLevels	GOGroups	% Associated Genes	Nr. Genes	Rich Factor	Associated Genes Found
KEGG:04622	RIG-I-like receptor signaling pathway	KEGG_25.05.2022	0.011227557	0.020851178	0.011227557	0.011227557	[-1]	Group01	5.714285851	4	2.430402121	[Ddx58, Dhx58, Irf7, Isg15]
KEGG:04979	Cholesterol metabolism	KEGG_25.05.2022	0.003167329	0.008235054	0.003167329	0.005806769	[-1]	Group02	8.163265228	4	3.548387097	[Angptl4, Apob, Apoc1, Cd36]
KEGG:05165	Human papillomavirus infection	KEGG_25.05.2022	0.005421123	0.012436693	0.005421123	0.006625816	[-1]	Group03	3.038674116	11	1.35906191	[Cdkn1a, Eif2ak2, Fas, Fzd10, Heyl, Isg15, Mx1, Oasl1, Oasl2, Ptgs2, Tnxb]
KEGG:05204	Chemical carcinogenesis	KEGG_25.05.2022	0.003915139	0.009543152	0.003915139	0.005383316	[-1]	Group04	5.952381134	5	2.464157706	[Cyp3a13, Hsd11b1, Nat2, Ptgs2, Ugt1a6a]
KEGG:04514	Cell adhesion molecules	KEGG_25.05.2022	0.001462138	0.004073099	0.001462138	0.003216704	[-1]	Group05	4.597701073	8	1.96043486	[Cd2, Cldn14, Cldn8, H2-DMb2, Icos, Sele, Selp, Siglec1]
KEGG:05144	Malaria	KEGG_25.05.2022	0.005471801	0.01185557	7.90662E-05	0.000217432	[-1]	Group06	7.017543793	4	2.304147465	[Ccl12, Cd36, Sele, Selp]
KEGG:05417	Lipid and atherosclerosis	KEGG_25.05.2022	1.48671E-05	0.000144955	7.90662E-05	0.000217432	[-1]	Group06	5.555555344	12	2.452802141	[Apob, Casp1, Ccl12, Cd36, Cxcl1, Cyp2j8, Fas, Hspa1b, Hspa5, Irf7, Sele, Selp]
KEGG:00590	Arachidonic acid metabolism	KEGG_25.05.2022	0.022360913	0.034883026	0.009473461	0.010420807	[-1]	Group07	4.651162624	4	1.993475897	[Cyp2j8, Gpx2, Pla2g4e, Ptgs2]
KEGG:00591	Linoleic acid metabolism	KEGG_25.05.2022	0.024287213	0.03382862	0.009473461	0.010420807	[-1]	Group07	6	3	2.419354839	[Cyp2j8, Cyp3a13, Pla2g4e]
KEGG:04370	VEGF signaling pathway	KEGG_25.05.2022	0.035608072	0.047886718	0.009473461	0.010420807	[-1]	Group07	5.172413826	3	2.294215795	[Hspb1, Pla2g4e, Ptgs2]
KEGG:04613	Neutrophil extracellular trap formation	KEGG_25.05.2022	0.014828099	0.026286175	0.003235813	0.005084849	[-1]	Group08	3.38164258	7	1.485568761	[Casp1, Fcgr4, Fpr1, H2ac6, H2bc6, H3c4, Selp]
KEGG:05150	Staphylococcus aureus infection	KEGG_25.05.2022	0.001070756	0.003212268	0.003235813	0.005084849	[-1]	Group08	5.46875	7	2.425655242	[C1s2, Dsg1c, Fcgr2b, Fcgr4, Fpr1, H2-DMb2, Selp]
KEGG:05322	Systemic lupus erythematosus	KEGG_25.05.2022	0.010418855	0.020316767	0.003235813	0.005084849	[-1]	Group08	4.05405426	6	1.774193548	[C1s2, Fcgr4, H2-DMb2, H2ac6, H2bc6, H3c4]
KEGG:04621	NOD-like receptor signaling pathway	KEGG_25.05.2022	0.017098561	0.028993212	4.84259E-07	5.32685E-06	[-1]	Group09	3.286385059	7	1.437425329	[Casp1, Ccl12, Cxcl1, Ifi204, Irf7, Oas1a, Oas2]
KEGG:05160	Hepatitis C	KEGG_25.05.2022	6.17039E-06	0.000120323	4.84259E-07	5.32685E-06	[-1]	Group09	6.666666508	11	2.921576202	[Cdkn1a, Cldn14, Cldn8, Ddx58, Eif2ak2, Fas, Ifit1, Irf7, Mx1, Oas1a, Oas2]
KEGG:05162	Measles	KEGG_25.05.2022	1.87113E-06	7.29739E-05	4.84259E-07	5.32685E-06	[-1]	Group09	7.534246445	11	3.364849833	[Ddx58, Eif2ak2, Fas, Fcgr2b, Hspa1b, Il2ra, Il2rg, Irf7, Mx1, Oas1a, Oas2]
KEGG:05164	Influenza A	KEGG_25.05.2022	9.71137E-06	0.000126248	4.84259E-07	5.32685E-06	[-1]	Group09	6.358381271	11	2.804041528	[Calcoco2, Casp1, Ccl12, Ddx58, Eif2ak2, Fas, H2-DMb2, Irf7, Mx1, Oas1a, Oas2]
KEGG:05169	Epstein-Barr virus infection	KEGG_25.05.2022	0.000138663	0.000772552	4.84259E-07	5.32685E-06	[-1]	Group09	4.761904716	11	2.139926429	[Cd247, Cdkn1a, Ddx58, Eif2ak2, Fas, Fcer2a, H2-DMb2, Irf7, Isg15, Oas1a, Oas2]
KEGG:05171	Coronavirus disease	KEGG_25.05.2022	0.001006822	0.003272173	4.84259E-07	5.32685E-06	[-1]	Group09	4.048583031	10	1.327989183	[C1s2, Casp1, Ccl12, Ddx58, Eif2ak2, Isg15, Mx1, Oas1a, Oas2, Selp]
KEGG:04060	Cytokine-cytokine receptor interaction	KEGG_25.05.2022	0.000272171	0.001179408	8.6413E-07	4.75272E-06	[-1]	Group10	4.1095891	12	1.81040158	[Ccl12, Ccl17, Ccl22, Cxcl1, Cxcl5, Fas, Il13ra2, Il1r1, Il27ra, Il2ra, Il2rg, Il7r]
KEGG:04061	Viral protein interaction with cytokine and cytokine receptor	KEGG_25.05.2022	0.000175327	0.000854718	8.6413E-07	4.75272E-06	[-1]	Group10	7.368421078	7	3.268251273	[Ccl12, Ccl17, Ccl22, Cxcl1, Cxcl5, Il2ra, Il2rg]
KEGG:04630	JAK-STAT signaling pathway	KEGG_25.05.2022	0.018497365	0.030058216	8.6413E-07	4.75272E-06	[-1]	Group10	3.571428537	6	1.55631013	[Cdkn1a, Il13ra2, Il27ra, Il2ra, Il2rg, Il7r]
KEGG:04657	IL-17 signaling pathway	KEGG_25.05.2022	1.93844E-05	0.000151198	8.6413E-07	4.75272E-06	[-1]	Group10	8.602150917	8	3.77487989	[Ccl12, Ccl17, Cxcl1, Cxcl5, Lcn2, Ptgs2, S100a8, S100a9]
KEGG:04658	Th1 and Th2 cell differentiation	KEGG_25.05.2022	0.024097484	0.034807477	8.6413E-07	4.75272E-06	[-1]	Group10	4.545454502	4	2.016129032	[Cd247, H2-DMb2, Il2ra, Il2rg]
KEGG:04659	Th17 cell differentiation	KEGG_25.05.2022	0.000326037	0.001271545	8.6413E-07	4.75272E-06	[-1]	Group10	6.666666508	7	2.956989247	[Cd247, H2-DMb2, Il1r1, Il27ra, Il2ra, Il2rg, Runx1]
KEGG:04668	TNF signaling pathway	KEGG_25.05.2022	0.000509811	0.001807513	8.6413E-07	4.75272E-06	[-1]	Group10	6.194690228	7	2.631219245	[Ccl12, Cxcl1, Cxcl5, Fas, Gm5431, Ptgs2, Sele]
KEGG:05323	Rheumatoid arthritis	KEGG_25.05.2022	0.023219274	0.034828912	8.6413E-07	4.75272E-06	[-1]	Group10	4.597701073	4	2.039302929	[Ccl12, Cxcl1, Cxcl5, H2-DMb2]
KEGG:05340	Primary immunodeficiency	KEGG_25.05.2022	0.010029542	0.020586954	8.6413E-07	4.75272E-06	[-1]	Group10	8.333333015	3	3.696236559	[Icos, Il2rg, Il7r]

This table lists significantly enriched KEGG pathways. "MPTP" refers to the acute MPTP model, "A53T" to the 16m A53T-α-synuclein model, and "MitoPark" to the 5 months MitoPark model.
