# Supplementary material for: Neurotoxicity, α-synuclein pathology, and mitochondrial dysfunction: A comparative study of different mouse models of Parkinson’s disease
Source: Neural Regen Res. 2025 Sep 29;21(10):5000–12. doi: 10.4103/NRR.NRR-D-25-00648 (PMC13568611; doi:10.4103/NRR.NRR-D-25-00648)
Supplement: Supplementary file 5 [file NRR-21-5000_Suppl4.pdf]

Additional Table 6. Pathway enrichment results from GSEA analysis of RNA-Seq data in the striatum of three PD mouse models.

MPTP

ID	Description	setSize	enrichmentScore	NES	pvalue	p.adjust	qvalue	rank	leading_edge	core_enrichment
CHEN_METABOLIC_SYNDROM_NETWORK	CHEN_METABOLIC_SYNDROM_NETWORK	804	0.52864138	1.871916162	1.44928E-05	0.007103861	0.006538028	1691	tags=33%, list=14%, signal=30%	Efemp2/Ifi27l2a/Vim/Otulinl/Pimreg/Emp3/Npdc1/Nrros/Akr1b8/Cbfb/Il11ra1/Anxa4/Spsb2/Lrrc25/Soat1/Slc11a1/Ccl9/Cd86/Clec4a3/Themis2/Dapp1/Lgals1/Rpe65/Tifab/Klhl6/Plekhn1/Fcgr4/Mthfsl/Fes/Snx20/Slamf9/S100a6/Fcgr2b/Rac2/Was/Mpeg1/Myo1f/Lyz2/Lacc1/Ccdc80/Lgals3bp/Gsdmd/Slc15a3/Olfml3/Cd74/Lipa/Gmfg/Cd180/Msantd3/Ifit3/Pik3ap1/Csf3r/Cd14/Layn/Ctsh/Capg/Pdlim4/Ccl3/Plxnb2/Arhgap18/Tlr7/Trem2/Lcp2/Alox5ap/Il18bp/Pycard/Ppic/Tep1/Pld4/Mfrp/P3h2/Apobec1/Cd68/P2ry6/Il10ra/Igsf6/Dock2/Pdgfrl/Fcgr1/Nckap1l/Il10rb/Fermt3/Col1a1/Samsn1/Ly86/Hpgds/S100a11/Dock8/Cybc1/Phldb1/Ptpre/Stab1/Padi2/Cd53/Icosl/Irf8/Csf1r/Clic1/Irf5/Calhm
REACTOME_NEUTROPHIL_DEGRANULATION	REACTOME_NEUTROPHIL_DEGRANULATION	321	0.534579667	1.761771915	1.59155E-05	0.007103861	0.006538028	1841	tags=27%, list=16%, signal=23%	Snap23/Nfkb1/Ifi204/Tmem63a/Serpina3n/Slc11a1/Pld1/Chil1/Fcgr4/Fcgr2b/Lyz2/Gsdmd/Capn1/Bst2/Acly/Gmfg/Cd14/Ctsh/Clec5a/Pycard/H2-D1/Cd68/Dock2/Nckap1l/Mgst1/S100a11/Stom/Naprt/Padi2/Cd53/Gusb/Tcirg1/Slc2a5/Tlr2/Ptafr/Tyrobp/Sptan1/Rnaset2a/Dok3/Gpr84/B2m/Grn/Fcer1g/Aldh3b1/Cpped1/Vcl/Tmc6/Cyba/Cab39/Hexb/Stk11ip/C3ar1/Serpinb1a/Prdx6/Dynlt1b/Man2b1/Anxa2/Fpr1/Ctsz/Lair1/Ctsc/Cd63/Stk10/Itgb2/Rab3d/Vps35l/Hvcn1/Pygb/Fuca1/Faf2/Nme2/Vamp8/Atp11b/Lamp2/Hspa1b/Ctsa/Npc2/Cyb5r3/Tnfrsf1b/Apaf1/Gsn/Aga/Diaph1/Cmtm6/Cnn2/Dbnl
REACTOME_INNATE_IMMUNE_SYSTEM	REACTOME_INNATE_IMMUNE_SYSTEM	619	0.486631183	1.693965265	1.48914E-05	0.007103861	0.006538028	1897	tags=26%, list=16%, signal=23%	Snap23/Nfkb1/Ifi204/Tmem63a/Cyld/Isg15/Serpina3n/Btk/C4b/Slc11a1/Clec4a3/Pld1/Chil1/Fcgr4/Dnm2/Fcgr2b/Rac2/Was/Ifih1/Lyz2/Tlr3/Gsdmd/Capn1/Bst2/Acly/Arpc3/Gmfg/Cd180/Cd14/Ctsh/Ly96/Clec5a/Tlr7/Trem2/Lcp2/Pycard/H2-D1/Pld4/Tlr9/Ptpn11/Cd68/Ube2l6/Dock2/Fcgr1/Nckap1l/Mgst1/Ly86/S100a11/Stom/Pcbp2/Naprt/Padi2/Psme2/Cd53/Gusb/Tcirg1/Ddx58/Slc2a5/Ncf2/C1qc/Tlr2/Ptafr/Tyrobp/Mef2c/Abl1/Lgmn/Sptan1/Lbp/Plcg2/Rnaset2a/Dok3/Gpr84/Tnfaip3/Lyn/B2m/Stat6/Pros1/Ube2k/Grn/Fcer1g/Vav1/Aldh3b1/Icam2/Cpped1/Vcl/Serping1/C1qb/Rnf135/Tmc6/Pld2/Cyba/Cab39/Mre11a/Hexb/Stk11ip/Nod1/Ripk1/C3ar1/S100a1/H2-
LEE_BMP2_TARGETS_UP	LEE_BMP2_TARGETS_UP	530	0.481020907	1.654302668	1.51782E-05	0.007103861	0.006538028	1740	tags=28%, list=15%, signal=25%	Chd3/Aamdc/Npdc1/Akr1b8/Synpo2/Itgb5/Slc7a7/Ifitm1/Usp18/Pld1/Tmem140/Rarres2/Tmem35b/Gbp2/Igfbp5/Aspg/Ifih1/Nupr1/Ilvbl/Ednra/Lyz2/Ccdc80/Gsdmd/H2az2/Pamr1/Cd14/Ctsh/Fxyd1/Plxnb2/Scarb1/Pgghg/Tmem176a/Pycard/Efemp1/H2-D1/Apobec1/AU021092/Phldb1/Resf1/Sema4a/Stom/Naprt/Abca9/Htra3/Slc39a8/Cln3/Tcn2/Pbxip1/Rgma/Etfbkmt/Grb7/Gbp7/Rnase4/Ddah2/Celf2/Grn/Rcsd1/Abca1/Prkd3/Serping1/Bmf/Rab29/Mxra8/F5/Zfp467/Aebp1/Wipi1/Dtx3l/Pltp/Col5a1/Mycn/Slc6a9/Nbl1/Ptprd/Rspo3/Vwa5a/Rhobtb1/Aqp1/Adcy6/Krt18/S100a1/Thbd/H2-T23/Serpinb1a/Maf/Jak2/Clu/Gcnt1/4931406C07Rik/Pi
SANA_RESPONSE_TO_IFNG_UP	SANA_RESPONSE_TO_IFNG_UP	38	0.815175753	2.047865992	6.93577E-05	0.017469106	0.016077665	1025	tags=55%, list=9%, signal=51%	Mx2/Cxcl10/Golm1/Parp14/Parp9/Gbp2/Ifih1/Lgals3bp/Cd74/Bst2/H2-DMa/Rnf213/Il18bp/H2-D1/Ube2l6/Samd9l/Ifi35/Vamp5/Serping1/Dtx3l/H2-T23
XIE_ST_HSC_S1PR3_OE_UP	XIE_ST_HSC_S1PR3_OE_UP	95	0.653524105	1.874364153	6.81002E-05	0.017469106	0.016077665	975	tags=37%, list=8%, signal=34%	Rtp4/Isg15/Mx2/Cxcl10/Slc7a7/Gbp5/Ifitm1/Usp18/Dapp1/Parp9/Gbp2/Ifih1/Lgals3bp/Bst2/Ifit3/Cd52/Rnf213/Pdlim4/Slfn5/Ifitm3/Samd9l/Eif2ak2/Ifi35/Ddx58/Ifi27/Parp12/Epsti1/Pros1/Abca1/Vcl/Bmf/Ifit2/Dtx3l/Vwa5a/Arap3
GRAESSMANN_RESPONSE_TO_MC_AND_SERUM_DEPRIVATION_UP	GRAESSMANN_RESPONSE_TO_MC_AND_SERUM_DEPRIVATION_UP	133	0.62420742	1.869877684	6.70309E-05	0.017469106	0.016077665	1071	tags=31%, list=9%, signal=28%	Ifi204/Irf1/Rtp4/Isg15/Mx2/Cxcl10/Ccl9/Usp18/Parp14/Themis2/Fas/Parp9/Fcgr4/Tmem140/Fes/Ifit1/Gbp2/Ifih1/Tlr3/Lgals3bp/Lipa/Bst2/Ifit3/Zfp672/Resf1/Eif2ak2/Ifi35/Trim12c/Ifi27/Parp12/Tlr2/Tor3a/Casp7/Gbp7/Ifit2/Stk11ip/Fjx1/Aqp1/H2-T23/Boc/Osgin2
YANG_BCL3_TARGETS_UP	YANG_BCL3_TARGETS_UP	211	0.54994343	1.737620166	8.17862E-05	0.019701736	0.018132464	1225	tags=26%, list=10%, signal=23%	Pbk/Rtp4/S100a3/Pimreg/Mx2/Cxcl10/Ckap2/Ccnb2/Ccl9/Parp14/Top2a/Cep128/Tmem140/Il6ra/Gbp2/Igfbp5/Ifit3/Vps54/A2m/Cd68/Ube2l6/Rem2/Cdc20/Coasy/Pex12/Loxl3/Eif2ak2/Ifi35/Taf9b/Ddx58/Mki67/Trim12c/Ednrb/Gnb4/Pex11g/Jade1/Aass/Hmgb2/Abca1/Ifit2/Ccna2/Dtx3l/Ptn/Rrm2/Rspo3/Thbd/Gpx7/Serpinb1a/Tgfbr2/Rxrg/Acss1/Trf/Ctsc/E2f1

A53T

ID	Description	setSize	enrichmentScore	NES	pvalue	p.adjust	qvalue	rank	leading_edge	core_enrichment
LEIN_CHOROID_PLEXUS_MARKERS	LEIN_CHOROID_PLEXUS_MARKERS	103	-0.716290544	-2.156893831	1.6415E-05	0.085787539	0.085787539	5032	tags=77%, list=22%, signal=60%	Acad8/Pltp/Spata17/Cfap44[illegible]/Pcolce2/Kif9/Tmem23[illegible]nip/Stra6/Cfh/Tgfbi/No[illegible]Nxn/Myo7a/Slc39a12/[illegible]2/Ggh/Dnali1/Tcn2/Kl[illegible]a/Ezr/Sulf1/Vwa3a/Mg[illegible]p1/Rsph9/Slco1c1/Aca[illegible]c21a/Cpq/Csrp2/Lgals[illegible]Nek11/F13a1/Tbc1d9/[illegible]b2/Cab39l/4932438H2[illegible]/Nid2/Spint2/Fzd4/Per[illegible]d1/Ace/Adh1/Fhad1/S[illegible]/St6galnac2/Col9a3/R[illegible]7/Pcolce/Calml4/Msx1/[illegible]a20a/Lbp/Lepr/Prr32/C[illegible]2/Cldn1/Lum/Trpv4/Ab[illegible]mprss11a/Otx2/Sostdc[illegible]Prlr/Slc4a5/F5/Col8a1/[illegible]4/Ttr/Kcne2/Folr1/Tme[illegible]Cdh3/Cldn2/Wfdc2
SMID_BREAST_CANCER_LUMINAL_B_DN	SMID_BREAST_CANCER_LUMINAL_B_DN	527	-0.411950988	-1.443827571	2.71308E-05	0.085787539	0.085787539	4873	tags=33%, list=21%, signal=26%	Il1r2/Tmem45a/Adamts1/A[illegible]/Adcy2/Fbxo17/Csgaln[illegible]Ctsc/Ccl11/Pcolce2/Fbl[illegible]abre/Tcf7l2/Cyp39a1/[illegible]/Eln/Pdpn/Cald1/Emcn[illegible]1/Stxbp6/Gabbr2/Mfap[illegible]p1/Isg20/Nfil3/Wls/Se[illegible]1/Angptl4/Tfpi/Mpzl2/[illegible]a3/Ltf/Nfib/Ccl5/Bace2[illegible]/Adm/Mfge8/Gzmk/Gs[illegible]ell/Trbc2/Hsd17b2/An[illegible]H2-[illegible]ghg2c/Fhod3/Nlrp1a/T[illegible]11b/Igf1/Plac8/Ptger4/[illegible]10/Pamr1/Fzd7/Tdo2/T[illegible]8/Clec10a/Lamp3/Csrp[illegible]Ptgs2/Crybg1/Serping1[illegible]2/Mylk/Saa1/Foxc1/Vc[illegible]Ace2/C7/Tap1/Prelp/H[illegible]Chrdl1/Capn6/Smco4/[illegible]/Ccr2/Krt23/Zic1/Cd2[illegible]ia/Fabp7/Perp/Dsg3/Sf[illegible]tras2/Slc43a3/Fbln1/V
REACTOME_PROLACTIN_RECEPTOR_SIGNALING	REACTOME_PROLACTIN_RECEPTOR_SIGNALING	13	-0.871124464	-1.799154078	0.00011085	0.154802702	0.154802702	646	tags=23%, list=3%, signal=22%	Prlr/Prl/Gh
MEISSNER_BRAIN_HCP_WITH_H3K27ME3	MEISSNER_BRAIN_HCP_WITH_H3K27ME3	225	-0.482328916	-1.582249324	9.10056E-05	0.154802702	0.154802702	3742	tags=46%, list=16%, signal=39%	Prom2/Espn/Slc30a2/Rem1/Aebp1/Alx4/Marcksl1/Crb3/Hnf1b/Cyp26a1/Chmp4c/Grin3b/Gbx2/Ncmap/Tmem30b/Vsx2/Pou2f3/Prdm6/Calcr/Gpr50/Foxc2/Pou4f3/Glp1r/Ltbp2/Sim1/Fgfr4/Spag16/Bmp8a/Cldn3/Alx3/Mfsd6l/Irx2/Phox2a/Igf2bp1/Fgf4/Nkx2-4/Gsc/Plbd1/Pitx2/Chrna3/Trp73/Bhlhe23/Gal/Hoxd8/Large2/Slc44a3/Dlx3/Dlx4/Dmrta1/Loxl1/Rax/Cblc/Krt18/Calb2/Aldh1a3/Barhl2/Epcam/Prrx2/Alx1/Onecut3/Col8a2/Col13a1/Scnn1b/Aqp5/Trpv4/Tmem54/Pitx1/Mesp1/Wdr86/Hoxb7/Fev/Tbx21/Emilin3/Ntf5/Utf1/Nags/Ffar4/Hoxc8/Igf2/Cldn7/Gfi1/Kcnk15/Foxd3/Mixl1/Lgr6/Ppm1n/Dmrt3/Kdf1/Crlf1/Hoxb9/Lbx2/Six2/Tfap2c/Foxl1/Lmx1a/Tlx2/Barhl1/Ga
MCBRYAN_PUBERTAL_BREAST_4_5WK_UP	MCBRYAN_PUBERTAL_BREAST_4_5WK_UP	264	-0.459163467	-1.528781905	0.000148723	0.154802702	0.154802702	4702	tags=33%, list=21%, signal=27%	Sox4/Igfbp6/Wwc1/Esrp2/Cald1/Hebp2/Nxn/Id2/Ano1/Tle3/Errfi1/Nfil3/Atp1a1/Ltf/Slc39a6/Mfge8/Pdlim4/Tmem40/Thbs2/Ezr/Ptprk/Cpt1a/Cxcl14/Rbp1/Atp6v1b1/Fbln7/Acta2/Tnfrsf12a/Prom2/Gas1/Plac8/Cck/Emb/St14/Hspa1b/Ocln/Kif22/Marcksl1/Grhl2/Esrp1/Hspa1a/Dusp6/Areg/Dsp/Pawr/Bcl6/Pof1b/Tmem30b/Irx1/Col9a1/Sdc1/Tead2/Perp/Sfrp1/Boc/Cldn3/Ripk4/H2-Eb1/Irx2/Upk3a/2610528J11Rik/Foxa1/Aldh1a7/Btn1a1/Ly6d/Thbs1/Irx5/Egr2/Serpinb5/Krt18/Fos/Npr3/Cdh1/Epcam/Cnn1/Pdzk1ip1/Ehf/Aqp5/Tspan8/Actg2/Igfbp2/Cldn7/Prlr/Krt8/Folr1/Tf
MIKKELSEN_NPC_HCP_WITH_H3K27ME3	MIKKELSEN_NPC_HCP_WITH_H3K27ME3	320	-0.445490257	-1.507333157	0.000130795	0.154802702	0.154802702	4432	tags=33%, list=19%, signal=27%	Amigo2/Tcerg1l/Kcnk3/Fezf2/Fam43b/Llgl2/Wnt10a/Rasgef1c/Skap1/Rab11fip1/Slc17a6/Cckbr/Ralyl/T/Htr1a/Cxcl14/Stbd1/Mpped1/Htr7/Kcnq1/Kcns3/Rtn4r/Crb3/Sowahb/Scube1/Cbln4/Cyp26a1/Il11/Bmp6/Ihh/Tfap2d/Neurod2/Prok2/Wnt4/Slc30a3/Car10/Pou2f3/Calcr/Pou4f3/Ltbp2/Lrat/Sim1/Nptxr/Scnn1g/Colec12/Sfrp1/Ovol2/Ripk4/Alx3/Hoxc6/Phlda2/Igf2bp1/Hoxc9/Fgf4/Ucn/Skor1/Gjb2/Trp73/Bhlhe23/Cpz/Jhy/Emx1/Dlx3/Cartpt/Hs3st6/Slc6a20a/Npr3/Cdh1/Gsx1/Alx1/D930020B18Rik/Onecut3/Hoxb8/Hoxb3/Col13a1/Sp8/Aldh1a2/Pitx1/Mesp1/Hoxb7/Fev/Tbx21/Utf1/Ffar4/Hoxc8/Igf2/Cldn7/Gfi1/Foxd3/Mixl1/Bnc2/Ppm1n/Dmrt3/Tfap2b/Slc6a3/Hoxb9/Atp12a/Tfap2c/Foxl
MEISSNER_BRAIN_HCP_WITH_H3K4ME3_AND_H3K27ME3	MEISSNER_BRAIN_HCP_WITH_H3K4ME3_AND_H3K27ME3	1066	-0.357085644	-1.296078364	0.00017135	0.154802702	0.154802702	5654	tags=37%, list=25%, signal=29%	Bub1/Mal2/Odf3b/Sigirr/Dcxr/Smoc2/Tnfaip8l3/Heyl/Smo/Mlkl/F3/Tmem98/Arx/Thsd7b/Syngr2/Itga8/Shisa2/C1ql1/Nrip3/Dsg2/Htra1/Crtac1/Sstr1/Wrap73/0610040J01Rik/Abt1/Nell1/Sdc4/Slc12a4/Pax6/Mecom/Mast1/F2rl1/Myo10/Stk17b/Svep1/Il4ra/Hmcn1/Prss12/Mmp14/Nfix/Swap70/Coa6/Hcn1/Tifa/Ablim3/Snagp/Hic1/Bhlha9/Mmp23/Pappa/Lhfpl2/Etl4/Nifk/Ptger2/Gldc/Pltp/Adgrg6/Kcnk9/Vstm2b/Kctd11/Fn1/Prkcq/Gnas/Prdm16/Cnn2/Cpne9/Lrp5/Cd83/Vax1/Egln3/Hapln4/Emp2/F11r/Eya4/Dpp10/Wnt1/Acer2/Parp14/Il17rd/Epha3/Hs3st3b1/Uhrf1/Ppm1l/Tcf7l2/Maff/Smad6/Smoc1/Slc7a10/Txnip/Eif4ebp1/Cavin1/Plekhf1/Epha2/Pou3f2/Pcdh8/Cgas/Ntn4/Lamc3/Trim
BOQUEST_STEM_CELL_CULTURED_VS_FRESH_UP	BOQUEST_STEM_CELL_CULTURED_VS_FRESH_UP	415	-0.42008006	-1.448710271	0.000210574	0.166458433	0.166458433	5431	tags=35%, list=24%, signal=27%	Fhl2/Ing1/Rai14/Me1/Adam12/Slc5a3/Myo10/Mettl7a1/Ppp1r3c/Adamtsl3/Tpm4/Id1/Pappa/Spry1/Dkk2/Ptger2/Pltp/Tsc22d3/Fn1/Nqo1/Serpine1/Hspb7/Cd83/Emp2/Mical2/Ggt5/Slc7a5/Epha3/Sox4/Srpx2/Apoc1/Txnip/Acsl5/Cfh/Col18a1/Spata6/Dusp1/Ano1/Ackr3/Sobp/Sema3g/Tmem176a/Top2a/Angptl4/Tfpi/Pclaf/Nfib/Apol7a/Aspm/Oxtr/Sulf1/Tgfb2/Klf4/Mgp/Cd34/Ifih1/Nox4/Col4a5/Slc7a11/Mybl1/Gpx3/Apold1/Acacb/Rai2/Tnfrsf11b/Igf1/Trib1/Rrm2/Cxcl10/Aebp1/Egr1/Atf3/Cyp4b1/Hspa1b/Kcns3/Wnt5a/Junb/Maf/Dcx/Npy1r/[illegible]Hspa1a/Btg2/Dsp[illegible]nf1/Vcam1/Fgl2/[illegible]

MitoPark

ID	Description	setSize	enrichmentScore	NES	pvalue	p.adjust	qvalue	rank	leading_edge	core_enrichment
XIE_ST_HSC_S1PR3_OE_UP	XIE_ST_HSC_S1PR3_OE_UP	23	-0.570934256	-2.077719121	0.000730518	0.105451188	0.093357189	148	tags=100%, list=47%, signal=57%	Rnf213/Parp10/Ifi27/Eif2ak2/Samd9l/Helz2/Dtx3l/Ddx60/Slfn5/Rtp4/Ifitm3/Dhx58/Irf7/Hspb1/Isg15/Oas2/Ifi44/Usp18/Oas1a/Isg20/Oasl1/Selp
ALTEMEIER_RESPONSE_TO_LPS_WITH_MECHANICAL_VENTILATION	ALTEMEIER_RESPONSE_TO_LPS_WITH_MECHANICAL_VENTILATION	14	-0.648088289	-2.03789131	0.001096644	0.105451188	0.093357189	90	tags=86%, list=29%, signal=64%	Irf7/Isg15/Oas2/Ifit1/Usp18/Bst1/Cxcl5/Ch25h/Tgm1/Cxcl1/Slfn4/Selp
BOYLAN_MULTIPLE_MYELOMA_PCA1_UP	BOYLAN_MULTIPLE_MYELOMA_PCA1_UP	10	-0.708747985	-2.000531502	0.001298286	0.105451188	0.093357189	41	tags=60%, list=13%, signal=54%	Isg20/S100a8/S100a9/Slfn4/Ltf/Lcn2
LEE_LIVER_CANCER_CIPROFIBRATE_UP	LEE_LIVER_CANCER_CIPROFIBRATE_UP	6	-0.829458473	-1.963039741	0.000521837	0.105451188	0.093357189	24	tags=67%, list=8%, signal=63%	S100a9/Tm4sf4/Lcn2/Sprr1a
REACTOME_ANTIMICROBIAL_PEPTIDES	REACTOME_ANTIMICROBIAL_PEPTIDES	5	-0.875966089	-1.944683835	0.000441235	0.105451188	0.093357189	27	tags=80%, list=9%, signal=74%	S100a8/S100a9/Ltf/Lcn2
MCBRYAN_PUBERTAL_BREAST_3_4WK_UP	MCBRYAN_PUBERTAL_BREAST_3_4WK_UP	6	-0.805610597	-1.906600113	0.001485228	0.105451188	0.093357189	13	tags=67%, list=4%, signal=65%	Hspa1b/Ltf/Lcn2/Sprr1a
BLANCO_MELO_BETA_INTERFERON_TREATED_BRONCHIAL_EPITHELIAL_CELLS_DN	BLANCO_MELO_BETA_INTERFERON_TREATED_BRONCHIAL_EPITHELIAL_CELLS_DN	5	-0.852425833	-1.892423417	0.000882471	0.105451188	0.093357189	27	tags=80%, list=9%, signal=74%	S100a8/S100a9/Sprr1b/Sprr1a
BLANCO_MELO_COVID19_BRONCHIAL_EPITHELIAL_CELLS_SARS_COV_2_INFECTION_UP	BLANCO_MELO_COVID19_BRONCHIAL_EPITHELIAL_CELLS_SARS_COV_2_INFECTION_UP	16	-0.60797947	-1.993101933	0.001908169	0.116298	0.10296	94	tags=75%, list=30%, signal=55%	Ifitm3/Irf7/Oas2/Cyp27b1/Ifi44/Oas1a/Cxcl5/Tgm1/Cxcl1/S100a8/S100a9/Sprr1a

BOCHKIS_FOXA2_TARGETS	BOCHKIS_FOXA2_TARGETS	294	0.509347543	1.665078268	9.59447E-05	0.021377225	0.019674497	1213	tags=18%, list=10%, signal=17%	Zscan12/Rdx/Ifi27l2a/Isg15/Nrros/Serpina3n/Il11ra1/Eaf1b2/Clec4a3/Chil1/1600014C10Rik/Nuf2/Tmem140/Bsc1a1/Prdx1/Depp1/Plod2/Rik3ap1/Apoc1/Scarb1/Zbscan5/Dhrs1/Ube2l6/Mlxipl/Pigo/Hmx4/Hacl1/Hagh/Ttc7/Chac2/Lyn/Pros1/Aass/Cyp4f14/Id3/Prl/Bbgb2/Fbp2a/Abca1/Efna1/Cd9/Thpo/Pld2/Zmym1/Mt2/Tnfrsf1a/Tfap4/Pedu6/Ap3m1/Pex12c/G
MOSERLE_IFNA_RESPONSE	MOSERLE_IFNA_RESPONSE	13	0.917196643	1.912025939	0.000161693	0.032299592	0.029726881	854	tags=85%, list=7%, signal=79%	Rtp4/Mx2/Cxcl10/Ifitm1/Usp18/Ifih1/Ifit3/Samd9l/Ddx58/Eaf...
HECKER_IFNB1_TARGETS	HECKER_IFNB1_TARGETS	41	0.780822106	1.985409032	0.000173259	0.033456347	0.0307915	1185	tags=63%, list=10%, signal=57%	Rtp4/Isg15/Mx2/Cxcl10/Parp9/Chil1/Tmem140/Lgals3bp/Ifit3/Samd9l/Eif2ak2/Ifi35/Ccl12/Ddx58/C1qc/Ifi27/Parp12/Epsti1/Pros1/Serping1/C1qb/Ifit2/Dtx3l/C3ar1/C
FULCHER_INFLAMMATORY_RESPONSE_LECTIN_VS_LPS_DN	FULCHER_INFLAMMATORY_RESPONSE_LECTIN_VS_LPS_DN	285	0.503298714	1.640325844	0.000192443	0.035251261	0.032443446	1466	tags=29%, list=13%, signal=26%	Rdx/Ifi204/Ifi27l2a/Mx2/Cxcl10/Synpo2/Ifitm1/Lcn2/Usp18/Depp1/Fas/Pdia6/Fcgr4/Tmem140/Gbp2/Ifih1/Npr1/Lacc1/Lgals3bp/Ifit3/Pik3ap1/Cst3r/Cd14/Rnf213/Apoc1/Scarb1/Fyb/Armcx1/Lcp2/Slfn5/Ube2l6/Pdgfrl/Zeb2/Ifitm3/Fcgr1/Ly96/Samd9l/Sema4a/Stom/Stab1/Ddx58/Trim12c/Klhdc8b/Slc39a8/Ifi27/Epsti1/Parvg/Gab4/Lgmn/Rnase2a/Lst1/Lyn/Lxn/Vamp5/Slc12a7/Ddah2/Serping1/Ifit2/I830077J02Rik/Mafb/Maml2/C3ar1/Maf/Tgfbr2/Fpr1/Lair1/Sat1/Ctsc/Plekha2/Hmox1/H2bc4/Myd88/Ctsl/Ccnl1/Npl/Samd4/Slc46a3/Casp6/Unc93b1
LIAN_LIPA_TARGETS_3M	LIAN_LIPA_TARGETS_3M	29	0.819536099	1.971355907	0.00024464	0.040722264	0.037478675	1245	tags=59%, list=11%, signal=53%	Slc11a1/Fcgr2b/Mpeg1/Slc15a3/Tlr7/Trem2/Apobec1/Cd68/Igsf6/C1qc/Fcer1g/C1qb/Mafb/C3ar1/Fabp7/C1qa/
ALTEMEIER_RESPONSE_TO_LPS_WITH_MECHANICAL_VENTILATION	ALTEMEIER_RESPONSE_TO_LPS_WITH_MECHANICAL_VENTILATION	58	0.713962822	1.910208876	0.000310452	0.04279912	0.039390107	1424	tags=57%, list=12%, signal=50%	Isg15/Mx2/Cxcl10/Ccl9/Usp18/Sla/Ifit1/Fcgr2b/Gbp2/Ifih1/Slc15a3/Csf3r/Cd14/Ccl3/Lcp2/Rcan1/Osmr/Fcgr1/Gem/Trim12c/Tlr2/Gpr84/Lst1/Tnfaip3/Ebi3/Fcer1g/Ifit2/Mt2/Nfil3/Litaf/Mt1/Tifa/
GRAESSMANN_APOPTOSIS_BY_SERUM_DEPRIVATION_UP	GRAESSMANN_APOPTOSIS_BY_SERUM_DEPRIVATION_UP	374	0.470887433	1.574125213	0.000298301	0.04279912	0.039390107	1545	tags=20%, list=13%, signal=18%	Ifi204/Irf1/Rtp4/Isg15/Mx2/Cxcl10/Wnt7b/C4b/Ccl9/Usp18/Parp14/Themis2/Fas/Parp9/Dtwd1/Trip4/Fcgr4/Tmem140/Fes/Septin7/Ifit1/Gbp2/Ifih1/Tlr3/Lgals3bp/Lipa/Bst2/Adamts10/Ifit3/Plp2/Il18bp/H2-D1/Apobec1/Osmr/Zeb2/Tpbg/Xdh/Zfp672/Resf1/Eif2ak2/Ifi35/Trim12c/Ifi27/Parp12/Tlr2/Tor3a/Casp7/Gbp7/Sirt4/Ifit2/Stk11ip/Slc6a9/Vwa5a/Fjx1/Aqp1/H2-T23/Boc/Osgin2/Apex2/Itsn1/Angpt1/Cip2a/Tirap/Hadh/Dkk3/Tifa/Myd88/Pdk4/Mfsd1/Csf1/Tmem168/Plpp3/S
BROWN_MYELOID_CELL_DEVELOPMENT_UP	BROWN_MYELOID_CELL_DEVELOPMENT_UP	100	0.62583524	1.807551139	0.000407297	0.052432754	0.048256407	1393	tags=34%, list=12%, signal=30%	Anxa4/Soat1/Ccl9/Clec4a3/Tifab/S100a6/Fcgr2b/Mpeg1/Slc15a3/Cd52/Capg/Clec5a/Trem2/Cd68/Fcgr1/S100a11/Xdh/Stom/Csf2ra/Tlr2/Tyrobp/Glipr2/Rnase4/Grn/Zyx/Hexb/Tnfrsf1a/Fpr1/Slc31a2/Sat1/Emcn/Vsir/Itgb2/C
COLINA_TARGETS_OF_4EBP1_AND_4EBP2	COLINA_TARGETS_OF_4EBP1_AND_4EBP2	246	0.510331442	1.639157152	0.000421353	0.053062982	0.048836436	1834	tags=32%, list=16%, signal=27%	Nfkb1/Pbk/Rtp4/Ifi27l2a/Isg15/C4b/Usp18/Top2a/Nuf2/Gbp2/Lgals3bp/Bst2/Plod2/Cd14/Ctsh/Ly96/Dhrs1/H2-D1/Skap2/Ifitm3/Scara3/Mgst1/Cdc20/Ifi35/Tcirg1/Igfbp2/Trim12c/Pon3/Parp12/Tor3a/Lbp/Slc16a3/B2m/Rnase4/Lxn/Vamp5/Celf2/Grn/Id3/S100a13/Ifit2/Plk1/Ccna2/Mt2/Hexb/Ppp1r3c/S100a1/Thbd/H2-T23/Gpx7/Fabp7/Pcbd2/Aup1/Foxc1/Ifngr1/Mt1/Nat8/Sod3/Cdk8/Vcam1/Foxp2/Fuca1/Grwd1/Mfsd1/Hnrnpr/Nek2/Plpp3/Melk/Abcc4/Lamp2/Pdpn/Lpar6/Tmem47/
CASTELLANO_NRAS_TARGETS_UP	CASTELLANO_NRAS_TARGETS_UP	48	0.733755422	1.909676151	0.000502304	0.058196903	0.053561433	1067	tags=35%, list=9%, signal=32%	Ifi27l2a/Zfp637/Isg15/Anxa4/Usp18/Ifit1/Gbp2/Ifih1/H2-D1/Ifi35/Pon3/Gja1/Lbp/B2
WP_SARSCOV2_INNATE_IMMUNITY_EVASION_AND_CELLSPECIFIC_IMMUNE_RESPONSE	WP_SARSCOV2_INNATE_IMMUNITY_EVASION_AND_CELLSPECIFIC_IMMUNE_RESPONSE	33	0.786095105	1.931650978	0.000609607	0.068780174	0.063301731	1120	tags=42%, list=10%, signal=38%	Nfkb1/Mx2/Cxcl10/Ifitm1/Lag3/Il6ra/Ccl3/Tlr7/Ddx58/Havcr2/Ifit2/Tgfb1/Ripk1/Mavs
ROZANOV_MMP14_TARGETS_UP	ROZANOV_MMP14_TARGETS_UP	176	0.542187871	1.678141378	0.000627995	0.068780174	0.063301731	1474	tags=29%, list=13%, signal=26%	Efemp2/Nnt/Cst7/Lamb1/Mx2/Ifitm1/Gbp2/Nupr1/Lgals3bp/Gmfg/H2-DMa/Plod2/Mia/Dok1/Pycard/Efemp1/H2-D1/Pdgfrl/Loxl3/Uaca/Htra3/Igfbp2/Ncf2/Tlr2/Lgmn/Col4a1/Kif1b/Slc12a7/Icam2/Daam2/Col5a1/Fkbp7/Prdx6/Mdk/Boc/Hcls1/Pik3ip1/Lmbr1l/Mmp14/Gjb2/Tmem74b/Herpud1/Pnpla7/Sod3/Ptgs1/Gpr55/H2-DMb1/Pygb/Serpinh1/Fuca
REACTOME_EXTRACELLULAR_MATRIX_ORGANIZATION	REACTOME_EXTRACELLULAR_MATRIX_ORGANIZATION	139	0.566789705	1.707421183	0.000684668	0.073449708	0.06759933	2135	tags=39%, list=18%, signal=32%	Efemp2/Lamb1/P4ha3/Itgb5/Capn1/Col20a1/Plod2/A2m/Icam1/Efemp1/P3h2/Col1a1/Col24a1/Loxl3/Col27a1/F11r/Col4a1/Dag1/Mmp2/Icam2/Loxl1/Aspn/Tgfb1/Col5a1/Col9a3/Lrp4/Mfap3/Mmp14/Bmp1/Itgb2/Spock3/Ctsl/Vcam1/Serpinh1/Itga1/Col1a2/Col11a2/Bmp4/Bgn/Lama5/Capns1/Bmp2/Plod3/Adam17/Plod1/Lamb2/Itga11/Mfap2/Bcan/Lamc1/Ncam1/Kdr/Itga9/Ctsb
GRANDVAUX_IFN_RESPONSE_NOT_VIA_IRF3	GRANDVAUX_IFN_RESPONSE_NOT_VIA_IRF3	7	0.951969178	1.758142866	0.000717611	0.075584	0.069563622	568	tags=100%, list=5%, signal=95%	Irf1/Mx2/Ifitm1/Gbp2/Ifitm3/Eif2ak2/Ifi35
KIM_ALL_DISORDERS_CALB1_CORR_UP	KIM_ALL_DISORDERS_CALB1_CORR_UP	488	-0.412154879	-1.468649215	0.00089124	0.088919398	0.081836836	3263	tags=39%, list=28%, signal=29%	Psma4/Opcml/Pcsk1/Togaram1/Stxbp1/Rnf11/Uba1/Scg5/Kars/Aldoa/Atp6v1b2/Glrb/Atrnl1/Psme3/Ywhab/Pdhb/Cct2/Cops3/Zfand5/Atp6v0a1/Slc25a11/Rraga/Cyc1/Ran/Got1/Bnip3/Dhcr24/Sub1/Mpc2/Zc3h15/Trim23/Slc25a3/Ube2a/Dusp3/Lrpprc/Ppp1r16b/Psmd1/Iscu/Sumo1/Tspyl5/Elmo1/Ube2e3/Eps15/Snu13/Gabbr2/Psmd11/Cox7a2/Hsp90aa1/Atp1a3/Prkar1a/Ppid/Tob1/Ndrg4/Zbtb18/Pafah1b1/Polr2e/Slc25a4/Wdr7/Ppp2r1a/Pfkp/Nmnat2/Pak1/Idh3b/Clstn1/Sorl1/Bhlhe40/Atp5c1/Pex19/Thy1/Sec23a/Hmgcr/Asns/Mllt11/Pnma2/Atp6v1c1/Prps1/Got2/Cdk5/Pisd/Rfk/Srsf2/Rad23b/Vdac2/Csrnp2/Stip1/Drg1/Pgk1/Dnaja1/Btbd8/Necap1/Atic/Prpf8/Psmc6/Tkt/Arpp19/Kif3c/
REACTOME_RRNA_MODIFICATION_IN_THE_MITOCHONDRION	REACTOME_RRNA_MODIFICATION_IN_THE_MITOCHONDRION	6	0.958828085	1.715992494	0.000944672	0.089712854	0.082567092	10	tags=17%, list=0%, signal=17%	Nsun4
REACTOME_DDX58_IFIH1_MEDIATED_INDUCTION_OF_INTERFERON_ALPHA_BETA	REACTOME_DDX58_IFIH1_MEDIATED_INDUCTION_OF_INTERFERON_ALPHA_BETA	47	0.713884429	1.851997397	0.001075101	0.09731344	0.08956228	1120	tags=26%, list=10%, signal=23%	Nfkb1/Cyld/Isg15/Ifih1/Ube2l6/Pcbp2/Ddx58/Tnfaip3/Ube2k/Rnf135/Ripk1/Mavs
NUYTTEN_EZH2_TARGETS_UP	NUYTTEN_EZH2_TARGETS_UP	732	0.398310258	1.401585315	0.001068689	0.09731344	0.08956228	1584	tags=19%, list=14%, signal=17%	Arl5b/Tspan9/Arhgap17/Irf1/Rtp4/Cyld/Mx2/Cpeb2/Cxcl10/S100a4/Tpi1/Phf20l1/Anxa4/Itgb5/Gbp5/Soat1/Ifitm1/Usp18/Parp14/Themis2/Fas/Parp9/Kbtbd8/Lima1/Tmem140/Il6ra/Gbp2/Ifih1/Tlr3/Slc15a3/Prdx1/Depp1/Lipa/Plod2/Plxnb2/Scarb1/Fyb/Papln/Uap1/Rcan1/Gulp1/Icam1/Klhl4/Slfn5/Efemp1/Ppic/H2-D1/Prrg1/Ube2l6/Tpbg/Zfhx4/Samd9l/Resf1/Stom/Vezt/Eif2ak2/Psme2/Gem/Kdm3a/Ddx58/Ncf2/Trim12c/Gja1/Parp12/Tlr2/Epsti1/Msi1/Lgmn/Slc16a3/Casp7/Glipr2/Slc37a2/Tnfaip3/Evc/Myo1e/B2m/Kif1b/Msra/Hk2/Fhip2a/Loxl1/Fbxo36/Adrb2/Bmf/Cab39/Dtx3l/Slc9a9/Col5a1/Mt2/Rgl1/Ankrd37/H2-T23/Jak2/Rhoc/Tgfbr2/Ap3m1/Dynlt1b/Anxa2/Baiap2/
DAUER_STAT3_TARGETS_DN	DAUER_STAT3_TARGETS_DN	29	0.783228346	1.884019299	0.001170776	0.10184415	0.093732112	799	tags=45%, list=7%, signal=42%	Isg15/Mx2/Ifitm1/Usp18/Ifih1/Tlr3/Ifit3/Ifi35/Ddx58/Trim12c/Ifi27/Parp12/Id3

JINESH_BLEBBISHIELD_VS_LIVE_CONTROL_DN	JINESH_BLEBBISHIELD_VS_LIVE_CONTROL_DN	16	-0.599393617	-1.964955455	0.002325581	0.116298	0.10296	80	tags=62%, list=26%, signal=49%	Oas2/Phf11d/Ifi44/Oas1a/Oasl1/Cxcl1/S100a8/S100a9/Lcn2/Sprr1a
MCDOWELL_ACUTE_LUNG_INJURY_UP	MCDOWELL_ACUTE_LUNG_INJURY_UP	8	-0.730481251	-1.912631973	0.00234	0.116298	0.10296	27	tags=50%, list=9%, signal=47%	S100a8/Slfn4/Ltf/Lcn2
CHYLA_CBFA2T3_TARGETS_DN	CHYLA_CBFA2T3_TARGETS_DN	16	-0.585369504	-1.91898062	0.003200159	0.122579937	0.108521473	50	tags=56%, list=16%, signal=50%	Slc38a5/Oas1a/Isg20/Oasl1/S100a8/S100a9/Slfn4/Ltf/Lcn2
CAIRO_LIVER_DEVELOPMENT_DN	CAIRO_LIVER_DEVELOPMENT_DN	11	-0.652198356	-1.90010679	0.003452956	0.122579937	0.108521473	41	tags=55%, list=13%, signal=49%	Isg20/Apob/S100a8/S100a9/Ltf/Lcn2
GERY_CEBP_TARGETS	GERY_CEBP_TARGETS	9	-0.695652786	-1.898047671	0.003408819	0.122579937	0.108521473	88	tags=89%, list=28%, signal=66%	Nnpb2/Ifi204/Hopa1b/Cxcl5/Cxcl1/S100a8/Slfn4/Lcn2
SENGUPTA_NASOPHARYNGEAL_CARCINOMA_WITH_LMP1_DN	SENGUPTA_NASOPHARYNGEAL_CARCINOMA_WITH_LMP1_DN	8	-0.718610977	-1.881551825	0.00338	0.122579937	0.108521473	27	tags=75%, list=9%, signal=70%	Cxcl5/S100a8/S100a9/Sprr1b/Lcn2/Sprr1a
REACTOME_NEUTROPHIL_DEGRANULATION	REACTOME_NEUTROPHIL_DEGRANULATION	14	-0.597805865	-1.879779958	0.004346699	0.135019341	0.119534225	77	tags=64%, list=25%, signal=51%	Ifi204/Hopa1b/Fcgr4/Bst1/Cxcl1/S100a8/S100a9/Ltf/Lcn2
LEE_DIFFERENTIATING_T_LYMPHOCYTE	LEE_DIFFERENTIATING_T_LYMPHOCYTE	5	0.802879313	1.789718886	0.004162517	0.135019341	0.119534225	18	tags=60%, list=6%, signal=57%	Cd247/Cd2/Icos
ZHOU_INFLAMMATORY_RESPONSE_LPS_UP	ZHOU_INFLAMMATORY_RESPONSE_LPS_UP	19	-0.543057628	-1.874486018	0.004694278	0.13723859	0.121498953	110	tags=79%, list=35%, signal=54%	Slfn5/Il1r1/Ptgs2/Rtp4/Irf7/Isg15/Oas2/Ccl12/Ifi44/Usp18/Oas1a/Ch25h/Oasl1/Tgm1/Cxcl1
ONDER_CDH1_TARGETS_2_DN	ONDER_CDH1_TARGETS_2_DN	19	-0.537799966	-1.85633456	0.005387526	0.147230177	0.130344623	41	tags=37%, list=13%, signal=34%	Isg20/Cxcl1/S100a8/S100a9/Sprr1b/Lcn2/Sprr1a
THUM_SYSTOLIC_HEART_FAILURE_UP	THUM_SYSTOLIC_HEART_FAILURE_UP	11	-0.633871175	-1.852539574	0.005628518	0.147230177	0.130344623	79	tags=73%, list=25%, signal=56%	Ccl12/Ifi204/Il2rg/Fcgr4/Oasl1/Cxcl1/S100a9/Selp
JAEGER_METASTASIS_DN	JAEGER_METASTASIS_DN	8	-0.69932956	-1.83106695	0.00612	0.147313544	0.130418429	27	tags=62%, list=9%, signal=59%	S100a8/S100a9/Ltf/Sprr1b/Sprr1a
BLANCO_MELO_COVID19_SARS_COV_2_INFECTION_CALU3_CELLS_UP	BLANCO_MELO_COVID19_SARS_COV_2_INFECTION_CALU3_CELLS_UP	28	-0.470067887	-1.802035439	0.006224516	0.147313544	0.130418429	150	tags=89%, list=48%, signal=51%	Nr4a3/Ddx58/Rnf213/Parp10/Eif2ak2/Samd9l/Helz2/Dtx3l/Ddx60/Slfn5/Ptgs2/Rtp4/Ddx58/Irf7/Isg15/Oas2/Phf11d/Ifi44/Usp18/Oas1a/Ch25h/Isg20/Oasl1/Cxcl1/Sele
HECKER_IFNB1_TARGETS	HECKER_IFNB1_TARGETS	16	-0.557432432	-1.827396669	0.006718346	0.151773549	0.134366925	148	tags=100%, list=47%, signal=55%	Ifi27/Eif2ak2/Samd9l/Dtx3l/Ddx60/Slfn5/Ptgs2/Rtp4/Ddx58/Irf7/Isg15/Oas2/Ccl12/Siglec1/Ifi44
MARKEY_RB1_ACUTE_LOF_DN	MARKEY_RB1_ACUTE_LOF_DN	14	-0.579562817	-1.822415322	0.007197974	0.155538834	0.137700376	90	tags=71%, list=29%, signal=53%	Irf7/Ifi27l2a/Isg15/Ccl12/Ifi44/Usp18/Ch25h/Isg20/Slfn4/Sprr1a
LENAOUR_DENDRITIC_CELL_MATURATION_DN	LENAOUR_DENDRITIC_CELL_MATURATION_DN	12	-0.603328966	-1.805291427	0.008439745	0.155994088	0.138103418	92	tags=83%, list=29%, signal=61%	Ifi2bcb/Irf7/Isg15/Il2rg/Bst1/Cxcl5/Lgals3/Cxcl1/S100a8/S100a9
REACTOME_INTERFERON_ALPHA_BETA_SIGNALING	REACTOME_INTERFERON_ALPHA_BETA_SIGNALING	9	-0.659554681	-1.799556114	0.008611753	0.155994088	0.138103418	94	tags=89%, list=30%, signal=64%	Ifitm3/Irf7/Isg15/Oas2/Usp18/Oas1a/Isg20/Oasl1
ICHIBA_GRAFT_VERSUS_HOST_DISEASE_35D_UP	ICHIBA_GRAFT_VERSUS_HOST_DISEASE_35D_UP	10	-0.636621051	-1.796944041	0.008788399	0.155994088	0.138103418	32	tags=50%, list=10%, signal=46%	Cxcl1/S100a8/S100a9/Slfn4/Ltf
BENNETT_SYSTEMIC_LUPUS_ERYTHEMATOSUS	BENNETT_SYSTEMIC_LUPUS_ERYTHEMATOSUS	7	-0.718032787	-1.793670278	0.007764193	0.155994088	0.138103418	94	tags=100%, list=30%, signal=71%	Irf7/Isg15/Oas2/Oas1a/Oasl1/S100a8

LEE_AGING_CEREBELLUM_UP	LEE_AGING_CEREBELLUM_UP	61	0.677053792	1.826612447	0.001188671	0.10184415	0.093732112	1245	tags=36%, list=11%, signal=32%	Cst7/Gfap/Itgb5/C4b/Slc11a1/Ifit1/Fcrls/Mpeg1/Lgals3bp/Ctsh/Cd68/C1qc/Trim12c/Ifi27/B2m/C1qb/Hexb/Selplg/Ctsz/Axl/C1qa/Hmox1
LENAOUR_DENDRITIC_CELL_MATURATION_DN	LENAOUR_DENDRITIC_CELL_MATURATION_DN	85	0.631420867	1.785340895	0.001195478	0.10184415	0.093732112	979	tags=28%, list=8%, signal=26%	Atp2a3/Isg15/S100a4/Emp3/Slc11a1/Chil1/Il6ra/Fcgr2b/Lyz2/Cd180/Cd14/Fyb/Emp1/Dok1/Pdlim7/Stab1/Csf1r/Ptafr/Lgmn/Lst1/Fcer1g/
WP_TYPE_II_INTERFERON_SIGNALING_IFNG	WP_TYPE_II_INTERFERON_SIGNALING_IFNG	16	0.861732041	1.869696226	0.001284384	0.10192379	0.093805409	1180	tags=75%, list=10%, signal=68%	Irf1/Isg15/Cxcl10/Icam1/H2-D1/Ptpn11/Eif2ak2/Irf8/Ifit
SANA_TNF_SIGNALING_UP	SANA_TNF_SIGNALING_UP	47	0.708350765	1.837641668	0.001265845	0.10192379	0.093805409	1477	tags=45%, list=13%, signal=39%	Mx2/Cxcl10/Atp13a3/Parp14/Ifih1/Lgals3bp/Slc15a3/Bst2/Icam1/H2-D1/Pdgfrl/Samd9l/Icosl/Ccl12/Tlr2/Tnfaip3/H2-T23/Sat1/Tapbp/Vcam1/Csf
CHICAS_RB1_TARGETS_CONFLUENT	CHICAS_RB1_TARGETS_CONFLUENT	365	0.453193325	1.512361946	0.001272185	0.10192379	0.093805409	1792	tags=27%, list=15%, signal=23%	Efemp2/Vim/S100a4/Emp3/Synpo2/Jdp2/Rarres1/Parp9/Nuf2/Hspb6/S100a6/Gbp2/Nupr1/Ccdc80/Olfml3/Depp1/Lipa/Tent5c/Ctsh/Capg/Arhgap18/4930503L19Rik/Tmem176a/Tspo/Pdgfrl/Ifitm3/Scara3/Col1a1/Apcdd1/Prss12/Samd9l/Pdlim7/Uaca/Shmt2/Kctd16/Lmna/Epb41l2/Lims2/Ica1/Slc14a1/Cnksr3/Lxn/Adgra2/Jade1/Zyx/Loxl1/Serping1/Ifit2/Cd9/Dtx3l/Col5a1/Ptn/Cmbl/Prdx6/Clu/Mdk/Tgfbr2/Phgdh/Anxa2/Rbp1/Nes/Axl/Cnn3/Hpgd/Pxk/Evl/Galnt15/Vsir/Plin3/Prkag2/Cbs/Ak1/Vcam1/Nt5dc2/Serpinh1/Col1a2/Flna/Nipsnap3b/Bmp4/Melk/Bgn/Ifitm2/Frmd5/Synpo/Notch2/Sulf1/Prim1/Myh9/Sipa1l2/Matn2/Mtus1/Tmem47/Pck2/Angptl2/Gsn/Fk
KEGG_CYTOKINE_CYTOKINE_RECEPTOR_INTERACTION	KEGG_CYTOKINE_CYTOKINE_RECEPTOR_INTERACTION	77	0.632427645	1.763961068	0.001574426	0.120008539	0.11044968	2105	tags=52%, list=18%, signal=43%	Cxcl10/Il11ra1/Ccl9/Fas/Il6ra/Ccl6/Tnfrsf13b/Csf3r/Ccl3/Il21r/Osmr/Il10ra/Cntf/Il10rb/Crlf2/Csf1r/Ccl12/Csf2ra/Acvr1/Cx3cr1/Prl/Tgfb1/Tnfrsf4/Tnfrsf1a/Ccl25/Tgfbr2/Ifngr1/Csf1/Tgfbr1/Bmpr1b/Ltbr/Tnfrsf1b/Tnfrsf11a/Bmp2/Gh/Il1rap/Egfr/Il13ra
REACTOME_INTERFERON_ALPHA_BETA_SIGNALING	REACTOME_INTERFERON_ALPHA_BETA_SIGNALING	32	0.762159192	1.86358448	0.001602983	0.120598444	0.110992599	1025	tags=53%, list=9%, signal=49%	Irf1/Isg15/Mx2/Ifitm1/Usp18/Gbp2/Bst2/Ifit3/H2-D1/Ptpn11/Ifitm3/Ifi35/Irf8/
REACTOME_CYTOKINE_SIGNALING_IN_IMMUNE_SYSTEM	REACTOME_CYTOKINE_SIGNALING_IN_IMMUNE_SYSTEM	399	0.437821126	1.471925422	0.001717596	0.122839921	0.113055539	1477	tags=22%, list=13%, signal=20%	Nfkb1/Irf1/Vim/Isg15/Mx2/Cxcl10/Il11ra1/Gbp5/Ifitm1/Usp18/Cd86/H3c8/Sla/Il6ra/Pomc/Gbp2/Tnfrsf13b/Gsdmd/Bst2/Ifit3/Csf3r/Ccl3/Il21r/Trim68/Ptpn7/Il18bp/Icam1/H2-D1/Ptpn11/Ube2l6/Osmr/Il10ra/Cntf/Ifitm3/Fcgr1/Il10rb/Crlf2/Eif2ak2/Psme2/Ifi35/Irf8/Csf1r/Irf5/Ddx58/Trim12c/Csf2ra/Ifi27/Ptafr/Mef2c/Lbp/Gbp7/Trim26/Lyn/Havcr2/B2m/Stat6/Mmp2/Ebi3/Msn/Vav1/Prl/Ifit2/Tgfb1/Mt2/Tnfrsf4/Nod1/Cdkn1b/Tnfrsf1a/H2-T23/Jak2/Ifi30/Anxa2/Fpr1/Ifngr1/Hmox1/Mapkapk3/S1pr1/Itgb2/H3c15/Psmd4/N
KIM_GLIS2_TARGETS_UP	KIM_GLIS2_TARGETS_UP	51	0.685925444	1.800974473	0.001884281	0.128518836	0.11828212	808	tags=43%, list=7%, signal=40%	Efemp2/Irf1/Vim/Isg15/Cxcl10/S100a4/Ccl9/S100a6/Fcgr2b/Cd74/H2-DMa/Icam1/Pycard/Osmr/Pdgfrl/Ifitm3/Col1a1/Ccl12/I
XU_AKT1_TARGETS_6HR	XU_AKT1_TARGETS_6HR	17	0.84244672	1.85041393	0.002029662	0.128697938	0.118446956	320	tags=29%, list=3%, signal=29%	Isg15/Mx2/Soat1/Ifih1/Ifit3
RADAEVA_RESPONSE_TO_IFNA1_UP	RADAEVA_RESPONSE_TO_IFNA1_UP	30	0.763367896	1.847200405	0.002043882	0.128697938	0.118446956	1121	tags=47%, list=10%, signal=42%	Irf1/Isg15/Mx2/Cxcl10/Ifitm1/Lgals3bp/Bst2/Ube2l6/Ifitm3/Ifi35/Ifi27/Cdkn1b/H
HUANG_GATA2_TARGETS_UP	HUANG_GATA2_TARGETS_UP	107	0.581953009	1.69529921	0.002015105	0.128697938	0.118446956	1193	tags=28%, list=10%, signal=25%	Ifi204/Emp3/Akr1b8/Ifitm1/Ccl9/Cmtm7/Lgals1/Fes/Fcgr2b/Myo1f/Lyz2/Depp1/Gmfg/Neurl3/Alox5ap/Tspo/Samsn1/Pik3cg/Tyrobp/Dok3/B2m/Cebpa/Cdkn1b/Selplg/H2-T23/Tgfbr2/Dynlt1b/Spi1/A
REACTOME_ADAPTIVE_IMMUNE_SYSTEM	REACTOME_ADAPTIVE_IMMUNE_SYSTEM	472	0.420251727	1.432527141	0.002013371	0.128697938	0.118446956	1740	tags=18%, list=15%, signal=16%	Snap23/Nfkb1/Npdc1/Mapkap1/Btk/Spsb2/Itgb5/Ifitm1/Cd86/Dapp1/Lag3/Kbtbd8/Fcgr4/Dnm2/Fcgr2b/Was/Cd74/H2-DMa/Pik3ap1/Cd14/Ctsh/Rnf213/Ly96/Fyb/Trem2/Lcp2/Icam1/H2-Oa/H2-D1/Ptpn11/Pja2/Ube2l6/Fcgr1/Herc4/Cdc20/Col1a1/Xdh/Psme2/Icosl/Ncf2/Tlr2/Tyrobp/Lgmn/Skp2/Plcg2/Lyn/B2m/Ube2cbp/Rnf111/Ube2k/Vav1/Icam2/Trim9/Cyba/Dtx3l/S100a1/H2-T23/Ifi30/Lair1/Trim36/Tapbp/Evl/Ctsc/Asb6/Tirap/Itgb2/Psmd4/Myd88/Ctsl/Vcam1/H2-DMb1/Spsb1/Rel/Col1a2/Pja1/Rnf182/Vamp8/Herc6/Fbxw4/Rela/Map3k7/Rasgrp2/Klhl3/Ctsa/Prkcq/Itpr1/Ct
PLASARI_TGFB1_TARGETS_10HR_DN	PLASARI_TGFB1_TARGETS_10HR_DN	157	0.530170773	1.619232051	0.002272614	0.14156187	0.130286257	1669	tags=32%, list=14%, signal=28%	Rtp4/Prickle2/Ccl9/Usp18/Parp9/Nuf2/Ifit1/Gbp2/Igfbp5/Ifit3/Arhgap18/Gulp1/Scara3/Mgst1/Zfhx4/Serhl/Eif2ak2/Ccl12/Rfc3/Mki67/Ednrb/Mef2c/Slc14a1/Cnksr3/Celf2/Ifit2/Zfp467/Ptprd/Rspo3/Vwa5a/Ppp1r3c/C3ar1/Aqp1/Thbd/Serpinb1a/Foxc1/Mt1/Eya4/Serpinb9/Tmem86a/Ddit4l/Pdlim2/Vcam1/Foxp2/Pdk4/Reps2/Plpp3/Herc6/Prim1/Mpped2/Palm
MARTINEZ_RB1_AND_TP53_TARGETS_UP	MARTINEZ_RB1_AND_TP53_TARGETS_UP	423	0.427238623	1.443343267	0.002543109	0.156725843	0.144242397	2116	tags=26%, list=18%, signal=22%	Pcx/Nsun4/Ifi204/Pbk/Ifi27l2a/Ndst2/Art3/Akr1b8/Ccl9/Lgals1/Pld1/Rarres2/Dnm2/Fcgr2b/Gbp2/Lyz2/Lgals3bp/Cd74/Prdx1/Uty/Lipa/Uap1/Git2/Alox5ap/H2-D1/Tspo/Pigo/Fermt3/Xdh/Stom/Eri2/Csf1r/Ncf2/Pon3/Gja1/Tyrobp/Skp2/Lbp/Slc14a1/Havcr2/B2m/Lxn/Msra/Tm6sf1/Celf2/Ide/Prkd3/C1qb/Ropn1l/Hmgcs2/Aqp1/Chd9/H2-T23/Serpinb1a/Tgfbr2/Ap3m1/Npm3/Prr13/Litaf/Trf/Emp2/Pdlim5/Asb6/Serpinb9/Bmp1/Hadh/Sod3/Rfc1/Tcf12/Lman1/Dusp22/Pdk4/Casp6/Fuca1/Virma/Ncoa4/Triap1/Ephx2/Sox4/Ccn1/Gna12/Slc25a10/Bphl/Hbs1l/Ccn3/Rgcc/Angptl2/Gna13/Parp3/Cldn5/Tgfbi/Ndrg1/Zc3h11a/Me1/Cep85/Cldn11/Klf3/H1f2/Egfr/Fads2/Selenof/I
KATSANOU_ELAVL1_TARGETS_UP	KATSANOU_ELAVL1_TARGETS_UP	110	0.572548852	1.674211247	0.002621786	0.158382546	0.145767141	1785	tags=40%, list=15%, signal=34%	Slc7a7/Fas/Slamf9/Nupr1/Mpeg1/Lyz2/Adamts10/Zfp458/Plod2/Cd52/Ctsh/Stom/Psme2/Kif21b/Igfbp2/C1qc/Trim12c/Zfp58/Mmp2/Ddah2/Serping1/C1qb/Ptp4a3/Fjx1/Ccl25/Cmbl/C3ar1/Lpcat2/Pgpep1/Zfp708/Foxc1/Nes/C1qa/Sat1/Ctsc/Plekha2/Emcn/Spsb1/Bmp4/Itpripl2/Lama5/Palmd/Aga/Pierce1
YANAGIHARA_ESX1_TARGETS	YANAGIHARA_ESX1_TARGETS	24	-0.793399993	-1.884010693	0.002769567	0.158838262	0.146186558	16	tags=12%, list=0%, signal=13%	Pclo/Pip5k1a/Tpr
TAVOR_CEBPA_TARGETS_DN	TAVOR_CEBPA_TARGETS_DN	21	0.803213751	1.831882804	0.003153185	0.158838262	0.146186558	1029	tags=52%, list=9%, signal=48%	Isg15/Itgb5/Plod2/A2m/Tbxas1/Cradd/Ifi27/Gja1/Icam2
KEGG_CITRATE_CYCLE_TCA_CYCLE	KEGG_CITRATE_CYCLE_TCA_CYCLE	26	0.773036796	1.827538643	0.003047552	0.158838262	0.146186558	303	tags=8%, list=3%, signal=8%	Pcx/Acly
WP_COMPLEMENT_SYSTEM	WP_COMPLEMENT_SYSTEM	34	0.733728004	1.811122889	0.002994898	0.158838262	0.146186558	2040	tags=59%, list=17%, signal=49%	Lamb1/C4b/Fcgr4/Was/Rps19/Icam1/Tlr2/Pros1/Icam2/Serping1/C3ar1/Selplg/Fpr1/Itgb2/Gna15/Lama5/Fkbp2
LEIN_ASTROCYTE_MARKERS	LEIN_ASTROCYTE_MARKERS	39	0.714963894	1.802894214	0.002858727	0.158838262	0.146186558	2019	tags=56%, list=17%, signal=47%	Gfap/Itgb5/Lyz2/Aqp4/Stab1/Gja1/Gldc/Id3/Emx2/Prdx6/Clu/C1qa/Mt1/Cd63/S1pr1/Plpp3/Dbi/Gsn/Dip2a/Cld
EINAV_INTERFERON_SIGNATURE_IN_CANCER	EINAV_INTERFERON_SIGNATURE_IN_CANCER	11	0.887203731	1.792016481	0.003066102	0.158838262	0.146186558	1066	tags=73%, list=9%, signal=66%	Isg15/Mx2/Bst2/Ifit3/Ube2l6/Eif2ak2/Ifi35/Ifi30
TSAI_RESPONSE_TO_IONIZING_RADIATION	TSAI_RESPONSE_TO_IONIZING_RADIATION	85	0.602832265	1.704506695	0.003108242	0.158838262	0.146186558	1180	tags=19%, list=10%, signal=17%	Ranbp1/Nfkb1/Cxcl10/Ifitm1/Fas/Zfp458/Ccl3/Ptpn11/Plcg2/Tnfaip3/Grn/Plk1/Ptn

JINESH_BLEBBISHIELD_TRANSFORMED_STEM_CELL_SPHERES_UP	JINESH_BLEBBISHIELD_TRANSFORMED_STEM_CELL_SPHERES_UP	7	-0.71602829	-1.788662978	0.008004322	0.155994088	0.138103418	63	tags=71%, list=20%, signal=58%	Hspa1b/Oas1a/S100a9/Lcn2/Sprr1a
ZHENG_IL22_SIGNALING_UP	ZHENG_IL22_SIGNALING_UP	7	-0.706296914	-1.764353671	0.010165489	0.174215456	0.154235011	49	tags=86%, list=16%, signal=74%	Ccl12/Cxcl5/Tgm1/Cxcl1/S100a8/S100a9
SMID_BREAST_CANCER_RELAPSE_IN_BONE_DN	SMID_BREAST_CANCER_RELAPSE_IN_BONE_DN	5	-0.766082164	-1.700736615	0.011231448	0.186067656	0.164727905	49	tags=80%, list=16%, signal=69%	Cxcl5/Lgals2/S100a8/S100a9
TAKEDA_TARGETS_OF_NUP98_HOXA9_FUSION_3D_UP	TAKEDA_TARGETS_OF_NUP98_HOXA9_FUSION_3D_UP	20	-0.498248868	-1.746962085	0.012142546	0.188588923	0.166960013	148	tags=95%, list=47%, signal=53%	Ddx58/Rnf213/Parp10/Ifi27/Eif2ak2/Samd9l/Helz2/Dtx3l/Ddx60/Slc5a3/Ptgs2/Irf7/Isg15/Oas2/Ifi44/Usp18/Oas1a/Ch25h/Oasl1
UROSEVIC_RESPONSE_TO_IMIQUIMOD	UROSEVIC_RESPONSE_TO_IMIQUIMOD	6	-0.72875817	-1.724717145	0.011962107	0.188588923	0.166960013	90	tags=100%, list=29%, signal=73%	Isg15/Oas2/Ccl12/Isg20/Oasl1
BLANCO_MELO_BRONCHIAL_EPITHELIAL_CELLS_INFLUENZA_A_DEL_NS1_INFECTION_UP	BLANCO_MELO_BRONCHIAL_EPITHELIAL_CELLS_INFLUENZA_A_DEL_NS1_INFECTION_UP	27	-0.449093522	-1.70598262	0.012887207	0.194089154	0.171829432	148	tags=89%, list=47%, signal=51%	Ddx58/Rnf213/Parp10/Ifi27/Eif2ak2/Samd9l/Helz2/Dtx3l/Angptl4/Fas/Rtp4/Ifitm3/Dhx58/Irf7/Ankk1/Oas2/Phf11d/Hspa1b/Ifi44/Usp18/Oas1a/Cxcl5/Oasl1/Cxcl1

NABA_MATRISOME	NABA_MATRISOME	377	0.434692094	1.454327096	0.002759572	0.158838262	0.146186558	1893	tags=33%, list=16%, signal=28%	Efemp2/Cst7/Lamb1/S100a3/Nrg2/Cxcl10/S100a4/Wnt7b/Serpina3n/Anxa4/P4ha3/Gpc5/Ccl9/Clec4a3/Thbs4/Lgals1/Sema4g/Ogfod1/S100a6/Ccl6/Sema4b/Igfbp5/Col20a1/Cela1/Adamts10/Plod2/Pamr1/Ctsh/Ccl3/Plxnb2/Clec5a/Emid1/Papln/A2m/Efemp1/P3h2/Cntf/Col1a1/S100a11/Prss12/Col24a1/Sema4a/Loxl3/Crhbp/Htra3/Ccl12/Igfbp2/C1qc/Col27a1/Vwa7/Col4a1/Bmp6/Mmp2/Impg1/Ebi3/Prl/S100a13/Loxl1/Aspn/Serping1/C1qb/Tgfb1/Crlf3/Thpo/Aebp1/Col5a1/Col9a3/Ptn/Rspo3/Vwa5a/Ccl25/Frem2/S100a1/Serpinb1a/Lgi4/Mdk/Gdf10/Pik3ip1/Anxa2/Mfap3/Ctsz/Angpt1/C1qa/Mmp14/Tspear/Ctsc/Emcn/Sema4d/Serpinb9/Bmp1/Anxa3/Ky/Wnt4/Spock3/Ctsl/Lman1/Vwa1/Pl
GAURNIER_PSMD4_TARGETS	GAURNIER_PSMD4_TARGETS	21	0.801251796	1.827408191	0.003346956	0.158925427	0.146266781	1492	tags=81%, list=13%, signal=71%	Nfkb1/Cxcl10/Ccl9/Cd86/H2-DMa/Ccl3/Il21r/H2-Oa/H2-D1/Ccl12/B2m/Tgfb1/C3ar1/H2-T23/Ifngr1/H2-
VILIMAS_NOTCH1_TARGETS_DN	VILIMAS_NOTCH1_TARGETS_DN	14	0.856496835	1.811335953	0.003241868	0.158925427	0.146266781	630	tags=71%, list=5%, signal=68%	Btk/Lrrc25/Ccl9/Lyz2/Csf3r/Trem2/Stom/Csf1r/Ncf2/Cs
DER_IFN_ALPHA_RESPONSE_UP	DER_IFN_ALPHA_RESPONSE_UP	41	0.703973194	1.790004057	0.003257273	0.158925427	0.146266781	1066	tags=37%, list=9%, signal=33%	Irf1/Isg15/Mx2/Ifitm1/Fas/Lipa/Bst2/Plod2/Ifit3/Ifi35/Trim26/B2m/H2-
WP_TOLLLIKE_RECEPTOR_SIGNALING_PATHWAY	WP_TOLLLIKE_RECEPTOR_SIGNALING_PATHWAY	55	0.660945734	1.754178185	0.003407126	0.158925427	0.146266781	1373	tags=31%, list=12%, signal=27%	Nfkb1/Cxcl10/Cd86/Tlr3/Cd14/Ly96/Ccl3/Tlr7/Tlr9/Pik3cg/Irf5/Tlr2/Lbp/Ripk1/Pi
LU_AGING_BRAIN_UP	LU_AGING_BRAIN_UP	206	0.488943839	1.541046968	0.003339991	0.158925427	0.146266781	2202	tags=32%, list=19%, signal=26%	Snap23/Rdx/Gfap/Vim/Gpc5/Nupr1/Zfp423/Plod2/Fxyd1/Efemp1/Ryr1/Zeb2/Stom/Mtrr/Gja1/Slc14a1/Wwtr1/Msn/Icam2/Wipi1/Mt2/Prdx6/Nfe2l2/Tgfbr2/Lrp4/Litaf/Mt1/Pdlim5/Mlc1/S1pr1/Anxa3/Cbs/Pdlim3/Gpr137b/Serpinh1/Septin10/Sh3glb1/Tst/Nkx2-2/Tax1bp3/Lamp2/Rhobtb3/Apod/Tpd52l2/Ptpn13/Tmem47/Gsn/Creb3l2/Cryab/Pon2/Tle4/Cldn5/Leprot/Cfh/Ndrg1/Iqgap1/Rassf2/Tln1/Myo10/Hspa2/Podxl/Cav2/
RUTELLA_RESPONSE_TO_HGF_UP	RUTELLA_RESPONSE_TO_HGF_UP	286	0.458376511	1.494621548	0.003268655	0.158925427	0.146266781	1941	tags=27%, list=17%, signal=23%	Tcf3/Rdx/Slc43a3/Cxcl10/Atp13a3/Ccl9/Rarres1/Chil1/Apoo/Ccl6/Prdx1/Ccl3/Apoc1/Clec5a/Il21r/Emp1/Fcgr1/Stom/Calhm2/Tcirg1/Ccl12/Csf2ra/Epb41l2/Slc1a2/Lxn/Mmp2/Ebi3/Tm6sf1/Hk2/Cd9/Lpar1/Pltp/Lhfpl2/Mt2/Hexb/Rgl1/Tnfrsf4/Vwa5a/Tpst1/C3ar1/Anxa2/Lmbr1l/Fzd5/Lair1/C1qa/Litaf/Mt1/Cd63/Dhrs4/Gpd2/Mctp1/Slc29a3/Gpr137b/Ctsl/Slc7a11/Npl/Scamp2/Ncoa4/Plpp3/Entpd1/Lamp2/Gna12/Rab13/Pck2/Itpr1/Acsl3/Mdm1/St3gal1/Sun2/Ndp/Leprot/Pl
LIAN_LIPA_TARGETS_6M	LIAN_LIPA_TARGETS_6M	36	0.718197939	1.788423685	0.003754693	0.168611928	0.155181737	1245	tags=56%, list=11%, signal=50%	Slc11a1/Fcgr4/Fcgr2b/Mpeg1/Slc15a3/Tlr7/Trem2/Apobec1/Cd68/Igsf6/Kif1a/C1qc/Slc37a2/Fcer1g/C1qb/Mafb/C3ar1/Fabp7/C1qa/Hmox
NABA_ECM_REGULATORS	NABA_ECM_REGULATORS	88	0.591573801	1.679591214	0.003808841	0.169727797	0.156208726	1922	tags=40%, list=16%, signal=33%	Cst7/Serpina3n/P4ha3/Ogfod1/Cela1/Adamts10/Plod2/Pamr1/Ctsh/A2m/P3h2/Prss12/Loxl3/Htra3/Mmp2/Loxl1/Serping1/Serpinb1a/Ctsz/Mmp14/Ctsc/Serpinb9/Bmp1/Ky/Ctsl/Serpinh1/Sulf1/Ctsa/Ctso/Adam11/Plod3/Fam20a/Mmp23/Adam17/Plod1
KYNG_ENVIRONMENTAL_STRESS_RESPONSE_NOT_BY_UV_IN_OLD	KYNG_ENVIRONMENTAL_STRESS_RESPONSE_NOT_BY_UV_IN_OLD	19	-0.816226467	-1.866905438	0.004044861	0.175856312	0.161849095	132	tags=16%, list=1%, signal=16%	Glrx/Prss23/Tpr
WP_TCA_CYCLE_AND_DEFICIENCY_OF_PYRUVATE_DEHYDROGENASE_COMPLEX_PDHC	WP_TCA_CYCLE_AND_DEFICIENCY_OF_PYRUVATE_DEHYDROGENASE_COMPLEX_PDHC	14	0.850467905	1.798585855	0.004191145	0.176120251	0.162092012	303	tags=14%, list=3%, signal=14%	Pcx/Acly
REACTOME_INTERFERON_GAMMA_SIGNALING	REACTOME_INTERFERON_GAMMA_SIGNALING	42	0.691316195	1.764132887	0.004225913	0.176120251	0.162092012	1416	tags=52%, list=12%, signal=46%	Irf1/Gbp5/Gbp2/Trim68/Icam1/H2-D1/Ptpn11/Fcgr1/Irf8/Irf5/Trim12c/Ptafr/Gbp7/Trim26/B2m/Mt2/H2-T23/Jak2/Ifi30/Ifngr1/Vcam
PEDRIOLI_MIR31_TARGETS_UP	PEDRIOLI_MIR31_TARGETS_UP	105	0.566326797	1.645638958	0.004217551	0.176120251	0.162092012	1536	tags=23%, list=13%, signal=20%	Coro2a/Taf5l/1700020L24Rik/Olfml3/Zfp366/Zfp667/P3h2/Phactr3/Gldc/Vsig10l/Rgma/Dok3/Nppc/Fam181a/Stk11ip/Nwd2/Zfp740/Cdnf/Fzd5/Opalin/Man2b2/Hs6
RASHI_RESPONSE_TO_IONIZING_RADIATION_6	RASHI_RESPONSE_TO_IONIZING_RADIATION_6	52	0.658516655	1.733244789	0.004446059	0.182666811	0.168117128	1586	tags=37%, list=14%, signal=32%	Emp3/Ccl9/Fas/Ifit1/Fcgr2b/Gbp2/Lyz2/Fermt3/Ccl12/Csf2ra/Tyrobp/Zfp688/Ptp4a3/Plekho1/Selplg/Acin1/Arntl/Unc93b1/D030056L22R
NAGY_STAGA_COMPONENTS_HUMAN	NAGY_STAGA_COMPONENTS_HUMAN	12	0.865445695	1.778941704	0.004528058	0.184725625	0.170011955	24	tags=17%, list=0%, signal=17%	Taf5l/Taf6l
WP_OVERVIEW_OF_PROINFLAMMATORY_AND_PROFIBROTIC_MEDIATORS	WP_OVERVIEW_OF_PROINFLAMMATORY_AND_PROFIBROTIC_MEDIATORS	21	0.79057523	1.803058237	0.004668123	0.188258436	0.173263372	968	tags=48%, list=8%, signal=44%	Nfkb1/Cxcl10/Ccl9/Ccl6/Ccl3/Cntf/Ccl12/Ebi3/Tgfb1/Ccl25
KAYO_CALORIE_RESTRICTION_MUSCLE_DN	KAYO_CALORIE_RESTRICTION_MUSCLE_DN	64	0.630478736	1.711825358	0.00467965	0.188258436	0.173263372	1822	tags=25%, list=16%, signal=21%	Ranbp1/Nnt/Acr/H2-DMa/Rcan1/Bmp6/Commd1/Kat5/Dynlt1b/Ddo/Hadh/Dbi/Gli3/Pcdhgb7/Vldlr/Cy
KYNG_DNA_DAMAGE_BY_4NQO_OR_GAMMA_RADIATION	KYNG_DNA_DAMAGE_BY_4NQO_OR_GAMMA_RADIATION	9	-0.902703918	-1.802200255	0.004815977	0.189788795	0.174671836	9	tags=22%, list=0%, signal=22%	Ube2n/Dctd
NABA_MATRISOME_ASSOCIATED	NABA_MATRISOME_ASSOCIATED	265	0.456157317	1.475614088	0.0048672	0.1905114	0.175336884	1504	tags=28%, list=13%, signal=25%	Cst7/S100a3/Nrg2/Cxcl10/S100a4/Wnt7b/Serpina3n/Anxa4/P4ha3/Gpc5/Ccl9/Clec4a3/Lgals1/Sema4g/Ogfod1/S100a6/Ccl6/Sema4b/Cela1/Adamts10/Plod2/Pamr1/Ctsh/Ccl3/Plxnb2/Clec5a/A2m/P3h2/Cntf/S100a11/Prss12/Sema4a/Loxl3/Crhbp/Htra3/Ccl12/C1qc/Bmp6/Mmp2/Ebi3/Prl/S100a13/Loxl1/Serping1/C1qb/Tgfb1/Crlf3/Thpo/Ptn/Ccl25/Frem2/S100a1/Serpinb1a/Mdk/Gdf10/Pik3ip1/Anxa2/Ctsz/Angpt1/C1qa/Mmp14/Ctsc/Emcn/Sema4d/Serpinb9/Bmp1/Anxa3/Ky/Wnt4/Ctsl/Lman1/Plx
HELLER_SILENCED_BY_METHYLATION_UP	HELLER_SILENCED_BY_METHYLATION_UP	136	0.526680969	1.582092952	0.005065958	0.195647289	0.180063692	2061	tags=49%, list=18%, signal=41%	Cst7/Isg15/Mx2/Cxcl10/S100a4/Zer1/Ifitm1/Usp18/Cd86/Dapp1/Top2a/H3c8/Chil1/Il6ra/S100a6/Ifih1/H2-DMa/Ifit3/Ctsh/Capg/Apoc1/Icam1/Cdc20/Samsn1/Crybb1/Ddx58/Ncf2/Ifi27/Mical1/Cx3cr1/Ebi3/Ddah2/S100a13/Plk1/Mafb/Tnfrsf4/Septin9/Clu/Tspan15/Sat1/Dennd5a/Bmp2k/H2bc4/Vcam1/H2-DMb1/Enpp2/Rel/Klf4/Ephx2/Vamp8/Zbtb43/Synpo/Herc6/Upb1/Smarca1/Rab13/Tnfrsf1b/Tnfrsf11a/Cdc42ep4/Ccnf/Olfml2b/Wls/Trip6/
PID_MYC_ACTIV_PATHWAY	PID_MYC_ACTIV_PATHWAY	61	-0.632280838	-1.736778235	0.005196167	0.198035505	0.182261683	400	tags=7%, list=3%, signal=6%	Eif4a1/Peg10/Mta1/Rcc1
DER_IFN_BETA_RESPONSE_UP	DER_IFN_BETA_RESPONSE_UP	62	0.632199889	1.709947006	0.005165556	0.198035505	0.182261683	1066	tags=27%, list=9%, signal=25%	Irf1/Isg15/Mx2/Ifitm1/Lipa/Bst2/Plod2/Ifit3/Ptpn11/Eif2ak2/Ifi35/Trim26/B2m/Ifit
KEGG_TOLL_LIKE_RECEPTOR_SIGNALING_PATHWAY	KEGG_TOLL_LIKE_RECEPTOR_SIGNALING_PATHWAY	55	0.646238459	1.715144451	0.005257696	0.199070815	0.183214529	1053	tags=27%, list=9%, signal=25%	Nfkb1/Cxcl10/Cd86/Tlr3/Cd14/Ly96/Ccl3/Tlr7/Tlr9/Pik3cg/Irf5/Tlr2/Lbp/Ripk1/Pi
ZHOU_INFLAMMATORY_RESPONSE_LPS_UP	ZHOU_INFLAMMATORY_RESPONSE_LPS_UP	182	0.515161379	1.601098206	0.001649077	0.122475644	0.112720277	1513	tags=21%, list=13%, signal=19%	Nfkb1/Rdx/Rtp4/Isg15/Cxcl10/Ccl9/Usp18/Rpe65/Ccl6/Ifih1/Anks6/Ifit3/Ccl3/Tlr7/Aqp4/Icam1/Slfn5/Irf8/Ccl12/Ddx58/C1qc/Tlr2/Slc1a2/Gpr84/Kif1b/Cobl/Ifit2/Ripk1/Trim36/Dennd5a/Litaf/Pdlim5/Cldn1/Foxp2/Spsb1/Rel/Klf4/Tent2/Plpp3
WP_TYROBP_CAUSAL_NETWORK_IN_MICROGLIA	WP_TYROBP_CAUSAL_NETWORK_IN_MICROGLIA	38	0.722465004	1.814960156	0.002670273	0.158838262	0.146186558	2078	tags=76%, list=18%, signal=63%	Nrros/Slc7a7/Capg/Pycard/Il10ra/Igsf6/Nckap1l/Samsn1/Loxl3/Tcirg1/Ncf2/C1qc/Tyrobp/Adap2/Lhfpl2/Maf/Hcls1/Apbb1ip/Itgb2/Sh2b3/Tgfbr1/Abcc4/Lyl1/Npc2/Tnfrsf1b/Creb3l2/Fkbp15/Il1
ICHIBA_GRAFT_VERSUS_HOST_DISEASE_D7_UP	ICHIBA_GRAFT_VERSUS_HOST_DISEASE_D7_UP	72	0.794211193	2.192482612	1.7168E-05	0.007103861	0.006538028	1433	tags=61%, list=12%, signal=54%	Ifi204/Irf1/Isg15/Cxcl10/Serpina3n/Slc11a1/Ccl9/Usp18/Fas/Ifit1/Gbp2/Mpeg1/Lyz2/Lgals3bp/Cd74/H2-DMa/Ifit3/Cd14/Lcp2/Il18bp/Icam1/Pld4/Ly86/Xdh/Psme2/Ifi35/Cd53/Irf8/Clic1/C1qc/Ifi27/Tyrobp/Lbp/Col4a1/Gbp7/C1qb/Cyba/Krt18/Tapbp/Litaf/Mt1/Ptgs1/Vcam

HOLLERN_EMT_BREAST_TUMOR_UP	HOLLERN_EMT_BREAST_TUMOR_UP	102	0.725968121	2.101784578	1.69601E-05	0.007103861	0.006538028	1202	tags=36%, list=10%, signal=33%	Efemp2/Lamb1/Phka1/Vim/S100a4/Emp3/Nrros/Ccl9/Clec4a3/Jdp2/Gpr34/Snx20/Fcgr2b/Was/Fcrls/Dok1/Tjap1/Scara3/B3gnt9/Phldb1/Loxl3/Csf1r/Htra3/Ccl12/Epb41l2/Gnb4/Adgra2/Stk17b/Loxl1/Mxra8/Lpar1/Fkbp7/Nbl1/Tgfbr2/Spi1/Axl/Fcgrt
VERHAAK_GLIOBLASTOMA_MESENCHYMAL	VERHAAK_GLIOBLASTOMA_MESENCHYMAL	128	0.704123312	2.098639764	1.67895E-05	0.007103861	0.006538028	1865	tags=55%, list=16%, signal=47%	Efemp2/Lamb1/S100a4/Emp3/Anxa4/Slc11a1/Themis2/Lgals1/Chil1/Fes/Fcgr2b/Rac2/Myo1f/Arhgap29/Cd14/Ly96/Uap1/Lcp2/Col1a1/S100a11/Stab1/Clic1/Tcirg1/Ncf2/Tlr2/Rab32/Slc16a3/Dok3/Tnfaip3/Stat6/Vamp5/Wwtr1/Grn/S100a13/Aldh3b1/Cyth4/Lhfpl2/Col5a1/Hexb/Mafb/Tnfrsf1a/Thbd/Gcnt1/Rbks/Ifi30/Tgfbr2/Pold4/Man2b1/Anxa2/Ctsz/Lair1/Cpq/Sat1/Swap70/Ctsc/Itgb2/Gna15/Fhod1/Hexa/Sh2b3/Mfsd1/Col1a2/Sqor/Pdpn/Myh9/Npc2/Tnfrsf1b/Tnfrsf1
MCLACHLAN_DENTAL_CARIES_UP	MCLACHLAN_DENTAL_CARIES_UP	125	0.685720311	2.038162816	1.67811E-05	0.007103861	0.006538028	1433	tags=50%, list=12%, signal=44%	Ifi204/Serpina3n/C4b/Thbs4/Chil1/Sla/Fcgr4/Fcgr2b/Rac2/Lyz2/Cd74/Depp1/H2-DMa/Tent5c/Cd14/Ctsh/Capg/Ly96/Ccl3/Apoc1/Fyb/Lcp2/Alox5ap/Icam1/Skap2/Il10ra/Samsn1/Stab1/Sash3/Cd53/Irf8/Csf1r/Ccl12/Ncf2/Tyrobp/Lbp/Tnfaip3/Lyn/Fcer1g/Arhgdib/Abca1/C1qb/Rab29/Ptp4a3/Cyba/Mt2/C3ar1/Ifi30/Hcls1/Man2b1/Fpr1/Nfil3/C1qa/Sat1/Mt1/Ctsc/Lrch4/Itgb2/Slc7a11/Vca
TAKEDA_TARGETS_OF_NUP98_HOXA9_FUSION_8D_DN	TAKEDA_TARGETS_OF_NUP98_HOXA9_FUSION_8D_DN	112	0.692379324	2.028924695	1.68962E-05	0.007103861	0.006538028	1196	tags=29%, list=10%, signal=27%	Coro2a/Cst7/Slc7a7/Slc11a1/Ccl9/Clec4a3/Tmem163/Chil1/Gpr34/Fcgr2b/Cd14/Fyb/Arhgap18/Clec5a/Emp1/Lcp2/Padi2/Akap13/Ncf2/C1qc/Lgmn/Glipr2/Tm6sf1/C1qb/Cd9/F5/Antxr1/Maf/Clu/Fpr1/Tspan15/C1qa/Mmp
MARKEY_RB1_ACUTE_LOF_DN	MARKEY_RB1_ACUTE_LOF_DN	164	0.622346538	1.910638176	1.65538E-05	0.007103861	0.006538028	1354	tags=42%, list=12%, signal=38%	Nsun4/Ifi27l2a/Isg15/Cxcl10/Anxa4/Ccl9/Usp18/Ifit1/Fcgr2b/Gbp2/Igfbp5/Rac2/Ifih1/Nupr1/Mpeg1/Lyz2/Lgals3bp/Gmfg/Ifit3/Cd52/Ctsh/Ccl3/Il18bp/H2-D1/Skap2/Apobec1/Ube2l6/Fcgr1/Nckap1l/Fermt3/Xdh/Stom/Stab1/Psme2/Ifi35/Csf1r/Ccl12/Ncf2/C1qc/Trim12c/Csf2ra/Tyrobp/Tor3a/Lgmn/Gbp7/Lst1/Grn/Vav1/Abca1/Serping1/C1qb/Ifit2/Mt2/Plekho1/Fkbp7/C3ar1/Selplg/H2-T23/Tmem229b/Hcls1/Fpr1
BLANCO_MELO_COVID19_SARS_COV_2_INFECTION_CALU3_CELLS_UP	BLANCO_MELO_COVID19_SARS_COV_2_INFECTION_CALU3_CELLS_UP	146	0.628535675	1.903673892	1.66528E-05	0.007103861	0.006538028	912	tags=32%, list=8%, signal=30%	Irf1/Rtp4/Isg15/Mx2/Cxcl10/Synpo2/Gbp5/Ifitm1/Usp18/Parp14/Themis2/Dapp1/Parp9/Tmem140/Ifih1/Nupr1/Tlr3/Slc15a3/Depp1/Bst2/Ifit3/Rnf213/Neurl3/Icam1/Slfn5/Ube2l6/Il10ra/Zeb2/Samd9l/Zfp799/Eif2ak2/Ifi35/Kif21b/Ddx58/Trim12c/Aldh1l2/Parp12/Ptafr/Epsti1/Col4a1/Lst1/Tnfaip3/Adrb2
QI_PLASMACYTOMA_UP	QI_PLASMACYTOMA_UP	143	0.625489755	1.890086821	1.66767E-05	0.007103861	0.006538028	1337	tags=34%, list=11%, signal=31%	Cst7/Irf1/Slc43a3/Dapp1/Fas/Lag3/Sla/Gbp2/Rac2/Capn1/Capg/Ccl3/Il21r/Dok1/Alox5ap/Il18bp/Tlr9/Ly86/Sema4a/Pdlim7/Ifi35/Cd53/Irf8/Csf1r/Trim12c/Gnb4/Casp7/Lst1/Tnfaip3/Stk17b/Mta3/Zyx/Vav1/S100a13/Arhgdib/Tgfb1/Mycn/Rgl1/Plekho1/Clu/Hcls1/Sh3bp2/Axl/Ifngr1/Swap70/Stk10/Itgb2/G
RODWELL_AGING_KIDNEY_UP	RODWELL_AGING_KIDNEY_UP	307	0.548157433	1.79925611	1.59406E-05	0.007103861	0.006538028	1467	tags=33%, list=13%, signal=29%	Tcf3/Vim/Emp3/C4b/Gbp5/Ifitm1/Parp14/Clec4a3/Cmtm7/Themis2/Rarres1/Fas/Rac2/Mpeg1/Myo1f/Lyz2/Lacc1/Slc15a3/Gmfg/H2-DMa/Cd14/Rnf213/Fyb/Rps19/Lcp2/Dok1/Pgghg/A2m/Slfn5/H2-D1/Osmr/Il10ra/Dock2/Zeb2/Tpbg/Col1a1/Cybc1/Samd9l/Pik3cg/Ptpre/Stab1/Tbxas1/Psme2/Cd53/Irf8/Csf1r/Clic1/Calhm2/Ddx58/C1qc/Col27a1/Parp12/Tyrobp/Parvg/Gnb4/Dok3/Glipr2/Lst1/Lyn/Stat6/Cx3cr1/Celf2/Grn/Fcer1g/Arhgdib/Loxl1/Akna/Serping1/C1qb/Tmc6/Aebp1/Dtx3l/Col5a1/Plekho1/Antxr1/Selplg/Rps25/H2-T23/Hcls1/Apbb1ip/Man2b1/Anxa2/Fnbp1/Rbp1/Axl/C1qa/Tapbp/Gfra1/Vsir/Stk10/Itgb2/Dkk3/Fhod1/Cldn1/Vcam1/Slc46a3/Trex1/Itga1
BLANCO_MELO_BRONCHIAL_EPITHELIAL_CELLS_INFLUENZA_A_DEL_NS1_INFECTION_UP	BLANCO_MELO_BRONCHIAL_EPITHELIAL_CELLS_INFLUENZA_A_DEL_NS1_INFECTION_UP	346	0.53586801	1.779040552	1.5794E-05	0.007103861	0.006538028	1546	tags=29%, list=13%, signal=26%	Nfkb1/Irf1/Rtp4/Cyld/Mx2/Cxcl10/Synpo2/Ifitm1/Usp18/Parp14/Themis2/Dapp1/Fas/Parp9/Kbtbd8/H3c8/Tmem140/Gbp2/Ifih1/Ednra/Tlr3/Lgals3bp/Gsdmd/Slc15a3/Ttll11/Depp1/Bst2/Ifit3/Pik3ap1/Pamr1/Rnf213/Fyb/Neurl3/Rcan1/Icam1/H2-D1/Tep1/Ube2l6/Il10ra/Ifitm3/Xdh/Samd9l/Zfp799/Akap7/Eif2ak2/Psme2/Ifi35/Icosl/Gem/Ddx58/Ncf2/Trim12c/Slc39a8/Ifi27/Parp12/Tlr2/Epsti1/Mef2c/Gnb4/Lgmn/Casp7/Trim26/Lyn/Gsap/B2m/Slc12a7/Ankk1/Efna1/Bmf/Ifit2/Cab39/Dtx3l/Nod1/Ripk1/C3ar1/H2-T23/Scn3a/Tmem229b/Ifi30/Gramd3/Fzd5/Trim36/Tapbp/Serpinb9/Ptgs1/Wnt4/Myd88/Ccnl1/Trex1/Unc93b1/Spsb1/Bbc3/Fa2h/Klf4/Csf1/Tent2/Rdh10/Bmp4/Slc2a
BLANCO_MELO_COVID19_SARS_COV_2_POS_PATIENT_LUNG_TISSUE_UP	BLANCO_MELO_COVID19_SARS_COV_2_POS_PATIENT_LUNG_TISSUE_UP	60	0.773299817	2.081328043	3.44394E-05	0.011735736	0.010800967	1251	tags=62%, list=11%, signal=55%	Rtp4/Isg15/Mx2/Cxcl10/Gbp5/Ifitm1/Clec4a3/Dapp1/Chil1/Was/Ifit3/Csf3r/Ly96/Fyb/Lcp2/Alox5ap/Igsf6/Ifitm3/S100a11/Samd9l/Eif2ak2/Padi2/Cd53/Ccl12/Tyrobp/Epsti1/Dok3/Gpr84/Fcer1g/Ifit2/F5/C3ar1/Apbb1ip/Spi
SCHUETZ_BREAST_CANCER_DUCTAL_INVASIVE_UP	SCHUETZ_BREAST_CANCER_DUCTAL_INVASIVE_UP	223	0.596362006	1.895630808	3.25961E-05	0.011735736	0.010800967	2105	tags=43%, list=18%, signal=36%	Efemp2/Lamb1/Ifi204/S100a4/Emp3/Slc7a7/Clec4a3/Lgals1/Fas/Fcgr4/Rarres2/Fcgr2b/Ednra/Olfml3/Cd74/H2-DMa/Ly96/Tlr7/Emp1/Lcp2/Ppic/Skap2/Zeb2/Fcgr1/Col1a1/Samsn1/Hpgds/Cd53/Irf8/Gem/Tyrobp/Lmcd1/Mef2c/Plcg2/Slc16a3/Col4a1/Lyn/Pros1/Mmp2/Tm6sf1/Celf2/Wwtr1/Msn/Fcer1g/Loxl1/Aspn/Serping1/C1qb/Mxra8/Aebp1/Col5a1/Mafb/Nbl1/C3ar1/Gpx7/Maf/Ifi30/Hcls1/Tgfbr2/Cpq/Axl/C1qa/Gimap6/Hmox1/Pdlim2/Elk3/Samd4/H2-DMb1/Serpinh1/Col1a2/Entpd1/Abcc4/Bgn/Sulf1/Enpep/Fli1/Ccn1/Evi2b/Rgcc/Tmem47/Angptl2/Tubb6/Tle4/Tgfbi/Igfbp7/Cfh/Olfml2b/Rassf2/Cavin3/Mfap2/Tacc1/
GAL_LEUKEMIC_STEM_CELL_DN	GAL_LEUKEMIC_STEM_CELL_DN	104	0.650532912	1.887919306	3.38903E-05	0.011735736	0.010800967	1225	tags=38%, list=10%, signal=34%	Ifi204/Tpi1/Ccnb2/Gpc5/Slc11a1/Cd86/Lgals1/Top2a/Fcgr2b/Gins2/Lyz2/Smc2/Slc15a3/Cd14/Capg/Klk6/Clec5a/Tep1/Igsf6/Fggy/Fcgr1/Cdc20/Galnt12/Igfbp2/Ncf2/Tlr2/Cx3cr1/Adap2/Mospd3/Plk1/Ccna2/Mafb/Rrm2/C3ar1/Ifi30/Osgin2/Fpr1/C1q
SANSOM_APC_TARGETS_DN	SANSOM_APC_TARGETS_DN	201	0.582367909	1.830558446	4.91537E-05	0.015819311	0.014559279	1566	tags=31%, list=13%, signal=28%	Pcx/Atp2a3/Aamdc/Cst7/Irf1/Otulinl/Npdc1/Akr1b8/Gpr12/Fas/Gbp2/Rac2/Dab1/Prrg2/Lipa/Cd52/Entpd5/Cideb/Dhrs1/Tep1/Pld4/Xdh/Ptpre/Cox7a1/Cd53/Hagh/Slc2a5/C1qc/Tyrobp/Entpd2/Vamp5/Vav1/S100a13/Arhgdib/Btd/Icam2/Tgfb1/Zfp467/Pltp/Plekho1/Nbl1/Ccl25/Cmbl/Selplg/H2-T23/Jak2/Fabp7/Rhoc/Cck/Gnpda1/Ace/Serpinb9/Stk10/Itgb2/Sod3/Cyp2s1/Casp6/H2-
BLANCO_MELO_HUMAN_PARAINFLUENZA_VIRUS_3_INFECTION_A594_CELLS_UP	BLANCO_MELO_HUMAN_PARAINFLUENZA_VIRUS_3_INFECTION_A594_CELLS_UP	99	0.654553944	1.888050088	6.79313E-05	0.017469106	0.016077665	900	tags=38%, list=8%, signal=36%	Irf1/Rtp4/Mx2/Cxcl10/Gbp5/Ifitm1/Usp18/Parp14/Themis2/Parp9/Tmem140/Gbp2/Ifih1/Tlr3/Slc15a3/Bst2/Ifit3/Pik3ap1/Ccl3/Cd68/Ube2l6/Zeb2/Ifitm3/Xdh/Samd9l/Eif2ak2/Ddx58/Ncf2/Trim12c/Aldh1l2/Parp12/Epsti1/Tnfaip3/B2m/Hk2/Serping1/I

LI_INDUCED_T_TO_NATURAL_KILLER_UP	LI_INDUCED_T_TO_NATURAL_KILLER_UP	203	0.559782954	1.761346399	6.55405E-05	0.017469106	0.016077665	1381	tags=32%, list=12%, signal=28%	Nfkb1/Vim/S100a4/Golm1/Serpina3n/Ifitm1/Lgals1/Lag3/Gpr34/Fes/Klk8/S100a6/Fcgr2b/Gbp2/Myo1f/Lgals3bp/Bscl2/Cd52/Capg/Ccl3/Neurl3/Emp1/Plp2/Tspo/Ifitm3/S100a11/Samd9l/Sema4a/Gramd1b/Pdlim7/Tfdp1/Taf9b/Lmna/Tyrobp/Plcg2/Glipr2/Havcr2/Myo1e/Cx3cr1/Hk2/Fcer1g/S100a13/Cd9/Pltp/Arap3/S100a1/Gcnt1/Ifi30/Castor1/Anxa2/Sh3bp2/Fnbp1/Nfil3/Prr13/Litaf/Mt1/Trf/Cd63/Susd3/Mapkapk
JAATINEN_HEMATOPOIETIC_STEM_CELL_DN	JAATINEN_HEMATOPOIETIC_STEM_CELL_DN	96	0.654404671	1.879413861	8.50239E-05	0.019701736	0.018132464	1102	tags=31%, list=9%, signal=29%	Cst7/Ifi204/Zer1/Cd86/Clec4a3/Chil1/Fcgr4/Fcgr2b/Mpeg1/Lyz2/Tent5c/Cd14/Ctsh/Ccl3/Fyb/Il21r/Slfn5/Il10ra/Igsf6/Tfdp1/Ncf2/Tyrobp/Slc14a1/Tnfaip3/Cx3cr1/Fcer1g/F5/Pik3r5/Ifi30/Fpr1
WALLACE_PROSTATE_CANCER_RACE_UP	WALLACE_PROSTATE_CANCER_RACE_UP	141	0.609187588	1.838052222	0.000100142	0.021485994	0.019774603	1636	tags=41%, list=14%, signal=36%	Irf1/Isg15/Mx2/Ifitm1/Cd86/Clec4a3/Themis2/Nr4a2/Sla/Fcgr2b/Rac2/Ednra/Lyz2/Gmfg/Ifit3/Cd52/Ly96/Fyb/Lcp2/Ptpn7/Alox5ap/H2-D1/Ube2l6/Il10ra/Ifitm3/Nckap1l/Samsn1/Stom/Serhl/Cd53/Irf8/Ccl12/Ifi27/Parp12/Mical1/Stk17b/Celf2/Fcer1g/Icam2/C1qb/Pltp/Hmgcs2/Thbd/H2-T23/Clu/Hcls1/Nfil3/C1qa/Gimap6/Ctsc/Serpinb9/Itgb2/Pdlim3/Pdk4/Ifitm2/Herc
WIELAND_UP_BY_HBV_INFECTION	WIELAND_UP_BY_HBV_INFECTION	59	0.736043221	1.974986745	0.000137781	0.028505982	0.026235439	1218	tags=53%, list=10%, signal=47%	Pbk/Cxcl10/Slc7a7/Themis2/Gbp2/Rac2/Lyz2/Cd74/H2-DMa/H2-D1/Ube2l6/Il10ra/Igsf6/Fcgr1/Psme2/Cd53/Ifi27/Parp12/Tyrobp/Rnaset2a/B2m/Fcer1g/Arhgdib/C1qb/Rgl1/Rrm2/C3ar1/H2-
GOLUB_ALL_VS_AML_UP	GOLUB_ALL_VS_AML_UP	19	0.87411086	1.959472926	0.000194725	0.035251261	0.032443446	9	tags=11%, list=0%, signal=11%	Tcf3/Atp2a3
TAKEDA_TARGETS_OF_NUP98_HOXA9_FUSION_10D_DN	TAKEDA_TARGETS_OF_NUP98_HOXA9_FUSION_10D_DN	70	0.686231552	1.88697952	0.00020637	0.036227307	0.033341748	1513	tags=44%, list=13%, signal=39%	Coro2a/Cst7/Ccl9/Rarres1/Chil1/Ccl3/Clec5a/Tlr7/Emp1/Trem2/A2m/Stab1/Padi2/C1qc/Lgmn/Hnrnpc/Id3/C1qb/Cd9/Lhfpl2/Rgl1/Fjx1/Maf/Ace/C1qa/Rit1/Anxa3/Np
DURAND_STROMA_S_UP	DURAND_STROMA_S_UP	197	0.544145686	1.706959621	0.000246035	0.040722264	0.037478675	2080	tags=33%, list=18%, signal=28%	Coro2a/Rtp4/Ifit1/Ccdc80/Car5b/Nudt14/H2-DMa/Cables1/Ctsh/Pdlim4/Fyb/Prrg1/Ube2l6/Osmr/Col1a1/Taf9b/Htra3/Slc39a8/Ifi27/Lgmn/Pbxip1/Slc37a2/Slc27a3/Mmp2/Loxl1/Lpar1/Arhgef28/Dtx3l/Ptn/Ralgapa1/Rhobtb1/St6galnac2/Serpinb1a/Maf/Fabp7/Castor1/Nes/Eya4/Plekha2/S1pr1/Rorb/Cldn1/Hykk/Rb1cc1/Pdk4/Col1a2/Plpp3/S100a16/Matn2/Tmem47/Ctso/St3gal1/Fam20a/Tgfbi/Mmp23/Ndrg1/Fut8/Inpp4b/Rassf2/Rtn
REACTOME_NUCLEAR_IMPORT_OF_REV_PROTEIN	REACTOME_NUCLEAR_IMPORT_OF_REV_PROTEIN	31	-0.822113788	-2.033459865	0.00025778	0.041481143	0.038177108	10	tags=13%, list=0%, signal=13%	Nup37/Nup98/Rcc1/Tpr
JECHLINGER_EPITHELIAL_TO_MESENCHYMAL_TRANSITION_UP	JECHLINGER_EPITHELIAL_TO_MESENCHYMAL_TRANSITION_UP	44	0.757141943	1.945332247	0.000312056	0.04279912	0.039390107	1444	tags=41%, list=12%, signal=36%	Lamb1/Vim/Isg15/C4b/Ifit1/Ifit3/Ppic/Cd68/Ifitm3/Rnaset2a/B2m/Mmp2/Cck/Phgdh/Ctsz/Fmo1/Ptgs1/Serpin
POOLA_INVASIVE_BREAST_CANCER_UP	POOLA_INVASIVE_BREAST_CANCER_UP	116	0.614446488	1.809854268	0.000286287	0.04279912	0.039390107	1070	tags=41%, list=9%, signal=38%	Cst7/Ifi204/Pbk/Cyld/Cxcl10/Btk/Ccnb2/Slc7a7/Soat1/Cd86/Themis2/Top2a/Sla/Fcgr4/Fcgr2b/Gins2/Lyz2/Cd74/Tbc1d31/Cd52/Ly96/Ccl3/Apoc1/Fyb/Lcp2/Dock2/Fcgr1/Cdc20/Samsn1/Cd53/Irf8/Ccl12/Ncf2/Slc16a3/Cks2/Lst1/Stk17b/Fcer1g/Loxl1/Cpped1/Ccna2/Blm/Col5a1/Mafb/Rrm2/Selplg/Ifi30/Hc
OISHI_CHOLANGIOMA_STEM_CELL_LIKE_DN	OISHI_CHOLANGIOMA_STEM_CELL_LIKE_DN	164	0.565306129	1.735520978	0.000281415	0.04279912	0.039390107	1398	tags=28%, list=12%, signal=25%	Efemp2/Atp2a3/Arhgap17/Emp3/Fam214b/Npdc1/Rarres1/Lima1/Gtf3a/Lyz2/Dep1/Cdyl/Pdlim4/Ccl3/Emp1/Efemp1/Prss12/Gem/Slc2a5/Ifi27/Adgra2/Cyp4f14/Hk2/Icam2/Tmc6/Mxra8/Aebp1/Pltp/Col5a1/Mt2/Rhobtb1/Septin9/Serpinb1a/Bcas1/Ifngr1/Hpgd/Mmp14/Mt1/Bmp1/Dkk3/Gna15/Rapgefl1/Elk3/Pdlim3/Myd88/Vcam
LINDGREN_BLADDER_CANCER_CLUSTER_2B	LINDGREN_BLADDER_CANCER_CLUSTER_2B	257	0.513127073	1.655483226	0.000290346	0.04279912	0.039390107	1741	tags=34%, list=15%, signal=29%	Cst7/Slc43a3/Emp3/Serpina3n/C4b/Ifitm1/Ccl9/Lgals1/Klhl6/Rarres2/Ccl6/Fcgr2b/Rac2/Ednra/Myo1f/Lyz2/Ccdc80/H2-DMa/Cd52/Tent5c/Fyb/Lcp2/Ptpn7/Alox5ap/A2m/Icam1/Efemp1/Igdcc4/Il10ra/Dock2/Pdgfrl/Ifitm3/Fermt3/Samsn1/Stom/Gem/Htra3/Epb41l2/Lmcd1/Parvg/Ttc7/Glipr2/Tnfaip3/Pros1/Mmp2/Ebi3/Celf2/Msn/Serping1/Daam2/Cyth4/Aebp1/Mt2/Mafb/Rgl1/Tpst1/C3ar1/Aqp1/Thbd/H2-T23/Maf/Ifi30/Mfng/Nfil3/Rbp1/Nes/Cnn3/Mt1/Bmp2k/Slc16a2/Dkk3/Vcam1/Sh2b3/Col1a2/Klf4/Tgfbr1/Entpd1/Mob3b/Ifitm2/Synpo/Pd
REN_ALVEOLAR_RHABDOMYOSARCOMA_DN	REN_ALVEOLAR_RHABDOMYOSARCOMA_DN	324	0.48644071	1.604834992	0.000317687	0.04279912	0.039390107	2045	tags=28%, list=17%, signal=24%	Efemp2/Snap23/Npdc1/Lima1/S100a6/Lgals3bp/Arpc3/Capg/Emp1/Uap1/Gulp1/Dok1/Efemp1/Ppic/Tspo/Skap2/Osmr/Scara3/Tpbg/Il10rb/Col1a1/Clic1/Gja1/Tmem184b/Rab32/Lxn/Stat6/Mmp2/Msn/Grn/Id3/Zyx/S100a13/Loxl1/Tgfb1/Wipi1/Lpar1/Col5a1/Hexb/Tpst1/Tnfrsf1a/Clu/Rhoc/Tgfbr2/Anxa2/Gramd3/Axl/Prr13/Mmp14/Tapbp/Swap70/Pdlim5/Bmp1/Pdlim2/Elk3/Man2b2/Lman1/Mfsd1/Plpp3/Lamp2/Papss1/Sqor/Myh9/Ccn1/Vkorc1/Ptpn13/Gns/Creb3l2/Rab23/Rrbp1/Cmtm6/Elovl5/Scpep1/Cnn2/Leprel/Nucb2/Tgfbi/Igfbp7/Fkbp10/Plod1/Wls/Igsf [illegible]
BLANCO_MELO_RESPIRATORY_SYNCYTIAL_VIRUS_INFECTION_A594_CELLS_UP	BLANCO_MELO_RESPIRATORY_SYNCYTIAL_VIRUS_INFECTION_A594_CELLS_UP	133	0.58683093	1.757912552	0.00036867	0.048538727	0.044672545	900	tags=30%, list=8%, signal=28%	Irf1/Mx2/Cxcl10/Gbp5/Ifitm1/Usp18/Parp14/Themis2/Parp9/Tmem140/Gbp2/Ifih1/Lacc1/Tlr3/Btn2/Plod2/Ifit3/Ccl3/Il21r/Emp1/Icam1/Slfn5/Cd68/Ube2l6/Osmr/Galnt12/Xdh/Samd9l/Ptpro/Ddx58/Ncf2/Parp12/Ptafr/Tnfaip3/B2m/Hk2/Abca1/Serpin
AGUIRRE_PANCREATIC_CANCER_COPY_NUMBER_DN	AGUIRRE_PANCREATIC_CANCER_COPY_NUMBER_DN	188	-0.532778624	-1.714855535	0.000432317	0.053285365	0.049041107	408	tags=4%, list=3%, signal=4%	Abi1/Pik3c3/Gmeb1/Pdhx/Pole2/Dctd/Hmgn2/Gide
CUI_TCF21_TARGETS_2_DN	CUI_TCF21_TARGETS_2_DN	622	0.420985007	1.465948787	0.000476439	0.057500186	0.052920211	1811	tags=18%, list=15%, signal=16%	Atp2a3/Snap23/Rorg/Ifi204/Arhgap17/Vim/Cpeb2/Phf20l1/Arl3/Nrros/Anxa4/Itgb5/Parp14/Clec4a3/Pld1/Lyz2/Tlr3/Mcu1/Lipa/Fam13c/Cdyl/Plod2/Cables1/Arhgap29/Vps54/Armcx1/Denn2b/4930503L19Rik/Rcan1/Gulp1/Slfn5/Med23/P3h2/Osmr/Zeb2/Fam241a/Il10rb/Uaca/Kcnk6/Ifi35/Gem/Hagh/Trim12c/Parp12/Ednrb/Mef2c/Chac2/Tnfaip3/Slc27a3/Myo1e/Kif1b/Tspan12/Adgra2/Pros1/Vamp5/Jade1/Celf2/Atp6a/Wwtr1/Scarn2/Vcl/Heatr5a/Daam2/Mafb/Gopc/Thbd/H2-T23/Tgfbr2/Itsn1/Stard8/Ncor1/Foxc1/Nfil3/Nes/Cyp20a1/Gimap6/Nostrin/Emcn/Gpd2/Vstm4/Pros2/Pdlim2/Ifpn2/Ak1/Npl/Samd4/Fosp2/Tor/Sh2b3/Klf6/Gpr146/Sh3glb1/Ncoa4/Plpp3/Clic5/
BLANCO_MELO_BETA_INTERFERON_TREATED_BRONCHIAL_EPITHELIAL_CELLS_UP	BLANCO_MELO_BETA_INTERFERON_TREATED_BRONCHIAL_EPITHELIAL_CELLS_UP	201	0.529707828	1.665031887	0.000491537	0.058111754	0.053483067	1771	tags=30%, list=15%, signal=26%	Arl5b/Irf1/Rtp4/Mx2/Cpeb2/Atp13a3/Gbp5/Usp18/Themis2/Dapp1/Fus/Parp9/Tmem140/Gbp2/Ifih1/Ednra/Gmfg/Fyb/Slfn5/Osmr/Samd9l/Ptpro/Eif2ak2/Ddx58/Trim12c/Parp12/Tlr2/Ptafr/Gch1/Casp7/Tnfaip3/Abca1/Adcb2/Ifit2/Cab39/Dtx3l/Tmem229b/Axl/Serpinb9/Mctp1/Myd88/Lrrd2/Slc7a11/Cldn1/Pdk4/Spsb1/Itga1/Csf1/Repo2/Itk2a12/Rp2/Apod/Samhd1/
REACTOME_POSTMITOTIC_NUCLEAR_PORE_COMPLEX_NPC_REFORMATION	REACTOME_POSTMITOTIC_NUCLEAR_PORE_COMPLEX_NPC_REFORMATION	26	-0.822163274	-1.978457348	0.000629268	0.068780174	0.063301731	24	tags=15%, list=0%, signal=15%	Nup37/Nup98/Tnpo1/Rcc1

MEBARKI_HCC_PROGENITOR_FZD8CRD_UP	MEBARKI_HCC_PROGENITOR_FZD8CRD_UP	279	0.486216352	1.581281497	0.000802478	0.083013489	0.076401341	1598	tags=24%, list=14%, signal=22%	Ranbp1/Ccp110/Taf5l/H4c17/Pbk/Vim/Ckap2l/Top2a/H3c8/Cep128/Nuf2/Gins2/Ccdc80/Pamr1/Layn/Fam72a/Zeb2/Slc13a3/Scara3/Cdc20/Phldb1/Loxl3/Tfdp1/Cenpq/Gem/Cenpw/Rfc3/Mki67/Mycl/Cks2/Slc37a2/Mcm8/Id3/Gm960/Hmgb2/Loxl1/Mboat1/Plk1/Cyth4/Ccna2/Faap24/Mdfic/Blm/Plekho1/Rrm2/Cep76/Ncapd3/Nes/E2f1/Ccdc134/Tex30/Anxa3/H3c15/Smc4/Pdlim3/Slc7a11/Fam111a/Panx2/Lrrc45/Cks1b/Micall1/Nek2/Melk/Tmpo/Rdm1/D030056L22Rik/P
WP_WNTBETACATENIN_SIGNALING_PATHWAY_IN_LEUKEMIA	WP_WNTBETACATENIN_SIGNALING_PATHWAY_IN_LEUKEMIA	16	0.869641948	1.886858315	0.000909772	0.088919398	0.081836836	5	tags=6%, list=0%, signal=6%	Tcf3
MORI_EMU_MYC_LYMPHOMA_BY_ONSET_TIME_UP	MORI_EMU_MYC_LYMPHOMA_BY_ONSET_TIME_UP	84	0.638450174	1.802253792	0.000888737	0.088919398	0.081836836	887	tags=13%, list=8%, signal=12%	Tcf3/Ranbp1/Rai1/Cbll1/Gins2/Inka1/Smarcd2/Cenps/
TAKEDA_TARGETS_OF_NUP98_HOXA9_FUSION_3D_UP	TAKEDA_TARGETS_OF_NUP98_HOXA9_FUSION_3D_UP	90	0.629816479	1.794474486	0.000920967	0.088919398	0.081836836	1395	tags=40%, list=12%, signal=35%	Isg15/Mx2/Cxcl10/Ifitm1/Usp18/Parp14/Sdsl/Parp9/Gbp2/Ifih1/Lgals3bp/Bst2/Ifit3/Rnf213/Ccl3/Col24a1/Samd9l/Eif2ak2/Crhbp/Ifi35/Ddx58/Ifi27/Parp12/Epsti1/Trim9/Serping1/Ifit2/Dtx3l/Mycn/Ptprd/Rhobtb1/Scn2a/Angpt1/Bmp2k/Ptgs1/Pla2g4a
SARRIO_EPITHELIAL_MESENCHYMAL_TRANSITION_DN	SARRIO_EPITHELIAL_MESENCHYMAL_TRANSITION_DN	92	0.622903801	1.77940815	0.001074407	0.09731344	0.08956228	1229	tags=23%, list=11%, signal=21%	Pcx/Tcf3/S100a4/Serpina3n/Rarres1/Gbp2/Ccl3/Ifitm3/Stom/Gem/F11r/Tnfaip3/Efna1/Cd9/Mafb/Clu/Nfe2l2/
PICCALUGA_ANGIOIMMUNOBLASTIC_LYMPHOMA_UP	PICCALUGA_ANGIOIMMUNOBLASTIC_LYMPHOMA_UP	136	0.562276579	1.689018332	0.001136915	0.101325329	0.093254616	2150	tags=47%, list=18%, signal=39%	Lamb1/Cxcl10/C4b/Tmem163/Pld1/Chil1/Rarres2/Igfbp5/Ccdc80/Depp1/Gjc1/Arhgap29/Apoc1/Tmem176a/A2m/Ppic/Osmr/Col1a1/Samd9l/Cox7a1/C1qc/Ifi27/Gja1/Col4a1/Wwtr1/Loxl1/Serping1/C1qb/Lpar1/Rgl1/Rspo3/Antxr1/Clu/C1qa/Ctsc/Pdlim5/Cd63/Emcn/Ctsl/Vcam1/Npl/Serpinh1/Enpp2/Fuca1/Col1a2/Septin10/Plpp3/Sulf1/Rhobtb3/Itpripl2/Pdpn/Rab13/Tjp1/Fam20a/Igfbp7/Cfh/Wls/Lamc1/Il18/Cav2/I
IIZUKA_LIVER_CANCER_PROGRESSION_L0_L1_UP	IIZUKA_LIVER_CANCER_PROGRESSION_L0_L1_UP	10	0.912203935	1.807417624	0.001215662	0.10192379	0.093805409	3	tags=10%, list=0%, signal=10%	Zscan12
SMID_BREAST_CANCER_NORMAL_LIKE_UP	SMID_BREAST_CANCER_NORMAL_LIKE_UP	201	0.513412254	1.613809969	0.001245228	0.10192379	0.093805409	1907	tags=36%, list=16%, signal=31%	Atp2a3/Cyld/Btk/Clec4a3/Themis2/Chil1/Sla/Il6ra/Rac2/Fcrls/Lyz2/Cd74/Depp1/Gmfg/Cd180/Cd52/Fxyd1/Ly96/Fyb/Il21r/Git2/Lcp2/Efemp1/Tep1/P3h2/Skap2/Dock2/Nckap1l/Irf8/Gem/Csf2ra/Mef2c/Lst1/Lyn/Pros1/Vamp5/Aass/Celf2/Id3/Icam2/Serping1/Pltp/Plekho1/Ptn/Aqp1/Thbd/Clu/Mt1/Gimap6/Trf/Ctsc/Emcn/Lrch4/Stk10/Kri1/Vcam1/Enpp2/Plpp3/Lyl1/Fli1/Evi2b/Gmip/Rasgrp2/Palmd/Matn2/Prkcq/Tnf
GRAHAM_CML_DIVIDING_VS_NORMAL_QUIESCENT_UP	GRAHAM_CML_DIVIDING_VS_NORMAL_QUIESCENT_UP	98	0.601290891	1.731942105	0.001496293	0.117135499	0.107805483	1540	tags=23%, list=13%, signal=21%	Ranbp1/Cst7/Pbk/Ccnb2/Top2a/Gins2/Smc2/Cdc20/Hpgds/Shmt2/Rfc3/Lmna/Pros1/Ccna2/Rrm2/Cep76/Npm3/Nt5dc2/Cks1b/Nek2/Melk
PETROVA_ENDOTHELIUM_LYMPHATIC_VS_BLOOD_DN	PETROVA_ENDOTHELIUM_LYMPHATIC_VS_BLOOD_DN	99	0.598788774	1.727196372	0.001545438	0.119369597	0.109861632	1621	tags=34%, list=14%, signal=30%	Isg15/Emp3/Itgb5/Slc7a7/Gbp2/Rac2/Plod2/Capg/Pdlim4/Uap1/Icam1/Efemp1/Ifi27/Tcn2/Stat6/Bmp6/Arhgdib/Icam2/Pltp/Mt2/Clu/Axl/Mmp14/Mt1/Ctsc/Hmox1/Mapkapk3/Anxa3/Pla2g4a/Col1a2/Bgn/Lyl1/Lamp2/La
KEGG_LEISHMANIA_INFECTION	KEGG_LEISHMANIA_INFECTION	35	0.746632638	1.851849836	0.001703963	0.122839921	0.113055539	1643	tags=49%, list=14%, signal=42%	Nfkb1/Fcgr4/Fcgr2b/H2-DMa/H2-Oa/Fcgr1/Ncf2/Tlr2/Tgfb1/Cyba/Jak2/Ifngr1/Itgb2/Myd88/H2-
ICHIBA_GRAFT_VERSUS_HOST_DISEASE_35D_UP	ICHIBA_GRAFT_VERSUS_HOST_DISEASE_35D_UP	83	0.621230184	1.750659243	0.001692597	0.122839921	0.113055539	1433	tags=49%, list=12%, signal=44%	Ifi204/S100a4/Serpina3n/S100a6/Gbp2/Mpeg1/Lyz2/Cd74/H2-DMa/Cd52/Cd14/Lcp2/Il18bp/Pld4/Cd68/Fcgr1/S100a11/Cd53/Csf1r/Clic1/C1qc/Tyrobp/Lgmn/Lbp/Grn/C1qb/Plk1/Cyba/Mt2/Selplg/H2-T23/Apbb1ip/Fpr1/C1qa/Tapbp/Litaf/Mt1/Ctsc/Itgb2/V
BIOCARTA_NPC_PATHWAY	BIOCARTA_NPC_PATHWAY	11	-0.900845189	-1.86435185	0.001930112	0.128518836	0.11828212	10	tags=18%, list=0%, signal=18%	Rangap1/Rcc1
YAN_ESCAPE_FROM_ANOIKIS	YAN_ESCAPE_FROM_ANOIKIS	11	0.895748984	1.809276593	0.001911804	0.128518836	0.11828212	757	tags=64%, list=6%, signal=60%	Nfkb1/Irf1/Mx2/Cxcl10/H2-DMa/Eif2ak2/Mmp2
BIOCARTA_MALATEX_PATHWAY	BIOCARTA_MALATEX_PATHWAY	7	0.935763218	1.728212913	0.001821628	0.128518836	0.11828212	303	tags=29%, list=3%, signal=28%	Pcx/Acly
THUM_SYSTOLIC_HEART_FAILURE_UP	THUM_SYSTOLIC_HEART_FAILURE_UP	298	0.462909522	1.514994632	0.001918864	0.128518836	0.11828212	1644	tags=28%, list=14%, signal=24%	Snap23/Rdx/Ifi204/Anxa4/Slc11a1/Fas/Pdia6/Gpr34/Fcgr4/Tmem35b/Fcgr2b/Rac2/Mpeg1/Lyz2/Cd74/Depp1/H2-DMa/Cd52/Cd14/Ly96/Mfn2/Tlr7/Alox5ap/Skap2/Ptpn11/Dock2/Scara3/Hpgds/Zfp672/Pik3cg/Pdlim7/Ppfibp1/Cd53/Irf8/Taf9b/Ccl12/C1qc/Tyrobp/Lst1/Lyn/Cnksr3/Lxn/Tspan12/Cx3cr1/Fcer1g/Arhgdib/Loxl1/Aspn/C1qb/Slc9a9/Tatdn3/Ppp1r3c/C3ar1/Jak2/Tgfbr2/Pik3ip1/Lair1/St3gal3/C1qa/Cnn3/Ctsc/Arntl/Itgb2/Dkk3/Ss18/Rarg/Lman1/Pla2g4a/Vcam1/Tnfaip8l2/Enpp2/Septin10/Clic5/Entpd1/Vamp8/Yth
CREIGHTON_ENDOCRINE_THERAPY_RESISTANCE_5	CREIGHTON_ENDOCRINE_THERAPY_RESISTANCE_5	351	0.449064825	1.493114015	0.001844669	0.128518836	0.11828212	2045	tags=23%, list=17%, signal=20%	Coro2a/Atp2a3/Cpeb2/Prickle2/Phf20l1/Lima1/Ccdc80/Emp1/Fam241a/Smim14/Zfp799/Stom/Serhl/Clic1/Epb41l2/Ica1/Mycl/Rab11a/Dag1/Ddah2/Mta3/S100a13/Fhip2a/Loxl1/Wipi1/Cab39/Lhfpl2/Ncam2/Pgpep1/Pllp/Bcas1/Fnbp1/Gramd3/Fzd5/Zfp275/Sycp2/Sat1/Litaf/Swap70/Nostrin/Anxa3/Fhod1/Lratd2/Samd4/Fuca1/Nipsnap3b/Hacd2/Vamp8/Rai2/Rick3/Sqor/Tmem41a/Myh9/Gramd4/Dbi/Ptpn13/Mtss1/Slc20a1/Gltp/Bcdin3d/Gm/Spire1/Chfr/St3gal1/Wdr37/Nucb2/Gpr157/Mab21/Sh3bp4/Sgsm2/Pbx3/Olfml2b/Miarc2/Wls/Klf3/Cspg4/Zfp36
SU_PLACENTA	SU_PLACENTA	9	-0.921778623	-1.840281887	0.001966156	0.128697938	0.118446956	392	tags=33%, list=3%, signal=32%	Adam12/Hmgb3/Gale
RIZ_ERYTHROID_DIFFERENTIATION_CCNE1	RIZ_ERYTHROID_DIFFERENTIATION_CCNE1	31	-0.756909689	-1.872180586	0.002624672	0.158382546	0.145767141	7	tags=10%, list=0%, signal=10%	Hnrnpdl/Hmgn2/Hmgb3
DUNNE_TARGETS_OF_AML1_MTG8_FUSION_UP	DUNNE_TARGETS_OF_AML1_MTG8_FUSION_UP	31	0.746842102	1.817193879	0.003069946	0.158838262	0.146186558	1119	tags=39%, list=10%, signal=35%	Cst7/Sla/Ifi6ra/Mpo/Lyz2/Csf3r/Alox5ap/Samsn1/Lst1/
WP_HAIR_FOLLICLE_DEVELOPMENT_CYTODIFFERENTIATION_PART_3_OF_3	WP_HAIR_FOLLICLE_DEVELOPMENT_CYTODIFFERENTIATION_PART_3_OF_3	42	0.700086543	1.78651347	0.003065519	0.158838262	0.146186558	2020	tags=40%, list=17%, signal=34%	Tcf3/Nfkb1/S100a4/Igfbp5/Klk6/Bmp6/Tgfb1/Dkk3/Bmp4/Gli2/Notch2/Fmo1/Gjb6/Adam17/Sox9/Egfr/Cd3
KEGG_SYSTEMIC_LUPUS_ERYTHEMATOSUS	KEGG_SYSTEMIC_LUPUS_ERYTHEMATOSUS	43	0.695934062	1.781371336	0.003137568	0.158838262	0.146186558	1442	tags=47%, list=12%, signal=41%	H4c17/C4b/Cd86/H3c8/H4c12/Fcgr4/Fcgr2b/H2ac18/H2az2/H2-DMa/H2-Oa/Fcgr1/C1qc/C1qb/H2ac6/C1qa/H3c15/H2bc4/H2-
ANDERSEN_CHOLANGIOCARCINOMA_CLASS2	ANDERSEN_CHOLANGIOCARCINOMA_CLASS2	57	0.661477964	1.764981634	0.00291681	0.158838262	0.146186558	1460	tags=35%, list=12%, signal=31%	Coro2a/Ccnb2/Depp1/Top2a/Capg/Klk6/Apobec1/Sccl/Cdc20/Galnt12/Slc16a3/Hk2/Aldh3b1/Tfcp2l1/Anxa3/Cyp2s1/Ctsl/Ube2l3b1/Fa2h
REACTOME_ANTIGEN_PROCESSING_CROSS_PRESENTATION	REACTOME_ANTIGEN_PROCESSING_CROSS_PRESENTATION	75	0.616824107	1.713930179	0.003133454	0.158838262	0.146186558	1381	tags=25%, list=12%, signal=22%	Snap23/Btk/Itgb5/Cd14/Ly96/H2-D1/Fcgr1/Psme2/Ncf2/Tlr2/B2m/Cyba/S100a1/H2-T23/Tapbp/Tirap/Psmd4/M
TONKS_TARGETS_OF_RUNX1_RUNX1T1_FUSION_HSC_DN	TONKS_TARGETS_OF_RUNX1_RUNX1T1_FUSION_HSC_DN	115	0.563209353	1.656927218	0.002933095	0.158838262	0.146186558	1088	tags=23%, list=9%, signal=22%	Cst7/Slc43a3/S100a4/Lgals1/Sla/Ifi6ra/Fcgr2b/Igfbp5/Cd52/Capg/Plp2/Lcp2/Alox5ap/Il10ra/Stab1/Sash3/Irf8/Igfbp2/Tyrobp/Slc16a3/Lst1/Mmp2/Myo1c/Cebpa/Rhns18
SENGUPTA_NASOPHARYNGEAL_CARCINOMA_UP	SENGUPTA_NASOPHARYNGEAL_CARCINOMA_UP	181	0.504280979	1.566441224	0.002868873	0.158838262	0.146186558	991	tags=18%, list=8%, signal=17%	Lamb1/Pbk/Cxcl10/Phf20l1/Sympo2/Fas/Top2a/Chil1/Nuf2/Fcgr4/Fcgr2b/Ifih1/Bst2/Zfp458/Cd14/Apoc1/Igsf6/Fcgr1/Col1a1/Vcrt/Rfc3/Col27a1/Gja1/Col4a1/Srcf10/Hmarcc2/Mcm8/Nedd1/Hmgb2/Ccna2/Col5a1/Rrm2/Gas4

CHARAFE_BREAST_CANCER_LUMINAL_VS_MESENCHYMAL_DN	CHARAFE_BREAST_CANCER_LUMINAL_VS_MESENCHYMAL_DN	325	0.446498806	1.473469947	0.002953412	0.158838262	0.146186558	1659	tags=21%, list=14%, signal=18%	Efemp2/Lamb1/Asxl1/Vim/Emp3/Soat1/Ckap2l/Cmtm7/Fas/Tmem35b/Ppp4r1/Msantd3/Layn/Vps54/Plp2/Gulp1/P3h2/Osmr/Cdc20/Prss12/Gja1/Epb41l2/Skp2/Col4a1/Glipr2/Tnfaip3/Lyn/Pros1/Mmp2/Wwtr1/Msn/Loxl1/Vcl/Prkd3/Tgfb1/Plk1/Lpar1/Mdfic/Col5a1/Maml2/Arap3/Antxr1/Serpinb1a/Tgfbr2/Anxa2/Itsn1/Foxc1/Axl/Dennd5a/Ctsc/Eya4/Stk10/Bche/Dkk3/Tlcd3a/Elk3/Ankrd28/Sh2b3/Itga1/Sh3glb1/Rnf182/Abcc4/Fli1/Foxq1/Ka
SMID_BREAST_CANCER_BASAL_UP	SMID_BREAST_CANCER_BASAL_UP	364	0.435568258	1.452980059	0.003144901	0.158838262	0.146186558	2097	tags=28%, list=18%, signal=24%	Pbk/Elf5/Rtp4/Slc43a3/Cxcl10/S100a4/Art3/Atp13a3/Ccnb2/Themis2/Rarres1/Chil1/Klk8/S100a6/Ifih1/Depp1/Gjc1/Cdyl/Plod2/Ccl3/Klk6/Mia/Rcan1/Zfp532/Icam1/P3h2/Ryr1/Cdc20/Galnt12/Padi2/Kif1a/Tfdp1/Ccl12/Mki67/Epb41l2/Skp2/Gldc/Lyn/Wwtr1/Msn/Prkd3/Ccna2/Ptp4a3/Mt2/Col9a3/Ugt8a/Gart/S100a1/Fabp7/Tra2b/Phgdh/Foxc1/Nfil3/Tfcp2l1/Rbp1/Angpt1/Nes/Cnn3/Mmp14/Mt1/Mlc1/Anxa3/Cbs/Scrg1/Ctsl/Cldn1/Pla2g4a/Nt5dc2/Pygb/Serpinh1/Flna/Col11a2/Micall1/Nek2/Melk/Mob3b/Ndufa4l2/Pdpn/Mtmr2/Fat1/Vldlr/Pals2/Rab23/Cryab/Arpc4/Adam17/Ndrg1/Nudt10/Pvr/Sox9/Folh1/Mfap2/Egfr/Anp32e/Myo10/Ncam1/Map2/Cand2/Inava/
BYSTRYKH_HEMATOPOIESIS_STEM_CELL_QTL_TRANS	BYSTRYKH_HEMATOPOIESIS_STEM_CELL_QTL_TRANS	522	0.408424233	1.402898551	0.002800097	0.158838262	0.146186558	1234	tags=14%, list=11%, signal=13%	Tcf3/Snap23/Nfkb1/Otulinl/Cxcl10/Npdc1/Serpina3n/Akr1b8/Cbfb/Ndufaf1/Zkscan3/Hnrnph2/Klk8/Gtf3a/Igfbp5/Mpeg1/Myo1f/Cd74/Prg2/Cd14/Ccl3/Plxnb2/Mia/Cox6b2/Il18bp/Stau1/Skap2/Cd68/Ifitm3/Fggy/Fcgr1/Cdc20/Phldb1/Coasy/Stom/Pcbp2/Tbxas1/Irf8/Cradd/Tcirg1/Igfbp2/Lmna/Tlk2/Ubtf/Tor3a/Lbp/Col4a1/Rab11a/Srsf10/Slc27a1/Stat6/Tspan12/Ddah2/Vav1/Prl/Icam2/Vcl/Serping1/Zfp688/Arid1a/Ankrd17/Arhgef28/Nr2c2ap/Adcy6/Thbd/Maf/Apbb1ip/Itsn1/Tfcp2l1/Has1/Prr13
KLEIN_TARGETS_OF_BCR_ABL1_FUSION	KLEIN_TARGETS_OF_BCR_ABL1_FUSION	16	0.838380553	1.819030603	0.003407185	0.158925427	0.146266781	1010	tags=50%, list=9%, signal=46%	Tcf3/Ifi204/Btk/Tnfrsf13b/Csf3r/Csf1r/Ighmbp2/Septin9
WP_ALANINE_AND_ASPARTATE_METABOLISM	WP_ALANINE_AND_ASPARTATE_METABOLISM	10	0.892583646	1.768542484	0.003429256	0.158925427	0.146266781	2	tags=10%, list=0%, signal=10%	Pcx
KEGG_B_CELL_RECEPTOR_SIGNALING_PATHWAY	KEGG_B_CELL_RECEPTOR_SIGNALING_PATHWAY	55	0.661089993	1.754561055	0.003407126	0.158925427	0.146266781	809	tags=20%, list=7%, signal=19%	Nfkb1/Btk/Ifitm1/Dapp1/Fcgr2b/Rac2/Pik3ap1/Pik3cg/Plcg2/Lyn/Vav1
GOLDRATH_ANTIGEN_RESPONSE	GOLDRATH_ANTIGEN_RESPONSE	224	0.481504255	1.531285507	0.003194263	0.158925427	0.146266781	1267	tags=26%, list=11%, signal=24%	S100a4/Tpi1/Emp3/Ckap2/Serpina3n/Anxa4/Ccnb2/Ccl9/Lgals1/Top2a/Lag3/H3c8/S100a6/Myo1f/Cd74/H2az2/Capg/Ccl3/Emp1/Plp2/Dhrs1/Pld4/Skap2/Cd68/Ifitm3/Mgst1/Cdc20/S100a11/Irf8/Gem/Lmna/Mki67/Tyrobp/Plcg2/Cks2/Rab11a/Lyn/Lxn/S100a13/Hmgb2/Vcl/Plk1/Ccna2/Mdfic/Mt2/Rrm2/H2-T23/Ifi30/Gnpda1/Anxa2/Itsn1/Nfil3/Mcm3/Dennd5a/
REACTOME_NEGATIVE_REGULATORS_OF_DDX58_IFIH1_SIGNALING	REACTOME_NEGATIVE_REGULATORS_OF_DDX58_IFIH1_SIGNALING	25	0.77337698	1.816864884	0.003607137	0.164536582	0.151430999	1120	tags=40%, list=10%, signal=36%	Cyld/Isg15/Ifih1/Ube2l6/Pcbp2/Ddx58/Tnfaip3/Ube2k/Rnf135/Mavs
WORSCHECH_TUMOR_EVASION_AND_TOLEROGENICITY_UP	WORSCHECH_TUMOR_EVASION_AND_TOLEROGENICITY_UP	12	0.87328071	1.795046741	0.003593697	0.164536582	0.151430999	388	tags=33%, list=3%, signal=32%	Nfkb1/Cxcl10/Ccl9/Lcp2
BOWIE_RESPONSE_TO_TAMOXIFEN	BOWIE_RESPONSE_TO_TAMOXIFEN	9	0.89972598	1.745237058	0.00372698	0.168611928	0.155181737	1180	tags=100%, list=10%, signal=90%	Irf1/Isg15/Mx2/Ifitm1/Ifit3/Ifi35/Ifi27/Ifit2/Ifngr1
TAKEDA_TARGETS_OF_NUP98_HOXA9_FUSION_10D_UP	TAKEDA_TARGETS_OF_NUP98_HOXA9_FUSION_10D_UP	96	0.579065788	1.663044773	0.003945109	0.173437408	0.159622861	1189	tags=28%, list=10%, signal=25%	Rtp4/Isg15/Mx2/Cxcl10/Ifitm1/Usp18/Nr4a2/Ifih1/Lgals3bp/Zfp458/Ifit3/Pld4/Galnt12/Samd9l/Eif2ak2/Gem/Ddx58/Ifi27/Epsti1/Adgra2/Trim9/Ifit2/Cebpzos/Krt18/
BENPORATH_PROLIFERATION	BENPORATH_PROLIFERATION	101	0.57285105	1.656702328	0.003951966	0.173437408	0.159622861	1510	tags=22%, list=13%, signal=19%	Ranbp1/Rdx/Pbk/Ccnb2/Top2a/Nuf2/Fance/H2az2/A2m/S100a11/Gnb4/Cks2/Rrm2/Gart/Mcm3/Rit1/Cnih4/Ctsl/Pdcd10/Cks1b/Nek2/P
MMS_MOUSE_LYMPH_HIGH_4HRS_UP	MMS_MOUSE_LYMPH_HIGH_4HRS_UP	29	-0.755024847	-1.847638068	0.004067797	0.175856312	0.161849095	707	tags=17%, list=6%, signal=16%	Psma3/Hnrnpdl/Atp6v1a/Noct/Hmgb3
BOYAULT_LIVER_CANCER_SUBCLASS_G5_DN	BOYAULT_LIVER_CANCER_SUBCLASS_G5_DN	17	0.824198851	1.810332926	0.00421956	0.176120251	0.162092012	889	tags=47%, list=8%, signal=44%	Emp3/Themis2/Icam1/Il10ra/S100a11/Celf2/Arhgdib/Cyba
SALVADOR_MARTIN_PEDIATRIC_TBD_ANTI_TNF_THERAPY_NONRESPONDER_POST_TREATMENT_UP	SALVADOR_MARTIN_PEDIATRIC_TBD_ANTI_TNF_THERAPY_NONRESPONDER_POST_TREATMENT_UP	7	0.919078658	1.697399059	0.004121663	0.176120251	0.162092012	846	tags=86%, list=7%, signal=80%	Irf1/Gbp5/Gbp2/Ube2l6/Fcgr1/Serping1
TAKEDA_TARGETS_OF_NUP98_HOXA9_FUSION_16D_DN	TAKEDA_TARGETS_OF_NUP98_HOXA9_FUSION_16D_DN	60	0.641688129	1.727096615	0.004425465	0.182666811	0.168117128	1758	tags=35%, list=15%, signal=30%	Coro2a/Rarres1/Chil1/Nupr1/Armcx1/Zeb2/Lrp3/Kif21b/Pltp/Maf/Ncor1/Rbp1/Anxa3/Hexa/Tent2/Zfp398/Plpp3/Sfxn5/Mtus1/Tmem37/D
CHUNG_BLISTER_CYTOTOXICITY_DN	CHUNG_BLISTER_CYTOTOXICITY_DN	28	0.748094083	1.790515634	0.004716404	0.188428486	0.173419877	838	tags=14%, list=7%, signal=13%	Snap23/Ifi204/Plcg2/Vcl
CHYLA_CBFA2T3_TARGETS_UP	CHYLA_CBFA2T3_TARGETS_UP	225	0.472453721	1.503211241	0.00475663	0.188733955	0.173701015	1470	tags=29%, list=13%, signal=26%	Taf5l/Cst7/Serpina3n/Tifab/Fcgr2b/1700020L24Rik/Depp1/Gmfg/Msantd3/Cables1/Clec5a/Cideb/Cracr2b/Il21r/Emp1/Alox5ap/Ppic/Pld4/Igsf6/S100a11/Uaca/Tcirg1/Ncf2/Sptan1/Lbp/Vsig10l/Ica1/Tcn2/Plcg2/Cx3cr1/Ebi3/Tm6sf1/Celf2/Mta3/Arhgdib/Cyth4/Tnfrsf1a/Rhobtb1/Chd9/Krt18/Gopc/Fnbp1l/Selplg/Serpinb1a/4931406C07Rik/Pik3ip1/Spi1/Mfng/Stard8/Tet2/Hpgd/Trf/Nostrin/Ctsc/Gfra1/Plekha2/Anxa3/Arl11/Tmem119/Ptgs1/Mct
KEGG_THYROID_CANCER	KEGG_THYROID_CANCER	21	-0.796006326	-1.850667623	0.005019198	0.19514237	0.179598991	169	tags=10%, list=1%, signal=9%	Ret/Tpr
SESTO_RESPONSE_TO_UV_C6	SESTO_RESPONSE_TO_UV_C6	33	-0.7258363	-1.812520519	0.00533014	0.199209682	0.183342335	103	tags=12%, list=1%, signal=12%	Rgs2/Calb2/Hmgn2/Gale
MULLIGHAN_MLL_SIGNATURE_2_UP	MULLIGHAN_MLL_SIGNATURE_2_UP	302	0.443518312	1.453555933	0.00531779	0.199209682	0.183342335	2389	tags=31%, list=20%, signal=26%	Chd3/S100a4/Cbfb/Ccl9/Thbs4/Lgals1/Sla/Il6ra/Fes/S100a6/Rac2/Myo1f/Capg/Plxnb2/Mfn2/Git2/Pycard/Fcgr1/Il10rb/Ptafr/Oaz1/Mical1/Cln3/Slc16a3/Lst1/Slc27a3/Nagpa/Grn/Aldh3b1/Adrb2/Rab29/Cyth4/Cyba/Hexb/Aspscr1/Cebpa/Selplg/Rbks/Med8/Hcls1/Apbb1ip/Spi1/Pold4/Hdac6/Lair1/Arntl/Cnih4/Plin3/Itgb2/Kri1/Rab3d/Ctsl/Pla2g4a/Trex1/Mfsd1/Fam53b/Flna/Tmpo/Lamp2/Abr/Fbxw4/Gna12/Upb1/Evi2b/Naga/Htr1f/Ctsa/Sec23b/Slc15a2/Rrbp1/Arpc4/Scpep1/Igfbp7/Malt1/Hnrnpul1/Pbx3/Plod1/Iqgap1/Rassf2/Zfp446/Lrp10/Akt2/Cand2/Meis1/Rangrf/Osbpl1a/Itga7/Tada3/Tgfa/Fosl2/Tns3/L

This table lists significantly enriched Gene Set Enrichment Analysis (GSEA) pathways. "MPTP" refers to the acute MPTP model, "A53T" to the 16 months A53T-α-synuclein model, and "MitoPark" to the 5m MitoPark model.
