# Supplementary material for: Neurotoxicity, α-synuclein pathology, and mitochondrial dysfunction: A comparative study of different mouse models of Parkinson’s disease
Source: Neural Regen Res. 2025 Sep 29;21(10):5000–12. doi: 10.4103/NRR.NRR-D-25-00648 (PMC13568611; doi:10.4103/NRR.NRR-D-25-00648)
Supplement: Supplementary file 6 [file NRR-21-5000_Suppl5.pdf]

Additional Table 8. Intersection analysis of DEPs across the three PD mouse models

**Only MPTP**		**Only A53T**		**Only MitoPark**		**A53T AND MitoPark NOT MPTP**		**A53T AND MPTP NOT MitoPark**		**MitoPark AND MPTP NOT A53T**		***A53T AND MitoPark AND MPTP***	
**Gene**	**Description**	**Gene**	**Description**	**Gene**	**Description**	**Gene**	**Description**	**Gene**	**Description**	**Gene**	**Description**	**Gene**	**Description**
Aass	Alpha-aminoadipic semialdehyde synthase, mitochondrial	*Aarsd1*	Alanyl-tRNA editing protein Aarsd1	*Abcc9*	ATP-binding cassette sub-family C member 9	*Ascc3*	Activating signal cointegrator 1 complex subunit 3	*Abca1*	Phospholipid-transporting ATPase ABCA1	*Aldh1a1*	Aldehyde dehydrogenase 1A1	*Sephs1*	Selenide, water dikinase 1
Abca8b	ABC-type organic anion transporter ABCA8B	*Abcb7*	Iron-sulfur clusters transporter ABCB7, mitochondrial	*Acot2*	Acyl-coenzyme A thioesterase 2, mitochondrial	*B4galnt1*	Beta-1,4 N-acetylgalactosaminyltransferase 1	*Adgrg1*	Adhesion G-protein coupled receptor G1	*Cnn3*	Calponin-3	*Timm8a1*	Mitochondrial import inner membrane translocase subunit
Abcd3	ATP-binding cassette sub-family D member 3	*Abhd14b*	Putative protein-lysine deacylase ABHD14B	*Agpat5*	1-acyl-sn-glycerol-3-phosphate acyltransferase epsilon	*Bcat2*	Branched-chain-amino-acid aminotransferase, mitochondrial	*Arpc5l*	Actin-related protein 2/3 complex subunit 5-like protein	*Cox7c*	Cytochrome c oxidase subunit 7C, mitochondrial		
Abhd10	Palmitoyl-protein thioesterase ABHD10, mitochondrial	*Abhd3*	Phospholipase ABHD3	*Akt2*	RAC-beta serine/threonine-protein kinase	*Cadps2*	Calcium-dependent secretion activator 2	*Arpp19*	cAMP-regulated phosphoprotein 19	*Ddc*	Aromatic-L-amino-acid decarboxylase		
Abhd4	(Lyso)-N-acylphosphatidylethanolamine lipase	*Abhd6*	Monoacylglycerol lipase ABHD6	*Alg1*	Chitobiosyldiphosphodolichol beta-mannosyltransferase	*Gprc5b*	G-protein coupled receptor family C group 5 member B	*Atp2b1*	Plasma membrane calcium-transporting ATPase 1	*Fam234a*	Protein FAM234A		
Abraxas2	BRISC complex subunit Abraxas 2	*Abracl*	Costars family protein ABRACL	*Amacr*	Alpha-methylacyl-CoA racemase	*Mtch1*	Mitochondrial carrier homolog 1	*Cables2*	CDK5 and ABL1 enzyme substrate 2	*Ildr2*	Immunoglobulin-like domain-containing receptor 2		
Acad10	Acyl-CoA dehydrogenase family member 10	*Acaa1a*	3-ketoacyl-CoA thiolase A, peroxisomal	*Ap2m1*	AP-2 complex subunit mu	*Plin3*	Perilipin-3	*Celf4*	CUGBP Elav-like family member 4	*Inpp5j*	Phosphatidylinositol 4,5-bisphosphate 5-phosphatase A		
Ache	Acetylcholinesterase	*Adam10*	Disintegrin and metalloproteinase domain-containing	*Arid1b*	AT-rich interactive domain-containing protein 1B	*Rabgef1*	Rab5 GDP/GTP exchange factor	*Cend1*	Cell cycle exit and neuronal differentiation protein 1	*Kank4*	KN motif and ankyrin repeat domain-containing protein 4		
Acot1	Acyl-coenzyme A thioesterase 1	*Adam15*	Disintegrin and metalloproteinase domain-containing	*Atp1b1*	Sodium/potassium-transporting ATPase subunit beta-1	*Rars2*	Probable arginine--tRNA ligase, mitochondrial	*Ckb*	Creatine kinase B-type	*Kcnc1*	Voltage-gated potassium channel KCNC1		
Acot11	Acyl-coenzyme A thioesterase 11	*Adam23*	Disintegrin and metalloproteinase domain-containing	*Atp5f1a*	ATP synthase F(1) complex subunit alpha, mitochondrial	*Rtn4rl1*	Reticulon-4 receptor-like 1	*Clcn2*	Chloride channel protein 2	*Lgals3bp*	Galectin-3-binding protein		
Acrbp	Acrosin-binding protein	*Add2*	Beta-adducin	*Atp5mg*	ATP synthase F(0) complex subunit g, mitochondrial	*Sf3b5*	Splicing factor 3B subunit 5	*Coasy*	Bifunctional coenzyme A synthase	*Mme*	Neprilysin		
Acsbg1	Long-chain-fatty-acid--CoA ligase ACSBG1	*Ahctf1*	Protein ELYS	*Atp5po*	ATP synthase peripheral stalk subunit OSCP, mitochondrial	*Stx2*	Syntaxin-2	*Cox7a1*	Cytochrome c oxidase subunit 7A1, mitochondrial	*Mydgf*	Myeloid-derived growth factor		
Actb	Actin, cytoplasmic 1	*Aida*	Axin interactor, dorsalization-associated protein	*Bmerb1*	bMERB domain-containing protein 1	*Supt4h1a*	Transcription elongation factor SPT4-A	*Cplx1*	Complexin-1	*Opa3*	Optic atrophy 3 protein homolog		
Actg2	Actin, gamma-enteric smooth muscle	*Aldh1a2*	Retinal dehydrogenase 2	*Bola1*	BolA-like protein 1	*Uprt*	Uracil phosphoribosyltransferase homolog	*Dhrs13*	Dehydrogenase/reductase SDR family member 13	*Pip5k1a*	Phosphatidylinositol 4-phosphate 5-kinase type-1 alpha		
Actn2	Alpha-actinin-2	*Alg2*	Programmed cell death protein 6	*C1qa*	Complement C1q subcomponent subunit	*Ydjc*	Carbohydrate deacetylase	*Dipk1b*	Divergent protein kinase domain 1B	*Pnkd*	Probable thioesterase PNKD		
Actr3b	Actin-related protein 3B	*Alkbh5*	RNA demethylase ALKBH5	*C1ra*	Complement C1r-A subcomponent			*Dnajb6*	DnaJ homolog subfamily B member 6	*Prag1*	Inactive tyrosine-protein kinase PRAG1		

Adcy3	Adenylate cyclase type 3	*Alyref*	THO complex subunit 4	*Ca11*	Gastrokine-1	*Dynlt1*	Dynein light chain Tctex-type 1	*Psmd5*	26S proteasome non-ATPase regulatory subunit 5
Adcy8	Adenylate cyclase type 8	*Ampd3*	AMP deaminase 3	*Ca8*	Carbonic anhydrase-related protein	*Fbxo2*	F-box only protein 2	*Ranbp9*	Importin-9
ADGRF3	Adhesion G-protein coupled receptor F3	*Ank3*	Ankyrin-3	*Cacng8*	Voltage-dependent calcium channel	*Frmd4a*	FERM domain-containing protein 4A	*Rpl35a*	Large ribosomal subunit protein eL33
Aif1l	Allograft inflammatory factor 1-like	*Ankmy2*	Ankyrin repeat and MYND domain-containing protein 2	*Cacybp*	Calcyclin-binding protein	*Gabra3*	Gamma-aminobutyric acid receptor subunit alpha-3	*Rps15a*	Small ribosomal subunit protein uS8
Ajap1	Adherens junction-associated protein 1	*Ap5z1*	AP-5 complex subunit zeta-1	*Cadm3*	Cell adhesion molecule 3	*Homer2*	Homer protein homolog 2	*S100a11*	Protein S100-A11
Ak2	Adenylate kinase 2, mitochondrial	*Apbb1*	Amyloid beta precursor protein binding family B member 1	*Cbs*	Cystathionine beta-synthase	*Ipo8*	Importin-8	*Sars2*	Serine--tRNA ligase, mitochondrial
Ak5	Adenylate kinase isoenzyme 5	*Arhgap1*	Rho GTPase-activating protein 1	*Cd81*	CD81 antigen	*Itpka*	Inositol-trisphosphate 3-kinase A	*Sdhaf2*	Succinate dehydrogenase assembly factor 2, mitochondrial
Akt1s1	Proline-rich AKT1 substrate 1	*Arhgap39*	Rho GTPase-activating protein 39	*Cdh12*	Cadherin-12	*Lamtor1*	Ragulator complex protein LAMTOR1	*Slc18a2*	Synaptic vesicular amine transporter
Aldh1b1	Aldehyde dehydrogenase X, mitochondrial	*Arl2bp*	ADP-ribosylation factor-like protein 2-binding protein	*Celsr2*	Cadherin EGF LAG seven-pass G-type receptor 2	*Mlf2*	Myeloid leukemia factor 2	*Slc25a5*	ADP/ATP translocase 2
Aldh1l1	Cytosolic 10-formyltetrahydrofolate dehydrogenase	*Atcay*	Caytaxin	*Chac2*	Putative glutathione-specific gamma-glutamylcyclotransferase	*Myl12b*	Myosin regulatory light chain 12B	*Slc6a3*	Sodium-dependent dopamine transporter
Ankrd63	Ankyrin repeat domain-containing protein 63	*Atp1a1*	Sodium/potassium-transporting ATPase subunit alpha-1	*Chrm2*	Muscarinic acetylcholine receptor M2	*Ndufa12*	NADH dehydrogenase [ubiquinone] 1 alpha subcomplex subunit 12	*Stxbp6*	Syntaxin-binding protein 6
Ano3	Anoctamin-3	*Atp1a4*	Sodium/potassium-transporting ATPase subunit alpha-4	*Clptm1l*	Lipid scramblase CLPTM1L	*Ndufaf3*	NADH dehydrogenase [ubiquinone] 1 alpha subcomplex assembly factor 3	*Th*	Tyrosine 3-monooxygenase
Anxa2	Annexin A2	*Atp2a2*	Sarcoplasmic/endoplasmic reticulum calcium ATPase 2	*Cmip*	C-Maf-inducing protein	*Nexn*	Nexilin	*Tia1*	Cytotoxic granule associated RNA binding protein TIA1
Anxa4	Annexin A4	*Atp2b2*	Plasma membrane calcium-transporting ATPase 2	*Cnnm3*	Metal transporter CNNM3	*Nptx1*	Neuronal pentraxin-1	*Tmem263*	Transmembrane protein 263
Ap4e1	AP-4 complex subunit epsilon-1	*Atp5f1e*	ATP synthase F(1) complex subunit epsilon, mitochondrial	*Commd4*	COMM domain-containing protein 4	*Pcyt1b*	Choline-phosphate cytidylyltransferase B	*Ttyh2*	Protein tweety homolog 2
Ap5m1	AP-5 complex subunit mu-1	*Atp8a2*	Phospholipid-transporting ATPase IB	*Coq8a*	Atypical kinase COQ8A, mitochondrial	*Pex1*	Peroxisomal ATPase PEX1		
Apba1	Amyloid-beta A4 precursor protein-binding family A	*Atp9a*	Probable phospholipid-transporting ATPase IIA	*Cox20*	Cytochrome c oxidase assembly protein COX20, mitochondrial	*Plxnc1*	Plexin-C1		
Apba2	Amyloid-beta A4 precursor protein-binding family A	*Bdnf*	Neurotrophic factor BDNF precursor form	*Cpeb3*	Cytoplasmic polyadenylation element-binding protein	*Rab26*	Ras-related protein Rab-26		

Apbb1ip	Amyloid beta A4 precursor protein-binding family B member 1-interacting	*Bud31*	Protein BUD31 homolog	*Cpt1a*	Carnitine O-palmitoyltransferase 1, liver isoform			*Rad23a*	UV excision repair protein RAD23 homolog A
Apex1	DNA repair nuclease/redox regulator APEX1	*C1qtnf5*	Complement C1q tumor necrosis factor-related protein 5	*Cs*	Citrate synthase, mitochondrial			*Ranbp6*	Ran-binding protein 6
Aplp2	Amyloid beta precursor like protein 2	*Ca1*	Carbonic anhydrase 1	*Ctu2*	Cytoplasmic tRNA 2-thiolation protein 2			*Shisa9*	Protein shisa-9
Arglu1	Arginine and glutamate-rich protein 1	*Ca10*	Carbonic anhydrase-related protein 10	*Cux1*	Protein CASP			*Sirpa*	Tyrosine-protein phosphatase non-receptor type substrate 1
Arhgap20	Rho GTPase-activating protein 20	*Cacnb3*	Voltage-dependent L-type calcium channel subunit beta-3	*Cyb5r4*	Cytochrome b5 reductase 4			*Skp1*	S-phase kinase-associated protein 1
Arhgap6	Rho GTPase-activating protein 6	*Cadps*	Calcium-dependent secretion activator 1	*Cyp46a1*	Cholesterol 24-hydroxylase			*Sorbs1*	Sorbin and SH3 domain-containing protein 1
Arhgef10	Rho guanine nucleotide exchange factor 10	*Calm1*	Calmodulin-1	*Cyp4f3*	Cytochrome P450 4F3			*Stat3*	Signal transducer and activator of transcription
Arhgef2	Rho guanine nucleotide exchange factor 2	*Camk2a*	Calcium/calmodulin-dependent protein kinase type II subunit	*Dda1*	DET1- and DDB1-associated protein 1			*Syap1*	Synapse-associated protein 1
Arpc1b	Actin-related protein 2/3 complex subunit 1B	*Camk2d*	Calcium/calmodulin-dependent protein kinase type II subunit	*Ddah2*	Putative hydrolase DDAH2			*Tceal3*	Transcription elongation factor A protein-like 3
Arpc3	Actin-related protein 2/3 complex subunit 3	*Camkk1*	Calcium/calmodulin-dependent protein kinase kinase 1	*Dhdh*	Trans-1,2-dihydrobenzene-1,2-diol dehydrogenase			*Tlnrd1*	Talin rod domain-containing protein 1
Asic1	Acid-sensing ion	*Capn5*	Calpain-5	*Dhfr*	Dihydrofolate reductase			*Tmod1*	Tropomodulin-1
Atp2b4	Plasma membrane calcium-transporting ATPase 4	*Cat*	Catalase	*Dnajc10*	DnaJ homolog subfamily C member 10			*Vasp*	Vasodilator-stimulated phosphoprotein
Atp5f1b	ATP synthase F(1) complex catalytic subunit beta, mitochondrial	*Cbarp*	Voltage-dependent calcium channel beta subunit-associated regulatory protein	*Dnajc12*	DnaJ homolog subfamily C member 12				
Atp5pb	ATP synthase peripheral stalk subunit b, mitochondrial	*Cbx3*	Chromobox protein homolog 3	*Dnm1*	Dynamin-1				
Atp5pd	ATP synthase peripheral stalk subunit d, mitochondrial	*Ccdc177*	Coiled-coil domain-containing protein 177	*Dop1b*	Protein DOP1B				
Atp6v1g1	V-type proton ATPase subunit G 1	*Cck*	Cholecystokinin	*Drg1*	Developmentally-regulated GTP-binding				
Atpaf1	ATP synthase mitochondrial F1 complex assembly factor 1	*Ccs*	Copper chaperone for superoxide dismutase	*Ecsit*	Evolutionarily conserved signaling intermediate in Toll pathway, mitochondrial				
Atxn10	Ataxin-10	*Ccsap*	Centriole, cilia and spindle-associated	*Efhd2*	EF-hand domain-containing protein D2				

B2m	Beta-2-microglobulin	*Cd47*	Leukocyte surface antigen CD47	*Eif5a2*	Eukaryotic translation initiation factor 5A-2
B4galt6	Beta-1,4-galactosyltransferase 6	*Cd55b*	Complement decay-accelerating factor transmembrane isoform	*Emb*	Embigin
Bag3	BAG family molecular chaperone regulator 3	*Cd99*	CD99 antigen	*Entpd1*	Ectonucleoside triphosphate diphosphohydrolase 1
Bap1	Ubiquitin carboxyl-terminal hydrolase BAP1	*Cdip1*	Cell death-inducing p53-target protein 1	*Entpd3*	Ectonucleoside triphosphate diphosphohydrolase 3
Bax	Apoptosis regulator BAX	*Cdk18*	Cyclin-dependent kinase 18	*Epha7*	Ephrin type-A receptor 7
Bbs2	BBSome complex member BBS2	*Cdk9*	Cyclin-dependent kinase 9	*Ephx1*	Epoxide hydrolase 1
Bcar3	Breast cancer anti-estrogen resistance protein 3 homolog	*Cerk*	Ceramide kinase	*Erbin*	Erbin
Bloc1s6	Biogenesis of lysosome-related organelles complex 1 subunit 6	*Cfdp1*	Craniofacial development protein 1	*Erich5*	Glutamate-rich protein 5
Bod1	Biorientation of chromosomes in cell division protein 1	*Chchd2*	Coiled-coil-helix-coiled-coil-helix domain-containing protein 2	*Fam210a*	Protein FAM210A
Brd4	Bromodomain-containing protein 4	*Cnih2*	Protein cornichon homolog 2	*Fam98b*	Protein FAM98B
Brix1	Ribosome biogenesis protein BRX1 homolog	*Cog3*	Conserved oligomeric Golgi complex subunit 3	*Fbxo31*	F-box only protein 31
Brsk1	Serine/threonine-protein kinase BRSK1	*Comtd1*	Catechol O-methyltransferase domain-containing	*Fcgr2*	Low affinity immunoglobulin gamma Fc region receptor II
C1qc	Complement C1q subcomponent subunit C	*Coq6*	Ubiquinone biosynthesis monooxygenase COQ6, mitochondrial	*Fgf12*	Fibroblast growth factor 12
C4b	Complement C4-B	*Coq7*	NADPH-dependent 3-demethoxyubiquinone 3-hydroxylase,	*Fkbp2*	Peptidyl-prolyl cis-trans isomerase FKBP2
Ca14	Carbonic anhydrase 14	*Coro1c*	Coronin-1C	*Fyco1*	FYVE and coiled-coil domain-containing protein 1
Camk1	Calcium/calmodulin-dependent protein kinase type 1	*Cplx3*	Complexin-3	*Gcat*	2-amino-3-ketobutyrate coenzyme A ligase, mitochondrial
Camk1d	Calcium/calmodulin-dependent protein kinase type 1D	*Cpne8*	Copine-8	*Gfm2*	Ribosome-releasing factor 2, mitochondrial
Camk2b	Calcium/calmodulin-dependent protein kinase type II subunit	*Cpox*	Oxygen-dependent coproporphyrinogen-III oxidase, mitochondrial	*Gipc2*	PDZ domain-containing protein GIPC2

Camkk2	Calcium/calmodulin-dependent protein kinase kinase 2	*Crip2*	Cysteine-rich protein 2	*Glb1*	Beta-galactosidase
Ccdc127	Coiled-coil domain-containing protein 127	*Crppa*	D-ribitol-5-phosphate cytidylyltransferase	*Glrx3*	Glutaredoxin-3
Ccdc136	Coiled-coil domain-containing protein 136	*Csrp1*	Cysteine and glycine-rich protein 1	*Gls2*	Glutaminase liver isoform, mitochondrial
Ccdc25	Coiled-coil domain-containing protein 25	*Cul2*	Cullin-2	*Gmppb*	Mannose-1-phosphate guanylyltransferase catalytic subunit beta
Ccdc51	Mitochondrial potassium channel	*Cyb5r1*	NADH-cytochrome b5 reductase 1	*Gnb4*	Guanine nucleotide-binding protein subunit
Ccdc85c	Coiled-coil domain-containing protein 85C	*Cyth3*	Cytohesin-3	*Gng11*	Guanine nucleotide-binding protein G(I)/G(S)/G(O) subunit
Ccdc92	Coiled-coil domain-containing protein 92	*Dcaf7*	DDB1- and CUL4-associated factor 7	*Gpn1*	GPN-loop GTPase 1
Cd151	CD151 antigen	*Dctn5*	Dynactin subunit 5	*Gpr37l1*	G-protein coupled receptor 37-like 1
Cdc5l	Cell division cycle 5-like protein	*Dcun1d2*	DCN1-like protein 2	*Grip1*	Glutamate receptor-interacting protein 1
Cdh2	Cadherin-2	*Dennd1a*	DENN domain-containing protein 1A	*Grk6*	G protein-coupled receptor kinase 6
Cdk11b	Cyclin-dependent kinase 11B	*Denr*	Density-regulated protein	*Gtf2a2*	Transcription initiation factor IIA subunit 2
Cdk19	Cyclin-dependent kinase 19	*Derpc*	Decreased expression in renal and prostate cancer protein	*Gtf2b*	Transcription initiation factor IIB
Cdk5r2	Cyclin-dependent kinase 5 activator 2	*Dhcr7*	7-dehydrocholesterol reductase	*Gtf2f1*	General transcription factor IIF subunit 1
Cds1	Phosphatidate cytidylyltransferase 1	*Dhx29*	ATP-dependent RNA helicase DHX29	*Guf1*	Translation factor Guf1, mitochondrial
Cfap298	Cilia- and flagella-associated protein 298	*Dlg1*	Disks large homolog 1	*Gxylt1*	Glucoside xylosyltransferase 1
Chchd1	Small ribosomal subunit protein mS37	*Dmwd*	Dystrophia myotonica WD repeat-containing	*Hebp1*	Heme-binding protein 1
Chd2	Chromodomain-helicase-DNA-binding	*Dnajc24*	DnaJ homolog subfamily C member 24	*Hmox2*	Heme oxygenase 2
Chdh	Choline dehydrogenase, mitochondrial	*Dus3l*	tRNA-dihydrouridine(47)	*Hpgds*	Hematopoietic prostaglandin D
Chmp1b2	Charged multivesicular body protein 1B2	*Dync2i2*	Cytoplasmic dynein 2 intermediate chain 2	*Icmt*	Protein-S-isoprenylcysteine O-
Chmp5	Charged multivesicular body protein 5	*Ecpas*	Proteasome adapter and scaffold protein ECM29	*Ier3ip1*	Immediate early response 3-interacting
Chn1	N-chimaerin	*Ehd2*	EH domain-containing protein 2	*Ift25*	Intraflagellar transport protein 25 homolog
Ckmt1	Creatine kinase U-type, mitochondrial	*Eif1*	Eukaryotic translation initiation factor 1	*Ighm*	Immunoglobulin heavy constant mu
Clic1	Chloride intracellular channel protein 1	*Eif2ak4*	eIF-2-alpha kinase GCN2	*Inmt*	Indolethylamine N-methyltransferase

Clmp	CXADR-like membrane protein	*Elfn1*	Protein ELFN1	*Ints14*	Integrator complex subunit 14			
Clu	Clusterin	*Elmo2*	Engulfment and cell motility protein 2	*Ints9*	Integrator complex subunit 9			
Cmbl	Carboxymethylenebutenolidase homolog	*Elp3*	Elongator complex protein 3	*Iqgap1*	Ras GTPase-activating-like protein IQGAP1			
Cnnm4	Metal transporter CNNM4	*Emc7*	Endoplasmic reticulum membrane protein complex subunit 7	*Iqgap2*	Ras GTPase-activating-like protein IQGAP2			
Cnot7	CCR4-NOT transcription complex	*Epb41l2*	Band 4.1-like protein 2	*Jpt1*	Jupiter microtubule associated homolog 1			
Coa3	Cytochrome c oxidase assembly factor 3 homolog, mitochondrial	*Epdr1*	Mammalian ependymin-related protein 1	*Jund*	Transcription factor JunD			
Commd6	COMM domain-containing protein 6	*Epg5*	Ectopic P granules protein 5 homolog	*Kcnc3*	Voltage-gated potassium channel KCNC3			
Commd8	COMM domain-containing protein 8	*Etnk1*	Ethanolamine kinase 1	*Kcnh2*	Voltage-gated inwardly rectifying potassium channel KCNH2			
Comt	Catechol O-methyltransferase	*Extl2*	Exostosin-like 2	*Klhl26*	Kelch-like protein 26			
Copg1	Coatomer subunit gamma-1	*Fbll1*	RNA 2'-O-methyltransferase	*Kpna4*	Importin subunit alpha-3			
Cops7b	COP9 signalosome complex subunit 7b	*Fbxl16*	F-box/LRR-repeat protein 16	*Kpna6*	Importin subunit alpha-7			
Coq3	Ubiquinone biosynthesis O-methyltransferase, mitochondrial	*Fbxo21*	F-box only protein 21	*Krt16*	Keratin, type I cytoskeletal 16			
Coro7	Coronin-7	*Fbxo3*	F-box only protein 3	*Lima1*	LIM domain and actin-binding protein 1			
Cox6b1	Cytochrome c oxidase subunit 6B1	*Foxp1*	Forkhead box protein P1	*Lrrc8b*	Volume-regulated anion channel subunit			
Cox7a2	Cytochrome c oxidase subunit 7A2,	*Foxp4*	Forkhead box protein P4	*Lrrc8d*	Volume-regulated anion channel subunit			
Cpne3	Copine-3	*Gabrb1*	Gamma-aminobutyric acid receptor subunit	*Lypla1*	Acyl-protein thioesterase 1			
Cracd	Capping protein-inhibiting regulator of actin dynamics	*Gcdh*	Glutaryl-CoA dehydrogenase, mitochondrial	*Marf1*	Meiosis regulator and mRNA stability factor 1			
Crebbp	Histone lysine acetyltransferase CREBBP	*Gcn1*	Stalled ribosome sensor GCN1	*Mast1*	Microtubule-associated serine/threonine-protein kinase 1			
Csmd1	CUB and sushi domain-containing protein 1	*Ggact*	Gamma-glutamylaminecyclotransferase	*Mfge8*	Lactadherin			
Cst3	Cystatin-C	*Glcci1*	Glucocorticoid-induced transcript 1 protein	*Mindy4*	Probable ubiquitin carboxyl-terminal hydrolase MINDY-4			
Cstb	Cystatin-B	*Glce*	D-glucuronyl C5-epimerase	*Mkln1*	Muskelin			

Ctnna3	Catenin alpha-3	*Glul*	Glutamine synthetase	*Mms19*	MMS19 nucleotide excision repair protein homolog
Custos	Protein CUSTOS	*Gmeb2*	Glucocorticoid modulatory element-	*Mogs*	Mannosyl-oligosaccharide
Cyp4f14	Leukotriene-B4 omega-hydroxylase 3	*Gsg1l*	Germ cell-specific gene 1-like protein	*Morf4l2*	Mortality factor 4-like protein 2
Czib	CXXC motif containing zinc binding protein	*Gtf3c1*	General transcription factor 3C polypeptide 1	*Mrpl51*	Large ribosomal subunit protein mL51
D3Ertd751e	UPF0462 protein C4orf33 homolog	*Gys1*	Glycogen [starch] synthase, muscle	*Mrps18b*	Small ribosomal subunit protein mS40
Dab2ip	Disabled homolog 2-interacting protein	*Haghl*	Hydroxyacylglutathione hydrolase-like protein	*Mrps23*	Small ribosomal subunit protein mS23
Dap	Death-associated protein 1	*Hba*	Hemoglobin subunit alpha	*Nap1l1*	Nucleosome assembly protein 1-like 1
Dbn1	Drebrin	*Hbb-b1*	Hemoglobin subunit beta-1	*Ndufa11*	NADH dehydrogenase [ubiquinone] 1 alpha subcomplex subunit 11
Dbnl	Drebrin-like protein	*Hdgf*	Hepatoma-derived growth factor	*Ndufaf2*	NADH dehydrogenase [ubiquinone] 1 alpha subcomplex assembly factor 2
Dcakd	Dephospho-CoA kinase domain-containing protein	*Hdhd3*	Haloacid dehalogenase-like hydrolase domain-containing protein 3	*Nectin1*	Nectin-1
Dcp1a	mRNA-decapping enzyme 1A	*Hebp2*	Heme-binding protein 2	*Nek7*	Serine/threonine-protein kinase Nek7
Dcps	m7GpppX diphosphatase	*Hivep2*	Transcription factor HIVEP2	*Nelfcd*	Negative elongation factor D
Dek	Protein DEK	*Hmgn5*	High mobility group nucleosome-binding domain-containing protein 5	*Nfib*	Nuclear factor 1 B-type
Des	Desmin	*Inpp5b*	Type II inositol 1,4,5-trisphosphate 5-phosphatase	*Nid1*	Nidogen-1
Dgki	Diacylglycerol kinase iota	*Ints3*	Integrator complex subunit 3	*Nlk*	Serine/threonine-protein kinase NLK
Dhrs1	Dehydrogenase/reductase SDR family member 1	*Iqsec3*	IQ motif and SEC7 domain-containing protein 3	*Nop53*	Ribosome biogenesis protein NOP53
Dhx32	Putative pre-mRNA-splicing factor ATP-dependent RNA helicase DHX32	*Jph1*	Junctophilin-1	*Nptn*	Neuroplastin
Dhx36	ATP-dependent DNA/RNA helicase DHX36	*Jph3*	Junctophilin-3	*Nrbp1*	Nuclear receptor-binding protein
Dip2a	Disco-interacting protein 2 homolog A	*Kcna1*	Potassium voltage-gated channel subfamily A member 1	*Ntmt1*	N-terminal Xaa-Pro-Lys N-methyltransferase 1

Dip2b	Disco-interacting protein 2 homolog B	*Kcna2*	Potassium voltage-gated channel subfamily A member 2	*Nudt10*	Diphosphoinositol polyphosphate phosphohydrolase 3-
Dipk2a	Divergent protein kinase domain 2A	*Kcnab2*	Voltage-gated potassium channel subunit beta-2	*Nufip2*	FMR1-interacting protein NUFIP2
Dkk3	Dickkopf-related protein 3	*Kcnip3*	Calsenilin isoform 4	*Otub2*	Ubiquitin thioesterase OTUB2
Dlgap4	Disks large-associated protein 4	*Kiaa0319*	Dyslexia-associated protein KIAA0319 homolog	*P4ha1*	Prolyl 4-hydroxylase subunit alpha-1
Dmkn	Dermokine	*Kiaa0513*	Uncharacterized protein KIAA0513	*Pafah1b2*	Platelet-activating factor acetylhydrolase IB subunit alpha2
Dnaja3	DnaJ homolog subfamily A member 3,	*Kif1b*	Kinesin-like protein KIF1B	*Pank1*	Pantothenate kinase 1
Dnm2	Dynamin-2	*Kirrel3*	Kin of IRRE-like protein 3	*Pcyox1l*	Prenylcysteine oxidase 1-like
Dnmt1	DNA (cytosine-5)-methyltransferase 1	*Klc4*	Kinesin light chain 4	*Pde8a*	High affinity cAMP-specific and IBMX-insensitive 3',5'-cyclic phosphodiesterase 8A
Dnph1	5-hydroxymethyl-dUMP N-hydrolase	*Klhdc10*	Kelch domain-containing protein 10	*Pdia4*	Protein disulfide-isomerase A4
Dph5	Diphthine methyl ester synthase	*Klhl22*	Kelch-like protein 22	*Pds5a*	Sister chromatid cohesion protein PDS5
Dpysl4	Dihydropyrimidinase-related protein 4	*L1cam*	Neural cell adhesion molecule L1	*Pex6*	Peroxisomal ATPase PEX6
Drd1	D(1A) dopamine receptor	*Lims1*	LIM and senescent cell antigen-like-containing domain protein 1	*Phaf1*	Phagosome assembly factor 1
Dynll1	Dynein light chain 1, cytoplasmic	*Lrrc49*	Leucine-rich repeat-containing protein 49	*Phkg2*	Phosphorylase b kinase gamma catalytic chain, liver/testis isoform
Ears2	Nondiscriminating glutamyl-tRNA synthetase EARS2,	*Lrrc4c*	Leucine-rich repeat-containing protein 4C	*Phyhd1*	Phytanoyl-CoA dioxygenase domain-containing protein 1
Ecel1	Endothelin-converting enzyme-like 1	*Maged1*	Melanoma-associated antigen D1	*Pin4*	Peptidyl-prolyl cis-trans isomerase NIMA-interacting 4
Echdc1	Ethylmalonyl-CoA decarboxylase	*Map1a*	Microtubule-associated protein 1A	*Plekhb1*	Pleckstrin homology domain-containing family B member 1
Ednrb	Endothelin receptor type B	*Mblac2*	Acyl-coenzyme A thioesterase MBLAC2	*Pmpcb*	Mitochondrial-processing peptidase
Eef1a1	Elongation factor 1-alpha 1	*Mboat7*	Membrane-bound acylglycerophosphatidyl inositol O-acyltransferase	*Pnp*	Purine nucleoside phosphorylase
Eef2k	Eukaryotic elongation factor 2 kinase	*Mdga2*	MAM domain-containing glycosylphosphatidylino	*Pofut1*	GDP-fucose protein O-fucosyltransferase 1

Efnb1	Ephrin-B1	*Mea1*	Male-enhanced antigen	*Ppm1e*	Protein phosphatase 1E
Eif6	Eukaryotic translation initiation factor 6	*Med15*	Mediator of RNA polymerase II transcription subunit 15	*Ppp1cb*	Serine/threonine-protein phosphatase PP1-beta catalytic subunit
Elapor1	Endosome/lysosome-associated apoptosis and autophagy regulator 1	*Memo1*	Protein MEMO1	*Prpf38b*	Pre-mRNA-splicing factor 38B
Endog	Endonuclease G, mitochondrial	*Mest*	Mesoderm-specific transcript protein	*Psen1*	Presenilin-1
Eno2	Gamma-enolase	*Metap1*	Methionine aminopeptidase 1	*Ptgfrn*	Prostaglandin F2 receptor negative
Enpp1	Ectonucleotide pyrophosphatase/phosphodiesterase family	*Mien1*	Migration and invasion enhancer 1	*Ptp4a3*	Protein tyrosine phosphatase type IVA 3
Ensa	Alpha-endosulfine	*Mrpl27*	Large ribosomal subunit protein bL27m	*Pym1*	Partner of Y14 and mago
Epb41	Protein 4.1	*Mrpl34*	Large ribosomal subunit protein bL34m	*Rab5if*	GEL complex subunit OPTI
Ergic1	Endoplasmic reticulum-Golgi intermediate compartment protein 1	*Mrpl43*	Large ribosomal subunit protein mL43	*Ranbp1*	Ran-specific GTPase-activating protein
Esd	S-formylglutathione hydrolase	*Mrps5*	Small ribosomal subunit protein uS5m	*Rbbp5*	Retinoblastoma-binding protein 5
Etfrf1	Electron transfer flavoprotein regulatory factor 1	*Mrtfa*	Myocardin-related transcription factor A	*Rbfox1*	RNA binding protein fox-1 homolog 1
Etnppl	Ethanolamine-phosphate phospho-lyase	*Mt-Cyb*	Cytochrome b	*Rbp1*	AT-rich interactive domain-containing protein 4A
Exosc10	Exosome complex component 10	*Mtnd4*	NADH-ubiquinone oxidoreductase chain 4	*Rdh14*	Retinol dehydrogenase 14
Fabp7	Fatty acid-binding protein, brain	*Mvd*	Diphosphomevalonate decarboxylase	*Reep2*	Receptor expression-enhancing protein 2
Fam13c	Protein FAM13C	*Mvk*	Mevalonate kinase	*Rimoc1*	RAB7A-interacting MON1-CCZ1 complex subunit 1
Fam171a2	Protein FAM171A2	*Mvp*	V-type proton ATPase 16 kDa proteolipid	*Rmdn2*	Regulator of microtubule dynamics
Farp1	FERM, ARHGEF and pleckstrin domain-containing protein 1	*Mycbp2*	E3 ubiquitin-protein ligase MYCBP2	*Rnf112*	RING finger protein 112
Fcho1	F-BAR domain only protein 1	*Myl1*	Myosin light chain 1/3, skeletal muscle isoform	*Rnf25*	E3 ubiquitin-protein ligase RNF25
Fgf13	Fibroblast growth factor 13	*Naa25*	N-alpha-acetyltransferase 25,	*Rock1*	Rho-associated protein kinase 1
Fhip2a	FHF complex subunit HOOK interacting protein 2A	*Napa*	Alpha-soluble NSF attachment protein	*Rpl31*	Large ribosomal subunit protein eL31
Fip1l1	Pre-mRNA 3'-end-processing factor FIP1	*Ncln*	BOS complex subunit NCLN	*Rpl7*	Large ribosomal subunit protein uL30

Flna	Filamin-A	*Ndor1*	NADPH-dependent diflavin oxidoreductase	*Rpl8*	Large ribosomal subunit protein uL2
Flnc	Filamin-C	*Ndrg1*	Protein NDRG1	*Rps26*	Small ribosomal subunit protein eS26
Foxo1	Forkhead box protein O1	*Ndufa9*	NADH dehydrogenase [ubiquinone] 1 alpha subcomplex subunit 9, mitochondrial	*Rrm2b*	Ribonucleoside-diphosphate reductase subunit M2 B
Frmd4b	FERM domain-containing protein 4B	*Nefm*	Neurofilament medium polypeptide	*Rybp*	RING1 and YY1-binding protein
Frrs1l	DOMON domain-containing protein FRRS1L	*Nfia*	Nuclear factor 1 A-type	*Scap*	Sterol regulatory element-binding protein cleavage-activating
Ftl1	Ferritin light chain 1	*Ngly1*	Peptide-N(4)-(N-acetyl-beta-glucosaminyl)asparagine amidase	*Scn3b*	Sodium channel regulatory subunit beta-3
Fundc1	FUN14 domain-containing protein 1	*Nipsnap3b*	Protein NipSnap homolog 3B	*Scoc*	Short coiled-coil protein
Fundc2	FUN14 domain-containing protein 2	*Nlrx1*	NLR family member X1	*Scrg1*	Scrapie-responsive protein 1
Gab1	GRB2-associated-binding protein 1	*Nop56*	Nucleolar protein 56	*Serpinc1*	Antithrombin-III
Gabra4	Gamma-aminobutyric acid receptor subunit	*Nrip2*	Nuclear receptor-interacting protein 2	*Sh3bp4*	SH3 domain-binding protein 4
Gabrb2	Gamma-aminobutyric acid receptor subunit	*Nrxn1*	Neurexin-1-beta	*Shisa4*	Protein shisa-4
Gabrd	Gamma-aminobutyric acid receptor subunit	*Nt5c3a*	Cytosolic 5'-nucleotidase 3A	*Shroom4*	Protein Shroom4
Ganc	Neutral alpha-glucosidase C	*Nt5dc3*	5'-nucleotidase domain-containing protein 3	*Slc25a15*	Mitochondrial ornithine transporter 1
Gap43	Neuromodulin	*Nt5m*	5'(3')-deoxyribonucleotidase, mitochondrial	*Slc25a21*	Mitochondrial 2-oxodicarboxylate carrier
Gbp2	Guanylate-binding protein 2	*Nudt3*	Diphosphoinositol polyphosphate phosphohydrolase 1	*Slc25a3*	Solute carrier family 25 member 3
Gdpd5	Glycerophosphodiester phosphodiesterase domain-containing	*Nudt4*	Diphosphoinositol polyphosphate phosphohydrolase 2	*Slc25a4*	ADP/ATP translocase 1
Gfap	Glial fibrillary acidic protein	*Numb*	Protein numb homolog	*Slc25a42*	Mitochondrial coenzyme A transporter
Gjb6	Gap junction beta-6 protein	*Nup188*	Nucleoporin NUP188	*Slc38a1*	Sodium-coupled neutral amino acid symporter 1
Gjc2	Gap junction gamma-2 protein	*Nxph1*	Neurexophilin-1	*Slc8a2*	Sodium/calcium exchanger 2
Glrx2	Glutaredoxin-2, mitochondrial	*Osbpl10*	Oxysterol-binding protein-related protein	*Slc8a3*	Sodium/calcium exchanger 3
Gnal	Guanine nucleotide-binding protein G(olf)	*Oxa1l*	Mitochondrial inner membrane protein	*Slc9a1*	Sodium/hydrogen exchanger 1

Gnb5	Guanine nucleotide-binding protein subunit	*Oxr1*	Oxidation resistance protein 1	*Snrpf*	Small nuclear ribonucleoprotein F
Gng12	Guanine nucleotide-binding protein G(I)/G(S)/G(O) subunit	*Padi2*	Protein-arginine deiminase type-2	*Snx10*	Sorting nexin-10
Gng4	Guanine nucleotide-binding protein G(I)/G(S)/G(O) subunit	*Pag1*	Phosphoprotein associated with glycosphingolipid-	*Snx24*	Sorting nexin-24
Gpcpd1	Glycerophosphocholine phosphodiesterase GPCPD1	*Pck2*	Phosphoenolpyruvate carboxykinase [GTP], mitochondrial	*Ssu72*	RNA polymerase II subunit A C-terminal domain phosphatase SSU72
Gpr158	Metabotropic glycine receptor	*Pcmtd1*	Protein-L-isoaspartate O-methyltransferase domain-containing	*Stk38*	Serine/threonine-protein kinase 38
Gprin3	G protein-regulated inducer of neurite	*Pcnp*	PEST proteolytic signal-containing nuclear	*Tacc2*	Transforming acidic coiled-coil-containing
Gramd1a	Protein Aster-A	*Pcp4*	Calmodulin regulator protein PCP4	*Tgm2*	Protein-glutamine gamma-
Grb10	Growth factor receptor-bound protein 10	*Pcsk1*	Neuroendocrine convertase 1	*Tgoln1*	Trans-Golgi network integral membrane protein 1
Grem2	Gremlin-2	*Pdcl3*	Phosducin-like protein 3	*Tiprl*	TIP41-like protein
Grik5	Glutamate receptor ionotropic, kainate 5	*Pdk1*	[Pyruvate dehydrogenase (acetyl-transferring)] kinase isozyme 1,	*Tmem169*	Transmembrane protein 169
Grk5	G protein-coupled receptor kinase 5	*Pdk3*	[Pyruvate dehydrogenase (acetyl-transferring)] kinase isozyme 3,	*Tmem177*	Transmembrane protein 177
Grm1	Metabotropic glutamate receptor 1	*Pecr*	Peroxisomal trans-2-enoyl-CoA reductase	*Tmem33*	Transmembrane protein 33
Gtpbp4	GTP-binding protein 4	*Pex13*	Peroxisomal membrane protein PEX13	*Tmem62*	Transmembrane protein 62
Gucy1b1	Guanylate cyclase soluble subunit beta-1	*Pex19*	Peroxisomal biogenesis factor 19	*Tnfrsf21*	Tumor necrosis factor receptor superfamily member 21
H1-2	Histone H1.2	*Phf24*	PHD finger protein 24	*Tor1a*	Torsin-1A
H2-K1	H-2 class I histocompatibility antigen, K-Q alpha	*Phgdh*	D-3-phosphoglycerate dehydrogenase	*Tsn*	Translin
H2bc12	Histone H2B type 1-K	*Pip4k2c*	Phosphatidylinositol 5-phosphate 4-kinase type-2 gamma	*Tsnax*	Translin-associated protein X
Hacd3	Very-long-chain (3R)-3-hydroxyacyl-CoA dehydratase 3	*Pja2*	E3 ubiquitin-protein ligase Praja-2	*Ttr*	Transthyretin
Hap1	Huntingtin-associated protein 1	*Plcb1*	1-phosphatidylinositol 4,5-bisphosphate phosphodiesterase beta-	*Tubg1*	Tubulin gamma-1 chain

Hapln3	Hyaluronan and proteoglycan link	*Pmm2*	Phosphomannomutase 2	*Tubgcp2*	Gamma-tubulin complex component 2
Hcn3	Potassium/sodium hyperpolarization-activated cyclic nucleotide-gated	*Polr2e*	DNA-directed RNA polymerases I, II, and III subunit RPABC1	*Ube2q1*	Ubiquitin-conjugating enzyme E2 Q1
Hdhd5	Haloacid dehalogenase-like hydrolase domain-containing 5	*Polr2i*	DNA-directed RNA polymerase II subunit RPB9	*Ubl5*	Ubiquitin-like protein 5
Hepacam	Hepatic and glial cell adhesion molecule	*Ppat*	Amidophosphoribosyltransferase	*Ubqln2*	Ubiquilin-2
Heph	Hephaestin	*Ppip5k2*	Inositol hexakisphosphate and diphosphoinositol-	*Ubxn4*	UBX domain-containing protein 4
Hexb	Beta-hexosaminidase subunit beta	*Ppp1r9b*	Neurabin-2	*Was*	Actin nucleation-promoting factor WAS
Hint3	Adenosine 5'-monophosphoramidase HINT3	*Ppp3ca*	Protein phosphatase 3 catalytic subunit alpha	*Wfs1*	Wolframin
Hk2	Hexokinase-2	*Ppp3cc*	Serine/threonine-protein phosphatase 2B catalytic subunit gamma isoform	*Xpo6*	Exportin-6
Hltf	Helicase-like transcription factor	*Ppp3r1*	Calcineurin subunit B type 1	*Ybx1*	Y-box-binding protein 1
Hmga1	High mobility group protein HMG-I/HMG-Y	*Ppp4c*	Serine/threonine-protein phosphatase 4 catalytic subunit	*Yod1*	Ubiquitin thioesterase OTU1
Hmgb1	High mobility group protein B1	*Ppwd1*	Peptidylprolyl isomerase domain and WD repeat-containing protein 1	*Ythdf2*	YTH domain-containing family protein 2
Hmgb2	High mobility group protein B2	*Praf2*	PRA1 family protein 2	*Ywhah*	14-3-3 protein eta
Hmgcs1	Hydroxymethylglutaryl-CoA synthase,	*Preb*	Guanine nucleotide-exchange factor SEC12	*Zcchc18*	Zinc finger CCHC domain-containing
Hnrnpa0	Heterogeneous nuclear ribonucleoprotein A0	*Prkaa1*	5'-AMP-activated protein kinase catalytic subunit alpha-1		
Hsdl1	Inactive hydroxysteroid dehydrogenase-like protein 1	*Prkra*	Interferon-inducible double-stranded RNA-dependent protein kinase activator A		
Hspa2	Heat shock-related 70 kDa protein 2	*Prpf40a*	Pre-mRNA-processing factor 40 homolog A		
Hspb11	Intraflagellar transport protein 25 homolog	*Prps1*	Ribose-phosphate pyrophosphokinase 1		
Hspe1	10 kDa heat shock protein, mitochondrial	*Prr7*	Proline-rich protein 7		
Htr4	5-hydroxytryptamine receptor 4	*Purg*	Purine-rich element-binding protein gamma		

Idh3a	Isocitrate dehydrogenase [NAD] subunit alpha, mitochondrial	*Pxk*	PX domain-containing protein kinase-like protein
Ifit3	Interferon-induced protein with tetratricopeptide repeats	*Pxn*	Paxillin
Ift81	Intraflagellar transport protein 81 homolog	*Rab11b*	Ras-related protein Rab-11B
Igfbp4	Insulin-like growth factor-binding protein 4	*Rab25*	Ras-related protein Rab-25
Igfbp5	Insulin-like growth factor-binding protein 5	*Rab2b*	Ras-related protein Rab-2B
Ina	Alpha-internexin	*Rab3ip*	Rab-3A-interacting
Inf2	Inverted formin-2	*Rad54l2*	Helicase ARIP4
Inka2	PAK4-inhibitor INKA2	*Rae1*	mRNA export factor
Ipo7	Importin-7	*Rc3h1*	Roquin-1
Irgm1	Immunity-related GTPase family M protein 1	*Rims2*	Regulating synaptic membrane exocytosis protein 2
Jak3	Tyrosine-protein kinase JAK3	*Rmdn1*	Regulator of microtubule dynamics
Jcad	Junctional cadherin 5-associated protein	*Rnaset2a*	Ribonuclease T2-A
Josd2	Josephin-2	*Rock2*	Rho-associated protein kinase 2
Kcna3	Potassium voltage-gated channel subfamily A member 3	*Rpl15*	Large ribosomal subunit protein eL15
Kcnab3	Voltage-gated potassium channel subunit beta-3	*Rpl18a*	Large ribosomal subunit protein eL20
Kcnb1	Potassium voltage-gated channel subfamily B member 1	*Rpl22l1*	Ribosomal protein eL22-like 1
Kcnd2	A-type voltage-gated potassium channel KCND2	*Rtn3*	Reticulon-3
Kcnip2	A-type potassium channel modulatory protein KCNIP2	*S100a1*	Protein S100-A1
Kctd4	BTB/POZ domain-containing protein KCTD4	*Sanbr*	SANT and BTB domain regulator of class switch recombination
Kdm1a	Lysine-specific histone demethylase 1A	*Scn4b*	Sodium channel regulatory subunit beta-
Kdsr	3-ketodihydrosphingosine	*Scn8a*	Sodium channel protein type 8 subunit alpha
Kiaa1671	Uncharacterized protein KIAA1671	*Sdf2*	Stromal cell-derived factor 2
Kif21b	Kinesin-like protein KIF21B	*Sec13*	Protein SEC13 homolog

Klc1	Kinesin light chain 1	*Sec61a1*	Protein transport protein Sec61 subunit alpha isoform 1								
Klhl20	Kelch-like protein 20	*Sec61a2*	Protein transport protein Sec61 subunit alpha isoform 2								
Kptn	KICSTOR complex protein kaptin	*Sema4a*	Semaphorin-4A								
Lamp2	Lysosome-associated membrane glycoprotein	*Sema4f*	Semaphorin-4F								
Larp7	La-related protein 7	*Septin4*	Septin-4								
Lcp1	Plastin-2	*Serpinb1a*	Leukocyte elastase inhibitor A								
Lemd2	LEM domain-containing protein 2	*Sf3b4*	Splicing factor 3B subunit 4								
Lgals1	Galectin-1	*Sgtb*	Small glutamine-rich tetratricopeptide repeat-containing protein beta								
Lgi3	Leucine-rich repeat LGI family member 3	*Sh3gl1*	Endophilin-A2								
Lingo1	Leucine-rich repeat and immunoglobulin-like domain-containing nogo receptor-interacting protein 1	*Sh3gl2*	Endophilin-A1								
Lingo3	Leucine-rich repeat and immunoglobulin-like domain-containing nogo receptor-interacting protein 3	*Sh3glb2*	Endophilin-B2								
Lipa	Lysosomal acid lipase/cholesteryl ester hydrolase	*Shd*	SH2 domain-containing adapter protein D								
Lmna	Prelamin-A/C	*Slc12a2*	Solute carrier family 12 member 2								
Lmnb2	Lamin-B2	*Slc17a7*	Vesicular glutamate transporter 1								
Lnpep	Leucyl-cystinyl aminopeptidase	*Slc24a2*	Sodium/potassium/calcium exchanger 2								
Lrrc58	Leucine-rich repeat-containing protein 58	*Slc25a10*	Mitochondrial dicarboxylate carrier								
Lsmem1	Leucine-rich single-pass membrane protein 1	*Slc25a14*	Brain mitochondrial carrier protein 1								
Manf	Mesencephalic astrocyte-derived	*Slc26a6*	Solute carrier family 26 member 6								
Map2k2	Dual specificity mitogen-activated protein kinase kinase 2	*Slc30a3*	Probable proton-coupled zinc antiporter SLC30A3								
Map3k13	Mitogen-activated protein kinase kinase	*Slc4a1*	Band 3 anion transport protein								

Mapk3	Mitogen-activated protein kinase 3	*Slc8a1*	Sodium/calcium exchanger 1
Marcks	Myristoylated alanine-rich C-kinase substrate	*Slmap*	Sarcolemmal membrane-associated
Matk	Megakaryocyte-associated tyrosine-	*Smad4*	Mothers against decapentaplegic
Mcat	Malonyl-CoA-acyl carrier protein transacylase, mitochondrial	*Smarcal1*	SWI/SNF-related matrix-associated actin-dependent regulator of chromatin subfamily A-
Mcm3ap	Germinal-center associated nuclear	*Sncb*	Beta-synuclein
Mcu	Calcium uniporter protein, mitochondrial	*Snrpg*	Small nuclear ribonucleoprotein G
Me2	Transcription factor E2-alpha	*Snu13*	NHP2-like protein 1
Meak7	MTOR-associated protein MEAK7	*Snx6*	Sorting nexin-6
Mef2c	Myocyte-specific enhancer factor 2C	*Sorbs3*	Vinexin
Micos13	MICOS complex subunit MIC13	*Spg21*	Maspardin
Miga2	Mitoguardin 2	*Sppl2b*	Signal peptide peptidase-like 2B
Mindy1	Ubiquitin carboxyl-terminal hydrolase	*Srsf2*	Serine/arginine-rich splicing factor 2
Mlc1	Membrane protein MLC1	*St8sia3*	Alpha-N-acetylneuraminate alpha-2,8-sialyltransferase
Mon1b	Vacuolar fusion protein MON1 homolog B	*Stau1*	Double-stranded RNA-binding protein Staufen homolog 1
Morn4	MORN repeat-containing protein 4	*Stx1a*	Syntaxin-1A
Mrfap1	MORF4 family-associated protein 1	*Sumo2*	Small ubiquitin-related modifier 2
Mrpl13	Large ribosomal subunit protein uL13m	*Syn1*	Synapsin-1
Mrpl19	Large ribosomal subunit protein bL19m	*Tdrp*	Testis development-related protein
Mrpl44	Large ribosomal subunit protein mL44	*Tenm3*	Teneurin-3
Mrps14	Small ribosomal subunit protein uS14m	*Them6*	Protein THEM6
Msh2	DNA mismatch repair protein Msh2	*Timm23*	Mitochondrial import inner membrane translocase subunit
Msn	Moesin	*Tjp1*	Tight junction protein 1
Mtatp8	ATP synthase F(0) complex subunit 8	*Tmem198*	Transmembrane protein 198

Mterf2	Transcription termination factor 2,	*Tmem205*	Transmembrane protein 205
Mtmr12	Myotubularin-related protein 12	*Tmem63b*	Mechanosensitive cation channel TMEM63B
Mtrex	Exosome RNA helicase MTR4	*Tmem88b*	Transmembrane protein 278
Myl4	Myosin light chain 4	*Tpbg*	Trophoblast
Myo1b	Unconventional myosin-Ib	*Tppp*	Tubulin polymerization-promoting protein
Naa50	N-alpha-acetyltransferase 50	*Trappc9*	Trafficking protein particle complex subunit
Naca	Nascent polypeptide-associated complex subunit alpha	*Trip4*	Activating signal cointegrator 1
Nap1l3	Nucleosome assembly protein 1-like 3	*Tsc22d3*	TSC22 domain family protein 3
Naprt	Nicotinate phosphoribosyltransfera	*Ttc3*	E3 ubiquitin-protein ligase TTC3
Natd1	Protein NATD1	*Tut7*	Terminal uridylyltransferase 7
Ncbp2	Nuclear cap-binding protein subunit 2	*Uap1*	UDP-N-acetylhexosamine
Ndrg4	Protein NDRG4	*Unc80*	Protein unc-80 homolog
Ndufa10	NADH dehydrogenase [ubiquinone] 1 alpha subcomplex subunit 10, mitochondrial	*Urm1*	Ubiquitin-related modifier 1
Ndufab1	Acyl carrier protein, mitochondrial	*Vps26c*	Vacuolar protein sorting-associated
Ndufb6	NADH dehydrogenase [ubiquinone] 1 beta subcomplex subunit 6	*Vps8*	Vacuolar protein sorting-associated protein 8 homolog
Nebl	Nebulette	*Wdr37*	WD repeat-containing protein 37
Nek3	Serine/threonine-protein kinase Nek3	*Wdr6*	tRNA (34-2'-O)-methyltransferase regulator WDR6
Ngef	Ephexin-1	*Xpr1*	Solute carrier family 53 member 1
Niban2	Protein Niban 2	*Yes1*	Tyrosine-protein kinase Yes
Nlgn3	Neuroligin-3	*Zdhhc8*	Palmitoyltransferase ZDHHC8
Nme3	Nucleoside diphosphate kinase 3	*Zmpste24*	CAAX prenyl protease 1 homolog
Nmral1	NmrA-like family domain-containing	*Znf830*	Zinc finger protein 830
Noct	Nocturnin	*Zyg11b*	Protein zyg-11 homolog
Nras	GTPase NRas		
Nrgn	Neurogranin		
Nrp2	Neuropilin-2		

Nrxn3	Neurexin-3-beta					
Nsg2	Neuronal vesicle trafficking-associated protein 2					
Ntrk3	NT-3 growth factor receptor					
Nucb2	Nucleobindin-2					
Nucks1	Nuclear ubiquitous casein and cyclin-dependent kinase					
Nudt12	NAD-capped RNA hydrolase NUDT12					
Nudt7	Peroxisomal coenzyme A diphosphatase					
Nwd2	NACHT and WD repeat domain-containing protein 2					
Ogfod1	Prolyl 3-hydroxylase OGFOD1					
Olfm2	Noelin-2					
Olfml3	Olfactomedin-like					
Osbpl8	Oxysterol-binding protein-related protein 8					
P2rx7	P2X purinoceptor 7					
Pacsin1	Protein kinase C and casein kinase substrate in neurons protein 1					
Paip1	Polyadenylate-binding protein-interacting protein 1					
Pald1	Paladin					
Pamr1	Inactive serine protease PAMR1					
Panx2	Pannexin-2					
Parvb	Beta-parvin					
Pcm1	Pericentriolar material 1 protein					
Pcna	Proliferating cell nuclear antigen					
Pcnx2	Pecanex-like protein 2					
Pdcd4	Programmed cell death protein 4					
Pde10a	cAMP and cAMP-inhibited cGMP 3',5'-cyclic phosphodiesterase					
Pde1b	Dual specificity calcium/calmodulin-dependent 3',5'-cyclic nucleotide phosphodiesterase 1B					

Pde4a	3',5'-cyclic-AMP phosphodiesterase 4A					
Pdk2	[Pyruvate dehydrogenase (acetyl-transferring)] kinase isozyme 2,					
Pdlim4	PDZ and LIM domain protein 4					
Pdpn	Podoplanin					
Pdyn	Proenkephalin-B					
Pelp1	Proline-, glutamic acid- and leucine-rich protein					
Pgrmc2	Membrane-associated progesterone receptor component 2					
Phactr1	Phosphatase and actin regulator 1					
Phactr4	Phosphatase and actin regulator 4					
Phka1	Phosphorylase b kinase regulatory subunit alpha, skeletal muscle isoform					
Phkb	Phosphorylase b kinase regulatory subunit beta					
Phlda3	Pleckstrin homology-like domain family A					
Pianp	PILR alpha-associated neural protein					
Picalm	Phosphatidylinositol-binding clathrin					
Pigt	GPI-anchor transamidase component					
Pik3ip1	Phosphoinositide-3-kinase-interacting					
Pip4p1	Type 1 phosphatidylinositol 4,5-bisphosphate 4-					
Pitpnc1	Cytoplasmic phosphatidylinositol transfer protein 1					
Pla2g4a	Cytosolic phospholipase A2					
Pla2g7	Platelet-activating factor acetylhydrolase					
Plcd3	1-phosphatidylinositol 4,5-bisphosphate phosphodiesterase delta-					
Plcg2	1-phosphatidylinositol 4,5-bisphosphate phosphodiesterase gamma-2					

Pld1	Phospholipase D1
Plekha7	Pleckstrin homology domain-containing family A member 7
Plekhg5	Pleckstrin homology domain-containing family G member 5
Plgrkt	Plasminogen receptor (KT)
Plin4	Perilipin-4
Pls1	Plastin-1
Pnck	Calcium/calmodulin-dependent protein kinase type 1B
Polr2f	DNA-directed RNA polymerases I, II, and III subunit RPABC2
Ppp1cc	Serine/threonine-protein phosphatase PP1-gamma catalytic subunit
Ppp1r16b	Protein phosphatase 1 regulatory inhibitor subunit 16B
Ppp2r2a	Serine/threonine-protein phosphatase 2A 55 kDa regulatory subunit B alpha isoform
Ppp4r1	Serine/threonine-protein phosphatase 4 regulatory subunit 1
Ppp4r4	Serine/threonine-protein phosphatase 4 regulatory subunit 4
Prkag1	5'-AMP-activated protein kinase subunit
Prkar1b	cAMP-dependent protein kinase type I-beta regulatory subunit
Prkar2b	cAMP-dependent protein kinase type II-beta regulatory subunit
Prkcb	Protein kinase C beta
Prmt8	Protein arginine N-methyltransferase 8
Prom1	Prominin-1
Prrt1	Proline-rich transmembrane protein 1
Psma2	Proteasome subunit alpha type-2
Psme1	Proteasome activator complex subunit 1

Psme2	Proteasome activator complex subunit 2						
Ptgr1	Prostaglandin reductase						
Ptgs1	Prostaglandin G/H synthase 1						
Ptk7	Inactive tyrosine-protein kinase 7						
Ptms	Parathymosin						
Ptpa	Serine/threonine-protein phosphatase 2A						
Ptpn5	Tyrosine-protein phosphatase non-receptor type 5						
Ptpn6	Tyrosine-protein phosphatase non-receptor type 6						
Pum3	Pumilio homolog 3						
Pus10	tRNA pseudouridine synthase Pus10						
Rab14	Ras-related protein Rab-						
Rab31	Ras-related protein Rab-						
Rab34	Ras-related protein Rab-						
Rab3a	Ras-related protein Rab-3A						
Rab3c	Ras-related protein Rab-3C						
Rab8b	Ras-related protein Rab-8B						
Rac1	Ras-related C3 botulinum toxin						
Rac2	Ras-related C3 botulinum toxin						
Ralgps2	Ras-specific guanine nucleotide-releasing factor RalGPS2						
Ralyl	RNA-binding Raly-like protein						
Rap1gap	Rap1 GTPase-activating protein 1						
Rasd2	GTP-binding protein						
Rasgrp2	RAS guanyl-releasing protein 2						
Raver1	Ribonucleoprotein PTB-binding 1						
Rbm39	RNA-binding protein 39						
Rbx1	E3 ubiquitin-protein ligase RBX1						
Rcan1	Calcipressin-1						
Rcc2	Protein RCC2						
Rem2	GTP-binding protein REM 2						

Rfc1	Replication factor C subunit 1					
Rgs8	Regulator of G-protein signaling 8					
Rgs9	Regulator of G-protein signaling 9					
Rhob	Rho-related GTP-binding protein RhoB					
Rhof	Rho-related GTP-binding protein RhoF					
Rnf157	E3 ubiquitin ligase					
Rpl23	Large ribosomal subunit protein uL14					
Rpl23a	Large ribosomal subunit protein uL23					
Rpl27a	Large ribosomal subunit protein uL15					
Rpl28	Large ribosomal subunit protein eL28					
Rpl37	Large ribosomal subunit protein eL37					
Rplp0	Large ribosomal subunit protein uL10					
Rplp2	Large ribosomal subunit protein P2					
Rps9	Small ribosomal subunit protein uS4					
Rragb	Ras-related GTP-binding protein B					
Rras2	Ras-related protein R-Ras2					
Rsu1	Ras suppressor protein 1					
Rtn4rl2	Reticulon-4 receptor-					
Ryr3	Ryanodine receptor 3					
S100a4	Protein S100-A4					
S100a6	Protein S100-A6					
Sar1b	Small COPII coat GTPase SAR1B					
Scaf8	SR-related and CTD-associated factor 8					
Sccpdh	Saccharopine dehydrogenase-like oxidoreductase					
Scn1b	Sodium channel regulatory subunit beta-					
Sec61b	Protein transport protein Sec61 subunit beta					
Sema7a	Semaphorin-7A					
Septin6	Septin-6					

Setd1a	Histone-lysine N-methyltransferase SETD1A					
Setd3	Actin-histidine N-methyltransferase					
Sez6	Seizure protein 6					
Sgpl1	Sphingosine-1-phosphate lyase 1					
Sgpp1	Sphingosine-1-phosphate phosphatase 1					
Sh3bgrl3	SH3 domain-binding glutamic acid-rich-like protein 3					
Sh3gl3	Endophilin-A3					
Shisa7	Protein shisa-7					
Shoc2	Leucine-rich repeat protein SHOC-2					
Sidt1	SID1 transmembrane family member 1					
Sigmar1	Sigma non-opioid intracellular receptor 1					
Slc10a4	Sodium/bile acid cotransporter 4					
Slc18a3	Vesicular acetylcholine transporter					
Slc1a3	Excitatory amino acid transporter 1					
Slc25a18	Mitochondrial glutamate carrier 2					
Slc25a33	Solute carrier family 25 member 33					
Slc25a35	Solute carrier family 25 member 35					
Slc35g2	Solute carrier family 35 member G2					
Slc6a9	Sodium- and chloride-dependent glycine transporter 1					
Slc7a5	Large neutral amino acids transporter small					
Slc9a6	Sodium/hydrogen exchanger 6					
Smg5	Nonsense-mediated mRNA decay factor SMG5					
Smpd3	Sphingomyelin phosphodiesterase 3					
Smpd4	Sphingomyelin phosphodiesterase 4					
Smu1	WD40 repeat-containing protein SMU1					

Snap25	Synaptosomal-associated protein 25					
Sncg	Gamma-synuclein					
Snrpn	Small nuclear ribonucleoprotein-associated protein N					
Snx19	Sorting nexin-19					
Sod1	Superoxide dismutase [Cu-Zn]					
Sox8	Transcription factor SOX-8					
Sparc	SPARC					
Spats2	Spermatogenesis-associated serine-rich protein 2					
Spcs1	Signal peptidase complex subunit 1					
Speg	Striated muscle-specific serine/threonine-protein kinase					
Spock3	Testican-3					
Sra1	Cytoplasmic FMR1-interacting protein 1					
Srgap1	SLIT-ROBO Rho GTPase-activating protein 1					
Ssh3	Protein phosphatase Slingshot homolog 3					
Stam	Signal transducing adapter molecule 1					
Stat1	Signal transducer and activator of transcription					
Stim1	Stromal interaction molecule 1					
Strbp	Spermatid perinuclear RNA-binding protein					
Strn	Striatin					
Stx3	Syntaxin-3					
Stx4	Syntaxin-4					
Suox	Sulfite oxidase, mitochondrial					
Sv2c	Synaptic vesicle glycoprotein 2C					
Syngr1	Synaptogyrin-1					
Sypl1	Synaptophysin-like protein 1					
Syt17	Synaptotagmin-17					
Taco1	Translational activator of cytochrome c oxidase					
Tagln2	Transgelin-2					
Tagln3	Transgelin-3					

Tbc1d8	TBC1 domain family member 8					
Tef	Transcriptional enhancer factor TEF-1					
Tgfa	Protransforming growth factor alpha					
Thada	tRNA (32-2'-O)-methyltransferase regulator THADA					
Thbs4	Thrombospondin-4					
Thtpa	Thiamine-triphosphatase					
Tiam2	Rho guanine nucleotide exchange factor TIAM2					
Timm10b	Mitochondrial import inner membrane translocase subunit					
Timm13	Mitochondrial import inner membrane translocase subunit					
Timm9	Mitochondrial import inner membrane translocase subunit					
Tm9sf3	Transmembrane 9 superfamily member 3					
Tmcc2	Transmembrane and coiled-coil domains protein 2					
Tmem100	Transmembrane protein 100					
Tmem106b	Transmembrane protein 106B					
Tmem229a	Transmembrane protein 229A					
Tmem38a	Trimeric intracellular cation channel type A					
Tmem68	DGAT1/2-independent enzyme synthesizing storage lipids					
Tmx3	Protein disulfide-isomerase TMX3					
Tnks	Poly [ADP-ribose] polymerase tankyrase-1					
Tomm70	Mitochondrial import receptor subunit TOM70					
Tp53bp2	Apoptosis-stimulating of p53 protein 2					
Tpi1	Triosephosphate					
Tpm4	Tropomyosin alpha-4					
Traf6	TNF receptor-associated factor 6					

Trim25	E3 ubiquitin/ISG15 ligase TRIM25					
Trmt6	tRNA (adenine(58)-N(1))-methyltransferase non-catalytic subunit					
Trp53i11	Tumor protein p53-inducible protein 11					
Tspan7	Tetraspanin-7					
Tspan9	Tetraspanin-9					
Ttll7	Tubulin polyglutamylase TTLL7					
Ttyh1	Protein tweety homolog					
Tuba1a	Tubulin alpha-1A chain					
Tubb2a	Tubulin beta-2A chain					
Tulp3	Tubby-related protein 3					
Tusc3	Dolichyl-diphosphooligosaccharide--protein glycosyltransferase					
Tut4	Terminal uridylyltransferase 4					
Txn	Thioredoxin					
Txndc17	Thioredoxin domain-containing protein 17					
Txndc9	Thioredoxin domain-containing protein 9					
Ube2i	SUMO-conjugating enzyme UBC9					
Ugdh	UDP-glucose 6-dehydrogenase					
Ugt1a1	UDP-glucuronosyltransferase 1A1					
Unc119	Protein unc-119 homolog A					
Unc45a	Protein unc-45 homolog					
Vamp3	Vesicle-associated membrane protein 3					
Vangl2	Vang-like protein 2					
Vav1	Proto-oncogene vav					
Vcl	Vinculin					
Vdac3	Non-selective voltage-gated ion channel					
Vim	Vimentin					
Vopp1	WW domain binding protein VOPP1					
Vrk3	Serine/threonine-protein kinase VRK3					
Wipf3	WAS/WASL-interacting protein family member 3					

Wipi1	WD repeat domain phosphoinositide-interacting protein 1						
Xkr5	XK-related protein 5						
Xkr9	XK-related protein 9						
Yme1l1	ATP-dependent zinc metalloprotease						
Yy1	Transcriptional repressor protein YY1						
Zbtb20	Zinc finger and BTB domain-containing protein 20						
Zdbf2	DBF4-type zinc finger-containing protein 2 homolog						
Zfp28	Zinc finger protein 28						
Zfyve16	Zinc finger FYVE domain-containing						
Zmym2	Zinc finger MYM-type protein 2						
Znf207	BUB3-interacting and GLEBS motif-containing protein						
Znf330	Zinc finger protein 330						
Znrd2	Protein ZNRD2						

Categories include "Only MPTP/A53T/MitoPark" (DEPs exclusive to each model), intersections such as "MPTP AND MitoPark NOT A53T" (shared by two models but not the third), and "MPTP AND A53T AND MitoPark" (common to all three); "MPTP" refers to the acute MPTP model, "A53T" to the 16 months A53T-α-synuclein model, and "MitoPark" to the 5 months MitoPark model.
