# Supplementary material for: Neurotoxicity, α-synuclein pathology, and mitochondrial dysfunction: A comparative study of different mouse models of Parkinson’s disease
Source: Neural Regen Res. 2025 Sep 29;21(10):5000–12. doi: 10.4103/NRR.NRR-D-25-00648 (PMC13568611; doi:10.4103/NRR.NRR-D-25-00648)
Supplement: Supplementary file 7 [file NRR-21-5000_Suppl6.pdf]

Additional Table 9. GO pathway identified from proteomic data analysis of the striatum in three PD mouse models

MPTP

ONTOLOGY	ID	Description	GeneRatio	BgRatio	Rich Factor	pvalue	p.adjust	qvalue	geneID	Count
BP	GO:0099504	synaptic vesicle cycle	28/607	227/28814	5.86	6.69153E-14	3.19654E-10	2.31456E-10	Actb/Apba1/Apba2/Brsk1/Cdh2/Cplx1/Ddc/Dgki/Dnm2/Drd1/Grik5/Nlgn3/Nrxn3/P2rx7/Pacsin1/Picalm/Pld1/Prkar1b/Prkcb/Rab3a/Sh3gl3/Slc10a4/Slc18a2/Slc18a3/Snap25/Sncg/Stx3/Th	28
BP	GO:0099003	vesicle-mediated transport in synapse	29/607	254/28814	5.42	1.77624E-13	4.24254E-10	3.07195E-10	Actb/Apba1/Apba2/Brsk1/Cdh2/Cplx1/Ddc/Dgki/Dnm2/Drd1/Grik5/Hap1/Nlgn3/Nrxn3/P2rx7/Pacsin1/Picalm/Pld1/Prkar1b/Prkcb/Rab3a/Sh3gl3/Slc10a4/Slc18a2/Slc18a3/Snap25/Sncg/Stx3/Th	29
BP	GO:0050808	synapse organization	39/607	494/28814	3.75	2.03537E-12	3.24098E-09	2.34674E-09	Ache/Actb/C1qc/Camk1/Camk2b/Cdh2/Dab2ip/Dbn1/Dbnl/Dhx36/Dip2a/Dnaja3/Drd1/Eef2k/Efnb1/Farp1/Fgf13/Flna/Gabrb2/Gap43/Itpka/Marcks/Mef2c/Ngef/Nlgn3/Nptx1/Nrp2/Nrxn3/Ntrk3/Picalm/Prrt1/Rab3a/Sez6/Shisa7/Slc9a6/Sncg/Sorbs1/	39
BP	GO:0006836	neurotransmitter transport	26/607	242/28814	5.1	1.28608E-11	1.53591E-08	1.11212E-08	Apba1/Apba2/Asic1/Brsk1/Cplx1/Ddc/Dgki/Drd1/Gfap/Grik5/Mef2c/Nrxn3/P2rx7/Pnkd/Prkcb/Rab3a/Slc10a4/Slc18a2/Slc18a3/Slc6a3/Slc6a9/Snap25/Sncg/Stx3/Sv2c	26
BP	GO:0007264	small GTPase mediated signal transduction	34/607	431/28814	3.74	5.56664E-11	5.31837E-08	3.85095E-08	Abca1/Adgrg1/Arhgef10/Arhgef2/Bcar3/Cdh2/Chn1/Dab2ip/Dbnl/Dgki/Dnaja3/Dnm2/Dnmt1/Hacd3/Nras/Nucb2/Pdpn/Phactr4/Plekhg5/Prag1/Rac1/Rac2/Ralgps2/Rap1gap/Rasgrp2/Rhob/Rhof/Rras2/Rsu1/Shoc2/Srgap1/Tiam2/Vangl2/Vav1	34
BP	GO:0007015	actin filament organization	35/607	458/28814	3.63	6.9324E-11	5.51935E-08	3.99647E-08	Actn2/Aif1l/Arhgap6/Arhgef10/Arhgef2/Arpc1b/Arpc3/Arpc5l/Coro7/Cracd/Ctnna3/Dbn1/Dbnl/Flna/Kank4/Kptn/Lcp1/Marcks/Mcu/Myo1b/Nebl/Pacsin1/Pdlim4/Phactr1/Pls1/Rac1/Rac2/Rhob/Rhof/Sirpa/Sorbs1/Ssh3/Tm	35
BP	GO:0031346	positive regulation of cell projection organization	34/607	443/28814	3.64	1.16121E-10	7.83595E-08	5.67388E-08	Ache/Camk1/Camk1d/Camk2b/Dab2ip/Dbn1/Dbnl/Dhx36/Dnm2/Dynll1/Eef1a1/Eef2k/Flna/Hap1/Itpka/Map2k2/Map3k13/Marcks/Ndrg4/Ntrk3/P2rx7/Pacsin1/Picalm/Plxnc1/Ptk7/Ptpn5/Rab8b/Rac1/Rac2/Scn1b/Sema7a/Sez6/Tiam2/Tmem106b	34
BP	GO:0007409	axonogenesis	36/607	493/28814	3.47	1.31228E-10	7.83595E-08	5.67388E-08	Ache/Actb/Aplp2/B4galt6/Brsk1/Cdh2/Cdk5r1/Chn1/Dbnl/Dip2b/Dnm2/Efnb1/Fgf13/Gap43/Map2k2/Map3k13/Mef2c/Nexn/Nlgn3/Nptx1/Nrp2/Ntrk3/Picalm/Plxna1/Rab3a/Rac1/Rac2/Scn1b/Sema7a/Slc9a6/Tiam2/Vangl2/Vasp/Vcl/Vim	36
BP	GO:0001505	regulation of neurotransmitter levels	25/607	254/28814	4.67	2.10929E-10	1.11956E-07	8.10658E-08	Ache/Apba1/Apba2/Asic1/Brsk1/Comt/Cplx1/Dgki/Drd1/Gfap/Grik5/Mef2c/Nrxn3/P2rx7/Pde1b/Pnkd/Prkcb/Rab3a/Slc18a2/Slc6a3/Slc6a9/Snap25/Sncg/Stx3/Th	25
BP	GO:0007611	learning or memory	28/607	322/28814	4.13	3.13236E-10	1.49633E-07	1.08347E-07	Adcy3/Adcy8/Asic1/B2m/Brsk1/Comt/Crebbp/Cxnd1/Dgki/Drd1/Fgf13/Mef2c/Mme/Ndrg4/Nlgn3/Nrxn3/Pde1b/Picalm/Prkar1b/Prkar2b/Prrt1/Ptgs1/Rcan1/Shisa7/Snap25/Tacr1/Th/Vdac3	28
BP	GO:1901652	response to peptide	32/607	420/28814	3.62	4.94687E-10	2.05786E-07	1.49006E-07	Ache/Adcy8/Arhgef2/Atp2b1/Bcar3/Chmp5/Ednrb/Eif6/Eppp1/Foxo1/Grb10/Jak3/Marcks/Mmk7/Nucb2/Nucks1/Oxbp8/Pdk2/Pld1/Prkcb/Rab31/Rab8b/Rac1/Rpl23/Slc25a33/Smpd3/Sorbs1/Stat1/Stat3/Syap1/Vim	32
BP	GO:0007626	locomotory behavior	25/607	265/28814	4.48	5.16943E-10	2.05786E-07	1.49006E-07	Adcy8/Apba1/Apba2/Aplp2/Cxnd1/Cxnb/Dbn1/Drd1/Emp1/Gnrn1/Hexb/Kcnd2/Olfm2/Pde1b/Rasd2/Rcan1/Sez6/Slc18a2/Slc6a3/Snap25/Sncg/Sod1/Strn/Th/Tmod1	25
BP	GO:0050890	cognition	29/607	359/28814	3.83	8.48298E-10	3.11717E-07	2.25709E-07	Adcy3/Adcy8/Asic1/B2m/Brsk1/Comt/Crebbp/Cxnd1/Dgki/Drd1/Fgf13/Mef2c/Mme/Ndrg4/Nlgn3/Nrxn3/Pde1b/Picalm/Prkar1b/Prkar2b/Prrt1/Ptgs1/Rcan1/Shisa7/Snap25/Tacr1/Th/Tmsb/Vdac	29
BP	GO:0022604	regulation of cell morphogenesis	28/607	338/28814	3.93	9.43397E-10	3.219E-07	2.33083E-07	Ache/Camk2b/Dbn1/Dbnl/Dhx36/Dnm2/Eef2k/Epb41/Flna/Hexb/Itpka/Map3k13/Marcks/Mas/Myl12b/Parvb/Pdpn/Picalm/Ptnc1/Pnrg1/Rac1/Rac2/Rcc2/Rhob/Rhof	28
BP	GO:0006091	generation of precursor metabolites and energy	32/607	436/28814	3.48	1.25094E-09	3.98364E-07	2.88463E-07	Acsl/Aldh11/Atp5pb/Bax/Cox7a1/Cox7a2/Cox7c/Eif6/Ctsa/Esrp1/Ezh1/Grb10/Hk2/Hmgb1/Idh3a/Mdh2/Ndufa10/Ndufs6/P2rx7/Pfkp1/Pkb/Ppp1cc/Sdha/Slc1a3/Slc25a18/Slc25a33/Sorbs1/Stat3/Taco1/Tpi	32
BP	GO:1990778	protein localization to cell periphery	29/607	370/28814	3.72	1.69923E-09	5.07326E-07	3.67346E-07	Actb/Actn2/Anxa2/Arf4/Bbs2/Camk2b/Cdh2/Cplx1/Epb41/Fgf13/Flna/Gpr158/Kcnb1/Nptx1/Pacsin1/Picalm/Pip5k1a/Pls1/Rab26/Rab31/Rab34/Rab3a/Rab8b/Rac1/Snap25/Sorbs1/St	29
BP	GO:0050767	regulation of neurogenesis	32/607	455/28814	3.34	3.54496E-09	9.42012E-07	6.82095E-07	Ache/Anxa2/Arhgef2/B2m/Camk2b/Dbn1/Dbnl/Dhx36/Dip2b/Eef2k/Fgf13/Gfap/Gjc2/Hap1/Hlrf/Hmgb1/Itpka/Kdm1a/Lingo1/Map2k2/Map3k13/Marcks/Mme/Ntrk3/Pcm1/Picalm/Plxnc1/Ppp1cc/Sema7a/Sox8/Tiam2/Vim	32
BP	GO:1901653	cellular response to peptide	26/607	313/28814	3.94	3.54955E-09	9.42012E-07	6.82095E-07	Actn2/Adcy8/Arhgef2/Atp2b1/Bcar3/Chmp5/Eppp1/Foxo1/Grb10/Jak3/Marcks/Nucb2/Nucks1/Oxbp8/Pdk2/Prkcb/Rab31/Rab8b/Rac1/Rpl23/Slc25a33/Smpd3/Sorbs	26
BP	GO:0072659	protein localization to plasma membrane	25/607	302/28814	3.93	7.60722E-09	1.91262E-06	1.38489E-06	Actb/Actn2/Anxa2/Arf4/Bbs2/Camk2b/Cdh2/Fgf13/Flna/Gpr158/Kcnb1/Pacsin1/Picalm/Pip5k1a/Pls1/Rab26/Rab31/Rab34/Rab3a/Rab8b/Rac1/Sorbs1/Stx	25
BP	GO:0034765	regulation of ion transmembrane transport	33/607	498/28814	3.15	8.73493E-09	2.08634E-06	1.51068E-06	Actn2/Atp2b4/Bax/Chn2/Ctn1/Dnm2/Drd1/Fgf13/Flna/Gjc2/Grik5/Hap1/Htr3/Hspa2/Kcna5/Kcnab3/Kcnb1/Kcnc1/Kcnd2/Kcnip2/Mef2c/Nlgn3/P2rx7/Pkig2/Prrt1/Pnn8/Rgs9/Scn1b/Shisa9/Slc6a9/Stim1/Tacr1/Tmem38a	33
BP	GO:0043434	response to peptide hormone	27/607	359/28814	3.57	1.48503E-08	3.28629E-06	2.37955E-06	Ache/Actn2/Adcy8/Atp2b1/Bcar3/Ednrb/Eif6/Eppp1/Foxo1/Grb10/Jak3/Marcks/Mmk7/Nucb2/Nucks1/Oxbp8/Pdk2/Pld1/Prkcb/Rab31/Rab8b/Rac1/Slc25a33/Sorbs1/	27
BP	GO:0010976	positive regulation of neuron projection development	21/607	225/28814	4.43	1.51347E-08	3.28629E-06	2.37955E-06	Camk1/Camk1d/Camk2b/Dab2ip/Dbn1/Dbnl/Dhx36/Eef2k/Flna/Hap1/Itpka/Marcks/Ndrg4/Ntrk3/Pacsin1/Ptk7/Ptpn5/Scn1b/Sez6/	21
BP	GO:0099173	postsynapse organization	20/607	207/28814	4.59	1.85768E-08	3.85931E-06	2.79374E-06	Actb/Camk2b/Cdh2/Dbn1/Dbnl/Dhx36/Dip2a/Dnaja3/Eef2k/Farp1/Gap43/Itpka/Marcks/Ngef/Nlgn3/Nptx1/	20
BP	GO:0016358	dendrite development	25/607	317/28814	3.74	2.00146E-08	3.98374E-06	2.88456E-06	Ache/Camk1/Camk1d/Camk2b/Crebbp/Dab2ip/Dbn1/Dbnl/Dhx36/Eef2k/Fgf13/Hap1/Itpka/Marcks/Mef2c/Ngef/Nlgn3/Pacsin1/Phactr1/Picalm/Rac1/Sez6/Slc9a6/St	25
BP	GO:0006887	exocytosis	29/607	414/28814	3.33	2.10582E-08	4.02381E-06	2.91357E-06	Apba1/Apba2/Bbs1/Cd63/Cdk5r1/Cplx1/Dgki/Drd1/Grik5/Hap1/Kcnb1/Lgi3/Nrxn3/P2rx7/Prkcb/Rab26/Rab31/Rab3a/Rab8b/Rac2/Scamp5/Snap25/Stx19/Stxbp6/Syngr1/Syt17/	29
BP	GO:0060998	regulation of dendritic spine development	13/607	85/28814	7.26	2.61255E-08	4.80005E-06	3.47564E-06	Camk1/Camk2b/Crebbp/Dbn1/Dbnl/Dhx36/Eef2k/Itpka/Marcks/Mef2c/Ngef/Nlgn3/	13
BP	GO:0051588	regulation of neurotransmitter transport	16/607	139/28814	5.46	4.2787E-08	7.57012E-06	5.48148E-06	Apba1/Apba2/Asic1/Cplx1/Dgki/Drd1/Gfap/Mef2c/Nrxn3/P2rx7/Pnkd/Prkcb/Rab3a/Slc10a4/Slc18a2/Sncg	16
BP	GO:0007269	neurotransmitter secretion	18/607	185/28814	4.62	8.55388E-08	1.3922E-05	1.00807E-05	Apba1/Apba2/Asic1/Brsk1/Cplx1/Dgki/Drd1/Grik5/Mef2c/Nrxn3/P2rx7/Pnkd/Prkcb/Rab3a/Slc6a9/Snap25/Sncg/Stx3	18

A53T

ONTOLOGY	ID	Description	GeneRatio	BgRatio	Rich Factor	pvalue	p.adjust	qvalue	geneID	Count
BP	GO:0042391	regulation of membrane potential	29/361	469/28814	4.94	2.11556E-12	7.73662E-09	6.01711E-09	Ank3/Atp1a1/Atp1a4/Atp2a2/Bdnf/Cacnb3/Camk2d/Cck/Celf4/Cnih2/Dlg1/Foxp1/Gabra3/Gabrb1/Hebp2/Kcna1/Kcna2/Kcnab2/Nrxn1/Ppp3ca/Rims2/Scn4b/Scn8a/Sh3gl1/Slc17a7/Slc8a1/Slmap/Stx1a/Zmpste24	29
BP	GO:0099003	vesicle-mediated transport in synapse	21/361	254/28814	6.6	1.19545E-11	2.18588E-08	1.70006E-08	Atp2a2/Cadps/Cadps2/Camk2a/Cplx1/Cplx3/Dennd1a/Gsg1l/Napa/Nrxn1/Numb/Ppp3cc/Ppp3r1/Rims2/Sh3gl1/Sh3gl2/Slc17a7/Sncb/Stx1a/Stx2/Syn1	21
BP	GO:0050808	synapse organization	27/361	494/28814	4.36	2.01405E-10	2.45512E-07	1.90946E-07	Adam10/Add2/Ank3/Apbb1/Arhgap39/Atp2b2/Bdnf/Cacnb3/Elfn1/Iqsec3/Itpka/Kirrel3/L1cam/Lrrc4c/Mdga2/Ndrg1/Nfia/Nptx1/Nrxn1/Numb/Rock2/Sema4a/Sncb/Sorbs1/Stau1/Syn1/Tpbg	27
BP	GO:0099504	synaptic vesicle cycle	18/361	227/28814	6.33	7.06499E-10	6.45917E-07	5.02358E-07	Atp2a2/Cadps/Cadps2/Camk2a/Cplx1/Cplx3/Dennd1a/Napa/Nrxn1/Ppp3cc/Rims2/Sh3gl1/Sh3gl2/Slc17a7/Sncb/Stx1a/Stx2/Syn1	18
BP	GO:1990778	protein localization to cell periphery	21/361	370/28814	4.53	1.13297E-08	8.28657E-06	6.44483E-06	Adam10/Ank3/Cacnb3/Camk2a/Camk2d/Cplx1/Dlg1/Ehd2/Epb41l2/Kcnip3/Kif1b/Nptx1/Nrxn1/Numb/Ppp1r9b/Rab26/Rock2/Slc4a1/Slmap/Sorbs1/Tpbg	21
BP	GO:0140029	exocytic process	10/361	93/28814	8.58	2.70408E-07	0.000146006	0.000113555	Cadps/Cadps2/Camk2a/Cplx1/Cplx3/Napa/Nrxn1/Rims2/Stx1a/Stx2	10
BP	GO:0006887	exocytosis	20/361	414/28814	3.86	3.4468E-07	0.000146006	0.000113555	Atp2a2/Atp9a/Cadps/Cadps2/Camk2a/Cbarp/Cplx1/Cplx3/Napa/Nrxn1/Rab11b/Rab25/Rab26/Rab2b/Rab3ip/Rabgef1/Rims2/Septin4/Stx1a/Stx2	20
BP	GO:0001508	action potential	12/361	147/28814	6.52	3.67343E-07	0.000146006	0.000113555	Ank3/Atp2a2/Cacnb3/Camk2d/Dlg1/Foxp1/Kcna1/Kcna2/Kcnab2/Scn4b/Scn8a/Slmap	12
BP	GO:0034765	regulation of ion transmembrane transport	22/361	498/28814	3.53	4.09185E-07	0.000146006	0.000113555	Ank3/Cacnb3/Calm1/Camk2d/Cbarp/Clcn2/Cnih2/Dlg1/Gsg1l/Jph3/Kcna1/Kcna2/Kcnab2/Kcnip3/Nrxn1/Plcb1/S100a1/Scn4b/Scn8a/Shisa9/Slc8a1/Slmap	22
BP	GO:1904062	regulation of cation transmembrane transport	19/361	381/28814	3.98	4.20482E-07	0.000146006	0.000113555	Ank3/Cacnb3/Calm1/Camk2d/Cbarp/Cnih2/Dlg1/Gsg1l/Jph3/Kcna1/Kcnab2/Kcnip3/Nrxn1/Plcb1/S100a1/Scn4b/Shisa9/Slc8a1/Slmap	19
BP	GO:0032412	regulation of ion transmembrane transporter activity	16/361	274/28814	4.66	4.39175E-07	0.000146006	0.000113555	Ank3/Cacnb3/Calm1/Camk2d/Cbarp/Cnih2/Dlg1/Gsg1l/Jph3/Kcna1/Nrxn1/Plcb1/S100a1/Scn4b/Shisa9/Slmap	16
BP	GO:2001257	regulation of cation channel activity	13/361	181/28814	5.73	5.26985E-07	0.000160599	0.000124905	Ank3/Cacnb3/Calm1/Cbarp/Cnih2/Dlg1/Gsg1l/Jph3/Kcna1/Nrxn1/S100a1/Shisa9/Slmap	13
BP	GO:0022898	regulation of transmembrane transporter activity	16/361	284/28814	4.5	7.07326E-07	0.000198976	0.000154753	Ank3/Cacnb3/Calm1/Camk2d/Cbarp/Cnih2/Dlg1/Gsg1l/Jph3/Kcna1/Nrxn1/Plcb1/S100a1/Scn4b/Shisa9/Slmap	16
BP	GO:0010959	regulation of metal ion transport	20/361	446/28814	3.58	1.09951E-06	0.000287208	0.000223374	Ank3/Atp1a1/Atp2b1/Atp2b2/Cacnb3/Calm1/Camk2a/Camk2d/Cbarp/Dlg1/Homer2/Jph3/Kcna1/Kcnab2/Kcnip3/Plcb1/S100a1/Scn4b/Slc8a1/Slmap	20
BP	GO:0032409	regulation of transporter activity	16/361	298/28814	4.29	1.33126E-06	0.000307553	0.000239198	Ank3/Cacnb3/Calm1/Camk2d/Cbarp/Cnih2/Dlg1/Gsg1l/Jph3/Kcna1/Nrxn1/Plcb1/S100a1/Scn4b/Shisa9/Slmap	16
BP	GO:0007409	axonogenesis	21/361	493/28814	3.4	1.3456E-06	0.000307553	0.000239198	Ank3/Apbb1/Atp8a2/Bdnf/Cck/Crppa/Foxp1/L1cam/Lrrc4c/Map1a/Mycbp2/Nefm/Nexn/Nptx1/Numb/Plxnc1/Rtn4rl1/Sema4a/Sema4f/Smad4/Vasp	21
BP	GO:0072659	protein localization to plasma membrane	16/361	302/28814	4.23	1.5838E-06	0.000340703	0.00026498	Ank3/Cacnb3/Camk2a/Camk2d/Dlg1/Ehd2/Kcnip3/Nrxn1/Numb/Ppp1r9b/Rab26/Rock2/Slc4a1/Slmap/Sorbs1/Tpbg	16
BP	GO:0060079	excitatory postsynaptic potential	9/361	92/28814	7.81	2.39719E-06	0.000463327	0.00036035	Bdnf/Cacnb3/Celf4/Nrxn1/Ppp3ca/Rims2/Sh3gl1/Slc17a7/Stx1a	9
BP	GO:0060078	regulation of postsynaptic membrane potential	10/361	118/28814	6.76	2.47342E-06	0.000463327	0.00036035	Bdnf/Cacnb3/Celf4/Gabra3/Nrxn1/Ppp3ca/Rims2/Sh3gl1/Slc17a7/Stx1a	10
BP	GO:0003012	muscle system process	19/361	430/28814	3.53	2.53392E-06	0.000463327	0.00036035	Apbb1/Atp1a1/Atp2a2/Atp2b1/Atp8a2/Calm1/Camk2d/Cdk9/Dlg1/Foxp1/Kcna1/Myl1/Ppp3ca/Rock2/Scn4b/Slc8a1/Slmap/Smad4/Tmod1	19
BP	GO:0035725	sodium ion transmembrane transport	11/361	150/28814	5.85	3.23593E-06	0.000563514	0.00043827	Ank3/Atp1a1/Atp1a4/Camk2d/Plcb1/Scn4b/Scn8a/Slc12a2/Slc24a2/Slc8a1/Slmap	11
BP	GO:0007269	neurotransmitter secretion	12/361	185/28814	5.18	4.17813E-06	0.000664322	0.000516673	Atp2a2/Cadps/Cadps2/Camk2a/Cplx1/Cplx3/Napa/Nrxn1/Rims2/Stx1a/Stx2/Syn1	12
BP	GO:0099643	signal release from synapse	12/361	185/28814	5.18	4.17813E-06	0.000664322	0.000516673	Atp2a2/Cadps/Cadps2/Camk2a/Cplx1/Cplx3/Napa/Nrxn1/Rims2/Stx1a/Stx2/Syn1	12
BP	GO:0007411	axon guidance	14/361	258/28814	4.33	5.4038E-06	0.000823404	0.000640398	Ank3/Apbb1/Bdnf/Crppa/Foxp1/L1cam/Mycbp2/Nexn/Plxnc1/Rtn4rl1/Sema4a/Sema4f/Smad4/Vasp	14
BP	GO:0097485	neuron projection guidance	14/361	259/28814	4.31	5.64879E-06	0.000826305	0.000642654	Ank3/Apbb1/Bdnf/Crppa/Foxp1/L1cam/Mycbp2/Nexn/Plxnc1/Rtn4rl1/Sema4a/Sema4f/Smad4/Vasp	14
BP	GO:0099565	chemical synaptic transmission, postsynaptic	9/361	103/28814	6.97	6.13789E-06	0.000863318	0.000671441	Bdnf/Cacnb3/Celf4/Nrxn1/Ppp3ca/Rims2/Sh3gl1/Slc17a7/Stx1a	9
BP	GO:0031644	regulation of nervous system process	12/361	194/28814	4.94	6.79866E-06	0.000920841	0.000716179	Cacnb3/Cck/Celf4/Cnih2/Nptx1/Nrxn1/Rims2/Rock2/Sh3gl1/Shisa9/Stx1a/Tppp	12
BP	GO:0016079	synaptic vesicle exocytosis	10/361	137/28814	5.83	9.43456E-06	0.001232221	0.000958353	Atp2a2/Cadps/Cadps2/Camk2a/Cplx1/Cplx3/Napa/Rims2/Stx1a/Stx2	10

MitoPark

ONTOLOGY	ID	Description	GeneRatio	BgRatio	Rich Factor	pvalue	p.adjust	qvalue	geneID	Count
BP	GO:0006520	cellular amino acid metabolic process	13/255	259/28814	5.67	6.11379E-07	0.001292515	0.001120335	Aldh1a1/Bcat2/Cbs/Ddah2/Ddc/Dhfr/Gcat/Gls2/Icmt/Rars2/Sars2/Sephs1/Th	13
BP	GO:0031331	positive regulation of cellular catabolic process	17/255	461/28814	4.17	8.96646E-07	0.001292515	0.001120335	Akt2/Cd81/Cpeb3/Cpt1a/Dda1/Fyco1/Nop53/Pafah1b2/Psen1/Rock1/Scoc/Sh3bp4/Slc25a4/Slc25a5/Ubqln2/Ybx1/Ythdf2	17
BP	GO:0044282	small molecule catabolic process	14/255	334/28814	4.74	1.92429E-06	0.001486954	0.001288873	Acot2/Akt2/Bcat2/Cbs/Cpt1a/Cyp46a1/Ddah2/Dhdh/Gcat/Glb1/Gls2/Inpp5j/Pnkd/Pnp	14
BP	GO:0098656	anion transmembrane transport	11/255	202/28814	6.15	2.06307E-06	0.001486954	0.001288873	Emb/Grk6/Lrrc8b/Lrrc8d/Psen1/Slc25a15/Slc25a21/Slc25a3/Slc25a4/Slc25a42/Slc25a5	11
BP	GO:0099003	vesicle-mediated transport in synapse	12/255	254/28814	5.34	3.11424E-06	0.001610516	0.001395974	Ap2m1/Cadps2/Chrm2/Ddc/Dnm1/Grip1/Psen1/Rock1/Slc18a2/Stx2/Th/Tor1a	12
BP	GO:0002181	cytoplasmic translation	8/255	101/28814	8.95	3.35175E-06	0.001610516	0.001395974	Cpeb3/Drg1/Rpl31/Rpl35a/Rpl8/Rps26/Ybx1/Ythdf2	8
BP	GO:0099504	synaptic vesicle cycle	11/255	227/28814	5.48	6.32806E-06	0.002606257	0.00225907	Ap2m1/Cadps2/Chrm2/Ddc/Dnm1/Psen1/Rock1/Slc18a2/Stx2/Th/Tor1a	11
BP	GO:0098657	import into cell	11/255	240/28814	5.18	1.07022E-05	0.003705476	0.003211859	Abcc9/Akt2/Atp1b1/Kcnh2/Psen1/Slc18a2/Slc6a3/Slc8a2/Slc8a3/Slc9a1/Tor1a	11
BP	GO:0006836	neurotransmitter transport	11/255	242/28814	5.14	1.15676E-05	0.003705476	0.003211859	Cadps2/Chrm2/Ddc/Kcnc3/Pnkd/Psen1/Slc18a2/Slc6a3/Stx2/Th/Tor1a	11
BP	GO:0008652	cellular amino acid biosynthetic process	6/255	62/28814	10.94	1.83909E-05	0.005302088	0.004595782	Aldh1a1/Bcat2/Cbs/Dhfr/Gls2/Sephs1	6
BP	GO:0010765	positive regulation of sodium ion transport	5/255	43/28814	13.14	3.81747E-05	0.009129571	0.007913395	Akt2/Atp1b1/Fgf12/Scn3b/Slc9a1	5
BP	GO:1900242	regulation of synaptic vesicle endocytosis	4/255	22/28814	20.54	3.86575E-05	0.009129571	0.007913395	Ap2m1/Dnm1/Rock1/Tor1a	4
BP	GO:1901605	alpha-amino acid metabolic process	9/255	184/28814	5.53	4.1167E-05	0.009129571	0.007913395	Bcat2/Cbs/Ddah2/Dhfr/Gcat/Gls2/Icmt/Sephs1/Th	9
BP	GO:0035725	sodium ion transmembrane transport	8/255	150/28814	6.03	6.01241E-05	0.012381278	0.010731933	Atp1b1/Fgf12/Scn3b/Slc6a3/Slc8a2/Slc8a3/Slc9a1/Ywhah	8
BP	GO:0098700	neurotransmitter loading into synaptic vesicle	3/255	10/28814	33.9	7.85103E-05	0.014273918	0.012372449	Ddc/Slc18a2/Th	3
BP	GO:0006814	sodium ion transport	10/255	248/28814	4.56	7.9217E-05	0.014273918	0.012372449	Akt2/Atp1b1/Fgf12/Scn3b/Slc38a1/Slc6a3/Slc8a2/Slc8a3/Slc9a1/Ywhah	10
BP	GO:0001505	regulation of neurotransmitter levels	10/255	254/28814	4.45	9.65018E-05	0.016365572	0.014185467	Cadps2/Chrm2/Kcnc3/Pnkd/Psen1/Slc18a2/Slc6a3/Stx2/Th/Tor1a	10
BP	GO:0042572	retinol metabolic process	4/255	29/28814	15.59	0.000119553	0.018616206	0.016136289	Aldh1a1/Rbp1/Rdh14/Ttr	4
BP	GO:0001963	synaptic transmission, dopaminergic	4/255	30/28814	15.07	0.00013699	0.018616206	0.016136289	Pnkd/Slc6a3/Th/Tor1a	4
BP	GO:2000649	regulation of sodium ion transmembrane transporter activity	5/255	56/28814	10.09	0.000137953	0.018616206	0.016136289	Atp1b1/Fgf12/Scn3b/Slc9a1/Ywhah	5
BP	GO:0001666	response to hypoxia	9/255	216/28814	4.71	0.000140662	0.018616206	0.016136289	Acot2/Atp1b1/Cbs/Hmox2/Nop53/Scap/Slc8a3/Slc9a1/Th	9
BP	GO:1900244	positive regulation of synaptic vesicle endocytosis	3/255	12/28814	28.25	0.000142059	0.018616206	0.016136289	Ap2m1/Dnm1/Tor1a	3
BP	GO:1901607	alpha-amino acid biosynthetic process	5/255	57/28814	9.91	0.000150135	0.018763115	0.016263627	Bcat2/Cbs/Dhfr/Gls2/Sephs1	5
BP	GO:1903421	regulation of synaptic vesicle recycling	4/255	31/28814	14.58	0.000156197	0.018763115	0.016263627	Ap2m1/Dnm1/Rock1/Tor1a	4
BP	GO:1901679	nucleotide transmembrane transport	3/255	13/28814	26.08	0.00018347	0.021157755	0.01833927	Slc25a4/Slc25a42/Slc25a5	3
BP	GO:0002028	regulation of sodium ion transport	6/255	97/28814	6.99	0.000228464	0.022933377	0.019878357	Akt2/Atp1b1/Fgf12/Scn3b/Slc9a1/Ywhah	6
BP	GO:0015867	ATP transport	3/255	14/28814	24.21	0.000231982	0.022933377	0.019878357	Slc25a4/Slc25a42/Slc25a5	3
BP	GO:0072530	purine-containing compound transmembrane transport	3/255	14/28814	24.21	0.000231982	0.022933377	0.019878357	Slc25a4/Slc25a42/Slc25a5	3

BP	GO:0099643	signal release from synapse	18/607	185/28814	4.62	8.55388E-08	1.3922E-05	1.00807E-05	Apba1/Apba2/Asic1/Brsk1/Cplx1/Dgki/Dnf1/Grik5/Mef2c/Nrxn3/P2rx7/Pnkd/Prkcb/Rab3a/Skrfo9/Snap25/Snc	18
BP	GO:0007265	Ras protein signal transduction	25/607	342/28814	3.47	8.79607E-08	1.3922E-05	1.00807E-05	Abca1/Adgrg1/Arhgef10/Arhgef2/Cdh2/Dab2ip/Dbnl/Dgki/Dnm2/Dnmt1/Hace1/Nras/Pdpn/Phactr4/Plekhg5/Rasgrf1/Rac2/Ralgps2/Rasgrp2/Rras2/Rsu1/Sbno2/Stg	25
BP	GO:0042391	regulation of membrane potential	30/607	469/28814	3.04	9.03459E-08	1.3922E-05	1.00807E-05	Actn2/Asic1/B2m/Bax/Cell/Chrna1/Dbn1/Dgki/Dnf1/Fgf13/Flna/Gabra3/Gabra4/Gabrb1/Gabrd/Grik5/Hcn3/Kcnh1/Kcnd2/Kcnip2/Mef2c/Nlgn3/Nrxn3/P2rx7/Prkar1b/Scn1b/Scn8/Slc25a	30
BP	GO:0007612	learning	18/607	187/28814	4.57	1.0079E-07	1.49195E-05	1.08029E-05	Adcy3/Asic1/Brsk1/Cntn/Crtc1/Dgki/Dnf1/Fgf13/Nfatc4/Nlgn3/Nrxn3/Pde1b/Pnkd/Ptgs1/Snap25/Tacr1/Th	18
BP	GO:0060999	positive regulation of dendritic spine development	11/607	65/28814	8.03	1.05865E-07	1.49195E-05	1.08029E-05	Camk1/Camk2b/Cnbbp/Dbn1/Dbnl/Dhx36/Eef2k/Itpka/Marcks/Nlgn3/Rac1	11
BP	GO:0050769	positive regulation of neurogenesis	23/607	298/28814	3.66	1.08867E-07	1.49195E-05	1.08029E-05	Ache/Camk2b/Dbn1/Dbnl/Dhx36/Eef2k/Gfap/Gjc2/Hap1/Itpka/Kdm1a/Map2k2/Map3k13/Marcks/Mme/Ntrk3/Picalm/Ptxtc1/Ppp1cc/Sem	23
BP	GO:0060996	dendritic spine development	15/607	130/28814	5.48	1.09311E-07	1.49195E-05	1.08029E-05	Camk1/Camk2b/Cnbbp/Dbn1/Dbnl/Dhx36/Dip2a/Eef2k/Itpka/Marcks/Mef2c/Ngef	15
BP	GO:0010720	positive regulation of cell development	26/607	378/28814	3.27	1.61695E-07	2.05107E-05	1.48514E-05	Ache/Camk2b/Dbn1/Dbnl/Dhx36/Dnm2/Eef2k/Flna/Gfap/Gjc2/Hap1/Itpka/Kdm1a/Map2k2/Map3k13/Marcks/Mme/Ntrk3/Picalm/Plxnc1/Ppp1cc/Rac1/Sema7a/Sox8	26
BP	GO:0048167	regulation of synaptic plasticity	26/607	378/28814	3.27	1.61695E-07	2.05107E-05	1.48514E-05	Adcy8/Brsk1/Camk2b/Dbn1/Dgki/Dnf1/Eepp1/Gfap/Grid1/Itpka/Kcnb1/Mef2c/Mme/Nlgn3/Nrxn/Prkar1b/Prrt1/Ptpn5/Rab3a/Rac1/Shisa7/Shisa9/Snap25/Stx3/Syap1	26
BP	GO:0090407	organophosphate biosynthetic process	30/607	482/28814	2.95	1.63158E-07	2.05107E-05	1.48514E-05	Abca8b/Abhd4/Adcy3/Adcy8/Ak2/Ak5/Atp5pb/Cds1/Chkb/Cktm1/Coasy/Dusp6/Dgki/Dip2a/Gucy1b1/Hdhd5/Hexb/Hmgcs1/Itpka/Nagm/Nme3/Pcyt1b/Pdk2/Pgp/Pip5k1a/Plcg4a/Plcg2/Ptdss1/St	30
BP	GO:1904062	regulation of cation transmembrane transport	26/607	381/28814	3.24	1.88578E-07	2.30984E-05	1.67252E-05	Actn2/Atp2b4/Bax/Dnm2/Drd1/Fgf13/Flna/Gjc2/Gnb5/Hap1/Hspa2/Kcnab3/Kcnc1/Kcnip2/Mef2c/Nlgn3/Pkcg2/Prrt1/Ptpn6/Rgs9/Scn1b/Shisa9/Slc6a9/Stim1/Tacr1/T	26
BP	GO:0016050	vesicle organization	22/607	287/28814	3.64	2.30901E-07	2.75849E-05	1.99738E-05	Abca1/Acsbg/Bloc1s6/Coda/13/Chmp1/Cplx1/Grik5/Lamtor1/P2rx7/Picalm/Pla2g6/Rab14/Rab34/Rab3a/Rab8b/Sar1b/Snap25/Stx19/Stx3/Stxbp6/Vamp3	22
BP	GO:0071868	cellular response to monoamine stimulus	11/607	72/28814	7.25	3.12894E-07	3.55879E-05	2.57686E-05	Atp2b4/Dnm2/Drd1/Flna/Gnb5/Gsk3b/Htr4/Mapk3/Nog2/Rgs8/Rgs9	11
BP	GO:0071870	cellular response to catecholamine stimulus	11/607	72/28814	7.25	3.12894E-07	3.55879E-05	2.57686E-05	Atp2b4/Dnm2/Drd1/Flna/Gnb5/Gsk3b/Htr4/Mapk3/Nog2/Rgs8/Rgs9	11
BP	GO:0071375	cellular response to peptide hormone stimulus	21/607	269/28814	3.71	3.20466E-07	3.56015E-05	2.57785E-05	Actn2/Adcy8/Atp2b1/Bcar3/Eepp1/Foxo1/Grb10/Jak3/Marcks/Ncoa1/Nucks1/Oxsm/Pdk2/Pck2/Prkcb/Rab31/Rab8b/Rac1/Slc25a33/Sorbs1/S	21
BP	GO:1903350	response to dopamine	10/607	59/28814	8.05	3.9706E-07	4.21502E-05	3.05202E-05	Dnm2/Drd1/Flna/Gnal/Gnb5/Htr4/Mapk3/Nog2/Rgs8/Rgs9	10
BP	GO:1903351	cellular response to dopamine	10/607	59/28814	8.05	3.9706E-07	4.21502E-05	3.05202E-05	Dnm2/Drd1/Flna/Gnal/Gnb5/Htr4/Mapk3/Nog2/Rgs8/Rgs9	10
BP	GO:0071867	response to monoamine	11/607	74/28814	7.06	4.16586E-07	4.2341E-05	3.06585E-05	Atp2b4/Dnm2/Drd1/Flna/Gnal/Gnb5/Htr4/Mapk3/Nog2/Rgs8/Rgs9	11
BP	GO:0071869	response to catecholamine	11/607	74/28814	7.06	4.16586E-07	4.2341E-05	3.06585E-05	Atp2b4/Dnm2/Drd1/Flna/Gnal/Gnb5/Htr4/Mapk3/Nog2/Rgs8/Rgs9	11
BP	GO:0099175	regulation of postsynapse organization	13/607	111/28814	5.56	6.44544E-07	6.41455E-05	4.64467E-05	Camk2b/Cdh2/Dbn1/Dbnl/Dhx36/Eef2k/Itpka/Marcks/Ngef/Nlgn3/Nptn1/Nrp2/Ntrk3	13
BP	GO:0051962	positive regulation of nervous system development	24/607	359/28814	3.17	8.02591E-07	7.82448E-05	5.66555E-05	Ache/Camk2b/Dbn1/Dbnl/Dhx36/Eef2k/Gfap/Gjc2/Hap1/Itpka/Kdm1a/Map2k2/Map3k13/Marcks/Mme/Nlgn3/Ntrk3/Picalm/Plxnc1/Ppp1cc/Sema7a/Sox8/Tiam2/Vi	24
BP	GO:0046034	ATP metabolic process	20/607	263/28814	3.61	9.20659E-07	8.58398E-05	6.21552E-05	Ak2/Ak5/Atp5pb/Cox7a1/Cox7a2/Cox7c/Eif6/Eno2/Eno3/Hexb/Ndufa10/Ndufa12/Ndufs7/Ndufb6/Prkaa1/Slc25a33/Stat3/Tpi1/Zfr	20
BP	GO:0050807	regulation of synapse organization	20/607	263/28814	3.61	9.20659E-07	8.58398E-05	6.21552E-05	Camk1/Camk2b/Cdh2/Dab2ip/Dbn1/Dbnl/Dhx36/Eef2k/Farp1/Itpka/Marcks/Mef2c/Ngef/Nlgn3/Nptn1/Nrp2/Ntrk3/Picalm/Sparc/Tuba1a	20
BP	GO:0098700	neurotransmitter loading into synaptic vesicle	5/607	10/28814	23.73	9.42313E-07	8.58398E-05	6.21552E-05	Ddc/Slc10a4/Slc18a2/Slc18a3/Th	5
BP	GO:0015980	energy derivation by oxidation of organic compounds	22/607	312/28814	3.35	9.52378E-07	8.58398E-05	6.21552E-05	Atp5pb/Bax/Cox7a1/Cox7a2/Cox7c/Eepp1/Esrrb/Gsk3b/Hmgb1/Idh3a/Mdh2/Ndufa10/Ndufa12/Ndufs7/Ndufb6/Prkaa1/Pkb/Ppp1cc/Sdha/Sdhd/Slc25a18/Slc25a33/Sorb	22
BP	GO:0001764	neuron migration	17/607	196/28814	4.12	9.95144E-07	8.80334E-05	6.37435E-05	Adgrg1/Arhgef2/Bax/Camk2b/Cdk5r1/Dab2ip/Dnf1/Fgf13/Flna/Mef2c/Nrp2/Ntrk3/Pcnt1/Phactr1/Rac1/Stat3/Vax	17
BP	GO:0010959	regulation of metal ion transport	27/607	446/28814	2.87	1.15279E-06	0.000100125	7.24989E-05	Actn2/Atp2b1/Atp2b4/B2m/Bax/Dnm2/Drd1/Eepp1/Fgf13/Flna/Gjc2/Gnb5/Hap1/Homer2/Hspa2/Jak3/Kcnab3/Kcnc1/Kcnip2/P2rx7/Pkcg2/Ptpn6/Rgs9/Scn1b/Slc6a9	27
BP	GO:0032868	response to insulin	19/607	246/28814	3.67	1.3619E-06	0.000116175	8.41202E-05	Ache/Atp2b1/Bcar3/Eif6/Enpp1/Foxo1/Grb10/Marcks/Mmk7/Ncoa2/Nucks1/Oxsm/Pck2/Pdk2/Prkcb/Rab31/Rab8b/Slc25a33/Sorbs1	19
BP	GO:0017157	regulation of exocytosis	19/607	247/28814	3.65	1.44696E-06	0.000120816	8.74807E-05	Apba1/Apba2/Cdk5r2/Dgki/Dnf1/Hap1/Kcnb1/Lgi3/Nrxn3/P2rx7/Pnkd/Prkcb/Rab26/Rab3a/Rab3c/Rab8b/Rac2/Snap25/Stam/Syt17	19
BP	GO:0050803	regulation of synapse structure or activity	20/607	271/28814	3.5	1.46689E-06	0.000120816	8.74807E-05	Camk1/Camk2b/Cdh2/Dab2ip/Dbn1/Dbnl/Dhx36/Eef2k/Farp1/Itpka/Marcks/Mef2c/Ngef/Nlgn3/Nptn1/Nrp2/Ntrk3/Picalm/Sparc/Tuba1a	20
BP	GO:0043254	regulation of protein-containing complex assembly	26/607	429/28814	2.88	1.77845E-06	0.000143994	0.000104264	Abca1/Arhgef2/Arpc1b/Atp2b4/Bax/Chchd3/Arpc5/Dbn1/Dnm1/Dnnl/Hmgb1/Itsn1/Ipp5/Ipp5/Kank4/Lamp2/Lcp1/Mme/Pkcg2/Rab3a/Rac1/Sar1b/Snh3/Stxbp6/Tmod	26
BP	GO:0061003	positive regulation of dendritic spine morphogenesis	7/607	29/28814	11.46	1.85543E-06	0.000144749	0.00010481	Camk2b/Dbn1/Dbnl/Dhx36/Eef2k/Itpka/Marcks	7
BP	GO:0010977	negative regulation of neuron projection development	15/607	162/28814	4.4	1.89734E-06	0.000144749	0.00010481	B2m/Dnp2b/Fgf13/Flna/Gfap/H2-K1/Inpp5j/Lgals1/Ngef/Nlgn3/Prag1/Rtn4rl2/Sema7a/Scrt/Vax	15
BP	GO:0010769	regulation of cell morphogenesis involved in differentiation	13/607	122/28814	5.06	1.90897E-06	0.000144749	0.00010481	Ache/Camk2b/Dbn1/Dbnl/Dhx36/Dnm2/Eef2k/Flna/Itpka/Marcks/Pdpn/Rac1/Rcc2	13
BP	GO:0046928	regulation of neurotransmitter secretion	13/607	122/28814	5.06	1.90897E-06	0.000144749	0.00010481	Apba1/Apba2/Asic1/Cplx1/Dgki/Dnf1/Mef2c/Nrxn3/P2rx7/Pnkd/Prkcb/Rab3a/Snc	13
BP	GO:0071900	regulation of protein serine/threonine kinase activity	25/607	352/28814	3.1	1.99988E-06	0.000149273	0.000108086	Ache/Atp2b4/Camk1/Cdk5r1/Dab2ip/Dgkq/Dusp6/Gadd45g/Gnb5/Ipgm1/Map2k2/Map3k13/Marcks/Ntrk3/P2rx7/Prkag1/Prkar1b/Prkar2b/Ptpn5/Ptpn6/Syap1/Tgfa/Trafd/Va	23
BP	GO:0050770	regulation of axonogenesis	16/607	185/28814	4.11	2.14134E-06	0.000157372	0.000113951	Ache/Brsk1/Cdh2/Chn1/Dbn1/Dbnl/Dnp2b/Fgf13/Map2k2/Map3k13/Ntrk3/Picalm/Plxnc1/Sema7a/Tiam2/Vax	16
BP	GO:0140694	non-membrane-bounded organelle assembly	24/607	381/28814	2.99	2.27829E-06	0.0001649	0.000119401	Abracl/Actn2/Arhgef10/Arhgef2/Brsk1/Cdc14/Chmp5/Cnot7/Dbnl/Edf1/Flna/Flnc/Lcp1/Mef2c/Mme/Nr4a1/Ntrk3/Ogfod1/Pls1/Rpl24/Rplp0	24
BP	GO:0061001	regulation of dendritic spine morphogenesis	9/607	56/28814	7.63	2.41489E-06	0.000172178	0.000124672	Camk2b/Dbn1/Dbnl/Dhx36/Eef2k/Itpka/Marcks/Ngef/Nlgn3	9

BP	GO:0099004	calmodulin dependent kinase signaling pathway	4/361	12/28814	26.61	1.1079E-05	0.001328059	0.00103289	Camk2a/Pcp4/Prkaa1/Zmpste24	4
BP	GO:0140115	export across plasma membrane	7/361	61/28814	9.16	1.11544E-05	0.001328059	0.00103289	Abca1/Atp1a1/Atp1a4/Atp2b1/Dlg1/Kcna2/Slc8a1	7
BP	GO:0031503	protein-containing complex localization	12/361	204/28814	4.7	1.13102E-05	0.001328059	0.00103289	Adam10/Camk2a/Cnih2/Cplx1/Dlg1/Dync2i2/Gsg1l/Nptx1/Nrxn1/Numb/Ppp3r1/Stau1	12
BP	GO:0006936	muscle contraction	15/361	314/28814	3.81	1.1621E-05	0.001328059	0.00103289	Apbb1/Atp1a1/Atp2a2/Atp2b1/Atp8a2/Calm1/Camk2d/Dlg1/Kcna1/Myl1/Rock2/Scn4b/Slc8a1/Slmap/Tmod1	15
BP	GO:0006836	neurotransmitter transport	13/361	242/28814	4.29	1.29996E-05	0.001440589	0.00112041	Atp2a2/Cadps/Cadps2/Camk2a/Cplx1/Cplx3/Napa/Nrxn1/Rims2/Slc17a7/Stx1a/Stx2/Syn1	13
BP	GO:0098876	vesicle-mediated transport to the plasma membrane	10/361	144/28814	5.54	1.46171E-05	0.001572198	0.001222768	Ank3/Arhgap1/Atp9a/Cplx1/Dennd1a/Ehd2/Epg5/Rab11b/Rab26/Vps26c	10
BP	GO:0016082	synaptic vesicle priming	5/361	26/28814	15.35	1.59051E-05	0.001661855	0.001292498	Cadps/Cadps2/Napa/Rims2/Stx1a	5
BP	GO:0006814	sodium ion transport	13/361	248/28814	4.18	1.68484E-05	0.001711522	0.001331126	Ank3/Atp1a1/Atp1a4/Camk2d/Cpox/Plcb1/Scn4b/Scn8a/Slc12a2/Slc17a7/Slc24a2/Slc8a1/Slmap	13
BP	GO:0045055	regulated exocytosis	14/361	287/28814	3.89	1.79893E-05	0.001778023	0.001382847	Atp2a2/Cadps/Cadps2/Camk2a/Cbarp/Cplx1/Cplx3/Napa/Rab11b/Rab26/Rabgef1/Rims2/Stx1a/Stx2	14
BP	GO:0098900	regulation of action potential	7/361	66/28814	8.47	1.88728E-05	0.001816255	0.001412581	Ank3/Atp2a2/Cacnb3/Camk2d/Dlg1/Foxp1/Kcnab2	7
BP	GO:0001505	regulation of neurotransmitter levels	13/361	254/28814	4.09	2.16634E-05	0.002018786	0.001570099	Atp2a2/Cadps/Cadps2/Camk2a/Cplx1/Cplx3/Napa/Nrxn1/Rims2/Slc17a7/Stx1a/Stx2/Syn1	13
BP	GO:0099072	regulation of postsynaptic membrane neurotransmitter receptor levels	8/361	93/28814	6.87	2.26615E-05	0.002018786	0.001570099	Adam10/Camk2a/Cplx1/Dlg1/Gsg1l/Nptx1/Numb/Ppp3r1	8
BP	GO:0015698	inorganic anion transport	10/361	152/28814	5.25	2.3379E-05	0.002018786	0.001570099	Clcn2/Gabra3/Gabrb1/Slc12a2/Slc17a7/Slc25a10/Slc25a14/Slc26a6/Slc4a1/Xpr1	10
BP	GO:0090257	regulation of muscle system process	13/361	256/28814	4.05	2.35165E-05	0.002018786	0.001570099	Atp1a1/Atp2a2/Atp2b1/Calm1/Camk2d/Cdk9/Dlg1/Foxp1/Kcna1/Ppp3ca/Rock2/Slc8a1/Smad4	13
BP	GO:0090150	establishment of protein localization to membrane	13/361	257/28814	4.04	2.44941E-05	0.002018786	0.001570099	Ank3/Cacnb3/Cplx1/Emc7/Oxa1l/Pex19/Rab11b/Rab26/Rab3ip/Rabgef1/Sec61a1/Sec61a2/Sgtb	13
BP	GO:0006839	mitochondrial transport	10/361	153/28814	5.22	2.47397E-05	0.002018786	0.001570099	Camk2a/Hebp2/Nptx1/Oxa1l/Prkaa1/Slc25a10/Slc25a14/Stat3/Timm23/Timm8a1	10
BP	GO:0019693	ribose phosphate metabolic process	16/361	376/28814	3.4	2.48415E-05	0.002018786	0.001570099	Ampd3/Atp2b2/Coasy/Gcdh/Mvd/Mvk/Nudt3/Nudt4/Pdk1/Pdk3/Ppat/Prkaa1/Prps1/Slc4a1/Stat3/Uprt	16
BP	GO:0070839	metal ion export	6/361	47/28814	10.19	2.58281E-05	0.002053335	0.001596969	Atp1a1/Atp1a4/Atp2b1/Dlg1/Kcna2/Slc8a1	6
BP	GO:0022604	regulation of cell morphogenesis	15/361	338/28814	3.54	2.74448E-05	0.002077187	0.00161552	Cfdp1/Coro1c/Dlg1/Epb41l2/Itpka/Lims1/Myl12b/Numb/Plxnc1/Prpf40a/Pxn/Rims2/Sema4a/Ttc3/Zmpste24	15
BP	GO:0048488	synaptic vesicle endocytosis	7/361	70/28814	7.98	2.78322E-05	0.002077187	0.00161552	Dennd1a/Ppp3cc/Sh3gl1/Sh3gl2/Slc17a7/Sncb/Stx1a	7
BP	GO:0140238	presynaptic endocytosis	7/361	70/28814	7.98	2.78322E-05	0.002077187	0.00161552	Dennd1a/Ppp3cc/Sh3gl1/Sh3gl2/Slc17a7/Sncb/Stx1a	7
BP	GO:0030007	cellular potassium ion homeostasis	4/361	15/28814	21.28	2.96548E-05	0.002168954	0.001686892	Atp1a1/Atp1a4/Camk2d/Slc12a2	4
BP	GO:0072594	establishment of protein localization to organelle	17/361	426/28814	3.19	3.14152E-05	0.002252651	0.001751986	Ipo8/Nup188/Oxa1l/Pex1/Pex13/Pex19/Ppp3ca/Ppp3r1/Prkaa1/Ranbp6/Sec13/Sec61a1/Sec61a2/Sgtb/Stat3/Timm23/Timm8a1	17
BP	GO:0007214	gamma-aminobutyric acid signaling pathway	5/361	30/28814	13.3	3.30678E-05	0.002325558	0.001808689	Bdnf/Gabra3/Gabrb1/Phf24/Slc12a2	5
BP	GO:0016197	endosomal transport	12/361	228/28814	4.2	3.40685E-05	0.002350728	0.001828265	Abca1/Ap5z1/Arhgap1/Atp9a/Coro1c/Dennd1a/Ehd2/Epg5/Lamtor1/Rab11b/Snx6/Vps26c	12
BP	GO:0006066	alcohol metabolic process	15/361	345/28814	3.47	3.47252E-05	0.002351667	0.001828995	Abca1/Cat/Clcn2/Dhcr7/Inpp5b/Itpka/Mvd/Mvk/Nudt3/Nudt4/Pck2/Pecr/Plcb1/Ppip5k2/Prkaa1	15
BP	GO:0001764	neuron migration	11/361	196/28814	4.48	4.02846E-05	0.002678557	0.002083232	Adgrg1/Apbb1/Camk2a/Cck/Elp3/Kirrel3/Mdga2/Mrtfa/Pex13/Septin4/Stat3	11
BP	GO:0097120	receptor localization to synapse	7/361	75/28814	7.45	4.3681E-05	0.002816563	0.002190566	Adam10/Camk2a/Cnih2/Cplx1/Dlg1/Nptx1/Nrxn1	7
BP	GO:0090407	organophosphate biosynthetic process	18/361	482/28814	2.98	4.39005E-05	0.002816563	0.002190566	Ampd3/Ckb/Coasy/Etnk1/Gcdh/Itpka/Mboat7/Mvd/Mvk/Pcyt1b/Pdk1/Pdk3/Pip4k2c/Ppat/Ppip5k2/Prps1/Stat3/Uprt	18
BP	GO:0070296	sarcoplasmic reticulum calcium ion transport	5/361	32/28814	12.47	4.57776E-05	0.002886356	0.002244846	Atp2a2/Calm1/Camk2d/Slc8a1/Zmpste24	5
BP	GO:0034599	cellular response to oxidative stress	13/361	274/28814	3.79	4.74937E-05	0.002901248	0.002256429	Atp2a2/Cat/Ccs/Chchd2/Coq7/Foxp1/Oxr1/Pdk1/Prkaa1/Prkra/Sirpa/Slc8a1/Stx2	13
BP	GO:0032456	endocytic recycling	7/361	76/28814	7.35	4.76005E-05	0.002901248	0.002256429	Arhgap1/Atp9a/Dennd1a/Ehd2/Epg5/Rab11b/Vps26c	7
BP	GO:0009117	nucleotide metabolic process	17/361	447/28814	3.04	5.70436E-05	0.003419811	0.002659738	Ampd3/Atp2b2/Coasy/Gcdh/Mvd/Mvk/Nt5m/Nudt3/Nudt4/Pdk1/Pdk3/Ppat/Prkaa1/Prps1/Slc4a1/Stat3/Uprt	17
BP	GO:0006605	protein targeting	13/361	280/28814	3.71	5.92334E-05	0.003493817	0.002717296	Ank3/Cacnb3/Pex1/Pex13/Pex19/Prkaa1/Rab3ip/Rabgef1/Sec61a1/Sec61a2/Sgtb/Timm23/Timm8a1	13
BP	GO:0006813	potassium ion transport	12/361	243/28814	3.94	6.30888E-05	0.003575581	0.002780888	Ank3/Atp1a1/Atp1a4/Calm1/Cpox/Dlg1/Kcna1/Kcna2/Kcnab2/Kcnip3/Slc12a2/Slc24a2	12
BP	GO:0071805	potassium ion transmembrane transport	11/361	206/28814	4.26	6.32272E-05	0.003575581	0.002780888	Ank3/Atp1a1/Atp1a4/Calm1/Dlg1/Kcna1/Kcna2/Kcnab2/Kcnip3/Slc12a2/Slc24a2	11
BP	GO:0009259	ribonucleotide metabolic process	15/361	364/28814	3.29	6.37201E-05	0.003575581	0.002780888	Ampd3/Atp2b2/Coasy/Gcdh/Mvd/Mvk/Nudt3/Nudt4/Pdk1/Pdk3/Ppat/Prkaa1/Slc4a1/Stat3/Uprt	15
BP	GO:0010644	cell communication by electrical coupling	4/361	18/28814	17.74	6.45306E-05	0.003575581	0.002780888	Ank3/Camk2d/Kcna1/Slc8a1	4
BP	GO:0006753	nucleoside phosphate metabolic process	17/361	455/28814	2.98	7.08563E-05	0.00385971	0.003001867	Ampd3/Atp2b2/Coasy/Gcdh/Mvd/Mvk/Nt5m/Nudt3/Nudt4/Pdk1/Pdk3/Ppat/Prkaa1/Prps1/Slc4a1/Stat3/Uprt	17

BP	GO:1905039	carboxylic acid transmembrane transport	7/255	138/28814	5.73	0.000237095	0.022933377	0.019878357	Akt2/Emb/Lrrc8d/Psen1/Slc25a15/Slc25a21/Slc38a1	7
BP	GO:0022898	regulation of transmembrane transporter activity	10/255	284/28814	3.98	0.000238641	0.022933377	0.019878357	Abcc9/Atp1b1/Cacng8/Fgf12/Grk6/Kcnc1/Scn3b/Slc25a4/Slc9a1/Ywhah	10
BP	GO:1903825	organic acid transmembrane transport	7/255	139/28814	5.69	0.000247812	0.023046537	0.019976442	Akt2/Emb/Lrrc8d/Psen1/Slc25a15/Slc25a21/Slc38a1	7
BP	GO:0006066	alcohol metabolic process	11/255	345/28814	3.6	0.000276719	0.024251351	0.02102076	Aldh1a1/Cyp46a1/Dhfr/Ephx1/Inpp5j/Lima1/Nudt10/Rbp1/Rdh14/Scap/Ttr	11
BP	GO:0098739	import across plasma membrane	8/255	191/28814	4.73	0.000317673	0.024251351	0.02102076	Abcc9/Akt2/Atp1b1/Kcnh2/Psen1/Slc8a2/Slc8a3/Slc9a1	8
BP	GO:1902305	regulation of sodium ion transmembrane transport	5/255	67/28814	8.43	0.000322296	0.024251351	0.02102076	Atp1b1/Fgf12/Scn3b/Slc9a1/Ywhah	5
BP	GO:0046394	carboxylic acid biosynthetic process	10/255	295/28814	3.83	0.000322699	0.024251351	0.02102076	Aldh1a1/Amacr/Bcat2/Cbs/Dhfr/Gls2/Hpgds/Rbp1/Scap/Sephs1	10
BP	GO:0016053	organic acid biosynthetic process	10/255	296/28814	3.82	0.000331433	0.024251351	0.02102076	Aldh1a1/Amacr/Bcat2/Cbs/Dhfr/Gls2/Hpgds/Rbp1/Scap/Sephs1	10
BP	GO:0036293	response to decreased oxygen levels	9/255	243/28814	4.19	0.0003369	0.024251351	0.02102076	Acot2/Atp1b1/Cbs/Hmox2/Nop53/Scap/Slc8a3/Slc9a1/Th	9
BP	GO:0032409	regulation of transporter activity	10/255	298/28814	3.79	0.000349496	0.024251351	0.02102076	Abcc9/Atp1b1/Cacng8/Fgf12/Grk6/Kcnc1/Scn3b/Slc25a4/Slc9a1/Ywhah	10
BP	GO:0009070	serine family amino acid biosynthetic process	3/255	16/28814	21.19	0.00035225	0.024251351	0.02102076	Cbs/Dhfr/Sephs1	3
BP	GO:0042416	dopamine biosynthetic process	3/255	16/28814	21.19	0.00035225	0.024251351	0.02102076	Ddc/Slc6a3/Th	3
BP	GO:1903423	positive regulation of synaptic vesicle recycling	3/255	16/28814	21.19	0.00035225	0.024251351	0.02102076	Ap2m1/Dnm1/Tor1a	3
BP	GO:0016236	macroautophagy	9/255	245/28814	4.15	0.00035766	0.024251351	0.02102076	Fyco1/Pafah1b2/Psen1/Rimoc1/Scoc/Slc25a4/Slc25a5/Ubqln2/Yod1	9
BP	GO:1901361	organic cyclic compound catabolic process	13/255	477/28814	3.08	0.000361709	0.024251351	0.02102076	Cpeb3/Cyp46a1/Entpd1/Entpd3/Hmox2/Nudt10/Pnp/Pym1/Rock1/Slc6a3/Ybx1/Ythdf2/Ywhah	13
BP	GO:0072659	protein localization to plasma membrane	10/255	302/28814	3.74	0.00038809	0.024483486	0.021221971	Akt2/Ap2m1/Atp1b1/Cd81/Grip1/Lypla1/Phaf1/Pip5k1a/Rock1/Scn3b	10
BP	GO:0036503	ERAD pathway	6/255	107/28814	6.34	0.000388268	0.024483486	0.021221971	Dnajc10/Tor1a/Ubqln2/Ubxn4/Wfs1/Yod1	6
BP	GO:0008016	regulation of heart contraction	8/255	197/28814	4.59	0.000390649	0.024483486	0.021221971	Abcc9/Atp1b1/Chrm2/Glrx3/Kcnh2/Scn3b/Slc9a1/Th	8
BP	GO:0010508	positive regulation of autophagy	7/255	151/28814	5.24	0.000409623	0.025126439	0.021779274	Fyco1/Pafah1b2/Rock1/Scoc/Sh3bp4/Slc25a4/Slc25a5	7
BP	GO:0007156	homophilic cell adhesion via plasma membrane adhesion molecules	6/255	112/28814	6.05	0.000495487	0.029760192	0.025795752	Cadm3/Cdh12/Celsr2/Emb/Nectin1/Nptn	6
BP	GO:0006820	anion transport	13/255	498/28814	2.95	0.000541879	0.031244717	0.02708252	Emb/Grk6/Lrrc8b/Lrrc8d/Psen1/Slc25a15/Slc25a21/Slc25a3/Slc25a4/Slc25a42/Slc25a5/Slc38a1/Ttyh2	13
BP	GO:0034765	regulation of ion transmembrane transport	13/255	498/28814	2.95	0.000541879	0.031244717	0.02708252	Abcc9/Atp1b1/Cacng8/Fgf12/Grk6/Kcnc1/Kcnc3/Kcnh2/Psen1/Scn3b/Slc25a4/Slc9a1/Ywhah	13
BP	GO:0016239	positive regulation of macroautophagy	5/255	76/28814	7.43	0.000578033	0.031282516	0.027115283	Fyco1/Pafah1b2/Scoc/Slc25a4/Slc25a5	5
BP	GO:0032543	mitochondrial translation	5/255	76/28814	7.43	0.000578033	0.031282516	0.027115283	Gfm2/Mrpl51/Mrps18b/Rars2/Sars2	5
BP	GO:0016241	regulation of macroautophagy	6/255	116/28814	5.84	0.000596788	0.031282516	0.027115283	Fyco1/Pafah1b2/Scoc/Slc25a4/Slc25a5/Ubqln2	6
BP	GO:0098659	inorganic cation import across plasma membrane	6/255	116/28814	5.84	0.000596788	0.031282516	0.027115283	Abcc9/Atp1b1/Kcnh2/Slc8a2/Slc8a3/Slc9a1	6
BP	GO:0099587	inorganic ion import across plasma membrane	6/255	116/28814	5.84	0.000596788	0.031282516	0.027115283	Abcc9/Atp1b1/Kcnh2/Slc8a2/Slc8a3/Slc9a1	6
BP	GO:1901606	alpha-amino acid catabolic process	5/255	78/28814	7.24	0.000651125	0.033521335	0.029055863	Bcat2/Cbs/Ddah2/Gcat/Gls2	5
BP	GO:0042417	dopamine metabolic process	4/255	46/28814	9.83	0.000730102	0.034328008	0.029755076	Ddc/Pnkd/Slc6a3/Th	4
BP	GO:1990542	mitochondrial transmembrane transport	5/255	80/28814	7.06	0.000730926	0.034328008	0.029755076	Slc25a15/Slc25a21/Slc25a4/Slc25a5/Slc8a3	5
BP	GO:0015837	amine transport	6/255	121/28814	5.6	0.000745219	0.034328008	0.029755076	Ddc/Entpd1/Psen1/Slc18a2/Th/Tor1a	6
BP	GO:0019439	aromatic compound catabolic process	12/255	451/28814	3.01	0.000746053	0.034328008	0.029755076	Cpeb3/Entpd1/Entpd3/Ephx1/Hmox2/Nudt10/Pnp/Pym1/Rock1/Slc6a3/Ybx1/Ythdf2	12
BP	GO:0043270	positive regulation of ion transport	10/255	329/28814	3.43	0.000752548	0.034328008	0.029755076	Akt2/Atp1b1/Cacng8/Fgf12/Kcnc1/Kcnh2/Psen1/Scn3b/Slc9a1/Wfs1	10
BP	GO:0009636	response to toxic substance	6/255	122/28814	5.56	0.000778051	0.034328008	0.029755076	Aldh1a1/Ddc/Dhfr/Ephx1/Inmt/Slc18a2	6
BP	GO:0046395	carboxylic acid catabolic process	8/255	219/28814	4.13	0.00078347	0.034328008	0.029755076	Acot2/Akt2/Bcat2/Cbs/Cpt1a/Ddah2/Gcat/Gls2	8
BP	GO:0070839	metal ion export	4/255	47/28814	9.62	0.00079252	0.034328008	0.029755076	Atp1b1/Kcnh2/Slc8a2/Slc8a3	4
BP	GO:0032412	regulation of ion transmembrane transporter activity	9/255	274/28814	3.71	0.000797897	0.034328008	0.029755076	Abcc9/Atp1b1/Cacng8/Fgf12/Grk6/Kcnc1/Scn3b/Slc9a1/Ywhah	9
BP	GO:0050767	regulation of neurogenesis	12/255	455/28814	2.98	0.000805276	0.034328008	0.029755076	Cux1/Epha7/Fbxo31/Gpr37l1/Mme/Nap1l1/Nptn/Psen1/Rnf112/Tgm2/Ythdf2/Ywhah	12
BP	GO:0015868	purine ribonucleotide transport	3/255	21/28814	16.14	0.000809679	0.034328008	0.029755076	Slc25a4/Slc25a42/Slc25a5	3

BP	GO:0048813	dendrite morphogenesis	16/607	187/28814	4.06	2.4649E-06	0.000173159	0.000125382	Ache/Camk2b/Dbn1/Dbnl/Dhx36/Dip2a/Eef2k/Farp1/Itpka/Marcks/Ngef/Nlgn3/Phactr1/Picalm/Rac1/Tmem10	16
BP	GO:0048284	organelle fusion	14/607	145/28814	4.58	2.53495E-06	0.0001755	0.000127076	Bax/Bloc1s6/Chmp5/Cplx1/Grik5/Miga2/Pla2g4a/Rab34/Rab3a/Rab8b/Snap25/Stx3/Stxbp6/Vamp3	14
BP	GO:1900271	regulation of long-term synaptic potentiation	9/607	57/28814	7.5	2.81455E-06	0.000192073	0.000139077	Adcy8/Drd1/Mme/Nlgn3/Nrgn/Prkar1b/Ptpn5/Rab3a/Shisa7	9
BP	GO:0032970	regulation of actin filament-based process	25/607	414/28814	2.87	2.98836E-06	0.000194627	0.000140927	Actn2/Arhgap6/Arhgef10/Arpc1b/Arpc3/Arpc5l/Cracd/Ctnna3/Dbn1/Dbnl/Fgf13/Flna/Kank4/Mef2c/Ntrk3/Pdlim4/Pdpn/Rac1/Rac2/Rhob/Rhof/Ssh3/Tmod1/Vangl2/	25
BP	GO:1900273	positive regulation of long-term synaptic potentiation	7/607	31/28814	10.72	3.01379E-06	0.000194627	0.000140927	Adcy8/Drd1/Mme/Nrgn/Prkar1b/Ptpn5/Shisa7	7
BP	GO:0048678	response to axon injury	10/607	73/28814	6.5	3.01495E-06	0.000194627	0.000140927	Bax/Enpp1/Flna/Gap43/Map2k2/Morn4/Ntrk3/Plcg2/Rtn4rl2/Sod1	10
BP	GO:0060997	dendritic spine morphogenesis	10/607	73/28814	6.5	3.01495E-06	0.000194627	0.000140927	Camk2b/Dbn1/Dbnl/Dhx36/Dip2a/Eef2k/Itpka/Marcks/Ngef/Nlgn3	10
BP	GO:0006906	vesicle fusion	12/607	109/28814	5.23	3.35073E-06	0.000213419	0.000154533	Bloc1s6/Chmp5/Cplx1/Grik5/Pla2g4a/Rab34/Rab3a/Rab8b/Snap25/Stx3/Stxbp6/V	12
BP	GO:1904064	positive regulation of cation transmembrane transport	15/607	170/28814	4.19	3.46117E-06	0.000216894	0.000157049	Actn2/Bax/Dnm2/Drd1/Flna/Gjc2/Hap1/Hspa2/Kcnc1/Kcnip2/Nlgn3/Plcg2/Rgs9/S	15
BP	GO:0045055	regulated exocytosis	20/607	287/28814	3.31	3.52451E-06	0.000216894	0.000157049	Apba1/Apba2/Cdk5r2/Cplx1/Dgki/Drd1/Grik5/Kcnb1/Nrxn3/P2rx7/Prkcb/Rab26/Rab31/Rab3a/Rac2/Snap25/Stx3/Syngr1/Syt17/Vamp3	20
BP	GO:0031345	negative regulation of cell projection organization	17/607	215/28814	3.75	3.54149E-06	0.000216894	0.000157049	B2m/Dip2b/Dnm2/Fgf13/Flna/Gfap/H2-K1/Inpp5j/Lgals1/Ngef/Nlgn3/Prag1/Rap1gap/Rtn4rl2/	17
BP	GO:0034767	positive regulation of ion transmembrane transport	16/607	193/28814	3.94	3.7157E-06	0.000224323	0.000162429	Actn2/Bax/Dnm2/Drd1/Flna/Gjc2/Hap1/Hspa2/Kcnc1/Kcnip2/Nlgn3/P2rx7/Plcg2/	16
BP	GO:0009117	nucleotide metabolic process	26/607	447/28814	2.76	3.75672E-06	0.000224323	0.000162429	Acot1/Acot11/Adcy3/Adcy8/Ak2/Ak5/Atp5pb/Coasy/Dcakd/Dip2a/Dnph1/Eif6/Eno2/Enpp1/Gucy1b1/Hk2/Hmgcs1/Naprt/Nme3/Nudt12/Nudt7/P2rx7/Pdk2/Stat3/Tpi1	26
BP	GO:0032535	regulation of cellular component size	24/607	393/28814	2.9	3.88072E-06	0.000228867	0.000165719	Actn2/Akt1s1/Arpc1b/Arpc3/Arpc5l/Ccdc51/Cracd/Dbn1/Dbnl/Dip2b/Dnm2/Fgf13/Inf2/Kank4/Map3k13/Ntrk3/P2rx7/Picalm/Pls1/Rac1/Sema7a/Ssh3/Tmod1/Vasp	24
BP	GO:0006644	phospholipid metabolic process	23/607	367/28814	2.97	4.01653E-06	0.000233987	0.000169426	Abca8b/Abhd4/Cds1/Dgki/Gpcpd1/Hdhd5/Hexb/Hmgcs1/Inpp5j/Itpka/Mtmr12/Pcyt1b/Pigt/Pip4p1/Pip5k1a/Pitpnc1/Pla2g4a/Pla2g7/Plcg2/Pld1/Sgpp1/Smpd3/Smpd	23
BP	GO:0006163	purine nucleotide metabolic process	23/607	369/28814	2.96	4.394E-06	0.000252893	0.000183116	Acot1/Acot11/Adcy3/Adcy8/Ak2/Atp5pb/Coasy/Dcakd/Dip2a/Dnph1/Eif6/Eno2/Enpp1/Gucy1b1/Hk2/Hmgcs1/Nme3/Nudt7/P2rx7/Pdk2/St	23
BP	GO:0034248	regulation of cellular amide metabolic process	26/607	453/28814	2.72	4.77182E-06	0.000266946	0.000193291	Clu/Cnot7/Coa3/Dcp1a/Dcps/Dhx36/Dph5/Eif6/Hap1/Igfbp5/Mapk3/Mrpl13/Ncbp2/Noct/Ogfod1/Paip1/Pdk2/Picalm/Pld1/Pum3/Rps9/Smpd3/Taco1/Tia1/Tut4/Vim	26
BP	GO:0044262	cellular carbohydrate metabolic process	20/607	293/28814	3.24	4.81108E-06	0.000266946	0.000193291	Abhd10/Arpp19/Coq3/Enpp1/Foxo1/Grb10/Hk2/Hmgb1/Igfbp4/Inpp5j/Itpka/Pdk2/Phka1/Phkb/Pla2g4a/Ppp1cc/Sorbs1/Stat3/Tpi1/Ugt1a1	20
BP	GO:0090174	organelle membrane fusion	12/607	113/28814	5.04	4.89612E-06	0.000266946	0.000193291	Bloc1s6/Chmp5/Cplx1/Grik5/Pla2g4a/Rab34/Rab3a/Rab8b/Snap25/Stx3/Stxbp6/Vamp3	12
BP	GO:0009150	purine ribonucleotide metabolic process	22/607	345/28814	3.03	4.91757E-06	0.000266946	0.000193291	Acot1/Acot11/Adcy3/Adcy8/Ak2/Atp5pb/Coasy/Dcakd/Dip2a/Eif6/Eno2/Enpp1/Gucy1b1/Hk2/Hmgcs1/Nme3/Nudt7/P2rx7/Pdk2/Stat3/Tp	22
BP	GO:0043087	regulation of GTPase activity	22/607	345/28814	3.03	4.91757E-06	0.000266946	0.000193291	Arhgap6/Arhgef10/Bcar3/Chn1/Dab2ip/Dgki/Gnb5/Ngef/Ntrk3/Picalm/Pip5k1a/Plxnc1/Ralgps2/Rap1gap/Rasgrp2/Rcc2/Rgs8/Rsu1/Sod1/	22
BP	GO:0022411	cellular component disassembly	25/607	427/28814	2.78	5.13569E-06	0.000274003	0.000198401	Actn2/Arhgef2/Bax/C1qc/Chmp5/Cracd/Endog/Fgf13/Fundc1/Fundc2/Hk2/Hspa2/Irgm1/Klc1/Lamp2/Lcp1/Ngef/Ogfod1/Pdpn/Rpl23/Scaf8/Sh3gl3/Slc25a5/Tmod1/	25
BP	GO:0006753	nucleoside phosphate metabolic process	26/607	455/28814	2.71	5.16228E-06	0.000274003	0.000198401	Acot1/Acot11/Adcy3/Adcy8/Ak2/Ak5/Atp5pb/Coasy/Dcakd/Dip2a/Dnph1/Eif6/Eno2/Enpp1/Gucy1b1/Hk2/Hmgcs1/Naprt/Nme3/Nudt12/Nudt7/P2rx7/Pdk2/Stat3/Tpi1	26
BP	GO:0016311	dephosphorylation	22/607	347/28814	3.01	5.39234E-06	0.000283068	0.000204965	Arpp19/Bod1/Ensa/Inpp5j/Mef2c/Mtmr12/Pip4p1/Ppp1cc/Ppp1r16b/Ppp2r2a/Ppp4r1/Ppp4r4/Ptpa/Ptpn5/Ptpn6/Rcan1/Sdhaf2/Sgpp1/Sm	22
BP	GO:0003012	muscle system process	25/607	430/28814	2.76	5.79873E-06	0.000301093	0.000218016	Actn2/Atp2b1/Atp2b4/Bbs2/Ctnna3/Dbn1/Drd1/Ednrb/Fgf13/Flna/Foxo1/Igfbp5/Lmna/Mef2c/Myl4/Ptgs1/Ryr3/Scn1b/Setd3/Smpd3/Sod1/Tmem38a/Tmod1/Tpm4/Y	25
BP	GO:0042063	gliogenesis	22/607	350/28814	2.98	6.18282E-06	0.000317584	0.000229957	Adgrg1/Arhgef10/B4galt6/Cdh2/Cdk5r2/Clu/Dab2ip/Drd1/Enpp1/Gap43/Gfap/Gjc2/Hexb/Lingo1/Mapk3/Nlgn3/Ntrk3/Ppp1cc/Sod1/Sox8/	22
BP	GO:0050773	regulation of dendrite development	13/607	137/28814	4.5	6.9706E-06	0.00035424	0.000256499	Ache/Camk1d/Camk2b/Dab2ip/Dbn1/Dbnl/Dhx36/Eef2k/Itpka/Marcks/Pacsin1/Sez	13
BP	GO:0034764	positive regulation of transmembrane transport	18/607	253/28814	3.38	8.08312E-06	0.000406453	0.000294306	Actn2/Bax/Dnm2/Drd1/Flna/Gjc2/Hap1/Hspa2/Kcnc1/Kenip2/Nlgn3/Osbpl8/P2rx7/Plcg2/Rgs9/Slc6a9/Sorbs1	18
BP	GO:0007163	establishment or maintenance of cell polarity	17/607	229/28814	3.52	8.21999E-06	0.00040903	0.000296172	Actb/Arhgef2/Brsk1/Coro7/Fgf13/Frmd4a/Frmd4b/Lmna/Msn/Nek3/Parvb/Ptk7/Rac1/Rac2/Rhob/Rhof/Vangl2	17
BP	GO:0015850	organic hydroxy compound transport	18/607	255/28814	3.35	9.00423E-06	0.000442979	0.000320753	Abca1/Abca8b/Abcd3/Actb/Anxa2/Drd1/Gramd1a/Kcnb1/Nucb2/Prkcb/Ptgs1/Rab3a/Slc10a4/Slc18a2/Slc6a3/	18
BP	GO:0071902	positive regulation of protein serine/threonine kinase activity	16/607	207/28814	3.67	9.08769E-06	0.000442979	0.000320753	Atp2b4/Camk1/Cdk5r2/Dab2ip/Dipk2a/Gab1/Irgm1/Map2k2/Map3k13/Marcks/Ntrk3/P2rx7/Syap1/Tgfa/Traf	16
BP	GO:0043547	positive regulation of GTPase activity	18/607	256/28814	3.34	9.49921E-06	0.000458361	0.000331892	Arhgap6/Arhgef10/Bcar3/Chn1/Gnb5/Ngef/Ntrk3/Picalm/Pip5k1a/Ralgps2/Rap1gap/Rasgrp2/Rcc2/Rgs8/Rsu1/	18
BP	GO:0032869	cellular response to insulin stimulus	16/607	208/28814	3.65	9.65673E-06	0.000461302	0.000334021	Atp2b1/Bcar3/Enpp1/Foxo1/Grb10/Marcks/Nucb2/Nucks1/Osbpl8/Pdk2/Prkcb/Rab31/Rab8b/Slc25a33/Sorbs	16
BP	GO:0110156	methylguanosine-cap decapping	5/607	15/28814	15.82	1.02881E-05	0.000486595	0.000352336	Cnot7/Dcp1a/Dcps/Noct/Nudt12	5
BP	GO:0006892	post-Golgi vesicle-mediated transport	11/607	102/28814	5.12	1.04374E-05	0.00048882	0.000353947	Bbs2/Dnm2/Klhl20/Myo1b/Rab14/Rab26/Rab31/Rab34/Stxbp6/Vamp3/Wipi1	11
BP	GO:0009259	ribonucleotide metabolic process	22/607	364/28814	2.87	1.14498E-05	0.000531028	0.000384508	Acot1/Acot11/Adcy3/Adcy8/Ak2/Atp5pb/Coasy/Dcakd/Dip2a/Eif6/Eno2/Enpp1/Gucy1b1/Hk2/Hmgcs1/Nme3/Nudt7/P2rx7/Pdk2/Stat3/Tp	22
BP	GO:0072521	purine-containing compound metabolic process	23/607	392/28814	2.79	1.17474E-05	0.000539591	0.000390709	Acot1/Acot11/Adcy3/Adcy8/Ak2/Atp5pb/Coasy/Dcakd/Dip2a/Dnph1/Eif6/Eno2/Enpp1/Gucy1b1/Hk2/Hmgcs1/Nme3/Nudt7/P2rx7/Pdk2/St	23
BP	GO:0098876	vesicle-mediated transport to the plasma membrane	13/607	144/28814	4.29	1.20021E-05	0.000546037	0.000395376	Actn2/Bbs2/Cplx1/Dnm2/Ndrg4/Rab14/Rab26/Rab31/Rab34/Snap25/Stx3/Stxbp6/Vamp3	13
BP	GO:0032956	regulation of actin cytoskeleton organization	22/607	366/28814	2.85	1.24676E-05	0.000561718	0.000406731	Actn2/Arhgap6/Arhgef10/Arpc1b/Arpc3/Arpc5l/Cracd/Dbn1/Dbnl/Flna/Kank4/Mef2c/Ntrk3/Pdlim4/Rac1/Rac2/Rhob/Rhof/Ssh3/Tmod1/V	22
BP	GO:0010770	positive regulation of cell morphogenesis involved in differentiation	11/607	104/28814	5.02	1.25819E-05	0.000561718	0.000406731	Ache/Camk2b/Dbn1/Dbnl/Dhx36/Dnm2/Eef2k/Flna/Itpka/Marcks/Rac1	11
BP	GO:0007212	dopamine receptor signaling pathway	7/607	38/28814	8.74	1.27292E-05	0.000563033	0.000407683	Drd1/Flna/Gnal/Gnb5/Nsg2/Rgs8/Rgs9	7

BP	GO:0036465	synaptic vesicle recycling	7/361	81/28814	6.9	7.17693E-05	0.00385971	0.003001867	Dennd1a/Ppp3cc/Sh3gl1/Sh3gl2/Slc17a7/Sncb/Stx1a	7
BP	GO:0006163	purine nucleotide metabolic process	15/361	369/28814	3.24	7.42186E-05	0.003933587	0.003059324	Ampd3/Atp2b2/Coasy/Gcdh/Mvd/Mvk/Nudt3/Nudt4/Pdk1/Pdk3/Ppat/Prkaa1/Prps1/Slc4a1/Stat3	15
BP	GO:1904375	regulation of protein localization to cell periphery	9/361	141/28814	5.09	7.54809E-05	0.003943337	0.003066907	Adam10/Camk2a/Camk2d/Dlg1/Epb41l3/Nrxn1/Numb/Ppp1r9b/Sorbs1	9
BP	GO:0043647	inositol phosphate metabolic process	6/361	57/28814	8.4	7.85713E-05	0.003971061	0.003088469	Inpp5b/Itpka/Nudt3/Nudt4/Plcb1/Ppip5k2	6
BP	GO:0086001	cardiac muscle cell action potential	6/361	57/28814	8.4	7.85713E-05	0.003971061	0.003088469	Ank3/Atp2a2/Camk2d/Dlg1/Scn4b/Smao	6
BP	GO:0043270	positive regulation of ion transport	14/361	329/28814	3.4	7.92692E-05	0.003971061	0.003088469	Ank3/Atp2b1/Atp2b2/Cacnb3/Calm1/Camk2a/Cck/Dlg1/Kcna1/Prpk4/Pdk1/S100a1/Scn4b/Slc12a2	14
BP	GO:0031032	actomyosin structure organization	11/361	213/28814	4.12	8.52794E-05	0.004214418	0.003277739	Cd47/Csrp1/Epb41l2/Foxp1/Pkn/Rock2/Smad4/Sorbs1/Sorbs3/Tjp1/Tmod1	11
BP	GO:0071806	protein transmembrane transport	6/361	58/28814	8.26	8.67212E-05	0.004228528	0.003288713	Abca1/Pex1/Pex13/Sec61a1/Sec61a2/Timm23	6
BP	GO:2001135	regulation of endocytic recycling	4/361	20/28814	15.96	0.000100169	0.004819985	0.003748715	Arfgap1/Atp8a/Ehd2/Rab11b	4
BP	GO:0098815	modulation of excitatory postsynaptic potential	6/361	60/28814	7.98	0.000105035	0.004988496	0.003879774	Cacnb3/Cplx4/Nrxn1/Rims2/Sh3gl1/Stx1a	6
BP	GO:0035418	protein localization to synapse	7/361	87/28814	6.42	0.000113119	0.005303544	0.004124801	Adam10/Camk2a/Cplx1/Dlg1/Map1a/Nptx1/Nrxn1	7
BP	GO:0048172	regulation of short-term neuronal synaptic plasticity	4/361	21/28814	15.2	0.00012252	0.00567157	0.004411031	Bdnf/Shisa9/Syap1/Syn1	4
BP	GO:0009150	purine ribonucleotide metabolic process	14/361	345/28814	3.24	0.000130553	0.005967907	0.004641505	Ampd3/Atp2b2/Coasy/Gcdh/Mvd/Mvk/Nudt3/Nudt4/Pdk1/Pdk3/Ppat/Prkaa1/Slc4a1/Stat3	14
BP	GO:0006835	dicarboxylic acid transport	7/361	90/28814	6.21	0.000140077	0.006288937	0.004891184	Bdnf/Cck/Slc12a2/Slc17a7/Slc25a10/Slc25a14/Slc26a6	7
BP	GO:0071326	cellular response to monosaccharide stimulus	9/361	153/28814	4.7	0.000141015	0.006288937	0.004891184	Nrxn1/Pck2/Pdk3/Plcb1/Ppp3ca/Prkaa1/Rab11b/Slc26a6/Smad4	9
BP	GO:0072521	purine-containing compound metabolic process	15/361	392/28814	3.05	0.000144464	0.006365097	0.004950417	Ampd3/Atp2b2/Coasy/Gcdh/Mvd/Mvk/Nudt3/Nudt4/Pdk1/Pdk3/Ppat/Prkaa1/Prps1/Slc4a1/Stat3	15
BP	GO:1902414	protein localization to cell junction	8/361	121/28814	5.28	0.000147872	0.006437714	0.005006894	Adam10/Camk2a/Cplx1/Dlg1/Map1a/Nptx1/Nrxn1/Tjp1	8
BP	GO:0034329	cell junction assembly	16/361	441/28814	2.9	0.000160868	0.00692129	0.005382868	Add2/Bdnf/Coro1c/Dlg1/Kirrel3/Lims1/Mdga2/Nptx1/Nrxn1/Numb/Pxn/Rock2/Sema4a/Sorbs1/Tjp1/Tpbg	16
BP	GO:0071375	cellular response to peptide hormone stimulus	12/361	269/28814	3.56	0.000164436	0.006962634	0.005415148	Atp2b1/Camk2a/Pdk2/Pdk4/Plcb1/Prkaa1/Pxn/Rock2/Slc26a6/Sorbs1/Stat3/Svep1	12
BP	GO:0006612	protein targeting to membrane	8/361	123/28814	5.19	0.000165641	0.006962634	0.005415148	Ank3/Cacnb3/Pex13/Rab3a/Rabgef1/Sec61a1/Sec61a2/Sgtb	8
BP	GO:0008360	regulation of cell shape	9/361	157/28814	4.58	0.000171358	0.007121086	0.005538384	Cfdp1/Dlg1/Epb41l2/Myl12b/Plxnc1/Ptf40a/Pxn/Sema4a/Zmpste24	9
BP	GO:0009306	protein secretion	15/361	399/28814	3	0.000175009	0.007191098	0.005592835	Abca1/Apbb1/C1qtnf5/Cplx1/Cplx3/Frmd4a/Glul/Nrxn1/Pcsk1/Ppp3ca/Preb/Rab11b/Rims2/Slc12a2/Stx1a	15
BP	GO:0035592	establishment of protein localization to extracellular region	15/361	400/28814	2.99	0.0001798	0.00722653	0.005620487	Abca1/Apbb1/C1qtnf5/Cplx1/Cplx3/Frmd4a/Glul/Nrxn1/Pcsk1/Ppp3ca/Preb/Rab11b/Rims2/Slc12a2/Stx1a	15
BP	GO:1901653	cellular response to peptide	13/361	313/28814	3.32	0.000179826	0.00722653	0.005620487	Atp2b1/Camk2a/Pdk2/Pdk4/Plcb1/Prkaa1/Pxn/Rock2/Slc26a6/Snx6/Sorbs1/Stat3/Syap1	13
BP	GO:0017038	protein import	11/361	233/28814	3.77	0.000187722	0.007461967	0.005803501	Ipo8/Nup188/Pex1/Pex13/Pex19/Ppp3ca/Ppp3r1/Ranbp6/Sec13/Stat3/Timm23	11
BP	GO:0019722	calcium-mediated signaling	10/361	196/28814	4.07	0.00019621	0.007715498	0.006000684	Atp2a2/Homer2/L1cam/Ppp1r9b/Ppp3ca/Ppp3cc/Ppp3r1/Prkaa1/Slc8a1/Zmpste24	10
BP	GO:0007613	memory	9/361	161/28814	4.46	0.000206955	0.007867012	0.006118523	Bdnf/Cck/Eif2ak4/Jph3/Map1a/Pde1/Plcb1/Slc17a7/Slc24a2	9
BP	GO:0071322	cellular response to carbohydrate stimulus	9/361	161/28814	4.46	0.000206955	0.007867012	0.006118523	Nrxn1/Pck2/Pdk3/Plcb1/Ppp3ca/Prkaa1/Rab11b/Slc26a6/Smad4	9
BP	GO:0071692	protein localization to extracellular region	15/361	406/28814	2.95	0.000211006	0.007867012	0.006118523	Abca1/Apbb1/C1qtnf5/Cplx1/Cplx3/Frmd4a/Glul/Nrxn1/Pcsk1/Ppp3ca/Preb/Rab11b/Rims2/Slc12a2/Stx1a	15
BP	GO:0031629	synaptic vesicle fusion to presynaptic active zone membrane	4/361	24/28814	13.3	0.000211166	0.007867012	0.006118523	Cplx1/Cplx3/Stx1a/Stx2	4
BP	GO:0034284	response to monosaccharide	10/361	198/28814	4.03	0.000212971	0.007867012	0.006118523	Glul/Nrxn1/Pck2/Pdk3/Plcb1/Ppp3ca/Prkaa1/Rab11b/Slc26a6/Smad4	10
BP	GO:0050905	neuromuscular process	10/361	198/28814	4.03	0.000212971	0.007867012	0.006118523	Atp2b2/Atp8a2/Jph3/Kcna1/Kcnab2/Map1a/Mycbp2/Nrxn1/Scn8a/Zmpste24	10
BP	GO:0099541	trans-synaptic signaling by lipid	3/361	10/28814	23.95	0.000219234	0.007936211	0.006172342	Abhd6/Nrxn1/Plcb1	3
BP	GO:0099542	trans-synaptic signaling by endocannabinoid	3/361	10/28814	23.95	0.000219234	0.007936211	0.006172342	Abhd6/Nrxn1/Plcb1	3
BP	GO:0034504	protein localization to nucleus	13/361	320/28814	3.24	0.000222992	0.007936211	0.006172342	Arl2bp/Dnajb6/Glul/Hdgf/Ipo8/Maged1/Nup188/Ppp3ca/Ppp3r1/Ranbp6/Sec13/Sko1/Stat3	13
BP	GO:0002028	regulation of sodium ion transport	7/361	97/28814	5.76	0.000223525	0.007936211	0.006172342	Ank3/Atp1a2/Camk2d/Plcb1/Scn4b/Slc9a1/Smaop	7
BP	GO:0006979	response to oxidative stress	15/361	409/28814	2.93	0.000228294	0.008027609	0.006243426	Atp2a2/Cat/Cza/Chchd2/Coq7/Foxp1/Ndufa12/Oxr1/Pdk1/Prkaa1/Prkra/Sirpa/Slc4a1/Slc8a1/Stx2	15
BP	GO:1905475	regulation of protein localization to membrane	10/361	200/28814	3.99	0.000230917	0.008042507	0.006255013	Adam10/Ank3/Cacnb3/Camk2a/Camk2d/Dlg1/Nrxn1/Numb/Ppp1r9b/Sorbs1	10
BP	GO:0099500	vesicle fusion to plasma membrane	4/361	25/28814	12.77	0.000248916	0.008507338	0.006616533	Cplx1/Cplx3/Stx1a/Stx2	4
BP	GO:2000311	regulation of AMPA receptor activity	4/361	25/28814	12.77	0.000248916	0.008507338	0.006616533	Cnih2/Gsg1l/Nrxn1/Shisa9	4
BP	GO:0017156	calcium-ion regulated exocytosis	6/361	71/28814	6.75	0.000267735	0.009016879	0.007012825	Atp2a2/Cadps/Cadps2/Cbarp/Rims2/Stx1a	6

BP	GO:0051503	adenine nucleotide transport	3/255	21/28814	16.14	0.000809679	0.034328008	0.029755076	Slc25a4/Slc25a42/Slc25a5	3
BP	GO:0016054	organic acid catabolic process	8/255	221/28814	4.09	0.000830978	0.034720421	0.030095215	Acot2/Akt2/Bcat2/Cbs/Cpt1a/Ddah2/Gcat/Gls2	8
BP	GO:0030433	ubiquitin-dependent ERAD pathway	5/255	84/28814	6.73	0.000912181	0.037568818	0.032564169	Dnajc10/Ubqln2/Ubxn4/Wfs1/Yod1	5
BP	GO:0015865	purine nucleotide transport	3/255	22/28814	15.41	0.000931419	0.037820868	0.032782643	Slc25a4/Slc25a42/Slc25a5	3
BP	GO:0006720	isoprenoid metabolic process	5/255	87/28814	6.49	0.001068608	0.042788846	0.037088822	Aldh1a1/Amacr/Rbp1/Rdh14/Ttr	5
BP	GO:0006979	response to oxidative stress	11/255	409/28814	3.04	0.001127851	0.044542394	0.038608775	Aldh1a1/Dhfr/Gpr37l1/Hmox2/Kpna4/Pde8a/Psen1/Rnf112/Rrm2b/Stx2/Tor1a	11
BP	GO:0030100	regulation of endocytosis	8/255	237/28814	3.81	0.00130024	0.05065664	0.043908525	Ap2m1/Dnm1/Ighm/Mkln1/Rabgef1/Rock1/Tor1a/Ubqln2	8
BP	GO:0060047	heart contraction	8/255	238/28814	3.8	0.001335402	0.051332864	0.044494667	Abcc9/Atp1b1/Chrm2/Glrx3/Kcnh2/Scn3b/Slc9a1/Th	8
BP	GO:0031623	receptor internalization	6/255	137/28814	4.95	0.001417714	0.053779863	0.046615695	Ap2m1/Cacng8/Cd81/Dnm1/Mkln1/Ubqln2	6
BP	GO:0045727	positive regulation of translation	6/255	138/28814	4.91	0.001471379	0.055090706	0.047751917	Cpeb3/Eif5a2/Guf1/Pym1/Ybx1/Ythdf2	6
BP	GO:2000765	regulation of cytoplasmic translation	3/255	26/28814	13.04	0.001532015	0.056355103	0.04884788	Cpeb3/Ybx1/Ythdf2	3
BP	GO:0070482	response to oxygen levels	9/255	302/28814	3.37	0.001568464	0.056355103	0.04884788	Acot2/Atp1b1/Cbs/Hmox2/Nop53/Scap/Slc8a3/Slc9a1/Th	9
BP	GO:0009063	cellular amino acid catabolic process	5/255	95/28814	5.95	0.001582667	0.056355103	0.04884788	Bcat2/Cbs/Ddah2/Gcat/Gls2	5
BP	GO:0035966	response to topologically incorrect protein	6/255	140/28814	4.84	0.001583338	0.056355103	0.04884788	Dnajc10/Tmem33/Tor1a/Ubxn4/Wfs1/Yod1	6
BP	GO:1900271	regulation of long-term synaptic potentiation	4/255	57/28814	7.93	0.001638065	0.05740254	0.049755784	Cpeb3/Cyp46a1/Mme/Nptn	4
BP	GO:0043161	proteasome-mediated ubiquitin-dependent protein catabolic process	11/255	430/28814	2.89	0.001676372	0.05740254	0.049755784	Ascc3/Dda1/Dnajc10/Fbxo31/Nop53/Psen1/Rybp/Ubqln2/Ubxn4/Wfs1/Yod1	11
BP	GO:0006862	nucleotide transport	3/255	27/28814	12.56	0.00171232	0.05740254	0.049755784	Slc25a4/Slc25a42/Slc25a5	3
BP	GO:0009713	catechol-containing compound biosynthetic process	3/255	27/28814	12.56	0.00171232	0.05740254	0.049755784	Ddc/Slc6a3/Th	3
BP	GO:0042423	catecholamine biosynthetic process	3/255	27/28814	12.56	0.00171232	0.05740254	0.049755784	Ddc/Slc6a3/Th	3
BP	GO:0001523	retinoid metabolic process	4/255	58/28814	7.79	0.001747321	0.057902588	0.05018922	Aldh1a1/Rbp1/Rdh14/Ttr	4
BP	GO:1990778	protein localization to cell periphery	10/255	370/28814	3.05	0.001808773	0.058935364	0.051084417	Akt2/Ap2m1/Atp1b1/Cd81/Grip1/Lypla1/Phaf1/Pip5k1a/Rock1/Scn3b	10
BP	GO:0003015	heart process	8/255	250/28814	3.62	0.001819371	0.058935364	0.051084417	Abcc9/Atp1b1/Chrm2/Glrx3/Kcnh2/Scn3b/Slc9a1/Th	8
BP	GO:0032469	endoplasmic reticulum calcium ion homeostasis	3/255	28/28814	12.11	0.001905345	0.061034539	0.052903954	Psen1/Tgm2/Wfs1	3
BP	GO:0016101	diterpenoid metabolic process	4/255	60/28814	7.53	0.001980806	0.062424833	0.054109043	Aldh1a1/Rbp1/Rdh14/Ttr	4
BP	GO:0050905	neuromuscular process	7/255	198/28814	3.99	0.001992052	0.062424833	0.054109043	Fgf12/Icmt/Opa3/Pnkd/Rbfox1/Slc6a3/Slc8a3	7
BP	GO:0001508	action potential	6/255	147/28814	4.61	0.002026841	0.062832078	0.054462038	Fgf12/Kcnh2/Scn3b/Slc8a2/Slc9a1/Ywhah	6
BP	GO:0140115	export across plasma membrane	4/255	61/28814	7.41	0.002105265	0.064568912	0.055967504	Atp1b1/Kcnh2/Slc8a2/Slc8a3	4
BP	GO:0034976	response to endoplasmic reticulum stress	8/255	257/28814	3.52	0.002159895	0.065159779	0.05647966	Dnajc10/Pdia4/Tmem33/Tor1a/Ubqln2/Ubxn4/Wfs1/Yod1	8
BP	GO:0016358	dendrite development	9/255	317/28814	3.21	0.002178464	0.065159779	0.05647966	Arid1b/Celsr2/Cpeb3/Cux1/Fbxo31/Grip1/Iqgap1/Psen1/Ywhah	9
BP	GO:0006584	catecholamine metabolic process	4/255	62/28814	7.29	0.002235018	0.065159779	0.05647966	Ddc/Pnkd/Slc6a3/Th	4
BP	GO:0009712	catechol-containing compound metabolic process	4/255	62/28814	7.29	0.002235018	0.065159779	0.05647966	Ddc/Pnkd/Slc6a3/Th	4
BP	GO:1904062	regulation of cation transmembrane transport	10/255	381/28814	2.97	0.002237537	0.065159779	0.05647966	Abcc9/Atp1b1/Cacng8/Fgf12/Kcnc1/Kcnh2/Scn3b/Slc25a4/Slc9a1/Ywhah	10
BP	GO:0010001	glial cell differentiation	8/255	259/28814	3.49	0.002265852	0.065324517	0.056622452	Akt2/C1qa/Gpr37l1/Nfib/Psen1/Rnf112/Slc8a3/Tnfrsf21	8
BP	GO:0002029	desensitization of G protein-coupled receptor signaling pathway	3/255	30/28814	11.3	0.002330776	0.065878701	0.057102812	Dnm1/Grk6/Ubqln2	3
BP	GO:0022401	negative adaptation of signaling pathway	3/255	30/28814	11.3	0.002330776	0.065878701	0.057102812	Dnm1/Grk6/Ubqln2	3
BP	GO:0006839	mitochondrial transport	6/255	153/28814	4.43	0.002476674	0.069322819	0.060088129	Pmpcb/Psen1/Slc25a4/Slc25a5/Slc9a1/Timm8a1	6
BP	GO:0086010	membrane depolarization during action potential	3/255	31/28814	10.94	0.002563767	0.070393711	0.061016364	Kcnh2/Scn3b/Ywhah	3
BP	GO:1900273	positive regulation of long-term synaptic potentiation	3/255	31/28814	10.94	0.002563767	0.070393711	0.061016364	Cpeb3/Mme/Nptn	3
BP	GO:1903522	regulation of blood circulation	8/255	266/28814	3.4	0.002669211	0.07164873	0.062104199	Abcc9/Atp1b1/Chrm2/Glrx3/Kcnh2/Scn3b/Slc9a1/Th	8
BP	GO:0010563	negative regulation of phosphorus metabolic process	11/255	457/28814	2.72	0.002684032	0.07164873	0.062104199	Dnajc10/Entpd1/Inpp5j/Iqgap1/Ppm1e/Psen1/Rabgef1/Rock1/Slc8a3/Stk38/Tiprl	11
BP	GO:0045936	negative regulation of phosphate metabolic process	11/255	457/28814	2.72	0.002684032	0.07164873	0.062104199	Dnajc10/Entpd1/Inpp5j/Iqgap1/Ppm1e/Psen1/Rabgef1/Rock1/Slc8a3/Stk38/Tiprl	11

BP	GO:0006936	muscle contraction	20/607	314/28814	3.02	1.33566E-05	0.000585362	0.000423851	Actn2/Atp2b1/Atp2b4/Bbs2/Ctnna3/Dbn1/Drd1/Ednrb/Fgf13/Flna/Myl4/Ptgs1/Ryr3/Scn1b/Setd3/Smpd3/Sod1	20
BP	GO:2000112	regulation of cellular macromolecule biosynthetic process	26/607	481/28814	2.57	1.36987E-05	0.000594899	0.000430756	Cnot7/Coa3/Dcp1a/Dcps/Dhx36/Dph5/Eif6/Enpp1/Grb10/Igfbp5/Jak3/Mapk3/Mrpl13/Ncbp2/Noct/Ogfod1/Paip1/Pld1/Pum3/Rps9/Slc6a9/Sorbs1/Taco1/Tia1/Tut4/Vi	26
BP	GO:0099601	regulation of neurotransmitter receptor activity	9/607	69/28814	6.19	1.41693E-05	0.000609792	0.000441541	Dlgap4/Mef2c/Nlgn3/Nptx1/Prrt1/Rgs9/Shisa7/Shisa9/Slc6a9	9
BP	GO:0015844	monoamine transport	11/607	106/28814	4.93	1.5099E-05	0.000643999	0.000466309	Actb/Drd1/Kcnb1/Prkcb/Ptgs1/Rab3a/Slc18a2/Slc6a3/Smpd3/Sncg/Syt17	11
BP	GO:0006898	receptor-mediated endocytosis	18/607	266/28814	3.21	1.59683E-05	0.000671712	0.000486376	Ache/Anxa2/Ap4e1/B2m/Clu/Dbnl/Dnm2/Fcho1/Hap1/Nlgn3/Pacsin1/Picalm/Plcg2/Ptpn5/Rab31/Sh3gl3/Snap25	18
BP	GO:0048872	homeostasis of number of cells	22/607	372/28814	2.81	1.603E-05	0.000671712	0.000486376	Adgrg1/B2m/Bap1/Bax/Cdh2/Dnaja3/Enpp1/Heph/Hmgb1/Hmgb2/Ildr2/Jak3/Kdm1a/Lipa/Mef2c/P2rx7/Rac1/Setd1a/Slc25a5/Sod1/Stat1/	22
BP	GO:0051937	catecholamine transport	10/607	88/28814	5.39	1.65138E-05	0.000685967	0.000496698	Actb/Drd1/Kcnb1/Prkcb/Ptgs1/Rab3a/Slc6a3/Smpd3/Sncg/Syt17	10
BP	GO:0050805	negative regulation of synaptic transmission	10/607	89/28814	5.33	1.82576E-05	0.000751867	0.000544414	Adcy8/Asic1/Celf4/Dgki/Drd1/Gnal/Kcnb1/Nlgn3/Pnkd/Prrt1	10
BP	GO:0019693	ribose phosphate metabolic process	22/607	376/28814	2.78	1.88903E-05	0.000771272	0.000558465	Acot1/Acot11/Adcy3/Adcy8/Ak2/Atp5pb/Coasy/Dcakd/Dip2a/Eif6/Eno2/Enpp1/Gucy1b1/Hk2/Hmgcs1/Nme3/Nudt7/P2rx7/Pdk2/Stat3/Tp	22
BP	GO:0051017	actin filament bundle assembly	14/607	173/28814	3.84	1.9533E-05	0.000790435	0.000572341	Aif1l/Arhgap6/Arhgef10/Dbn1/Flna/Kank4/Lcp1/Marcks/Myo1b/Pdlim4/Phactr1/Pls1/Rac1/Sorbs1	14
BP	GO:0106027	neuron projection organization	11/607	109/28814	4.79	1.96906E-05	0.000790435	0.000572341	Camk2b/Dbn1/Dbnl/Dhx36/Dip2a/Eef2k/Itpka/Marcks/Ngef/Nlgn3/Pls1	11
BP	GO:0110154	RNA decapping	5/607	17/28814	13.96	2.04715E-05	0.000809475	0.000586127	Cnot7/Dcp1a/Dcps/Noct/Nudt12	5
BP	GO:0009991	response to extracellular stimulus	23/607	406/28814	2.69	2.05038E-05	0.000809475	0.000586127	Bbs2/Brsk1/Comt/Dap/Dcps/Elapor1/Enpp1/Foxo1/Htr4/Kcnb1/Kptn/Lamp2/Mapk3/Meak7/Noct/Nucb2/P2rx7/Pdk2/Prkag1/Rragb/Sod1/	23
BP	GO:0048499	synaptic vesicle membrane organization	6/607	28/28814	10.17	2.16406E-05	0.000847354	0.000613555	Cplx1/Grik5/Rab3a/Snap25/Stx3/Syngr1	6
BP	GO:0061572	actin filament bundle organization	14/607	176/28814	3.78	2.36825E-05	0.000919768	0.000665989	Aif1l/Arhgap6/Arhgef10/Dbn1/Flna/Kank4/Lcp1/Marcks/Myo1b/Pdlim4/Phactr1/Pls1/Rac1/Sorbs1	14
BP	GO:0043270	positive regulation of ion transport	20/607	329/28814	2.89	2.61127E-05	0.00100597	0.000728406	Actn2/Atp2b1/Bax/Dnm2/Drd1/Flna/Gjc2/Hap1/Hspa2/Jak3/Kcnc1/Kcnip2/Nlgn3/P2rx7/Pla2g4a/Plcg2/Rgs9/	20
BP	GO:0006650	glycerophospholipid metabolic process	18/607	277/28814	3.08	2.74055E-05	0.001047329	0.000758354	Abhd4/Cds1/Dgki/Gpcpd1/Hdhd5/Inpp5j/Itpka/Mtmr12/Pcyt1b/Pigt/Pip4p1/Pip5k1a/Pitpnc1/Pla2g4a/Pla2g7/	18
BP	GO:0045860	positive regulation of protein kinase activity	21/607	358/28814	2.78	2.80011E-05	0.001061596	0.000768684	Adcy8/Atp2b4/Camk1/Camkk2/Cdk5r2/Clu/Dab2ip/Dipk2a/Gab1/Irgm1/Map2k2/Map3k13/Marcks/Ntrk3/Osbpl8/P2rx7/Syap1/Tgfa/Traf	21
BP	GO:1990138	neuron projection extension	15/607	204/28814	3.49	3.08919E-05	0.001161974	0.000841367	Dbn1/Dbnl/Dip2b/Dnm2/Kdm1a/Map3k13/Nlgn3/Nrp2/Ntrk3/Picalm/Rnf157/Se	15
BP	GO:0050775	positive regulation of dendrite morphogenesis	8/607	59/28814	6.44	3.19063E-05	0.001190755	0.000862206	Ache/Camk2b/Dbn1/Dbnl/Dhx36/Eef2k/Itpka/Marcks	8
BP	GO:2001257	regulation of cation channel activity	14/607	181/28814	3.67	3.23346E-05	0.001197382	0.000867004	Actn2/Fgf13/Gnb5/Hap1/Kcnc1/Mef2c/Nlgn3/Plcg2/Prrt1/Rgs9/Scn1b/Shisa9/Slc6	14
BP	GO:0060560	developmental growth involved in morphogenesis	18/607	281/28814	3.04	3.31008E-05	0.001216328	0.000880723	Dbn1/Dbnl/Dip2b/Dnm2/Fgf13/Kdm1a/Map3k13/Nlgn3/Nrp2/Ntrk3/Picalm/Ptk7/Rnf157/Sema7a/Slc9a6/Syt	18
BP	GO:0016079	synaptic vesicle exocytosis	12/607	137/28814	4.16	3.48818E-05	0.001271988	0.000921026	Apba1/Apba2/Cplx1/Dgki/Drd1/Grik5/Nrxn3/P2rx7/Prkcb/Rab3a/Snap25/Stx3	12
BP	GO:0019932	second-messenger-mediated signaling	19/607	309/28814	2.92	3.53557E-05	0.001279501	0.000926466	Adcy8/Atp2b4/Camkk2/Drd1/Ednrb/Grm1/Gucy1b1/Homer2/Irgm1/Mcu/Nucb2/Pde10a/Pdk2/Plcg2/Rasd2/Rcan1/Rnf157/Tmem100/Tm	19
BP	GO:0019439	aromatic compound catabolic process	24/607	451/28814	2.53	3.73504E-05	0.001336308	0.000967598	Aldh1l1/Apex1/Bax/Cnot7/Comt/Dcp1a/Dcps/Dhx36/Dnph1/Endog/Enpp1/Exosc10/Hnrnpa0/Mtrex/Ncbp2/Noct/Nudt12/Nudt7/Paip1/Slc6a3/Smg5/Tut4/Ugt1a1/Vi	24
BP	GO:0007266	Rho protein signal transduction	12/607	138/28814	4.13	3.74849E-05	0.001336308	0.000967598	Abca1/Adgrg1/Arhgef10/Arhgef2/Cdh2/Hacd3/Pdpn/Phactr4/Plekhg5/Prag1/Srgap1/	12
BP	GO:0022898	regulation of transmembrane transporter activity	18/607	284/28814	3.01	3.80396E-05	0.001346038	0.000974644	Actb/Actn2/Dnm2/Fgf13/Gnb5/Hap1/Hspa2/Kcnc1/Mef2c/Nlgn3/Plcg2/Prrt1/Rgs9/Scn1b/Shisa9/Slc6a9/Stim1/	18
BP	GO:0072594	establishment of protein localization to organelle	23/607	426/28814	2.56	4.32422E-05	0.001518881	0.001099797	Bag3/Bax/Clu/Drd1/Flna/Hk2/Ipo7/Ipo8/Lamp2/Lmna/Mon1b/Pex1/Ptpn5/Rab8b/Ranbp6/Sec61b/Spcs1/Stam/Stat3/Timm13/Timm8a1/Ti	23
BP	GO:0030900	forebrain development	22/607	398/28814	2.62	4.45329E-05	0.0015528	0.001124357	Adgrg1/Aplp2/B2m/Bax/Bbs2/Ccdc85c/Cdh2/Cdk5r2/Dab2ip/Drd1/Fabp7/Fgf13/Gdpd5/Hap1/Kdm1a/Nrp2/Pcm1/Phactr1/Rac1/Rtn4rl2/S	22
BP	GO:0010563	negative regulation of phosphorus metabolic process	24/607	457/28814	2.49	4.60042E-05	0.00157718	0.001142011	Actb/Akt1s1/Arpp19/Bax/Bod1/Dab2ip/Dnaja3/Drd1/Dynll1/Enpp1/Ensa/Grb10/Inpp5j/Ntrk3/Pik3ip1/Ppp1r16b/Ppp4r4/Prkar1b/Prkar2b/Ptpa/Ptpn5/Ptpn6/Sirpa/Sta	24
BP	GO:0045936	negative regulation of phosphate metabolic process	24/607	457/28814	2.49	4.60042E-05	0.00157718	0.001142011	Actb/Akt1s1/Arpp19/Bax/Bod1/Dab2ip/Dnaja3/Drd1/Dynll1/Enpp1/Ensa/Grb10/Inpp5j/Ntrk3/Pik3ip1/Ppp1r16b/Ppp4r4/Prkar1b/Prkar2b/Ptpa/Ptpn5/Ptpn6/Sirpa/Sta	24
BP	GO:0009306	protein secretion	22/607	399/28814	2.62	4.62226E-05	0.00157718	0.001142011	Abca1/Adcy8/Bloc1s6/Copg1/Cplx1/Dynll1/Ednrb/Ensa/Frmd4a/Ildr2/Kcnb1/Mcu/Olfm2/P2rx7/Rab34/Rab3a/Rab3c/Rab8b/Rac1/Slc18a2	22
BP	GO:0042417	dopamine metabolic process	7/607	46/28814	7.22	4.6667E-05	0.001577848	0.001142494	Comt/Ddc/Drd1/Pde1b/Pnkd/Slc6a3/Th	7
BP	GO:0062012	regulation of small molecule metabolic process	21/607	371/28814	2.69	4.69802E-05	0.001577848	0.001142494	Arpp19/Atp2b4/Clcn2/Comt/Coq3/Dkk3/Eif6/Enpp1/Foxo1/Grb10/Hmgb1/Igfbp4/Nucb2/P2rx7/Pdk2/Pla2g4a/Sod1/Sorbs1/Stat3/Ugt1a1/	21
BP	GO:1900076	regulation of cellular response to insulin stimulus	9/607	80/28814	5.34	4.7233E-05	0.001577848	0.001142494	Atp2b1/Enpp1/Grb10/Marcks/Nucb2/Nucks1/Osbpl8/Prkcb/Sorbs1	9
BP	GO:0035592	establishment of protein localization to extracellular region	22/607	400/28814	2.61	4.79694E-05	0.001591319	0.001152248	Abca1/Adcy8/Bloc1s6/Copg1/Cplx1/Dynll1/Ednrb/Ensa/Frmd4a/Ildr2/Kcnb1/Mcu/Olfm2/P2rx7/Rab34/Rab3a/Rab3c/Rab8b/Rac1/Slc18a2	22
BP	GO:0031667	response to nutrient levels	21/607	373/28814	2.67	5.07485E-05	0.001664257	0.001205061	Bbs2/Brsk1/Comt/Dap/Dcps/Elapor1/Enpp1/Foxo1/Htr4/Kcnb1/Kptn/Lamp2/Mapk3/Meak7/Nucb2/Pdk2/Prkag1/Rragb/Sod1/Suox/Wipi	21
BP	GO:0006066	alcohol metabolic process	20/607	345/28814	2.75	5.08649E-05	0.001664257	0.001205061	Abca1/Abhd4/Aldh1a1/Aplp2/Clcn2/Coq3/Dkk3/Ednrb/Enpp1/Hmgcs1/Idh3a/Inpp5j/Itpka/Pip4p1/Pla2g4a/Plcg2/Sgpp1/Sod1/Thtpa/Tpi1	20
BP	GO:0043279	response to alkaloid	9/607	81/28814	5.27	5.21572E-05	0.001694932	0.001227273	Adcy8/Crebbp/Drd1/Gnal/Homer2/Ppp2r2a/Slc1a3/Slc6a3/Tmem38a	9
BP	GO:0031331	positive regulation of cellular catabolic process	24/607	461/28814	2.47	5.27331E-05	0.001702069	0.00123244	Bag3/Bax/Clu/Cnot7/Dab2ip/Dcp1a/Dcps/Dhx36/Elapor1/Endog/Foxo1/Hmgb1/Irgm1/Mapk3/Msn/Noct/P2rx7/Paip1/Rad23a/Rbx1/Slc25a	24
BP	GO:0050772	positive regulation of axonogenesis	10/607	101/28814	4.7	5.50633E-05	0.001753584	0.001269741	Ache/Dbn1/Dbnl/Map2k2/Map3k13/Ntrk3/Picalm/Plxnc1/Sema7a/Tiam2	10
BP	GO:0097061	dendritic spine organization	10/607	101/28814	4.7	5.50633E-05	0.001753584	0.001269741	Camk2b/Dbn1/Dbnl/Dhx36/Dip2a/Eef2k/Itpka/Marcks/Ngef/Nlgn3	10
BP	GO:0000290	deadenylation-dependent decapping of nuclear-transcribed mRNA	4/607	11/28814	17.26	5.72026E-05	0.001809648	0.001310337	Cnot7/Dcp1a/Dcps/Noct	4
BP	GO:0071692	protein localization to extracellular region	22/607	406/28814	2.57	5.97477E-05	0.001877728	0.001359632	Abca1/Adcy8/Bloc1s6/Copg1/Cplx1/Dynll1/Ednrb/Ensa/Frmd4a/Ildr2/Kcnb1/Mcu/Olfm2/P2rx7/Rab34/Rab3a/Rab3c/Rab8b/Rac1/Slc18a2	22
BP	GO:0048762	mesenchymal cell differentiation	16/607	242/28814	3.14	6.15244E-05	0.00192093	0.001390914	Cdh2/Dab2ip/Ednrb/Efnb1/Flna/Mapk3/Mef2c/Nrp2/Pdcd4/Pdpn/Phactr4/Sdhaf2/Sema7a/Sox8/Tmem100/Va	16

BP	GO:0060048	cardiac muscle contraction	8/361	132/28814	4.84	0.000268756	0.009016879	0.007012825	Atp1a1/Atp2a2/Calm1/Camk2d/Dlg1/Myl3/Scn4b/Slc8a1	8
BP	GO:0048168	regulation of neuronal synaptic plasticity	6/361	72/28814	6.65	0.000289031	0.009510859	0.007397015	Bdnf/Camk2a/Jph3/Shisa9/Syap1/Syn1	6
BP	GO:0006817	phosphate ion transport	4/361	26/28814	12.28	0.000291281	0.009510859	0.007397015	Slc17a7/Slc25a10/Slc25a14/Xpr1	4
BP	GO:0006883	cellular sodium ion homeostasis	4/361	26/28814	12.28	0.000291281	0.009510859	0.007397015	Atp1a1/Atp1a4/Slc12a2/Slc8a1	4
BP	GO:0016050	vesicle organization	12/361	287/28814	3.34	0.000297647	0.009591461	0.007459703	Abca1/Cadps/Coro1c/Cplx1/Cplx3/Lamtor1/Preb/Sec13/Slc17a7/Stx1a/Stx2/Vps8	12
BP	GO:0050974	detection of mechanical stimulus involved in sensory perception	5/361	47/28814	8.49	0.000298996	0.009591461	0.007459703	Atp2b2/Cacnb3/Kcna1/Phf24/Slc12a2	5
BP	GO:0043502	regulation of muscle adaptation	7/361	102/28814	5.48	0.000304659	0.009688147	0.0075349	Atp2a2/Camk2d/Cdk9/Foxp1/Ppp3ca/Rock2/Smad4	7
BP	GO:0017157	regulation of exocytosis	11/361	247/28814	3.55	0.000309844	0.0097681	0.007597083	Atp2a2/Atp9a/Cadps/Cadps2/Cbarp/Rab26/Rab2b/Rabgef1/Rims2/Secisn4/Stx1a	11
BP	GO:0048167	regulation of synaptic plasticity	14/361	378/28814	2.96	0.000332101	0.010380298	0.008073217	Abhd6/Atp2b2/Bdnf/Camk2a/Eif2ak4/Itpka/Jph3/Map1a/Rims2/Shisa9/Slc24a2/Stau1/Syap1/Syn1	14
BP	GO:0046390	ribose phosphate biosynthetic process	9/361	172/28814	4.18	0.000337768	0.010467951	0.008141388	Ampd3/Coasy/Gcdh/Pdk3/Prkg1/Ppat/Prps1/Stat3/Uprt	9
BP	GO:0062197	cellular response to chemical stress	13/361	336/28814	3.09	0.000356091	0.010842776	0.008432906	Atp2a2/Cat/Cys/Chchd2/Coq7/Foxp1/Oxr1/Pdk1/Prkaa1/Prkra/Sirpa/Slc8a1/Stx2	13
BP	GO:0044262	cellular carbohydrate metabolic process	12/361	293/28814	3.27	0.000358714	0.010842776	0.008432906	Arpp19/Gys1/Inpp5b/Itpka/Pck2/Pdk1/Pdk3/Ppp5k2/Slc4a1/Sorbs1/Stat3/Zmpste24	12
BP	GO:0010611	regulation of cardiac muscle hypertrophy	6/361	75/28814	6.39	0.000360983	0.010842776	0.008432906	Camk2d/Cdk9/Foxp1/Ppp3ca/Rock2/Smad4	6
BP	GO:0035196	production of miRNAs involved in gene silencing by miRNA	5/361	49/28814	8.14	0.000364165	0.010842776	0.008432906	Ppp3ca/Prkra/Stat3/Tut7/Zmpste24	5
BP	GO:0009743	response to carbohydrate	10/361	212/28814	3.76	0.000367342	0.010842776	0.008432906	Glul/Nrxn1/Pck2/Pdk3/Ptbp1/Ppp3ca/Prkaa1/Rab11b/Slc26a6/Smad4	10
BP	GO:0006941	striated muscle contraction	9/361	174/28814	4.13	0.000367652	0.010842776	0.008432906	Atp1a1/Atp1a2/Atp8a2/Calm1/Camk2d/Dlg1/Myl3/Scn4b/Slc8a1	9
BP	GO:0010038	response to metal ion	12/361	295/28814	3.25	0.000381292	0.011155082	0.008675801	Alg2/Ank3/Calm1/Cpne8/Kcna1/Kcnip3/Nptx1/Nrxn1/Ppp3ca/Prkaa1/Slc12a2/Slc30a3	12
BP	GO:0043161	proteasome-mediated ubiquitin-dependent protein catabolic process	15/361	430/28814	2.78	0.00038733	0.011168075	0.008685905	Ascc3/Cul2/Ecpas/Fbxl16/Fbxo2/Klhdc10/Klhl22/Map1a/Pcnp/Rad23a/Sgtb/Skp1/Sumo2/Trip4/Zyg11b	15
BP	GO:0140056	organelle localization by membrane tethering	6/361	76/28814	6.3	0.000387844	0.011168075	0.008685905	Atp2a2/Camk2a/Nrxn1/Rims2/Stx1a/Stx2	6
BP	GO:0048499	synaptic vesicle membrane organization	4/361	28/28814	11.4	0.000391129	0.011174678	0.008691041	Cplx1/Cplx3/Stx1a/Stx2	4
BP	GO:0042762	regulation of sulfur metabolic process	3/361	12/28814	19.95	0.000394506	0.011183774	0.008698115	Abcb7/Pdk1/Pdk3	3
BP	GO:0003300	cardiac muscle hypertrophy	7/361	107/28814	5.22	0.000407895	0.01147441	0.008924156	Atp2a2/Camk2d/Cdk9/Foxp1/Ppp3ca/Rock2/Smad4	7
BP	GO:0098661	inorganic anion transmembrane transport	6/361	77/28814	6.22	0.000416242	0.011615891	0.009034192	Gabra3/Gabrb1/Slc12a2/Slc25a10/Slc25a14/Xpr1	6
BP	GO:0046165	alcohol biosynthetic process	8/361	141/28814	4.53	0.000419277	0.011615891	0.009034192	Cln2/Dhcr7/Itpka/Mvd/Mvk/Pck2/Ppip5k2/Prkaa1	8
BP	GO:0009612	response to mechanical stimulus	9/361	178/28814	4.04	0.00043398	0.011932812	0.009280675	Atp1a1/Atp2b2/Atp8a2/Cacnb3/Kcna1/Nrxn1/Phf24/Slc12a2/Slc8a1	9
BP	GO:0014743	regulation of muscle hypertrophy	6/361	78/28814	6.14	0.000446236	0.012178259	0.009471571	Camk2d/Cdk9/Foxp1/Ppp3ca/Rock2/Smad4	6
BP	GO:1901888	regulation of cell junction assembly	10/361	220/28814	3.63	0.000491282	0.013221533	0.010282971	Bdnf/Coro1c/Lims1/Mdga2/Nptx1/Nrxn1/Rock2/Sema4a/Tjp1/Tpbg	10
BP	GO:0006816	calcium ion transport	15/361	440/28814	2.72	0.000491695	0.013221533	0.010282971	Atp2a2/Atp2b1/Atp2b2/Cacnb3/Calm1/Camk2a/Camk2d/Cbarp/Homer2/Jph3/Ppp3ca/S100a1/Slc24a2/Slc8a1/Zmpste24	15
BP	GO:0014897	striated muscle hypertrophy	7/361	111/28814	5.03	0.000509155	0.0134926	0.010493791	Atp2a2/Camk2d/Cdk9/Foxp1/Ppp3ca/Rock2/Smad4	7
BP	GO:0030004	cellular monovalent inorganic cation homeostasis	7/361	111/28814	5.03	0.000509155	0.0134926	0.010493791	Atp1a1/Atp1a4/Camk2d/Slc12a2/Slc25a14/Slc4a1/Slc8a1	7
BP	GO:0070918	primary sncRNA processing	5/361	53/28814	7.53	0.000526089	0.013841052	0.010764798	Ppp3ca/Prkra/Stat3/Tut7/Zmpste24	5
BP	GO:0022406	membrane docking	6/361	81/28814	5.91	0.000546424	0.014273367	0.011101028	Atp2a2/Camk2a/Nrxn1/Rims2/Stx1a/Stx2	6
BP	GO:0014896	muscle hypertrophy	7/361	113/28814	4.94	0.000566787	0.014700281	0.011433059	Atp2a2/Camk2d/Cdk9/Foxp1/Ppp3ca/Rock2/Smad4	7
BP	GO:0065002	intracellular protein transmembrane transport	5/361	54/28814	7.39	0.000573882	0.014717397	0.01144637	Pex1/Pex13/Sec61a1/Sec61a2/Timm23	5
BP	GO:0086010	membrane depolarization during action potential	4/361	31/28814	10.3	0.000583545	0.014717397	0.01144637	Ank3/Scn4b/Scn8a/Slmap	4
BP	GO:0099633	protein localization to postsynaptic specialization membrane	4/361	31/28814	10.3	0.000583545	0.014717397	0.01144637	Adam10/Camk2a/Dlg1/Nptx1	4
BP	GO:0099645	neurotransmitter receptor localization to postsynaptic specialization membrane	4/361	31/28814	10.3	0.000583545	0.014717397	0.01144637	Adam10/Camk2a/Dlg1/Nptx1	4
BP	GO:0007612	learning	9/361	187/28814	3.84	0.000619665	0.015521323	0.012071619	Apbb1/Bdnf/Cdk/Eif2ak4/Jph3/Map1a/Nrxn1/Slc24a2/Tpbg	9
BP	GO:1903076	regulation of protein localization to plasma membrane	7/361	115/28814	4.86	0.000629488	0.015660108	0.012179558	Camk2a/Camk2d/Dlg1/Nkx2-1/Numb/Prnp/Sorbs1	7
BP	GO:0035435	phosphate ion transmembrane transport	3/361	14/28814	17.1	0.000640687	0.015831039	0.012312499	Slc25a10/Slc25a14/Xpr1	3
BP	GO:0043434	response to peptide hormone	13/361	359/28814	2.89	0.000662109	0.016159939	0.012568299	Atp2a2/Atp2b1/Camk2a/Pck2/Pip4k2c/Pld1/Prkaa1/Pxn/Rock2/Slc26a6/Sorbs1/Stat3/Syap1	13
BP	GO:0019751	polyol metabolic process	7/361	116/28814	4.82	0.000662836	0.016159939	0.012568299	Inpp5b/Itpka/Nudt3/Nudt4/Pck2/Plcb1/Ppip5k2	7
BP	GO:1903539	protein localization to postsynaptic membrane	5/361	56/28814	7.13	0.000679156	0.016339955	0.012708305	Adam10/Camk2a/Cplx1/Dlg1/Nptx1	5
BP	GO:2000649	regulation of sodium ion transmembrane transporter activity	5/361	56/28814	7.13	0.000679156	0.016339955	0.012708305	Ank3/Camk2d/Plcb1/Scn4b/Slmap	5
BP	GO:0071331	cellular response to hexose stimulus	8/361	152/28814	4.2	0.000689531	0.01648115	0.012818119	Nrxn1/Pck2/Pdk3/Ppp3ca/Prkaa1/Rab11b/Slc26a6/Smad4	8

BP	GO:0034470	ncRNA processing	10/255	393/28814	2.88	0.002794894	0.071838077	0.062268322	Ctu2/Ints14/Ints8/Nop53/Prn4/Rpl35a/Rpl7/Tsn/Tsnax/Ythdf2	10
BP	GO:0023058	adaptation of signaling pathway	3/255	32/28814	10.59	0.002810645	0.071838077	0.062268322	Dnm1/Grk6/Ubqln2	3
BP	GO:0070536	protein K63-linked deubiquitination	3/255	32/28814	10.59	0.002810645	0.071838077	0.062268322	Nop53/Otub2/Yod1	3
BP	GO:0098810	neurotransmitter reuptake	3/255	32/28814	10.59	0.002810645	0.071838077	0.062268322	Slc18a2/Slc6a3/Tor1a	3
BP	GO:0035637	multicellular organismal signaling	6/255	157/28814	4.32	0.002815714	0.071838077	0.062268322	Abcc9/Cacng8/Fgf12/Kcnh2/Scn3b/Slc9a1	6
BP	GO:0035967	cellular response to topologically incorrect protein	5/255	109/28814	5.18	0.002888419	0.07241142	0.062765289	Dnajc10/Tmem33/Tor1a/Wfs1/Yod1	5
BP	GO:0140053	mitochondrial gene expression	5/255	109/28814	5.18	0.002888419	0.07241142	0.062765289	Gfm2/Mrpl51/Mrps18b/Rars2/Sars2	5
BP	GO:0006721	terpenoid metabolic process	4/255	67/28814	6.75	0.002967118	0.07360421	0.063799184	Aldh1a1/Rbp1/Rdh14/Ttr	4
BP	GO:0006914	autophagy	11/255	464/28814	2.68	0.00301259	0.07360421	0.063799184	Fyco1/Pafah1b2/Psen1/Rmoc1/Rock1/Scoc/Sh3bp4/Slc25a4/Slc25a5/Ubqln2/Yod1	11
BP	GO:0061919	process utilizing autophagic mechanism	11/255	464/28814	2.68	0.00301259	0.07360421	0.063799184	Fyco1/Pafah1b2/Psen1/Rmoc1/Rock1/Scoc/Sh3bp4/Slc25a4/Slc25a5/Ubqln2/Yod1	11
BP	GO:0030968	endoplasmic reticulum unfolded protein response	4/255	68/28814	6.65	0.003130976	0.075141579	0.065131755	Dnajc10/Tmem33/Wfs1/Yod1	4
BP	GO:0061337	cardiac conduction	4/255	68/28814	6.65	0.003130976	0.075141579	0.065131755	Abcc9/Kcnh2/Scn3b/Slc9a1	4
BP	GO:0070085	glycosylation	7/255	215/28814	3.68	0.003153705	0.075141579	0.065131755	Alg1/B4galnt1/Gmppb/Gxylt1/Mogs/Pofut2/Psen1	7
BP	GO:0120254	olefinic compound metabolic process	6/255	162/28814	4.19	0.003287022	0.076047947	0.065917383	Aldh1a1/Cyp46a1/Ephx1/Rbp1/Rdh14/Ttr	6
BP	GO:0006607	NLS-bearing protein import into nucleus	2/255	10/28814	22.6	0.003350014	0.076047947	0.065917383	Kpna4/Kpna6	2
BP	GO:0014908	myotube differentiation involved in skeletal muscle regeneration	2/255	10/28814	22.6	0.003350014	0.076047947	0.065917383	Cd81/Ptgfrn	2
BP	GO:0035871	protein K11-linked deubiquitination	2/255	10/28814	22.6	0.003350014	0.076047947	0.065917383	Otub2/Yod1	2
BP	GO:0051583	dopamine uptake involved in synaptic transmission	2/255	10/28814	22.6	0.003350014	0.076047947	0.065917383	Slc6a3/Tor1a	2
BP	GO:0051934	catecholamine uptake involved in synaptic transmission	2/255	10/28814	22.6	0.003350014	0.076047947	0.065917383	Slc6a3/Tor1a	2
BP	GO:0008380	RNA splicing	10/255	404/28814	2.8	0.003398553	0.076547106	0.066350048	Akt2/Ilf2/Prpf38b/Rbfox1/Rps26/Sf3b5/Snrpf/Tia1/Ubl5/Ybx1	10
BP	GO:0048488	synaptic vesicle endocytosis	4/255	70/28814	6.46	0.003476997	0.077109084	0.066837164	Ap2m1/Dnm1/Rock1/Tor1a	4
BP	GO:0140238	presynaptic endocytosis	4/255	70/28814	6.46	0.003476997	0.077109084	0.066837164	Ap2m1/Dnm1/Rock1/Tor1a	4
BP	GO:0034250	positive regulation of cellular amide metabolic process	6/255	166/28814	4.08	0.00370451	0.081527503	0.070666992	Cpeb3/Eif5a2/Guf1/Pym1/Ybx1/Ythdf2	6
BP	GO:0010506	regulation of autophagy	8/255	283/28814	3.19	0.003883373	0.083825876	0.072659192	Fyco1/Pafah1b2/Rock1/Scoc/Sh3bp4/Slc25a4/Slc25a5/Ubqln2	8
BP	GO:0000423	mitophagy	3/255	36/28814	9.42	0.003942186	0.083825876	0.072659192	Rmoc1/Slc25a4/Slc25a5	3
BP	GO:0009154	purine ribonucleotide catabolic process	3/255	36/28814	9.42	0.003942186	0.083825876	0.072659192	Entpd1/Nudt10/Pnp	3
BP	GO:0042168	heme metabolic process	3/255	36/28814	9.42	0.003942186	0.083825876	0.072659192	Cyb5r4/Hebp1/Hmox2	3
BP	GO:0016311	dephosphorylation	9/255	347/28814	2.93	0.003958553	0.083825876	0.072659192	Inpp5j/Iqgap1/Ppm1e/Ppp1cb/Ppp4c/Rock1/Sdhaf2/Ssu72/Tipr1	9
BP	GO:0006986	response to unfolded protein	5/255	118/28814	4.79	0.004057864	0.083825876	0.072659192	Dnajc10/Tmem33/Ubxn4/Wfs1/Yod1	5
BP	GO:0061635	regulation of protein complex stability	2/255	11/28814	20.54	0.004070629	0.083825876	0.072659192	Ecsit/Scoc	2
BP	GO:1901029	negative regulation of mitochondrial outer membrane permeabilization involved in apoptotic signaling pathway	2/255	11/28814	20.54	0.004070629	0.083825876	0.072659192	Slc25a4/Slc25a5	2
BP	GO:1903599	positive regulation of autophagy of mitochondrion	2/255	11/28814	20.54	0.004070629	0.083825876	0.072659192	Slc25a4/Slc25a5	2
BP	GO:0042063	gliogenesis	9/255	350/28814	2.91	0.00418555	0.085581146	0.074180638	Akt2/Bmerb1/C1qa/Gpr37l1/Nf1/Psen1/Rnf112/Slc8a3/Tnfrsf21	9
BP	GO:0009069	serine family amino acid metabolic process	3/255	37/28814	9.16	0.004262277	0.086536228	0.075008491	Cbs/Dhfr/Sephs1	3
BP	GO:0015711	organic anion transport	9/255	353/28814	2.88	0.004422599	0.088552512	0.07675618	Emb/Lrrc8d/Psen1/Slc25a15/Slc25a21/Slc25a4/Slc25a42/Slc25a5/Slc38a1	9
BP	GO:0010611	regulation of cardiac muscle hypertrophy	4/255	75/28814	6.03	0.004453734	0.088552512	0.07675618	Glrx3/Rock1/Slc25a4/Slc9a1	4
BP	GO:1901292	nucleoside phosphate catabolic process	4/255	75/28814	6.03	0.004453734	0.088552512	0.07675618	Entpd1/Entpd3/Nudt10/Pnp	4
BP	GO:1905168	positive regulation of double-strand break repair via homologous recombination	3/255	38/28814	8.92	0.004597696	0.090788743	0.078694516	Mms19/Morf4l2/Was	3
BP	GO:0006941	striated muscle contraction	6/255	174/28814	3.9	0.004655916	0.091030395	0.078903977	Abcc9/Atp1b1/Kcnh2/Scn3b/Slc8a3/Slc9a1	6
BP	GO:0034754	cellular hormone metabolic process	5/255	122/28814	4.63	0.004673083	0.091030395	0.078903977	Aldh1a1/Cyp46a1/Rbp1/Rdh14/Ttr	5
BP	GO:0032000	positive regulation of fatty acid beta-oxidation	2/255	12/28814	18.83	0.004856343	0.093965341	0.081447951	Akt2/Cpt1a	2
BP	GO:0050890	cognition	9/255	359/28814	2.83	0.00492804	0.094197487	0.081649172	Cpeb3/Dop1b/Mme/Nptn/Psen1/Shroom4/Slc8a2/Slc8a3/Th	9
BP	GO:0036474	cell death in response to hydrogen peroxide	3/255	39/28814	8.69	0.004948648	0.094197487	0.081649172	Gpr37l1/Pde8a/Rnf112	3
BP	GO:0022411	cellular component disassembly	10/255	427/28814	2.65	0.004994952	0.094197487	0.081649172	Bmerb1/C1qa/Eif5a2/Fyco1/[illegible]/Lima1/Pym1/Rmoc1/Slc25a4/Slc25a5	10
BP	GO:1901617	organic hydroxy compound biosynthetic process	7/255	234/28814	3.38	0.004999034	0.094197487	0.081649172	Amacr/Ddc/Dhfr/Ephx1/Scap/Slc6a3/Th	7

BP	GO:0048172	regulation of short-term neuronal synaptic plasticity	5/607	21/28814	11.3	6.27809E-05	0.001947432	0.001410104	Rab3a/Shisa7/Shisa9/Syap1/Syngr1	5
BP	GO:0007416	synapse assembly	14/607	193/28814	3.44	6.52287E-05	0.002010306	0.001455629	Ache/Cdh2/Dbnl/Eef2k/Efnb1/Farp1/Fgf13/Gabrb2/Gap43/Mef2c/Nlgn3/Nptx1/Nrxn3/Ntrk3	14
BP	GO:0035308	negative regulation of protein dephosphorylation	6/607	34/28814	8.38	6.93946E-05	0.002103022	0.001522764	Arpp19/Bod1/Ensa/Ppp1r16b/Ppp4r4/Ptpa	6
BP	GO:0010469	regulation of signaling receptor activity	12/607	147/28814	3.88	6.95578E-05	0.002103022	0.001522764	Aplp2/Dlgap4/Grem2/Mef2c/Nlgn3/Nptx1/Prrt1/Rgs9/Shisa7/Shisa9/Slc6a9/Tgfa	12
BP	GO:0043467	regulation of generation of precursor metabolites and energy	12/607	147/28814	3.88	6.95578E-05	0.002103022	0.001522764	Cox7a1/Cox7a2/Eif6/Enpp1/Grb10/Hmgb1/P2rx7/Slc25a33/Sorbs1/Stat3/Taco1/Zbtb20	12
BP	GO:0006816	calcium ion transport	23/607	440/28814	2.48	7.06114E-05	0.002114622	0.001531163	Asic1/Atp2b1/Atp2b4/Bax/Camk2b/Drd1/Ednrb/Epb41/Gjc2/Gnb5/Hap1/Homer2/Hspa2/Jak3/Mcu/P2rx7/Plcg2/Prkcb/Ptpn6/Rgs9/Ryr3/	23
BP	GO:0032409	regulation of transporter activity	18/607	298/28814	2.87	7.08268E-05	0.002114622	0.001531163	Actb/Actn2/Dnm2/Fgf13/Gnb5/Hap1/Hspa2/Kcnc1/Mef2c/Nlgn3/Plcg2/Prrt1/Rgs9/Scn1b/Shisa9/Slc6a9/Stim1/	18
BP	GO:0034329	cell junction assembly	23/607	441/28814	2.48	7.3059E-05	0.002167719	0.001569609	Ache/Actb/Actn2/Arhgap6/Cdh2/Dbnl/Eef2k/Efnb1/Farp1/Fgf13/Gabrb2/Gap43/Gjb6/Mef2c/Nlgn3/Nptx1/Nrxn3/Ntrk3/Pip5k1a/Rac1/Rcc	23
BP	GO:0048259	regulation of receptor-mediated endocytosis	11/607	126/28814	4.14	7.56148E-05	0.002229703	0.001614491	Anxa2/B2m/Clu/Dnm2/Hap1/Pacsin1/Picalm/Plcg2/Ptpn5/Sh3gl3/Unc119	11
BP	GO:0070588	calcium ion transmembrane transport	18/607	300/28814	2.85	7.71322E-05	0.002260493	0.001636786	Asic1/Atp2b1/Atp2b4/Bax/Drd1/Ednrb/Gjc2/Gnb5/Hap1/Hspa2/Mcu/P2rx7/Plcg2/Ptpn6/Rgs9/Ryr3/Stim1/T	18
BP	GO:0060249	anatomical structure homeostasis	19/607	328/28814	2.75	7.85902E-05	0.002289178	0.001657556	Aldh1a1/Bag3/Bap1/Bax/Bbs2/Cracd/Enpp1/Homer2/Ildr2/Lamp2/Lipa/P2rx7/Pla2g4a/Prom1/Ptgs1/Rac1/Rac	19
BP	GO:0001975	response to amphetamine	5/607	22/28814	10.79	7.98422E-05	0.002311554	0.001673758	Asic1/Drd1/Gnal/Pde1b/Slc18a2	5
BP	GO:0032412	regulation of ion transmembrane transporter activity	17/607	274/28814	2.95	8.05456E-05	0.002317871	0.001678332	Actn2/Dnm2/Fgf13/Gnb5/Hap1/Hspa2/Kcnc1/Mef2c/Nlgn3/Plcg2/Prrt1/Rgs9/Scn1b/Shisa9/Slc6a9/Stim1/Taco1	17
BP	GO:0008286	insulin receptor signaling pathway	11/607	127/28814	4.11	8.12377E-05	0.002323789	0.001682617	Bcar3/Enpp1/Foxo1/Grb10/ [illegible]	11
BP	GO:0032496	response to lipopolysaccharide	21/607	386/28814	2.58	8.25369E-05	0.002346897	0.00169935	[illegible]	21
BP	GO:0061684	chaperone-mediated autophagy	4/607	12/28814	15.82	8.43741E-05	0.002384942	0.001726898	Bag3/Clu/Gfap/Lamp2	4
BP	GO:0045333	cellular respiration	15/607	223/28814	3.19	8.53935E-05	0.002398744	0.001736891	[illegible]	15
BP	GO:0006109	regulation of carbohydrate metabolic process	14/607	196/28814	3.36	8.58667E-05	0.002398744	0.001736891	[illegible]	14
BP	GO:1902903	regulation of supramolecular fiber organization	21/607	388/28814	2.57	8.87488E-05	0.002464844	0.001784753	[illegible]	21
BP	GO:1901361	organic cyclic compound catabolic process	24/607	477/28814	2.39	8.93634E-05	0.002467567	0.001786725	[illegible]	24
BP	GO:0006821	chloride transport	10/607	107/28814	4.44	8.99989E-05	0.002470557	0.00178889	[illegible]	10
BP	GO:0061351	neural precursor cell proliferation	14/607	199/28814	3.34	9.06149E-05	0.002473528	0.001791041	[illegible]	14
BP	GO:0048588	developmental cell growth	17/607	277/28814	2.91	9.19884E-05	0.002496754	0.001807859	[illegible]	17
BP	GO:0042053	regulation of dopamine metabolic process	5/607	23/28814	10.32	0.000100259	0.002601815	0.001949099	[illegible]	5
BP	GO:0009132	nucleoside diphosphate metabolic process	11/607	130/28814	4.02	0.000100302	0.002601815	0.001949099	[illegible]	11
BP	GO:0006605	protein targeting	17/607	280/28814	2.88	0.000104836	0.002797777	0.002025825	[illegible]	17
BP	GO:0018209	peptidyl-serine modification	20/607	364/28814	2.61	0.000105892	0.002810261	0.002034864	[illegible]	20
BP	GO:0046434	organophosphate catabolic process	12/607	154/28814	3.7	0.00010876	0.002870417	0.002078422	[illegible]	12
BP	GO:0046677	response to antibiotic	6/607	37/28814	7.7	0.0001137	0.00297294	0.002152657	[illegible]	6
BP	GO:0006417	regulation of translation	21/607	395/28814	2.52	0.000113889	0.00297294	0.002152657	[illegible]	21
BP	GO:0014745	negative regulation of muscle adaptation	4/607	13/28814	14.61	0.000119844	0.003111393	0.002252909	[illegible]	4
BP	GO:0018105	peptidyl-serine phosphorylation	19/607	339/28814	2.66	0.000120965	0.00312351	0.002261682	[illegible]	19
BP	GO:0051098	regulation of binding	21/607	397/28814	2.51	0.000122145	0.003137022	0.002271466	[illegible]	21
BP	GO:0031669	cellular response to nutrient levels	14/607	205/28814	3.24	0.000124187	0.003145671	0.002277729	[illegible]	14
BP	GO:0014075	response to amine	5/607	24/28814	9.89	0.000124457	0.003145671	0.002277729	[illegible]	5
BP	GO:0031629	synaptic vesicle fusion to presynaptic active zone membrane	5/607	24/28814	9.89	0.000124457	0.003145671	0.002277729	[illegible]	5
BP	GO:0008360	regulation of cell shape	12/607	157/28814	3.63	0.000130636	0.003269358	0.002367289	[illegible]	12
BP	GO:0071805	potassium ion transmembrane transport	14/607	206/28814	3.23	0.00013072	0.003269358	0.002367289	[illegible]	14
BP	GO:0021987	cerebral cortex development	10/607	112/28814	4.24	0.000131982	0.003282821	0.002377037	[illegible]	10
BP	GO:0060292	long-term synaptic depression	6/607	38/28814	7.5	0.000132632	0.003282821	0.002377037	[illegible]	6
BP	GO:0001894	tissue homeostasis	17/607	286/28814	2.82	0.000135334	0.003331136	0.002412021	[illegible]	17
BP	GO:0010001	glial cell differentiation	16/607	259/28814	2.93	0.000135979	0.003331136	0.002412021	[illegible]	16
BP	GO:0031589	cell-substrate adhesion	20/607	373/28814	2.55	0.000146851	0.003579113	0.002591577	[illegible]	20
BP	GO:2001222	regulation of neuron migration	7/607	55/28814	6.04	0.000150244	0.00364134	0.002636635	[illegible]	7
BP	GO:0042069	regulation of catecholamine metabolic process	5/607	25/28814	9.49	0.00015288	0.00364134	0.002636635	[illegible]	5
BP	GO:0099500	vesicle fusion to plasma membrane	5/607	25/28814	9.49	0.00015288	0.00364134	0.002636635	[illegible]	5
BP	GO:0033108	mitochondrial respiratory chain complex assembly	9/607	93/28814	4.59	0.000153846	0.00364134	0.002636635	[illegible]	9
BP	GO:0010563	negative regulation of phosphorus metabolic process	15/361	457/28814	2.62	0.000724439	0.017092087	0.013293272	Aida/Arpp19/Coro1c/Mvp/Pcp4/Pip4k2c/Ppp1r9b/Ppp4c/Rabgef1/Rock2/Sh3gl2/Sirpa/Slc4a1/Slc8a1/Stat3	15
BP	GO:0045936	negative regulation of phosphate metabolic process	15/361	457/28814	2.62	0.000724439	0.017092087	0.013293272	Aida/Arpp19/Coro1c/Mvp/Pcp4/Pip4k2c/Ppp1r9b/Ppp4c/Rabgef1/Rock2/Sh3gl2/Sirpa/Slc4a1/Slc8a1/Stat3	15
BP	GO:0062237	protein localization to postsynapse	5/361	57/28814	7	0.000736904	0.017222344	0.013394578	Adam10/Camk2a/Cplx1/Dlg1/Nptx1	5
BP	GO:0007015	actin filament organization	15/361	458/28814	2.61	0.000740641	0.017222344	0.013394578	Add2/Arpc5l/Cd47/Coro1c/Dlg1/Elmo2/Ppp1r9b/Pxn/Rock2/Sirpa/Sorbs1/Sorbs3/Tjp1/Tmod1/Vasp	15
BP	GO:0009156	ribonucleoside monophosphate biosynthetic process	4/361	33/28814	9.67	0.000744088	0.017222344	0.013394578	Ampd3/Ppat/Prps1/Uprt	4
BP	GO:0007416	synapse assembly	9/361	193/28814	3.72	0.00077623	0.017801413	0.013844946	Add2/Bdnf/Kirrel3/Mdga2/Nptx1/Nrxn1/Numb/Sema4a/Tpbg	9
BP	GO:0032026	response to magnesium ion	3/361	15/28814	15.96	0.000793446	0.017801413	0.013844946	Ank3/Kcna1/Kcnip3	3
BP	GO:0036376	sodium ion export across plasma membrane	3/361	15/28814	15.96	0.000793446	0.017801413	0.013844946	Atp1a1/Atp1a4/Slc8a1	3
BP	GO:0038166	angiotensin-activated signaling pathway	3/361	15/28814	15.96	0.000793446	0.017801413	0.013844946	Camk2a/Rock2/Slc26a6	3
BP	GO:0098917	retrograde trans-synaptic signaling	3/361	15/28814	15.96	0.000793446	0.017801413	0.013844946	Abhd6/Bdnf/Plcb1	3
BP	GO:0006720	isoprenoid metabolic process	6/361	87/28814	5.5	0.000798586	0.017807489	0.013849671	Aldh1a2/Crppa/Dhrs13/Mvd/Mvk/Pecr	6
BP	GO:0032970	regulation of actin filament-based process	14/361	414/28814	2.7	0.000811471	0.017985148	0.013987844	Abracl/Add2/Arpc5l/Atp1a1/Atp2a2/Cd47/Dlg1/Pxn/Rock2/Smad4/Sorbs3/Tjp1/Tmod1/Vasp	14
BP	GO:0007611	learning or memory	12/361	322/28814	2.97	0.000824574	0.018069918	0.014053774	Apbb1/Bdnf/Cck/Eif2ak4/Jph3/Map1a/Nrxn1/Pja2/Plcb1/Slc17a7/Slc24a2/Tpbg	12
BP	GO:0055075	potassium ion homeostasis	4/361	34/28814	9.39	0.000835038	0.018069918	0.014053774	Atp1a1/Atp1a4/Camk2d/Slc12a2	4
BP	GO:0060487	lung epithelial cell differentiation	4/361	34/28814	9.39	0.000835038	0.018069918	0.014053774	Foxp1/Foxp4/Numb/Ppp3r1	4
BP	GO:0009746	response to hexose	9/361	195/28814	3.68	0.00083506	0.018069918	0.014053774	Glul/Nrxn1/Pck2/Pdk3/Ppp3ca/Prkaa1/Rab11b/Slc26a6/Smad4	9
BP	GO:0060047	heart contraction	10/361	238/28814	3.35	0.000900043	0.019361517	0.015058307	Atp1a1/Atp2a2/Calm1/Camk2d/Dlg1/Myl1/S100a1/Scn4b/Slc8a1/Zmpste24	10
BP	GO:0032984	protein-containing complex disassembly	10/361	239/28814	3.34	0.000929136	0.019397706	0.015086453	Add2/Atp2a2/Ccsap/Epg5/Map1a/Napa/Sh3gl1/Sh3gl2/Tmod1/Zmpste24	10
BP	GO:1901652	response to peptide	14/361	420/28814	2.66	0.000931361	0.019397706	0.015086453	Atp2a2/Atp2b1/Camk2a/Pck2/Pip4k2c/Plcb1/Prkaa1/Pxn/Rock2/Slc26a6/Snx6/Sorbs1/Stat3/Syap1	14
BP	GO:0048278	vesicle docking	5/361	60/28814	6.65	0.000932242	0.019397706	0.015086453	Camk2a/Nrxn1/Rims2/Stx1a/Stx2	5
BP	GO:0006084	acetyl-CoA metabolic process	4/361	35/28814	9.12	0.000933551	0.019397706	0.015086453	Mvd/Mvk/Pdk1/Pdk3	4
BP	GO:0008299	isoprenoid biosynthetic process	4/361	35/28814	9.12	0.000933551	0.019397706	0.015086453	Aldh1a2/Crppa/Mvd/Mvk	4
BP	GO:0060479	lung cell differentiation	4/361	35/28814	9.12	0.000933551	0.019397706	0.015086453	Foxp1/Foxp4/Numb/Ppp3r1	4
BP	GO:0021681	cerebellar granular layer development	3/361	16/28814	14.97	0.000967514	0.01998982	0.015546966	Atp2b2/Cend1/Nrxn1	3
BP	GO:0033866	nucleoside bisphosphate biosynthetic process	4/361	36/28814	8.87	0.001039964	0.021128594	0.016432642	Coasy/Gcdh/Pdk1/Pdk3	4
BP	GO:0034030	ribonucleoside bisphosphate biosynthetic process	4/361	36/28814	8.87	0.001039964	0.021128594	0.016432642	Coasy/Gcdh/Pdk1/Pdk3	4
BP	GO:0034033	purine nucleoside bisphosphate biosynthetic process	4/361	36/28814	8.87	0.001039964	0.021128594	0.016432642	Coasy/Gcdh/Pdk1/Pdk3	4
BP	GO:0051924	regulation of calcium ion transport	11/361	287/28814	3.06	0.001073332	0.021686045	0.016866196	Atp2b1/Atp2b2/Cacnb3/Calm1/Camk2a/Camk2d/Cbarp/Homer2/Jph3/S100a1/Slc8a1	11
BP	GO:0050982	detection of mechanical stimulus	5/361	62/28814	6.44	0.001082281	0.021688886	0.016868405	Atp2b2/Cacnb3/Kcna1/Phf24/Slc12a2	5
BP	GO:0009260	ribonucleotide biosynthetic process	8/361	163/28814	3.92	0.001085334	0.021688886	0.016868405	Ampd3/Coasy/Gcdh/Pdk1/Pdk3/Ppat/Stat3/Uprt	8
BP	GO:0016126	sterol biosynthetic process	5/361	63/28814	6.33	0.001163674	0.02264181	0.017609536	Cyb5r1/Dhcr7/Mvd/Mvk/Prkaa1	5
BP	GO:0006744	ubiquinone biosynthetic process	3/361	17/28814	14.09	0.001163976	0.02264181	0.017609536	Coq6/Coq7/Ndufa9	3
BP	GO:0008272	sulfate transport	3/361	17/28814	14.09	0.001163976	0.02264181	0.017609536	Slc25a10/Slc25a14/Slc26a6	3
BP	GO:0086012	membrane depolarization during cardiac muscle cell action potential	3/361	17/28814	14.09	0.001163976	0.02264181	0.017609536	Ank3/Scn4b/Slmap	3
BP	GO:1904889	regulation of excitatory synapse assembly	3/361	17/28814	14.09	0.001163976	0.02264181	0.017609536	Nptx1/Nrxn1/Sema4a	3
BP	GO:0051899	membrane depolarization	6/361	94/28814	5.09	0.001196974	0.023160506	0.018012949	Ank3/Camk2d/Cck/Scn4b/Scn8a/Slmap	6
BP	GO:0043500	muscle adaptation	7/361	129/28814	4.33	0.001237826	0.023824894	0.018529673	Atp2a2/Camk2d/Cdk9/Foxp1/Ppp3ca/Rock2/Smad4	7
BP	GO:0099173	postsynapse organization	9/361	207/28814	3.47	0.001268906	0.02429524	0.018895482	Adam10/Arhgap39/Itpka/Ndrg1/Nptx1/Nrxn1/Numb/Rock2/Sorbs1	9
BP	GO:0030866	cortical actin cytoskeleton organization	4/361	38/28814	8.4	0.001277824	0.024338562	0.018929175	Dlg1/Ehd2/Epb41l2/Rock2	4
BP	GO:0003015	heart process	10/361	250/28814	3.19	0.001303227	0.024693795	0.019205456	Atp1a1/Atp2a2/Calm1/Camk2d/Dlg1/Myl1/S100a1/Scn4b/Slc8a1/Zmpste24	10
BP	GO:0006164	purine nucleotide biosynthetic process	8/361	168/28814	3.8	0.001316827	0.02482287	0.019305843	Ampd3/Coasy/Gcdh/Pdk1/Pdk3/Ppat/Prps1/Stat3	8
BP	GO:0051235	maintenance of location	12/361	342/28814	2.8	0.001379241	0.025178083	0.019582108	Ank3/Arl2bp/B4galnt1/Calm1/Camk2d/Jph3/Mest/Plin3/Skp1/Slc17a7/Slc30a3/Slc8a1	12
BP	GO:0006625	protein targeting to peroxisome	3/361	18/28814	13.3	0.001383865	0.025178083	0.019582108	Pex1/Pex13/Pex19	3
BP	GO:0035633	maintenance of blood-brain barrier	3/361	18/28814	13.3	0.001383865	0.025178083	0.019582108	Sh3gl2/Slc12a2/Tjp1	3
BP	GO:0072662	protein localization to peroxisome	3/361	18/28814	13.3	0.001383865	0.025178083	0.019582108	Pex1/Pex13/Pex19	3
BP	GO:0072663	establishment of protein localization to peroxisome	3/361	18/28814	13.3	0.001383865	0.025178083	0.019582108	Pex1/Pex13/Pex19	3
BP	GO:0099150	regulation of postsynaptic specialization assembly	3/361	18/28814	13.3	0.001383865	0.025178083	0.019582108	Nptx1/Nrxn1/Numb	3
BP	GO:0044270	cellular nitrogen compound catabolic process	10/255	428/28814	2.64	0.005075784	0.095022626	0.082364391	Cpeb3/Entpd1/Entpd3/Hmox2/Nudt10/Pnp/Pym1/Rock1/Ybx1/Ythdf2	10
BP	GO:0014743	regulation of muscle hypertrophy	4/255	78/28814	5.79	0.005120454	0.095240448	0.082553197	Glrx3/Rock1/Slc25a4/Slc9a1	4
BP	GO:0046700	heterocycle catabolic process	10/255	429/28814	2.63	0.005157639	0.095317129	0.082619663	Cpeb3/Entpd1/Entpd3/Hmox2/Nudt10/Pnp/Pym1/Rock1/Ybx1/Ythdf2	10
BP	GO:0003012	muscle system process	10/255	430/28814	2.63	0.005240526	0.096232077	0.083412728	Abcc9/Atp1b1/Chrm2/Glrx3/Kcnh2/Rock1/Scn3b/Slc25a4/Slc8a3/Slc9a1	10
BP	GO:0006195	purine nucleotide catabolic process	3/255	40/28814	8.47	0.005315331	0.096987977	0.084067933	Entpd1/Nudt10/Pnp	3
BP	GO:0009308	amine metabolic process	5/255	126/28814	4.48	0.00535187	0.097040518	0.084113475	Ddc/Inmt/Pnkd/Slc6a3/Th	5
BP	GO:0001504	neurotransmitter uptake	3/255	41/28814	8.27	0.005697933	0.099876749	0.086571883	Slc18a2/Slc6a3/Tor1a	3
BP	GO:0010613	positive regulation of cardiac muscle hypertrophy	3/255	41/28814	8.27	0.005697933	0.099876749	0.086571883	Rock1/Slc25a4/Slc9a1	3
BP	GO:0035794	positive regulation of mitochondrial membrane permeability	3/255	41/28814	8.27	0.005697933	0.099876749	0.086571883	Slc25a4/Slc25a5/Slc9a1	3
BP	GO:1901381	positive regulation of potassium ion transmembrane transport	3/255	41/28814	8.27	0.005697933	0.099876749	0.086571883	Atp1b1/Kcnc1/Kcnh2	3
BP	GO:1901524	regulation of mitophagy	2/255	13/28814	17.38	0.005705956	0.099876749	0.086571883	Slc25a4/Slc25a5	2
BP	GO:0031109	microtubule polymerization or depolymerization	5/255	128/28814	4.41	0.005716151	0.099876749	0.086571883	Bmerb1/Drg1/Inpp5j/Tubg1/Tubgcp2	5
CC	GO:0098793	presynapse	22/263	493/28739	4.88	1.24237E-09	4.77071E-07	3.6748E-07	Cadm3/Cadps2/Chrm2/Cyp46a1/Ddc/Dnm1/Entpd1/Kcnc3/Mme/Nectin1/Nptn/Phaf1/Psen1/Rnf112/Slc18a2/Slc6a3/Slc8a2/Slc8a3/Stx2/Th/Tor1a/Ywhah	22
CC	GO:0005743	mitochondrial inner membrane	19/263	460/28739	4.51	5.88404E-08	1.12974E-05	8.70219E-06	Coq8a/Cox20/Cox7c/Cpt1a/Dhfr/Gcat/Guf1/Ndufa11/Pmpcb/Psen1/Rab5if/Slc25a15/Slc25a21/Slc25a3/Slc25a4/Slc25a42/Slc25a5/Timm8a1/Tmem177	19
CC	GO:0008021	synaptic vesicle	11/263	205/28739	5.86	3.28392E-06	0.000420341	0.000323783	Ddc/Dnm1/Entpd1/Mme/Phaf1/Psen1/Rnf112/Slc18a2/Stx2/Th/Tor1a	11
CC	GO:0070382	exocytic vesicle	11/263	228/28739	5.27	9.04532E-06	0.00086835	0.000668877	Ddc/Dnm1/Entpd1/Mme/Phaf1/Psen1/Rnf112/Slc18a2/Stx2/Th/Tor1a	11
CC	GO:0097060	synaptic membrane	14/263	390/28739	3.92	1.64803E-05	0.001265686	0.000974939	Cacng8/Cadm3/Cadps2/Chrm2/Dnm1/Entpd1/Epha7/Grip1/Kcnc3/Nectin1/Nptn/Psen1/Slc6a3/Stx2	14
CC	GO:0031252	cell leading edge	14/263	412/28739	3.71	3.01241E-05	0.001927942	0.001485065	Akt2/Cacng8/Inpp5j/Iqgap1/Iqgap2/Kcnc1/Kcnc3/Lima1/Mkln1/Pip5k1a/Rock1/S100a11/Stx2/Tubg1	14
CC	GO:0030133	transport vesicle	12/263	316/28739	4.15	3.86145E-05	0.00211828	0.00163168	Ddc/Dnm1/Entpd1/Mme/Phaf1/Psen1/Rnf112/Slc18a2/Stx2/Tgoln1/Th/Tor1a	12
CC	GO:0005635	nuclear envelope	14/263	434/28739	3.52	5.28414E-05	0.002393039	0.001843322	Agpat5/Cacybp/Eif5a2/Jpt1/Kcnh2/Kpna4/Lypla1/Mrps23/Psen1/Ranbp1/Sephs1/Tmem33/Tor1a/Ybx1	14
CC	GO:0005840	ribosome	10/263	236/28739	4.63	6.92693E-05	0.002393039	0.001843322	Mrpl51/Mrps18b/Mrps23/Nufip2/Rpl31/Rpl35a/Rpl7/Rpl8/Rps15a/Rps26	10
CC	GO:0010494	cytoplasmic stress granule	6/263	76/28739	8.63	7.09597E-05	0.002393039	0.001843322	Nufip2/Rbfox1/Rock1/Tia1/Ybx1/Ythdf2	6
CC	GO:1990351	transporter complex	13/263	393/28739	3.61	7.58944E-05	0.002393039	0.001843322	Abcc9/Atp1b1/Cacng8/Kcnc1/Kcnc3/Kcnh2/Lrrc8b/Lrrc8d/Ndufa11/Scn3b/Slc9a1/Timm8a1/Ttyh2	13
CC	GO:0005844	polysome	6/263	77/28739	8.51	7.63721E-05	0.002393039	0.001843322	Drg1/Nufip2/Rpl31/Rpl7/Rpl8/Rps26	6
CC	GO:0044391	ribosomal subunit	9/263	195/28739	5.04	8.3379E-05	0.002393039	0.001843322	Mrpl51/Mrps18b/Mrps23/Rpl31/Rpl35a/Rpl7/Rpl8/Rps15a/Rps26	9
CC	GO:0001726	ruffle	8/263	153/28739	5.71	8.72462E-05	0.002393039	0.001843322	Akt2/Inpp5j/Iqgap1/Lima1/Mkln1/Pip5k1a/Rock1/S100a11	8
CC	GO:0044306	neuron projection terminus	9/263	212/28739	4.64	0.000157073	0.004021075	0.003097374	Chrm2/Kcnc3/Mme/Scrg1/Slc18a2/Slc6a3/Slc8a2/Slc8a3/Th	9
CC	GO:1902495	transmembrane transporter complex	12/263	372/28739	3.52	0.000180217	0.004325219	0.003331652	Abcc9/Atp1b1/Cacng8/Kcnc1/Kcnc3/Kcnh2/Lrrc8b/Lrrc8d/Ndufa11/Scn3b/Slc9a1/Ttyh2	12
CC	GO:0150034	distal axon	12/263	375/28739	3.5	0.000194018	0.004382523	0.003375792	Chrm2/Inpp5j/Iqgap1/Kcnc3/Nectin1/Psen1/Slc18a2/Slc6a3/Slc8a2/Slc8a3/Th/Tor1a	12
CC	GO:0042734	presynaptic membrane	7/263	132/28739	5.79	0.000221246	0.004719925	0.003635688	Cadm3/Cadps2/Dnm1/Nectin1/Nptn/Slc6a3/Stx2	7
CC	GO:0042788	polysomal ribosome	4/263	35/28739	12.49	0.000287113	0.005561337	0.004283815	Nufip2/Rpl31/Rpl8/Rps26	4
CC	GO:0045121	membrane raft	12/263	393/28739	3.34	0.000297244	0.005561337	0.004283815	Atp1b1/Gprc5b/Grip1/Iqgap1/Mme/Psen1/Rtn4rl1/Slc25a4/Slc25a5/Slc6a3/Slc9a1/Stx2	12
CC	GO:0098857	membrane microdomain	12/263	394/28739	3.33	0.000304136	0.005561337	0.004283815	Atp1b1/Gprc5b/Grip1/Iqgap1/Mme/Psen1/Rtn4rl1/Slc25a4/Slc25a5/Slc6a3/Slc9a1/Stx2	12
CC	GO:0098984	neuron to neuron synapse	11/263	361/28739	3.33	0.000536698	0.009367823	0.007215894	Cacng8/Chrm2/Cnn3/Entpd1/Nectin1/Nptn/Rnf112/Rpl7/Rpl8/Slc8a2/Slc8a3	11
CC	GO:0022626	cytosolic ribosome	6/263	111/28739	5.91	0.000563268	0.00940412	0.007243854	Rpl31/Rpl35a/Rpl7/Rpl8/Rps15a/Rps26	6
CC	GO:0019898	extrinsic component of membrane	10/263	318/28739	3.44	0.000749919	0.0119987	0.00924242	Cdh12/Coq8a/Gnb4/Gng11/Inpp5j/Iqgap1/Mfge8/Rnf112/Snx10/Tor1a	10
CC	GO:0042383	sarcolemma	7/263	165/28739	4.64	0.000845338	0.012984399	0.010001689	Abcc9/Atp1b1/Cacng8/Psen1/Slc8a2/Slc8a3/Slc9a1	7
CC	GO:0032279	asymmetric synapse	10/263	339/28739	3.22	0.001214822	0.017941982	0.013820441	Cacng8/Chrm2/Cnn3/Entpd1/Nptn/Rnf112/Rpl7/Rpl8/Slc8a2/Slc8a3	10
CC	GO:0098798	mitochondrial protein-containing complex	9/263	286/28739	3.44	0.001358735	0.019324234	0.01488517	Cox7c/Mrpl51/Mrps18b/Mrps23/Ndufa11/Pmpcb/Slc25a4/Slc25a5/Timm8a1	9
CC	GO:0034702	ion channel complex	9/263	294/28739	3.35	0.001641539	0.02251254	0.017341074	Abcc9/Cacng8/Kcnc1/Kcnc3/Kcnh2/Lrrc8b/Lrrc8d/Scn3b/Ttyh2	9
CC	GO:0005689	U12-type spliceosomal complex	3/263	27/28739	12.14	0.001883812	0.02386129	0.018379996	Sf3b5/Snrpf/Ybx1	3
CC	GO:0043679	axon terminus	7/263	190/28739	4.03	0.001905495	0.02386129	0.018379996	Chrm2/Kcnc3/Slc18a2/Slc6a3/Slc8a2/Slc8a3/Th	7
CC	GO:0031965	nuclear membrane	8/263	244/28739	3.58	0.001926302	0.02386129	0.018379996	Jpt1/Kpna4/Lypla1/Mrps23/Psen1/Sephs1/Tor1a/Ybx1	8
CC	GO:0022625	cytosolic large ribosomal subunit	4/263	63/28739	6.94	0.002674085	0.032089018	0.024717692	Rpl31/Rpl35a/Rpl7/Rpl8	4
CC	GO:0005669	transcription factor TFIID complex	3/263	31/28739	10.57	0.002818026	0.032791577	0.025258863	Gtf2a2/Gtf2b/Gtf2f1	3
CC	GO:0014069	postsynaptic density	9/263	330/28739	2.98	0.003551079	0.040106303	0.030893287	Cacng8/Cnn3/Entpd1/Nptn/Rnf112/Rpl7/Rpl8/Slc8a2/Slc8a3	9
CC	GO:0030315	T-tubule	4/263	69/28739	6.33	0.003718931	0.040801981	0.031429157	Abcc9/Atp1b1/Cacng8/Slc9a1	4

BP	GO:0140029	exocytic process	9/607	93/28814	4.59	0.000153846	0.00364134	0.002636635	Bloc1s6/Cplx1/Grik5/Kcnb1/Rab3a/Rab3c/Rab8b/Snap25/Stx3	9
BP	GO:0033238	regulation of cellular amine metabolic process	6/607	39/28814	7.3	0.000153978	0.00364134	0.002636635	Atp2b4/Comt/Drd1/Pde1b/Pnkd/Slc6a3	6
BP	GO:0033674	positive regulation of kinase activity	22/607	435/28814	2.4	0.000161257	0.003794697	0.002747678	Adcy8/Atp2b4/Camk1/Camkk2/Cdk5r2/Clu/Dab2ip/Dipk2a/Gab1/Irgm1/Map2k2/Map3k13/Marcks/Ntrk3/Osbpl8/P2rx7/Rac1/Syap1/Tgfa/Traf6/Unc119/Vangl2	22
BP	GO:0007613	memory	12/607	161/28814	3.54	0.00016559	0.003877575	0.002807689	Adcy8/Asic1/Comt/Crebbp/Csmd1/Drd1/Fgf13/Ptgs1/Rcan1/Shisa7/Snap25/Th	12
BP	GO:0031668	cellular response to extracellular stimulus	15/607	237/28814	3	0.000167526	0.003903772	0.002826657	Brsk1/Comt/Dap/Dcps/Elapor1/Foxo1/Kcnb1/Kptn/Lamp2/Mapk3/P2rx7/Pdk2/Prkag1/Rragb/Wipi1	15
BP	GO:0006643	membrane lipid metabolic process	13/607	186/28814	3.32	0.000170737	0.003959273	0.002866844	Abca8b/B4galt6/Bax/Hacd3/Hexb/Kdsr/P2rx7/Pigt/Scepdh/Sgpl1/Sgpp1/Smpd3/Smpd4	13
BP	GO:0002237	response to molecule of bacterial origin	21/607	407/28814	2.45	0.000171894	0.00396685	0.002872331	Abca1/B2m/Chmp5/Dab2ip/Ednrb/Gbp2/Hmgb1/Hmgb2/Hnrnpa0/Irgm1/Mapk3/Mef2c/Noct/P2rx7/Pdcd4/Plcg2/Sirpa/Stat1/Traf6/Tut4/Vi	21
BP	GO:0006119	oxidative phosphorylation	10/607	116/28814	4.09	0.000176513	0.004053864	0.002935336	Atp5pb/Cox7a1/Cox7a2/Cox7c/Msh2/Ndufa10/Ndufa12/Ndufb6/Sdhaf2/Slc25a33	10
BP	GO:0031032	actomyosin structure organization	14/607	213/28814	3.12	0.000185365	0.004208181	0.003047075	Actn2/Arhgap6/Arhgef10/Cnn3/Epb41/Flnc/Kank4/Mef2c/Nebl/Pdlim4/Phactr1/Rac1/Sorbs1/Tmod1	14
BP	GO:1902115	regulation of organelle assembly	14/607	213/28814	3.12	0.000185365	0.004208181	0.003047075	Arhgef2/Chmp5/Dnm2/Dynll1/Elapor1/Gap43/Hap1/Lcp1/Msn/Nptx1/Ntrk3/Stam/Vdac3/Wipi1	14
BP	GO:0061025	membrane fusion	12/607	163/28814	3.49	0.000185875	0.004208181	0.003047075	Bloc1s6/Chmp5/Cplx1/Grik5/Pla2g4a/Rab34/Rab3a/Rab8b/Snap25/Stx3/Stxbp6/Vamp3	12
BP	GO:0016052	carbohydrate catabolic process	11/607	140/28814	3.73	0.0001938	0.004366904	0.003162004	Abhd10/Eif6/Eno2/Enpp1/Hexb/Hk2/Hmgb1/P2rx7/Stat3/Tpi1/Zbtb20	11
BP	GO:0010038	response to metal ion	17/607	295/28814	2.74	0.000195606	0.004386904	0.003176485	Adcy8/B2m/Camk2b/Cpne3/Enpp1/Kcnb1/Mapk3/Mef2c/Nptx1/P2rx7/Pla2g4a/Plcg2/Rasgrp2/Smpd3/Sod1/Stim1/Syt17	17
BP	GO:0030534	adult behavior	13/607	189/28814	3.27	0.000199943	0.004463208	0.003231736	Bbs2/Cend1/Crebbp/Cstb/Drd1/Enpp1/Homer2/Nlgn3/Nrxn3/Sez6/Slc10a4/Sncg/Tmod1	13
BP	GO:0060078	regulation of postsynaptic membrane potential	10/607	118/28814	4.02	0.000203138	0.004513439	0.003268107	Celf4/Dbn1/Dgki/Gabra3/Gabra4/Grik5/Mef2c/Nlgn3/Prkar1b/Sez6	10
BP	GO:0008064	regulation of actin polymerization or depolymerization	12/607	165/28814	3.45	0.000208243	0.004605456	0.003334735	Actn2/Arpc1b/Arpc3/Arpc5l/Cracd/Dbn1/Dbnl/Kank4/Rac1/Ssh3/Tmod1/Vasp	12
BP	GO:0009395	phospholipid catabolic process	7/607	58/28814	5.73	0.000210829	0.004619859	0.003345164	Gpcpd1/Pla2g4a/Pla2g7/Plcg2/Pld1/Smpd3/Smpd4	7
BP	GO:0015872	dopamine transport	7/607	58/28814	5.73	0.000210829	0.004619859	0.003345164	Drd1/Prkcb/Rab3a/Slc6a3/Smpd3/Sncg/Syt17	7
BP	GO:0007040	lysosome organization	8/607	77/28814	4.93	0.000217398	0.00469646	0.003400629	Abca1/Chmp5/Hexb/Lamtor1/P2rx7/Rab14/Rab34/Tmem106b	8
BP	GO:0080171	lytic vacuole organization	8/607	77/28814	4.93	0.000217398	0.00469646	0.003400629	Abca1/Chmp5/Hexb/Lamtor1/P2rx7/Rab14/Rab34/Tmem106b	8
BP	GO:0006090	pyruvate metabolic process	10/607	119/28814	3.99	0.000217666	0.00469646	0.003400629	Eif6/Eno2/Enpp1/Hk2/Me2/P2rx7/Pdk2/Stat3/Tpi1/Zbtb20	10
BP	GO:0021700	developmental maturation	19/607	355/28814	2.54	0.000218257	0.00469646	0.003400629	B4galt6/Bap1/Bloc1s6/Camk2b/Cdk5r2/Cend1/Dab2ip/Ednrb/Enpp1/Kcnb1/Kcnip2/Map3k13/Picalm/Rab3a/Rac1/Sez6/Sox10/Sox8/Tut	19
BP	GO:0048675	axon extension	11/607	142/28814	3.68	0.00021943	0.004700525	0.003403573	Dbn1/Dbnl/Dip2b/Dnm2/Map1k13/Nlgn3/Nrp2/Ntrk3/Sema7a/Slc9a6/Vcl	11
BP	GO:0099171	presynaptic modulation of chemical synaptic transmission	4/607	15/28814	12.66	0.000221245	0.004718258	0.003416413	P2rx7/Pkdcg5/Prkcb/Rab3a	4
BP	GO:2000113	negative regulation of cellular macromolecule biosynthetic process	14/607	217/28814	3.06	0.000224674	0.004770075	0.003453933	Cnot7/Dcp1a/Dcps/Dhx36/Eif6/Enpp1/Grb10/Igfbp5/Lsk3/Mrpl13/Noct/Paip1/Tia1/Tut4	14
BP	GO:0006631	fatty acid metabolic process	22/607	446/28814	2.34	0.000228633	0.004822469	0.003491871	Abcd3/Acot1/Acot11/Acsbg1/Cyp4f14/Echdc1/Eif6/Enpp1/Hacd3/Lipa/Mcat/Ndufab1/Nucb2/Nudt7/Pdk2/Pdpn/Pla2g4a/Prkag1/Prkar2b/	22
BP	GO:0060485	mesenchyme development	17/607	299/28814	2.7	0.000229161	0.004822469	0.003491871	Cdh2/Dab2ip/Ednrb/Efnb1/Flna/Mapk3/Mef2c/Nrp2/Pdcd4/Pdpn/Phactr4/Ptk7/Sdhaf2/Sema7a/Sox8/Tmem100	17
BP	GO:0006904	vesicle docking involved in exocytosis	6/607	42/28814	6.78	0.000234689	0.004917138	0.003560419	Bloc1s6/Kcnb1/Rab3a/Rab3c/Rab8b/Stx3	6
BP	GO:0030832	regulation of actin filament length	12/607	168/28814	3.39	0.000246678	0.00508186	0.003679691	Actn2/Arpc1b/Arpc3/Arpc5l/Cracd/Dbn1/Dbnl/Kank4/Rac1/Ssh3/Tmod1/Vasp	12
BP	GO:0014033	neural crest cell differentiation	9/607	99/28814	4.32	0.000247714	0.00508186	0.003679691	Cdh2/Ednrb/Efnb1/Mapk3/Mef2c/Nrp2/Phactr4/Sema7a/Sox8	9
BP	GO:0046031	ADP metabolic process	9/607	99/28814	4.32	0.000247714	0.00508186	0.003679691	Ak2/Eif6/Eno2/Enpp1/Hk2/Me2/P2rx7/Stat3/Tpi1/Zbtb20	9
BP	GO:0071496	cellular response to external stimulus	17/607	301/28814	2.68	0.00024774	0.00508186	0.003679691	Bag3/Brsk1/Comt/Dap/Dcps/Elapor1/Foxo1/Kcnb1/Kptn/Lamp2/Mapk3/P2rx7/Pdk2/Prkag1/Rac1/Rragb/Wipi1	17
BP	GO:0006665	sphingolipid metabolic process	11/607	144/28814	3.63	0.00024787	0.00508186	0.003679691	Abca8b/B4galt6/Bax/Hacd3/Hexb/Kdsr/P2rx7/Sgpl1/Sgpp1/Smpd3/Smpd4	11
BP	GO:0015837	amine transport	10/607	121/28814	3.92	0.000249347	0.005090296	0.0036858	Ddc/Kcnb1/P2rx7/Prkcb/Rab3a/Slc18a2/Slc6a3/Sncg/Syt17/Th	10
BP	GO:0046626	regulation of insulin receptor signaling pathway	8/607	79/28814	4.81	0.000259824	0.005281614	0.00382433	Enpp1/Grb10/Marcks/Nucb2/Nucks1/Osbpl8/Prkcb/Sorbs1	8
BP	GO:0048278	vesicle docking	7/607	60/28814	5.54	0.000261167	0.005286428	0.003827816	Bloc1s6/Kcnb1/Ndrg4/Rab3a/Rab3c/Rab8b/Stx3	7
BP	GO:0030833	regulation of actin filament polymerization	11/607	146/28814	3.58	0.000279361	0.005584823	0.004043879	Arpc1b/Arpc3/Arpc5l/Cracd/Dbn1/Dbnl/Kank4/Rac1/Ssh3/Tmod1/Vasp	11
BP	GO:0048193	Golgi vesicle transport	16/607	276/28814	2.75	0.000279417	0.005584823	0.004043879	Bbs2/Copg1/Dapk2/Dnm2/Ergic1/Kif1b/Myo1b/Rab14/Rab26/Rab31/Rab34/Rab8b/Sar1b/Scfd1/Vamp3/Wasl	16
BP	GO:0110053	regulation of actin filament organization	16/607	276/28814	2.75	0.000279417	0.005584823	0.004043879	Actn2/Arhgap6/Arhgef10/Arpc1b/Arpc3/Arpc5l/Cracd/Dbn1/Dbnl/Flna/Kank4/Pdlim4/Rac1/Ssh3/Tmod1/Vasp	16
BP	GO:0019722	calcium-mediated signaling	13/607	196/28814	3.15	0.00028519	0.005660109	0.004098392	Atp2b4/Camkk2/Drd1/Ednrb/Grm1/Homer2/Itgam1/Myo/Pdk2/Plcg2/Rcan1/Tmem1	13
BP	GO:0006470	protein dephosphorylation	15/607	249/28814	2.86	0.000285553	0.005660109	0.004098392	Arpp19/Bod1/Ensa/Ppp1cc/Ppp1r16b/Ppp2r2a/Ppp4r1/Ppp6r4/Ppa/Ptpn5/Ptpn6/R	15
BP	GO:0008654	phospholipid biosynthetic process	14/607	223/28814	2.98	0.000297011	0.005862907	0.004245235	Abca8b/Abhd4/Cds1/Dgki/Hdhd5/Hexb/Hmgcs1/Inpka/Pcyt1b/Pigt/Pip5k1a/Pla2g4	14
BP	GO:0042789	mRNA transcription by RNA polymerase II	6/607	44/28814	6.47	0.000304769	0.005966729	0.004320411	Anxa2/Ensp1/Flna/Hlf/Ncebp2/Stat3	6
BP	GO:0099084	postsynaptic specialization organization	6/607	44/28814	6.47	0.000304769	0.005966729	0.004320411	Cdh2/Dbn1/Gap43/Nlgn3/Nptx1/Ntrk3	6
BP	GO:0036465	synaptic vesicle recycling	8/607	81/28814	4.69	0.000308792	0.006020811	0.00435957	Actb/Dnm2/Nlgn3/Pacsin1/Picalm/Rab3a/Sh3gl3/Sncg	8
BP	GO:0001919	regulation of receptor recycling	5/607	29/28814	8.18	0.000318784	0.00612879	0.004437756	Actn/Anxa2/Chmp5/Lamtor1/Vamp3	5
BP	GO:0090023	positive regulation of neutrophil chemotaxis	5/607	29/28814	8.18	0.000318784	0.00612879	0.004437756	Camk1d/Mcu/Rac1/Rac2/Tbbs4	5
BP	GO:0006584	catecholamine metabolic process	7/607	62/28814	5.36	0.000320745	0.00612879	0.004437756	Comt/Ddc/Drd1/Pde1b/Ptk7/Slc6a3/Th	7
BP	GO:0009712	catechol-containing compound metabolic process	7/607	62/28814	5.36	0.000320745	0.00612879	0.004437756	Comt/Ddc/Drd1/Pde1b/Ptk7/Slc6a3/Th	7
BP	GO:0031102	neuron projection regeneration	7/607	62/28814	5.36	0.000320745	0.00612879	0.004437756	Enpp1/Flna/Gap43/Gfap/Map2k2/Ntrk3/Rtn4r2	7
BP	GO:0032386	regulation of intracellular transport	18/607	337/28814	2.54	0.000324552	0.006176834	0.004472544	Actn2/Anxa2/Bag3/Camk1/Dbn1/Flna/Hax1/Kcnb1/Lcp1/Map2k2/Mapk3/Mkl1/Mkm	18
BP	GO:0009135	purine nucleoside diphosphate metabolic process	9/607	103/28814	4.15	0.000333486	0.006271899	0.004541379	Ak2/Eif6/Eno2/Enpp1/Hk2/P2rx7/Stat3/Tpi1/Zbtb20	9
BP	GO:0009179	purine ribonucleoside diphosphate metabolic process	9/607	103/28814	4.15	0.000333486	0.006271899	0.004541379	Ak2/Eif6/Eno2/Enpp1/Hk2/P2rx7/Stat3/Tpi1/Zbtb20	9
BP	GO:0032479	regulation of type I interferon production	9/607	103/28814	4.15	0.000333486	0.006271899	0.004541379	Crebbp/Dhx36/Hmgb1/Hmgb2/Plcg2/Ptpn6/Stat1/Yy1/Zbtb20	9
BP	GO:0044270	cellular nitrogen compound catabolic process	21/607	426/28814	2.33	0.000337676	0.006325798	0.004580407	Aldh1l1/Apex1/Bax/Cnot7/Dcp1a/Dcps/Dhx36/Dxo/Endog/Enpp1/Exosc10/Hexb/Mrnip/Ncbp2/Noct/Nudt12/Nudt7/Pnp/Smg5/Tut4	21
BP	GO:0032414	positive regulation of ion transmembrane transporter activity	10/607	126/28814	3.77	0.000345818	0.006444401	0.004666285	Actn2/Dnm2/Hap1/Hspa2/Kcnc1/Nlgn3/Plcg2/Rgs9/Sl	10

BP	GO:1901663	quinone biosynthetic process	3/361	18/28814	13.3	0.001383865	0.025178083	0.019582108	Coq6/Coq7/Ndufa9	3
BP	GO:0070252	actin-mediated cell contraction	6/361	98/28814	4.89	0.001484311	0.026871903	0.020899466	Atp1a1/Atp2a2/Camk2d/Dlg1/Epdr1/Scn4b	6
BP	GO:0070588	calcium ion transmembrane transport	11/361	300/28814	2.93	0.00152773	0.027385281	0.021298743	Atp2a2/Atp2b1/Atp2b2/Cacnb3/Calm1/Camk2d/Cbarp/Jph3/S100a1/Slc24a2/Slc8a1	11
BP	GO:0009123	nucleoside monophosphate metabolic process	5/361	67/28814	5.96	0.001535133	0.027385281	0.021298743	Ampd3/Nt5m/Ppat/Prps1/Uprt	5
BP	GO:1902305	regulation of sodium ion transmembrane transport	5/361	67/28814	5.96	0.001535133	0.027385281	0.021298743	Ank3/Camk2d/Plcb1/Scn4b/Slmap	5
BP	GO:0006743	ubiquinone metabolic process	3/361	19/28814	12.6	0.001628165	0.028764246	0.022371226	Coq6/Coq7/Ndufa9	3
BP	GO:0015919	peroxisomal membrane transport	3/361	19/28814	12.6	0.001628165	0.028764246	0.022371226	Pex1/Pex13/Pex19	3
BP	GO:0009165	nucleotide biosynthetic process	9/361	215/28814	3.34	0.001647854	0.028972117	0.022532896	Ampd3/Coasy/Gcdh/Pdk1/Pdk3/Ppat/Prps1/Stat3/Uprt	9
BP	GO:0008277	regulation of G protein-coupled receptor signaling pathway	7/361	136/28814	4.11	0.001678353	0.029138495	0.022662295	Abhd6/Camk2a/Homer2/Phf24/Plcb1/Ppp1r9b/Rock2	7
BP	GO:0009582	detection of abiotic stimulus	7/361	136/28814	4.11	0.001678353	0.029138495	0.022662295	Atp2b2/Atp8a2/Cacnb3/Kcna1/Phf24/Slc12a2/Slc24a2	7
BP	GO:0001666	response to hypoxia	9/361	216/28814	3.33	0.001700996	0.029138495	0.022662295	Alkbh5/Camk2d/Cat/Chchd2/Pdk1/Pdk3/Rock2/Slc8a1/Smad4	9
BP	GO:0010613	positive regulation of cardiac muscle hypertrophy	4/361	41/28814	7.79	0.001702205	0.029138495	0.022662295	Camk2d/Cdk9/Ppp3ca/Rock2	4
BP	GO:2000463	positive regulation of excitatory postsynaptic potential	4/361	41/28814	7.79	0.001702205	0.029138495	0.022662295	Cacnb3/Nrxn1/Rims2/Stx1a	4
BP	GO:0072522	purine-containing compound biosynthetic process	8/361	175/28814	3.65	0.001705124	0.029138495	0.022662295	Ampd3/Coasy/Gcdh/Pdk1/Pdk3/Ppat/Prps1/Stat3	8
BP	GO:0009581	detection of external stimulus	7/361	137/28814	4.08	0.001750115	0.029499932	0.022943401	Atp2b2/Atp8a2/Cacnb3/Kcna1/Phf24/Slc12a2/Slc24a2	7
BP	GO:0099601	regulation of neurotransmitter receptor activity	5/361	69/28814	5.78	0.001750475	0.029499932	0.022943401	Cnih2/Gsg1l/Nptx1/Nrxn1/Shisa9	5
BP	GO:1904377	positive regulation of protein localization to cell periphery	5/361	69/28814	5.78	0.001750475	0.029499932	0.022943401	Dlg1/Epb41l2/Nrxn1/Ppp1r9b/Sorbs1	5
BP	GO:0014742	positive regulation of muscle hypertrophy	4/361	42/28814	7.6	0.001863014	0.031160867	0.024235184	Camk2d/Cdk9/Ppp3ca/Rock2	4
BP	GO:0086003	cardiac muscle cell contraction	5/361	70/28814	5.7	0.001866073	0.031160867	0.024235184	Atp1a1/Atp2a2/Camk2d/Dlg1/Scn4b	5
BP	GO:0006541	glutamine metabolic process	3/361	20/28814	11.97	0.001897811	0.031262592	0.0243143	Glul/Phgdh/Ppat	3
BP	GO:0006620	post-translational protein targeting to endoplasmic reticulum membrane	3/361	20/28814	11.97	0.001897811	0.031262592	0.0243143	Sec61a1/Sec61a2/Sgtb	3
BP	GO:0050966	detection of mechanical stimulus involved in sensory perception of pain	3/361	20/28814	11.97	0.001897811	0.031262592	0.0243143	Cacnb3/Kcna1/Phf24	3
BP	GO:1901293	nucleoside phosphate biosynthetic process	9/361	220/28814	3.27	0.001927466	0.03160871	0.024583491	Ampd3/Coasy/Gcdh/Pdk1/Pdk3/Ppat/Prps1/Stat3/Uprt	9
BP	GO:0055117	regulation of cardiac muscle contraction	5/361	71/28814	5.62	0.00198716	0.032442153	0.025231697	Atp1a1/Atp2a2/Calm1/Dlg1/Slc8a1	5
BP	GO:0009124	nucleoside monophosphate biosynthetic process	4/361	43/28814	7.42	0.002034044	0.032913717	0.025598453	Ampd3/Ppat/Prps1/Uprt	4
BP	GO:0042181	ketone biosynthetic process	4/361	43/28814	7.42	0.002034044	0.032913717	0.025598453	Clcn2/Coq6/Coq7/Ndufa9	4
BP	GO:0050890	cognition	12/361	359/28814	2.67	0.00206575	0.033279499	0.025882938	Apbb1/Bdnf/Cck/Eif2ak4/Jph3/Map1a/Nrxn1/Pja2/Plcb1/Slc17a7/Slc24a2/Tpbg	12
BP	GO:0010666	positive regulation of cardiac muscle cell apoptotic process	3/361	21/28814	11.4	0.002193693	0.03487972	0.0271275	Camk2a/Camk2d/Smad4	3
BP	GO:0098962	regulation of postsynaptic neurotransmitter receptor activity	3/361	21/28814	11.4	0.002193693	0.03487972	0.0271275	Cnih2/Nptx1/Shisa9	3
BP	GO:1904385	cellular response to angiotensin	3/361	21/28814	11.4	0.002193693	0.03487972	0.0271275	Camk2a/Rock2/Slc26a6	3
BP	GO:1903532	positive regulation of secretion by cell	12/361	363/28814	2.64	0.002262443	0.035817122	0.027856559	Apbb1/Cadps/Cadps2/Cck/Frmd4a/Glul/Pcp4/Pcsk1/Rab2b/Slc12a2/Smad4/Stx1a	12
BP	GO:0006821	chloride transport	6/361	107/28814	4.48	0.002320767	0.036582086	0.028451505	Clcn2/Gabra3/Gabrb1/Slc12a2/Slc26a6/Slc4a1	6
BP	GO:0017158	regulation of calcium ion-dependent exocytosis	4/361	45/28814	7.09	0.002408064	0.037633723	0.029269409	Atp2a2/Cadps/Cbarp/Stx1a	4
BP	GO:0086009	membrane repolarization	4/361	45/28814	7.09	0.002408064	0.037633723	0.029269409	Atp1a1/Cacnb3/Scn4b/Zmpste24	4
BP	GO:0055001	muscle cell development	9/361	228/28814	3.15	0.002452501	0.038165084	0.029682673	Atp2a2/Camk2d/Csrp1/Foxp1/Ppp3ca/Slc8a1/Smad4/Tmod1/Zmpste24	9
BP	GO:0010663	positive regulation of striated muscle cell apoptotic process	3/361	22/28814	10.88	0.002516654	0.038347521	0.029824562	Camk2a/Camk2d/Smad4	3
BP	GO:0043574	peroxisomal transport	3/361	22/28814	10.88	0.002516654	0.038347521	0.029824562	Pex1/Pex13/Pex19	3
BP	GO:0048791	calcium ion-regulated exocytosis of neurotransmitter	3/361	22/28814	10.88	0.002516654	0.038347521	0.029824562	Atp2a2/Cadps/Rims2	3
BP	GO:0071398	cellular response to fatty acid	3/361	22/28814	10.88	0.002516654	0.038347521	0.029824562	Foxp1/Pdk3/Plcb1	3
BP	GO:1900242	regulation of synaptic vesicle endocytosis	3/361	22/28814	10.88	0.002516654	0.038347521	0.029824562	Ppp3cc/Sh3gl1/Slc17a7	3
BP	GO:2001258	negative regulation of cation channel activity	4/361	46/28814	6.94	0.002611696	0.039630585	0.030822457	Ank3/Cacnb3/Calm1/Cbarp	4
BP	GO:0033865	nucleoside bisphosphate metabolic process	6/361	110/28814	4.35	0.002666106	0.039958815	0.031077736	Coasy/Gcdh/Mvd/Mvk/Pdk1/Pdk3	6
BP	GO:0033875	ribonucleoside bisphosphate metabolic process	6/361	110/28814	4.35	0.002666106	0.039958815	0.031077736	Coasy/Gcdh/Mvd/Mvk/Pdk1/Pdk3	6
BP	GO:0034032	purine nucleoside bisphosphate metabolic process	6/361	110/28814	4.35	0.002666106	0.039958815	0.031077736	Coasy/Gcdh/Mvd/Mvk/Pdk1/Pdk3	6
BP	GO:0110053	regulation of actin filament organization	10/361	276/28814	2.89	0.002686565	0.040101093	0.031188393	Add2/Arpc5l/Cd47/Dlg1/Pxn/Rock2/Sorbs3/Tjp1/Tmod1/Vasp	10
BP	GO:0030534	adult behavior	8/361	189/28814	3.38	0.002750299	0.040885547	0.031798497	Bdnf/Cend1/Homer2/Nrxn1/Oxr1/Scn8a/Tmod1/Zmpste24	8
BP	GO:0110020	regulation of actomyosin structure organization	6/361	111/28814	4.31	0.002789361	0.041298348	0.032119551	Cd47/Pxn/Rock2/Smad4/Sorbs3/Tjp1	6
BP	GO:0007628	adult walking behavior	4/361	47/28814	6.79	0.002826831	0.041612808	0.03236412	Cend1/Oxr1/Scn8a/Zmpste24	4
BP	GO:0010880	regulation of release of sequestered calcium ion into cytosol by sarcoplasmic reticulum	3/361	23/28814	10.41	0.002867495	0.041612808	0.03236412	Calm1/Camk2d/Slc8a1	3
BP	GO:0099149	regulation of postsynaptic neurotransmitter receptor internalization	3/361	23/28814	10.41	0.002867495	0.041612808	0.03236412	Gsg1l/Numb/Ppp3r1	3
BP	GO:0099170	postsynaptic modulation of chemical synaptic transmission	3/361	23/28814	10.41	0.002867495	0.041612808	0.03236412	Plcb1/Ppp3ca/Ppp3r1	3
BP	GO:1900120	regulation of receptor binding	3/361	23/28814	10.41	0.002867495	0.041612808	0.03236412	Adam15/Atp2a2/Bdnf	3
BP	GO:1901617	organic hydroxy compound biosynthetic process	9/361	234/28814	3.07	0.002916146	0.041836803	0.032538331	Clcn2/Cyb5r1/Dhcr7/Itpka/Mvd/Mvk/Pck2/Ppip5k2/Prkaa1	9
BP	GO:1905477	positive regulation of protein localization to membrane	6/361	112/28814	4.28	0.002916833	0.041836803	0.032538331	Ank3/Cacnb3/Dlg1/Nrxn1/Ppp1r9b/Sorbs1	6
BP	GO:0071333	cellular response to glucose stimulus	7/361	150/28814	3.72	0.00291725	0.041836803	0.032538331	Nrxn1/Pck2/Pdk3/Ppp3ca/Prkaa1/Rab11b/Smad4	7
BP	GO:0032413	negative regulation of ion transmembrane transporter activity	5/361	78/28814	5.12	0.003001253	0.042873372	0.033344516	Ank3/Cacnb3/Calm1/Camk2d/Cbarp	5

CC	GO:0005790	smooth endoplasmic reticulum	3/263	35/28739	9.37	0.003994385	0.042391323	0.032653404	Pdia4/Psen1/Th	3
CC	GO:0009925	basal plasma membrane	8/263	276/28739	3.17	0.004084581	0.042391323	0.032653404	Atp1b1/Cd81/Entpd1/Erbin/Shroom4/Slc38a1/Slc9a1/Stx2	8
CC	GO:0032809	neuronal cell body membrane	3/263	38/28739	8.63	0.005045854	0.049224362	0.037916792	Kcnc1/Kcnc3/Slc6a3	3
CC	GO:0034663	endoplasmic reticulum chaperone complex	2/263	12/28739	18.21	0.00518304	0.049224362	0.037916792	Dnajc10/Pdia4	2
CC	GO:0005759	mitochondrial matrix	8/263	288/28739	3.04	0.005255669	0.049224362	0.037916792	Cs/Dhfr/Mrpl51/Mrps18b/Mrps23/Pin4/Pmpcb/Slc25a5	8
CC	GO:0015934	large ribosomal subunit	5/263	122/28739	4.48	0.005374609	0.049224362	0.037916792	Mrpl51/Rpl31/Rpl35a/Rpl7/Rpl8	5
CC	GO:0099572	postsynaptic specialization	9/263	352/28739	2.79	0.005383915	0.049224362	0.037916792	Cacng8/Cnn3/Entpd1/Nptn/Rnf112/Rpl7/Rpl8/Slc8a2/Slc8a3	9
CC	GO:0015935	small ribosomal subunit	4/263	78/28739	5.6	0.005756869	0.051410177	0.039600492	Mrps18b/Mrps23/Rps15a/Rps26	4
CC	GO:0036464	cytoplasmic ribonucleoprotein granule	7/263	234/28739	3.27	0.005976289	0.051956026	0.040020952	Iqgap1/Nufip2/Rbfox1/Rock1/Tia1/Ybx1/Ythdf2	7
CC	GO:0031074	nucleocytoplasmic transport complex	2/263	13/28739	16.81	0.006088597	0.051956026	0.040020952	Kpna4/Kpna6	2
CC	GO:0044298	cell body membrane	3/263	41/28739	8	0.006249211	0.052167328	0.040183715	Kcnc1/Kcnc3/Slc6a3	3
CC	GO:0045178	basal part of cell	8/263	301/28739	2.9	0.006797207	0.055332857	0.042622074	Atp1b1/Cd81/Entpd1/Erbin/Shroom4/Slc38a1/Slc9a1/Stx2	8
CC	GO:0090533	cation-transporting ATPase complex	2/263	14/28739	15.61	0.007060703	0.055332857	0.042622074	Atp1b1/Slc9a1	2
CC	GO:1990124	messenger ribonucleoprotein complex	2/263	14/28739	15.61	0.007060703	0.055332857	0.042622074	Cpeb3/Ybx1	2
CC	GO:0016323	basolateral plasma membrane	7/263	246/28739	3.11	0.007775683	0.059717243	0.045999302	Atp1b1/Cd81/Entpd1/Erbin/Slc38a1/Slc9a1/Stx2	7
CC	GO:0035770	ribonucleoprotein granule	7/263	252/28739	3.04	0.008812313	0.065418273	0.05039072	Iqgap1/Nufip2/Rbfox1/Rock1/Tia1/Ybx1/Ythdf2	7
CC	GO:0034705	potassium channel complex	4/263	89/28739	4.91	0.009116979	0.065418273	0.05039072	Abcc9/Kcnc1/Kcnc3/Kcnh2	4
CC	GO:0000930	gamma-tubulin complex	2/263	16/28739	13.66	0.009199445	0.065418273	0.05039072	Tubg1/Tubgcp2	2
CC	GO:0098533	ATPase dependent transmembrane transport complex	2/263	16/28739	13.66	0.009199445	0.065418273	0.05039072	Atp1b1/Slc9a1	2
CC	GO:0044291	cell-cell contact zone	4/263	90/28739	4.86	0.009473957	0.066145448	0.050950852	Atp1b1/Nectin1/Slc9a1/Ywhah	4
CC	GO:0098978	glutamatergic synapse	8/263	323/28739	2.71	0.010155097	0.069634953	0.053638766	Ap2m1/Cacng8/Cadps2/Dnm1/Epha7/Erbin/Grip1/Ywhah	8
CC	GO:0098688	parallel fiber to Purkinje cell synapse	2/263	17/28739	12.86	0.010363564	0.069817696	0.05377953	Cadm3/Cadps2	2
CC	GO:0032590	dendrite membrane	3/263	50/28739	6.56	0.010819495	0.071632517	0.05517746	Cacng8/Kcnc1/Kcnc3	3
CC	GO:0031312	extrinsic component of organelle membrane	3/263	51/28739	6.43	0.011419336	0.072954975	0.05619613	Coq8a/Snx10/Tor1a	3
CC	GO:0005847	mRNA cleavage and polyadenylation specificity factor complex	2/263	18/28739	12.14	0.011589202	0.072954975	0.05619613	Pip5k1a/Ssu72	2
CC	GO:0032039	integrator complex	2/263	18/28739	12.14	0.011589202	0.072954975	0.05619613	Ints14/Ints9	2
CC	GO:0030173	integral component of Golgi membrane	3/263	52/28739	6.3	0.012038002	0.07455795	0.057430877	B4galnt1/Cux1/Ier3ip1	3
CC	GO:0031228	intrinsic component of Golgi membrane	3/263	54/28739	6.07	0.013332163	0.079992978	0.061617398	B4galnt1/Cux1/Ier3ip1	3
CC	GO:0071011	precatalytic spliceosome	3/263	54/28739	6.07	0.013332163	0.079992978	0.061617398	Prpf38b/Sf3b5/Snrpf	3
CC	GO:0043209	myelin sheath	6/263	215/28739	3.05	0.014563402	0.086036096	0.066272321	Atp1b1/Dnm1/Gnb4/Slc25a3/Slc25a4/Slc25a5	6
CC	GO:0031256	leading edge membrane	5/263	158/28739	3.46	0.015347777	0.089211539	0.068718318	Akt2/Cacng8/Kcnc1/Kcnc3/Pip5k1a	5
CC	GO:0030673	axolemma	2/263	21/28739	10.41	0.01562316	0.089211539	0.068718318	Kcnc1/Kcnc3	2
CC	GO:0031301	integral component of organelle membrane	8/263	350/28739	2.5	0.015797877	0.089211539	0.068718318	B4galnt1/Cpt1a/Cux1/Ier3ip1/Slc25a3/Tmem177/Tmem33/Wfs1	8
CC	GO:0005849	mRNA cleavage factor complex	2/263	22/28739	9.93	0.017082891	0.095070004	0.073231007	Pip5k1a/Ssu72	2
CC	GO:0034703	cation channel complex	6/263	224/28739	2.93	0.017470635	0.095838914	0.073823286	Abcc9/Cacng8/Kcnc1/Kcnc3/Kcnh2/Scn3b	6
MF	GO:0022853	active ion transmembrane transporter activity	11/254	238/28275	5.14	1.13504E-05	0.004084379	0.003271419	Abcc9/Atp1b1/Cox7c/Slc18a2/Slc25a4/Slc25a5/Slc38a1/Slc6a3/Slc8a2/Slc8a3/Slc9a1	11
MF	GO:0003735	structural constituent of ribosome	9/254	164/28275	6.11	1.86623E-05	0.004084379	0.003271419	Mrpl51/Mrps18b/Mrps23/Rpl31/Rpl35a/Rpl7/Rpl8/Rps15a/Rps26	9
MF	GO:0008509	anion transmembrane transporter activity	12/254	313/28275	4.27	2.93061E-05	0.004084379	0.003271419	Lrrc8b/Lrrc8d/Slc18a2/Slc25a15/Slc25a21/Slc25a4/Slc25a42/Slc25a5/Slc38a1/Slc6a3/Slc9a1/Ttyh2	12
MF	GO:0046873	metal ion transmembrane transporter activity	14/254	421/28275	3.7	3.1001E-05	0.004084379	0.003271419	Abcc9/Atp1b1/Cacng8/Kcnc1/Kcnc3/Kcnh2/Psen1/Scn3b/Slc18a2/Slc38a1/Slc6a3/Slc8a2/Slc8a3/Slc9a1	14
MF	GO:0015081	sodium ion transmembrane transporter activity	8/254	145/28275	6.14	5.2459E-05	0.00552918	0.004428645	Atp1b1/Scn3b/Slc18a2/Slc38a1/Slc6a3/Slc8a2/Slc8a3/Slc9a1	8
MF	GO:0005347	ATP transmembrane transporter activity	3/254	11/28275	30.36	0.000112073	0.009843717	0.007884411	Slc25a4/Slc25a42/Slc25a5	3
MF	GO:0044548	S100 protein binding	3/254	14/28275	23.85	0.000242352	0.01670614	0.013380929	Cacybp/Iqgap1/S100a11	3
MF	GO:0022804	active transmembrane transporter activity	12/254	396/28275	3.37	0.000268435	0.01670614	0.013380929	Abcc9/Atp1b1/Cox7c/Slc18a2/Slc25a3/Slc25a4/Slc25a5/Slc38a1/Slc6a3/Slc8a2/Slc8a3/Slc9a1	12
MF	GO:0017110	nucleoside-diphosphatase activity	3/254	15/28275	22.26	0.000300932	0.01670614	0.013380929	Entpd1/Entpd3/Slc25a42	3
MF	GO:0005516	calmodulin binding	8/254	192/28275	4.64	0.000363007	0.01670614	0.013380929	Cnn3/Iqgap1/Iqgap2/Phkg2/Slc8a2/Slc8a3/Slc9a1/Wfs1	8
MF	GO:0015291	secondary active transmembrane transporter activity	9/254	242/28275	4.14	0.000363984	0.01670614	0.013380929	Slc18a2/Slc25a3/Slc25a4/Slc25a5/Slc38a1/Slc6a3/Slc8a2/Slc8a3/Slc9a1	9
MF	GO:0044325	transmembrane transporter binding	7/254	147/28275	5.3	0.000380658	0.01670614	0.013380929	Abcc9/Ap2m1/Fgf12/Kcnc1/Nptn/Scn3b/Ywhah	7
MF	GO:0000295	adenine nucleotide transmembrane transporter activity	3/254	17/28275	19.64	0.000443806	0.01670614	0.013380929	Slc25a4/Slc25a42/Slc25a5	3
MF	GO:0005346	purine ribonucleotide transmembrane transporter activity	3/254	17/28275	19.64	0.000443806	0.01670614	0.013380929	Slc25a4/Slc25a42/Slc25a5	3
MF	GO:0015216	purine nucleotide transmembrane transporter activity	3/254	19/28275	17.58	0.000624081	0.021926062	0.017561871	Slc25a4/Slc25a42/Slc25a5	3
MF	GO:0015297	antiporter activity	5/254	83/28275	6.71	0.000923201	0.030407925	0.024355494	Slc25a4/Slc25a5/Slc8a2/Slc8a3/Slc9a1	5
MF	GO:0015215	nucleotide transmembrane transporter activity	3/254	23/28275	14.52	0.001110751	0.034433293	0.027579648	Slc25a4/Slc25a42/Slc25a5	3
MF	GO:0015491	cation:cation antiporter activity	3/254	24/28275	13.91	0.00126105	0.036920729	0.029571981	Slc8a2/Slc8a3/Slc9a1	3
MF	GO:0016667	oxidoreductase activity, acting on a sulfur group of donors	4/254	54/28275	8.25	0.00141391	0.038793789	0.031072225	Dnajc10/Glrx3/Pcyox1l/Pdia4	4
MF	GO:0019842	vitamin binding	6/254	136/28275	4.91	0.00147225	0.038793789	0.031072225	Cbs/Ddc/Dhfr/Gcat/P4ha1/Rbp1	6
MF	GO:0005525	GTP binding	10/254	359/28275	3.1	0.001615884	0.040551006	0.032479683	Dnm1/Drg1/Gfm2/Gmppb/Gpn1/Guf1/Rnf112/Tgm2/Tuba1/Uprt	10

BP	GO:1903829	positive regulation of protein localization	23/607	491/28814	2.22	0.000346705	0.006444401	0.004666285	Adcy8/Atp2b4/Bag3/Camk1/Dynll1/Epb41/Flna/Frmd4a/Kcnb1/Larp7/Mcu/Meak7/Msn/Nlgn3/P2rx7/Pcm1/Pls1/Ptpn5/Rab34/Rac1/Sar1	23
BP	GO:0046700	heterocycle catabolic process	21/607	429/28814	2.32	0.000348238	0.006447795	0.004668742	Aldh1l1/Apex1/Bax/Cnot7/Dcp1a/Dcps/Dhx36/Dnph1/Endog/Enpp1/Exosc10/Hnrnpa0/Mtrex/Ncbp2/Noct/Nudt12/Nudt7/Paip1/Smg5/Tu	21
BP	GO:0034655	nucleobase-containing compound catabolic process	20/607	399/28814	2.38	0.000353432	0.006510817	0.004714376	Apex1/Bax/Cnot7/Dcp1a/Dcps/Dhx36/Dnph1/Endog/Enpp1/Exosc10/Hnrnpa0/Mtrex/Ncbp2/Noct/Nudt12/Nudt7/Paip1/Smg5/Tut4/Vim	20
BP	GO:0006893	Golgi to plasma membrane transport	7/607	63/28814	5.27	0.000354367	0.006510817	0.004714376	Bbs2/Dnm2/Rab26/Rab31/Rab34/Stxbp6/Vamp3	7
BP	GO:0006165	nucleoside diphosphate phosphorylation	9/607	104/28814	4.11	0.00035838	0.006559319	0.004749495	Ak5/Eif6/Eno2/Enpp1/Hk2/P2rx7/Stat3/Tpi1/Zbtb20	9
BP	GO:0002262	myeloid cell homeostasis	13/607	201/28814	3.07	0.000363576	0.006629023	0.004799967	B2m/Bap1/Bax/Heph/Hmgb1/Hmgb2/Jak3/Kdm1a/Setd1a/Slc25a5/Sod1/Stat1/Stat	13
BP	GO:0060291	long-term synaptic potentiation	15/607	255/28814	2.79	0.000367635	0.006651379	0.004816154	Adcy8/Camk2b/Drd1/Enpp1/Gfap/Mme/Nlgn3/Nrgn/Prkar1b/Prrt1/Ptpn5/Rab3a/S	15
BP	GO:0090136	epithelial cell-cell adhesion	4/607	17/28814	11.17	0.000373053	0.006651379	0.004816154	Ctnna3/Plekha7/Thbs4/Vcl	4
BP	GO:0098974	postsynaptic actin cytoskeleton organization	4/607	17/28814	11.17	0.000373053	0.006651379	0.004816154	Actb/Dbn1/Dbnl/Farp1	4
BP	GO:0099188	postsynaptic cytoskeleton organization	4/607	17/28814	11.17	0.000373053	0.006651379	0.004816154	Actb/Dbn1/Dbnl/Farp1	4
BP	GO:0007189	adenylate cyclase-activating G protein-coupled receptor signaling pathway	11/607	151/28814	3.46	0.000373157	0.006651379	0.004816154	Abca1/Adcy3/Adcy8/Adgrg1/Atp2b4/Drd1/Gnal/Grk5/Htr4/Pde10a/Pde4a	11
BP	GO:0009152	purine ribonucleotide biosynthetic process	11/607	151/28814	3.46	0.000373157	0.006651379	0.004816154	Adcy3/Adcy8/Ak2/Atp5pb/Coasy/Dcakd/Dip2a/Gucy1b1/Nme3/Pdk2/Stat3	11
BP	GO:0001963	synaptic transmission, dopaminergic	5/607	30/28814	7.91	0.000375959	0.006651691	0.004816381	Drd1/Pnkd/Rasd2/Slc6a3/Th	5
BP	GO:0007214	gamma-aminobutyric acid signaling pathway	5/607	30/28814	7.91	0.000375959	0.006651691	0.004816381	Gabra3/Gabra4/Gabrb2/Htr4/Shisa7	5
BP	GO:0098656	anion transmembrane transport	13/607	202/28814	3.05	0.000381276	0.006720862	0.004866466	Ano3/Gabra3/Gabra4/Gabrb2/Gabrd/Gfap/Grm1/Slc1a3/Slc25a18/Slc25a5/Slc6a9/	13
BP	GO:0051235	maintenance of location	18/607	342/28814	2.5	0.000386836	0.006778736	0.004908372	Bax/Cds1/Dbn1/Drd1/Enpp1/Flna/Ftl1/Grik5/Hap1/Hexb/Hk2/Osbpl8/P2rx7/Plcg2/Ptpn6/Ryr3/Skp1/Tmem38a	18
BP	GO:0007249	I-kappaB kinase/NF-kappaB signaling	14/607	229/28814	2.9	0.000388465	0.006778736	0.004908372	Brd4/Dab2ip/Dhx36/Dnaja3/Ednrb/Hacd3/Plcg2/Prkcb/S100a4/Sirpa/Stat1/Traf6/T	14
BP	GO:0048638	regulation of developmental growth	20/607	402/28814	2.36	0.000388816	0.006778736	0.004908372	Bbs2/Dbn1/Dbnl/Dip2b/Dnm2/Fgf13/Map3k13/Mef2c/Naca/Ntrk3/Picalm/Pls1/Rnf157/Sema7a/Sgpl1/Slc6a3/	20
BP	GO:0045807	positive regulation of endocytosis	10/607	128/28814	3.71	0.000392264	0.006813976	0.004933888	Anxa2/B2m/Cd151/Clu/Dnm2/Eef2k/Picalm/Plcg2/Ptpn5/Rab31	10
BP	GO:0015698	inorganic anion transport	11/607	152/28814	3.44	0.00039478	0.006831602	0.004946651	Ano3/Clcn2/Clic1/Enpp1/Gabra3/Gabra4/Gabrb2/Gabr	11
BP	GO:2000300	regulation of synaptic vesicle exocytosis	8/607	84/28814	4.52	0.000396139	0.006831602	0.004946651	Apba1/Apba2/Dgki/Drd1/Nrxn3/P2rx7/Prkcb/Rab3a	8
BP	GO:0090150	establishment of protein localization to membrane	15/607	257/28814	2.77	0.000399156	0.006858878	0.004966401	Bax/Bbs2/Cplx1/Kcnb1/Naca/Rab26/Rab31/Rab34/Rab8b/Sec61b/Snap25/Stx3/Ti	15
BP	GO:0030010	establishment of cell polarity	11/607	153/28814	3.41	0.000417444	0.007146389	0.005174583	Arhgef2/Brsk1/Coro7/Fgf13/Frmd4a/Frmd4b/Lmna/Msn/Nek3/Ptk7/Vangl2	11
BP	GO:0031503	protein-containing complex localization	13/607	204/28814	3.03	0.00041888	0.007146389	0.005174583	Cplx1/Dbn1/Dynll1/Eif6/Hap1/Hspb11/Ift81/Nptx1/Pacsin1/Pcm1/Rcc2/Snap25/Stx	13
BP	GO:0006820	anion transport	23/607	498/28814	2.19	0.000422184	0.007177123	0.005196837	Abcd3/Ano3/Apba1/Clcn2/Clic1/Enpp1/Gabra3/Gabra4/Gabrb2/Gabrd/Gfap/Grm1/P2rx7/Pla2g4a/Slc10a4/Slc1a3/Slc25a18/Slc25a5/Slc6a9/Slc7a5/Ttyh1/Ttyh2/Vda	23
BP	GO:0006081	cellular aldehyde metabolic process	7/607	65/28814	5.11	0.000430066	0.007242897	0.005244463	Clcn2/Dhrs13/Dkk3/Ednrb/Esd/Pnkd/Tpi1	7
BP	GO:0051924	regulation of calcium ion transport	16/607	287/28814	2.65	0.000430185	0.007242897	0.005244463	Atp2b1/Bax/Drd1/Epb41/Gjc2/Gnb5/Hap1/Homer2/Hspa2/Jak3/P2rx7/Plcg2/Ptpn6/	16
BP	GO:0051090	regulation of DNA-binding transcription factor activity	21/607	436/28814	2.29	0.000430601	0.007242897	0.005244463	Adcy8/Anxa4/Arhgef2/Camk1d/Clu/Commd6/Crebbp/Dab2ip/Dap/Dnaja3/Enpp1/Flna/Kdm1a/Map3k13/Mapk3/Plcg2/Prkcb/Stat3/Traf6/T	21
BP	GO:0097485	neuron projection guidance	15/607	259/28814	2.75	0.000432967	0.007257138	0.005254775	Cdk5r2/Chn1/Dpysl4/Efnb1/Gap43/Mef2c/Nexn/Nrp2/Plxnc1/Rac1/Rac2/Scn1b/Se	15
BP	GO:0043277	apoptotic cell clearance	6/607	47/28814	6.06	0.00043951	0.007312953	0.005295189	Hmgb1/Rab14/Rac1/Rac2/Xkr5/Xkr9	6
BP	GO:0071624	positive regulation of granulocyte chemotaxis	5/607	31/28814	7.66	0.000440551	0.007312953	0.005295189	Camk1d/Mcu/Rac1/Rac2/Thbs4	5
BP	GO:0032606	type I interferon production	9/607	107/28814	3.99	0.000442421	0.007312953	0.005295189	Crebbp/Dhx36/Hmgb1/Hmgb2/Plcg2/Sirpa/Stat1/Yy1/	9
BP	GO:0046939	nucleotide phosphorylation	9/607	107/28814	3.99	0.000442421	0.007312953	0.005295189	Ak5/Eif6/Eno2/Enpp1/Hk2/P2rx7/Stat3/Tpi1/Zbtb20	9
BP	GO:0042692	muscle cell differentiation	21/607	438/28814	2.28	0.000457053	0.007502892	0.005432721	Actn2/Camk1/Cdh2/Dnmt1/Ednrb/Flnc/Igfbp5/Kdm1a/Lmna/Mef2c/Naca/Nebl/Olfm2/Pdcd4/Rcan1/Setd3/Speg/Stim1/Tmod1/Vangl2/Yy	21
BP	GO:0070997	neuron death	21/607	438/28814	2.28	0.000457053	0.007502892	0.005432721	Ache/Akt1s1/Bax/Clu/Dpysl4/Eno2/Enpp1/Gabrb2/Grik5/Kcnb1/Meak7/Mef2c/Msh2/Picalm/Pigt/Ptpn5/Rras2/Sigmar1/Sncg/Sod1/Stat3	21
BP	GO:0051592	response to calcium ion	10/607	131/28814	3.62	0.000471583	0.007650849	0.005539854	Adcy8/Cpne3/Kcnb1/Mef2c/P2rx7/Pla2g4a/Plcg2/Rasgr	10
BP	GO:0060134	prepulse inhibition	4/607	18/28814	10.55	0.000471681	0.007650849	0.005539854	Dbn1/Fabp7/Nlgn3/Slc6a3	4
BP	GO:0097091	synaptic vesicle clustering	4/607	18/28814	10.55	0.000471681	0.007650849	0.005539854	Cdh2/Nlgn3/Picalm/Rab3a	4
BP	GO:0072665	protein localization to vacuole	7/607	66/28814	5.03	0.000472472	0.007650849	0.005539854	Clu/Kptn/Lamp2/Meak7/Mon1b/Stam/Zfyve16	7
BP	GO:0090596	sensory organ morphogenesis	17/607	319/28814	2.53	0.000482927	0.007793717	0.005643303	Aldh1a1/Bax/Bcar3/Enpp1/Gjb6/Mapk3/Phactr4/Pls1/Prom1/Ptk7/Rac1/Sod1/Sox8	17
BP	GO:0001941	postsynaptic membrane organization	6/607	48/28814	5.93	0.000493454	0.007936805	0.00574691	Cdh2/Dbn1/Dnaja3/Nlgn3/Shisa7/Sorbs1	6
BP	GO:0042593	glucose homeostasis	16/607	291/28814	2.61	0.000500159	0.008017655	0.005805452	Adcy8/Csmd1/Dynll1/Enpp1/Foxo1/Hk2/Igfbp5/Kcnb1/Mcu/Nucks1/Pdk2/Rab34/Rac1/Slc18a2/Stat3/Zbtb20	16
BP	GO:0140053	mitochondrial gene expression	9/607	109/28814	3.92	0.000506968	0.008099623	0.005864804	Chchd1/Coa3/Ears2/Mrpl44/Mrps14/Mterf2/Sars2/Slc25a33/Taco1	9
BP	GO:0009416	response to light stimulus	17/607	321/28814	2.51	0.00051823	0.008238433	0.005965314	Bax/Brsk1/Crebbp/Dhx36/Drd1/Gnb5/Kdm1a/Mme/Msh2/Ndrg4/Nlgn3/Pcna/Pde1b/Ppp1cc/Rgs9/Slc1a3/Yy	17
BP	GO:0033500	carbohydrate homeostasis	16/607	292/28814	2.6	0.000519106	0.008238433	0.005965314	Adcy8/Csmd1/Dynll1/Enpp1/Foxo1/Hk2/Igfbp5/Kcnb1/Mcu/Nucks1/Pdk2/Rab34/Rac1/Slc18a2/Stat3/Zbtb20	16
BP	GO:0042180	cellular ketone metabolic process	14/607	236/28814	2.82	0.000524597	0.008298006	0.00600845	Atp2b4/Clcn2/Comt/Coq3/Cyp4f14/Dkk3/Ednrb/Eif6/Kdsr/Nucb2/Pdk2/Pla2g4a/	14
BP	GO:0033865	nucleoside bisphosphate metabolic process	9/607	110/28814	3.88	0.000542026	0.008489369	0.006147013	Acot1/Acot11/Coasy/Dcakd/Dip2a/Enpp1/Hmgcs1/Nud	9
BP	GO:0033875	ribonucleoside bisphosphate metabolic process	9/607	110/28814	3.88	0.000542026	0.008489369	0.006147013	Acot1/Acot11/Coasy/Dcakd/Dip2a/Enpp1/Hmgcs1/Nudt7/Pdk2	9
BP	GO:0034032	purine nucleoside bisphosphate metabolic process	9/607	110/28814	3.88	0.000542026	0.008489369	0.006147013	Acot1/Acot11/Coasy/Dcakd/Dip2a/Enpp1/Hmgcs1/Nudt7/Pdk2	9
BP	GO:0030100	regulation of endocytosis	14/607	237/28814	2.8	0.000547014	0.008523826	0.006171963	Anxa2/B2m/Cd151/Clu/Dnm2/Eef2k/Hap1/Pacsin1/Picalm/Plcg2/Ptpn5/Rab31/Sh3	14
BP	GO:0007009	plasma membrane organization	11/607	158/28814	3.3	0.000547795	0.008523826	0.006171963	Ano3/Chmp5/Clu/Dmkn/Dnm2/P2rx7/Pacsin1/Rab3a/Sod1/Xkr5/Xkr9	11
BP	GO:0071526	semaphorin-plexin signaling pathway	6/607	49/28814	5.81	0.000552413	0.008567777	0.006203787	Flna/Mef2c/Nrp2/Plxnc1/Rac1/Sema7a	6
BP	GO:0032411	positive regulation of transporter activity	10/607	134/28814	3.54	0.000563774	0.00868758	0.006290534	Actn2/Dnm2/Hap1/Hspa2/Kcnc1/Nlgn3/Plcg2/Rgs9/Slc6a9/Stim1	10

BP	GO:0009152	purine ribonucleotide biosynthetic process	7/361	151/28814	3.7	0.003026963	0.042925127	0.033384768	Ampd3/Coasy/Gcdh/Pdk1/Pdk3/Ppat/Stat3	7
BP	GO:0009749	response to glucose	8/361	192/28814	3.33	0.003028352	0.042925127	0.033384768	Glul/Nnmt/Pck2/Pdk3/Ppp3ca/Prkaa1/Rab11b/Smad4	8
BP	GO:0030100	regulation of endocytosis	9/361	237/28814	3.03	0.003172572	0.044795739	0.034839626	Gsg1l/Numb/Ppp3ca/Ppp3cc/Ptpn3/Rabgef1/Sh3gl2/Sh3gl3/Slc17a7	9
BP	GO:0043254	regulation of protein-containing complex assembly	13/361	429/28814	2.42	0.003202263	0.045041064	0.035030426	Abca1/Add2/Arda/Arpc5l/Cdk/Dlg1/Napa/Nkfn/Preb/Stx1a/Tmod1/Tppp/Vasp	13
BP	GO:0030002	cellular anion homeostasis	3/361	24/28814	9.98	0.00324697	0.045148933	0.035114321	Ckb/Slc12a2/Xpr1	3
BP	GO:0098901	regulation of cardiac muscle cell action potential	3/361	24/28814	9.98	0.00324697	0.045148933	0.035114321	Atp2a2/Camk2d/Dlg1	3
BP	GO:1990776	response to angiotensin	3/361	24/28814	9.98	0.00324697	0.045148933	0.035114321	Camk2a/Rock2/Slc26a6	3
BP	GO:0019228	neuronal action potential	4/361	49/28814	6.52	0.003292859	0.04527062	0.035208962	Ank3/Kcna1/Kcna2/Scn8a	4
BP	GO:0033173	calcineurin-NFAT signaling cascade	4/361	49/28814	6.52	0.003292859	0.04527062	0.035208962	Homer2/Ppp3ca/Ppp3cc/Ppp3r1	4
BP	GO:1990573	potassium ion import across plasma membrane	4/361	49/28814	6.52	0.003292859	0.04527062	0.035208962	Atp1a1/Atp1a4/Dlg1/Slc12a2	4
BP	GO:0016125	sterol metabolic process	7/361	154/28814	3.63	0.003375105	0.045963656	0.035747967	Abca1/Cat/Cyb5r1/Dhcr7/Mvd/Mvk/Prkaa1	7
BP	GO:0046434	organophosphate catabolic process	7/361	154/28814	3.63	0.003375105	0.045963656	0.035747967	Abhd6/Ampd3/Inpp5b/Nt5m/Nudt3/Nudt4/Pcbi	7
BP	GO:0019216	regulation of lipid metabolic process	12/361	382/28814	2.51	0.00341683	0.045963656	0.035747967	Abhd6/Atp1a1/Clcn2/Dhcr7/Lamtor1/Mboat7/Pdk1/Pdk3/Pip4k2b/Prkaa1/Sorbs1/Zmpste24	12
BP	GO:0030038	contractile actin filament bundle assembly	6/361	116/28814	4.13	0.003470676	0.045963656	0.035747967	Cd47/Pxn/Rock2/Sorbs1/Sorbs3/Tjp1	6
BP	GO:0043149	stress fiber assembly	6/361	116/28814	4.13	0.003470676	0.045963656	0.035747967	Cd47/Pxn/Rock2/Sorbs1/Sorbs3/Tjp1	6
BP	GO:0098659	inorganic cation import across plasma membrane	6/361	116/28814	4.13	0.003470676	0.045963656	0.035747967	Atp1a1/Atp1a4/Dlg1/Slc12a2/Slc24a2/Slc8a1	6
BP	GO:0099587	inorganic ion import across plasma membrane	6/361	116/28814	4.13	0.003470676	0.045963656	0.035747967	Atp1a1/Atp1a4/Dlg1/Slc12a2/Slc24a2/Slc8a1	6
BP	GO:0006913	nucleocytoplasmic transport	11/361	334/28814	2.63	0.003494618	0.045963656	0.035747967	Adam10/Alkbh5/Alyref/Ipo8/Nup188/Ppp3ca/Ppp3r1/Rae1/Ranbp6/Sec13/Stat3	11
BP	GO:0051169	nuclear transport	11/361	334/28814	2.63	0.003494618	0.045963656	0.035747967	Adam10/Alkbh5/Alyref/Ipo8/Nup188/Ppp3ca/Ppp3r1/Rae1/Ranbp6/Sec13/Stat3	11
BP	GO:0043279	response to alkaloid	5/361	81/28814	4.93	0.003533504	0.045963656	0.035747967	Bdnf/Homer2/Ppp1r9b/Prkaa1/Slc8a1	5
BP	GO:1900542	regulation of purine nucleotide metabolic process	5/361	81/28814	4.93	0.003533504	0.045963656	0.035747967	Pdk1/Pdk3/Prkaa1/Slc4a1/Stat3	5
BP	GO:0008016	regulation of heart contraction	8/361	197/28814	3.24	0.003539888	0.045963656	0.035747967	Atp1a1/Atp2a2/Calm1/Dlg1/S100a1/Scn4b/Slc8a1/Zmpste24	8
BP	GO:0015931	nucleobase-containing compound transport	8/361	197/28814	3.24	0.003539888	0.045963656	0.035747967	Ahctf1/Alkbh5/Alyref/Cd47/Eprs/Nup188/Rae1/Sec13	8
BP	GO:0003254	regulation of membrane depolarization	4/361	50/28814	6.39	0.003544367	0.045963656	0.035747967	Ank3/Camk2d/Cdk/Smap	4
BP	GO:0031146	SCF-dependent proteasomal ubiquitin-dependent protein catabolic process	4/361	50/28814	6.39	0.003544367	0.045963656	0.035747967	Cul2/Fbxl16/Fbxo2/Skp1	4
BP	GO:0090659	walking behavior	4/361	50/28814	6.39	0.003544367	0.045963656	0.035747967	Cend1/Cwr1/Scn8a/Zmpste24	4
BP	GO:0006109	regulation of carbohydrate metabolic process	8/361	198/28814	3.22	0.003649776	0.04690963	0.036483692	Arpp19/Pdk1/Pdk3/Prkaa1/Slc4a1/Sorbs1/Stat3/Zmpste24	8
BP	GO:0014808	release of sequestered calcium ion into cytosol by sarcoplasmic reticulum	3/361	25/28814	9.58	0.003655796	0.04690963	0.036483692	Calm1/Camk2d/Slc8a1	3
BP	GO:1902683	regulation of receptor localization to synapse	3/361	25/28814	9.58	0.003655796	0.04690963	0.036483692	Adam10/Camk2a/Cnih2	3
BP	GO:0006091	generation of precursor metabolites and energy	13/361	436/28814	2.38	0.003668904	0.046913228	0.036486491	Bdnf/Cat/Chchd2/Coq7/Cox7a1/Gys1/Ndor1/Ndufa12/Oxa1l/Prkaa1/Slc4a1/Sorbs1/Stat3	13
BP	GO:0006140	regulation of nucleotide metabolic process	5/361	82/28814	4.87	0.003725067	0.047300595	0.036787763	Pdk1/Pdk3/Prkaa1/Slc4a1/Stat3	5
BP	GO:0045123	cellular extravasation	5/361	82/28814	4.87	0.003725067	0.047300595	0.036787763	Add2/Cd47/Cd99/Plcb1/Stpa	5
BP	GO:0036293	response to decreased oxygen levels	9/361	243/28814	2.96	0.003738572	0.047307811	0.036793376	Alkbh5/Camk2d/Cat/Chchd2/Pdk1/Pdk3/Rock2/Slc8a1/Smad4	9
BP	GO:0035637	multicellular organismal signaling	7/361	157/28814	3.56	0.00375297	0.047326243	0.036807711	Ank3/Kcna1/Kcna2/Scn4b/Scn8a/Slc8a1/Zmpste24	7
BP	GO:0051028	mRNA transport	6/361	118/28814	4.06	0.003775226	0.047443309	0.036898758	Ahctf1/Alkbh5/Alyref/Nup188/Rae1/Sec13	6
BP	GO:0045332	phospholipid translocation	4/361	51/28814	6.26	0.003808609	0.047524217	0.036961684	Abca1/Atp8a2/Atp9a/Slc4a1	4
BP	GO:0042692	muscle cell differentiation	13/361	438/28814	2.37	0.00381191	0.047524217	0.036961684	Atp2a2/Bdnf/Camk2d/Cdk9/Csrp1/Ehd2/Fosl1/Mrtfa/Ppp3ca/Slc8a1/Smad4/Tmod1/Zmpste24	13
BP	GO:0008202	steroid metabolic process	11/361	338/28814	2.6	0.003820651	0.047524217	0.036961684	Abca1/Acaa1a/Atp1a1/Cat/Clcn2/Cyb5r1/Dhcr7/Lamtor1/Mvd/Mvk/Prkaa1	11
BP	GO:1902903	regulation of supramolecular fiber organization	12/361	388/28814	2.47	0.003866986	0.047896326	0.03725109	Add2/Arpc5l/Cd47/Dlg1/Map1a/Pxn/Rock2/Smad4/Sorbs3/Tjp1/Tmod1/Vasp	12
BP	GO:0007009	plasma membrane organization	7/361	158/28814	3.54	0.003885803	0.047896326	0.03725109	Ank3/Atp2a2/Ehd2/Ndrg1/Sh3gl2/Slc4a1/Stx2	7
BP	GO:1903829	positive regulation of protein localization	14/361	491/28814	2.28	0.003889857	0.047896326	0.03725109	Ank3/Apbb1/Cacnb3/Dlg1/Epb41l2/Frmd4a/Glul/Map1a/Nrxn1/Pcsk1/Ppp1r9b/Prkaa1/Rock2/Sorbs1	14
BP	GO:0006090	pyruvate metabolic process	6/361	119/28814	4.02	0.003934724	0.048286193	0.037554306	Pck2/Pdk1/Pdk3/Prkaa1/Slc4a1/Stat3	6
BP	GO:0060401	cytosolic calcium ion transport	8/361	201/28814	3.18	0.003995336	0.048866028	0.03800527	Atp2a2/Atp2b1/Cacnb3/Calm1/Camk2d/Sph3/Slc24a2/Slc8a1	8
BP	GO:0051954	positive regulation of amine transport	4/361	52/28814	6.14	0.00408588	0.049095468	0.038183715	Cck/Ptgs4/Slc12a2/Stx1a	4
BP	GO:0060428	lung epithelium development	4/361	52/28814	6.14	0.00408588	0.049095468	0.038183715	Foxp1/Foxp4/Numb/Ppp3r1	4
BP	GO:0035384	thioester biosynthetic process	3/361	26/28814	9.21	0.004094645	0.049095468	0.038183715	Gcdh/Pdk1/Pdk3	3
BP	GO:0071616	acyl-CoA biosynthetic process	3/361	26/28814	9.21	0.004094645	0.049095468	0.038183715	Gcdh/Pdk1/Pdk3	3
BP	GO:1902473	regulation of protein localization to synapse	3/361	26/28814	9.21	0.004094645	0.049095468	0.038183715	Adam10/Camk2a/Dlg1	3
BP	GO:1903514	release of sequestered calcium ion into cytosol by endoplasmic reticulum	3/361	26/28814	9.21	0.004094645	0.049095468	0.038183715	Calm1/Camk2d/Slc8a1	3
BP	GO:0008088	axo-dendritic transport	5/361	84/28814	4.75	0.004130332	0.049200726	0.038265579	Cnih2/Kif1b/Map1a/Nefm/Stau1	5
BP	GO:2001259	positive regulation of cation channel activity	5/361	84/28814	4.75	0.004130332	0.049200726	0.038265579	Ank3/Cacnb3/Calm1/Kcna1/S100a1	5
BP	GO:0051963	regulation of synapse assembly	6/361	121/28814	3.96	0.004268617	0.050461186	0.039245895	Bdnf/Mif/Ngp2/Nptx1/Nrxn1/Sema4a	6
BP	GO:0032535	regulation of cellular component size	12/361	393/28814	2.44	0.004277541	0.050461186	0.039245895	Add2/Arpc5l/Atp2b2/Bdnf/Ehd2/Map1a/Nefm/Pxn/Rock2/Sema4a/Slc12a2/Tmod1/Vasp	12

MF	GO:0015605	organophosphate ester transmembrane transporter activity	3/254	28/28275	11.93	0.001987795	0.044617728	0.03573696	Slc25a4/Slc25a42/Slc25a5	3
MF	GO:0140142	nucleocytoplasmic carrier activity	3/254	28/28275	11.93	0.001987795	0.044617728	0.03573696	Kpna4/Kpna6/Xpo6	3
MF	GO:0003729	mRNA binding	10/254	372/28275	2.99	0.002094862	0.044617728	0.03573696	Cpeb3/Dhfr/Marf1/Rbfox1/Rpl7/Rps26/Tia1/Ybx1/Ythdf2	10
MF	GO:0051117	ATPase binding	5/254	100/28275	5.57	0.002116591	0.044617728	0.03573696	Atp1b1/Dnajc10/Psen1/Snx10/Wls	5
MF	GO:0015298	solute:cation antiporter activity	3/254	29/28275	11.52	0.00220255	0.044644002	0.035758005	Slc8a2/Slc8a3/Slc9a1	3
MF	GO:0005251	delayed rectifier potassium channel activity	3/254	30/28275	11.13	0.002431164	0.04590302	0.036796287	Kcnc1/Kcnc3/Kcnh2	3
MF	GO:0019001	guanyl nucleotide binding	10/254	382/28275	2.91	0.002536239	0.04590302	0.036796287	Dnm1/Drg1/Gfm2/Gnpb/Gpn1/Guf1/Rnf112/Tgm2/Tubg1/Uprt	10
MF	GO:0032561	guanyl ribonucleotide binding	10/254	382/28275	2.91	0.002536239	0.04590302	0.036796287	Dnm1/Drg1/Gfm2/Gnpb/Gpn1/Guf1/Rnf112/Tgm2/Tubg1/Uprt	10
MF	GO:0051020	GTPase binding	9/254	321/28275	3.12	0.002615197	0.04590302	0.036796287	Iqgap1/Iqgap2/Rabgef1/Ranbp9/Rock1/Sh3bp4/Wasl/Xpo6/Ybx1	9
MF	GO:0016922	nuclear receptor binding	6/254	155/28275	4.31	0.002842762	0.048272108	0.038663968	Grip1/Grl2b/Jund/Mms19/Taco2/Ywhah	6
MF	GO:0030371	translation repressor activity	3/254	32/28275	10.44	0.002931134	0.048272108	0.038663968	Cpeb3/Dhfr/Marf1	3
MF	GO:0015079	potassium ion transmembrane transporter activity	6/254	157/28275	4.25	0.003028897	0.04837057	0.038742832	Abcc9/Atp1b1/Kcnc1/Kcnc3/Kcnh2/Slc9a1	6
MF	GO:0004382	guanosine-diphosphatase activity	2/254	10/28275	22.26	0.003449237	0.048399778	0.038766226	Entpd1/Entpd3	2
MF	GO:0008504	monoamine transmembrane transporter activity	2/254	10/28275	22.26	0.003449237	0.048399778	0.038766226	Slc18a2/Slc6a3	2
MF	GO:0015368	calcium:cation antiporter activity	2/254	10/28275	22.26	0.003449237	0.048399778	0.038766226	Slc8a2/Slc8a3	2
MF	GO:0045134	uridine-diphosphatase activity	2/254	10/28275	22.26	0.003449237	0.048399778	0.038766226	Entpd1/Entpd3	2
MF	GO:0016251	RNA polymerase II general transcription initiation factor activity	3/254	34/28275	9.82	0.003489927	0.048399778	0.038766226	Gtf2a2/Gtf2b/Gtf2f1	3
MF	GO:0015035	protein-disulfide reductase activity	3/254	35/28275	9.54	0.00379203	0.051241019	0.041041943	Dnajc10/Glrx3/Pdia4	3
MF	GO:0043022	ribosome binding	4/254	72/28275	6.18	0.004056361	0.053442562	0.042805288	Cpeb3/Eif5a2/Guf1/Pym1	4
MF	GO:0015378	sodium:chloride symporter activity	2/254	11/28275	20.24	0.004190829	0.05386748	0.04314563	Slc18a2/Slc6a3	2
MF	GO:0031267	small GTPase binding	8/254	285/28275	3.12	0.004423612	0.05445074	0.043612797	Iqgap1/Iqgap2/Rabgef1/Ranbp9/Rock1/Sh3bp4/Wasl/Xpo6	8
MF	GO:0017080	sodium channel regulator activity	3/254	37/28275	9.03	0.00444285	0.05445074	0.043612797	Fgf12/Scn3b/Ywhah	3
MF	GO:1901338	catecholamine binding	2/254	12/28275	18.55	0.004999307	0.059878059	0.047959855	Slc6a3/Th	2
MF	GO:0140223	general transcription initiation factor activity	3/254	39/28275	8.56	0.005157307	0.060397791	0.04837614	Gtf2a2/Gtf2b/Gtf2f1	3
MF	GO:0003924	GTPase activity	8/254	294/28275	3.03	0.005315229	0.060894041	0.048773615	Dnm1/Drg1/Gfm2/Gnb4/Gpn1/Guf1/Rnf112/Tubg1	8
MF	GO:0000900	mRNA regulatory element binding translation repressor activity	2/254	13/28275	17.13	0.005873419	0.063169217	0.050595938	Cpeb3/Dhfr	2
MF	GO:0008097	5S rRNA binding	2/254	13/28275	17.13	0.005873419	0.063169217	0.050595938	Nop53/Rpl7	2
MF	GO:0015373	anion:sodium symporter activity	2/254	13/28275	17.13	0.005873419	0.063169217	0.050595938	Slc18a2/Slc6a3	2
MF	GO:0015036	disulfide oxidoreductase activity	3/254	42/28275	7.95	0.006351867	0.066948674	0.053623126	Dnajc10/Glrx3/Pdia4	3
MF	GO:0005216	ion channel activity	10/254	438/28275	2.54	0.006568129	0.067859624	0.054352759	Abcc9/Cacng8/Kcnc1/Kcnc3/Kcnh2/Lrrc8b/Lrrc8d/Psen1/Scn3b/Ttyh2	10
MF	GO:0008514	organic anion transmembrane transporter activity	6/254	185/28275	3.61	0.006695826	0.067859624	0.054352759	Slc25a15/Slc25a21/Slc25a4/Slc25a42/Slc25a5/Slc38a1	6
MF	GO:0099106	ion channel regulator activity	5/254	136/28275	4.09	0.007808605	0.077644049	0.062189681	Abcc9/Cacng8/Fgf12/Scn3b/Ywhah	5
MF	GO:0005048	signal sequence binding	3/254	46/28275	7.26	0.008181439	0.079844788	0.063952382	Ap2m1/Kpna4/Kpna6	3
MF	GO:0008276	protein methyltransferase activity	4/254	89/28275	5	0.008554976	0.081972223	0.065656369	Fam98b/Icmt/Nbmt1/Rbbp5	4
MF	GO:0016746	acyltransferase activity	7/254	257/28275	3.03	0.008857448	0.082076025	0.06573951	Agpat5/Cpt1a/Cs/Gcat/Glt2b/Pafah1b2/Tgm2	7
MF	GO:0005092	GDP-dissociation inhibitor activity	2/254	16/28275	13.91	0.008877293	0.082076025	0.06573951	Ranbp1/Sh3bp4	2
MF	GO:0015932	nucleobase-containing compound transmembrane transporter activity	3/254	48/28275	6.96	0.009200484	0.08359755	0.066958154	Slc25a4/Slc25a42/Slc25a5	3
MF	GO:0016247	channel regulator activity	5/254	144/28275	3.87	0.009854128	0.08640863	0.069209748	Abcc9/Cacng8/Fgf12/Scn3b/Ywhah	5
MF	GO:0061608	nuclear import signal receptor activity	2/254	17/28275	13.1	0.010001758	0.08640863	0.069209748	Kpna4/Kpna6	2
MF	GO:1990380	Lys48-specific deubiquitinase activity	2/254	17/28275	13.1	0.010001758	0.08640863	0.069209748	Mindy4/Yod1	2
MF	GO:0015103	inorganic anion transmembrane transporter activity	5/254	146/28275	3.81	0.010417843	0.088551669	0.070926234	Lrrc8b/Lrrc8d/Slc18a2/Slc6a3/Ttyh2	5
MF	GO:0048306	calcium-dependent protein binding	4/254	95/28275	4.69	0.010700921	0.089514057	0.071697068	S100a11/Slc9a1/Stx2/Wfs1	4
MF	GO:0015377	cation:chloride symporter activity	2/254	18/28275	12.37	0.011185847	0.092108461	0.073775078	Slc18a2/Slc6a3	2
MF	GO:0030170	pyridoxal phosphate binding	3/254	52/28275	6.42	0.011452154	0.092250296	0.073888682	Cbs/Ddc/Gcat	3
MF	GO:0008017	microtubule binding	7/254	271/28275	2.88	0.011618982	0.092250296	0.073888682	Cnn3/Dnm1/Drg1/Mast1/Reep2/Rmdn2/Tubgcp2	7
MF	GO:0016790	thiolester hydrolase activity	3/254	53/28275	6.3	0.012060181	0.092250296	0.073888682	Acot2/Lypla1/Pnkd	3
MF	GO:0070279	vitamin B6 binding	3/254	53/28275	6.3	0.012060181	0.092250296	0.073888682	Cbs/Ddc/Gcat	3
MF	GO:0015267	channel activity	10/254	482/28275	2.31	0.012319518	0.092250296	0.073888682	Abcc9/Cacng8/Kcnc1/Kcnc3/Kcnh2/Lrrc8b/Lrrc8d/Psen1/Scn3b/Ttyh2	10
MF	GO:0022803	passive transmembrane transporter activity	10/254	482/28275	2.31	0.012319518	0.092250296	0.073888682	Abcc9/Cacng8/Kcnc1/Kcnc3/Kcnh2/Lrrc8b/Lrrc8d/Psen1/Scn3b/Ttyh2	10
MF	GO:0035259	nuclear glucocorticoid receptor binding	2/254	19/28275	11.72	0.012428408	0.092250296	0.073888682	Grip1/Ywhah	2
MF	GO:1901505	carbohydrate derivative transmembrane transporter activity	3/254	54/28275	6.18	0.012686432	0.092857637	0.074375137	Slc25a4/Slc25a42/Slc25a5	3
MF	GO:0043177	organic acid binding	5/254	156/28275	3.57	0.013569282	0.097959061	0.078461167	Dgah2/Ddc/Dhfr/P4ha1/Th	5

BP	GO:0035303	regulation of dephosphorylation	10/607	134/28814	3.54	0.000563774	0.00868758	0.006290534	Arpp19/Bod1/Ensa/Mef2c/Ppp1r16b/Ppp4r4/Ptpa/Rcan1/Smg5/Vrk3	10
BP	GO:0071482	cellular response to light stimulus	9/607	111/28814	3.85	0.000579046	0.008879797	0.006429716	Bax/Crebbp/Dhx36/Gnb5/Kdm1a/Mme/Pcna/Rgr9/Yy1	9
BP	GO:0014032	neural crest cell development	8/607	89/28814	4.27	0.000585608	0.008879797	0.006429716	Cdh2/Ednrb/Efnb1/Mapk3/Nrp2/Phactr4/Sema7a/Sox8	8
BP	GO:0043652	engulfment of apoptotic cell	4/607	19/28814	9.99	0.000587556	0.008879797	0.006429716	Rac1/Rac2/Xkr5/Xkr9	4
BP	GO:0090083	regulation of inclusion body assembly	4/607	19/28814	9.99	0.000587556	0.008879797	0.006429716	Clu/Dnajb6/Hap1/Hspa2	4
BP	GO:1900452	regulation of long-term synaptic depression	4/607	19/28814	9.99	0.000587556	0.008879797	0.006429716	Adcy8/Dgki/Gnal/Kcnb1	4
BP	GO:1905874	regulation of postsynaptic density organization	4/607	19/28814	9.99	0.000587556	0.008879797	0.006429716	Cdh2/Dbn1/Nptx1/Ntrk3	4
BP	GO:1903305	regulation of regulated secretory pathway	12/607	185/28814	3.08	0.00058926	0.008879797	0.006429716	Apba1/Apba2/Cdk5r2/Dgki/Dnd1/Kcnb1/Nrxn3/P2rx7/Pdcd6/Rab3a/Rac2/Syt17	12
BP	GO:0060541	respiratory system development	15/607	267/28814	2.67	0.000593877	0.008899554	0.006444021	Clcn2/Hmgb1/Hspb11/Igfbp5/Lipa/Map2k2/Mapk3/Pdpn/Pik3/Rab3a/Smpd3/Spar	15
BP	GO:0030323	respiratory tube development	14/607	239/28814	2.78	0.000594297	0.008899554	0.006444021	Clcn2/Hmgb1/Hspb11/Igfbp5/Lipa/Map2k2/Mapk3/Pdpn/Pik3/Rab3a/Smpd3/Spar	14
BP	GO:0009299	mRNA transcription	6/607	50/28814	5.7	0.000616699	0.009177481	0.006645264	Anxa2/Enpp1/Flna/Hltf/Ncbp2/Stat3	6
BP	GO:0035305	negative regulation of dephosphorylation	6/607	50/28814	5.7	0.000616699	0.009177481	0.006645264	Arpp19/Bod1/Ensa/Ppp1r16b/Ppp4r4/Ptpa	6
BP	GO:0010876	lipid localization	21/607	449/28814	2.22	0.000629348	0.009333463	0.006758208	Abca1/Abca8b/Abcd3/Anxa2/Anxa2/Cds1/Enpp1/Fabp7/Gramd1a/Hexb/Ncbp2/Osbpl8/P2rx7/Pitpnc1/Pla2g4a/Sar1b/Sgpp1/Sigmar1/Slc10	21
BP	GO:0006096	glycolytic process	8/607	90/28814	4.22	0.000631088	0.009333463	0.006758208	Eif6/Eno2/Enpp1/Hk2/P2rx7/Stat3/Tpi1/Zbtb20	8
BP	GO:0008277	regulation of G protein-coupled receptor signaling pathway	10/607	136/28814	3.49	0.000633152	0.009335084	0.006759381	Atp2b4/Camk2b/Dnm2/Grk5/Homer2/Pde10a/Pde4a/Rasd/Rnf157/Tab3	10
BP	GO:0007033	vacuole organization	12/607	187/28814	3.05	0.000648164	0.009527016	0.006898556	Abca1/Bag3/Chmp5/Elapor1/Hexb/Irgm1/Lamtor1/P2rx7/Rab14/Rab34/Tmem106	12
BP	GO:0048488	synaptic vesicle endocytosis	7/607	70/28814	4.75	0.000676578	0.009883835	0.007156723	Actb/Dnm2/Nlgn3/Pacsin1/Picalm/Sh3gl3/Snca	7
BP	GO:0140238	presynaptic endocytosis	7/607	70/28814	4.75	0.000676578	0.009883835	0.007156723	Actb/Dnm2/Nlgn3/Pacsin1/Picalm/Sh3gl3/Snca	7
BP	GO:0006757	ATP generation from ADP	8/607	91/28814	4.17	0.000679376	0.009894442	0.007164403	Eif6/Eno2/Enpp1/Hk2/P2rx7/Stat3/Tpi1/Zbtb20	8
BP	GO:0099068	postsynapse assembly	5/607	34/28814	6.98	0.000684926	0.009944964	0.007200985	Cdh2/Gap43/Nlgn3/Nptx1/Ntrk3	5
BP	GO:0006402	mRNA catabolic process	14/607	243/28814	2.73	0.000699332	0.010080596	0.007299194	Apex1/Cnot7/Dcp1a/Dcps/Dhx36/Exosc10/Hnrnpa0/Ncbp2/Noct/Nudt12/Paip1/S	14
BP	GO:0006813	potassium ion transport	14/607	243/28814	2.73	0.000699332	0.010080596	0.007299194	Actn2/Ccdc51/Dnm2/Flna/Hcn3/Kcna3/Kcnab3/Kcnb1/Kcnc1/Kcnd2/Kcnip2/Slc1	14
BP	GO:0009185	ribonucleoside diphosphate metabolic process	9/607	114/28814	3.75	0.000702708	0.010080596	0.007299194	Ak2/Eif6/Eno2/Enpp1/Hk2/P2rx7/Stat3/Tpi1/Zbtb20	9
BP	GO:0031532	actin cytoskeleton reorganization	9/607	114/28814	3.75	0.000702708	0.010080596	0.007299194	Flna/Gab1/Mcu/Ntrk3/Parvb/Pdlim4/Phactr1/Pip5k1a/Pik3	9
BP	GO:0009260	ribonucleotide biosynthetic process	11/607	163/28814	3.2	0.000710497	0.010131468	0.00733603	Adcy3/Adcy8/Ak2/Atp5pb/Coasy/Dcakd/Dip2a/Gucy1b1/Nme3/Pdk2/Stat3	11
BP	GO:0021543	pallium development	11/607	163/28814	3.2	0.000710497	0.010131468	0.00733603	Adgrg1/Bax/Bbs2/Ccdc85c/Cdh2/Cdk5r2/Dab2ip/Drd1/Fgf13/Phactr1/Rac1	11
BP	GO:0106070	regulation of adenylate cyclase-activating G protein-coupled receptor signaling pathway	4/607	20/28814	9.49	0.000722276	0.01026879	0.007435462	Atp2b4/Grk5/Pde10a/Pde4a	4
BP	GO:0060079	excitatory postsynaptic potential	8/607	92/28814	4.13	0.000730596	0.010356251	0.007498792	Celf4/Dbn1/Dgki/Grik5/Mef2c/Nlgn3/Prkar1b/Stx6	8
BP	GO:0017156	calcium-ion regulated exocytosis	7/607	71/28814	4.68	0.000737155	0.010417067	0.007543733	Cdk5r2/Kcnb1/P2rx7/Rab3a/Snap25/Syt17/Vamp3	7
BP	GO:0045646	regulation of erythrocyte differentiation	6/607	52/28814	5.48	0.000762554	0.010745495	0.007780637	B2m/Hmgb2/Kdm1a/Sesd1/Stat1/Stat3	6
BP	GO:0051648	vesicle localization	12/607	191/28814	2.98	0.000780853	0.010945919	0.00792576	Bloc1s6/Cdh2/Dapk2a/Hap1/Myo1b/Nlgn3/Picalm/Rab3a/Sar1b/Smpd3/Stam/Wipi1	12
BP	GO:0048814	regulation of dendrite morphogenesis	8/607	93/28814	4.08	0.000784878	0.010945919	0.00792576	Ache/Camk2b/Dbn1/Dbnl/Dhx36/Ezh2/Itpka/Marcks	8
BP	GO:0090162	establishment of epithelial cell polarity	5/607	35/28814	6.78	0.000785369	0.010945919	0.00792576	Frmd4a/Frmd4b/Mas/Pik3/Vangl2	5
BP	GO:0009060	aerobic respiration	11/607	165/28814	3.16	0.000785943	0.010945919	0.00792576	Atp5pb/Cox7a1/Cox7a2/Cox7c/Idh3a/Mdh2/Ndufa10/Ndufa12/Ndufb6/Sdhaf2/Slc2	11
BP	GO:0071222	cellular response to lipopolysaccharide	16/607	304/28814	2.5	0.00079016	0.011071913	0.008016991	Abca1/B2m/Chmp5/Dab2ip/Gbp2/Hmgb1/Irgm1/Mapk3/Mef2c/Pde4a/Plcg2/Sirpa	16
BP	GO:0045428	regulation of nitric oxide biosynthetic process	7/607	72/28814	4.62	0.000801943	0.011071913	0.008016991	Atp2b4/Clu/Dnm2/Dynll1/Rac1/Sirpa/Smad3	7
BP	GO:0048168	regulation of neuronal synaptic plasticity	7/607	72/28814	4.62	0.000801943	0.011071913	0.008016991	Camk2b/Rab3a/Rac1/Shisa7/Shisa9/Syap1/Syngr1	7
BP	GO:0032103	positive regulation of response to external stimulus	21/607	458/28814	2.18	0.000809705	0.011146864	0.008071261	Camk1d/Cdk19/Flna/Hmgb1/Hmgb2/Irgm1/Lgals1/Mapk3/Mcu/Ntrk3/Pdcd4/Pla2g4a/Pla2g7/Plcg2/Rab34/Rac1/Rac2/Stx3/Thbs4/Traf6/	21
BP	GO:0034249	negative regulation of cellular amide metabolic process	13/607	219/28814	2.82	0.000814684	0.011183183	0.00809756	Clu/Cnot7/Dcp1a/Dcps/Dhx36/Eif6/Hap1/Igfbp5/Mrpl13/Noct/Paip1/Tia1/Tut4	13
BP	GO:0034250	positive regulation of cellular amide metabolic process	11/607	166/28814	3.15	0.000826088	0.01130723	0.00818738	Clu/Coa3/Dhx36/Eif6/Mapk3/Paip1/Picalm/Pld1/Rps9/Smpd3/Vim	11
BP	GO:0050806	positive regulation of synaptic transmission	17/607	335/28814	2.41	0.000833403	0.011374755	0.008236274	Adcy8/Camk2b/Drd1/Enpp1/Gfap/Hap1/Mme/Nlgn3/Nrgn/Prkar1b/Prrt1/Ptpn5/Rab3a/Shisa7/Slc1a3/Snap25/	17
BP	GO:0022904	respiratory electron transport chain	8/607	94/28814	4.04	0.000842355	0.011431613	0.008277443	Cox7c/Etfrf1/Ndufa10/Ndufa12/Ndufb6/Sdhaf2/Slc1a3/Slc25a18	8
BP	GO:0035304	regulation of protein dephosphorylation	8/607	94/28814	4.04	0.000842355	0.011431613	0.008277443	Arpp19/Bod1/Ensa/Ppp1r16b/Ppp4r4/Ptpa/Rcan1/Vrk3	8
BP	GO:0006939	smooth muscle contraction	9/607	117/28814	3.65	0.000847114	0.011432542	0.008278116	Atp2b1/Atp2b4/Bbs2/Drd1/Ednrb/Ptgs1/Setd3/Smpd3/Sod1	9
BP	GO:0048639	positive regulation of developmental growth	13/607	220/28814	2.81	0.000849603	0.011432542	0.008278116	Bbs2/Dbn1/Dbnl/Map3k13/Mef2c/Naca/Ntrk3/Picalm/Pls1/Rnf157/Sema7a/Slc6a3/	13
BP	GO:1901293	nucleoside phosphate biosynthetic process	13/607	220/28814	2.81	0.000849603	0.011432542	0.008278116	Adcy3/Adcy8/Ak2/Ak5/Atp5pb/Coasy/Dcakd/Dip2a/Gucy1b1/Naprt/Nme3/Pdk2/Stat3	13
BP	GO:0001936	regulation of endothelial cell proliferation	11/607	167/28814	3.13	0.000867918	0.01161357	0.008409196	Hmgb1/Hmgb2/Jcad/Mef2c/Mydgf/Nras/Ppp1r16b/Sparc/Stat1/Stat3/Thbs4	11
BP	GO:0009267	cellular response to starvation	11/607	167/28814	3.13	0.000867918	0.01161357	0.008409196	Brsk1/Comt/Dap/Elapor1/Foxo1/Kptn/Lamp2/Mapk3/Prkag1/Rragb/Wipi1	11
BP	GO:0045652	regulation of megakaryocyte differentiation	4/607	21/28814	9.04	0.000877452	0.011663857	0.008445608	Eif6/Hmgb2/Kdm1a/Mef2c	4
BP	GO:0098962	regulation of postsynaptic neurotransmitter receptor activity	4/607	21/28814	9.04	0.000877452	0.011663857	0.008445608	Dlgap4/Nptx1/Shisa7/Shisa9	4
BP	GO:0001558	regulation of cell growth	21/607	461/28814	2.16	0.000879001	0.011663857	0.008445608	Bap1/Dab2ip/Dbn1/Dbnl/Dip2b/Dnm2/Dnph1/Enpp1/Fgf13/Gng4/Igfbp4/Igfbp5/Lamtor1/Map3k13/Ntrk3/Picalm/Rnf157/Sema7a/Slc25a	21
BP	GO:0031644	regulation of nervous system process	12/607	194/28814	2.94	0.00089464	0.011829014	0.008565195	Celf4/Comt/Dbn1/Dlgap4/Ednrb/Grm1/Nlgn3/Nptx1/Prkar1b/Shisa7/Shisa9/Tmem100	12
BP	GO:0014741	negative regulation of muscle hypertrophy	5/607	36/28814	6.59	0.0008964	0.011829014	0.008565195	Atp2b4/Foxo1/Igfbp5/Lmna/Yy1	5
BP	GO:0014031	mesenchymal cell development	8/607	95/28814	4	0.00090316	0.011852738	0.008582373	Cdh2/Ednrb/Efnb1/Mapk3/Nrp2/Phactr4/Sema7a/Sox8	8
BP	GO:0048864	stem cell development	8/607	95/28814	4	0.00090316	0.011852738	0.008582373	Cdh2/Ednrb/Efnb1/Mapk3/Nrp2/Phactr4/Sema7a/Sox8	8
BP	GO:0006164	purine nucleotide biosynthetic process	11/607	168/28814	3.11	0.000911486	0.011896642	0.008614163	Adcy3/Adcy8/Ak2/Atp5pb/Coasy/Dcakd/Dip2a/Gucy1b1/Nme3/Pdk2/Stat3	11
BP	GO:0046474	glycerophospholipid biosynthetic process	11/607	168/28814	3.11	0.000911486	0.011896642	0.008614163	Abhd4/Cds1/Dgki/Hdhd5/Itpka/Pcyt1b/Pigt/Pip5k1a/Pla2g4a/Plcg2/Pld1	11
BP	GO:1903311	regulation of mRNA metabolic process	16/607	308/28814	2.47	0.000917143	0.011937857	0.008644007	Apex1/Celf4/Cnot7/Dcp1a/Dcps/Dhx36/Hnrnpa0/Larp7/Ncbp2/Noct/Paip1/Raver1	16
BP	GO:0031103	axon regeneration	6/607	54/28814	5.27	0.000933703	0.012120383	0.00877617	Enpp1/Flna/Gap43/Map2k2/Ntrk3/Rtn4rl2	6
BP	GO:0006914	autophagy	21/607	464/28814	2.15	0.000953352	0.012254331	0.00887316	Bag3/Chmp5/Clu/Dap/Elapor1/Endog/Foxo1/Fundc1/Fundc2/Gfap/Gramd1a/Hap1/Hk2/Hmgb1/Irgm1/Lamp2/Mapk3/Rragb/Slc25a5/Stat	21
BP	GO:0061919	process utilizing autophagic mechanism	21/607	464/28814	2.15	0.000953352	0.012254331	0.00887316	Bag3/Chmp5/Clu/Dap/Elapor1/Endog/Foxo1/Fundc1/Fundc2/Gfap/Gramd1a/Hap1/Hk2/Hmgb1/Irgm1/Lamp2/Mapk3/Rragb/Slc25a5/Stat	21
BP	GO:0030041	actin filament polymerization	11/607	169/28814	3.09	0.000956849	0.012254331	0.00887316	Arpc1b/Arpc3/Arpc5l/Cracd/Dbn1/Dbnl/Kank4/Rac1/Ssh3/Tmod1/Vasp	11
BP	GO:0051100	negative regulation of binding	11/607	169/28814	3.09	0.000956849	0.012254331	0.00887316	Actb/B2m/Bax/Camk1/Ckmt1/Gtpbp4/Kdm1a/Larp7/Mapk3/Rap1gap/Tnks	11
BP	GO:0071248	cellular response to metal ion	11/607	169/28814	3.09	0.000956849	0.012254331	0.00887316	Adcy8/B2m/Cpne3/Kcnb1/Mapk3/Mef2c/Nptx1/Plcg2/Rasgrp2/Smpd3/Syt17	11

BP	GO:0034470	ncRNA processing	12/361	393/28814	2.44	0.004277541	0.050461186	0.039245895	Dus3l/Elp3/Fbll1/Ints3/Ppp3ca/Prkra/Snu13/Stat3/Tut7/Urm1/Wdr6/Zmpste24	12
BP	GO:0034204	lipid translocation	4/361	53/28814	6.02	0.004376475	0.051297342	0.03989621	Abca1/Atp8a2/Atp9a/Slc4a1	4
BP	GO:0097720	calcineurin-mediated signaling	4/361	53/28814	6.02	0.004376475	0.051297342	0.03989621	Homer2/Ppp3ca/Ppp3cc/Ppp3r1	4
BP	GO:0006820	anion transport	14/361	498/28814	2.24	0.004400054	0.051408936	0.039983001	Bdnf/Cck/Cd47/Clcn2/Gabra3/Gabrb1/Rab11b/Slc12a2/Slc17a7/Slc25a10/Slc25a14/Slc26a6/Slc4a1/Xpr1	14
BP	GO:0046928	regulation of neurotransmitter secretion	6/361	122/28814	3.93	0.004443194	0.051747646	0.040246432	Atp2a2/Camk2a/Cplx1/Cplx3/Rims2/Stx1a	6
BP	GO:0015740	C4-dicarboxylate transport	3/361	27/28814	8.87	0.004564154	0.052987649	0.041210837	Slc12a2/Slc25a10/Slc25a14	3
BP	GO:0021543	pallium development	7/361	163/28814	3.43	0.004604228	0.053283735	0.041441116	Adgrg1/Kcna1/Kirrel3/Mboat7/Pex13/Plcb1/Trappc9	7
BP	GO:0009161	ribonucleoside monophosphate metabolic process	4/361	54/28814	5.91	0.004680683	0.053827853	0.0418643	Ampd3/Ppat/Prps1/Uprt	4
BP	GO:0098926	postsynaptic signal transduction	4/361	54/28814	5.91	0.004680683	0.053827853	0.0418643	Plcb1/Prr7/Sorbs3/Stat3	4
BP	GO:0006937	regulation of muscle contraction	7/361	164/28814	3.41	0.004759182	0.05459029	0.042432968	Atp1a1/Atp2a2/Atp2b1/Calm1/Dlg1/Kcna1/Slc8a1	7
BP	GO:0032410	negative regulation of transporter activity	5/361	87/28814	4.59	0.004795858	0.054775987	0.042601702	Ank3/Cacnb3/Calm1/Camk2d/Cbarp	5
BP	GO:0015748	organophosphate ester transport	6/361	124/28814	3.86	0.004808064	0.054775987	0.042601702	Abca1/Atp8a2/Atp9a/Cd47/Epo5/Slc4a1	6
BP	GO:0042326	negative regulation of phosphorylation	12/361	400/28814	2.39	0.004910326	0.055456286	0.043130805	Aida/Coro1c/Mvp/Pcp4/Pip4k2c/Ppp4c/Rabgef1/Sh3gl2/Sirpa/Slc4a1/Slc8a1/Stat3	12
BP	GO:0009060	aerobic respiration	7/361	165/28814	3.39	0.004918034	0.055456286	0.043130805	Bdnf/Cat/Chchd2/Coq7/Cox7a1/Ndufa12/Oxa1l	7
BP	GO:0010675	regulation of cellular carbohydrate metabolic process	7/361	165/28814	3.39	0.004918034	0.055456286	0.043130805	Arpp19/Pdk1/Pdk3/Slc4a1/Sorbs1/Stat3/Zmpste24	7
BP	GO:0051261	protein depolymerization	6/361	125/28814	3.83	0.004998539	0.055456286	0.043130805	Add2/Ccsap/Map1a/Sh3gl1/Sh3gl2/Tmod3	6
BP	GO:0006637	acyl-CoA metabolic process	5/361	88/28814	4.54	0.005033673	0.055456286	0.043130805	Gcdh/Mvd/Mvk/Pdk1/Pdk3	5
BP	GO:0006942	regulation of striated muscle contraction	5/361	88/28814	4.54	0.005033673	0.055456286	0.043130805	Atp1a1/Atp2a2/Calm1/Dlg1/Slc8a1	5
BP	GO:0035383	thioester metabolic process	5/361	88/28814	4.54	0.005033673	0.055456286	0.043130805	Gcdh/Mvd/Mvk/Pdk1/Pdk3	5
BP	GO:0061484	hematopoietic stem cell homeostasis	3/361	28/28814	8.55	0.005064917	0.055456286	0.043130805	Adgrg1/Cadps2/Septin4	3
BP	GO:0070050	neuron cellular homeostasis	3/361	28/28814	8.55	0.005064917	0.055456286	0.043130805	Map1a/Prkaa1/Slc17a7	3
BP	GO:0098698	postsynaptic specialization assembly	3/361	28/28814	8.55	0.005064917	0.055456286	0.043130805	Nptx1/Nrxn1/Numb	3
BP	GO:0098884	postsynaptic neurotransmitter receptor internalization	3/361	28/28814	8.55	0.005064917	0.055456286	0.043130805	Gsg1l/Numb/Ppp3r1	3
BP	GO:0140239	postsynaptic endocytosis	3/361	28/28814	8.55	0.005064917	0.055456286	0.043130805	Gsg1l/Numb/Ppp3r1	3
BP	GO:2000178	negative regulation of neural precursor cell proliferation	3/361	28/28814	8.55	0.005064917	0.055456286	0.043130805	Bdnf/Cend1/Lims1	3
BP	GO:0032414	positive regulation of ion transmembrane transporter activity	6/361	126/28814	3.8	0.005194495	0.056705279	0.044102201	Ank3/Cacnb3/Calm1/Kcna1/Plcb1/S100a1	6
BP	GO:1901019	regulation of calcium ion transmembrane transporter activity	5/361	89/28814	4.48	0.005279726	0.057464159	0.044692416	Cacnb3/Calm1/Cbarp/Jph3/S100a1	5
BP	GO:0048016	inositol phosphate-mediated signaling	4/361	56/28814	5.7	0.005331066	0.057850766	0.044993097	Homer2/Ppp3ca/Ppp3cc/Ppp3r1	4
BP	GO:0055067	monovalent inorganic cation homeostasis	7/361	168/28814	3.33	0.005418577	0.058626442	0.045596375	Atp1a1/Atp1a4/Camk2d/Slc12a2/Slc26a6/Slc4a1/Slc8a1	7
BP	GO:0021537	telencephalon development	9/361	258/28814	2.78	0.005532035	0.059353812	0.046162082	Adgrg1/Kcna1/Kirrel3/Mboat7/Numb/Pex13/Plcb1/Rtn4rl1/Trappc9	9
BP	GO:1901379	regulation of potassium ion transmembrane transport	5/361	90/28814	4.43	0.005534164	0.059524819	0.046295083	Ank3/Dlg1/Kcna1/Kcnab2/Kcnip3	5
BP	GO:0006694	steroid biosynthetic process	7/361	169/28814	3.31	0.005593623	0.059679391	0.0464153	Atp1a1/Clcn2/Cyb5r1/Dhcr7/Mvd/Mvk/Prkaa1	7
BP	GO:0071248	cellular response to metal ion	7/361	169/28814	3.31	0.005593623	0.059679391	0.0464153	Ank3/Cpne8/Kcna1/Nptx1/Nrxn1/Prkaa1/Slc12a2	7
BP	GO:1904861	excitatory synapse assembly	3/361	29/28814	8.26	0.005597493	0.059679391	0.0464153	Nptx1/Nrxn1/Sema4a	3
BP	GO:0006695	cholesterol biosynthetic process	4/361	57/28814	5.6	0.005677795	0.060010684	0.046672961	Dhcr7/Mvd/Mvk/Prkaa1	4
BP	GO:1901607	alpha-amino acid biosynthetic process	4/361	57/28814	5.6	0.005677795	0.060010684	0.046672961	Bcat2/Glul/Phgdh/Sephs1	4
BP	GO:1902653	secondary alcohol biosynthetic process	4/361	57/28814	5.6	0.005677795	0.060010684	0.046672961	Dhcr7/Mvd/Mvk/Prkaa1	4
BP	GO:0001678	cellular glucose homeostasis	7/361	170/28814	3.29	0.00577287	0.060839725	0.047317743	Nrxn1/Pck2/Pdk3/Ppp3ca/Prkaa1/Rab11b/Smad4	7
BP	GO:0030048	actin filament-based movement	6/361	129/28814	3.71	0.005816161	0.060944701	0.047399388	Atp1a1/Atp2a2/Camk2d/Dlg1/Epdr1/Scn4b	6
BP	GO:0031929	TOR signaling	6/361	129/28814	3.71	0.005816161	0.060944701	0.047399388	Klhl22/Lamtor1/Prkaa1/Sec13/Syap1/Zmpste24	6
BP	GO:0019932	second-messenger-mediated signaling	10/361	309/28814	2.58	0.005912707	0.061779336	0.04804852	Atp2a2/Homer2/L1cam/Ppp1r9b/Ppp3ca/Ppp3cc/Ppp3r1/Prkaa1/Slc8a1/Zmpste24	10
BP	GO:0007631	feeding behavior	6/361	130/28814	3.68	0.006034952	0.062261175	0.048423267	Atp8a2/Bdnf/Cck/Eif2ak4/Pex13/Stat3	6
BP	GO:0010257	NADH dehydrogenase complex assembly	4/361	58/28814	5.5	0.006039243	0.062261175	0.048423267	Ndufa12/Ndufa9/Ndufaf3/Oxa1l	4
BP	GO:0032981	mitochondrial respiratory chain complex I assembly	4/361	58/28814	5.5	0.006039243	0.062261175	0.048423267	Ndufa12/Ndufa9/Ndufaf3/Oxa1l	4
BP	GO:0051496	positive regulation of stress fiber assembly	4/361	58/28814	5.5	0.006039243	0.062261175	0.048423267	Cd47/Pxn/Rock2/Sorbs3	4
BP	GO:0050954	sensory perception of mechanical stimulus	10/361	310/28814	2.57	0.006043948	0.062261175	0.048423267	Atp2b2/Cacnb3/Homer2/Kcna1/Map1a/Phf24/Ppip5k2/Scn8a/Slc12a2/Tip1	10
BP	GO:0030258	lipid modification	8/361	216/28814	2.96	0.00611686	0.062835274	0.04886977	Acaa1a/B4galnt1/Gcdh/Inpp5b/Lamtor1/Mboat7/Pex13/Prkaa1	8
BP	GO:0009225	nucleotide-sugar metabolic process	3/361	30/28814	7.98	0.006162405	0.063103157	0.049078114	Extl2/Pmm2/Uap1	3
BP	GO:1901342	regulation of vasculature development	10/361	311/28814	2.57	0.006177449	0.063103157	0.049078114	Adam10/Glul/Pdcl3/Pxn/Rock2/S100a1/Sema4a/Slc12a2/Stat3/Tip1	10
BP	GO:0046034	ATP metabolic process	9/361	263/28814	2.73	0.006214175	0.063301496	0.049232371	Ampd3/Bdnf/Chchd2/Coq7/Cox7a1/Ndufa12/Prkaa1/Slc4a1/Stat3	9
BP	GO:0051592	response to calcium ion	6/361	131/28814	3.66	0.006259678	0.063587894	0.049455116	Alg2/Calm1/Cpne8/Nrxn1/Ppp3ca/Prkaa1	6
BP	GO:0006403	RNA localization	7/361	173/28814	3.23	0.006336422	0.064011868	0.049784859	Ahctf1/Alkbh5/Alyref/Nup188/Rae1/Sec13/Stau1	7
BP	GO:0051017	actin filament bundle assembly	7/361	173/28814	3.23	0.006336422	0.064011868	0.049784859	Add2/Cd47/Pxn/Rock2/Sorbs1/Sorbs3/Tip1	7
BP	GO:0097035	regulation of membrane lipid distribution	4/361	59/28814	5.41	0.006415675	0.064104813	0.049857146	Abca1/Atp8a2/Atp9a/Slc4a1	4
BP	GO:0007626	locomotory behavior	9/361	265/28814	2.71	0.006518104	0.064104813	0.049857146	Atp2b2/Cend1/Gprc5b/Oxr1/Pex13/Scn8a/Tmod1/Zdhhc8/Zmpste24	9
BP	GO:1903169	regulation of calcium ion transmembrane transport	7/361	174/28814	3.21	0.006533081	0.064104813	0.049857146	Cacnb3/Calm1/Camk2d/Cbarp/Jph3/S100a1/Slc8a1	7
BP	GO:0006616	SRP-dependent cotranslational protein targeting to membrane, translocation	2/361	10/28814	15.96	0.006591033	0.064104813	0.049857146	Sec61a1/Sec61a2	2
BP	GO:0014883	transition between fast and slow fiber	2/361	10/28814	15.96	0.006591033	0.064104813	0.049857146	Atp2a2/Ppp3ca	2
BP	GO:0021684	cerebellar granular layer formation	2/361	10/28814	15.96	0.006591033	0.064104813	0.049857146	Atp2b2/Nrxn1	2
BP	GO:0021707	cerebellar granule cell differentiation	2/361	10/28814	15.96	0.006591033	0.064104813	0.049857146	Atp2b2/Nrxn1	2
BP	GO:0033572	transferrin transport	2/361	10/28814	15.96	0.006591033	0.064104813	0.049857146	Arhgap1/Rab11b	2
BP	GO:0035864	response to potassium ion	2/361	10/28814	15.96	0.006591033	0.064104813	0.049857146	Nptx1/Slc12a2	2
BP	GO:0035865	cellular response to potassium ion	2/361	10/28814	15.96	0.006591033	0.064104813	0.049857146	Nptx1/Slc12a2	2
BP	GO:0061762	CAMKK-AMPK signaling cascade	2/361	10/28814	15.96	0.006591033	0.064104813	0.049857146	Prkaa1/Zmpste24	2

BP	GO:0040013	negative regulation of locomotion	17/607	340/28814	2.37	0.000980024	0.012517579	0.009063774	Adgrg1/Apex1/Atp2b4/Dab2ip/Dbn1/Drd1/Gtpbp4/Hmgb1/Igfbp5/Mef2c/Ndrg4/Osbpl8/Rac1/Rhob/Sema7a/S...	17
BP	GO:1903242	regulation of cardiac muscle hypertrophy in response to stress	3/607	10/28814	14.24	0.000999552	0.012699096	0.009195208	Atp2b4/Foxo1/Lmna	3
BP	GO:2000807	regulation of synaptic vesicle clustering	3/607	10/28814	14.24	0.000999552	0.012699096	0.009195208	Cdh2/Nlgn3/Picalm	3
BP	GO:0008306	associative learning	9/607	120/28814	3.56	0.001014765	0.01277248	0.009248344	Adcy3/Asic1/Brsk1/Csmd1/Drd1/Ndrg4/Nlgn3/Pde1b/Snap25	9
BP	GO:0014812	muscle cell migration	9/607	120/28814	3.56	0.001014765	0.01277248	0.009248344	Apex1/Enpp1/Grb10/Igfbp5/Mef2c/Naca/Ndrg4/S100a11/Thbs4	9
BP	GO:2000177	regulation of neural precursor cell proliferation	9/607	120/28814	3.56	0.001014765	0.01277248	0.009248344	Adgrg1/Cdh2/Cend1/Flna/Foxo1/Gjc2/Kdm1a/Rsu1/Setd1a	9
BP	GO:0001964	startle response	5/607	37/28814	6.41	0.001018697	0.01277248	0.009248344	Csmd1/Dbn1/Fabp7/Nlgn3/Slc6a3	5
BP	GO:0090022	regulation of neutrophil chemotaxis	5/607	37/28814	6.41	0.001018697	0.01277248	0.009248344	Camk1d/Mcu/Rac1/Rac2/Thbs4	5
BP	GO:1902476	chloride transmembrane transport	6/607	55/28814	5.18	0.001029637	0.012875855	0.009323195	Ano3/Gabra3/Gabra4/Gabrb2/Gabrd/Slc1a3	6
BP	GO:0051928	positive regulation of calcium ion transport	10/607	145/28814	3.27	0.001038418	0.012951759	0.009378156	Atp2b1/Bax/Drd1/Gjc2/Hap1/Hspa2/Jak3/P2rx7/Plcg2/Stim1	10
BP	GO:0051651	maintenance of location in cell	13/607	225/28814	2.74	0.001043608	0.012982595	0.009400484	Bax/Dbn1/Drd1/Ftl1/Grik5/Hap1/Hk2/P2rx7/Plcg2/Ptpn6/Ryr3/Skp1/Tmem38a	13
BP	GO:0006874	cellular calcium ion homeostasis	22/607	500/28814	2.09	0.001049736	0.01298529	0.009402436	Adcy8/Atp2b1/Atp2b4/Bax/Drd1/Ednrb/Grm1/Hap1/Hexb/Hmgb1/Jak3/Mcu/Nucb2/P2rx7/Plcg2/Prkcb/Ptpn6/Ryr3/Smpd3/Stim1/Thada/T...	22
BP	GO:0017121	plasma membrane phospholipid scrambling	4/607	22/28814	8.63	0.001054698	0.01298529	0.009402436	Ano3/P2rx7/Xkr5/Xkr9	4
BP	GO:0051602	response to electrical stimulus	4/607	22/28814	8.63	0.001054698	0.01298529	0.009402436	Actb/Grm1/P2rx7/Rab3a	4
BP	GO:0071800	podosome assembly	4/607	22/28814	8.63	0.001054698	0.01298529	0.009402436	Arhgef2/Dbnl/Lcp1/Msn	4
BP	GO:0008154	actin polymerization or depolymerization	12/607	198/28814	2.88	0.001067554	0.013109786	0.009492581	Actn2/Arpc1b/Arpc3/Arpc5l/Cracd/Dbn1/Dbnl/Kank4/Rac1/Ssh3/Tmod1/Vasp	12
BP	GO:0000956	nuclear-transcribed mRNA catabolic process	9/607	121/28814	3.53	0.001076272	0.013115692	0.009496857	Cnot7/Dcp1a/Dcps/Dhx36/Exosc10/Ncbp2/Noct/Paip1/Smg5	9
BP	GO:0044106	cellular amine metabolic process	9/607	121/28814	3.53	0.001076272	0.013115692	0.009496857	Abhd4/Atp2b4/Comt/Ddc/Drd1/Pde1b/Pnkd/Slc6a3/Th	9
BP	GO:1902414	protein localization to cell junction	9/607	121/28814	3.53	0.001076272	0.013115692	0.009496857	Actb/Cplx1/Flna/Klc1/Nlgn3/Nptx1/Snap25/Stx3/Vcl	9
BP	GO:0046390	ribose phosphate biosynthetic process	11/607	172/28814	3.04	0.001104264	0.013358101	0.009672382	Adcy3/Adcy8/Ak2/Atp5pb/Coasy/Dcakd/Dip2a/Gucy1b1/Nme3/Pdk2/Stat3	11
BP	GO:0032543	mitochondrial translation	7/607	76/28814	4.37	0.001107349	0.013358101	0.009672382	Chchd1/Coa3/Ears2/Mrpl44/Mrps14/Sars2/Taco1	7
BP	GO:0080164	regulation of nitric oxide metabolic process	7/607	76/28814	4.37	0.001107349	0.013358101	0.009672382	Atp2b4/Clu/Dnm2/Dynll1/Rac1/Sirpa/Smpd3	7
BP	GO:0140056	organelle localization by membrane tethering	7/607	76/28814	4.37	0.001107349	0.013358101	0.009672382	Bloc1s6/Kcnb1/Ndrg4/Rab3a/Rab3c/Rab8b/Stx3	7
BP	GO:0051271	negative regulation of cellular component movement	16/607	314/28814	2.42	0.00112198	0.013466574	0.009750925	Adgrg1/Apex1/Atp2b4/Dab2ip/Dbn1/Drd1/Gtpbp4/Hmgb1/Igfbp5/Mef2c/Ndrg4/Osbpl8/Rac1/Rhob/Srgap1/St...	16
BP	GO:0071219	cellular response to molecule of bacterial origin	16/607	314/28814	2.42	0.00112198	0.013466574	0.009750925	Abca1/B2m/Chmp5/Dab2ip/Gbp2/Hmgb1/Irgm1/Mapk3/Mef2c/Pdcd4/Plcg2/Sirpa/Stat1/Traf6/Tut4/Vim	16
BP	GO:0009636	response to toxic substance	9/607	122/28814	3.5	0.001140761	0.013623534	0.009864578	Aldh1a1/Bax/Ddc/Esd/Gjc2/Mapk3/Scn1b/Slc18a2/Sod1	9
BP	GO:0021782	glial cell development	9/607	122/28814	3.5	0.001140761	0.013623534	0.009864578	Arhgef10/B4galt6/Clu/Drd1/Enpp1/Gfap/Ntrk3/Sod1/Vim	9
BP	GO:0006401	RNA catabolic process	15/607	285/28814	2.5	0.001149944	0.013698957	0.00991919	Apex1/Cnot7/Dcp1a/Dcps/Dhx36/Exosc10/Hnrnpa0/Mtrex/Ncbp2/Noct/Nudt12/Paip1/Smg5/Tut4/Vim	15
BP	GO:0090257	regulation of muscle system process	14/607	256/28814	2.6	0.001155519	0.013731124	0.009942482	Atp2b1/Atp2b4/Ctnna3/Dbn1/Fgf13/Foxo1/Igfbp5/Lmna/Mef2c/Ptgs1/Setd3/Sod1/Tmem38a/Vcl	14
BP	GO:0016197	endosomal transport	13/607	228/28814	2.71	0.001176852	0.013947541	0.010099186	Abca1/Actn2/Ap5m1/Chmp5/Lamtor1/Ndrg4/Nsg2/Picalm/Rab14/Snap25/Stam/Vamp3/Zfyve16	13
BP	GO:0001938	positive regulation of endothelial cell proliferation	8/607	99/28814	3.84	0.00118249	0.013947541	0.010099186	Hmgb1/Hmgb2/Jcad/Mydgf/Nras/Ppp1r16b/Stat3/Thbs4	8
BP	GO:0002690	positive regulation of leukocyte chemotaxis	8/607	99/28814	3.84	0.00118249	0.013947541	0.010099186	Camk1d/Hmgb1/Mapk3/Mcu/Pla2g7/Rac1/Rac2/Thbs4	8
BP	GO:0005977	glycogen metabolic process	7/607	77/28814	4.32	0.001196345	0.014041616	0.010167304	Enpp1/Grb10/Hmgb1/Phka1/Phkb/Ppp1cc/Sorbs1	7
BP	GO:0006073	cellular glucan metabolic process	7/607	77/28814	4.32	0.001196345	0.014041616	0.010167304	Enpp1/Grb10/Hmgb1/Phka1/Phkb/Ppp1cc/Sorbs1	7
BP	GO:0006979	response to oxidative stress	19/607	409/28814	2.21	0.001211079	0.014162422	0.010254778	Aldh1a1/Apex1/Cst3/Endog/Foxo1/Gab1/Glrx2/Mapk3/Meak7/Naprt/Ndufa12/Pdk2/Ptgs1/Rcan1/Rhob/Sigmar1/Sirpa/Smpd3/Sod1	19
BP	GO:1903169	regulation of calcium ion transmembrane transport	11/607	174/28814	3	0.001212567	0.014162422	0.010254778	Bax/Drd1/Gjc2/Gnb5/Hap1/Hspa2/Plcg2/Ptpn6/Rgs9/Stim1/Tmem38a	11
BP	GO:0007411	axon guidance	14/607	258/28814	2.58	0.00124404	0.014494583	0.01049529	Cdk5r2/Chn1/Efnb1/Gap43/Mef2c/Nexn/Nrp2/Plxnc1/Rac1/Rac2/Scn1b/Sema7a/Vangl2/Vax...	14
BP	GO:0072522	purine-containing compound biosynthetic process	11/607	175/28814	2.98	0.001269908	0.014759161	0.010686867	Adcy3/Adcy8/Ak2/Atp5pb/Coasy/Dcakd/Dip2a/Gucy1b1/Nme3/Pdk2/Stat3	11
BP	GO:0019233	sensory perception of pain	10/607	149/28814	3.19	0.001276553	0.014759161	0.010686867	Comt/Ednrb/Grm1/Kcnd2/Mapk3/Mme/P2rx7/Prkar1b/Ptgs1/Tmem100	10
BP	GO:0030218	erythrocyte differentiation	10/607	149/28814	3.19	0.001276553	0.014759161	0.010686867	B2m/Bap1/Heph/Hmgb2/Jak3/Kdm1a/Setd1a/Slc25a5/Stat1/Stat3	10
BP	GO:0015748	organophosphate ester transport	9/607	124/28814	3.45	0.001279107	0.014759161	0.010686867	Abca1/Ano3/Osbpl8/P2rx7/Pitpnc1/Slc25a33/Slc25a5/Xkr5/Xkr9	9
BP	GO:0044042	glucan metabolic process	7/607	78/28814	4.26	0.001290845	0.014858717	0.010758953	Enpp1/Grb10/Hmgb1/Phka1/Phkb/Ppp1cc/Sorbs1	7
BP	GO:0031281	positive regulation of cyclase activity	5/607	39/28814	6.09	0.001299857	0.014926479	0.010808019	Adcy3/Drd1/Gnal/Mapk3/Stim1	5
BP	GO:0050673	epithelial cell proliferation	21/607	476/28814	2.09	0.001307448	0.014977646	0.010845068	B2m/Bax/Dab2ip/Ednrb/Fabp7/Hmgb1/Hmgb2/Igfbp4/Igfbp5/Jcad/Map2k2/Mef2c/Mydgf/Nras/Ppp1r16b/Rap1gap/Sparc/Stat1/Stat3/Tgfa/Thbs4	21
BP	GO:0046486	glycerolipid metabolic process	18/607	381/28814	2.24	0.001337246	0.015242834	0.011037086	Abhd4/Cds1/Dgki/Gpcpd1/Hdhd5/Inpp5j/Itpka/Mtmr12/Pcyt1b/Pigt/Pip4p1/Pip5k1a/Pitpnc1/Pla2g4a/Pla2g7/Plcg2/Pld1/Sgpp1...	18
BP	GO:0090630	activation of GTPase activity	8/607	101/28814	3.76	0.001345874	0.015242834	0.011037086	Arhgef10/Ngef/Ntrk3/Pip5k1a/Rap1gap/Rcc2/Tbc1d8/Tiam2	8
BP	GO:0010612	regulation of cardiac muscle adaptation	3/607	11/28814	12.95	0.001352933	0.015242834	0.011037086	Atp2b4/Foxo1/Lmna	3
BP	GO:0031630	regulation of synaptic vesicle fusion to presynaptic active zone membrane	3/607	11/28814	12.95	0.001352933	0.015242834	0.011037086	Cplx1/Grik5/Rab3a	3
BP	GO:0060020	Bergmann glial cell differentiation	3/607	11/28814	12.95	0.001352933	0.015242834	0.011037086	Gfap/Mapk3/Vim	3
BP	GO:0072584	caveolin-mediated endocytosis	3/607	11/28814	12.95	0.001352933	0.015242834	0.011037086	Mapk3/Mlc1/Unc119	3
BP	GO:1901632	regulation of synaptic vesicle membrane organization	3/607	11/28814	12.95	0.001352933	0.015242834	0.011037086	Cplx1/Grik5/Rab3a	3
BP	GO:0071277	cellular response to calcium ion	7/607	79/28814	4.21	0.001391083	0.015601305	0.011296649	Adcy8/Cpne3/Kcnb1/Mef2c/Plce2/Rasgrp2/Syt17	7
BP	GO:0071478	cellular response to radiation	11/607	177/28814	2.95	0.001391282	0.015601305	0.011296649	Bax/Crebbp/Dhx36/Gnb5/Kdm1a/Mme/Nucks1/Pcna/Rgs9/Rhob/Yy1	11
BP	GO:0050921	positive regulation of chemotaxis	10/607	151/28814	3.14	0.001411306	0.015788776	0.011432394	Camk1d/Hmgb1/Mapk3/Mcu/Ntrk3/Pla2g7/Rac1/Rac2/Stx3/Thbs4	10
BP	GO:0051056	regulation of small GTPase mediated signal transduction	13/607	233/28814	2.65	0.001430341	0.015923949	0.01153027	Abca1/Adgrg1/Arhgef10/Arhgef2/Cdh2/Chn1/Dab2ip/Dgki/Dnm2/Prag1/Ralgps2/Rap1gap/Shoc2	13
BP	GO:0009308	amine metabolic process	9/607	126/28814	3.39	0.00143067	0.015923949	0.01153027	Abhd4/Atp2b4/Comt/Ddc/Drd1/Pde1b/Pnkd/Slc6a3/Th	9
BP	GO:0150115	cell-substrate junction organization	8/607	102/28814	3.72	0.00143403	0.015923949	0.01153027	Actn2/Arhgap6/Pip5k1a/Rab8b/Rac1/Rcc2/Sorbs1/Vcl	8
BP	GO:0120032	regulation of plasma membrane bounded cell projection assembly	12/607	205/28814	2.78	0.001436722	0.015923949	0.01153027	Dbn1/Dnm2/Dynll1/Gap43/Hap1/P2rx7/Pld1/Rac1/Rac2/Rap1gap/Rcc2/Vdac3	12
BP	GO:0001881	receptor recycling	5/607	40/28814	5.93	0.001460129	0.016048741	0.01162063	Ache/Anxa2/Chmp5/Lamtor1/Vamp3	5
BP	GO:0019884	antigen processing and presentation of exogenous antigen	5/607	40/28814	5.93	0.001460129	0.016048741	0.01162063	B2m/H2-K1/Psme1/Psme2/Traf6	5
BP	GO:1902624	positive regulation of neutrophil migration	5/607	40/28814	5.93	0.001460129	0.016048741	0.01162063	Camk1d/Mcu/Rac1/Rac2/Thbs4	5
BP	GO:0030336	negative regulation of cell migration	15/607	292/28814	2.44	0.00146142	0.016048741	0.01162063	Adgrg1/Apex1/Atp2b4/Dab2ip/Drd1/Gtpbp4/Hmgb1/Igfbp5/Mef2c/Ndrg4/Osbpl8/Rac1/Rhob/Srgap1/Stat3	15
BP	GO:1900006	positive regulation of dendrite development	4/607	24/28814	7.91	0.001481868	0.016210013	0.011737404	Dab2ip/Pacsin1/Sez6/Tmem106b	4
BP	GO:0007034	vacuolar transport	10/607	152/28814	3.12	0.001482892	0.016210013	0.011737404	Chmp5/Clu/Lamp2/Mon1b/Rab34/Rhob/Rnf157/Stam/Tmem106b/Zfyve16	10

BP	GO:0072344	rescue of stalled ribosome	2/361	10/28814	15.96	0.006591033	0.064104813	0.049857146	Ascc3/Trip4	2
BP	GO:0090148	membrane fission	2/361	10/28814	15.96	0.006591033	0.064104813	0.049857146	Coro1c/Sh3gl2	2
BP	GO:1903799	negative regulation of production of miRNAs involved in gene silencing by miRNA	2/361	10/28814	15.96	0.006591033	0.064104813	0.049857146	Ppp3ca/Zmpste24	2
BP	GO:1903522	regulation of blood circulation	9/361	266/28814	2.7	0.006674288	0.064742362	0.050352996	Atp1a1/Atp2a2/Atp2b1/Calm1/Dlg1/S100a1/Scn4b/Slc8a1/Zmpste24	9
BP	GO:0002026	regulation of the force of heart contraction	3/361	31/28814	7.72	0.00676014	0.064892356	0.050469653	Atp1a1/Atp2a2/Slc8a1	3
BP	GO:0021533	cell differentiation in hindbrain	3/361	31/28814	7.72	0.00676014	0.064892356	0.050469653	Atp2b2/Cend1/Nrxn1	3
BP	GO:1903421	regulation of synaptic vesicle recycling	3/361	31/28814	7.72	0.00676014	0.064892356	0.050469653	Ppp3cc/Sh3gl1/Slc17a7	3
BP	GO:0051047	positive regulation of secretion	12/361	417/28814	2.3	0.006760729	0.064892356	0.050469653	Apbb1/Cadps/Cadps2/Cck/Frmd4a/Glul/Pcp4/Pcsk1/Rab2b/Slc12a2/Smad4/Stx1a	12
BP	GO:0030522	intracellular receptor signaling pathway	8/361	220/28814	2.9	0.006805416	0.064998628	0.050552306	Aldh1a2/Bdnf/Foxp1/Nrx1/Padi2/Pdk3/Stat3/Trip4	8
BP	GO:0055078	sodium ion homeostasis	4/361	60/28814	5.32	0.006807349	0.064998628	0.050552306	Atp1a1/Atp1a4/Slc12a2/Slc8a1	4
BP	GO:0032956	regulation of actin cytoskeleton organization	11/361	366/28814	2.4	0.006840693	0.065146909	0.05066763	Add2/Arpc5l/Cd47/Dlg1/Pxn/Rock2/Smad4/Sorbs3/Tjp1/Tmod1/Vasp	11
BP	GO:0061572	actin filament bundle organization	7/361	176/28814	3.17	0.006939978	0.065916838	0.051266438	Add2/Cd47/Pxn/Rock2/Sorbs1/Sorbs3/Tjp1	7
BP	GO:0032411	positive regulation of transporter activity	6/361	134/28814	3.57	0.006970373	0.065916838	0.051266438	Ank3/Cacnb3/Calm1/Kcna1/Plcb1/S100a1	6
BP	GO:0006644	phospholipid metabolic process	11/361	367/28814	2.39	0.006975613	0.065916838	0.051266438	Abhd3/Abhd6/Etnk1/Inpp5b/Itpka/Mboat7/Mvd/Mvk/Pcyt1b/Pip4k2c/Plcb1	11
BP	GO:0006397	mRNA processing	13/361	472/28814	2.2	0.007015237	0.066064401	0.051381204	Alkbh5/Alyref/Bud31/Cdk9/Celf4/Plcb1/Ppwd1/Prpf40a/Sf3b4/Sf3b5/Snrpa/Snu13/Srsf2	13
BP	GO:0016358	dendrite development	10/361	317/28814	2.52	0.007027359	0.066064401	0.051381204	Adam10/Bdnf/Camk2a/Itpka/Map1a/Numb/Ppp1r9b/Ppp3ca/Rock2/Tpbg	10
BP	GO:0006606	protein import into nucleus	7/361	178/28814	3.14	0.007365388	0.069064677	0.053714652	Ipo8/Nup188/Ppp3ca/Ppp3r1/Ranbp6/Sec13/Stat3	7
BP	GO:0015833	peptide transport	10/361	320/28814	2.49	0.007484927	0.069956345	0.054408141	Abca1/Cplx1/Cplx3/Glul/Nrxn1/Ppp3ca/Rab11b/Rims2/Slc26a6/Stx1a	10
BP	GO:0050803	regulation of synapse structure or activity	9/361	271/28814	2.65	0.007498739	0.069956345	0.054408141	Adam10/Bdnf/Itpka/Mdga2/Nptx1/Nrxn1/Sema4a/Slc17a7/Tpbg	9
BP	GO:0062012	regulation of small molecule metabolic process	11/361	371/28814	2.37	0.007536234	0.070127245	0.054541058	Arpp19/Atcay/Clcn2/Dhcr7/Pdk1/Pdk3/Prkaa1/Slco4a1/Sorbs1/Stat3/Zmpste24	11
BP	GO:0008652	cellular amino acid biosynthetic process	4/361	62/28814	5.15	0.007637431	0.070530519	0.054854701	Bcat2/Glul/Phgdh/Sephs1	4
BP	GO:0051932	synaptic transmission, GABAergic	4/361	62/28814	5.15	0.007637431	0.070530519	0.054854701	Bdnf/Gabra3/Phf24/Tpbg	4
BP	GO:1903078	positive regulation of protein localization to plasma membrane	4/361	62/28814	5.15	0.007637431	0.070530519	0.054854701	Dlg1/Nrxn1/Ppp1r9b/Sorbs1	4
BP	GO:0051651	maintenance of location in cell	8/361	225/28814	2.84	0.007746716	0.071359543	0.05549947	Ank3/Arl2bp/Calm1/Camk2d/Jph3/Skp1/Slc30a3/Slc8a1	8
BP	GO:1904063	negative regulation of cation transmembrane transport	5/361	98/28814	4.07	0.007889144	0.07186165	0.055889981	Ank3/Cacnb3/Calm1/Camk2d/Cbarp	5
BP	GO:0010807	regulation of synaptic vesicle priming	2/361	11/28814	14.51	0.007989341	0.07186165	0.055889981	Napa/Stx1a	2
BP	GO:0031630	regulation of synaptic vesicle fusion to presynaptic active zone membrane	2/361	11/28814	14.51	0.007989341	0.07186165	0.055889981	Cplx1/Cplx3	2
BP	GO:0042117	monocyte activation	2/361	11/28814	14.51	0.007989341	0.07186165	0.055889981	Adam10/Foxp1	2
BP	GO:0042118	endothelial cell activation	2/361	11/28814	14.51	0.007989341	0.07186165	0.055889981	Foxp1/Smad4	2
BP	GO:0045472	response to ether	2/361	11/28814	14.51	0.007989341	0.07186165	0.055889981	Foxp1/Plcb1	2
BP	GO:0060052	neurofilament cytoskeleton organization	2/361	11/28814	14.51	0.007989341	0.07186165	0.055889981	Atp8a2/Nefm	2
BP	GO:1901632	regulation of synaptic vesicle membrane organization	2/361	11/28814	14.51	0.007989341	0.07186165	0.055889981	Cplx1/Cplx3	2
BP	GO:1990504	dense core granule exocytosis	2/361	11/28814	14.51	0.007989341	0.07186165	0.055889981	Cadps/Cadps2	2
BP	GO:0021697	cerebellar cortex formation	3/361	33/28814	7.26	0.008055857	0.07186165	0.055889981	Atp2b2/Cend1/Nrxn1	3
BP	GO:0062149	detection of stimulus involved in sensory perception of pain	3/361	33/28814	7.26	0.008055857	0.07186165	0.055889981	Cacnb3/Kcna1/Phf24	3
BP	GO:0009064	glutamine family amino acid metabolic process	4/361	63/28814	5.07	0.00807633	0.07186165	0.055889981	Atcay/Glul/Phgdh/Ppat	4
BP	GO:0030865	cortical cytoskeleton organization	4/361	63/28814	5.07	0.00807633	0.07186165	0.055889981	Dlg1/Ehd2/Epb41l2/Rock2	4
BP	GO:0044091	membrane biogenesis	4/361	63/28814	5.07	0.00807633	0.07186165	0.055889981	Ank3/Nrxn1/Pex19/Stx2	4
BP	GO:0014033	neural crest cell differentiation	5/361	99/28814	4.03	0.008225603	0.072805724	0.05662423	Aldh1a2/Coro1c/Sema4a/Sema4f/Smad4	5
BP	GO:0051492	regulation of stress fiber assembly	5/361	99/28814	4.03	0.008225603	0.072805724	0.05662423	Cd47/Pxn/Rock2/Sorbs3/Tjp1	5
BP	GO:0000910	cytokinesis	7/361	182/28814	3.07	0.008273703	0.072805724	0.05662423	Ahctf1/Ank3/Calm1/Prpf40a/Rock2/Septin4/Stx2	7
BP	GO:0051170	import into nucleus	7/361	182/28814	3.07	0.008273703	0.072805724	0.05662423	Ipo8/Nup188/Ppp3ca/Ppp3r1/Ranbp6/Sec13/Stat3	7
BP	GO:0051588	regulation of neurotransmitter transport	6/361	139/28814	3.45	0.008281975	0.072805724	0.05662423	Atp2a2/Camk2a/Cplx1/Cplx3/Rims2/Stx1a	6
BP	GO:0071674	mononuclear cell migration	7/361	183/28814	3.05	0.008513115	0.074639994	0.058050822	Adam10/Cd47/Cd99/Padi2/Plcb1/Sirpa/Slc12a2	7
BP	GO:0061077	chaperone-mediated protein folding	4/361	64/28814	4.99	0.008531451	0.074639994	0.058050822	Dnajb6/Pdcl3/Pex19/Sdf2	4
BP	GO:0006650	glycerophospholipid metabolic process	9/361	277/28814	2.59	0.008588284	0.074779412	0.058159253	Abhd3/Abhd6/Etnk1/Inpp5b/Itpka/Mboat7/Pcyt1b/Pip4k2c/Plcb1	9
BP	GO:0048588	developmental cell growth	9/361	277/28814	2.59	0.008588284	0.074779412	0.058159253	Bdnf/Camk2d/Eif2ak4/Foxp1/L1cam/Rims2/Sema4a/Sema4f/Sh3gl2	9
BP	GO:0035308	negative regulation of protein dephosphorylation	3/361	34/28814	7.04	0.008754646	0.075154321	0.058450837	Arpp19/Ppp1r9b/Rock2	3
BP	GO:0045920	negative regulation of exocytosis	3/361	34/28814	7.04	0.008754646	0.075154321	0.058450837	Atp9a/Cbarp/Rabgef1	3
BP	GO:0060306	regulation of membrane repolarization	3/361	34/28814	7.04	0.008754646	0.075154321	0.058450837	Cacnb3/Scn4b/Zmpste24	3
BP	GO:0099068	postsynapse assembly	3/361	34/28814	7.04	0.008754646	0.075154321	0.058450837	Nptx1/Nrxn1/Numb	3
BP	GO:0099560	synaptic membrane adhesion	3/361	34/28814	7.04	0.008754646	0.075154321	0.058450837	Lrrc4c/Mdga2/Nrxn1	3
BP	GO:1901020	negative regulation of calcium ion transmembrane transporter activity	3/361	34/28814	7.04	0.008754646	0.075154321	0.058450837	Cacnb3/Calm1/Cbarp	3
BP	GO:0006790	sulfur compound metabolic process	10/361	328/28814	2.43	0.008817987	0.075344344	0.058598626	Abcb7/Gcdh/Glo1/Hbb-b1/Hmgcr/Mvd/Mvk/Pdk1/Pdk3/Phgdh	10
BP	GO:0051146	striated muscle cell differentiation	10/361	328/28814	2.43	0.008817987	0.075344344	0.058598626	Bdnf/Camk2d/Csrp1/Ehd2/Foxp1/Ppp3ca/Slc8a1/Smad4/Tmod1/Zmpste24	10
BP	GO:0002181	cytoplasmic translation	5/361	101/28814	3.95	0.008928117	0.07575435	0.058917506	Denr/Dhx29/Rpl15/Rpl18a/Rpl22l1	5
BP	GO:0009062	fatty acid catabolic process	5/361	101/28814	3.95	0.008928117	0.07575435	0.058917506	Abhd3/Acaa1a/Gcdh/Pck2/Pex13	5
BP	GO:0050795	regulation of behavior	5/361	101/28814	3.95	0.008928117	0.07575435	0.058917506	Cck/Eif2ak4/Kcna2/Nrxn1/Stat3	5
BP	GO:0006081	cellular aldehyde metabolic process	4/361	65/28814	4.91	0.009003026	0.075896791	0.059028289	Aldh1a2/Cox2/Dhrs13/Hagh	4
BP	GO:0009166	nucleotide catabolic process	4/361	65/28814	4.91	0.009003026	0.075896791	0.059028289	Ampd3/Nt5m/Nudt3/Nudt4	4
BP	GO:0050770	regulation of axonogenesis	7/361	185/28814	3.02	0.009007166	0.075896791	0.059028289	Bdnf/L1cam/Lrrc4c/Nefm/Plxnc1/Sema4a/Sema4f	7
BP	GO:0046486	glycerolipid metabolic process	11/361	381/28814	2.3	0.009091859	0.076434314	0.059446344	Abhd3/Abhd6/Cat/Etnk1/Inpp5b/Itpka/Mboat7/Pck2/Pcyt1b/Pip4k2c/Plcb1	11
BP	GO:0044743	protein transmembrane import into intracellular organelle	3/361	35/28814	6.84	0.009487874	0.077381493	0.060183007	Pex1/Pex13/Timm23	3
BP	GO:0099590	neurotransmitter receptor internalization	3/361	35/28814	6.84	0.009487874	0.077381493	0.060183007	Gsg1l/Numb/Ppp3r1	3

BP	GO:0006826	iron ion transport	6/607	59/28814	4.83	0.001490993	0.016224313	0.011747759	B2m/Dnm2/Heph/Myo1b/Parms2/Slc6a9	6
BP	GO:0071622	regulation of granulocyte chemotaxis	6/607	59/28814	4.83	0.001490993	0.016224313	0.011747759	Camk1d/Mapk3/Mou/Rac1/Rac2/Thbs4	6
BP	GO:0051145	smooth muscle cell differentiation	7/607	80/28814	4.15	0.001497295	0.016255863	0.011770603	Dnmt1/Ednrb/Mef2c/Olfm2/Pdcd4/Rcan1/Vangl2	7
BP	GO:0045785	positive regulation of cell adhesion	21/607	482/28814	2.07	0.001523181	0.016499397	0.011946943	Adgrg1/Apbb1ip/B2m/Dbn1/Dnaja3/Dnm2/Efnb1/Fcho1/Flna/Hmgb1/Itk3/Lgals1/Pdpn/Ptpn6/Rac1/Rap1gap/Rsu1/Sirpa/Stx3/Traf6/Vav	21
BP	GO:0099565	chemical synaptic transmission, postsynaptic	8/607	103/28814	3.69	0.001526707	0.016500185	0.011947513	Cdf4/Dbn1/Dgki/Grik5/Mef2c/Nlgn3/Prkar1b/Sez6	8
BP	GO:0006839	mitochondrial transport	10/607	153/28814	3.1	0.001557399	0.016756069	0.012132795	Bag3/Bax/Bk2/Nptx1/Ppm1k/Slc25a5/Stat3/Timm13/Tim...	10
BP	GO:0010565	regulation of cellular ketone metabolic process	10/607	153/28814	3.1	0.001557399	0.016756069	0.012132795	Atp2b4/Cln2/Comt/Coq3/Dkk3/Edb/Nucb2/Pdk2/Pla...	10
BP	GO:0030324	lung development	13/607	236/28814	2.61	0.001603102	0.01719808	0.012452848	Cln2/Hmgb1/Hspb11/Igfbp5/Lipa/Map2k2/Mapk3/Pdpn/Pdk7/Rab3a/Smpd3/Spar...	13
BP	GO:0022406	membrane docking	7/607	81/28814	4.1	0.001609721	0.01719808	0.012452848	Bloc1s6/Kcnb1/Ndrg4/Rab3a/Rab3c/Rab8b/Stx3	7
BP	GO:0046394	carboxylic acid biosynthetic process	15/607	295/28814	2.41	0.001615057	0.01719808	0.012452848	Aass/Abcd3/Acsbg1/Aldh1a1/Atp2b4/Edb/Hacd3/Mcat/Ndufab1/Pla2g4a/Prkag1/Ptgs1/Sephs1/Slc1a3/Ugdh	15
BP	GO:0022900	electron transport chain	8/607	104/28814	3.65	0.001624068	0.01719808	0.012452848	Cox7c/Etfrf1/Ndufa10/Ndufa12/Ndufb6/Sdhaf2/Slc1a3/...	8
BP	GO:0060491	regulation of cell projection assembly	12/607	208/28814	2.74	0.001624345	0.01719808	0.012452848	Dbn1/Dnm2/Dyrk1/Gap43/Hap1/P2rx7/Pld1/Rac1/Rac...	12
BP	GO:0000288	nuclear-transcribed mRNA catabolic process, deadenylation-dependent decay	6/607	60/28814	4.75	0.00162764	0.01719808	0.012452848	Cnot7/Dcp1a/Dcps/Dhx36/Nocr/Paip1	6
BP	GO:1900024	regulation of substrate adhesion-dependent cell spreading	6/607	60/28814	4.75	0.00162764	0.01719808	0.012452848	Dbn1/Dnm2/Flna/Pdpn/Rac1/Rac2	6
BP	GO:0001667	ameboidal-type cell migration	20/607	452/28814	2.1	0.001628657	0.01719808	0.012452848	Atp2b4/Cdh2/Dab2ip/Ednrb/Efnb1/Hmgb1/Icad/Mef2c/Nrp2/Phactr4/Pip5k1a/Plcg2/Plekhg5/Rac1/Rcc2/Rhob/Sema7a/Sgpl1/Sox8/Sparc	20
BP	GO:0032365	intracellular lipid transport	5/607	41/28814	5.79	0.001634484	0.01719808	0.012452848	Abca1/Abcd3/Anxa2/Gramd1a/Sgpp1	5
BP	GO:1901381	positive regulation of potassium ion transmembrane transport	5/607	41/28814	5.79	0.001634484	0.01719808	0.012452848	Actn2/Dnm2/Flna/Kcnc1/Kcnip2	5
BP	GO:0016053	organic acid biosynthetic process	15/607	296/28814	2.41	0.001669179	0.017524543	0.012689234	Aass/Abcd3/Acsbg1/Aldh1a1/Atp2b4/Edb/Hacd3/Mcat/Ndufab1/Pla2g4a/Prkag1/Ptgs1/Sephs1/Slc1a3/Ugdh	15
BP	GO:0032271	regulation of protein polymerization	12/607	209/28814	2.73	0.001691194	0.017716738	0.012828399	Arpc1b/Arpc3/Arpc5l/Cracd/Dbn1/Dbnl/Inppl/Kank4/...	12
BP	GO:0006869	lipid transport	18/607	390/28814	2.19	0.001728969	0.018056922	0.013074721	Abca1/Abca8b/Abcd3/Anxa3/Anxa2/Fabp7/Gramd1a/Nucb2/Osbpl8/P2rx7/Pitpnc1/Pla2g4a/Sar1b/Sgpp1/Sgms...	18
BP	GO:0046628	positive regulation of insulin receptor signaling pathway	4/607	25/28814	7.6	0.001735007	0.018056922	0.013074721	Mzmlo/Nucks1/Osbpl8/Sorbs1	4
BP	GO:2000311	regulation of AMPA receptor activity	4/607	25/28814	7.6	0.001735007	0.018056922	0.013074721	Mef2c/Nlgn3/Ptrt1/Shisa9	4
BP	GO:0006790	sulfur compound metabolic process	16/607	328/28814	2.32	0.001754672	0.018221882	0.013194166	Acot1/Acot11/Clic1/Comt/Dip2a/Dnmt1/Ednrb/Enpp1/Hmgcs1/Nudt7/Pdk2/Sod1/...	16
BP	GO:0042551	neuron maturation	6/607	61/28814	4.67	0.001773594	0.018361321	0.013295131	B4galt6/Ednrb/Kcnb1/Kcnn2/Mapk3/Rac1	6
BP	GO:0098693	regulation of synaptic vesicle cycle	3/607	12/28814	11.87	0.001775786	0.018361321	0.013295131	Pld1/Prkar1b/Slc10a4	3
BP	GO:0032984	protein-containing complex disassembly	13/607	239/28814	2.58	0.001792801	0.018497212	0.013393528	Actn2/Arhgef2/Chmp5/Cracd/Fgf13/Irgm1/Klc1/Lamp2/Ogfod1/Rpl23/Scaf/Sh3gl3	13
BP	GO:0016042	lipid catabolic process	16/607	329/28814	2.31	0.001809476	0.018629026	0.013488972	Abcd3/Abhd4/Cyp4f14/Ech...	16
BP	GO:0001935	endothelial cell proliferation	11/607	183/28814	2.85	0.001813679	0.018632134	0.013491223	Hmgb1/Hmgb2/Icad/Mef2c/Mydgf/Nrau/Ppp1r16b/Sparc...	11
BP	GO:0043001	Golgi to plasma membrane protein transport	5/607	42/28814	5.65	0.001823645	0.018694319	0.01353625	Bbs2/Rab26/Rab31/Rab34/Vamp3	5
BP	GO:0048863	stem cell differentiation	13/607	240/28814	2.57	0.001860011	0.019003167	0.013759882	Cdh2/Chd2/Dhx36/Ednrb/Efnb1/Mapk3/Mef2c/Nrp2/Phactr4/Sema7a/Smd1a/Sox8	13
BP	GO:0071214	cellular response to abiotic stimulus	16/607	330/28814	2.3	0.001865708	0.019003167	0.013759882	Actb/Asic1/Bag3/Bax/Crebbp/Dhx36/Gnb5/Gria1/Kdm1a/Mmc/Nucks1/Pcna/Rac1/...	16
BP	GO:0104004	cellular response to environmental stimulus	16/607	330/28814	2.3	0.001865708	0.019003167	0.013759882	Actb/Asic1/Bag3/Bax/Crebbp/Dhx36/Gnb5/Gria1/Kdm1a/Mmc/Nucks1/Pcna/Rac1/...	16
BP	GO:0006809	nitric oxide biosynthetic process	7/607	84/28814	3.96	0.001986748	0.020107408	0.014559444	Atp2b4/Cln/Dnm2/Dyrll1/Rac1/Sirna/Smnd3	7
BP	GO:0048708	astrocyte differentiation	7/607	84/28814	3.96	0.001986748	0.020107408	0.014559444	Drd1/Gfap/Mapk3/Ntrk3/Sox8/Stat3/Vim	7
BP	GO:2001259	positive regulation of cation channel activity	7/607	84/28814	3.96	0.001986748	0.020107408	0.014559444	Actn2/Hap1/Kcnc1/Nlgn3/P2rx7/Ras9/Stim1	7
BP	GO:0097164	ammonium ion metabolic process	4/607	26/28814	7.3	0.00201664	0.02035588	0.014739359	Achn/Chdh/Ddc/Pde1b	4
BP	GO:0042220	response to cocaine	5/607	43/28814	5.52	0.002028344	0.02035588	0.014739359	Crebbp/Drd1/Homer2/Slc1a3/Slc6a3	5
BP	GO:0048873	homeostasis of number of cells within a tissue	5/607	43/28814	5.52	0.002028344	0.02035588	0.014739359	Bax/Ikb2/Lipa/P2rx7/Rac1	5
BP	GO:0061462	protein localization to lysosome	5/607	43/28814	5.52	0.002028344	0.02035588	0.014739359	Clu/Kptn/Lamp2/Mea47/Zfyve16	5
BP	GO:0008361	regulation of cell size	12/607	214/28814	2.66	0.002060272	0.020580806	0.014902224	Akt1s1/Cobl/Dbn1/Dbnl/Dip2b/Dnm2/Fgf13/Map3k13/Ntrk3/P2rx7/Rac1/Sema...	12
BP	GO:0006576	cellular biogenic amine metabolic process	8/607	108/28814	3.52	0.002063681	0.020580806	0.014902224	Abhd4/Comt/Ddc/Drd1/Pde1b/Pnkd/Slc6a3/Th	8
BP	GO:0033555	multicellular organismal response to stress	8/607	108/28814	3.52	0.002063681	0.020580806	0.014902224	Asic1/Comt/Drd1/Ednrb/Mef2c/Prkar1b/Thbs4/Vdac3	8
BP	GO:0046467	membrane lipid biosynthetic process	9/607	133/28814	3.21	0.002078503	0.020685431	0.014977981	Abca8b/B4galt6/Hacd3/Kdsr/P2rx7/Pigt/Sccpdh/Smpd3/Smpd4	9
BP	GO:0001755	neural crest cell migration	6/607	63/28814	4.52	0.002095035	0.020720456	0.015003342	Ednrb/Efnb1/Nrp2/Phactr4/Sema7a/Sox8	6
BP	GO:0030865	cortical cytoskeleton organization	6/607	63/28814	4.52	0.002095035	0.020720456	0.015003342	Epb41/Plk1/Rac1/Rac2/Rhob/Rhof	6
BP	GO:0048260	positive regulation of receptor-mediated endocytosis	6/607	63/28814	4.52	0.002095035	0.020720456	0.015003342	Anxa2/Il6ra/Clu/Dnm2/Pla2g2/Ppm5	6
BP	GO:0009165	nucleotide biosynthetic process	12/607	215/28814	2.65	0.002141462	0.02112668	0.015297483	Adcy3/Adcy8/Ak2/Atp5pb/Cmny/Dcakd/Dip2a/Gucy1b1/Napri/Nme1/Pdk2/Stat3	12
BP	GO:0032388	positive regulation of intracellular transport	11/607	187/28814	2.79	0.002149375	0.02112668	0.015297483	Actn2/Anxa2/Bag3/Camk1/Flna/Kcnb1/Mk1/Mon/Pcm1/Pppn5/Sar1b	11
BP	GO:0042594	response to starvation	11/607	187/28814	2.79	0.002149375	0.02112668	0.015297483	Brsk1/Comt/Dap/Eloper1/Fo.../Kptn/Lamp2/Mapk3/P...	11
BP	GO:0043488	regulation of mRNA stability	10/607	160/28814	2.97	0.002168227	0.02126821	0.015399962	Apex1/Cnot7/Dcp1a/Dcps/Dhx36/Hnrnpab/Nocr/Paip1/Tut4/Vim	10
BP	GO:0032755	positive regulation of interleukin-6 production	8/607	109/28814	3.48	0.002186985	0.021408257	0.015501368	Arhgef2/Hmgb1/P2rx7/Pkg2/Stat3/Traf6/Tut4/Zbtb20	8
BP	GO:0009314	response to radiation	19/607	431/28814	2.09	0.002193478	0.021427904	0.015515594	Bax/Brsk1/Crebbp/Dhx36/Drd1/Gnb5/Kdm1a/Mmc/Msh2/Ndrg4/Nlgn3/Nucks1/Pcna/Pde1b/Ppp1cc/Rgs9/Rb...	19
BP	GO:2000146	negative regulation of cell motility	15/607	305/28814	2.33	0.00222098	0.021721683	0.015728315	Adgrg1/Apex1/Atp2b4/Dab2ip/Drd1/Gpihp4/Hmgb1/Igfbp5/Mef2c/Ndrg4/Osbpl8/...	15
BP	GO:0034101	erythrocyte homeostasis	10/607	161/28814	2.95	0.002269356	0.02189864	0.015856446	B2m/Bap1/Heph/Hmgb2/Isk3/Kdm1a/Smd1a/Slc25a5/Stat5...	10
BP	GO:0008347	glial cell migration	6/607	64/28814	4.45	0.002271339	0.02189864	0.015856446	Adgrg1/Cdk5r2/Dab2ip/Enro1/Hexb/Vim	6
BP	GO:0006895	Golgi to endosome transport	3/607	13/28814	10.95	0.002272569	0.02189864	0.015856446	Klhl20/Rab14/Wipi1	3
BP	GO:0071236	cellular response to antibiotic	3/607	13/28814	10.95	0.002272569	0.02189864	0.015856446	Mef2c/Pla2g4a/Rpl23	3
BP	GO:0042326	negative regulation of phosphorylation	18/607	400/28814	2.14	0.002273706	0.02189864	0.015856446	Actb/Akt1s1/Bax/Dab2ip/Dnaja3/Drd1/Dyrrl1/Enpp1/Grb10/Inpp5j/Ntrk3/Pik3ip1/Prkar1b/Prkar2b/Ptpn5/Pt...	18
BP	GO:0048041	focal adhesion assembly	7/607	86/28814	3.86	0.002273755	0.02189864	0.015856446	Actn2/Arhgap6/Pip5k1a/Rac1/Rac2/Sorbs1/Vcl	7
BP	GO:0005976	polysaccharide metabolic process	8/607	110/28814	3.45	0.002316002	0.022068399	0.015979366	Enpp1/Grb10/Hmgb1/Phka1/Pkb/Ppp1cc/Smpd3/Sorb...	8
BP	GO:0014074	response to purine-containing compound	8/607	110/28814	3.45	0.002316002	0.022068399	0.015979366	Enpp1/Gnal/Hmgcs1/Igfbp5/P2rx7/Pkg2/Slc6a3/Tmem38a	8
BP	GO:0034446	substrate adhesion-dependent cell spreading	8/607	110/28814	3.45	0.002316002	0.022068399	0.015979366	Dbn1/Dnm2/Flna/Parvb/Pdpn/Rac1/Rac2/Vamp3	8
BP	GO:0014829	vascular associated smooth muscle contraction	4/607	27/28814	7.03	0.002328339	0.022068399	0.015979366	Atp2b1/Bbs2/Ednrb/Smpd3	4
BP	GO:0031338	regulation of vesicle fusion	4/607	27/28814	7.03	0.002328339	0.022068399	0.015979366	Cplx1/Grik5/Pla2g4a/Rab3a	4
BP	GO:0070841	inclusion body assembly	4/607	27/28814	7.03	0.002328339	0.022068399	0.015979366	Clu/Dnajb6/Hap1/Hspa2	4
BP	GO:0090382	phagosome maturation	4/607	27/28814	7.03	0.002328339	0.022068399	0.015979366	P2rx7/Rab14/Rab31/Rab34	4
BP	GO:2000310	regulation of NMDA receptor activity	4/607	27/28814	7.03	0.002328339	0.022068399	0.015979366	Mef2c/Nlgn3/Rgs9/Slc6a9	4

BP	GO:0006020	inositol metabolic process	2/361	12/28814	13.3	0.009508308	0.077381493	0.060183007	Itpka/Ppip5k2	2
BP	GO:0006086	acetyl-CoA biosynthetic process from pyruvate	2/361	12/28814	13.3	0.009508308	0.077381493	0.060183007	Pdk1/Pdk3	2
BP	GO:0010649	regulation of cell communication by electrical coupling	2/361	12/28814	13.3	0.009508308	0.077381493	0.060183007	Ank3/Camk2d	2
BP	GO:0010998	regulation of translational initiation by eIF2 alpha phosphorylation	2/361	12/28814	13.3	0.009508308	0.077381493	0.060183007	Eif2ak4/Hbb-b1	2
BP	GO:0016558	protein import into peroxisome matrix	2/361	12/28814	13.3	0.009508308	0.077381493	0.060183007	Pex1/Pex13	2
BP	GO:0021683	cerebellar granular layer morphogenesis	2/361	12/28814	13.3	0.009508308	0.077381493	0.060183007	Atp2b2/Nrxn1	2
BP	GO:0060433	bronchus development	2/361	12/28814	13.3	0.009508308	0.077381493	0.060183007	Foxp1/Foxp4	2
BP	GO:0072697	protein localization to cell cortex	2/361	12/28814	13.3	0.009508308	0.077381493	0.060183007	Epb41l2/Ppp1r9b	2
BP	GO:0098693	regulation of synaptic vesicle cycle	2/361	12/28814	13.3	0.009508308	0.077381493	0.060183007	Nrxn1/Syn1	2
BP	GO:1900244	positive regulation of synaptic vesicle endocytosis	2/361	12/28814	13.3	0.009508308	0.077381493	0.060183007	Ppp3cc/Sh3gl1	2
BP	GO:1990253	cellular response to leucine starvation	2/361	12/28814	13.3	0.009508308	0.077381493	0.060183007	Eif2ak4/Gcn1	2
BP	GO:0042594	response to starvation	7/361	187/28814	2.99	0.009521923	0.077381493	0.060183007	Cadps2/Eif2ak4/Gcn1/Glul/Pck2/Prkaa1/Xpr1	7
BP	GO:0048813	dendrite morphogenesis	7/361	187/28814	2.99	0.009521923	0.077381493	0.060183007	Adam10/Camk2a/Itpka/Numb/Ppp3ca/Rock2/Tpbg	7
BP	GO:0009100	glycoprotein metabolic process	10/361	332/28814	2.4	0.009549289	0.077431819	0.060222148	Alg2/Cog3/Crppa/Extl2/Fbxo2/Glce/Ngly1/Plcb1/Pmm2/St8sia3	10
BP	GO:0007160	cell-matrix adhesion	8/361	234/28814	2.73	0.009683508	0.078173488	0.060798977	Adam15/Coro1c/Epdr1/L1cam/Lims1/Pxn/Sirpa/Sorbs1	8
BP	GO:0030073	insulin secretion	8/361	234/28814	2.73	0.009683508	0.078173488	0.060798977	Cplx1/Cplx3/Glul/Nrxn1/Ppp3ca/Rab11b/Rims2/Stx1a	8
BP	GO:0044282	small molecule catabolic process	10/361	334/28814	2.39	0.009931885	0.080001991	0.062221084	Abhd3/Acaa1a/Bcat2/Gcdh/Haghl/Inpp5b/Nudt3/Pck2/Pex13/Ppat	10
BP	GO:0022900	electron transport chain	5/361	104/28814	3.84	0.010057902	0.080480108	0.062592937	Bdnf/Chchd2/Coq7/Ndor1/Ndufa12	5
BP	GO:0055013	cardiac muscle cell development	5/361	104/28814	3.84	0.010057902	0.080480108	0.062592937	Camk2d/Foxp1/Slc8a1/Smad4/Zmpste24	5
BP	GO:0008203	cholesterol metabolic process	6/361	145/28814	3.3	0.01007927	0.080480108	0.062592937	Abca1/Cat/Dhcr7/Mvd/Mvk/Prkaa1	6
BP	GO:0051928	positive regulation of calcium ion transport	6/361	145/28814	3.3	0.01007927	0.080480108	0.062592937	Atp2b1/Atp2b2/Cacnb3/Calm1/Camk2a/S100a1	6
BP	GO:0042180	cellular ketone metabolic process	8/361	236/28814	2.71	0.010158845	0.080938769	0.062949658	Atcay/Clcn2/Coq6/Coq7/Haghl/Ndufa9/Pdk1/Pdk3	8
BP	GO:0009154	purine ribonucleotide catabolic process	3/361	36/28814	6.65	0.010255866	0.081357268	0.063275143	Ampd3/Nudt3/Nudt4	3
BP	GO:0070542	response to fatty acid	3/361	36/28814	6.65	0.010255866	0.081357268	0.063275143	Foxp1/Pdk3/Plcb1	3
BP	GO:0032233	positive regulation of actin filament bundle assembly	4/361	68/28814	4.7	0.010518696	0.083081799	0.064616387	Cd47/Pxn/Rock2/Sorbs3	4
BP	GO:0042775	mitochondrial ATP synthesis coupled electron transport	4/361	68/28814	4.7	0.010518696	0.083081799	0.064616387	Bdnf/Chchd2/Coq7/Ndufa12	4
BP	GO:0031346	positive regulation of cell projection organization	12/361	443/28814	2.16	0.010609372	0.083306452	0.064791109	Apbb1/Atp8a2/Bdnf/Coro1c/Gprc5b/Itpka/L1cam/Mien1/Numb/Pcp4/Plxnc1/Tenm3	12
BP	GO:0000302	response to reactive oxygen species	7/361	191/28814	2.93	0.010615479	0.083306452	0.064791109	Cat/Ccs/Foxp1/Prkaa1/Sirpa/Slc4a1/Slc8a1	7
BP	GO:0051648	vesicle localization	7/361	191/28814	2.93	0.010615479	0.083306452	0.064791109	Atp9a/Cnih2/Kif1b/Nrxn1/Preb/Rab11b/Syn1	7
BP	GO:0010469	regulation of signaling receptor activity	6/361	147/28814	3.26	0.010735534	0.083709698	0.065104732	Bud31/Cnih2/Gsg1l/Nptx1/Nrxn1/Shisa9	6
BP	GO:0043467	regulation of generation of precursor metabolites and energy	6/361	147/28814	3.26	0.010735534	0.083709698	0.065104732	Chchd2/Cox7a1/Prkaa1/Slc4a1/Sorbs1/Stat3	6
BP	GO:1901136	carbohydrate derivative catabolic process	6/361	147/28814	3.26	0.010735534	0.083709698	0.065104732	Ampd3/Fbxo2/Ngly1/Nt5m/Nudt3/Nudt4	6
BP	GO:0032006	regulation of TOR signaling	5/361	106/28814	3.76	0.010863305	0.084073638	0.065387784	Klhl22/Lamtor1/Prkaa1/Sec13/Zmpste24	5
BP	GO:0042093	T-helper cell differentiation	4/361	69/28814	4.63	0.011058279	0.084073638	0.065387784	Foxp1/Rc3h1/Sema4a/Stat3	4
BP	GO:0007031	peroxisome organization	3/361	37/28814	6.47	0.011058918	0.084073638	0.065387784	Pex1/Pex13/Pex19	3
BP	GO:0010661	positive regulation of muscle cell apoptotic process	3/361	37/28814	6.47	0.011058918	0.084073638	0.065387784	Camk2a/Camk2d/Smad4	3
BP	GO:0030808	regulation of nucleotide biosynthetic process	3/361	37/28814	6.47	0.011058918	0.084073638	0.065387784	Pdk1/Pdk3/Stat3	3
BP	GO:0090075	relaxation of muscle	3/361	37/28814	6.47	0.011058918	0.084073638	0.065387784	Atp2a2/Camk2d/Slc8a1	3
BP	GO:1900371	regulation of purine nucleotide biosynthetic process	3/361	37/28814	6.47	0.011058918	0.084073638	0.065387784	Pdk1/Pdk3/Stat3	3
BP	GO:0001731	formation of translation preinitiation complex	2/361	13/28814	12.28	0.011144709	0.084073638	0.065387784	Denr/Dhx29	2
BP	GO:0009226	nucleotide-sugar biosynthetic process	2/361	13/28814	12.28	0.011144709	0.084073638	0.065387784	Pmm2/Uap1	2
BP	GO:0030953	astral microtubule organization	2/361	13/28814	12.28	0.011144709	0.084073638	0.065387784	Dlg1/Tppp	2
BP	GO:0060368	regulation of Fc receptor mediated stimulatory signaling pathway	2/361	13/28814	12.28	0.011144709	0.084073638	0.065387784	Cd47/Rabgef1	2
BP	GO:0060965	negative regulation of miRNA-mediated gene silencing	2/361	13/28814	12.28	0.011144709	0.084073638	0.065387784	Ppp3ca/Zmpste24	2
BP	GO:1900121	negative regulation of receptor binding	2/361	13/28814	12.28	0.011144709	0.084073638	0.065387784	Adam15/Atp2a2	2
BP	GO:1904181	positive regulation of membrane depolarization	2/361	13/28814	12.28	0.011144709	0.084073638	0.065387784	Ank3/Cck	2
BP	GO:2001223	negative regulation of neuron migration	2/361	13/28814	12.28	0.011144709	0.084073638	0.065387784	Adgrg1/Stat3	2
BP	GO:0040013	negative regulation of locomotion	10/361	340/28814	2.35	0.011150045	0.084073638	0.065387784	Abhd6/Adam15/Adgrg1/Coro1c/Padi2/Plcb1/Rabgef1/Sema4a/Sema4f/Stat3	10
BP	GO:0001657	ureteric bud development	5/361	107/28814	3.73	0.011282057	0.084373176	0.065620748	Bdnf/Cat/Dlg1/Maged1/Smad4	5
BP	GO:0034766	negative regulation of ion transmembrane transport	5/361	107/28814	3.73	0.011282057	0.084373176	0.065620748	Ank3/Cacnb3/Calm1/Camk2d/Cbarp	5
BP	GO:0072163	mesonephric epithelium development	5/361	107/28814	3.73	0.011282057	0.084373176	0.065620748	Bdnf/Cat/Dlg1/Maged1/Smad4	5
BP	GO:0072164	mesonephric tubule development	5/361	107/28814	3.73	0.011282057	0.084373176	0.065620748	Bdnf/Cat/Dlg1/Maged1/Smad4	5
BP	GO:0019233	sensory perception of pain	6/361	149/28814	3.21	0.011421517	0.084895298	0.066026825	Cacnb3/Cck/Kcna1/Kcna2/Kcnip3/Phf24	6
BP	GO:0050657	nucleic acid transport	6/361	149/28814	3.21	0.011421517	0.084895298	0.066026825	Ahctf1/Alkbh5/Alyref/Nup188/Rae1/Sec13	6
BP	GO:0050658	RNA transport	6/361	149/28814	3.21	0.011421517	0.084895298	0.066026825	Ahctf1/Alkbh5/Alyref/Nup188/Rae1/Sec13	6
BP	GO:0042593	glucose homeostasis	9/361	291/28814	2.47	0.011595186	0.085467716	0.06647202	Gprc5b/Nrxn1/Pck2/Pdk3/Ppp3ca/Prkaa1/Rab11b/Smad4/Stat3	9
BP	GO:0002294	CD4-positive, alpha-beta T cell differentiation involved in immune response	4/361	70/28814	4.56	0.011615383	0.085467716	0.06647202	Foxp1/Rc3h1/Sema4a/Stat3	4
BP	GO:0015800	acidic amino acid transport	4/361	70/28814	4.56	0.011615383	0.085467716	0.06647202	Bdnf/Cck/Slc12a2/Slc17a7	4
BP	GO:0042773	ATP synthesis coupled electron transport	4/361	70/28814	4.56	0.011615383	0.085467716	0.06647202	Bdnf/Chchd2/Coq7/Ndufa12	4
BP	GO:0043462	regulation of ATP-dependent activity	4/361	70/28814	4.56	0.011615383	0.085467716	0.06647202	Dnajc24/Napa/Oxa1l/Pxk	4
BP	GO:0060562	epithelial tube morphogenesis	11/361	395/28814	2.22	0.011673582	0.085664074	0.066624737	Aldh1a2/Coq7/Dlg1/Foxp1/Maged1/Ppp3r1/Pxn/Rock2/Slc12a2/Smad4/Vasp	11
BP	GO:0048762	mesenchymal cell differentiation	8/361	242/28814	2.64	0.011688918	0.085664074	0.066624737	Adam15/Aldh1a2/Coro1c/Ppp3r1/Rock2/Sema4a/Sema4f/Smad4	8
BP	GO:0033500	carbohydrate homeostasis	9/361	292/28814	2.46	0.01183664	0.086497843	0.067273195	Gprc5b/Nrxn1/Pck2/Pdk3/Ppp3ca/Prkaa1/Rab11b/Smad4/Stat3	9
BP	GO:0086004	regulation of cardiac muscle cell contraction	3/361	38/28814	6.3	0.011897297	0.086497843	0.067273195	Atp1a1/Atp2a2/Dlg1	3
BP	GO:1901385	regulation of voltage-gated calcium channel activity	3/361	38/28814	6.3	0.011897297	0.086497843	0.067273195	Cacnb3/Cbarp/S100a1	3
BP	GO:1901661	quinone metabolic process	3/361	38/28814	6.3	0.011897297	0.086497843	0.067273195	Coq6/Coq7/Ndufa9	3
BP	GO:0006906	vesicle fusion	5/361	109/28814	3.66	0.012152287	0.087928144	0.068385604	Cplx1/Cplx3/Stx1a/Stx2/Vps8	5

BP	GO:0045765	regulation of angiogenesis	15/607	307/28814	2.32	0.002371367	0.022431723	0.016242443	Atp2b4/Dab2ip/Gab1/Hk2/Hmgb1/Jcad/Mydgf/Nras/Ppp1r16b/Prkcb/Rhob/Sparc/Stat1/Stat3/Stim1	15
BP	GO:0006112	energy reserve metabolic process	7/607	87/28814	3.82	0.002428732	0.022928958	0.016602483	Enpp1/Grb10/Hmgb1/Phka1/Phkb/Ppp1cc/Sorbs1	7
BP	GO:0051259	protein complex oligomerization	13/607	248/28814	2.49	0.002476197	0.023330953	0.016893561	Adcy8/B2m/Kcna3/Kcnb1/Kcnc1/Kcnd2/Kctd4/Mcu/Prmt8/Sigmar1/Ugdh/Vasp/Y	13
BP	GO:0072583	clathrin-dependent endocytosis	5/607	45/28814	5.27	0.002487297	0.023343455	0.016902613	Dnm2/Fcho1/Picalm/Sh3gl3/Unc119	5
BP	GO:0097178	ruffle assembly	5/607	45/28814	5.27	0.002487297	0.023343455	0.016902613	Aif1l/Dbnl/Pip5k1a/Rac1/Rcc2	5
BP	GO:0071216	cellular response to biotic stimulus	16/607	340/28814	2.23	0.002512994	0.023538381	0.017043756	Abca1/B2m/Chmp5/Dab2ip/Gbp2/Hmgb1/Irgm1/Mapk3/Mef2c/Pdcd4/Plcg2/Sirpa/Stat1/Traf6/Tut4/Vim	16
BP	GO:0043122	regulation of I-kappaB kinase/NF-kappaB signaling	11/607	191/28814	2.73	0.00253384	0.023687192	0.017151508	Brd4/Dab2ip/Dhx36/Dnaja3/Plcg2/Prkcb/S100a4/Stat1/Traf6/Trim25/Ube2i	11
BP	GO:0031623	receptor internalization	9/607	137/28814	3.12	0.002542305	0.023719901	0.017175191	Ache/Dnm2/Hap1/Pacsin1/Picalm/Plcg2/Ptpn5/Rab31/Snap25	9
BP	GO:0001738	morphogenesis of a polarized epithelium	7/607	88/28814	3.78	0.002591713	0.024086793	0.017440852	Actb/Ajap1/Brsk1/Msn/Ptk7/Rac1/Vangl2	7
BP	GO:0050766	positive regulation of phagocytosis	7/607	88/28814	3.78	0.002591713	0.024086793	0.017440852	Camk1d/Dnm2/Plcg2/Rab31/Rap1gap/Sirpa/Sod1	7
BP	GO:0003299	muscle hypertrophy in response to stress	4/607	28/28814	6.78	0.002671655	0.024638019	0.017839986	Atp2b4/Foxo1/Lmna/Mef2c	4
BP	GO:0014898	cardiac muscle hypertrophy in response to stress	4/607	28/28814	6.78	0.002671655	0.024638019	0.017839986	Atp2b4/Foxo1/Lmna/Mef2c	4
BP	GO:0015874	norepinephrine transport	4/607	28/28814	6.78	0.002671655	0.024638019	0.017839986	Actb/Kcnb1/Ptgs1/Slc6a3	4
BP	GO:0098698	postsynaptic specialization assembly	4/607	28/28814	6.78	0.002671655	0.024638019	0.017839986	Gap43/Nlgn3/Nptx1/Ntrk3	4
BP	GO:1901342	regulation of vasculature development	15/607	311/28814	2.29	0.002680864	0.024675311	0.017866988	Atp2b4/Dab2ip/Gab1/Hk2/Hmgb1/Jcad/Mydgf/Nras/Ppp1r16b/Prkcb/Rhob/Sparc/Stat1/Stat3/Stim1	15
BP	GO:0010675	regulation of cellular carbohydrate metabolic process	10/607	165/28814	2.88	0.002712439	0.024917926	0.018042662	Arpp19/Enpp1/Foxo1/Grb10/Hmgb1/Igfbp4/Pdk2/Sorbs1/Stat3/Ugt1a1	10
BP	GO:0032881	regulation of polysaccharide metabolic process	5/607	46/28814	5.16	0.002743031	0.025150597	0.018211135	Enpp1/Grb10/Hmgb1/Smpd3/Sorbs1	5
BP	GO:0034644	cellular response to UV	7/607	89/28814	3.73	0.002762966	0.025236494	0.018273331	Bax/Crebbp/Dhx36/Kdm1a/Mme/Pcna/Yy1	7
BP	GO:1904427	positive regulation of calcium ion transmembrane transport	7/607	89/28814	3.73	0.002762966	0.025236494	0.018273331	Bax/Drd1/Gjc2/Hap1/Hspa2/Plcg2/Stim1	7
BP	GO:0032675	regulation of interleukin-6 production	10/607	166/28814	2.86	0.002833359	0.025712386	0.018617917	Arhgef2/Hmgb1/P2rx7/Plcg2/Ptpn6/Sirpa/Stat3/Traf6/Tut4/Zbtb20	10
BP	GO:0031915	positive regulation of synaptic plasticity	3/607	14/28814	10.17	0.002847363	0.025712386	0.018617917	Adcy8/Dbn1/Snap25	3
BP	GO:0032341	aldosterone metabolic process	3/607	14/28814	10.17	0.002847363	0.025712386	0.018617917	Clcn2/Dkk3/Ednrb	3
BP	GO:0036296	response to increased oxygen levels	3/607	14/28814	10.17	0.002847363	0.025712386	0.018617917	Atp6v1g1/Foxo1/Pdpn	3
BP	GO:0045176	apical protein localization	3/607	14/28814	10.17	0.002847363	0.025712386	0.018617917	Actb/Rab14/Vangl2	3
BP	GO:0060263	regulation of respiratory burst	3/607	14/28814	10.17	0.002847363	0.025712386	0.018617917	Camk1d/Rac1/Rac2	3
BP	GO:0032608	interferon-beta production	6/607	67/28814	4.25	0.002867598	0.02579758	0.018679605	Crebbp/Hmgb1/Hmgb2/Sirpa/Yy1/Zbtb20	6
BP	GO:0032648	regulation of interferon-beta production	6/607	67/28814	4.25	0.002867598	0.02579758	0.018679605	Crebbp/Hmgb1/Hmgb2/Sirpa/Yy1/Zbtb20	6
BP	GO:0000723	telomere maintenance	9/607	140/28814	3.05	0.002941182	0.026374439	0.019097299	Apex1/Dhx36/Exosc10/Lmna/Mapk3/Pcna/Smg5/Tnks/Yy1	9
BP	GO:0046209	nitric oxide metabolic process	7/607	90/28814	3.69	0.002942762	0.026374439	0.019097299	Atp2b4/Clu/Dnm2/Dynll1/Rac1/Sirpa/Smpd3	7
BP	GO:0044242	cellular lipid catabolic process	12/607	224/28814	2.54	0.002996609	0.02680674	0.019410321	Abcd3/Echdc1/Gpcpd1/Hexb/Nudt7/Pla2g4a/Pla2g7/Plcg2/Pld1/Sgpl1/Smpd3/Smpd4	12
BP	GO:0014887	cardiac muscle adaptation	4/607	29/28814	6.55	0.003048114	0.027165746	0.019670271	Atp2b4/Foxo1/Lmna/Mef2c	4
BP	GO:0030318	melanocyte differentiation	4/607	29/28814	6.55	0.003048114	0.027165746	0.019670271	Bloc1s6/Ednrb/Enpp1/Mef2c	4
BP	GO:0062013	positive regulation of small molecule metabolic process	10/607	168/28814	2.83	0.003088107	0.027290246	0.01976042	Arpp19/Comt/Foxo1/Hmgb1/Nucb2/P2rx7/Pla2g4a/Sorbs1/Stat3/Zbtb20	10
BP	GO:0022029	telencephalon cell migration	6/607	68/28814	4.19	0.003090211	0.027290246	0.01976042	Adgrg1/Cdk5r2/Dab2ip/Drd1/Fgf13/Rac1	6
BP	GO:0032481	positive regulation of type I interferon production	6/607	68/28814	4.19	0.003090211	0.027290246	0.01976042	Dhx36/Hmgb1/Hmgb2/Plcg2/Stat1/Zbtb20	6
BP	GO:0050433	regulation of catecholamine secretion	6/607	68/28814	4.19	0.003090211	0.027290246	0.01976042	Kcnb1/Prkcb/Ptgs1/Rab3a/Sncg/Syt17	6
BP	GO:0017148	negative regulation of translation	11/607	196/28814	2.66	0.003090647	0.027290246	0.01976042	Cnot7/Dcp1a/Dcps/Dhx36/Eif6/Igfbp5/Mrpl13/Noct/Paip1/Tia1/Tut4	11
BP	GO:0015914	phospholipid transport	7/607	91/28814	3.65	0.003131378	0.027548053	0.019947093	Abca1/Ano3/Osbpl8/P2rx7/Pitpnc1/Xkr5/Xkr9	7
BP	GO:1903578	regulation of ATP metabolic process	7/607	91/28814	3.65	0.003131378	0.027548053	0.019947093	Cox7a1/Cox7a2/Eif6/P2rx7/Slc25a33/Stat3/Zbtb20	7
BP	GO:0006006	glucose metabolic process	12/607	226/28814	2.52	0.003219913	0.028274861	0.020473363	Arpp19/Eno2/Enpp1/Foxo1/Ganc/Grb10/Hk2/Hmgb1/Igfbp4/Pdk2/Sorbs1/Tpi1	12
BP	GO:0030219	megakaryocyte differentiation	5/607	48/28814	4.94	0.003310726	0.029018968	0.021012159	Eif6/Hmgb2/Kdm1a/Mef2c/Ptpn6	5
BP	GO:2001057	reactive nitrogen species metabolic process	7/607	92/28814	3.61	0.003329089	0.029055243	0.021038425	Atp2b4/Clu/Dnm2/Dynll1/Rac1/Sirpa/Smpd3	7
BP	GO:0007188	adenylate cyclase-modulating G protein-coupled receptor signaling pathway	12/607	227/28814	2.51	0.003336506	0.029055243	0.021038425	Abca1/Adcy3/Adcy8/Adgrg1/Atp2b4/Drd1/Flna/Gnal/Grk5/Htr4/Pde10a/Pde4a	12
BP	GO:0010810	regulation of cell-substrate adhesion	12/607	227/28814	2.51	0.003336506	0.029055243	0.021038425	Ajap1/Arhgap6/Dbn1/Dnm2/Flna/Lgals1/Pdpn/Rac1/Rac2/Rcc2/Rsu1/Vcl	12
BP	GO:0050905	neuromuscular process	11/607	198/28814	2.64	0.003339194	0.029055243	0.021038425	Aplp2/Camk2b/Csmd1/Dbn1/Fabp7/Hexb/Nlgn3/Opa3/Pnkd/Slc1a3/Slc6a3	11
BP	GO:0034614	cellular response to reactive oxygen species	9/607	143/28814	2.99	0.0033879	0.029372046	0.021267817	Apex1/Endog/Mapk3/Pdk2/Rhob/Sigmar1/Sirpa/Smpd3/Sod1	9
BP	GO:1903313	positive regulation of mRNA metabolic process	9/607	143/28814	2.99	0.0033879	0.029372046	0.021267817	Celf4/Cnot7/Dcp1a/Dcps/Dhx36/Ncbp2/Noct/Paip1/Tut4	9
BP	GO:0007041	lysosomal transport	8/607	117/28814	3.25	0.003394503	0.029375983	0.021270668	Chmp5/Clu/Lamp2/Rab34/Rhob/Rnf157/Tmem106b/Zfyve16	8
BP	GO:0014912	negative regulation of smooth muscle cell migration	4/607	30/28814	6.33	0.003459215	0.029720626	0.021520218	Apex1/Igfbp5/Mef2c/Ndrg4	4
BP	GO:0030149	sphingolipid catabolic process	4/607	30/28814	6.33	0.003459215	0.029720626	0.021520218	Hexb/Sgpl1/Smpd3/Smpd4	4
BP	GO:0045648	positive regulation of erythrocyte differentiation	4/607	30/28814	6.33	0.003459215	0.029720626	0.021520218	Hmgb2/Kdm1a/Stat1/Stat3	4
BP	GO:1900078	positive regulation of cellular response to insulin stimulus	4/607	30/28814	6.33	0.003459215	0.029720626	0.021520218	Marcks/Nucks1/Osbpl8/Sorbs1	4
BP	GO:0021537	telencephalon development	13/607	258/28814	2.39	0.00347213	0.029732016	0.021528465	Adgrg1/Bax/Bbs2/Ccdc85c/Cdh2/Cdk5r2/Dab2ip/Drd1/Fgf13/Phactr1/Rac1/Rtn4rl2/Sema7a	13
BP	GO:0002730	regulation of dendritic cell cytokine production	3/607	15/28814	9.49	0.003503892	0.029732016	0.021528465	Dhx36/Jak3/Plcg2	3
BP	GO:0015936	coenzyme A metabolic process	3/607	15/28814	9.49	0.003503892	0.029732016	0.021528465	Coasy/Dcakd/Nudt7	3
BP	GO:0021819	layer formation in cerebral cortex	3/607	15/28814	9.49	0.003503892	0.029732016	0.021528465	Adgrg1/Cdk5r2/Dab2ip	3
BP	GO:0032026	response to magnesium ion	3/607	15/28814	9.49	0.003503892	0.029732016	0.021528465	Enpp1/Plcg2/Smpd3	3
BP	GO:0032530	regulation of microvillus organization	3/607	15/28814	9.49	0.003503892	0.029732016	0.021528465	Pld1/Pls1/Rap1gap	3
BP	GO:1902806	regulation of cell cycle G1/S phase transition	10/607	171/28814	2.78	0.003504108	0.029732016	0.021528465	Apex1/Atp2b4/Crebbp/Gjc2/Larp7/Pdpn/Plcg2/Psme1/Psme2/Ptpn6	10
BP	GO:0099072	regulation of postsynaptic membrane neurotransmitter receptor levels	7/607	93/28814	3.57	0.003536176	0.029950913	0.021686965	Cplx1/Frrs1l/Hap1/Nptx1/Pacsin1/Snap25/Stx3	7
BP	GO:1905475	regulation of protein localization to membrane	11/607	200/28814	2.61	0.003603519	0.030467276	0.022060855	Actb/Atp2b4/Camk2b/Cdh2/Dbn1/Kcnb1/Picalm/Pls1/Shisa7/Sorbs1/Stx3	11
BP	GO:0072666	establishment of protein localization to vacuole	5/607	49/28814	4.84	0.003624171	0.030533796	0.022109021	Clu/Lamp2/Mon1b/Stam/Zfyve16	5
BP	GO:0002293	alpha-beta T cell differentiation involved in immune response	4/361	71/28814	4.5	0.012190202	0.087928144	0.068385604	Foxp1/Rc3h1/Sema4a/Stat3	4
BP	GO:0030239	myofibril assembly	4/361	71/28814	4.5	0.012190202	0.087928144	0.068385604	Csrp1/Foxp1/Smad4/Tmod1	4
BP	GO:0050432	catecholamine secretion	4/361	71/28814	4.5	0.012190202	0.087928144	0.068385604	Cadps/Kcna2/Pcp4/Stx1a	4
BP	GO:0072001	renal system development	10/361	345/28814	2.31	0.012249036	0.088178589	0.068580386	Aldh1a2/Bdnf/Cat/Dlg1/Kirrel3/Maged1/Nfia/Sec61a1/Smad4/Zmpste24	10
BP	GO:0030900	forebrain development	11/361	398/28814	2.21	0.012293152	0.088322309	0.068692163	Adgrg1/Aldh1a2/Kcna1/Kirrel3/Mboat7/Mrtfa/Numb/Pex13/Plcb1/Rtn4rl1/Trappc9	11
BP	GO:0051236	establishment of RNA localization	6/361	152/28814	3.15	0.012507669	0.089654758	0.069728467	Ahctf1/Alkbh5/Alyref/Nup188/Rae1/Sec13	6
BP	GO:0001823	mesonephros development	5/361	110/28814	3.63	0.01260401	0.089654758	0.069728467	Bdnf/Cat/Dlg1/Maged1/Smad4	5
BP	GO:0055006	cardiac cell development	5/361	110/28814	3.63	0.01260401	0.089654758	0.069728467	Camk2d/Foxp1/Slc8a1/Smad4/Zmpste24	5
BP	GO:0051481	negative regulation of cytosolic calcium ion concentration	3/361	39/28814	6.14	0.012771243	0.089654758	0.069728467	Atp2b1/Calm1/Slc8a1	3
BP	GO:1905145	cellular response to acetylcholine	3/361	39/28814	6.14	0.012771243	0.089654758	0.069728467	Plcb1/Rock2/Sorbs1	3
BP	GO:0002287	alpha-beta T cell activation involved in immune response	4/361	72/28814	4.43	0.012782927	0.089654758	0.069728467	Foxp1/Rc3h1/Sema4a/Stat3	4
BP	GO:0055002	striated muscle cell development	4/361	72/28814	4.43	0.012782927	0.089654758	0.069728467	Csrp1/Foxp1/Smad4/Tmod1	4
BP	GO:0010882	regulation of cardiac muscle contraction by calcium ion signaling	2/361	14/28814	11.4	0.012895379	0.089654758	0.069728467	Atp2a2/Slc8a1	2
BP	GO:0016081	synaptic vesicle docking	2/361	14/28814	11.4	0.012895379	0.089654758	0.069728467	Camk2a/Rims2	2
BP	GO:0030644	cellular chloride ion homeostasis	2/361	14/28814	11.4	0.012895379	0.089654758	0.069728467	Ckb/Slc12a2	2
BP	GO:0031054	pre-miRNA processing	2/361	14/28814	11.4	0.012895379	0.089654758	0.069728467	Prkra/Tut7	2
BP	GO:0060149	negative regulation of post-transcriptional gene silencing	2/361	14/28814	11.4	0.012895379	0.089654758	0.069728467	Ppp3ca/Zmpste24	2
BP	GO:0060967	negative regulation of gene silencing by RNA	2/361	14/28814	11.4	0.012895379	0.089654758	0.069728467	Ppp3ca/Zmpste24	2
BP	GO:0061140	lung secretory cell differentiation	2/361	14/28814	11.4	0.012895379	0.089654758	0.069728467	Foxp1/Foxp4	2
BP	GO:0097709	connective tissue replacement	2/361	14/28814	11.4	0.012895379	0.089654758	0.069728467	Ppp3ca/Rock2	2
BP	GO:0099151	regulation of postsynaptic density assembly	2/361	14/28814	11.4	0.012895379	0.089654758	0.069728467	Nptx1/Nrxn1	2
BP	GO:1900369	negative regulation of post-transcriptional gene silencing by RNA	2/361	14/28814	11.4	0.012895379	0.089654758	0.069728467	Ppp3ca/Zmpste24	2
BP	GO:0008344	adult locomotory behavior	5/361	111/28814	3.6	0.013066965	0.090675315	0.0705222	Cend1/Oxr1/Scn8a/Tmod1/Zmpste24	5
BP	GO:0048638	regulation of developmental growth	11/361	402/28814	2.18	0.013157837	0.091132972	0.07087814	Atp8a2/Bdnf/Dlg1/Foxp1/L1cam/Plcb1/Rims2/Sema4a/Sema4f/Stat3/Zmpste24	11
BP	GO:0002790	peptide secretion	9/361	298/28814	2.41	0.013365038	0.092329226	0.071808519	Abca1/Cplx1/Cplx3/Glul/Nrxn1/Ppp3ca/Rab11b/Rims2/Stx1a	9
BP	GO:0032102	negative regulation of response to external stimulus	11/361	403/28814	2.18	0.013381047	0.092329226	0.071808519	Cacnb3/Cck/Mvk/Nlrx1/Nrxn1/Padi2/Rabgef1/Rtn4rl1/Sema4a/Sema4f/Slc12a2	11
BP	GO:0021987	cerebral cortex development	5/361	112/28814	3.56	0.01354127	0.092996998	0.072327876	Adgrg1/Mboat7/Pex13/Plcb1/Trappc9	5
BP	GO:0043266	regulation of potassium ion transport	5/361	112/28814	3.56	0.01354127	0.092996998	0.072327876	Ank3/Dlg1/Kcna1/Kcnab2/Kcnip3	5
BP	GO:0006195	purine nucleotide catabolic process	3/361	40/28814	5.99	0.013680969	0.092996998	0.072327876	Ampd3/Nudt3/Nudt4	3
BP	GO:0031297	replication fork processing	3/361	40/28814	5.99	0.013680969	0.092996998	0.072327876	Alyref/Cdk9/Smarcal1	3
BP	GO:0051154	negative regulation of striated muscle cell differentiation	3/361	40/28814	5.99	0.013680969	0.092996998	0.072327876	Bdnf/Foxp1/Smad4	3
BP	GO:0060259	regulation of feeding behavior	3/361	40/28814	5.99	0.013680969	0.092996998	0.072327876	Cck/Eif2ak4/Stat3	3
BP	GO:1903670	regulation of sprouting angiogenesis	3/361	40/28814	5.99	0.013680969	0.092996998	0.072327876	Glul/S100a1/Tjp1	3
BP	GO:0006470	protein dephosphorylation	8/361	249/28814	2.56	0.013681265	0.092996998	0.072327876	Arpp19/Calm1/Ppp1r9b/Ppp3ca/Ppp3cc/Ppp3r1/Ppp4c/Rock2	8
BP	GO:0046578	regulation of Ras protein signal transduction	7/361	201/28814	2.78	0.013745115	0.093257674	0.072530614	Abca1/Adgrg1/Camk2d/Cyth3/Dennd1a/Iqsec3/Rabgef1	7
BP	GO:0090174	organelle membrane fusion	5/361	113/28814	3.53	0.014027042	0.094994242	0.073881221	Cplx1/Cplx3/Stx1a/Stx2/Vps8	5
BP	GO:0001558	regulation of cell growth	12/361	461/28814	2.08	0.014139698	0.095580178	0.074336929	Adam10/Adam15/Bdnf/Foxp1/Klhl22/L1cam/Lamtor1/Ppp1r9b/Rims2/Sema4a/Sema4f/Smad4	12
BP	GO:0042886	amide transport	10/361	353/28814	2.26	0.014174891	0.095641282	0.074384452	Abca1/Cplx1/Cplx3/Glul/Nrxn1/Ppp3ca/Rab11b/Rims2/Slc26a6/Stx1a	10
BP	GO:0070482	response to oxygen levels	9/361	302/28814	2.38	0.014462316	0.096715299	0.075219762	Alkbh5/Camk2d/Cat/Chchd2/Pdk1/Pdk3/Rock2/Slc8a1/Smad4	9
BP	GO:0060964	regulation of miRNA-mediated gene silencing	3/361	41/28814	5.84	0.014626661	0.096715299	0.075219762	Ppp3ca/Stat3/Zmpste24	3
BP	GO:0042982	amyloid precursor protein metabolic process	4/361	75/28814	4.26	0.014670333	0.096715299	0.075219762	Adam10/Dlg1/Rock2/Rtn3	4
BP	GO:1901292	nucleoside phosphate catabolic process	4/361	75/28814	4.26	0.014670333	0.096715299	0.075219762	Ampd3/Nt5m/Nudt3/Nudt4	4
BP	GO:0021700	developmental maturation	10/361	355/28814	2.25	0.014689907	0.096715299	0.075219762	Aldh1a2/Ank3/Cend1/Nfia/Nrxn1/Plcb1/Septin4/Slc17a7/Slc26a6/Tut7	10
BP	GO:0006188	IMP biosynthetic process	2/361	15/28814	10.64	0.014757215	0.096715299	0.075219762	Ampd3/Ppat	2
BP	GO:0010771	negative regulation of cell morphogenesis involved in differentiation	2/361	15/28814	10.64	0.014757215	0.096715299	0.075219762	Coro1c/Ttc3	2
BP	GO:0010960	magnesium ion homeostasis	2/361	15/28814	10.64	0.014757215	0.096715299	0.075219762	Ank3/Kcna1	2
BP	GO:0021819	layer formation in cerebral cortex	2/361	15/28814	10.64	0.014757215	0.096715299	0.075219762	Adgrg1/Mboat7	2
BP	GO:0030320	cellular monovalent inorganic anion homeostasis	2/361	15/28814	10.64	0.014757215	0.096715299	0.075219762	Ckb/Slc12a2	2
BP	GO:0031000	response to caffeine	2/361	15/28814	10.64	0.014757215	0.096715299	0.075219762	Prkaa1/Slc8a1	2
BP	GO:0042574	retinal metabolic process	2/361	15/28814	10.64	0.014757215	0.096715299	0.075219762	Aldh1a2/Dhrs13	2
BP	GO:0043558	regulation of translational initiation in response to stress	2/361	15/28814	10.64	0.014757215	0.096715299	0.075219762	Eif2ak4/Hbb-b1	2
BP	GO:0061577	calcium ion transmembrane transport via high voltage-gated calcium channel	2/361	15/28814	10.64	0.014757215	0.096715299	0.075219762	Cacnb3/Camk2d	2
BP	GO:1901386	negative regulation of voltage-gated calcium channel activity	2/361	15/28814	10.64	0.014757215	0.096715299	0.075219762	Cacnb3/Cbarp	2
BP	GO:1901387	positive regulation of voltage-gated calcium channel activity	2/361	15/28814	10.64	0.014757215	0.096715299	0.075219762	Cacnb3/S100a1	2
BP	GO:1990138	neuron projection extension	7/361	204/28814	2.74	0.014800679	0.096826624	0.075306345	Bdnf/Eif2ak4/L1cam/Rims2/Sema4a/Sema4f/Sh3gl2	7
BP	GO:1902652	secondary alcohol metabolic process	6/361	158/28814	3.03	0.014893841	0.097262102	0.075645035	Abca1/Cat/Dhcr7/Mvd/Mvk/Prkaa1	6
BP	GO:0034764	positive regulation of transmembrane transport	8/361	253/28814	2.52	0.014925361	0.097294201	0.07567	Ank3/Cacnb3/Calm1/Kcna1/Plcb1/S100a1/Slc26a6/Sorbs1	8
BP	GO:0032231	regulation of actin filament bundle assembly	5/361	115/28814	3.47	0.015033436	0.097824333	0.076082307	Cd47/Pxn/Rock2/Sorbs3/Tjp1	5
BP	GO:0031669	cellular response to nutrient levels	7/361	205/28814	2.73	0.015165031	0.098505358	0.076611971	Cadps2/Eif2ak4/Gcn1/Glul/Ndor1/Prkaa1/Xpr1	7
BP	GO:0046847	filopodium assembly	4/361	76/28814	4.2	0.015336448	0.099442184	0.077340581	Mien1/Nrxn1/Ppp1r9b/Rab3ip	4
CC	GO:0098793	presynapse	38/367	493/28739	6.04	8.42655E-19	3.85936E-16	2.43039E-16	Adam10/Adam23/Apbb1/Atp2b1/Bdnf/Cadps2/Calm1/Cck/Cplx1/Cplx3/Dlg1/Glul/Kcna1/Kcna2/Kcnab2/Kcnip3/Kirrel3/L1cam/Nefm/Nrxn1/Pcsk1/Ppp3cc/Rab11b/Rab26/Rab2b/Rims2/Septin4/Sh3gl1/Sh3gl2/Slc17a7/Slc30a3/Slc8a1/Sncb/Stx1a/Stx2/Syap1/Syn1/Tpbg	38
CC	GO:0098978	glutamatergic synapse	30/367	323/28739	7.27	2.8437E-17	6.51207E-15	4.10091E-15	Abhd6/Adam10/Adam23/Apbb1/Arhgap39/Arpc5l/Atp2b1/Atp2b2/Cadps/Cadps2/Cplx1/Dlg1/Gsg1l/Homer2/Lrrc4c/Mdga2/Napa/Nptx1/Nrxn1/Numb/Plcb1/Ppp3ca/Ppp3cc/Ppp3r1/Rims2/Rock2/Sh3gl1/Sh3gl2/Shisa9/Slc30a3	30

BP	GO:0089718	amino acid import across plasma membrane	5/607	49/28814	4.84	0.003624171	0.030533796	0.022109021	Gfap/Grm1/Slc1a3/Slc6a9/Slc7a5	5
BP	GO:0051650	establishment of vesicle localization	10/607	172/28814	2.76	0.003652247	0.030716167	0.022241073	Bloc1s6/Dipk2a/Hap1/Myo1b/Picalm/Rab3a/Sar1b/Smpd3/Stam/Wipi1	10
BP	GO:0006909	phagocytosis	17/607	386/28814	2.09	0.003689335	0.030973557	0.022427445	Abca1/Camk1d/Dnm2/Hmgb1/P2rx7/Plcg2/Rab14/Rab31/Rab34/Rac1/Rac2/Rap1gap/Sirpa/Sod1/Vav1/Xkr5/Xkr9	17
BP	GO:0097529	myeloid leukocyte migration	12/607	230/28814	2.48	0.003706895	0.031018578	0.022460044	Camk1d/Ednrb/Enpp1/Hmgb1/Mapk3/Mcu/Pla2g7/Rac1/Rac2/Sirpa/Thbs4/Vav1	12
BP	GO:0045927	positive regulation of growth	15/607	322/28814	2.21	0.003707987	0.031018578	0.022460044	Bbs2/Dbn1/Dbnl/Dnph1/Map3k13/Mef2c/Naca/Ntrk3/Picalm/Pls1/Rnf157/Sema7a/Slc25a33/Slc6a3/Syt17	15
BP	GO:0032200	telomere organization	9/607	145/28814	2.95	0.003714178	0.031018578	0.022460044	Apex1/Dhx36/Exosc10/Lmna/Mapk3/Pcna/Smg5/Tnks/Yy1	9
BP	GO:0046578	regulation of Ras protein signal transduction	11/607	201/28814	2.6	0.003741818	0.031114245	0.022529314	Abca1/Adgrg1/Arhgef10/Arhgef2/Cdh2/Dab2ip/Dgki/Dnm2/Prag1/Ralgps2/Shoc2	11
BP	GO:0060401	cytosolic calcium ion transport	11/607	201/28814	2.6	0.003741818	0.031114245	0.022529314	Atp2b1/Bax/Drd1/Hap1/Mcu/P2rx7/Plcg2/Ptpn6/Rgs9/Ryr3/Tmem38a	11
BP	GO:0032204	regulation of telomere maintenance	7/607	94/28814	3.53	0.003752921	0.031114245	0.022529314	Dhx36/Exosc10/Lmna/Mapk3/Smg5/Tnks/Yy1	7
BP	GO:0055072	iron ion homeostasis	7/607	94/28814	3.53	0.003752921	0.031114245	0.022529314	Atp6v1g1/B2m/Ftl1/Heph/Picalm/Slc6a9/Sod1	7
BP	GO:0050764	regulation of phagocytosis	8/607	119/28814	3.19	0.003764713	0.031114245	0.022529314	Camk1d/Dnm2/Hmgb1/Plcg2/Rab31/Rap1gap/Sirpa/Sod1	8
BP	GO:0051209	release of sequestered calcium ion into cytosol	8/607	119/28814	3.19	0.003764713	0.031114245	0.022529314	Bax/Drd1/Hap1/P2rx7/Plcg2/Ptpn6/Ryr3/Tmem38a	8
BP	GO:0021885	forebrain cell migration	6/607	71/28814	4.01	0.003835645	0.031536795	0.022835276	Adgrg1/Cdk5r2/Dab2ip/Drd1/Fgf13/Rac1	6
BP	GO:0042273	ribosomal large subunit biogenesis	6/607	71/28814	4.01	0.003835645	0.031536795	0.022835276	Brix1/Eif6/Gtpbp4/Rpl23a/Rpl35a/Rplp0	6
BP	GO:0050432	catecholamine secretion	6/607	71/28814	4.01	0.003835645	0.031536795	0.022835276	Kcnb1/Prkcb/Ptgs1/Rab3a/Sncg/Syt17	6
BP	GO:0001654	eye development	18/607	421/28814	2.03	0.003894615	0.031966629	0.023146512	Ache/Aldh1a1/Bax/Bcar3/Celf4/Clcn2/Hmgb1/Ntrk3/Phactr4/Prom1/Slc6a3/Sox8/Stat3/Th/Tmod1/Tulp3/Vim/Yy1	18
BP	GO:0045762	positive regulation of adenylate cyclase activity	4/607	31/28814	6.13	0.003906424	0.032008556	0.02317687	Adcy3/Drd1/Gnal/Stim1	4
BP	GO:0051283	negative regulation of sequestering of calcium ion	8/607	120/28814	3.16	0.003961138	0.032364383	0.023434519	Bax/Drd1/Hap1/P2rx7/Plcg2/Ptpn6/Ryr3/Tmem38a	8
BP	GO:0043487	regulation of RNA stability	10/607	174/28814	2.73	0.0039634	0.032364383	0.023434519	Apex1/Cnot7/Dcp1a/Dcps/Dhx36/Hnrnpa0/Noct/Paip1/Tut4/Vim	10
BP	GO:0061157	mRNA destabilization	7/607	95/28814	3.5	0.003979606	0.032441261	0.023490185	Cnot7/Dcp1a/Dcps/Dhx36/Noct/Paip1/Tut4	7
BP	GO:0048771	tissue remodeling	11/607	203/28814	2.57	0.004031133	0.032805322	0.023753796	Bax/Enpp1/Flna/Igfbp5/Lipa/Mef2c/P2rx7/Rac1/Rac2/Thbs4/Traf6	11
BP	GO:0001508	action potential	9/607	147/28814	2.91	0.004064583	0.032965221	0.023869576	Ctnna3/Drd1/Fgf13/Flna/Kcnb1/Kcnd2/Ntrk3/P2rx7/Scn1b	9
BP	GO:0046488	phosphatidylinositol metabolic process	9/607	147/28814	2.91	0.004064583	0.032965221	0.023869576	Cds1/Inpp5j/Itpka/Mtmr12/Pigt/Pip4p1/Pip5k1a/Pitpnc1/Plcg2	9
BP	GO:0017038	protein import	12/607	233/28814	2.44	0.004109702	0.033176236	0.024022369	Bag3/Clu/Drd1/Flna/Ipo7/Ipo8/Lmna/Pex1/Ptpn5/Rab8b/Ranbp6/Stat3	12
BP	GO:0044843	cell cycle G1/S phase transition	12/607	233/28814	2.44	0.004109702	0.033176236	0.024022369	Apex1/Atp2b4/Brd4/Camk2b/Crebbp/Gjc2/Larp7/Pdpn/Plcg2/Psme1/Psme2/Ptpn6	12
BP	GO:2000179	positive regulation of neural precursor cell proliferation	6/607	72/28814	3.96	0.004111436	0.033176236	0.024022369	Adgrg1/Flna/Gjc2/Kdm1a/Rsu1/Setd1a	6
BP	GO:0032635	interleukin-6 production	10/607	175/28814	2.71	0.004126642	0.033242784	0.024070555	Arhgef2/Hmgb1/P2rx7/Plcg2/Ptpn6/Sirpa/Stat3/Traf6/Tut4/Zbtb20	10
BP	GO:0150063	visual system development	18/607	424/28814	2.02	0.004189666	0.033522209	0.024272881	Ache/Aldh1a1/Bax/Bcar3/Celf4/Clcn2/Hmgb1/Ntrk3/Phactr4/Prom1/Slc6a3/Sox8/Stat3/Th/Tmod1/Tulp3/Vim/Yy1	18
BP	GO:0007044	cell-substrate junction assembly	7/607	96/28814	3.46	0.004216519	0.033522209	0.024272881	Actn2/Arhgap6/Pip5k1a/Rac1/Rcc2/Sorbs1/Vcl	7
BP	GO:0072655	establishment of protein localization to mitochondrion	7/607	96/28814	3.46	0.004216519	0.033522209	0.024272881	Bag3/Bax/Hk2/Ptpn5/Timm13/Timm8a1/Timm9	7
BP	GO:0002371	dendritic cell cytokine production	3/607	16/28814	8.9	0.004245538	0.033522209	0.024272881	Dhx36/Jak3/Plcg2	3
BP	GO:0008212	mineralocorticoid metabolic process	3/607	16/28814	8.9	0.004245538	0.033522209	0.024272881	Clcn2/Dkk3/Ednrb	3
BP	GO:0032688	negative regulation of interferon-beta production	3/607	16/28814	8.9	0.004245538	0.033522209	0.024272881	Crebbp/Sirpa/Yy1	3
BP	GO:0042416	dopamine biosynthetic process	3/607	16/28814	8.9	0.004245538	0.033522209	0.024272881	Ddc/Slc6a3/Th	3
BP	GO:0043951	negative regulation of cAMP-mediated signaling	3/607	16/28814	8.9	0.004245538	0.033522209	0.024272881	Nucb2/Pde10a/Rnf157	3
BP	GO:0051151	negative regulation of smooth muscle cell differentiation	3/607	16/28814	8.9	0.004245538	0.033522209	0.024272881	Dnmt1/Pdcd4/Rcan1	3
BP	GO:0070885	negative regulation of calcineurin-NFAT signaling cascade	3/607	16/28814	8.9	0.004245538	0.033522209	0.024272881	Atp2b4/Homer2/Rcan1	3
BP	GO:0106057	negative regulation of calcineurin-mediated signaling	3/607	16/28814	8.9	0.004245538	0.033522209	0.024272881	Atp2b4/Homer2/Rcan1	3
BP	GO:2000479	regulation of cAMP-dependent protein kinase activity	3/607	16/28814	8.9	0.004245538	0.033522209	0.024272881	Atp2b4/Prkar1b/Prkar2b	3
BP	GO:1901214	regulation of neuron death	17/607	392/28814	2.06	0.004300148	0.033783205	0.024461865	Akt1s1/Bax/Clu/Eno2/Gabrb2/Grik5/Kcnb1/Meak7/Mef2c/Msh2/Picalm/Ptpn5/Rras2/Sigmar1/Sncg/Sod1/Stat3	17
BP	GO:0042088	T-helper 1 type immune response	5/607	51/28814	4.65	0.004313953	0.033783205	0.024461865	Hmgb1/Jak3/Nras/Pla2g4a/Traf6	5
BP	GO:0043268	positive regulation of potassium ion transport	5/607	51/28814	4.65	0.004313953	0.033783205	0.024461865	Actn2/Dnm2/Flna/Kcnc1/Kcnip2	5
BP	GO:0045332	phospholipid translocation	5/607	51/28814	4.65	0.004313953	0.033783205	0.024461865	Abca1/Ano3/P2rx7/Xkr5/Xkr9	5

CC	GO:0097060	synaptic membrane	28/367	390/28739	5.62	2.21862E-13	3.38709E-11	2.13299E-11	Abhd6/Adam10/Adam23/Ank3/Apbb1/Atp2b1/Atp2b2/Cadps2/Cnih2/Cplx3/Dlg1/Gabra3/Gabrb1/Gsg1l/Iqsec3/Kcna1/L1cam/Lrrc4c/Nrxn1/Ppp1r9b/Prr7/Rims2/Sema4f/Shisa9/Slc8a1/Stx1a/Stx2/Syap1	28
CC	GO:1990351	transporter complex	24/367	393/28739	4.78	3.37207E-10	3.86102E-08	2.43144E-08	Abhd6/Atp1a1/Atp1a4/Atp2a2/Atp8a2/Cacnb3/Calm1/Clcn2/Cnih2/Gabra3/Gabrb1/Kcna1/Kcna2/Kcnab2/Kcnip3/Ndufa12/Ndufa9/Pex13/Scn4b/Scn8a/Shisa9/Slc26a6/Timm8a1/Unc80	24
CC	GO:0043194	axon initial segment	8/367	27/28739	23.2	1.17737E-09	9.26771E-08	5.83625E-08	Ank3/Camk2d/Cck/Kcna1/Kcna2/Kcnab2/Map1a/Scn8a	8
CC	GO:0099572	postsynaptic specialization	22/367	352/28739	4.89	1.21411E-09	9.26771E-08	5.83625E-08	Adam10/Add2/Atp1a1/Atp2b2/Camk2a/Cnih2/Dlg1/Gabra3/Gsg1l/Homer2/Iqsec3/Kcnab2/Lrrc4c/Map1a/Nefm/Ppp1r9b/Prr7/Rpl18a/Shisa9/Slc8a1/Stx1a/Svn1	22
CC	GO:0098984	neuron to neuron synapse	22/367	361/28739	4.77	1.9391E-09	1.20817E-07	7.6083E-08	Adam10/Add2/Atp1a1/Atp2b2/Camk2a/Cnih2/Dlg1/Gsg1l/Homer2/Iqsec3/Kcnab2/Lrrc4c/Map1a/Nefm/Ppp1r9b/Prr7/Rpl18a/Shisa9/Slc30a3/Slc8a1/Stx1a/Svn1	22
CC	GO:0014069	postsynaptic density	21/367	330/28739	4.98	2.11033E-09	1.20817E-07	7.6083E-08	Adam10/Add2/Atp1a1/Atp2b2/Camk2a/Cnih2/Dlg1/Gsg1l/Homer2/Iqsec3/Kcnab2/Lrrc4c/Map1a/Nefm/Ppp1r9b/Prr7/Rpl18a/Shisa9/Slc8a1/Stx1a/Svn1	21
CC	GO:0032279	asymmetric synapse	21/367	339/28739	4.85	3.40506E-09	1.7328E-07	1.09121E-07	Adam10/Add2/Atp1a1/Atp2b2/Camk2a/Cnih2/Dlg1/Gsg1l/Homer2/Iqsec3/Kcnab2/Lrrc4c/Map1a/Nefm/Ppp1r9b/Prr7/Rpl18a/Shisa9/Slc8a1/Stx1a/Svn1	21
CC	GO:0042734	presynaptic membrane	13/367	132/28739	7.71	1.59463E-08	6.17643E-07	3.88955E-07	Adam23/Apbb1/Atp2b1/Cadps2/Cplx3/Dlg1/Kcna1/L1cam/Nrxn1/Rims2/Stx1a/Stx2/Syap1	13
CC	GO:0044306	neuron projection terminus	16/367	212/28739	5.91	1.66716E-08	6.17643E-07	3.88955E-07	Atcay/Bdnf/Cck/Cplx1/Cplx3/Glul/Kcna1/Kcna2/Kcnab2/Kcnip3/L1cam/Pcsk1/Septin4/Slc8a1/Sncb/Tpbg	16
CC	GO:1902495	transmembrane transporter complex	21/367	372/28739	4.42	1.73038E-08	6.17643E-07	3.88955E-07	Abhd6/Atp1a1/Atp1a4/Atp2a2/Cacnb3/Calm1/Clcn2/Cnih2/Gabra3/Gabrb1/Kcna1/Kcna2/Kcnab2/Kcnip3/Ndufa12/Ndufa9/Scn4b/Scn8a/Shisa9/Slc26a6/Unc80	21
CC	GO:0045211	postsynaptic membrane	18/367	273/28739	5.16	1.75314E-08	6.17643E-07	3.88955E-07	Abhd6/Adam10/Ank3/Apbb1/Atp2b2/Cadps2/Cnih2/Dlg1/Gabra3/Gabrb1/Gsg1l/Iqsec3/Lrrc4c/Prr7/Sema4f/Shisa9/Slc8a1/Syap1	18
CC	GO:0150034	distal axon	21/367	375/28739	4.39	1.98766E-08	6.50247E-07	4.09487E-07	Apbb1/Bdnf/Calm1/Cbarp/Cck/Cplx1/Cplx3/Glul/Kcna1/Kcna2/Kcnab2/Kcnip3/L1cam/Nrxn1/Pcsk1/Ppp1r9b/Septin4/Slc8a1/Sncb/Syap1/Tpbg	21
CC	GO:0043679	axon terminus	15/367	190/28739	6.18	2.57099E-08	7.8501E-07	4.94352E-07	Bdnf/Cck/Cplx1/Cplx3/Glul/Kcna1/Kcna2/Kcnab2/Kcnip3/L1cam/Pcsk1/Septin4/Slc8a1/Sncb/Tpbg	15
CC	GO:0030133	transport vesicle	19/367	316/28739	4.71	3.09171E-08	8.85002E-07	5.57322E-07	Adam10/Atp2b1/Bdnf/C1qtnf5/Calm1/Cplx3/Dlg1/Kirrel3/Rab11b/Rab26/Rab3ip/Sec13/Septin4/Slc17a7/Slc30a3/Spg21/Stx1a/Stx2/Svn1	19
CC	GO:0070382	exocytic vesicle	16/367	228/28739	5.5	4.62923E-08	1.24717E-06	7.85392E-07	Adam10/Atp2b1/Bdnf/Calm1/Cplx3/Dlg1/Kirrel3/Rab11b/Rab26/Rab3ip/Septin4/Slc17a7/Slc30a3/Stx1a/Stx2/Svn1	16
CC	GO:0008021	synaptic vesicle	15/367	205/28739	5.73	7.05609E-08	1.79538E-06	1.13063E-06	Adam10/Atp2b1/Bdnf/Calm1/Cplx3/Dlg1/Kirrel3/Rab11b/Rab26/Septin4/Slc17a7/Slc30a3/Stx1a/Stx2/Svn1	15
CC	GO:0098685	Schaffer collateral - CA1 synapse	10/367	83/28739	9.43	1.08548E-07	2.61659E-06	1.64777E-06	Apbb1/Camk2a/Cplx1/Gsg1l/Lrrc4c/Nrxn1/Ppp3ca/Ppp3r1/Rock2/Syn1	10
CC	GO:0044304	main axon	10/367	85/28739	9.21	1.36525E-07	3.12641E-06	1.96883E-06	Ank3/Apbb1/Camk2d/Cck/Dlg1/Kcna1/Kcna2/Kcnab2/Map1a/Scn8a	10
CC	GO:0030016	myofibril	15/367	224/28739	5.24	2.24269E-07	4.8912E-06	3.08019E-06	Ank3/Calm1/Csrp1/Dnajb6/Jph1/Myl1/Myl12b/Nexn/Ppp3ca/Rpl15/S100a1/Scn8a/Slc4a1/Slc8a1/Tmod1	15
CC	GO:0098982	GABA-ergic synapse	9/367	69/28739	10.21	2.36378E-07	4.92096E-06	3.09893E-06	Abhd6/Atp2b1/Atp2b2/Gabra3/Iqsec3/Mdga2/Nrxn1/Plcb1/Rims2	9
CC	GO:0030017	sarcomere	14/367	199/28739	5.51	3.12294E-07	6.21873E-06	3.91619E-06	Ank3/Calm1/Csrp1/Dnajb6/Jph1/Myl12b/Nexn/Ppp3ca/Rpl15/S100a1/Scn8a/Slc4a1/Slc8a1/Tmod1	14
CC	GO:0043292	contractile fiber	15/367	237/28739	4.96	4.6236E-07	8.82338E-06	5.55644E-06	Ank3/Calm1/Csrp1/Dnajb6/Jph1/Myl1/Myl12b/Nexn/Ppp3ca/Rpl15/S100a1/Scn8a/Slc4a1/Slc8a1/Tmod1	15
CC	GO:0098563	intrinsic component of synaptic vesicle membrane	6/367	25/28739	18.79	6.00605E-07	1.10031E-05	6.92908E-06	Atp2b1/Cplx3/Rab26/Slc17a7/Slc30a3/Syn1	6
CC	GO:0043209	myelin sheath	14/367	215/28739	5.1	7.95199E-07	1.40077E-05	8.82123E-06	Atp1a1/Calm1/Ckb/Dlg1/Glul/Hbb-b1/Napa/Ndrg1/Nefm/Phgdh/Plcb1/Septin4/Syn1/Tppp	14
CC	GO:0030672	synaptic vesicle membrane	9/367	81/28739	8.7	9.51184E-07	1.55587E-05	9.79791E-06	Atp2b1/Calm1/Cplx3/Dlg1/Rab26/Slc17a7/Slc30a3/Stx1a/Syn1	9
CC	GO:0099501	exocytic vesicle membrane	9/367	81/28739	8.7	9.51184E-07	1.55587E-05	9.79791E-06	Atp2b1/Calm1/Cplx3/Dlg1/Rab26/Slc17a7/Slc30a3/Stx1a/Syn1	9
CC	GO:0030018	Z disc	11/367	132/28739	6.53	1.10677E-06	1.74794E-05	1.10075E-05	Ank3/Csrp1/Dnajb6/Jph1/Myl12b/Nexn/Ppp3ca/S100a1/Scn8a/Slc4a1/Slc8a1	11
CC	GO:0034702	ion channel complex	16/367	294/28739	4.26	1.42962E-06	2.18255E-05	1.37444E-05	Abhd6/Cacnb3/Calm1/Clcn2/Cnih2/Gabra3/Gabrb1/Kcna1/Kcna2/Kcnab2/Kcnip3/Scn4b/Scn8a/Shisa9/Slc26a6/Unc80	16
CC	GO:0098562	cytoplasmic side of membrane	13/367	195/28739	5.22	1.51343E-06	2.23597E-05	1.40808E-05	Atp2a2/Atp2b1/Cdip1/Cyth3/Dlg1/Kcnab2/Mien1/Ppp1r9b/Ppp3ca/Slc4a1/Sppl2b/Syap1/Yes1	13
CC	GO:0043197	dendritic spine	13/367	198/28739	5.14	1.79479E-06	2.56879E-05	1.61767E-05	Adam10/Apbb1/Atp2b1/Atp2b2/Camk2a/Cnih2/Fbxo2/Itpka/Numb/Ppp1r9b/Ppp3ca/Shisa9/Slc8a1	13
CC	GO:0014704	intercalated disc	8/367	67/28739	9.35	2.21712E-06	3.07709E-05	1.93777E-05	Ank3/Atp1a1/Atp2a2/Camk2d/Scn4b/Slc4a1/Slc8a1/Tjp1	8
CC	GO:0044309	neuron spine	13/367	203/28739	5.01	2.36805E-06	3.1899E-05	2.00881E-05	Adam10/Apbb1/Atp2b1/Atp2b2/Camk2a/Cnih2/Fbxo2/Itpka/Numb/Ppp1r9b/Ppp3ca/Shisa9/Slc8a1	13
CC	GO:0031674	I band	11/367	145/28739	5.94	2.79165E-06	3.65307E-05	2.30048E-05	Ank3/Csrp1/Dnajb6/Jph1/Myl12b/Nexn/Ppp3ca/S100a1/Scn8a/Slc4a1/Slc8a1	11
CC	GO:0030658	transport vesicle membrane	10/367	119/28739	6.58	3.16266E-06	4.0236E-05	2.53382E-05	Atp2b1/Calm1/Cplx3/Dlg1/Rab26/Sec13/Slc17a7/Slc30a3/Stx1a/Syn1	10
CC	GO:0032591	dendritic spine membrane	4/367	13/28739	24.09	1.7077E-05	0.000211386	0.000133118	Atp2b1/Atp2b2/Ppp1r9b/Shisa9	4
CC	GO:0009898	cytoplasmic side of plasma membrane	11/367	177/28739	4.87	1.87205E-05	0.000225632	0.000142089	Atp2a2/Atp2b1/Cyth3/Dlg1/Kcnab2/Mien1/Ppp1r9b/Ppp3ca/Slc4a1/Syap1/Yes1	11
CC	GO:0044291	cell-cell contact zone	8/367	90/28739	6.96	2.04241E-05	0.000239852	0.000151045	Ank3/Atp1a1/Atp2a2/Camk2d/Scn4b/Slc4a1/Slc8a1/Tjp1	8
CC	GO:0031300	intrinsic component of organelle membrane	16/367	370/28739	3.39	2.57505E-05	0.000294843	0.000185675	Atp2b1/B4galnt1/Cplx3/Dhcr7/Emc7/Oxa1l/Pex13/Preb/Rab26/Sec61a1/Slc17a7/Slc30a3/Sppl2b/Syn1/Timm23/Zmpste24	16
CC	GO:0099240	intrinsic component of synaptic membrane	9/367	123/28739	5.73	3E-05	0.000335121	0.000211039	Abhd6/Adam23/Atp2b1/Cplx3/Gabra3/Gsg1l/Lrrc4c/Nrxn1/Shisa9	9
CC	GO:0034703	cation channel complex	12/367	224/28739	4.2	3.44783E-05	0.000375978	0.000236768	Abhd6/Cacnb3/Calm1/Cnih2/Kcna1/Kcna2/Kcnab2/Kcnip3/Scn4b/Scn8a/Shisa9/Unc80	12
CC	GO:0048786	presynaptic active zone	7/367	74/28739	7.41	4.51554E-05	0.000480957	0.000302878	Cplx3/Nrxn1/Rims2/Slc17a7/Stx1a/Stx2/Syn1	7
CC	GO:0045121	membrane raft	16/367	393/28739	3.19	5.29152E-05	0.0005508	0.000346861	Abca1/Add2/Atp1a1/Atp2b1/Atp2b2/Cd55b/Coro1c/Dlg1/Ehd2/Gprc5b/L1cam/Lamtor1/Pag1/Rtn4rl1/Sorbs1/Stx2	16
CC	GO:0098857	membrane microdomain	16/367	394/28739	3.18	5.45254E-05	0.000554948	0.000349473	Abca1/Add2/Atp1a1/Atp2b1/Atp2b2/Cd55b/Coro1c/Dlg1/Ehd2/Gprc5b/L1cam/Lamtor1/Pag1/Rtn4rl1/Sorbs1/Stx2	16

BP	GO:0045740	positive regulation of DNA replication	5/607	51/28814	4.65	0.004313953	0.033783205	0.024461865	Bcar3/Cst3/Endog/Pcna/Rac1	5
BP	GO:0001776	leukocyte homeostasis	8/607	122/28814	3.11	0.00437763	0.034163922	0.024737536	Bap1/Bax/Dnaja3/Enpp1/Hmgb1/Jak3/Mef2c/P2rx7	8
BP	GO:0051282	regulation of sequestering of calcium ion	8/607	122/28814	3.11	0.00437763	0.034163922	0.024737536	Bax/Drd1/Hap1/P2rx7/Plcg2/Ptpn6/Ryr3/Tmem38a	8
BP	GO:0007007	inner mitochondrial membrane organization	4/607	32/28814	5.93	0.004391176	0.034163922	0.024737536	Bax/Micos13/Timm13/Timm9	4
BP	GO:0051150	regulation of smooth muscle cell differentiation	4/607	32/28814	5.93	0.004391176	0.034163922	0.024737536	Dnmt1/Olfm2/Pdcd4/Rcan1	4
BP	GO:0009411	response to UV	9/607	149/28814	2.87	0.004440303	0.034489964	0.024973618	Bax/Brsk1/Crebbp/Dhx36/Kdm1a/Mme/Msh2/Pcna/Y…	9
BP	GO:0010721	negative regulation of cell development	11/607	206/28814	2.53	0.004498262	0.034883439	0.025258527	Arhgef2/B2m/Dip2b/Ednrb/Fgf13/Kdm1a/Lingo1/Ntrk3/…	11
BP	GO:0042542	response to hydrogen peroxide	8/607	123/28814	3.09	0.004598102	0.035588436	0.025769003	Apex1/Endog/Glrx2/Rhob/Sigmar1/Sirpa/Smpd3/Sod1	8
BP	GO:0002790	peptide secretion	14/607	298/28814	2.23	0.00460676	0.035588436	0.025769003	Abca1/Adcy8/Cplx1/Dynll1/Ensa/Ildr2/Kcnb1/Mcu/Nucb2/Rab8b/Rac1/Slc18a2/Sm…	14
BP	GO:0048880	sensory system development	18/607	428/28814	2	0.004611522	0.035588436	0.025769003	Ache/Aldh1a1/Bax/Bcar3/Celf4/Clcn2/Hmgb1/Ntrk3/Phactr4/Prom1/Slc6a3/Sox8/Stat3/Th/Tmod1/Tulp3/Vim/Y…	18
BP	GO:0035725	sodium ion transmembrane transport	9/607	150/28814	2.85	0.004638032	0.035700102	0.025849858	Asic1/Atp2b4/Dnm2/Fgf13/Hcn3/Scn1b/Slc6a3/Slc6a9/…	9
BP	GO:0061013	regulation of mRNA catabolic process	10/607	178/28814	2.67	0.004648412	0.035700102	0.025849858	Apex1/Cnot7/Dcp1a/Dcps/Dhx36/Hnrnpa0/Noct/Paip1/…	10
BP	GO:1902904	negative regulation of supramolecular fiber organization	10/607	178/28814	2.67	0.004648412	0.035700102	0.025849858	Arhgap6/Arhgef2/Clu/Cracd/Dbn1/Fgf13/Inpp5j/Kank4/Ssh3/Tmod1	10
BP	GO:0014888	striated muscle adaptation	5/607	52/28814	4.56	0.004691762	0.035857941	0.025964147	Atp2b4/Foxo1/Igfbp5/Lmna/Mef2c	5
BP	GO:0048066	developmental pigmentation	5/607	52/28814	4.56	0.004691762	0.035857941	0.025964147	Bax/Bloc1s6/Ednrb/Enpp1/Mef2c	5
BP	GO:1902622	regulation of neutrophil migration	5/607	52/28814	4.56	0.004691762	0.035857941	0.025964147	Camk1d/Mcu/Rac1/Rac2/Thbs4	5
BP	GO:0034394	protein localization to cell surface	6/607	74/28814	3.85	0.00470661	0.035857941	0.025964147	Actn2/Flna/Picalm/Prrt1/Stx3/Vcl	6
BP	GO:0030148	sphingolipid biosynthetic process	7/607	98/28814	3.39	0.004722176	0.035857941	0.025964147	Abca8b/B4galt6/Hacd3/Kdsr/P2rx7/Smpd3/Smpd4	7
BP	GO:0032091	negative regulation of protein binding	7/607	98/28814	3.39	0.004722176	0.035857941	0.025964147	Actb/B2m/Bax/Camk1/Ckmt1/Kdm1a/Mapk3	7
BP	GO:0044264	cellular polysaccharide metabolic process	7/607	98/28814	3.39	0.004722176	0.035857941	0.025964147	Enpp1/Grb10/Hmgb1/Phka1/Pkh/Ppp1cc/Sorbs1	7
BP	GO:1903532	positive regulation of secretion by cell	16/607	363/28814	2.09	0.004729015	0.035857941	0.025964147	Adcy8/Cdk5r2/Dynll1/Frmd4a/Kcnb1/Mcu/Nucb2/P2rx7/Pla2g4a/Rab34/Rab3a/Rab8b/Rac1/Smpd3/Snap23/St…	16
BP	GO:0051208	sequestering of calcium ion	8/607	124/28814	3.06	0.004826999	0.036542903	0.026460117	Bax/Drd1/Hap1/P2rx7/Plcg2/Ptpn6/Ryr3/Tmem38a	8
BP	GO:0010508	positive regulation of autophagy	9/607	151/28814	2.83	0.004842543	0.036544749	0.026461453	Bag3/Elapor1/Endog/Foxo1/Hmgb1/Irgm1/Mapk3/Slc25a5/Wipi1	9
BP	GO:0051147	regulation of muscle cell differentiation	9/607	151/28814	2.83	0.004842543	0.036544749	0.026461453	Camk1/Dnmt1/Mef2c/Naca/Olfm2/Pdcd4/Rcan1/Setd3/Yy1	9
BP	GO:2000108	positive regulation of leukocyte apoptotic process	4/607	33/28814	5.75	0.004914868	0.03703206	0.026814308	Bax/Jak3/Mef2c/P2rx7	4
BP	GO:0019935	cyclic-nucleotide-mediated signaling	7/607	99/28814	3.36	0.0049915	0.037491185	0.027146752	Adcy8/Ednrb/Gucy1b1/Nucb2/Pde10a/Rasd2/Rxfp3	7
BP	GO:0050779	RNA destabilization	7/607	99/28814	3.36	0.0049915	0.037491185	0.027146752	Cnot7/Dcp1a/Dcps/Dhx36/Noct/Paip1/Tut4	7
BP	GO:0071241	cellular response to inorganic substance	12/607	239/28814	2.38	0.005020574	0.037589114	0.027217661	Adcy8/Cbr2/Cpne3/Foxo1/Kcnb1/Mapk3/Mef2c/Nptx1/Plcg2/Rasgrp2/Smpd3/Syt…	12
BP	GO:0031333	negative regulation of protein-containing complex assembly	9/607	152/28814	2.81	0.005053989	0.037589114	0.027217661	Arhgef2/Clu/Cracd/Hmgb1/Inpp5j/Kank4/Lamp2/Ssh3/Tmod1	9
BP	GO:0001845	phagolysosome assembly	3/607	17/28814	8.38	0.005075357	0.037589114	0.027217661	P2rx7/Rab14/Rab34	3
BP	GO:0010566	regulation of ketone biosynthetic process	3/607	17/28814	8.38	0.005075357	0.037589114	0.027217661	Clcn2/Coq3/Dkk3	3
BP	GO:0051589	negative regulation of neurotransmitter transport	3/607	17/28814	8.38	0.005075357	0.037589114	0.027217661	Asic1/Pnkd/Slc18a2	3
BP	GO:0071801	regulation of podosome assembly	3/607	17/28814	8.38	0.005075357	0.037589114	0.027217661	Arhgef2/Lcp1/Msn	3
BP	GO:0090493	catecholamine uptake	3/607	17/28814	8.38	0.005075357	0.037589114	0.027217661	Actb/Slc6a3/Smpd3	3
BP	GO:1902285	semaphorin-plexin signaling pathway involved in neuron projection guidance	3/607	17/28814	8.38	0.005075357	0.037589114	0.027217661	Mef2c/Nrp2/Plxnc1	3
BP	GO:1903358	regulation of Golgi organization	3/607	17/28814	8.38	0.005075357	0.037589114	0.027217661	Dnm2/Map2k2/Mapk3	3
BP	GO:0021795	cerebral cortex cell migration	5/607	53/28814	4.48	0.005092488	0.037599402	0.02722511	Adgrg1/Cdk5r2/Dab2ip/Fgf13/Rac1	5
BP	GO:0034204	lipid translocation	5/607	53/28814	4.48	0.005092488	0.037599402	0.02722511	Abca1/Ano3/P2rx7/Xkr5/Xkr9	5
BP	GO:0006913	nucleocytoplasmic transport	15/607	334/28814	2.13	0.005174935	0.038090392	0.027580628	Bag3/Camk1/Drd1/Eif6/Flna/Ipo7/Ipo8/Lmna/Mcm3ap/Ncbp2/Ppp1cc/Prkag1/Ptpn…	15
BP	GO:0051169	nuclear transport	15/607	334/28814	2.13	0.005174935	0.038090392	0.027580628	Bag3/Camk1/Drd1/Eif6/Flna/Ipo7/Ipo8/Lmna/Mcm3ap/Ncbp2/Ppp1cc/Prkag1/Ptpn…	15
BP	GO:0008406	gonad development	12/607	240/28814	2.37	0.00518692	0.038119872	0.027601974	Adgrg1/Bax/Csmd1/Flna/Hmgb2/Msh2/Pcyt1b/Pla2g4…	12
BP	GO:0061014	positive regulation of mRNA catabolic process	7/607	100/28814	3.32	0.005272209	0.038630148	0.027971457	Cnot7/Dcp1a/Dcps/Dhx36/Noct/Paip1/Tut4	7
BP	GO:0006633	fatty acid biosynthetic process	9/607	153/28814	2.79	0.005272526	0.038630148	0.027971457	Abcd3/Acsbg1/Eif6/Hacd3/Mcat/Ndufab1/Pla2g4a/Prk…	9
BP	GO:0002688	regulation of leukocyte chemotaxis	8/607	126/28814	3.01	0.005310888	0.038851623	0.028131823	Camk1d/Hmgb1/Mapk3/Mcu/Pla2g7/Rac1/Rac2/Thbs4	8
BP	GO:0016239	positive regulation of macroautophagy	6/607	76/28814	3.75	0.005362827	0.039111795	0.028320209	Bag3/Elapor1/Irgm1/Mapk3/Slc25a5/Wipi1	6
BP	GO:1905515	non-motile cilium assembly	6/607	76/28814	3.75	0.005362827	0.039111795	0.028320209	Bbs2/Dnm2/Dynll1/Hap1/Pcm1/Vangl2	6
BP	GO:0051170	import into nucleus	10/607	182/28814	2.61	0.005423264	0.039432168	0.028552186	Bag3/Drd1/Flna/Ipo7/Ipo8/Lmna/Prkag1/Ptpn5/Ranbp…	10
BP	GO:0051260	protein homooligomerization	10/607	182/28814	2.61	0.005423264	0.039432168	0.028552186	Adcy8/B2m/Kcna3/Kcnb1/Kcnc1/Kcnd2/Kctd4/Prmt8/Sigmar1/Vasp	10
BP	GO:0010614	negative regulation of cardiac muscle hypertrophy	4/607	34/28814	5.58	0.005478861	0.039655329	0.028713774	Atp2b4/Foxo1/Lmna/Yy1	4
BP	GO:0046466	membrane lipid catabolic process	4/607	34/28814	5.58	0.005478861	0.039655329	0.028713774	Hexb/Sgpl1/Smpd3/Smpd4	4
BP	GO:0046475	glycerophospholipid catabolic process	4/607	34/28814	5.58	0.005478861	0.039655329	0.028713774	Gpcpd1/Pla2g4a/Pla2g7/Smpd4	4
BP	GO:0007617	mating behavior	5/607	54/28814	4.4	0.005516853	0.039749635	0.028782059	Aplp2/Drd1/Ednrb/Hexb/Th	5
BP	GO:0045773	positive regulation of axon extension	5/607	54/28814	4.4	0.005516853	0.039749635	0.028782059	Dbn1/Dbnl/Map3k13/Ntrk3/Sema7a	5
BP	GO:0045912	negative regulation of carbohydrate metabolic process	5/607	54/28814	4.4	0.005516853	0.039749635	0.028782059	Enpp1/Grb10/Smpd3/Stat3/Ugt1a1	5
BP	GO:0045766	positive regulation of angiogenesis	10/607	183/28814	2.59	0.005631911	0.0404566	0.02929396	Gab1/Hk2/Hmgb1/Jcad/Mydgf/Nras/Prkcb/Rhob/Stat3/…	10
BP	GO:1904018	positive regulation of vasculature development	10/607	183/28814	2.59	0.005631911	0.0404566	0.02929396	Gab1/Hk2/Hmgb1/Jcad/Mydgf/Nras/Prkcb/Rhob/Stat3/…	10
BP	GO:0043113	receptor clustering	6/607	77/28814	3.7	0.005714924	0.040929825	0.029636615	Cdh2/Dnaja3/Flna/Grik5/Shisa7/Sorbs1	6
BP	GO:0098661	inorganic anion transmembrane transport	6/607	77/28814	3.7	0.005714924	0.040929825	0.029636615	Ano3/Gabra3/Gabra4/Gabrb2/Gabrd/Slc1a3	6
BP	GO:0045017	glycerolipid biosynthetic process	11/607	213/28814	2.45	0.005756029	0.041109467	0.029766691	Abhd4/Cds1/Dgki/Hdhd5/Itpka/Pcyt1b/Pigt/Pip5k1a/Pla2g4a/Plcg2/Pld1	11
BP	GO:0008202	steroid metabolic process	15/607	338/28814	2.11	0.005757219	0.041109467	0.029766691	Abca1/Abcd3/Aplp2/Clcn2/Comt/Dkk3/Ednrb/Enpp1/Hmgcs1/Lamtor1/Lipa/Pip4p…	15
BP	GO:0019882	antigen processing and presentation	8/607	128/28814	2.97	0.005830959	0.041511908	0.030058092	B2m/H2-K1/Psme1/Psme2/Rab34/Rab3c/Rab8b/Traf6	8
BP	GO:0061387	regulation of extent of cell growth	8/607	128/28814	2.97	0.005830959	0.041511908	0.030058092	Dbn1/Dbnl/Dip2b/Dnm2/Fgf13/Map3k13/Ntrk3/Sema7…	8
BP	GO:0070585	protein localization to mitochondrion	7/607	102/28814	3.26	0.005868953	0.04172022	0.030208927	Bag3/Bax/Hk2/Ptpn5/Timm13/Timm8a1/Timm9	7
BP	GO:0045137	development of primary sexual characteristics	12/607	244/28814	2.33	0.005896699	0.041855171	0.030306643	Adgrg1/Bax/Csmd1/Flna/Hmgb2/Msh2/Pcyt1b/Pla2g4…	12
BP	GO:0050678	regulation of epithelial cell proliferation	17/607	405/28814	1.99	0.005910767	0.041876214	0.03032188	B2m/Bax/Dab2ip/Ednrb/Hmgb1/Hmgb2/Jcad/Mef2c/Mydgf/Nras/Ppp1r16b/Rap1gap/Sparc/Stat1/Stat3/Tgfa…	17
BP	GO:0014041	regulation of neuron maturation	3/607	18/28814	7.91	0.005996092	0.041876214	0.03032188	Ednrb/Map3k13/Rac1	3
BP	GO:0032793	positive regulation of CREB transcription factor activity	3/607	18/28814	7.91	0.005996092	0.041876214	0.03032188	Adcy8/Camk1d/Crebbp	3

CC	GO:0016323	basolateral plasma membrane	12/367	246/28739	3.82	8.49646E-05	0.000845952	0.00053273	Abca1/Ank3/Atp1a1/Atp2b1/Dlg1/Numb/Slc12a2/Slc26a6/Slc4a1/Slc8a1/Stx2/Tjp1	12
CC	GO:0043204	perikaryon	9/367	141/28739	5	8.72564E-05	0.000850285	0.000535459	Bdnf/Cck/Clcn2/Glul/Kcna2/Nefm/Pcsk1/Septin4/Syap1	9
CC	GO:0005743	mitochondrial inner membrane	17/367	460/28739	2.89	0.000101903	0.000972326	0.000612313	Abcb7/Bdnf/Coq6/Coq7/Cox7a1/Cpox/Dhrs13/Gcdh/Ndufa12/Ndufa9/Ndufaf3/Oxa1l/Slc25a10/Slc25a14/Stat3/Timm23/Timm8a1	17
CC	GO:0099699	integral component of synaptic membrane	8/367	113/28739	5.54	0.000104592	0.000977619	0.000615646	Abhd6/Adam23/Atp2b1/Gabra3/Gsg1l/Lrrc4c/Nrxn1/Shisa9	8
CC	GO:0005758	mitochondrial intermembrane space	7/367	91/28739	6.02	0.000168767	0.00154591	0.000973522	Arl2bp/Cat/Ccs/Chchd2/Cpox/Timm23/Timm8a1	7
CC	GO:0016328	lateral plasma membrane	6/367	67/28739	7.01	0.00021558	0.001935994	0.001219173	Ank3/Atp1a1/C1qtnf5/Coro1c/Dlg1/Slc12a2	6
CC	GO:0044224	juxtaparanode region of axon	3/367	10/28739	23.49	0.000231892	0.002042434	0.001286203	Kcna1/Kcna2/Kcnab2	3
CC	GO:0009925	basal plasma membrane	12/367	276/28739	3.4	0.000247996	0.002143064	0.001349574	Abca1/Ank3/Atp1a1/Atp2b1/Dlg1/Numb/Slc12a2/Slc26a6/Slc4a1/Slc8a1/Stx2/Tjp1	12
CC	GO:0030315	T-tubule	6/367	69/28739	6.81	0.000253485	0.002149932	0.001353899	Ank3/Atp1a1/Cacnb3/Camk2d/Dlg1/Slc8a1	6
CC	GO:0005778	peroxisomal membrane	5/367	46/28739	8.51	0.000294673	0.002410006	0.001517678	Cat/Pecr/Pex1/Pex13/Pex19	5
CC	GO:0031903	microbody membrane	5/367	46/28739	8.51	0.000294673	0.002410006	0.001517678	Cat/Pecr/Pex1/Pex13/Pex19	5
CC	GO:0031201	SNARE complex	5/367	48/28739	8.16	0.0003605	0.002896646	0.001824135	Cplx1/Cplx3/Napa/Stx1a/Stx2	5
CC	GO:0031970	organelle envelope lumen	7/367	104/28739	5.27	0.000384612	0.003037112	0.001912592	Arl2bp/Cat/Ccs/Chchd2/Cpox/Timm23/Timm8a1	7
CC	GO:0048787	presynaptic active zone membrane	4/367	28/28739	11.19	0.00042033	0.003262902	0.002054781	Cplx3/Nrxn1/Stx1a/Stx2	4
CC	GO:0032590	dendrite membrane	5/367	50/28739	7.83	0.000436872	0.003334789	0.002100051	Atp2b1/Atp2b2/Gabra3/Ppp1r9b/Shisa9	5
CC	GO:0045178	basal part of cell	12/367	301/28739	3.12	0.000540138	0.004055463	0.002553889	Abca1/Ank3/Atp1a1/Atp2b1/Dlg1/Numb/Slc12a2/Slc26a6/Slc4a1/Slc8a1/Stx2/Tjp1	12
CC	GO:0031301	integral component of organelle membrane	13/367	350/28739	2.91	0.000624451	0.004556392	0.002869343	Atp2b1/B4galnt1/Dhcr7/Emc7/Oxa1l/Pex13/Preb/Sec61a1/Slc17a7/Slc30a3/Sppl2b/Timm23/Zmpste24	13
CC	GO:0005963	magnesium-dependent protein serine/threonine phosphatase complex	4/367	31/28739	10.1	0.000626753	0.004556392	0.002869343	Ppp3ca/Ppp3cc/Ppp3r1/Ppp4c	4
CC	GO:0090533	cation-transporting ATPase complex	3/367	14/28739	16.78	0.00067719	0.004751806	0.002992404	Atp1a1/Atp1a4/Atp2a2	3
CC	GO:0005777	peroxisome	8/367	149/28739	4.2	0.000684758	0.004751806	0.002992404	Acaa1a/Cat/Mvd/Mvk/Pecr/Pex1/Pex13/Pex19	8
CC	GO:0042579	microbody	8/367	149/28739	4.2	0.000684758	0.004751806	0.002992404	Acaa1a/Cat/Mvd/Mvk/Pecr/Pex1/Pex13/Pex19	8
CC	GO:0016528	sarcoplasm	6/367	85/28739	5.53	0.000779748	0.005251829	0.003307288	Ank3/Atp2a2/Camk2d/Jph1/Jph3/S100a1	6
CC	GO:0099634	postsynaptic specialization membrane	6/367	85/28739	5.53	0.000779748	0.005251829	0.003307288	Atp2b2/Dlg1/Gabra3/Gsg1l/Lrrc4c/Shisa9	6
CC	GO:0098839	postsynaptic density membrane	5/367	60/28739	6.53	0.001014874	0.00659436	0.004152734	Atp2b2/Dlg1/Gsg1l/Lrrc4c/Shisa9	5
CC	GO:0033270	paranode region of axon	3/367	16/28739	14.68	0.00102227	0.00659436	0.004152734	Ank3/Dlg1/Kcna1	3
CC	GO:0098533	ATPase dependent transmembrane transport complex	3/367	16/28739	14.68	0.00102227	0.00659436	0.004152734	Atp1a1/Atp1a4/Atp2a2	3
CC	GO:0043195	terminal bouton	6/367	90/28739	5.22	0.001052555	0.00669542	0.004216376	Bdnf/Cck/Cplx1/Cplx3/L1cam/Sncb	6
CC	GO:0031594	neuromuscular junction	6/367	91/28739	5.16	0.00111494	0.006995104	0.004405099	Ank3/Apbb1/Camk2d/Dlg1/Nefm/Trip4	6
CC	GO:0010008	endosome membrane	11/367	284/28739	3.03	0.001149243	0.00711288	0.004479267	Abhd6/Arhgap1/Atp9a/Cdip1/Coro1c/Ehd2/Lamtor1/Ndrg1/Rab11b/Snx6/Sppl2b	11
CC	GO:0005684	U2-type spliceosomal complex	6/367	95/28739	4.95	0.001393259	0.008508166	0.005357935	Bud31/Prpf40a/Sf3b4/Sf3b5/Snrpg/Snu13	6
CC	GO:0005681	spliceosomal complex	9/367	208/28739	3.39	0.001495501	0.009012361	0.005675447	Alyref/Bud31/Ppwd1/Prpf40a/Sf3b4/Sf3b5/Snrpg/Snu13/Srsf2	9
CC	GO:0030285	integral component of synaptic vesicle membrane	3/367	19/28739	12.36	0.001719384	0.010095873	0.006357778	Atp2b1/Slc17a7/Slc30a3	3
CC	GO:0033268	node of Ranvier	3/367	19/28739	12.36	0.001719384	0.010095873	0.006357778	Ank3/Dlg1/Scn8a	3
CC	GO:0005923	bicellular tight junction	7/367	138/28739	3.97	0.002030955	0.011774397	0.007414812	Ank3/C1qtnf5/Cyth3/Dlg1/Frmd4a/Tjp1/Vasp	7
CC	GO:0032589	neuron projection membrane	5/367	72/28739	5.44	0.002295872	0.013143865	0.008277221	Atp2b1/Atp2b2/Gabra3/Ppp1r9b/Shisa9	5
CC	GO:0097470	ribbon synapse	3/367	22/28739	10.68	0.002656228	0.014919959	0.009395699	Atp2a2/Cplx3/Sh3gl2	3
CC	GO:0031252	cell leading edge	13/367	412/28739	2.47	0.002671259	0.014919959	0.009395699	Apbb1/Arhgap1/Atp2b1/Atp2b2/Coro1c/Cyth3/Gabra3/Kcna2/Ppp1r9b/Pxn/Shisa9/Stx2/Vasp	13
CC	GO:0008076	voltage-gated potassium channel complex	5/367	75/28739	5.22	0.002745593	0.01515038	0.009540805	Calm1/Kcna1/Kcna2/Kcnab2/Kcnip3	5
CC	GO:0016529	sarcoplasmic reticulum	5/367	76/28739	5.15	0.002908734	0.015859524	0.009987381	Ank3/Atp2a2/Camk2d/Jph1/S100a1	5
CC	GO:0034707	chloride channel complex	4/367	48/28739	6.53	0.003269378	0.017616177	0.011093616	Clcn2/Gabra3/Gabrb1/Slc26a6	4
CC	GO:0070160	tight junction	7/367	151/28739	3.63	0.003361155	0.017900103	0.011272416	Ank3/C1qtnf5/Cyth3/Dlg1/Frmd4a/Tjp1/Vasp	7
CC	GO:0005840	ribosome	9/367	236/28739	2.99	0.003496417	0.018406426	0.011591268	Dhx29/Eif2ak4/Mrpl27/Mrpl34/Mrpl43/Mrps5/Rpl15/Rpl18a/Rpl22l1	9
CC	GO:0098889	intrinsic component of presynaptic membrane	4/367	51/28739	6.14	0.004075207	0.021209598	0.013356539	Adam23/Atp2b1/Cplx3/Nrxn1	4
CC	GO:0005635	nuclear envelope	13/367	434/28739	2.35	0.004139932	0.021304371	0.013416221	Ahctf1/Cbx3/Dctn5/Dhcr7/Gabrb1/Ipo8/Nrxn1/Nup188/Rae1/Sec13/Sephs1/Stx1a/Zmpste24	13
CC	GO:0045177	apical part of cell	14/367	487/28739	2.25	0.004278745	0.02176033	0.013703357	Atp1a1/Atp2b1/Atp2b2/C1qtnf5/Cacnb3/Cd55b/Homer2/Kcna1/Myl12b/Numb/Prkaa1/Slc12a2/Slc26a6/Tjp1	14
CC	GO:0008287	protein serine/threonine phosphatase complex	4/367	52/28739	6.02	0.004371071	0.02176033	0.013703357	Ppp3ca/Ppp3cc/Ppp3r1/Ppp4c	4
CC	GO:0071005	U2-type precatalytic spliceosome	4/367	52/28739	6.02	0.004371071	0.02176033	0.013703357	Sf3b4/Sf3b5/Snrpg/Snu13	4
CC	GO:0005874	microtubule	13/367	438/28739	2.32	0.004466431	0.021995971	0.013851749	Calm1/Ccsap/Dlg1/Kcnab2/Kif1b/Klc4/Klhl22/Lrrc49/Map1a/Ndrg1/Rmdn1/Slc8a1/Tppp	13
CC	GO:1903293	phosphatase complex	4/367	53/28739	5.91	0.004681076	0.022493427	0.014165017	Ppp3ca/Ppp3cc/Ppp3r1/Ppp4c	4
CC	GO:0099055	integral component of postsynaptic membrane	5/367	85/28739	4.61	0.004706499	0.022493427	0.014165017	Abhd6/Gabra3/Gsg1l/Lrrc4c/Shisa9	5
CC	GO:0030426	growth cone	8/367	203/28739	3.09	0.004746758	0.022493427	0.014165017	Apbb1/Calm1/Cbarp/L1cam/Nrxn1/Ppp1r9b/Sncb/Syap1	8
CC	GO:0005689	U12-type spliceosomal complex	3/367	27/28739	8.7	0.004813004	0.022493427	0.014165017	Sf3b4/Sf3b5/Snrpg	3
CC	GO:0060077	inhibitory synapse	3/367	27/28739	8.7	0.004813004	0.022493427	0.014165017	Iqsec3/Nrxn1/Rims2	3
CC	GO:0055038	recycling endosome membrane	4/367	54/28739	5.8	0.005005523	0.022925295	0.014436982	Atp9a/Ehd2/Ndrg1/Rab11b	4
CC	GO:0071011	precatalytic spliceosome	4/367	54/28739	5.8	0.005005523	0.022925295	0.014436982	Sf3b4/Sf3b5/Snrpg/Snu13	4
CC	GO:0043296	apical junction complex	7/367	163/28739	3.36	0.005100292	0.023128056	0.014564669	Ank3/C1qtnf5/Cyth3/Dlg1/Frmd4a/Tjp1/Vasp	7
CC	GO:0042383	sarcolemma	7/367	165/28739	3.32	0.005445736	0.024452424	0.015398676	Ank3/Atp1a1/Cacnb3/Camk2d/Dlg1/Ppp3r1/Slc8a1	7
CC	GO:0034705	potassium channel complex	5/367	89/28739	4.4	0.005715471	0.025170053	0.015850597	Calm1/Kcna1/Kcna2/Kcnab2/Kcnip3	5
CC	GO:0098936	intrinsic component of postsynaptic membrane	5/367	89/28739	4.4	0.005715471	0.025170053	0.015850597	Abhd6/Gabra3/Gsg1l/Lrrc4c/Shisa9	5
CC	GO:0030427	site of polarized growth	8/367	210/28739	2.98	0.005797246	0.02525676	0.015905199	Apbb1/Calm1/Cbarp/L1cam/Nrxn1/Ppp1r9b/Sncb/Syap1	8
CC	GO:0005732	sno(s)RNA-containing ribonucleoprotein complex	3/367	29/28739	8.1	0.005900597	0.02525676	0.015905199	Fbll1/Nop56/Snu13	3
CC	GO:0032281	AMPA glutamate receptor complex	3/367	29/28739	8.1	0.005900597	0.02525676	0.015905199	Abhd6/Cnih2/Shisa9	3
CC	GO:0097525	spliceosomal snRNP complex	4/367	58/28739	5.4	0.006453567	0.027367904	0.017234672	Prpf40a/Sf3b5/Snrpg/Snu13	4
CC	GO:0016600	flotillin complex	2/367	10/28739	15.66	0.006839043	0.028218753	0.017770486	Coro1c/Sorbs1	2
CC	GO:0031080	nuclear pore outer ring	2/367	10/28739	15.66	0.006839043	0.028218753	0.017770486	Ahctf1/Sec13	2
CC	GO:0099569	presynaptic cytoskeleton	2/367	10/28739	15.66	0.006839043	0.028218753	0.017770486	Nefm/Rims2	2
CC	GO:0030027	lamellipodium	7/367	176/28739	3.11	0.007667859	0.031229879	0.019666713	Apbb1/Coro1c/Kcna2/Ppp1r9b/Pxn/Stx2/Vasp	7

BP	GO:0035493	SNARE complex assembly	3/607	18/28814	7.91	0.005996092	0.041876214	0.03032188	Snap25/Stxbp6/Vamp3	3
BP	GO:0046184	aldehyde biosynthetic process	3/607	18/28814	7.91	0.005996092	0.041876214	0.03032188	Clcn2/Dkk3/Tpi1	3
BP	GO:0048268	clathrin coat assembly	3/607	18/28814	7.91	0.005996092	0.041876214	0.03032188	Fcho1/Nsg2/Picalm	3
BP	GO:0070102	interleukin-6-mediated signaling pathway	3/607	18/28814	7.91	0.005996092	0.041876214	0.03032188	Gab1/Stat3/Tut4	3
BP	GO:0099150	regulation of postsynaptic specialization assembly	3/607	18/28814	7.91	0.005996092	0.041876214	0.03032188	Gap43/Nptx1/Ntrk3	3
BP	GO:1902855	regulation of non-motile cilium assembly	3/607	18/28814	7.91	0.005996092	0.041876214	0.03032188	Dnm2/Dynll1/Hap1	3
BP	GO:2000369	regulation of clathrin-dependent endocytosis	3/607	18/28814	7.91	0.005996092	0.041876214	0.03032188	Dnm2/Sh3gl3/Unc119	3
BP	GO:2000641	regulation of early endosome to late endosome transport	3/607	18/28814	7.91	0.005996092	0.041876214	0.03032188	Map2k2/Mapk3/Msn	3
BP	GO:0035051	cardiocyte differentiation	10/607	185/28814	2.57	0.006067976	0.042123949	0.030501261	Actn2/Dhx36/Lmna/Mapk3/Mef2c/Nebl/Pdcd4/Speg/Vangl2/Yy1	10
BP	GO:0051258	protein polymerization	13/607	276/28814	2.24	0.006070203	0.042123949	0.030501261	Arpc1b/Arpc3/Arpc5l/Cracd/Dbn1/Dbnl/Fgf13/Inpp5j/Kank4/Rac1/Ssh3/Tmod1/Vasp	13
BP	GO:0014743	regulation of muscle hypertrophy	6/607	78/28814	3.65	0.006083598	0.042123949	0.030501261	Atp2b4/Foxo1/Igfbp5/Lmna/Mef2c/Yy1	6
BP	GO:0000038	very long-chain fatty acid metabolic process	4/607	35/28814	5.43	0.006084472	0.042123949	0.030501261	Abcd3/Acot1/Acsbg1/Hacd3	4
BP	GO:0006084	acetyl-CoA metabolic process	4/607	35/28814	5.43	0.006084472	0.042123949	0.030501261	Dip2a/Hmgcs1/Nudt7/Pdk2	4
BP	GO:1904738	vascular associated smooth muscle cell migration	4/607	35/28814	5.43	0.006084472	0.042123949	0.030501261	Enpp1/Grb10/Igfbp5/Mef2c	4
BP	GO:0030048	actin filament-based movement	8/607	129/28814	2.94	0.006105087	0.042144508	0.030516147	Ctnna3/Dbn1/Fgf13/Flna/Myo1b/Pdpn/Scn1b/Wipf3	8
BP	GO:0031929	TOR signaling	8/607	129/28814	2.94	0.006105087	0.042144508	0.030516147	Akt1s1/Endog/Kptn/Lamtor1/Meak7/Pip4p1/Rragb/Syap1	8
BP	GO:0014066	regulation of phosphatidylinositol 3-kinase signaling	7/607	103/28814	3.23	0.006185575	0.042577076	0.030829363	Dab2ip/Dipk2a/Mydgf/Ntrk3/Pik3ip1/Ppp1r16b/Ptpn6	7
BP	GO:0021761	limbic system development	7/607	103/28814	3.23	0.006185575	0.042577076	0.030829363	Bax/Bbs2/Cdk5r2/Drd1/Fgf13/Hap1/Nrp2	7
BP	GO:0043010	camera-type eye development	16/607	374/28814	2.03	0.006248034	0.042945122	0.031095858	Ache/Aldh1a1/Bax/Bcar3/Celf4/Clcn2/Ntrk3/Phactr4/Prom1/Slc6a3/Sox8/Th/Tmod1/Tulp3/Vim/Yy1	16
BP	GO:0043406	positive regulation of MAP kinase activity	8/607	130/28814	2.92	0.00638889	0.043850186	0.031751201	Dab2ip/Gab1/Map3k13/Ntrk3/P2rx7/Tgfa/Traf6/Vangl2	8
BP	GO:0043090	amino acid import	5/607	56/28814	4.24	0.006439361	0.044069952	0.031910329	Gfap/Grm1/Slc1a3/Slc6a9/Slc7a5	5
BP	GO:0071709	membrane assembly	5/607	56/28814	4.24	0.006439361	0.044069952	0.031910329	Cdh2/Chmp5/Dmkn/Nlgn3/Picalm	5
BP	GO:0008643	carbohydrate transport	9/607	158/28814	2.7	0.006477043	0.044264429	0.032051147	Drd1/Enpp1/Grb10/Hk2/Osbpl8/Pls1/Prkcb/Smpd3/Sorbs1	9
BP	GO:0006814	sodium ion transport	12/607	248/28814	2.3	0.006481376	0.045595619	0.03301504	Asic1/Atp2b4/Dnm2/Ednrb/Fgf13/Hcn3/P2rx7/Scn1b/Slc10a4/Slc6a3/Slc6a9/Slc9a6	12
BP	GO:0006623	protein targeting to vacuole	4/607	36/28814	5.27	0.006732981	0.045686722	0.033081007	Clu/Lamp2/Mon1b/Zfyve16	4
BP	GO:0033866	nucleoside bisphosphate biosynthetic process	4/607	36/28814	5.27	0.006732981	0.045686722	0.033081007	Coasy/Dcakd/Dip2a/Pdk2	4
BP	GO:0034030	ribonucleoside bisphosphate biosynthetic process	4/607	36/28814	5.27	0.006732981	0.045686722	0.033081007	Coasy/Dcakd/Dip2a/Pdk2	4
BP	GO:0034033	purine nucleoside bisphosphate biosynthetic process	4/607	36/28814	5.27	0.006732981	0.045686722	0.033081007	Coasy/Dcakd/Dip2a/Pdk2	4
BP	GO:0043393	regulation of protein binding	11/607	218/28814	2.4	0.006812728	0.046162273	0.033425345	Actb/Anxa2/Aplp2/B2m/Bax/Camk1/Ckmt1/Epb41/Gre[illegible]	11
BP	GO:1990542	mitochondrial transmembrane transport	6/607	80/28814	3.56	0.006872418	0.046500765	0.033670442	Ccdc51/Mcu/Slc1a5/Slc25a18/Slc25a33/Slc25a5	6
BP	GO:0031529	ruffle organization	5/607	57/28814	4.16	0.006938911	0.046818047	0.03390018	Arf1/Dbnl/Pip5k1a/Rac1/Rac2	5
BP	GO:0086001	cardiac muscle cell action potential	5/607	57/28814	4.16	0.006938911	0.046818047	0.03390018	Ctnna3/Fgf13/Flna/Kcnd2/Scn1b	5
BP	GO:0010906	regulation of glucose metabolic process	8/607	132/28814	2.88	0.006986373	0.046901525	0.033960625	Arpp19/Enpp1/Foxo1/Grb10/Hmgb1/Igfbp4/Pdk2/Sorbs1	8
BP	GO:0043244	regulation of protein-containing complex disassembly	8/607	132/28814	2.88	0.006986373	0.046901525	0.033960625	Actn2/Arhgef2/Cracd/Fgf13/Irgm1/Ogfod1/Scaf8/Tmod1	8
BP	GO:0015919	peroxisomal membrane transport	3/607	19/28814	7.5	0.007010192	0.046901525	0.033960625	Abcd3/Pex1/Rab8b	3
BP	GO:0021854	hypothalamus development	3/607	19/28814	7.5	0.007010192	0.046901525	0.033960625	Bax/Hap1/Nrp2	3
BP	GO:0048199	vesicle targeting, to, from or within Golgi	3/607	19/28814	7.5	0.007010192	0.046901525	0.033960625	Dipk2a/Sar1b/Wipi1	3
BP	GO:0048484	enteric nervous system development	3/607	19/28814	7.5	0.007010192	0.046901525	0.033960625	Ednrb/Phactr4/Sox8	3
BP	GO:0060326	cell chemotaxis	14/607	314/28814	2.12	0.007198815	0.048096137	0.034825624	Camk1d/Ednrb/Gab1/Hmgb1/Hmgb2/Mapk3/Mcu/Pip5k1a/Pla2g7/Plekhg5/Rac1/Rac2/Thbs4/Vav1	14
BP	GO:0014909	smooth muscle cell migration	7/607	106/28814	3.13	0.007211972	0.048116746	0.034840547	Apex1/Enpp1/Grb10/Igfbp5/Mef2c/Ndrg4/S100a11	7
BP	GO:0009914	hormone transport	16/607	380/28814	2	0.007229653	0.048167435	0.03487725	Adcy8/Cplx1/Dynll1/Ensa/Ildr2/Kcnb1/Mcu/Niban2/Nucb2/Rab8b/Rac1/Slc18a2/Slc7a5/Smpd3/Snap25/Snx19	16
BP	GO:0032088	negative regulation of NF-kappaB transcription factor activity	6/607	81/28814	3.52	0.007293434	0.048457208	0.03508707	Anxa4/Commd6/Dab2ip/Dap/Dnaja3/Tut4	6
BP	GO:0034308	primary alcohol metabolic process	6/607	81/28814	3.52	0.007293434	0.048457208	0.03508707	Abhd4/Aldh1a1/Clcn2/Dkk3/Ednrb/Thtpa	6
BP	GO:0010506	regulation of autophagy	13/607	283/28814	2.18	0.007424954	0.049186074	0.03561483	Bag3/Dap/Elapor1/Endog/Foxo1/Gfap/Hmgb1/Irgm1/Mapk3/Rragb/Slc25a5/Stat3/Wipi1	13
BP	GO:0070873	regulation of glycogen metabolic process	4/607	37/28814	5.13	0.007425622	0.049186074	0.03561483	Enpp1/Grb10/Hmgb1/Sorbs1	4
BP	GO:0010257	NADH dehydrogenase complex assembly	5/607	58/28814	4.09	0.007464916	0.049186074	0.03561483	Ndufa10/Ndufa12/Ndufab1/Ndufaf3/Ndufb6	5
BP	GO:0032981	mitochondrial respiratory chain complex I assembly	5/607	58/28814	4.09	0.007464916	0.049186074	0.03561483	Ndufa10/Ndufa12/Ndufab1/Ndufaf3/Ndufb6	5
BP	GO:0043470	regulation of carbohydrate catabolic process	5/607	58/28814	4.09	0.007464916	0.049186074	0.03561483	Eif6/Hmgb1/P2rx7/Stat3/Zbtb20	5
BP	GO:0043666	regulation of phosphoprotein phosphatase activity	5/607	58/28814	4.09	0.007464916	0.049186074	0.03561483	Bod1/Ppp4r4/Ptpa/Rcan1/Vrk3	5
BP	GO:0000302	response to reactive oxygen species	10/607	191/28814	2.49	0.007535281	0.049581318	0.035901019	Apex1/Endog/Glrx2/Mapk3/Pdk2/Rhob/Sigmar1/Sirpa/Smpd3/Sod1	10
BP	GO:0007006	mitochondrial membrane organization	7/607	107/28814	3.11	0.007580596	0.049758902	0.036029605	Bax/Hk2/Micos13/Slc25a5/Stat3/Timm13/Timm9	7
BP	GO:0019216	regulation of lipid metabolic process	16/607	382/28814	1.99	0.007583103	0.049758902	0.036029605	Clcn2/Dab2ip/Dkk3/Eif6/Lamtor1/Nucb2/Opa3/Pdk2/Pik3ip1/Pla2g4a/Plcg2/Rac1/Smpd3/Sod1/Sorbs1/Zbtb20	16
BP	GO:0045913	positive regulation of carbohydrate metabolic process	6/607	82/28814	3.47	0.007732762	0.050671337	0.036690284	Arpp19/Foxo1/Hmgb1/P2rx7/Sorbs1/Zbtb20	6
BP	GO:0045637	regulation of myeloid cell differentiation	11/607	222/28814	2.35	0.007762613	0.050797264	0.036781466	B2m/C1qc/Eif6/Hmgb1/Hmgb2/Kdm1a/Mef2c/Setd1a/Stat1/Stat3/Traf6	11
BP	GO:0051047	positive regulation of secretion	17/607	417/28814	1.94	0.007803244	0.05099329	0.036923405	Adcy8/Cdk5r2/Dynll1/Ednrb/Frmd4a/Kcnb1/Mcu/Nucb2/P2rx7/Pla2g4a/Rab34/Rab3a/Rab8b/Rac1/Smpd3/Snap25/Stam	17
BP	GO:0050768	negative regulation of neurogenesis	9/607	163/28814	2.62	0.007882513	0.051440937	0.037247539	Arhgef2/B2m/Dip2b/Fgf13/Kdm1a/Lingo1/Ntrk3/Pcm1/Sema7a	9
BP	GO:0031123	RNA 3'-end processing	7/607	108/28814	3.08	0.007962957	0.051895012	0.037576327	Dhx36/Exosc10/Fip1l1/Larp7/Ncbp2/Scaf8/Tut4	7
BP	GO:0031279	regulation of cyclase activity	5/607	59/28814	4.02	0.008018057	0.051900079	0.037579996	Adcy3/Drd1/Gnal/Mapk3/Stim1	5
BP	GO:0043388	positive regulation of DNA binding	5/607	59/28814	4.02	0.008018057	0.051900079	0.037579996	Brd4/Hmgb1/Hmgb2/Niban2/Traf6	5
BP	GO:0051930	regulation of sensory perception of pain	5/607	59/28814	4.02	0.008018057	0.051900079	0.037579996	Comt/Ednrb/Grm1/Prkar1b/Tmem100	5
BP	GO:0097035	regulation of membrane lipid distribution	5/607	59/28814	4.02	0.008018057	0.051900079	0.037579996	Abca1/Ano3/P2rx7/Xkr5/Xkr9	5
BP	GO:1902475	L-alpha-amino acid transmembrane transport	5/607	59/28814	4.02	0.008018057	0.051900079	0.037579996	Grm1/Slc1a3/Slc25a18/Slc6a9/Slc7a5	5
BP	GO:0006071	glycerol metabolic process	3/607	20/28814	7.12	0.008119825	0.052205117	0.037800869	Coq3/Pla2g4a/Tpi1	3
BP	GO:0032495	response to muramyl dipeptide	3/607	20/28814	7.12	0.008119825	0.052205117	0.037800869	Arhgef2/Chmp5/Vim	3
BP	GO:0045019	negative regulation of nitric oxide biosynthetic process	3/607	20/28814	7.12	0.008119825	0.052205117	0.037800869	Atp2b4/Dynll1/Sirpa	3
BP	GO:0098969	neurotransmitter receptor transport to postsynaptic membrane	3/607	20/28814	7.12	0.008119825	0.052205117	0.037800869	Cplx1/Snap25/Stx3	3
BP	GO:1904406	negative regulation of nitric oxide metabolic process	3/607	20/28814	7.12	0.008119825	0.052205117	0.037800869	Atp2b4/Dynll1/Sirpa	3
CC	GO:0099060	integral component of postsynaptic specialization membrane	4/367	61/28739	5.13	0.007705188	0.031229879	0.019666713	Gabra3/Gsg1l/Lrrc4c/Shisa9	4
CC	GO:0042641	actomyosin	5/367	97/28739	4.04	0.008173991	0.032223824	0.02029264	Myl12b/Pxn/Sorbs1/Stx1a/Tlnrd1	5
CC	GO:0031461	cullin-RING ubiquitin ligase complex	8/367	223/28739	2.81	0.008206809	0.032223824	0.02029264	Cul2/Dcaf7/Fbxl16/Fbxo2/Klhdc10/Klhl22/Skp1/Zyg11b	8
CC	GO:0005890	sodium:potassium-exchanging ATPase complex	2/367	11/28739	14.24	0.008288644	0.032223824	0.02029264	Atp1a1/Atp1a4	2
CC	GO:0098684	photoreceptor ribbon synapse	2/367	11/28739	14.24	0.008288644	0.032223824	0.02029264	Cplx3/Sh3gl2	2
CC	GO:0016324	apical plasma membrane	11/367	369/28739	2.33	0.008302208	0.032223824	0.02029264	Atp1a1/Atp2b1/Atp2b2/C1qtnf5/Cacnb3/Cd55b/Kcna1/Prkaa1/Slc12a2/Slc26a6/Tjp1	11
CC	GO:0098948	intrinsic component of postsynaptic specialization membrane	4/367	63/28739	4.97	0.008622426	0.033185473	0.020898229	Gabra3/Gsg1l/Lrrc4c/Shisa9	4
CC	GO:0060076	excitatory synapse	4/367	64/28739	4.89	0.009106642	0.034757016	0.021887893	Cadps2/Elfn1/Ppp3r1/Slc17a7	4
CC	GO:0008180	COP9 signalosome	3/367	34/28739	6.91	0.009220593	0.034901088	0.021978621	Epb41l2/Tmod1/Wdr6	3
CC	GO:0005883	neurofilament	2/367	12/28739	13.05	0.009862947	0.036725444	0.023127491	Nefm/Pcp4	2
CC	GO:0098945	intrinsic component of presynaptic active zone membrane	2/367	12/28739	13.05	0.009862947	0.036725444	0.023127491	Cplx3/Nrxn1	2
CC	GO:0005912	adherens junction	6/367	142/28739	3.31	0.009988437	0.036892775	0.023232867	Cyth3/Dlg1/Frmd4a/Ndrg1/Sorbs1/Tjp1	6
CC	GO:0019005	SCF ubiquitin ligase complex	4/367	66/28739	4.75	0.010127451	0.037106982	0.023367761	Cul2/Fbxl16/Fbxo2/Skp1	4
CC	GO:0030532	small nuclear ribonucleoprotein complex	4/367	67/28739	4.68	0.010664501	0.038764615	0.02441164	Prpf40a/Sf3b5/Snrpg/Snu13	4
CC	GO:0043198	dendritic shaft	4/367	68/28739	4.61	0.011219607	0.040374613	0.02542552	Cnih2/Kirrel3/Map1a/Slc8a1	4
CC	GO:0031234	extrinsic component of cytoplasmic side of plasma membrane	5/367	105/28739	3.73	0.011283735	0.040374613	0.02542552	Atp2a2/Cyth3/Kcnab2/Syap1/Yes1	5
CC	GO:0099523	presynaptic cytosol	2/367	13/28739	12.05	0.011558546	0.041037319	0.025842853	Ppp3cc/Sh3gl2	2
CC	GO:0098798	mitochondrial protein-containing complex	9/367	286/28739	2.46	0.01171512	0.04127327	0.025991441	Mrpl27/Mrpl34/Mrpl43/Mrps5/Ndufa12/Ndufa9/Pdk1/Timm23/Timm8a1	9
CC	GO:0032040	small-subunit processome	3/367	38/28739	6.18	0.012521744	0.043446658	0.027360111	Fbll1/Nop56/Snu13	3
CC	GO:0032809	neuronal cell body membrane	3/367	38/28739	6.18	0.012521744	0.043446658	0.027360111	Atp2b1/Atp2b2/Kcna2	3
CC	GO:0044391	ribosomal subunit	7/367	195/28739	2.81	0.012986289	0.044719701	0.028161797	Dhx29/Mrpl27/Mrpl34/Mrpl43/Mrps5/Rpl15/Rpl18a	7
CC	GO:0005665	RNA polymerase II, core complex	2/367	14/28739	11.19	0.013372105	0.045593797	0.028712251	Polr2e/Polr2i	2
CC	GO:0099061	integral component of postsynaptic density membrane	3/367	39/28739	6.02	0.01343922	0.045593797	0.028712251	Gsg1l/Lrrc4c/Shisa9	3
CC	GO:0030176	integral component of endoplasmic reticulum membrane	6/367	155/28739	3.03	0.014880566	0.050112495	0.031557857	Dhcr7/Emc7/Preb/Sec61a1/Sppl2b/Zmpste24	6
CC	GO:0071004	U2-type prespliceosome	2/367	15/28739	10.44	0.015300352	0.050335284	0.031698156	Prpf40a/Snrpg	2
CC	GO:0071010	prespliceosome	2/367	15/28739	10.44	0.015300352	0.050335284	0.031698156	Prpf40a/Snrpg	2
CC	GO:0044298	cell body membrane	3/367	41/28739	5.73	0.015386331	0.050335284	0.031698156	Atp2b1/Atp2b2/Kcna2	3
CC	GO:0099146	intrinsic component of postsynaptic density membrane	3/367	41/28739	5.73	0.015386331	0.050335284	0.031698156	Gsg1l/Lrrc4c/Shisa9	3
CC	GO:0005643	nuclear pore	4/367	78/28739	4.02	0.01780704	0.057841306	0.036425001	Ahctf1/Nup188/Rae1/Sec13	4
CC	GO:0005876	spindle microtubule	4/367	79/28739	3.96	0.018573274	0.058362087	0.036752958	Calm1/Ccsap/Klhl22/Rmdn1	4
CC	GO:0120114	Sm-like protein family complex	4/367	79/28739	3.96	0.018573274	0.058362087	0.036752958	Prpf40a/Sf3b5/Snrpg/Snu13	4
CC	GO:0008328	ionotropic glutamate receptor complex	3/367	44/28739	5.34	0.018589667	0.058362087	0.036752958	Abhd6/Cnih2/Shisa9	3
CC	GO:0099056	integral component of presynaptic membrane	3/367	44/28739	5.34	0.018589667	0.058362087	0.036752958	Adam23/Atp2b1/Nrxn1	3
CC	GO:0031227	intrinsic component of endoplasmic reticulum membrane	6/367	163/28739	2.88	0.018604508	0.058362087	0.036752958	Dhcr7/Emc7/Preb/Sec61a1/Sppl2b/Zmpste24	6
CC	GO:0030139	endocytic vesicle	7/367	211/28739	2.6	0.019188152	0.059109717	0.03722377	Abca1/Ecpas/Ehd2/Nrxn1/Rab11b/Rabgef1/Sec61a1	7
CC	GO:0030684	preribosome	4/367	80/28739	3.92	0.019359667	0.059109717	0.03722377	Fbll1/Nop56/Snu13/Wdr3	4
CC	GO:0001518	voltage-gated sodium channel complex	2/367	17/28739	9.21	0.019488138	0.059109717	0.03722377	Scn4b/Scn8a	2
CC	GO:0030867	rough endoplasmic reticulum membrane	2/367	17/28739	9.21	0.019488138	0.059109717	0.03722377	Sec61a1/Sec61a2	2
CC	GO:0098688	parallel fiber to Purkinje cell synapse	2/367	17/28739	9.21	0.019488138	0.059109717	0.03722377	Cadps2/Ppp3r1	2
CC	GO:0015934	large ribosomal subunit	5/367	122/28739	3.21	0.02036811	0.06137233	0.038648629	Mrpl27/Mrpl34/Mrpl43/Rpl15/Rpl18a	5
CC	GO:0098878	neurotransmitter receptor complex	3/367	46/28739	5.11	0.020914838	0.062607815	0.039426664	Abhd6/Cnih2/Shisa9	3
CC	GO:0016460	myosin II complex	2/367	18/28739	8.7	0.021741448	0.064367704	0.040534937	Myl1/Myl12b	2
CC	GO:0005925	focal adhesion	6/367	169/28739	2.78	0.02178383	0.064367704	0.040534937	Lims1/Nexn/Pxn/Sorbs1/Sorbs3/Vasp	6
CC	GO:0005685	U1 snRNP	2/367	19/28739	8.24	0.024096985	0.070295662	0.044268011	Prpf40a/Snrpg	2
CC	GO:1902711	GABA-A receptor complex	2/367	19/28739	8.24	0.024096985	0.070295662	0.044268011	Gabra3/Gabrb1	2
CC	GO:0001725	stress fiber	4/367	88/28739	3.56	0.026390948	0.076004485	0.047863087	Myl12b/Pxn/Sorbs1/Tlnrd1	4
CC	GO:0097517	contractile actin filament bundle	4/367	88/28739	3.56	0.026390948	0.076004485	0.047863087	Myl12b/Pxn/Sorbs1/Tlnrd1	4
CC	GO:0099522	cytosolic region	2/367	20/28739	7.83	0.026551785	0.076004485	0.047863087	Ppp3cc/Sh3gl2	2
CC	GO:0005791	rough endoplasmic reticulum	4/367	89/28739	3.52	0.027363912	0.077362171	0.048718076	Glul/Pcsk1/Sec61a1/Sec61a2	4
CC	GO:0016363	nuclear matrix	4/367	89/28739	3.52	0.027363912	0.077362171	0.048718076	Ahctf1/Dcaf7/Prpf40a/Sorbs1	4
CC	GO:0019897	extrinsic component of plasma membrane	6/367	179/28739	2.62	0.027862879	0.078289563	0.049302092	Atp2a2/Cyth3/Kcnab2/Numb/Syap1/Yes1	6
CC	GO:0000313	organellar ribosome	4/367	90/28739	3.48	0.028358058	0.078715096	0.049570067	Mrpl27/Mrpl34/Mrpl43/Mrps5	4
CC	GO:0005761	mitochondrial ribosome	4/367	90/28739	3.48	0.028358058	0.078715096	0.049570067	Mrpl27/Mrpl34/Mrpl43/Mrps5	4
CC	GO:1902710	GABA receptor complex	2/367	21/28739	7.46	0.029102945	0.080296077	0.050565675	Gabra3/Gabrb1	2
CC	GO:0030055	cell-substrate junction	6/367	182/28739	2.58	0.029884349	0.081958276	0.051612428	Lims1/Nexn/Pxn/Sorbs1/Sorbs3/Vasp	6
CC	GO:0044853	plasma membrane raft	5/367	136/28739	2.88	0.030687873	0.083660987	0.052684694	Add2/Atp1a1/Coro1c/Ehd2/Sorbs1	5
CC	GO:0005686	U2 snRNP	2/367	22/28739	7.12	0.031747617	0.086037919	0.054181544	Sf3b5/Snrpg	2
CC	GO:0000151	ubiquitin ligase complex	9/367	341/28739	2.07	0.032057645	0.086367066	0.054388821	Cul2/Dcaf7/Dcun1d2/Fbxl16/Fbxo2/Klhdc10/Klhl22/Skp1/Zyg11b	9
CC	GO:0005901	caveola	4/367	96/28739	3.26	0.034771107	0.092588181	0.058306508	Atp1a1/Coro1c/Ehd2/Sorbs1	4
CC	GO:0071013	catalytic step 2 spliceosome	4/367	96/28739	3.26	0.034771107	0.092588181	0.058306508	Alyref/Bud31/Ppwd1/Snrpg	4
CC	GO:0000922	spindle pole	5/367	141/28739	2.78	0.035042507	0.092771493	0.058421947	Calm1/Klhl22/Rae1/Rmdn1/Rock2	5
CC	GO:0032432	actin filament bundle	4/367	97/28739	3.23	0.035915069	0.094535068	0.059532541	Myl12b/Pxn/Sorbs1/Tlnrd1	4
MF	GO:0044325	transmembrane transporter binding	15/359	147/28275	8.04	6.96963E-10	4.32117E-07	3.4408E-07	Ank3/Atp2a2/Calm1/Camk2d/Cbarp/Dlg1/Kcnab2/Kcnip3/Ppp1r9b/Rims2/Scn4b/Sh3gl1/Slc8a1/Stx1a/Yes1	15
MF	GO:0005516	calmodulin binding	13/359	192/28275	5.33	1.19034E-06	0.000254663	0.000202779	Add2/Atp2b1/Atp2b2/Camk2a/Camk2d/Camkk1/Itpka/Pcp4/Plcb1/Ppp3ca/Ppp3cc/Slc8a1/Tjp1	13
MF	GO:0015662	P-type ion transporter activity	6/359	30/28275	15.75	1.84441E-06	0.000254663	0.000202779	Atp1a1/Atp1a4/Atp2a2/Atp2b1/Atp2b2/Cpox	6
MF	GO:0140358	P-type transmembrane transporter activity	6/359	30/28275	15.75	1.84441E-06	0.000254663	0.000202779	Atp1a1/Atp1a4/Atp2a2/Atp2b1/Atp2b2/Cpox	6
MF	GO:0046873	metal ion transmembrane transporter activity	19/359	421/28275	3.55	2.25114E-06	0.000254663	0.000202779	Atp1a1/Atp1a4/Atp2a2/Atp2b1/Atp2b2/Cacnb3/Cpox/Kcna1/Kcna2/Kcnab2/Kcnip3/Scn4b/Scn8a/Sec61a1/Slc12a2/Slc17a7/Slc24a2/Slc30a3/Slc8a1	19

BP	GO:0021799	cerebral cortex radially oriented cell migration	4/607	38/28814	5	0.008163584	0.052345555	0.037902558	Adgrg1/Cdk5r2/Dab2ip/Rac1	4
BP	GO:0097106	postsynaptic density organization	4/607	38/28814	5	0.008163584	0.052345555	0.037902558	Cdh2/Dbn1/Nptx1/Ntrk3	4
BP	GO:0042310	vasoconstriction	7/607	109/28814	3.05	0.008359347	0.053457295	0.038707551	Atp2b1/Bbs2/Drd1/Ednrb/Manf/Ptgs1/Smpd3	7
BP	GO:0043255	regulation of carbohydrate biosynthetic process	7/607	109/28814	3.05	0.008359347	0.053457295	0.038707551	Arpp19/Enpp1/Foxo1/Grb10/Pdk2/Smpd3/Sorbs1	7
BP	GO:0015833	peptide transport	14/607	320/28814	2.08	0.008427556	0.053753725	0.038922191	Abca1/Adcy8/Cplx1/Dynll1/Ensa/Ildr2/Kcnb1/Mcu/Nucb2/Rab8b/Rac1/Slc18a2/Smpd3/Snx19	14
BP	GO:0042886	amide transport	15/607	353/28814	2.02	0.008428206	0.053753725	0.038922191	Abca1/Adcy8/Cplx1/Dynll1/Ensa/Ildr2/Kcnb1/Mcu/Nucb2/Rab8b/Rac1/Sgpp1/Slc18a2/Smpd3/Snx19	15
BP	GO:0035924	cellular response to vascular endothelial growth factor stimulus	5/607	60/28814	3.96	0.008599004	0.054624258	0.03955253	Atp2b4/Dab2ip/Gab1/Jcad/Nrp2	5
BP	GO:0051931	regulation of sensory perception	5/607	60/28814	3.96	0.008599004	0.054624258	0.03955253	Comt/Ednrb/Grm1/Prkar1b/Tmem100	5
BP	GO:0097479	synaptic vesicle localization	5/607	60/28814	3.96	0.008599004	0.054624258	0.03955253	Bloc1s6/Cdh2/Nlgn3/Picalm/Rab3a	5
BP	GO:0051222	positive regulation of protein transport	14/607	321/28814	2.07	0.008647568	0.05477165	0.039659253	Adcy8/Bag3/Camk1/Dynll1/Flna/Frmd4a/Kcnb1/Mcu/P2rx7/Pcm1/Ptpn5/Rab34/Rac1/Sar1b	14
BP	GO:0006275	regulation of DNA replication	8/607	137/28814	2.77	0.008664284	0.05477165	0.039659253	Bcar3/Cst3/Endog/Gtpbp4/Nucks1/Pcna/Rac1/Yv1	8
BP	GO:0002260	lymphocyte homeostasis	6/607	84/28814	3.39	0.008668069	0.05477165	0.039659253	Bax/Dnaja3/Enpp1/Jak3/Mef2c/P2rx7	6
BP	GO:0050848	regulation of calcium-mediated signaling	6/607	84/28814	3.39	0.008668069	0.05477165	0.039659253	Atp2b4/Homer2/Pdk2/Plcg2/Rcan1/Tmem100	6
BP	GO:0019318	hexose metabolic process	12/607	257/28814	2.22	0.008748165	0.055204732	0.039972841	Arpp19/Eno2/Enpp1/Foxo1/Ganc/Grb10/Hk2/Hmgb1/Igfbp4/Pdk2/Sorbs1/Tpi1	12
BP	GO:0042098	T cell proliferation	11/607	226/28814	2.31	0.008812489	0.05553728	0.040213634	Bax/Cd151/Dnaja3/Efnb1/Hmgb1/Jak3/Msn/P2rx7/Ptpn6/Rac2/Traf6	11
BP	GO:0016032	viral process	15/607	355/28814	2.01	0.008848616	0.055618207	0.040272232	Bax/Brd4/Chmp5/Cnot7/Crebbp/Hacd3/Larp7/Lgals1/Mon1b/Nucks1/Rad23a/Spcs1/Stat1/Trim25/Ube2i	15
BP	GO:0045787	positive regulation of cell cycle	15/607	355/28814	2.01	0.008848616	0.055618207	0.040272232	Apex1/Brd4/Camk2b/Cdk5r2/Crebbp/Gdpd5/Hmgb1/Hspa2/Larp7/Plcg2/Rad23a/Rcc2/Rps15a/Smpd3/Tqfa	15
BP	GO:0002931	response to ischemia	4/607	39/28814	4.87	0.00894801	0.055875348	0.040458423	Abraxas2/Hk2/Panx2/Rcan1	4
BP	GO:0032885	regulation of polysaccharide biosynthetic process	4/607	39/28814	4.87	0.00894801	0.055875348	0.040458423	Enpp1/Grb10/Smpd3/Sorbs1	4
BP	GO:0050931	pigment cell differentiation	4/607	39/28814	4.87	0.00894801	0.055875348	0.040458423	Bloc1s6/Ednrb/Enpp1/Mef2c	4
BP	GO:0070884	regulation of calcineurin-NFAT signaling cascade	4/607	39/28814	4.87	0.00894801	0.055875348	0.040458423	Atp2b4/Homer2/Plcg2/Rcan1	4
BP	GO:1905145	cellular response to acetylcholine	4/607	39/28814	4.87	0.00894801	0.055875348	0.040458423	Ache/Atp2b4/Rgs8/Sorbs1	4
BP	GO:0030072	peptide hormone secretion	13/607	290/28814	2.13	0.009008353	0.056178723	0.040678092	Adcy8/Cplx1/Dynll1/Ensa/Ildr2/Kcnb1/Mcu/Nucb2/Rab8b/Rac1/Slc18a2/Smpd3/Snx19	13
BP	GO:0045727	positive regulation of translation	8/607	138/28814	2.75	0.009033152	0.056186674	0.04068385	Coa3/Dhx36/Eif6/Mapk3/Paip1/Pld1/Rps9/Vim	8
BP	GO:0055076	transition metal ion homeostasis	8/607	138/28814	2.75	0.009033152	0.056186674	0.04068385	Aplp2/Atp6v1g1/B2m/Ftl1/Heph/Picalm/Slc6a9/Sod1	8
BP	GO:0051966	regulation of synaptic transmission, glutamatergic	6/607	85/28814	3.35	0.009164896	0.056468038	0.04088758	Cdh2/Dgki/Drd1/Grm1/Mef2c/Nlgn3	6
BP	GO:1902117	positive regulation of organelle assembly	6/607	85/28814	3.35	0.009164896	0.056468038	0.04088758	Dynll1/Elapor1/Hap1/Lcp1/Msn/Wipi1	6
BP	GO:0010970	transport along microtubule	9/607	167/28814	2.56	0.009165113	0.056468038	0.04088758	Bloc1s6/Copg1/Dynll1/Hap1/Hspb11/Ift81/Klc1/Pcm1/Sod1	9
BP	GO:0051961	negative regulation of nervous system development	9/607	167/28814	2.56	0.009165113	0.056468038	0.04088758	Arhgef2/B2m/Dip2b/Fgf13/Kdm1a/Lingo1/Ntrk3/Pcm1/Sema7a	9
BP	GO:0008344	adult locomotory behavior	7/607	111/28814	2.99	0.009195387	0.056468038	0.04088758	Cend1/Cstb/Drd1/Enpp1/Sez6/Sncg/Tmod1	7
BP	GO:0002011	morphogenesis of an epithelial sheet	5/607	61/28814	3.89	0.009208416	0.056468038	0.04088758	Cd151/Flna/Pdpn/Phactr4/Vangl2	5
BP	GO:0010656	negative regulation of muscle cell apoptotic process	5/607	61/28814	3.89	0.009208416	0.056468038	0.04088758	Acot1/Bag3/Dipk2a/Dnmt1/Naca	5
BP	GO:0042596	fear response	5/607	61/28814	3.89	0.009208416	0.056468038	0.04088758	Asic1/Drd1/Mef2c/Prkar1b/Vdac3	5
BP	GO:0099172	presynapse organization	5/607	61/28814	3.89	0.009208416	0.056468038	0.04088758	Efnb1/Farp1/Nlgn3/Ntrk3/Picalm	5
BP	GO:0140115	export across plasma membrane	5/607	61/28814	3.89	0.009208416	0.056468038	0.04088758	Abca1/Atp2b1/Kcnb1/Kcnip2/Rgs9	5
BP	GO:1903409	reactive oxygen species biosynthetic process	5/607	61/28814	3.89	0.009208416	0.056468038	0.04088758	Eef1a1/Plcg2/Slc25a33/Sod1/Stat3	5
BP	GO:0006622	protein targeting to lysosome	3/607	21/28814	6.78	0.009326887	0.056685163	0.041044797	Clu/Lamp2/Zfyve16	3
BP	GO:0007190	activation of adenylate cyclase activity	3/607	21/28814	6.78	0.009326887	0.056685163	0.041044797	Adcy3/Drd1/Gnal	3
BP	GO:0007252	I-kappaB phosphorylation	3/607	21/28814	6.78	0.009326887	0.056685163	0.041044797	Dab2ip/Plcg2/Sirpa	3
BP	GO:0048148	behavioral response to cocaine	3/607	21/28814	6.78	0.009326887	0.056685163	0.041044797	Crebbp/Drd1/Homer2	3
BP	GO:0051204	protein insertion into mitochondrial membrane	3/607	21/28814	6.78	0.009326887	0.056685163	0.041044797	Bax/Timm13/Timm9	3
BP	GO:0060333	interferon-gamma-mediated signaling pathway	3/607	21/28814	6.78	0.009326887	0.056685163	0.041044797	Dnaja3/Irgm1/Stat1	3
BP	GO:1903540	establishment of protein localization to postsynaptic membrane	3/607	21/28814	6.78	0.009326887	0.056685163	0.041044797	Cplx1/Snap25/Stx3	3
BP	GO:0055001	muscle cell development	11/607	228/28814	2.29	0.009376997	0.056917298	0.041212882	Actn2/Flnc/Kdm1a/Lmna/Mef2c/Naca/Nebl/Rcan1/Speg/Tmod1/Yy1	11
BP	GO:0009410	response to xenobiotic stimulus	14/607	325/28814	2.04	0.009573383	0.057987084	0.041987497	Aldh1a1/Dnmt1/Drd1/Mef2c/P2rx7/Pcna/Pde4a/Prkcb/Ptpn5/Slc10a4/Slc18a2/Slc1a3/Sod1/Ugt1a1	14
BP	GO:0030705	cytoskeleton-dependent intracellular transport	10/607	198/28814	2.4	0.009577519	0.057987084	0.041987497	Bloc1s6/Copg1/Dynll1/Hap1/Hspb11/Ift81/Klc1/Myo1b/Pcm1/Sod1	10
BP	GO:0071346	cellular response to interferon-gamma	7/607	112/28814	2.97	0.00963562	0.058265006	0.042188735	Dnaja3/Gbp2/Irgm1/Sirpa/Stat1/Vamp3/Vim	7
BP	GO:0010921	regulation of phosphatase activity	6/607	86/28814	3.31	0.009681732	0.058469827	0.042337043	Bod1/Mef2c/Ppp4r4/Ptpa/Rcan1/Vrk3	6
BP	GO:0006284	base-excision repair	4/607	40/28814	4.75	0.009779996	0.058692264	0.042498106	Apex1/Hmga1/Hmgb1/Pcna	4
BP	GO:0006739	NADP metabolic process	4/607	40/28814	4.75	0.009779996	0.058692264	0.042498106	Aldh1l1/Me2/Noct/Nudt12	4
BP	GO:0051349	positive regulation of lyase activity	4/607	40/28814	4.75	0.009779996	0.058692264	0.042498106	Adcy3/Drd1/Gnal/Stim1	4
BP	GO:0070849	response to epidermal growth factor	4/607	40/28814	4.75	0.009779996	0.058692264	0.042498106	Dab2ip/Eef1a1/Mapk3/Svap1	4
BP	GO:0106056	regulation of calcineurin-mediated signaling	4/607	40/28814	4.75	0.009779996	0.058692264	0.042498106	Atp2b4/Homer2/Plcg2/Rcan1	4
BP	GO:0008652	cellular amino acid biosynthetic process	5/607	62/28814	3.83	0.009846938	0.058872121	0.042628337	Aass/Aldh1a1/Atp2b4/Sephs1/Slc1a3	5
BP	GO:0031663	lipopolysaccharide-mediated signaling pathway	5/607	62/28814	3.83	0.009846938	0.058872121	0.042628337	Mapk3/Plcg2/Stat1/Traf6/Tut4	5
BP	GO:0051932	synaptic transmission, GABAergic	5/607	62/28814	3.83	0.009846938	0.058872121	0.042628337	Gabra3/Gabra4/Gabrb2/Hap1/Rac1	5
BP	GO:0050920	regulation of chemotaxis	11/607	230/28814	2.27	0.009969002	0.059527401	0.043102815	Camk1d/Hmgb1/Mapk3/Mcu/Ntrk3/Pla2g7/Rac1/Rac2/Sema7a/Stx3/Tbs4	11
BP	GO:0030516	regulation of axon extension	7/607	113/28814	2.94	0.010091047	0.060180941	0.043576032	Dbn1/Dbnl/Dip2b/Dnm2/Map3k13/Ntrk3/Sema7a	7

MF	GO:0016247	channel regulator activity	11/359	144/28275	6.02	2.46448E-06	0.000254663	0.000202779	Arpp19/Cacnb3/Calm1/Camk2d/Cnih2/Dlg1/Kcnab2/Kcnip3/Nrxn1/Scn4b/Stx1a	11
MF	GO:0022804	active transmembrane transporter activity	18/359	396/28275	3.58	3.76949E-06	0.000333869	0.000265848	Abca1/Abcb7/Atp1a1/Atp1a4/Atp2a2/Atp2b1/Atp2b2/Cox7a1/Cpox/Ipo8/Slc12a2/Slc17a7/Slc24a2/Slc25a10/Slc25a14/Slc26a6/Slc4a1/Slc8a1	18
MF	GO:0042626	ATPase-coupled transmembrane transporter activity	9/359	102/28275	6.95	6.29526E-06	0.000487882	0.000388484	Abca1/Abcb7/Atp1a1/Atp1a4/Atp2a2/Atp2b1/Atp2b2/Cpox/Ipo8	9
MF	GO:0060090	molecular adaptor activity	18/359	421/28275	3.37	8.72235E-06	0.000600873	0.000478454	Ank3/Cul2/Dlg1/Ecpas/Frmd4a/Homer2/Klhdc10/Lamtor1/Map1a/Napa/Pxn/Septin4/Sgtb/Skp1/Sorbs1/Stat3/Stx1a/Stx2	18
MF	GO:0099106	ion channel regulator activity	10/359	136/28275	5.79	9.91114E-06	0.000614491	0.000489297	Arpp19/Cacnb3/Calm1/Camk2d/Dlg1/Kcnab2/Kcnip3/Nrxn1/Scn4b/Stx1a	10
MF	GO:0022853	active ion transmembrane transporter activity	13/359	238/28275	4.3	1.25028E-05	0.000704706	0.000561132	Atp1a1/Atp1a4/Atp2a2/Atp2b1/Atp2b2/Cox7a1/Cpox/Slc12a2/Slc17a7/Slc24a2/Slc26a6/Slc4a1/Slc8a1	13
MF	GO:0051020	GTPase binding	15/359	321/28275	3.68	1.75473E-05	0.000879974	0.000700693	Abca1/Arhgap1/Coro1c/Dennd1a/Ipo8/Itpka/Lamtor1/Mycbp2/Ndrg1/Preb/Rab3ip/Rabgef1/Rims2/Rock2/Sh3gl1	15
MF	GO:0015103	inorganic anion transmembrane transporter activity	10/359	146/28275	5.39	1.84511E-05	0.000879974	0.000700693	Clcn2/Gabra3/Gabrb1/Slc12a2/Slc17a7/Slc25a10/Slc25a14/Slc26a6/Slc4a1/Xpr1	10
MF	GO:0005048	signal sequence binding	6/359	46/28275	10.27	2.45207E-05	0.00105997	0.000844017	Cat/Pex13/Pex19/Ranbp6/Sec61a1/Sec61a2	6
MF	GO:0015399	primary active transmembrane transporter activity	10/359	152/28275	5.18	2.6142E-05	0.00105997	0.000844017	Abca1/Abcb7/Atp1a1/Atp1a4/Atp2a2/Atp2b1/Atp2b2/Cox7a1/Cpox/Ipo8	10
MF	GO:0019829	ATPase-coupled cation transmembrane transporter activity	6/359	47/28275	10.05	2.78107E-05	0.00105997	0.000844017	Atp1a1/Atp1a4/Atp2a2/Atp2b1/Atp2b2/Cpox	6
MF	GO:0048306	calcium-dependent protein binding	8/359	95/28275	6.63	2.90637E-05	0.00105997	0.000844017	Alg2/Calm1/Kcnip3/Nrxn1/S100a1/Stx1a/Stx2/Svn1	8
MF	GO:0008320	protein transmembrane transporter activity	4/359	16/28275	19.69	4.12236E-05	0.001417658	0.001128831	Abca1/Sec61a1/Sec61a2/Timm23	4
MF	GO:0050839	cell adhesion molecule binding	14/359	307/28275	3.59	4.34444E-05	0.001417658	0.001128831	Adam15/Ank3/Cd47/Kirrel3/L1cam/Lrrc4c/Ndrg1/Nexn/Nrxn1/Numb/Pxn/Sirpa/Tenm3/Tjp1	14
MF	GO:0008022	protein C-terminus binding	12/359	232/28275	4.07	4.57884E-05	0.00141944	0.00113025	Atp2a2/Atp2b2/Dlg1/Kcnip3/Pex1/Ppp1r9b/Prkaa1/Pxk/Sh3gl1/Sh3gl2/Slc4a1/Tjp1	12
MF	GO:0005216	ion channel activity	17/359	438/28275	3.06	5.21772E-05	0.001540468	0.001226621	Cacnb3/Clcn2/Gabra3/Gabrb1/Kcna1/Kcna2/Kcnab2/Kcnip3/Scn4b/Scn8a/Sec61a1/Slc12a2/Slc17a7/Slc24a2/Slc26a6/Tmem63b/Unc80	17
MF	GO:0015081	sodium ion transmembrane transporter activity	9/359	145/28275	4.89	0.000103429	0.002914809	0.00232096	Atp1a1/Atp1a4/Cpox/Scn4b/Scn8a/Slc12a2/Slc17a7/Slc24a2/Slc8a1	9
MF	GO:0008307	structural constituent of muscle	4/359	21/28275	15	0.000128925	0.003197349	0.002545936	Csrp1/Jph1/Myl1/Nexn	4
MF	GO:0022884	macromolecule transmembrane transporter activity	4/359	21/28275	15	0.000128925	0.003197349	0.002545936	Abca1/Sec61a1/Sec61a2/Timm23	4
MF	GO:0140318	protein transporter activity	4/359	21/28275	15	0.000128925	0.003197349	0.002545936	Abca1/Sec61a1/Sec61a2/Timm23	4
MF	GO:0030165	PDZ domain binding	8/359	120/28275	5.25	0.000152744	0.003642361	0.002900284	Atp2b1/Atp2b2/Dlg1/Kirrel3/L1cam/Shisa9/Slc26a6/Tmem88b	8
MF	GO:0015267	channel activity	17/359	482/28275	2.78	0.000165424	0.003662965	0.00291669	Cacnb3/Clcn2/Gabra3/Gabrb1/Kcna1/Kcna2/Kcnab2/Kcnip3/Scn4b/Scn8a/Sec61a1/Slc12a2/Slc17a7/Slc24a2/Slc26a6/Tmem63b/Unc80	17
MF	GO:0022803	passive transmembrane transporter activity	17/359	482/28275	2.78	0.000165424	0.003662965	0.00291669	Cacnb3/Clcn2/Gabra3/Gabrb1/Kcna1/Kcna2/Kcnab2/Kcnip3/Scn4b/Scn8a/Sec61a1/Slc12a2/Slc17a7/Slc24a2/Slc26a6/Tmem63b/Unc80	17
MF	GO:0016627	oxidoreductase activity, acting on the CH-CH group of donors	6/359	65/28275	7.27	0.000176613	0.003775871	0.003006593	Acaa1a/Cpox/Dhcr7/Dus3l/Gcdh/Pecr	6
MF	GO:0015079	potassium ion transmembrane transporter activity	9/359	157/28275	4.51	0.000188973	0.003905443	0.003109767	Atp1a1/Atp1a4/Cpox/Kcna1/Kcna2/Kcnab2/Kcnip3/Slc12a2/Slc24a2	9
MF	GO:0004683	calmodulin-dependent protein kinase activity	4/359	24/28275	13.13	0.000222119	0.004157041	0.003310105	Camk2a/Camk2d/Camkk1/Itpka	4
MF	GO:0035254	glutamate receptor binding	6/359	68/28275	6.95	0.000226691	0.004157041	0.003310105	Atp2b2/Calm1/Camk2a/Dlg1/Homer2/Map1a	6
MF	GO:0005391	P-type sodium:potassium-exchanging transporter activity	3/359	10/28275	23.63	0.000227967	0.004157041	0.003310105	Atp1a1/Atp1a4/Cpox	3
MF	GO:0008554	P-type sodium transporter activity	3/359	10/28275	23.63	0.000227967	0.004157041	0.003310105	Atp1a1/Atp1a4/Cpox	3
MF	GO:0140104	molecular carrier activity	6/359	70/28275	6.75	0.000265851	0.00470936	0.003749898	Emc7/Hbb-b1/Oxa1l/Pex19/Ranbp6/Urm1	6
MF	GO:0030674	protein-macromolecule adaptor activity	13/359	326/28275	3.14	0.0003025	0.00493626	0.003930571	Ank3/Cul2/Frmd4a/Homer2/Klhdc10/Map1a/Napa/Pxn/Skp1/Sorbs1/Stat3/Stx1a/Stx2	13
MF	GO:0017124	SH3 domain binding	8/359	133/28275	4.74	0.000308965	0.00493626	0.003930571	Adam10/Adam15/Arhgap1/Dennd1a/Elmo2/Sh3gl1/Sirpa/Vasp	8
MF	GO:0005388	P-type calcium transporter activity	3/359	11/28275	21.48	0.000310507	0.00493626	0.003930571	Atp2a2/Atp2b1/Atp2b2	3
MF	GO:0008556	P-type potassium transmembrane transporter activity	3/359	11/28275	21.48	0.000310507	0.00493626	0.003930571	Atp1a1/Atp1a4/Cpox	3
MF	GO:0000287	magnesium ion binding	10/359	211/28275	3.73	0.000392335	0.006081195	0.004842242	Atp8a2/Atp9a/Cerk/Glul/Kcnip3/Mvk/Nudt3/Prps1/Septin4/Tppp	10
MF	GO:0016018	cyclosporin A binding	3/359	13/28275	18.18	0.000528146	0.007986604	0.006359452	Ppp3ca/Ppp3r1/Ppwd1	3
MF	GO:0015108	chloride transmembrane transporter activity	7/359	111/28275	4.97	0.000550556	0.008127259	0.006471451	Clcn2/Gabra3/Gabrb1/Slc12a2/Slc17a7/Slc26a6/Slc4a1	7
MF	GO:0003779	actin binding	15/359	444/28275	2.66	0.000617418	0.008902313	0.007088599	Add2/Arpc5l/Coro1c/Epb41l2/Hdgf/Homer2/Map1a/Mrtfa/Nexn/Ppp1r9b/Pxk/Slc4a1/Tlnrd1/Tmod1/Vasp	15
MF	GO:0015297	antiporter activity	6/359	83/28275	5.69	0.000666793	0.009395719	0.007481481	Slc24a2/Slc25a10/Slc25a14/Slc26a6/Slc4a1/Slc8a1	6
MF	GO:0016779	nucleotidyltransferase activity	7/359	120/28275	4.59	0.000875297	0.011823109	0.009414326	Coasy/Crppa/Pcyt1b/Polr2e/Polr2i/Tut7/Uap1	7
MF	GO:0098632	cell-cell adhesion mediator activity	4/359	34/28275	9.27	0.000877198	0.011823109	0.009414326	Cd47/Lrrc4c/Nexn/Sirpa	4
MF	GO:0022836	gated channel activity	12/359	323/28275	2.93	0.000948389	0.012510668	0.009961805	Cacnb3/Clcn2/Gabra3/Gabrb1/Kcna1/Kcna2/Kcnab2/Kcnip3/Scn4b/Scn8a/Slc17a7/Tmem63b	12
MF	GO:0015116	sulfate transmembrane transporter activity	3/359	16/28275	14.77	0.001005304	0.012985176	0.010339639	Slc25a10/Slc25a14/Slc26a6	3
MF	GO:0031267	small GTPase binding	11/359	285/28275	3.04	0.001127533	0.014266748	0.01136011	Abca1/Arhgap1/Coro1c/Dennd1a/Ipo8/Itpka/Mycbp2/Ndrg1/Rabgef1/Rims2/Rock2	11
MF	GO:0005326	neurotransmitter transmembrane transporter activity	3/359	17/28275	13.9	0.00120929	0.014668424	0.011679951	Cplx1/Cplx3/Slc17a7	3
MF	GO:0140597	protein carrier chaperone	3/359	17/28275	13.9	0.00120929	0.014668424	0.011679951	Emc7/Oxa1l/Pex19	3
MF	GO:0005261	cation channel activity	12/359	333/28275	2.84	0.001230255	0.014668424	0.011679951	Cacnb3/Kcna1/Kcna2/Kcnab2/Kcnip3/Scn4b/Scn8a/Sec61a1/Slc12a2/Slc24a2/Tmem63b/Unc80	12
MF	GO:0015085	calcium ion transmembrane transporter activity	7/359	134/28275	4.11	0.001661747	0.019439308	0.015478838	Atp2a2/Atp2b1/Atp2b2/Cacnb3/Sec61a1/Slc24a2/Slc8a1	7
MF	GO:0005246	calcium channel regulator activity	4/359	41/28275	7.68	0.001786522	0.020511917	0.016332919	Cacnb3/Calm1/Nrxn1/Stx1a	4
MF	GO:0033218	amide binding	14/359	452/28275	2.44	0.002089235	0.023551371	0.018753129	Apbb1/Cat/Epdr1/Fbxo2/Gcdh/Memo1/Pex13/Pex19/Ppp3ca/Ppp3r1/Ppwd1/Ranbp6/Sec61a1/Sec61a2	14
MF	GO:0043022	ribosome binding	5/359	72/28275	5.47	0.002238697	0.024763308	0.019718152	Gcn1/Ncln/Oxa1l/Sec61a1/Sec61a2	5
MF	GO:0099095	ligand-gated anion channel activity	3/359	21/28275	11.25	0.00227797	0.024763308	0.019718152	Gabra3/Gabrb1/Slc17a7	3
MF	GO:0098631	cell adhesion mediator activity	4/359	44/28275	7.16	0.002324465	0.024763308	0.019718152	Cd47/Lrrc4c/Nexn/Sirpa	4
MF	GO:0008509	anion transmembrane transporter activity	11/359	313/28275	2.77	0.002356508	0.024763308	0.019718152	Clcn2/Gabra3/Gabrb1/Slc12a2/Slc17a7/Slc24a2/Slc25a10/Slc25a14/Slc26a6/Slc4a1/Xpr1	11
MF	GO:0030506	ankyrin binding	3/359	23/28275	10.27	0.002976925	0.030652098	0.024407189	Atp1a1/Slc4a1/Slc8a1	3
MF	GO:0019902	phosphatase binding	9/359	233/28275	3.04	0.003092321	0.030652098	0.024407189	Dlg1/Mvp/Ppp1r9b/Ppp3r1/Pxn/Sh3gl1/Sirpa/Stat3/Stau1	9
MF	GO:0030695	GTPase regulator activity	13/359	422/28275	2.43	0.003114649	0.030652098	0.024407189	Arhgap1/Arhgap39/Arl2bp/Cyth3/Dennd1a/Iqsec3/Lamtor1/Mycbp2/Plcb1/Preb/Rab3ip/Rabgef1/Sirpa	13
MF	GO:0060589	nucleoside-triphosphatase regulator activity	13/359	422/28275	2.43	0.003114649	0.030652098	0.024407189	Arhgap1/Arhgap39/Arl2bp/Cyth3/Dennd1a/Iqsec3/Lamtor1/Mycbp2/Plcb1/Preb/Rab3ip/Rabgef1/Sirpa	13

BP	GO:0008584	male gonad development	8/607	141/28814	2.69	0.01021017	0.060643802	0.043911182	Adgrg1/Bax/Csmd1/Flna/Hmgb2/Msh2/Sgpl1/Sox8	8
BP	GO:1904375	regulation of protein localization to cell periphery	8/607	141/28814	2.69	0.01021017	0.060643802	0.043911182	Actb/Atp2b4/Camk2b/Epb41/Picalm/Pls1/Sorbs1/Stx3	8
BP	GO:0035418	protein localization to synapse	6/607	87/28814	3.27	0.010218991	0.060643802	0.043911182	Cplx1/Klc1/Nlgn3/Nptx1/Snap25/Stx3	6
BP	GO:0010950	positive regulation of endopeptidase activity	9/607	170/28814	2.51	0.010226199	0.060643802	0.043911182	Bax/Dap/Dnaja3/Hmgb1/Picalm/Psme1/Psme2/Stat1/Stat3	9
BP	GO:0043405	regulation of MAP kinase activity	10/607	200/28814	2.37	0.010232134	0.060643802	0.043911182	Dab2ip/Gab1/Map3k13/Ntrk3/P2rx7/Ptpn5/Ptpn6/Tgfa/Traf6/Vangl2	10
BP	GO:0044091	membrane biogenesis	5/607	63/28814	3.77	0.010515206	0.062167251	0.045014287	Cdh2/Chmp5/Dmkn/Nlgn3/Picalm	5
BP	GO:0045744	negative regulation of G protein-coupled receptor signaling pathway	5/607	63/28814	3.77	0.010515206	0.062167251	0.045014287	Atp2b4/Camk2b/Dnm2/Grk5/Rnf157	5
BP	GO:0046546	development of primary male sexual characteristics	8/607	142/28814	2.67	0.010626691	0.062171291	0.045017213	Adgrg1/Bax/Csmd1/Flna/Hmgb2/Msh2/Sgpl1/Sox8	8
BP	GO:0006684	sphingomyelin metabolic process	3/607	22/28814	6.47	0.010633022	0.062171291	0.045017213	Abca8b/Smpd3/Smpd4	3
BP	GO:0036344	platelet morphogenesis	3/607	22/28814	6.47	0.010633022	0.062171291	0.045017213	Bap1/Mef2c/Ptpn6	3
BP	GO:0043574	peroxisomal transport	3/607	22/28814	6.47	0.010633022	0.062171291	0.045017213	Abcd3/Pex1/Rab8b	3
BP	GO:0050849	negative regulation of calcium-mediated signaling	3/607	22/28814	6.47	0.010633022	0.062171291	0.045017213	Atp2b4/Homer2/Rcan1	3
BP	GO:0060252	positive regulation of glial cell proliferation	3/607	22/28814	6.47	0.010633022	0.062171291	0.045017213	Gfap/Ppp1cc/Vim	3
BP	GO:0061081	positive regulation of myeloid leukocyte cytokine production involved in immune response	3/607	22/28814	6.47	0.010633022	0.062171291	0.045017213	Dhx36/Plcg2/Sema7a	3
BP	GO:0098877	neurotransmitter receptor transport to plasma membrane	3/607	22/28814	6.47	0.010633022	0.062171291	0.045017213	Cplx1/Snap25/Stx3	3
BP	GO:2001224	positive regulation of neuron migration	3/607	22/28814	6.47	0.010633022	0.062171291	0.045017213	Arhgef2/Dab2ip/Flna	3
BP	GO:0001504	neurotransmitter uptake	4/607	41/28814	4.63	0.010660592	0.062180279	0.045023721	Gfap/Slc18a2/Slc6a3/Slc6a9	4
BP	GO:0046627	negative regulation of insulin receptor signaling pathway	4/607	41/28814	4.63	0.010660592	0.062180279	0.045023721	Enpp1/Grb10/Nucb2/Prkcb	4
BP	GO:0045444	fat cell differentiation	12/607	264/28814	2.16	0.010675856	0.062193372	0.045033201	Bbs2/Cds1/Enpp1/Foxo1/Grk5/Noct/Nucb2/Nudt7/Opa3/Osbpl8/Sox8/Syap1	12
BP	GO:0051480	regulation of cytosolic calcium ion concentration	16/607	397/28814	1.91	0.010697436	0.062243184	0.045069269	Adcy8/Atp2b1/Atp2b4/Bax/Drd1/Ednrb/Grm1/Hap1/Hmgb1/Jak3/P2rx7/Plcg2/Ptpn6/Ryr3/Smpd3/Tmem38a	16
BP	GO:0006637	acyl-CoA metabolic process	6/607	88/28814	3.24	0.010777085	0.062478318	0.045239525	Acot1/Acot11/Dip2a/Hmgcs1/Nudt7/Pdk2	6
BP	GO:0035383	thioester metabolic process	6/607	88/28814	3.24	0.010777085	0.062478318	0.045239525	Acot1/Acot11/Dip2a/Hmgcs1/Nudt7/Pdk2	6
BP	GO:0040014	regulation of multicellular organism growth	6/607	88/28814	3.24	0.010777085	0.062478318	0.045239525	Bbs2/Pls1/Sgpl1/Slc6a3/Sod1/Stat3	6
BP	GO:0032231	regulation of actin filament bundle assembly	7/607	115/28814	2.89	0.01104864	0.06386079	0.046240551	Arhgap6/Arhgef10/Dbn1/Flna/Kank4/Pdlim4/Rac1	7
BP	GO:1903076	regulation of protein localization to plasma membrane	7/607	115/28814	2.89	0.01104864	0.06386079	0.046240551	Actb/Atp2b4/Camk2b/Picalm/Pls1/Sorbs1/Stx3	7
BP	GO:0042471	ear morphogenesis	8/607	143/28814	2.66	0.011055657	0.06386079	0.046240551	Enpp1/Gjb6/Mapk3/Pls1/Ptk7/Rac1/Sod1/Vangl2	8
BP	GO:0061077	chaperone-mediated protein folding	5/607	64/28814	3.71	0.011213839	0.064696265	0.046845504	Clu/Dnajb6/Hspa2/Hspe1/Unc45a	5
BP	GO:1903522	regulation of blood circulation	12/607	266/28814	2.14	0.011282778	0.06501548	0.047076643	Atp2b1/Atp2b4/Ctnna3/Drd1/Ednrb/Fgf13/Flna/Myl4/Ptgs1/Scn1b/Th/Tmem38a	12
BP	GO:0046323	glucose import	6/607	89/28814	3.2	0.011356419	0.065360978	0.047326813	Drd1/Enpp1/Grb10/Hk2/Osbpl8/Sorbs1	6
BP	GO:0048017	inositol lipid-mediated signaling	9/607	173/28814	2.47	0.011377158	0.065401547	0.047356188	Dab2ip/Dipk2a/Mydgf/Ntrk3/Pik3ip1/Plcd3/Pld1/Ppp1r16b/Ptpn6	9
BP	GO:0051054	positive regulation of DNA metabolic process	13/607	299/28814	2.06	0.011420026	0.065525725	0.047446103	Actb/Bax/Bcar3/Cst3/Dhx36/Endog/Hmgb1/Mapk3/Msh2/Pcna/Rac1/Tnks/Yy1	13
BP	GO:0019751	polyol metabolic process	7/607	116/28814	2.86	0.011551376	0.065525725	0.047446103	Coq3/Inpp5j/Itpka/Pla2g4a/Plcg2/Sgpl1/Tpi1	7
BP	GO:0030038	contractile actin filament bundle assembly	7/607	116/28814	2.86	0.011551376	0.065525725	0.047446103	Arhgap6/Arhgef10/Kank4/Pdlim4/Phactr1/Rac1/Sorbs1	7
BP	GO:0043149	stress fiber assembly	7/607	116/28814	2.86	0.011551376	0.065525725	0.047446103	Arhgap6/Arhgef10/Kank4/Pdlim4/Phactr1/Rac1/Sorbs1	7
BP	GO:0051952	regulation of amine transport	7/607	116/28814	2.86	0.011551376	0.065525725	0.047446103	Kcnb1/P2rx7/Prkcb/Ptgs1/Rab3a/Sncg/Syt17	7
BP	GO:0000184	nuclear-transcribed mRNA catabolic process, nonsense-mediated decay	4/607	42/28814	4.52	0.011590797	0.065525725	0.047446103	Dcp1a/Exosc10/Ncbp2/Smg5	4
BP	GO:0014046	dopamine secretion	4/607	42/28814	4.52	0.011590797	0.065525725	0.047446103	Prkcb/Rab3a/Sncg/Syt17	4
BP	GO:0014059	regulation of dopamine secretion	4/607	42/28814	4.52	0.011590797	0.065525725	0.047446103	Prkcb/Rab3a/Sncg/Syt17	4
BP	GO:0031076	embryonic camera-type eye development	4/607	42/28814	4.52	0.011590797	0.065525725	0.047446103	Aldh1a1/Phactr4/Th/Tulp3	4
BP	GO:0033363	secretory granule organization	4/607	42/28814	4.52	0.011590797	0.065525725	0.047446103	Abca1/Acrbp/Ccdc136/Snx19	4
BP	GO:0038084	vascular endothelial growth factor signaling pathway	4/607	42/28814	4.52	0.011590797	0.065525725	0.047446103	Dab2ip/Gab1/Jcad/Nrp2	4
BP	GO:0045022	early endosome to late endosome transport	4/607	42/28814	4.52	0.011590797	0.065525725	0.047446103	Flna/Map2k2/Mapk3/Msn	4
BP	GO:0099174	regulation of presynapse organization	4/607	42/28814	4.52	0.011590797	0.065525725	0.047446103	Farp1/Nlgn3/Ntrk3/Picalm	4
BP	GO:1905144	response to acetylcholine	4/607	42/28814	4.52	0.011590797	0.065525725	0.047446103	Ache/Atp2b4/Rgs8/Sorbs1	4
BP	GO:1901987	regulation of cell cycle phase transition	16/607	401/28814	1.89	0.011678736	0.065944826	0.047749567	Apex1/Atp2b4/Brd4/Brsk1/Cdc5l/Crebbp/Gjc2/Hspa2/Larp7/Msh2/Pdpn/Plcg2/Psme1/Psme2/Ptpn6/Rcc2	16
BP	GO:0060119	inner ear receptor cell development	5/607	65/28814	3.65	0.011943443	0.06711005	0.048593287	Gabrb2/Pls1/Rac1/Sod1/Vangl2	5
BP	GO:0006835	dicarboxylic acid transport	6/607	90/28814	3.16	0.011957394	0.06711005	0.048593287	Apba1/Gfap/Grm1/P2rx7/Slc1a3/Slc25a18	6
BP	GO:0010827	regulation of glucose transmembrane transport	6/607	90/28814	3.16	0.011957394	0.06711005	0.048593287	Enpp1/Grb10/Hk2/Osbpl8/Prkcb/Sorbs1	6
BP	GO:1901379	regulation of potassium ion transmembrane transport	6/607	90/28814	3.16	0.011957394	0.06711005	0.048593287	Actn2/Dnm2/Flna/Kcnab3/Kcnc1/Kcnip2	6
BP	GO:0070661	leukocyte proliferation	15/607	368/28814	1.93	0.012002329	0.06711005	0.048593287	Bap1/Bax/Cd151/Clu/Dnaja3/Efnb1/Hmgb1/Jak3/Mapk3/Mef2c/Msn/P2rx7/Ptpn6/Rac2/Traf6	15
BP	GO:0006654	phosphatidic acid biosynthetic process	3/607	23/28814	6.19	0.01203963	0.06711005	0.048593287	Abhd4/Dgki/Pld1	3
BP	GO:0009310	amine catabolic process	3/607	23/28814	6.19	0.01203963	0.06711005	0.048593287	Atp2b4/Comt/Slc6a3	3
BP	GO:0019400	alditol metabolic process	3/607	23/28814	6.19	0.01203963	0.06711005	0.048593287	Coq3/Pla2g4a/Tpi1	3
BP	GO:0046716	muscle cell cellular homeostasis	3/607	23/28814	6.19	0.01203963	0.06711005	0.048593287	Bag3/Lamp2/Sod1	3
BP	GO:1900120	regulation of receptor binding	3/607	23/28814	6.19	0.01203963	0.06711005	0.048593287	Anxa2/B2m/Grem2	3
BP	GO:1902992	negative regulation of amyloid precursor protein catabolic process	3/607	23/28814	6.19	0.01203963	0.06711005	0.048593287	Clu/Hap1/Picalm	3
BP	GO:0035249	synaptic transmission, glutamatergic	7/607	117/28814	2.84	0.012070449	0.067125188	0.048604248	Cdh2/Dgki/Drd1/Grik5/Grm1/Mef2c/Nlgn3	7
BP	GO:1904659	glucose transmembrane transport	7/607	117/28814	2.84	0.012070449	0.067125188	0.048604248	Drd1/Enpp1/Grb10/Hk2/Osbpl8/Prkcb/Sorbs1	7
BP	GO:0030183	B cell differentiation	9/607	175/28814	2.44	0.01219657	0.067747692	0.049054993	Bax/Enpp1/Jak3/Lgals1/Msh2/Plcg2/Ptpn6/Slc25a5/Yy1	9
BP	GO:0000082	G1/S transition of mitotic cell cycle	10/607	206/28814	2.3	0.012401166	0.068724325	0.049762157	Apex1/Brd4/Camk2b/Crebbp/Gjc2/Larp7/Pdpn/Psme1/Psme2/Ptpn6	10
BP	GO:0031334	positive regulation of protein-containing complex assembly	10/607	206/28814	2.3	0.012401166	0.068724325	0.049762157	Abca1/Bax/Clu/Cracd/Dab2ip/Lcp1/Msn/Plcg2/Rac1/Vasp	10
BP	GO:2000045	regulation of G1/S transition of mitotic cell cycle	8/607	146/28814	2.6	0.01241933	0.068745234	0.049777297	Apex1/Crebbp/Gjc2/Larp7/Pdpn/Psme1/Psme2/Ptpn6	8
BP	GO:0030099	myeloid cell differentiation	17/607	439/28814	1.84	0.012518355	0.069213173	0.050116124	B2m/Bap1/C1qc/Eif6/Enpp1/Hoph/Hmgb1/Hmgb2/Jak3/Kdm1a/Mef2c/Ptpn6/Setd1a/Slc25a5/Stat1/Stat3/Traf6	17
BP	GO:0016572	histone phosphorylation	4/607	43/28814	4.42	0.012571562	0.06921786	0.050119517	Drd1/Mapk3/Prkdc/Vrk3	4
BP	GO:0032781	positive regulation of ATP-dependent activity	4/607	43/28814	4.42	0.012571562	0.06921786	0.050119517	Dnm2/Hspa2/Msh2/Myl4	4
BP	GO:0097484	dendrite extension	4/607	43/28814	4.42	0.012571562	0.06921786	0.050119517	Picalm/Rnf157/Slc9a6/Syt17	4
BP	GO:0035023	regulation of Rho protein signal transduction	6/607	91/28814	3.13	0.012580407	0.06921786	0.050119517	Abca1/Adgrg1/Arhgef10/Arhgef2/Cdh2/Prag1	6
BP	GO:0008585	female gonad development	7/607	118/28814	2.82	0.012606142	0.06921786	0.050119517	Bax/Csmd1/Pcyt1b/Pla2g4a/Rac1/Sgpl1/Sod1	7
BP	GO:0008645	hexose transmembrane transport	7/607	118/28814	2.82	0.012606142	0.06921786	0.050119517	Drd1/Enpp1/Grb10/Hk2/Osbpl8/Prkcb/Sorbs1	7
BP	GO:0051494	negative regulation of cytoskeleton organization	9/607	176/28814	2.43	0.012622395	0.069227534	0.050126522	Arhgap6/Arhgef2/Cracd/Dbn1/Fgf13/Inpp5j/Kank4/Ssh3/Tmod1	9
BP	GO:0002720	positive regulation of cytokine production involved in immune response	5/607	66/28814	3.6	0.01270461	0.069598537	0.050395159	B2m/Dhx36/Plcg2/Sema7a/Traf6	5
BP	GO:0021953	central nervous system neuron differentiation	10/607	207/28814	2.29	0.012793957	0.07000771	0.050691435	B2m/B4galt6/Cend1/Drd1/Gdpd5/Lingo1/Nrp2/Rac1/Scn1b/Tulp3	10

MF	GO:0042277	peptide binding	12/359	374/28275	2.53	0.003205253	0.031050885	0.024724729	Apbb1/Cat/Fbxo2/Memo1/Pex13/Pex19/Ppp3ca/Ppp3r1/Ppwd1/Ranbp6/Sec61a1/Sec61a2	12
MF	GO:0005244	voltage-gated ion channel activity	8/359	192/28275	3.28	0.003280251	0.031288551	0.024913974	Cacnb3/Clcn2/Kcna1/Kcna2/Kcnab2/Kcnip3/Scn4b/Scn8a	8
MF	GO:0022832	voltage-gated channel activity	8/359	193/28275	3.26	0.003385272	0.031801042	0.025322052	Cacnb3/Clcn2/Kcna1/Kcna2/Kcnab2/Kcnip3/Scn4b/Scn8a	8
MF	GO:0015459	potassium channel regulator activity	4/359	50/28275	6.3	0.003715612	0.034383271	0.02737819	Arpp19/Dlg1/Kcnab2/Kcnip3	4
MF	GO:0019894	kinesin binding	4/359	51/28275	6.18	0.003992106	0.036398617	0.028982939	Atcay/Dlg1/Kcna2/Klc4	4
MF	GO:0005254	chloride channel activity	5/359	83/28275	4.74	0.004149581	0.036635367	0.029171455	Clcn2/Gabra3/Gabrb1/Slc17a7/Slc26a6	5
MF	GO:0000149	SNARE binding	6/359	119/28275	3.97	0.00419503	0.036635367	0.029171455	Abca1/Cplx1/Cplx3/Napa/Stx1a/Stx2	6
MF	GO:0005085	guanyl-nucleotide exchange factor activity	8/359	200/28275	3.15	0.00419534	0.036635367	0.029171455	Cyth3/Dennd1a/Iqsec3/Lamtor1/Mycbp2/Preb/Rab3ip/Rabgef1	8
MF	GO:0019888	protein phosphatase regulator activity	5/359	85/28275	4.63	0.004592917	0.039550122	0.031492372	Arpp19/Calm1/Elfn1/Ppp1r9b/Ppp3r1	5
MF	GO:0050660	flavin adenine dinucleotide binding	5/359	86/28275	4.58	0.004826763	0.040994425	0.03264242	Coq6/Cyb5r1/Dus3l/Gcdh/Ndor1	5
MF	GO:0003735	structural constituent of ribosome	7/359	164/28275	3.36	0.005108669	0.042802362	0.034082016	Mrpl27/Mrpl34/Mrpl43/Mrps5/Rpl15/Rpl18a/Rpl22l1	7
MF	GO:0005310	dicarboxylic acid transmembrane transporter activity	3/359	28/28275	8.44	0.005254991	0.042869667	0.034135609	Slc25a10/Slc25a14/Slc26a6	3
MF	GO:0140326	ATPase-coupled intramembrane lipid transporter activity	3/359	28/28275	8.44	0.005254991	0.042869667	0.034135609	Abca1/Atp8a2/Atp9a	3
MF	GO:0043021	ribonucleoprotein complex binding	7/359	171/28275	3.22	0.006387695	0.051433386	0.040954598	Dhx29/Eif1/Gcn1/Ncln/Oxa1l/Sec61a1/Sec61a2	7
MF	GO:0015368	calcium:cation antiporter activity	2/359	10/28275	15.75	0.006762958	0.053756843	0.042804685	Slc24a2/Slc8a1	2
MF	GO:0005253	anion channel activity	5/359	94/28275	4.19	0.007010285	0.054337367	0.043266936	Clcn2/Gabra3/Gabrb1/Slc17a7/Slc26a6	5
MF	GO:0008200	ion channel inhibitor activity	3/359	31/28275	7.62	0.007011273	0.054337367	0.043266936	Calm1/Camk2d/Stx1a	3
MF	GO:0030515	snoRNA binding	3/359	32/28275	7.38	0.007664795	0.058668804	0.046715907	Nop56/Nudt4/Snu13	3
MF	GO:0016634	oxidoreductase activity, acting on the CH-CH group of donors, oxygen as acceptor	2/359	11/28275	14.32	0.008196836	0.060220016	0.047951082	Acaa1a/Cpox	2
MF	GO:0016653	oxidoreductase activity, acting on NAD(P)H, heme protein as acceptor	2/359	11/28275	14.32	0.008196836	0.060220016	0.047951082	Cyb5r1/Ndor1	2
MF	GO:0019855	calcium channel inhibitor activity	2/359	11/28275	14.32	0.008196836	0.060220016	0.047951082	Calm1/Stx1a	2
MF	GO:0016248	channel inhibitor activity	3/359	33/28275	7.16	0.008353099	0.060220016	0.047951082	Calm1/Camk2d/Stx1a	3
MF	GO:0030507	spectrin binding	3/359	33/28275	7.16	0.008353099	0.060220016	0.047951082	Add2/Ank3/Epb41l2	3
MF	GO:0051117	ATPase binding	5/359	100/28275	3.94	0.009044533	0.063986475	0.050950182	Abca1/Pex19/Ppp3ca/S100a1/Snu13	5
MF	GO:0008253	5'-nucleotidase activity	2/359	12/28275	13.13	0.009754179	0.063986475	0.050950182	Nt5dc3/Nt5m	2
MF	GO:0030492	hemoglobin binding	2/359	12/28275	13.13	0.009754179	0.063986475	0.050950182	Hbb-b1/Slc4a1	2
MF	GO:0031386	protein tag	2/359	12/28275	13.13	0.009754179	0.063986475	0.050950182	Sumo2/Urm1	2
MF	GO:0031402	sodium ion binding	2/359	12/28275	13.13	0.009754179	0.063986475	0.050950182	Atp1a1/Scn8a	2
MF	GO:0031432	titin binding	2/359	12/28275	13.13	0.009754179	0.063986475	0.050950182	Calm1/Camk2d	2
MF	GO:0032036	myosin heavy chain binding	2/359	12/28275	13.13	0.009754179	0.063986475	0.050950182	Myl12b/Stx1a	2
MF	GO:0045503	dynein light chain binding	2/359	12/28275	13.13	0.009754179	0.063986475	0.050950182	Dync2i2/Glul	2
MF	GO:0019208	phosphatase regulator activity	5/359	102/28275	3.86	0.009804379	0.063986475	0.050950182	Arpp19/Calm1/Elfn1/Ppp1r9b/Ppp3r1	5
MF	GO:0017166	vinculin binding	2/359	13/28275	12.12	0.01143164	0.071592087	0.057006263	Pxn/Sorbs3	2
MF	GO:0022851	GABA-gated chloride ion channel activity	2/359	13/28275	12.12	0.01143164	0.071592087	0.057006263	Gabra3/Gabrb1	2
MF	GO:0035256	G protein-coupled glutamate receptor binding	2/359	13/28275	12.12	0.01143164	0.071592087	0.057006263	Calm1/Homer2	2
MF	GO:1990226	histone methyltransferase binding	2/359	13/28275	12.12	0.01143164	0.071592087	0.057006263	Cbx3/Nop56	2
MF	GO:0035591	signaling adaptor activity	4/359	70/28275	4.5	0.012145601	0.075302724	0.059960913	Homer2/Pxn/Sorbs1/Stat3	4
MF	GO:0030159	signaling receptor complex adaptor activity	3/359	38/28275	6.22	0.012328838	0.075681976	0.060262898	Homer2/Pxn/Sorbs1	3
MF	GO:0015291	secondary active transmembrane transporter activity	8/359	242/28275	2.6	0.012574739	0.07643469	0.060862257	Slc12a2/Slc17a7/Slc24a2/Slc25a10/Slc25a14/Slc26a6/Slc4a1/Slc8a1	8
MF	GO:0008252	nucleotidase activity	2/359	14/28275	11.25	0.013225934	0.078095988	0.062185091	Nt5dc3/Nt5m	2
MF	GO:0044548	S100 protein binding	2/359	14/28275	11.25	0.013225934	0.078095988	0.062185091	Atp2a2/S100a1	2
MF	GO:0047372	acylglycerol lipase activity	2/359	14/28275	11.25	0.013225934	0.078095988	0.062185091	Abhd3/Abhd6	2
MF	GO:0004864	protein phosphatase inhibitor activity	3/359	40/28275	5.91	0.014173804	0.082128583	0.065396104	Arpp19/Elfn1/Ppp1r9b	3
MF	GO:0071949	FAD binding	3/359	40/28275	5.91	0.014173804	0.082128583	0.065396104	Coq6/Cyb5r1/Ndor1	3
MF	GO:0005543	phospholipid binding	12/359	459/28275	2.06	0.015057175	0.085299828	0.067921255	Abca1/Aida/Cadps/Cpne8/Cyth3/Dennd1a/Epdr1/Osbpl10/Pcyt1b/Plcb1/Pxk/Snx6	12
MF	GO:0015556	C4-dicarboxylate transmembrane transporter activity	2/359	15/28275	10.5	0.01513384	0.085299828	0.067921255	Slc25a10/Slc25a14	2
MF	GO:0032977	membrane insertase activity	2/359	15/28275	10.5	0.01513384	0.085299828	0.067921255	Emc7/Oxa1l	2
MF	GO:0017022	myosin binding	4/359	76/28275	4.15	0.016024438	0.089505869	0.071270378	Myl12b/Rab11b/Rab25/Stx1a	4
MF	GO:0015562	efflux transmembrane transporter activity	2/359	16/28275	9.85	0.017152203	0.094109431	0.074936032	Slc26a6/Xpr1	2
MF	GO:0043024	ribosomal small subunit binding	2/359	16/28275	9.85	0.017152203	0.094109431	0.074936032	Dhx29/Eif1	2
MF	GO:0019905	syntaxin binding	4/359	79/28275	3.99	0.018223829	0.098710435	0.07859965	Abca1/Cplx1/Cplx3/Napa	4
MF	GO:0019212	phosphatase inhibitor activity	3/359	44/28275	5.37	0.018309194	0.098710435	0.07859965	Arpp19/Elfn1/Ppp1r9b	3

BP	GO:1904951	positive regulation of establishment of protein localization	14/607	337/28814	1.97	0.012824641	0.070095319	0.050754871	Adcy8/Bag3/Camk1/Dynll1/Flna/Frmd4a/Kcnb1/Mcu/P2rx7/Pcm1/Ptpn5/Rab34/Rac1/Sar1b	14
BP	GO:0046879	hormone secretion	15/607	372/28814	1.91	0.013131479	0.071690372	0.051909823	Adcy8/Cplx1/Dynll1/Ensa/Ildr2/Kcnb1/Mcu/Niban2/Nucb2/Rab8b/Rac1/Slc18a2/Smpd3/Snap25/Snx19	15
BP	GO:0043491	protein kinase B signaling	10/607	208/28814	2.28	0.013196032	0.071878501	0.052046044	Igfbp5/Lemd2/Lingo1/Mydgf/Ntrk3/Osbpl8/Phlda3/Rasd2/Smpd3/Tmem100	10
BP	GO:0072330	monocarboxylic acid biosynthetic process	10/607	208/28814	2.28	0.013196032	0.071878501	0.052046044	Abcd3/Acsbg1/Aldh1a1/Eif6/Hacd3/Mcat/Ndufab1/Pla2g4a/Prkag1/Ptgs1	10
BP	GO:0097581	lamellipodium organization	6/607	92/28814	3.1	0.013225848	0.071958855	0.052104227	Dnm2/Parvb/Pdpn/Rac1/Rac2/Vcl	6
BP	GO:0051091	positive regulation of DNA-binding transcription factor activity	12/607	272/28814	2.09	0.013263871	0.072083633	0.052194577	Adcy8/Arhgef2/Camk1d/Clu/Crebbp/Kdm1a/Map3k13/Plcg2/Prkcb/Stat3/Traf6/Trim25	12
BP	GO:1901616	organic hydroxy compound catabolic process	5/607	67/28814	3.54	0.013497918	0.07260595	0.052572778	Comt/Cyp4f14/Inpp5j/Slc6a3/Tpi1	5
BP	GO:1990874	vascular associated smooth muscle cell proliferation	5/607	67/28814	3.54	0.013497918	0.07260595	0.052572778	Dnmt1/Enpp1/Igfbp5/Mef2c/Pdcd4	5
BP	GO:0006606	protein import into nucleus	9/607	178/28814	2.4	0.013507093	0.07260595	0.052572778	Bag3/Drd1/Flna/Ipo7/Ipo8/Lmna/Ptpn5/Ranbp6/Stat3	9
BP	GO:0008210	estrogen metabolic process	3/607	24/28814	5.93	0.013547882	0.07260595	0.052572778	Comt/Sgpl1/Ugt1a1	3
BP	GO:0009190	cyclic nucleotide biosynthetic process	3/607	24/28814	5.93	0.013547882	0.07260595	0.052572778	Adcy3/Adcy8/Gucy1b1	3
BP	GO:0032515	negative regulation of phosphoprotein phosphatase activity	3/607	24/28814	5.93	0.013547882	0.07260595	0.052572778	Bod1/Ppp4r4/Ptpa	3
BP	GO:0032727	positive regulation of interferon-alpha production	3/607	24/28814	5.93	0.013547882	0.07260595	0.052572778	Dhx36/Hmgb1/Stat1	3
BP	GO:0034405	response to fluid shear stress	3/607	24/28814	5.93	0.013547882	0.07260595	0.052572778	Abca1/Mef2c/P2rx7	3
BP	GO:0052652	cyclic purine nucleotide metabolic process	3/607	24/28814	5.93	0.013547882	0.07260595	0.052572778	Adcy3/Adcy8/Gucy1b1	3
BP	GO:0097150	neuronal stem cell population maintenance	3/607	24/28814	5.93	0.013547882	0.07260595	0.052572778	Cdh2/Foxo1/Pcm1	3
BP	GO:1902547	regulation of cellular response to vascular endothelial growth factor stimulus	3/607	24/28814	5.93	0.013547882	0.07260595	0.052572778	Atp2b4/Dab2ip/Jcad	3
BP	GO:0046839	phospholipid dephosphorylation	4/607	44/28814	4.32	0.013603786	0.07260595	0.052572778	Inpp5j/Mtmr12/Pip4p1/Sgpp1	4
BP	GO:0098927	vesicle-mediated transport between endosomal compartments	4/607	44/28814	4.32	0.013603786	0.07260595	0.052572778	Flna/Map2k2/Mapk3/Msn	4
BP	GO:1900026	positive regulation of substrate adhesion-dependent cell spreading	4/607	44/28814	4.32	0.013603786	0.07260595	0.052572778	Dbn1/Dnm2/Flna/Rac1	4
BP	GO:1901021	positive regulation of calcium ion transmembrane transporter activity	4/607	44/28814	4.32	0.013603786	0.07260595	0.052572778	Hap1/Hspa2/Plcg2/Stim1	4
BP	GO:0030307	positive regulation of cell growth	10/607	209/28814	2.27	0.013607521	0.07260595	0.052572778	Dbn1/Dbnl/Dnph1/Map3k13/Ntrk3/Picalm/Rnf157/Sema7a/Slc25a33/Syt17	10
BP	GO:0005996	monosaccharide metabolic process	12/607	273/28814	2.09	0.013618365	0.07260595	0.052572778	Arpp19/Eno2/Enpp1/Foxo1/Ganc/Grb10/Hk2/Hmgb1/Igfbp4/Pdk2/Sorbs1/Tpi1	12
BP	GO:0042060	wound healing	15/607	374/28814	1.9	0.013726212	0.073099347	0.052930039	Ajap1/Anxa2/Bloc1s6/Cd151/Chmp5/Flna/Fundc2/Hmgb1/Naca/Pdpn/Pla2g4a/Ptk7/Ptpn6/Rab3a/Vangl2	15
BP	GO:0007346	regulation of mitotic cell cycle	18/607	479/28814	1.78	0.013748971	0.073139013	0.05295876	Apex1/Brd4/Brsk1/Cdk11b/Crebbp/Gjc2/Hmgb1/Hspa2/Larp7/Msh2/Pdpn/Psme1/Psme2/Ptpn6/Rcc2/Smpd3/Sra1/Tgfa	18
BP	GO:0042490	mechanoreceptor differentiation	6/607	93/28814	3.06	0.013894101	0.07366495	0.053339583	Gabrb2/Ntrk3/Pls1/Rac1/Sod1/Vangl2	6
BP	GO:0045104	intermediate filament cytoskeleton organization	6/607	93/28814	3.06	0.013894101	0.07366495	0.053339583	Des/Dnajb6/Gfap/Ina/Sod1/Vim	6
BP	GO:0070301	cellular response to hydrogen peroxide	6/607	93/28814	3.06	0.013894101	0.07366495	0.053339583	Apex1/Endog/Rhob/Sigmar1/Sirpa/Smpd3	6
BP	GO:0015749	monosaccharide transmembrane transport	7/607	121/28814	2.75	0.014315702	0.075358366	0.054565757	Drd1/Enpp1/Grb10/Hk2/Osbpl8/Prkcb/Sorbs1	7
BP	GO:0046545	development of primary female sexual characteristics	7/607	121/28814	2.75	0.014315702	0.075358366	0.054565757	Bax/Csmd1/Pcyt1b/Pla2g4a/Rac1/Sgpl1/Sod1	7
BP	GO:1901989	positive regulation of cell cycle phase transition	7/607	121/28814	2.75	0.014315702	0.075358366	0.054565757	Apex1/Brd4/Crebbp/Hspa2/Larp7/Plcg2/Rcc2	7
BP	GO:0019098	reproductive behavior	5/607	68/28814	3.49	0.014323926	0.075358366	0.054565757	Aplp2/Drd1/Ednrb/Hexb/Th	5
BP	GO:0019933	cAMP-mediated signaling	5/607	68/28814	3.49	0.014323926	0.075358366	0.054565757	Adcy8/Nucb2/Pde10a/Rasd2/Rnf157	5
BP	GO:0042775	mitochondrial ATP synthesis coupled electron transport	5/607	68/28814	3.49	0.014323926	0.075358366	0.054565757	Cox7c/Ndufa10/Ndufa12/Ndufb6/Sdhaf2	5
BP	GO:0061951	establishment of protein localization to plasma membrane	5/607	68/28814	3.49	0.014323926	0.075358366	0.054565757	Bbs2/Rab26/Rab31/Rab34/Vamp3	5
BP	GO:0032259	methylation	15/607	376/28814	1.89	0.014341656	0.075368638	0.054573195	Brd4/Comt/Coq3/Crebbp/Dnmt1/Dph5/Kdm1a/Larp7/Lmna/Prmt8/Setd1a/Setd3/Thada/Trmt6/Zdbf2	15
BP	GO:0010952	positive regulation of peptidase activity	9/607	180/28814	2.37	0.014436928	0.075785942	0.054875358	Bax/Dap/Dnaja3/Hmgb1/Picalm/Psme1/Psme2/Stat1/Stat3	9
BP	GO:0021954	central nervous system neuron development	6/607	94/28814	3.03	0.014585545	0.076398189	0.055318676	B4galt6/Drd1/Lingo1/Nrp2/Rac1/Scn1b	6
BP	GO:0045103	intermediate filament-based process	6/607	94/28814	3.03	0.014585545	0.076398189	0.055318676	Des/Dnajb6/Gfap/Ina/Sod1/Vim	6
BP	GO:0017158	regulation of calcium ion-dependent exocytosis	4/607	45/28814	4.22	0.014688322	0.07660056	0.055465209	Cdk5r2/Kcnb1/P2rx7/Syt17	4
BP	GO:0034198	cellular response to amino acid starvation	4/607	45/28814	4.22	0.014688322	0.07660056	0.055465209	Dap/Kptn/Mapk3/Rragb	4
BP	GO:1902742	apoptotic process involved in development	4/607	45/28814	4.22	0.014688322	0.07660056	0.055465209	Bax/Enpp1/Xkr5/Xkr9	4
BP	GO:1902991	regulation of amyloid precursor protein catabolic process	4/607	45/28814	4.22	0.014688322	0.07660056	0.055465209	Clu/Hap1/Picalm/Ranbp9	4
BP	GO:0034754	cellular hormone metabolic process	7/607	122/28814	2.72	0.014920628	0.07727199	0.05628099	Aldh1a1/Clcn2/Comt/Dkk3/Ednrb/Sgpl1/Ugt1a1	7
BP	GO:0002082	regulation of oxidative phosphorylation	3/607	25/28814	5.7	0.015158729	0.078157387	0.056592483	Cox7a1/Cox7a2/Slc25a33	3
BP	GO:0006298	mismatch repair	3/607	25/28814	5.7	0.015158729	0.078157387	0.056592483	Hmgb1/Msh2/Pcna	3
BP	GO:0015813	L-glutamate transmembrane transport	3/607	25/28814	5.7	0.015158729	0.078157387	0.056592483	Grm1/Slc1a3/Slc25a18	3
BP	GO:0090151	establishment of protein localization to mitochondrial membrane	3/607	25/28814	5.7	0.015158729	0.078157387	0.056592483	Bax/Timm13/Timm9	3
BP	GO:0101023	vascular endothelial cell proliferation	3/607	25/28814	5.7	0.015158729	0.078157387	0.056592483	Hmgb1/Mef2c/Stat3	3
BP	GO:0150105	protein localization to cell-cell junction	3/607	25/28814	5.7	0.015158729	0.078157387	0.056592483	Actb/Flna/Vcl	3
BP	GO:1902993	positive regulation of amyloid precursor protein catabolic process	3/607	25/28814	5.7	0.015158729	0.078157387	0.056592483	Clu/Picalm/Ranbp9	3
BP	GO:1905562	regulation of vascular endothelial cell proliferation	3/607	25/28814	5.7	0.015158729	0.078157387	0.056592483	Hmgb1/Mef2c/Stat3	3
BP	GO:0006879	cellular iron ion homeostasis	5/607	69/28814	3.44	0.015183181	0.078157387	0.056592483	Atp6v1g1/Ftl1/Heph/Slc6a9/Sod1	5
BP	GO:1901224	positive regulation of NIK/NF-kappaB signaling	5/607	69/28814	3.44	0.015183181	0.078157387	0.056592483	Crebbp/Hmgb1/Pdcd4/Smpd3/Traf6	5
BP	GO:1904377	positive regulation of protein localization to cell periphery	5/607	69/28814	3.44	0.015183181	0.078157387	0.056592483	Atp2b4/Epb41/Pls1/Sorbs1/Stx3	5
BP	GO:0019226	transmission of nerve impulse	6/607	95/28814	3	0.015300552	0.07867679	0.056968573	Drd1/Kcnd2/Ntrk3/P2rx7/Scn1b/Sod1	6
BP	GO:0010927	cellular component assembly involved in morphogenesis	7/607	123/28814	2.7	0.015543539	0.079840309	0.057811059	Acrbp/Actn2/Ccdc136/Flnc/Mef2c/Nebl/Tmod1	7
BP	GO:1900077	negative regulation of cellular response to insulin stimulus	4/607	46/28814	4.13	0.015825968	0.081116579	0.058735184	Enpp1/Grb10/Nucb2/Prkcb	4
BP	GO:1903115	regulation of actin filament-based movement	4/607	46/28814	4.13	0.015825968	0.081116579	0.058735184	Ctnna3/Dbn1/Fgf13/Pdpn	4
BP	GO:0003333	amino acid transmembrane transport	6/607	96/28814	2.97	0.016039488	0.081872128	0.059282265	Gfap/Grm1/Slc1a3/Slc25a18/Slc6a9/Slc7a5	6
BP	GO:0015800	acidic amino acid transport	5/607	70/28814	3.39	0.01607621	0.081872128	0.059282265	Apba1/Grm1/P2rx7/Slc1a3/Slc25a18	5
BP	GO:0042773	ATP synthesis coupled electron transport	5/607	70/28814	3.39	0.01607621	0.081872128	0.059282265	Cox7c/Ndufa10/Ndufa12/Ndufb6/Sdhaf2	5
BP	GO:0043462	regulation of ATP-dependent activity	5/607	70/28814	3.39	0.01607621	0.081872128	0.059282265	Cnn3/Dnm2/Hspa2/Msh2/Myl4	5
BP	GO:0046324	regulation of glucose import	5/607	70/28814	3.39	0.01607621	0.081872128	0.059282265	Enpp1/Grb10/Hk2/Osbpl8/Sorbs1	5
BP	GO:0048662	negative regulation of smooth muscle cell proliferation	5/607	70/28814	3.39	0.01607621	0.081872128	0.059282265	Comt/Igfbp5/Mef2c/Ndrg4/Pdcd4	5
BP	GO:0030258	lipid modification	10/607	216/28814	2.2	0.016762316	0.085023112	0.061563841	Abcd3/Dgki/Echdc1/Inpp5j/Lamtor1/Mtmr12/Nucb2/Pip4p1/Pla2g7/Sgpp1	10
BP	GO:0002718	regulation of cytokine production involved in immune response	6/607	97/28814	2.94	0.01680271	0.085023112	0.061563841	B2m/Dhx36/Jak3/Plcg2/Sema7a/Traf6	6
BP	GO:0010660	regulation of muscle cell apoptotic process	6/607	97/28814	2.94	0.01680271	0.085023112	0.061563841	Acot1/Bag3/Dlgk2a/Dnmt1/Naca/Pdcd4	6
BP	GO:0021801	cerebral cortex radial glia-guided migration	3/607	26/28814	5.48	0.016872914	0.085023112	0.061563841	Adgrg1/Cdk5r2/Dab2ip	3
BP	GO:0022030	telencephalon glial cell migration	3/607	26/28814	5.48	0.016872914	0.085023112	0.061563841	Adgrg1/Cdk5r2/Dab2ip	3
BP	GO:0030859	polarized epithelial cell differentiation	3/607	26/28814	5.48	0.016872914	0.085023112	0.061563841	Ajap1/Msn/Ptk7	3
BP	GO:0032098	regulation of appetite	3/607	26/28814	5.48	0.016872914	0.085023112	0.061563841	Bbs2/Htr4/Nucb2	3
BP	GO:0045730	respiratory burst	3/607	26/28814	5.48	0.016872914	0.085023112	0.061563841	Camk1d/Rac1/Rac2	3

BP	GO:1900151	regulation of nuclear-transcribed mRNA catabolic process, deadenylation-dependent decay	3/607	26/28814	5.48	0.016872914	0.085023112	0.061563841	Cnot7/Dhx36/Paip1	3
BP	GO:1901018	positive regulation of potassium ion transmembrane transporter activity	3/607	26/28814	5.48	0.016872914	0.085023112	0.061563841	Actn2/Dnm2/Kcnc1	3
BP	GO:0021536	diencephalon development	5/607	71/28814	3.34	0.017003526	0.085212247	0.061700791	Bax/Hap1/Kdm1a/Nrp2/Slc6a3	5
BP	GO:0030239	myofibril assembly	5/607	71/28814	3.34	0.017003526	0.085212247	0.061700791	Actn2/Flnc/Mef2c/Nebl/Tmod1	5
BP	GO:0006110	regulation of glycolytic process	4/607	47/28814	4.04	0.017017476	0.085212247	0.061700791	Eif6/P2rx7/Stat3/Zbtb20	4
BP	GO:0007215	glutamate receptor signaling pathway	4/607	47/28814	4.04	0.017017476	0.085212247	0.061700791	Frrs1l/Grm1/Homer2/Kcnb1	4
BP	GO:0010907	positive regulation of glucose metabolic process	4/607	47/28814	4.04	0.017017476	0.085212247	0.061700791	Arpp19/Foxo1/Hmgb1/Sorbs1	4
BP	GO:0070839	metal ion export	4/607	47/28814	4.04	0.017017476	0.085212247	0.061700791	Atp2b1/Kcnb1/Kcnip2/Rgs9	4
BP	GO:0048593	camera-type eye morphogenesis	8/607	155/28814	2.45	0.017249888	0.086195308	0.062412609	Aldh1a1/Bax/Bcar3/Phactr4/Prom1/Sox8/Th/Yy1	8
BP	GO:0097553	calcium ion transmembrane import into cytosol	8/607	155/28814	2.45	0.017249888	0.086195308	0.062412609	Bax/Drd1/Hap1/P2rx7/Plcg2/Ptpn6/Ryr3/Tmem38a	8
BP	GO:0048592	eye morphogenesis	9/607	186/28814	2.3	0.017509982	0.087328433	0.063233086	Aldh1a1/Bax/Bcar3/Phactr4/Prom1/Sox8/Stat3/Th/Yy1	9
BP	GO:0018958	phenol-containing compound metabolic process	7/607	126/28814	2.64	0.017522812	0.087328433	0.063233086	Comt/Ddc/Drd1/Pde1b/Pnkd/Slc6a3/Th	7
BP	GO:0003015	heart process	11/607	250/28814	2.09	0.017587496	0.087328433	0.063233086	Atp2b4/Ctnna3/Ednrb/Fgf13/Flna/Myl4/Rac1/Scn1b/Sod1/Th/Tmem38a	11
BP	GO:0070252	actin-mediated cell contraction	6/607	98/28814	2.91	0.017590571	0.087328433	0.063233086	Ctnna3/Dbn1/Fgf13/Flna/Pdpn/Scn1b	6
BP	GO:0007191	adenylate cyclase-activating dopamine receptor signaling pathway	2/607	10/28814	9.49	0.017823995	0.087328433	0.063233086	Drd1/Gnal	2
BP	GO:0015937	coenzyme A biosynthetic process	2/607	10/28814	9.49	0.017823995	0.087328433	0.063233086	Coasy/Dcakd	2
BP	GO:0019336	phenol-containing compound catabolic process	2/607	10/28814	9.49	0.017823995	0.087328433	0.063233086	Comt/Slc6a3	2
BP	GO:0021826	substrate-independent telencephalic tangential migration	2/607	10/28814	9.49	0.017823995	0.087328433	0.063233086	Drd1/Rac1	2
BP	GO:0021843	substrate-independent telencephalic tangential interneuron migration	2/607	10/28814	9.49	0.017823995	0.087328433	0.063233086	Drd1/Rac1	2
BP	GO:0021979	hypothalamus cell differentiation	2/607	10/28814	9.49	0.017823995	0.087328433	0.063233086	Hap1/Nrp2	2
BP	GO:0033572	transferrin transport	2/607	10/28814	9.49	0.017823995	0.087328433	0.063233086	Dnm2/Myo1b	2
BP	GO:0045654	positive regulation of megakaryocyte differentiation	2/607	10/28814	9.49	0.017823995	0.087328433	0.063233086	Hmgb2/Kdm1a	2
BP	GO:0061762	CAMKK-AMPK signaling cascade	2/607	10/28814	9.49	0.017823995	0.087328433	0.063233086	Camkk2/Irgm1	2
BP	GO:0071257	cellular response to electrical stimulus	2/607	10/28814	9.49	0.017823995	0.087328433	0.063233086	Actb/Grm1	2
BP	GO:0090129	positive regulation of synapse maturation	2/607	10/28814	9.49	0.017823995	0.087328433	0.063233086	Camk2b/Dab2ip	2
BP	GO:0097119	postsynaptic density protein 95 clustering	2/607	10/28814	9.49	0.017823995	0.087328433	0.063233086	Cdh2/Dbn1	2
BP	GO:0098883	synapse pruning	2/607	10/28814	9.49	0.017823995	0.087328433	0.063233086	C1qc/Ngef	2
BP	GO:0110095	cellular detoxification of aldehyde	2/607	10/28814	9.49	0.017823995	0.087328433	0.063233086	Aldh1a1/Esd	2
BP	GO:1903589	positive regulation of blood vessel endothelial cell proliferation involved in sprouting angiogenesis	2/607	10/28814	9.49	0.017823995	0.087328433	0.063233086	Jcad/Ppp1r16b	2
BP	GO:0031099	regeneration	8/607	156/28814	2.43	0.017859491	0.087412691	0.063294096	Enpp1/Flna/Gap43/Gfap/Map2k2/Naca/Ntrk3/Rtn4rl2	8
BP	GO:0055002	striated muscle cell development	5/607	72/28814	3.3	0.017965622	0.087842145	0.063605057	Actn2/Flnc/Mef2c/Nebl/Tmod1	5
BP	GO:0001933	negative regulation of protein phosphorylation	14/607	352/28814	1.89	0.018023214	0.088033633	0.06374371	Actb/Akt1s1/Bax/Dab2ip/Dnaja3/Drd1/Enpp1/Inpp5j/Ntrk3/Prkar1b/Prkar2b/Ptpn5/Ptpn6/Sirpa	14
BP	GO:0043123	positive regulation of I-kappaB kinase/NF-kappaB signaling	7/607	127/28814	2.62	0.018220275	0.088844146	0.06433059	Brd4/Dhx36/Prkcb/S100a4/Traf6/Trim25/Ube2i	7
BP	GO:0000959	mitochondrial RNA metabolic process	4/607	48/28814	3.96	0.018263544	0.088844146	0.06433059	Ears2/Mterf2/Sars2/Slc25a33	4
BP	GO:0042073	intraciliary transport	4/607	48/28814	3.96	0.018263544	0.088844146	0.06433059	Dynll1/Hspb11/Ift81/Pcm1	4
BP	GO:0043949	regulation of cAMP-mediated signaling	4/607	48/28814	3.96	0.018263544	0.088844146	0.06433059	Nucb2/Pde10a/Rasd2/Rnf157	4
BP	GO:0006672	ceramide metabolic process	6/607	99/28814	2.88	0.018403415	0.089161371	0.064560287	B4galt6/Hexb/P2rx7/Sgpl1/Smpd3/Smpd4	6
BP	GO:0032418	lysosome localization	6/607	99/28814	2.88	0.018403415	0.089161371	0.064560287	Lamtor1/Pip4p1/Rab34/Rab3a/Rac2/Tmem106b	6
BP	GO:1901992	positive regulation of mitotic cell cycle phase transition	6/607	99/28814	2.88	0.018403415	0.089161371	0.064560287	Apex1/Brd4/Crebbp/Hspa2/Larp7/Rcc2	6
BP	GO:1990849	vacuolar localization	6/607	99/28814	2.88	0.018403415	0.089161371	0.064560287	Lamtor1/Pip4p1/Rab34/Rab3a/Rac2/Tmem106b	6
BP	GO:0002687	positive regulation of leukocyte migration	8/607	157/28814	2.42	0.018484392	0.089197642	0.06458655	Camk1d/Hmgb1/Mapk3/Mcu/Pla2g7/Rac1/Rac2/Thbs4	8
BP	GO:0035637	multicellular organismal signaling	8/607	157/28814	2.42	0.018484392	0.089197642	0.06458655	Ctnna3/Drd1/Flna/Kcnd2/Ntrk3/P2rx7/Scn1b/Sod1	8
BP	GO:0051092	positive regulation of NF-kappaB transcription factor activity	8/607	157/28814	2.42	0.018484392	0.089197642	0.06458655	Arhgef2/Clu/Map3k13/Plcg2/Prkcb/Stat3/Traf6/Trim25	8
BP	GO:0001659	temperature homeostasis	9/607	188/28814	2.27	0.018633024	0.089197642	0.06458655	Ache/Acot11/Aldh1a1/Drd1/Ednrb/Grb10/Kdm1a/Lnpep/Stat3	9
BP	GO:0048659	smooth muscle cell proliferation	9/607	188/28814	2.27	0.018633024	0.089197642	0.06458655	Comt/Dnmt1/Enpp1/Igfbp5/Mef2c/Ndrg4/Pdcd4/Smpd3/Traf6	9
BP	GO:0000027	ribosomal large subunit assembly	3/607	27/28814	5.27	0.018690986	0.089197642	0.06458655	Brix1/Rpl23a/Rplp0	3
BP	GO:0002093	auditory receptor cell morphogenesis	3/607	27/28814	5.27	0.018690986	0.089197642	0.06458655	Pls1/Rac1/Sod1	3
BP	GO:0006862	nucleotide transport	3/607	27/28814	5.27	0.018690986	0.089197642	0.06458655	P2rx7/Slc25a33/Slc25a5	3
BP	GO:0009713	catechol-containing compound biosynthetic process	3/607	27/28814	5.27	0.018690986	0.089197642	0.06458655	Ddc/Slc6a3/Th	3
BP	GO:0010829	negative regulation of glucose transmembrane transport	3/607	27/28814	5.27	0.018690986	0.089197642	0.06458655	Enpp1/Grb10/Prkcb	3
BP	GO:0015740	C4-dicarboxylate transport	3/607	27/28814	5.27	0.018690986	0.089197642	0.06458655	Gfap/Slc1a3/Slc25a18	3
BP	GO:0032069	regulation of nuclease activity	3/607	27/28814	5.27	0.018690986	0.089197642	0.06458655	Hmgb1/Hmgb2/Pcna	3
BP	GO:0032528	microvillus organization	3/607	27/28814	5.27	0.018690986	0.089197642	0.06458655	Pld1/Pls1/Rap1gap	3
BP	GO:0042423	catecholamine biosynthetic process	3/607	27/28814	5.27	0.018690986	0.089197642	0.06458655	Ddc/Slc6a3/Th	3
BP	GO:0046473	phosphatidic acid metabolic process	3/607	27/28814	5.27	0.018690986	0.089197642	0.06458655	Abhd4/Dgki/Pld1	3
BP	GO:0002822	regulation of adaptive immune response based on somatic recombination of immune receptors built from immunoglobulin superfamily domains	10/607	220/28814	2.16	0.01879182	0.089500024	0.064805499	B2m/H2-K1/Hmgb1/Jak3/Mef2c/Msh2/P2rx7/Pla2g4a/Ptpn6/Traf6	10
BP	GO:1901888	regulation of cell junction assembly	10/607	220/28814	2.16	0.01879182	0.089500024	0.064805499	Arhgap6/Eef2k/Farp1/Mef2c/Nlgn3/Nptx1/Ntrk3/Rac1/Rcc2/Vcl	10
BP	GO:0071621	granulocyte chemotaxis	7/607	128/28814	2.6	0.018937012	0.09004423	0.06519955	Camk1d/Mapk3/Mcu/Rac1/Rac2/Thbs4/Vav1	7
BP	GO:0019080	viral gene expression	5/607	73/28814	3.25	0.018962976	0.09004423	0.06519955	Brd4/Larp7/Mon1b/Nucks1/Spcs1	5
BP	GO:0043112	receptor metabolic process	5/607	73/28814	3.25	0.018962976	0.09004423	0.06519955	Ache/Anxa2/Chmp5/Lamtor1/Vamp3	5
BP	GO:0034504	protein localization to nucleus	13/607	320/28814	1.93	0.018981482	0.09004423	0.06519955	Bag3/Dnajb6/Drd1/Flna/Ipo7/Ipo8/Larp7/Lmna/Osbpl8/Ptpn5/Ranbp6/Skp1/Stat3	13
BP	GO:0043409	negative regulation of MAPK cascade	9/607	189/28814	2.26	0.019213742	0.091055603	0.065931869	Dab2ip/Foxo1/Lemd2/P2rx7/Ptpn5/Ptpn6/Ranbp9/Sirpa/Vrk3	9
BP	GO:0010677	negative regulation of cellular carbohydrate metabolic process	4/607	49/28814	3.88	0.019564822	0.091989326	0.066607963	Enpp1/Grb10/Stat3/Ugt1a1	4
BP	GO:0016601	Rac protein signal transduction	4/607	49/28814	3.88	0.019564822	0.091989326	0.066607963	Dbnl/Dnm2/Rac1/Rac2	4
BP	GO:0033173	calcineurin-NFAT signaling cascade	4/607	49/28814	3.88	0.019564822	0.091989326	0.066607963	Atp2b4/Homer2/Plcg2/Rcan1	4
BP	GO:0035196	production of miRNAs involved in gene silencing by miRNA	4/607	49/28814	3.88	0.019564822	0.091989326	0.066607963	Map2k2/Pus10/Stat3/Tut4	4
BP	GO:0043029	T cell homeostasis	4/607	49/28814	3.88	0.019564822	0.091989326	0.066607963	Bax/Dnaja3/Jak3/P2rx7	4
BP	GO:0045761	regulation of adenylate cyclase activity	4/607	49/28814	3.88	0.019564822	0.091989326	0.066607963	Adcy3/Drd1/Gnal/Stim1	4
BP	GO:1901985	positive regulation of protein acetylation	4/607	49/28814	3.88	0.019564822	0.091989326	0.066607963	Camk1/Dip2a/Dip2b/Mapk3	4
BP	GO:1990928	response to amino acid starvation	4/607	49/28814	3.88	0.019564822	0.091989326	0.066607963	Dap/Kptn/Mapk3/Rragb	4
BP	GO:0014013	regulation of gliogenesis	7/607	129/28814	2.58	0.01967327	0.092317497	0.066845586	Gfap/Gjc2/Lingo1/Ntrk3/Ppp1cc/Sox8/Vim	7
BP	GO:0034219	carbohydrate transmembrane transport	7/607	129/28814	2.58	0.01967327	0.092317497	0.066845586	Drd1/Enpp1/Grb10/Hk2/Osbpl8/Prkcb/Sorbs1	7
BP	GO:0097530	granulocyte migration	8/607	159/28814	2.39	0.019780856	0.092731255	0.067145181	Camk1d/Mapk3/Mcu/Rac1/Rac2/Sirpa/Thbs4/Vav1	8
BP	GO:0051656	establishment of organelle localization	16/607	427/28814	1.78	0.019893898	0.093169757	0.067462693	Arhgef2/Bloc1s6/Chmp5/Copg1/Dipk2a/Eif6/Hap1/Myo1b/Pcm1/Picalm/Rab3a/Rac2/Sar1b/Smpd3/Stam/Wipi1	16
BP	GO:0030032	lamellipodium assembly	5/607	74/28814	3.21	0.019996046	0.093464882	0.067676389	Dnm2/Parvb/Rac1/Rac2/Vcl	5
BP	GO:0045109	intermediate filament organization	5/607	74/28814	3.21	0.019996046	0.093464882	0.067676389	Des/Dnajb6/Gfap/Ina/Vim	5
BP	GO:0045862	positive regulation of proteolysis	14/607	357/28814	1.86	0.020070921	0.09360958	0.067781162	Bax/Clu/Dab2ip/Dap/Dnaja3/Hmgb1/Picalm/Plgrkt/Psme1/Psme2/Rad23a/Rbx1/Stat1/Stat3	14
BP	GO:0002181	cytoplasmic translation	6/607	101/28814	2.82	0.020105386	0.09360958	0.067781162	Dhx36/Dph5/Paip1/Rpl35a/Rplp0/Rplp2	6

BP	GO:0032147	activation of protein kinase activity	6/607	101/28814	2.82	0.020105386	0.09360958	0.067781162	Adcy8/Camkk2/Cdk5r2/Ntrk3/Osbpl8/Traf6	6
BP	GO:0043473	pigmentation	6/607	101/28814	2.82	0.020105386	0.09360958	0.067781162	Bax/Bloc1s6/Ednrb/Enpp1/Mef2c/Sparc	6
BP	GO:0035264	multicellular organism growth	10/607	223/28814	2.13	0.020428301	0.094949472	0.068751356	Apba1/Apba2/Bbs2/Enpp1/Pls1/Sgpl1/Slc6a3/Smpd3/Sod1/Stat3	10
BP	GO:0006865	amino acid transport	8/607	160/28814	2.37	0.020452796	0.094949472	0.068751356	Apba1/Gfap/Grm1/P2rx7/Slc1a3/Slc25a18/Slc6a9/Slc7a5	8
BP	GO:2000377	regulation of reactive oxygen species metabolic process	8/607	160/28814	2.37	0.020452796	0.094949472	0.068751356	Eef1a1/Eif6/Foxo1/Hk2/Plcg2/Slc25a33/Sod1/Stat3	8
BP	GO:0021895	cerebral cortex neuron differentiation	3/607	28/28814	5.09	0.020613306	0.095231878	0.068955841	Drd1/Gdpd5/Rac1	3
BP	GO:0034390	smooth muscle cell apoptotic process	3/607	28/28814	5.09	0.020613306	0.095231878	0.068955841	Dipk2a/Dnmt1/Pdcd4	3
BP	GO:0034391	regulation of smooth muscle cell apoptotic process	3/607	28/28814	5.09	0.020613306	0.095231878	0.068955841	Dipk2a/Dnmt1/Pdcd4	3
BP	GO:0045070	positive regulation of viral genome replication	3/607	28/28814	5.09	0.020613306	0.095231878	0.068955841	Cnot7/Hacd3/Rad23a	3
BP	GO:0090103	cochlea morphogenesis	3/607	28/28814	5.09	0.020613306	0.095231878	0.068955841	Ptk7/Rac1/Vangl2	3
BP	GO:0003254	regulation of membrane depolarization	4/607	50/28814	3.8	0.020921908	0.095826488	0.069386389	B2m/Hcn3/P2rx7/Scn1b	4
BP	GO:0045806	negative regulation of endocytosis	4/607	50/28814	3.8	0.020921908	0.095826488	0.069386389	Pacsin1/Picalm/Sh3gl3/Unc119	4
BP	GO:0090102	cochlea development	4/607	50/28814	3.8	0.020921908	0.095826488	0.069386389	Gabrb2/Ptk7/Rac1/Vangl2	4
BP	GO:1902743	regulation of lamellipodium organization	4/607	50/28814	3.8	0.020921908	0.095826488	0.069386389	Dnm2/Pdpn/Rac1/Rac2	4
BP	GO:1903426	regulation of reactive oxygen species biosynthetic process	4/607	50/28814	3.8	0.020921908	0.095826488	0.069386389	Eef1a1/Plcg2/Slc25a33/Stat3	4
BP	GO:0010657	muscle cell apoptotic process	6/607	102/28814	2.79	0.020995163	0.095826488	0.069386389	Acot1/Bag3/Dipk2a/Dnmt1/Naca/Pdcd4	6
BP	GO:0043502	regulation of muscle adaptation	6/607	102/28814	2.79	0.020995163	0.095826488	0.069386389	Atp2b4/Foxo1/Igfbp5/Lmna/Mef2c/Yy1	6
BP	GO:0007422	peripheral nervous system development	5/607	75/28814	3.16	0.021065273	0.095826488	0.069386389	Arhgef10/Ednrb/Ntrk3/Sod1/Sox8	5
BP	GO:0010611	regulation of cardiac muscle hypertrophy	5/607	75/28814	3.16	0.021065273	0.095826488	0.069386389	Atp2b4/Foxo1/Lmna/Mef2c/Yy1	5
BP	GO:0042982	amyloid precursor protein metabolic process	5/607	75/28814	3.16	0.021065273	0.095826488	0.069386389	Clu/Hap1/Picalm/Ranbp9/Tmcc2	5
BP	GO:0097120	receptor localization to synapse	5/607	75/28814	3.16	0.021065273	0.095826488	0.069386389	Cplx1/Dbn1/Nptx1/Snap25/Stx3	5
BP	GO:0016482	cytosolic transport	8/607	161/28814	2.36	0.021140789	0.095826488	0.069386389	Flna/Klhl20/Map2k2/Mapk3/Msn/Rab14/Vamp3/Wipi1	8
BP	GO:0018205	peptidyl-lysine modification	15/607	395/28814	1.8	0.021308619	0.095826488	0.069386389	Actb/Brd4/Crebbp/Dip2a/Dip2b/Dnmt1/Kdm1a/Lmna/Mapk3/Naa50/Ndufab1/Rasd2/Setd1a/Setd3/Ube2i	15
BP	GO:0006171	cAMP biosynthetic process	2/607	11/28814	8.63	0.021484231	0.095826488	0.069386389	Adcy3/Adcy8	2
BP	GO:0006521	regulation of cellular amino acid metabolic process	2/607	11/28814	8.63	0.021484231	0.095826488	0.069386389	Atp2b4/Comt	2
BP	GO:0010572	positive regulation of platelet activation	2/607	11/28814	8.63	0.021484231	0.095826488	0.069386389	Pdpn/Pla2g4a	2
BP	GO:0019227	neuronal action potential propagation	2/607	11/28814	8.63	0.021484231	0.095826488	0.069386389	Ntrk3/Scn1b	2
BP	GO:0021892	cerebral cortex GABAergic interneuron differentiation	2/607	11/28814	8.63	0.021484231	0.095826488	0.069386389	Drd1/Rac1	2
BP	GO:0031053	primary miRNA processing	2/607	11/28814	8.63	0.021484231	0.095826488	0.069386389	Pus10/Stat3	2
BP	GO:0031442	positive regulation of mRNA 3'-end processing	2/607	11/28814	8.63	0.021484231	0.095826488	0.069386389	Dhx36/Ncbp2	2
BP	GO:0032342	aldosterone biosynthetic process	2/607	11/28814	8.63	0.021484231	0.095826488	0.069386389	Clcn2/Dkk3	2
BP	GO:0038128	ERBB2 signaling pathway	2/607	11/28814	8.63	0.021484231	0.095826488	0.069386389	Cpne3/Tgfa	2
BP	GO:0043568	positive regulation of insulin-like growth factor receptor signaling pathway	2/607	11/28814	8.63	0.021484231	0.095826488	0.069386389	Igfbp4/Igfbp5	2
BP	GO:0060052	neurofilament cytoskeleton organization	2/607	11/28814	8.63	0.021484231	0.095826488	0.069386389	Ina/Sod1	2
BP	GO:0060056	mammary gland involution	2/607	11/28814	8.63	0.021484231	0.095826488	0.069386389	Bax/Igfbp5	2
BP	GO:0060900	embryonic camera-type eye formation	2/607	11/28814	8.63	0.021484231	0.095826488	0.069386389	Aldh1a1/Phactr4	2
BP	GO:0061517	macrophage proliferation	2/607	11/28814	8.63	0.021484231	0.095826488	0.069386389	Clu/Mapk3	2
BP	GO:0070782	phosphatidylserine exposure on apoptotic cell surface	2/607	11/28814	8.63	0.021484231	0.095826488	0.069386389	Xkr5/Xkr9	2
BP	GO:0086100	endothelin receptor signaling pathway	2/607	11/28814	8.63	0.021484231	0.095826488	0.069386389	Bcar3/Ednrb	2
BP	GO:0090084	negative regulation of inclusion body assembly	2/607	11/28814	8.63	0.021484231	0.095826488	0.069386389	Dnajb6/Hspa2	2
BP	GO:0097104	postsynaptic membrane assembly	2/607	11/28814	8.63	0.021484231	0.095826488	0.069386389	Cdh2/Nlgn3	2
BP	GO:0098870	action potential propagation	2/607	11/28814	8.63	0.021484231	0.095826488	0.069386389	Ntrk3/Scn1b	2
BP	GO:1900454	positive regulation of long-term synaptic depression	2/607	11/28814	8.63	0.021484231	0.095826488	0.069386389	Adcy8/Kcnb1	2
BP	GO:1901339	regulation of store-operated calcium channel activity	2/607	11/28814	8.63	0.021484231	0.095826488	0.069386389	Plcg2/Stim1	2
BP	GO:1905288	vascular associated smooth muscle cell apoptotic process	2/607	11/28814	8.63	0.021484231	0.095826488	0.069386389	Dnmt1/Pdcd4	2
BP	GO:1905459	regulation of vascular associated smooth muscle cell apoptotic process	2/607	11/28814	8.63	0.021484231	0.095826488	0.069386389	Dnmt1/Pdcd4	2
BP	GO:2000009	negative regulation of protein localization to cell surface	2/607	11/28814	8.63	0.021484231	0.095826488	0.069386389	Actn2/Picalm	2
BP	GO:0120254	olefinic compound metabolic process	8/607	162/28814	2.34	0.021845018	0.097344825	0.070485791	Aldh1a1/Clcn2/Cyp4f14/Dhrs13/Dkk3/Ednrb/Pla2g4a/Ptgs1	8
BP	GO:0030593	neutrophil chemotaxis	6/607	103/28814	2.77	0.02191122	0.097548834	0.070633511	Camk1d/Mcu/Rac1/Rac2/Thbs4/Vav1	6
BP	GO:0033002	muscle cell proliferation	11/607	259/28814	2.02	0.02216523	0.098522086	0.071338227	Comt/Dipk2a/Dnmt1/Enpp1/Igfbp5/Mef2c/Ndrg4/Pdcd4/Smpd3/Stat3/Traf6	11
BP	GO:0006626	protein targeting to mitochondrion	5/607	76/28814	3.12	0.022171079	0.098522086	0.071338227	Bag3/Ptpn5/Timm13/Timm8a1/Timm9	5
BP	GO:0000737	DNA catabolic process, endonucleolytic	3/607	29/28814	4.91	0.022640059	0.099404008	0.071976812	Apex1/Bax/Endog	3
BP	GO:0010592	positive regulation of lamellipodium assembly	3/607	29/28814	4.91	0.022640059	0.099404008	0.071976812	Dnm2/Rac1/Rac2	3
BP	GO:0010971	positive regulation of G2/M transition of mitotic cell cycle	3/607	29/28814	4.91	0.022640059	0.099404008	0.071976812	Brd4/Hspa2/Rcc2	3
BP	GO:0022010	central nervous system myelination	3/607	29/28814	4.91	0.022640059	0.099404008	0.071976812	B4galt6/Clu/Enpp1	3
BP	GO:0032291	axon ensheathment in central nervous system	3/607	29/28814	4.91	0.022640059	0.099404008	0.071976812	B4galt6/Clu/Enpp1	3
BP	GO:0032480	negative regulation of type I interferon production	3/607	29/28814	4.91	0.022640059	0.099404008	0.071976812	Crebbp/Sirpa/Yy1	3
BP	GO:0032647	regulation of interferon-alpha production	3/607	29/28814	4.91	0.022640059	0.099404008	0.071976812	Dhx36/Hmgb1/Stat1	3
BP	GO:0033198	response to ATP	3/607	29/28814	4.91	0.022640059	0.099404008	0.071976812	Enpp1/P2rx7/Plcg2	3
BP	GO:0042133	neurotransmitter metabolic process	3/607	29/28814	4.91	0.022640059	0.099404008	0.071976812	Ache/Comt/Th	3
BP	GO:0051570	regulation of histone H3-K9 methylation	3/607	29/28814	4.91	0.022640059	0.099404008	0.071976812	Dnmt1/Kdm1a/Lmna	3
BP	GO:0099637	neurotransmitter receptor transport	3/607	29/28814	4.91	0.022640059	0.099404008	0.071976812	Cplx1/Snap25/Stx3	3
BP	GO:1903649	regulation of cytoplasmic transport	3/607	29/28814	4.91	0.022640059	0.099404008	0.071976812	Map2k2/Mapk3/Msn	3
BP	GO:1903861	positive regulation of dendrite extension	3/607	29/28814	4.91	0.022640059	0.099404008	0.071976812	Picalm/Rnf157/Syt17	3
BP	GO:0051146	striated muscle cell differentiation	13/607	328/28814	1.88	0.022684772	0.099508867	0.072052739	Actn2/Cdh2/Flnc/Igfbp5/Lmna/Mef2c/Naca/Nebl/Rcan1/Speg/Stim1/Tmod1/Yy1	13
BP	GO:0002685	regulation of leukocyte migration	10/607	227/28814	2.09	0.022768998	0.099695238	0.072187687	Camk1d/Hmgb1/Mapk3/Mcu/Msn/Pla2g7/Rac1/Rac2/Smpd3/Thbs4	10
BP	GO:0033673	negative regulation of kinase activity	10/607	227/28814	2.09	0.022768998	0.099695238	0.072187687	Actb/Akt1s1/Dab2ip/Dnaja3/Drd1/Pik3ip1/Prkar1b/Prkar2b/Ptpn5/Ptpn6	10
BP	GO:0003158	endothelium development	7/607	133/28814	2.5	0.022818304	0.099819634	0.07227776	Ednrb/Msn/Pdpn/Ppp1r16b/Rhob/Tmem100/Vcl	7
CC	GO:0098793	presynapse	48/627	493/28739	4.46	5.40508E-18	3.10251E-15	1.87755E-15	Ache/Actb/Adcy8/Apba1/Apba2/Atp2b1/Brsk1/Cdh2/Cplx1/Ddc/Dgki/Drd1/Gnb5/Grik5/Grm1/Gucy1b1/Hap1/Hcn3/Lgi3/Marcks/Mme/Nrxn3/P2rx7/Pacsin1/Pdyn/Picalm/Prkcb/Prrt1/Ptpn5/Rab26/Rab3a/Rab3c/Rab8b/Septin6/Sh3gl3/Slc10a4/Slc18a2/Slc18a3/Slc6a3/Slc9a6/Snap25/Sncg/Stx3/Sv2c/Syap1/Syngr1/Th/Vdac3	48
CC	GO:0031252	cell leading edge	42/627	412/28739	4.67	1.3363E-16	3.83519E-14	2.32095E-14	Actb/Aif1l/Anxa2/Arhgef2/Arpc3/Atp2b1/Cdh2/Ctnna3/Dbn1/Dbnl/Dnm2/Eef1a1/Frmd4b/Gabra3/Gabra4/Inpp5j/Jcad/Kcnb1/Kcnc1/Kptn/Lcp1/Pacsin1/Parvb/Pdlim4/Pdpn/Phactr4/Pip5k1a/Plcg2/Pld1/Plekhg5/Rab34/Rac1/Rac2/S100a11/S100a6/Shisa7/Shisa9/Snap25/Sra1/Stx3/Vasp/Vim	42

CC	GO:0097060	synaptic membrane	38/627	390/28739	4.47	1.66977E-14	3.19483E-12	1.93342E-12	Ache/Adcy8/Apba1/Atp2b1/Cdh2/Comt/Dbn1/Dbnl/Dgki/Dnaja3/Dnm2/Drd1/Eno2/Gabra3/Gabra4/Gabrb2/Gabrd/Grik5/Grm1/Kcnb1/Kcnd2/Nlgn3/Nrp2/Nrxn3/Olfm2/Pdlim4/Picalm/Prrt1/Ptpn5/Rgs9/Shisa7/Shisa9/Sigmar1/Slc6a3/Snap25/Strn/Stx3/Syap1	38
CC	GO:0044309	neuron spine	27/627	203/28739	6.1	6.74547E-14	9.67975E-12	5.85791E-12	Apba1/Apba2/Asic1/Atp2b1/Cnn3/Comt/Dbn1/Dgki/Dip2a/Drd1/Eef2k/Fbxo2/Grm1/Itpka/Kcnd2/Marcks/Nrgn/Pdlim4/Ppp1cc/Prkar2b/Rac1/Sez6/Shisa7/Shisa9/Slc1a3/Slc9a6/Strn	27
CC	GO:0043197	dendritic spine	26/627	198/28739	6.02	2.66742E-13	3.0622E-11	1.85316E-11	Apba1/Apba2/Asic1/Atp2b1/Cnn3/Comt/Dbn1/Dgki/Dip2a/Drd1/Eef2k/Fbxo2/Grm1/Itpka/Kcnd2/Marcks/Nrgn/Pdlim4/Ppp1cc/Prkar2b/Rac1/Sez6/Shisa7/Shisa9/Slc1a3/Strn	26
CC	GO:0150034	distal axon	35/627	375/28739	4.28	6.51042E-13	6.2283E-11	3.76919E-11	Actb/Arpc3/Brsk1/Cdk5r2/Clu/Cplx1/Dbn1/Dgki/Dnm2/Drd1/Eno2/Fgf13/Flna/Grik5/Hap1/Hcn3/Inpp5j/Klc1/Marcks/P2rx7/Pacsin1/Pdyn/Prkcb/Rab3a/Septin6/Slc18a2/Slc18a3/Slc6a3/Slc9a6/Snap25/Sncg/Stim1/Stx3/Syap1/Th	35
CC	GO:0008021	synaptic vesicle	25/627	205/28739	5.59	4.09813E-12	3.36047E-10	2.03366E-10	Apba1/Apba2/Atp2b1/Brsk1/Ddc/Dgki/Lgi3/Mme/Picalm/Prrt1/Ptpn5/Rab26/Rab3a/Rab3c/Rab8b/Septin6/Slc10a4/Slc18a2/Slc18a3/Snap25/Stx3/Sv2c/Syngr1/Th/Vdac3	25
CC	GO:0070382	exocytic vesicle	26/627	228/28739	5.23	7.17533E-12	5.1483E-10	3.1156E-10	Apba1/Apba2/Atp2b1/Brsk1/Ddc/Dgki/Lgi3/Mme/Picalm/Prrt1/Ptpn5/Rab26/Rab3a/Rab3c/Rab8b/Septin6/Slc10a4/Slc18a2/Slc18a3/Snap25/Stx3/Sv2c/Syngr1/Syt17/Th/Vdac3	26
CC	GO:0005884	actin filament	20/627	133/28739	6.89	1.22053E-11	7.78429E-10	4.71083E-10	Actb/Aif1l/Arpc3/Dbn1/Dbnl/Dnaja3/Flna/Gjb6/Gng12/Kptn/Lcp1/Myo1b/Pdlim4/Pls1/Rac1/Rac2/Tmod1/Tpm4/Vcl/Wipf3	20
CC	GO:0030133	transport vesicle	30/627	316/28739	4.35	1.92951E-11	1.10754E-09	6.70252E-10	Apba1/Apba2/Atp2b1/Bloc1s6/Brsk1/Ddc/Dgki/Lgi3/Mme/Nrgn/Picalm/Prrt1/Ptpn5/Rab14/Rab26/Rab3a/Rab3c/Rab8b/Sar1b/Septin6/Slc10a4/Slc18a2/Slc18a3/Snap25/Stx3/Sv2c/Syngr1/Syt17/Th/Vdac3	30
CC	GO:0005743	mitochondrial inner membrane	36/627	460/28739	3.59	5.01154E-11	2.61511E-09	1.58259E-09	Abca8b/Abcd3/Ak2/Atp5pb/Ccdc51/Chdh/Ckmt1/Clu/Coa3/Coq3/Cox6b1/Cox7a1/Cox7a2/Cox7c/Dhrs1/Dhrs13/Endog/Mcu/Micos13/Ndufa10/Ndufa12/Ndufab1/Ndufaf3/Ndufb6/Slc1a3/Slc25a18/Slc25a33/Slc25a35/Slc25a5/Stat3/Timm10b/Timm13/Timm8a1/Timm9/Vdac3/Yme1l1	36
CC	GO:0045211	postsynaptic membrane	27/627	273/28739	4.53	7.80435E-11	3.73308E-09	2.25915E-09	Ache/Cdh2/Comt/Dbn1/Dbnl/Dnaja3/Dnm2/Drd1/Gabra3/Gabra4/Gabrb2/Gabrd/Grik5/Grm1/Kcnb1/Kcnd2/Nlgn3/Nrp2/Pdlim4/Picalm/Prrt1/Rgs9/Shisa7/Shisa9/Sigmar1/Strn/Syap1	27
CC	GO:1990351	transporter complex	32/627	393/28739	3.73	2.22134E-10	9.80805E-09	5.93555E-09	Ccdc51/Clcn2/Clic1/Gabra3/Gabra4/Gabrb2/Gabrd/Grik5/Hspa2/Kcna3/Kcnab3/Kcnb1/Kcnc1/Kcnd2/Kcnip2/Mcu/Ndufa10/Ndufa12/Ndufab1/Ndufb6/Olfm2/Ptpa/Ryr3/Scn1b/Shisa9/Snap25/Timm10b/Timm13/Timm8a1/Timm9/Ttyh1/Ttyh2	32
CC	GO:0043679	axon terminus	21/627	190/28739	5.07	1.3376E-09	5.48416E-08	3.31886E-08	Actb/Cplx1/Dgki/Drd1/Grik5/Hap1/Hcn3/Marcks/P2rx7/Pacsin1/Pdyn/Prkcb/Rab3a/Septin6/Slc18a2/Slc18a3/Slc6a3/Slc9a6/Sncg/Stx3/Th	21
CC	GO:0044306	neuron projection terminus	22/627	212/28739	4.76	1.801E-09	6.89181E-08	4.17073E-08	Actb/Cplx1/Dgki/Drd1/Grik5/Hap1/Hcn3/Marcks/Mme/P2rx7/Pacsin1/Pdyn/Prkcb/Rab3a/Septin6/Slc18a2/Slc18a3/Slc6a3/Slc9a6/Sncg/Stx3/Th	22
CC	GO:0030027	lamellipodium	20/627	176/28739	5.21	2.02791E-09	7.27512E-08	4.4027E-08	Actb/Arpc3/Cdh2/Ctnna3/Dbn1/Dbnl/Dnm2/Kptn/Parvb/Pdlim4/Pdpn/Phactr4/Pip5k1a/Pld1/Plekhg5/Rac1/Rac2/Snap25/Stx3/Vasp	20
CC	GO:0005635	nuclear envelope	32/627	434/28739	3.38	2.61509E-09	8.5167E-08	5.15406E-08	Ache/Anxa4/Bax/Chmp5/Clic1/Cst3/Ednrb/Grk5/Gtpbp4/Hacd3/Ipo7/Ipo8/Lemd2/Lmna/Lmnb2/Mapk3/Mcm3ap/Mrps14/Nucb2/Osbpl8/P2rx7/Pcm1/Pgrmc2/Pla2g4a/Ptgs1/Rac2/S100a6/Sephs1/Sigmar1/Smpd4/Tmem38a/Tnks	32
CC	GO:0031253	cell projection membrane	27/627	320/28739	3.87	2.67074E-09	8.5167E-08	5.15406E-08	Adcy3/Aif1l/Arhgef2/Atp2b1/Bbs2/Dnm2/Drd1/Eef1a1/Gabra3/Gabra4/Gap43/Jcad/Kcnb1/Kcnc1/Msn/Ndrg4/Pacsin1/Pdpn/Pip5k1a/Plcg2/Prkcb/Prom1/Rac1/Shisa7/Shisa9/Slc7a5/Ttyh1	27
CC	GO:0032432	actin filament bundle	15/627	97/28739	7.09	3.03981E-09	9.18341E-08	5.55754E-08	Actb/Anxa2/Bag3/Flna/Lcp1/Marcks/Myl12b/Nebl/Pdlim4/Pls1/Sorbs1/Tnrd1/Tpm4/Vangl2/Vcl	15
CC	GO:0098978	glutamatergic synapse	27/627	323/28739	3.83	3.26744E-09	9.37756E-08	5.67503E-08	Actb/Adcy8/Apba1/Arpc5l/Atp2b1/Cplx1/Dbn1/Dgki/Dlgap4/Drd1/Grm1/Homer2/Kcnd2/Ngef/Nptx1/Nrp2/Olfm2/Ppp2r2a/Prkar1b/Prkar2b/Rac1/Rgs9/Sh3gl3/Shisa7/Shisa9/Snap25/Stx3	27
CC	GO:0099572	postsynaptic specialization	28/627	352/28739	3.65	5.01943E-09	1.37198E-07	8.30282E-08	Actb/Adcy8/Arhgef2/Camk2b/Cdh2/Cnn3/Dbn1/Dbnl/Dgki/Dlgap4/Eef2k/Gabra3/Gabrb2/Gap43/Grik5/Grm1/Homer2/Kcnd2/Nlgn3/Nrgn/Picalm/Prrt1/Rgs9/Rplp0/Shisa7/Shisa9/Sigmar1/Strn	28
CC	GO:0001726	ruffle	18/627	153/28739	5.39	7.45628E-09	1.94541E-07	1.17731E-07	Aif1l/Anxa2/Arhgef2/Dbnl/Dnm2/Eef1a1/Frmd4b/Inpp5j/Jcad/Lcp1/Pacsin1/Pdpn/Pip5k1a/Plcg2/Rab34/Rac1/S100a11/S100a6	18
CC	GO:0043209	myelin sheath	21/627	215/28739	4.48	1.2399E-08	3.09435E-07	1.87261E-07	Actb/Anxa2/Atp5pb/Ckb/Ckmt1/Eef1a1/Eno2/Gfap/Gjc2/Gnb5/Hspa2/Idh3a/Ina/Msn/Ndufa10/Pacsin1/Slc25a5/Snap25/Sod1/Tagln3/Tuba1a	21
CC	GO:0045121	membrane raft	29/627	393/28739	3.38	1.43678E-08	3.4363E-07	2.07955E-07	Abca1/Actb/Adcy3/Adcy8/Anxa2/Atp2b1/Cdh2/Drd1/Ednrb/Efnb1/Eno2/Kcna3/Kcnd2/Lamp2/Lamtor1/Mapk3/Mlc1/Mme/P2rx7/Pdpn/Prkar2b/Rtn4rl2/Slc25a5/Slc6a3/Snap25/Sorbs1/Stim1/Tuba1a/Vcl	29
CC	GO:0098857	membrane microdomain	29/627	394/28739	3.37	1.51967E-08	3.48916E-07	2.11154E-07	Abca1/Actb/Adcy3/Adcy8/Anxa2/Atp2b1/Cdh2/Drd1/Ednrb/Efnb1/Eno2/Kcna3/Kcnd2/Lamp2/Lamtor1/Mapk3/Mlc1/Mme/P2rx7/Pdpn/Prkar2b/Rtn4rl2/Slc25a5/Slc6a3/Snap25/Sorbs1/Stim1/Tuba1a/Vcl	29
CC	GO:1902495	transmembrane transporter complex	28/627	372/28739	3.45	1.66807E-08	3.68258E-07	2.22859E-07	Ccdc51/Clcn2/Clic1/Gabra3/Gabra4/Gabrb2/Gabrd/Grik5/Hspa2/Kcna3/Kcnab3/Kcnb1/Kcnc1/Kcnd2/Kcnip2/Mcu/Ndufa10/Ndufa12/Ndufab1/Ndufb6/Olfm2/Ptpa/Ryr3/Scn1b/Shisa9/Snap25/Ttyh1/Ttyh2	28
CC	GO:0005938	cell cortex	25/627	305/28739	3.76	1.84464E-08	3.92157E-07	2.37322E-07	Actb/Actn2/Anxa2/Cdh2/Dbn1/Dbnl/Eef1a1/Eno2/Epb41/Flna/Map2k2/Marcks/Myl12b/Osbpl8/Pls1/Prkcb/Rac1/Rac2/Rhob/Rhof/Septin6/Stim1/Stxbp6/Tmod1/Tpm4	25

CC	GO:0034702	ion channel complex	24/627	294/28739	3.74	3.83571E-08	7.86321E-07	4.75859E-07	Ccdc51/Clcn2/Clic1/Gabra3/Gabra4/Gabrb2/Gabrd/Grik5/Hspa2/Kcna3/Kcnab3/Kcnb1/Kcnc1/Kcnd2/Kcnip2/Mcu/Olfm2/Ptpa/Ryr3/Scn1b/Shisa9/Snap25/Ttyh1/Ttyh2	24
CC	GO:0098562	cytoplasmic side of membrane	19/627	195/28739	4.47	6.34809E-08	1.25648E-06	7.60389E-07	Ajap1/Atp2b1/Farp1/Gfap/Gnal/Gnb5/Gng12/Gng4/Gramd1a/Map2k2/Msn/Prmt8/Rac1/Rgs8/S100a6/Snap25/Syap1/Th/Traf6	19
CC	GO:0009898	cytoplasmic side of plasma membrane	18/627	177/28739	4.66	7.32098E-08	1.36502E-06	8.26073E-07	Ajap1/Atp2b1/Farp1/Gnal/Gnb5/Gng12/Gng4/Gramd1a/Map2k2/Msn/Prmt8/Rac1/Rgs8/S100a6/Snap25/Syap1/Th/Traf6	18
CC	GO:0031256	leading edge membrane	17/627	158/28739	4.93	7.37207E-08	1.36502E-06	8.26073E-07	Aif1l/Arhgef2/Atp2b1/Dnm2/Eef1a1/Gabra3/Gabra4/Jcad/Kcnb1/Kcnc1/Pacsin1/Pdpn/Pip5k1a/Plcg2/Rac1/Shisa7/Shisa9	17
CC	GO:0043204	perikaryon	16/627	141/28739	5.2	8.45022E-08	1.51576E-06	9.17294E-07	Camk2b/Ckmt1/Clcn2/Dhx36/Dip2b/Endog/Eno2/Gjc2/Grik5/Kcnb1/Pde10a/Ptpn5/Rtn4rl2/Syap1/Th/Tmem100	16
CC	GO:0016323	basolateral plasma membrane	21/627	246/28739	3.91	1.27589E-07	2.21928E-06	1.34304E-06	Abca1/Abca8b/Adcy8/Ajap1/Anxa2/Atp2b1/Atp2b4/Cdh2/Cnnm4/Epb41/Heph/Mlc1/Msn/Ndrg4/Pdpn/Pianp/Slc1a3/Slc6a9/Slc7a5/Tgfa/Vangl2	21
CC	GO:0030139	endocytic vesicle	19/627	211/28739	4.13	2.20649E-07	3.72507E-06	2.25431E-06	Abca1/B2m/Dab2ip/Dnm2/Ftl1/H2-K1/Irgm1/Lamp2/Nlgn3/Pip4p1/Pld1/Plekhg5/Rab14/Rab31/Rab34/Rab8b/Slc18a3/Vamp3/Vim	19
CC	GO:0005769	early endosome	24/627	327/28739	3.36	2.78539E-07	4.56804E-06	2.76445E-06	Anxa2/H2-K1/Hmgb1/Map2k2/Mapk3/Mlc1/Mme/Myo1b/Nsg2/Pdlim4/Picalm/Rab14/Rab31/Rab34/Rac1/Rap1gap/Rcc2/Rhob/Rnf157/Sh3gl3/Slc9a6/Snx19/Vamp3/Zfyve16	24
CC	GO:0014069	postsynaptic density	24/627	330/28739	3.33	3.28942E-07	5.2448E-06	3.174E-06	Actb/Adcy8/Arhgef2/Camk2b/Cdh2/Cnn3/Dbn1/Dbnl/Dgki/Eef2k/Gap43/Grik5/Grm1/Homer2/Kcnd2/Nrgn/Picalm/Prrt1/Rgs9/Rplp0/Shisa7/Shisa9/Sigmar1/Strn	24
CC	GO:0098798	mitochondrial protein-containing complex	22/627	286/28739	3.53	3.92922E-07	6.0956E-06	3.68888E-06	Atp5pb/Bax/Cox6b1/Cox7a2/Cox7c/Idh3a/Mcu/Micos13/Mrpl13/Mrpl19/Mrpl44/Mrps14/Ndufa10/Ndufa12/Ndufab1/Ndufb6/Pdk2/Slc25a5/Timm10b/Timm13/Timm8a1/Timm9	22
CC	GO:0098984	neuron to neuron synapse	25/627	361/28739	3.17	4.69334E-07	6.81628E-06	4.12502E-06	Actb/Adcy8/Arhgef2/Camk2b/Cdh2/Cnn3/Dbn1/Dbnl/Dgki/Eef2k/Gap43/Grik5/Grm1/Homer2/Kcnd2/Nrgn/Picalm/Prkar1b/Prrt1/Rgs9/Rplp0/Shisa7/Shisa9/Sigmar1/Strn	25
CC	GO:0001725	stress fiber	12/627	88/28739	6.25	4.75002E-07	6.81628E-06	4.12502E-06	Actb/Anxa2/Bag3/Lcp1/Myl12b/Nebl/Pdlim4/Sorbs1/Tlnrd1/Tpm4/Vangl2/Vcl	12
CC	GO:0097517	contractile actin filament bundle	12/627	88/28739	6.25	4.75002E-07	6.81628E-06	4.12502E-06	Actb/Anxa2/Bag3/Lcp1/Myl12b/Nebl/Pdlim4/Sorbs1/Tlnrd1/Tpm4/Vangl2/Vcl	12
CC	GO:0032279	asymmetric synapse	24/627	339/28739	3.25	5.34986E-07	7.48981E-06	4.53262E-06	Actb/Adcy8/Arhgef2/Camk2b/Cdh2/Cnn3/Dbn1/Dbnl/Dgki/Eef2k/Gap43/Grik5/Grm1/Homer2/Kcnd2/Nrgn/Picalm/Prrt1/Rgs9/Rplp0/Shisa7/Shisa9/Sigmar1/Strn	24
CC	GO:0009925	basal plasma membrane	21/627	276/28739	3.49	8.59025E-07	1.174E-05	7.10472E-06	Abca1/Abca8b/Adcy8/Ajap1/Anxa2/Atp2b1/Atp2b4/Cdh2/Cnnm4/Epb41/Heph/Mlc1/Msn/Ndrg4/Pdpn/Pianp/Slc1a3/Slc6a9/Slc7a5/Tgfa/Vangl2	21
CC	GO:0099699	integral component of synaptic membrane	13/627	113/28739	5.27	1.17019E-06	1.56206E-05	9.45316E-06	Adcy8/Atp2b1/Cdh2/Drd1/Gabra3/Gabrb2/Gabrd/Grik5/Kcnd2/Nlgn3/Nrp2/Shisa7/Shisa9	13
CC	GO:0042734	presynaptic membrane	14/627	132/28739	4.86	1.23474E-06	1.61078E-05	9.74796E-06	Ache/Adcy8/Apba1/Atp2b1/Cdh2/Drd1/Grik5/Grm1/Nrxn3/Picalm/Slc6a3/Snap25/Stx3/Syap1	14
CC	GO:0098791	Golgi apparatus subcompartment	22/627	307/28739	3.28	1.2934E-06	1.6498E-05	9.98412E-06	Ap4e1/Coro7/Dnm2/Elapor1/Flna/H2-K1/Klhl20/Lamp2/Mme/Myo1b/Nsg2/Nucb2/Pld1/Rab14/Rab31/Rab34/Rac1/Smpd3/Smpd4/Snap25/Syt17/Wipi1	22
CC	GO:0032809	neuronal cell body membrane	8/627	38/28739	9.65	1.34753E-06	1.68148E-05	1.01758E-05	Adcy8/Atp2b1/Dab2ip/Kcnb1/Kcnc1/Kcnd2/Rgs8/Slc6a3	8
CC	GO:0042641	actomyosin	12/627	97/28739	5.67	1.37969E-06	1.68498E-05	1.0197E-05	Actb/Anxa2/Bag3/Lcp1/Myl12b/Nebl/Pdlim4/Sorbs1/Tlnrd1/Tpm4/Vangl2/Vcl	12
CC	GO:0030175	filopodium	12/627	99/28739	5.56	1.71965E-06	2.05642E-05	1.24449E-05	Actn2/Dbn1/Fgf13/Gap43/Lcp1/Morn4/Msn/Myo1b/Pdpn/Snap25/Ttyh1/Vasp	12
CC	GO:0031965	nuclear membrane	19/627	244/28739	3.57	2.01286E-06	2.35792E-05	1.42695E-05	Anxa4/Cst3/Ednrb/Grk5/Gtpbp4/Hacd3/Lemd2/Lmna/Lmnb2/Mcm3ap/Mrps14/Nucb2/Osbpl8/P2rx7/Pcm1/Sephs1/Smpd4/Tmem38a/Tnks	19
CC	GO:0019897	extrinsic component of plasma membrane	16/627	179/28739	4.1	2.17634E-06	2.49844E-05	1.51198E-05	Anxa2/Cdh2/Dnaja3/Farp1/Gnal/Gnb5/Gng12/Gng4/Gramd1a/Olfm2/Picalm/Rac1/Rgs8/S100a6/Snap25/Syap1	16
CC	GO:0005770	late endosome	19/627	246/28739	3.54	2.27123E-06	2.55625E-05	1.54697E-05	Anxa2/Ap5m1/Chmp5/Cst3/Elapor1/Irgm1/Lamp2/Lamtor1/Map2k2/Mapk3/Mon1b/Nsg2/Pip4p1/Prkar1b/Rab14/Rab31/Rab34/Rhob/Slc9a6	19
CC	GO:0099055	integral component of postsynaptic membrane	11/627	85/28739	5.93	2.4164E-06	2.61701E-05	1.58374E-05	Cdh2/Drd1/Gabra3/Gabrb2/Gabrd/Grik5/Kcnd2/Nlgn3/Nrp2/Shisa7/Shisa9	11
CC	GO:0099634	postsynaptic specialization membrane	11/627	85/28739	5.93	2.4164E-06	2.61701E-05	1.58374E-05	Cdh2/Gabra3/Gabrb2/Grik5/Grm1/Kcnd2/Nlgn3/Prrt1/Rgs9/Shisa7/Shisa9	11
CC	GO:0044298	cell body membrane	8/627	41/28739	8.94	2.48545E-06	2.64194E-05	1.59883E-05	Adcy8/Atp2b1/Dab2ip/Kcnb1/Kcnc1/Kcnd2/Rgs8/Slc6a3	8
CC	GO:0005912	adherens junction	14/627	142/28739	4.52	2.96194E-06	3.09118E-05	1.8707E-05	Actb/Ajap1/Ccdc85c/Cdh2/Ctnna3/Frmd4a/Frmd4b/Jcad/Msn/Niban2/Pdlim4/Plekha7/Sorbs1/Vcl	14
CC	GO:0099240	intrinsic component of synaptic membrane	13/627	123/28739	4.84	3.06887E-06	3.14559E-05	1.90362E-05	Adcy8/Atp2b1/Cdh2/Drd1/Gabra3/Gabrb2/Gabrd/Grik5/Kcnd2/Nlgn3/Nrp2/Shisa7/Shisa9	13
CC	GO:0045178	basal part of cell	21/627	301/28739	3.2	3.41988E-06	3.44388E-05	2.08414E-05	Abca1/Abca8b/Adcy8/Ajap1/Anxa2/Atp2b1/Atp2b4/Cdh2/Cnnm4/Epb41/Heph/Mlc1/Msn/Ndrg4/Pdpn/Pianp/Slc1a3/Slc6a9/Slc7a5/Tgfa/Vangl2	21
CC	GO:0098936	intrinsic component of postsynaptic membrane	11/627	89/28739	5.67	3.82331E-06	3.78376E-05	2.28982E-05	Cdh2/Drd1/Gabra3/Gabrb2/Gabrd/Grik5/Kcnd2/Nlgn3/Nrp2/Shisa7/Shisa9	11
CC	GO:0030427	site of polarized growth	17/627	210/28739	3.71	4.0976E-06	3.92966E-05	2.37812E-05	Arpc3/Cdk5r2/Clu/Dbn1/Dbnl/Dnm2/Eno2/Fgf13/Flna/Hap1/Inpp5j/Klc1/Marcks/Snap25/Stim1/Stx3/Syap1	17
CC	GO:0032587	ruffle membrane	10/627	73/28739	6.28	4.10766E-06	3.92966E-05	2.37812E-05	Aif1l/Arhgef2/Dnm2/Eef1a1/Jcad/Pacsin1/Pdpn/Pip5k1a/Plcg2/Rac1	10
CC	GO:0005925	focal adhesion	15/627	169/28739	4.07	4.90551E-06	4.61601E-05	2.79348E-05	Actn2/Aif1l/Bcar3/Cpne3/Lcp1/Map2k2/Mapk3/Msn/Nexn/Parvb/Prag1/Rsu1/Sorbs1/Vasp/Vcl	15
CC	GO:0045335	phagocytic vesicle	12/627	113/28739	4.87	6.97115E-06	6.45394E-05	3.90574E-05	Abca1/B2m/H2-K1/Irgm1/Lamp2/Pip4p1/Rab14/Rab31/Rab34/Rab8b/Vamp3/Vim	12
CC	GO:0034707	chloride channel complex	8/627	48/28739	7.64	8.58276E-06	7.81985E-05	4.73235E-05	Clcn2/Clic1/Gabra3/Gabra4/Gabrb2/Gabrd/Ttyh1/Ttyh2	8
CC	GO:0030426	growth cone	16/627	203/28739	3.61	1.09556E-05	9.82583E-05	5.94631E-05	Arpc3/Cdk5r2/Clu/Dbn1/Dnm2/Eno2/Fgf13/Flna/Hap1/Inpp5j/Klc1/Marcks/Snap25/Stim1/Stx3/Syap1	16
CC	GO:0030055	cell-substrate junction	15/627	182/28739	3.78	1.20539E-05	0.000106445	6.44177E-05	Actn2/Aif1l/Bcar3/Cpne3/Lcp1/Map2k2/Mapk3/Msn/Nexn/Parvb/Prag1/Rsu1/Sorbs1/Vasp/Vcl	15

CC	GO:0098858	actin-based cell projection	17/627	229/28739	3.4	1.29114E-05	0.00011229	6.79549E-05	Actn2/Bbs2/Dbn1/Fgf13/Gap43/Homer2/Lcp1/Morn4/Msn/Myo1b/Pdpn/Pls1/Prom1/Slc7a5/Snap25/Ttyh1/Vasp	17
CC	GO:0098685	Schaffer collateral - CA1 synapse	10/627	83/28739	5.52	1.32298E-05	0.000113342	6.85911E-05	Actb/Adcy8/Apba1/Apba2/Cplx1/Dgki/Grm1/Ina/Prkar1b/Stx3	10
CC	GO:0015934	large ribosomal subunit	12/627	122/28739	4.51	1.53312E-05	0.000127538	7.71824E-05	Mrpl13/Mrpl19/Mrpl44/Ndufab1/Rpl23/Rpl23a/Rpl27a/Rpl28/Rpl35a/Rpl37/Rplp0/Rplp2	12
CC	GO:0030863	cortical cytoskeleton	12/627	122/28739	4.51	1.53312E-05	0.000127538	7.71824E-05	Actb/Actn2/Cdh2/Dbn1/Dbnl/Eef1a1/Epb41/Flna/Pls1/Prkcb/Tmod1/Tpm4	12
CC	GO:0002102	podosome	6/627	26/28739	10.58	1.67073E-05	0.000136999	8.29082E-05	Actb/Arhgef2/Dbnl/Lcp1/Tpm4/Vcl	6
CC	GO:0036464	cytoplasmic ribonucleoprotein granule	17/627	234/28739	3.33	1.70877E-05	0.000138146	8.36018E-05	Actb/Cnot7/Dcp1a/Dcps/Dhx36/Ina/Marcks/Noct/Ogfod1/Psma2/Rac1/Rpl28/Rplp0/Tia1/Trim25/Tuba1a/Tut4	17
CC	GO:0031234	extrinsic component of cytoplasmic side of plasma membrane	11/627	105/28739	4.8	1.90514E-05	0.000151882	9.19148E-05	Farp1/Gnal/Gnb5/Gng12/Gng4/Gramd1a/Rac1/Rgs8/S100a6/Snap25/Svap1	11
CC	GO:0043292	contractile fiber	17/627	237/28739	3.29	2.01358E-05	0.000158328	9.58156E-05	Actn2/Atp2b4/Bag3/Cst3/Dek/Des/Dnajb6/Flna/Mef2c/Myl12b/Myl4/Nebl/Nexn/Pdlim4/Ryr3/Tmod1/Vcl	17
CC	GO:0031300	intrinsic component of organelle membrane	22/627	370/28739	2.73	2.51025E-05	0.000194714	0.000117835	Atp2b1/B2m/Ccdc51/Coa3/Ergic1/Fundc1/Fundc2/Gramd1a/H2-K1/Hacd3/Lamp2/Lemd2/Mcu/Micos13/P2rx7/Pigt/Rab26/Rab3a/Slc10a4/Spcs1/Stim1/Vopp1	22
CC	GO:0044391	ribosomal subunit	15/627	195/28739	3.53	2.72638E-05	0.000208659	0.000126274	Mrpl13/Mrpl19/Mrpl44/Mrps14/Ndufab1/Rpl23/Rpl23a/Rpl27a/Rpl28/Rpl35a/Rpl37/Rplp0/Rplp2/Rps15a/Rps9	15
CC	GO:0005802	trans-Golgi network	17/627	246/28739	3.17	3.23872E-05	0.000244609	0.00014803	Ap4e1/Coro7/Dnm2/Elapor1/Flna/Klhl20/Lamp2/Mme/Myo1b/Nsg2/Rab14/Rab31/Rac1/Smpd4/Snap25/Syt17/Wipi1	17
CC	GO:0030018	Z disc	12/627	132/28739	4.17	3.38454E-05	0.000252302	0.000152686	Actn2/Atp2b4/Bag3/Des/Dnajb6/Flna/Myl12b/Nebl/Nexn/Pdlim4/Ryr3/Vcl	12
CC	GO:0030017	sarcomere	15/627	199/28739	3.45	3.45292E-05	0.0002541	0.000153774	Actn2/Atp2b4/Bag3/Des/Dnajb6/Flna/Mef2c/Myl12b/Myl4/Nebl/Nexn/Pdlim4/Ryr3/Tmod1/Vcl	15
CC	GO:0030135	coated vesicle	15/627	200/28739	3.44	3.65925E-05	0.000264496	0.000160066	Copg1/Dipk2a/Dnm2/Ergic1/Fcho1/Nrgn/Pacsin1/Picalm/Rab14/Rab3a/Rab8b/Sar1b/Slc18a3/Vamp3/Vangl2	15
CC	GO:0034703	cation channel complex	16/627	224/28739	3.27	3.68636E-05	0.000264496	0.000160066	Ccdc51/Grik5/Hspa2/Kcna3/Kcnab3/Kcnb1/Kcnc1/Kcnd2/Kcnip2/Mcu/Olfm2/Ptpa/Ryr3/Scn1b/Shisa9/Snap25	16
CC	GO:0035770	ribonucleoprotein granule	17/627	252/28739	3.09	4.38612E-05	0.000310819	0.000188099	Actb/Cnot7/Dcp1a/Dcps/Dhx36/Ina/Marcks/Noct/Ogfod1/Psma2/Rac1/Rpl28/Rplp0/Tia1/Trim25/Tuba1a/Tut4	17
CC	GO:0099060	integral component of postsynaptic specialization membrane	8/627	61/28739	6.01	5.22334E-05	0.000365634	0.000221271	Cdh2/Gabra3/Gabrb2/Grik5/Kcnd2/Nlgn3/Shisa7/Shisa9	8
CC	GO:0030658	transport vesicle membrane	11/627	119/28739	4.24	6.12806E-05	0.000423796	0.000256469	Atp2b1/Nrgn/Prrt1/Rab26/Rab3a/Sar1b/Slc10a4/Slc18a2/Slc18a3/Sv2c/Syngr1	11
CC	GO:0022625	cytosolic large ribosomal subunit	8/627	63/28739	5.82	6.61214E-05	0.000446514	0.000270217	Rpl23/Rpl23a/Rpl27a/Rpl28/Rpl35a/Rpl37/Rplp0/Rplp2	8
CC	GO:0098948	intrinsic component of postsynaptic specialization membrane	8/627	63/28739	5.82	6.61214E-05	0.000446514	0.000270217	Cdh2/Gabra3/Gabrb2/Grik5/Kcnd2/Nlgn3/Shisa7/Shisa9	8
CC	GO:0005840	ribosome	16/627	236/28739	3.11	6.87923E-05	0.000459148	0.000277863	Chchd1/Mrpl13/Mrpl19/Mrpl44/Mrps14/Ndufab1/Rpl23/Rpl23a/Rpl27a/Rpl28/Rpl35a/Rpl37/Rplp0/Rplp2/Rps15a/Rps9	16
CC	GO:0098800	inner mitochondrial membrane protein complex	12/627	143/28739	3.85	7.41854E-05	0.000489453	0.000296203	Atp5pb/Cox6b1/Cox7a2/Cox7c/Mcu/Micos13/Ndufa10/Ndufa12/Ndufab1/Ndufb6/Timm10b/Timm9	12
CC	GO:0019898	extrinsic component of membrane	19/627	318/28739	2.74	8.2557E-05	0.000538496	0.000325883	Anxa2/Bloc1s6/Cdh2/Dnaja3/Farp1/Gnal/Gnb5/Gng12/Gng4/Gramd1a/Inpp5j/Olfm2/Picalm/Rac1/Rgs8/S100a6/Snap25/Svap1/Wipi1	19
CC	GO:0031674	I band	12/627	145/28739	3.79	8.48288E-05	0.000547098	0.000331088	Actn2/Atp2b4/Bag3/Des/Dnajb6/Flna/Myl12b/Nebl/Nexn/Pdlim4/Ryr3/Vcl	12
CC	GO:0098981	cholinergic synapse	4/627	12/28739	15.28	9.66225E-05	0.000616237	0.000372929	Dlgap4/Pld1/Slc10a4/Slc18a3	4
CC	GO:0016528	sarcoplasm	9/627	85/28739	4.85	9.96584E-05	0.000628238	0.000380192	Camk2b/Flnc/Hk2/Manf/Mtmr12/Ryr3/Stim1/Thbs4/Tmem38a	9
CC	GO:0032590	dendrite membrane	7/627	50/28739	6.42	0.000100693	0.000628238	0.000380192	Atp2b1/Gabra3/Gabra4/Kcnb1/Kcnc1/Shisa7/Shisa9	7
CC	GO:0043198	dendritic shaft	8/627	68/28739	5.39	0.000114723	0.000708077	0.000428508	Arhgef2/Asic1/Dbn1/Drd1/Flna/Inpp5j/Prkar2b/Sez6	8
CC	GO:0030016	myofibril	15/627	224/28739	3.07	0.000131778	0.000804686	0.000486973	Actn2/Atp2b4/Bag3/Des/Dnajb6/Flna/Mef2c/Myl12b/Myl4/Nebl/Nexn/Pdlim4/Ryr3/Tmod1/Vcl	15
CC	GO:0005916	fascia adherens	4/627	13/28739	14.1	0.000137159	0.000828729	0.000501523	Cdh2/Ctnna3/Des/Vcl	4
CC	GO:0034705	potassium channel complex	9/627	89/28739	4.64	0.000142448	0.000851718	0.000515436	Ccdc51/Grik5/Kcna3/Kcnab3/Kcnb1/Kcnc1/Kcnd2/Kcnip2/Snap25	9
CC	GO:0022626	cytosolic ribosome	10/627	111/28739	4.13	0.000162632	0.000962381	0.000582405	Rpl23/Rpl23a/Rpl27a/Rpl28/Rpl35a/Rpl37/Rplp0/Rplp2/Rps15a/Rps9	10
CC	GO:0005652	nuclear lamina	4/627	14/28739	13.1	0.000188714	0.001105322	0.000668909	Eif6/Lmna/Lmnb2/Pcna	4
CC	GO:0048786	presynaptic active zone	8/627	74/28739	4.96	0.00020865	0.001209749	0.000732106	Adcy8/Apba1/Cdh2/Dgki/Gucy1b1/Rab3a/Snap25/Stx3	8
CC	GO:0016529	sarcoplasmic reticulum	8/627	76/28739	4.82	0.000251296	0.00144244	0.000872924	Camk2b/Hk2/Manf/Mtmr12/Ryr3/Stim1/Thbs4/Tmem38a	8
CC	GO:0030670	phagocytic vesicle membrane	5/627	27/28739	8.49	0.000263774	0.001499075	0.000907197	B2m/H2-K1/Lamp2/Pip4p1/Rab31	5
CC	GO:0031301	integral component of organelle membrane	19/627	350/28739	2.49	0.000282659	0.001590647	0.000962614	Atp2b1/B2m/Ccdc51/Coa3/Ergic1/Fundc1/Fundc2/H2-K1/Hacd3/Lamp2/Lemd2/Mcu/Micos13/P2rx7/Pigt/Slc10a4/Spcs1/Stim1/Vopp1	19
CC	GO:0030672	synaptic vesicle membrane	8/627	81/28739	4.53	0.000389802	0.002151405	0.001301969	Atp2b1/Prrt1/Rab26/Rab3a/Slc10a4/Slc18a2/Sv2c/Syngr1	8
CC	GO:0099501	exocytic vesicle membrane	8/627	81/28739	4.53	0.000389802	0.002151405	0.001301969	Atp2b1/Prrt1/Rab26/Rab3a/Slc10a4/Slc18a2/Sv2c/Syngr1	8
CC	GO:0030666	endocytic vesicle membrane	6/627	45/28739	6.11	0.000415984	0.002274047	0.001376188	B2m/H2-K1/Lamp2/Pip4p1/Rab31/Slc18a3	6
CC	GO:0031527	filopodium membrane	4/627	17/28739	10.78	0.000425924	0.002306417	0.001395778	Gap43/Msn/Pdpn/Ttyh1	4
CC	GO:0005746	mitochondrial respirasome	8/627	85/28739	4.31	0.000540585	0.002899959	0.001754972	Cox6b1/Cox7a1/Cox7a2/Cox7c/Ndufa10/Ndufa12/Ndufab1/Ndufb6	8
CC	GO:1902711	GABA-A receptor complex	4/627	19/28739	9.65	0.000670025	0.003561059	0.002155052	Gabra3/Gabra4/Gabrb2/Gabrd	4
CC	GO:0005921	gap junction	5/627	33/28739	6.94	0.000696203	0.003666243	0.002218705	Dbn1/Des/Gjb6/Gjc2/Panx2	5
CC	GO:0098982	GABA-ergic synapse	7/627	69/28739	4.65	0.000761548	0.003973897	0.002404889	Atp2b1/Drd1/Gabra3/Gabrb2/Gabrd/Kcnd2/Nrxn3	7
CC	GO:0043195	terminal bouton	8/627	90/28739	4.07	0.000791998	0.004095556	0.002478514	Cplx1/Grik5/P2rx7/Rab3a/Slc18a2/Slc18a3/Stx3/Th	8
CC	GO:0031594	neuromuscular junction	8/627	91/28739	4.03	0.000852044	0.004366727	0.002642618	Ache/Des/Dlgap4/Dnaja3/P2rx7/Syngr1/Thbs4/Tuba1a	8
CC	GO:0030136	clathrin-coated vesicle	9/627	114/28739	3.62	0.000899124	0.00456723	0.002763957	Dnm2/Fcho1/Nrgn/Picalm/Rab14/Rab3a/Rab8b/Slc18a3/Vamp3	9
CC	GO:0008287	protein serine/threonine phosphatase complex	6/627	52/28739	5.29	0.000914179	0.004602972	0.002785587	Ppp1cc/Ppp2r2a/Ppp4r1/Ppp4r4/Ptpa/Shoc2	6
CC	GO:0032589	neuron projection membrane	7/627	72/28739	4.46	0.000983119	0.004907047	0.002969605	Atp2b1/Gabra3/Gabra4/Kcnb1/Kcnc1/Shisa7/Shisa9	7
CC	GO:1902710	GABA receptor complex	4/627	21/28739	8.73	0.000999417	0.004945391	0.00299281	Gabra3/Gabra4/Gabrb2/Gabrd	4
CC	GO:1903293	phosphatase complex	6/627	53/28739	5.19	0.001012134	0.004965512	0.003004986	Ppp1cc/Ppp2r2a/Ppp4r1/Ppp4r4/Ptpa/Shoc2	6
CC	GO:0042383	sarcolemma	11/627	165/28739	3.06	0.001042812	0.005072662	0.00306983	Anxa2/Atp2b4/Cdh2/Des/Flnc/Kcnd2/Msn/Ryr3/Scn1b/Smpd4/Vcl	11
CC	GO:0099571	postsynaptic cytoskeleton	3/627	10/28739	13.75	0.001106059	0.00533511	0.003228656	Actb/Ina/Kptn	3
CC	GO:0070469	respirasome	8/627	95/28739	3.86	0.001129776	0.005404096	0.003270405	Cox6b1/Cox7a1/Cox7a2/Cox7c/Ndufa10/Ndufa12/Ndufab1/Ndufb6	8
CC	GO:0045177	apical part of cell	22/627	487/28739	2.07	0.001171063	0.00555529	0.003361903	Adcy8/Anxa4/Atp2b1/Cdh2/Cyp4f14/Gjb6/Homer2/Kcnb1/Mlc1/Msn/Myl12b/Myo1b/Pcm1/Pdpn/Pld1/Prom1/Rab14/Slc6a9/Slc7a5/Stx3/Vamp3/Vangl2	22
CC	GO:0001891	phagocytic cup	4/627	22/28739	8.33	0.001200586	0.005648658	0.003418407	Dnm2/Lcp1/Rab31/Rac1	4
CC	GO:0008076	voltage-gated potassium channel complex	7/627	75/28739	4.28	0.001252491	0.005844956	0.003537201	Kcna3/Kcnab3/Kcnb1/Kcnc1/Kcnd2/Kcnip2/Snap25	7

CC	GO:0016324	apical plasma membrane	18/627	369/28739	2.24	0.00137985	0.006387368	0.003865453	Adcy8/Anxa4/Atp2b1/Cdh2/Cyp4f14/Gjb6/Kcnb1/Mlc1/Msn/Pdpn/Pld1/Prom1/Rab14/Slc6a9/Slc7a5/Stx3/Vamp3/Vangl2	18
CC	GO:0010008	endosome membrane	15/627	284/28739	2.42	0.00156452	0.007184275	0.004347718	Anxa2/Chmp5/H2-K1/Lamp2/Lamtor1/Myo1b/Nsg2/Pip4p1/Rac1/Rcc2/Rhob/Slc9a6/Snx19/Wipi1/Zfyve16	15
CC	GO:0005759	mitochondrial matrix	15/627	288/28739	2.39	0.001792725	0.008166857	0.004942349	Acot11/Chchd1/Coasy/Dnaja3/Glrx2/Hspe1/Idh3a/Mrpl13/Mrpl19/Mrpl44/Mrps14/Mterf2/Ndufab1/Pdk2/Slc25a5	15
CC	GO:0060187	cell pole	3/627	12/28739	11.46	0.001962795	0.008840872	0.005350244	Msn/Rab8b/Vangl2	3
CC	GO:0098563	intrinsic component of synaptic vesicle membrane	4/627	25/28739	7.33	0.001971484	0.008840872	0.005350244	Atp2b1/Rab26/Rab3a/Slc10a4	4
CC	GO:0031985	Golgi cisterna	6/627	61/28739	4.51	0.002114288	0.009407763	0.00569331	H2-K1/Nsg2/Nucb2/Pld1/Rab34/Smpd3	6
CC	GO:0046930	pore complex	4/627	26/28739	7.05	0.002290148	0.010111883	0.006119423	Bax/Plekha7/Slc25a5/Vdac3	4
CC	GO:0030662	coated vesicle membrane	7/627	84/28739	3.82	0.002417073	0.01059084	0.006409274	Copg1/Dipk2a/Nrgn/Rab3a/Sar1b/Slc18a3/Vamp3	7
CC	GO:0032591	dendritic spine membrane	3/627	13/28739	10.58	0.002510486	0.010834727	0.006556868	Atp2b1/Shisa7/Shisa9	3
CC	GO:0044754	autolysosome	3/627	13/28739	10.58	0.002510486	0.010834727	0.006556868	Ftl1/Irgm1/Lamp2	3
CC	GO:0099056	integral component of presynaptic membrane	5/627	44/28739	5.21	0.00261681	0.011209319	0.00678356	Adcy8/Atp2b1/Cdh2/Drd1/Grik5	5
CC	GO:0005795	Golgi stack	7/627	86/28739	3.73	0.002762748	0.011746797	0.007108826	H2-K1/Nsg2/Nucb2/Pld1/Rab14/Rab34/Smpd3	7
CC	GO:0030864	cortical actin cytoskeleton	7/627	87/28739	3.69	0.002949193	0.012356472	0.007477784	Actn2/Cdh2/Dbn1/Dbnl/Eef1a1/Pls1/Prkcb	7
CC	GO:0098803	respiratory chain complex	7/627	87/28739	3.69	0.002949193	0.012356472	0.007477784	Cox6b1/Cox7a2/Cox7c/Ndufa10/Ndufa12/Ndufab1/Ndufb6	7
CC	GO:0048787	presynaptic active zone membrane	4/627	28/28739	6.55	0.003030422	0.012601718	0.0076262	Apba1/Cdh2/Snap25/Stx3	4
CC	GO:0044853	plasma membrane raft	9/627	136/28739	3.03	0.003051636	0.012601718	0.0076262	Adcy8/Cdh2/Drd1/Kcnd2/Mapk3/Mlc1/Slc6a3/Sorbs1/Stim1	9
CC	GO:0005798	Golgi-associated vesicle	7/627	89/28739	3.61	0.003350822	0.013738369	0.008314069	Copg1/Dipk2a/Nrgn/Pacsin1/Rab14/Rab8b/Slc18a3	7
CC	GO:0014704	intercalated disc	6/627	67/28739	4.1	0.003405707	0.013864369	0.00839032	Cdh2/Ctnna3/Des/Fgf13/Scn1b/Vcl	6
CC	GO:0097440	apical dendrite	4/627	29/28739	6.32	0.003455401	0.013967608	0.008452798	Clu/Enpp1/Flna/Sez6	4
CC	GO:0044291	cell-cell contact zone	7/627	90/28739	3.57	0.003566626	0.014316387	0.008663869	Ajap1/Cdh2/Ctnna3/Des/Fgf13/Scn1b/Vcl	7
CC	GO:0034399	nuclear periphery	8/627	115/28739	3.19	0.003770554	0.01501442	0.009086299	Eif6/Lmna/Lmnb2/Pcna/Scaf8/Sorbs1/Sparc/Yy1	8
CC	GO:0005758	mitochondrial intermembrane space	7/627	91/28739	3.53	0.003792841	0.01501442	0.009086299	Ak2/Sod1/Suox/Timm10b/Timm13/Timm8a1/Timm9	7
CC	GO:0031201	SNARE complex	5/627	48/28739	4.77	0.003842288	0.01510598	0.009141708	Bloc1s6/Cplx1/Snap25/Stx3/Vamp3	5
CC	GO:0030140	trans-Golgi network transport vesicle	4/627	30/28739	6.11	0.00391913	0.015298683	0.009258326	Nrgn/Rab14/Rab8b/Slc18a3	4
CC	GO:0032421	stereocilium bundle	6/627	69/28739	3.99	0.003944608	0.015298683	0.009258326	Bbs2/Homer2/Morn4/Pls1/Prom1/Rac1	6
CC	GO:0098889	intrinsic component of presynaptic membrane	5/627	51/28739	4.49	0.004997518	0.019252185	0.011650873	Adcy8/Atp2b1/Cdh2/Drd1/Grik5	5
CC	GO:0005901	caveola	7/627	96/28739	3.34	0.00509121	0.019482364	0.011790171	Adcy8/Drd1/Kcnd2/Mapk3/Mlc1/Slc6a3/Sorbs1	7
CC	GO:0005767	secondary lysosome	3/627	17/28739	8.09	0.005594211	0.021265412	0.012869221	Ftl1/Irgm1/Lamp2	3
CC	GO:0030117	membrane coat	7/627	98/28739	3.27	0.005694638	0.021364197	0.012929002	Ap4e1/Ap5m1/Copg1/Dipk2a/Picalm/Sar1b/Slc18a3	7
CC	GO:0048475	coated membrane	7/627	98/28739	3.27	0.005694638	0.021364197	0.012929002	Ap4e1/Ap5m1/Copg1/Dipk2a/Picalm/Sar1b/Slc18a3	7
CC	GO:0070160	tight junction	9/627	151/28739	2.73	0.006049687	0.022548832	0.01364591	Actb/Arhgef2/Ccdc85c/Clmp/Frmd4a/Frmd4b/Ildr2/Strn/Vasp	9
CC	GO:0044305	calyx of Held	3/627	18/28739	7.64	0.006605412	0.024461332	0.0148033	Actb/Cplx1/Prkcb	3
CC	GO:0005834	heterotrimeric G-protein complex	4/627	35/28739	5.24	0.006873315	0.025290273	0.015304951	Gnal/Gnb5/Gng12/Gng4	4
CC	GO:0031970	organelle envelope lumen	7/627	104/28739	3.09	0.007827073	0.028616178	0.017317695	Ak2/Sod1/Suox/Timm10b/Timm13/Timm8a1/Timm9	7
CC	GO:0032592	integral component of mitochondrial membrane	6/627	80/28739	3.44	0.008096991	0.02941565	0.017801512	Ccdc51/Coa3/Fundc1/Fundc2/Mcu/Micos13	6
CC	GO:0070069	cytochrome complex	4/627	37/28739	4.96	0.008378605	0.030058245	0.018190392	Cox6b1/Cox7a2/Cox7c/Uqt1a1	4
CC	GO:1905360	GTPase complex	4/627	37/28739	4.96	0.008378605	0.030058245	0.018190392	Gnal/Gnb5/Gng12/Gng4	4
CC	GO:0032420	stereocilium	5/627	58/28739	3.95	0.008611496	0.030701857	0.018579888	Bbs2/Homer2/Morn4/Pls1/Prom1	5
CC	GO:0005751	mitochondrial respiratory chain complex IV	3/627	20/28739	6.88	0.008935085	0.031658882	0.019159052	Cox6b1/Cox7a2/Cox7c	3
CC	GO:0098573	intrinsic component of mitochondrial membrane	6/627	82/28739	3.35	0.009099526	0.032043728	0.01939195	Ccdc51/Coa3/Fundc1/Fundc2/Mcu/Micos13	6
CC	GO:0043296	apical junction complex	9/627	163/28739	2.53	0.009772738	0.034204584	0.020699638	Actb/Arhgef2/Ccdc85c/Clmp/Frmd4a/Frmd4b/Plekha7/Strn/Vasp	9
CC	GO:0098839	postsynaptic density membrane	5/627	60/28739	3.82	0.009907947	0.034467646	0.020858836	Grik5/Grm1/Prrt1/Rgs9/Shisa9	5
CC	GO:0031902	late endosome membrane	6/627	84/28739	3.27	0.010187738	0.03522748	0.021318666	Anxa2/Chmp5/Lamp2/Lamtor1/Nsg2/Pip4p1	6
CC	GO:0030660	Golgi-associated vesicle membrane	4/627	40/28739	4.58	0.011016011	0.037863414	0.022913858	Copg1/Dipk2a/Nrgn/Slc18a3	4
CC	GO:0005849	mRNA cleavage factor complex	3/627	22/28739	6.25	0.011687767	0.039696912	0.024023438	Fip1l1/Pip5k1a/Scaf8	3
CC	GO:0097449	astrocyte projection	3/627	22/28739	6.25	0.011687767	0.039696912	0.024023438	Adgrg1/Gfap/Mlc1	3
CC	GO:0005776	autophagosome	6/627	87/28739	3.16	0.011988719	0.040479557	0.024497073	Chmp5/Ftl1/Hap1/Irgm1/Lamp2/Wipi1	6
CC	GO:0060076	excitatory synapse	5/627	64/28739	3.58	0.012890151	0.043268695	0.02618498	Adcy8/Dbn1/Dgki/Nlgn3/Shisa7	5
CC	GO:0000313	organellar ribosome	6/627	90/28739	3.06	0.014002758	0.046193007	0.02795469	Chchd1/Mrpl13/Mrpl19/Mrpl44/Mrps14/Ndufab1	6
CC	GO:0005761	mitochondrial ribosome	6/627	90/28739	3.06	0.014002758	0.046193007	0.02795469	Chchd1/Mrpl13/Mrpl19/Mrpl44/Mrps14/Ndufab1	6
CC	GO:0030667	secretory granule membrane	6/627	90/28739	3.06	0.014002758	0.046193007	0.02795469	Ccdc136/Lamp2/Msn/Rab26/Sparc/Stx3	6
CC	GO:0030141	secretory granule	17/627	432/28739	1.8	0.014808073	0.048570481	0.029393469	Acrbp/Ccdc136/Dynll1/Hexb/Lamp2/Msn/Pla2g4a/Rab14/Rab26/Rab3a/Sh3gl3/Slc18a2/Slc6a9/Sod1/Sparc/Stx3/Vamp3	17
CC	GO:0030665	clathrin-coated vesicle membrane	4/627	44/28739	4.17	0.015287894	0.049859383	0.030173476	Nrgn/Rab3a/Slc18a3/Vamp3	4
CC	GO:0098862	cluster of actin-based cell projections	10/627	206/28739	2.23	0.015480604	0.050202637	0.030381204	Bbs2/Flna/Homer2/Mme/Morn4/Myo1b/Pls1/Prkcb/Prom1/Rac1	10
CC	GO:0005774	vacuolar membrane	11/627	239/28739	2.11	0.016434694	0.052997273	0.032072437	Anxa2/Atp6v1g1/Chmp5/Gfap/H2-K1/Lamp2/Lamtor1/Meak7/Pip4p1/Tmem106b/Wipi1	11
CC	GO:0045277	respiratory chain complex IV	3/627	25/28739	5.5	0.016635131	0.053297942	0.032254394	Cox6b1/Cox7a2/Cox7c	3
CC	GO:0005874	microtubule	17/627	438/28739	1.78	0.01671364	0.053297942	0.032254394	Abraxas2/Arhgef2/Bicd1/Chmp5/Clmp/Dnm2/Dynll1/Fgf13/Kif21b/Klc1/Map2k2/Marcks/Rcc2/Stim1/Ttll7/Tuba1a/Tubb2a	17
CC	GO:0019867	outer membrane	10/627	209/28739	2.19	0.016954485	0.053471836	0.032359629	Bax/Fundc1/Fundc2/Hk2/Miga2/Ntrk3/Nucb2/Ppp1cc/Smpd4/Vdac3	10
CC	GO:0031968	organelle outer membrane	10/627	209/28739	2.19	0.016954485	0.053471836	0.032359629	Bax/Fundc1/Fundc2/Hk2/Miga2/Ntrk3/Nucb2/Ppp1cc/Smpd4/Vdac3	10
CC	GO:0030315	T-tubule	5/627	69/28739	3.32	0.017401448	0.054581591	0.033031221	Atp2b4/Cdh2/Kcnd2/Msn/Scn1b	5
CC	GO:0005905	clathrin-coated pit	5/627	70/28739	3.27	0.018414132	0.057419287	0.034748514	Adcy8/Dnm2/Fcho1/Picalm/Slc18a3	5
CC	GO:0097038	perinuclear endoplasmic reticulum	3/627	26/28739	5.29	0.018506216	0.057419287	0.034748514	Clu/Gdpd5/Kcnd2	3
CC	GO:0016600	flotillin complex	2/627	10/28739	9.17	0.0190418	0.058449161	0.035371765	Slc6a3/Sorbs1	2
CC	GO:0097197	tetraspanin-enriched microdomain	2/627	10/28739	9.17	0.0190418	0.058449161	0.035371765	Pdpn/Tspan9	2
CC	GO:1902911	protein kinase complex	7/627	126/28739	2.55	0.020770239	0.063415517	0.038377261	Brd4/Cdk11b/Cdk5r2/Pcna/Phka1/Phkb/Prkag1	7
CC	GO:0031305	integral component of mitochondrial inner membrane	4/627	49/28739	3.74	0.021924372	0.066306275	0.040126665	Ccdc51/Coa3/Mcu/Micos13	4
CC	GO:0005765	lysosomal membrane	9/627	187/28739	2.21	0.022063586	0.066306275	0.040126665	Anxa2/Atp6v1g1/Chmp5/Gfap/H2-K1/Lamp2/Meak7/Pip4p1/Tmem106b	9
CC	GO:0098852	lytic vacuole membrane	9/627	187/28739	2.21	0.022063586	0.066306275	0.040126665	Anxa2/Atp6v1g1/Chmp5/Gfap/H2-K1/Lamp2/Meak7/Pip4p1/Tmem106b	9
CC	GO:0031901	early endosome membrane	6/627	100/28739	2.75	0.022398233	0.066961385	0.04052312	H2-K1/Rac1/Rcc2/Slc9a6/Snx19/Zfyve16	6
CC	GO:0005775	vacuolar lumen	2/627	11/28739	8.33	0.022940811	0.067876419	0.041076872	Lamp2/Nsg2	2
CC	GO:0042824	MHC class I peptide loading complex	2/627	11/28739	8.33	0.022940811	0.067876419	0.041076872	B2m/H2-K1	2
CC	GO:0005747	mitochondrial respiratory chain complex I	4/627	50/28739	3.67	0.023431855	0.067928712	0.041108518	Ndufa10/Ndufa12/Ndufab1/Ndufb6	4
CC	GO:0030964	NADH dehydrogenase complex	4/627	50/28739	3.67	0.023431855	0.067928712	0.041108518	Ndufa10/Ndufa12/Ndufab1/Ndufb6	4
CC	GO:0031304	intrinsic component of mitochondrial inner membrane	4/627	50/28739	3.67	0.023431855	0.067928712	0.041108518	Ccdc51/Coa3/Mcu/Micos13	4
CC	GO:0045271	respiratory chain complex I	4/627	50/28739	3.67	0.023431855	0.067928712	0.041108518	Ndufa10/Ndufa12/Ndufab1/Ndufb6	4
CC	GO:0140534	endoplasmic reticulum protein-containing complex	7/627	130/28739	2.47	0.024156847	0.069678544	0.042167466	B2m/H2-K1/Pigt/Sec61b/Spcs1/Tusc3/Ugt1a1	7
CC	GO:0030137	COPI-coated vesicle	3/627	29/28739	4.74	0.024791395	0.070797317	0.042844516	Copg1/Dipk2a/Pacsin1	3
CC	GO:0098686	hippocampal mossy fiber to CA3 synapse	3/627	29/28739	4.74	0.024791395	0.070797317	0.042844516	Adcy8/Grik5/Prkar1b	3
CC	GO:0031227	intrinsic component of endoplasmic reticulum membrane	8/627	163/28739	2.25	0.027046742	0.075981959	0.045982113	B2m/Ergic1/Gramd1a/H2-K1/Hacd3/Pigt/Spcs1/Stim1	8
CC	GO:0030126	COPI vesicle coat	2/627	12/28739	7.64	0.027136414	0.075981959	0.045982113	Copg1/Dipk2a	2
CC	GO:0031143	pseudopodium	2/627	12/28739	7.64	0.027136414	0.075981959	0.045982113	Mapk3/Msn	2
CC	GO:1990635	proximal dendrite	2/627	12/28739	7.64	0.027136414	0.075981959	0.045982113	Kcnb1/Ptprr	2
CC	GO:0005902	microvillus	6/627	105/28739	2.62	0.02764951	0.077042809	0.04662411	Bbs2/Msn/Myo1b/Pdpn/Prom1/Slc7a5	6

CC	GO:0005963	magnesium-dependent protein serine/threonine phosphatase complex	3/627	31/28739	4.44	0.029541321	0.081522685	0.049335203	Ppp2r2a/Ppp4r1/Ptpa	3
CC	GO:0031045	dense core granule	3/627	31/28739	4.44	0.029541321	0.081522685	0.049335203	Slc18a2/Slc6a9/Sod1	3
CC	GO:0030120	vesicle coat	4/627	54/28739	3.4	0.030080061	0.082612224	0.04999456	Copg1/Dipk2a/Sar1b/Slc18a3	4
CC	GO:0097730	non-motile cilium	8/627	167/28739	2.2	0.030628238	0.083717185	0.050663251	Drd1/Gnb5/Prom1/Ptgs1/Rac1/Rgs9/Ttll7/Tulp3	8
CC	GO:0000137	Golgi cis cisterna	2/627	13/28739	7.05	0.031613855	0.084796042	0.051316145	Nsg2/Smpd3	2
CC	GO:0012510	trans-Golgi network transport vesicle membrane	2/627	13/28739	7.05	0.031613855	0.084796042	0.051316145	Nrgn/Slc18a3	2
CC	GO:0044327	dendritic spine head	2/627	13/28739	7.05	0.031613855	0.084796042	0.051316145	Drd1/Nrgn	2
CC	GO:0098691	dopaminergic synapse	2/627	13/28739	7.05	0.031613855	0.084796042	0.051316145	Slc10a4/Slc18a2	2
CC	GO:0005923	bicellular tight junction	7/627	138/28739	2.33	0.032028417	0.085508425	0.051747259	Arhgef2/Ccdc85c/Clmp/Frmd4a/Frmd4b/Strn/Vasp	7
CC	GO:0005811	lipid droplet	6/627	109/28739	2.52	0.032392336	0.085682953	0.051852878	Abhd4/Anxa2/Plin4/Sccpdh/Sigmar1/Traf6	6
CC	GO:1902554	serine/threonine protein kinase complex	6/627	109/28739	2.52	0.032392336	0.085682953	0.051852878	Brd4/Cdk11b/Cdk5r2/Pcna/Phka1/Phkb	6
CC	GO:0005882	intermediate filament	7/627	139/28739	2.31	0.033119642	0.08720493	0.052773935	Des/Eif6/Gfap/Ina/Lmna/Lmnb2/Vim	7
CC	GO:0000932	P-body	5/627	83/28739	2.76	0.035204139	0.09227021	0.055839298	Cnot7/Dcp1a/Dcps/Noct/Psma2	5
CC	GO:0060170	ciliary membrane	4/627	57/28739	3.22	0.03572515	0.093169635	0.056383604	Adcy3/Bbs2/Drd1/Prom1	4
CC	GO:0000164	protein phosphatase type 1 complex	2/627	14/28739	6.55	0.036358882	0.093169635	0.056383604	Ppp1cc/Shoc2	2
CC	GO:0000940	outer kinetochore	2/627	14/28739	6.55	0.036358882	0.093169635	0.056383604	Bod1/Crebbp	2
CC	GO:0005639	integral component of nuclear inner membrane	2/627	14/28739	6.55	0.036358882	0.093169635	0.056383604	Lemd2/P2rx7	2
CC	GO:0031229	intrinsic component of nuclear inner membrane	2/627	14/28739	6.55	0.036358882	0.093169635	0.056383604	Lemd2/P2rx7	2
CC	GO:0008180	COP9 signalosome	3/627	34/28739	4.04	0.03749796	0.095661463	0.057891588	Cops7b/Dynll1/Tmod1	3
CC	GO:0001917	photoreceptor inner segment	4/627	58/28739	3.16	0.037733568	0.095836585	0.057997567	Dnm2/Eno2/Gnb5/Rgs9	4
MF	GO:0003779	actin binding	44/606	444/28275	4.62	3.62241E-17	3.07905E-14	2.20395E-14	Actn2/Actr3b/Adcy8/Aif1l/Arpc1b/Arpc3/Arpc5l/Bloc1s6/Cdk5r2/Cnn3/Coro7/Ctnna3/Dbn1/Dbnl/Epb41/Flna/Flnc/Gbp2/Gjb6/Homer2/Inf2/Klhl20/Kptn/Lcp1/Marcks/Msn/Myl4/Myo1b/Nebl/Nexn/Parvb/Pdlim4/Phactr1/Phactr4/Pls1/S100a4/Setd3/Ssh3/Tlnrd1/Tmod1/Tpm4/Vasp/Vcl/Wipf3	44
MF	GO:0019903	protein phosphatase binding	26/606	185/28275	6.56	3.4465E-14	1.46476E-11	1.04846E-11	Adcy8/Atp2b4/Bod1/Cdc5l/Cdh2/Dab2ip/Drd1/Foxo1/Jak3/Lmna/Pdlim4/Phactr1/Phactr4/Ppp1cc/Ppp1r16b/Ppp2r2a/Ptpa/Shoc2/Sirpa/Slc6a3/Smg5/Sod1/Stat3/Strn/Vim/Vrk3	26
MF	GO:0019902	phosphatase binding	27/606	233/28275	5.41	1.26776E-12	3.59197E-10	2.5711E-10	Adcy8/Atp2b4/Bod1/Cdc5l/Cdh2/Dab2ip/Drd1/Foxo1/Jak3/Lmna/Mapk3/Pdlim4/Phactr1/Phactr4/Ppp1cc/Ppp1r16b/Ppp2r2a/Ptpa/Shoc2/Sirpa/Slc6a3/Smg5/Sod1/Stat3/Strn/Vim/Vrk3	27
MF	GO:0005516	calmodulin binding	23/606	192/28275	5.59	2.99817E-11	6.37111E-09	4.56038E-09	Adcy3/Adcy8/Atp2b1/Atp2b4/Camk1/Camk1d/Camk2b/Camkk2/Cnn3/Eef1a1/Eef2k/Epb41/Gap43/Itpka/Marcks/Myo1b/Nrgn/Pde1b/Phka1/Phkb/Pnck/Ryr3/Strn	23
MF	GO:0051015	actin filament binding	24/606	218/28275	5.14	6.7046E-11	1.13978E-08	8.15844E-09	Actn2/Actr3b/Aif1l/Arpc1b/Arpc3/Arpc5l/Bloc1s6/Coro7/Ctnna3/Dbn1/Dbnl/Flna/Gjb6/Kptn/Lcp1/Marcks/Myl4/Myo1b/Nebl/Nexn/Pls1/Tmod1/Tpm4/Vcl	24
MF	GO:0003924	GTPase activity	27/606	294/28275	4.28	2.79425E-10	3.95852E-08	2.83347E-08	Dnm2/Eef1a1/Gbp2/Gnal/Gng4/Gtpbp4/Irgm1/Nras/Rab14/Rab26/Rab31/Rab34/Rab3a/Rab3c/Rab8b/Rac1/Rac2/Rasd2/Rem2/Rhob/Rhof/Rragb/Rras2/Sar1b/Septin6/Tuba1a/Tubb2a	27
MF	GO:0051721	protein phosphatase 2A binding	10/606	38/28275	12.28	5.23184E-09	5.60292E-07	4.01051E-07	Adcy8/Bod1/Dab2ip/Foxo1/Ppp2r2a/Ptpa/Slc6a3/Smg5/Strn/Vim	10
MF	GO:0015267	channel activity	33/606	482/28275	3.19	5.9325E-09	5.60292E-07	4.01051E-07	Ano3/Anxa2/Asic1/Atp5pb/Bax/Ccdc51/Clcn2/Clic1/Gabra3/Gabra4/Gabrb2/Gabrd/Gjb6/Gjc2/Grik5/Hcn3/Kcna3/Kcnab3/Kcnb1/Kcnc1/Kcnd2/Kcnip2/Mcu/P2rx7/Panx2/Ryr3/Scn1b/Snap25/Stim1/Tmem38a/Ttyh1/Ttyh2/Vdac3	33
MF	GO:0022803	passive transmembrane transporter activity	33/606	482/28275	3.19	5.9325E-09	5.60292E-07	4.01051E-07	Ano3/Anxa2/Asic1/Atp5pb/Bax/Ccdc51/Clcn2/Clic1/Gabra3/Gabra4/Gabrb2/Gabrd/Gjb6/Gjc2/Grik5/Hcn3/Kcna3/Kcnab3/Kcnb1/Kcnc1/Kcnd2/Kcnip2/Mcu/P2rx7/Panx2/Ryr3/Scn1b/Snap25/Stim1/Tmem38a/Ttyh1/Ttyh2/Vdac3	33
MF	GO:0019001	guanyl nucleotide binding	28/606	382/28275	3.42	2.00907E-08	1.49349E-06	1.06902E-06	Dnm2/Eef1a1/Gbp2/Gnal/Gtpbp4/Gucy1b1/Irgm1/Nras/Pde10a/Rab14/Rab26/Rab31/Rab34/Rab3a/Rab3c/Rab8b/Rac1/Rac2/Rasd2/Rem2/Rhob/Rhof/Rragb/Rras2/Sar1b/Septin6/Tuba1a/Tubb2a	28
MF	GO:0032561	guanyl ribonucleotide binding	28/606	382/28275	3.42	2.00907E-08	1.49349E-06	1.06902E-06	Dnm2/Eef1a1/Gbp2/Gnal/Gtpbp4/Gucy1b1/Irgm1/Nras/Pde10a/Rab14/Rab26/Rab31/Rab34/Rab3a/Rab3c/Rab8b/Rac1/Rac2/Rasd2/Rem2/Rhob/Rhof/Rragb/Rras2/Sar1b/Septin6/Tuba1a/Tubb2a	28
MF	GO:0005525	GTP binding	27/606	359/28275	3.51	2.10846E-08	1.49349E-06	1.06902E-06	Dnm2/Eef1a1/Gbp2/Gnal/Gtpbp4/Gucy1b1/Irgm1/Nras/Rab14/Rab26/Rab31/Rab34/Rab3a/Rab3c/Rab8b/Rac1/Rac2/Rasd2/Rem2/Rhob/Rhof/Rragb/Rras2/Sar1b/Septin6/Tuba1a/Tubb2a	27
MF	GO:0005543	phospholipid binding	31/606	459/28275	3.15	2.35302E-08	1.53851E-06	1.10125E-06	Abca1/Actn2/Anxa2/Anxa4/Apba1/Cpne3/Dab2ip/Epb41/Fundc2/Gap43/Hmgb1/Marcks/Mme/Myo1b/Nrgn/Osbpl8/Pacsin1/Pcyt1b/Phlda3/Picalm/Pitpnc1/Pla2g4a/Pla2g7/Pld1/Smpd3/Snx19/Stxbp6/Syt17/Tulp3/Wipi1/Zfyve16	31
MF	GO:0005216	ion channel activity	29/606	438/28275	3.09	1.01998E-07	6.19276E-06	4.43272E-06	Ano3/Anxa2/Asic1/Atp5pb/Ccdc51/Clcn2/Clic1/Gabra3/Gabra4/Gabrb2/Gabrd/Grik5/Hcn3/Kcna3/Kcnab3/Kcnb1/Kcnc1/Kcnd2/Kcnip2/Mcu/P2rx7/Ryr3/Scn1b/Snap25/Stim1/Tmem38a/Ttyh1/Ttyh2/Vdac3	29
MF	GO:0022836	gated channel activity	24/606	323/28275	3.47	1.60337E-07	9.08575E-06	6.50349E-06	Asic1/Ccdc51/Clcn2/Clic1/Gabra3/Gabra4/Gabrb2/Gabrd/Grik5/Hcn3/Kcna3/Kcnab3/Kcnb1/Kcnc1/Kcnd2/Kcnip2/P2rx7/Ryr3/Scn1b/Snap25/Tmem38a/Ttyh1/Ttyh2/Vdac3	24
MF	GO:0008081	phosphoric diester hydrolase activity	12/606	86/28275	6.51	3.04236E-07	1.61625E-05	1.1569E-05	Apex1/Enpp1/Gdpd5/Gpcpd1/Pde10a/Pde1b/Pde4a/Plcd3/Plcg2/Pld1/Smpd3/Smpd4	12
MF	GO:0004683	calmodulin-dependent protein kinase activity	7/606	24/28275	13.61	5.05877E-07	2.4787E-05	1.77423E-05	Camk1/Camk1d/Camk2b/Camkk2/Eef2k/Itpka/Pnck	7
MF	GO:0046873	metal ion transmembrane transporter activity	27/606	421/28275	2.99	5.24902E-07	2.4787E-05	1.77423E-05	Anxa2/Asic1/Atp2b1/Atp2b4/Ccdc51/Cnnm4/Grik5/Hcn3/Kcna3/Kcnab3/Kcnb1/Kcnc1/Kcnd2/Kcnip2/Mcu/Ryr3/Scn1b/Slc10a4/Slc18a2/Slc1a3/Slc6a3/Slc6a9/Slc9a6/Snap25/Stim1/Tmem38a/Tusc3	27
MF	GO:0005200	structural constituent of cytoskeleton	10/606	66/28275	7.07	1.3624E-06	6.09493E-05	4.36269E-05	Actb/Arpc1b/Arpc3/Camk2b/Des/Gfap/Ina/Tuba1a/Tubb2a/Vim	10
MF	GO:0030695	GTPase regulator activity	26/606	422/28275	2.87	1.79249E-06	6.91761E-05	4.95155E-05	Arhgap20/Arhgap6/Arhgef10/Arhgef2/Bcar3/Chn1/Dab2ip/Dgki/Dnaja3/Farp1/Gnb5/Lamtor1/Ngef/Nucb2/Plekhg5/Ralgps2/Rap1gap/Rasgrp2/Rcc2/Rgs8/Rgs9/Sirpa/Srgap1/Tbc1d8/Tiam2/Vav1	26
MF	GO:0060589	nucleoside-triphosphatase regulator activity	26/606	422/28275	2.87	1.79249E-06	6.91761E-05	4.95155E-05	Arhgap20/Arhgap6/Arhgef10/Arhgef2/Bcar3/Chn1/Dab2ip/Dgki/Dnaja3/Farp1/Gnb5/Lamtor1/Ngef/Nucb2/Plekhg5/Ralgps2/Rap1gap/Rasgrp2/Rcc2/Rgs8/Rgs9/Sirpa/Srgap1/Tbc1d8/Tiam2/Vav1	26

MF	GO:0035091	phosphatidylinositol binding	20/606	270/28275	3.46	1.79483E-06	6.91761E-05	4.95155E-05	Actn2/Anxa2/Apba1/Dab2ip/Epb41/Fundc2/Gap43/Myo1b/Nrgn/Osbpl8/Phlda3/Picalm/Pitpnc1/Pla2g4a/Pld1/Snx19/Stxbp6/Tulp3/Wipi1/Zfyve16	20
MF	GO:0019208	phosphatase regulator activity	12/606	102/28275	5.49	1.96979E-06	6.91761E-05	4.95155E-05	Arpp19/Bod1/Ensa/Mtmr12/Phactr1/Phactr4/Ppp1r16b/Ppp2r2a/Ppp4r1/Ppp4r4/Ptpa/Rcan1	12
MF	GO:0051020	GTPase binding	22/606	321/28275	3.2	2.01208E-06	6.91761E-05	4.95155E-05	Abca1/Anxa2/Arhgef2/Dgki/Farp1/Flna/Inf2/Ipo7/Ipo8/Itpka/Lamtor1/Lcp1/Picalm/Rab34/Rac1/Ranbp9/Rap1gap/Rcc2/Rragb/Sod1/Srgap1/Vdl	22
MF	GO:0019888	protein phosphatase regulator activity	11/606	85/28275	6.04	2.03459E-06	6.91761E-05	4.95155E-05	Arpp19/Bod1/Ensa/Phactr1/Phactr4/Ppp1r16b/Ppp2r2a/Ppp4r1/Ppp4r4/Ptpa/Rcan1	11
MF	GO:1901981	phosphatidylinositol phosphate binding	16/606	185/28275	4.04	2.66355E-06	8.70777E-05	6.23293E-05	Actn2/Anxa2/Apba1/Dab2ip/Fundc2/Gap43/Myo1b/Nrgn/Osbpl8/Phlda3/Picalm/Pla2g4a/Snx19/Stxbp6/Wipi1/Zfyve16	16
MF	GO:0042578	phosphoric ester hydrolase activity	23/606	357/28275	3.01	3.35958E-06	0.000105765	7.57052E-05	Apex1/Enpp1/Gdpd5/Gpcpd1/Inpp5j/Mtmr12/Nudt7/Pald1/Pde10a/Pde1b/Pde4a/Pip4p1/Plcd3/Plcg2/Pld1/Ppp1cc/Ptpa/Ptpn5/Ptpn6/Sgpp1/Smpd3/Smpd4/Ssh3	23
MF	GO:0045309	protein phosphorylated amino acid binding	9/606	61/28275	6.88	5.77942E-06	0.000175447	0.000125583	Bcar3/Grb10/Mapk3/Plcg2/Ptpn5/Ptpn6/Scaf8/Sirpa/Vav1	9
MF	GO:0008157	protein phosphatase 1 binding	7/606	34/28275	9.61	6.52835E-06	0.000191348	0.000136965	Cdc5l/Lmna/Phactr1/Phactr4/Ppp1cc/Ppp1r16b/Shoc2	7
MF	GO:0061629	RNA polymerase II-specific DNA-binding transcription factor binding	23/606	377/28275	2.85	8.22518E-06	0.000233047	0.000166812	Actb/Anxa4/Apex1/Bbs2/Commd6/Crebbp/Dnaja3/Dnmt1/Grm1/Hmga1/Hmgb1/Kdm1a/Mef2c/Pcna/Prkcb/Rbx1/Setd1a/Setd3/Sra1/Stat1/Stat3/Strn/Wipi1	23
MF	GO:0003735	structural constituent of ribosome	14/606	164/28275	3.98	1.28964E-05	0.00035361	0.00025311	Mrpl13/Mrpl19/Mrpl44/Mrps14/Rpl23/Rpl23a/Rpl27a/Rpl28/Rpl35a/Rpl37/Rplp0/Rplp2/Rps15a/Rps9	14
MF	GO:0031267	small GTPase binding	19/606	285/28275	3.11	1.46298E-05	0.000388603	0.000278158	Abca1/Anxa2/Arhgef2/Dgki/Farp1/Flna/Inf2/Ipo7/Ipo8/Itpka/Picalm/Rab34/Rac1/Ranbp9/Rap1gap/Rcc2/Sod1/Srgap1/Vdl	19
MF	GO:0051219	phosphoprotein binding	11/606	110/28275	4.67	2.51088E-05	0.000646742	0.000462931	Bcar3/Dpysl4/Epb41/Grb10/Mapk3/Plcg2/Ptpn5/Ptpn6/Scaf8/Sirpa/Vav1	11
MF	GO:0015108	chloride transmembrane transporter activity	11/606	111/28275	4.62	2.73396E-05	0.000683491	0.000489236	Ano3/Clcn2/Clic1/Gabra3/Gabra4/Gabrb2/Gabrd/Slc18a2/Slc6a3/Ttyh1/Ttyh2	11
MF	GO:0005253	anion channel activity	10/606	94/28275	4.96	3.41483E-05	0.000829317	0.000593616	Ano3/Clcn2/Clic1/Gabra3/Gabra4/Gabrb2/Gabrd/Ttyh1/Ttyh2/Vdac3	10
MF	GO:0099106	ion channel regulator activity	12/606	136/28275	4.12	3.82702E-05	0.000903603	0.000646789	Arpp19/Atp2b4/Ensa/Fgf13/Flna/Kcnab3/Kcnip2/Nrxn3/Prkcb/Rem2/Scn1b/Stim1	12
MF	GO:0022804	active transmembrane transporter activity	22/606	396/28275	2.59	5.31111E-05	0.00122012	0.000873349	Abca1/Abca8b/Abcd3/Atp2b1/Atp2b4/Atp6v1g1/Cox7a1/Cox7a2/Cox7c/Ipo8/Mcu/Ndufa10/Slc10a4/Slc18a2/Slc18a3/Slc1a3/Slc25a18/Slc25a5/Slc6a3/Slc6a9/Slc7a5/Slc9a6	22
MF	GO:0001784	phosphotyrosine residue binding	7/606	47/28275	6.95	5.99771E-05	0.001326409	0.000949429	Bcar3/Grb10/Mapk3/Plcg2/Ptpn5/Ptpn6/Vav1	7
MF	GO:0005267	potassium channel activity	11/606	121/28275	4.24	6.08588E-05	0.001326409	0.000949429	Ccdc51/Grik5/Hcn3/Kcna3/Kcnab3/Kcnb1/Kcnc1/Kcnd2/Kcnip2/Snap25/Tmem38a	11
MF	GO:0016247	channel regulator activity	12/606	144/28275	3.89	6.69899E-05	0.001423535	0.001018952	Arpp19/Atp2b4/Ensa/Fgf13/Flna/Kcnab3/Kcnip2/Nrxn3/Prkcb/Rem2/Scn1b/Stim1	12
MF	GO:0005254	chloride channel activity	9/606	83/28275	5.06	7.21669E-05	0.001496143	0.001070924	Ano3/Clcn2/Clic1/Gabra3/Gabra4/Gabrb2/Gabrd/Ttyh1/Ttyh2	9
MF	GO:0015103	inorganic anion transmembrane transporter activity	12/606	146/28275	3.83	7.65602E-05	0.001549433	0.001109068	Ano3/Clcn2/Clic1/Gabra3/Gabra4/Gabrb2/Gabrd/Slc18a2/Slc6a3/Ttyh1/Ttyh2/Vdac3	12
MF	GO:0030552	cAMP binding	5/606	22/28275	10.6	8.65751E-05	0.001711368	0.001224979	Hcn3/Pde10a/Pde4a/Prkar1b/Prkar2b	5
MF	GO:0001786	phosphatidylserine binding	8/606	68/28275	5.49	0.000101442	0.00195968	0.001402719	Anxa2/Gap43/Hmgb1/Marcks/Mme/Osbpl8/Smpd3/Syt17	8
MF	GO:0005261	cation channel activity	19/606	333/28275	2.66	0.000119523	0.002234877	0.001599702	Anxa2/Asic1/Atp5pb/Ccdc51/Grik5/Hcn3/Kcna3/Kcnab3/Kcnb1/Kcnc1/Kcnd2/Kcnip2/Mcu/P2rx7/Ryr3/Scn1b/Snap25/Stim1/Tmem38a	19
MF	GO:0051087	chaperone binding	10/606	109/28275	4.28	0.000120946	0.002234877	0.001599702	Bag3/Bax/Dnajb6/Gnb5/H2-K1/Hspa2/Hspe1/Pdpn/Sod1/Timm9	10
MF	GO:0016790	thiolester hydrolase activity	7/606	53/28275	6.16	0.00013157	0.002379463	0.001703195	Abcd3/Abhd10/Acot1/Acot11/Esd/Nudt7/Pnkd	7
MF	GO:0047485	protein N-terminus binding	11/606	133/28275	3.86	0.000142854	0.002529711	0.001810741	Adcy8/Clu/Dab2ip/Epb41/Kcnb1/Kcnip2/Pelp1/Ppp1cc/Slc6a3/Snap25/Traf6	11
MF	GO:0015079	potassium ion transmembrane transporter activity	12/606	157/28275	3.57	0.000153075	0.00265539	0.0019007	Ccdc51/Grik5/Hcn3/Kcna3/Kcnab3/Kcnb1/Kcnc1/Kcnd2/Kcnip2/Slc9a6/Snap25/Tmem38a	12
MF	GO:0003713	transcription coactivator activity	16/606	258/28275	2.89	0.000157892	0.00268417	0.001921301	Actn2/Apex1/Brd4/Crebbp/Hmga1/Hmgb1/Hmgb2/Kdm1a/Naca/Niban2/Nucks1/Prkcb/Rbm39/Setd3/Sra1/Trim25	16
MF	GO:0008509	anion transmembrane transporter activity	18/606	313/28275	2.68	0.000162366	0.002706101	0.001936998	Ano3/Clcn2/Clic1/Gabra3/Gabra4/Gabrb2/Gabrd/Slc18a2/Slc1a3/Slc25a18/Slc25a5/Slc6a3/Slc6a9/Slc7a5/Slc9a6/Ttyh1/Ttyh2/Vdac3	18
MF	GO:0019887	protein kinase regulator activity	14/606	208/28275	3.14	0.000172548	0.002820495	0.002018881	Cdk5r2/Dab2ip/Fgf13/Hmgb1/Inka2/Irgm1/Map2k2/Map3k13/P2rx7/Prkag1/Prkar1b/Prkar2b/Rac2/Tgfa	14
MF	GO:0015271	outward rectifier potassium channel activity	4/606	14/28275	13.33	0.000176244	0.00282656	0.002023222	Kcna3/Kcnb1/Kcnd2/Kcnip2	4
MF	GO:0004674	protein serine/threonine kinase activity	22/606	441/28275	2.33	0.000247451	0.003895069	0.002788049	Brd4/Brsk1/Camk1/Camk1d/Camk2b/Camkk2/Cdk11b/Cdk19/Cpne3/Eef2k/Grk5/Itpka/Map2k2/Map3k13/Mapk3/Nek3/Pdk2/Pnck/Prkag1/Prkcb/Speg/Vrk3	22
MF	GO:0004620	phospholipase activity	9/606	98/28275	4.28	0.000260241	0.004021912	0.002878842	Abhd4/Gdpd5/Pla2g4a/Pla2g7/Plcd3/Plcg2/Pld1/Smpd3/Smpd4	9
MF	GO:0015081	sodium ion transmembrane transporter activity	11/606	145/28275	3.54	0.000304375	0.004552478	0.003258616	Asic1/Cnnm4/Grik5/Hcn3/Scn1b/Slc10a4/Slc18a2/Slc1a3/Slc6a3/Slc6a9/Slc9a6	11
MF	GO:0016298	lipase activity	10/606	122/28275	3.82	0.000305284	0.004552478	0.003258616	Abhd4/Gdpd5/Lipa/Pla2g4a/Pla2g7/Plcd3/Plcg2/Pld1/Smpd3/Smpd4	10
MF	GO:0016829	lyase activity	13/606	195/28275	3.11	0.000319513	0.004629035	0.003313414	Adcy3/Adcy8/Ddc/Echdc1/Eno2/Etnppl/Gucy1b1/Hacd3/Hmga1/Hmgb1/Me2/Sgpl1/Tpi1	13
MF	GO:0003684	damaged DNA binding	7/606	61/28275	5.35	0.000321309	0.004629035	0.003313414	Apex1/Crebbp/Hmgb1/Hmgb2/Msh2/Pcna/Rad23a	7
MF	GO:0005085	guanyl-nucleotide exchange factor activity	13/606	200/28275	3.03	0.000407386	0.0057713	0.004131036	Arhgef10/Arhgef2/Bcar3/Farp1/Lamtor1/Ngef/Nucb2/Plekhg5/Ralgps2/Rasgrp2/Rcc2/Tiam2/Vav1	13
MF	GO:0097110	scaffold protein binding	8/606	84/28275	4.44	0.000443709	0.006182832	0.004425606	Atp2b4/Dynll1/Map2k2/Mapk3/Nlgn3/P2rx7/Plcg2/Vim	8
MF	GO:0015399	primary active transmembrane transporter activity	11/606	152/28275	3.38	0.000455481	0.006244496	0.004469744	Abca1/Abca8b/Abcd3/Atp2b1/Atp2b4/Atp6v1g1/Cox7a1/Cox7a2/Cox7c/Ipo8/Ndufa10	11
MF	GO:0016922	nuclear receptor binding	11/606	155/28275	3.31	0.000537197	0.007247902	0.005187972	Dnmt1/Grm1/Hmga1/Kdm1a/Pcna/Prkcb/Sra1/Stat1/Stat3/Strn/Wipi1	11
MF	GO:0017124	SH3 domain binding	10/606	133/28275	3.51	0.000606551	0.007940748	0.005683904	Arhgap6/Dab2ip/Dnm2/Grik5/Inpp5j/Picalm/Ptpn6/Sirpa/Vasp/Wipf3	10
MF	GO:0005249	voltage-gated potassium channel activity	8/606	88/28275	4.24	0.000607234	0.007940748	0.005683904	Hcn3/Kcna3/Kcnab3/Kcnb1/Kcnc1/Kcnd2/Kcnip2/Snap25	8
MF	GO:0004890	GABA-A receptor activity	4/606	19/28275	9.82	0.000626722	0.008071415	0.005777434	Gabra3/Gabra4/Gabrb2/Gabrd	4
MF	GO:0098960	postsynaptic neurotransmitter receptor activity	7/606	69/28275	4.73	0.000685888	0.008701559	0.006228484	Drd1/Gabra3/Gabra4/Gabrb2/Gabrd/Grik5/Grm1	7
MF	GO:0019207	kinase regulator activity	14/606	240/28275	2.72	0.000730804	0.009135045	0.006538769	Cdk5r2/Dab2ip/Fgf13/Hmgb1/Inka2/Irgm1/Map2k2/Map3k13/P2rx7/Prkag1/Prkar1b/Prkar2b/Rac2/Tgfa	14
MF	GO:0035591	signaling adaptor activity	7/606	70/28275	4.67	0.000748231	0.009217344	0.006597678	Dab2ip/Gnb5/Grb10/Homer2/Map2k2/Sorbs1/Stat3	7
MF	GO:0030295	protein kinase activator activity	9/606	116/28275	3.62	0.000898437	0.010909596	0.007808974	Cdk5r2/Dab2ip/Fgf13/Hmgb1/Irgm1/Map2k2/Map3k13/P2rx7/Tgfa	9
MF	GO:0050998	nitric-oxide synthase binding	4/606	21/28275	8.89	0.0009354	0.011179632	0.008002263	Actb/Atp2b4/Cdh2/Dnm2	4

MF	GO:0005244	voltage-gated ion channel activity	12/606	192/28275	2.92	0.000946981	0.011179632	0.008002263	Clcn2/Clic1/Hcn3/Kcna3/Kcnab3/Kcnb1/Kcnc1/Kcnd2/Kcnip2/Scn1b/Snap25/Vdac3	12
MF	GO:0022832	voltage-gated channel activity	12/606	193/28275	2.9	0.000990591	0.011534274	0.008256112	Clcn2/Clic1/Hcn3/Kcna3/Kcnab3/Kcnb1/Kcnc1/Kcnd2/Kcnip2/Scn1b/Snap25/Vdac3	12
MF	GO:0048306	calcium-dependent protein binding	8/606	95/28275	3.93	0.001008137	0.01157995	0.008288806	Anxa2/Anxa4/Atp2b4/Cpne3/S100a11/S100a4/S100a6/Snap25	8
MF	GO:0008504	monoamine transmembrane transporter activity	3/606	10/28275	14	0.001050555	0.011906289	0.008522396	Slc18a2/Slc18a3/Slc6a3	3
MF	GO:0000217	DNA secondary structure binding	5/606	37/28275	6.31	0.001099637	0.012009367	0.008596179	Hmga1/Hmgb1/Mef2c/Msh2/Yy1	5
MF	GO:0030551	cyclic nucleotide binding	5/606	37/28275	6.31	0.001099637	0.012009367	0.008596179	Hcn3/Pde10a/Pde4a/Prkar1b/Prkar2b	5
MF	GO:0016917	GABA receptor activity	4/606	22/28275	8.48	0.001124028	0.012009367	0.008596179	Gabra3/Gabra4/Gabrb2/Gabrd	4
MF	GO:0048156	tau protein binding	4/606	22/28275	8.48	0.001124028	0.012009367	0.008596179	Clu/Hspa2/Picalm/Ppp2r2a	4
MF	GO:0019843	rRNA binding	7/606	75/28275	4.35	0.001130293	0.012009367	0.008596179	Brix1/Rpl23/Rpl23a/Rpl37/Rplp0/Rps9/Taco1	7
MF	GO:0044325	transmembrane transporter binding	10/606	147/28275	3.17	0.001309328	0.013739866	0.009834852	Actn2/Fgf13/Flna/Hap1/Kcnab3/Kcnb1/Kcnc1/Kcnip2/Scn1b/Snap25	10
MF	GO:0019209	kinase activator activity	9/606	123/28275	3.41	0.001359944	0.014096981	0.010090471	Cdk5r2/Dab2ip/Fgf13/Hmgb1/Irgm1/Map2k2/Map3k13/P2rx7/Tgfa	9
MF	GO:0004857	enzyme inhibitor activity	19/606	409/28275	2.17	0.001472523	0.014875541	0.010647756	Anxa2/Aplp2/Arpp19/Atp2b4/Bod1/Cst3/Cstb/Dgki/Dynll1/Ensa/Inka2/Phactr1/Phactr4/Prkar1b/Prkar2b/Rpl23/Rpl37/Spock3/Ugt1a1	19
MF	GO:0043539	protein serine/threonine kinase activator activity	6/606	58/28275	4.83	0.001487554	0.014875541	0.010647756	Cdk5r2/Dab2ip/Irgm1/Map2k2/Map3k13/P2rx7	6
MF	GO:0050840	extracellular matrix binding	6/606	58/28275	4.83	0.001487554	0.014875541	0.010647756	Ache/Adgrg1/Anxa2/Lgals1/Sparc/Thbs4	6
MF	GO:0019003	GDP binding	7/606	79/28275	4.13	0.001534143	0.015163037	0.010853542	Gbp2/Nras/Rab14/Rab31/Rab8b/Rhob/Rras2	7
MF	GO:0004864	protein phosphatase inhibitor activity	5/606	40/28275	5.83	0.00157474	0.015245603	0.010912642	Arpp19/Bod1/Ensa/Phactr1/Phactr4	5
MF	GO:0030742	GTP-dependent protein binding	4/606	24/28275	7.78	0.001578368	0.015245603	0.010912642	Rab34/Rab3a/Rab3c/Rac1	4
MF	GO:0004016	adenylate cyclase activity	3/606	12/28275	11.66	0.001865378	0.017423856	0.012471813	Adcy3/Adcy8/Gucy1b1	3
MF	GO:0070513	death domain binding	3/606	12/28275	11.66	0.001865378	0.017423856	0.012471813	Bax/Dap/Irgm1	3
MF	GO:1901338	catecholamine binding	3/606	12/28275	11.66	0.001865378	0.017423856	0.012471813	Drd1/Slc6a3/Th	3
MF	GO:0005547	phosphatidylinositol-3,4,5-trisphosphate binding	5/606	42/28275	5.55	0.001965625	0.018160664	0.012999212	Fundc2/Myo1b/Nrgn/Phlda3/Zfyve16	5
MF	GO:0022853	active ion transmembrane transporter activity	13/606	238/28275	2.55	0.002005996	0.018334368	0.013123547	Atp2b1/Atp2b4/Atp6v1g1/Cox7a1/Cox7a2/Cox7c/Slc10a4/Slc18a2/Slc1a3/Slc25a5/Slc6a3/Slc6a9/Slc9a6	13
MF	GO:0003725	double-stranded RNA binding	7/606	84/28275	3.89	0.002187709	0.019782476	0.014160088	Dhx36/Hmgb1/Mrpl44/Msn/Sidt1/Strbp/Vim	7
MF	GO:0022851	GABA-gated chloride ion channel activity	3/606	13/28275	10.77	0.002386574	0.021299011	0.015245608	Gabra3/Gabra4/Gabrb2	3
MF	GO:0019212	phosphatase inhibitor activity	5/606	44/28275	5.3	0.002423003	0.021299011	0.015245608	Arpp19/Bod1/Ensa/Phactr1/Phactr4	5
MF	GO:0072341	modified amino acid binding	8/606	109/28275	3.42	0.002430593	0.021299011	0.015245608	Anxa2/Gap43/Hmgb1/Marcks/Mme/Osbpl8/Smpd3/Syt17	8
MF	GO:0030594	neurotransmitter receptor activity	8/606	110/28275	3.39	0.002573186	0.022152785	0.01585673	Drd1/Gabra3/Gabra4/Gabrb2/Gabrd/Grik5/Grm1/Htr4	8
MF	GO:0015276	ligand-gated ion channel activity	9/606	135/28275	3.11	0.002580148	0.022152785	0.01585673	Asic1/Ccdc51/Gabra3/Gabra4/Gabrb2/Gabrd/Grik5/P2rx7/Ryr3	9
MF	GO:0050839	cell adhesion molecule binding	15/606	307/28275	2.28	0.00278561	0.023677684	0.016948237	Cd151/Cdh2/Ctnna3/Gfap/Hmgb1/Lcp1/Msn/Nexn/Nlgn3/Prom1/Ptk7/Ptpn6/Sema7a/Sirpa/Thbs4	15
MF	GO:0022834	ligand-gated channel activity	9/606	137/28275	3.07	0.002848792	0.023974983	0.01716104	Asic1/Ccdc51/Gabra3/Gabra4/Gabrb2/Gabrd/Grik5/P2rx7/Ryr3	9
MF	GO:0005545	1-phosphatidylinositol binding	3/606	14/28275	10	0.002989391	0.024669732	0.017658335	Epb41/Picalm/Zfyve16	3
MF	GO:0044548	S100 protein binding	3/606	14/28275	10	0.002989391	0.024669732	0.017658335	Anxa2/S100a11/S100a6	3
MF	GO:0005546	phosphatidylinositol-4,5-bisphosphate binding	7/606	90/28275	3.63	0.003234497	0.026435791	0.018922461	Actn2/Anxa2/Apba1/Myo1b/Phlda3/Picalm/Stxbp6	7
MF	GO:1902936	phosphatidylinositol bisphosphate binding	8/606	115/28275	3.25	0.003386984	0.027418446	0.019625835	Actn2/Anxa2/Apba1/Myo1b/Phlda3/Picalm/Stxbp6/Wipi1	8
MF	GO:0032266	phosphatidylinositol-3-phosphate binding	5/606	48/28275	4.86	0.003562179	0.028564645	0.020446272	Dab2ip/Phlda3/Pla2g4a/Snx19/Wipi1	5
MF	GO:0005230	extracellular ligand-gated ion channel activity	6/606	69/28275	4.06	0.003616673	0.028682929	0.020530939	Gabra3/Gabra4/Gabrb2/Gabrd/Grik5/P2rx7	6
MF	GO:0005523	tropomyosin binding	3/606	15/28275	9.33	0.003677672	0.028682929	0.020530939	Nebl/S100a6/Tmod1	3
MF	GO:0016289	CoA hydrolase activity	4/606	30/28275	6.22	0.003678164	0.028682929	0.020530939	Abcd3/Acot1/Acot11/Nudt7	4
MF	GO:0016887	ATP hydrolysis activity	13/606	258/28275	2.35	0.004006043	0.030955785	0.022157825	Abca1/Abca8b/Abcd3/Atp2b1/Atp6v1g1/Chd2/Hspa2/Hspe1/Kif21b/Msh2/Pex1/Rfc1/Yme1l1	13
MF	GO:0000149	SNARE binding	8/606	119/28275	3.14	0.004171204	0.03194165	0.022863497	Abca1/Cplx1/Kcnb1/Picalm/Snap25/Stx3/Syt17/Vamp3	8
MF	GO:0015459	potassium channel regulator activity	5/606	50/28275	4.67	0.004256478	0.032017754	0.022917972	Arpp19/Ensa/Flna/Kcnab3/Kcnip2	5
MF	GO:0030331	nuclear estrogen receptor binding	5/606	50/28275	4.67	0.004256478	0.032017754	0.022917972	Dnmt1/Grm1/Pcna/Strn/Wipi1	5
MF	GO:0019894	kinesin binding	5/606	51/28275	4.57	0.004637526	0.034193892	0.024475628	Actb/Arhgef10/Dcp1a/Kcnc1/Klc1	5
MF	GO:0022829	wide pore channel activity	4/606	32/28275	5.83	0.004666461	0.034193892	0.024475628	Gjb6/Gjc2/Panx2/Vdac3	4
MF	GO:0051059	NF-kappaB binding	4/606	32/28275	5.83	0.004666461	0.034193892	0.024475628	Anxa4/Apex1/Commd6/Dnaja3	4
MF	GO:1904315	transmitter-gated ion channel activity involved in regulation of postsynaptic membrane potential	5/606	52/28275	4.49	0.005042199	0.036631359	0.026220341	Gabra3/Gabra4/Gabrb2/Gabrd/Grik5	5
MF	GO:0016831	carboxy-lyase activity	4/606	33/28275	5.66	0.005221503	0.037094829	0.026552088	Ddc/Echdc1/Me2/Sgpl1	4
MF	GO:0000400	four-way junction DNA binding	3/606	17/28275	8.23	0.005324199	0.037094829	0.026552088	Hmgb1/Msh2/Yy1	3
MF	GO:0004029	aldehyde dehydrogenase (NAD+) activity	3/606	17/28275	8.23	0.005324199	0.037094829	0.026552088	Aldh1a1/Aldh1b1/Aldh1l1	3
MF	GO:0005237	inhibitory extracellular ligand-gated ion channel activity	3/606	17/28275	8.23	0.005324199	0.037094829	0.026552088	Gabra3/Gabra4/Gabrb2	3
MF	GO:0005326	neurotransmitter transmembrane transporter activity	3/606	17/28275	8.23	0.005324199	0.037094829	0.026552088	Cplx1/Slc18a3/Slc6a3	3
MF	GO:0030374	nuclear receptor coactivator activity	5/606	53/28275	4.4	0.005471259	0.037809514	0.027063652	Actn2/Hmga1/Kdm1a/Prkcb/Sra1	5
MF	GO:0044389	ubiquitin-like protein ligase binding	15/606	332/28275	2.11	0.005712799	0.039160314	0.028030541	Ckb/Clu/Foxo1/Hltf/Prkar2b/Rbx1/Rpl23/Slc25a5/Smg5/Sorbs1/Stam/Stat1/Tcp1/Trpf6/Vcl	15
MF	GO:0051117	ATPase binding	7/606	100/28275	3.27	0.005777344	0.039263749	0.028104578	Abca1/Atp6v1g1/Drd1/Pelp1/Rab3a/Rac1/Tmem106b	7
MF	GO:0017022	myosin binding	6/606	76/28275	3.68	0.005820273	0.039263749	0.028104578	Hap1/Myl12b/Rab14/Rab3a/Rab3c/Snap25	6
MF	GO:0000287	magnesium ion binding	11/606	211/28275	2.43	0.006082629	0.040710507	0.029140152	Brsk1/Comt/Dhx36/Endog/Eno2/Idh3a/Msh2/Nudt12/Nudt7/P2rx7/Thtpa	11
MF	GO:0004129	cytochrome-c oxidase activity	3/606	18/28275	7.78	0.006288385	0.041601216	0.029777713	Cox7a1/Cox7a2/Cox7c	3
MF	GO:0005544	calcium-dependent phospholipid binding	5/606	55/28275	4.24	0.006405554	0.041601216	0.029777713	Anxa2/Anxa4/Cpne3/Pla2g4a/Syt17	5
MF	GO:0099529	neurotransmitter receptor activity involved in regulation of postsynaptic membrane potential	5/606	55/28275	4.24	0.006405554	0.041601216	0.029777713	Gabra3/Gabra4/Gabrb2/Gabrd/Grik5	5
MF	GO:0042626	ATPase-coupled transmembrane transporter activity	7/606	102/28275	3.2	0.006427389	0.041601216	0.029777713	Abca1/Abca8b/Abcd3/Atp2b1/Atp2b4/Atp6v1g1/Ipo8	7
MF	GO:0016776	phosphotransferase activity, phosphate group as acceptor	4/606	35/28275	5.33	0.006480424	0.041601216	0.029777713	Ak2/Ak5/Itpka/Nme3	4
MF	GO:0004030	aldehyde dehydrogenase [NAD(P)+] activity	3/606	19/28275	7.37	0.007349947	0.045550595	0.032604637	Aldh1a1/Aldh1b1/Aldh1l1	3
MF	GO:0004435	phosphatidylinositol phospholipase C activity	3/606	19/28275	7.37	0.007349947	0.045550595	0.032604637	Gdpd5/Plcd3/Plcg2	3
MF	GO:0004629	phospholipase C activity	3/606	19/28275	7.37	0.007349947	0.045550595	0.032604637	Gdpd5/Plcd3/Plcg2	3
MF	GO:0016675	oxidoreductase activity, acting on a heme group of donors	3/606	19/28275	7.37	0.007349947	0.045550595	0.032604637	Cox7a1/Cox7a2/Cox7c	3
MF	GO:0022824	transmitter-gated ion channel activity	5/606	57/28275	4.09	0.00744635	0.045550595	0.032604637	Gabra3/Gabra4/Gabrb2/Gabrd/Grik5	5
MF	GO:0022835	transmitter-gated channel activity	5/606	57/28275	4.09	0.00744635	0.045550595	0.032604637	Gabra3/Gabra4/Gabrb2/Gabrd/Grik5	5
MF	GO:0016835	carbon-oxygen lyase activity	6/606	80/28275	3.5	0.00744979	0.045550595	0.032604637	Echdc1/Eno2/Etnppl/Hacd3/Hmga1/Tpi1	6
MF	GO:0015078	proton transmembrane transporter activity	7/606	105/28275	3.11	0.007502451	0.045550595	0.032604637	Atp5pb/Atp6v1g1/Cox7a1/Cox7a2/Cox7c/Slc25a5/Slc9a6	7
MF	GO:0031489	myosin V binding	3/606	20/28275	7	0.008511079	0.050946599	0.036467039	Rab14/Rab3a/Rab3c	3
MF	GO:0099186	structural constituent of postsynapse	3/606	20/28275	7	0.008511079	0.050946599	0.036467039	Actb/Camk2b/Ina	3
MF	GO:0030159	signaling receptor complex adaptor activity	4/606	38/28275	4.91	0.008660684	0.051479593	0.036848551	Gnb5/Grb10/Homer2/Sorbs1	4
MF	GO:0016614	oxidoreductase activity, acting on CH-OH group of donors	9/606	164/28275	2.56	0.009100744	0.053719668	0.038451973	Chdh/Dhrs1/Dhrs13/Idh3a/Kcnab3/Kdsr/Me2/Ptgr1/Ugdh	9
MF	GO:0005319	lipid transporter activity	9/606	165/28275	2.55	0.009449005	0.055011333	0.039376533	Abca1/Abca8b/Abcd3/Ano3/Gramd1a/Osbpl8/Pitpnc1/Slc10a4/Xkr9	9
MF	GO:0052689	carboxylic ester hydrolase activity	9/606	165/28275	2.55	0.009449005	0.055011333	0.039376533	Abhd10/Abhd4/Ache/Acot1/Acot11/Esd/Lipa/Pla2g4a/Pla2g7	9
MF	GO:0015631	tubulin binding	16/606	386/28275	1.93	0.00970467	0.055384255	0.039643467	Abraxas2/Arhgef2/Brsk1/Cnn3/Dip2b/Dnm2/Fgf13/Gjb6/Ift81/Kif21b/Klc1/Rcc2/Sncg/Stim1/Strbp/Ttll7	16
MF	GO:0004550	nucleoside diphosphate kinase activity	3/606	21/28275	6.67	0.009773692	0.055384255	0.039643467	Ak2/Ak5/Nme3	3
MF	GO:0070300	phosphatidic acid binding	3/606	21/28275	6.67	0.009773692	0.055384255	0.039643467	Nrgn/Pitpnc1/Smpd3	3

MF	GO:0099095	ligand-gated anion channel activity	3/606	21/28275	6.67	0.009773692	0.055384255	0.039643467	Gabra3/Gabra4/Gabrb2	3
MF	GO:0060090	molecular adaptor activity	17/606	421/28275	1.88	0.009993853	0.056256788	0.040268017	Actn2/Ap4e1/Dab2ip/Dlgap4/Frmd4a/Gnb5/Grb10/Homer2/Lamtor1/Map2k2/Septin6/Skp1/Snap25/Sorbs1/Stat3/Stx3/Vamp3	17
MF	GO:0005243	gap junction channel activity	3/606	22/28275	6.36	0.011139428	0.061969093	0.044356825	Gjb6/Gjc2/Panx2	3
MF	GO:0030674	protein-macromolecule adaptor activity	14/606	326/28275	2	0.011272507	0.061969093	0.044356825	Actn2/Ap4e1/Dab2ip/Frmd4a/Gnb5/Grb10/Homer2/Map2k2/Skp1/Snap25/Sorbs1/Stat3/Stx3/Vamp3	14
MF	GO:0005246	calcium channel regulator activity	4/606	41/28275	4.55	0.011300246	0.061969093	0.044356825	Nrxn3/Prkcb/Rem2/Stim1	4
MF	GO:0043548	phosphatidylinositol 3-kinase binding	4/606	41/28275	4.55	0.011300246	0.061969093	0.044356825	Dab2ip/Dnm2/Pik3ip1/Rasd2	4
MF	GO:0004540	ribonuclease activity	7/606	114/28275	2.86	0.011525475	0.062799062	0.044950908	Apex1/Cnot7/Endog/Exosc10/Mrpl44/Noct/Nudt12	7
MF	GO:0051082	unfolded protein binding	6/606	88/28275	3.18	0.01165501	0.06290073	0.04502368	Clu/Dnaja3/Dnajb6/Hspa2/Hspe1/Naca	6
MF	GO:0022843	voltage-gated cation channel activity	8/606	142/28275	2.63	0.011692136	0.06290073	0.04502368	Hcn3/Kcna3/Kcnab3/Kcnb1/Kcnc1/Kcnd2/Kcnip2/Snap25	8
MF	GO:0031072	heat shock protein binding	8/606	143/28275	2.61	0.012160454	0.065008716	0.046532555	Bax/Dnaja3/Dnajb6/Gbp2/Gucy1b1/Hspa2/Slc18a2/Unc45a	8
MF	GO:0022839	ion gated channel activity	4/606	42/28275	4.44	0.012282838	0.065252578	0.046707108	Asic1/Tmem38a/Ttyh1/Ttyh2	4
MF	GO:0005096	GTPase activator activity	11/606	236/28275	2.17	0.013392577	0.070706151	0.050610718	Arhgap20/Arhgap6/Chn1/Dab2ip/Gnb5/Rap1gap/Rgs8/Rgs9/Srgap1/Tbc1d8/Tiam2	11
MF	GO:0140098	catalytic activity, acting on RNA	15/606	368/28275	1.9	0.013829198	0.072560605	0.051938118	Apex1/Cnot7/Dhx32/Dhx36/Ears2/Endog/Exosc10/Mrpl44/Mtrex/Noct/Nudt12/Polr2f/Pus10/Sars2/Tut4	15
MF	GO:0004114	3',5'-cyclic-nucleotide phosphodiesterase activity	3/606	24/28275	5.83	0.014185576	0.072798859	0.052108657	Pde10a/Pde1b/Pde4a	3
MF	GO:0017075	syntaxin-1 binding	3/606	24/28275	5.83	0.014185576	0.072798859	0.052108657	Cplx1/Snap25/Vamp3	3
MF	GO:0031681	G-protein beta-subunit binding	3/606	24/28275	5.83	0.014185576	0.072798859	0.052108657	Gng12/Gng4/Rasd2	3
MF	GO:0016791	phosphatase activity	12/606	270/28275	2.07	0.014217189	0.072798859	0.052108657	Enpp1/Inpp5j/Mtmr12/Noct/Pald1/Pip4p1/Ppp1cc/Ptpa/Ptpn5/Ptpn6/Sgpp1/Ssh3	12
MF	GO:0005272	sodium channel activity	4/606	44/28275	4.24	0.014407996	0.07307655	0.052307426	Asic1/Grik5/Hcn3/Scn1b	4
MF	GO:0001540	amyloid-beta binding	5/606	67/28275	3.48	0.014443365	0.07307655	0.052307426	Apba1/Apba2/Clu/Cst3/Fbxo2	5
MF	GO:0030165	PDZ domain binding	7/606	120/28275	2.72	0.014954247	0.075213669	0.053837153	Apba1/Atp2b1/Atp2b4/Gng12/Grik5/Map2k2/Shisa9	7
MF	GO:0048487	beta-tubulin binding	4/606	45/28275	4.15	0.01555233	0.077761648	0.055660969	Fgf13/Gjb6/Sncg/Ttll7	4
MF	GO:0009975	cyclase activity	3/606	25/28275	5.6	0.015868046	0.078417669	0.056130542	Adcy3/Adcy8/Gucy1b1	3
MF	GO:0016849	phosphorus-oxygen lyase activity	3/606	25/28275	5.6	0.015868046	0.078417669	0.056130542	Adcy3/Adcy8/Gucy1b1	3
MF	GO:0015370	solute:sodium symporter activity	5/606	69/28275	3.38	0.016237398	0.079779124	0.057105057	Slc10a4/Slc18a2/Slc1a3/Slc6a3/Slc6a9	5
MF	GO:0120013	lipid transfer activity	4/606	46/28275	4.06	0.016752256	0.081835733	0.058577156	Abca1/Gramd1a/Osbpl8/Pitpnc1	4
MF	GO:0008013	beta-catenin binding	6/606	96/28275	2.92	0.017305673	0.083695987	0.059908707	Ajap1/Cdh2/Ctnna3/Foxo1/Setd1a/Skp1	6
MF	GO:0004112	cyclic-nucleotide phosphodiesterase activity	3/606	26/28275	5.38	0.017657783	0.083695987	0.059908707	Pde10a/Pde1b/Pde4a	3
MF	GO:0008408	3'-5' exonuclease activity	4/606	47/28275	3.97	0.018008524	0.083695987	0.059908707	Apex1/Cnot7/Exosc10/Noct	4
MF	GO:0016616	oxidoreductase activity, acting on the CH-OH group of donors, NAD or NADP as acceptor	8/606	154/28275	2.42	0.018259635	0.083695987	0.059908707	Dhrs1/Dhrs13/Idh3a/Kcnab3/Kdsr/Me2/Ptgr1/Ugdh	8
MF	GO:0003680	minor groove of adenine-thymine-rich DNA binding	2/606	10/28275	9.33	0.018413117	0.083695987	0.059908707	Hmga1/Mef2c	2
MF	GO:0004017	adenylate kinase activity	2/606	10/28275	9.33	0.018413117	0.083695987	0.059908707	Ak2/Ak5	2
MF	GO:0031628	opioid receptor binding	2/606	10/28275	9.33	0.018413117	0.083695987	0.059908707	Flna/Pdyn	2
MF	GO:0043878	glyceraldehyde-3-phosphate dehydrogenase (NAD+) (non-phosphorylating) activity	2/606	10/28275	9.33	0.018413117	0.083695987	0.059908707	Aldh1a1/Aldh1b1	2
MF	GO:0047499	calcium-independent phospholipase A2 activity	2/606	10/28275	9.33	0.018413117	0.083695987	0.059908707	Pla2g4a/Pla2g7	2
MF	GO:0050543	icosatetraenoic acid binding	2/606	10/28275	9.33	0.018413117	0.083695987	0.059908707	Cyp4f14/Stx3	2
MF	GO:0050786	RAGE receptor binding	2/606	10/28275	9.33	0.018413117	0.083695987	0.059908707	Hmgb2/S100a4	2
MF	GO:0070878	primary miRNA binding	2/606	10/28275	9.33	0.018413117	0.083695987	0.059908707	Pus10/Stat3	2
MF	GO:1990948	ubiquitin ligase inhibitor activity	2/606	10/28275	9.33	0.018413117	0.083695987	0.059908707	Rpl23/Rpl37	2
MF	GO:0031625	ubiquitin protein ligase binding	13/606	315/28275	1.93	0.019166668	0.085987192	0.061548727	Ckb/Clu/Foxo1/Hltf/Prkar2b/Rbx1/Rpl23/Slc25a5/Smg5/Sorbs1/Tpi1/Traf6/Vcl	13
MF	GO:0001223	transcription coactivator binding	4/606	48/28275	3.89	0.019321828	0.085987192	0.061548727	Crebbp/Foxo1/Rpl23/Stat1	4
MF	GO:0015485	cholesterol binding	4/606	48/28275	3.89	0.019321828	0.085987192	0.061548727	Gramd1a/Osbpl8/Prom1/Sidt1	4
MF	GO:0032813	tumor necrosis factor receptor superfamily binding	4/606	48/28275	3.89	0.019321828	0.085987192	0.061548727	Dab2ip/Nucb2/Stat1/Traf6	4
MF	GO:0035035	histone acetyltransferase binding	3/606	27/28275	5.18	0.019555277	0.086180683	0.061687226	Pcna/Pla2g4a/Stat1	3
MF	GO:0043177	organic acid binding	8/606	156/28275	2.39	0.019568085	0.086180683	0.061687226	Cyp4f14/Ddc/Hmgcs1/Ogfod1/Slc1a3/Stx3/Th/Ugt1a1	8
MF	GO:0004527	exonuclease activity	5/606	73/28275	3.2	0.020256598	0.088753134	0.063528559	Apex1/Cnot7/Enpp1/Exosc10/Noct	5
MF	GO:0140097	catalytic activity, acting on DNA	10/606	221/28275	2.11	0.021460159	0.091556614	0.06553526	Apex1/Chd2/Dhx36/Dnmt1/Endog/Hltf/Hmga1/Msh2/Pcna/Rfc1	10
MF	GO:0005158	insulin receptor binding	3/606	28/28275	5	0.021560825	0.091556614	0.06553526	Enpp1/Grb10/Sorbs1	3
MF	GO:0035925	mRNA 3'-UTR AU-rich region binding	3/606	28/28275	5	0.021560825	0.091556614	0.06553526	Dhx36/Hnrnpa0/Tia1	3
MF	GO:0047617	acyl-CoA hydrolase activity	3/606	28/28275	5	0.021560825	0.091556614	0.06553526	Abcd3/Acot1/Acot11	3
MF	GO:0015453	oxidoreduction-driven active transmembrane transporter activity	4/606	50/28275	3.73	0.022122055	0.091556614	0.06553526	Cox7a1/Cox7a2/Cox7c/Ndufa10	4
MF	GO:0005388	P-type calcium transporter activity	2/606	11/28275	8.48	0.022189015	0.091556614	0.06553526	Atp2b1/Atp2b4	2
MF	GO:0008503	benzodiazepine receptor activity	2/606	11/28275	8.48	0.022189015	0.091556614	0.06553526	Gabra3/Gabra4	2
MF	GO:0015378	sodium:chloride symporter activity	2/606	11/28275	8.48	0.022189015	0.091556614	0.06553526	Slc18a2/Slc6a3	2
MF	GO:0030983	mismatched DNA binding	2/606	11/28275	8.48	0.022189015	0.091556614	0.06553526	Msh2/Pcna	2
MF	GO:0031996	thioesterase binding	2/606	11/28275	8.48	0.022189015	0.091556614	0.06553526	Rac1/Traf6	2
MF	GO:0032404	mismatch repair complex binding	2/606	11/28275	8.48	0.022189015	0.091556614	0.06553526	Msh2/Pcna	2
MF	GO:0055105	ubiquitin-protein transferase inhibitor activity	2/606	11/28275	8.48	0.022189015	0.091556614	0.06553526	Rpl23/Rpl37	2
MF	GO:0016796	exonuclease activity, active with either ribo- or deoxyribonucleic acids and producing 5'-phosphomonoesters	4/606	51/28275	3.66	0.0236101	0.095345443	0.068247264	Apex1/Cnot7/Exosc10/Noct	4
MF	GO:0016830	carbon-carbon lyase activity	4/606	51/28275	3.66	0.0236101	0.095345443	0.068247264	Ddc/Echdc1/Me2/Sgpl1	4
MF	GO:0000175	3'-5'-exoribonuclease activity	3/606	29/28275	4.83	0.023674539	0.095345443	0.068247264	Cnot7/Exosc10/Noct	3
MF	GO:0005343	organic acid:sodium symporter activity	3/606	29/28275	4.83	0.023674539	0.095345443	0.068247264	Slc10a4/Slc1a3/Slc6a9	3
MF	GO:0043236	laminin binding	3/606	29/28275	4.83	0.023674539	0.095345443	0.068247264	Ache/Lgals1/Thbs4	3
MF	GO:0001221	transcription coregulator binding	7/606	132/28275	2.47	0.023881687	0.095345443	0.068247264	Cdc5l/Crebbp/Foxo1/Hmga1/Rpl23/Stat1/Ube2i	7
MF	GO:0016874	ligase activity	8/606	162/28275	2.3	0.023892446	0.095345443	0.068247264	Acsbg1/Atp5pb/Dip2a/Ears2/Naprt/Sars2/Trim25/Ttll7	8

This table lists significantly enriched Gene Ontology (GO) pathways. "MPTP" refers to the acute MPTP model, "A53T" to the 16 months A53T-α-synuclein model, and "MitoPark" to the 5 months MitoPark model.
