# Supplementary material for: Neurotoxicity, α-synuclein pathology, and mitochondrial dysfunction: A comparative study of different mouse models of Parkinson’s disease
Source: Neural Regen Res. 2025 Sep 29;21(10):5000–12. doi: 10.4103/NRR.NRR-D-25-00648 (PMC13568611; doi:10.4103/NRR.NRR-D-25-00648)
Supplement: Supplementary file 8 [file NRR-21-5000_Suppl7.pdf]

Additional Table 10. KEGG pathway identified from proteomic data analysis of the striatum in three PD mouse models

MPTP

ID	Term	Ontology Source	Term PValue	Term PValue Corrected with Benjamini-Hochberg	Group PValue	Group PValue Corrected with Benjamini-Hochberg	GOLevels	GOGroups	% Associated Genes	Nr. Genes	Rich Factor	Associated Genes Found
KEGG:04070	Phosphatidylinositol signaling system	KEGG_25.05.2022	0.019662663	0.046045475	0.019662663	0.019662663	[-1]	Group01	8.333333015	8	3.696236559	[Cds1, Dgki, Itpka, Pip4p1, Pip5k1a, Plcd3, Plcg2, Prkcb]
KEGG:04140	Autophagy	KEGG_25.05.2022	0.017399043	0.041298909	0.017399043	0.018848963	[-1]	Group02	7.746478634	11	2.921576202	[Akt1s1, Camkk2, Lamp2, Map2k2, Mapk3, Nras, Rab8a, Rragb, Rras2, Traf6, [illegible]]
KEGG:00730	Thiamine metabolism	KEGG_25.05.2022	0.014361238	0.035473719	0.014361238	0.016996009	[-1]	Group03	20	3	8.870967742	[Ak1, Ak5, Thtpa]
KEGG:04721	Synaptic vesicle cycle	KEGG_25.05.2022	7.288E-05	0.001123567	7.288E-05	0.000189488	[-1]	Group04	14.28571415	11	6.33640553	[Atp6v1g1, Cplx1, Dnm2, [illegible], Slc18a2, Slc18a3, [illegible], Slc6a3, Slc6a9, Snap25, Stx3]
KEGG:04910	Insulin signaling pathway	KEGG_25.05.2022	0.00360701	0.013084251	0.00360701	0.005210125	[-1]	Group05	8.633093834	12	3.801841518	[Foxo1, Hk2, Map2k2, Mapk3, Nras, Phka1, Phkb, Ppp1cc, Prkag1, Prkar1b, [illegible], Sorbs1]
KEGG:03010	Ribosome	KEGG_25.05.2022	0.012097411	0.032435089	0.008298473	0.010788013	[-1]	Group06	7.262569904	13	1.954620011	[Mrpl13, Mrpl19, Mrps14, Rpl23, Rpl23a, Rpl27a, Rpl28, Rpl35a, Rpl37, Rplp0, Rplp2, Rps15a, Rps9]
KEGG:05171	Coronavirus disease	KEGG_25.05.2022	0.004057609	0.014163353	0.008298473	0.010788013	[-1]	Group06	7.28744936	18	2.390380529	[C1qc, C4b, Mapk3, Plcg2, Prkcb, Rpl23, Rpl23a, Rpl27a, Rpl28, Rpl35a, Rpl37, Rplp0, Rplp2, Rps15a, Rps9, Stat1, Stat3, Traf6]
KEGG:04211	Longevity regulating pathway	KEGG_25.05.2022	0.013757836	0.034865748	0.003084074	0.00501162	[-1]	Group07	8.888889313	8	3.94265233	[Adcy3, Adcy8, Akt1s1, Bax, Camkk2, Foxo1, Nras, Prkag1]
KEGG:04213	Longevity regulating pathway	KEGG_25.05.2022	0.001398722	0.006469089	0.003084074	0.00501162	[-1]	Group07	12.9032259	8	5.817028027	[Adcy3, Adcy8, Akt1s1, Foxo1, Hspa2, Nras, Prkag1, Sod1]
KEGG:04713	Circadian entrainment	KEGG_25.05.2022	0.000633465	0.00404107	0.00022568	0.000488974	[-1]	Group08	11.22449017	11	4.879032258	[Adcy3, Adcy8, Camk2b, Gnas, Gnb5, Gng12, Gng4, Gucy1b1, Mapk3, Prkcb, Ryr3]
KEGG:04726	Serotonergic synapse	KEGG_25.05.2022	0.000201509	0.001775197	0.00022568	0.000488974	[-1]	Group08	10.68702316	14	4.532611255	[Ddc, Gabrb2, Gnas, Gnb5, Gng12, Gng4, Htr4, Kcnd2, Mapk3, Nras, Pla2g4a, Prkcb, Ptgs1, Slc18a2]
KEGG:04728	Dopaminergic synapse	KEGG_25.05.2022	0.000276543	0.002046421	0.00022568	0.000488974	[-1]	Group08	10.37036991	14	4.532611255	[Camk2b, Comt, Ddc, Drd1, Gnas, Gnb5, Gng12, Gng4, Ppp1cc, Ppp2r2a, Prkcb, Slc18a2, Slc6a3, Th]
KEGG:05030	Cocaine addiction	KEGG_25.05.2022	0.000239305	0.001924843	0.00022568	0.000488974	[-1]	Group08	16.66666603	8	7.392473118	[Ddc, Drd1, Gnas, Pdyn, [illegible], Slc18a2, Slc6a3, Th]
KEGG:05031	Amphetamine addiction	KEGG_25.05.2022	0.000136963	0.00168921	0.00022568	0.000488974	[-1]	Group08	14.49275398	10	6.428212494	[Camk2b, Ddc, Drd1, Gnas, Pdyn, Ppp1cc, Prkcb, Slc18a2, Slc6a3, Th]
KEGG:05034	Alcoholism	KEGG_25.05.2022	0.006442571	0.020201283	0.00022568	0.000488974	[-1]	Group08	7.317073345	15	3.198666253	[Camkk2, Ddc, Drd1, Gnas, Gnb5, Gng12, Gng4, H2bc3, Mapk3, Nras, Pdyn, Ppp1cc, Slc18a2, Slc6a3, Th]
KEGG:04014	Ras signaling pathway	KEGG_25.05.2022	0.001795687	0.008102489	1.33619E-05	8.68525E-05	[-1]	Group09	7.659574509	18	3.368721927	[Gab1, Gnb5, Gng12, Gng4, Map2k2, Mapk3, Nras, Pla2g4a, Plcg2, Pld1, Prkcb, Rac2, Rac3, Rasgrp2, Rras2, Shoc2, Tgfa, Tiam1]
KEGG:04713	Circadian entrainment	KEGG_25.05.2022	0.000633465	0.00404107	1.33619E-05	8.68525E-05	[-1]	Group09	11.22449017	11	4.879032258	[Adcy3, Adcy8, Camk2b, Gnas, Gnb5, Gng12, Gng4, Gucy1b1, Mapk3, Prkcb, Ryr3]
KEGG:04724	Glutamatergic synapse	KEGG_25.05.2022	3.99484E-05	0.000923807	1.33619E-05	8.68525E-05	[-1]	Group09	12.38938046	14	5.307416598	[Adcy3, Adcy8, Gnas, Gnb5, Gng12, Gng4, Grik5, Grm1, Homer2, Mapk3, Pla2g4a, Pld1, Prkcb, Slc1a3]
KEGG:04725	Cholinergic synapse	KEGG_25.05.2022	0.001918006	0.008448359	1.33619E-05	8.68525E-05	[-1]	Group09	9.821428299	11	4.279852858	[Ache, Adcy3, Adcy8, Camk2b, Gnb5, Gng12, Gng4, Mapk3, Nras, Prkcb, Slc18a3]
KEGG:04726	Serotonergic synapse	KEGG_25.05.2022	0.000201509	0.001775197	1.33619E-05	8.68525E-05	[-1]	Group09	10.68702316	14	4.532611255	[Ddc, Gabrb2, Gnas, Gnb5, Gng12, Gng4, Htr4, Kcnd2, Mapk3, Nras, Pla2g4a, Prkcb, Ptgs1, Slc18a2]
KEGG:04728	Dopaminergic synapse	KEGG_25.05.2022	0.000276543	0.002046421	1.33619E-05	8.68525E-05	[-1]	Group09	10.37036991	14	4.532611255	[Camk2b, Comt, Ddc, Drd1, Gnas, Gnb5, Gng12, Gng4, Ppp1cc, Ppp2r2a, Prkcb, Slc18a2, Slc6a3, Th]
KEGG:04730	Long-term depression	KEGG_25.05.2022	0.001124907	0.005624536	1.33619E-05	8.68525E-05	[-1]	Group09	13.33333302	8	5.913978495	[Gnas, Grm1, Gucy1b1, Map2k2, Mapk3, Nras, Pla2g4a, Prkcb]
KEGG:04912	GnRH signaling pathway	KEGG_25.05.2022	0.001200186	0.005693189	1.33619E-05	8.68525E-05	[-1]	Group09	11.11111069	10	4.928315412	[Adcy3, Adcy8, Camk2b, Gnas, Map2k2, Mapk3, Nras, Pla2g4a, Pld1, Prkcb]
KEGG:05034	Alcoholism	KEGG_25.05.2022	0.006442571	0.020201283	1.33619E-05	8.68525E-05	[-1]	Group09	7.317073345	15	3.198666253	[Camkk2, Ddc, Drd1, Gnas, Gnb5, Gng12, Gng4, H2bc3, Mapk3, Nras, Pdyn, Ppp1cc, Slc18a2, Slc6a3, Th]
KEGG:00190	Oxidative phosphorylation	KEGG_25.05.2022	0.002835967	0.010930288	4.41764E-05	0.000143573	[-1]	Group10	8.888889313	12	3.77487989	[Atp5b, Atp5h, Atp5pb, Atp6v1g1, Cox6b1, Cox7a1, Cox7a2, Cox7c, Ndufa10, Ndufa12, Ndufab1, Ndufb6]
KEGG:04714	Thermogenesis	KEGG_25.05.2022	6.2737E-05	0.001055123	4.41764E-05	0.000143573	[-1]	Group10	9.090909004	21	3.913662239	[Actb, Adcy3, Adcy8, Akt1s1, Atp5b, Atp5h, Atp5pb, Coa3, Cox6b1, Cox7a1, Cox7a2, Cox7c, Gnas, Kdm1a, Ndufa10, Ndufa12, Ndufab1, Ndufaf3, Ndufb6, Nras, Prkag1]
KEGG:05010	Alzheimer disease	KEGG_25.05.2022	0.006355662	0.020272372	4.41764E-05	0.000143573	[-1]	Group10	6.266318321	24	2.736545319	[Atp5b, Atp5h, Atp5pb, Cox6b1, Cox7a1, Cox7a2, Cox7c, Klc1, Map2k2, Mapk3, Mcu, Mme, Ndufa10, Ndufa12, Ndufab1, Ndufb6, Nras, Psma2, Ryr3, Slc25a5, Tuba4a, Tubb2a, Vdac3, Wipi1]
KEGG:05012	Parkinson disease	KEGG_25.05.2022	1.62345E-07	1.50169E-05	4.41764E-05	0.000143573	[-1]	Group10	10.60606098	28	4.532611255	[Atp5b, Atp5h, Atp5pb, Bax, Camk2b, Cox6b1, Cox7a1, Cox7a2, Cox7c, Drd1, Gnas, Klc1, Mcu, Ndufa10, Ndufa12, Ndufab1, Ndufb6, Psma2, Ryr3, Slc18a2, Slc25a5, Slc6a3, Sod1, Th, Tuba4a, Tubb2a, Txn1, Vdac3]
KEGG:05014	Amyotrophic lateral sclerosis	KEGG_25.05.2022	0.008471073	0.025690958	4.41764E-05	0.000143573	[-1]	Group10	6.233062267	23	2.713194921	[Actb, Atp5b, Atp5h, Atp5pb, Bax, Cox6b1, Cox7a1, Cox7a2, Cox7c, Hap1, Klc1, Mcu, Ndufa10, Ndufa12, Ndufab1, Ndufb6, Psma2, Rab8a, Sigmar1, Sod1, Tuba4a, Tubb2a, Wipi1]
KEGG:05016	Huntington disease	KEGG_25.05.2022	0.000176564	0.001633221	4.41764E-05	0.000143573	[-1]	Group10	7.947020054	24	3.456221198	[Atp5b, Atp5h, Atp5pb, Bax, Cox6b1, Cox7a1, Cox7a2, Cox7c, Crebbp, Hap1, Klc1, Ndufa10, Ndufa12, Ndufab1, Ndufb6, Polr2f, Psma2, Slc1a3, Slc25a5, Sod1, Tuba4a, Tubb2a, Vdac3, Wipi1]
KEGG:05020	Prion disease	KEGG_25.05.2022	7.96011E-06	0.000245437	4.41764E-05	0.000143573	[-1]	Group10	9.32835865	25	4.061798417	[Atp5b, Atp5h, Atp5pb, Bax, C1qc, Cox6b1, Cox7a1, Cox7a2, Cox7c, Hspa2, Klc1, Mapk3, Mcu, Ndufa10, Ndufa12, Ndufab1, Ndufb6, Psma2, Rac2, Ryr3, Slc25a5, Sod1, Tuba4a, Tubb2a, Vdac3]
KEGG:05022	Pathways of neurodegeneration	KEGG_25.05.2022	0.000149109	0.001622656	4.41764E-05	0.000143573	[-1]	Group10	7.006369591	33	3.043055462	[Atp5b, Atp5h, Atp5pb, Bax, Camk2b, Cox6b1, Cox7a1, Cox7a2, Cox7c, Grm1, Hap1, Klc1, Map2k2, Mapk3, Mcu, Ndufa10, Ndufa12, Ndufab1, Ndufb6, Nras, Pdyn, Prkcb, Psma2, Rab8a, Ryr3, Sigmar1, Slc25a5, Slc6a3, Sod1, Tuba4a, Tubb2a, Vdac3, Wipi1]
KEGG:05208	Chemical carcinogenesis	KEGG_25.05.2022	0.001150001	0.005598691	4.41764E-05	0.000143573	[-1]	Group10	8.108108521	18	3.486406536	[Atp5b, Atp5h, Atp5pb, Cox6b1, Cox7a1, Cox7a2, Cox7c, Map2k2, Mapk3, Ndufa10, Ndufa12, Ndufab1, Ndufb6, Nras, Pld1, Slc25a5, Sod1, Vdac3]
KEGG:05415	Diabetic cardiomyopathy	KEGG_25.05.2022	0.000158952	0.001547691	4.41764E-05	0.000143573	[-1]	Group10	9.004739761	19	3.883603389	[Atp5b, Atp5h, Atp5pb, Camk2b, Cox6b1, Cox7a1, Cox7a2, Cox7c, Ndufa10, Ndufa12, Ndufab1, Ndufb6, Pdk2, Ppp1cc, Prkcb, Ptpa, Rac2, Slc25a5, Vdac3]
KEGG:04015	Rap1 signaling pathway	KEGG_25.05.2022	0.00406363	0.013921698	1.49924E-05	6.49673E-05	[-1]	Group11	7.476635456	16	3.300825206	[Actb, Adcy3, Adcy8, Apbb1ip, Gnas, Map2k2, Mapk3, Nras, Prkcb, Rac2, Rac3, Rap1gap, Rasgrp2, Tiam1, Vasp, Vav1]
KEGG:04144	Endocytosis	KEGG_25.05.2022	0.003985176	0.014178031	1.49924E-05	6.49673E-05	[-1]	Group11	6.985294342	19	3.121266428	[2610002M06Rik, Actr3b, Arpc1b, Arpc3, Arpc5l, Chmp5, Dnm2, Grk5, H2-K1, Hspa2, Pip5k1a, Pld1, Rab22a, Rab8a, Sh3gl3, Stam, Traf6, Wipf3, Zfyve16]
KEGG:04510	Focal adhesion	KEGG_25.05.2022	0.005569688	0.018077057	1.49924E-05	6.49673E-05	[-1]	Group11	7.462686539	15	3.293676142	[Actb, Flna, Flnc, Mapk3, Myl12b, Parvb, Pip5k1a, Ppp1cc, Prkcb, Rac2, Rac3, Thbs4, Vasp, Vav1, Vcl]
KEGG:04530	Tight junction	KEGG_25.05.2022	0.000793751	0.004894797	1.49924E-05	6.49673E-05	[-1]	Group11	8.982035637	15	3.913662239	[Actb, Actr3b, Arhgef2, Arpc1b, Arpc3, Arpc5l, Msn, Myl12b, Pcna, Ppp2r2a, Prkag1, Rab8a, Tiam1, Tuba4a, Vasp]
KEGG:04650	Natural killer cell mediated cytotoxicity	KEGG_25.05.2022	0.010214157	0.028203271	1.49924E-05	6.49673E-05	[-1]	Group11	8.264462471	10	3.696236559	[H2-K1, Map2k2, Mapk3, Nras, Plcg2, Prkcb, Ptpn6, Rac2, Rac3, Vav1]
KEGG:04662	B cell receptor signaling pathway	KEGG_25.05.2022	0.002090776	0.0089952	1.49924E-05	6.49673E-05	[-1]	Group11	11.11111069	9	4.752304147	[Map2k2, Mapk3, Nras, Plcg2, Prkcb, Ptpn6, Rac2, Rac3, Vav1]
KEGG:04664	Fc epsilon RI signaling pathway	KEGG_25.05.2022	0.002104029	0.008846485	1.49924E-05	6.49673E-05	[-1]	Group11	12.12121201	8	5.376344086	[Map2k2, Mapk3, Nras, Pla2g4a, Plcg2, Rac2, Rac3, Vav1]
KEGG:04666	Fc gamma R-mediated phagocytosis	KEGG_25.05.2022	6.44238E-07	3.9728E-05	1.49924E-05	6.49673E-05	[-1]	Group11	16.30434799	15	7.077899794	[Actr3b, Arpc1b, Arpc3, Arpc5l, Dnm2, Mapk3, Marcks, Pip5k1a, Pla2g4a, Plcg2, Pld1, Prkcb, Rac2, Vasp, Vav1]
KEGG:04810	Regulation of actin cytoskeleton	KEGG_25.05.2022	9.38129E-05	0.001239671	1.49924E-05	6.49673E-05	[-1]	Group11	9.090909004	20	3.77487989	[Actb, Actr3b, Arpc1b, Arpc3, Arpc5l, Gng12, Map2k2, Mapk3, Msn, Myl12b, Nras, Pip5k1a, Ppp1cc, Rac2, Rac3, Rras2, Ssh3, Tiam1, Vav1, Vcl]
KEGG:05100	Bacterial invasion of epithelial cells	KEGG_25.05.2022	0.000309214	0.002200174	1.49924E-05	6.49673E-05	[-1]	Group11	13.15789509	10	5.760368664	[Actb, Actr3b, Arpc1b, Arpc3, Arpc5l, Ctnna3, Dnm2, Gab1, Septin6, Vcl]
KEGG:05132	Salmonella infection	KEGG_25.05.2022	8.18959E-05	0.001165442	1.49924E-05	6.49673E-05	[-1]	Group11	8.695652008	22	3.856942496	[Actb, Actr3b, Anxa2, Arpc1b, Arpc3, Arpc5l, Bax, Dnm2, Dynll1, Dynlt1b, Flna, Flnc, Klc1, Map2k2, Mapk3, Myl12b, Rhob, Skp1, Traf6, Tuba4a, Tubb2a, Txn1]
KEGG:05135	Yersinia infection	KEGG_25.05.2022	0.000859696	0.004970118	1.49924E-05	6.49673E-05	[-1]	Group11	9.70149231	13	4.239800759	[Actb, Actr3b, Arpc1b, Arpc3, Arpc5l, Map2k2, Mapk3, Pip5k1a, Rac2, Rac3, Traf6, Vav1, Wipf3]
KEGG:05205	Proteoglycans in cancer	KEGG_25.05.2022	0.000322211	0.002207744	1.49924E-05	6.49673E-05	[-1]	Group11	8.780488014	18	3.913662239	[Actb, Camk2b, Flna, Flnc, Gab1, Map2k2, Mapk3, Msn, Nras, Pdcd4, Plcg2, Ppp1cc, Prkcb, Ptpn6, Rras2, Stat3, Tiam1, Vav1]
KEGG:04012	ErbB signaling pathway	KEGG_25.05.2022	0.009266584	0.026786219	1.01053E-10	1.31369E-09	[-1]	Group12	9.523809433	8	4.224270353	[Camk2b, Gab1, Map2k2, Mapk3, Nras, Plcg2, Prkcb, Tgfa]
KEGG:04014	Ras signaling pathway	KEGG_25.05.2022	0.001795687	0.008102489	1.01053E-10	1.31369E-09	[-1]	Group12	7.659574509	18	3.368721927	[Gab1, Gnb5, Gng12, Gng4, Map2k2, Mapk3, Nras, Pla2g4a, Plcg2, Pld1, Prkcb, Rac2, Rac3, Rasgrp2, Rras2, Shoc2, Tgfa, Tiam1]
KEGG:04015	Rap1 signaling pathway	KEGG_25.05.2022	0.00406363	0.013921698	1.01053E-10	1.31369E-09	[-1]	Group12	7.476635456	16	3.300825206	[Actb, Adcy3, Adcy8, Apbb1ip, Gnas, Map2k2, Mapk3, Nras, Prkcb, Rac2, Rac3, Rap1gap, Rasgrp2, Tiam1, Vasp, Vav1]
KEGG:04020	Calcium signaling pathway	KEGG_25.05.2022	1.07735E-06	4.98276E-05	1.01053E-10	1.31369E-09	[-1]	Group12	10.41666698	25	4.348513599	[Adcy3, Adcy8, Atp2b1, Atp2b4, Camk1, Camk2b, Drd1, Ednrb, Gnas, Grm1, Htr4, Itpka, Mcu, Ntrk3, P2rx7, Pde1b, Phka1, Phkb, Plcd3, Plcg2, Prkcb, Ryr3, Slc25a5, Stim1, Vdac3]
KEGG:04022	cGMP-PKG signaling pathway	KEGG_25.05.2022	0.009954444	0.027902609	1.01053E-10	1.31369E-09	[-1]	Group12	7.514451027	13	3.3524006	[Adcy3, Adcy8, Atp2b1, Atp2b4, Ednrb, Gucy1b1, Map2k2, Mapk3, Mef2c, Ppp1cc, Slc25a5, Vasp, Vdac3]
KEGG:04024	cAMP signaling pathway	KEGG_25.05.2022	9.38129E-05	0.001239671	1.01053E-10	1.31369E-09	[-1]	Group12	9.090909004	20	3.960253456	[Adcy3, Adcy8, Atp2b1, Atp2b4, Camk2b, Crebbp, Drd1, Gnas, Htr4, Map2k2, Mapk3, Pde10a, Pde4a, Pld1, Ppp1cc, Rac2, Rac3, Rras2, Tiam1, Vav1]
KEGG:04062	Chemokine signaling pathway	KEGG_25.05.2022	0.000142202	0.001644208	1.01053E-10	1.31369E-09	[-1]	Group12	9.375	18	4.115397406	[Adcy3, Adcy8, Gnb5, Gng12, Gng4, Grk5, Jak3, Mapk3, Nras, Plcg2, Prkcb, Rac2, Rac3, Rasgrp2, Stat1, Stat3, Tiam1, Vav1]
KEGG:04066	HIF-1 signaling pathway	KEGG_25.05.2022	0.006810481	0.020998985	1.01053E-10	1.31369E-09	[-1]	Group12	8.771929741	10	3.791011856	[Camk2b, Crebbp, Eno2, Hk2, Map2k2, Mapk3, Plcg2, Prkcb, Rbx1, Stat3]
KEGG:04071	Sphingolipid signaling pathway	KEGG_25.05.2022	0.004258162	0.01432291	1.01053E-10	1.31369E-09	[-1]	Group12	8.870967865	11	3.872247824	[Bax, Map2k2, Mapk3, Nras, Pld1, Ppp2r2a, Prkcb, Rac2, Rac3, Sgpl1, Sgpp1]
KEGG:04072	Phospholipase D signaling pathway	KEGG_25.05.2022	0.000234124	0.001968773	1.01053E-10	1.31369E-09	[-1]	Group12	10.06711388	15	4.465252219	[Adcy3, Adcy8, Dgki, Dnm2, Gab1, Gnas, Grm1, Map2k2, Mapk3, Nras, Pip5k1a, Pla2g4a, Plcg2, Pld1, Rras2]
KEGG:04261	Adrenergic signaling in cardiomyocytes	KEGG_25.05.2022	0.011436814	0.03111486	1.01053E-10	1.31369E-09	[-1]	Group12	7.894736767	12	3.41191067	[Adcy3, Adcy8, Atp2b1, Atp2b4, Camk2b, Gnas, Mapk3, Myl4, Ppp1cc, Ppp2r2a, Scn1b, Tpm4]
KEGG:04360	Axon guidance	KEGG_25.05.2022	0.012952838	0.033281598	1.01053E-10	1.31369E-09	[-1]	Group12	7.182320595	13	3.185706648	[Camk2b, Efnb1, Mapk3, Myl12b, Ngef, Nras, Plcg2, Plxnc1, Rac2, Rac3, Sema7a, Srgap1, Ssh3]
KEGG:04370	VEGF signaling pathway	KEGG_25.05.2022	0.000895668	0.005021172	1.01053E-10	1.31369E-09	[-1]	Group12	13.79310322	8	6.117908788	[Map2k2, Mapk3, Nras, Pla2g4a, Plcg2, Prkcb, Rac2, Rac3]
KEGG:04371	Apelin signaling pathway	KEGG_25.05.2022	0.001057457	0.005589413	1.01053E-10	1.31369E-09	[-1]	Group12	9.489050865	13	4.118663594	[Adcy3, Adcy8, Gnb5, Gng12, Gng4, Map2k2, Mapk3, Mef2c, Myl4, Nras, Prkag1, Rras2, Ryr3]
KEGG:04510	Focal adhesion	KEGG_25.05.2022	0.005569688	0.018077057	1.01053E-10	1.31369E-09	[-1]	Group12	7.462686539	15	3.293676142	[Actb, Flna, Flnc, Mapk3, Myl12b, Parvb, Pip5k1a, Ppp1cc, Prkcb, Rac2, Rac3, Thbs4, Vasp, Vav1, Vcl]
KEGG:04520	Adherens junction	KEGG_25.05.2022	0.00081148	0.004842703	1.01053E-10	1.31369E-09	[-1]	Group12	12.67605591	9	4.339060309	[Actb, Crebbp, Ctnna3, Mapk3, Ptpn6, Rac2, Rac3, Sorbs1, Vcl]
KEGG:04540	Gap junction	KEGG_25.05.2022	4.36426E-05	0.000897098	1.01053E-10	1.31369E-09	[-1]	Group12	13.95348835	12	6.189047262	[Adcy3, Adcy8, Drd1, Gnas, Grm1, Gucy1b1, Map2k2, Mapk3, Nras, Prkcb, Tuba4a, Tubb2a]
KEGG:04611	Platelet activation	KEGG_25.05.2022	2.82884E-05	0.000747622	1.01053E-10	1.31369E-09	[-1]	Group12	12.0967741	15	5.280337942	[Actb, Adcy3, Adcy8, Apbb1ip, Gnas, Gucy1b1, Mapk3, Myl12b, Pla2g4a, Plcg2, Ppp1cc, Ptgs1, Rasgrp2, Stim1, Vasp]

A53T

ID	Term	Ontology Source	Term PValue	Term PValue Corrected with Benjamini-Hochberg	Group PValue	Group PValue Corrected with Benjamini-Hochberg	GOLevels	GOGroups	% Associated Genes	Nr. Genes	Rich Factor	Associated Genes Found
KEGG 03013	Nucleocytoplasmic transport	KEGG_25.05.2022	0.007255716	0.021767147	0.007255716	0.010883573	[-1]	Group1	5.932203293	7	2.609108159	[Ahctf1, Alyref, Ipo8, Nup188, Rae1, Sec13, Sumo2]
KEGG:04721	Synaptic vesicle cycle	KEGG_25.05.2022	0.003408465	0.013992647	0.003408465	0.007669047	[-1]	Group2	7.792207718	6	3.456221198	[Cplx1, Cplx3, Napa, Slc17a7, Stx1a, Stx2]
KEGG:03040	Spliceosome	KEGG_25.05.2022	0.004029652	0.015715642	0.004029652	0.007253374	[-1]	Group3	5.97014904	8	0.339883822	[Alyref, Bud31, Prpf40a, Sf3b4, Sf3b5, Snrpg, Snu13, Srsf2]
KEGG:04964	Proximal tubule bicarbonate reclamation	KEGG_25.05.2022	0.00071313	0.005056743	0.00071313	0.003209087	[-1]	Group4	18.18181801	4	8.064516129	[Atp1a1, Atp1a4, Pck2, Slc25a10]
KEGG:03060	Protein export	KEGG_25.05.2022	0.017292796	0.040873881	0.017292796	0.017292796	[-1]	Group5	10.34482765	3	3.168202765	[Oxa1l, Sec61a1, Sec61a2]
KEGG:04141	Protein processing in endoplasmic reticulum	KEGG_25.05.2022	0.017007153	0.041454937	0.017007153	0.019133048	[-1]	Group6	4.651162624	8	2.004738473	[Eif2ak4, Fbxo2, Preb, Rad23a, Sec13, Sec61a1, Sec61a2, Skp1]
KEGG:04146	Peroxisome	KEGG_25.05.2022	0.001227087	0.00683663	0.001227087	0.003681262	[-1]	Group7	8.13953495	7	3.568780126	[Acaa1a, Cat, Mvk, Pecr, Pex1, Pex13, Pex19]
KEGG:04020	Calcium signaling pathway	KEGG_25.05.2022	0.006144845	0.020839041	1.57204E-05	0.000141484	[-1]	Group8	4.583333492	11	1.913345984	[Atp2a2, Atp2b1, Atp2b2, Calm1, Camk2a, Camk2d, Plcb1, Ppp3ca, Ppp3cc, Ppp3r1, Slc8a1]
KEGG:04022	cGMP-PKG signaling pathway	KEGG_25.05.2022	2.4812E-05	0.000967666	1.57204E-05	0.000141484	[-1]	Group8	7.514451027	13	3.3524006	[Atp1a1, Atp1a4, Atp2a2, Atp2b1, Atp2b2, Calm1, Plcb1, Ppp3ca, Ppp3cc, Ppp3r1, Rock2, Slc8a1, Vasp]
KEGG:04024	cAMP signaling pathway	KEGG_25.05.2022	0.00934625	0.024300249	1.57204E-05	0.000141484	[-1]	Group8	4.545454502	10	1.980126728	[Atp1a1, Atp1a4, Atp2a2, Atp2b1, Atp2b2, Bdnf, Calm1, Camk2a, Camk2d, Rock2]
KEGG:04114	Oocyte meiosis	KEGG_25.05.2022	0.008288341	0.023088949	1.57204E-05	0.000141484	[-1]	Group8	5.785123825	7	2.524259114	[Calm1, Camk2a, Camk2d, Ppp3ca, Ppp3cc, Ppp3r1, Skp1]
KEGG:04260	Cardiac muscle contraction	KEGG_25.05.2022	0.006213468	0.020193772	1.57204E-05	0.000141484	[-1]	Group8	6.896551609	6	2.990213846	[Atp1a1, Atp1a4, Atp2a2, Cacnb3, Cox7a1, Slc8a1]
KEGG:04261	Adrenergic signaling in cardiomyocytes	KEGG_25.05.2022	3.15155E-05	0.000819403	1.57204E-05	0.000141484	[-1]	Group8	7.894736767	12	3.41191067	[Atp1a1, Atp1a4, Atp2a2, Atp2b1, Atp2b2, Cacnb3, Calm1, Camk2a, Camk2d, Plcb1, Scn4b, Slc8a1]
KEGG:04310	Wnt signaling pathway	KEGG_25.05.2022	0.005365369	0.019928515	1.57204E-05	0.000141484	[-1]	Group8	5.263157845	9	2.281105991	[Camk2a, Camk2d, Plcb1, Ppp3ca, Ppp3cc, Ppp3r1, Rock2, Skp1, Smad4]
KEGG:04360	Axon guidance	KEGG_25.05.2022	0.000170897	0.002665998	1.57204E-05	0.000141484	[-1]	Group8	6.629834175	12	2.94065229	[Camk2a, Camk2d, L1cam, Lrrc4c, Myl12b, Plxnc1, Ppp3ca, Ppp3cc, Ppp3r1, Rock2, Sema4a, Sema4f]
KEGG:04720	Long-term potentiation	KEGG_25.05.2022	0.000268783	0.002995015	1.57204E-05	0.000141484	[-1]	Group8	10.44776154	7	4.634087626	[Calm1, Camk2a, Camk2d, Plcb1, Ppp3ca, Ppp3cc, Ppp3r1]
KEGG:04724	Glutamatergic synapse	KEGG_25.05.2022	0.005751057	0.02039011	1.57204E-05	0.000141484	[-1]	Group8	6.194690228	7	2.653708299	[Glul, Homer2, Plcb1, Ppp3ca, Ppp3cc, Ppp3r1, Slc17a7]
KEGG:04911	Insulin secretion	KEGG_25.05.2022	0.000221853	0.002884091	1.57204E-05	0.000141484	[-1]	Group8	9.302325249	8	4.126031508	[Atp1a1, Atp1a4, Camk2a, Camk2d, Cck, Plcb1, Rims2, Stx1a]
KEGG:04921	Oxytocin signaling pathway	KEGG_25.05.2022	0.000669405	0.005801514	1.57204E-05	0.000141484	[-1]	Group8	6.5359478	10	2.880184332	[Cacnb3, Calm1, Camk2a, Camk2d, Plcb1, Ppp3ca, Ppp3cc, Ppp3r1, Prkaa1, Rock2]
KEGG:04922	Glucagon signaling pathway	KEGG_25.05.2022	4.21403E-06	0.000328694	1.57204E-05	0.000141484	[-1]	Group8	10.57692337	11	4.691377171	[Calm1, Camk2a, Camk2d, Gys1, Pck2, Plcb1, Ppp3ca, Ppp3cc, Ppp3r1, Ppp4c, Prkaa1]
KEGG:04924	Renin secretion	KEGG_25.05.2022	0.014776602	0.037179839	1.57204E-05	0.000141484	[-1]	Group8	6.578947544	5	2.918081494	[Calm1, Plcb1, Ppp3ca, Ppp3cc, Ppp3r1]
KEGG:04925	Aldosterone synthesis and secretion	KEGG_25.05.2022	0.000708565	0.005526808	1.57204E-05	0.000141484	[-1]	Group8	7.843137264	8	3.41191067	[Atp1a1, Atp1a4, Atp2b1, Atp2b2, Calm1, Camk2a, Camk2d, Plcb1]
KEGG:04961	Endocrine and other factor-regulated calcium reabsorption	KEGG_25.05.2022	0.001025006	0.006662538	1.57204E-05	0.000141484	[-1]	Group8	9.836065292	6	4.362771021	[Atp1a1, Atp1a4, Atp2b1, Atp2b2, Plcb1, Slc8a1]
KEGG:04970	Salivary secretion	KEGG_25.05.2022	0.001145266	0.006871596	1.57204E-05	0.000141484	[-1]	Group8	8.235294342	7	3.568780126	[Atp1a1, Atp1a4, Atp2b1, Atp2b2, Calm1, Plcb1, Slc12a2]
KEGG:04971	Gastric acid secretion	KEGG_25.05.2022	0.002986445	0.012941261	1.57204E-05	0.000141484	[-1]	Group8	8	6	3.548387097	[Atp1a1, Atp1a4, Calm1, Camk2a, Camk2d, Plcb1]
KEGG:04972	Pancreatic secretion	KEGG_25.05.2022	0.001466324	0.007624885	1.57204E-05	0.000141484	[-1]	Group8	7.017543793	8	3.085553997	[Atp1a1, Atp1a4, Atp2a2, Atp2b1, Atp2b2, Cck, Plcb1, Slc12a2]
KEGG:04978	Mineral absorption	KEGG_25.05.2022	6.73772E-05	0.001313854	1.57204E-05	0.000141484	[-1]	Group8	12.9629631	7	5.64516129	[Atp1a1, Atp1a4, Atp2b1, Atp2b2, Clcn2, Slc26a6, Slc8a1]
KEGG:05010	Alzheimer disease	KEGG_25.05.2022	0.019278992	0.044228274	1.57204E-05	0.000141484	[-1]	Group8	3.655352592	14	1.596318103	[Adam10, Apbb1, Atp2a2, Atp5e, Calm1, Cox7a1, Klc4, Ndufa12, Ndufa9, Plcb1, Ppp3ca, Ppp3cc, Ppp3r1, Rtn3]
KEGG:05014	Amyotrophic lateral sclerosis	KEGG_25.05.2022	0.00248968	0.012137189	1.57204E-05	0.000141484	[-1]	Group8	4.336043358	16	1.887439945	[Alyref, Atp5e, Cat, Ccs, Cox7a1, Dctn5, Klc4, Ndufa12, Ndufa9, Nefm, Nup188, Ppp3ca, Ppp3cc, Ppp3r1, Rae1, Sec13]
KEGG:05022	Pathways of neurodegeneration	KEGG_25.05.2022	0.002522963	0.011575946	1.57204E-05	0.000141484	[-1]	Group8	4.033970356	19	1.752062236	[Atp2a2, Atp5e, Bdnf, Calm1, Camk2a, Camk2d, Cat, Ccs, Cox7a1, Dctn5, Klc4, Ndufa12, Ndufa9, Nefm, Plcb1, Ppp3ca, Ppp3cc, Ppp3r1, Stx1a]
KEGG:05031	Amphetamine addiction	KEGG_25.05.2022	0.000322983	0.00314908	1.57204E-05	0.000141484	[-1]	Group8	10.14492798	7	4.499766246	[Calm1, Camk2a, Camk2d, Ppp3ca, Ppp3cc, Ppp3r1, Stx1a]
KEGG:05415	Diabetic cardiomyopathy	KEGG_25.05.2022	0.007056347	0.022015803	1.57204E-05	0.000141484	[-1]	Group8	4.739336491	10	2.044001784	[Atp2a2, Atp5e, Camk2a, Camk2d, Cox7a1, Gys1, Ndufa12, Ndufa9, Pdk3, Plcb1]
KEGG:05417	Lipid and atherosclerosis	KEGG_25.05.2022	0.008267113	0.023882771	1.57204E-05	0.000141484	[-1]	Group8	4.629629612	10	2.044001784	[Abca1, Calm1, Camk2a, Camk2d, Plcb1, Ppp3ca, Ppp3cc, Ppp3r1, Rock2, Stat3]

MitoPark

ID	Term	Ontology Source	Term PValue	Term PValue Corrected with Benjamini-Hochberg	Group PValue	Group PValue Corrected with Benjamini-Hochberg	GOLevels	GOGroups	% Associated Genes	Nr. Genes	Rich Factor	Associated Genes Found
KEGG:05231	Choline metabolism in cancer	KEGG_25.05.2022	0.013331912	0.041021269	0.013331912	0.015998295	[-1]	Group1	5.102040768	5	2.263001975	[Akt2, Lypla1, Pip5k1a, Slc22a4, Was]
KEGG:04520	Adherens junction	KEGG_25.05.2022	0.01890818	0.044489838	0.01890818	0.01890818	[-1]	Group2	5.633802891	4	1.928471248	[Iqgap1, Nectin1, Nlk, Was]
KEGG:04714	Thermogenesis	KEGG_25.05.2022	0.006011475	0.026717667	0.006011475	0.009017212	[-1]	Group3	3.896103859	9	1.677283817	[Arid1b, Atp5a1, Atp5l, Atp5o, Cox20, Cox7c, Cpt1a, Ndufa11, Ndufaf2]
KEGG:04721	Synaptic vesicle cycle	KEGG_25.05.2022	0.004920414	0.024602069	0.00029373	0.001762381	[-1]	Group4	6.493506432	5	2.880184332	[Ap2m1, Dnm1, Slc18a2, Slc6a3, Stx2]
KEGG:04728	Dopaminergic synapse	KEGG_25.05.2022	0.000707723	0.028308932	0.00029373	0.001762381	[-1]	Group4	5.925925732	8	2.590063574	[Akt2, Ddc, Gnb4, Gng11, Ppp1cb, Slc18a2, Slc6a3, Th]
KEGG:05012	Parkinson disease	KEGG_25.05.2022	0.01375203	0.039291516	0.00029373	0.001762381	[-1]	Group4	3.409090996	9	1.456910761	[Atp5a1, Atp5o, Cox7c, Ndufa11, Slc18a2, Slc25a4, Slc25a5, Slc6a3, Th]
KEGG:05030	Cocaine addiction	KEGG_25.05.2022	0.004871978	0.027839873	0.00029373	0.001762381	[-1]	Group4	8.333333015	4	3.696236559	[Ddc, Slc18a2, Slc6a3, Th]
KEGG:05031	Amphetamine addiction	KEGG_25.05.2022	0.003065574	0.030655744	0.00029373	0.001762381	[-1]	Group4	7.246376991	5	3.214118747	[Ddc, Ppp1cb, Slc18a2, Slc6a3, Th]
KEGG:04022	cGMP-PKG signaling pathway	KEGG_25.05.2022	0.003428735	0.027429881	0.000420254	0.001260763	[-1]	Group5	4.624277592	8	2.063015754	[Akt2, Atp1b1, Ppp1cb, Rock1, Slc25a4, Slc25a5, Slc8a2, Slc8a3]
KEGG:04260	Cardiac muscle contraction	KEGG_25.05.2022	0.001535278	0.030705551	0.000420254	0.001260763	[-1]	Group5	6.896551609	6	2.990213846	[Atp1b1, Cacng8, Cox7c, Slc8a2, Slc8a3, Slc9a1]
KEGG:04261	Adrenergic signaling in cardiomyocytes	KEGG_25.05.2022	0.006239993	0.024959974	0.000420254	0.001260763	[-1]	Group5	4.605263233	7	1.990281224	[Akt2, Atp1b1, Cacng8, Ppp1cb, Slc8a2, Slc8a3, Slc9a1]
KEGG:04371	Apelin signaling pathway	KEGG_25.05.2022	0.013994029	0.03731741	0.000420254	0.001260763	[-1]	Group5	4.379561901	6	1.900921659	[Akt2, Gnb4, Gng11, Slc8a2, Slc8a3, Slc9a1]
KEGG:04961	Endocrine and other factor-regulated calcium reabsorption	KEGG_25.05.2022	0.001777851	0.02370468	0.000420254	0.001260763	[-1]	Group5	8.196721077	5	3.635642517	[Ap2m1, Atp1b1, Dnm1, Slc8a2, Slc8a3]
KEGG:04978	Mineral absorption	KEGG_25.05.2022	0.007412614	0.02695496	0.000420254	0.001260763	[-1]	Group5	7.407407284	4	3.225806452	[Atp1b1, Hmox2, Slc8a2, Slc8a3]
KEGG:05012	Parkinson disease	KEGG_25.05.2022	0.01375203	0.039291516	0.000420254	0.001260763	[-1]	Group5	3.409090996	9	1.456910761	[Atp5a1, Atp5o, Cox7c, Ndufa11, Slc18a2, Slc25a4, Slc25a5, Slc6a3, Th]
KEGG:05030	Cocaine addiction	KEGG_25.05.2022	0.004871978	0.027839873	0.000420254	0.001260763	[-1]	Group5	8.333333015	4	3.696236559	[Ddc, Slc18a2, Slc6a3, Th]
KEGG:05208	Chemical carcinogenesis	KEGG_25.05.2022	0.014623557	0.036558893	0.000420254	0.001260763	[-1]	Group5	3.603603601	8	1.549514016	[Akt2, Atp5a1, Atp5o, Cox7c, Ephx1, Ndufa11, Slc25a4, Slc25a5]
KEGG:05415	Diabetic cardiomyopathy	KEGG_25.05.2022	0.011017553	0.036725178	0.000420254	0.001260763	[-1]	Group5	3.791469097	8	1.635201427	[Akt2, Atp5a1, Atp5o, Cox7c, Ndufa11, Ppp1cb, Slc25a4, Slc25a5]

KEGG:04650	Natural killer cell mediated cytotoxicity	KEGG_25.05.2022	0.010214157	0.028203271	1.01053E-10	1.31369E-09	[-1]	Group12	8.264462471	10	3.696236559	[H2-K1, Map2k2, Mapk3, Nras, Plcg2, Prkcb, Ptpn6, Rac2, Rac3, Vav1]
KEGG:04662	B cell receptor signaling pathway	KEGG_25.05.2022	0.002090776	0.0089952	1.01053E-10	1.31369E-09	[-1]	Group12	11.11111069	9	4.752304147	[Map2k2, Mapk3, Nras, Plcg2, Prkcb, Ptpn6, Rac2, Rac3, Vav1]
KEGG:04664	Fc epsilon RI signaling pathway	KEGG_25.05.2022	0.002104029	0.008846485	1.01053E-10	1.31369E-09	[-1]	Group12	12.12121201	8	5.376344086	[Map2k2, Mapk3, Nras, Pla2g4a, Plcg2, Rac2, Rac3, Vav1]
KEGG:04666	Fc gamma R-mediated phagocytosis	KEGG_25.05.2022	6.44238E-07	3.9728E-05	1.01053E-10	1.31369E-09	[-1]	Group12	16.30434799	15	7.077899794	[Actr3b, Arpc1b, Arpc3, Arpc5l, Dnm2, Mapk3, Marcks, Pip5k1a, Pla2g4a, Plcg2, Pld1, Prkcb, Rac2, Vasp, Vav1]
KEGG:04670	Leukocyte transendothelial migration	KEGG_25.05.2022	0.008625281	0.025736727	1.01053E-10	1.31369E-09	[-1]	Group12	8.474575996	10	3.727297371	[Actb, Ctnna3, Msn, Myl12b, Plcg2, Prkcb, Rac2, Vasp, Vav1, Vcl]
KEGG:04713	Circadian entrainment	KEGG_25.05.2022	0.000633465	0.00404107	1.01053E-10	1.31369E-09	[-1]	Group12	11.22449017	11	4.879032258	[Adcy3, Adcy8, Camk2b, Gnas, Gnb5, Gng12, Gng4, Gucy1b1, Mapk3, Prkcb, Ryr3]
KEGG:04720	Long-term potentiation	KEGG_25.05.2022	0.000526975	0.003481802	1.01053E-10	1.31369E-09	[-1]	Group12	13.43283558	9	5.958112662	[Adcy8, Camk2b, Crebbp, Grm1, Map2k2, Mapk3, Nras, Ppp1cc, Prkcb]
KEGG:04722	Neurotrophin signaling pathway	KEGG_25.05.2022	0.010214157	0.028203271	1.01053E-10	1.31369E-09	[-1]	Group12	8.264462471	10	3.66568915	[Bax, Camk2b, Gab1, Map2k2, Mapk3, Matk, Nras, Ntrk3, Plcg2, Traf6]
KEGG:04723	Retrograde endocannabinoid signaling	KEGG_25.05.2022	6.21676E-05	0.001150101	1.01053E-10	1.31369E-09	[-1]	Group12	10.81081104	16	4.608294931	[Adcy3, Adcy8, Gabra3, Gabra4, Gabrb2, Gabrd, Gnb5, Gng12, Gng4, Grm1, Mapk3, Ndufa10, Ndufa12, Ndufab1, Ndufb6, Prkcb]
KEGG:04724	Glutamatergic synapse	KEGG_25.05.2022	3.99484E-05	0.000923807	1.01053E-10	1.31369E-09	[-1]	Group12	12.38938046	14	5.307416598	[Adcy3, Adcy8, Gnas, Gnb5, Gng12, Gng4, Grik5, Grm1, Homer2, Mapk3, Pla2g4a, Pld1, Prkcb, Slc1a3]
KEGG:04725	Cholinergic synapse	KEGG_25.05.2022	0.001918006	0.008448359	1.01053E-10	1.31369E-09	[-1]	Group12	9.821428299	11	4.279852858	[Ache, Adcy3, Adcy8, Camk2b, Gnb5, Gng12, Gng4, Mapk3, Nras, Prkcb, Slc18a3]
KEGG:04726	Serotonergic synapse	KEGG_25.05.2022	0.000201509	0.001775197	1.01053E-10	1.31369E-09	[-1]	Group12	10.68702316	14	4.532611255	[Ddc, Gabrb2, Gnas, Gnb5, Gng12, Gng4, Htr4, Kcnd2, Mapk3, Nras, Pla2g4a, Prkcb, Ptgs1, Slc18a2]
KEGG:04727	GABAergic synapse	KEGG_25.05.2022	0.000273527	0.00210844	1.01053E-10	1.31369E-09	[-1]	Group12	12.35955048	11	5.36157391	[Adcy3, Adcy8, Gabra3, Gabra4, Gabrb2, Gabrd, Gnb5, Gng12, Gng4, Hap1, Prkcb]
KEGG:04728	Dopaminergic synapse	KEGG_25.05.2022	0.000276543	0.002046421	1.01053E-10	1.31369E-09	[-1]	Group12	10.37036991	14	4.532611255	[Camk2b, Comt, Ddc, Drd1, Gnas, Gnb5, Gng12, Gng4, Ppp1cc, Ppp2r2a, Prkcb, Slc18a2, Slc6a3, Th]
KEGG:04730	Long-term depression	KEGG_25.05.2022	0.001124907	0.005624536	1.01053E-10	1.31369E-09	[-1]	Group12	13.33333302	8	5.913978495	[Gnas, Grm1, Gucy1b1, Map2k2, Mapk3, Nras, Pla2g4a, Prkcb]
KEGG:04810	Regulation of actin cytoskeleton	KEGG_25.05.2022	9.38129E-05	0.001239671	1.01053E-10	1.31369E-09	[-1]	Group12	9.090909004	20	3.77487989	[Actb, Actr3b, Arpc1b, Arpc3, Arpc5l, Gng12, Map2k2, Mapk3, Msn, Myl12b, Nras, Pip5k1a, Ppp1cc, Rac2, Rac3, Rras2, Ssh3, Tiam1, Vav1, Vcl]
KEGG:04912	GnRH signaling pathway	KEGG_25.05.2022	0.001200186	0.005693189	1.01053E-10	1.31369E-09	[-1]	Group12	11.11111069	10	4.928315412	[Adcy3, Adcy8, Camk2b, Gnas, Map2k2, Mapk3, Nras, Pla2g4a, Pld1, Prkcb]
KEGG:04916	Melanogenesis	KEGG_25.05.2022	0.002665847	0.01072134	1.01053E-10	1.31369E-09	[-1]	Group12	10	10	4.435483871	[Adcy3, Adcy8, Camk2b, Crebbp, Ednrb, Gnas, Map2k2, Mapk3, Nras, Prkcb]
KEGG:04919	Thyroid hormone signaling pathway	KEGG_25.05.2022	0.003308581	0.012241751	1.01053E-10	1.31369E-09	[-1]	Group12	9.166666985	11	4.065860215	[Actb, Crebbp, Foxo1, Map2k2, Mapk3, Nras, Plcd3, Plcg2, Prkcb, Rcan1, Stat1]
KEGG:04921	Oxytocin signaling pathway	KEGG_25.05.2022	1.62917E-06	6.02792E-05	1.01053E-10	1.31369E-09	[-1]	Group12	12.41830063	19	5.47235023	[Actb, Adcy3, Adcy8, Camk1, Camk2b, Camkk2, Eef2k, Gnas, Gucy1b1, Map2k2, Mapk3, Mef2c, Nras, Pla2g4a, Ppp1cc, Prkag1, Prkcb, Rcan1, Ryr3]
KEGG:04928	Parathyroid hormone synthesis, secretion and action	KEGG_25.05.2022	0.013758779	0.034396946	1.01053E-10	1.31369E-09	[-1]	Group12	8.333333015	9	3.441323693	[Adcy3, Adcy8, Gnas, Mapk3, Mef2c, Naca, Pde4a, Pld1, Prkcb]
KEGG:04933	AGE-RAGE signaling pathway in diabetic complications	KEGG_25.05.2022	0.009085817	0.026680574	1.01053E-10	1.31369E-09	[-1]	Group12	8.910891533	9	3.95241137	[Bax, Foxo1, Mapk3, Nras, Plcd3, Plcg2, Prkcb, Stat1, Stat3]
KEGG:04935	Growth hormone synthesis, secretion and action	KEGG_25.05.2022	0.002537039	0.010430049	1.01053E-10	1.31369E-09	[-1]	Group12	9.482758522	11	4.170113041	[Adcy3, Adcy8, Crebbp, Gnas, Map2k2, Mapk3, Nras, Plcg2, Prkcb, Stat1, Stat3]
KEGG:04970	Salivary secretion	KEGG_25.05.2022	0.002919039	0.011020863	1.01053E-10	1.31369E-09	[-1]	Group12	10.5882349	9	4.588431591	[Adcy3, Adcy8, Atp2b1, Atp2b4, Cst3, Gnas, Gucy1b1, Prkcb, Ryr3]
KEGG:05032	Morphine addiction	KEGG_25.05.2022	9.14656E-08	1.69211E-05	1.01053E-10	1.31369E-09	[-1]	Group12	17.58241844	16	7.630939993	[Adcy3, Adcy8, Drd1, Gabra3, Gabra4, Gabrb2, Gabrd, Gnas, Gnb5, Gng12, Gng4, Grk5, Pde10a, Pde1b, Pde4a, Prkcb]
KEGG:05135	Yersinia infection	KEGG_25.05.2022	0.000859696	0.004970118	1.01053E-10	1.31369E-09	[-1]	Group12	9.70149231	13	4.239800759	[Actb, Actr3b, Arpc1b, Arpc3, Arpc5l, Map2k2, Mapk3, Pip5k1a, Rac2, Rac3, Traf6, Vav1, Wipf3]
KEGG:05161	Hepatitis B	KEGG_25.05.2022	0.015190203	0.036976151	1.01053E-10	1.31369E-09	[-1]	Group12	7.361963272	12	3.245476003	[Bax, Crebbp, Jak3, Map2k2, Mapk3, Nras, Pcna, Prkcb, Stat1, Stat3, Traf6, Vdac3]
KEGG:05170	Human immunodeficiency virus 1 infection	KEGG_25.05.2022	0.012321891	0.032106336	1.01053E-10	1.31369E-09	[-1]	Group12	6.666666508	16	2.93255132	[B2m, Bax, Gnb5, Gng12, Gng4, H2-K1, Map2k2, Mapk3, Nras, Plcg2, Prkcb, Rac2, Rac3, Rbx1, Skp1, Traf6]
KEGG:05200	Pathways in cancer	KEGG_25.05.2022	0.015434738	0.037083462	1.01053E-10	1.31369E-09	[-1]	Group12	5.524861813	30	2.423761678	[Adcy3, Adcy8, Bax, Camk2b, Crebbp, Ctnna3, Ednrb, Foxo1, Gnas, Gnb5, Gng12, Gng4, Jak3, Map2k2, Mapk3, Msh2, Nras, Plcg2, Pld1, Plekhg5, Prkcb, Rac2, Rac3, Rasgrp2, Rbx1, Skp1, Stat1, Stat3, Tgfa, Traf6]
KEGG:05205	Proteoglycans in cancer	KEGG_25.05.2022	0.000322211	0.002207744	1.01053E-10	1.31369E-09	[-1]	Group12	8.780488014	18	3.913662239	[Actb, Camk2b, Flna, Flnc, Gab1, Map2k2, Mapk3, Msn, Nras, Pdcd4, Plcg2, Ppp1cc, Prkcb, Ptpn6, Rras2, Stat3, Tiam1, Vav1]
KEGG:05210	Colorectal cancer	KEGG_25.05.2022	0.012114219	0.032016151	1.01053E-10	1.31369E-09	[-1]	Group12	9.090909004	8	4.032258065	[Bax, Map2k2, Mapk3, Msh2, Nras, Rac2, Rac3, Tgfa]
KEGG:05211	Renal cell carcinoma	KEGG_25.05.2022	0.009695549	0.027595025	1.01053E-10	1.31369E-09	[-1]	Group12	10.29411793	7	4.435483871	[Crebbp, Gab1, Map2k2, Mapk3, Nras, Rbx1, Tgfa]
KEGG:05212	Pancreatic cancer	KEGG_25.05.2022	0.005101919	0.016854553	1.01053E-10	1.31369E-09	[-1]	Group12	10.52631569	8	4.66893039	[Bax, Mapk3, Pld1, Rac2, Rac3, Stat1, Stat3, Tgfa]
KEGG:05214	Glioma	KEGG_25.05.2022	0.00109813	0.005643169	1.01053E-10	1.31369E-09	[-1]	Group12	12.16216183	9	5.322580645	[Bax, Camk1, Camk2b, Map2k2, Mapk3, Nras, Plcg2, Prkcb, Tgfa]
KEGG:05223	Non-small cell lung cancer	KEGG_25.05.2022	0.000899303	0.004893265	1.01053E-10	1.31369E-09	[-1]	Group12	12.5	9	5.544354839	[Bax, Jak3, Map2k2, Mapk3, Nras, Plcg2, Prkcb, Stat3, Tgfa]
KEGG:05231	Choline metabolism in cancer	KEGG_25.05.2022	0.000158881	0.001632947	1.01053E-10	1.31369E-09	[-1]	Group12	12.24489784	12	5.43120474	[Dgki, Gpcpd1, Map2k2, Mapk3, Nras, Pcyt1b, Pip5k1a, Pla2g4a, Pld1, Prkcb, Rac2, Rac3]

This table lists significantly enriched KEGG pathways. "MPTP" refers to the acute MPTP model, "A53T" to the 16 months A53T-α-synuclein model, and "MitoPark" to the 5 months MitoPark model.
