# Supplementary material for: Neurotoxicity, α-synuclein pathology, and mitochondrial dysfunction: A comparative study of different mouse models of Parkinson’s disease
Source: Neural Regen Res. 2025 Sep 29;21(10):5000–12. doi: 10.4103/NRR.NRR-D-25-00648 (PMC13568611; doi:10.4103/NRR.NRR-D-25-00648)
Supplement: Supplementary file 9 [file NRR-21-5000_Suppl8.pdf]

Additional Table 11. Pathway enrichment results from GSEA analysis of proteomic data in the striatum of three PD mouse models

MPTP

ID	Description	setSize	enrichment Score	NES	pvalue	p.adjust	qvalue	rank	leading_edge	core_enrichment
NABA_MATRISOME_ASSOCIATED	NABA_MATRISOME_ASSOCIATED	113	0.5895553	1.998751	1.838E-05	0.0151915	0.0147792	899	tags=35%, list=14%, signal=30%	S100a11/Serpinc1/Cxcl14/Tgfa/S100a4/Agt/Serpinf2/Serpina3n/Lgals1/S100a6/Bmp1/Hpx/C1qc/Mug1/Pamr1/Serpina1b/Itih3/Serpina3k/Ogfod1/Anxa4/Plxnc1/Plod3/Pik3ip1/Sdc3/Anxa2/Fgf13/C1qa/Ctss/Cst3/Htra1/S100b/Cstb/S100a10/Ctsl/C1qb/Adam9/Efn1/Sema4b/Serpini1
BOCHKIS_FOXA2_TARGETS	BOCHKIS_FOXA2_TARGETS	179	0.4947471	1.7810959	1.791E-05	0.0151915	0.0147792	717	tags=21%, list=11%, signal=19%	Serpinc1/Eif1ax/Fitl1/Fgb/Chac2/Pnpla2/Mapkbp1/Serpinf2/Cnr2/Serpina3n/Dhx8/Hpx/Stard5/Dhrs1/Urod/Phip2a/Fmo2/Mrt4/Mug1/Fahr/Psmg3/Chmp5/Serpina1b/Itpkc/Cyp4f14/Banf1/Dph7/Cd82/Serpina3k/Cd9/Abca1/Cdh2/Fdft1/Gpt/Pamr1/Hmgb2/Igfbp4/Tip2
DEMAGALHAES_AGING_UP	DEMAGALHAES_AGING_UP	32	0.768799	2.09557	3.836E-05	0.0221063	0.0215064	795	tags=53%, list=12%, signal=47%	Apod/Gfap/S100a4/C4b/S100a6/Vwf/Sgk1/Litaf/C1qc/Gbp2/Clu/Msn/Il33/C1qa/Ctss/B2m/C1qb
NABA_MATRISOME	NABA_MATRISOME	177	0.4960777	1.7835149	3.584E-05	0.0221063	0.0215064	834	tags=30%, list=13%, signal=27%	S100a11/Serpinc1/Cxcl14/Tgfa/Fgb/S100a4/Agt/Serpinf2/Serpina3n/Fgg/Lgals1/S100a6/Bmp1/Vwf/Fga/Hpx/C1qc/Mug1/Thbs4/Pamr1/Igfbp5/Sparc/Serpina1b/Hapln2/Itih3/Serpina3k/Ogfod1/Anxa4/Plxnc1/Tinagl1/Plod3/Pik3ip1/Sdc3/Anxa2/Vwa5b1/Fgf13/C1qa/Hapln3/Ctss/Col3/Cst3/Htra1/S100b/Igfbp4/Cstb/Lgi3/S100a10/Ctsl/Fbln1/C1qb/Adam9/Efn1
KEGG_COMPLEMENT_AND_COAGULATION_CASCADES	KEGG_COMPLEMENT_AND_COAGULATION_CASCADES	15	0.8572992	1.9883942	5.884E-05	0.0273195	0.0265781	620	tags=80%, list=9%, signal=73%	Serpinc1/Fgb/C4b/Serpinf2/Vwf/Fga/C1qc/Cfh/Serpina1b/C1qa/F3
WP_COMPLEMENT_AND_COAGULATION_CASCADES	WP_COMPLEMENT_AND_COAGULATION_CASCADES	16	0.8424734	1.9831593	9.759E-05	0.0389315	0.037875	620	tags=69%, list=9%, signal=62%	Serpinc1/Fgb/C4b/Serpinf2/Vwf/C1qc/Cfh/Serpina1b/Clu/C1qa/F3
LEE_AGING_CEREBELLUM_UP	LEE_AGING_CEREBELLUM_UP	42	0.6742676	1.9394056	0.0002279	0.073855	0.0718507	795	tags=36%, list=12%, signal=32%	Ifit1/Hmox1/Gfap/Agt/C4b/Lgals3bp/Nos3/C1qc/Irgm1/Cdk4/Hexb/C1qa/Ctss/B2m/C1qb
WP_COMPLEMENT_SYSTEM	WP_COMPLEMENT_SYSTEM	21	0.7786678	1.9435537	0.0002719	0.0772318	0.0751359	287	tags=33%, list=4%, signal=32%	Fgb/C4b/Fgg/Fga/Apoa1/Alb/Cfh
LEE_AGING_NEOCORTEX_UP	LEE_AGING_NEOCORTEX_UP	51	0.6390082	1.904652	0.000283	0.0772318	0.0751359	940	tags=35%, list=14%, signal=30%	Apod/Gfap/C4b/Vim/Lgals3bp/Lat/C1qc/Itih3/Kcna1/Cd9/C1qa/Ctss/Ptgs1/S100a10/B2m/Hsd17b12/Sema4b/Sec23b
ZWANG_EGF_PERSISTENTLY_UP	ZWANG_EGF_PERSISTENTLY_UP	19	0.7759123	1.895811	0.0005646	0.121993	0.1186824	237	tags=16%, list=4%, signal=15%	Pus10/Ifit3/Ugdh
NABA_SECRETED_FACTORS	NABA_SECRETED_FACTORS	27	0.7161375	1.8860796	0.0006924	0.1298006	0.1262781	757	tags=37%, list=12%, signal=33%	S100a11/Cxcl14/Tgfa/S100a4/S100a6/Bmp1/Pik3ip1/Fgf13/S100b/S100a10
WP_NICOTINE_EFFECT_ON_DOPAMINERGIC_NEURONS	WP_NICOTINE_EFFECT_ON_DOPAMINERGIC_NEURONS	12	-0.843816	-1.884352	0.0008943	0.1531276	0.1489721	43	tags=33%, list=1%, signal=33%	Ppp1r1b/Slc18a2/Ddc/Th
HECKER_IFNB1_TARGETS	HECKER_IFNB1_TARGETS	18	0.7731446	1.8678137	0.0009153	0.1531276	0.1489721	795	tags=56%, list=12%, signal=49%	Ifit3/Lgals3bp/C1qc/Parp12/Myl9/Stat1/Clu/C1qa/Cmtm5/C1qb
REACTOME_INTEGRIN_SIGNALING	REACTOME_INTEGRIN_SIGNALING	18	0.7336385	1.8686343	0.0009857	0.1573117	0.1530426	114	tags=22%, list=2%, signal=21%	Rapgef3/Fgb/Fgg/Vwf/Fga
WUNDER_INFLAMMATORY_RESPONSE_AND_CHOLESTEROL_UP	WUNDER_INFLAMMATORY_RESPONSE_AND_CHOLESTEROL_UP	21	0.747025	1.8645735	0.0010877	0.1659023	0.1614001	782	tags=48%, list=12%, signal=42%	Ctrb1/Casp7/Gbp2/Serpina1b/Stat1/H2-D1/Ctss/Aif1/Psme2/B2m
WP_DOPAMINERGIC_NEUROGENESIS	WP_DOPAMINERGIC_NEUROGENESIS	5	-0.949319	-1.731985	0.0006167	0.1230093	0.1196711	43	tags=80%, list=1%, signal=80%	Slc18a2/Ddc/Slc6a3/Th
REACTOME_FORMATION_OF_THE_CORNIFIED_ENVELOPE	REACTOME_FORMATION_OF_THE_CORNIFIED_ENVELOPE	16	-0.893135	-2.12791	2.051E-05	0.0151915	0.0147792	307	tags=56%, list=5%, signal=54%	Ppl/Krt2/Sprr1a/Krt1/Krt18/Krt76/Krt5/Krt6a/Krt13
REACTOME_KERATINIZATION	REACTOME_KERATINIZATION	16	-0.893135	-2.12791	2.051E-05	0.0151915	0.0147792	307	tags=56%, list=5%, signal=54%	Ppl/Krt2/Sprr1a/Krt1/Krt18/Krt76/Krt5/Krt6a/Krt13
WAKABAYASHI_ADIPOGENESIS_PPARG_BOUND_36HR	WAKABAYASHI_ADIPOGENESIS_PPARG_BOUND_36HR	17	0.845726	2.0181053	1.948E-05	0.0151915	0.0147792	503	tags=41%, list=8%, signal=38%	Pus10/Dynlrb1/Lgals3bp/Mef2c/Cuedc1/Disp2/Plod3
LI_INDUCED_T_TO_NATURAL_KILLER_UP	LI_INDUCED_T_TO_NATURAL_KILLER_UP	112	0.5609313	1.8997063	1.837E-05	0.0151915	0.0147792	787	tags=30%, list=12%, signal=27%	S100a11/Hk2/Ifit3/S100a4/Prr7/Serpina3n/Lgals1/Vim/S100a6/Gpr34/Lgals3bp/Sgk1/Litaf/Glipr2/Selenos/Ifitm3/Gbp2/Prrm2/Plcg2/Bscl2/Bag3/Fcer1g/Mthfd1l/Mindy1/Serpina3k/Cd9/Syt1/Anxa2/Scd1/Lmna/Cstb/S100a10/Ctsl/Gng2
REACTOME_COMMON_PATHWAY_OF_FIBRIN_CLOT_FORMATION	REACTOME_COMMON_PATHWAY_OF_FIBRIN_CLOT_FORMATION	5	0.9820247	1.7830782	2.011E-05	0.0151915	0.0147792	114	tags=80%, list=2%, signal=79%	Serpinc1/Fgb/Fgg/Fga
RICKMAN_TUMOR_DIFFERENTIATED_WELL_VS_MODERATELY_DN	RICKMAN_TUMOR_DIFFERENTIATED_WELL_VS_MODERATELY_DN	41	-0.681186	-1.981372	6.322E-05	0.0273195	0.0265781	607	tags=32%, list=9%, signal=29%	Sqor/Numb/Arpc5l/Ccdc85c/Nocd/Cdc42ep2/Ajap1/Lrfn4/Impa2/Rras2/Dmkn/Krt6a/Dusp14
GOLDRATH_ANTIGEN_RESPONSE	GOLDRATH_ANTIGEN_RESPONSE	124	0.5365594	1.8431221	5.487E-05	0.0273195	0.0265781	757	tags=27%, list=12%, signal=25%	S100a11/Eif1ax/S100a4/Nudt4/Serpina3n/Rora/Lgals1/S100a6/Slc4a1/Dhrs1/Litaf/Mt2/Ifitm3/Unc119/Plcg2/Bag3/Serpina3k/Anxa4/Anxa2/Casp3/Scpep1/Fen1/Tpm4/Ald/Cst3/Ybx3/Hmgb2/Vcl/Hspa2/Lmna/Serinc3/Pcna/Sh3bgrl/S100a10
MARKEY_RB1_ACUTE_LOF_DN	MARKEY_RB1_ACUTE_LOF_DN	92	0.5611228	1.8453206	0.0001295	0.0479759	0.0466739	870	tags=35%, list=13%, signal=31%	Ifit1/Cxcl14/Apod/Ifit3/Ube2a/Tor1aip2/Lgals3bp/Retreg1/C1qc/Mt2/Nckap1/Csf1r/Gbp2/Rac2/Igfbp5/Npy/Vav1/Ptpn6/Irgm1/Stat1/H2-D1/Scpl1/Anxa4/Abca1/Lcp1/Rab8b/Pla2g7/Psme2/Gbp7/C1qb/Gabra/Bid
WP_NETWORK_MAP_OF_SARSCOV2_SIGNALING_PATHWAY	WP_NETWORK_MAP_OF_SARSCOV2_SIGNALING_PATHWAY	69	0.6007495	1.8852488	0.0002055	0.0710323	0.0691046	547	tags=26%, list=8%, signal=24%	Apod/Fgb/Apoa2/Agt/Fgg/Lgals3bp/Parp2/Fga/Apoa1/Alb/Mavs/Ifitm3/Cfh/Ptgs6/Stat1/Itih4/Il33/Casp3
REACTOME_PLATELET_ACTIVATION_SIGNALING_AND_AGGREGATION	REACTOME_PLATELET_ACTIVATION_SIGNALING_AND_AGGREGATION	156	0.4779781	1.8306969	0.0002706	0.0772318	0.0751359	619	tags=24%, list=9%, signal=22%	Rapgef3/Fgb/Serpinf2/Serpina3n/Fgg/Lgals3bp/Vwf/Fga/Lhfpl2/Apoa1/Alb/Gna14/Gng4/Vav3/Stxbp2/Rac2/Vav1/Selenop/Ptpn6/Sparc/Plcg2/Serpina1b/Prkch/Lamp2/Clu/Itih3/Pdpn/Fcer1g/Serpina3k/Abcc4/Cd9/Itpr2/Tagln2/Gng12/P2ry1/Rab27b/Gng5
REACTOME_RESPONSE_TO_ELEVATED_PLATELET_CYTOSOLIC_CA2	REACTOME_RESPONSE_TO_ELEVATED_PLATELET_CYTOSOLIC_CA2	70	0.5882009	1.8500346	0.0003549	0.0920311	0.0895336	681	tags=33%, list=10%, signal=30%	Fgb/Serpinf2/Serpina3n/Fgg/Lgals3bp/Vwf/Fga/Lhfpl2/Apoa1/Alb/Stxbp2/Selenop/Sparc/Serpina1b/Lamp2/Clu/Itih3/Serpina3k/Abcc4/Cd9/Tagln2/Rab27b/Vcl
CAIRO_LIVER_DEVELOPMENT_DN	CAIRO_LIVER_DEVELOPMENT_DN	89	0.5414533	1.7710014	0.000426	0.105201	0.102346	589	tags=21%, list=9%, signal=20%	Gc/Serpinc1/Hmox1/Fgb/Agt/Serpinf2/Fgg/Fga/Lcat/Ltf/Fahr/Cfh/Serpina1b/Itih3/Gm2a/Lcp1/Gnk5/C1qa/Entpd5
TAKADA_GASTRIC_CANCER_COPY_NUMBER_DN	TAKADA_GASTRIC_CANCER_COPY_NUMBER_DN	13	0.8343278	1.877196	0.0005111	0.1152367	0.1121094	32	tags=15%, list=0%, signal=15%	Pum3/Rbbt2
MCLACHLAN_DENTAL_CARIES_UP	MCLACHLAN_DENTAL_CARIES_UP	57	0.6093188	1.8508062	0.0005074	0.1152367	0.1121094	1093	tags=44%, list=17%, signal=37%	Eif1ax/C4b/Serpina3n/Mt2/Thbs4/Csf1r/Rac2/Serpina1b/Fcer1g/Serpina3k/Abca1/Lcp1/C1qa/Ctss/Tnfaip8/Slc7a11/Igfbp4/Pla2g7/Pecam1/C1qb/Rab31/Arpc1b/Apoe/Sod2/S100a9
REACTOME_FORMATION_OF_FIBRIN_CLOT_CLOTTING_CASCADE	REACTOME_FORMATION_OF_FIBRIN_CLOT_CLOTTING_CASCADE	9	0.884425	1.8326328	0.0006151	0.1230093	0.1196711	114	tags=56%, list=2%, signal=55%	Serpinc1/Fgb/Fgg/Vwf/Fga
WP_BLOOD_CLOTTING_CASCADE	WP_BLOOD_CLOTTING_CASCADE	6	0.9317557	1.7604837	0.0007008	0.1298006	0.1262781	114	tags=83%, list=2%, signal=82%	Fgb/Serpinf2/Fgg/Vwf/Fga
EBAUER_TARGETS_OF_PAX3_FOXO1_FUSION_DN	EBAUER_TARGETS_OF_PAX3_FOXO1_FUSION_DN	23	-0.735546	-1.898511	0.0008288	0.1482078	0.1441858	19	tags=22%, list=0%, signal=22%	Bmerb1/Ubxn4/Aktip/Dzip3/Zfyve16
HSIAO_LIVER_SPECIFIC_GENES	HSIAO_LIVER_SPECIFIC_GENES	84	0.5358441	1.7374982	0.001001	0.1573117	0.1530426	633	tags=30%, list=10%, signal=27%	Serpinc1/Fgb/Apoa2/Agt/C4b/Serpinf2/Serpina3n/Fgg/Fga/Hpx/Apoa1/Alb/Lcat/Mug1/Fahr/Cfh/Selenop/Serpina1b/Cyp4f14/Lamp2/Itih3/Serpina3k/Cdh2/Rgl2/Gpt

"MPTP" refers to the acute MPTP model, "A53T" to the 16 months A53T-α-synuclein model, and "MitoPark" to the 5 months MitoPark model.

A53T

ID	Description	setSize	enrichment Score	NES	pvalue	p.adjust	qvalue	rank	leading_edge	core_enrichment
REACTOME_SIGNALING_BY_PDGF	REACTOME_SIGNALING_BY_PDGF	28	0.8593775	2.0418691	2.117E-05	0.0282492	0.0278495	114	tags=18%, list=2%, signal=18%	Col6a2/Thbs1/Col6a1/Ptpn12/Col4a2
REACTOME_FORMATION_OF_THE_CORNIFIED_ENVELOPE	REACTOME_FORMATION_OF_THE_CORNIFIED_ENVELOPE	19	-0.908712	-1.999039	1.902E-05	0.0282492	0.0278495	357	tags=68%, list=5%, signal=65%	Krt5/Krt79/Krt10/Krt1/Krt18/Krt76/Krt16/Krt17/Krt6a/Krt73/Pkp2/Krt32/Krt8
REACTOME_KERATINIZATION	REACTOME_KERATINIZATION	19	-0.908712	-1.999039	1.902E-05	0.0282492	0.0278495	357	tags=68%, list=5%, signal=65%	Krt5/Krt79/Krt10/Krt1/Krt18/Krt76/Krt16/Krt17/Krt6a/Krt73/Pkp2/Krt32/Krt8
HSIAO_LIVER_SPECIFIC_GENES	HSIAO_LIVER_SPECIFIC_GENES	108	0.6659842	1.9800268	2.159E-05	0.0282492	0.0278495	575	tags=35%, list=9%, signal=33%	F10/Cyp2a5/Cpb2/Afm/Serpind1/Serping1/Apob/Orm1/Itih4/Apoh/Itih2/Hp/Serpinf2/Serpinf1/Apoc3/Rbp4/Hrg/Apcs/Serpina6/C3/Cthrc/Hpx/Itih3/Gc/Serpinc1/Klkb1/C4b/Alb/Apoa2/Ahsg/Serpinc1/Cfb/Msmo1/Apoa1/F2/Vtn/Rbm4/Cp
BROWNE_HCMV_INFECTION_30MIN_UP	BROWNE_HCMV_INFECTION_30MIN_UP	23	0.8501119	1.9514427	6.323E-05	0.0551434	0.0543631	317	tags=39%, list=5%, signal=37%	Col6a2/Thbs1/Col6a1/Gpx3/Socs3/Pdyn/Alb/St8sia3/Gc
REACTOME_FOLDING_OF_ACTIN_BY_CCT_TRIC	REACTOME_FOLDING_OF_ACTIN_BY_CCT_TRIC	10	0.9416876	1.8746674	6.266E-05	0.0551434	0.0543631	3	tags=10%, list=0%, signal=10%	Cct6b
NABA_ECM_REGULATORS	NABA_ECM_REGULATORS	57	0.69184	1.8594191	0.0001487	0.1085009	0.1069655	467	tags=33%, list=7%, signal=31%	F10/Plod3/A2m/Serpind1/Serping1/Itih4/Itih2/Serpinf2/Serpinf1/Hrg/Serpina6/Fam20b/Itih3/Ctsl/Mug1/Timp2/Serpinc1/Ctsh/Adam15
HOFMANN_MYELODYSPLASTIC_SYNDROM_HIGH_RISK_UP	HOFMANN_MYELODYSPLASTIC_SYNDROM_HIGH_RISK_UP	6	0.9661388	1.7596326	0.0001659	0.1085009	0.1069655	50	tags=33%, list=1%, signal=33%	Elk1/Coil
NABA_MATRISOME	NABA_MATRISOME	191	0.5183821	1.6623217	0.0002637	0.1533076	0.1511381	560	tags=20%, list=8%, signal=19%	F10/Col6a2/Thbs1/Col12a1/Col6a1/Plod3/A2m/Dcn/Serpind1/Serping1/Bdnf/Itih4/Col4a2/Itih2/Serpinf2/Serpinf1/Hrg/Serpina6/Sdc2/Crhbp/Hpx/Fam20b/Sema4f/Itih3/Ctsf/Mug1/Fgb/Cbln1/Timp2/Efn1/Serpinc1/Ctsh/Megf9/Adam15/Plxnc1/F2/Vtn/Plxdc1/Fn1
AMIT_SERUM_RESPONSE_120_MCF10A	AMIT_SERUM_RESPONSE_120_MCF10A	16	-0.877364	-1.874084	0.0003047	0.1594242	0.1571682	258	tags=25%, list=4%, signal=24%	Krt10/Sowahc/Rybp/Cd55b
CUI_TCF21_TARGETS_UP	CUI_TCF21_TARGETS_UP	20	0.8397442	1.8830385	0.0003997	0.1743196	0.1718528	157	tags=25%, list=2%, signal=24%	Col6a2/Col6a1/Dcn/Cyp7b1/Serpinf1
JACKSON_DNMT1_TARGETS_DN	JACKSON_DNMT1_TARGETS_DN	13	0.8927489	1.8567384	0.000378	0.1743196	0.1718528	279	tags=31%, list=4%, signal=30%	Col6a2/Col6a1/Peg3/Pold1

MitoPark

ID	Description	setSize	enrichment Score	NES	pvalue	p.adjust	qvalue	rank	leading_edge	core_enrichment
NABA_ECM_REGULATORS	NABA_ECM_REGULATORS	57	-0.835086	-2.112077	1.673E-05	0.0041033	0.0039082	254	tags=35%, list=4%, signal=34%	Cpn2/Ctsh/F10/Serpina3k/Serpinf1/F2/Mug1/Itih4/Itih2/Plg/Serpina1b/Serpinc1/Serpina1e/Serpind1/Serpinf2/Serpina6/A2m/Serping1/Serpina3n/Hrg
KEGG_COMPLEMENT_AND_COAGULATION_CASCADES	KEGG_COMPLEMENT_AND_COAGULATION_CASCADES	25	-0.91344	-2.037039	1.698E-05	0.0041033	0.0039082	336	tags=76%, list=5%, signal=72%	Cfh/C4b/F10/Fga/F2/C3/Fgb/Fgg/Plg/Serpina1b/Serpinc1/Serpina1e/Klkb1/Serpind1/Serpinf2/Cpb2/A2m/Serping1/Cfb
REACTOME_REGULATION_OF_INSULIN_LIKE_GROWTH_FACTOR_IGF_TRANSPORT_AND_UPTAKE_BY_INSULIN_LIKE_GROWTH_FACTOR_BINDING_PROTEINS_IGFBPS	REACTOME_REGULATION_OF_INSULIN_LIKE_GROWTH_FACTOR_IGF_TRANSPORT_AND_UPTAKE_BY_INSULIN_LIKE_GROWTH_FACTOR_BINDING_PROTEINS_IGFBPS	56	-0.800929	-2.020421	1.673E-05	0.0041033	0.0039082	425	tags=39%, list=6%, signal=37%	Cp/Fn1/Tgoln1/Mbtps1/C4b/Igfbp7/Lamb2/Fga/Alb/Ahsg/F2/C3/Fgg/Apoa1/Itih2/Apoa2/Plg/Serpina1b/Serpinc1/Serpina1e/Serpind1/Apob
WP_COMPLEMENT_AND_COAGULATION_CASCADES	WP_COMPLEMENT_AND_COAGULATION_CASCADES	25	-0.898872	-2.004552	1.698E-05	0.0041033	0.0039082	336	tags=64%, list=5%, signal=61%	Cfh/C4b/F10/F2/C3/Fgb/Plg/Serpina1b/Serpinc1/Serpina1e/Klkb1/Serpind1/Serpinf2/Cpb2/Serping1/Cfb
NABA_MATRISOME_ASSOCIATED	NABA_MATRISOME_ASSOCIATED	134	-0.658646	-1.881877	1.584E-05	0.0041033	0.0039082	495	tags=23%, list=7%, signal=22%	Fam20b/C1qa/Nrg3/Sema6d/Fgf12/Fgf13/Il18/Cpn2/Ctsh/F10/Serpina3k/Serpinf1/F2/Hpx/Mug1/Itih4/Itih2/Plg/Serpina1b/Serpinc1/Serpina1e/S100a8/Serpind1/Serpinf2/Serpina6/A2m/Serping1/Serpina3n/Il17f/S100a9/Hrg
REACTOME_SCAVENGING_OF_HEME_FROM_PLASMA	REACTOME_SCAVENGING_OF_HEME_FROM_PLASMA	7	-0.97916	-1.784545	1.725E-05	0.0041033	0.0039082	125	tags=86%, list=2%, signal=84%	Alb/Hpx/Apoa1/Igkv12-41/Hp/Ighv1-62-3
REACTOME_INNATE_IMMUNE_SYSTEM	REACTOME_INNATE_IMMUNE_SYSTEM	521	-0.517435	-1.698044	1.366E-05	0.0041033	0.0039082	361	tags=10%, list=5%, signal=10%	Myd88/Rhof/Isg15/Iqgap2/Ubb/Cfh/C4b/Cmtm6/Birc2/Ptpn6/Lgmn/Irak1/Stom/Mef2c/Dgat1/Cpn2/Atp6v0a2/Ttr/B2m/Ctsh/Atp11b/Fga/Ypel5/Fcer1g/Serpina3k/Ahsg/Polr3f/F2/C3/H2-Q10/Orm1/Rab37/Fgb/Fgg/Serpina1b/Ighg1/Ncf1/Serpina1e/Cd93/S100a8/Igkv12-41/Hp/Ighg2b/Lyz2/Cpb2/Was/Serping1/Cfb/Serpina3n/Saa1/S100a9/Ighv1-62-3/Apob
WP_SELENIUM_MICRONUTRIENT_NETWORK	WP_SELENIUM_MICRONUTRIENT_NETWORK	47	-0.812054	-1.993747	3.361E-05	0.0062012	0.0059064	144	tags=23%, list=2%, signal=23%	Fga/Serpina3k/Alb/F2/Fgb/Fgg/Apoa1/Plg/Serpina3n/Saa1/Apob
REACTOME_PLASMA_LIPOPROTEIN_ASSEMBLY_REMODELING_AND_CLEARANCE	REACTOME_PLASMA_LIPOPROTEIN_ASSEMBLY_REMODELING_AND_CLEARANCE	35	-0.838685	-1.969602	3.381E-05	0.0062012	0.0059064	394	tags=34%, list=6%, signal=32%	Lmf1/Mbtps1/Lcat/Apoc1/Alb/Apoa4/Apoa1/Apoa2/Plg/A2m/Apoc3/Apob
REACTOME_COMPLEMENT_CASCADE	REACTOME_COMPLEMENT_CASCADE	20	-0.883776	-1.905356	5.082E-05	0.0078404	0.0074676	336	tags=60%, list=5%, signal=57%	Cfh/C4b/Cpn2/F2/C3/Ighg1/Igkv12-41/Ighg2b/Cpb2/Serping1/Cfb/Ighv1-62-3
KEGG_HUNTINGTONS_DISEASE	KEGG_HUNTINGTONS_DISEASE	112	-0.668722	-1.865277	4.824E-05	0.0078404	0.0074676	1294	tags=56%, list=19%, signal=46%	Ndufs8/Ndufb8/Sdhc/Plcb3/Atp5pb/Ap2m1/Grin2b/Ndufa4/Uqcrfs1/Cox6b1/Cox7a1/Uqcr10/Slc25a5/Grin1/Uqcrb/Slc25a4/Cox7a2l/Ndufs4/Ndufs3/Ndufa5/Tgm2/Crebbp/Ndufc2/Ndufs7/Uqcrc2/Cyc1/Ndufb10/Ndufs6/Ndufs5/Ndufa10/Uqcrh/Ndufa6/Ndufb1/Ndufa9/Ndufb5/Cox4i1/Ndufs1/Ndufv2/Ndufs2/Ndufa8/Ndufv1/Uqcrq/Ndufa2/Vdac2/Vdac1/Ndufb2/Ndufv3/Ndufb6/Vdac3/Cox6c/Ndufab1/Cox5b/Ndufa7/Ndufb7/Cox5a/Ndufb9/Ndufb4/Ndufa1/Cox6a1/Ndufb3/Cox7c/Tbpl1/Cox7b
REACTOME_REGULATION_OF_TLR_BY_ENDOGENOUS_LIGAND	REACTOME_REGULATION_OF_TLR_BY_ENDOGENOUS_LIGAND	9	-0.959467	-1.828563	6.833E-05	0.0095612	0.0091067	144	tags=67%, list=2%, signal=65%	Fga/Fgb/Fgg/S100a8/S100a9/Apob
VANASSE_BCL2_TARGETS_UP	VANASSE_BCL2_TARGETS_UP	16	-0.902486	-1.88186	0.0001018	0.0133155	0.0126825	120	tags=44%, list=2%, signal=43%	Cwc22/Ighm/Apoa2/Ighg1/Ighg2b/Serping1/Cfb
REACTOME_AMYLOID_FIBER_FORMATION	REACTOME_AMYLOID_FIBER_FORMATION	32	-0.818371	-1.895032	0.0001863	0.0216652	0.0206352	340	tags=31%, list=5%, signal=30%	Ubb/Gga2/Ttr/B2m/Apcs/Fga/Apoa4/Apoa1/Lyz2/Saa1
KEGG_ALZHEIMERS_DISEASE	KEGG_ALZHEIMERS_DISEASE	107	-0.659827	-1.828394	0.0001938	0.0220456	0.0209975	1294	tags=54%, list=19%, signal=44%	Ndufs8/Ndufb8/Sdhc/Plcb3/Ncstn/Atp5pb/Grin2b/Ndufa4/Uqcrfs1/Cox6b1/Cox7a1/Uqcr10/Grin1/Uqcrb/Calm1/Cox7a2l/Ndufs4/Ndufs3/Ndufa5/Ndufc2/Ndufs7/Uqcrc2/Cyc1/Ndufb10/Ndufs6/Ndufs5/Ndufa10/Uqcrh/Atp2a2/Ndufa6/Ndufb1/Ndufa9/Ndufb
LENAOUR_DENDRITIC_CELL_MATURATION_DN	LENAOUR_DENDRITIC_CELL_MATURATION_DN	61	-0.723275	-1.849233	0.0002166	0.0241213	0.0229746	477	tags=23%, list=7%, signal=22%	Gla/Csf1r/Isg15/Lgmn/Fcer1g/Serpinf1/C3/Serpina1b/Ncf1/Serpina1e/S100a8/Lyz2/Atp2a3/S100a9
PID_SYNDECAN_1_PATHWAY	PID_SYNDECAN_1_PATHWAY	12	0.9189384	1.876516	0.0002914	0.0287685	0.0274008	54	tags=25%, list=1%, signal=25%	Col6a1/Col6a2/Col1a1
KEGG_ONE_CARBON_POOL_BY_FOLATE	KEGG_ONE_CARBON_POOL_BY_FOLATE	12	0.9157899	1.8700867	0.0002914	0.0287685	0.0274008	102	tags=25%, list=2%, signal=25%	Mthfd2l/Shmt1/Dhfr
WP_LEPTININSULIN_SIGNALING_OVERLAP	WP_LEPTININSULIN_SIGNALING_OVERLAP	7	0.9558896	1.8106386	0.0003093	0.0299076	0.0284857	4	tags=14%, list=0%, signal=14%	Socs3
REACTOME_NCAM_SIGNALING_FOR_NEURITE_OUT_GROWTH	REACTOME_NCAM_SIGNALING_FOR_NEURITE_OUT_GROWTH	32	0.7897917	1.8903893	0.000415	0.0381008	0.0362894	44	tags=9%, list=1%, signal=9%	Col6a1/Col6a2/Col4a2
REACTOME_METABOLISM_OF_FAT_SOLUBLE_VITAMINS	REACTOME_METABOLISM_OF_FAT_SOLUBLE_VITAMINS	23	-0.843085	-1.855841	0.0004415	0.0391609	0.0372991	365	tags=39%, list=5%, signal=37%	Retsat/Ttr/Vkorc1l1/Akr1c6/Apoa4/Apoa1/Apoa2/Apoc3/Apob
WP_NUCLEAR_RECEPTORS_METAPATHWAY	WP_NUCLEAR_RECEPTORS_METAPATHWAY	114	-0.623553	-1.743526	0.000594	0.0457107	0.0435375	506	tags=17%, list=8%, signal=16%	Slc6a1/Gstm2/Slc6a15/Slc6a5/Fgf13/Birc2/Mgst3/Stom/Myof/Slc6a2/Jund/Rgs2/Apoa1/Apoa2/Serpina1b/Serpina1e/Slc6a3/Cyp2a5/Apoc3
RASHI_RESPONSE_TO_IONIZING_RADIATION_6	RASHI_RESPONSE_TO_IONIZING_RADIATION_6	30	-0.804696	-1.84327	0.0006623	0.0502273	0.0478393	311	tags=33%, list=5%, signal=32%	Ddx18/Gbp2/Ighm/Ifit1/Ighg1/S100a8/Ighg2b/Lyz2/S100a9/Ighv1-62-3
SENESE_HDAC1_AND_HDAC2_TARGETS_UP	SENESE_HDAC1_AND_HDAC2_TARGETS_UP	100	-0.627123	-1.722286	0.0008273	0.0593024	0.056483	921	tags=26%, list=14%, signal=23%	Tmod1/Kdsr/Nap1l1/Cpeb4/Iqgap1/Il1rapl1/Hmga1/Pde4dip/Tgm2/Sorbs2/Nrip3/Dpy19l1/Bicd2/Fn1/Ptpn12/Cbfb/Atp11b/C3/Slc16a3/Gch1/Otub2/Rgs2/Serpina1b/Serpina1e/Homer3/Saa1
WP_FOLATE_METABOLISM	WP_FOLATE_METABOLISM	34	-0.779102	-1.820698	0.0009138	0.0600942	0.0572371	144	tags=32%, list=2%, signal=32%	Fga/Serpina3k/Alb/F2/Fgb/Fgg/Apoa1/Plg/Serpina3n/Saa1/Apob
AZARE_STAT3_TARGETS	AZARE_STAT3_TARGETS	17	-0.862041	-1.812773	0.0009164	0.0600942	0.0572371	410	tags=24%, list=6%, signal=22%	Fn1/Itgb6/S100a9/Thbs1
REACTOME_INITIAL_TRIGGERING_OF_COMPLEMENT	REACTOME_INITIAL_TRIGGERING_OF_COMPLEMENT	10	-0.918468	-1.780474	0.0009017	0.0600942	0.0572371	107	tags=80%, list=2%, signal=79%	C1qa/C4b/C3/Ighg1/Igkv12-41/Ighg2b/Cfb/Ighv1-62-3
REACTOME_TRANSPORT_OF_SMALL_MOLECULES	REACTOME_TRANSPORT_OF_SMALL_MOLECULES	362	-0.476139	-1.5147	0.0008614	0.0600942	0.0572371	1061	tags=28%, list=16%, signal=25%	Pmpcb/Npc2/Psmb3/Emb/Psme4/Slc8a2/Unc79/Slc12a5/Atp1a3/Slc25a5/Stoml2/Atp2b2/Hmox1/Atp6v1g1/Slc26a6/Atp6v0d1/Fxyd7/Slc4a4/Cyb5r4/Slc25a4/Calm1/Abca1/Slc9a6/Slc12a6/Slc13a3/Nipal3/Erlin2/Slc6a7/Pdzd11/Slc4a8/Npc1/Zdhhc8/Lrrc8b/Abca5/Psmd10/Slc13a5/Abcg4/Atp2a2/Arl2/Fxyd6/Atp6v0a1/Atp1a1/Ano2/Slc38a3/Magt1/Atp6v1g2/Phb2/Abcg2/Casq2/Slc6a1/Atp1b1/Clcn6/Mcu/Gnb4/Trpv2/Atp9b/Slc8a3/Vdac2/Cp/Mmgt1/Slc6a15/Vdac1/Slc6a5/Lmf1/Vdac3/Slc4a10/Derl1/Mbtps1/Slc4a7/Ubb/Slc25a29/Atp1a4/Slc35b4/Stom/Lcat/Atp6v0a2/Mb/Slc4a2/Abcc9/Apoc1/Slc4a1/Kcnj11/Slc12a4/Atp11b/Slc6a2/Alb/Apoa4/Tpcn1/Slc16a3/Slc38a1/Apoa1/Slc18a2/Apoa2/Plg/Slc36a1/Slc6a3/Slc36a4/A2m/Atp2a3/Apoc3/Aqp1/Apob
REACTOME_FCERI_MEDIATED_MAPK_ACTIVATION	REACTOME_FCERI_MEDIATED_MAPK_ACTIVATION	21	-0.839233	-1.823418	0.0009314	0.060175	0.0573141	136	tags=14%, list=2%, signal=14%	Fcer1g/Igkv12-41/Ighv1-62-3
WP_LIPID_PARTICLES_COMPOSITION	WP_LIPID_PARTICLES_COMPOSITION	6	-0.958462	-1.698419	0.0009547	0.0601897	0.0573282	256	tags=83%, list=4%, signal=80%	Lcat/Apoa1/Apoa2/Apoc3/Apob
ZHENG_IL22_SIGNALING_UP	ZHENG_IL22_SIGNALING_UP	17	-0.860458	-1.809445	0.0009843	0.0611341	0.0582277	540	tags=47%, list=8%, signal=43%	Slitrk1/Sbno2/Stom/C3/S100a8/Hp/Cfb/S100a9
REACTOME_METABOLISM_OF_STEROID_HORMONES	REACTOME_METABOLISM_OF_STEROID_HORMONES	13	-0.891277	-1.801472	0.0010356	0.0622929	0.0593314	111	tags=38%, list=2%, signal=38%	Hsd17b3/Hsd3b1/Cyp11a1/Serpina6/Pomc
REACTOME_FCGR_ACTIVATION	REACTOME_FCGR_ACTIVATION	8	-0.932456	-1.740556	0.0011169	0.0651784	0.0620797	70	tags=50%, list=1%, signal=50%	Ighg1/Igkv12-41/Ighg2b/Ighv1-62-3
WP_COMPLEMENT_SYSTEM	WP_COMPLEMENT_SYSTEM	36	-0.760065	-1.791741	0.0014032	0.0741731	0.0706467	336	tags=47%, list=5%, signal=45%	Cfh/C4b/Apcs/F10/Fga/Alb/C3/Fgb/Fgg/Apoa1/Plg/Cd93/Klkb1/Was/Serping1/Cfb/Thbs1
WP_PPARALPHA_PATHWAY	WP_PPARALPHA_PATHWAY	11	-0.900259	-1.770675	0.0015655	0.0803138	0.0764955	80	tags=36%, list=1%, signal=36%	Cpt1a/Apoa1/Apoa2/Apoc3
WP_CLASSICAL_PATHWAY_OF_STEROIDOGENESIS_WITH_GLUCOCORTICOID_AND_MINERALOCORTICOID_METABOLISM	WP_CLASSICAL_PATHWAY_OF_STEROIDOGENESIS_WITH_GLUCOCORTICOID_AND_MINERALOCORTICOID_METABOLISM	6	-0.950618	-1.684518	0.0015622	0.0803138	0.0764955	111	tags=50%, list=2%, signal=49%	Hsd17b3/Hsd3b1/Cyp11a1
REACTOME_IRAK4_DEFICIENCY_TLR2_4	REACTOME_IRAK4_DEFICIENCY_TLR2_4	8	-0.926083	-1.728658	0.0016324	0.0805862	0.0767549	361	tags=75%, list=5%, signal=71%	Myd88/Fga/Fgb/Fgg/S100a8/S100a9
WP_ONECARBON_METABOLISM	WP_ONECARBON_METABOLISM	19	0.8296257	1.8167309	0.001731	0.0831054	0.0791544	102	tags=16%, list=2%, signal=16%	Tcn2/Shmt1/Dhfr
HINATA_NFKB_TARGETS_KERATINOCYTE_DN	HINATA_NFKB_TARGETS_KERATINOCYTE_DN	8	-0.921507	-1.720116	0.0020619	0.0946497	0.0901498	85	tags=25%, list=1%, signal=25%	Rgs2/Thbs1
REACTOME_NCAM1_INTERACTIONS	REACTOME_NCAM1_INTERACTIONS	15	0.8583923	1.8128521	0.002095	0.0953296	0.0907974	44	tags=20%, list=1%, signal=20%	Col6a1/Col6a2/Col4a2
BOCHKIS_FOXA2_TARGETS	BOCHKIS_FOXA2_TARGETS	193	-0.527519	-1.575384	0.00224	0.1001882	0.095425	502	tags=16%, list=8%, signal=15%	Nuf2/Ndufa8/Tmem121b/Ndufa2/Gstm2/Ghitm/Tmem259/Dhcr7/Isg15/Chac2/Apoh/Aldh1a7/Ttr/Coch/Gcat/Apoc1/Trim13/Serpina3k/Ahsg/Hpx/Mug1/Fgb/Plg/Serpina1b/Serpinc1/Serpina1e/Klkb1/Serpinf2/Serpina6/Cyp2a5/Serpina3n
WUNDER_INFLAMMATORY_RESPONSE_AND_CHOLESTEROL_UP	WUNDER_INFLAMMATORY_RESPONSE_AND_CHOLESTEROL_UP	23	-0.804647	-1.771231	0.0024793	0.1069086	0.1018259	245	tags=26%, list=4%, signal=25%	Gbp2/B2m/H2-Q10/Krt16/Serpina1b/Serpina1e
HOEGERKORP_CD44_TARGETS_DIRECT_UP	HOEGERKORP_CD44_TARGETS_DIRECT_UP	12	-0.87871	-1.752671	0.0025333	0.1069086	0.1018259	125	tags=33%, list=2%, signal=33%	Alb/Plg/Serpinc1/Aqp1
PID_INTEGRIN2_PATHWAY	PID_INTEGRIN2_PATHWAY	11	-0.888961	-1.748454	0.0025183	0.1069086	0.1018259	185	tags=55%, list=3%, signal=53%	Itgb2/F10/Fga/C3/Fgb/Fgg
LIAN_LIPA_TARGETS_6M	LIAN_LIPA_TARGETS_6M	21	-0.81326	-1.766986	0.0026757	0.1085825	0.1034202	142	tags=43%, list=2%, signal=42%	Hmox1/C1qb/Neu1/C1qa/Aar2/Fcer1g/Slc6a2/Cd5l/S100a9
REACTOME_TOLL_LIKE_RECEPTOR_CASCADES	REACTOME_TOLL_LIKE_RECEPTOR_CASCADES	80	-0.633595	-1.686215	0.002615	0.1085825	0.1034202	630	tags=21%, list=9%, signal=19%	Itgb2/Mapkapk2/Atf2/Mef2a/Myd88/Ubb/Birc2/Lgmn/Irak1/Mef2c/Fga/Fgb/Fgg/S100a8/Saa1/S100a9/Apob
WP_TRANSSULFURATION_AND_ONECARBON_METABOLISM	WP_TRANSSULFURATION_AND_ONECARBON_METABOLISM	22	0.7997271	1.7970798	0.0028457	0.1135796	0.1081797	102	tags=14%, list=2%, signal=13%	Mthfd2l/Shmt1/Dhfr
REACTOME_ECM_PROTEOGLYCANS	REACTOME_ECM_PROTEOGLYCANS	30	0.7521721	1.7815304	0.0028457	0.1135796	0.1081797	269	tags=23%, list=4%, signal=22%	Col6a1/Col6a2/Col4a2/Col1a1/Dcn/Itga7/Sparc
REACTOME_METABOLISM_OF_FOLATE_AND_PTERINES	REACTOME_METABOLISM_OF_FOLATE_AND_PTERINES	9	0.9084247	1.7802944	0.0029908	0.116796	0.1112432	102	tags=33%, list=2%, signal=33%	Mthfd2l/Shmt1/Dhfr
REACTOME_DISEASES_OF_IMMUNE_SYSTEM	REACTOME_DISEASES_OF_IMMUNE_SYSTEM	13	-0.863213	-1.744746	0.0032427	0.1225125	0.116688	361	tags=46%, list=5%, signal=44%	Myd88/Fga/Fgb/Fgg/S100a8/S100a9
WP_VITAMIN_B12_DISORDERS	WP_VITAMIN_B12_DISORDERS	5	0.9543975	1.7105064	0.0032671	0.1225125	0.116688	2	tags=20%, list=0%, signal=20%	Tcn2
NABA_MATRISOME	NABA_MATRISOME	192	-0.519659	-1.550583	0.0032572	0.1225125	0.116688	254	tags=17%, list=4%, signal=17%	Cpn2/Igfbp7/Lamb2/Coch/Ctsh/F10/Col18a1/Fga/Serpina3k/Ints14/Serpinf1/F2/Hpx/Mug1/Fgb/Fgg/Itih4/Itih2/Plg/Serpina1b/Serpinc1/Serpina1e/S100a8/Serpind1/Serpinf2/Serpina6/A2m/Serping1/Serpina3n/Il17f/S100a9/Thbs1/Hrg
REACTOME_ROLE_OF_PHOSPHOLIPIDS_IN_PHAGOCYTOSIS	REACTOME_ROLE_OF_PHOSPHOLIPIDS_IN_PHAGOCYTOSIS	19	-0.816542	-1.746612	0.0035093	0.128421	0.1223156	70	tags=26%, list=1%, signal=26%	Pik3ca/Ighg1/Igkv12-41/Ighg2b/Ighv1-62-3
RUAN_RESPONSE_TO_TNF_TROGLITAZONE_UP	RUAN_RESPONSE_TO_TNF_TROGLITAZONE_UP	10	-0.888651	-1.722674	0.0035387	0.1285966	0.1224828	425	tags=60%, list=6%, signal=56%	Cp/Gbp2/Serpina3k/H2-Q10/Hp/Serpina3n
WP_VALPROIC_ACID_PATHWAY	WP_VALPROIC_ACID_PATHWAY	7	-0.924539	-1.684998	0.0036054	0.1292961	0.1231491	24	tags=29%, list=0%, signal=28%	Acadsb/Cyp2a5
REACTOME_METABOLISM_OF_STEROIDS	REACTOME_METABOLISM_OF_STEROIDS	67	-0.646807	-1.676859	0.0037558	0.1330963	0.1267686	411	tags=21%, list=6%, signal=20%	Ebp/Dhcr7/Mbtps1/Lgmn/Msmo1/Akr1c6/Alb/Hsd17b3/Gc/Hsd3b1/Cyp11a1/Serpina6/Acacb/Pomc
KAMIKUBO_MYELOID_CEBPA_NETWORK	KAMIKUBO_MYELOID_CEBPA_NETWORK	13	-0.859903	-1.738056	0.0038369	0.1338583	0.1274943	64	tags=46%, list=1%, signal=46%	Anxa1/Il18/S100a8/Hp/Lyz2/S100a9
REACTOME_NEUTROPHIL_DEGRANULATION	REACTOME_NEUTROPHIL_DEGRANULATION	282	-0.47431	-1.474335	0.0038229	0.1338583	0.1274943	772	tags=15%, list=12%, signal=13%	Tubb5/Ctsz/Kcmf1/Gusb/Ndufc2/Neu1/Degs1/Itgb2/Prcp/Plekho2/Atp6v0a1/Magt1/Mospd2/Gla/Krt1/Rhof/Iqgap2/Cmtm6/Ptpn6/Stom/Dgat1/Ttr/B2m/Ctsh/Atp11b/Ypel5/Fcer1g/Serpina3k/Ahsg/C3/H2-Q10/Orm1/Rab37/Serpina1b/Serpina1e/Cd93/S100a8/Hp/Lyz2/Serpina3n/S100a9
SCHOEN_NFKB_SIGNALING	SCHOEN_NFKB_SIGNALING	7	-0.922311	-1.680937	0.0039676	0.134966	0.1285494	130	tags=71%, list=2%, signal=70%	Fn1/Serpina3k/C3/Serpina3n/Thbs1
WP_METABOLIC_PATHWAY_OF_LDL_HDL_AND_TG_INCLUDING_DISEASES	WP_METABOLIC_PATHWAY_OF_LDL_HDL_AND_TG_INCLUDING_DISEASES	7	-0.920742	-1.678076	0.0042954	0.1450182	0.1381237	256	tags=57%, list=4%, signal=55%	Lcat/Apoa1/Apoa2/Apob
WP_SERINE_METABOLISM	WP_SERINE_METABOLISM	7	0.9242676	1.7507406	0.0044012	0.1466973	0.1397229	102	tags=29%, list=2%, signal=28%	Shmt1/Dhfr
REACTOME_FCGAMMA_RECEPTOR_FCGR_DEPENDENT_PHAGOCYTOSIS	REACTOME_FCGAMMA_RECEPTOR_FCGR_DEPENDENT_PHAGOCYTOSIS	71	-0.632874	-1.65566	0.0046489	0.1520466	0.1448179	70	tags=8%, list=1%, signal=8%	Abl1/Ighg1/Igkv12-41/Ighg2b/Was/Ighv1-62-3
REACTOME_TOLL_LIKE_RECEPTOR_TLR1_TLR2_CASCADE	REACTOME_TOLL_LIKE_RECEPTOR_TLR1_TLR2_CASCADE	53	-0.67394	-1.686282	0.0047385	0.1540174	0.146695	626	tags=25%, list=9%, signal=22%	Mapkapk2/Atf2/Mef2a/Myd88/Ubb/Irak1/Mef2c/Fga/Fgb/Fgg/S100a8/Saa1/S100a9
REACTOME_SIGNALING_BY_LEPTIN	REACTOME_SIGNALING_BY_LEPTIN	6	0.9352184	1.7258002	0.0050716	0.1608463	0.1531993	4	tags=17%, list=0%, signal=17%	Socs3
COATES_MACROPHAGE_M1_VS_M2_DN	COATES_MACROPHAGE_M1_VS_M2_DN	34	-0.729875	-1.705657	0.0050257	0.1608463	0.1531993	185	tags=24%, list=3%, signal=23%	C1qb/Xdh/Mfge8/F10/Ighm/H2-Q10/Cd5l/Thbs1
GOZGIT_ESR1_TARGETS_DN	GOZGIT_ESR1_TARGETS_DN	280	-0.46946	-1.458184	0.0050677	0.1608463	0.1531993	1065	tags=21%, list=16%, signal=18%	Epha4/Diras2/Npc2/Hk1/Tcf4/Ephx1/Prkca/Ugcg/Gabarapl1/Shank2/Sytl2/Mb21d2/Cab39l/Ppm1e/Fam114a1/Epb41l5/Ctsf/Ppm1l/Ifit3/Slc12a6/Ppm1k/Strbp/Fut9/Ucp3/Dgkd/Tgm2/Cpt1a/Acot2/Abca5/Tceal1/Sgsm2/Ptprk/Nfix/Atp1b1/Gfra1/Cp/Fgf13/Tmsb4x/Sat2/C4b/Slc25a29/Dnajc12/Ythdc1/Nnt/Gbp2/Ift122/Epha7/Col18a1/Atp11b/Serpina3k/Wls/Serpina1b/Serpina1e/Pgpep1/Atp2a3/Cfb/Serpina3n/Thbs1/Rerg
REACTOME_P130CAS_LINKAGE_TO_MAPK_SIGNALING_FOR_INTEGRINS	REACTOME_P130CAS_LINKAGE_TO_MAPK_SIGNALING_FOR_INTEGRINS	13	-0.851052	-1.720167	0.0052291	0.1648431	0.157006	410	tags=38%, list=6%, signal=36%	Fn1/Apbb1ip/Fga/Fgb/Fgg
REACTOME_FCGR3A_MEDIATED_IL10_SYNTHESIS	REACTOME_FCGR3A_MEDIATED_IL10_SYNTHESIS	28	-0.756204	-1.713601	0.0054521	0.1708445	0.1627221	70	tags=18%, list=1%, signal=18%	Calm1/Ighg1/Igkv12-41/Ighg2b/Ighv1-62-3

BRUINS_UVC_RESPONSE_VIA_TP53_GROUP_A	BRUINS_UVC_RESPONSE_VIA_TP53_GROUP_A	351	-0.447446	-1.419473	0.0056914	0.1753617	0.1670245	644	tags=15%, list=10%, signal=14%	Degs1/Pnkd/Cacng2/Tapbp/Cx3cl1/Med15/Nrgn/Ildr2/Slitrk1/Has1/Abcg2/Ifitm3/Kit/Gla/Alpl/Nol9/Map3k4/Dab2/Gpr155/Dhcr7/Cnn1/Flnc/Mgst3/Irak1/Itgb6/Glipr2/Mef2c/Dchs1/Actn3/Kcnj11/Apcs/Pgm5/Serpina3k/Ahsg/Apoa4/Fmo5/Hsd17b3/Stk38/Krt16/Gnpnat1/Hpx/Bpifa3/Cd93/Klkb1/Cyp11a1/Gipc2/Cpb2/Serping1/Fetub/Cfb/Serpina3n
PEREZ_TP53_TARGETS	PEREZ_TP53_TARGETS	405	0.3968012	1.3701384	0.0057981	0.1753838	0.1670457	765	tags=17%, list=12%, signal=16%	Abcb6/Kin/Col6a1/Aifm2/Ece1/Micall2/Limd1/Rmdn2/Rapgef5/Eppk1/Fyco1/Gid4/Baiap3/Disp2/Amotl1/Carns1/Fitm2/Vamp5/Gpr37/Ccnk/Ttyh2/Lpcat3/Lgals9/Foxp4/Slc35g2/Gls2/Elapor1/Gstt2/Gng11/Sdhaf1/Gab1/Mindy1/Aspa/Tgs1/Cracd/Lbh/Pcmtd2/Cnnm3/Insyn2a/Frmd8/Camk1/Poglut1/Ube2q1/Arhgef17/Adcyap1r1/Txlna/Taf5/Mlc1/Atpaf2/Gramd4/Sema4d/Cryab/Ganc/Snx32/Arnt2/Dvl3/Cpe/Llgl1/Frmd5/Plaat3/Ankrd46/S1pr5/Mad1l1/Abhd4/Lifr/Map7d2/Nsg1
ONDER_CDH1_TARGETS_2_UP	ONDER_CDH1_TARGETS_2_UP	115	0.5360664	1.5936587	0.0059134	0.1778436	0.1693885	450	tags=14%, list=7%, signal=13%	Nid1/Col6a1/Col6a2/Slc22a4/Col1a1/Dcn/Postn/Prr3/Itga7/Sparc/Enpp1/Gng11/Anxa6/Mme/Marcks/Fam50a
LIANG_SILENCED_BY_METHYLATION_UP	LIANG_SILENCED_BY_METHYLATION_UP	11	-0.866717	-1.704703	0.0061087	0.1816286	0.1729935	497	tags=55%, list=7%, signal=51%	Krt17/Lgmn/Krt8/C3/Saa1/Aqp1
LEE_CALORIE_RESTRICTION_MUSCLE_DN	LEE_CALORIE_RESTRICTION_MUSCLE_DN	21	-0.789647	-1.715682	0.0061813	0.1817235	0.1730839	302	tags=24%, list=5%, signal=23%	Mapkapk2/Irak1/Rit1/Ighm/S100a9
REACTOME_SCAVENGING_BY_CLASS_F_RECEPTORS	REACTOME_SCAVENGING_BY_CLASS_F_RECEPTORS	5	-0.940975	-1.614436	0.006509	0.1881844	0.1792377	9	tags=60%, list=0%, signal=60%	Hsp90aa1/Calr/Apob
MENSE_HYPOXIA_UP	MENSE_HYPOXIA_UP	36	0.7066633	1.7262608	0.0066825	0.1910902	0.1820053	16	tags=6%, list=0%, signal=6%	Abcb6/Jmjd6
AZARE_NEOPLASTIC_TRANSFORMATION_BY_STAT3_UP	AZARE_NEOPLASTIC_TRANSFORMATION_BY_STAT3_UP	59	-0.646365	-1.643817	0.0067296	0.1913903	0.1822911	657	tags=19%, list=10%, signal=17%	Ptprk/Ptbp3/Kcnma1/Fn1/Palld/Slc4a7/Cfh/Itgb6/S100a8/S100a9/Thbs1
KEGG_PARKINSONS_DISEASE	KEGG_PARKINSONS_DISEASE	85	-0.762063	-2.046269	1.64E-05	0.0041033	0.0039082	1056	tags=67%, list=16%, signal=57%	Ndufa4/Uqcrfs1/Cox6b1/Cox7a1/Uqcr10/Slc25a5/Uqcrb/Slc25a4/Cox7a2l/Ndufs4/Ndufs3/Ndufa5/Ndufc2/Ndufs7/Uqcrc2/Cyc1/Ndufb10/Ndufs6/Ndufs5/Ndufa10/Uqcrh/Lrrk2/Ndufa6/Ndufb1/Ndufa9/Ndufb5/Cox4i1/Ndufs1/Ndufv2/Ndufs2/Ndufa8/Ndufv1/Uqcrq/Ndufa2/Vdac2/Vdac1/Ndufb2/Ndufv3/Ndufb6/Vdac3/Cox6c/Ndufab1/Cox5b/Ndufa7/Ndufb7/Cox5a/Ubb/Ndufb9/Ndufb4/Ndufa1/Cox6a1/Ndufb3/Cox7c/Th/Slc18a2/Slc6a3/Cox7b
WP_ELECTRON_TRANSPORT_CHAIN_OXPHOS_SYSTEM_IN_MITOCHONDRIA	WP_ELECTRON_TRANSPORT_CHAIN_OXPHOS_SYSTEM_IN_MITOCHONDRIA	67	-0.774181	-2.007079	1.662E-05	0.0041033	0.0039082	1056	tags=76%, list=16%, signal=65%	Ndufa4/Uqcrfs1/Cox6b1/Cox7a1/Uqcr10/Slc25a5/Uqcrb/Slc25a4/Cox7a2l/Ndufs4/Ndufs3/Ndufa12/Ucp3/Ndufa5/Ndufc2/Ndufs7/Uqcrc2/Ndufb10/Ndufs6/Ndufs5/Ndufa10/Uqcrh/Slc25a14/Ndufa6/Ndufb1/Ndufa9/Ndufb5/Cox4i1/Ndufs1/Ndufv2/Ndufs2/Ndufa8/Ndufv1/Uqcrq/Ndufa2/Ndufb2/Ndufv3/Ndufb6/Cox6c/Ndufab1/Cox5b/Ndufa7/Ndufb7/Cox5a/Ndufb9/Ndufb4/Ndufa1/Cox6a1/Ndufb3/Cox7c/Cox7b
KEGG_OXIDATIVE_PHOSPHORYLATION	KEGG_OXIDATIVE_PHOSPHORYLATION	78	-0.741988	-1.96839	1.646E-05	0.0041033	0.0039082	1056	tags=68%, list=16%, signal=58%	Ndufa4/Uqcrfs1/Cox6b1/Cox7a1/Uqcr10/Atp6v1g1/Atp6v0d1/Uqcrb/Cox7a2l/Ndufs4/Ndufs3/Ndufa5/Ndufc2/Ndufs7/Uqcrc2/Cyc1/Ndufb10/Ndufs6/Ndufs5/Ndufa10/Uqcrh/Atp6v0a1/Ndufa6/Ndufb1/Ndufa9/Atp6v1g2/Ndufb5/Cox4i1/Ndufs1/Ndufv2/Ndufs2/Ndufa8/Ndufv1/Uqcrq/Ndufa2/Ndufb2/Ndufv3/Ndufb6/Cox6c/Ndufab1/Cox5b/Ndufa7/Ndufb7/Cox5a/Ndufa11/Ndufb9/Ndufb4/Ndufa1/Cox6a1/Atp6v0a2/Ndufb3/Cox7c/Cox7b
REACTOME_RESPIRATORY_ELECTRON_TRANSPORT	REACTOME_RESPIRATORY_ELECTRON_TRANSPORT	86	-0.712031	-1.914895	1.638E-05	0.0041033	0.0039082	1294	tags=67%, list=19%, signal=55%	Ndufs8/Ndufb8/Sdhc/Ecsit/Ndufaf3/Ndufaf2/Ndufa4/Uqcrfs1/Cox6b1/Uqcr10/Tmem126b/Uqcrb/Cox7a2l/Ndufs4/Ndufs3/Ndufa12/Ndufa5/Ndufc2/Ndufs7/Uqcrc2/Cyc1/Ndufb10/Ndufs6/Ndufs5/Cox19/Ndufa10/Uqcrh/Ndufa6/Ndufb1/Ndufa9/Ndufb5/Ndufb11/Cox4i1/Ndufs1/Ndufv2/Ndufa13/Ndufs2/Ndufa8/Ndufv1/Uqcrq/Ndufa2/Ndufb2/Ndufv3/Ndufb6/Cox6c/Ndufab1/Cox5b/Ndufa7/Ndufb7/Cox5a/Ndufa11/Ndufb9/Ndufb4/Ndufa1/Cox6a1/Ndufb3/Cox7c/Cox7b
REACTOME_RESPIRATORY_ELECTRON_TRANSPORT_ATP_SYNTHESIS_BY_CHEMIOSMOTIC_COUPLING_AND_HEAT_PRODUCTION_BY_UNCOUPLING_PROTEINS	REACTOME_RESPIRATORY_ELECTRON_TRANSPORT_ATP_SYNTHESIS_BY_CHEMIOSMOTIC_COUPLING_AND_HEAT_PRODUCTION_BY_UNCOUPLING_PROTEINS	91	-0.709049	-1.921863	3.268E-05	0.0062012	0.0059064	1329	tags=68%, list=20%, signal=55%	Slc25a27/Ndufs8/Ndufb8/Sdhc/Ecsit/Atp5pb/Ndufaf3/Ndufaf2/Ndufa4/Uqcrfs1/Cox6b1/Uqcr10/Tmem126b/Uqcrb/Cox7a2l/Ndufs4/Ndufs3/Ndufa12/Ucp3/Ndufa5/Ndufc2/Ndufs7/Uqcrc2/Cyc1/Ndufb10/Ndufs6/Ndufs5/Cox19/Ndufa10/Uqcrh/Slc25a14/Ndufa6/Ndufb1/Ndufa9/Ndufb5/Ndufb11/Cox4i1/Ndufs1/Ndufv2/Ndufa13/Ndufs2/Ndufa8/Ndufv1/Uqcrq/Ndufa2/Ndufb2/Ndufv3/Ndufb6/Cox6c/Ndufab1/Cox5b/Ndufa7/Ndufb7/Cox5a/Ndufa11/Ndufb9/Ndufb4/Ndufa1/Cox6a1/Ndufb3/Cox7c/Cox7b
REACTOME_THE_CITRIC_ACID_TCA_CYCLE_AND_RESPIRATORY_ELECTRON_TRANSPORT	REACTOME_THE_CITRIC_ACID_TCA_CYCLE_AND_RESPIRATORY_ELECTRON_TRANSPORT	131	-0.653293	-1.860935	3.178E-05	0.0062012	0.0059064	1354	tags=52%, list=20%, signal=42%	Cs/Slc25a27/Ndufs8/Ndufb8/Sdhc/Ecsit/Atp5pb/Mpc1/Ndufaf3/Ndufaf2/Ndufa4/Uqcrfs1/Cox6b1/Uqcr10/Suclg1/Tmem126b/Uqcrb/Cox7a2l/Ndufs4/Ndufs3/Ndufa12/Ucp3/Ndufa5/Ndufc2/Ndufs7/Uqcrc2/Cyc1/Ndufb10/Ndufs6/Ndufs5/Cox19/Ndufa10/Uqcrh/Slc25a14/Ndufa6/Ndufb1/Ndufa9/Ndufb5/Ndufb11/Cox4i1/Ndufs1/Ndufv2/Ndufa13/Ndufs2/Ndufa8/Ndufv1/Uqcrq/Ndufa2/Vdac1/Ndufb2/Ndufv3/Ndufb6/Cox6c/Ndufab1/Cox5b/Ndufa7/Ndufb7/Cox5a/Ndufa11/Ndufb9/Ndufb4/Nnt/Ndufa1/Cox6a1/Ndufb3/Cox7c/Slc16a3/Cox7b
MOOTHA_VOXPHOS	MOOTHA_VOXPHOS	66	-0.746648	-1.931517	4.988E-05	0.0078404	0.0074676	1078	tags=67%, list=16%, signal=56%	Surf2/Ndufa4/Uqcrfs1/Cox6b1/Cox7a1/Uqcr10/Uqcrb/Cox7a2l/Ndufs4/Ndufs3/Ndufa5/Ndufc2/Ndufs7/Uqcrc2/Cyc1/Ndufs6/Ndufs5/Ndufa10/Uqcrh/Ndufa6/Ndufb1/Ndufa9/Ndufb5/Cox4i1/Ndufs1/Ndufa13/Ndufs2/Ndufa8/Uqcrq/Ndufa2/Ndufb2/Ndufb6/Cox6c/Ndufab1/Cox5b/Ndufa7/Ndufb7/Cox5a/Ndufb4/Ndufa1/Cox6a1/Ndufb3/Cox7c/Cox7b
REACTOME_COMPLEX_I_BIOGENESIS	REACTOME_COMPLEX_I_BIOGENESIS	49	-0.764804	-1.890667	0.0001847	0.0216652	0.0206352	775	tags=80%, list=12%, signal=71%	Ndufs8/Ndufb8/Ecsit/Ndufaf3/Ndufaf2/Tmem126b/Ndufa4/Ndufs3/Ndufa12/Ndufa5/Ndufc2/Ndufs7/Ndufb10/Ndufs6/Ndufs5/Ndufa10/Ndufa6/Ndufb1/Ndufa9/Ndufb5/Ndufb11/Ndufs1/Ndufv2/Ndufa13/Ndufs2/Ndufa8/Ndufv1/Ndufa2/Ndufb2/Ndufv3/Ndufb6/Ndufab1/Ndufa7/Ndufb7/Ndufa11/Ndufb9/Ndufb4/Ndufa1/Ndufb3
WP_MITOCHONDRIAL_COMPLEX_I_ASSEMBLY_MODEL_OXPHOS_SYSTEM	WP_MITOCHONDRIAL_COMPLEX_I_ASSEMBLY_MODEL_OXPHOS_SYSTEM	47	-0.761898	-1.870605	0.0002521	0.02638	0.0251258	775	tags=77%, list=12%, signal=68%	Ndufs8/Ecsit/Ndufaf3/Ndufaf2/Tmem126b/Ndufa4/Ndufs3/Ndufa12/Ndufa5/Ndufc2/Ndufb10/Ndufs6/Ndufa10/Ndufa6/Ndufb1/Ndufa9/Ndufb5/Ndufb11/Ndufs1/Ndufv2/Ndufa13/Ndufs2/Tmem70/Ndufa8/Ndufv1/Ndufa2/Ndufb2/Ndufv3/Ndufb6/Ndufab1/Ndufa7/Ndufb7/Ndufb9/Ndufb4/Ndufa1/Ndufb3
WP_OXIDATIVE_PHOSPHORYLATION	WP_OXIDATIVE_PHOSPHORYLATION	36	-0.797369	-1.879678	0.0002874	0.0287685	0.0274008	775	tags=78%, list=12%, signal=69%	Ndufa4/Ndufs3/Ndufa5/Ndufc2/Ndufs7/Ndufb10/Ndufs6/Ndufs5/Ndufa10/Ndufa6/Ndufb1/Ndufa9/Ndufb5/Ndufs1/Ndufv2/Ndufs2/Ndufa8/Ndufv1/Ndufa2/Ndufb2/Ndufv3/Ndufb6/Ndufab1/Ndufa7/Ndufb7/Ndufa11/Ndufb9/Ndufb4
WP_DOPAMINERGIC_NEUROGENESIS	WP_DOPAMINERGIC_NEUROGENESIS	6	-0.966369	-1.71243	0.0004166	0.0381008	0.0362894	228	tags=83%, list=3%, signal=81%	Aldh1a7/Ddc/Th/Slc18a2/Slc6a3
MOOTHA_MITOCHONDRIA	MOOTHA_MITOCHONDRIA	334	-0.449499	-1.419818	0.0061512	0.1817235	0.1730839	1061	tags=23%, list=16%, signal=21%	Pmpcb/Ndufa4/Coq8a/Uqcrfs1/Hk1/Cox6b1/Cox7a1/Suclg1/Slc25a5/Atp5mpl/Mtx2/Mrpl42/Slc25a23/Got2/Uqcrb/Mrps18a/Slc25a4/Slc9a6/Tomm20/Ndufs4/Ndufs3/Ucp3/Ndufa5/Slc25a11/Slc25a20/Ndufc2/Cpt1a/Uqcrc2/Cyc1/Mrps12/Ndufb6/Tomm40/Ndufs5/Ndufa10/Uqcrh/Slc25a14/Ndufa6/Ndufb1/Bcat2/Dut/Ndufb5/Cox4i1/Mtco1/Ndufs1/Ndufv2/Timm10/Ndufs2/Ndufa8/Ndufv1/Vdac2/Timm8a1/Mtch2/Ndufb2/Sbno2/Ndufb6/Vdac3/Cox6c/Ndufab1/Cox5b/Ndufa7/Ndufb7/Cox5a/Ndufb4/Mtch1/Slc25a15/Nnt/Ndufa1/Sirt4/Cox6a1/Lcat/Ndufb3/Mb/Cox7c/Slc25a21/Timm17a/Cyp11a1/Cox7b
HSIAO_LIVER_SPECIFIC_GENES	HSIAO_LIVER_SPECIFIC_GENES	109	-0.854686	-2.374797	1.613E-05	0.0041033	0.0039082	256	tags=43%, list=4%, signal=42%	Lcat/Cpn2/Apoh/Aldh1a7/Ttr/Msmo1/Apoc1/Apcs/F10/Akr1c6/Fga/Serpina3k/Alb/Ahsg/Fmo5/Serpinf1/F2/C3/Orm1/Hpx/Gch1/Mug1/Fgb/Fgg/Apoa4/Itih4/Itih2/Apoa2/Plg/Serpina1b/Serpinc1/Serpina1e/Klkb1/Serpind1/Hp/Serpinf2/Serpina6/Cpb2/Serpina1/Cyp2a5/Cfb/Serpina3n/Apoc3/Saa1/Afm/Apob/Hrg
CAIRO_LIVER_DEVELOPMENT_DN	CAIRO_LIVER_DEVELOPMENT_DN	105	-0.786758	-2.17487	1.619E-05	0.0041033	0.0039082	293	tags=38%, list=4%, signal=37%	Enpp3/C1qa/Gstm2/Ebp/Fn1/Cfh/Dcxr/Lcat/Gcat/Ctsh/F10/Col18a1/Fga/Ahsg/Serpinf1/F2/C3/Fgb/Fgg/Itih4/Itih2/Plg/Serpina1b/Serpinc1/Serpina1e/Klkb1/S100a8/Gc/Serpind1/Hp/Serpinf2/Lyz2/Cpb2/A2m/Serping1/Rdh5/S100a9/Afm/Aqp1/Apob
REACTOME_RESPONSE_TO_ELEVATED_PLATELET_CYTOSOLIC_CA2	REACTOME_RESPONSE_TO_ELEVATED_PLATELET_CYTOSOLIC_CA2	78	-0.801116	-2.125248	1.646E-05	0.0041033	0.0039082	410	tags=28%, list=6%, signal=27%	Fn1/Tmsb4x/Lgals3bp/Apoh/Fga/Serpina3k/Alb/Ahsg/Orm1/Fgb/Fgg/Apoa1/Itih4/Plg/Serpina1b/Serpina1e/Serpinf2/A2m/Serping1/Serpina3n/Thbs1/Hrg
WP_NETWORK_MAP_OF_SARSCOV2_SIGNALING_PATHWAY	WP_NETWORK_MAP_OF_SARSCOV2_SIGNALING_PATHWAY	75	-0.772634	-2.039609	1.648E-05	0.0041033	0.0039082	410	tags=28%, list=6%, signal=27%	Fn1/Il18/Myd88/Lgals3bp/Cfh/Ptpn6/Irak1/Apoh/Apoc1/Pias1/Fga/Alb/Fgb/Fgg/Apoa1/Itih4/Apoa2/Hp/Cfb/Saa1/Hrg
HOSHIDA_LIVER_CANCER_SUBCLASS_S3	HOSHIDA_LIVER_CANCER_SUBCLASS_S3	137	-0.693685	-1.987719	1.582E-05	0.0041033	0.0039082	506	tags=23%, list=8%, signal=22%	Slc6a1/Ndufv2/Alpl/Cp/Cox5b/Isg15/Iqgap2/Cfh/C4b/Lcat/Apoh/Aldh1a7/Msmo1/Apcs/Col18a1/Nfib/Serpina3k/F2/Gch1/Fgb/Fgg/Apoa1/Itih4/Plg/Serpinc1/Klkb1/Serpina6/Cpb2/Serping1/Cfb/Serpina3n/Hrg
SU_LIVER	SU_LIVER	19	-0.928385	-1.985849	1.695E-05	0.0041033	0.0039082	254	tags=47%, list=4%, signal=46%	Cpn2/Apcs/Akr1c6/Itih2/Plg/Serpinc1/Serpind1/Afm/Apob
REACTOME_FORMATION_OF_FIBRIN_CLOT_CLOTTING_CASCADE	REACTOME_FORMATION_OF_FIBRIN_CLOT_CLOTTING_CASCADE	14	-0.95022	-1.942719	1.698E-05	0.0041033	0.0039082	185	tags=71%, list=3%, signal=70%	F10/Fga/F2/Fgb/Fgg/Serpinc1/Klkb1/Serpind1/A2m/Serping1
CAIRO_HEPATOBLASTOMA_DN	CAIRO_HEPATOBLASTOMA_DN	100	-0.699373	-1.920708	1.622E-05	0.0041033	0.0039082	296	tags=17%, list=4%, signal=16%	Slc25a15/Gpr88/Nnt/Lcat/Pxmp2/Hpx/Gch1/Mug1/Itih4/Plg/Hp/Mfap3l/Acacb/Serping1/Fetub/Cyp2a5/Hrg
REACTOME_HEMOSTASIS	REACTOME_HEMOSTASIS	321	-0.606914	-1.910149	1.453E-05	0.0041033	0.0039082	456	tags=14%, list=7%, signal=14%	Atp1b1/Kcnma1/Gnb4/Slc8a3/Fn1/Tuba3a/Tmsb4x/Lgals3bp/Esam/Kcnmb4/Ptpn6/Prkch/Apbb1ip/Apoh/Cables1/F10/Fga/Fcer1g/Serpina3k/Alb/Ahsg/Ighm/F2/Slc16a3/Orm1/Fgb/Fgg/Apoa1/Itih4/Plg/Serpina1b/Serpinc1/Serpina1e/Klkb1/Igkv12-41/Serpind1/Serpinf2/A2m/Serping1/Atp2a3/Serpina3n/Thbs1/Ighv1-62-3/Apob/Hrg
REACTOME_PLATELET_ACTIVATION_SIGNALING_AND_AGGREGATION	REACTOME_PLATELET_ACTIVATION_SIGNALING_AND_AGGREGATION	163	-0.636215	-1.86218	1.559E-05	0.0041033	0.0039082	362	tags=16%, list=5%, signal=15%	Tmsb4x/Lgals3bp/Ptpn6/Prkch/Apbb1ip/Apoh/Fga/Fcer1g/Serpina3k/Alb/Ahsg/F2/Orm1/Fgb/Fgg/Apoa1/Itih4/Plg/Serpina1b/Serpina1e/Serpinf2/A2m/Serping1/Serpina3n/Thbs1/Hrg
WP_BLOOD_CLOTTING_CASCADE	WP_BLOOD_CLOTTING_CASCADE	9	-0.97232	-1.853058	1.708E-05	0.0041033	0.0039082	185	tags=89%, list=3%, signal=87%	F10/Fga/F2/Fgb/Fgg/Plg/Klkb1/Serpinf2
MCLACHLAN_DENTAL_CARIES_UP	MCLACHLAN_DENTAL_CARIES_UP	73	-0.740459	-1.944832	3.304E-05	0.0062012	0.0059064	200	tags=30%, list=3%, signal=30%	Apoc1/Ctsh/Fcer1g/Serpina3k/Igkc/Ighm/C3/Orm1/Rgs2/Serpina1b/Ighg1/Serpina1e/Cd93/S100a8/Igkv12-41/Ighg2b/Lyz2/Cfb/Serpina3n/Saa1/S100a9/Ighv1-62-3
REACTOME_PLASMA_LIPOPROTEIN_REMODELING	REACTOME_PLASMA_LIPOPROTEIN_REMODELING	15	-0.91664	-1.895604	3.393E-05	0.0062012	0.0059064	394	tags=67%, list=6%, signal=63%	Lmf1/Mbtps1/Lcat/Alb/Apoa4/Apoa1/Apoa2/Plg/Apoc3/Apob
REACTOME_CHYLOMICRON_ASSEMBLY	REACTOME_CHYLOMICRON_ASSEMBLY	8	-0.970715	-1.81197	3.437E-05	0.0062012	0.0059064	122	tags=62%, list=2%, signal=61%	Apoa4/Apoa1/Apoa2/Apoc3/Apob
LEE_LIVER_CANCER_SURVIVAL_UP	LEE_LIVER_CANCER_SURVIVAL_UP	70	-0.741932	-1.937063	4.966E-05	0.0078404	0.0074676	545	tags=23%, list=8%, signal=21%	Fbxo31/Slc38a3/Echdc2/Ndufs2/Dcxr/Cpn2/Apcs/F10/Serpinf1/Serpinc1/S100a8/Serpind1/Cpb2/Serping1/Rdh5/Apoc3
REACTOME_PLASMA_LIPOPROTEIN_ASSEMBLY	REACTOME_PLASMA_LIPOPROTEIN_ASSEMBLY	14	-0.922463	-1.88597	5.094E-05	0.0078404	0.0074676	200	tags=50%, list=3%, signal=49%	Apoc1/Apoa4/Apoa1/Apoa2/A2m/Apoc3/Apob
REACTOME_COMMON_PATHWAY_OF_FIBRIN_CLOT_FORMATION	REACTOME_COMMON_PATHWAY_OF_FIBRIN_CLOT_FORMATION	8	-0.964981	-1.801267	6.873E-05	0.0095612	0.0091067	185	tags=88%, list=3%, signal=85%	F10/Fga/F2/Fgb/Fgg/Serpinc1/Serpind1
BIOCARTA_FIBRINOLYSIS_PATHWAY	BIOCARTA_FIBRINOLYSIS_PATHWAY	6	-0.979358	-1.735447	6.943E-05	0.0095612	0.0091067	144	tags=100%, list=2%, signal=98%	F2/Fgb/Fgg/Plg/Cpb2
REACTOME_CHYLOMICRON_REMODELING	REACTOME_CHYLOMICRON_REMODELING	6	-0.978811	-1.734478	6.943E-05	0.0095612	0.0091067	122	tags=83%, list=2%, signal=82%	Apoa4/Apoa1/Apoa2/Apoc3/Apob
SENGUPTA_NASOPHARYNGEAL_CARCINOMA_WITH_LMP1_DN	SENGUPTA_NASOPHARYNGEAL_CARCINOMA_WITH_LMP1_DN	43	-0.789371	-1.912077	8.413E-05	0.0112885	0.0107518	238	tags=28%, list=4%, signal=27%	Clic6/Serpina3k/Ighm/Serpina1b/Serpina1e/S100a8/Gc/Mfap3l/Acacb/Cfb/Serpina3n/S100a9
WP_STATIN_INHIBITION_OF_CHOLESTEROL_PRODUCTION	WP_STATIN_INHIBITION_OF_CHOLESTEROL_PRODUCTION	13	-0.925819	-1.871287	0.0001188	0.0146955	0.0139968	269	tags=62%, list=4%, signal=59%	Dgat1/Lcat/Apoc1/Apoa4/Apoa1/Apoa2/Apoc3/Apob
REACTOME_INTRINSIC_PATHWAY_OF_FIBRIN_CLOT_FORMATION	REACTOME_INTRINSIC_PATHWAY_OF_FIBRIN_CLOT_FORMATION	10	-0.944302	-1.830554	0.0001191	0.0146955	0.0139968	185	tags=70%, list=3%, signal=68%	F10/F2/Serpinc1/Klkb1/Serpind1/A2m/Serping1
BIOCARTA_EXTRINSIC_PATHWAY	BIOCARTA_EXTRINSIC_PATHWAY	7	-0.971379	-1.770365	0.0001208	0.0146955	0.0139968	185	tags=86%, list=3%, signal=83%	F10/Fga/F2/Fgb/Fgg/Serpinc1
WP_COVID19_THROMBOSIS_AND_ANTICOAGULATION	WP_COVID19_THROMBOSIS_AND_ANTICOAGULATION	5	-0.979211	-1.680036	0.0002275	0.0247983	0.0236193	144	tags=100%, list=2%, signal=98%	F2/Fgb/Fgg/Plg
SERVITJA_LIVER_HNF1A_TARGETS_DN	SERVITJA_LIVER_HNF1A_TARGETS_DN	47	-0.760167	-1.866355	0.0002521	0.02638	0.0251258	245	tags=32%, list=4%, signal=31%	Cfh/Gbp2/Ddc/Apcs/Akr1c6/Fmo5/Plg/Serpina1b/Serpina1e/Hsd3b1/Cpb2/Serping1/Saa1/Afm/Hrg
REACTOME_ANTIGEN_PROCESSING_CROSS_PRESENTATION	REACTOME_ANTIGEN_PROCESSING_CROSS_PRESENTATION	71	-0.703412	-1.840195	0.0003143	0.0299076	0.0284857	361	tags=14%, list=5%, signal=13%	Myd88/Ubb/B2m/Fga/H2-Q10/Fgb/Fgg/Ncf1/S100a8/S100a9
OHGUCHI_LIVER_HNF4A_TARGETS_DN	OHGUCHI_LIVER_HNF4A_TARGETS_DN	39	-0.772774	-1.843045	0.0004223	0.0381008	0.0362894	319	tags=36%, list=5%, signal=34%	Enpp3/Cpn2/Ddc/Ctsh/Serpina3k/Apoa4/Serpinf1/Mug1/Serpina1b/Serpina1e/Hsd3b1/Rdh5/Serpina3n/Hrg
ICHIBA_GRAFT_VERSUS_HOST_DISEASE_35D_UP	ICHIBA_GRAFT_VERSUS_HOST_DISEASE_35D_UP	51	-0.736623	-1.831979	0.0004694	0.0402701	0.0383556	653	tags=39%, list=10%, signal=36%	Litaf/Itgb2/Tapbp/Snx10/C1qa/Csf1r/Tmsb4x/Lgmn/Apbb1ip/Gbp2/Nop2/Stx3/Serpina3k/Orm1/Hpx/S100a8/Cd5l/Lyz2/Serpina3n/S100a9
PILON_KLF1_TARGETS_UP	PILON_KLF1_TARGETS_UP	236	-0.531437	-1.622339	0.0004665	0.0402701	0.0383556	468	tags=15%, list=7%, signal=15%	C1qa/Sema6d/Dab2/Ebp/Fn1/C2cd2/Esam/Tlk1/Chac2/Apoh/Igfbp7/Ttr/Abcc9/Krt8/Col18a1/Nlk/Fga/Alb/Ahsg/F2/Hpx/Fgb/Fgg/Apoa1/Itih2/Apoa2/Plg/Serpinc1/Gc/Serpinf2/Serpina6/A2m/Serping1/Cfb/Afm/Apob
WP_ALTERNATIVE_PATHWAY_OF_FETAL_ANDROGEN_SYNTHESIS	WP_ALTERNATIVE_PATHWAY_OF_FETAL_ANDROGEN_SYNTHESIS	6	-0.964523	-1.709158	0.0005034	0.0418115	0.0398236	165	tags=67%, list=2%, signal=65%	Akr1c6/Hsd17b3/Hsd3b1/Cyp11a1
CHARAFE_BREAST_CANCER_LUMINAL_VS_MESENCHYMAL_DN	CHARAFE_BREAST_CANCER_LUMINAL_VS_MESENCHYMAL_DN	215	0.5125092	1.654115	0.0004967	0.0418115	0.0398236	376	tags=9%, list=6%, signal=9%	Socs3/Popdc3/Col6a1/Stx2/Col6a2/Plp2/Col4a2/Slc16a7/Ppp1r18/Dipk1a/Gid4/Stradb/Timp2/Eif5a2/Nap1l5/Sparc/Hrh1/Bag2/Gng11/Akap12
NAKAYAMA_SOFT_TISSUE_TUMORS_PCA1_UP	NAKAYAMA_SOFT_TISSUE_TUMORS_PCA1_UP	27	-0.816934	-1.840932	0.0005267	0.0430652	0.0410178	468	tags=52%, list=7%, signal=48%	C1qa/Csf1r/Cfh/Fhl2/Igkc/C3/Serpina1b/Ighg1/Serpina1e/Igkv12-41/Ighg2b/Lyz2/Cfb/Thbs1
ZHAN_MULTIPLE_MYELOMA_DN	ZHAN_MULTIPLE_MYELOMA_DN	18	-0.861759	-1.828048	0.000594	0.0457107	0.0435375	228	tags=56%, list=3%, signal=54%	Smpdl3a/Gldc/Hnmt/Itgb2/Aldh1a7/Apoc1/Ctsh/Lyz2/A2m/S100a9
BOYLAN_MULTIPLE_MYELOMA_PCA1_UP	BOYLAN_MULTIPLE_MYELOMA_PCA1_UP	43	-0.751041	-1.81923	0.0005889	0.0457107	0.0435375	130	tags=28%, list=2%, signal=28%	Ifitm3/Cp/Pawr/Serpina3k/C3/Ighg1/S100a8/Hp/Ighg2b/Lyz2/Serpina3n/S100a9
REACTOME_PARASITE_INFECTION	REACTOME_PARASITE_INFECTION	50	-0.730098	-1.810514	0.0005871	0.0457107	0.0435375	70	tags=12%, list=1%, signal=12%	Abl1/Ighg1/Igkv12-41/Ighg2b/Was/Ighv1-62-3
HUMMERICH_BENIGN_SKIN_TUMOR_UP	HUMMERICH_BENIGN_SKIN_TUMOR_UP	8	-0.939799	-1.754261	0.0007045	0.0526658	0.0501619	105	tags=50%, list=2%, signal=49%	Krt16/S100a8/Serpind1/S100a9
POOLA_INVASIVE_BREAST_CANCER_UP	POOLA_INVASIVE_BREAST_CANCER_UP	48	-0.730286	-1.799498	0.0007892	0.0581683	0.0554029	410	tags=27%, list=6%, signal=26%	Fn1/Il18/Rhof/Apoc1/Fcer1g/Ighm/Slc16a3/Slc18a2/Ighg1/Ighg2b/Lyz2/Serpina6/Ighv1-62-3
SMID_BREAST_CANCER_ERBB2_UP	SMID_BREAST_CANCER_ERBB2_UP	56	-0.708583	-1.78747	0.0008029	0.0583535	0.0555793	432	tags=18%, list=7%, signal=17%	Fa2h/Itgb6/Ddc/Gcat/Hpx/Fgb/Fgg/S100a8/Igkv12-41/S100a9
REACTOME_VISUAL_PHOTOTRANSDUCTION	REACTOME_VISUAL_PHOTOTRANSDUCTION	39	-0.756938	-1.805277	0.0009121	0.0600942	0.0572371	424	tags=26%, list=6%, signal=24%	Sag/Retsat/Ttr/Akr1c6/Apoa4/Apoa1/Apoa2/Rdh5/Apoc3/Apob
BURTON_ADIPOGENESIS_PEAK_AT_2HR	BURTON_ADIPOGENESIS_PEAK_AT_2HR	12	-0.902362	-1.799847	0.0008501	0.0600942	0.0572371	85	tags=25%, list=1%, signal=25%	Dnajb6/Rgs2/Thbs1
CHEOK_RESPONSE_TO_HD_MTX_UP	CHEOK_RESPONSE_TO_HD_MTX_UP	10	-0.91811	-1.77978	0.0009187	0.0600942	0.0572371	64	tags=40%, list=1%, signal=40%	Mylk/S100a8/Lyz2/S100a9
SMID_BREAST_CANCER_LUMINAL_B_DN	SMID_BREAST_CANCER_LUMINAL_B_DN	171	-0.559574	-1.647827	0.0009453	0.0601897	0.0573282	134	tags=11%, list=2%, signal=11%	Nfib/Igkc/Ighm/Wls/C3/H2-Q10/Krt16/Rgs2/Ighg1/Ncf1/S100a8/Igkv12-41/Ighg2b/Lyz2/Serpina1/Saa1/S100a9/Ighv1-62-3/Aqp1
PID_BARD1_PATHWAY	PID_BARD1_PATHWAY	8	0.9361784	1.8063977	0.0010047	0.0611341	0.0582277	64	tags=25%, list=1%, signal=25%	Prkdc/Xrcc5
RIGGINS_TAMOXIFEN_RESISTANCE_DN	RIGGINS_TAMOXIFEN_RESISTANCE_DN	117	-0.605062	-1.698049	0.0009932	0.0611341	0.0582277	632	tags=18%, list=10%, signal=17%	Cnn3/Scn1a/Mapkapk2/Tmem33/Ifitm3/Pdlim3/Palld/Isg15/Lgals3bp/Sp3/Ptpn12/Nnt/Serinc5/Myof/Serpina3k/Alb/Rgs2/S100a8/Mfap3l/Serpina3n/Gtf2e1
CHIANG_LIVER_CANCER_SUBCLASS_PROLIFERATION_DN	CHIANG_LIVER_CANCER_SUBCLASS_PROLIFERATION_DN	60	-0.693505	-1.767819	0.0010515	0.0625278	0.0595551	293	tags=23%, list=4%, signal=23%	Gne/Itih1/Aass/Gpld1/Sorbs2/Slc38a3/Echdc2/Dcxr/Col18a1/Plg/Serpinc1/Cpb2/Serping1/Afm
REACTOME_COLLAGEN_FORMATION	REACTOME_COLLAGEN_FORMATION	19	0.8415311	1.8428015	0.0011215	0.0651784	0.0620797	90	tags=26%, list=1%, signal=26%	Col6a1/Col6a2/Col4a2/Col1a1/Cd151
SARRIO_EPITHELIAL_MESENCHYMAL_TRANSITION_DN	SARRIO_EPITHELIAL_MESENCHYMAL_TRANSITION_DN	49	-0.718274	-1.77564	0.0011583	0.0651784	0.0620797	286	tags=22%, list=4%, signal=22%	Plekho2/Ifitm3/Stom/Gbp2/Lamb2/Serpina3k/Krt16/Rgs2/S100a8/Cfb/Serpina3n
REACTOME_DISSOLUTION_OF_FIBRIN_CLOT	REACTOME_DISSOLUTION_OF_FIBRIN_CLOT	7	-0.94448	-1.72134	0.0011385	0.0651784	0.0620797	75	tags=43%, list=1%, signal=42%	Plg/Serpinf2/Hrg
SHEDDEN_LUNG_CANCER_GOOD_SURVIVAL_A12	SHEDDEN_LUNG_CANCER_GOOD_SURVIVAL_A12	105	-0.615882	-1.70251	0.0011492	0.0651784	0.0620797	124	tags=10%, list=2%, signal=10%	Igkc/Ighm/Ppt2/Apoa2/Ighg1/Igkv12-41/Ighg2b/Atp2a3/Cfb/Saa1/Ighv1-62-3

TARTE_PLASMA_CELL_VS_PLASMABLAST_UP	TARTE_PLASMA_CELL_VS_PLASMABLAST_UP	162	-0.562794	-1.645979	0.0011863	0.0660403	0.0629006	521	tags=20%, list=8%, signal=19%	Phb2/Krt6a/Ifitm3/Kcnma1/Krt1/Dab2/Cd34/C4b/Lgmn/Rab32/Aldh1a7/Apoc1/B2m/Fcer1g/Krt32/Serpina3k/Igkc/H2-Q10/Jund/Rgs2/Itih4/Serpina1b/Serpina1e/S100a8/Hp/Lyz2/Serping1/Cyp2a5/Cfb/Serpina3n/Apoc3/S100a9
REACTOME_ASSEMBLY_OF_COLLAGEN_FIBRILS_AND_OTHER_MULTIMERIC_STRUCTURES	REACTOME_ASSEMBLY_OF_COLLAGEN_FIBRILS_AND_OTHER_MULTIMERIC_STRUCTURES	13	0.8854372	1.8290932	0.0012165	0.0663128	0.0631601	90	tags=38%, list=1%, signal=38%	Col6a1/Col6a2/Col4a2/Col1a1/Cd151
MEBARKI_HCC_PROGENITOR_FZD8CRD_DN	MEBARKI_HCC_PROGENITOR_FZD8CRD_DN	131	-0.587841	-1.674494	0.0012076	0.0663128	0.0631601	335	tags=18%, list=5%, signal=17%	C4b/Apbb1ip/Dgat1/Ddc/Abcc9/Apoc1/Ctsh/F10/Fga/Alb/Serpinf1/Hsd17b3/Orm1/Rab37/Fgb/Itih4/Serpina1b/Serpina1e/Klkb1/S100a8/Hp/Acacb/Afm
WP_PEROXIREDOXIN_2_INDUCED_OVARIAN_FAILURE	WP_PEROXIREDOXIN_2_INDUCED_OVARIAN_FAILURE	6	-0.954587	-1.691551	0.0012324	0.0664851	0.0633242	51	tags=50%, list=1%, signal=50%	Mapk10/Hsd3b1/Cyp11a1
ANDERSEN_LIVER_CANCER_KRT19_DN	ANDERSEN_LIVER_CANCER_KRT19_DN	33	-0.772805	-1.797804	0.0013198	0.0704733	0.0671228	446	tags=24%, list=7%, signal=23%	Gstm2/Retsat/Aldh1a7/Serpina3k/Serpind1/Serping1/Serpina3n/Afm
JINESH_BLEBBISHIELD_TO_IMMUNE_CELL_FUSION_PBSHMS_DN	JINESH_BLEBBISHIELD_TO_IMMUNE_CELL_FUSION_PBSHMS_DN	142	-0.57488	-1.654608	0.0014205	0.0743336	0.0707996	950	tags=23%, list=14%, signal=20%	Mtx2/Akr1b8/Slc25a23/Psmg1/Slc26a6/Anxa5/Cab39l/Gldc/Ifit3/Hnmt/Xdh/Csrp2/Tbc1d4/Rhobtb2/Ifitm3/Kit/Cp/C2cd2/Isg15/Cfh/Lgmn/Apoc1/Akr1c6/Nfib/Wls/C3/Rgs2/S100a8/Sgce/Cfb/S100a9/Afm
REACTOME_ROLE_OF_LAT2_NTAL_LAB_ON_CALCIUM_MOBILIZATION	REACTOME_ROLE_OF_LAT2_NTAL_LAB_ON_CALCIUM_MOBILIZATION	13	-0.881703	-1.782121	0.0016299	0.0805862	0.0767549	136	tags=31%, list=2%, signal=30%	Pik3ca/Fcer1g/Igkv12-41/Ighv1-62-3
KEGG_NON_HOMOLOGOUS_END_JOINING	KEGG_NON_HOMOLOGOUS_END_JOINING	5	0.9625638	1.7251424	0.0016102	0.0805862	0.0767549	64	tags=40%, list=1%, signal=40%	Prkdc/Xrcc5
WP_FAMILIAL_HYPERLIPIDEMIA_TYPE_2	WP_FAMILIAL_HYPERLIPIDEMIA_TYPE_2	7	-0.938452	-1.710355	0.0015871	0.0805862	0.0767549	256	tags=71%, list=4%, signal=69%	Lcat/Apoa4/Apoa1/Apoa2/Apob
HUMMERICH_MALIGNANT_SKIN_TUMOR_UP	HUMMERICH_MALIGNANT_SKIN_TUMOR_UP	7	-0.937476	-1.708576	0.0016906	0.0826794	0.0787486	153	tags=57%, list=2%, signal=56%	Col18a1/Krt16/S100a8/S100a9
WP_FAMILIAL_HYPERLIPIDEMIA_TYPE_3	WP_FAMILIAL_HYPERLIPIDEMIA_TYPE_3	8	-0.924717	-1.726108	0.0017183	0.0831054	0.0791544	256	tags=50%, list=4%, signal=48%	Lcat/Apoa4/Apoa1/Apoa2
REACTOME_CREATION_OF_C4_AND_C2_ACTIVATORS	REACTOME_CREATION_OF_C4_AND_C2_ACTIVATORS	7	-0.936352	-1.706527	0.0017768	0.0845278	0.0805091	70	tags=71%, list=1%, signal=71%	C1qa/Ighg1/Igkv12-41/Ighg2b/Ighv1-62-3
GAL_LEUKEMIC_STEM_CELL_UP	GAL_LEUKEMIC_STEM_CELL_UP	54	0.6792464	1.7830626	0.0019361	0.0904612	0.0861605	232	tags=13%, list=3%, signal=13%	Trim23/Col6a1/Kbtbd4/Pik3ip1/Gemin6/Itfg2/Pex1
PID_DNA_PK_PATHWAY	PID_DNA_PK_PATHWAY	7	0.9376596	1.7761076	0.001927	0.0904612	0.0861605	64	tags=29%, list=1%, signal=28%	Prkdc/Xrcc5
BLANCO_MELO_HUMAN_PARAINFLUENZA_VIRUS_3_INFECTION_A594_CELLS_DN	BLANCO_MELO_HUMAN_PARAINFLUENZA_VIRUS_3_INFECTION_A594_CELLS_DN	28	-0.7846	-1.777949	0.0019702	0.0912414	0.0869036	321	tags=25%, list=5%, signal=24%	Shc2/Cers4/Plekhh3/Rab37/Serpinf2/Serpina6/Rerg
GAUSSMANN_MLL_AF4_FUSION_TARGETS_D_UP	GAUSSMANN_MLL_AF4_FUSION_TARGETS_D_UP	20	0.8197376	1.8106607	0.0021724	0.0980003	0.0933411	315	tags=30%, list=5%, signal=29%	Popdc3/Vsir/Adra2c/Qpct/Adcyap1/Rab9b
WIELAND_UP_BY_HBV_INFECTION	WIELAND_UP_BY_HBV_INFECTION	32	-0.761666	-1.763726	0.0023203	0.1029013	0.0980091	245	tags=25%, list=4%, signal=24%	Gbp2/B2m/Fcer1g/H2-Q10/Ighg1/Cd5l/Ighg2b/Lyz2
CHANG_POU5F1_TARGETS_DN	CHANG_POU5F1_TARGETS_DN	5	0.9580799	1.7171061	0.0023803	0.104675	0.0996985	4	tags=20%, list=0%, signal=20%	Socs3
VERHAAK_AML_WITH_NPM1_MUTATED_UP	VERHAAK_AML_WITH_NPM1_MUTATED_UP	51	-0.696615	-1.73248	0.0024142	0.1052776	0.1002725	102	tags=22%, list=2%, signal=21%	Snx10/C1qa/Lgals3bp/Slc16a3/Gch1/Serpina1b/Ncf1/Serpina1e/Homer3/Cfb/Thbs1
KIM_LRRC3B_TARGETS	KIM_LRRC3B_TARGETS	13	-0.868606	-1.755647	0.0025297	0.1069086	0.1018259	347	tags=54%, list=5%, signal=51%	Tapbp/Isg15/B2m/H2-Q10/Serpina1b/Serpina1e/Lyz2
ICHIBA_GRAFT_VERSUS_HOST_DISEASE_D7_UP	ICHIBA_GRAFT_VERSUS_HOST_DISEASE_D7_UP	46	-0.710418	-1.737777	0.0026074	0.1085825	0.1034202	836	tags=37%, list=13%, signal=33%	C1qb/Ifit3/Xdh/Litaf/Tapbp/Snx10/Tmsb4x/Isg15/Lgals3bp/Krt18/Gbp2/Gbp7/Serpina3k/Ifit1/Cd5l/Lyz2/Serpina3n
SMID_BREAST_CANCER_NORMAL_LIKE_UP	SMID_BREAST_CANCER_NORMAL_LIKE_UP	128	-0.571453	-1.621918	0.0026767	0.1085825	0.1034202	374	tags=16%, list=6%, signal=16%	Krt5/Cnn1/Cfh/Mef2c/Aldh1a7/Aoc3/F10/Slc12a4/Col18a1/Igkc/Ighm/C3/Ighg1/Ncf1/Ighg2b/Lyz2/Acacb/Serping1/Atp2a3/Saa1/Aqp1
ACEVEDO_LIVER_CANCER_DN	ACEVEDO_LIVER_CANCER_DN	259	-0.490131	-1.510788	0.0026654	0.1085825	0.1034202	511	tags=13%, list=8%, signal=13%	Kank4/Cox4i1/Tmem256/Entpd5/Alpl/Mrpl53/Ndfip2/Retsat/Cers4/Man2b2/Dgat1/Trip6/Lcat/Ttr/Inmt/Krt8/Fga/Serpinf1/C3/Jund/Orm1/Nop53/Mug1/Apoa1/Itih2/Apoa2/Plg/Klkb1/Zbtb48/Serpinf2/Serpina6/Acacb/Rdh5/Apob
WP_FAMILIAL_HYPERLIPIDEMIA_TYPE_1	WP_FAMILIAL_HYPERLIPIDEMIA_TYPE_1	8	-0.916158	-1.710133	0.0028867	0.1135796	0.1081797	394	tags=62%, list=6%, signal=59%	Lmf1/Lcat/Apoa4/Apoa1/Apoa2
WP_FAMILIAL_HYPERLIPIDEMIA_TYPE_5	WP_FAMILIAL_HYPERLIPIDEMIA_TYPE_5	8	-0.916158	-1.710133	0.0028867	0.1135796	0.1081797	394	tags=62%, list=6%, signal=59%	Lmf1/Lcat/Apoa4/Apoa1/Apoa2
CLASPER_LYMPHATIC_VESSELS_DURING_METASTASIS_DN	CLASPER_LYMPHATIC_VESSELS_DURING_METASTASIS_DN	13	0.8648937	1.7866555	0.003017	0.1169462	0.1113863	22	tags=15%, list=0%, signal=15%	Col6a1/Col6a2
REACTOME_HDL_REMODELING	REACTOME_HDL_REMODELING	7	-0.927452	-1.690306	0.0031569	0.1214695	0.1156945	256	tags=57%, list=4%, signal=55%	Lcat/Alb/Apoa1/Apoc3
REACTOME_ANTIMICROBIAL_PEPTIDES	REACTOME_ANTIMICROBIAL_PEPTIDES	7	-0.926939	-1.689372	0.0032776	0.1225125	0.116688	64	tags=57%, list=1%, signal=57%	Pdzd11/S100a8/Lyz2/S100a9
MATTIOLI_MGUS_VS_PCL	MATTIOLI_MGUS_VS_PCL	70	-0.64272	-1.678039	0.0033275	0.1234945	0.1176233	193	tags=10%, list=3%, signal=10%	B2m/Jund/Ighg1/S100a8/Igkv12-41/Ighg2b/Ighv1-62-3
SERVITJA_ISLET_HNF1A_TARGETS_DN	SERVITJA_ISLET_HNF1A_TARGETS_DN	31	-0.756099	-1.741508	0.0034082	0.1256002	0.1196289	225	tags=26%, list=3%, signal=25%	Ndufs2/Ttr/Ddc/Rab37/Gc/Clic5/Cpb2/Hpgds
WILSON_PROTEASES_AT_TUMOR_BONE_INTERFACE_UP	WILSON_PROTEASES_AT_TUMOR_BONE_INTERFACE_UP	11	-0.879451	-1.729751	0.0036073	0.1292961	0.1231491	57	tags=27%, list=1%, signal=27%	Usp33/Serpind1/Serping1
REACTOME_SENSORY_PERCEPTION	REACTOME_SENSORY_PERCEPTION	94	-0.599415	-1.632033	0.0037642	0.1330963	0.1267686	539	tags=14%, list=8%, signal=13%	Ano2/Kcnma1/Sag/Retsat/Ttr/Akr1c6/Apoa4/Apoa1/Apoa2/Clic5/Rdh5/Apoc3/Apob
BOYAULT_LIVER_CANCER_SUBCLASS_G3_DN	BOYAULT_LIVER_CANCER_SUBCLASS_G3_DN	28	-0.764635	-1.732707	0.0039405	0.134966	0.1285494	839	tags=39%, list=13%, signal=34%	Gpld1/Slc25a20/Sorbs2/Fxyd6/Slc38a3/Cfh/Cpn2/Col18a1/Serpinf1/Cpb2/Serping1
WP_FAMILIAL_HYPERLIPIDEMIA_TYPE_4	WP_FAMILIAL_HYPERLIPIDEMIA_TYPE_4	9	-0.898687	-1.712727	0.0038947	0.134966	0.1285494	394	tags=56%, list=6%, signal=52%	Lmf1/Lcat/Apoa4/Apoa1/Apoa2
TUOMISTO_TUMOR_SUPPRESSION_BY_COL13A1_UP	TUOMISTO_TUMOR_SUPPRESSION_BY_COL13A1_UP	5	-0.949031	-1.628257	0.0039719	0.134966	0.1285494	73	tags=80%, list=1%, signal=79%	Rpl14/Serpina1b/Serpina1e/Lyz2
KEGG_BLADDER_CANCER	KEGG_BLADDER_CANCER	16	-0.833193	-1.737369	0.0043596	0.1462431	0.1392904	13	tags=19%, list=0%, signal=19%	Map2k1/Raf1/Thbs1
BLANCO_MELO_RESPIRATORY_SYNCYTIAL_VIRUS_INFECTION_A594_CELLS_DN	BLANCO_MELO_RESPIRATORY_SYNCYTIAL_VIRUS_INFECTION_A594_CELLS_DN	28	-0.760804	-1.724026	0.004535	0.1501989	0.143058	253	tags=32%, list=4%, signal=31%	Apoh/Mb/Ddc/Kcnj11/Akr1c6/Rab37/Serpinf2/Serpina6/Rerg
KEGG_CARDIAC_MUSCLE_CONTRACTION	KEGG_CARDIAC_MUSCLE_CONTRACTION	48	-0.687837	-1.694898	0.0045674	0.1503207	0.1431741	1152	tags=56%, list=17%, signal=47%	Cacnb2/Cacna2d1/Uqcrfs1/Cox6b1/Cox7a1/Uqcr10/Atp1a3/Uqcrb/Cox7a2l/Slc9a6/Uqcrc2/Cyc1/Cacng2/Uqcrh/Atp2a2/Atp1a1/Cacng8/Cox4i1/Atp1b1/Uqcrq/Cox6c/Cox5b/Cox5a/Atp1a4/Cox6a1/Cox7c/Cox7b
DESERT_PERIPORTAL_HEPATOCELLULAR_CARCINOMA_SUBCLASS_UP	DESERT_PERIPORTAL_HEPATOCELLULAR_CARCINOMA_SUBCLASS_UP	58	-0.659132	-1.67089	0.0049703	0.1605528	0.1529197	98	tags=22%, list=1%, signal=22%	Gpr88/Lcat/Hpx/Gch1/Apoa1/Itih4/Plg/Klkb1/Mfap3l/Acacb/Fetub/Afm/Hrg
DING_LUNG_CANCER_BY_MUTATION_RATE	DING_LUNG_CANCER_BY_MUTATION_RATE	10	0.8825538	1.7559304	0.0055309	0.1722807	0.1640901	12	tags=10%, list=0%, signal=10%	Prkdc
GAJATE_RESPONSE_TO_TRABECTEDIN_DN	GAJATE_RESPONSE_TO_TRABECTEDIN_DN	9	0.8938004	1.7516343	0.0057162	0.1753617	0.1670245	185	tags=22%, list=3%, signal=22%	Abcb6/Fars2
ACEVEDO_LIVER_TUMOR_VS_NORMAL_ADJACENT_TISSUE_DN	ACEVEDO_LIVER_TUMOR_VS_NORMAL_ADJACENT_TISSUE_DN	108	-0.571935	-1.58688	0.0057303	0.1753617	0.1670245	282	tags=13%, list=4%, signal=13%	Cers4/Man2b2/Trip6/Lcat/Inmt/C3/Jund/Rgs2/Plg/Klkb1/Zbtb48/Tbpl1/A2m/Afm
SANSOM_APC_TARGETS_DN	SANSOM_APC_TARGETS_DN	135	-0.544061	-1.556122	0.0057937	0.1753838	0.1670457	630	tags=21%, list=9%, signal=20%	Itgb2/Cx3cl1/Atp6v0a1/Galm/Atp1b1/Entpd5/Sipa1/Gstm2/Ndfip2/Lsp1/Il18/Mcfd2/Mgst3/Dgat1/Ace/Gpr89/Gbp2/Ddc/Kcnj11/Usp2/Alb/Ighm/Rgs2/Ighg1/Ncf1/Ighg2b/Atp2a3/Rdh5/Ighv1-62-3
KIM_LIVER_CANCER_POOR_SURVIVAL_DN	KIM_LIVER_CANCER_POOR_SURVIVAL_DN	17	-0.816486	-1.716976	0.0059568	0.1781261	0.1696576	344	tags=35%, list=5%, signal=34%	Iqgap2/F10/Plg/Serpinc1/Serpinf2/Apoc3
REACTOME_PLASMA_LIPOPROTEIN_CLEARANCE	REACTOME_PLASMA_LIPOPROTEIN_CLEARANCE	17	-0.815397	-1.714687	0.0062623	0.1820591	0.1734036	200	tags=24%, list=3%, signal=23%	Npc1/Apoc1/Apoa1/Apob
HOEGERKORP_CD44_TARGETS_TEMPORAL_DN	HOEGERKORP_CD44_TARGETS_TEMPORAL_DN	9	-0.887761	-1.691904	0.006252	0.1820591	0.1734036	44	tags=33%, list=1%, signal=33%	Mef2a/Lyz2/Apoc3
REACTOME_COLLAGEN_DEGRADATION	REACTOME_COLLAGEN_DEGRADATION	12	0.8580142	1.7521058	0.0065801	0.189195	0.1802002	54	tags=33%, list=1%, signal=33%	Col6a1/Col6a2/Col4a2/Col1a1
FONTAINE_FOLLICULAR_THYROID_ADENOMA_DN	FONTAINE_FOLLICULAR_THYROID_ADENOMA_DN	28	-0.748163	-1.695381	0.0068449	0.193618	0.1844129	38	tags=14%, list=1%, signal=14%	Lpar1/Vdac1/Was/Cox7b

This table lists significantly enriched Gene Set Enrichment Analysis (GSEA) pathways. "MPTP" refers to the acute MPTP model, "A53T" to the 16 months A53T-α-synuclein model, and "MitoPark" to the 5m MitoPark model.
