# Supplementary material for: Neurotoxicity, α-synuclein pathology, and mitochondrial dysfunction: A comparative study of different mouse models of Parkinson’s disease
Source: Neural Regen Res. 2025 Sep 29;21(10):5000–12. doi: 10.4103/NRR.NRR-D-25-00648 (PMC13568611; doi:10.4103/NRR.NRR-D-25-00648)
Supplement: Supplementary file 10 [file NRR-21-5000_Suppl9.pdf]

MPTP

@id	compartment::cytoskeleton	compartment::cytosol	compartment::endoplasmic reticulum	compartment::endosome	compartment::extracellular	compartment::golgi apparatus	compartment::lysosome	compartment::mitochondrion	compartment::nucleus	compartment::peroxisome	compartment::plasma membrane	display name	MCODE::Score (4)
stringdb:10090.ENSMUSP00000004378	2.973005	4.325015	1.945752	1.6387	2.470426	1.564001	1.925086	2.48648	2.658811	1.409707	3.430624	Eno2	4.761904762
stringdb:10090.ENSMUSP00000099881	2.816951	3.112491	1.828466	1.6788	2.293909	1.525427	1.713641	2.276165	3.304418	1.073964	5	Gap43	4.464285714
stringdb:10090.ENSMUSP00000033093	2.324492	5	5	1.639338	2.579166	1.557638	2.554044	5	4.571239	2.02636	2.85946	Bax	4
stringdb:10090.ENSMUSP00000009036	1.770051	3.336447	1.890267	1.292094	1.515784	1.176341	1.630348	5	2.495873	1.267408	1.800435	Vdac3	5
stringdb:10090.ENSMUSP00000000642	3.480203	4.604876	3.552263	1.965115	2.996419	1.803267	2.449961	4.739245	2.961558	2.798309	2.837444	Hk2	5
stringdb:10090.ENSMUSP00000055308	2.723113	3.040312	2.820611	2.188359	3.016529	2.057136	2.737	3.261483	5	2.702094	2.944404	Foxo1	4.690909091
stringdb:10090.ENSMUSP00000005493	2.855961	2.502121	2.207465	1.849163	2.987282	1.618035	2.053671	2.740277	2.665251	1.815808	5	Slc1a3	4
stringdb:10090.ENSMUSP00000086795	2.029143	3.899512	1.786699	1.531055	5	1.482051	1.719508	2.040215	2.664272	1.082629	3.274135	Lgals1	4
stringdb:10090.ENSMUSP00000028062	5	3.992512	2.005374	1.786206	2.493416	1.467659	1.872065	2.79559	4.399424	2.738272	5	Vim	4.491666667
stringdb:10090.ENSMUSP00000001051	2.245088	4.737698	1.565709	1.339944	2.155408	1.008352	1.39368	2.731717	3.899752	0.776788	4.390025	S100a6	4
stringdb:10090.ENSMUSP00000096148	1.616375	1.366436	1.783278	0.786367	1.906043	0.735083	0.922109	1.449184	1.566344		5	Scn1b	4
stringdb:10090.ENSMUSP00000071202		3.021848			0.46875			4.550866	2.731212		0.234375	Chchd1	5
stringdb:10090.ENSMUSP00000152412	1.177368	3.562148	0.706923		0.827155			2.020424	4.423451		0.644635	Gtpbp4	4.761904762
stringdb:10090.ENSMUSP00000032124	0.543688	0.665703			0.832165			4.642845	2.672817		0.736213	Mrpl19	4.285714286
stringdb:10090.ENSMUSP00000146501	2.00092	3.361868	1.667234	3.431289	2.067128	3.749544	2.210069	1.934748	4.349286	1.058501	5	Picalm	4
stringdb:10090.ENSMUSP00000109938	5	3.853013	2.281214	2.071591	2.408014	5	2.018066	2.395688	4.63524	1.302562	4.548176	Flna	4.375

A53T

display name	compartment::cytoskeleton	compartment::cytosol	compartment::endoplasmic reticulum	compartment::endosome	compartment::extracellular	compartment::golgi apparatus	compartment::lysosome	compartment::mitochondrion	compartment::nucleus	compartment::peroxisome	compartment::plasma membrane	MCODE::Score
Oxa1l		1.025302	0.52228		0.506031			4.699987	1.142971		2.70093	3.238095238
Sh3gl1	3.71413	4.028782	1.32828	3.842809	1.422843	1.940333	1.834918	1.449173	2.930341		1.921117	3
Napa	1.420931	3.192846	1.058056	0.998726	1.615851	1.275856	1.051153	1.33307	2.401868		2.881687	3
Nefm	5	3.062045	1.309034	1.13858	1.703324	0.839095	1.236682	1.863972	3.758279	0.78071	1.809469	2.7
Mrpl27					0.28125			4.57564				3.238095238
Cnih2	0.893842		3.490228	0.81084	2.337882	1.118671		0.629957	0.728529		4.678446	3
Fbxo3	1.24821	3.544306	0.997941	0.881559	1.259204	0.865052	1.361212	1.108492	3.554098	0.28125	1.268031	3
Mrps5		1.293834	0.818251		0.1875		0.762932	4.588938	1.390148			3.238095238
Rae1	4.334845	2.705464	1.295227	0.924224	1.63393	1.007613	1.077283	1.289534	4.615221		1.470186	2.4
Rars2	1.112267	1.94137	0.961284		0.877941		0.68477	4.615444	2.031522	1.577927	1.009167	3
Sh3gl2	1.798203	4.567639	1.469073	5	1.530197	1.73141	1.928611	1.78222	2.961969	0.637323	2.019175	2.7
Sec61a1	0.977499	1.43048	4.487618	0.995014	1.243583	1.147602	1.136137	1.393771	1.38104	0.851043	3.141663	4
Sec13	2.294743	3.6283	4.503445	2.016343	1.815738	2.564806	4.391959	1.524884	4.525272	1.183894	1.883245	2.7
Kcna2	1.998153	1.874961	3.746768	1.067639	2.190469	1.104662	1.163775	1.707679	1.95582	0.64899	5	3.238095238
Ppp1r9b	5	3.33016	1.361843	1.204136	1.561999	0.917466	0.942288	1.411106	3.046116		4.384186	2.7
Mrpl34		0.28125			1.03125			4.646248	0.28125			4

MitoPark

@id	compartment::cytoskeleton	compartment::cytosol	compartment::endoplasmic reticulum	compartment::endosome	compartment::extracellular	compartment::golgi apparatus	compartment::lysosome	compartment::mitochondrion	compartment::nucleus	compartment::peroxisome	compartment::plasma membrane	display name	MCODE::Score (5)	stringdb::database identifier
stringdb:10090.ENSMUSP00000000219	2.888668	3.710043	3.386917	1.957435	2.820562	1.79867	2.387174	3.368194	5	1.700489	3.635745	Th	0.5	10090.ENSMUSP00000000219
stringdb:10090.ENSMUSP00000098786	5	2.552521	5	4.030664	2.959999	5	3.634663	4.571903	5	1.789228	5	Psen1	0.285714286	10090.ENSMUSP00000098786
stringdb:10090.ENSMUSP00000019109	1.835195	4.713813	1.582117	1.20912	1.515793	1.032565	1.142746	3.13603	2.906263	0.698432	2.987455	Ywhah	0	10090.ENSMUSP00000019109
stringdb:10090.ENSMUSP00000155657	1.429033	5	1.364308	1.033358	1.366818	0.819404	1.258126	1.933287	1.830667	0.656337	1.421858	Rpl8	1.714285714	10090.ENSMUSP00000155657
stringdb:10090.ENSMUSP00000001166	0.938092	1.087863	0.877677		1.146971		0.346253	0.933219	0.92534		4.32935	Cnnm3	0	10090.ENSMUSP00000001166
stringdb:10090.ENSMUSP00000158269	2.120033	3.434165	2.485287	1.760341	2.572374	1.985998	2.024351	3.086426	4.514474	2.355462	2.905014	Cbs	0.666666667	10090.ENSMUSP00000158269
stringdb:10090.ENSMUSP00000002452	1.065707	1.990939	1.045482		2.392208			5	1.920976	0.751617	0.978396	Ndufa11	1.2	10090.ENSMUSP00000002452
stringdb:10090.ENSMUSP00000136247	0.878855	3.15328	1.34559	1.297572	1.443745	0.639266	1.330782	5	4.371123	1.20601	1.559307	Ecsit	2	10090.ENSMUSP00000136247
stringdb:10090.ENSMUSP00000047960	1.18973	1.318877	0.980392	0.623482	1.118618		0.800858	5	1.445194	0.85636	1.096517	Atp5l	3	10090.ENSMUSP00000047960
stringdb:10090.ENSMUSP00000130532	0.787504	1.197138	0.914584		0.768401		0.523569	4.629409	2.726328	0.899291	0.697155	Ndufaf2	2	10090.ENSMUSP00000130532
stringdb:10090.ENSMUSP00000115419	1.123581	1.490396	1.215906	0.701744	1.306167		0.911398	5	1.875741	1.126177	1.244466	Cox7c	3	10090.ENSMUSP00000115419
stringdb:10090.ENSMUSP00000014370	1.527769	3.491887	1.142245	0.812824	1.1688	0.794704	1.027514	1.245494	5		1.23479	Cacybp	0.666666667	10090.ENSMUSP00000014370
stringdb:10090.ENSMUSP00000111309	5	3.962457	1.209234	1.040673	1.370817	1.121596	0.845399	1.712064	2.169636		1.372435	Ranbp1	2	10090.ENSMUSP00000111309
stringdb:10090.ENSMUSP00000066621	1.173559	4.393393	1.113938	0.549559	0.967588			2.00014	3.356912		0.930536	Glrx3	0.666666667	10090.ENSMUSP00000066621
stringdb:10090.ENSMUSP00000015100	1.653182	4.082389	1.835173	1.729046	1.588359	1.314381	1.247406	1.45607	3.832646	0.718687	1.723357	Ppp1cb	0.666666667	10090.ENSMUSP00000015100
stringdb:10090.ENSMUSP00000138618	1.109185	1.027176	0.985156	0.858516	1.27012	0.747859		0.938584	1.148067		5	Cacng8	0.666666667	10090.ENSMUSP00000138618

stringdb:10090.ENSMUSP00000103402	5	2.800514	1.45957	0.846481	1.243206	0.791741	0.994056	1.405736	3.57502		1.821173	Tmod1	4	10090.ENSMUSP00000103402	[illegible]	[illegible]
stringdb:10090.ENSMUSP00000022369	5	3.987867	2.305211	2.127901	2.843021	1.957913	2.167438	2.432823	3.009236	2.086767	5	Vcl	4.608333333	10090.ENSMUSP00000022369	[illegible]	[illegible]
stringdb:10090.ENSMUSP00000023165	2.558969	2.740186	2.356509	1.930272	2.582488	1.82405	2.129632	2.714209	5	2.217125	2.620741	Crebbp	4.636363636	10090.ENSMUSP00000023165	[illegible]	[illegible]
stringdb:10090.ENSMUSP00000004949	2.472607	5	2.539878	2.820158	2.992787	2.029339	2.74291	2.801217	4.74379	1.971178	4.601779	Traf6	4.464285714	10090.ENSMUSP00000004949	[illegible]	[illegible]
stringdb:10090.ENSMUSP00000004377	1.713735	4.684117	1.604902	1.235696	1.915018	0.792703	1.324423	1.571476	3.802453	0.82668	5	Ptpn6	4.761904762	10090.ENSMUSP00000004377	[illegible]	[illegible]
stringdb:10090.ENSMUSP00000079991	2.426035	4.778748	2.119761	1.963176	2.373262	1.294466	1.98432	1.94223	3.245288	0.935524	4.558688	Plcg2	4	10090.ENSMUSP00000079991	[illegible]	[illegible]
stringdb:10090.ENSMUSP00000147784	1.80187	4.43221	2.019715	1.462518	1.89981	0.821542	1.253727	1.446956	3.424421	0.757021	2.10821	Gab1	4.761904762	10090.ENSMUSP00000147784	[illegible]	[illegible]
stringdb:10090.ENSMUSP00000014290	3.473959	5	0.534799		0.828833			0.998289	2.400019		5	Apbb1ip	4	10090.ENSMUSP00000014290	[illegible]	[illegible]
stringdb:10090.ENSMUSP00000082682	2.613648	3.244275	3.694034	5	4.680697	1.947483	2.80908	3.066495	4.87312	2.083909	4.247391	Hmgb1	4.464285714	10090.ENSMUSP00000082682	[illegible]	[illegible]
stringdb:10090.ENSMUSP00000009727	1.10476	0.936745	0.644328	1.00869	1.243878	0.86335	0.961715	1.086815	1.106776		3.516194	Syngr1	4	10090.ENSMUSP00000009727	[illegible]	[illegible]
stringdb:10090.ENSMUSP00000020024	2.631962	4.741898	1.670997	1.196731	2.553494	1.087546	1.481368	2.090145	2.966591	1.870113	2.650803	Fabp7	4	10090.ENSMUSP00000020024	[illegible]	[illegible]
stringdb:10090.ENSMUSP00000114304	2.51976	5	1.462415	1.030325	1.998106	0.889718	1.454398	4.475121	2.945628	1.413743	1.952876	Aldh1l1	4	10090.ENSMUSP00000114304	[illegible]	[illegible]
stringdb:10090.ENSMUSP00000070019	4.393655	4.763146	1.878388	1.50883	2.081497	1.085633	1.688088	2.174754	5	1.356556	5	Prkcb	4.027777778	10090.ENSMUSP00000070019	[illegible]	[illegible]
stringdb:10090.ENSMUSP00000033075	1.978612	3.593635	1.871489	3.463204	3.520614	5	2.245532	1.736059	1.677041	0.80069	5	Stx4a	4.285714286	10090.ENSMUSP00000033075	[illegible]	[illegible]
stringdb:10090.ENSMUSP00000074448	2.63931	2.938632	2.740106	5	3.770626	2.654308	5	2.843164	2.733286	2.629226	4.57584	Lamp2	4	10090.ENSMUSP00000074448	[illegible]	[illegible]
stringdb:10090.ENSMUSP00000048770	1.025578	5	0.782923	1.623113	1.072272	0.720299	5	1.421687	2.522455		5	Atp6v1g1	4	10090.ENSMUSP00000048770	[illegible]	[illegible]

stringdb:10090.ENSMUSP00000046748	1.337657	5			0.891511	0.501694		0.756364	2.243285		5	Cplx3	3	10090.ENSMUSP00000046748	[illegible]	[illegible]
stringdb:10090.ENSMUSP00000048719	1.46835	1.811807	0.786319		1.083077			1.013142	3.452145		1.394027	Rims2	2.4	10090.ENSMUSP00000048719	[illegible]	[illegible]
stringdb:10090.ENSMUSP00000055225	2.002973	4.362404	4.418638	1.102702	2.130602	1.267395	1.296642	1.916235	2.780747	0.951049	5	Kcna1	3.238095238	10090.ENSMUSP00000055225	[illegible]	[illegible]
stringdb:10090.ENSMUSP00000057989	3.301113	2.935442	4.5521	2.543635	4.855076	2.349755	2.647071	3.984671	4.062456	2.240888	3.619107	Bdnf	4	10090.ENSMUSP00000057989	[illegible]	[illegible]
stringdb:10090.ENSMUSP00000064280	3.325864	4.552377	3.732433	1.676062	1.830969	3.133745	1.26958	1.454034	3.920308		5	Dlg1	4	10090.ENSMUSP00000064280	[illegible]	[illegible]
stringdb:10090.ENSMUSP00000065836	1.431579	1.811302	1.030028	0.64772	0.507682	0.8274		0.682104	4.348883		3.131394	Nup188	2.4	10090.ENSMUSP00000065836	[illegible]	[illegible]
stringdb:10090.ENSMUSP00000073591	0.521141		0.819032		3.344681	0.729824		0.507559	0.933479	0.407974	2.964185	Gsg1l	3	10090.ENSMUSP00000073591	[illegible]	[illegible]
stringdb:10090.ENSMUSP00000073832	0.501826	2.068682			0.589008			4.593276	3.601257	0.484375	0.574147	Ndufaf3	3	10090.ENSMUSP00000073832	[illegible]	[illegible]
stringdb:10090.ENSMUSP00000077532	1.351037	4.650879	1.061754	0.622563	1.196041		0.74895	5	1.769325		1.291032	Ppp3cc	2.4	10090.ENSMUSP00000077532	[illegible]	[illegible]
stringdb:10090.ENSMUSP00000080842	3.876722	1.893898	1.669485	1.165751	2.15172	1.168317	1.148173	1.776538	2.026118	0.599016	4.806107	Scn8a	2.7	10090.ENSMUSP00000080842	[illegible]	[illegible]
stringdb:10090.ENSMUSP00000081000	1.669035	2.080222	2.064493	1.148692	1.798441	0.833185	1.483606	5	2.424301	1.39866	3.529683	mt-Nd4	4	10090.ENSMUSP00000081000	[illegible]	[illegible]
stringdb:10090.ENSMUSP00000081003	1.91267	2.383773	1.907775	1.404354	2.083054	1.1628	1.801876	5	2.693586	1.798524	3.840536	mt-Cytb	4	10090.ENSMUSP00000081003	[illegible]	[illegible]
stringdb:10090.ENSMUSP00000082489	2.620076	1.992643	1.788901	1.87454	2.492261	1.607641	1.827789	2.356886	2.617038	1.08811	4.626663	Slc17a7	4	10090.ENSMUSP00000082489	[illegible]	[illegible]
stringdb:10090.ENSMUSP00000095322			0.84375					4.432915	0.625	0.578125		Mrpl43	4	10090.ENSMUSP00000095322	[illegible]	[illegible]
stringdb:10090.ENSMUSP00000096193	1.207807	1.437818	1.20797		1.648615		0.795692	4.796751	1.939529	1.441375	1.22988	Cox7a1	4	10090.ENSMUSP00000096193	[illegible]	[illegible]
stringdb:10090.ENSMUSP00000099944	1.964612	4.718266	1.913882	1.438815	2.020315	1.328203	1.557887	2.505962	2.358568	1.97194	4.415667	Ppp3r1	2.4	10090.ENSMUSP00000099944	[illegible]	[illegible]

[illegible]	5	3.881727	2.096345	2.011402	2.507106	3.823576	1.998141	2.771626	3.465303	1.497277	5	Rock1	0.5	[illegible]	[illegible]	[illegible]
[illegible]	0.841039	1.36023	1.146129		1.215091		0.759079	4.743783	2.35504	0.57623	2.624282	Atp5o	2.2	[illegible]	[illegible]	[illegible]
[illegible]	1.728324	2.528699	3.377337	0.582119	2.032343		0.837509	1.252612	3.948639		1.084351	Eif5a2	3	[illegible]	[illegible]	[illegible]
[illegible]	1.842175	2.912379	1.829695	1.24522	1.806962	0.948474	1.593224	5	2.078754	1.483311	3.70468	Atp5a1	2.2	[illegible]	[illegible]	[illegible]
[illegible]		1.59375						4.178267	2.871875			Mrps23	3	[illegible]	[illegible]	[illegible]
[illegible]		0.5						5	1.984415			Mrpl51	3	[illegible]	[illegible]	[illegible]
[illegible]	1.744223	3.127281	1.364272	1.014148	1.90273	0.813386	1.044403	4.703116	3.485432	1.026302	1.659712	Sdhaf2	0	[illegible]	[illegible]	[illegible]
[illegible]	0.759952	1.495957			0.537427			4.649313	2.951666	1.03125		Mrps18b	3	[illegible]	[illegible]	[illegible]
[illegible]	1.493986	4.072938	1.28417	3.984014	1.2313	1.840651	1.996378	1.367452	3.54099	0.606169	1.717774	Rabgef1	0	[illegible]	[illegible]	[illegible]
[illegible]	1.426276	2.258773	5	1.221788	1.750497	1.580947	1.362848	1.476364	1.502152	0.67302	2.1831	Dnajc10	0.666666667	[illegible]	[illegible]	[illegible]
[illegible]	1.516127	5	1.039831	4.198227	1.110502	0.572262	1.288418	1.084198	5		4.239646	Mkln1	0	[illegible]	[illegible]	[illegible]
[illegible]	1.695473	3.847843	0.608749	0.595646	1.062767	0.889058	0.854453	0.863133	4.542094		3.494492	Ranbp9	0.666666667	[illegible]	[illegible]	[illegible]
[illegible]	0.921941	3.257366	3.748796	0.909205	1.186789	0.975489	1.176471	1.50611	3.945468	0.592427	1.054702	Ubxn4	0	[illegible]	[illegible]	[illegible]
[illegible]	2.134414	4.118517	2.122583	2.023649	1.635474	1.645851	2.248294	2.176251	4.241447	1.068303	3.054374	Ubqln2	0.285714286	[illegible]	[illegible]	[illegible]
[illegible]	1.848176	2.206332	1.957249	1.362416	1.902777	1.340062	1.590761	2.478817	1.999672	1.065074	5	Atp1b1	0.666666667	[illegible]	[illegible]	[illegible]
[illegible]	2.356324	2.521796	3.267434	2.148376	2.5981	1.823945	2.150603	3.447461	3.42223	1.378207	5	Slc9a1	0.5	[illegible]	[illegible]	[illegible]

stringdb:10090.ENSMUSP00000033131	1.394313	2.704859	1.408292	4.506262	1.591745	1.234268	5	1.656929	2.836145	0.9502	1.529851	Lamtor1	4	10090.ENSMUSP00000033131	[illegible]	5y39_F|AF-[illegible]
stringdb:10090.ENSMUSP00000039013	1.321221	3.221053	1.438281	2.468417	1.462721	1.184643	4.530077	1.669506	2.561804	0.939934	1.528022	Rragb	4	10090.ENSMUSP00000039013	[illegible]	AF-Q6NTA4
stringdb:10090.ENSMUSP00000129767	1.35168	4.629342	1.359157	0.800006	1.568395	0.527752	1.215243	1.900521	4.471022	1.05289	1.298251	Psma2	4	10090.ENSMUSP00000129767	[illegible]	4r67_B|AF-P49722
stringdb:10090.ENSMUSP00000132945	1.550241	4.030625	1.033894	1.484076	1.091479	1.361018	1.292187	1.092043	2.488893		3.587329	Rab3c	4	10090.ENSMUSP00000132945	[illegible]	4rkf_A|6y7g_B|AF-P62823
stringdb:10090.ENSMUSP00000082822	5	3.201963	1.04459	1.358867	1.274226	1.062121	1.5204	1.481146	3.985974		1.663047	Arpc1b	4	10090.ENSMUSP00000082822	[illegible]	7tpt_C|AF-Q9WV32
stringdb:10090.ENSMUSP00000105719	3.684276	4.473257	1.704445	5	1.788388	2.072417	3.796915	1.810623	1.769184	0.568649	3.905092	Rab3a	4.107142857	10090.ENSMUSP00000105719	[illegible]	1zbd_A|3rab_A|AF-P63011
stringdb:10090.ENSMUSP00000121111	3.202256	5	2.974867	5	1.932594	5	1.499296	5	5	1.078381	3.22209	Map2k2	4	10090.ENSMUSP00000121111	[illegible]	1jky[illegible]|AF-Q63932
stringdb:10090.ENSMUSP00000024978	5	1.266469	1.202642		3.202302			4.34159	1.062306	1.218944	1.038167	Nme3	5	10090.ENSMUSP00000024978	[illegible]	1zs6_A|AF-Q9WV85
stringdb:10090.ENSMUSP00000017408	1.21107	4.254386	1.14969	0.615711	1.584113		0.794414	1.308636	4.757947		1.038234	Exosc10	4.761904762	10090.ENSMUSP00000017408	[illegible]	2cpr[illegible]|AF-P56960
stringdb:10090.ENSMUSP00000075573		1.6875	2						4.600836			Pum3	4.761904762	10090.ENSMUSP00000075573	[illegible]	4wzr_A|AF-Q8BKS9
stringdb:10090.ENSMUSP00000027464		1.5						4.573938	2		2	Mrpl44	4.285714286	10090.ENSMUSP00000027464	[illegible]	4ce4_h|7of0_c|7qh7_c|AF-Q9CY73
stringdb:10090.ENSMUSP00000029142	5	2.487276	1.447271	0.522784	2.527656	0.662577	0.940155	1.641866	5	0.421875	1.228503	Eif6	5	10090.ENSMUSP00000029142	[illegible]	AF-O55135
stringdb:10090.ENSMUSP00000033290		1.876184						3.362353	3.866111			Dhx32	5	10090.ENSMUSP00000033290	[illegible]	AF-Q8BZS9
stringdb:10090.ENSMUSP00000022855	0.584841	2.047842	0.706623					0.774074	4.301036		0.538249	Brix1	4.761904762	10090.ENSMUSP00000022855	[illegible]	AF-Q9DCA5
stringdb:10090.ENSMUSP00000081782	1.640874	3.930451	1.281557	1.21529	1.262771	0.878484	1.182808	1.68998	5	0.28125	2.052548	Ipo7	4	10090.ENSMUSP00000081782	[illegible]	6n88_A|AF-Q9EPL8
stringdb:10090.ENSMUSP00000046759	1.397576	2.883757	1.238184	1.050124	1.490421	0.87752	1.342929	1.852507	4.149121	0.910106	4.298588	Ipo8	4	10090.ENSMUSP00000046759	[illegible]	AF-Q7TMY7

[illegible]	Camk2a	[illegible]	1hkx_A|6ts3_D|6w4o_A|7b56_B|7ujq_A|AF-P11798
[illegible]	Sec61a2	[illegible]	
[illegible]	Plcb1	[illegible]	
[illegible]	Sumo2	[illegible]	2rpq_A|5tvp_Q|AF-P61957
[illegible]	Kcnab2	[illegible]	
[illegible]	Nrxn1	[illegible]	2h0b_A|2r16_A|2r1b_A|3asi_A|3poy_A|3r05_A|6cw1_A|AF-Q9CS84
[illegible]	Abhd6	[illegible]	
[illegible]	Shisa9	[illegible]	AF-Q9CZN4
[illegible]	Ndufa12	[illegible]	6ztq_q|AF-Q7TMF3
[illegible]	Ank3	[illegible]	4o6x_A|6m3p_E|AF-G5E8K5
[illegible]	Camk2d	[illegible]	
[illegible]	Ndufa9	[illegible]	6ztq_P|AF-Q9DC69
[illegible]	Ppp4c	[illegible]	
[illegible]	Calm1	[illegible]	6hr1_A

[illegible]	Rab5if	[illegible]	AF-Q9CQT9
[illegible]	Gng11	[illegible]	AF-P61952
[illegible]	Chrm2	[illegible]	AF-Q9ERZ4
[illegible]	Was	[illegible]	1cee_B|1t84_A|2a3z_C|AF-P70315
[illegible]	Shisa4	[illegible]	AF-Q8CA71
[illegible]	Nap1l1	[illegible]	7ue3_B|AF-P28656
[illegible]	Kcnc1	[illegible]	7jmk_A|AF-P15388
[illegible]	Tia1	[illegible]	2mjn_A|2rne_A|5ith_A|5o2v_A|5o3j_A|AF-P52912
[illegible]	Tgoln1	[illegible]	AF-Q62313
[illegible]	Bola1	[illegible]	1v60_A|AF-Q9D8S9
[illegible]	Plekhb1	[illegible]	2dhy_A|AF-Q9QYE9
[illegible]	Ybx1	[illegible]	1h95_A|6lmr_A|AF-P62960
[illegible]	Nufip2	[illegible]	AF-Q5F2E7
[illegible]	Cpeb3	[illegible]	2rug_A|AF-Q7TN99
[illegible]	Sf3b5	[illegible]	6ff7_a|AF-Q923D4
[illegible]	Mtch1	[illegible]	AF-Q791T5

stringdb:10090.ENSMUSP00000022281	0.653192	1.6875	0.593845					0.921875	4.567211	0.375		Mtrex	4.761904762	10090.ENSMUSP00000022281	Exosome RNA helicase MTR4; Catalyzes the ATP-dependent unwinding of RNA duplexes with a single-stranded 3' RNA extension. Central subunit of many protein complexes, namely TRAMP-like, nuclear exosome targeting (NEXT) and poly(A) tail exosome targeting (PAXT). NEXT functions as an RNA exosome cofactor that directs a subset of non-coding short-lived RNAs for exosomal degradation. NEXT is involved in surveillance and turnover of aberrant transcripts and non-coding RNAs. PAXT directs a subset of long and polyadenylated	label: attribute="display name" labelsize=12 labelAlignment=left position=northeast outline=true outlineColor=white outlineTransparency=95 outlineWidth=10 background=false color=black dropShadow=false	https://version-12-0.string-db.org//images/Protein pictures/pdb/6d/6d6r_M.png	rgb(117,229,239)	MADAFGDELFSVFEDDSTSAAGAKKDKEKEKWKGPPGSADKAGKRLDTKLQSESASGGKNKRDLDVEGTDEPIFGKKPRIEDSINEDLSLADLMPRVKVQSVETVEGCTHEVALPADEDYIPLKPRVGKAAKEYPFILDAFQREAIQCVDNNQSVLVSAHTSAGKTVCAEYAIALALREKQRVIFTSPIKALSNQKYREMYEEFQDVGLMTGDVTINPTASCLVMTTEILRSMLYRGSEVMREVAWVIFDEIHYMRDSERGVVWEETIILLPDNVHYVFLSATIPNARQFAEWICHLHKQPCHVIYTDYRPTPLQHYIFPAGGDGLHLVVDENGDFREDNFNTAMQVLRDAGDLAKGDQKGRKGGTKGPSNVFKIVKMIMERNFQPVIIFSFSKKDCEAYALQMTKLDFNTDEEKKMVEEVFNNAIDCLSDEDKKLPQVEHVLPLLKRGI	6d6r_M|AF-Q9CZU3
stringdb:10090.ENSMUSP00000080257	1.914812	1.87158	1.996094	1.046495	1.988644	1.376252	1.512906	1.717758	2.055373	0.62478	5	Kcnd2	4	10090.ENSMUSP00000080257	Potassium voltage-gated channel subfamily D member 2; Voltage-gated potassium channel that mediates transmembrane potassium transport in excitable membranes, primarily in the brain, but also in rodent heart. Mediates the major part of the dendritic A-type current I(SA) in brain neurons. This current is activated at membrane potentials that are below the threshold for action potentials. It regulates neuronal excitability, prolongs the latency before the first spike in a series of action potentials, regulates the frequency of repetitive action	label: attribute="display name" labelsize=12 labelAlignment=left position=northeast outline=true outlineColor=white outlineTransparency=95 outlineWidth=10 background=false color=black dropShadow=false	https://version-12-0.string-db.org//images/Protein pictures/pdb/7f/7f3f_A.png	rgb(239,237,117)	MAAGVAAWLPFARAAAIGWMPVASGPMPAPPRQERKRTQDALIVLNVSGTRFQTWQDTLERYPDTLLGSSERDFFYHPETQQYFFDRDPDIFRHILNFYRTGKLHYPRHECISAYDEELAFFGLIPEIIGDCCYEEYKDRRRENAERLQDDADTDNTGESALPTMTARQRVWRAFENPHTSTMALVFYYVTGFFIAVSVIANVVETVPCGSSPGHIKELPCGERYAVAFFCLDTACVMIFTVEYLLRLAAAPSRYRFVRSVMSIIDVVAILPYYIGLVMTDNEDVSGAFVTLRVFRVFRIFKFSRHSQGLRILGYTLKSCASELGFLLFSLTMAIIIFATVMFYAEKGSSASKFTSIPAAFWYTIVTMTTLGYGDMVPKTIAGKIFGSICSLSGVLVIALPVPVIVSNFSRIYHQNQRADKRRAQKKARLARIRAAKSGSANAYMQSKRNGLLSNQL	1nn7_A|1s6c_B|7e7z_A|7e87_B|7e8e_C|7f3f_A|AF-Q9Z0V2
stringdb:10090.ENSMUSP00000086485	1.57053	4.331202	1.482067	0.73509	1.58513	0.690582	0.978723	1.950947	2.539126	1.08937	1.888656	Pde10a	4	10090.ENSMUSP00000086485	cAMP and cAMP-inhibited cGMP 3',5'-cyclic phosphodiesterase 10A; Plays a role in signal transduction by regulating the intracellular concentration of cyclic nucleotides. Can hydrolyze both cAMP and cGMP, but has higher affinity for cAMP and is more efficient with cAMP as substrate. May play a critical role in regulating cAMP and cGMP levels in the striatum, a region of the brain that contributes to the control of movement and cognition.	label: attribute="display name" labelsize=12 labelAlignment=left position=northeast outline=true outlineColor=white outlineTransparency=95 outlineWidth=10 background=false color=black dropShadow=false	https://version-12-0.string-db.org//images/Protein pictures/sm/515/5153b3eb488159df60e57c36.png	rgb(239,207,117)	MEDGPSNNASCFRRLTECFLSPSLTDEKVKAYLSLHPQVLDEFVSESVSAETVEKWLKRKTNKAKDEPSPKEVSRYQDTNMQGVVYELNSYIEQRLDTGGDNHLLLYELSSIIRIATKADGFALYFLGECNNSLCVFIPPGMKEGQPRLIPAGPITQGTTISAYVAKSRKTLLVEDILGDERFPRGTGLESGTRIQSVLCLPIVTAIGDLIGILELYRHWGKEAFCLSHQEVATANLAWASVAIHQVQVCRGLAKQTELNDFLLDVSKTYFDNIVAIDSLLEHIMIYAKNLVNADRCALFQVDHKNKELYSDLFDIGEEKEGKPIFKKTKEIRFSIEKGIAGQVARTGEVLNIPDAYADPRFNREVDLYTGYTTRNILCMPIVSRGSVIGVVQMVNKISGSAFSKTDENNFKMFAVFCALALHCANMYHRIRHSECIYRVTMEKLSYHSICTSEEWQ	2zmf_A|3lxg_A|5ede_A|AF-Q8CA95
stringdb:10090.ENSMUSP00000156199	1.822914	1.508029	1.237627	0.823938	1.742851	0.883593	1.050421	5	5	0.679651	1.529755	Hmga1	4	10090.ENSMUSP00000156199	High mobility group AT-hook 1.	label: attribute="display name" labelsize=12 labelAlignment=left position=northeast outline=true outlineColor=white outlineTransparency=95 outlineWidth=10 background=false color=black dropShadow=false		rgb(117,117,239)	MSESGSKSSQPLASKQEKDGTEKRGRGRPRKQPPVSPGTALVGSQKEPSEVPTPKRPRGRPKGSKNKGAAKTRKVTTAPGRKPRGRPKKLEKEEEEGISQESSEEEQ	
stringdb:10090.ENSMUSP00000069752	1.688976	3.321139	1.290945	1.320038	1.466741	3.462797	1.321356	1.829096	2.563162	0.854974	3.725089	Rras2	4	10090.ENSMUSP00000069752	Ras-related protein R-Ras2; It is a plasma membrane-associated GTP-binding protein with GTPase activity. Might transduce growth inhibitory signals across the cell membrane, exerting its effect through an effector shared with the Ras proteins but in an antagonistic fashion.	label: attribute="display name" labelsize=12 labelAlignment=left position=northeast outline=true outlineColor=white outlineTransparency=95 outlineWidth=10 background=false color=black dropShadow=false	https://version-12-0.string-db.org//images/Protein pictures/pdb/2e/2ery_A.png	rgb(117,117,239)	MAAAGWRDGSGQEKYRLVVVGGGGVGKSALTIQFIQSYFVTDYDPTIEDSYTKQCVIDDRAARLDILDTAGQEEFGAMREQYMRTGEGFLLVFSVTDRGSFEEIYKFQRQILRVKDRDEFPMILIGNKADLDHQRQVTQEEGQQLARQLKVTYMEASAKIRMNVDQAFHELVRVIRKFQEQECPPSPEPTRKEKDKKGCHCVIF	2ery_A|AF-P62071
stringdb:10090.ENSMUSP00000023007	1.630456	2.716208	1.738797	0.913521	1.708737	0.859425	0.913169	1.561618	1.791193		5	Adcy8	4.761904762	10090.ENSMUSP00000023007	Adenylate cyclase type 8; Catalyzes the formation of cAMP in response to calcium entry leadings to cAMP signaling activation that affect processes suche as synaptic plasticity and insulin secretion. Plays a role in many brain functions, such as learning, memory, drug addiction, and anxiety modulation through regulation of synaptic plasticity by modulating long-term memory and long-term potentiation (LTP) through CREB transcription factor activity modulation. Plays a central role in insulin secretion by controlling glucose homeostasis through glucagon-like peptide 1	label: attribute="display name" labelsize=12 labelAlignment=left position=northeast outline=true outlineColor=white outlineTransparency=95 outlineWidth=10 background=false color=black dropShadow=false	https://version-12-0.string-db.org//images/Protein pictures/sm/b76/b7697853026d51a2f29e2b36.png	rgb(239,136,117)	MELSDVHCLSGSEELYTIQPTPPAGDDGSSRPQRLLWQTAVRHITEQRFIHGHRGGGGGGVSRKASNPAGSGPNHHAPQLSSDSVLPLYSLGPGERAHNTGGTKVFPERSGSGSASGSGGGGDLGFLHLDCAPSNSDFFLNGGYSYRGVIFPTLRNSFKSRDLERLYQRYFLGQRRKSEVVMNVLDVLTKLTLLVLHLSLASAPMDPLKGILLGFFTGIEVVICALVVVRKDNTSHTYLQYSGVVTWVAMTTQILAAGLGYGLLGDGIGYVLFTLFATYSMLPLPLTWAILAGLGTSLLQVTLQVLIPRLAVFSINQVLAQVVLFMCMNTAGIFISYLSDRAQRQAFLETRRCVEARLRLETENQRQERLVLSVLPRFVVLEMINDMTNVEDEHLQHQFHRIYIHRYENVSILFADVKGFTNLSTTLSAQELVRMLNELFARFDRLA	AF-P97490
stringdb:10090.ENSMUSP00000115406	2.006915	1.568898	1.439726	1.08482	1.753284	4.234672	0.938682	1.425056	1.953127	0.751852	4.763944	Adcy3	4.761904762	10090.ENSMUSP00000115406	Adenylate cyclase type 3; Catalyzes the formation of the signaling molecule cAMP in response to G-protein signaling. Participates in signaling cascades triggered by odorant receptors via its function in cAMP biosynthesis. Required for the perception of odorants. Required for normal sperm motility and normal male fertility. Plays a role in regulating insulin levels and body fat accumulation in response to a high fat diet.	label: attribute="display name" labelsize=12 labelAlignment=left position=northeast outline=true outlineColor=white outlineTransparency=95 outlineWidth=10 background=false color=black dropShadow=false	https://version-12-0.string-db.org//images/Protein pictures/sm/403/403e49bc3edfd4b7d9482b36.png	rgb(239,148,117)	MPRNQGFSDPEYSAEYSAEYSVSLPSDPDRGVGRTHEISVRNSGSCLCLPRFMRLTFVPESLENLYQTYFKRQRHETLLVLVVFAALFDCYVVVMCAVVFSSDKLAPLMVAGFGLVLDIILFVLCKKGLLPDRVSRKVVPYLLWLLISAQIFSYLGLNFSRAHAASDTVGWQAFFVFSFFITLPLSLSPIVIISVVSCVVHTLVLGVTVAQQQQDELEGMQLLREILANVFLYLCAIIVGIMSYYMADRKHRKAFLEARQSLEVKMNLEEQSQQQENLMLSILPKHVADEMLKDMKKDESQKDQQQFNTMYMYRHENVSILFADIVGFTQLSSACSAQELVKLLNELFARFDKLAAKYHQLRIKILGDCYYCICGLPDYREDHAVCSILMGLAMVEAISYVREKTKTGVDMRVGVHTGTVLGGVLGQKRWQYDVWSTDVTVANKMEA	AF-Q8VHH7
stringdb:10090.ENSMUSP00000023132	1.199875	4.712406	1.164828	0.635552	1.391168	0.819558	0.86381	1.906349	2.206784	0.46875	1.73497	Pde1b	4	10090.ENSMUSP00000023132	Calcium/calmodulin-dependent 3',5'-cyclic nucleotide phosphodiesterase 1B; Cyclic nucleotide phosphodiesterase with a dual-specificity for the second messengers cAMP and cGMP, which are key regulators of many important physiological processes. Has a preference for cGMP as a substrate (By similarity).	label: attribute="display name" labelsize=12 labelAlignment=left position=northeast outline=true outlineColor=white outlineTransparency=95 outlineWidth=10 background=false color=black dropShadow=false	https://version-12-0.string-db.org//images/Protein pictures/pdb/1t/1taz_A.png	rgb(117,239,218)	MELSPRSPPEMLESDCPSPLELKSAPSKKMWIKLRSLLRYMVKQLENGEVNIEELKKNLEYTASLLEAVYIDETRQILDTEDELRELRSDAVPSEVRDWLASTFTQQTRAKGRRAEEKPKFRSIVHAVQAGIFVERMFRRTYTSVGPTYSTAVHNCLKNLDLWCFDVFSLNRAADDHALRTIVFELLTRHSLISRFKIPTVFLMSFLEALETGYGKYKNPYHNQIHAADVTQTVHCFLLRTGMVHCLSEIEVLAIIFAAAIHDYEHTGTTNSFHIQTKSECAILYNDRSVLENHHISSVFRMMQDDEMNIFINLTKDEFAELRALVIEMVLATDMSCHFQQVKTMKTALQQLERIDKSKALSLLLHAADISHPTKQWSVHSRWTKALMEEFFRQGDKEAELGLPFSPLCDRTSTLVAQSQIGFIDFIVEPTFSVLTDVAEKSVQPLADDDSKPKS	1taz_A|AF-Q01065
stringdb:10090.ENSMUSP00000124562	1.637436	1.922296	4.021245	1.147159	4.370257	1.711639	1.468821	2.239094	1.945697	0.946479	1.706653	Manf	4	10090.ENSMUSP00000124562	Mesencephalic astrocyte-derived neurotrophic factor; Selectively promotes the survival of dopaminergic neurons of the ventral mid-brain. Modulates GABAergic transmission to the dopaminergic neurons of the substantia nigra. Enhances spontaneous, as well as evoked, GABAergic inhibitory postsynaptic currents in dopaminergic neurons. Inhibits cell proliferation and endoplasmic reticulum (ER) stress-induced cell death. Retained in the ER/sarcoplasmic reticulum (SR) through association with the endoplasmic reticulum	label: attribute="display name" labelsize=12 labelAlignment=left position=northeast outline=true outlineColor=white outlineTransparency=95 outlineWidth=10 background=false color=black dropShadow=false	https://version-12-0.string-db.org//images/Protein pictures/pdb/2r/2rqy_A.png	rgb(117,153,239)	MWATRGLAVALALSVLPDSRALRPGDCEVCISYLGRFYQDLKDRDVTFSPATIEEELIKFCREARGKENRLCYYIGATDDAATKIINEVSKPLAHHIPVEKICEKLKKKDSQICELKYDKQIDLSTVDLKKLRVKELKKILDDWGEMCKGCAEKSDYIRKINELMPKYAPKAASARTDL	2kve_A|2rqy_A|6h9u_B|AF-Q9CXI5
stringdb:10090.ENSMUSP00000028059	1.792287	2.728425	0.746446	0.903996	1.332186	0.73652		1.059054	2.570983		1.391197	Rsu1	4	10090.ENSMUSP00000028059	Ras suppressor protein 1; Potentially plays a role in the Ras signal transduction pathway. Capable of suppressing v-Ras transformation in vitro.	label: attribute="display name" labelsize=12 labelAlignment=left position=northeast outline=true outlineColor=white outlineTransparency=95 outlineWidth=10 background=false color=black dropShadow=false	https://version-12-0.string-db.org//images/Protein pictures/pdb/7d/7d2t_A.png	rgb(117,239,133)	MSKSLKKLVEESREKNQPEVDMSDRGISSMLDVNGLFSLAHITQLVLSHNKLTTVPPNVAELKNLEVLNFFNNQIEELPTQISSLQKLKHLNLGMNRLNTLPRGFGSLPALEVLDLTYNNLNEHSLPGNFFYLTTLRALYLSDNDFEILPPDIGKLTKLQILSLRDNDLISLPKEIGELTQLKELHIQGNRLTVLPPELGNLDLTGQKQVFKAENNPWVTPIADQFQLGVSHVFEYIRSETYKYLYGRHMQANPEPPKKNNDKSKKISRKPLAAKNK	7d2s_A|7d2t_A|AF-Q01730
stringdb:10090.ENSMUSP00000023072	3.884351	2.741553	0.911128	1.035141	1.690903	0.608365	0.92943	2.043258	2.411843		3.736772	Parvb	4.523809524	10090.ENSMUSP00000023072	Beta-parvin; Adapter protein that plays a role in integrin signaling via ILK and in activation of the GTPases CDC42 and RAC1 by guanine exchange factors, such [illegible]	label: attribute="display name" labelsize=12 labelAlignment=left position=northeast outline=true outlineColor=white outlineTransparency=9 [illegible]	https://version-12-0.string-db.org//images/Protein pictures/pdb/4e/4edn_A.png	rgb(117,239,195)	MSSAPPRSPTPRAPKMKKDESFLGKLGGTLARKKKTREVTDLQEGKSAINSPMAPALVDIHPEDTQLEENEERTMIDPTSREDPKFKELVKVLLDWINDVLAEERIIVKQLEEDLYDGQVLQ [illegible]	4edn_A|AF-Q9ES46
[illegible]	[illegible]	[illegible]	[illegible]	[illegible]	[illegible]	[illegible]	[illegible]	[illegible]	[illegible]	[illegible]	[illegible]	Pde4a	[illegible]	[illegible]	[illegible]	[illegible]	[illegible]	[illegible]	[illegible]	[illegible]
[illegible]	[illegible]	[illegible]	[illegible]	[illegible]	[illegible]	[illegible]	[illegible]	[illegible]	[illegible]	[illegible]	[illegible]	Psmd5	[illegible]	[illegible]	[illegible]	[illegible]	[illegible]	[illegible]	[illegible]	[illegible]
[illegible]	[illegible]	[illegible]	[illegible]	[illegible]	[illegible]	[illegible]	[illegible]	[illegible]	[illegible]	[illegible]	[illegible]	Psme1	[illegible]	[illegible]	[illegible]	[illegible]	[illegible]	[illegible]	[illegible]	[illegible]
[illegible]	[illegible]	[illegible]	[illegible]	[illegible]	[illegible]	[illegible]	[illegible]	[illegible]	[illegible]	[illegible]	[illegible]	Frrs1l	[illegible]	[illegible]	[illegible]	[illegible]	[illegible]	[illegible]	[illegible]	[illegible]
[illegible]	[illegible]	[illegible]	[illegible]	[illegible]	[illegible]	[illegible]	[illegible]	[illegible]	[illegible]	[illegible]	[illegible]	Hcn3	[illegible]	[illegible]	[illegible] facilitate the permeation of sodium ions. Belongs to the potassium channel HCN family.	[illegible] color=black dropShadow=false	[illegible]	[illegible]	[illegible] RAITRYLRTWFLVDLISSIPVDYIFLVVELEPRLDAEVYKTARALRIVRFTKILSLLRLLRLSRLIRYIHQWEEIFHMTYDLASAVVRIFNLIGMMLLLCHWDGCLQFLVPMLQDFPSDCWVSMNRMVNHSWGRQYSHALFKAMSHMLCIGYGQQAPVGMPDVWLTMLSMIVGATCYAMFIGHATALIQSLDSSRRQYQEKYKQVEQYMSFHKLPADTRQRIHEYYEHRYQGKMFDEESILGELSEPLREEIINFTCRGLVAHMPLFAHADPSFVTAVLTKLRFEVFQPGDLV	[illegible]
stringdb:10090.ENSMUSP00000125142	1.475132	2.723981	1.789987	0.607439	1.392941	0.932451	0.630548	1.410955	2.791757		5	Kcnip2	4	10090.ENSMUSP00000125142	Kv channel-interacting protein 2; Regulatory subunit of Kv4/D (Shal)-type voltage-gated rapidly inactivating A-type potassium channels. Modulates channel density, inactivation kinetics and rate of recovery from inactivation in a calcium-dependent and isoform-specific manner. In vitro, modulates KCND2/Kv4.2 and KCND3/Kv4.3 currents. Involved in KCND2 and KCND3 trafficking to the cell surface. Essential for the expression of I(To) currents in the heart. Required for normal protein levels of KCND2	label: attribute="display name" labelsize=12 labelAlignment=left position=northeast outline=true outlineColor=white outlineTransparency=95 outlineWidth=10 background=false color=black dropShadow=false	https://version-12-0.string-db.org//images/Protein pictures/pdb/7u/7ukh_I.png	rgb(117,239,139)	MRGQGRKESLSESRDLDGSYDQLTGHPPGPSKKALKQRFLKLLPCCGPQALPSVSETLAAPASLRPHRPRPLDPDSVEDEFELSTVCHRPEGLEQLQEQTKFTRRELQVLYRGFKNECPSGIVNEENFKQIYSQFFPQGDSSNYATFLFNAFDTNHDGSVSFEDFVAGLSVILRGTIDDRLNWAFNLYDLNKDGCITKEEMLDIMKSIYDMMGKYTYPALREEAPREHVESFFQKMDRNKDGVVTIEEFIESCQQDENIMRSMQLFDNVI	7ukh_I|AF-Q9JJ69
stringdb:10090.ENSMUSP00000100025	1.471798	4.092714	1.247995	4.046252	1.809399	1.737755	2.154878	1.124933	2.39178		1.851216	Stam	4	10090.ENSMUSP00000100025	Signal transducing adapter molecule 1; Involved in intracellular signal transduction mediated by cytokines and growth factors. Upon IL-2 and GM-CSL stimulation, it plays a role in signaling leading to DNA synthesis and MYC induction. May also play a role in T-cell development. Involved in down-regulation of receptor tyrosine kinase via multivesicular body (MVBs) when complexed with HGS (ESCRT-0 complex). The ESCRT-0 complex binds ubiquitin and acts as sorting machinery that recognizes ubiquitinated receptors and transfers them to further sequential	label: attribute="display name" labelsize=12 labelAlignment=left position=northeast outline=true outlineColor=white outlineTransparency=95 outlineWidth=10 background=false color=black dropShadow=false	https://version-12-0.string-db.org//images/Protein pictures/pdb/3l/3ldz_A.png	rgb(125,239,117)	MPLFATNPFDQDVEKATSELNTAEDWGLILDICDKVGQSRTGPKDCLRSIMRRVNHKDPHVAMQALTLLGACVSNCGKIFHLEVCSRDFASEVSNVLNKGHPKVCEKLKALMVEWTDEFKNDPQLSLISAMIKNLKEQGVTFPAIGSQAAEQAKASPALVAKDPGTVATKKEEEDLAKAIELSLKEQRQQSAPVSTLYPSTSNLLTNHQHEGRKVRAVYDFEAAEDNELTFKAGEIITVLDDSDPNWWKGETHQGVGLFPSNFVTADLTAEPEMIKTEKKTVQFNDDVQIETIEPEPEPAFIDEDKMDQLLQMLQSTDPSDNQPDLPELLHLEAMCHQMGPLIDEKLEDIDRKHSELSELNVKVMEALSLYTKLMNEDPMYSMYAKLQSQQYYLQSSAVSASQVYPGPAQSGTYLVAGSAQMTHLQSYSLPPEQLSSISQGAV	2l0a_A|3ldz_A|AF-P70297
stringdb:10090.ENSMUSP00000155364	1.987521	2.667989	1.524955	1.024928	1.58711	1.128016	2.477897	1.795835	5	0.935278	1.815796	Rbfox1	0	10090.ENSMUSP00000155364	RNA binding protein fox-1 homolog 1; RNA-binding protein that regulates alternative splicing events by binding to 5'-UGCAUGU-3' elements. Prevents binding of U2AF2 to the 3'-splice site. Regulates alternative splicing of tissue- specific exons and of differentially spliced exons during erythropoiesis.	label: attribute="display name" labelsize=12 labelAlignment=left position=northeast outline=true outlineColor=white outlineTransparency=95 outlineWidth=10 background=false color=black dropShadow=false	https://version-12-0.string-db.org//images/Protein pictures/pdb/4z/4zka_C.png	rgb(117,239,168)	MLASQGVLLHSYGVPMIVPAAPYFPGLMQGNQEAAAAPDTMAQPYASAQFAPPQNGIPAEYTAPHPHPAPEYTGQTTVPDHTLNLYPPTQTHSEQSADTSAQTVSGTATQTDDAAPTDGQPQTQPSENTESKSQPKRLHVSNIPFRFRDPDLRQMFGQFGKILDVEIIFNERGSKGFGFVTFENSADADRAREKLHGTVVEGRKIEVNNATARVMTNKKTVNPYTNGWKLNPVVGAVYSPDFYAGTVLLCQANQEGSSMYSGPSSLVYTSAMPGFPYPAATAAAAYRGAHLRGRGRTVYNTFRAAAPPPPIPAYGGVVYQEPVYGNKLLQGGYAAYRYAQPTPATAAAYSDSYGRVYAADPYHHTLAPAPTYGVGAMNAFAPLTDAKTRSHADDVGLVLSSLQASIYRGGYNRFAPY	2err_A|4zka_C|AF-Q9JJ43
stringdb:10090.ENSMUSP00000123234	1.901728	3.143219	3.610025	4.378083	1.653267	2.033601	1.380445	2.323264	2.226434		4.440619	Grip1	0.666666667	10090.ENSMUSP00000123234	Glutamate receptor-interacting protein 1; May play a role as a localized scaffold for the assembly of a multiprotein signaling complex and as mediator of the trafficking of its binding partners at specific subcellular location in neurons (By similarity). Through complex formation with NSG1, GRIA2 and STX12 controls the intracellular fate of AMPAR and the endosomal sorting of the GRIA2 subunit toward recycling and membrane targeting (By similarity).	label: attribute="display name" labelsize=12 labelAlignment=left position=northeast outline=true outlineColor=white outlineTransparency=95 outlineWidth=10 background=false color=black dropShadow=false	https://version-12-0.string-db.org//images/Protein pictures/pdb/2q/2qt5_A.png	rgb(239,234,117)	MIAVSFKCRCQILRRLTKDESPYTKSASQTKPPDGALAVRRQSIPEEFKGSTVVELMKKEGTTLGLTVSGGIDKDGKPRVSNLRQGGIAARSDQLDVGDYIKAVNGINLAKFRHDEIISLLKNVGERVVLEVEYELPPVSVQGSSVMFRTVEVTLHKEGNTFGFVIRGGAHDDRNKSRPVVITCVRPGGPADREGTIKPGDRLLSVDGIRLLGTTHAEAMSILKQCGQEATLLIEYDVSVMDSVATASGPLLVEVAKTPGASLGVALTTSVCCNKQVIVIDKIKSASIADRCGALHVGDHILSIDGTSMEYCTLAEATQFLANTTDQVKLEILPHHQTRLALKGPDHVKIQRSDRQHPWDAWASNQCGVHTNHHHNTYHPDHCRVPALTFPKALPPNSPPAMVPSSSPTSMSAYSLSSLNMGTLPRSLYSTSPRGTMMRRRLKKKDFKS	1m5z_A|1n7f_A|1p1d_A|1v5q_A|2jil_B|2qt5_A|AF-Q925T6
stringdb:10090.ENSMUSP00000099650	1.237106	4.683003	1.137619	1.116041	0.852224	0.791509	0.649289	1.178721	4.619769		1.823166	Kpna6	2	10090.ENSMUSP00000099650	Importin subunit alpha-7; Functions in nuclear protein import as an adapter protein for nuclear receptor KPNB1. Binds specifically and directly to substrates containing either a simple or bipartite NLS motif. Docking of the importin/substrate complex to the nuclear pore complex (NPC) is mediated by KPNB1 through binding to nucleoporin FxFG repeats and the complex is subsequently translocated through the pore by an energy requiring, Ran-dependent mechanism. At the nucleoplasmic side of the NPC, Ran	label: attribute="display name" labelsize=12 labelAlignment=left position=northeast outline=true outlineColor=white outlineTransparency=95 outlineWidth=10 background=false color=black dropShadow=false	https://version-12-0.string-db.org//images/Protein pictures/pdb/4u/4uad_A.png	rgb(117,117,239)	METMASPGKDNYRMKSYKNNALNPEEMRRRREEEGIQLRKQKREQQLFKRRNVELINEEAAMFDSLLMDSYVSSTTGESVITREMVEMLFSDDSDLQLATTQKFRKLLSKEPSPPIDEVINTPGVVDRFVEFLKRNENCTLQFEAAWALTNIASGTSQQTKIVIEAGAVPIFIELLNSDFEDVQEQAVWALGNIAGDSSLCRDYVLNCSILNPLLTLLTKSTRLTMTRNAVWALSNLCRGKNPPPEFAKVSPCLPVLSRLLFSSDSDLLADACWALSYLSDGPNEKIQAVIDSGVCRRLVELLMHNDYKVASPALRAVGNIVTGDDIQTQVILNCSALPCLLHLLSSSKESIRKEACWTISNITAGNRAQIQAVIDANIFPVLIEILQKAEFRTRKEAAWAITNATSGGTPEQIRYLVSLGCIKPLCDLLTVMDSKIVQVALNGLENILRLGEQE	4u2x_D|4uad_A|AF-O35345
stringdb:10090.ENSMUSP00000141227	1.595408	4.697772	1.212565	1.03978	1.475634	0.806141	0.937864	1.607407	5	0.645355	1.484708	Kpna4	2	10090.ENSMUSP00000141227	Importin subunit alpha-3; Functions in nuclear protein import as an adapter protein for nuclear receptor KPNB1. Binds specifically and directly to substrates containing either a simple or bipartite NLS motif. Docking of the importin/substrate complex to the nuclear pore complex (NPC) is mediated by KPNB1 through binding to nucleoporin FxFG repeats and the complex is subsequently translocated through the pore by an energy requiring, Ran-dependent mechanism. At the nucleoplasmic side of the NPC, Ran	label: attribute="display name" labelsize=12 labelAlignment=left position=northeast outline=true outlineColor=white outlineTransparency=95 outlineWidth=10 background=false color=black dropShadow=false	https://version-12-0.string-db.org//images/Protein pictures/pdb/5t/5tbk_A.png	rgb(117,239,163)	MADNEKLDNQRLKNFKNKGRDLETMRRQRNEVVVELRKNKRDEHLLKRRNVPQEDICEDSDIDGDYRVQNTSLEAIVQNASSDNQGIQLSAVQAARKLLSSDRNPPIDDLIKSGILPILVHCLERDDNPSLQFEAAWALTNIASGTSEQTQAVVQSNAVPLFLRLLHSPHQNVCEQAVWALGNIIGDGPQCRDYVISLGVVKPLLSFISPSIPITFLRNVTWVMVNLCRHKDPPPPMETIQEILPALCVLIHHTDVNILVDTVWALSYLTDAGNEQIQMVIDSGIVPHLVPLLSHQEVKVQTAALRAVGNIVTGTDEQTQVVLNCDALSHFPALLTHPKEKINKEAVWFLSNITAGNQQQVQAVIDANLVPMIIHLLDKGDFGTQKEAAWAISNLTISGRKDQVAYLIQQNVIPPFCNLLTVKDAQVVQVVLDGLSNILKMAED	5tbk_A|5xzx_A|6wx8_A|AF-O35343

stringdb:10090.ENSMUSP00000032874	1.679033	3.4607	1.140296	3.791866	1.190896	1.894264	1.753238	1.458887	2.821898		1.770233	Sh3gl3	4	10090.ENSMUSP00000032874	Endophilin-A3; Implicated in endocytosis. May recruit other proteins to membranes with high curvature (By similarity); Belongs to the endophilin family.	label: attribute="display name" labelsize=12 labelAlignment=left position=northeast outline=true outlineColor=white outlineTransparency=95 outlineWidth=10 background=false color=black dropShadow=false	https://version-12-0.string-db.org//images/Protein pictures/pdb/2z/2z0v_A.png	rgb(117,239,151)	MSVAGLKKQFHKASQLFSEKISGAEGTKLDEEFLNMEKKIDITSKAVAEILSKATEYLQPNPAYRAKLGMLNTVSKLRGQVKATGYPQTEGLLGDCMLKYGKELGEDSAFGNSLVDVGEALKLMAEVKDSLDINVKQTFIDPLQLLQDKDLKEIGHHLRKLEGRRLDYDYKKRRVGKIPEEEIRQAVEKFEESKELAERSMFNFLENDVEQVSQLAVFVEAALDYHRQSTEILQELQSKLELRISLASKVPKREFMPKPVNMSSTDANGVGPSSSSKTPGTDTPSDQPCCRGLYDFEPENEGELGFKEGDIITLTNQIDENWYEGMLRGESGFFPINYVEVIVPLPP	2ew3_A|2z0v_A|AF-Q62421
stringdb:10090.ENSMUSP00000103514	2.191681	5	1.996842	2.010928	2.06766	1.414086	2.427506	2.753314	2.917272	1.512527	2.296701	Akt1s1	4	10090.ENSMUSP00000103514	Proline-rich AKT1 substrate 1; Subunit of mTORC1, which regulates cell growth and survival in response to nutrient and hormonal signals. mTORC1 is activated in response to growth factors or amino acids. Growth factor-stimulated mTORC1 activation involves a AKT1-mediated phosphorylation of TSC1- TSC2, which leads to the activation of the RHEB GTPase that potently activates the protein kinase activity of mTORC1. Amino acid-signaling to mTORC1 requires its relocalization to the lysosomes mediated by the Ragulator complex	label: attribute="display name" labelsize=12 labelAlignment=left position=northeast outline=true outlineColor=white outlineTransparency=95 outlineWidth=10 background=false color=black dropShadow=false	https://version-12-0.string-db.org//images/Protein pictures/pdb/6s/6sb0_T.png	rgb(117,117,239)	MTQEAAMPFEGGDGAWPAMLATGTARMASGRPEELWEAVVGAAERFQARTGTELVLLTAAPPPPPRPGPCAYAAHGRGALAEAARRCLHDIAQAHRAATATRPPGPPPAPQPPSPAPSPPPRPALAREDEEEDEDEPTETETSGERLGGSDNGGLFMMDEDATLQDLPPFCESDPESTDDGSLSEETPAGPTACPQPPATALPTQQYAKSLPVSVPVWAFKEKRTEARSSDEENGPPSSPDLDRIAASMRALVLREAEDTQVFGDLPRPRLNTSDFQKLKRKY	5wbu_R|6sb0_T|AF-Q9D1F4

This table lists significant protein-protein interaction (PPI) hub nodes. "MPTP" denotes the acute MPTP model, "A53T" the 16m A53T-α-synuclein model, and "MitoPark" the 5m MitoPark model.
